# Supplementary material for: Integrating multiple sources of ecological data to unveil macroscale species abundance
Source: Nat Commun. 2020 Apr 3;11:1695. doi: 10.1038/s41467-020-15407-5 (PMC7125090; doi:10.1038/s41467-020-15407-5)
Supplement: Supplementary file 1 — Supplementary Information [file 41467_2020_15407_MOESM1_ESM.pdf]

Supplementary information: Integrating multiple sources of ecological data to unveil macroscale species abundance

K. Fukaya et al.

Contents

Supplementary Note 1. Statistical inference of the integrated model **2**

Supplementary Note 2. Additional details on the model application to the woody plant community **4**

Supplementary Note 3. Additional tables and figures **9**

Supplementary Note 4. Maps of the abundance of species with lower extinction risks **18**

Supplementary References **372**

Supplementary Note 1. Statistical inference of the integrated model

Model fitting procedure

As a class of general hierarchical models, the integrated model, described in *Integrating cell-level occurrence information* in the *Methods* section, can be fitted to data by using either maximum marginal likelihood (also known as empirical Bayes) or fully Bayesian approach. Let us denote $\mathbf{D}$ as the vector of all data, including the partially-observed latent state z_{ij} for notational convenience. We denote the vector of all parameters (i.e. $\mu, \eta, \sigma_1, \sigma_2, \sigma_3, \tau_1$, and τ_2) and the vector of all other random effects (i.e. $e_i^{(1)}, e_j^{(2)}, e_{ij}^{(3)}, u_i^{(1)}$, and $u_j^{(2)}$) by $\boldsymbol{\theta}$ and $\boldsymbol{\xi}$, respectively. In both approaches, inference is based on a joint distribution of data and random effects, $p(\mathbf{D}, \boldsymbol{\xi} \mid \boldsymbol{\theta})$, which is also known as a complete data likelihood (King 2014) and is described for the integrated model in the following section. In the maximum marginal likelihood approach, the estimation can be achieved via a two-stage procedure, where parameters are estimated by maximising a marginal likelihood $p(\mathbf{D} \mid \boldsymbol{\theta}) = \int p(\mathbf{D}, \boldsymbol{\xi} \mid \boldsymbol{\theta}) d\boldsymbol{\xi}$ and then, maximum a posteriori probability (MAP) estimates of random effects can be obtained conditionally on the parameter estimates $\hat{\boldsymbol{\theta}}$ by maximising $p(\mathbf{D}, \boldsymbol{\xi} \mid \hat{\boldsymbol{\theta}})$. Although an evaluation of the marginal likelihood may be computationally challenging, some recently developed software, such as **AD Model Builder** (Fournier *et al.* 2012) and **Template Model Builder** (Kristensen *et al.* 2016), can efficiently approximate the marginal likelihood of a wide class of hierarchical models by using the Laplace approximation. In contrast, in the fully Bayesian approach, the focus of inference is the joint posterior distribution of parameters and random effects $p(\boldsymbol{\theta}, \boldsymbol{\xi} \mid \mathbf{D}) = \frac{p(\mathbf{D}, \boldsymbol{\xi} \mid \boldsymbol{\theta}) p(\boldsymbol{\theta})}{\int \int p(\mathbf{D}, \boldsymbol{\xi} \mid \boldsymbol{\theta}) p(\boldsymbol{\theta}) d\boldsymbol{\xi} d\boldsymbol{\theta}}$, where a prior distribution for parameters $p(\boldsymbol{\theta})$ is needed to be specified. Although the integration over parameters and random effects is not tractable in general, a Markov chain Monte Carlo (MCMC) method can be used to obtain random samples from the posterior distribution. Several generic software tools are available to run MCMC for a vast array of hierarchical models (e.g. Plummer 2003, Carpenter *et al.* 2017).

Likelihood of the model

By letting $\mathbf{y}_{ij} = (y_{ij1}, \ldots, y_{ijK_j})$ and $\mathbf{D}_{ij} = (\mathbf{y}_{ij}, z_{ij})$, the complete data likelihood of the model can be expressed as:

$$p(\mathbf{D}, \boldsymbol{\xi} \mid \boldsymbol{\theta}) = p(\boldsymbol{\xi} \mid \boldsymbol{\theta}) \prod_{i,j} p(\mathbf{D}_{ij} \mid \boldsymbol{\xi}, \boldsymbol{\theta}),$$

where $p(\boldsymbol{\xi} \mid \boldsymbol{\theta})$ represents a joint probability density of random effects that is determined by the system model (Equations 4–6 and 10–11) and $p(\mathbf{D}_{ij} \mid \boldsymbol{\xi}, \boldsymbol{\theta})$ is the conditional likelihood derived from

the observation model (Equations 2 and 8). Under the independent normal assumption, the former is described as:

$$p(\boldsymbol{\xi} \mid \boldsymbol{\theta}) = \left\{ \prod_i \mathcal{N}(e_i^{(1)} \mid 0, \sigma_1^2) \mathcal{N}(u_i^{(1)} \mid 0, \tau_1^2) \right\} \left\{ \prod_j \mathcal{N}(e_j^{(2)} \mid 0, \sigma_2^2) \mathcal{N}(u_j^{(2)} \mid 0, \tau_2^2) \right\} \prod_{i,j} \mathcal{N}(e_{ij}^{(3)} \mid 0, \sigma_3^2),$$

where $\mathcal{N}(x \mid 0, \sigma^2)$ denotes the probability density of a normal distribution with mean 0 and variance σ^2 evaluated at x. The latter takes two cases depending on whether the presence-absence of species i in grid cell j is known (denoted by $m_{ij} = 1$) or not (denoted by $m_{ij} = 0$):

$$p(\mathbf{D}_{ij} \mid \boldsymbol{\xi}, \boldsymbol{\theta}) = \begin{cases} \psi_{ij}^{z_{ij}} (1 - \psi_{ij})^{1 - z_{ij}} \left[\prod_{k=1}^{K_j} \{1 - \exp(-z_{ij} d_{ij} a_{jk})\}^{y_{ijk}} \exp(-z_{ij} d_{ij} a_{jk})^{(1 - y_{ijk})} \right] \\ \qquad\qquad\qquad\qquad\qquad\qquad m_{ij} = 1 \\ \psi_{ij} \left[\prod_{k=1}^{K_j} \exp(-d_{ij} a_{jk}) \right] + (1 - \psi_{ij}) \qquad m_{ij} = 0, \end{cases} \tag{1}$$

where in the former case, the conditional likelihood is given as a joint likelihood of $\mathbf{y}_{ij}$ and z_{ij}, and in the latter case, it is given by the marginalised likelihood of $\mathbf{y}_{ij}$ because z_{ij} is missing. We note that d_{ij} and ψ_{ij} are respectively a function of $\boldsymbol{\xi}$ and $\boldsymbol{\theta}$ (Equations 3 and 9), although that is not expressed explicitly in the right-hand side of the equations.

We note that in this integrated model, geographical grid cells that contain no detection-nondetection observations but have cell-level presence-absence information for some species can still contribute to the inference. Let us now assume that the set of geographical areas of interest is divided into J geographical grid cells, in which cell j ($j = 1, \ldots, J$) contains $K_j \geq 0$ plots. Then, for cell j such that $K_j > 0$, the conditional likelihood is expressed by Equation 1, and for other cells ($K_j = 0$), it is written as:

$$p(\mathbf{D}_{ij} \mid \boldsymbol{\xi}, \boldsymbol{\theta}) = \begin{cases} \psi_{ij}^{z_{ij}} (1 - \psi_{ij})^{1 - z_{ij}} & m_{ij} = 1 \\ 1 & m_{ij} = 0. \end{cases}$$

Supplementary Note 2. Additional details on the model application to the woody plant community

As noted in the main text, the hierarchical models can be extended in various ways by, for example, adding environmental covariates and modelling correlation structure of random effects explicitly. Nevertheless, given that our specific data set required a large amount of computer memory and computation time to fit the models, we were unable to conduct a thorough comparison of such model variants that we could potentially obtain. Therefore, we examined a limited set of nested models that were built sequentially by adding a new model component to the previous simpler model variant. First, as the simplest baseline model, we fitted a model for spatially replicated detection-nondetection observations. This model does not use the cell-level species occurrence information and therefore is without data integration. Second, we fitted an integrated model that simultaneously explains replicated detection-nondetection observations and cell-level occurrence information. Third, we fitted a model in which a correlation between the two cell random effects is explicitly accounted for because the results of the second model suggested a negative correlation between them. Finally, to explain geographical variation in individual density and occurrence of species more explicitly, we further considered a series of models with two covariates that are related to climate and potential human impact. We did not include models with geographic and/or phylogenetic random effects because of their additional computational burden, although an introduction of such structured random effects may result in further improvement.

The fitted models are described below. All the models were fitted to data by using the maximum marginal likelihood estimation procedure implemented in the **Template Model Builder** (Kristensen *et al.* 2016), with the aid of **TMB** package (version 1.7.15) run in **R** (versions 3.5.0 and 3.5.1). The models were compared based on AIC and three other benchmarks of predictive performance that we described in the main text.

Model A: without data integration This model is identical to that described in *A model for spatially replicated detection-nondetection data* in the *Methods* section: i.e. a Bernoulli GLMM with complementary log-log link specified with Equations 1–6. Based on the assumption of a superposed homogeneous Poisson point process, the occurrence of each species in replicated local plots is described in terms of individual density of species.

Model B: data integration This model is identical to that described in *Integrating cell-level occurrence information* in the *Methods* section, which is specified with Equations 2–11. By accounting for the "zero-inflated" nature of underlying species abundance, it integrates plot-level detection-nondetection

observations and cell-level presence-absence information in a joint distribution of these data.

Model C: correlated random effects Model B is extended to account for the correlation between the two cell random effects, $e_j^{(2)}$ and $u_j^{(2)}$. Specifically, the independent normal distributions of the random effects (Equations 5 and 11) are replaced with the following bivariate normal distribution:

$$\begin{pmatrix} e_j^{(2)} \\ u_j^{(2)} \end{pmatrix} \sim \mathcal{N}_2 \left(\begin{bmatrix} 0 \\ 0 \end{bmatrix}, \begin{bmatrix} \sigma_2^2 & \rho\sigma_2\tau_2 \\ \rho\sigma_2\tau_2 & \tau_2^2 \end{bmatrix} \right),$$

where, as in the previous model, σ_2^2 and τ_2^2 represents the marginal variance of $e_j^{(2)}$ and $u_j^{(2)}$, respectively, whereas an additional parameter $\rho \in (-1, 1)$ represents the coefficient of correlation between them.

Model D: covariates Model C is extended to include covariates in the linear predictor for conditional density (Equation 3) and occurrence probability (Equation 9), respectively. Given that the model has two linear predictors that span over two dimensions (i.e. species and grid cells), there is a wide array of potential ways to add covariates into them. For example, the predictors may involve species-specific covariates or cell-specific covariates, or both. They may even have a set of covariates that differ from each other. Because we were able to have a number of covariates, including climatic, topographic, and geologic variables, in addition to species-specific properties, there were vast modelling options. Nevertheless, given the limited computational resources, we only used two cell-specific covariates, actual evapotranspiration (AET) and the human influence index (HII; Sanderson *et al.* 2002), to explain geographic variation in both conditional density and occurrence probability. AET was selected based on a preliminary assessment of the correlation between estimated values of $e_j^{(2)}$ and $u_j^{(2)}$ in Model C and potential climate-related covariates, in which AET had the strongest correlation. HII was selected as an additional covariate that was related to potential human impact. Although it had a positive correlation with AET (correlation coefficient: 0.485), there was no indication of serious collinearity (variance inflation factor: 1.308). We did not consider species-specific responses to these covariates.

We fitted two univariate models, a model with additive covariate effects, and a model with interaction between the covariates. In each model, the linear predictors (Equations 3 and 9) were replaced with the following:

Univariate (AET)

$$\log d_{ij} = \mu + \beta_1 x_{1j} + e_i^{(1)} + e_j^{(2)} + e_{ij}^{(3)}$$

$$\text{logit } \psi_{ij} = \eta + \gamma_1 x_{1j} + u_i^{(1)} + u_j^{(2)}$$

Univariate (HII)

$$\log d_{ij} = \mu + \beta_2 x_{2j} + e_i^{(1)} + e_j^{(2)} + e_{ij}^{(3)}$$

$$\text{logit } \psi_{ij} = \eta + \gamma_2 x_{2j} + u_i^{(1)} + u_j^{(2)}$$

Additive

$$\log d_{ij} = \mu + \beta_1 x_{1j} + \beta_2 x_{2j} + e_i^{(1)} + e_j^{(2)} + e_{ij}^{(3)}$$

$$\text{logit } \psi_{ij} = \eta + \gamma_1 x_{1j} + \gamma_2 x_{2j} + u_i^{(1)} + u_j^{(2)}$$

Interaction

$$\log d_{ij} = \mu + \beta_1 x_{1j} + \beta_2 x_{2j} + \beta_3 x_{1j} x_{2j} + e_i^{(1)} + e_j^{(2)} + e_{ij}^{(3)}$$

$$\text{logit } \psi_{ij} = \eta + \gamma_1 x_{1j} + \gamma_2 x_{2j} + \gamma_3 x_{1j} x_{2j} + u_i^{(1)} + u_j^{(2)},$$

where x_{1j} and x_{2j} respectively represent the values of AET and HII in cell j. Both covariates were scaled to have mean 0 and variance 1.

The AET data were obtained from Global Aridity Index and Potential Evapotranspiration Climate Database v1, provided by CGIAR consortium for spatial information (https://cgiarcsi.community/). The HII data were diverted from Kusumoto *et al.* (2017). Both datasets were matched to the grid specification adopted in this study to obtain an average value for each 10-km grid cell and to use it as the covariate. Geographic distribution of these covariates is shown in Supplementary Figure 2.

Result of the model comparison is summarized in Supplementary Table 1. Overall, the predictive performance of the model improved as the model became more complex. Specifically, among the models with data integration (i.e. Models B, C, and D), the increase in the model complexity resulted in a consistent decrease in AIC value. In each independent dataset for validation, the increase in the model complexity led to, in most cases, lower root mean square error (RMSE) of prediction although such an improvement was not evident in bias. In general, model estimates and validation dataset became more strongly correlated on a log-log scale as the model complexity increased, except when predicting individual density per unit area of natural forest in GMFT. Comprehensively, these results indicate that data integration, explicit consideration of the correlation between random effects, and inclusion of covariates can improve the predictive performance of the model.

We can thus reasonably conclude that Model D with an interaction effect was the best model. The estimates (and standard errors) of parameters were: $\hat{\mu} = 4.759$ (0.043), $\hat{\beta}_1 = -0.444$ (0.013), $\hat{\beta}_2 = 0.034$ (0.012), $\hat{\beta}_3 = -0.079$ (0.012), $\hat{\eta} = -3.273$ (0.074), $\hat{\gamma}_1 = 0.631$ (0.013), $\hat{\gamma}_2 = -0.087$ (0.013), $\hat{\gamma}_3 = 0.033$ (0.012), $\hat{\sigma}_1 = 1.371$ (0.030), $\hat{\sigma}_2 = 0.612$ (0.009), $\hat{\sigma}_3 = 1.219$ (0.003), $\hat{\tau}_1 = 2.551$ (0.052), $\hat{\tau}_2 = 0.727$ (0.008), and $\hat{\rho} = -0.596$ (0.013). The fitted regression surface within the range of observed covariates is shown in Supplementary Figure 3.

To further characterise the prediction of the model, we estimated RMSE and bias of the prediction of the individual density for each species and plotted them against average individual density (conditional on the presence of species) and area of species occupancy (Supplementary Figure 4). The logarithm of RMSE was more strongly correlated with the log of individual density than with the log of the area of occupancy. The bias tended to be smaller in most species, whereas a relatively smaller number of species with higher individual density had a particularly large negative bias. In particular, in NFI dataset that systematically covered the entire region, extreme bias only occurred in species with significantly narrower geographic distribution. As shown in Supplementary Table 2, a considerable fraction of predictive errors was indeed positive, indicating that the negative bias has occurred due to the failure of the model to predict extremely high species abundance that arises with lower frequency. These results suggest that, in terms of RMSE and bias, the prediction was in general reasonable for most species with lower density, whereas it was more difficult for species with higher density. The prediction can particularly be difficult for species with significantly narrow geographic distribution, for which the number of grid cells within the range of the species was limited. Additionally, we were able to obtain the correlation coefficient of log individual density between model prediction and validation dataset for each species. This quantity varied, however, largely where specific patterns along the average individual density and the area of species occupancy were rather unclear (Supplementary Figure 4).

In terms of the community-level abundance, the level of total individual density varied among the validation datasets, reflecting the difference in the size of individuals that are sampled or estimated (Supplementary Figure 5). The levels of individual density in FDP and NFI datasets were comparable but were slightly lower, on average, than the level of individual density predicted by the fitted model. On the other hand, in FSLE dataset, in which presumably smaller individuals were sampled as compared with FDP and NFI (see *Woody plant communities in East Asian islands* in the *Methods* section), the level of individual density was higher than that of the model prediction. Note that these tendencies in the forest inventory datasets were consistent with the bias observed in the prediction of the individual density of species (Table 1 in the main text). The level of individual density in GMFT dataset was lower than any other validation datasets. As implied by the positive bias (Table 1 in the

main text), it was also below that of the model prediction.

Supplementary Note 3. Additional tables and figures

Supplementary Table 1. **Summary of the preliminary model comparison.** Predictive performance of the fitted models to predict individual density per 1 km^2 natural forest was examined with four validation datasets (FDP, NFI, FSLE, and GMFT) and three benchmarks (RMSE, Bias, and Corr). The individual density of species was validated with FDP, NFI, and FSLE datasets, whereas the total density of woody plants was validated with the GMFT dataset. For GMFT, in which individual density was validated at the scale of the 10-km grid cell, the correlation coefficient calculated based on individual density per grid cell is also shown in the parenthesis. See Supplementary Note 2 for more details. Abbreviations: $\log L$ – Log likelihood; AIC – Akaike information criterion; FDP – Forest dynamics plots; NFI – National forest inventory; FSLE – Forest sampling plots along latitudinal and elevational gradients; GMFT – Global map of forest trees; RMSE – Root mean square error of the model prediction for individual density; Bias – Bias of the model prediction for individual density; Corr – Correlation coefficient of the log individual density between model prediction and validation dataset. The log likelihood and AIC of model A are not shown because they cannot be compared with other models; model A specifies the likelihood of replicated detection-nondetection data, whereas the remaining models specify the joint likelihood of the detection-nondetection data and cell-level presence-absence data.

			FDP			NFI			FSLE			GMFT		
	$\log L$	AIC	RMSE	Bias	Corr	RMSE	Bias	Corr	RMSE	Bias	Corr	RMSE	Bias	Corr
Model A	—	—	3105	19.7	0.319	3068	−16.7	0.247	5997	−442	0.397	179762	53073	−0.053 (0.851)
Model B	−3445607	6891229	2872	59.0	0.357	2866	17.9	0.270	5872	−338	0.480	170435	47311	−0.062 (0.812)
Model C	−3444659	6889334	2852	54.7	0.358	2854	26.2	0.276	5846	−346	0.485	162333	50645	−0.064 (0.865)
Model D														
AET	−3443483	6886987	2858	56.5	0.358	2855	28.1	0.275	5849	−334	0.485	161917	50969	−0.057 (0.869)
HII	−3444499	6889015	2855	55.9	0.358	2855	27.5	0.275	5847	−339	0.485	162095	50678	−0.057 (0.867)
Additive	−3443459	6886941	2859	56.7	0.358	2855	28.3	0.275	5849	−333	0.485	161906	50943	−0.056 (0.870)
Interaction	−3443437	6886903	2850	54.8	0.358	2854	27.2	0.276	5844	−344	0.485	161709	50788	−0.063 (0.868)

Supplementary Table 2. **Summary of the statistical distribution of the prediction errors.** Individual density of species per 1 km^2 natural forest was predicted by the best fit model and was then validated with three independent datasets (FDP, NFI, and FSLE). See Supplementary Note 2 for details. Abbreviations: FDP – Forest dynamics plots; NFI – National forest inventory; FSLE – Forest sampling plots along latitudinal and elevational gradients.

		Percentile						
Dataset	Mean	6.25%	12.5%	25%	50%	75%	87.5%	93.75%
FDP	54.8	7.85×10^{-2}	9.57×10^{-1}	6.12	47.4	1.96×10^{2}	5.34×10^{2}	1.06×10^{3}
NFI	27.2	1.99×10^{-1}	9.30×10^{-1}	5.19	41.5	2.07×10^{2}	5.24×10^{2}	1.05×10^{3}
FSLE	−344	-4.70×10^{-2}	2.74×10^{-1}	2.69	30.2	1.34×10^{2}	3.78×10^{2}	8.59×10^{2}

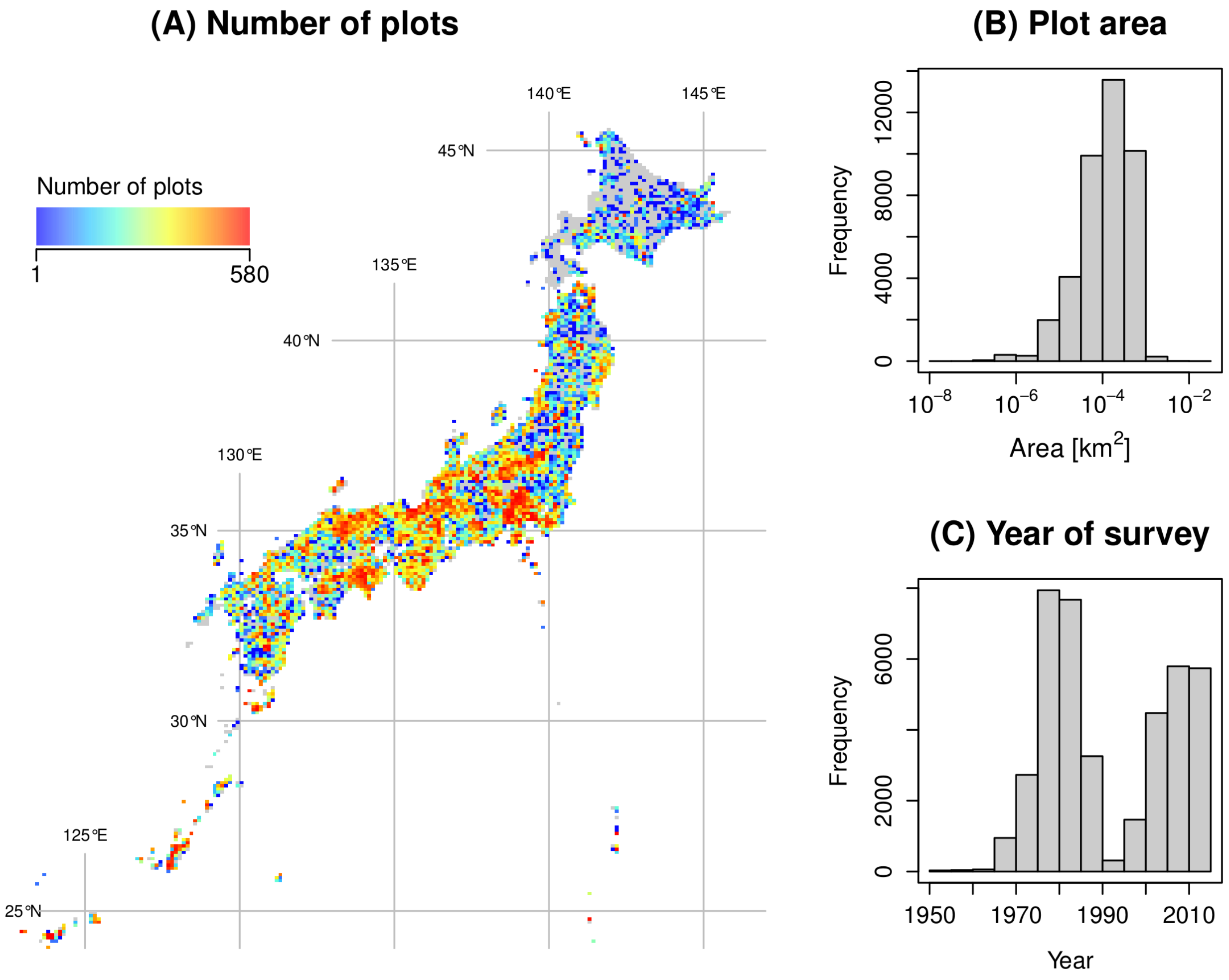

Supplementary Figure 1. **Summary of the 40,516 vegetation survey plots.** (A) The number of vegetation survey plots in each 10-km grid cell. Grid cells without any vegetation survey plot are shown in gray. (B) A histogram of the area of vegetation survey plots. (C) A histogram of the year of the vegetation survey. Note that for the year of survey (C), no information was available in 52 plots.

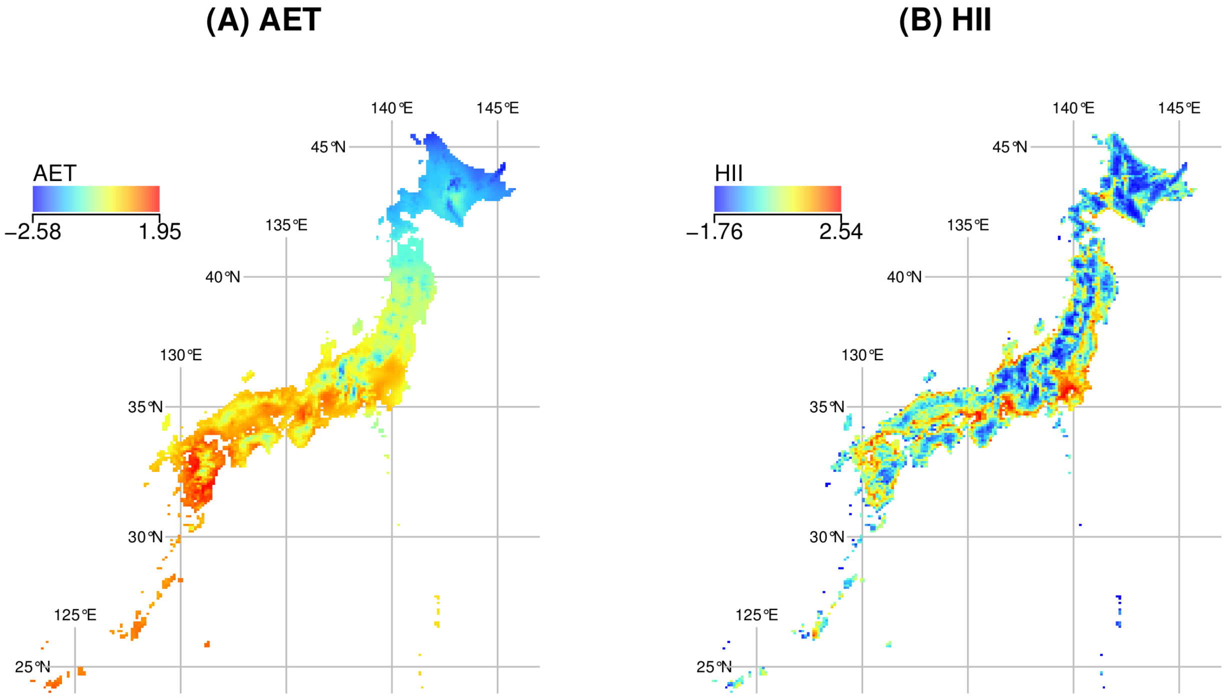

Supplementary Figure 2. **Geographic distribution of the values of covariates.** Both covariates were scaled to have mean 0 and variance 1.

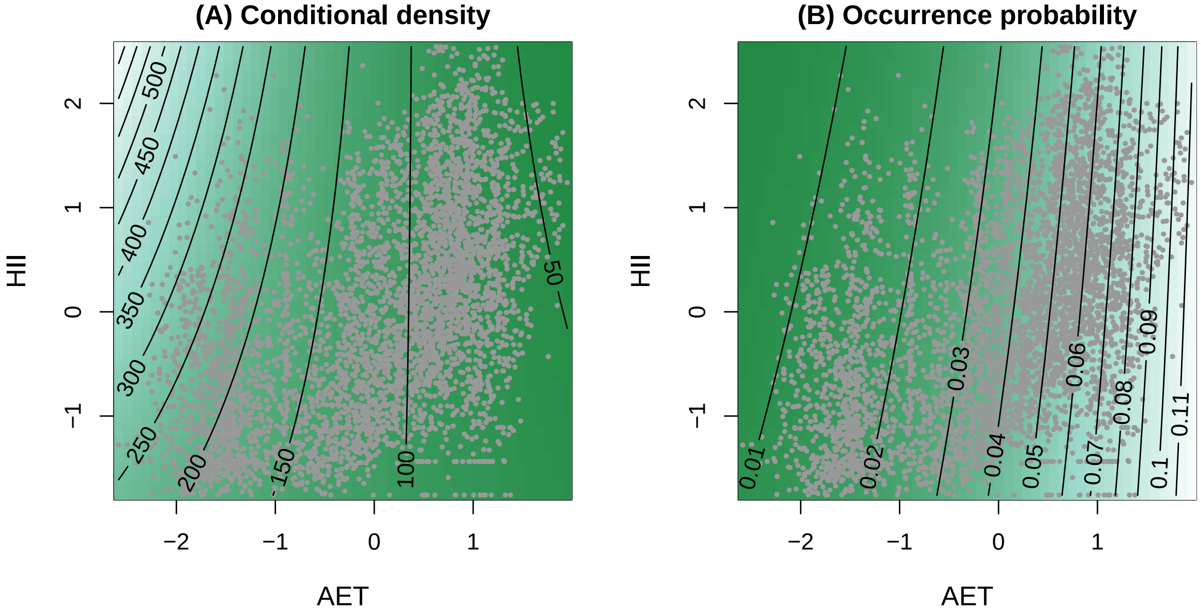

Supplementary Figure 3. **The fitted regression surface of the best model.** The estimated relationship between covariates (AET and HII) and (A) conditional density (d) and (B) occurrence probability (ψ). Gray circles indicate the observed values of these covariates that were scaled to have mean 0 and variance 1. See Supplementary Note 2 for details.

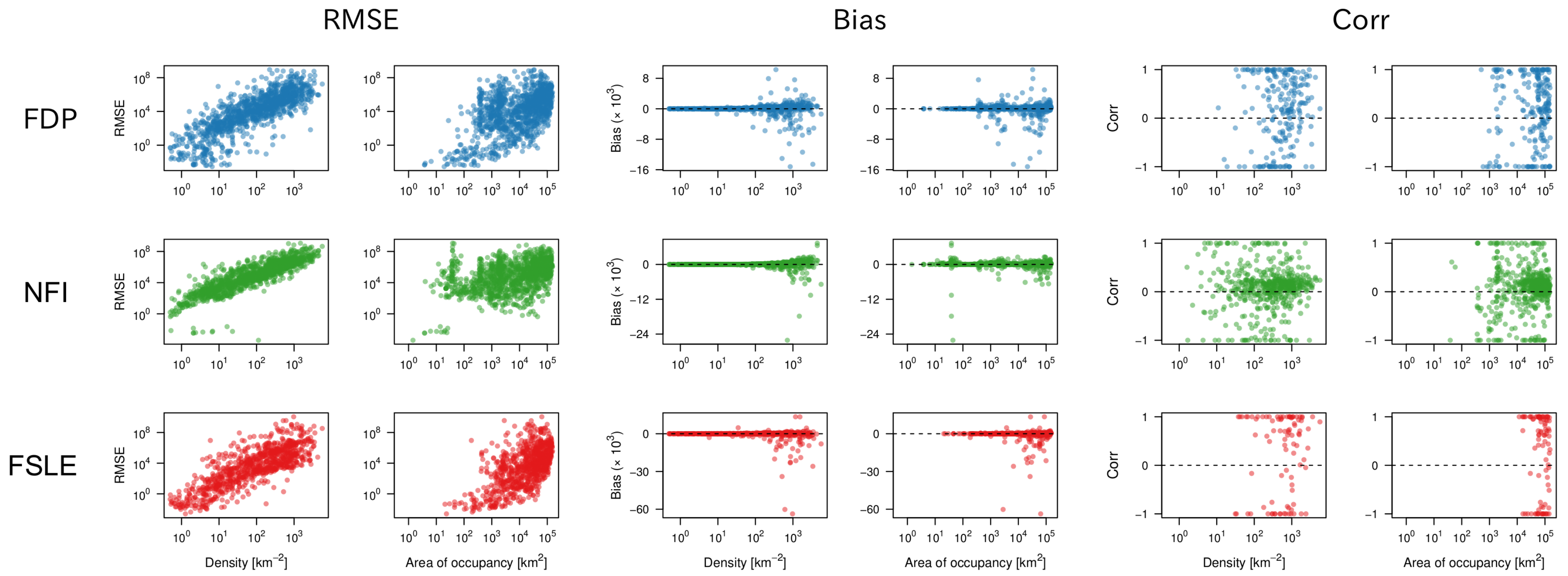

Supplementary Figure 4. **Performance of the best model to predict species-level individual density.** For each validation dataset (FDP, NFI, and FSLE), values of the three predictive benchmarks (RMSE, Bias, and Corr) of each species were plotted against the logarithm of mean conditional density and the logarithm of area of species occupancy. To obtain mean conditional density, density estimates were averaged over grid cells where the species is not absent. See Supplementary Note 2 for more details. Abbreviations: RMSE – Root mean square error of the model prediction for individual density; Bias – Bias of the model prediction for individual density; Corr – Correlation coefficient of the log individual density between model prediction and validation dataset.

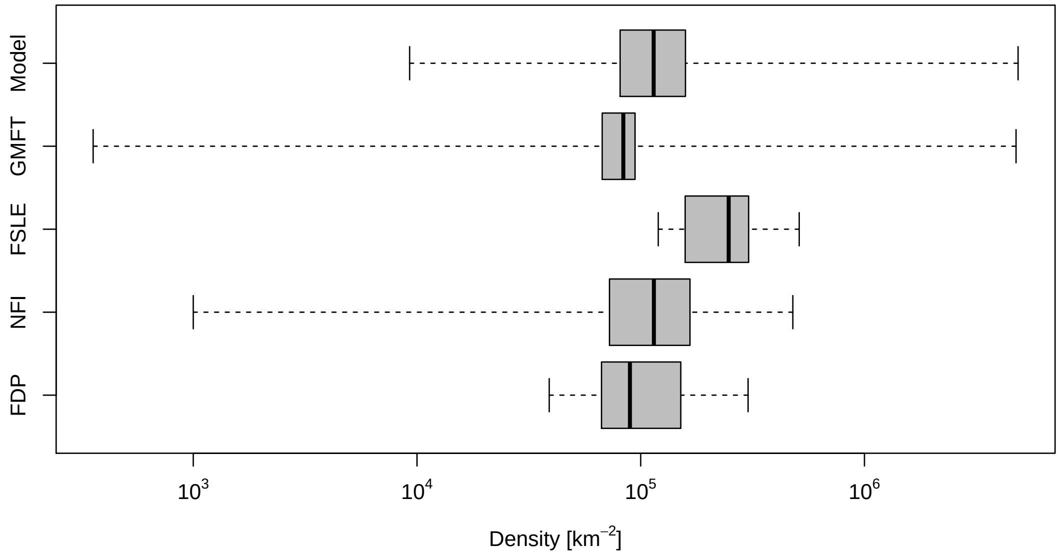

Supplementary Figure 5. **Statistical distribution of the total individual density of woody plants.** The individual density of all species per 1 km^2 natural forest was obtained for each grid cell from the validation datasets (FDP, NFI, FSLE, and GMFT) and the best fit model (Model). Middle line, median; box, first and third quartiles; whiskers, minima and maxima. See Supplementary Note 2 for details. Abbreviations: FDP – Forest dynamics plots; NFI – National forest inventory; FSLE – Forest sampling plots along latitudinal and elevational gradients; GMFT – Global map of forest trees.

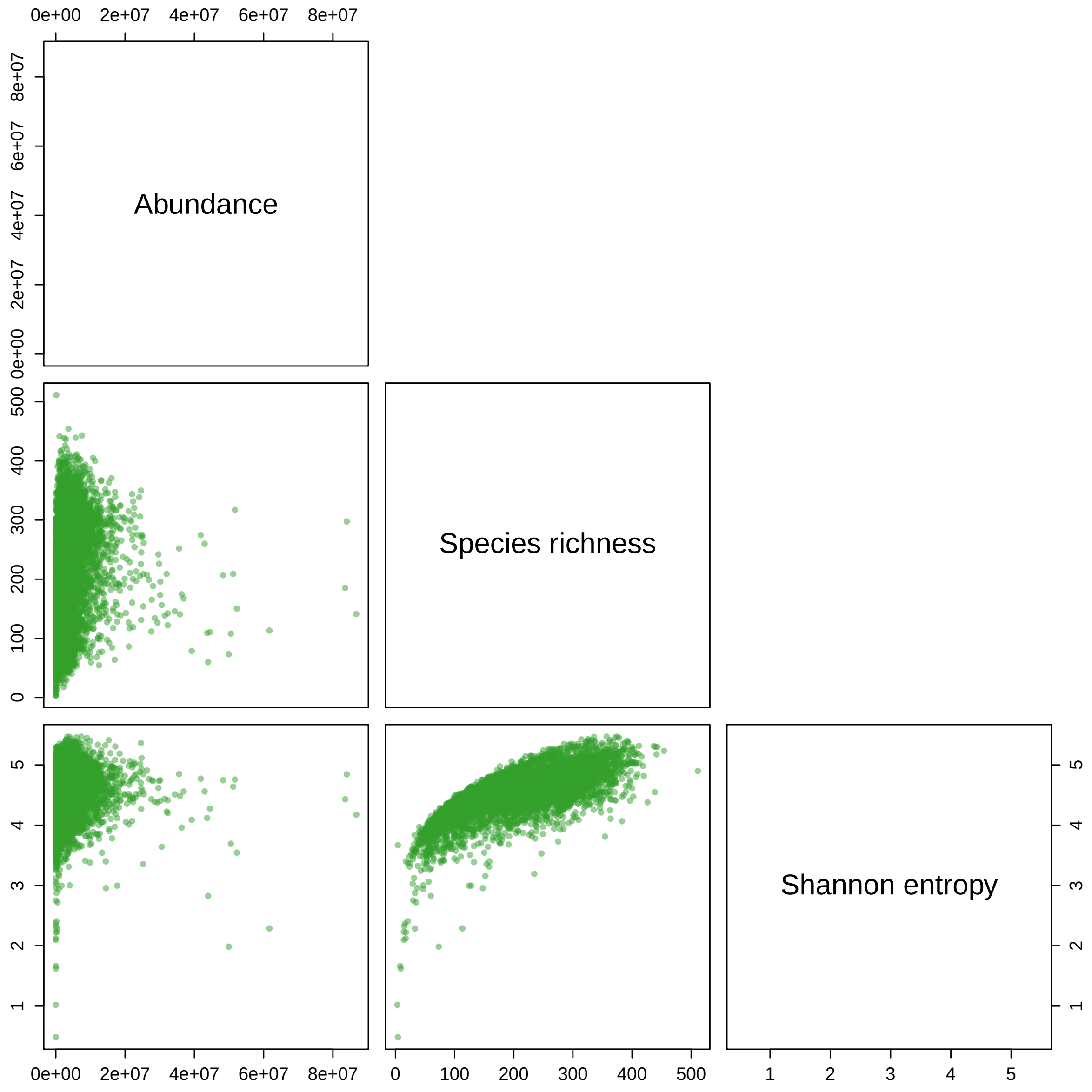

Supplementary Figure 6. **A scatter plot matrix of community properties estimated in 10-km grid cells.** Total number of individuals (abundance), number of species (species richness), and species diversity index (Shannon entropy) were obtained based on the estimates of the fitted model. Fig. 2 in the main text shows the geographic patterns of these variables.

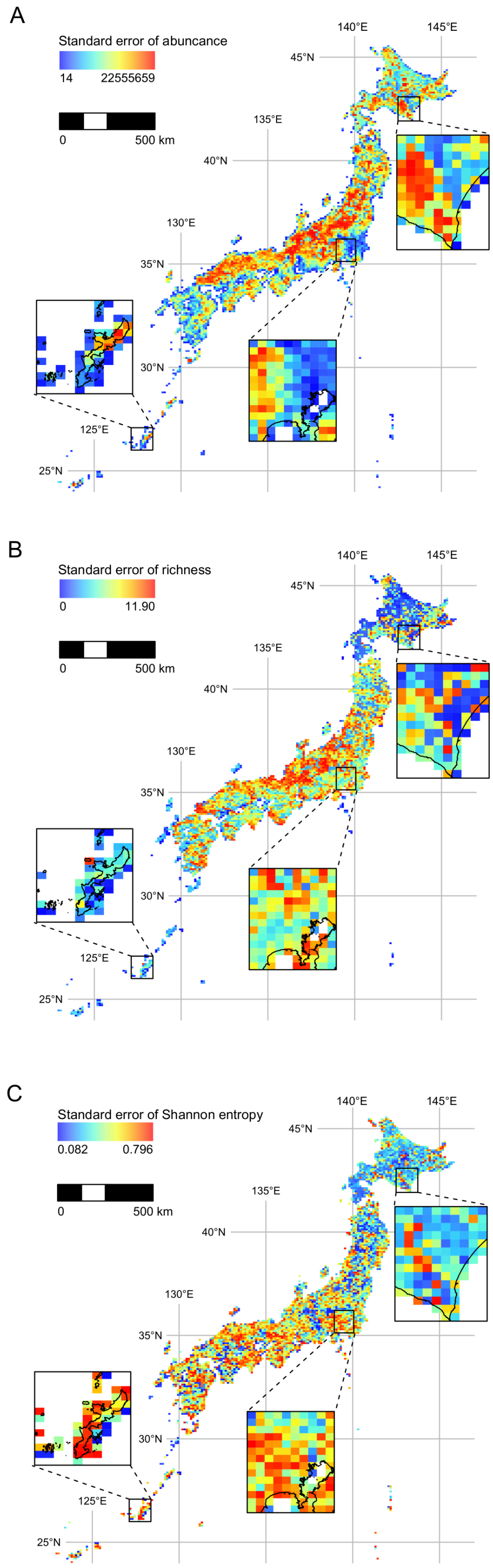

Supplementary Figure 7. **Maps of the standard error of community properties estimated in 10-km grid cells.** (A) total number of individuals (abundance), (B) number of species (species richness) and (C) species diversity index (Shannon entropy). To illustrate finer spatial patterns, three arbitrarily selected sections are enlarged. Fig. 2 in the main text shows the geographic patterns of the estimates.

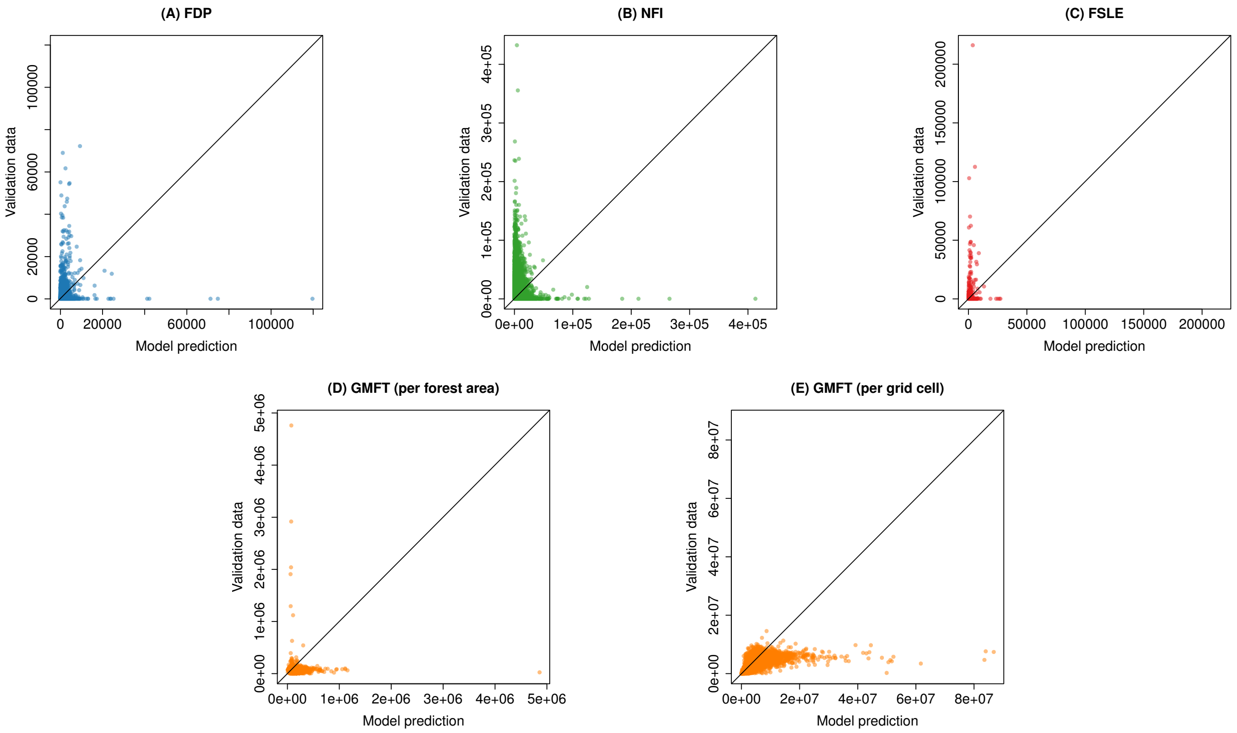

Supplementary Figure 8. **Model prediction of individual density to validation data.** Scatter diagrams of individual density per 1 km^2 natural forest between the model prediction and the four validation datasets (FDP, NFI, FSLE, and GMFT). The diagonal lines are the identity lines. For GMFT, in which individual density was validated at the scale of the 10-km grid cell, a scatter diagram based on individual density per grid cell is also shown in panel E. The relationships are also shown on the logarithmic scale in Fig. 3 in the main text. Abbreviations: FDP – Forest dynamics plots; NFI – National forest inventory; FSLE – Forest sampling plots along latitudinal and elevational gradients; GMFT – Global map of forest trees.

Supplementary Note 4. Maps of the abundance of species with lower extinction risks

We present a geographic map of estimated species abundance, along with a map of the associated standard error, for 1,057 species. The maps of 169 species that are classified in the at-risk categories in the national red list of Japan are not shown for conservation purpose. We also excluded 22 native species that are not registered in the national red list but have an area of occupancy smaller than 100 km^2, which simultaneously indicate rather limited abundance (see Fig. 5 in the main text). The species that meet this criterion are listed below; most of them are endemic to oceanic islands, especially Bonin (Ogasawara) Islands.

- *Callicarpa subpubescens*
- *Distylium lepidotum*
- *Elaeagnus rotundata*
- *Elaeocarpus photiniifolius*
- *Ficus boninsimae*
- *Freycinetia boninensis*
- *Hibiscus glaber*
- *Ligustrum micranthum*
- *Livistona boninensis*
- *Machilus kobu*
- *Melicope quadrilocularis*
- *Myoporum boninense*
- *Neisosperma nakaianum*
- *Neolitsea boninensis*
- *Neolitsea gilva*
- *Osteomeles schwerinae*
- *Pandanus boninensis*
- *Pisonia grandis*
- *Pittosporum boninense**
- *Psychotria boninensis*
- *Rhamnus chugokuensis*
- *Sasa jotanii*

* A variety of the species is categorised as EN.

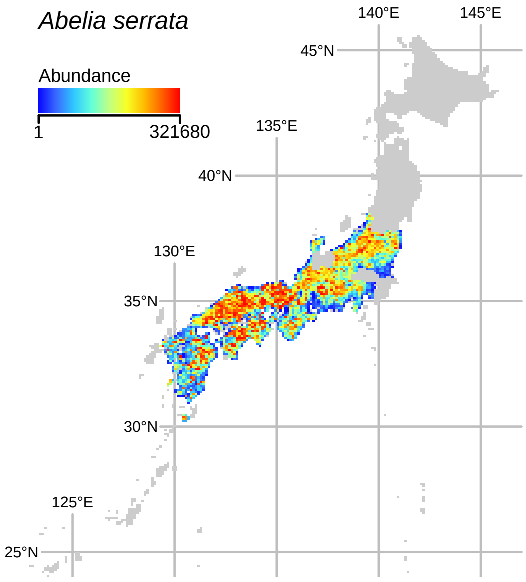

Abelia serrata
Abundance
1
321680
140°E
145°E
45°N
135°E
40°N
130°E
35°N
30°N
125°E
25°N

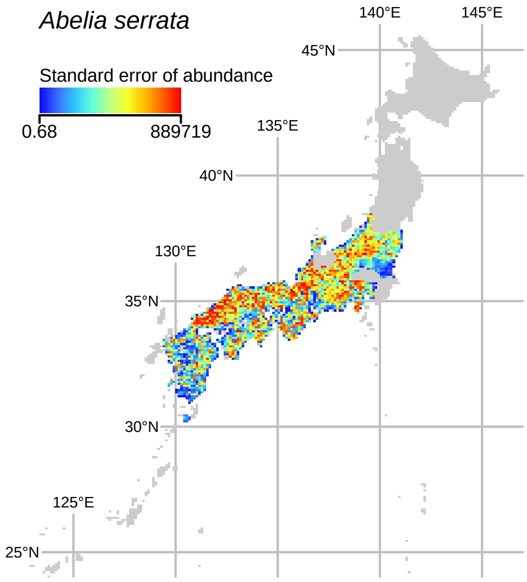

Abelia serrata
Standard error of abundance
0.68
889719
140°E
145°E
45°N
135°E
40°N
130°E
35°N
30°N
125°E
25°N

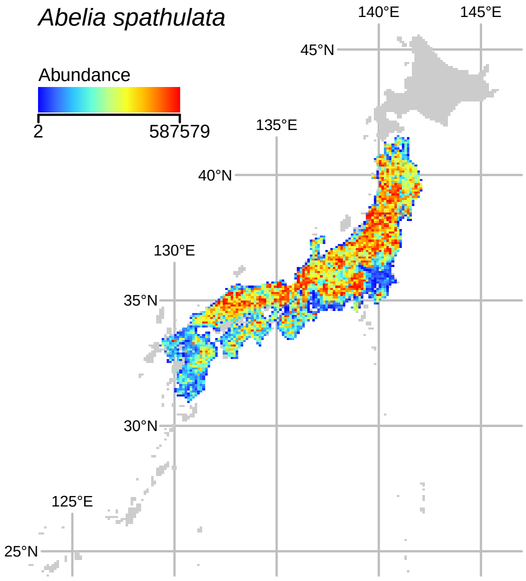

Abelia spathulata
Abundance
2
587579
140°E
145°E
45°N
135°E
40°N
130°E
35°N
30°N
125°E
25°N

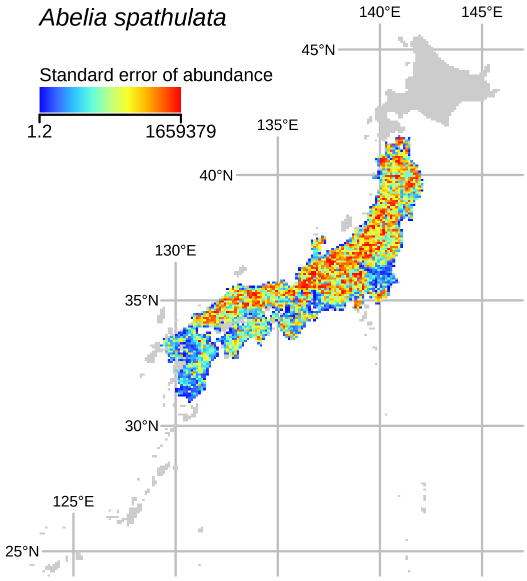

Abelia spathulata
Standard error of abundance
1.2
1659379
140°E
145°E
45°N
135°E
40°N
130°E
35°N
30°N
125°E
25°N

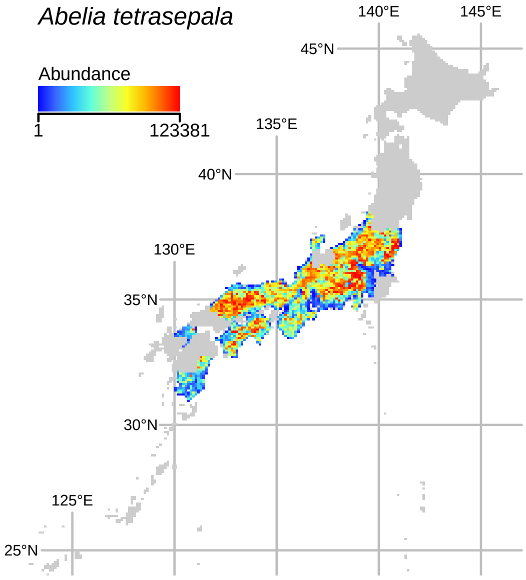

Abelia tetrasepala
Abundance
1
123381
140°E
145°E
45°N
135°E
40°N
130°E
35°N
30°N
125°E
25°N

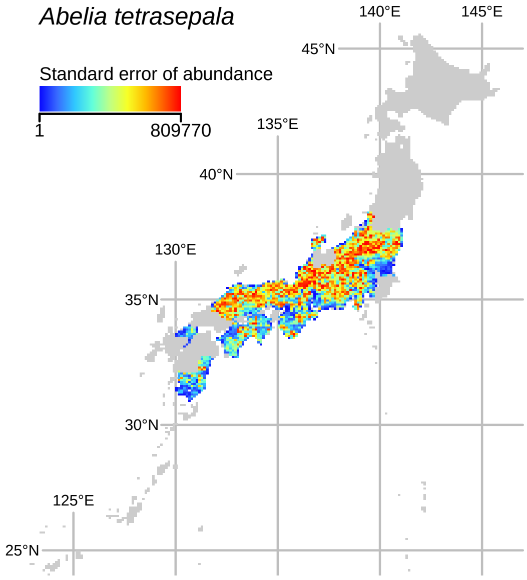

Abelia tetrasepala
Standard error of abundance
1
809770
140°E
145°E
45°N
135°E
40°N
130°E
35°N
30°N
125°E
25°N

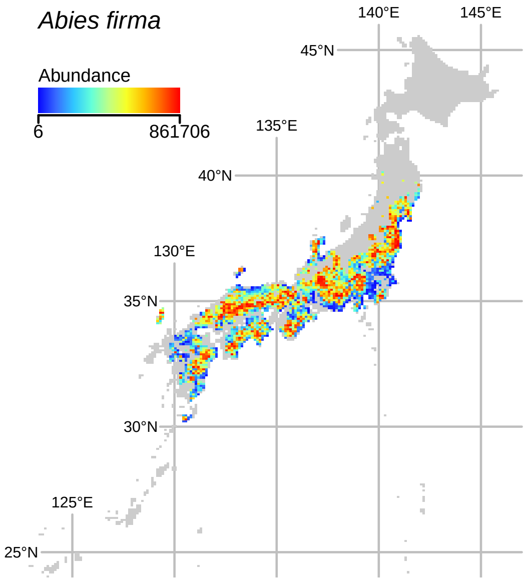
Abies firma
Abundance
6
861706
140°E
145°E
45°N
135°E
40°N
130°E
35°N
30°N
125°E
25°N

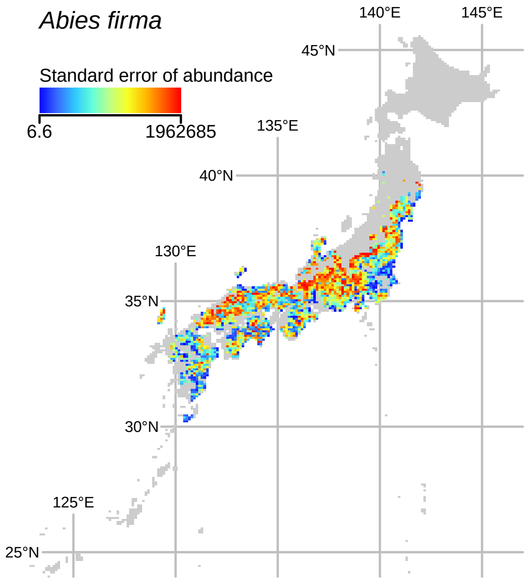
Abies firma
Standard error of abundance
6.6
1962685
140°E
145°E
45°N
135°E
40°N
130°E
35°N
30°N
125°E
25°N

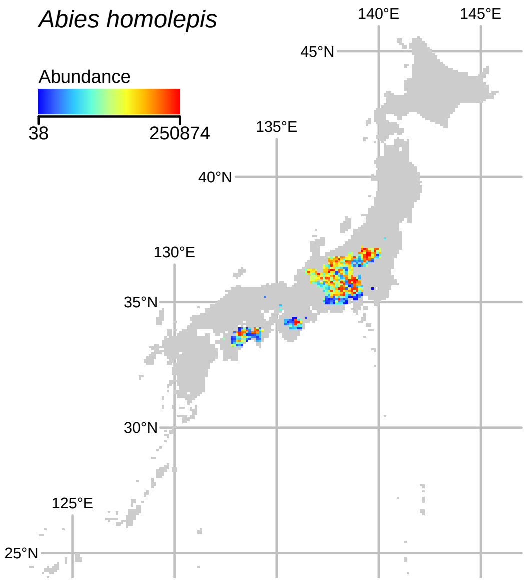
Abies homolepis
Abundance
38
250874
140°E
145°E
45°N
135°E
40°N
130°E
35°N
30°N
125°E
25°N

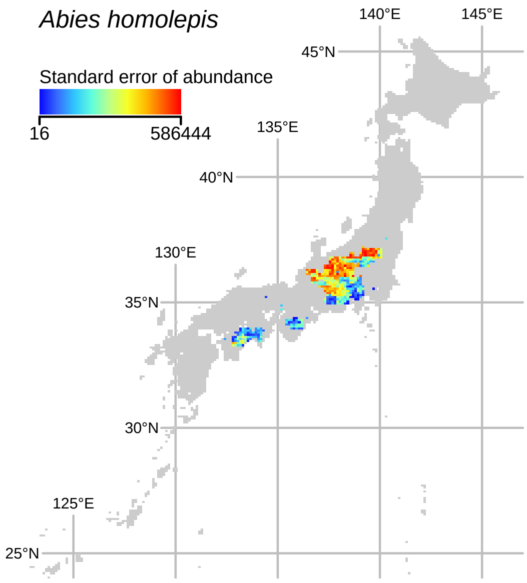
Abies homolepis
Standard error of abundance
16
586444
140°E
145°E
45°N
135°E
40°N
130°E
35°N
30°N
125°E
25°N

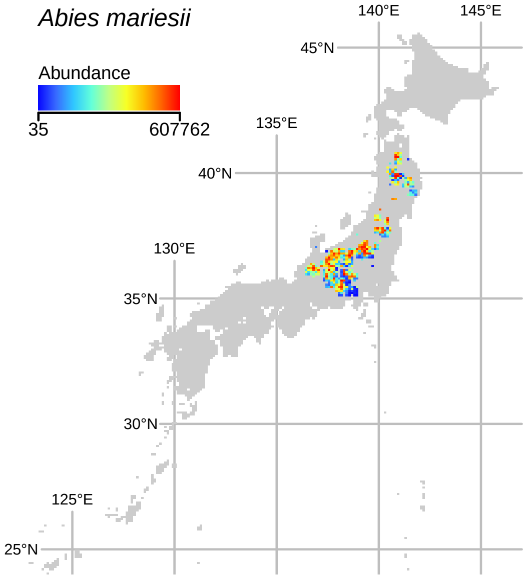
Abies mariesii
Abundance
35
607762
140°E
145°E
45°N
135°E
40°N
130°E
35°N
30°N
125°E
25°N

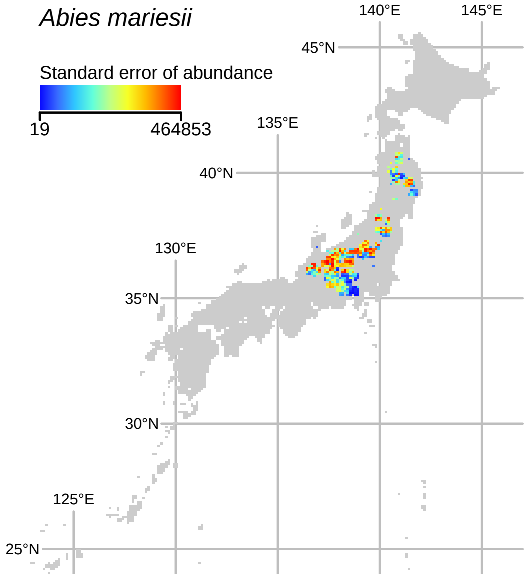
Abies mariesii
Standard error of abundance
19
464853
140°E
145°E
45°N
135°E
40°N
130°E
35°N
30°N
125°E
25°N

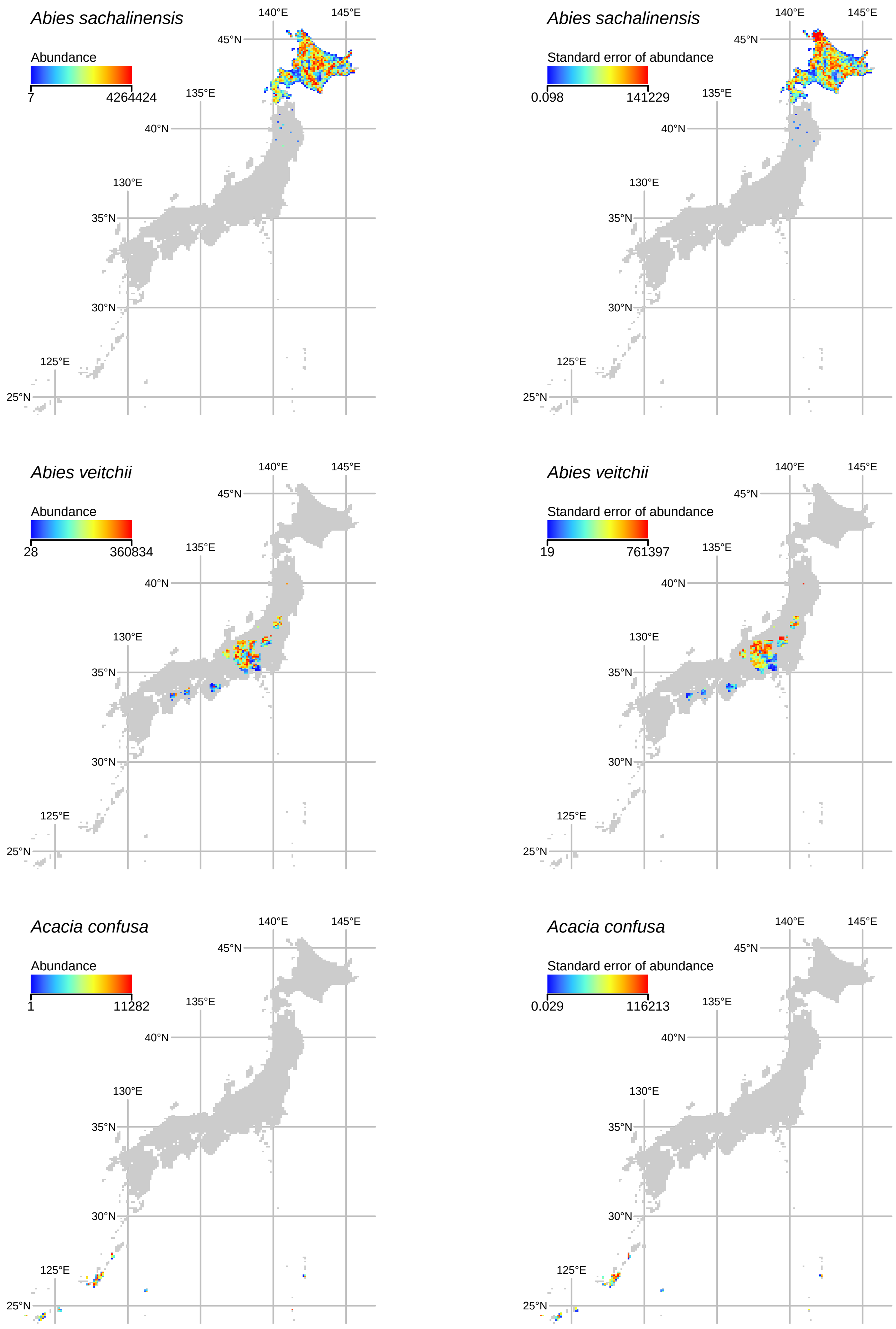
Abies sachalinensis
Abundance
7
4264424
Abies sachalinensis
Standard error of abundance
0.098
141229
Abies veitchii
Abundance
28
360834
Abies veitchii
Standard error of abundance
19
761397
Acacia confusa
Abundance
1
11282
Acacia confusa
Standard error of abundance
0.029
116213
125°E
130°E
135°E
140°E
145°E
25°N
30°N
35°N
40°N
45°N

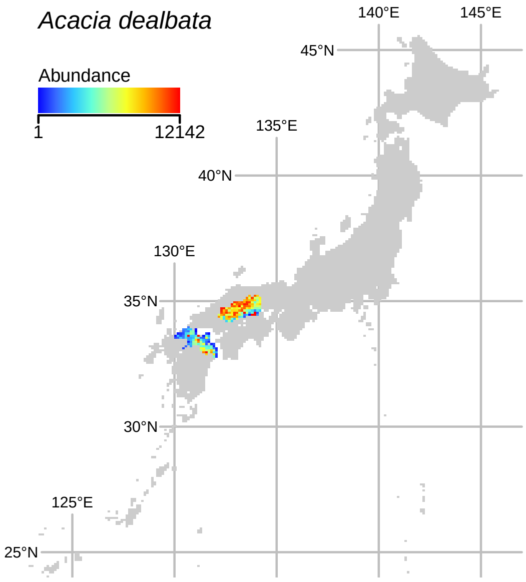
Acacia dealbata
Abundance
1
12142
140°E
145°E
45°N
135°E
40°N
130°E
35°N
30°N
125°E
25°N

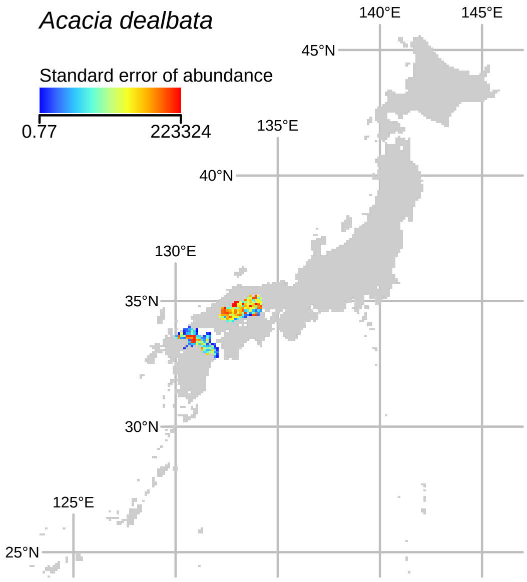
Acacia dealbata
Standard error of abundance
0.77
223324
140°E
145°E
45°N
135°E
40°N
130°E
35°N
30°N
125°E
25°N

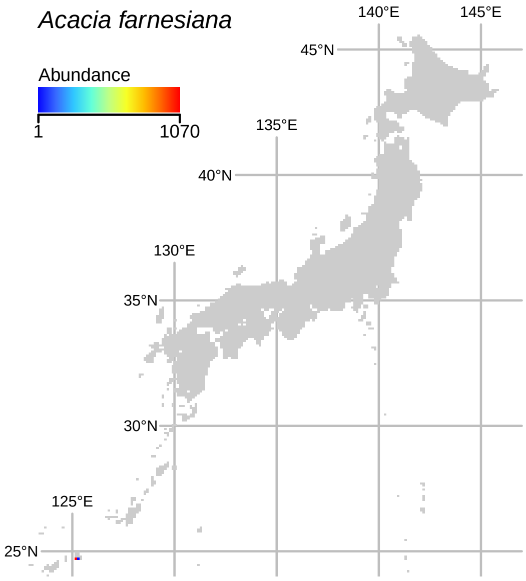
Acacia farnesiana
Abundance
1
1070
140°E
145°E
45°N
135°E
40°N
130°E
35°N
30°N
125°E
25°N

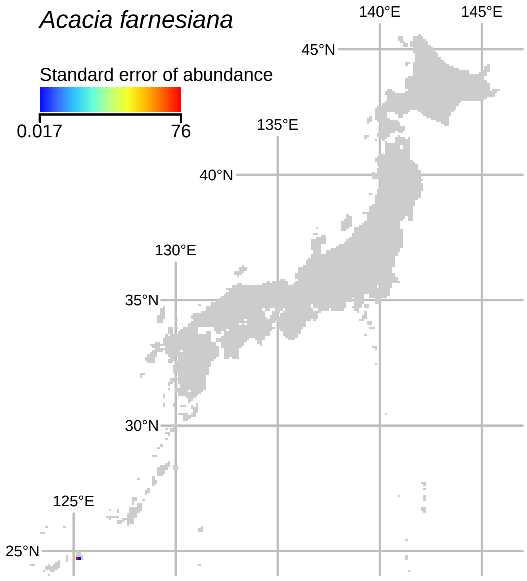
Acacia farnesiana
Standard error of abundance
0.017
76
140°E
145°E
45°N
135°E
40°N
130°E
35°N
30°N
125°E
25°N

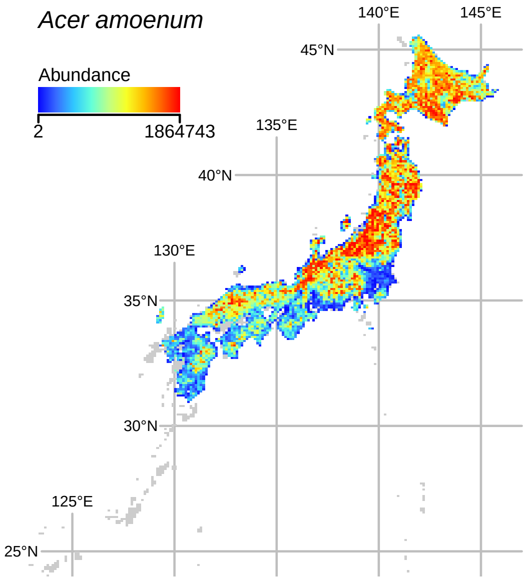
Acer amoenum
Abundance
2
1864743
140°E
145°E
45°N
135°E
40°N
130°E
35°N
30°N
125°E
25°N

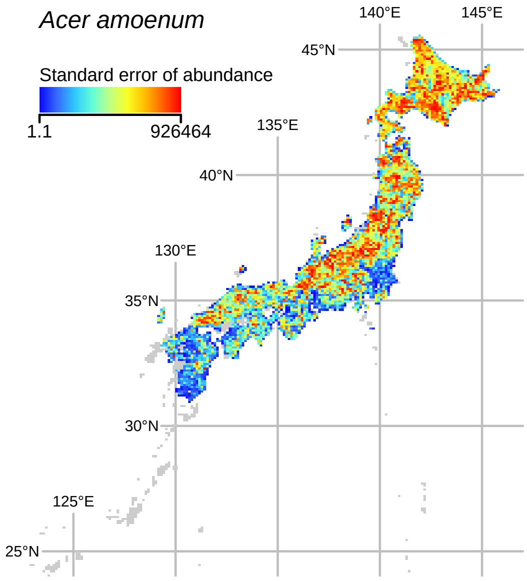
Acer amoenum
Standard error of abundance
1.1
926464
140°E
145°E
45°N
135°E
40°N
130°E
35°N
30°N
125°E
25°N

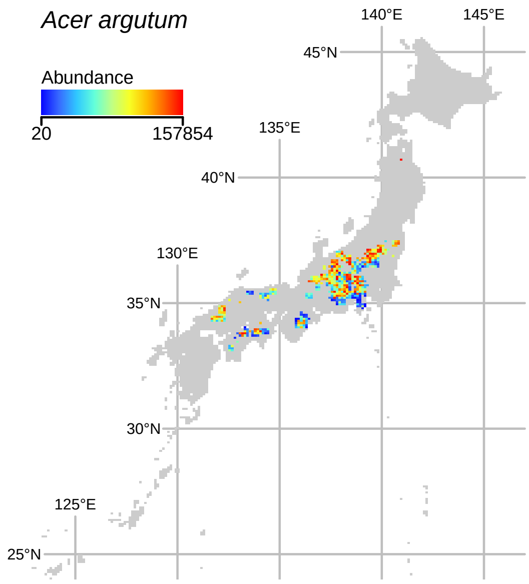
Acer argutum
Abundance
20
157854
140°E
145°E
45°N
135°E
40°N
130°E
35°N
30°N
125°E
25°N

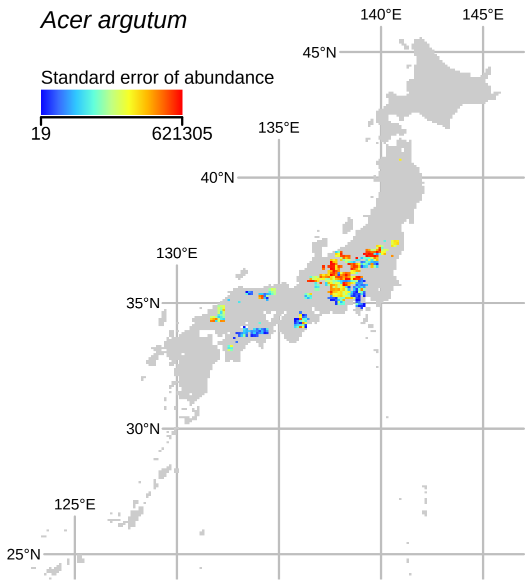
Acer argutum
Standard error of abundance
19
621305
140°E
145°E
45°N
135°E
40°N
130°E
35°N
30°N
125°E
25°N

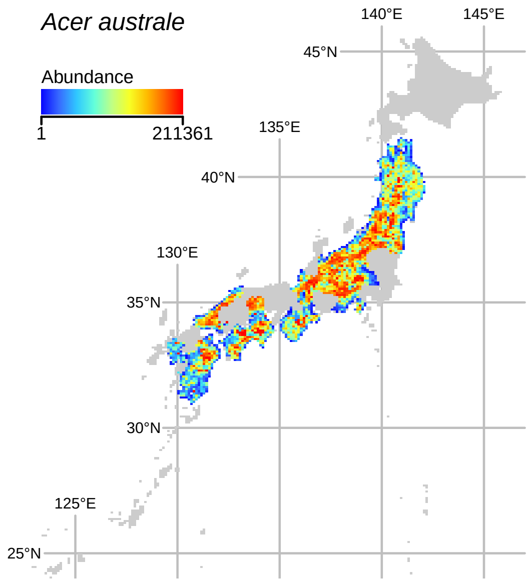
Acer australe
Abundance
1
211361
140°E
145°E
45°N
135°E
40°N
130°E
35°N
30°N
125°E
25°N

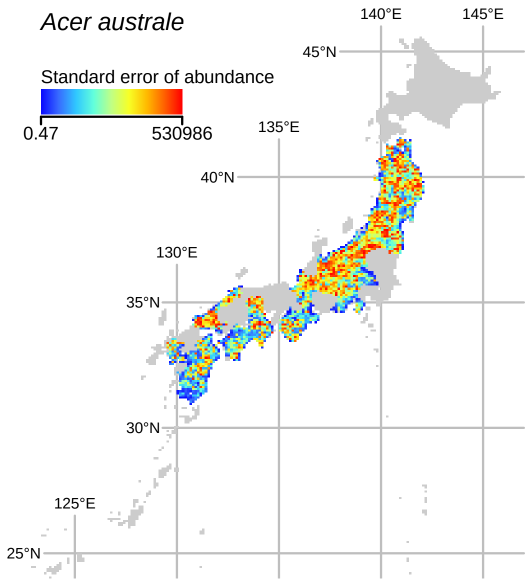
Acer australe
Standard error of abundance
0.47
530986
140°E
145°E
45°N
135°E
40°N
130°E
35°N
30°N
125°E
25°N

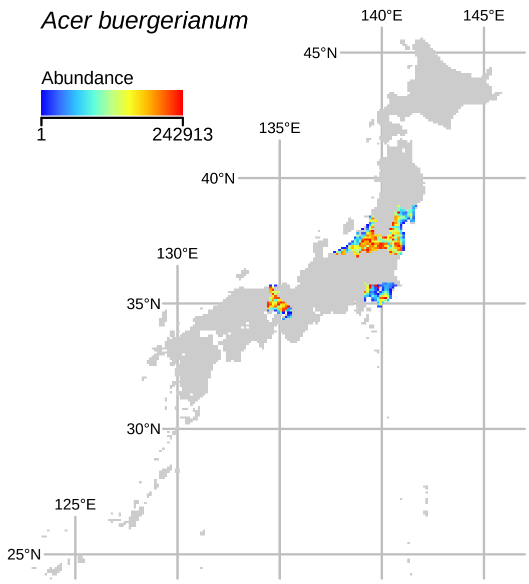
Acer buergerianum
Abundance
1
242913
140°E
145°E
45°N
135°E
40°N
130°E
35°N
30°N
125°E
25°N

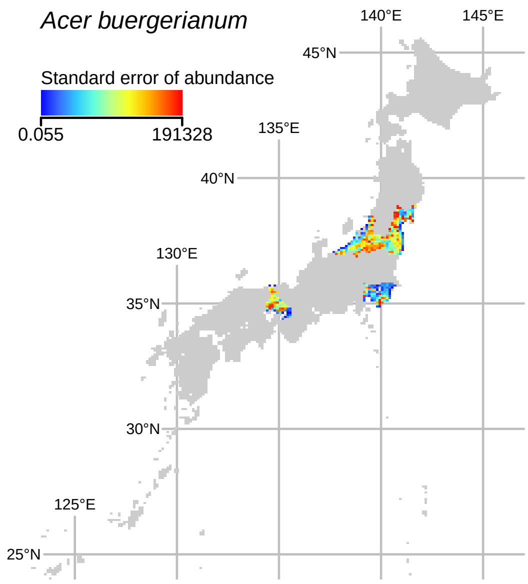
Acer buergerianum
Standard error of abundance
0.055
191328
140°E
145°E
45°N
135°E
40°N
130°E
35°N
30°N
125°E
25°N

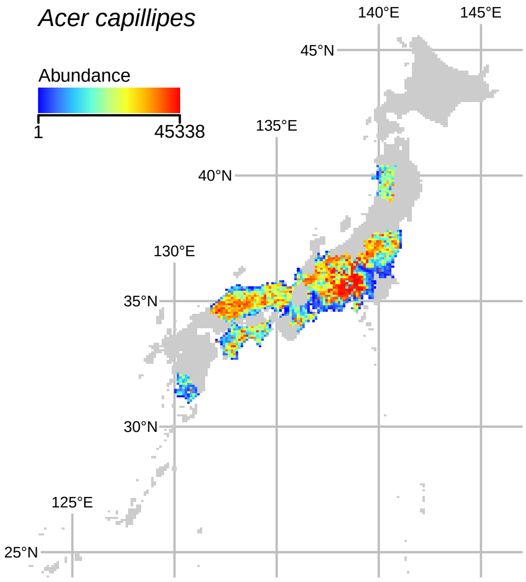
Acer capillipes
Abundance
1
45338
140°E
145°E
135°E
130°E
125°E
45°N
40°N
35°N
30°N
25°N

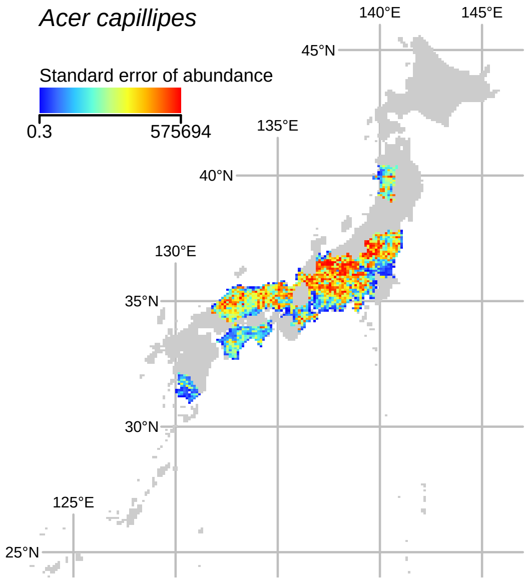
Acer capillipes
Standard error of abundance
0.3
575694
140°E
145°E
135°E
130°E
125°E
45°N
40°N
35°N
30°N
25°N

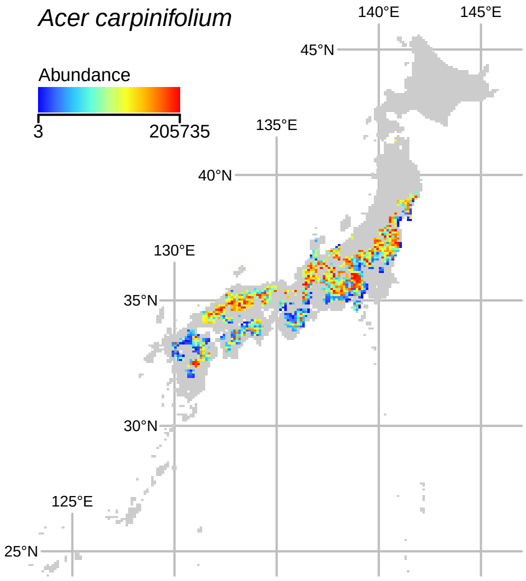
Acer carpinifolium
Abundance
3
205735
140°E
145°E
135°E
130°E
125°E
45°N
40°N
35°N
30°N
25°N

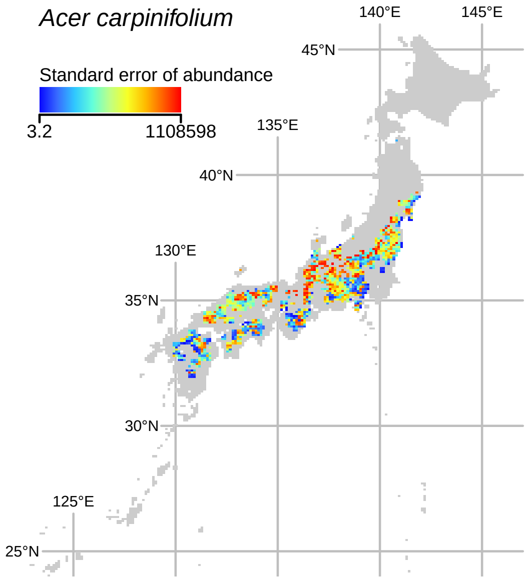
Acer carpinifolium
Standard error of abundance
3.2
1108598
140°E
145°E
135°E
130°E
125°E
45°N
40°N
35°N
30°N
25°N

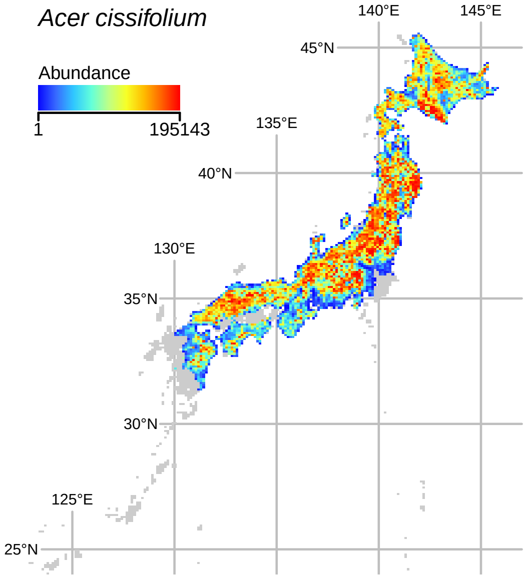
Acer cissifolium
Abundance
1
195143
140°E
145°E
135°E
130°E
125°E
45°N
40°N
35°N
30°N
25°N

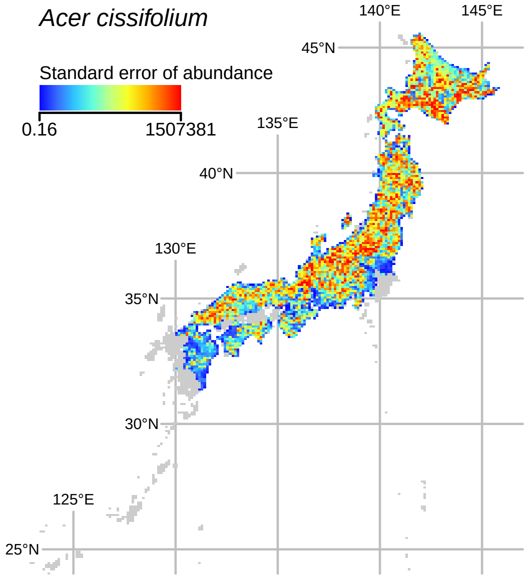
Acer cissifolium
Standard error of abundance
0.16
1507381
140°E
145°E
135°E
130°E
125°E
45°N
40°N
35°N
30°N
25°N

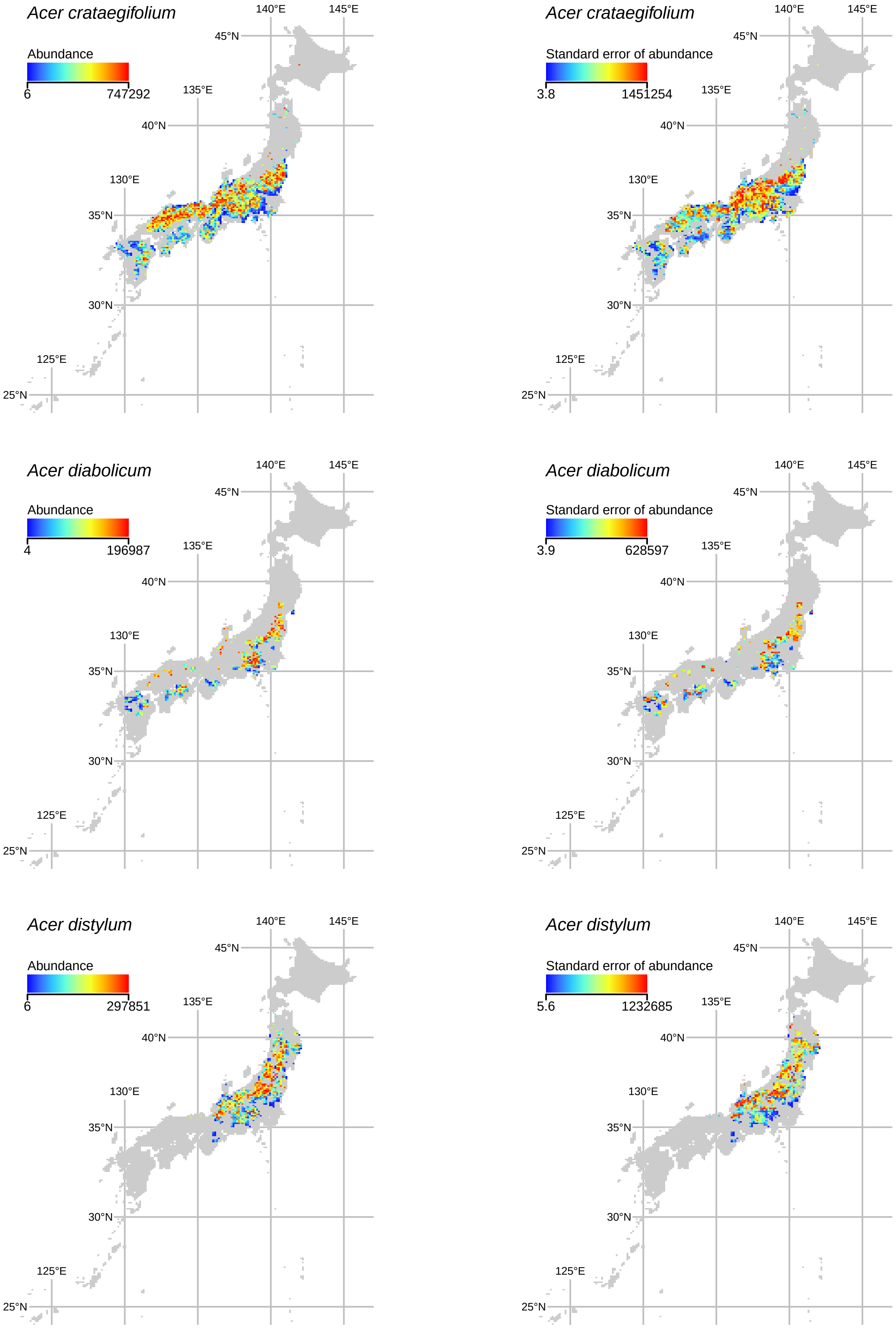

Acer crataegifolium
Abundance
6
747292
Acer crataegifolium
Standard error of abundance
3.8
1451254
Acer diabolicum
Abundance
4
196987
Acer diabolicum
Standard error of abundance
3.9
628597
Acer distylum
Abundance
6
297851
Acer distylum
Standard error of abundance
5.6
1232685
125°E
130°E
135°E
140°E
145°E
25°N
30°N
35°N
40°N
45°N

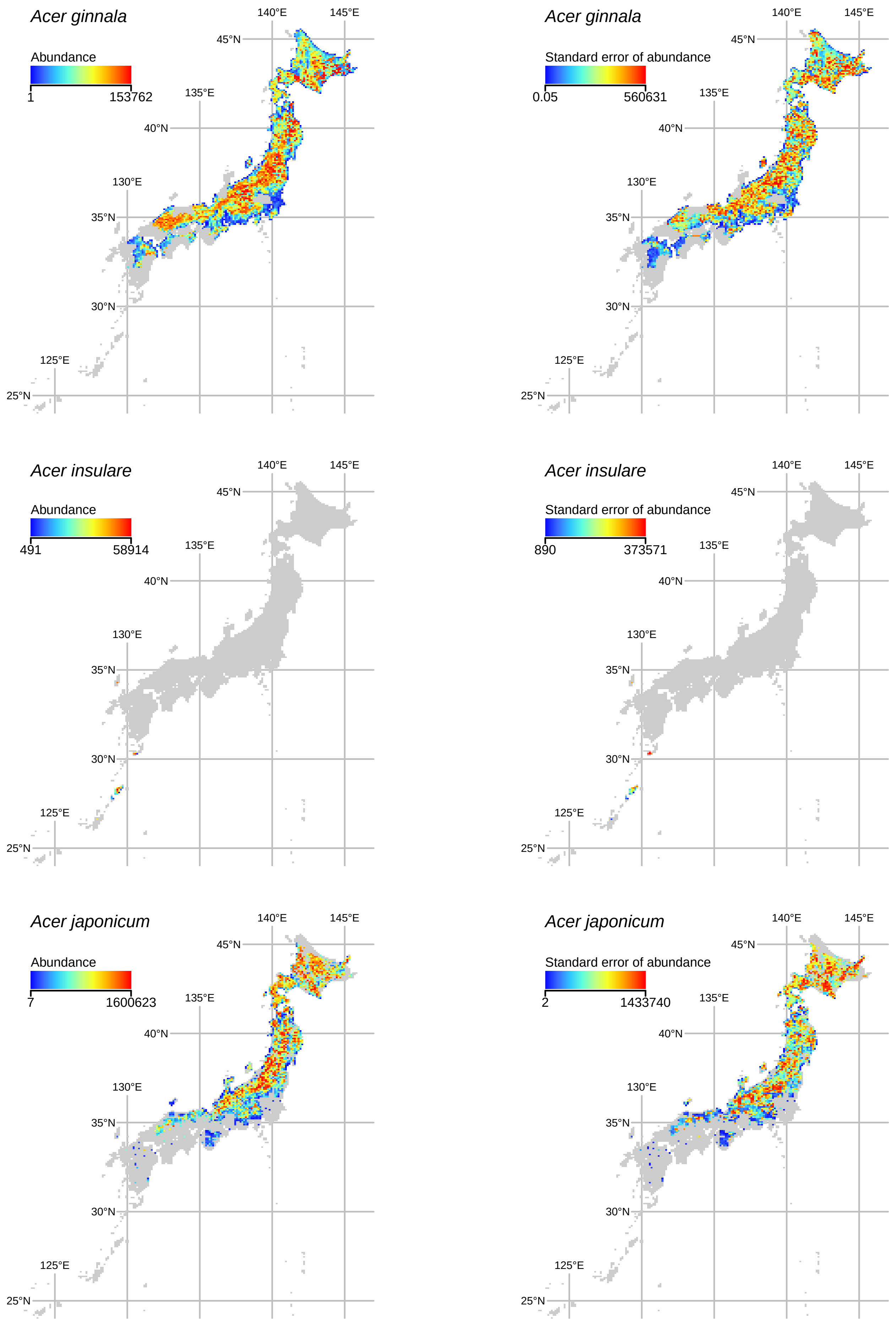
Acer ginnala
Abundance
1
153762
Acer ginnala
Standard error of abundance
0.05
560631
Acer insulare
Abundance
491
58914
Acer insulare
Standard error of abundance
890
373571
Acer japonicum
Abundance
7
1600623
Acer japonicum
Standard error of abundance
2
1433740
125°E
130°E
135°E
140°E
145°E
25°N
30°N
35°N
40°N
45°N

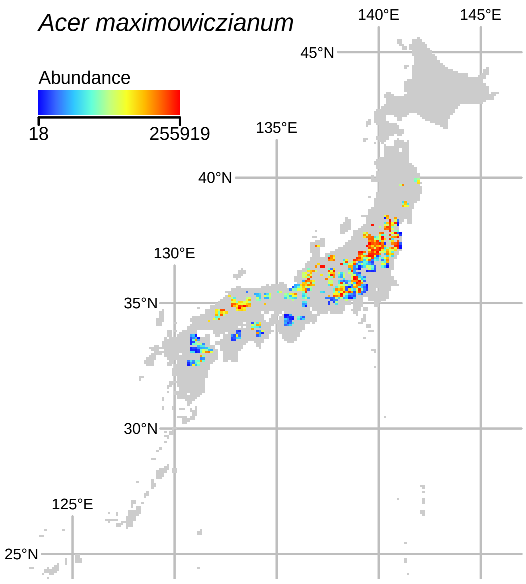
Acer maximowiczianum
Abundance
18
255919
140°E
145°E
45°N
135°E
40°N
130°E
35°N
30°N
125°E
25°N

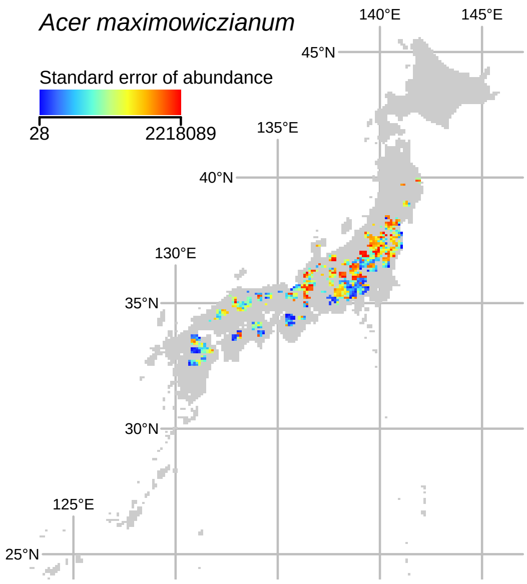
Acer maximowiczianum
Standard error of abundance
28
2218089
140°E
145°E
45°N
135°E
40°N
130°E
35°N
30°N
125°E
25°N

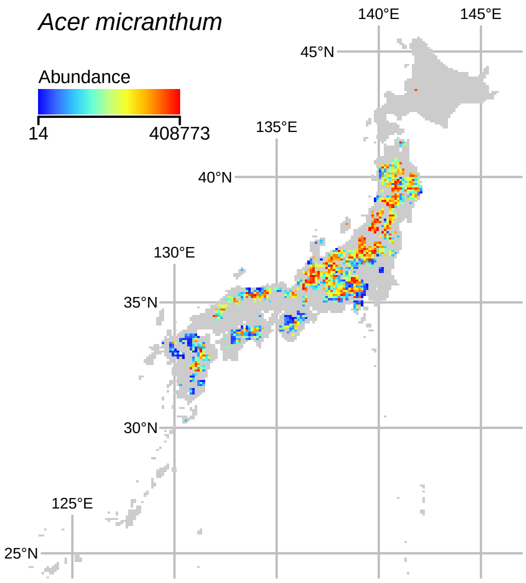
Acer micranthum
Abundance
14
408773
140°E
145°E
45°N
135°E
40°N
130°E
35°N
30°N
125°E
25°N

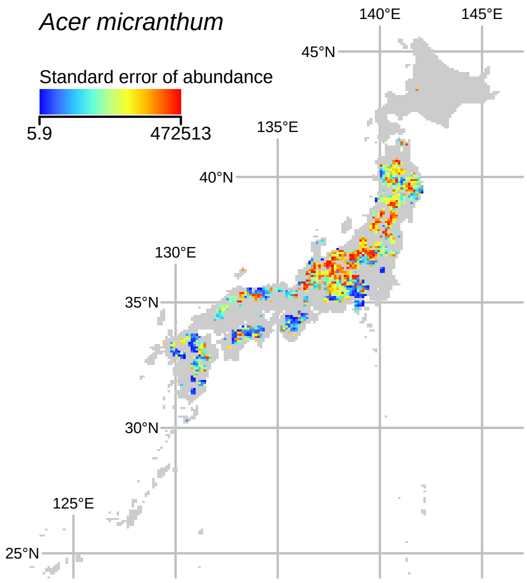
Acer micranthum
Standard error of abundance
5.9
472513
140°E
145°E
45°N
135°E
40°N
130°E
35°N
30°N
125°E
25°N

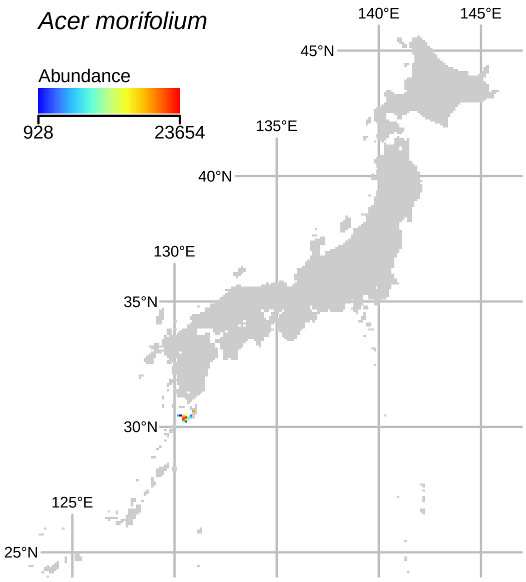
Acer morifolium
Abundance
928
23654
140°E
145°E
45°N
135°E
40°N
130°E
35°N
30°N
125°E
25°N

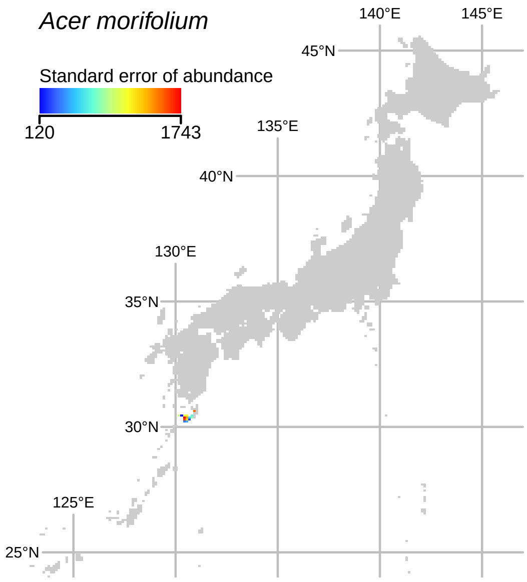
Acer morifolium
Standard error of abundance
120
1743
140°E
145°E
45°N
135°E
40°N
130°E
35°N
30°N
125°E
25°N

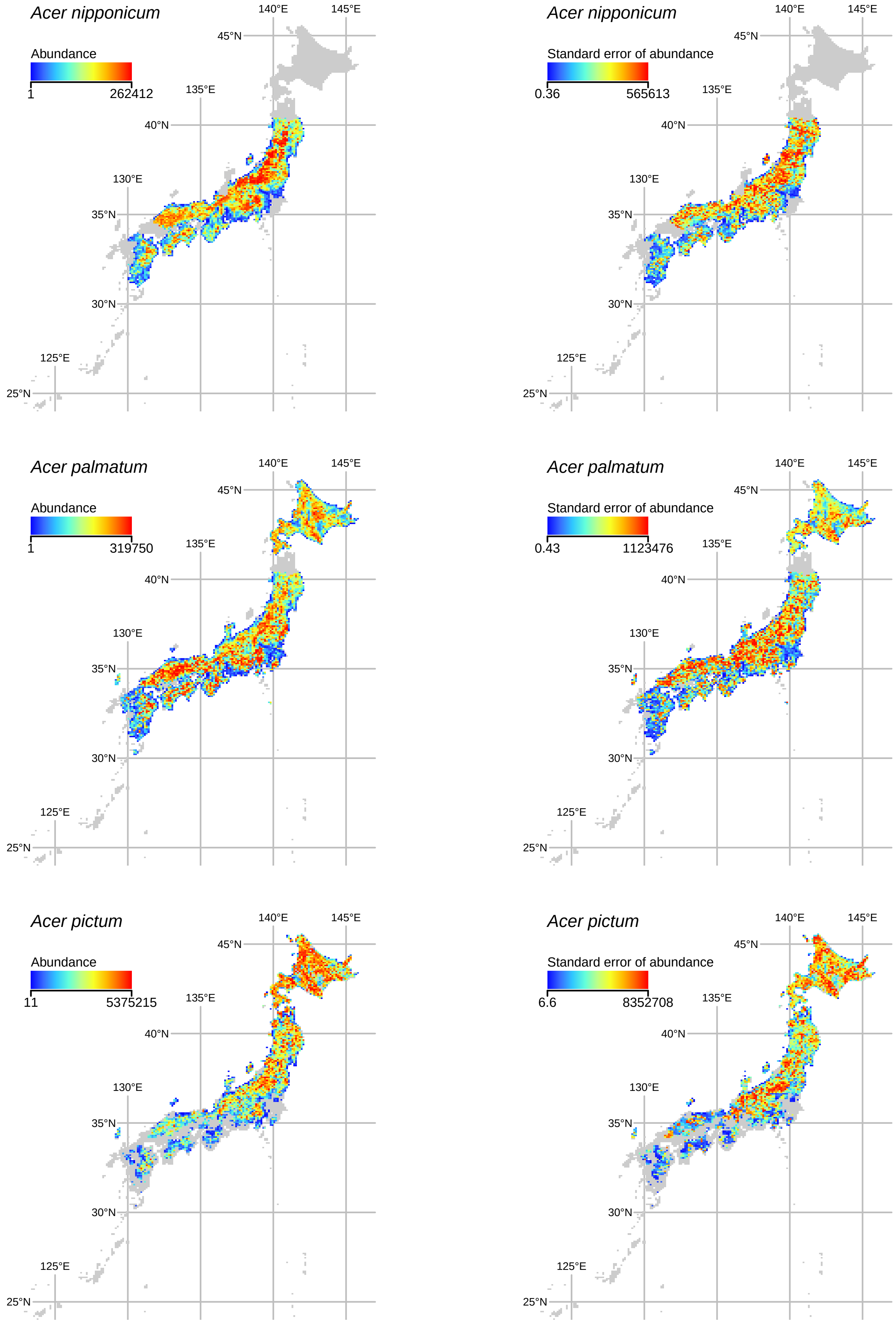
Acer nipponicum
Abundance
1
262412
Acer nipponicum
Standard error of abundance
0.36
565613
Acer palmatum
Abundance
1
319750
Acer palmatum
Standard error of abundance
0.43
1123476
Acer pictum
Abundance
11
5375215
Acer pictum
Standard error of abundance
6.6
8352708
125°E
130°E
135°E
140°E
145°E
25°N
30°N
35°N
40°N
45°N

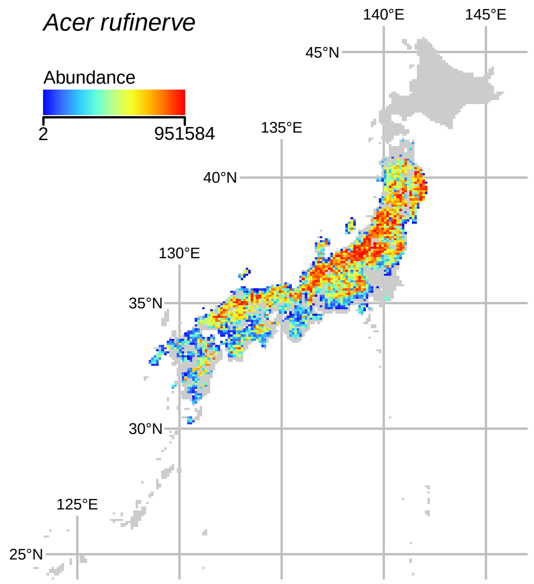
Acer rufinerve
Abundance
2
951584
140°E
145°E
45°N
135°E
40°N
130°E
35°N
30°N
125°E
25°N

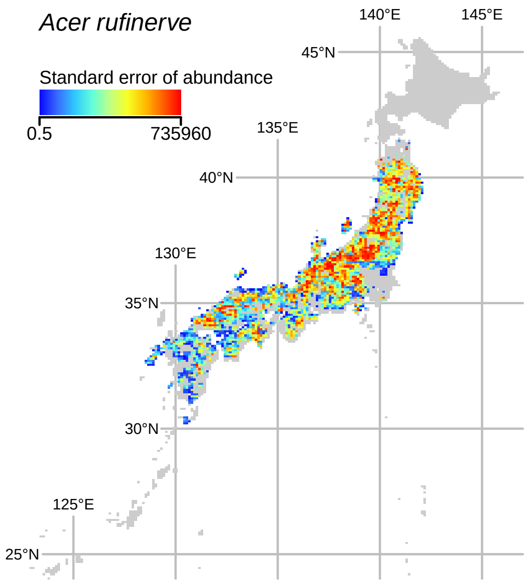
Acer rufinerve
Standard error of abundance
0.5
735960
140°E
145°E
45°N
135°E
40°N
130°E
35°N
30°N
125°E
25°N

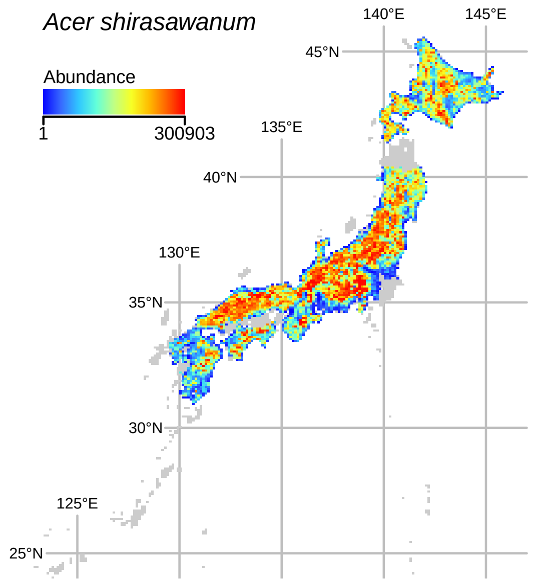
Acer shirasawanum
Abundance
1
300903
140°E
145°E
45°N
135°E
40°N
130°E
35°N
30°N
125°E
25°N

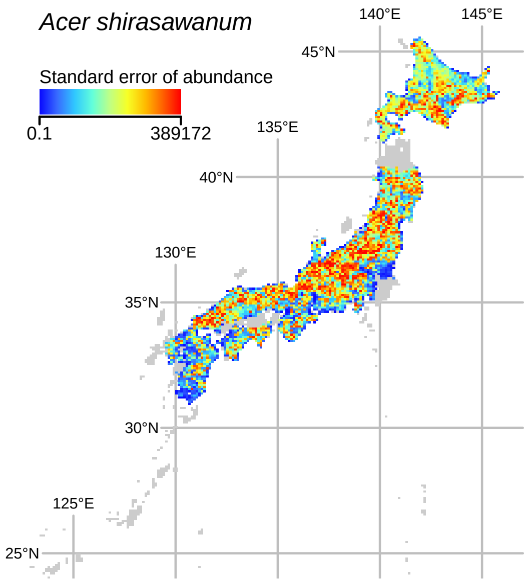
Acer shirasawanum
Standard error of abundance
0.1
389172
140°E
145°E
45°N
135°E
40°N
130°E
35°N
30°N
125°E
25°N

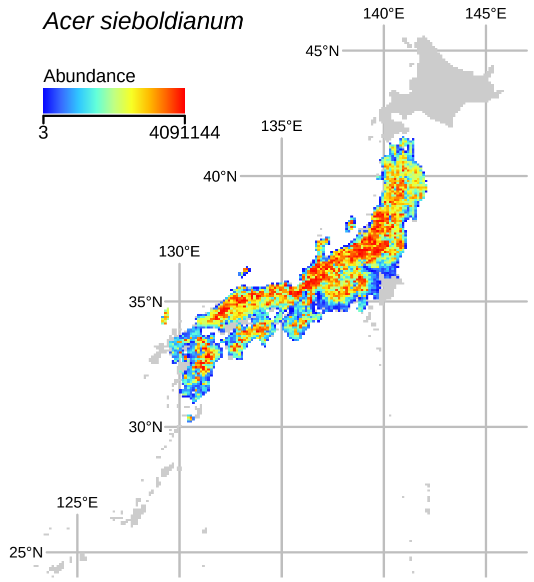
Acer sieboldianum
Abundance
3
4091144
140°E
145°E
45°N
135°E
40°N
130°E
35°N
30°N
125°E
25°N

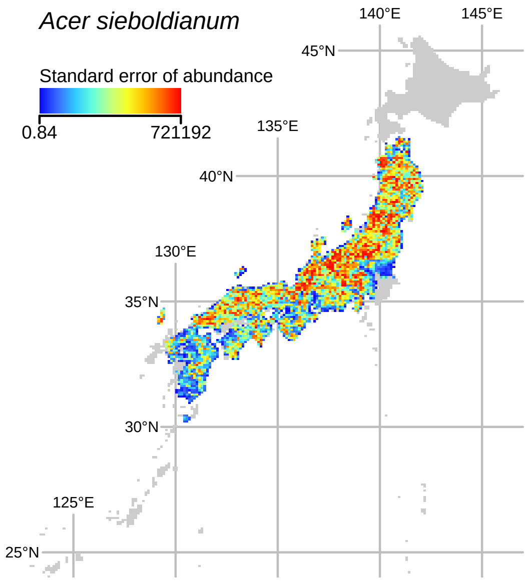
Acer sieboldianum
Standard error of abundance
0.84
721192
140°E
145°E
45°N
135°E
40°N
130°E
35°N
30°N
125°E
25°N

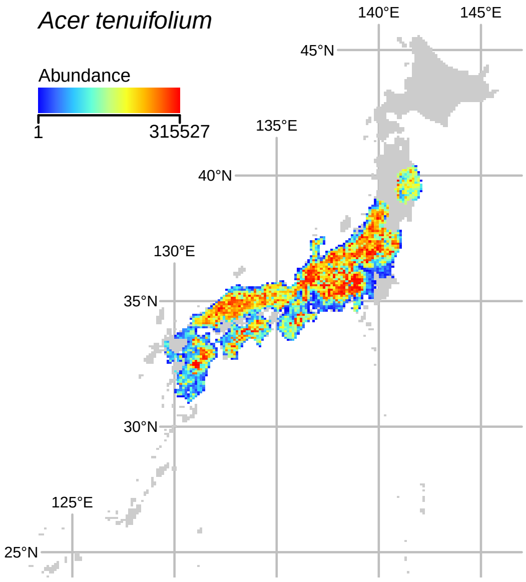
Acer tenuifolium
Abundance
1
315527
140°E
145°E
45°N
135°E
40°N
130°E
35°N
30°N
125°E
25°N

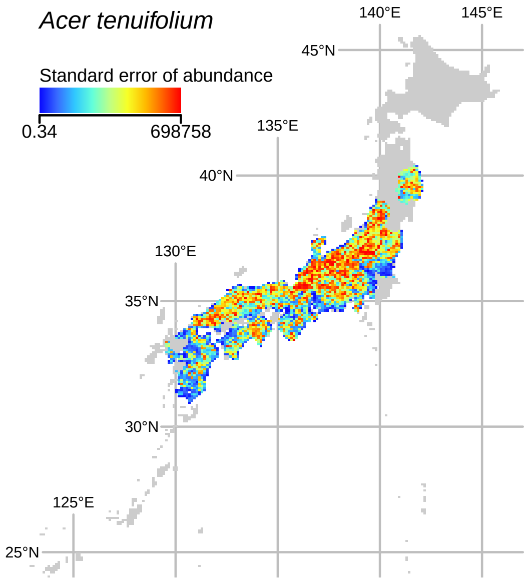
Acer tenuifolium
Standard error of abundance
0.34
698758
140°E
145°E
45°N
135°E
40°N
130°E
35°N
30°N
125°E
25°N

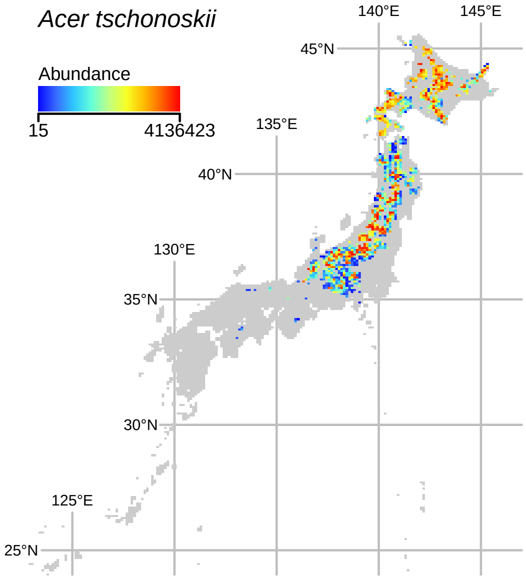
Acer tschonoskii
Abundance
15
4136423
140°E
145°E
45°N
135°E
40°N
130°E
35°N
30°N
125°E
25°N

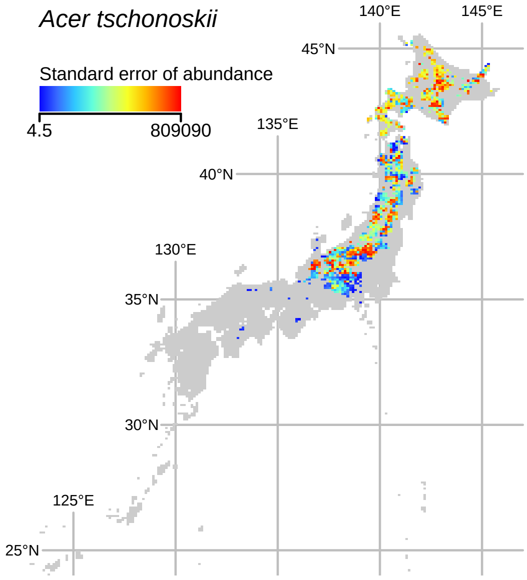
Acer tschonoskii
Standard error of abundance
4.5
809090
140°E
145°E
45°N
135°E
40°N
130°E
35°N
30°N
125°E
25°N

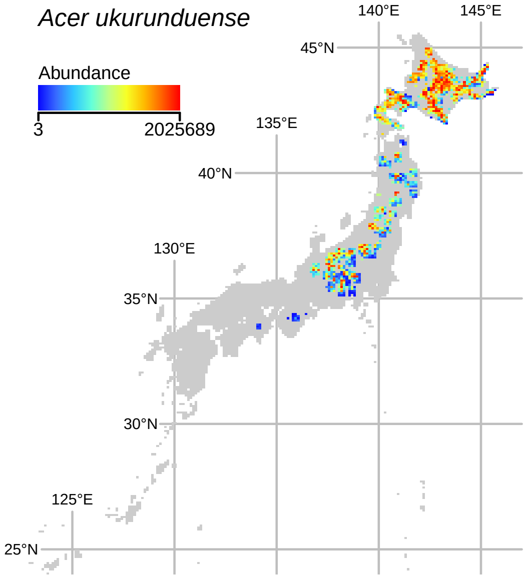
Acer ukurunduense
Abundance
3
2025689
140°E
145°E
45°N
135°E
40°N
130°E
35°N
30°N
125°E
25°N

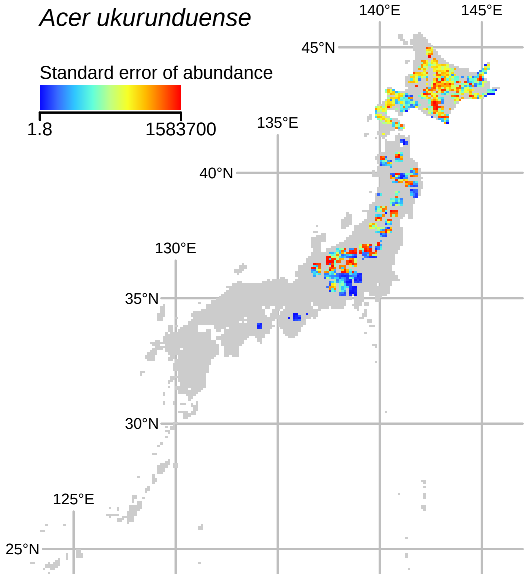
Acer ukurunduense
Standard error of abundance
1.8
1583700
140°E
145°E
45°N
135°E
40°N
130°E
35°N
30°N
125°E
25°N

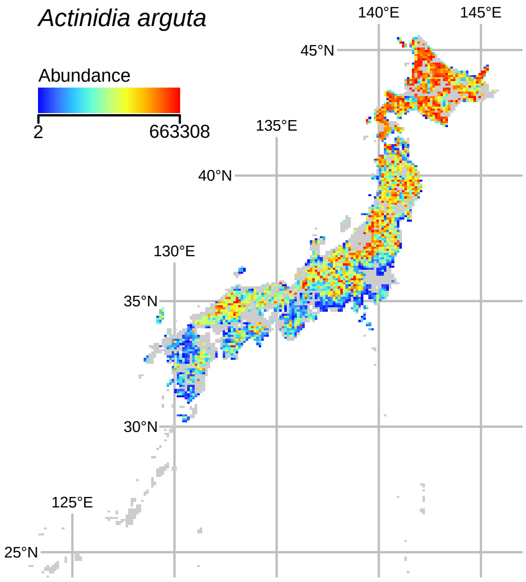
Actinidia arguta
Abundance
2
663308
140°E
145°E
45°N
135°E
40°N
130°E
35°N
30°N
125°E
25°N

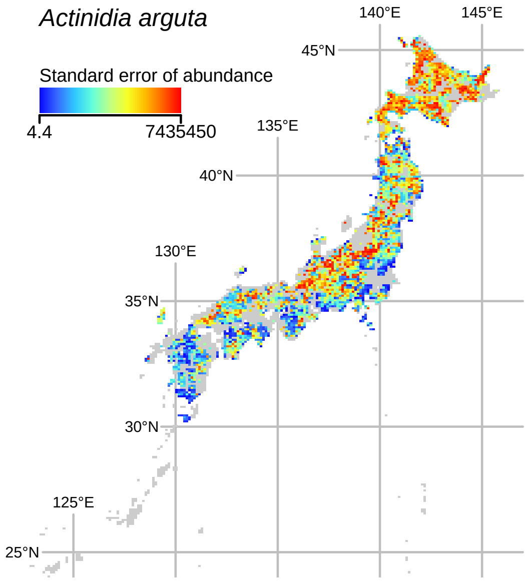
Actinidia arguta
Standard error of abundance
4.4
7435450
140°E
145°E
45°N
135°E
40°N
130°E
35°N
30°N
125°E
25°N

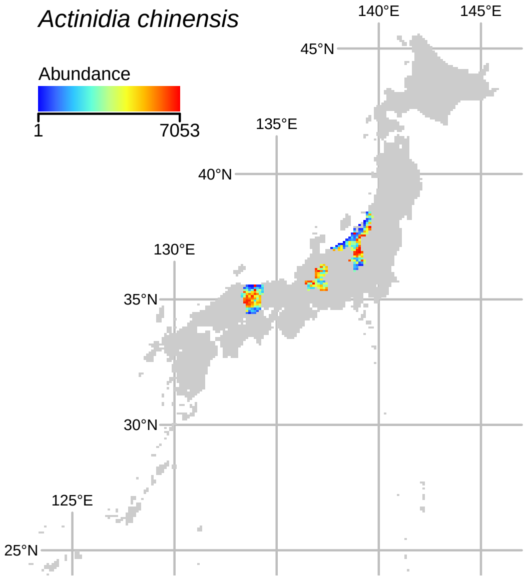
Actinidia chinensis
Abundance
1
7053
140°E
145°E
45°N
135°E
40°N
130°E
35°N
30°N
125°E
25°N

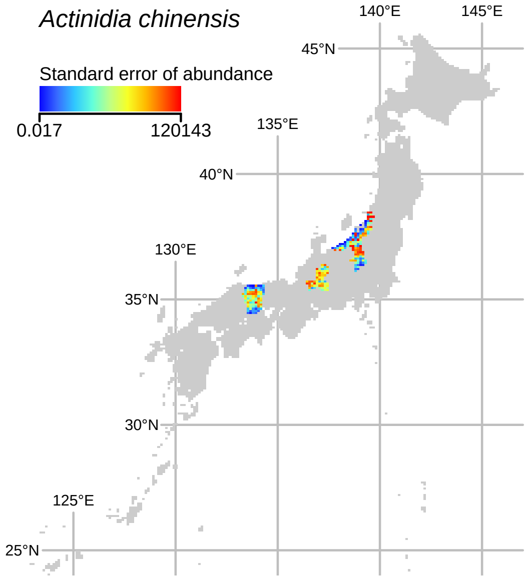
Actinidia chinensis
Standard error of abundance
0.017
120143
140°E
145°E
45°N
135°E
40°N
130°E
35°N
30°N
125°E
25°N

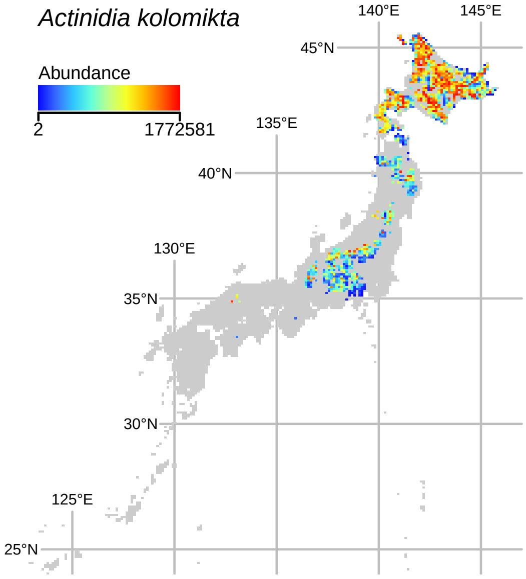
Actinidia kolomikta
Abundance
2
1772581
140°E
145°E
45°N
135°E
40°N
130°E
35°N
30°N
125°E
25°N

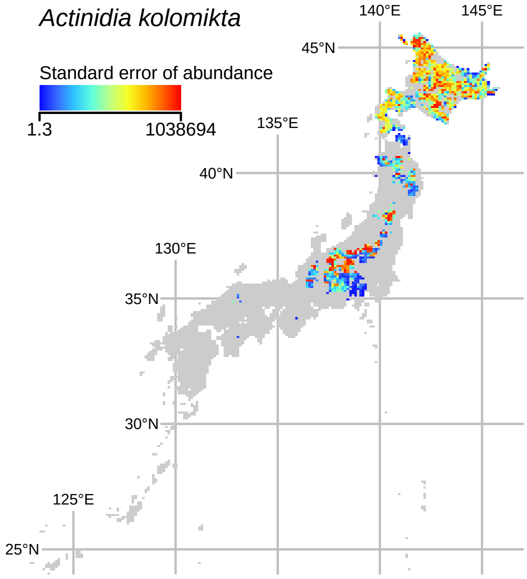
Actinidia kolomikta
Standard error of abundance
1.3
1038694
140°E
145°E
45°N
135°E
40°N
130°E
35°N
30°N
125°E
25°N

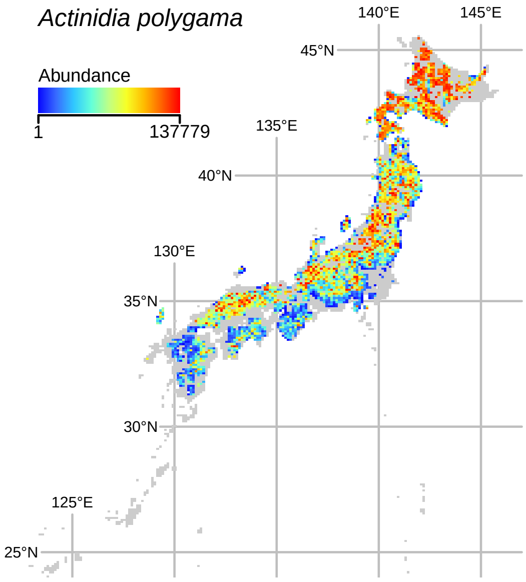
Actinidia polygama
Abundance
1
137779
140°E
145°E
45°N
135°E
40°N
130°E
35°N
30°N
125°E
25°N

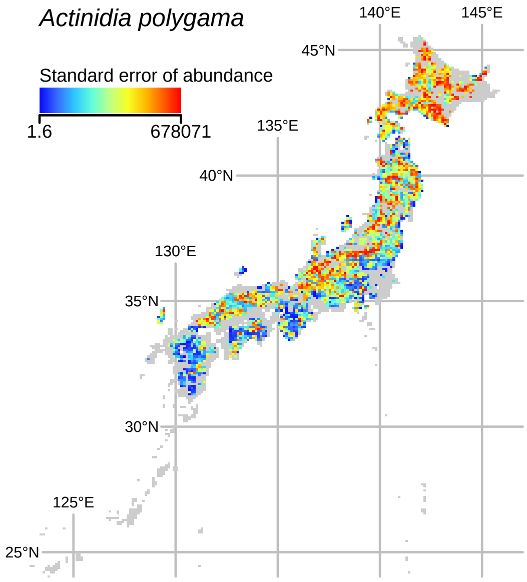
Actinidia polygama
Standard error of abundance
1.6
678071
140°E
145°E
45°N
135°E
40°N
130°E
35°N
30°N
125°E
25°N

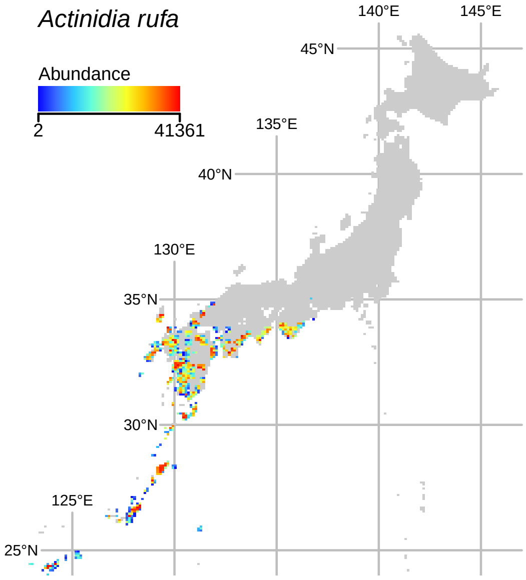
Actinidia rufa
Abundance
2
41361
140°E
145°E
45°N
135°E
40°N
130°E
35°N
30°N
125°E
25°N

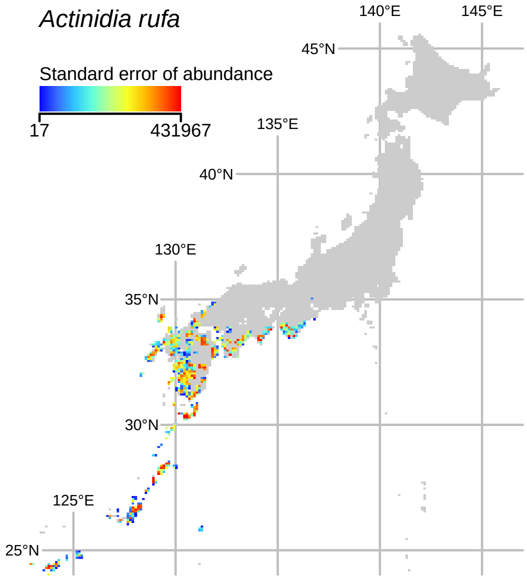
Actinidia rufa
Standard error of abundance
17
431967
140°E
145°E
45°N
135°E
40°N
130°E
35°N
30°N
125°E
25°N

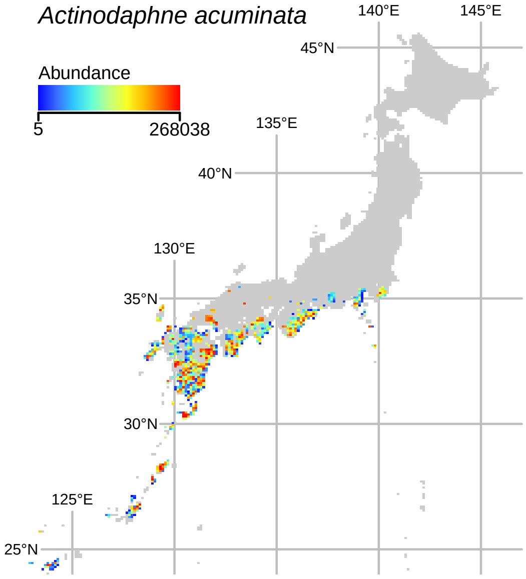
Actinodaphne acuminata
Abundance
5
268038
140°E
145°E
45°N
135°E
40°N
130°E
35°N
30°N
125°E
25°N

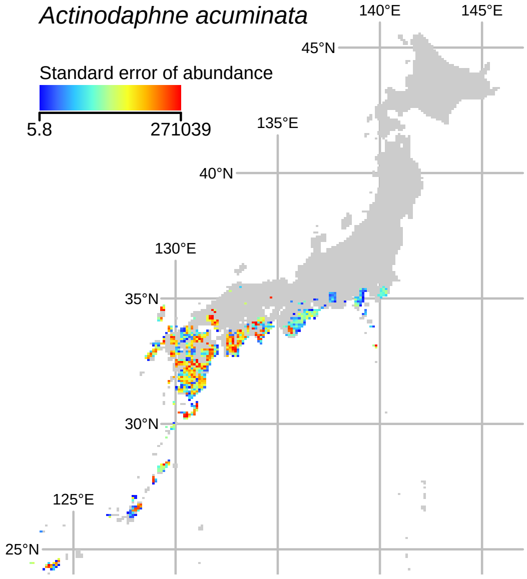
Actinodaphne acuminata
Standard error of abundance
5.8
271039
140°E
145°E
45°N
135°E
40°N
130°E
35°N
30°N
125°E
25°N

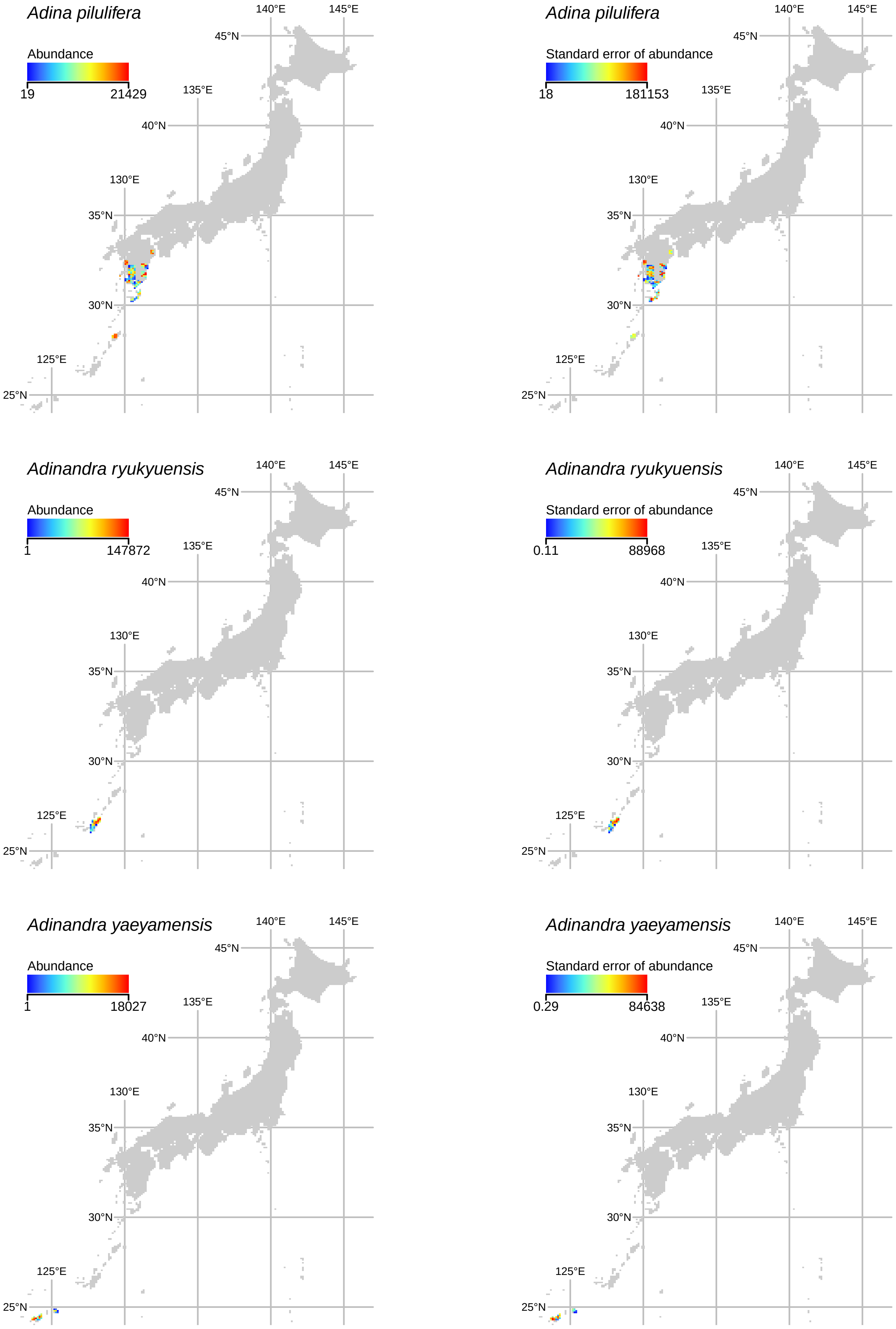
Adina pilulifera
Abundance
19
21429
Adina pilulifera
Standard error of abundance
18
181153
Adinandra ryukyuensis
Abundance
1
147872
Adinandra ryukyuensis
Standard error of abundance
0.11
88968
Adinandra yaeyamensis
Abundance
1
18027
Adinandra yaeyamensis
Standard error of abundance
0.29
84638
140°E
145°E
45°N
135°E
40°N
130°E
35°N
30°N
125°E
25°N

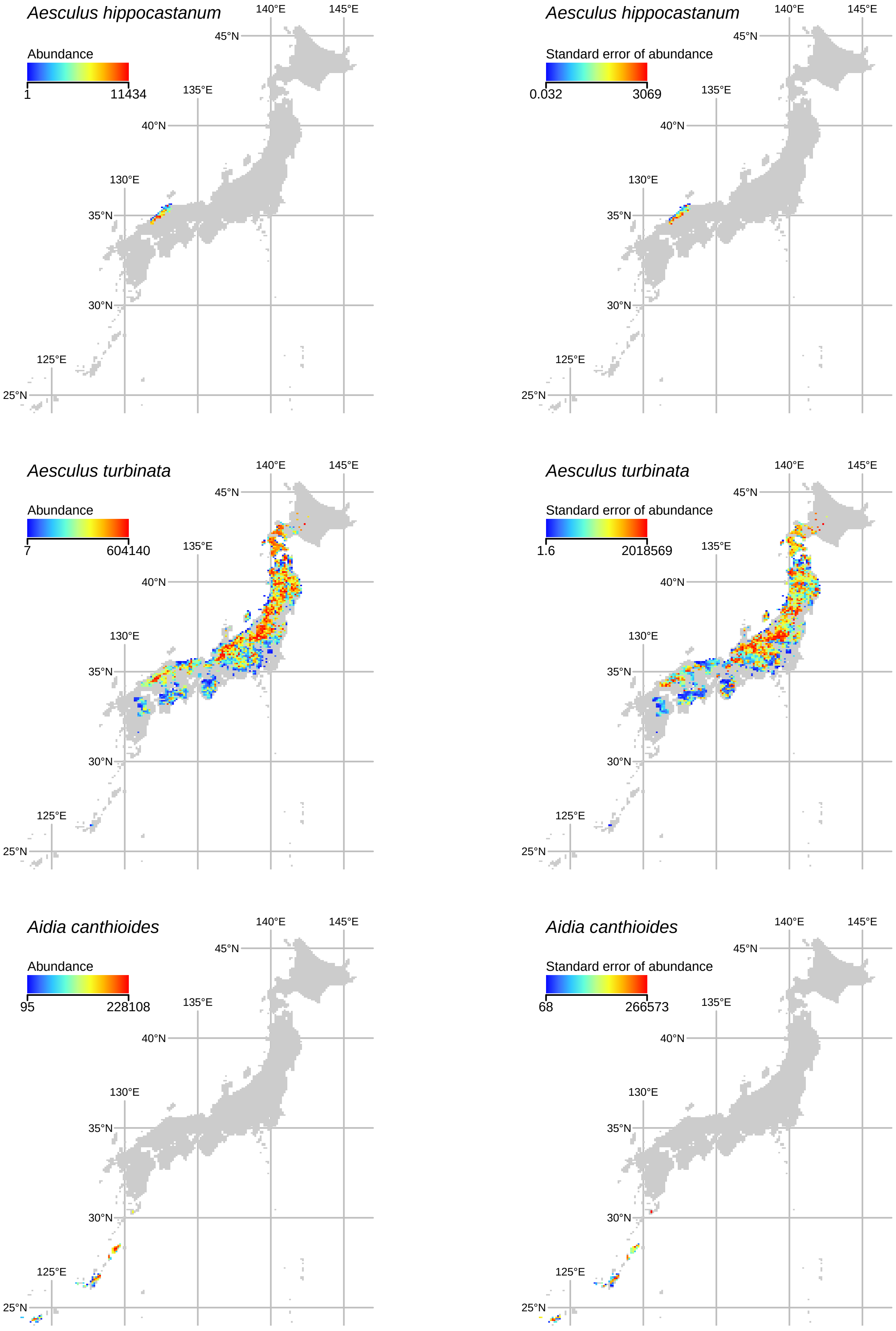
Aesculus hippocastanum
Abundance
1
11434
Aesculus hippocastanum
Standard error of abundance
0.032
3069
Aesculus turbinata
Abundance
7
604140
Aesculus turbinata
Standard error of abundance
1.6
2018569
Aidia canthioides
Abundance
95
228108
Aidia canthioides
Standard error of abundance
68
266573
125°E
130°E
135°E
140°E
145°E
25°N
30°N
35°N
40°N
45°N

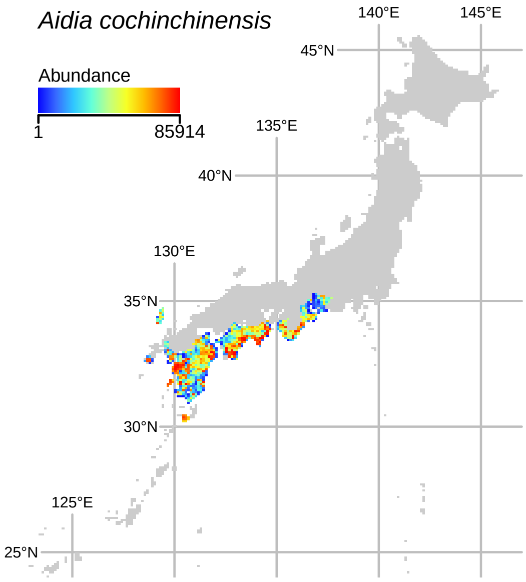
Aidia cochinchinensis
Abundance
1
85914
140°E
145°E
135°E
130°E
125°E
45°N
40°N
35°N
30°N
25°N

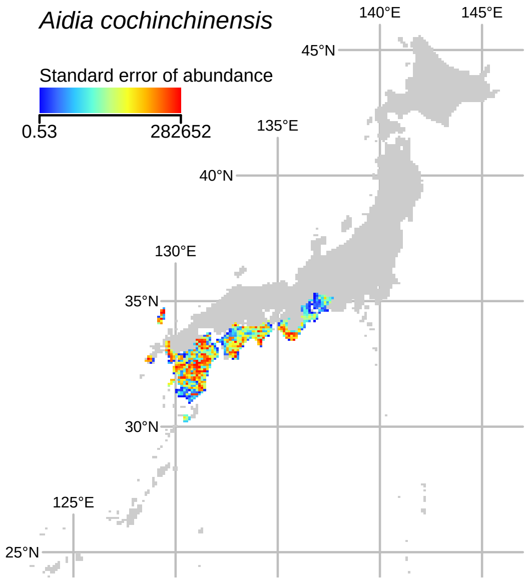
Aidia cochinchinensis
Standard error of abundance
0.53
282652
140°E
145°E
135°E
130°E
125°E
45°N
40°N
35°N
30°N
25°N

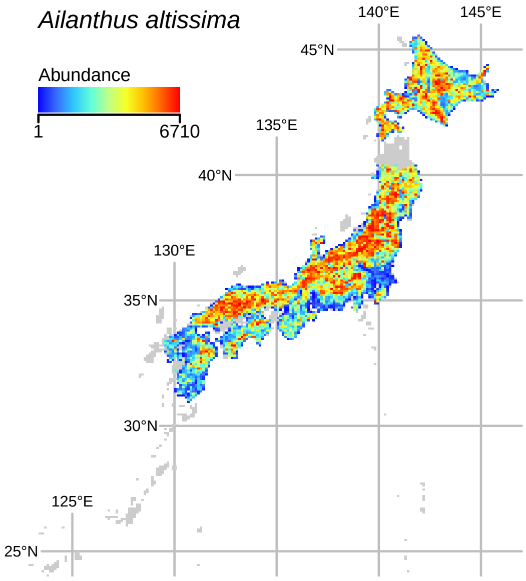
Ailanthus altissima
Abundance
1
6710
140°E
145°E
135°E
130°E
125°E
45°N
40°N
35°N
30°N
25°N

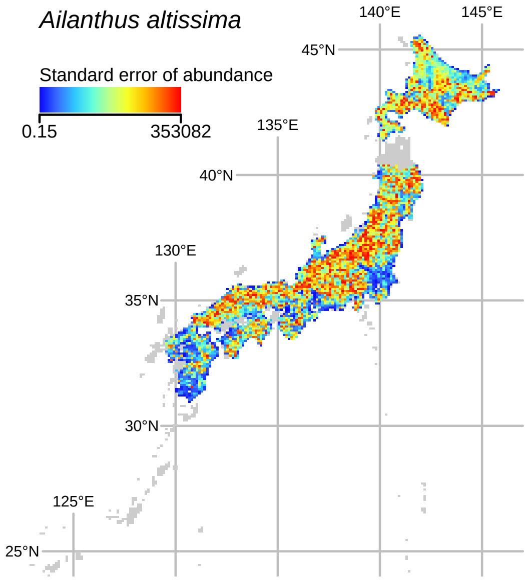
Ailanthus altissima
Standard error of abundance
0.15
353082
140°E
145°E
135°E
130°E
125°E
45°N
40°N
35°N
30°N
25°N

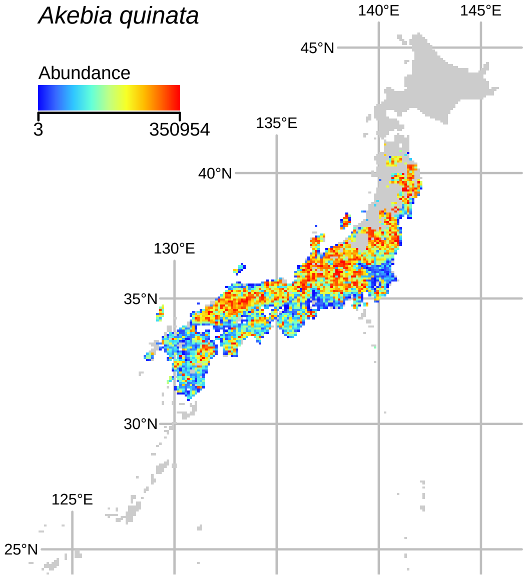
Akebia quinata
Abundance
3
350954
140°E
145°E
135°E
130°E
125°E
45°N
40°N
35°N
30°N
25°N

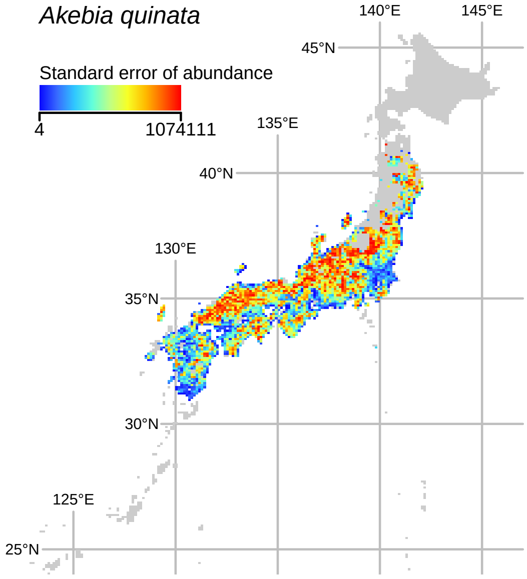
Akebia quinata
Standard error of abundance
4
1074111
140°E
145°E
135°E
130°E
125°E
45°N
40°N
35°N
30°N
25°N

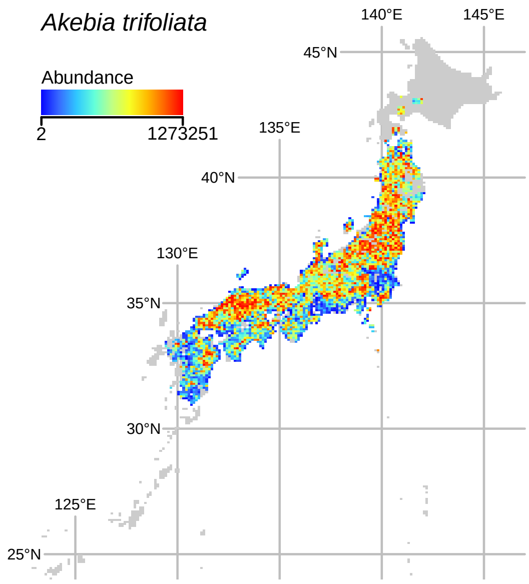
Akebia trifoliata
Abundance
2
1273251
140°E
145°E
45°N
135°E
40°N
130°E
35°N
30°N
125°E
25°N

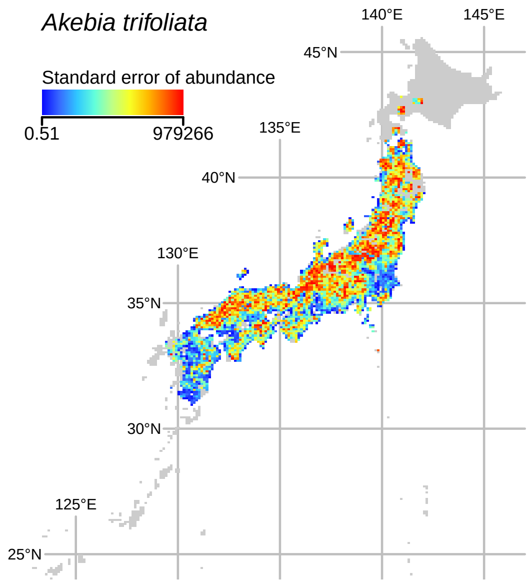
Akebia trifoliata
Standard error of abundance
0.51
979266
140°E
145°E
45°N
135°E
40°N
130°E
35°N
30°N
125°E
25°N

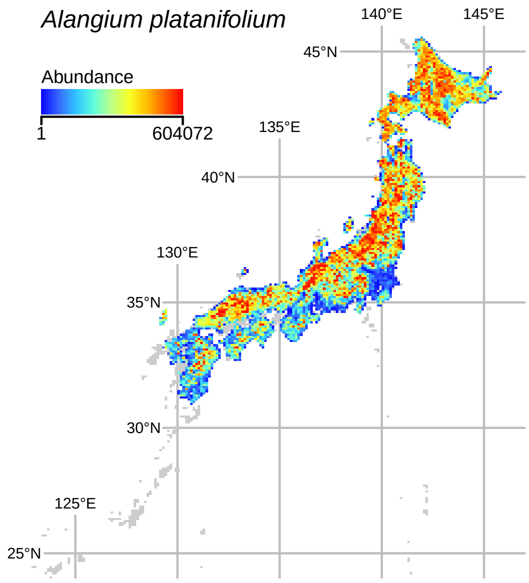
Alangium platanifolium
Abundance
1
604072
140°E
145°E
45°N
135°E
40°N
130°E
35°N
30°N
125°E
25°N

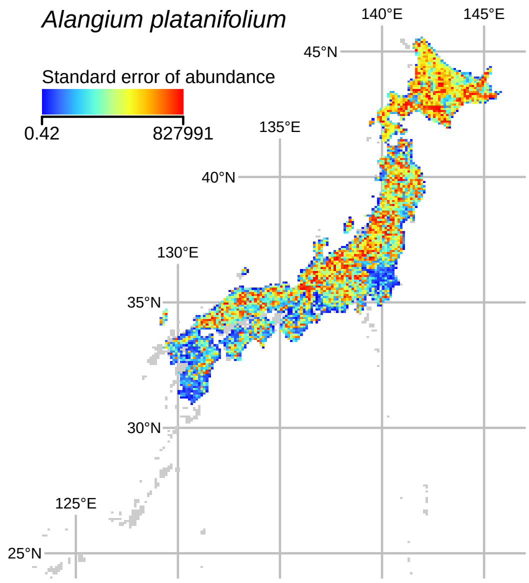
Alangium platanifolium
Standard error of abundance
0.42
827991
140°E
145°E
45°N
135°E
40°N
130°E
35°N
30°N
125°E
25°N

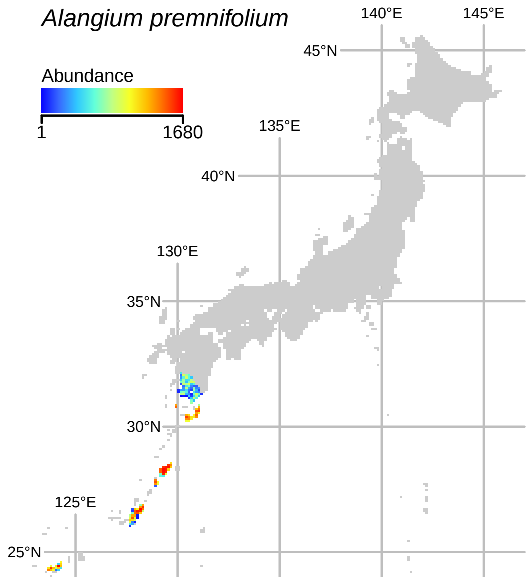
Alangium premnifolium
Abundance
1
1680
140°E
145°E
45°N
135°E
40°N
130°E
35°N
30°N
125°E
25°N

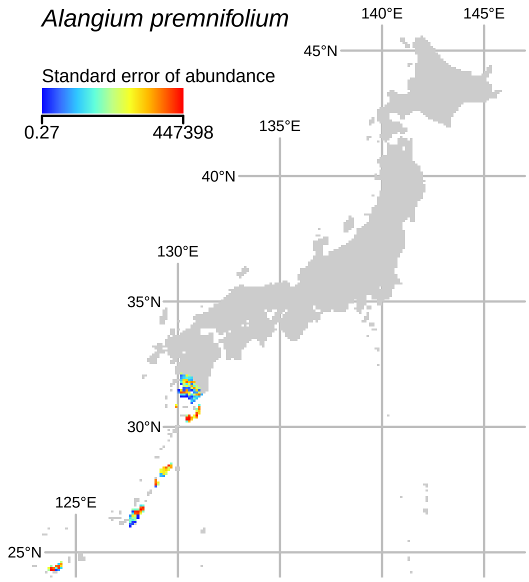
Alangium premnifolium
Standard error of abundance
0.27
447398
140°E
145°E
45°N
135°E
40°N
130°E
35°N
30°N
125°E
25°N

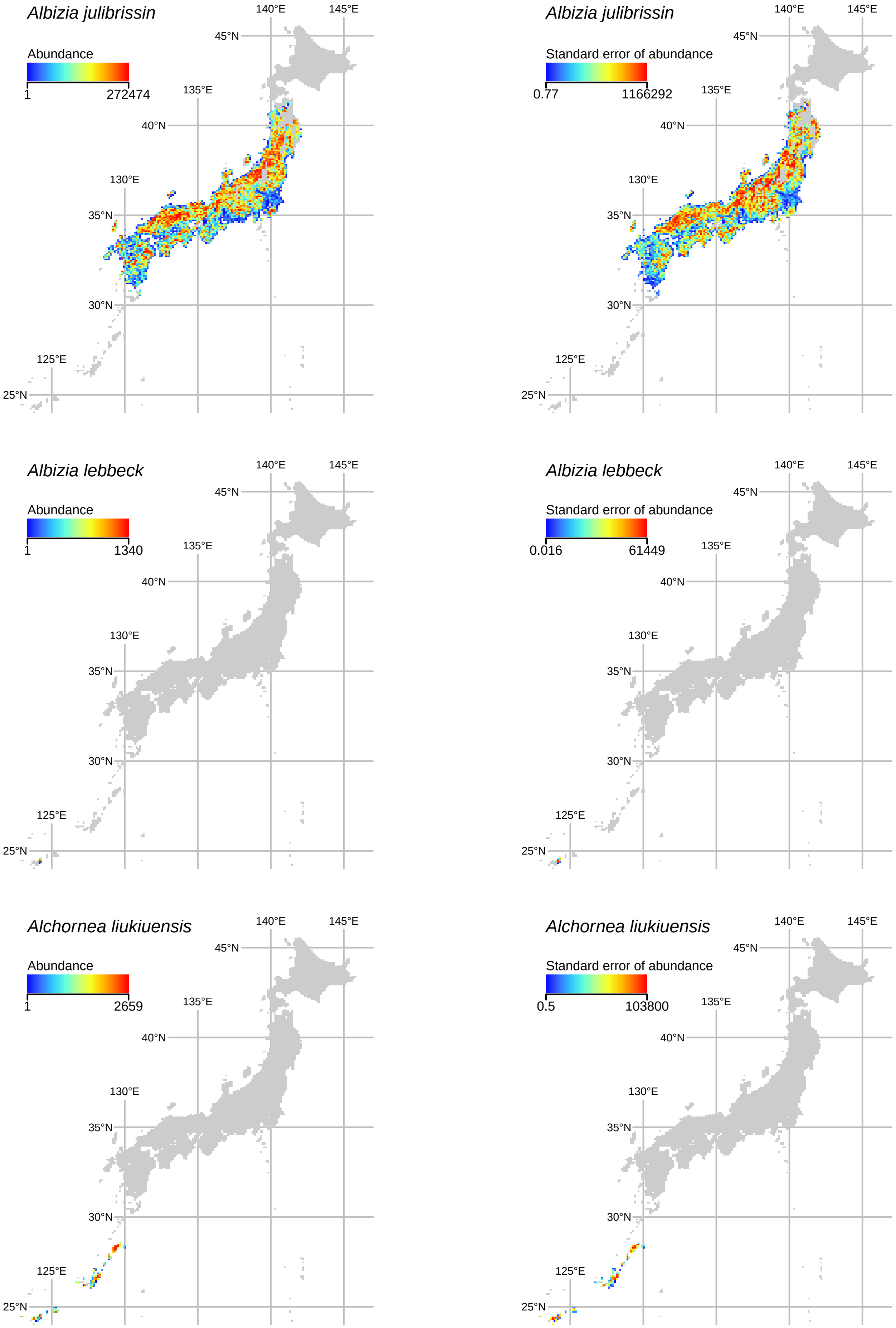
Albizia julibrissin
Abundance
1
272474
Albizia julibrissin
Standard error of abundance
0.77
1166292
Albizia lebbeck
Abundance
1
1340
Albizia lebbeck
Standard error of abundance
0.016
61449
Alchornea liukiuensis
Abundance
1
2659
Alchornea liukiuensis
Standard error of abundance
0.5
103800
125°E
130°E
135°E
140°E
145°E
25°N
30°N
35°N
40°N
45°N

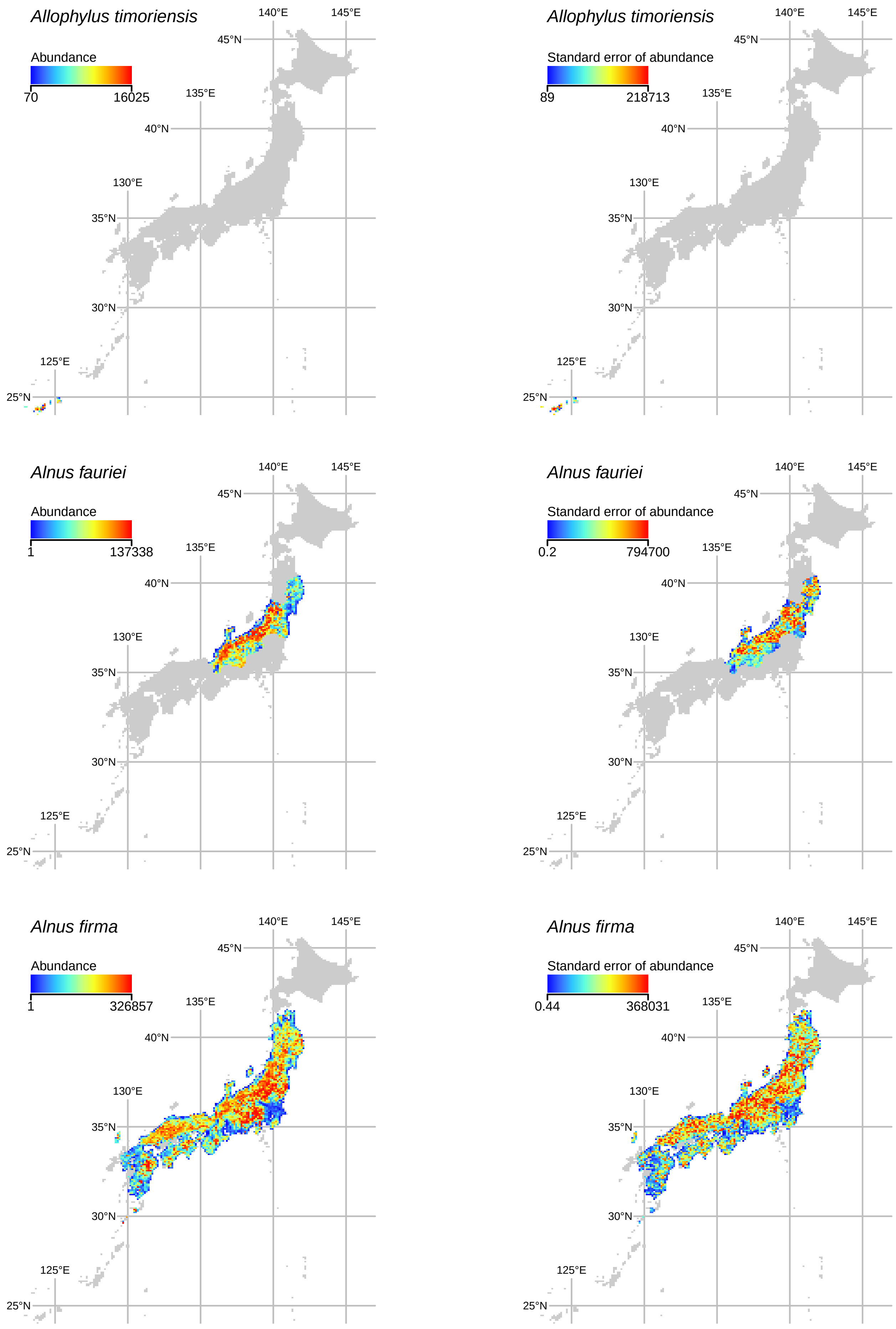
Allophylus timoriensis
Abundance
70
16025
Allophylus timoriensis
Standard error of abundance
89
218713
Alnus fauriei
Abundance
1
137338
Alnus fauriei
Standard error of abundance
0.2
794700
Alnus firma
Abundance
1
326857
Alnus firma
Standard error of abundance
0.44
368031
125°E
130°E
135°E
140°E
145°E
25°N
30°N
35°N
40°N
45°N

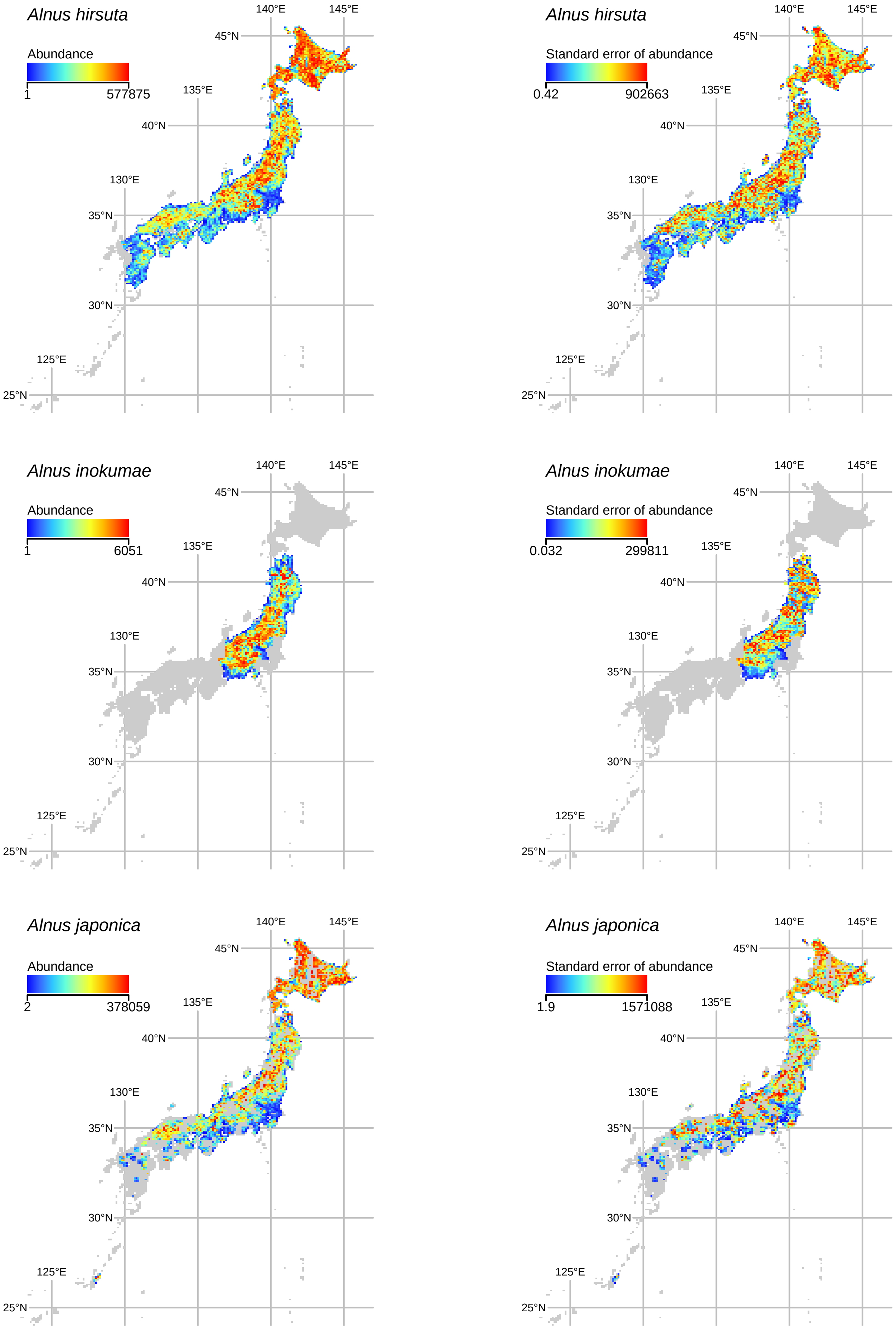
Alnus hirsuta
Abundance
1
577875
Alnus hirsuta
Standard error of abundance
0.42
902663
Alnus inokumae
Abundance
1
6051
Alnus inokumae
Standard error of abundance
0.032
299811
Alnus japonica
Abundance
2
378059
Alnus japonica
Standard error of abundance
1.9
1571088
125°E
130°E
135°E
140°E
145°E
25°N
30°N
35°N
40°N
45°N

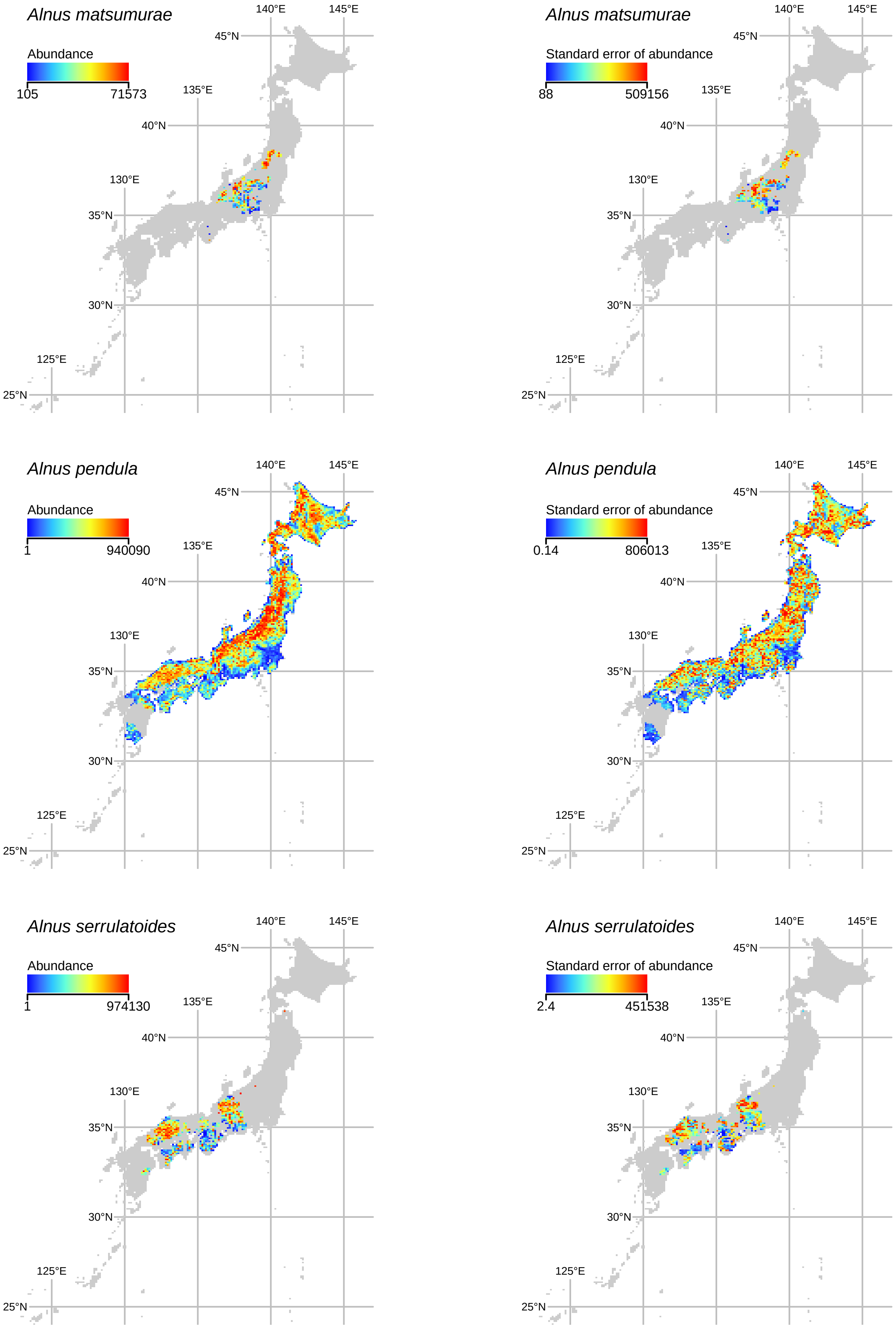
Alnus matsumurae
Abundance
105
71573
Alnus matsumurae
Standard error of abundance
88
509156
Alnus pendula
Abundance
1
940090
Alnus pendula
Standard error of abundance
0.14
806013
Alnus serrulatoides
Abundance
1
974130
Alnus serrulatoides
Standard error of abundance
2.4
451538
125°E
130°E
135°E
140°E
145°E
25°N
30°N
35°N
40°N
45°N

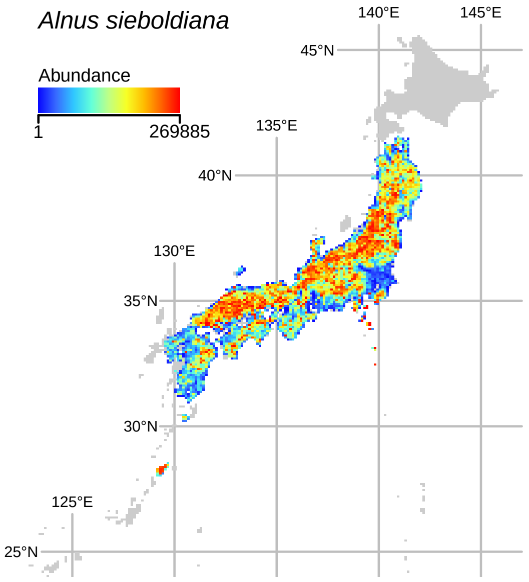
Alnus sieboldiana
Abundance
1
269885
140°E
145°E
45°N
135°E
40°N
130°E
35°N
30°N
125°E
25°N

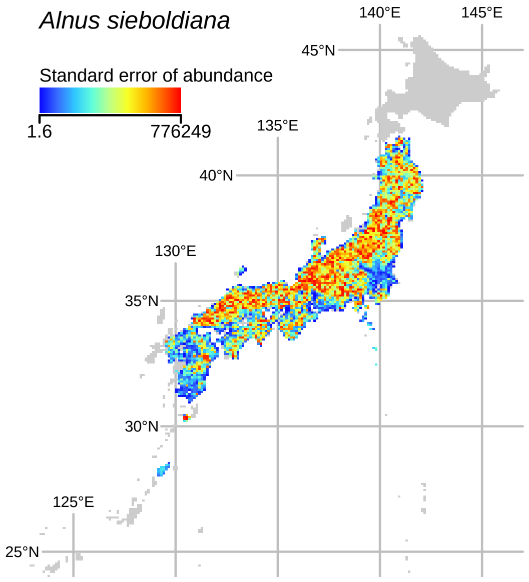
Alnus sieboldiana
Standard error of abundance
1.6
776249
140°E
145°E
45°N
135°E
40°N
130°E
35°N
30°N
125°E
25°N

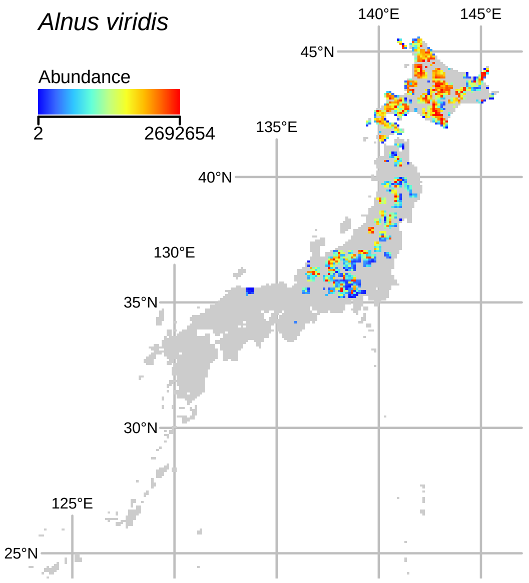
Alnus viridis
Abundance
2
2692654
140°E
145°E
45°N
135°E
40°N
130°E
35°N
30°N
125°E
25°N

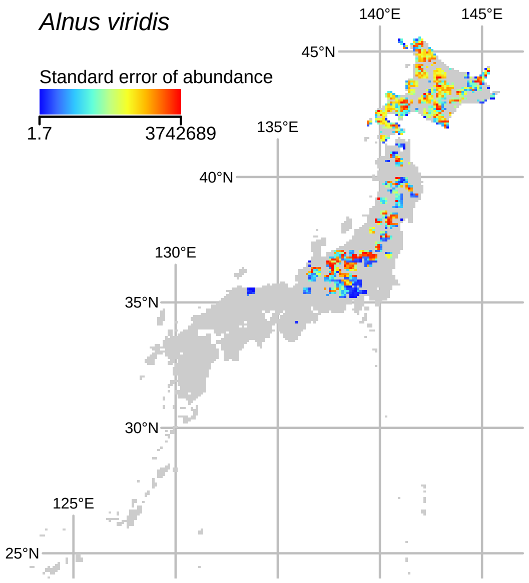
Alnus viridis
Standard error of abundance
1.7
3742689
140°E
145°E
45°N
135°E
40°N
130°E
35°N
30°N
125°E
25°N

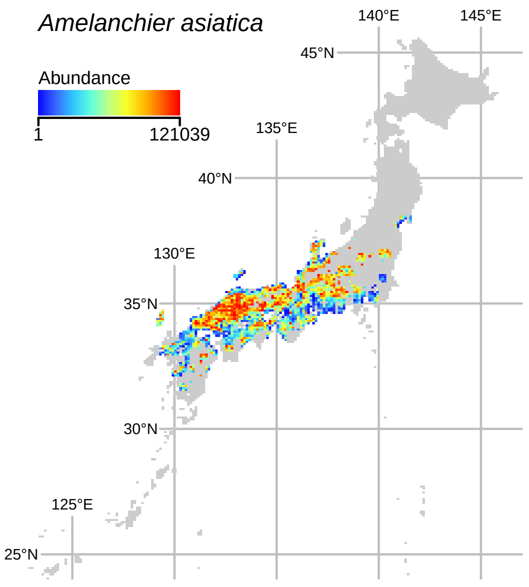
Amelanchier asiatica
Abundance
1
121039
140°E
145°E
45°N
135°E
40°N
130°E
35°N
30°N
125°E
25°N

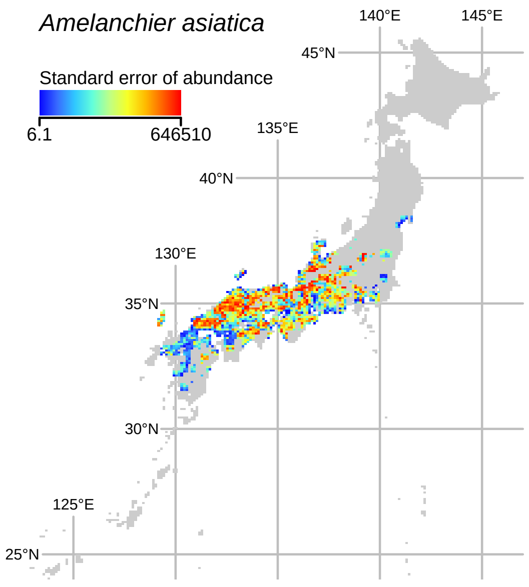
Amelanchier asiatica
Standard error of abundance
6.1
646510
140°E
145°E
45°N
135°E
40°N
130°E
35°N
30°N
125°E
25°N

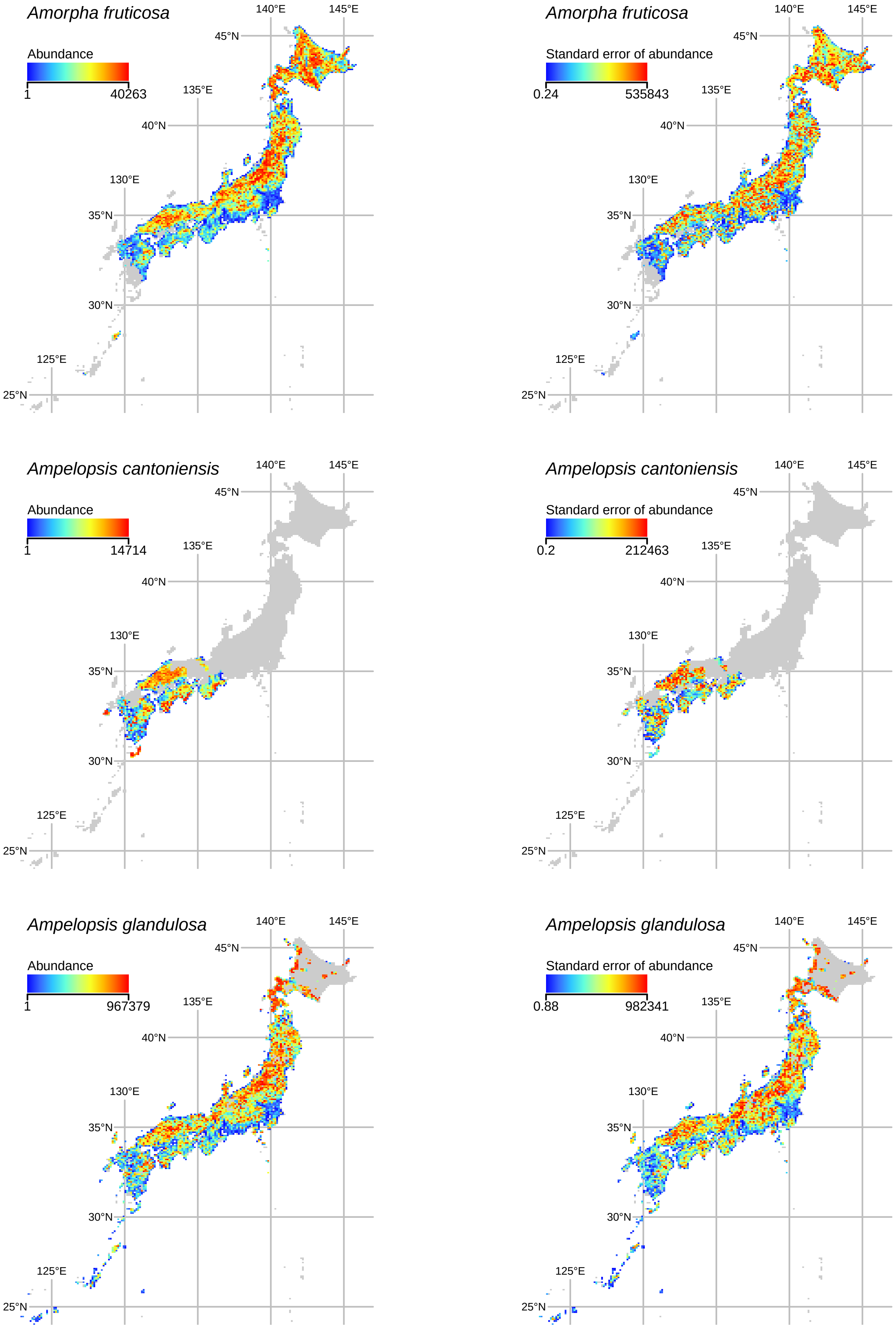
Amorpha fruticosa
Abundance
1
40263
Amorpha fruticosa
Standard error of abundance
0.24
535843
Ampelopsis cantoniensis
Abundance
1
14714
Ampelopsis cantoniensis
Standard error of abundance
0.2
212463
Ampelopsis glandulosa
Abundance
1
967379
Ampelopsis glandulosa
Standard error of abundance
0.88
982341
125°E
130°E
135°E
140°E
145°E
25°N
30°N
35°N
40°N
45°N

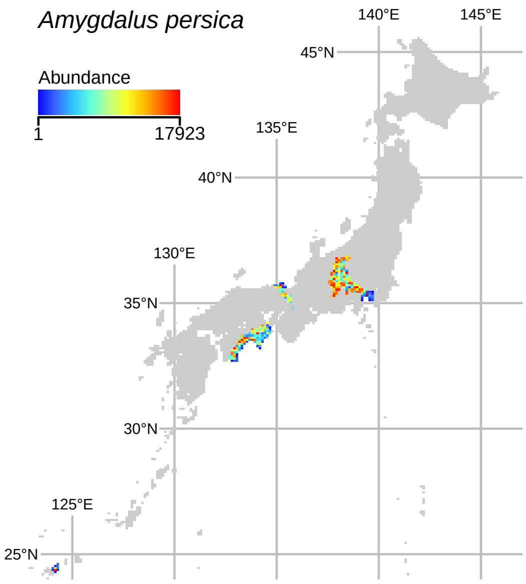
Amygdalus persica
Abundance
1
17923
140°E
145°E
135°E
130°E
125°E
45°N
40°N
35°N
30°N
25°N

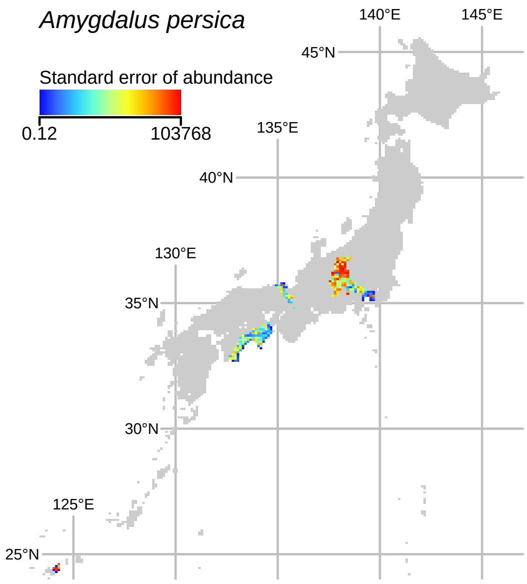
Amygdalus persica
Standard error of abundance
0.12
103768
140°E
145°E
135°E
130°E
125°E
45°N
40°N
35°N
30°N
25°N

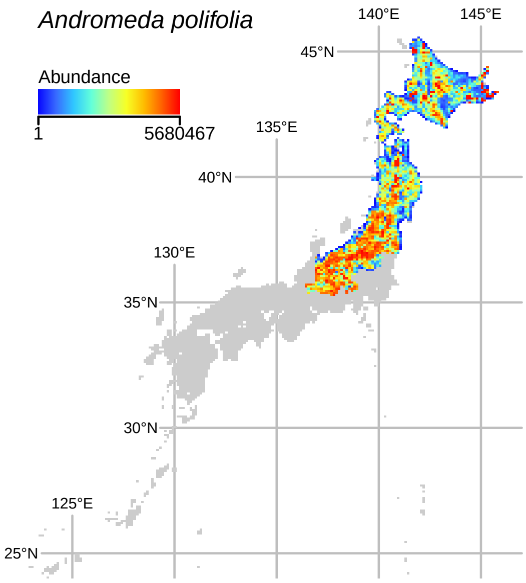
Andromeda polifolia
Abundance
1
5680467
140°E
145°E
135°E
130°E
125°E
45°N
40°N
35°N
30°N
25°N

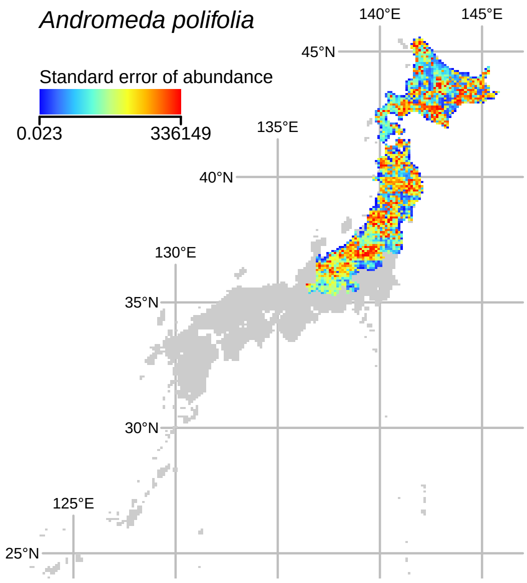
Andromeda polifolia
Standard error of abundance
0.023
336149
140°E
145°E
135°E
130°E
125°E
45°N
40°N
35°N
30°N
25°N

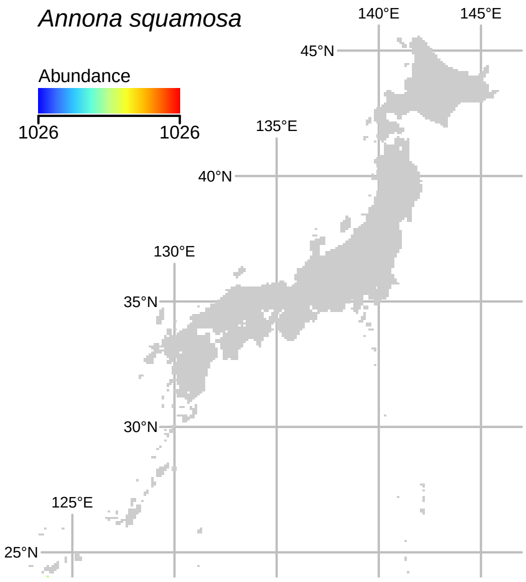
Annona squamosa
Abundance
1026
1026
140°E
145°E
135°E
130°E
125°E
45°N
40°N
35°N
30°N
25°N

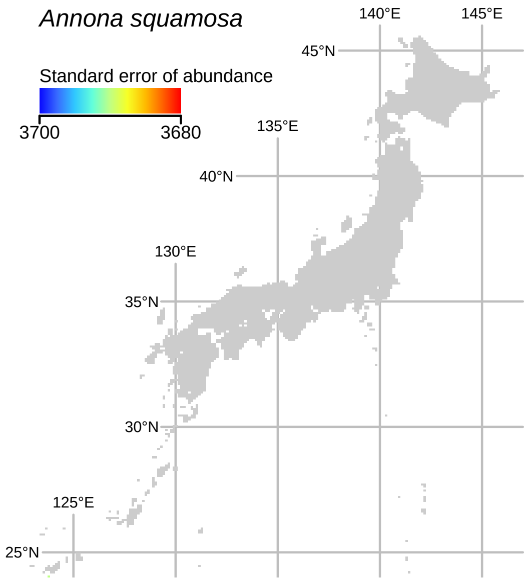
Annona squamosa
Standard error of abundance
3700
3680
140°E
145°E
135°E
130°E
125°E
45°N
40°N
35°N
30°N
25°N

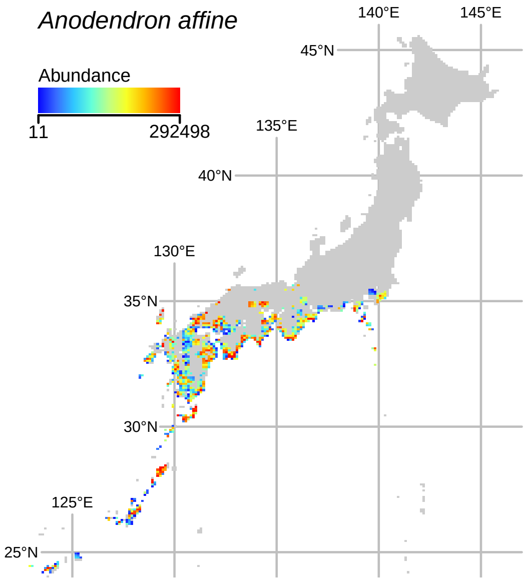
Anodendron affine
Abundance
11
292498
140°E
145°E
45°N
135°E
40°N
130°E
35°N
30°N
125°E
25°N

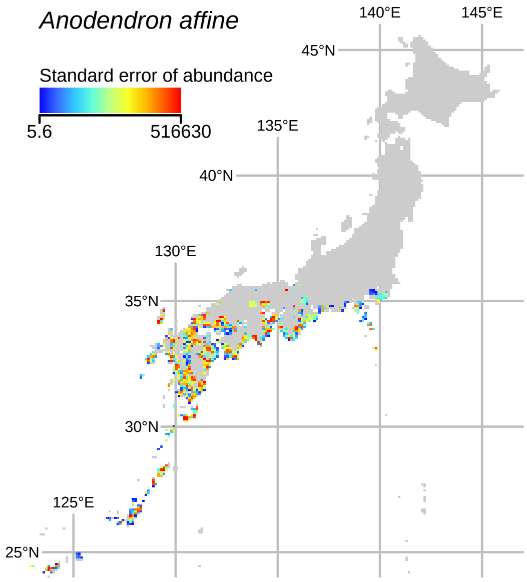
Anodendron affine
Standard error of abundance
5.6
516630
140°E
145°E
45°N
135°E
40°N
130°E
35°N
30°N
125°E
25°N

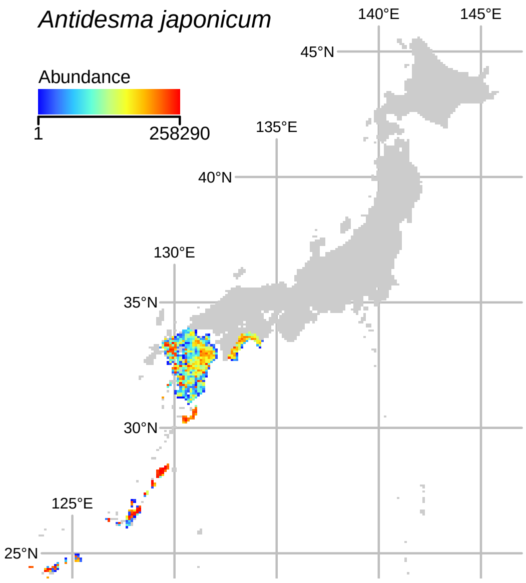
Antidesma japonicum
Abundance
1
258290
140°E
145°E
45°N
135°E
40°N
130°E
35°N
30°N
125°E
25°N

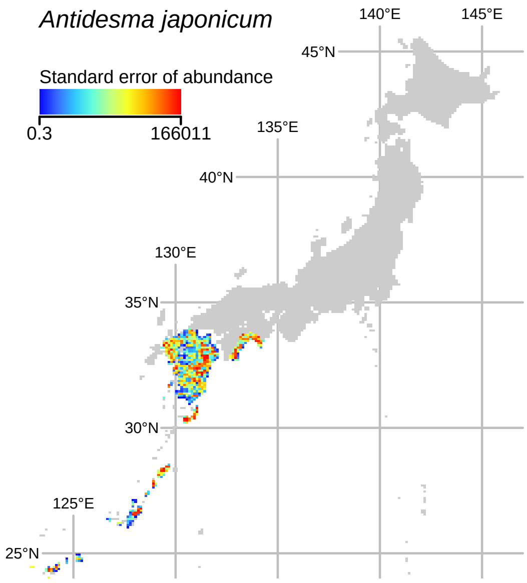
Antidesma japonicum
Standard error of abundance
0.3
166011
140°E
145°E
45°N
135°E
40°N
130°E
35°N
30°N
125°E
25°N

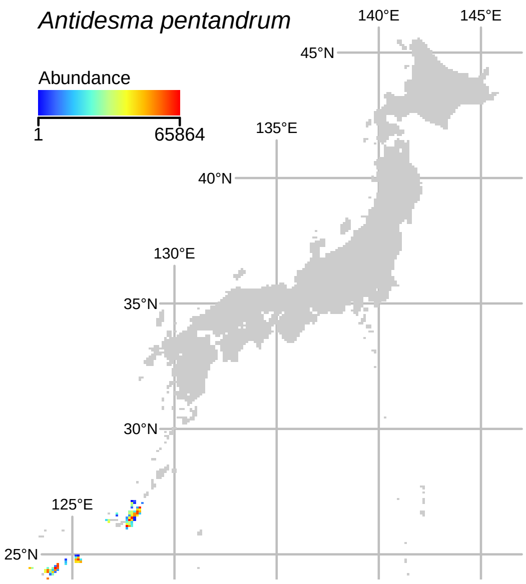
Antidesma pentandrum
Abundance
1
65864
140°E
145°E
45°N
135°E
40°N
130°E
35°N
30°N
125°E
25°N

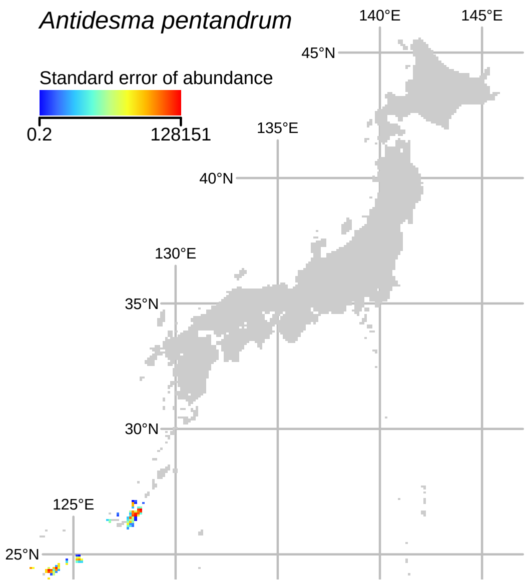
Antidesma pentandrum
Standard error of abundance
0.2
128151
140°E
145°E
45°N
135°E
40°N
130°E
35°N
30°N
125°E
25°N

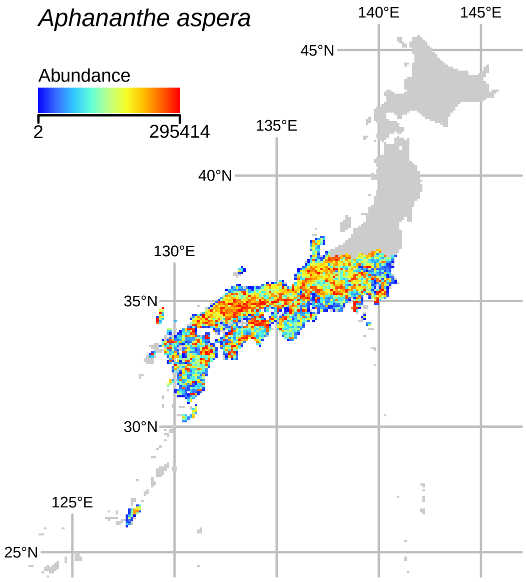
Aphananthe aspera
Abundance
2
295414
140°E
145°E
45°N
135°E
40°N
130°E
35°N
30°N
125°E
25°N

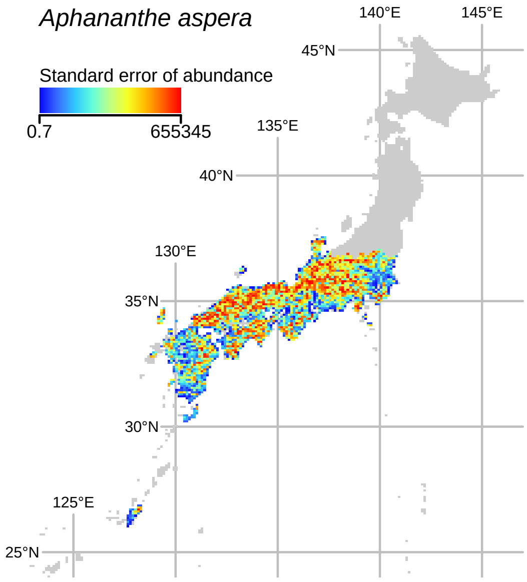
Aphananthe aspera
Standard error of abundance
0.7
655345
140°E
145°E
45°N
135°E
40°N
130°E
35°N
30°N
125°E
25°N

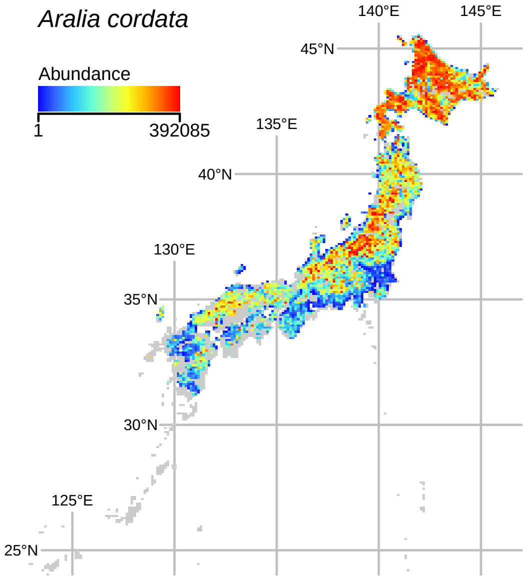
Aralia cordata
Abundance
1
392085
140°E
145°E
45°N
135°E
40°N
130°E
35°N
30°N
125°E
25°N

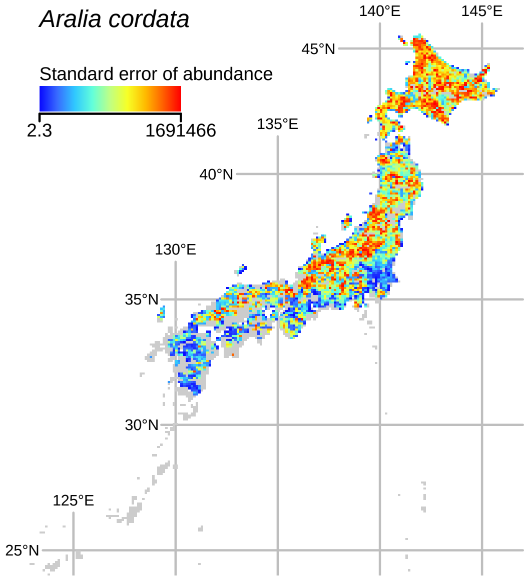
Aralia cordata
Standard error of abundance
2.3
1691466
140°E
145°E
45°N
135°E
40°N
130°E
35°N
30°N
125°E
25°N

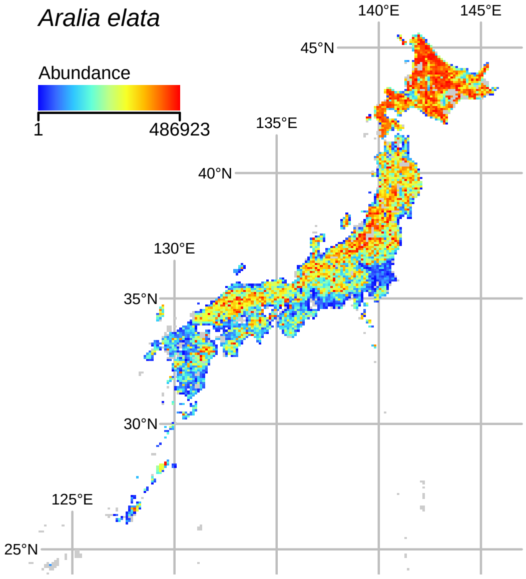
Aralia elata
Abundance
1
486923
140°E
145°E
45°N
135°E
40°N
130°E
35°N
30°N
125°E
25°N

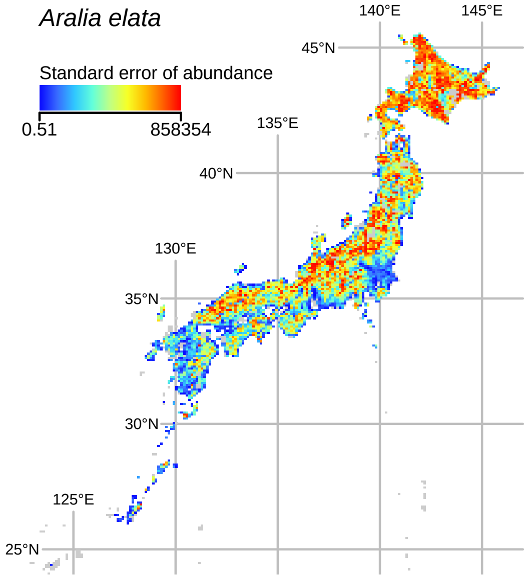
Aralia elata
Standard error of abundance
0.51
858354
140°E
145°E
45°N
135°E
40°N
130°E
35°N
30°N
125°E
25°N

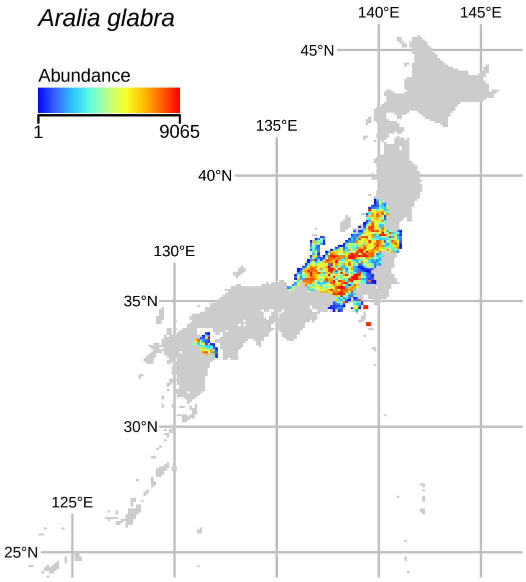
Aralia glabra
Abundance
1
9065
140°E
145°E
45°N
135°E
40°N
130°E
35°N
30°N
125°E
25°N

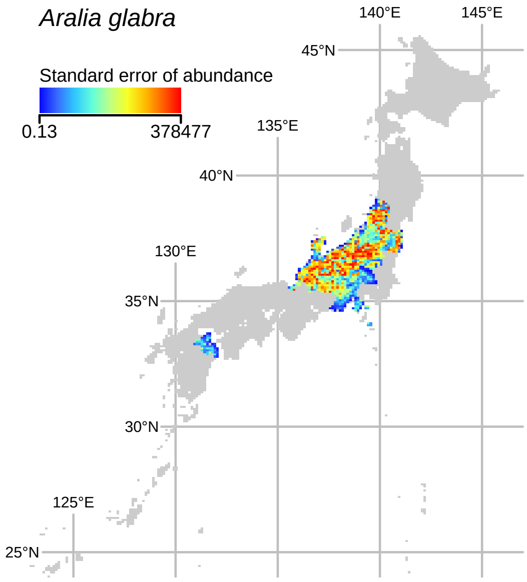
Aralia glabra
Standard error of abundance
0.13
378477
140°E
145°E
45°N
135°E
40°N
130°E
35°N
30°N
125°E
25°N

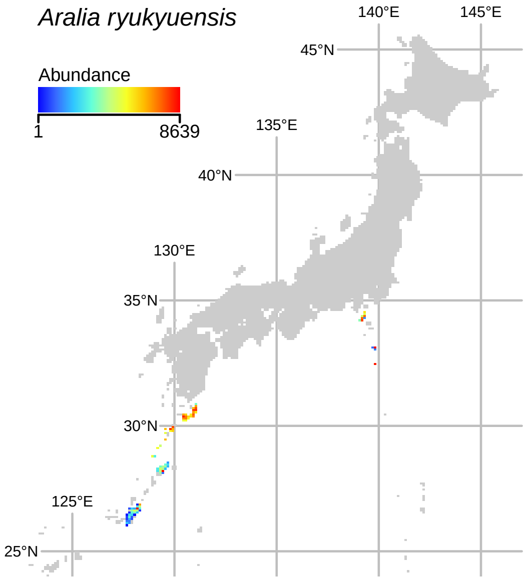
Aralia ryukyuensis
Abundance
1
8639
140°E
145°E
45°N
135°E
40°N
130°E
35°N
30°N
125°E
25°N

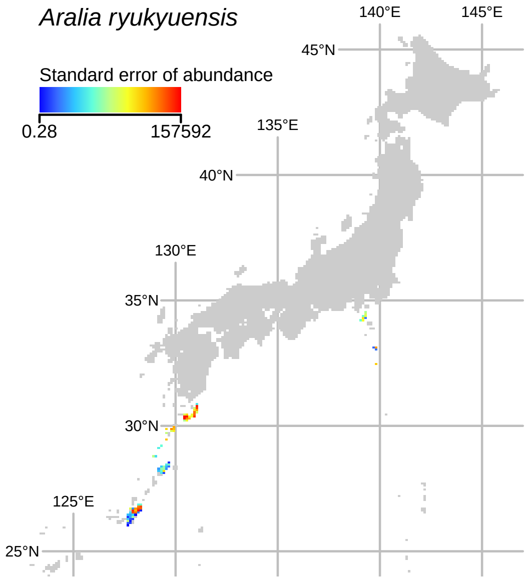
Aralia ryukyuensis
Standard error of abundance
0.28
157592
140°E
145°E
45°N
135°E
40°N
130°E
35°N
30°N
125°E
25°N

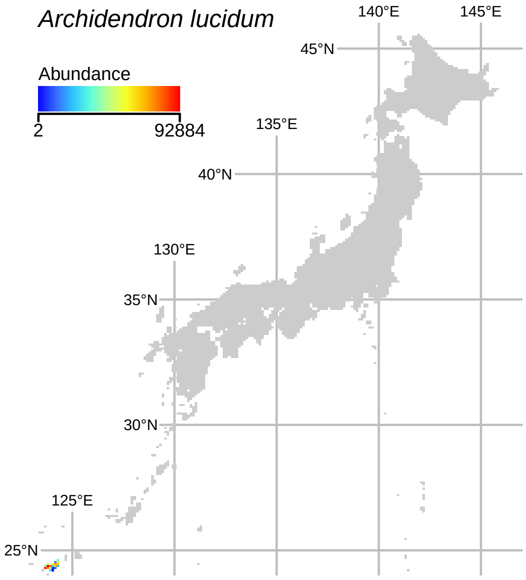
Archidendron lucidum
Abundance
2
92884
140°E
145°E
45°N
135°E
40°N
130°E
35°N
30°N
125°E
25°N

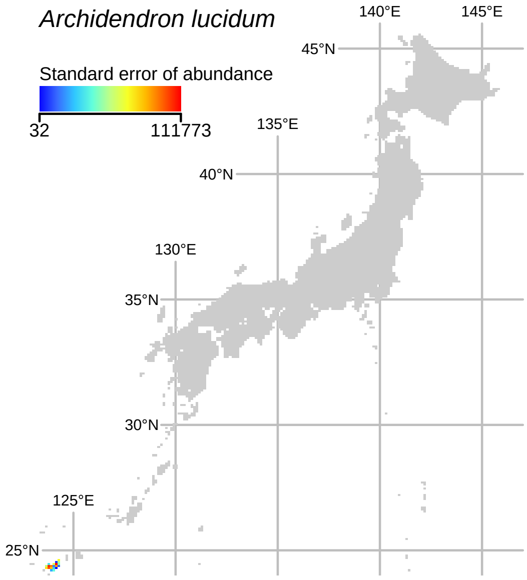
Archidendron lucidum
Standard error of abundance
32
111773
140°E
145°E
45°N
135°E
40°N
130°E
35°N
30°N
125°E
25°N

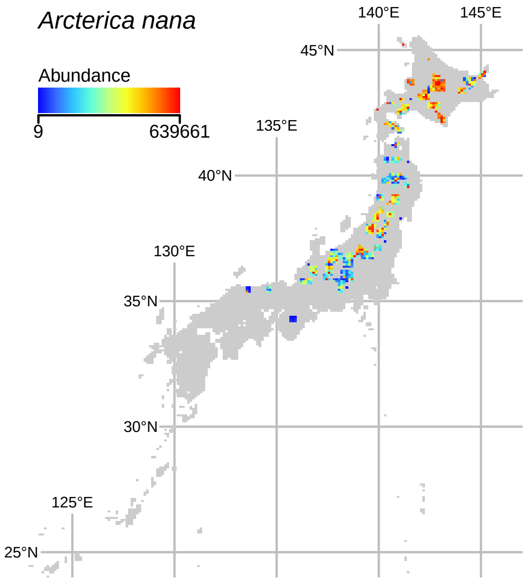
Arcterica nana
Abundance
9
639661
140°E
145°E
45°N
135°E
40°N
130°E
35°N
30°N
125°E
25°N

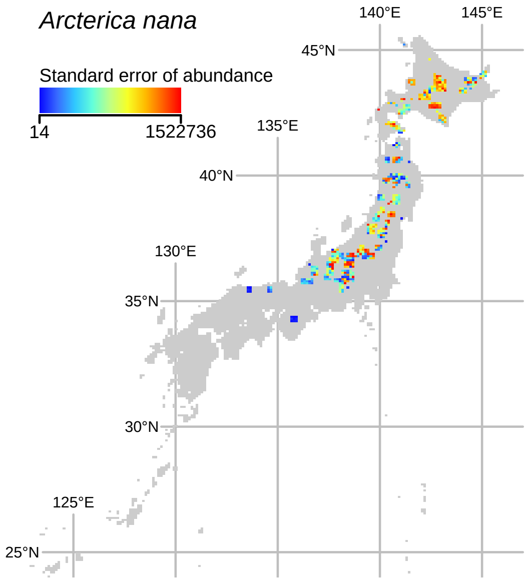
Arcterica nana
Standard error of abundance
14
1522736
140°E
145°E
45°N
135°E
40°N
130°E
35°N
30°N
125°E
25°N

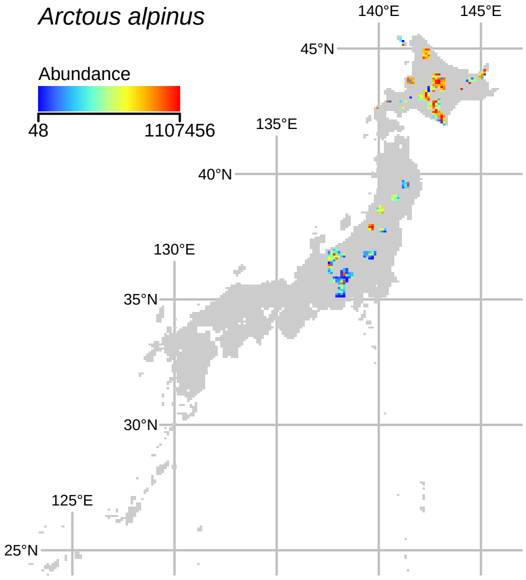
Arctous alpinus
Abundance
48
1107456
140°E
145°E
45°N
135°E
40°N
130°E
35°N
30°N
125°E
25°N

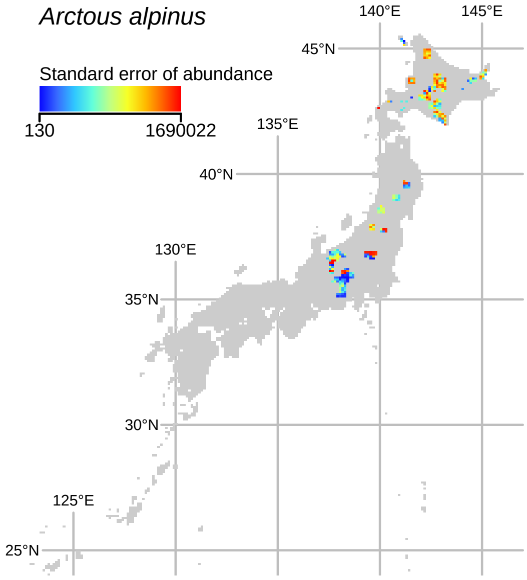
Arctous alpinus
Standard error of abundance
130
1690022
140°E
145°E
45°N
135°E
40°N
130°E
35°N
30°N
125°E
25°N

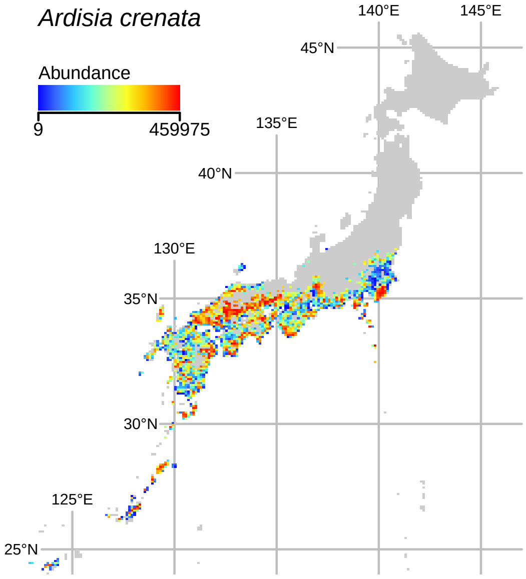
Ardisia crenata
Abundance
9
459975
140°E
145°E
45°N
135°E
40°N
130°E
35°N
30°N
125°E
25°N

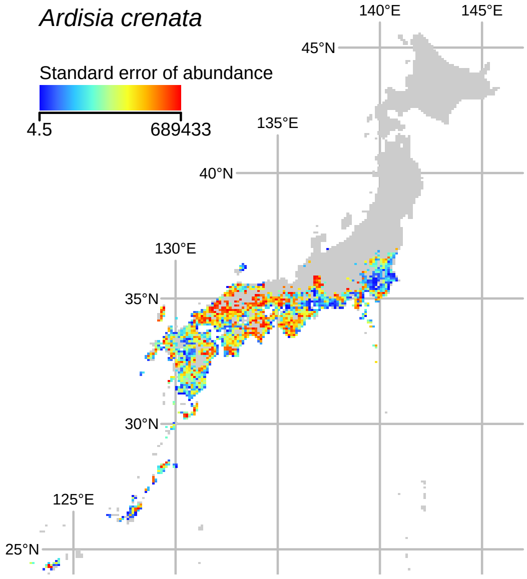
Ardisia crenata
Standard error of abundance
4.5
689433
140°E
145°E
45°N
135°E
40°N
130°E
35°N
30°N
125°E
25°N

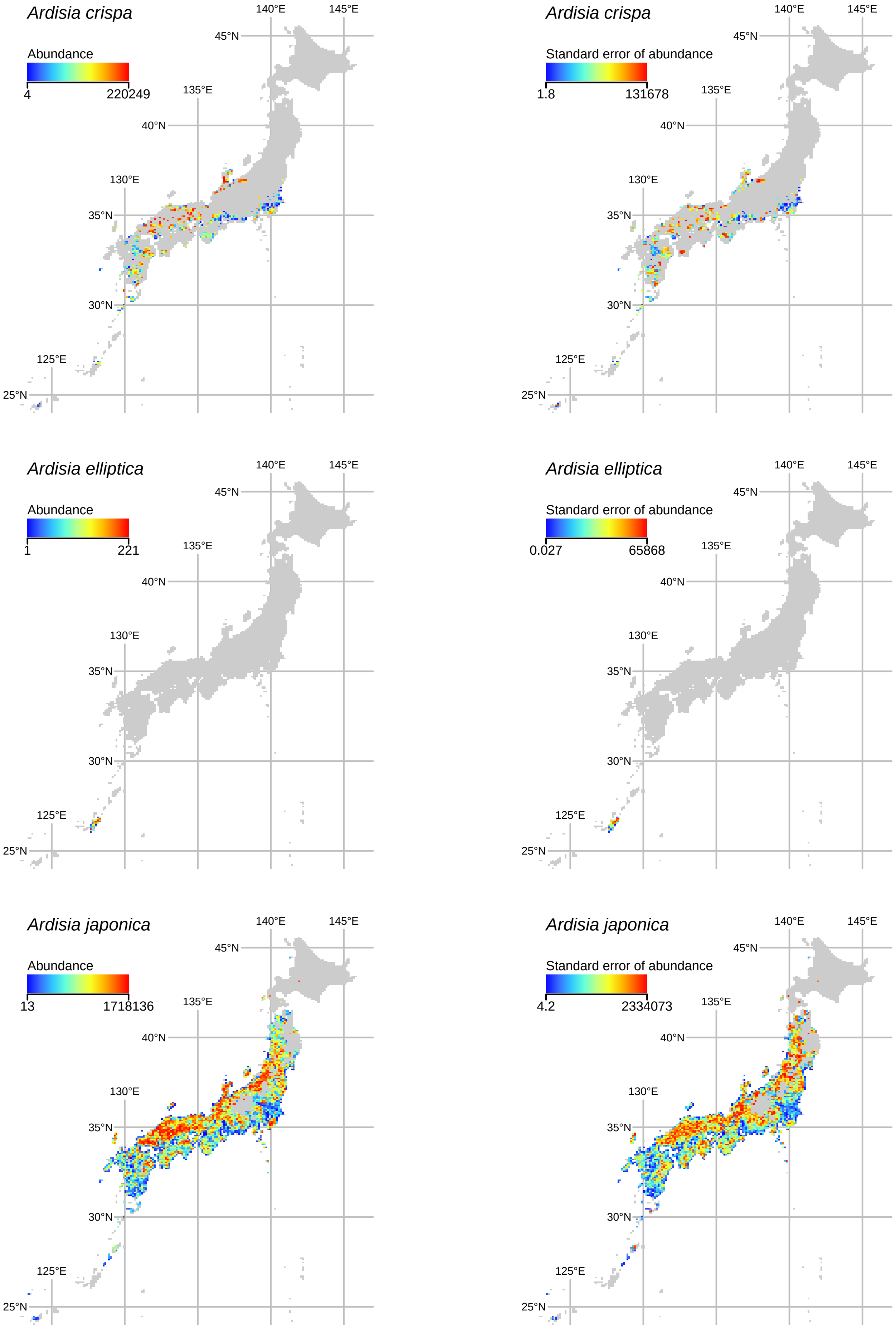
Ardisia crispa
Abundance
4
220249
Ardisia crispa
Standard error of abundance
1.8
131678
Ardisia elliptica
Abundance
1
221
Ardisia elliptica
Standard error of abundance
0.027
65868
Ardisia japonica
Abundance
13
1718136
Ardisia japonica
Standard error of abundance
4.2
2334073
125°E
130°E
135°E
140°E
145°E
25°N
30°N
35°N
40°N
45°N

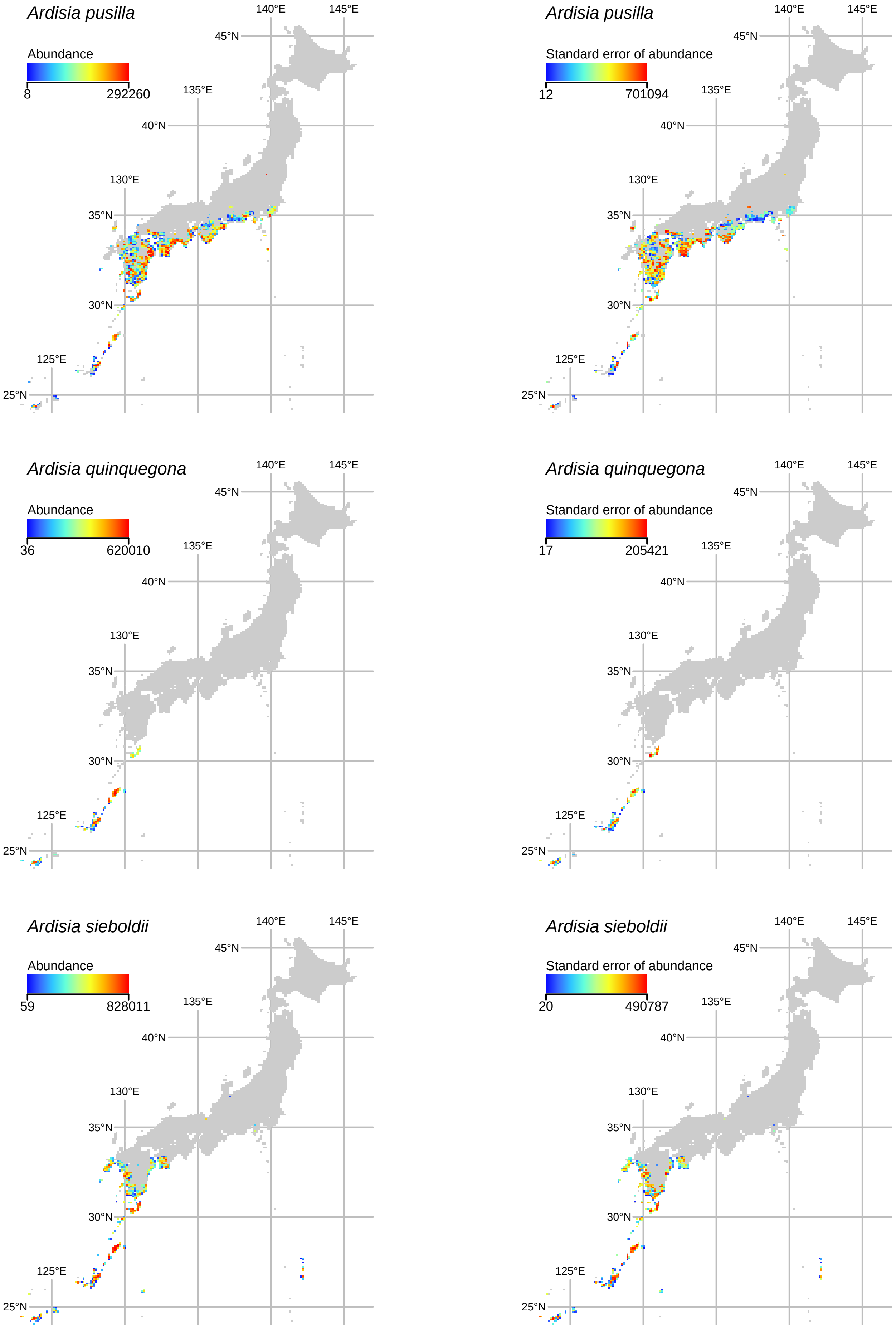
Ardisia pusilla
Abundance
8
292260
Ardisia pusilla
Standard error of abundance
12
701094
Ardisia quinquegona
Abundance
36
620010
Ardisia quinquegona
Standard error of abundance
17
205421
Ardisia sieboldii
Abundance
59
828011
Ardisia sieboldii
Standard error of abundance
20
490787
125°E
130°E
135°E
140°E
145°E
25°N
30°N
35°N
40°N
45°N

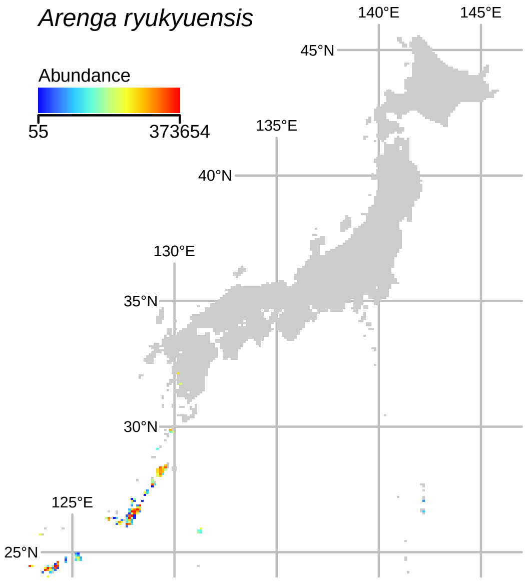
Arenga ryukyuensis
Abundance
55
373654
140°E
145°E
45°N
135°E
40°N
130°E
35°N
30°N
125°E
25°N

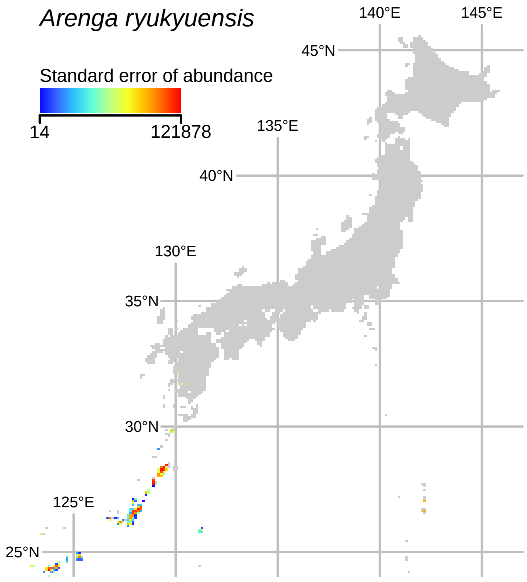
Arenga ryukyuensis
Standard error of abundance
14
121878
140°E
145°E
45°N
135°E
40°N
130°E
35°N
30°N
125°E
25°N

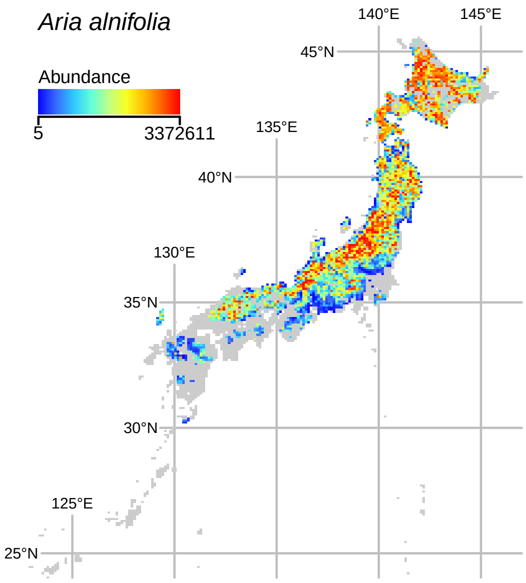
Aria alnifolia
Abundance
5
3372611
140°E
145°E
45°N
135°E
40°N
130°E
35°N
30°N
125°E
25°N

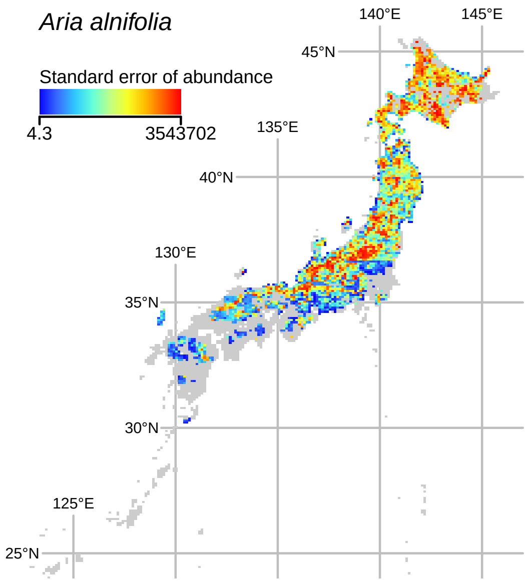
Aria alnifolia
Standard error of abundance
4.3
3543702
140°E
145°E
45°N
135°E
40°N
130°E
35°N
30°N
125°E
25°N

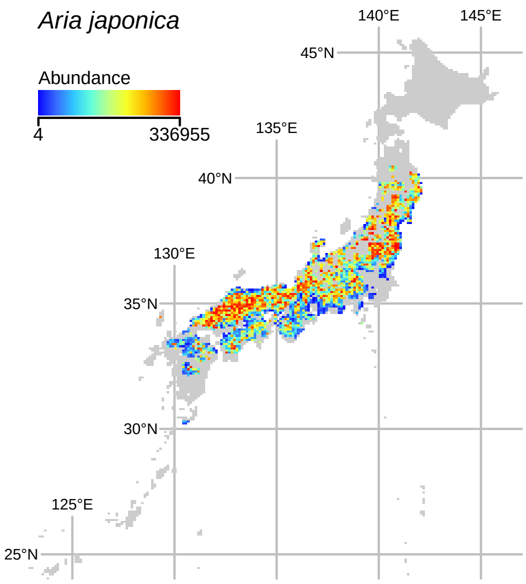
Aria japonica
Abundance
4
336955
140°E
145°E
45°N
135°E
40°N
130°E
35°N
30°N
125°E
25°N

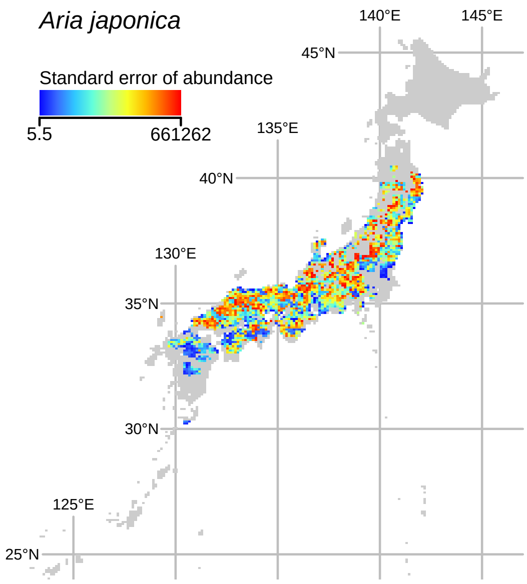
Aria japonica
Standard error of abundance
5.5
661262
140°E
145°E
45°N
135°E
40°N
130°E
35°N
30°N
125°E
25°N

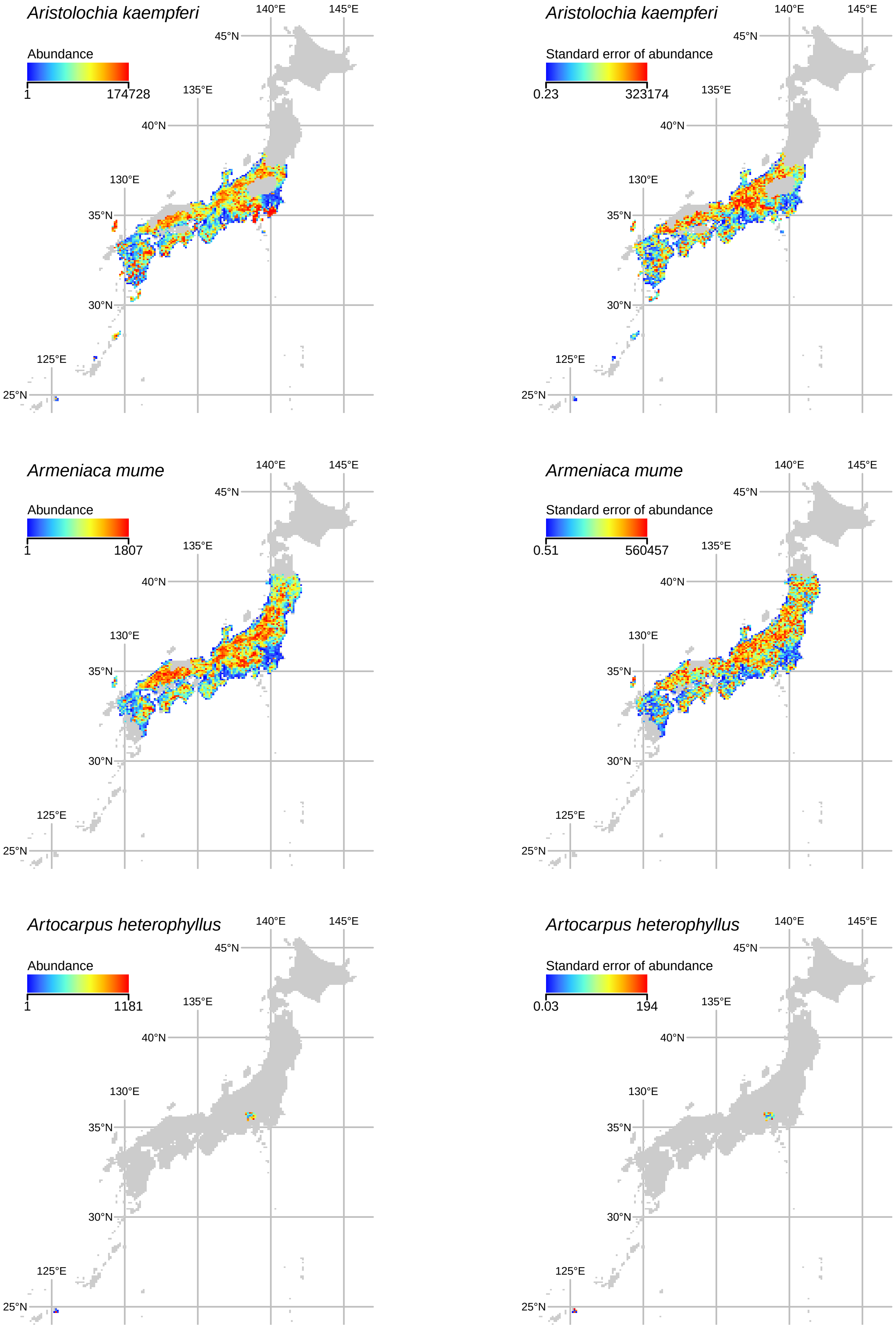
Aristolochia kaempferi
Abundance
1
174728
Aristolochia kaempferi
Standard error of abundance
0.23
323174
Armeniaca mume
Abundance
1
1807
Armeniaca mume
Standard error of abundance
0.51
560457
Artocarpus heterophyllus
Abundance
1
1181
Artocarpus heterophyllus
Standard error of abundance
0.03
194
140°E
145°E
135°E
130°E
125°E
45°N
40°N
35°N
30°N
25°N

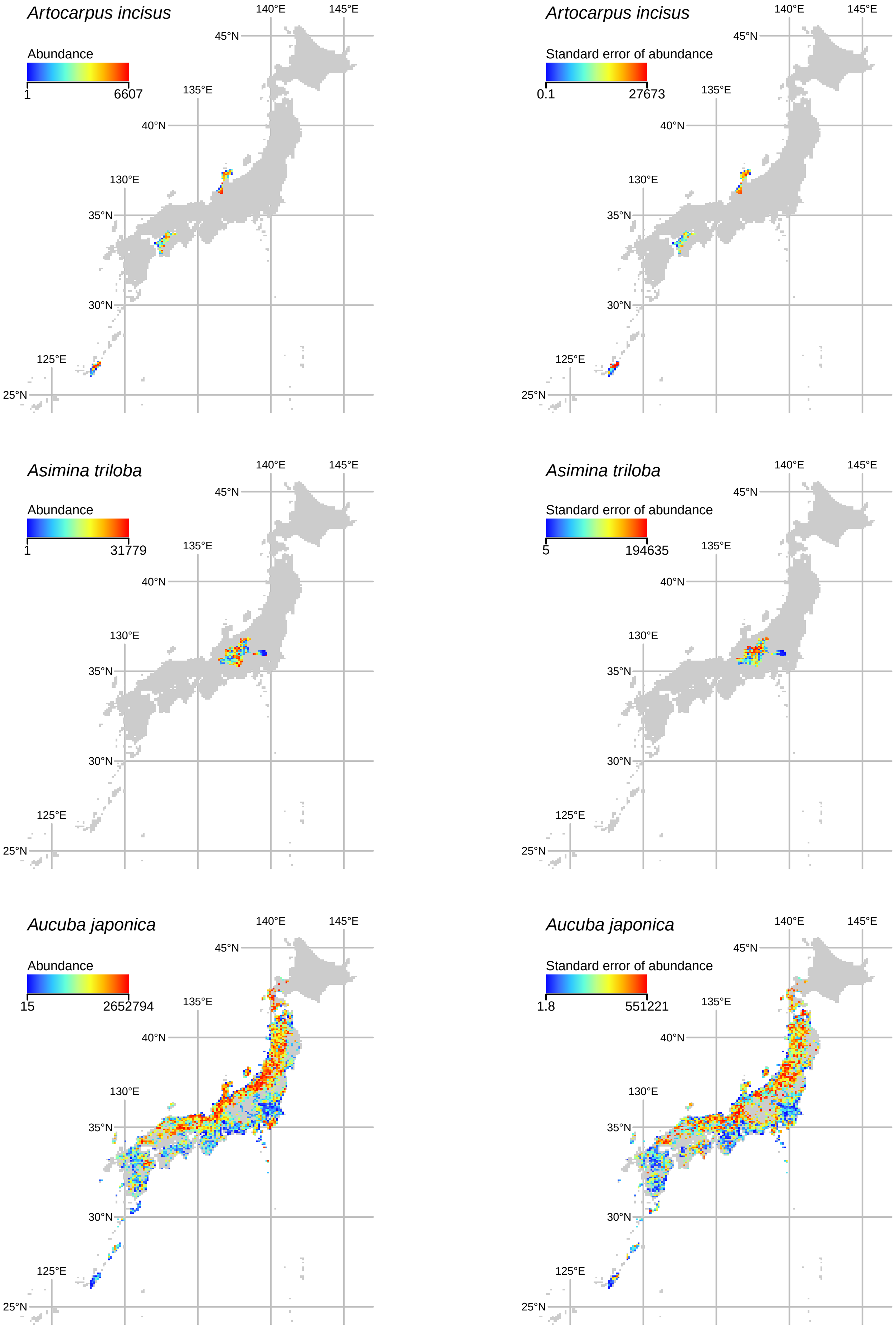
Artocarpus incisus
Abundance
1
6607
Artocarpus incisus
Standard error of abundance
0.1
27673
Asimina triloba
Abundance
1
31779
Asimina triloba
Standard error of abundance
5
194635
Aucuba japonica
Abundance
15
2652794
Aucuba japonica
Standard error of abundance
1.8
551221
125°E
130°E
135°E
140°E
145°E
25°N
30°N
35°N
40°N
45°N

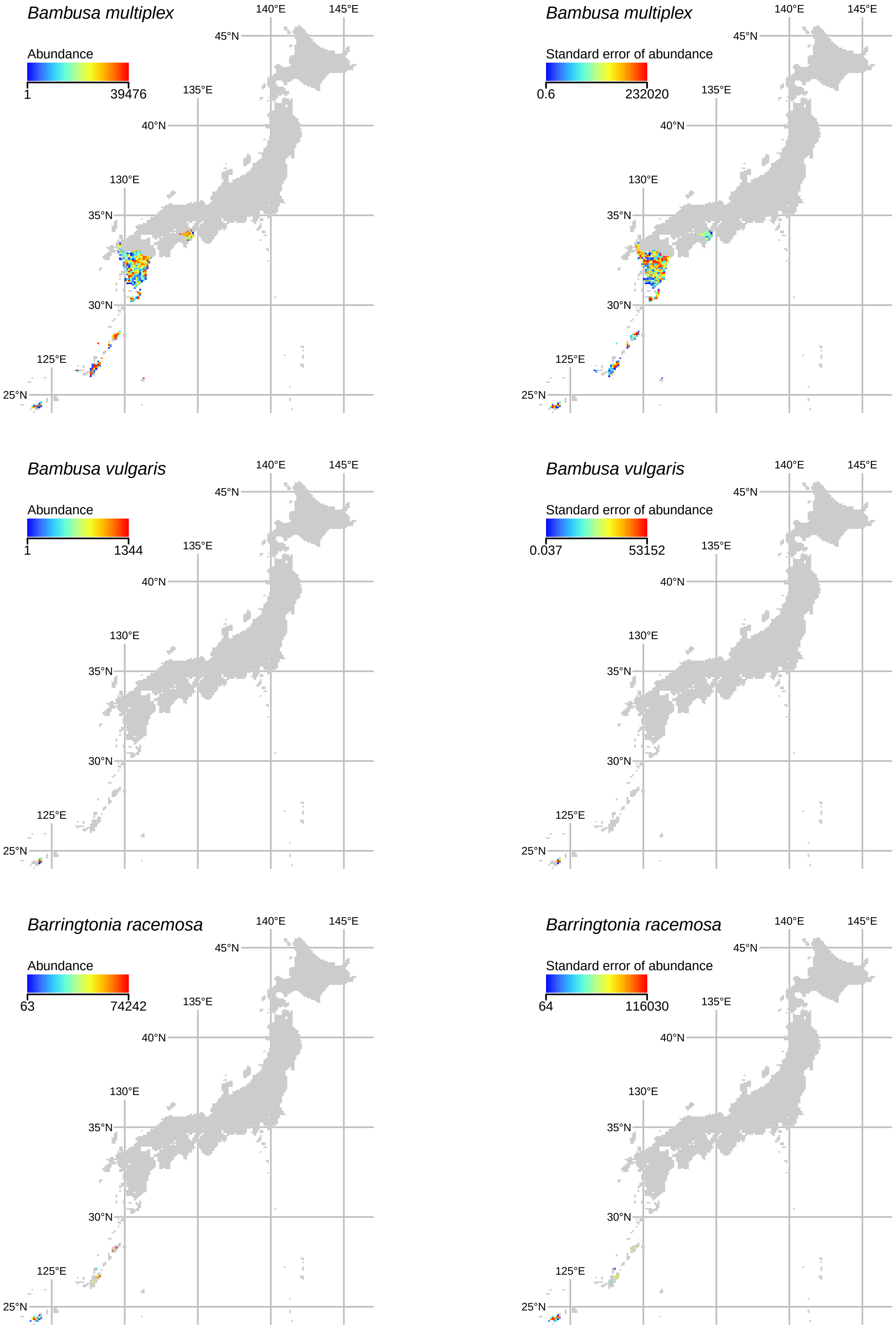
Bambusa multiplex
Abundance
1
39476
Bambusa multiplex
Standard error of abundance
0.6
232020
Bambusa vulgaris
Abundance
1
1344
Bambusa vulgaris
Standard error of abundance
0.037
53152
Barringtonia racemosa
Abundance
63
74242
Barringtonia racemosa
Standard error of abundance
64
116030
140°E
145°E
45°N
135°E
40°N
130°E
35°N
30°N
125°E
25°N

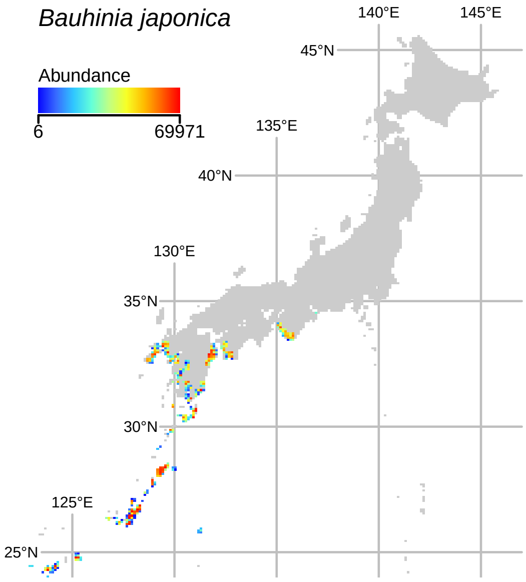
Bauhinia japonica
Abundance
6
69971
140°E
145°E
45°N
135°E
40°N
130°E
35°N
30°N
125°E
25°N

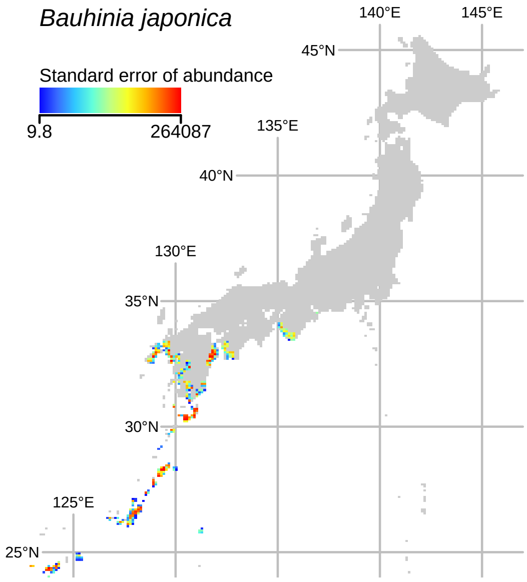
Bauhinia japonica
Standard error of abundance
9.8
264087
140°E
145°E
45°N
135°E
40°N
130°E
35°N
30°N
125°E
25°N

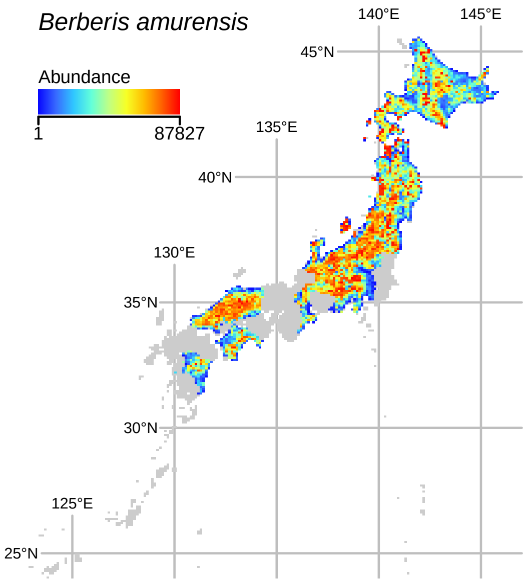
Berberis amurensis
Abundance
1
87827
140°E
145°E
45°N
135°E
40°N
130°E
35°N
30°N
125°E
25°N

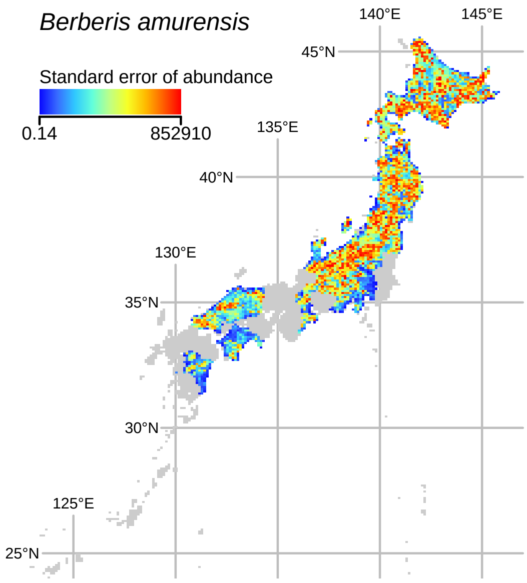
Berberis amurensis
Standard error of abundance
0.14
852910
140°E
145°E
45°N
135°E
40°N
130°E
35°N
30°N
125°E
25°N

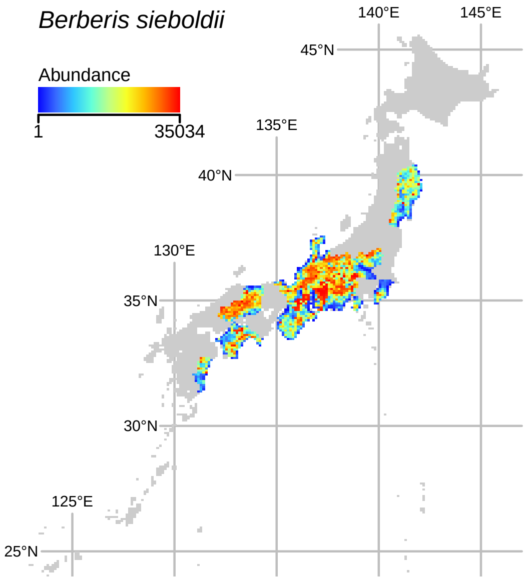
Berberis sieboldii
Abundance
1
35034
140°E
145°E
45°N
135°E
40°N
130°E
35°N
30°N
125°E
25°N

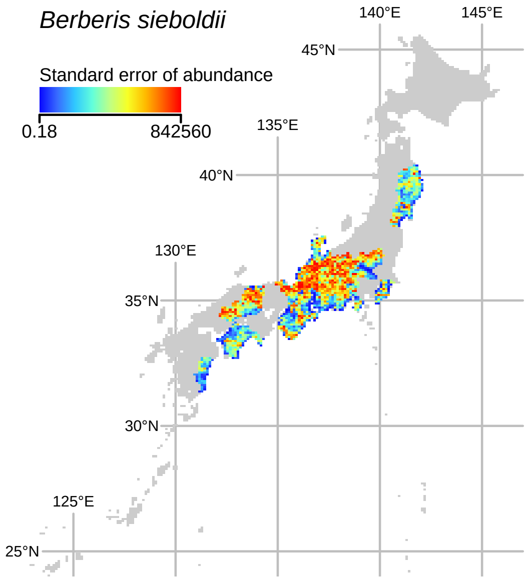
Berberis sieboldii
Standard error of abundance
0.18
842560
140°E
145°E
45°N
135°E
40°N
130°E
35°N
30°N
125°E
25°N

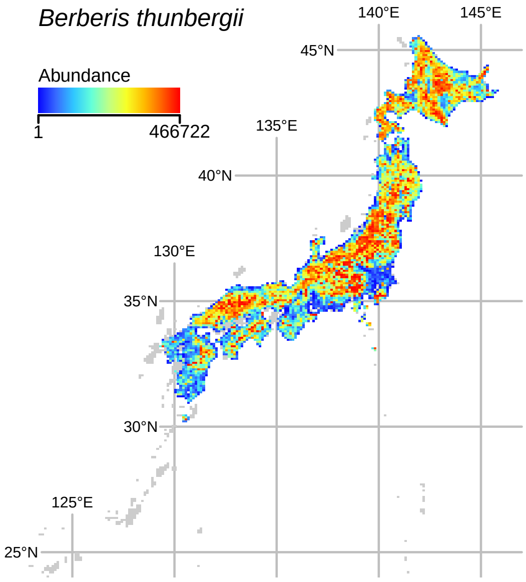
Berberis thunbergii
Abundance
1
466722
140°E
145°E
135°E
130°E
125°E
45°N
40°N
35°N
30°N
25°N

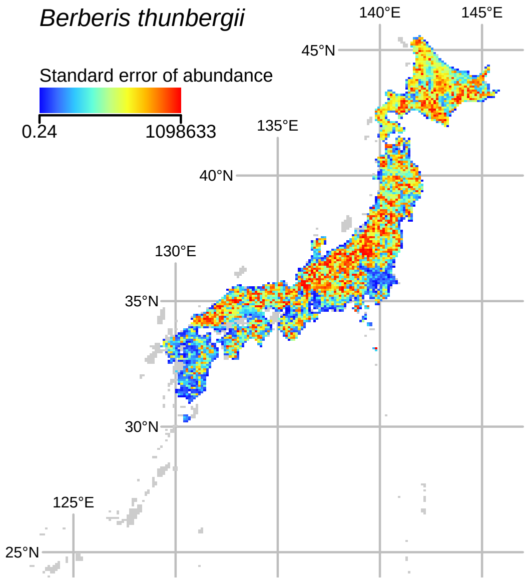
Berberis thunbergii
Standard error of abundance
0.24
1098633
140°E
145°E
135°E
130°E
125°E
45°N
40°N
35°N
30°N
25°N

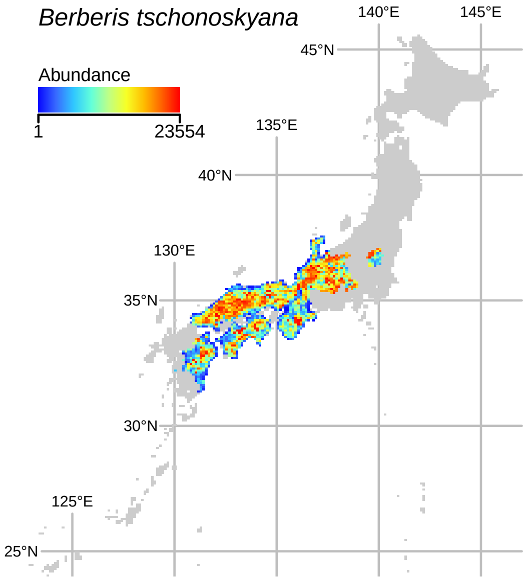
Berberis tschonoskyana
Abundance
1
23554
140°E
145°E
135°E
130°E
125°E
45°N
40°N
35°N
30°N
25°N

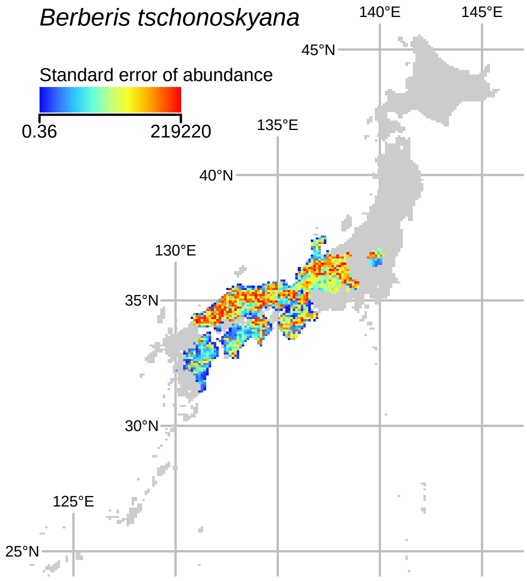
Berberis tschonoskyana
Standard error of abundance
0.36
219220
140°E
145°E
135°E
130°E
125°E
45°N
40°N
35°N
30°N
25°N

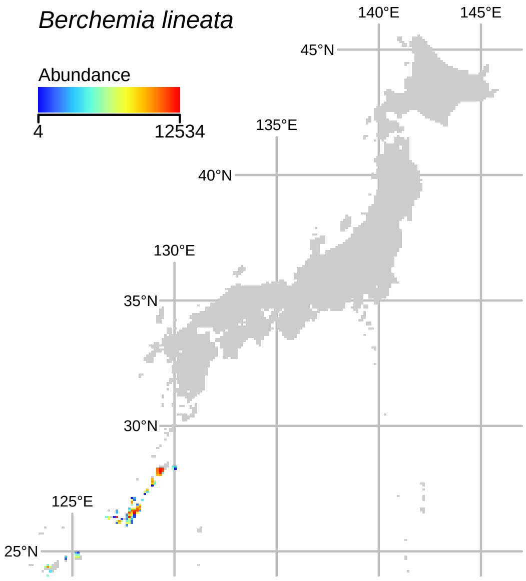
Berchemia lineata
Abundance
4
12534
140°E
145°E
135°E
130°E
125°E
45°N
40°N
35°N
30°N
25°N

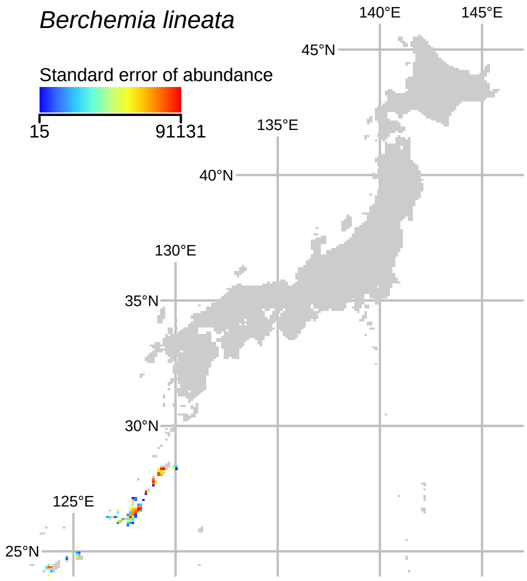
Berchemia lineata
Standard error of abundance
15
91131
140°E
145°E
135°E
130°E
125°E
45°N
40°N
35°N
30°N
25°N

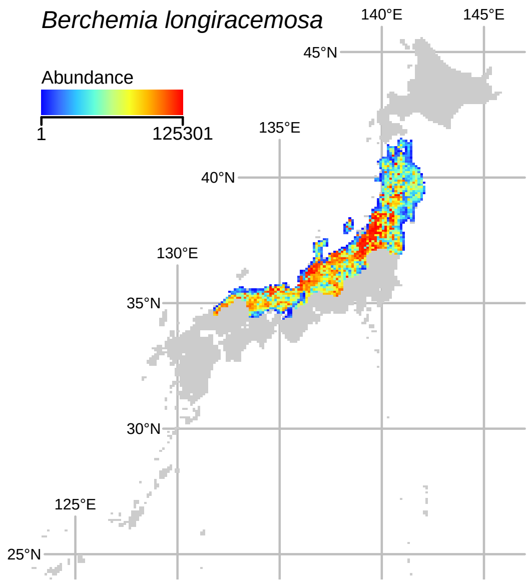
Berchemia longiracemosa
Abundance
1
125301
140°E
145°E
45°N
135°E
40°N
130°E
35°N
30°N
125°E
25°N

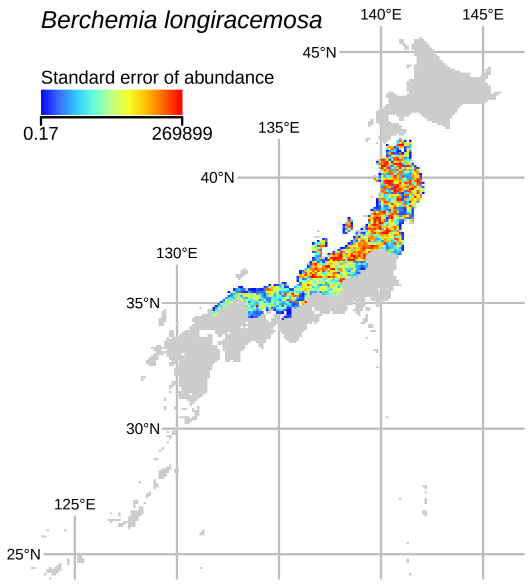
Berchemia longiracemosa
Standard error of abundance
0.17
269899
140°E
145°E
45°N
135°E
40°N
130°E
35°N
30°N
125°E
25°N

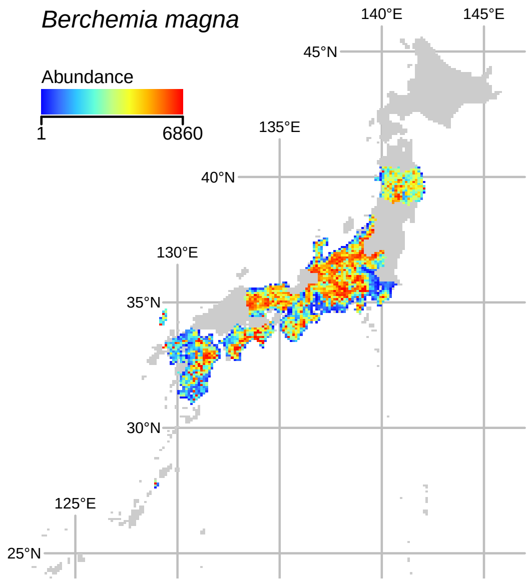
Berchemia magna
Abundance
1
6860
140°E
145°E
45°N
135°E
40°N
130°E
35°N
30°N
125°E
25°N

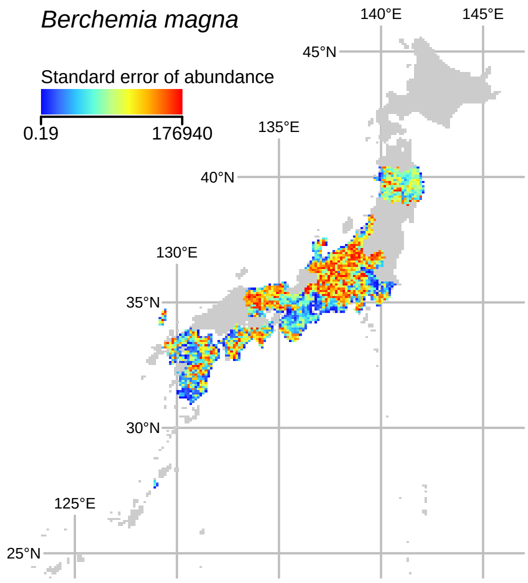
Berchemia magna
Standard error of abundance
0.19
176940
140°E
145°E
45°N
135°E
40°N
130°E
35°N
30°N
125°E
25°N

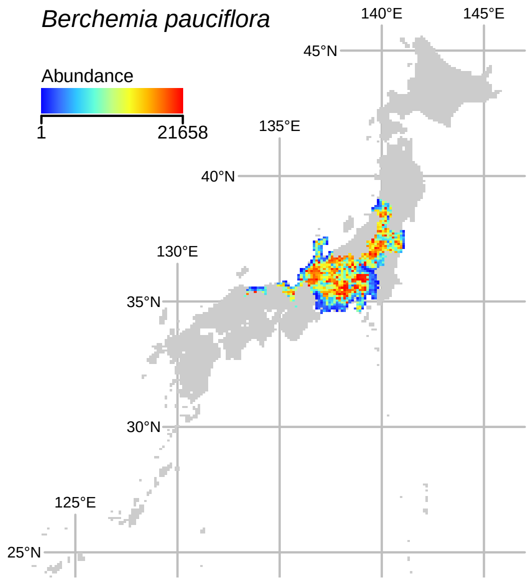
Berchemia pauciflora
Abundance
1
21658
140°E
145°E
45°N
135°E
40°N
130°E
35°N
30°N
125°E
25°N

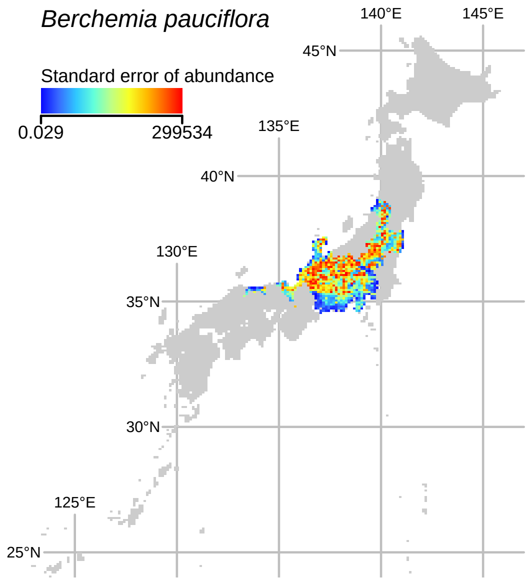
Berchemia pauciflora
Standard error of abundance
0.029
299534
140°E
145°E
45°N
135°E
40°N
130°E
35°N
30°N
125°E
25°N

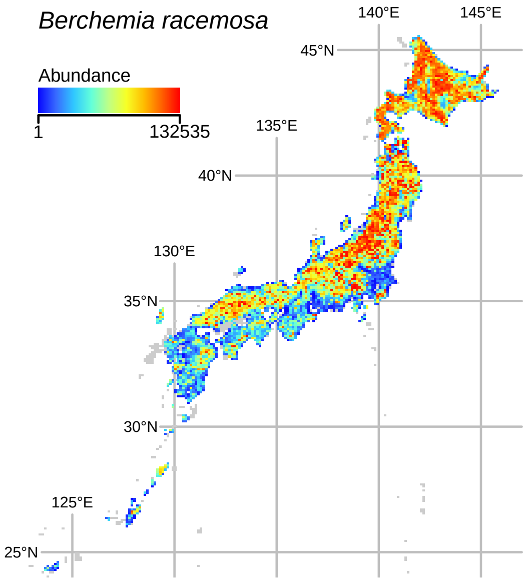
Berchemia racemosa
Abundance
1
132535
140°E
145°E
135°E
130°E
125°E
45°N
40°N
35°N
30°N
25°N

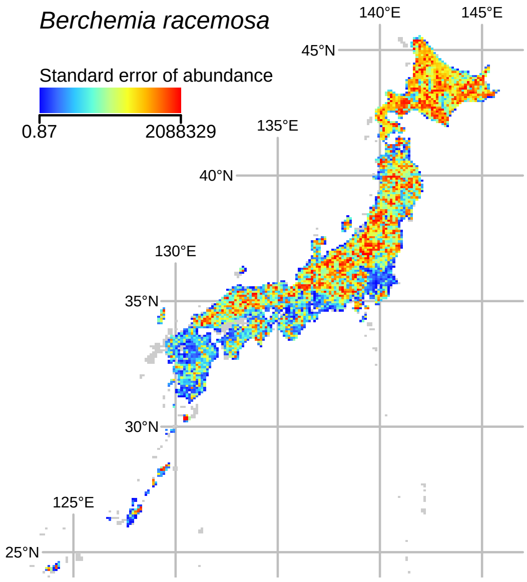
Berchemia racemosa
Standard error of abundance
0.87
2088329
140°E
145°E
135°E
130°E
125°E
45°N
40°N
35°N
30°N
25°N

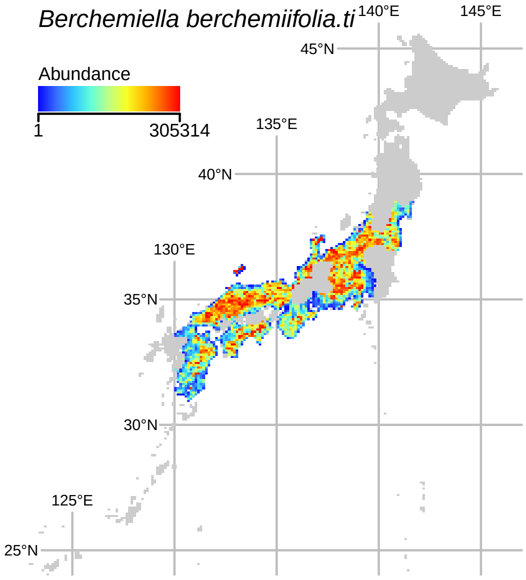
Berchemiella berchemiifolia.ti
Abundance
1
305314
140°E
145°E
135°E
130°E
125°E
45°N
40°N
35°N
30°N
25°N

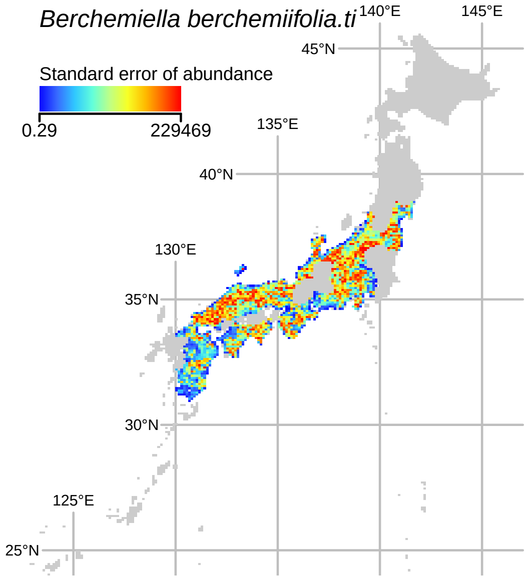
Berchemiella berchemiifolia.ti
Standard error of abundance
0.29
229469
140°E
145°E
135°E
130°E
125°E
45°N
40°N
35°N
30°N
25°N

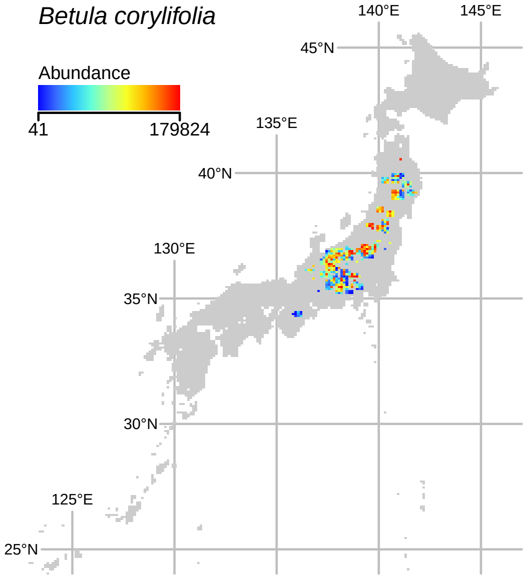
Betula corylifolia
Abundance
41
179824
140°E
145°E
135°E
130°E
125°E
45°N
40°N
35°N
30°N
25°N

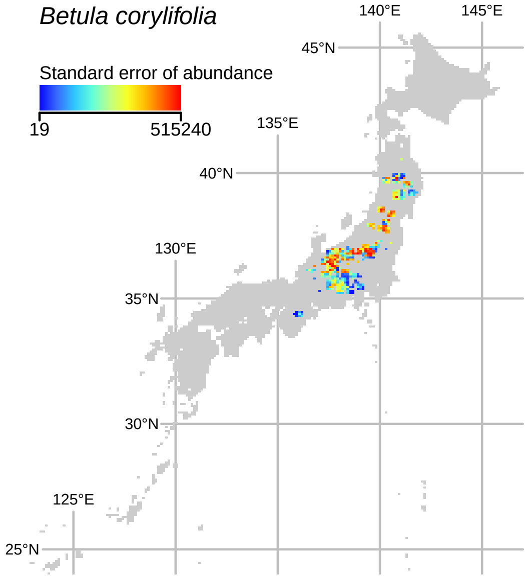
Betula corylifolia
Standard error of abundance
19
515240
140°E
145°E
135°E
130°E
125°E
45°N
40°N
35°N
30°N
25°N

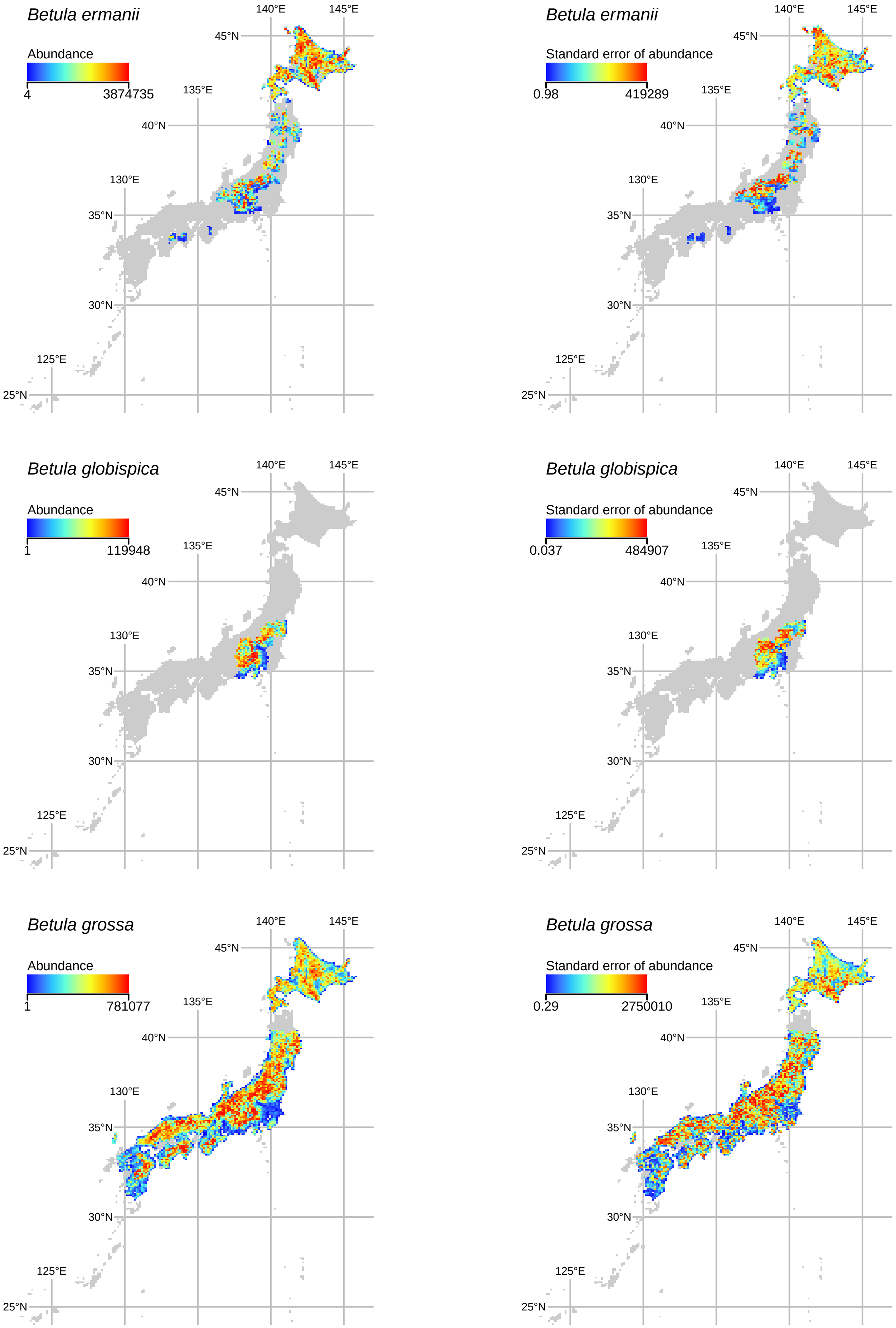
Betula ermanii
Abundance
4
3874735
Betula ermanii
Standard error of abundance
0.98
419289
Betula globispica
Abundance
1
119948
Betula globispica
Standard error of abundance
0.037
484907
Betula grossa
Abundance
1
781077
Betula grossa
Standard error of abundance
0.29
2750010
125°E
130°E
135°E
140°E
145°E
25°N
30°N
35°N
40°N
45°N

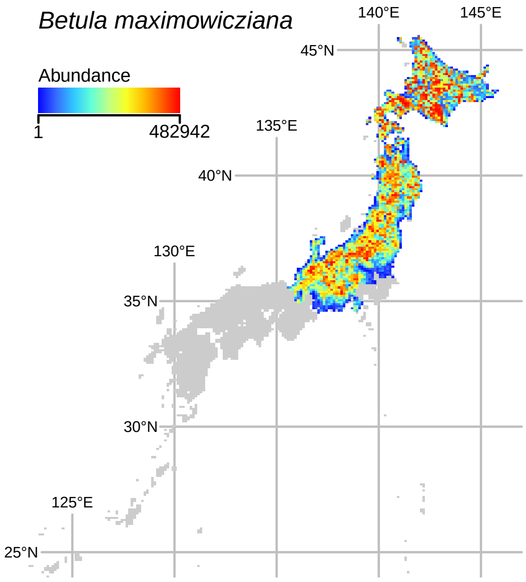
Betula maximowicziana
Abundance
1
482942
140°E
145°E
135°E
130°E
125°E
45°N
40°N
35°N
30°N
25°N

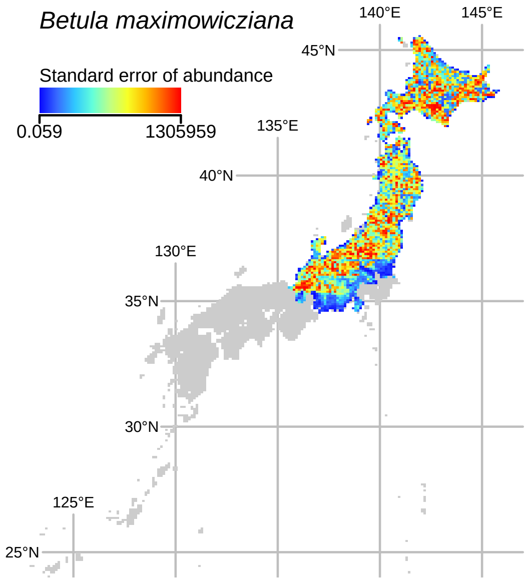
Betula maximowicziana
Standard error of abundance
0.059
1305959
140°E
145°E
135°E
130°E
125°E
45°N
40°N
35°N
30°N
25°N

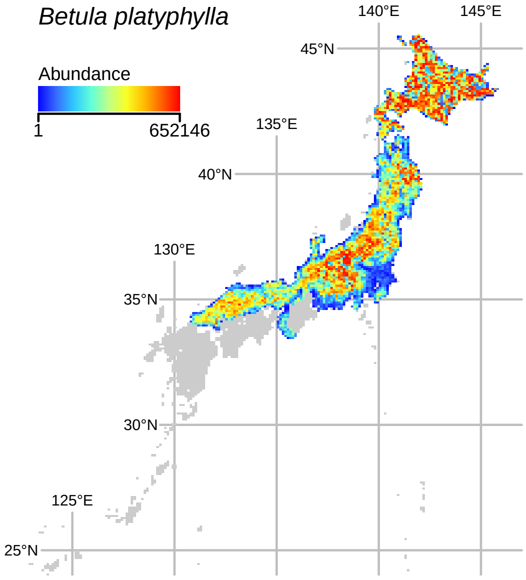
Betula platyphylla
Abundance
1
652146
140°E
145°E
135°E
130°E
125°E
45°N
40°N
35°N
30°N
25°N

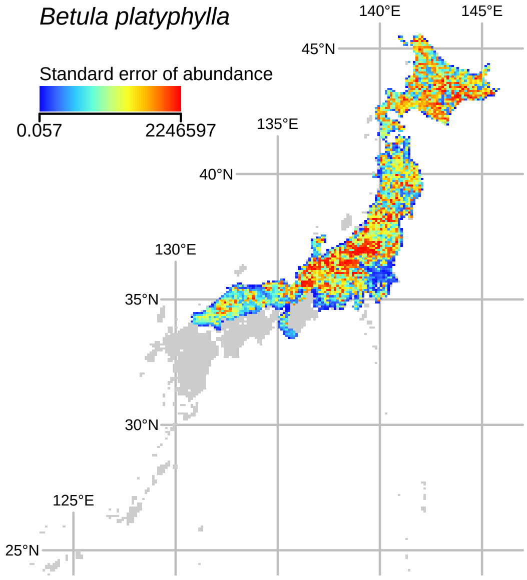
Betula platyphylla
Standard error of abundance
0.057
2246597
140°E
145°E
135°E
130°E
125°E
45°N
40°N
35°N
30°N
25°N

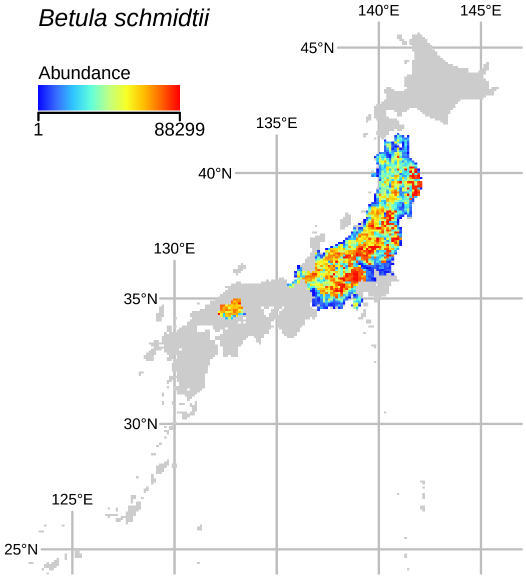
Betula schmidtii
Abundance
1
88299
140°E
145°E
135°E
130°E
125°E
45°N
40°N
35°N
30°N
25°N

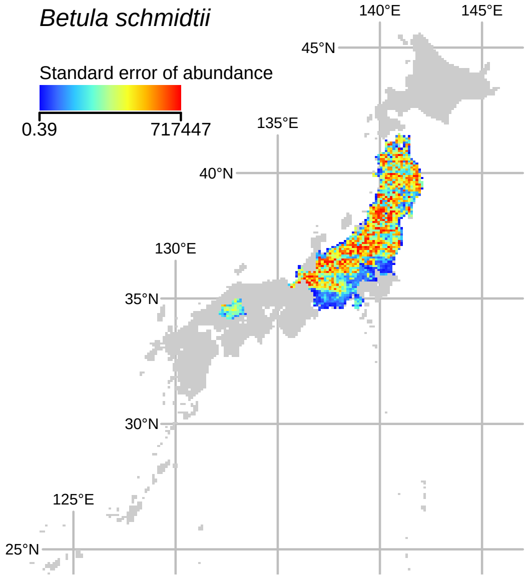
Betula schmidtii
Standard error of abundance
0.39
717447
140°E
145°E
135°E
130°E
125°E
45°N
40°N
35°N
30°N
25°N

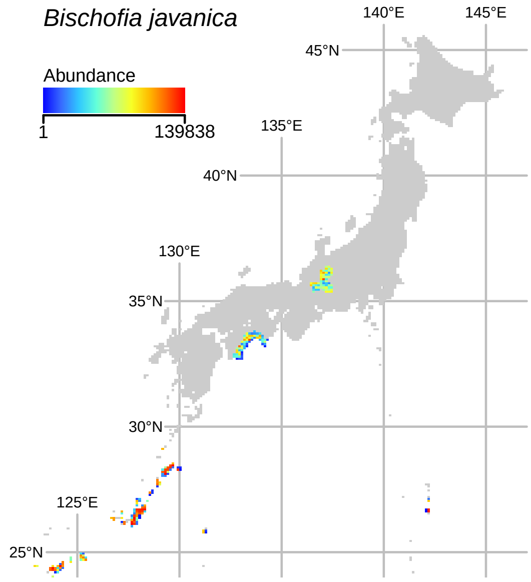
Bischofia javanica
Abundance
1
139838
140°E
145°E
45°N
135°E
40°N
130°E
35°N
30°N
125°E
25°N

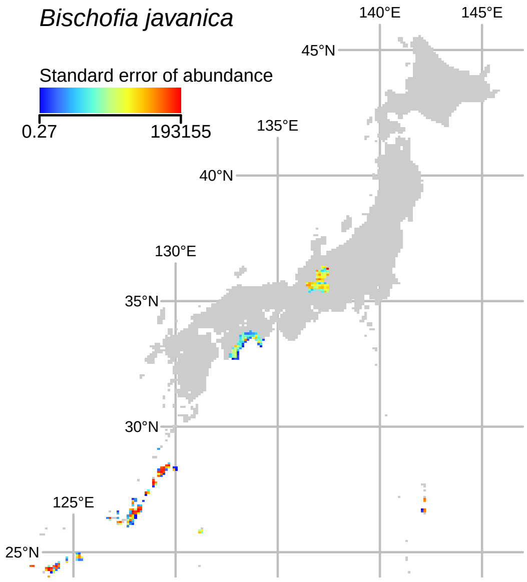
Bischofia javanica
Standard error of abundance
0.27
193155
140°E
145°E
45°N
135°E
40°N
130°E
35°N
30°N
125°E
25°N

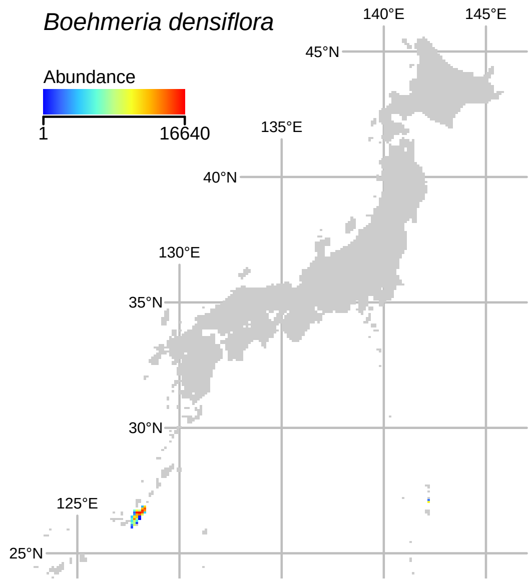
Boehmeria densiflora
Abundance
1
16640
140°E
145°E
45°N
135°E
40°N
130°E
35°N
30°N
125°E
25°N

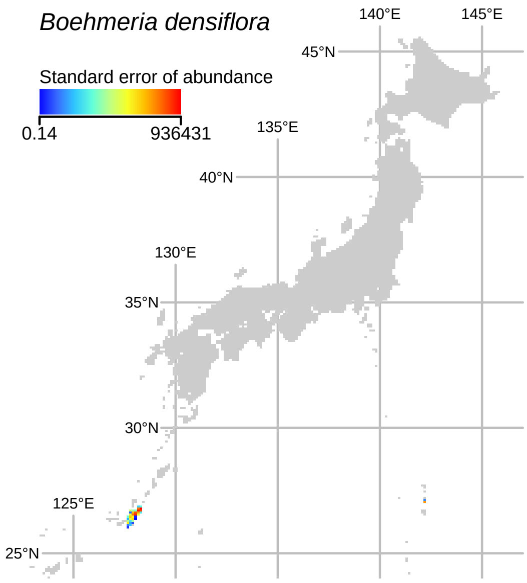
Boehmeria densiflora
Standard error of abundance
0.14
936431
140°E
145°E
45°N
135°E
40°N
130°E
35°N
30°N
125°E
25°N

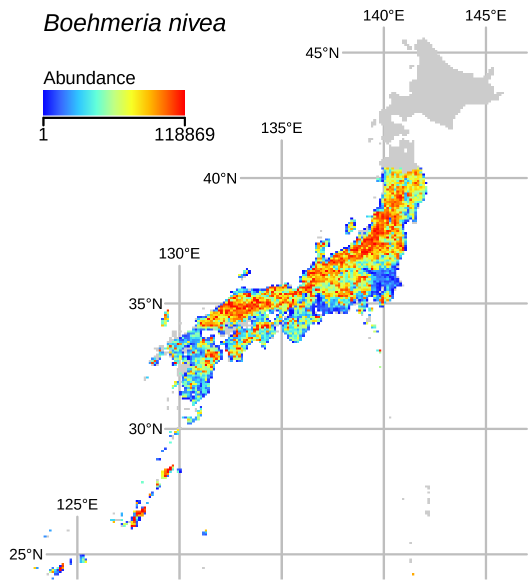
Boehmeria nivea
Abundance
1
118869
140°E
145°E
45°N
135°E
40°N
130°E
35°N
30°N
125°E
25°N

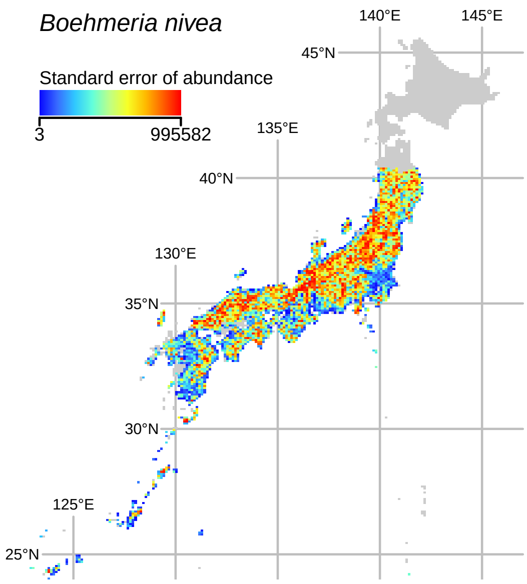
Boehmeria nivea
Standard error of abundance
3
995582
140°E
145°E
45°N
135°E
40°N
130°E
35°N
30°N
125°E
25°N

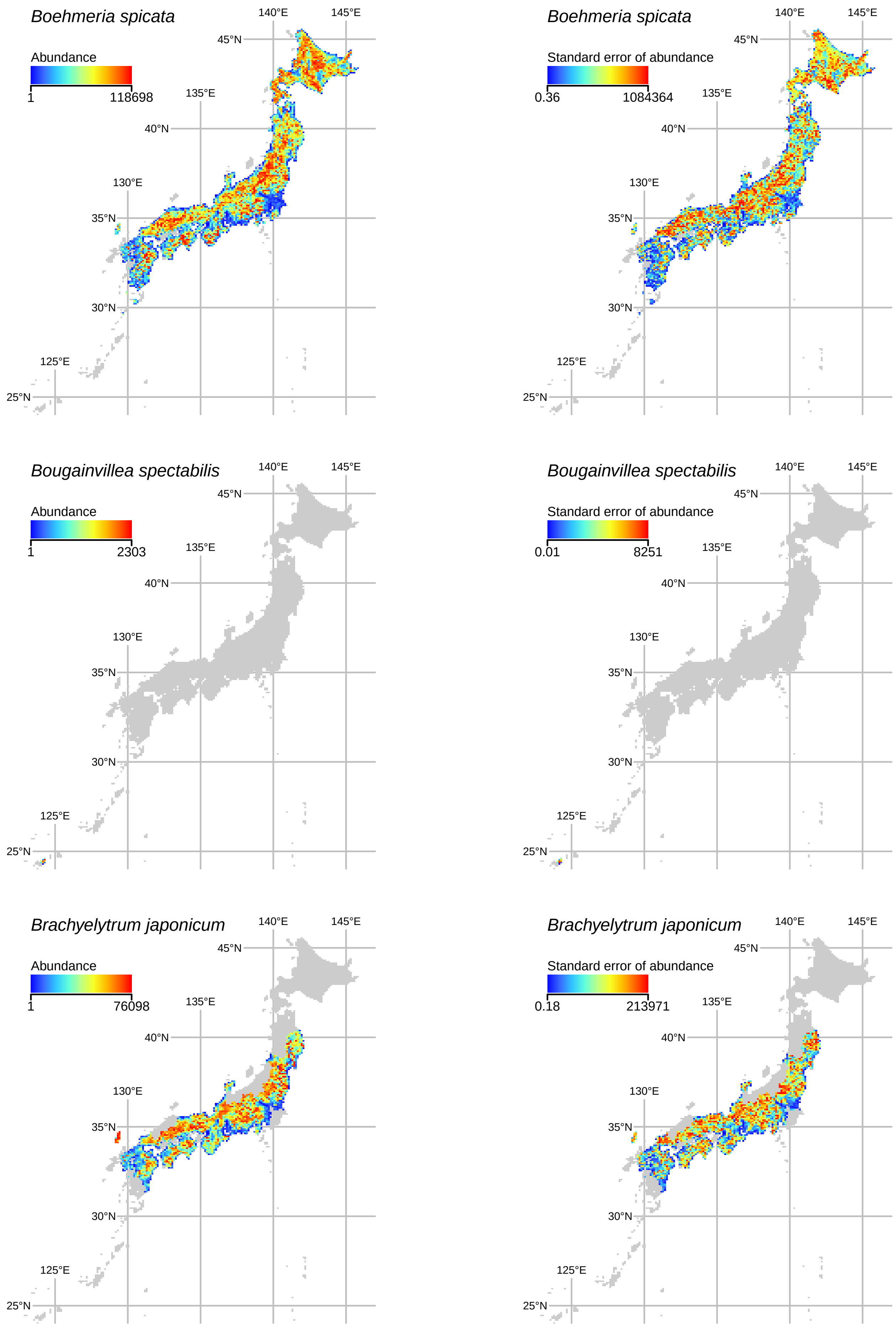
Boehmeria spicata
Abundance
1
118698
Boehmeria spicata
Standard error of abundance
0.36
1084364
Bougainvillea spectabilis
Abundance
1
2303
Bougainvillea spectabilis
Standard error of abundance
0.01
8251
Brachyelytrum japonicum
Abundance
1
76098
Brachyelytrum japonicum
Standard error of abundance
0.18
213971
125°E
130°E
135°E
140°E
145°E
25°N
30°N
35°N
40°N
45°N

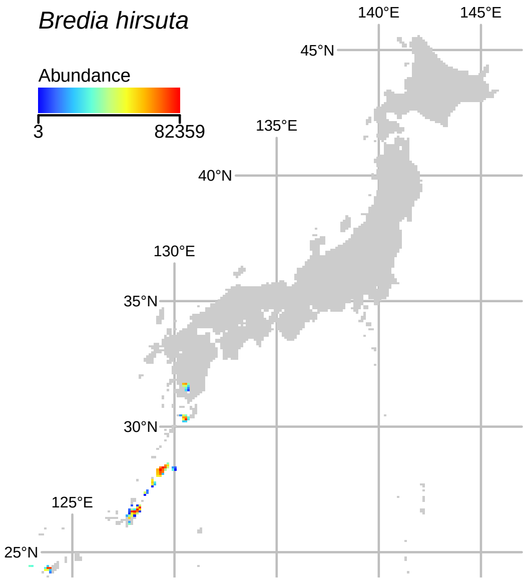
Bredia hirsuta
Abundance
3
82359
140°E
145°E
45°N
135°E
40°N
130°E
35°N
30°N
125°E
25°N

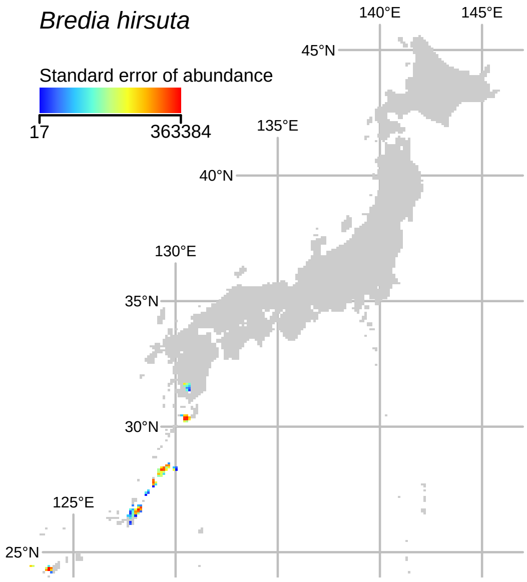
Bredia hirsuta
Standard error of abundance
17
363384
140°E
145°E
45°N
135°E
40°N
130°E
35°N
30°N
125°E
25°N

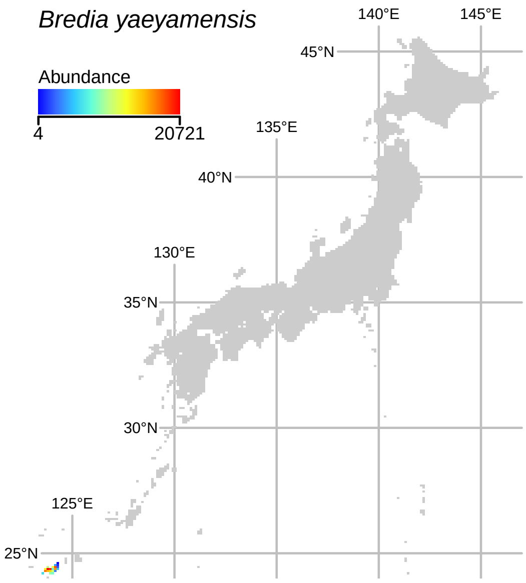
Bredia yaeyamensis
Abundance
4
20721
140°E
145°E
45°N
135°E
40°N
130°E
35°N
30°N
125°E
25°N

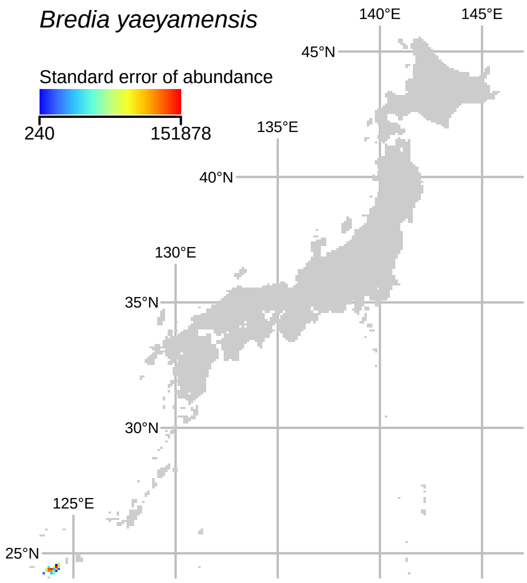
Bredia yaeyamensis
Standard error of abundance
240
151878
140°E
145°E
45°N
135°E
40°N
130°E
35°N
30°N
125°E
25°N

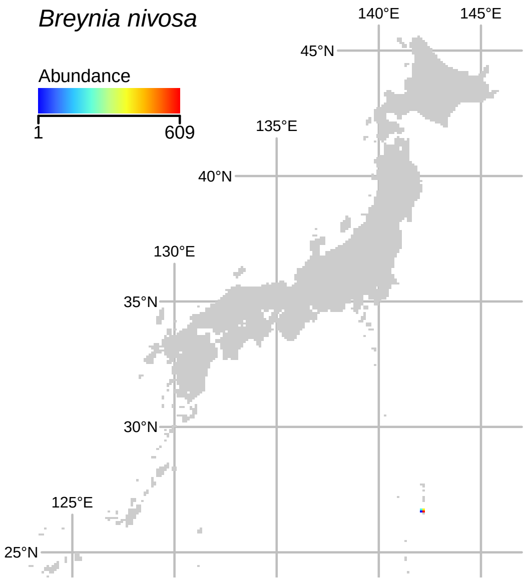
Breynia nivosa
Abundance
1
609
140°E
145°E
45°N
135°E
40°N
130°E
35°N
30°N
125°E
25°N

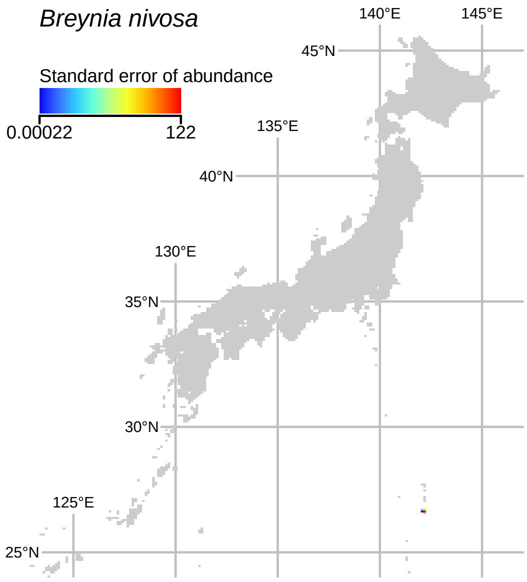
Breynia nivosa
Standard error of abundance
0.00022
122
140°E
145°E
45°N
135°E
40°N
130°E
35°N
30°N
125°E
25°N

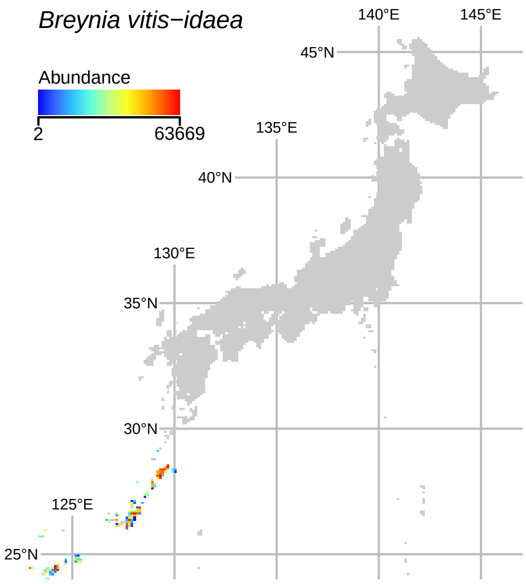
Breynia vitis−idaea
Abundance
2
63669
140°E
145°E
45°N
135°E
40°N
130°E
35°N
30°N
125°E
25°N

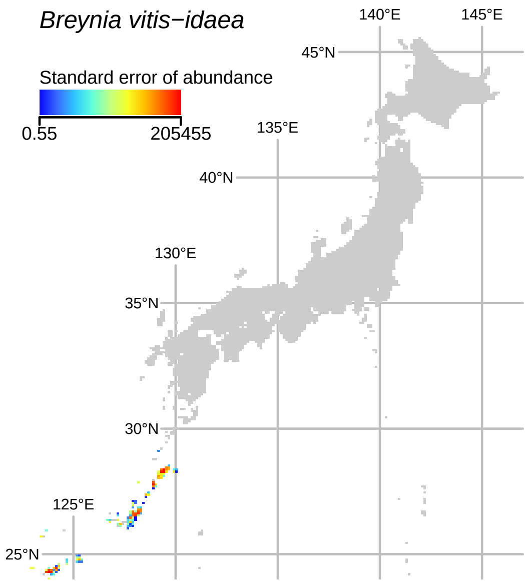
Breynia vitis−idaea
Standard error of abundance
0.55
205455
140°E
145°E
45°N
135°E
40°N
130°E
35°N
30°N
125°E
25°N

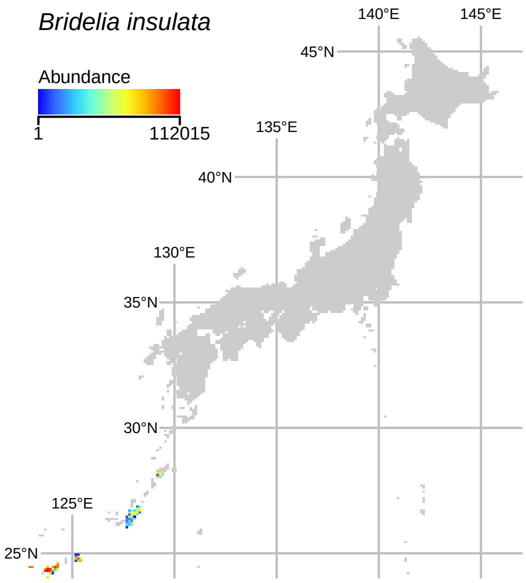
Bridelia insulata
Abundance
1
112015
140°E
145°E
45°N
135°E
40°N
130°E
35°N
30°N
125°E
25°N

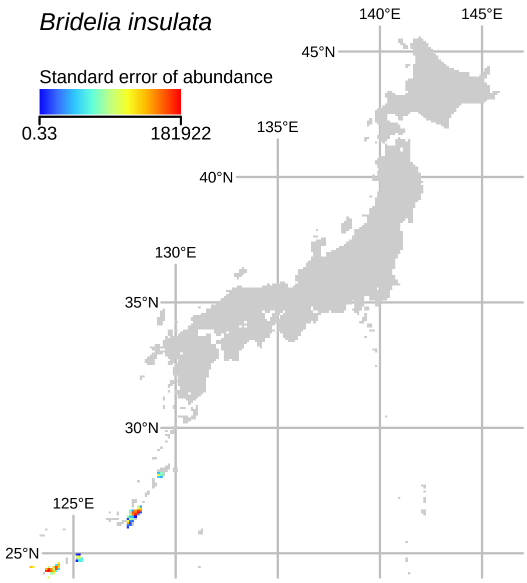
Bridelia insulata
Standard error of abundance
0.33
181922
140°E
145°E
45°N
135°E
40°N
130°E
35°N
30°N
125°E
25°N

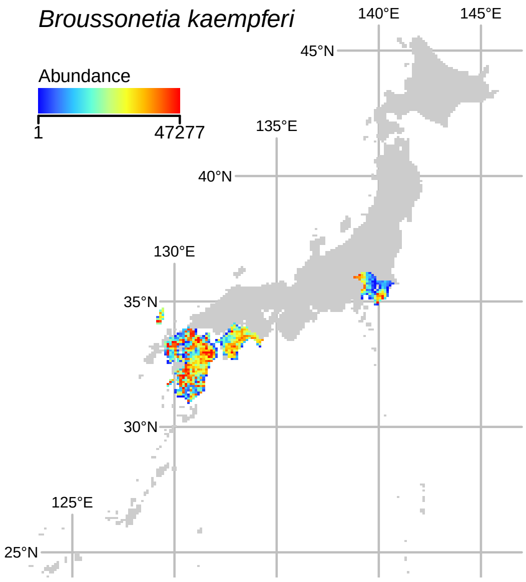
Broussonetia kaempferi
Abundance
1
47277
140°E
145°E
45°N
135°E
40°N
130°E
35°N
30°N
125°E
25°N

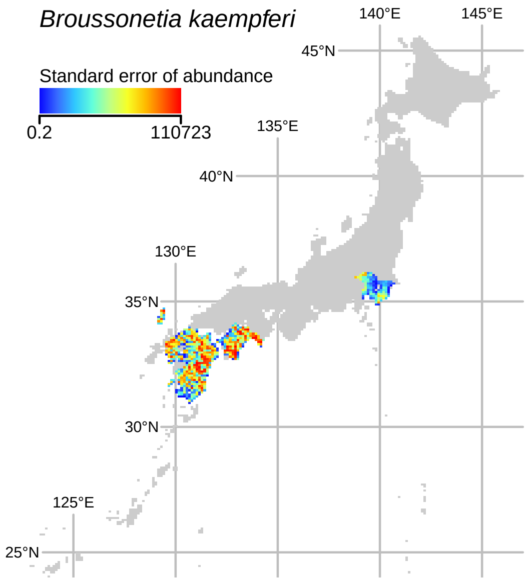
Broussonetia kaempferi
Standard error of abundance
0.2
110723
140°E
145°E
45°N
135°E
40°N
130°E
35°N
30°N
125°E
25°N

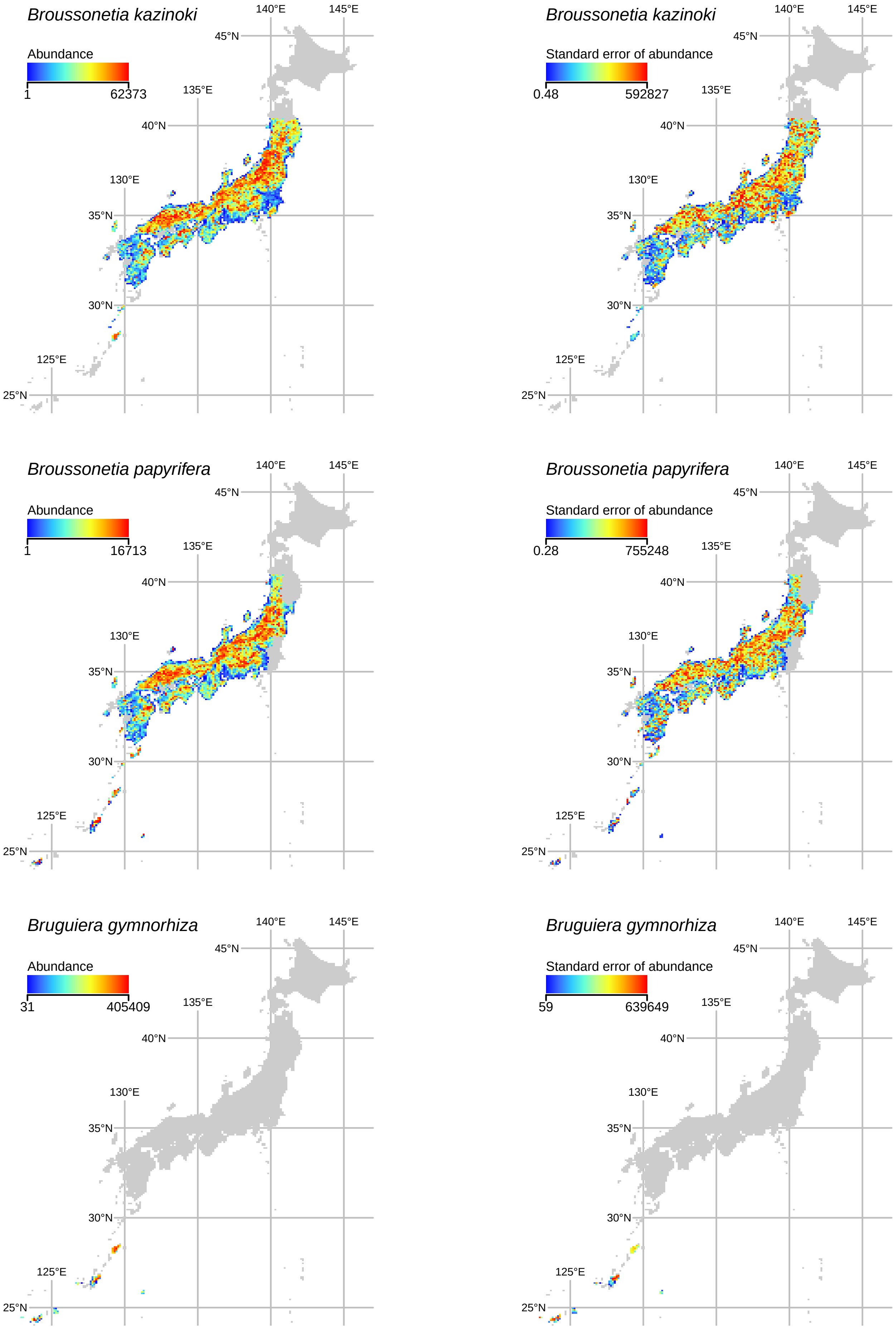
Broussonetia kazinoki
Abundance
1
62373
Broussonetia kazinoki
Standard error of abundance
0.48
592827
Broussonetia papyrifera
Abundance
1
16713
Broussonetia papyrifera
Standard error of abundance
0.28
755248
Bruguiera gymnorhiza
Abundance
31
405409
Bruguiera gymnorhiza
Standard error of abundance
59
639649
125°E
130°E
135°E
140°E
145°E
25°N
30°N
35°N
40°N
45°N

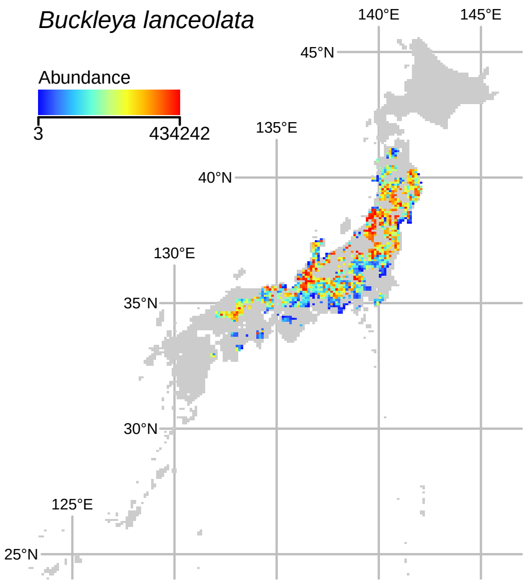
Buckleya lanceolata
Abundance
3
434242
140°E
145°E
45°N
135°E
40°N
130°E
35°N
30°N
125°E
25°N

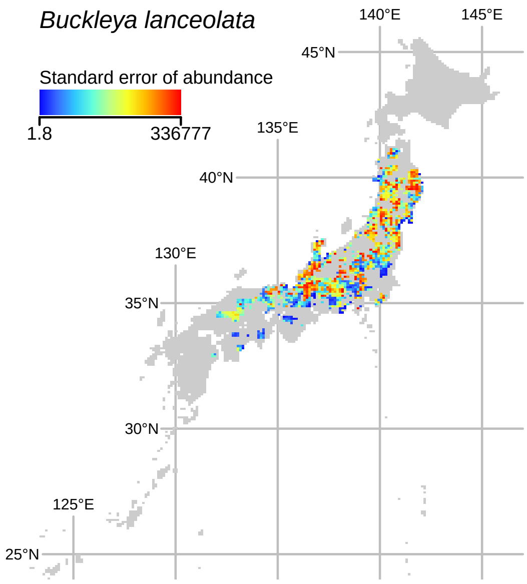
Buckleya lanceolata
Standard error of abundance
1.8
336777
140°E
145°E
45°N
135°E
40°N
130°E
35°N
30°N
125°E
25°N

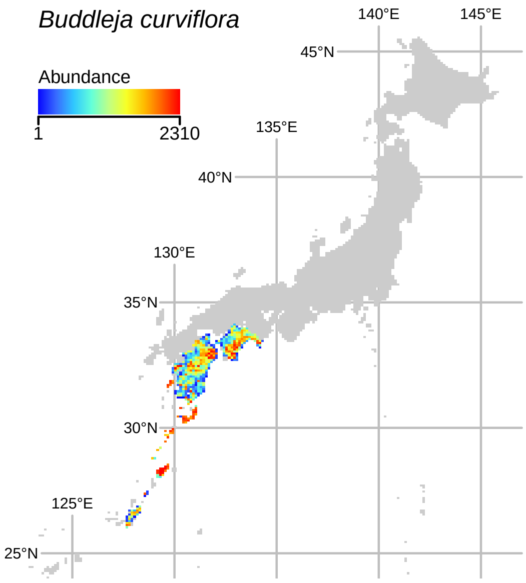
Buddleja curviflora
Abundance
1
2310
140°E
145°E
45°N
135°E
40°N
130°E
35°N
30°N
125°E
25°N

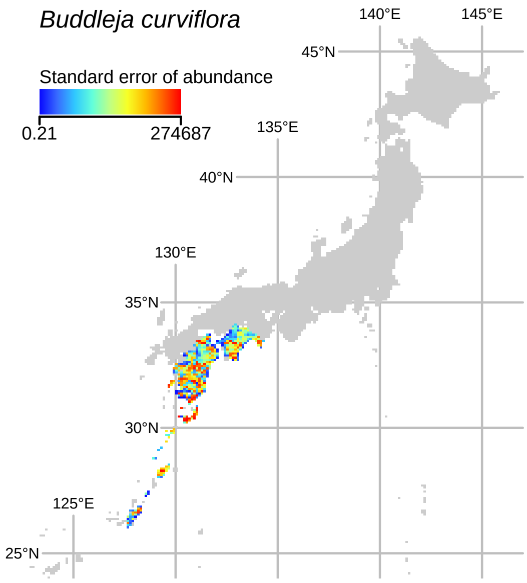
Buddleja curviflora
Standard error of abundance
0.21
274687
140°E
145°E
45°N
135°E
40°N
130°E
35°N
30°N
125°E
25°N

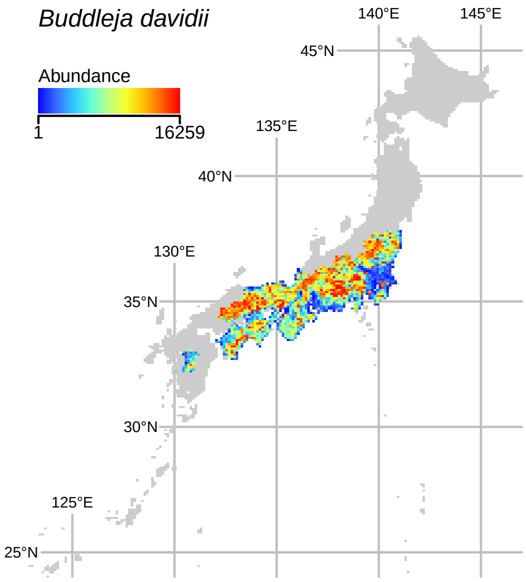
Buddleja davidii
Abundance
1
16259
140°E
145°E
45°N
135°E
40°N
130°E
35°N
30°N
125°E
25°N

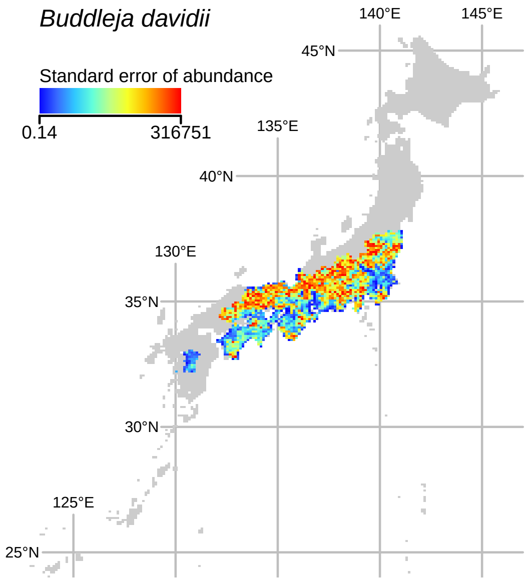
Buddleja davidii
Standard error of abundance
0.14
316751
140°E
145°E
45°N
135°E
40°N
130°E
35°N
30°N
125°E
25°N

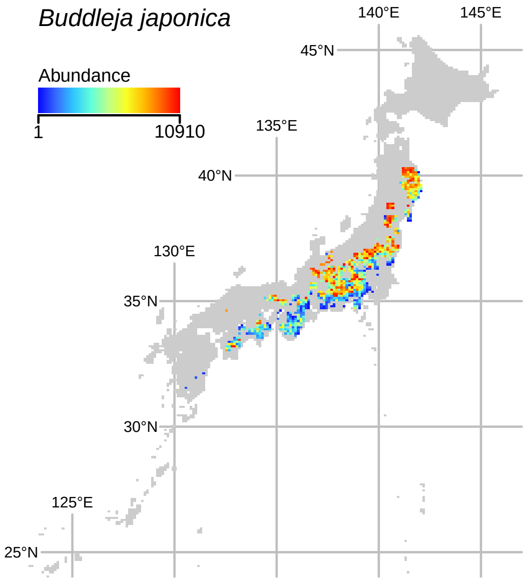
Buddleja japonica
Abundance
1
10910
140°E
145°E
135°E
130°E
125°E
45°N
40°N
35°N
30°N
25°N

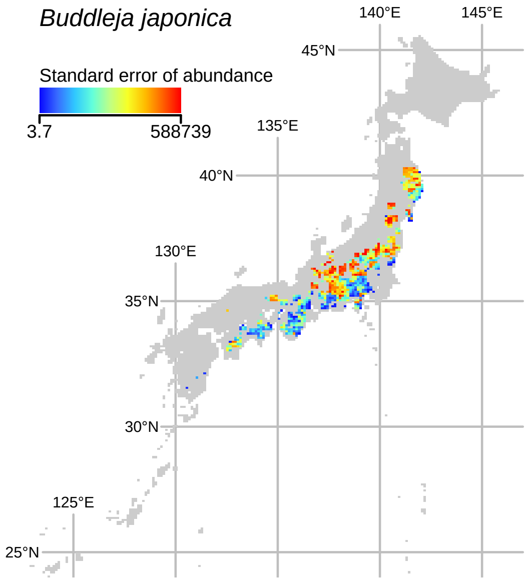
Buddleja japonica
Standard error of abundance
3.7
588739
140°E
145°E
135°E
130°E
125°E
45°N
40°N
35°N
30°N
25°N

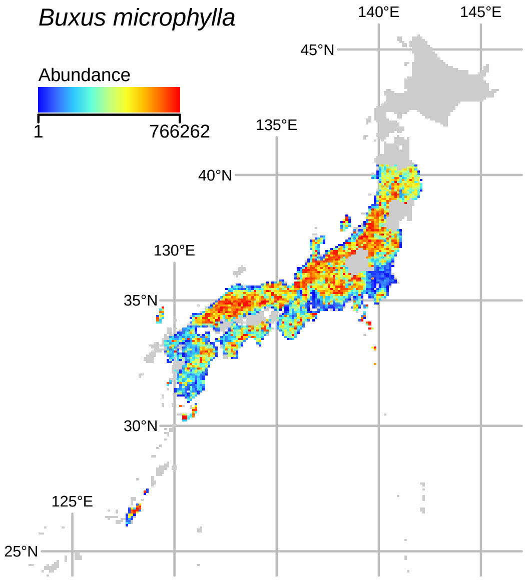
Buxus microphylla
Abundance
1
766262
140°E
145°E
135°E
130°E
125°E
45°N
40°N
35°N
30°N
25°N

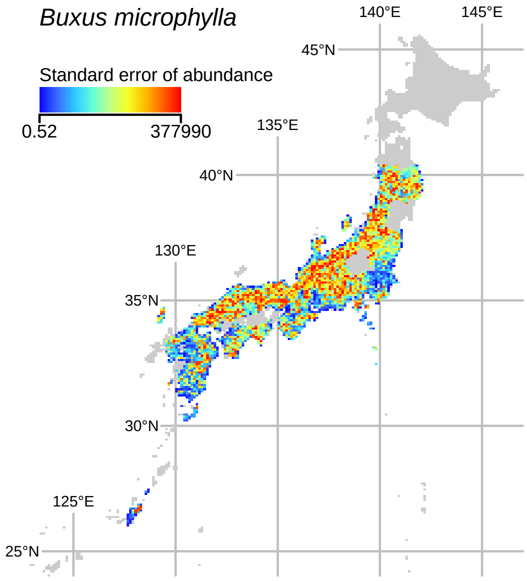
Buxus microphylla
Standard error of abundance
0.52
377990
140°E
145°E
135°E
130°E
125°E
45°N
40°N
35°N
30°N
25°N

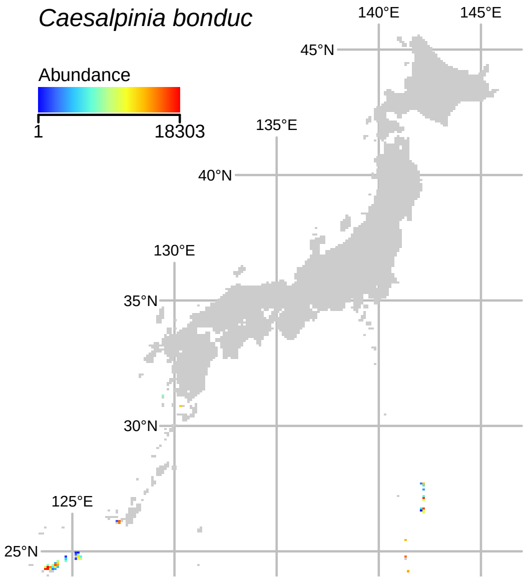
Caesalpinia bonduc
Abundance
1
18303
140°E
145°E
135°E
130°E
125°E
45°N
40°N
35°N
30°N
25°N

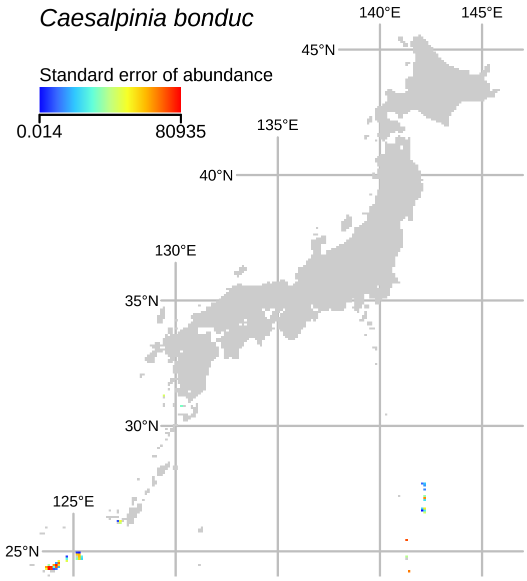
Caesalpinia bonduc
Standard error of abundance
0.014
80935
140°E
145°E
135°E
130°E
125°E
45°N
40°N
35°N
30°N
25°N

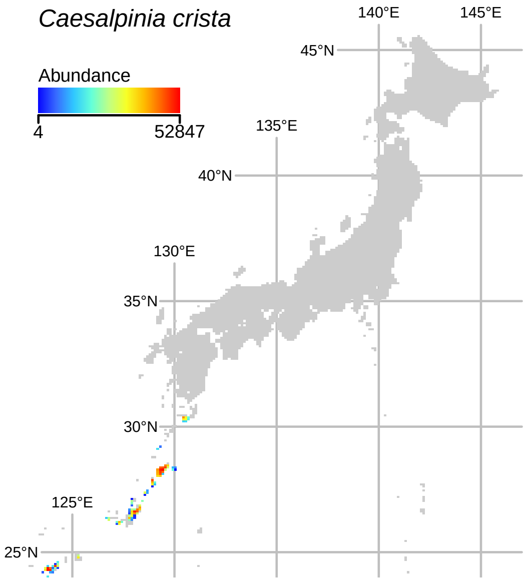
Caesalpinia crista
Abundance
4
52847
140°E
145°E
45°N
135°E
40°N
130°E
35°N
30°N
125°E
25°N

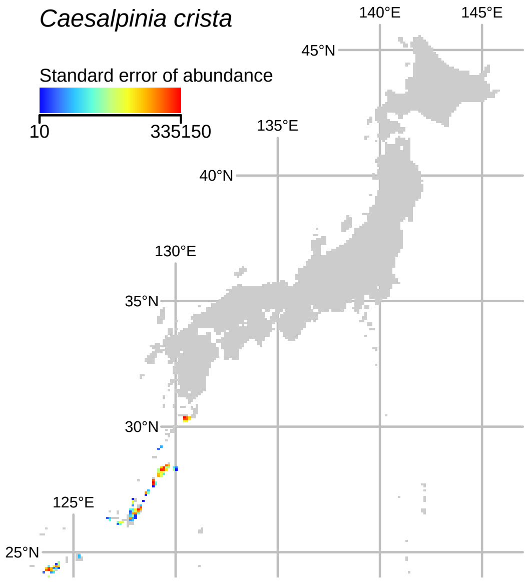
Caesalpinia crista
Standard error of abundance
10
335150
140°E
145°E
45°N
135°E
40°N
130°E
35°N
30°N
125°E
25°N

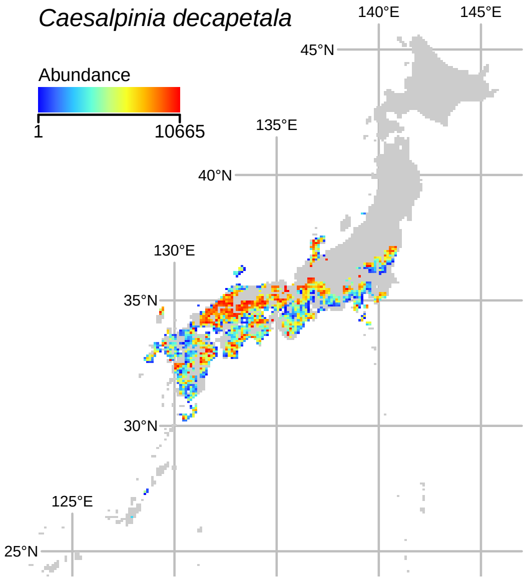
Caesalpinia decapetala
Abundance
1
10665
140°E
145°E
45°N
135°E
40°N
130°E
35°N
30°N
125°E
25°N

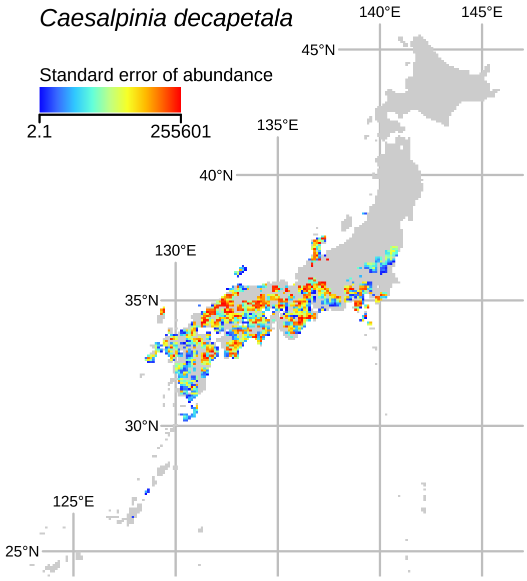
Caesalpinia decapetala
Standard error of abundance
2.1
255601
140°E
145°E
45°N
135°E
40°N
130°E
35°N
30°N
125°E
25°N

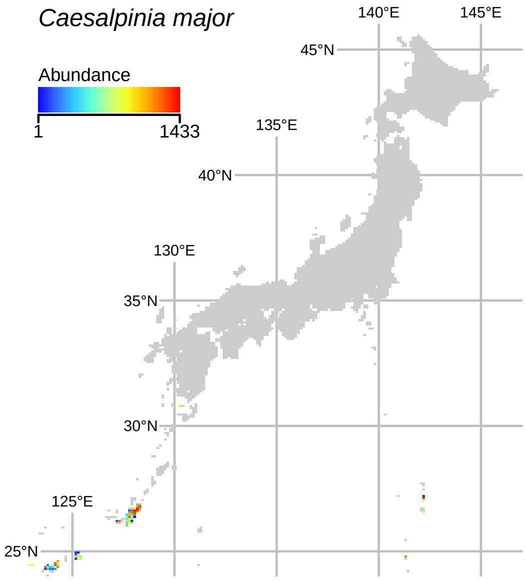
Caesalpinia major
Abundance
1
1433
140°E
145°E
45°N
135°E
40°N
130°E
35°N
30°N
125°E
25°N

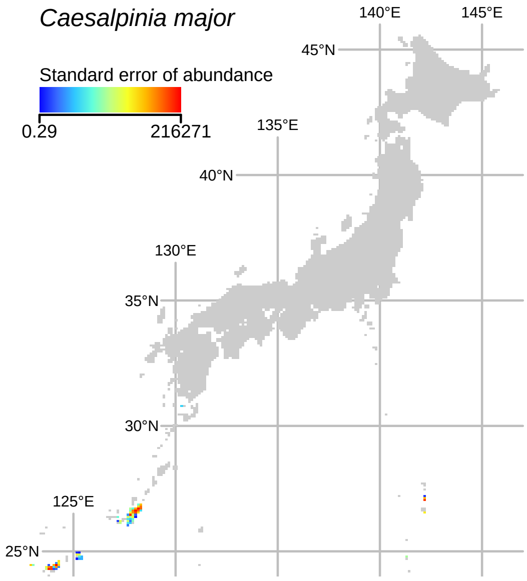
Caesalpinia major
Standard error of abundance
0.29
216271
140°E
145°E
45°N
135°E
40°N
130°E
35°N
30°N
125°E
25°N

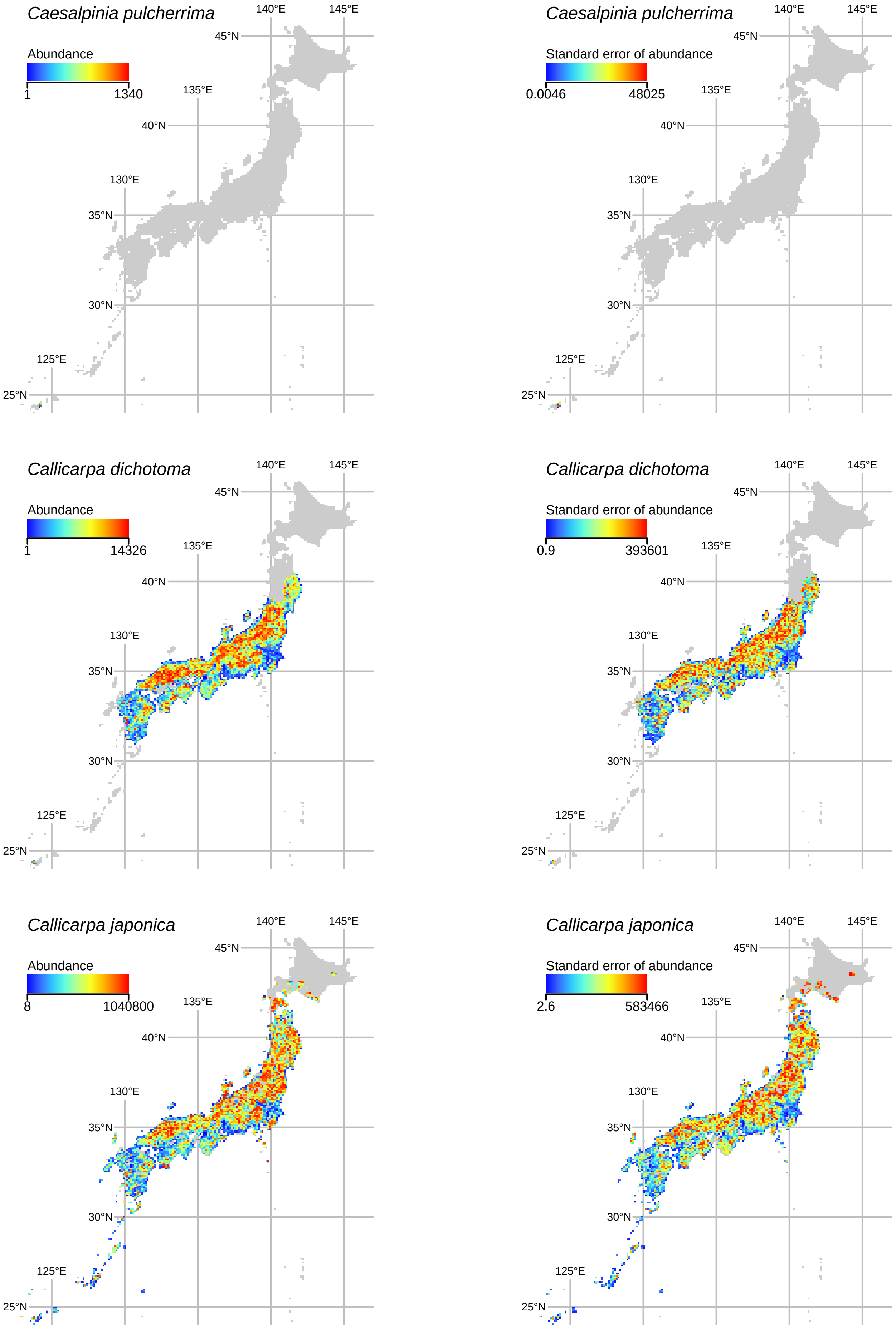

Caesalpinia pulcherrima
Abundance
1
1340
Caesalpinia pulcherrima
Standard error of abundance
0.0046
48025
Callicarpa dichotoma
Abundance
1
14326
Callicarpa dichotoma
Standard error of abundance
0.9
393601
Callicarpa japonica
Abundance
8
1040800
Callicarpa japonica
Standard error of abundance
2.6
583466
140°E
145°E
135°E
130°E
125°E
45°N
40°N
35°N
30°N
25°N

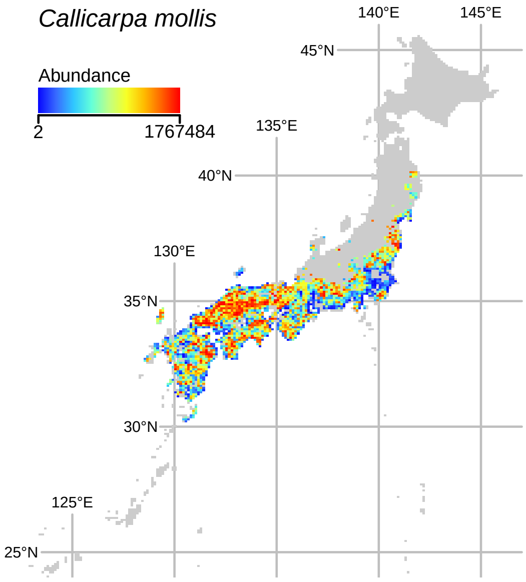
Callicarpa mollis
Abundance
2
1767484
140°E
145°E
45°N
135°E
40°N
130°E
35°N
30°N
125°E
25°N

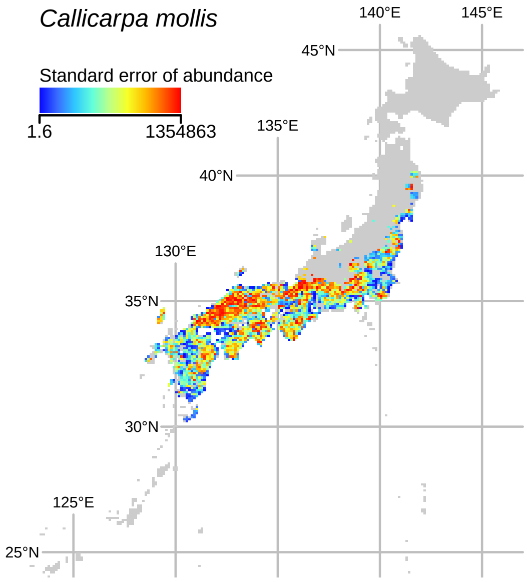
Callicarpa mollis
Standard error of abundance
1.6
1354863
140°E
145°E
45°N
135°E
40°N
130°E
35°N
30°N
125°E
25°N

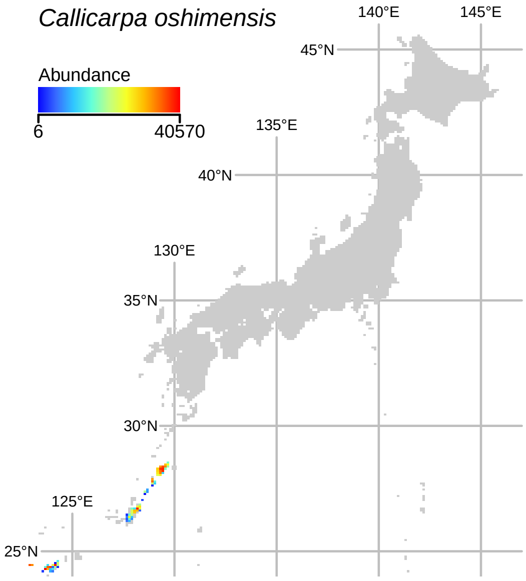
Callicarpa oshimensis
Abundance
6
40570
140°E
145°E
45°N
135°E
40°N
130°E
35°N
30°N
125°E
25°N

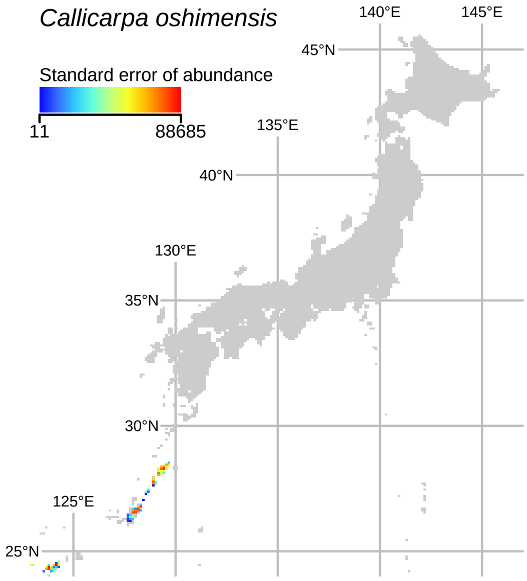
Callicarpa oshimensis
Standard error of abundance
11
88685
140°E
145°E
45°N
135°E
40°N
130°E
35°N
30°N
125°E
25°N

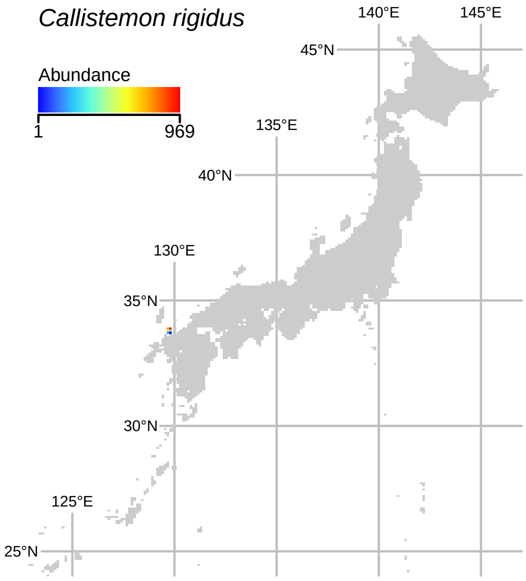
Callistemon rigidus
Abundance
1
969
140°E
145°E
45°N
135°E
40°N
130°E
35°N
30°N
125°E
25°N

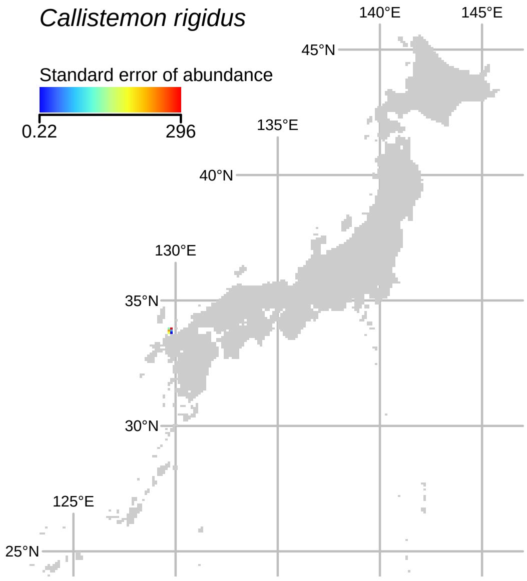
Callistemon rigidus
Standard error of abundance
0.22
296
140°E
145°E
45°N
135°E
40°N
130°E
35°N
30°N
125°E
25°N

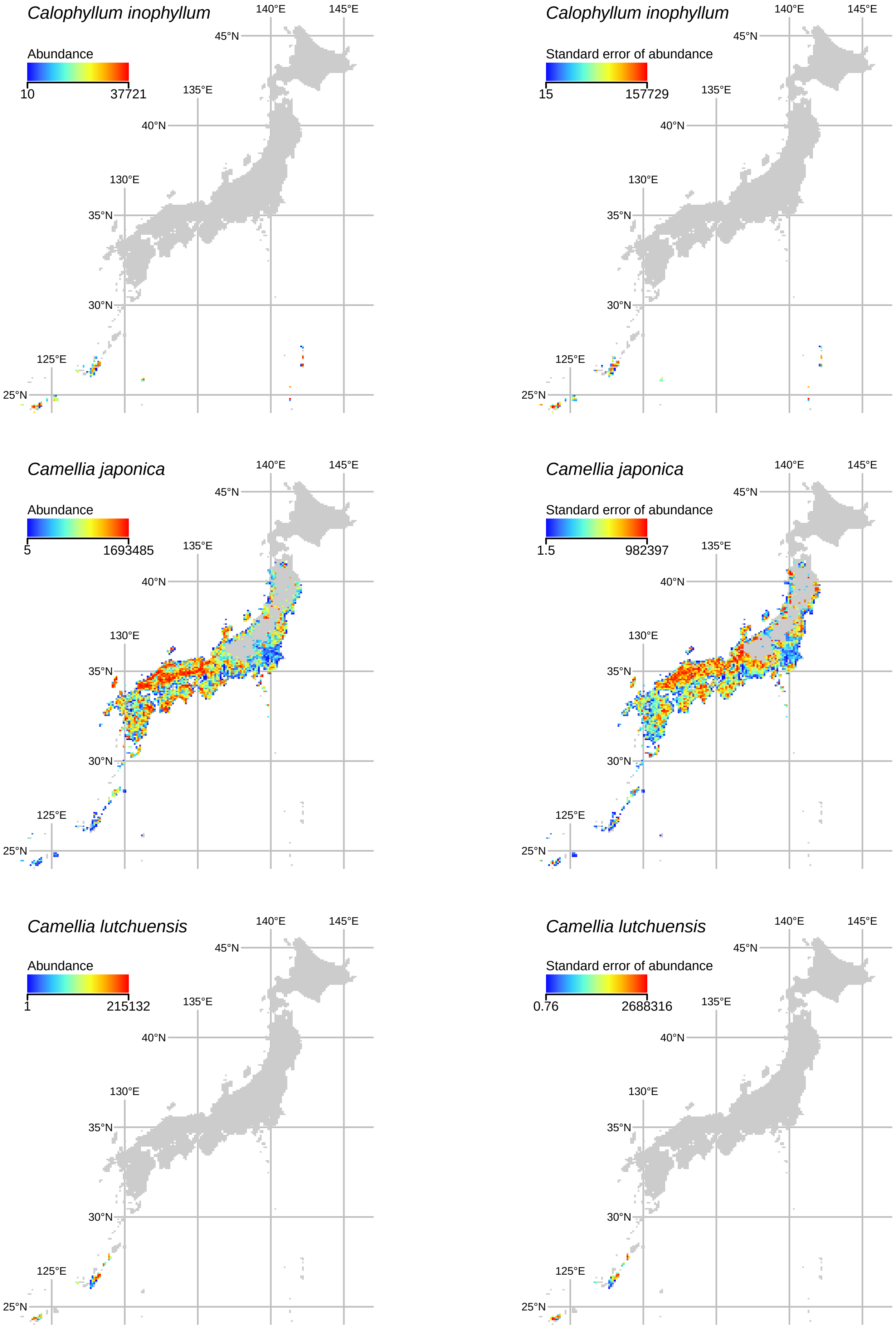
Calophyllum inophyllum
Abundance
10
37721
Calophyllum inophyllum
Standard error of abundance
15
157729
Camellia japonica
Abundance
5
1693485
Camellia japonica
Standard error of abundance
1.5
982397
Camellia lutchuensis
Abundance
1
215132
Camellia lutchuensis
Standard error of abundance
0.76
2688316
125°E
130°E
135°E
140°E
145°E
25°N
30°N
35°N
40°N
45°N

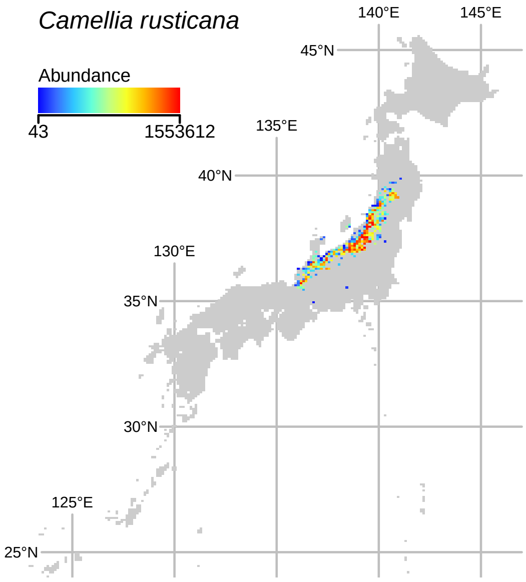
Camellia rusticana
Abundance
43
1553612
140°E
145°E
135°E
130°E
125°E
45°N
40°N
35°N
30°N
25°N

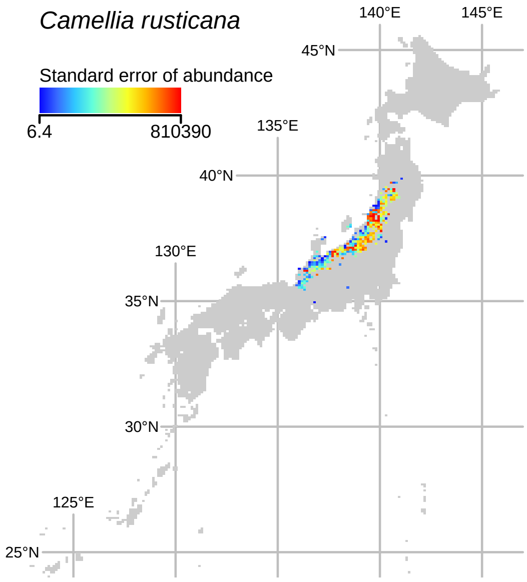
Camellia rusticana
Standard error of abundance
6.4
810390
140°E
145°E
135°E
130°E
125°E
45°N
40°N
35°N
30°N
25°N

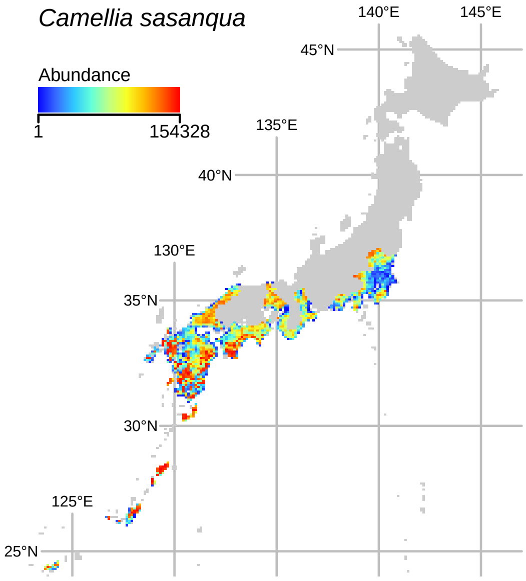
Camellia sasanqua
Abundance
1
154328
140°E
145°E
135°E
130°E
125°E
45°N
40°N
35°N
30°N
25°N

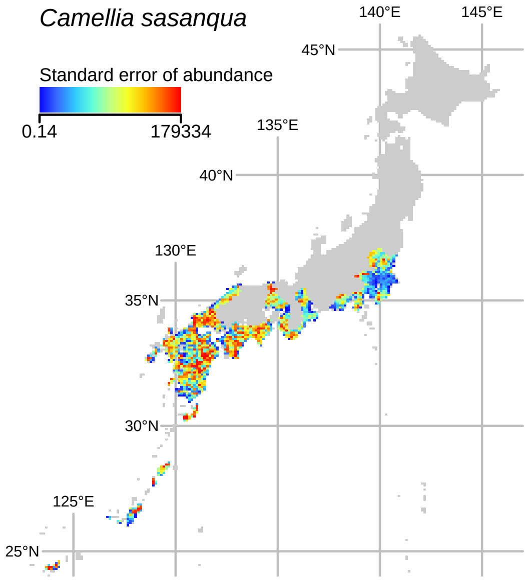
Camellia sasanqua
Standard error of abundance
0.14
179334
140°E
145°E
135°E
130°E
125°E
45°N
40°N
35°N
30°N
25°N

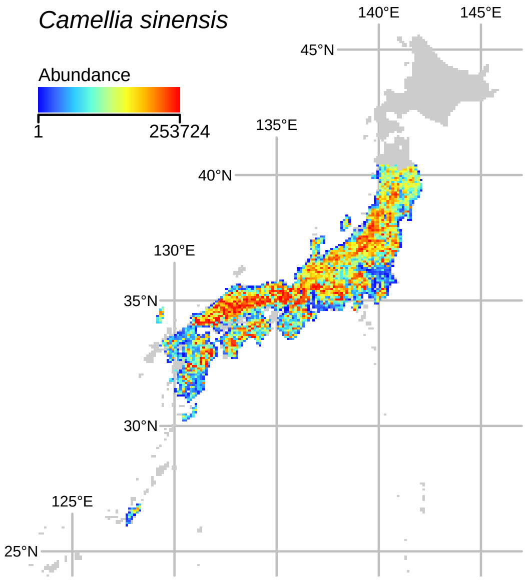
Camellia sinensis
Abundance
1
253724
140°E
145°E
135°E
130°E
125°E
45°N
40°N
35°N
30°N
25°N

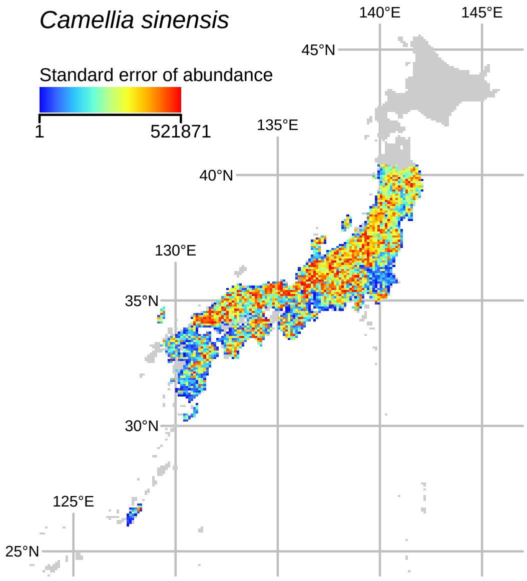
Camellia sinensis
Standard error of abundance
1
521871
140°E
145°E
135°E
130°E
125°E
45°N
40°N
35°N
30°N
25°N

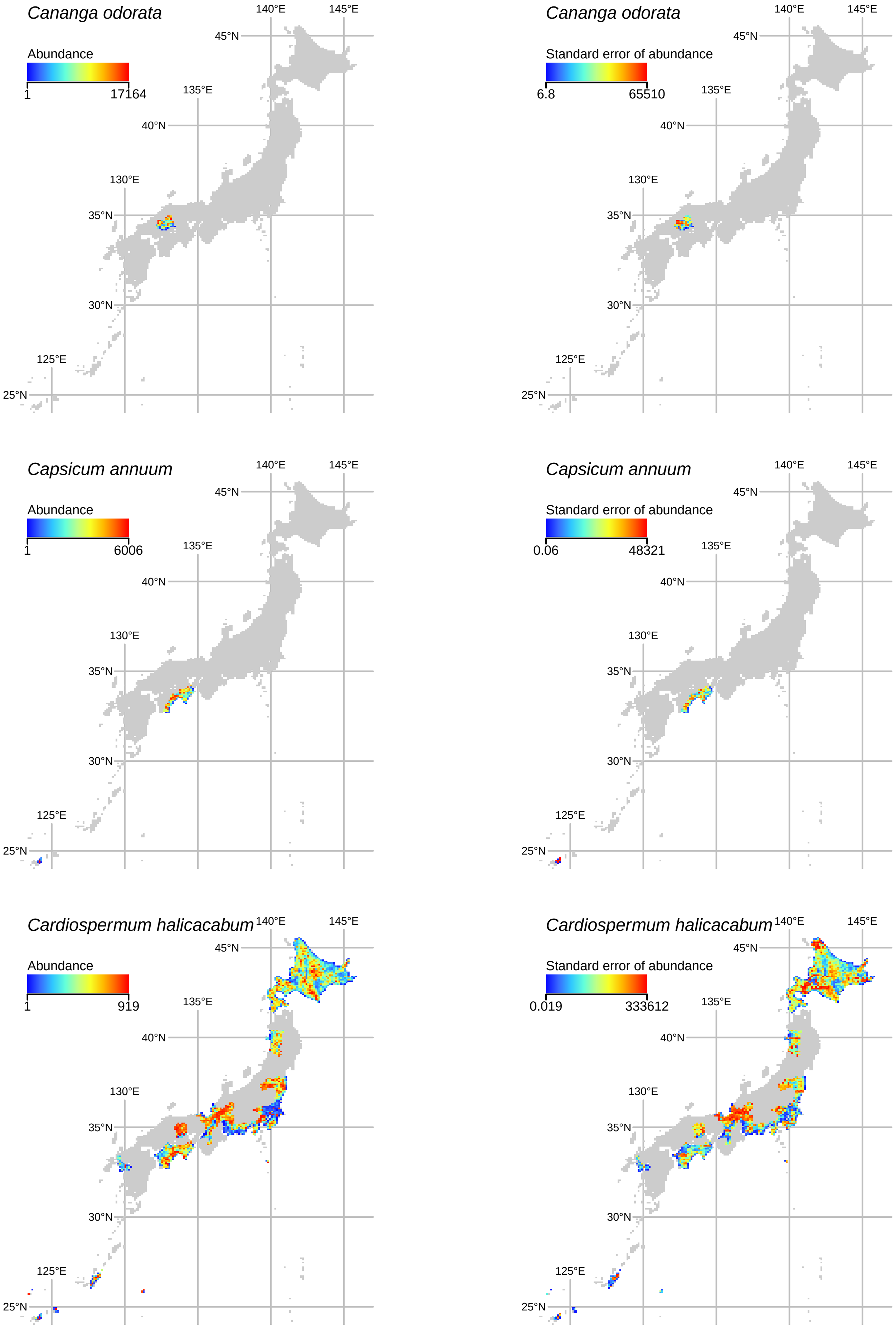
Cananga odorata
Abundance
1
17164
140°E
145°E
45°N
135°E
40°N
130°E
35°N
30°N
125°E
25°N
Cananga odorata
Standard error of abundance
6.8
65510
140°E
145°E
45°N
135°E
40°N
130°E
35°N
30°N
125°E
25°N
Capsicum annuum
Abundance
1
6006
140°E
145°E
45°N
135°E
40°N
130°E
35°N
30°N
125°E
25°N
Capsicum annuum
Standard error of abundance
0.06
48321
140°E
145°E
45°N
135°E
40°N
130°E
35°N
30°N
125°E
25°N
Cardiospermum halicacabum
Abundance
1
919
140°E
145°E
45°N
135°E
40°N
130°E
35°N
30°N
125°E
25°N
Cardiospermum halicacabum
Standard error of abundance
0.019
333612
140°E
145°E
45°N
135°E
40°N
130°E
35°N
30°N
125°E
25°N

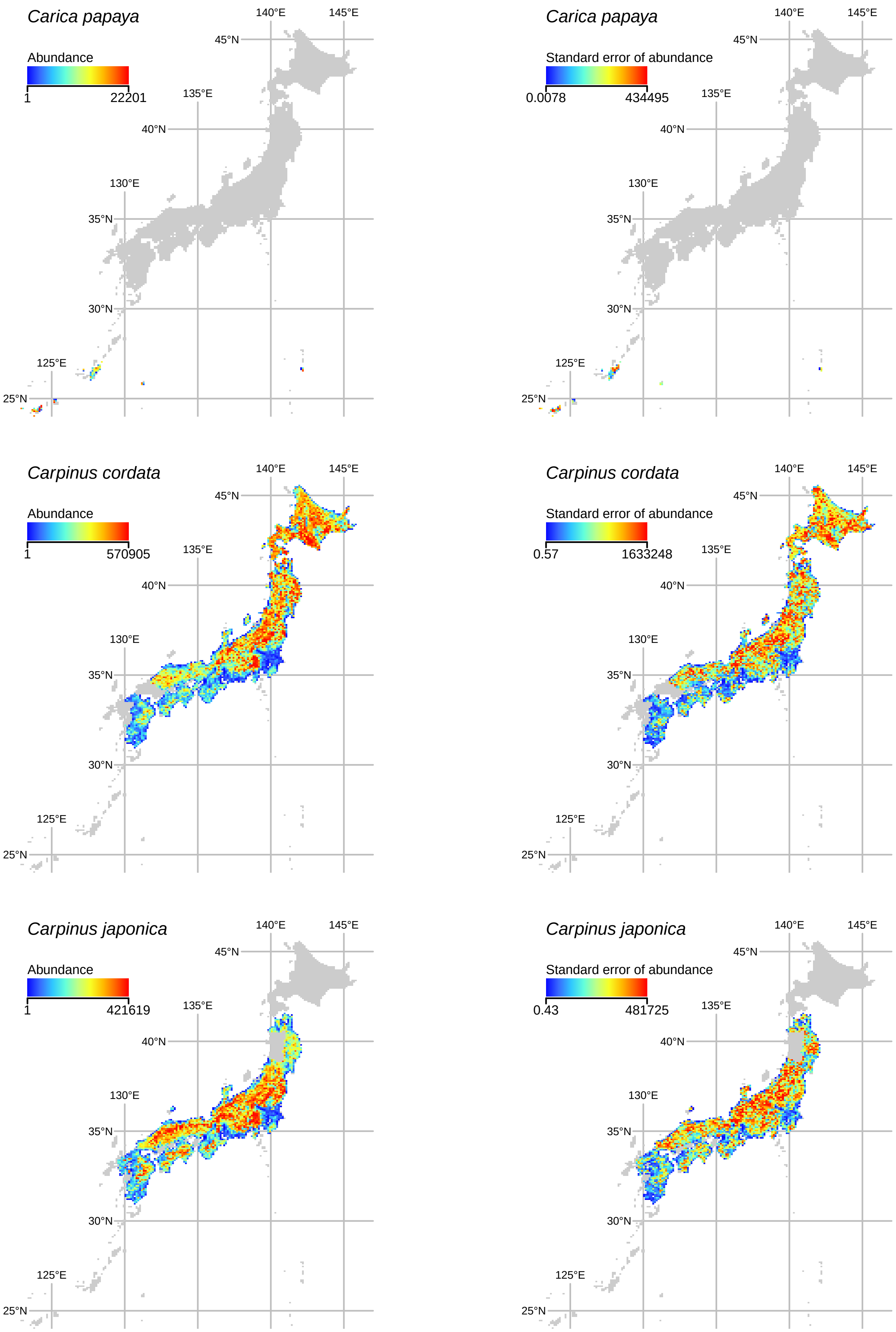
Carica papaya
Abundance
1
22201
Carica papaya
Standard error of abundance
0.0078
434495
Carpinus cordata
Abundance
1
570905
Carpinus cordata
Standard error of abundance
0.57
1633248
Carpinus japonica
Abundance
1
421619
Carpinus japonica
Standard error of abundance
0.43
481725
125°E
130°E
135°E
140°E
145°E
25°N
30°N
35°N
40°N
45°N

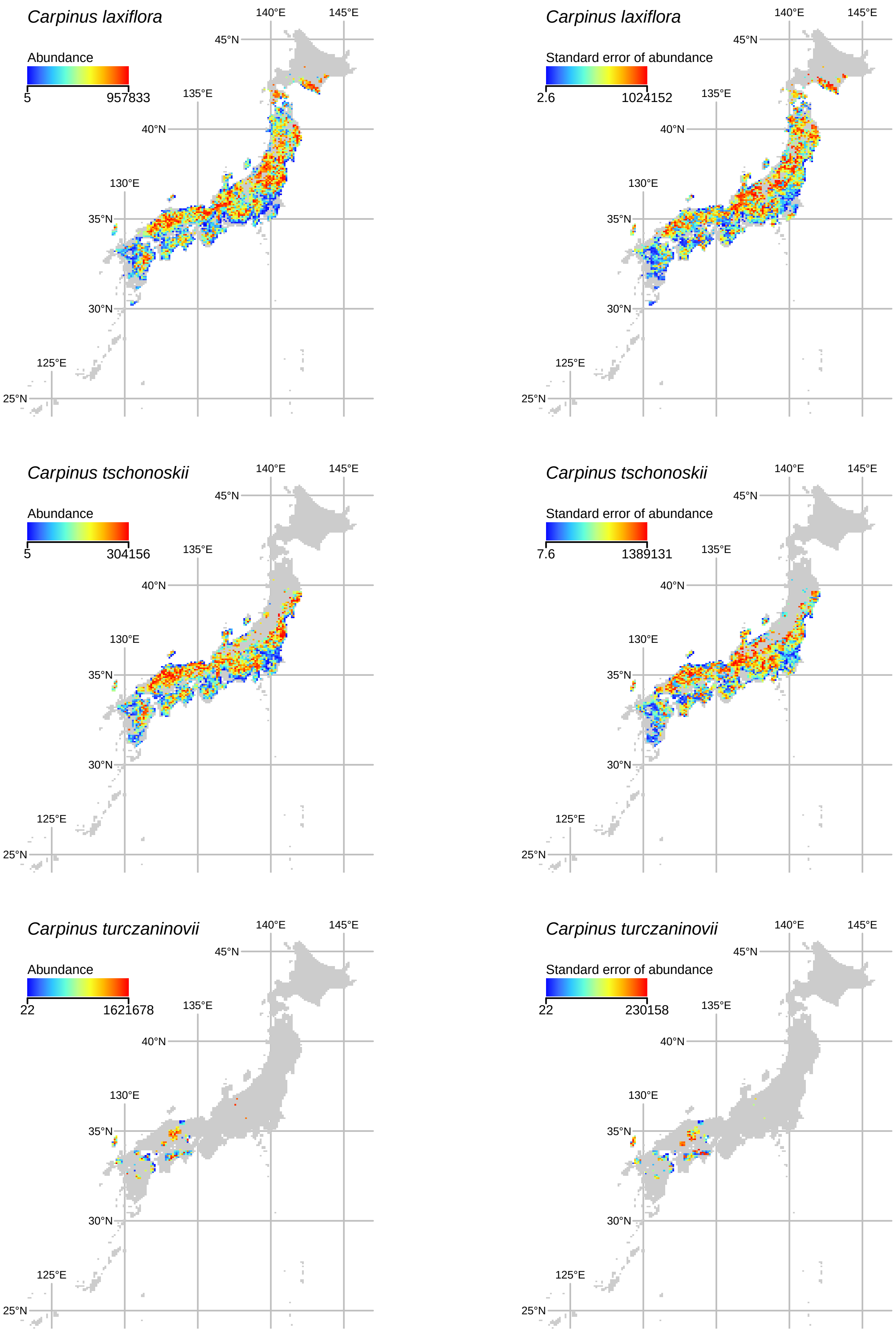
Carpinus laxiflora
Abundance
5
957833
Carpinus laxiflora
Standard error of abundance
2.6
1024152
Carpinus tschonoskii
Abundance
5
304156
Carpinus tschonoskii
Standard error of abundance
7.6
1389131
Carpinus turczaninovii
Abundance
22
1621678
Carpinus turczaninovii
Standard error of abundance
22
230158
125°E
130°E
135°E
140°E
145°E
25°N
30°N
35°N
40°N
45°N

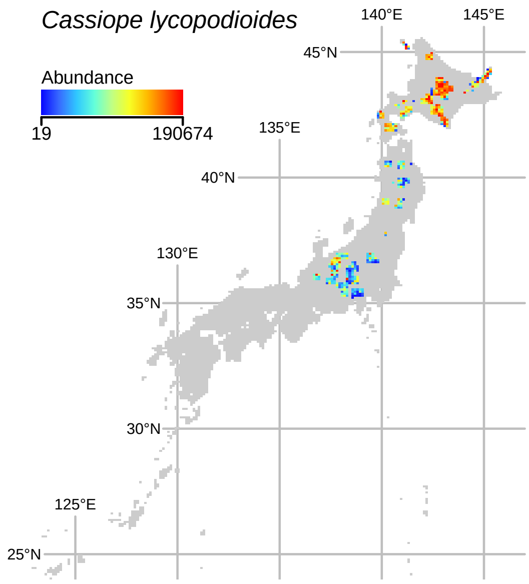
Cassiope lycopodioides
Abundance
19
190674
140°E
145°E
45°N
135°E
40°N
130°E
35°N
30°N
125°E
25°N

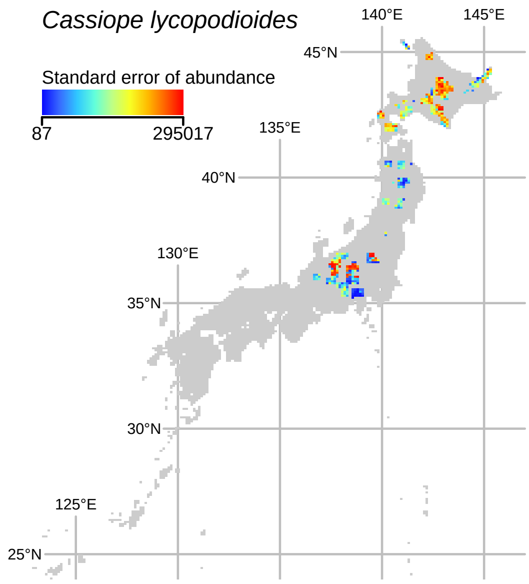
Cassiope lycopodioides
Standard error of abundance
87
295017
140°E
145°E
45°N
135°E
40°N
130°E
35°N
30°N
125°E
25°N

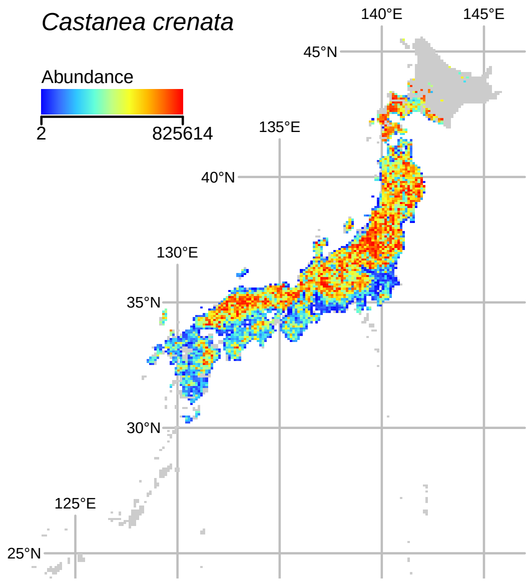
Castanea crenata
Abundance
2
825614
140°E
145°E
45°N
135°E
40°N
130°E
35°N
30°N
125°E
25°N

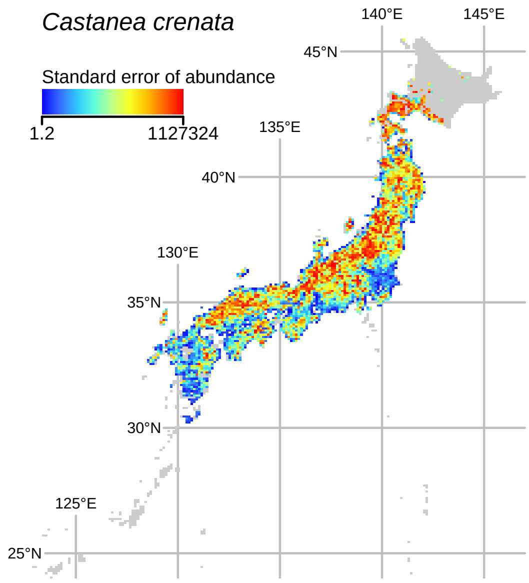
Castanea crenata
Standard error of abundance
1.2
1127324
140°E
145°E
45°N
135°E
40°N
130°E
35°N
30°N
125°E
25°N

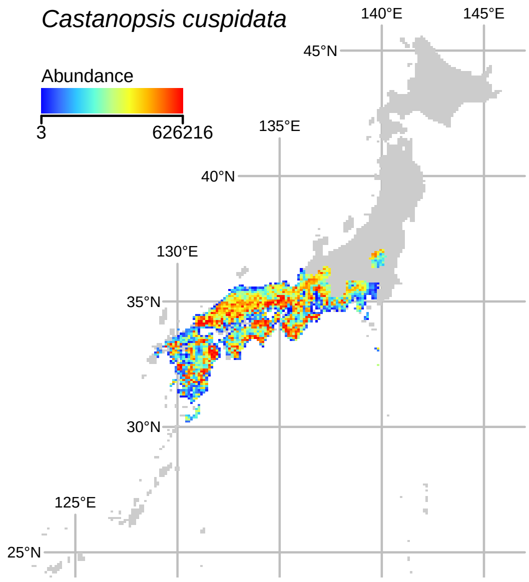
Castanopsis cuspidata
Abundance
3
626216
140°E
145°E
45°N
135°E
40°N
130°E
35°N
30°N
125°E
25°N

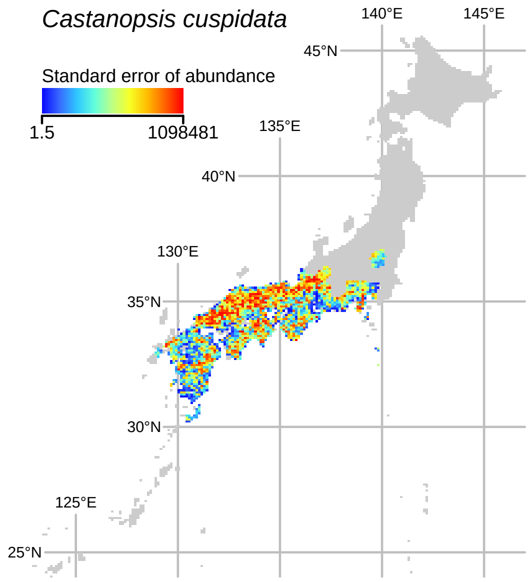
Castanopsis cuspidata
Standard error of abundance
1.5
1098481
140°E
145°E
45°N
135°E
40°N
130°E
35°N
30°N
125°E
25°N

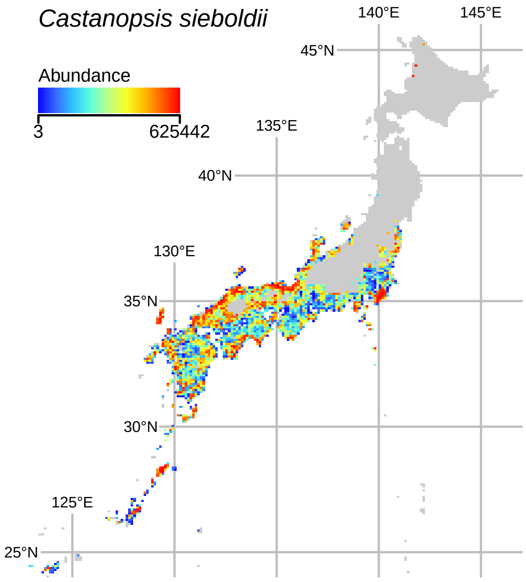
Castanopsis sieboldii
Abundance
3
625442
140°E
145°E
45°N
135°E
40°N
130°E
35°N
30°N
125°E
25°N

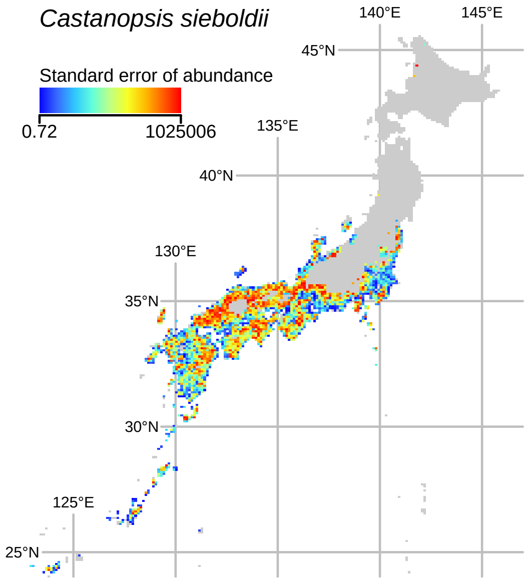
Castanopsis sieboldii
Standard error of abundance
0.72
1025006
140°E
145°E
45°N
135°E
40°N
130°E
35°N
30°N
125°E
25°N

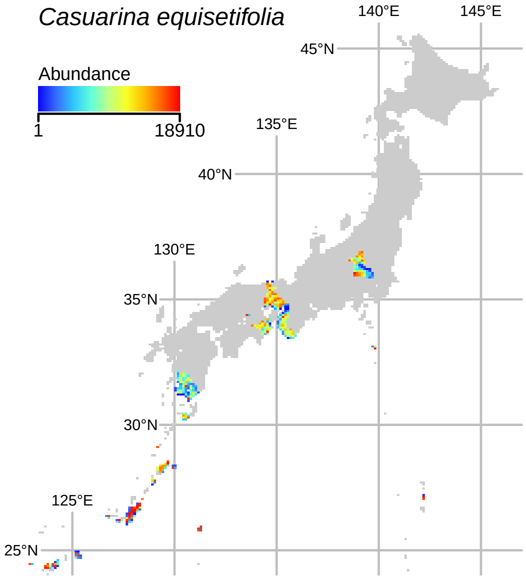
Casuarina equisetifolia
Abundance
1
18910
140°E
145°E
45°N
135°E
40°N
130°E
35°N
30°N
125°E
25°N

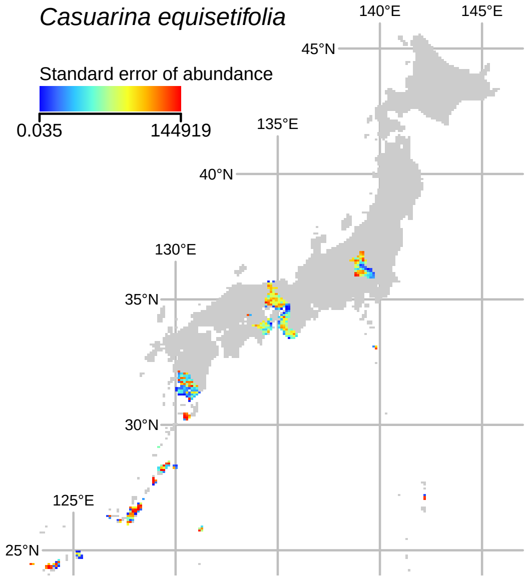
Casuarina equisetifolia
Standard error of abundance
0.035
144919
140°E
145°E
45°N
135°E
40°N
130°E
35°N
30°N
125°E
25°N

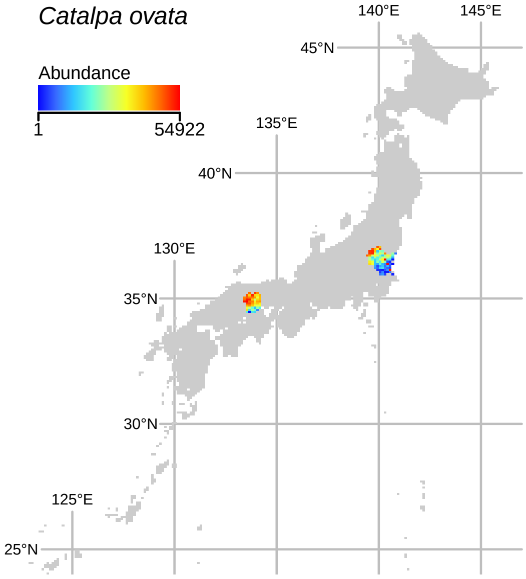
Catalpa ovata
Abundance
1
54922
140°E
145°E
45°N
135°E
40°N
130°E
35°N
30°N
125°E
25°N

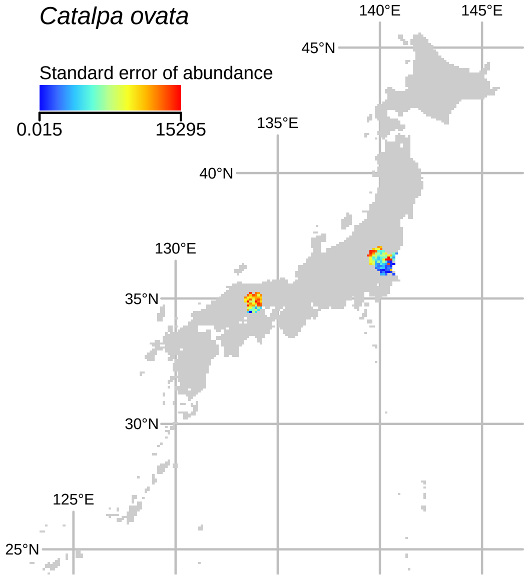
Catalpa ovata
Standard error of abundance
0.015
15295
140°E
145°E
45°N
135°E
40°N
130°E
35°N
30°N
125°E
25°N

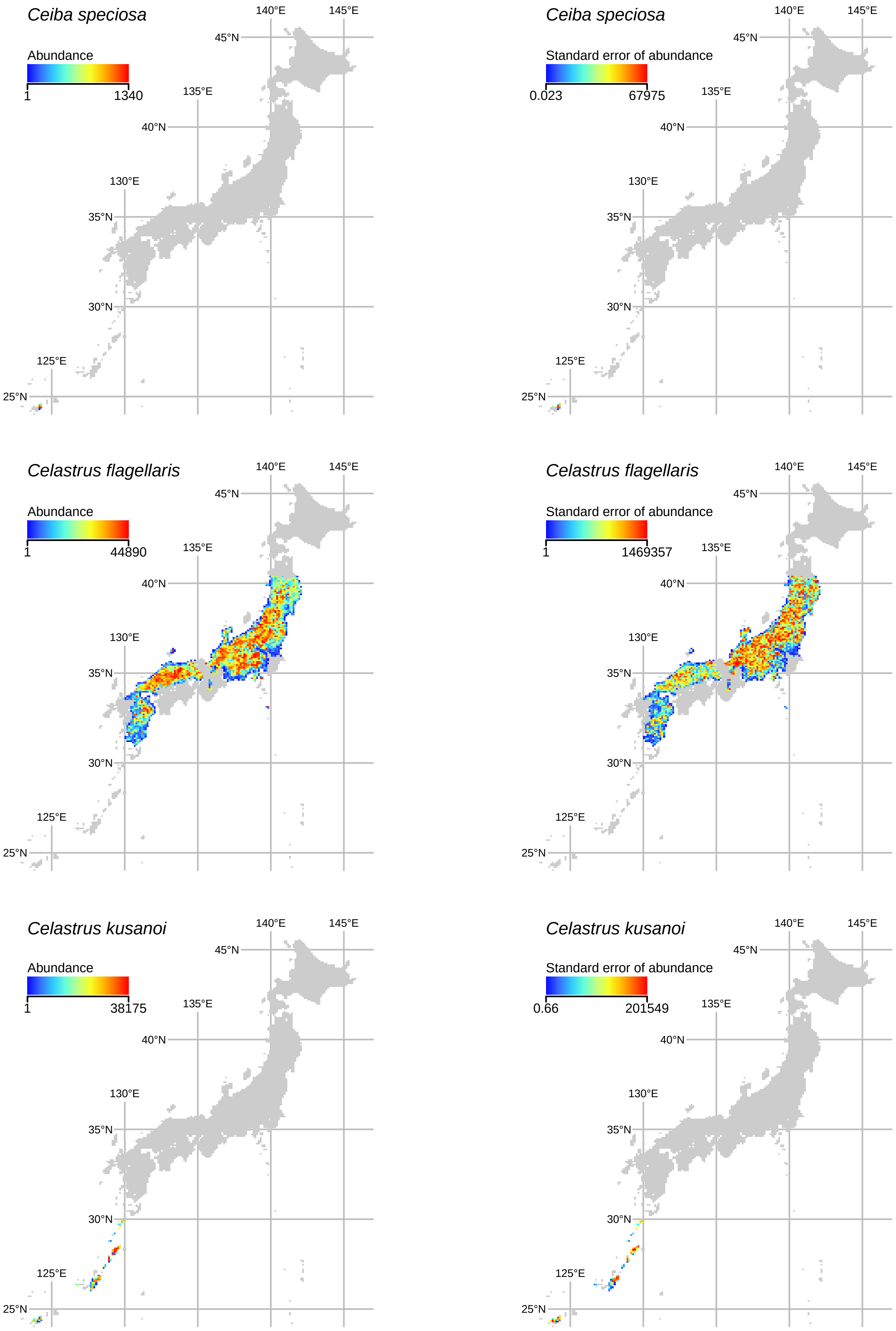
Ceiba speciosa
Abundance
1
1340
Ceiba speciosa
Standard error of abundance
0.023
67975
Celastrus flagellaris
Abundance
1
44890
Celastrus flagellaris
Standard error of abundance
1
1469357
Celastrus kusanoi
Abundance
1
38175
Celastrus kusanoi
Standard error of abundance
0.66
201549
140°E
145°E
45°N
135°E
40°N
130°E
35°N
30°N
125°E
25°N

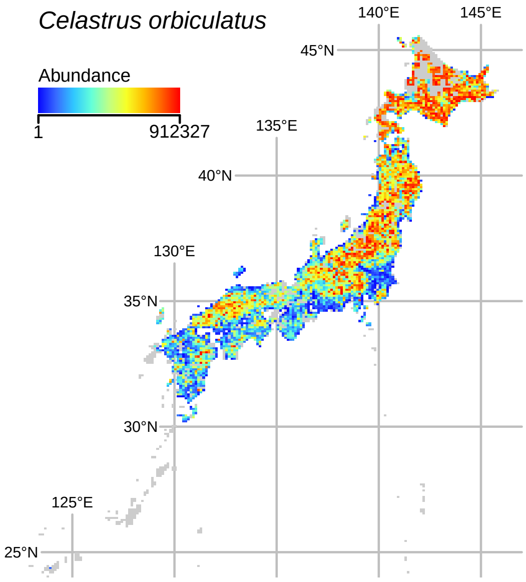
Celastrus orbiculatus
Abundance
1
912327
140°E
145°E
45°N
135°E
40°N
130°E
35°N
30°N
125°E
25°N

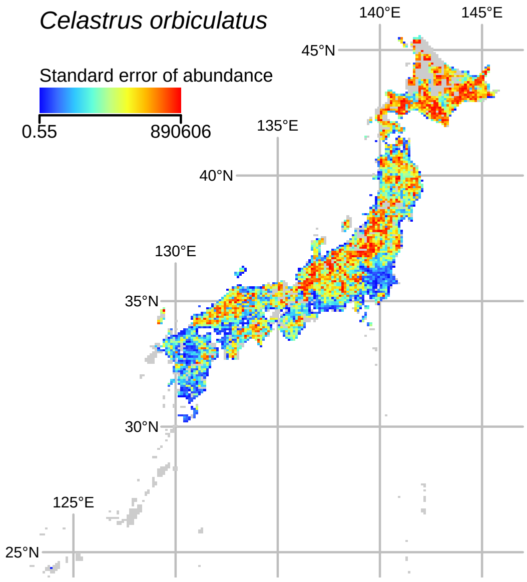
Celastrus orbiculatus
Standard error of abundance
0.55
890606
140°E
145°E
45°N
135°E
40°N
130°E
35°N
30°N
125°E
25°N

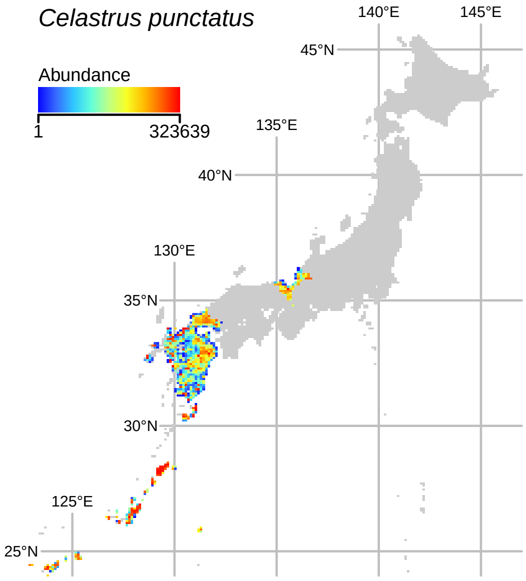
Celastrus punctatus
Abundance
1
323639
140°E
145°E
45°N
135°E
40°N
130°E
35°N
30°N
125°E
25°N

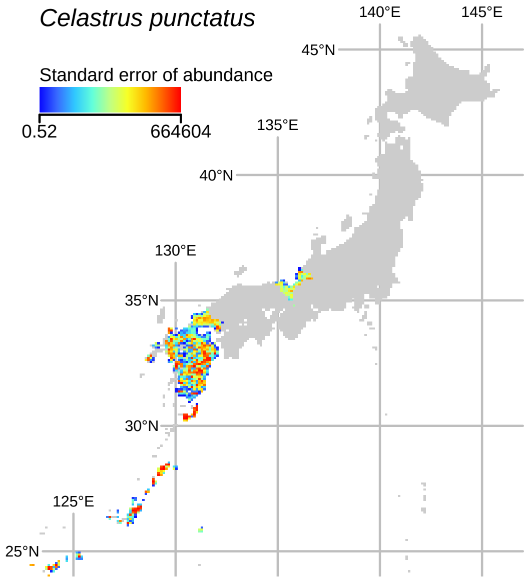
Celastrus punctatus
Standard error of abundance
0.52
664604
140°E
145°E
45°N
135°E
40°N
130°E
35°N
30°N
125°E
25°N

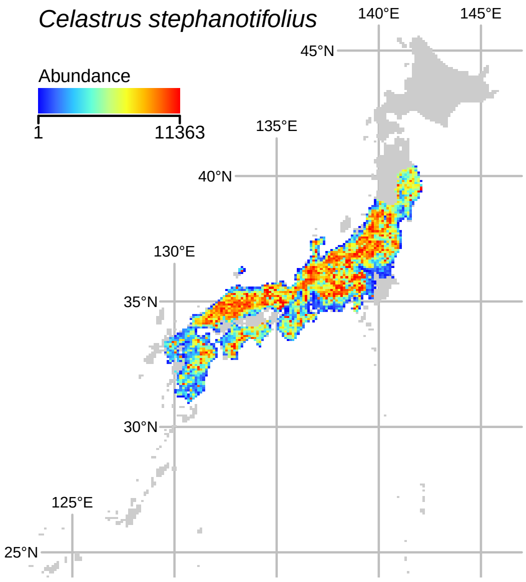
Celastrus stephanotifolius
Abundance
1
11363
140°E
145°E
45°N
135°E
40°N
130°E
35°N
30°N
125°E
25°N

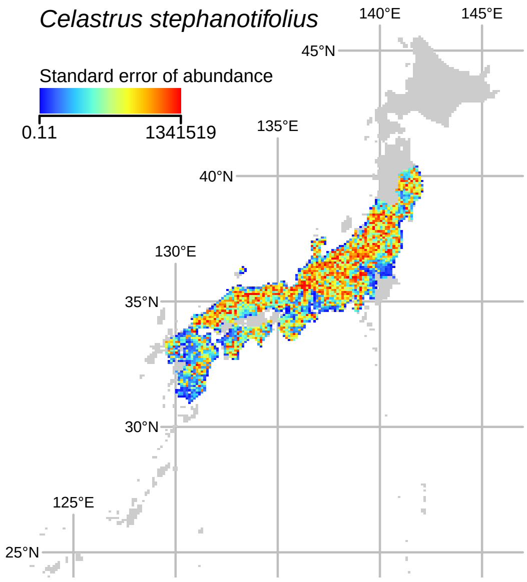
Celastrus stephanotifolius
Standard error of abundance
0.11
1341519
140°E
145°E
45°N
135°E
40°N
130°E
35°N
30°N
125°E
25°N

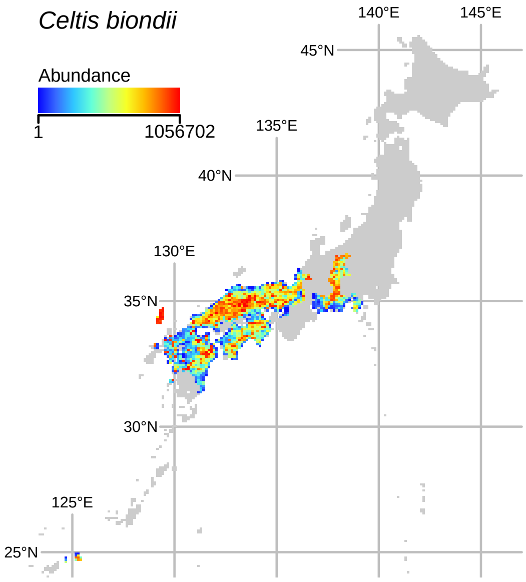
Celtis biondii
Abundance
1
1056702
140°E
145°E
45°N
135°E
40°N
130°E
35°N
30°N
125°E
25°N

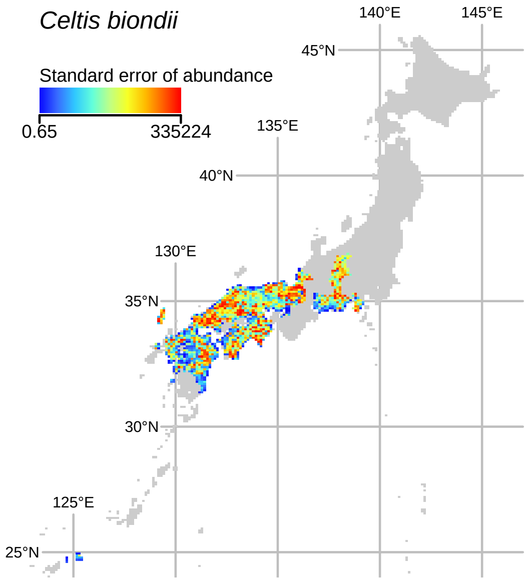
Celtis biondii
Standard error of abundance
0.65
335224
140°E
145°E
45°N
135°E
40°N
130°E
35°N
30°N
125°E
25°N

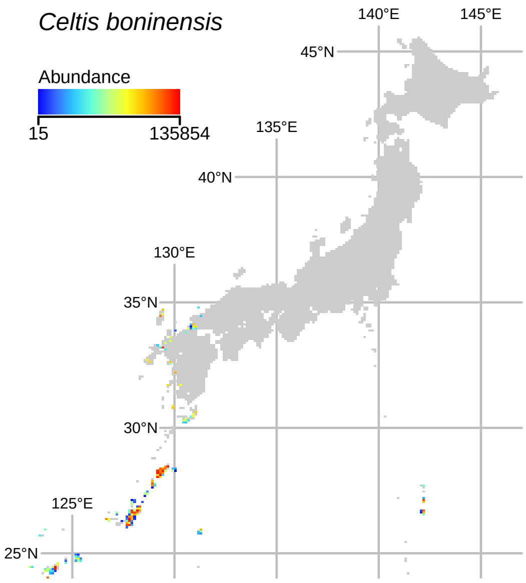
Celtis boninensis
Abundance
15
135854
140°E
145°E
45°N
135°E
40°N
130°E
35°N
30°N
125°E
25°N

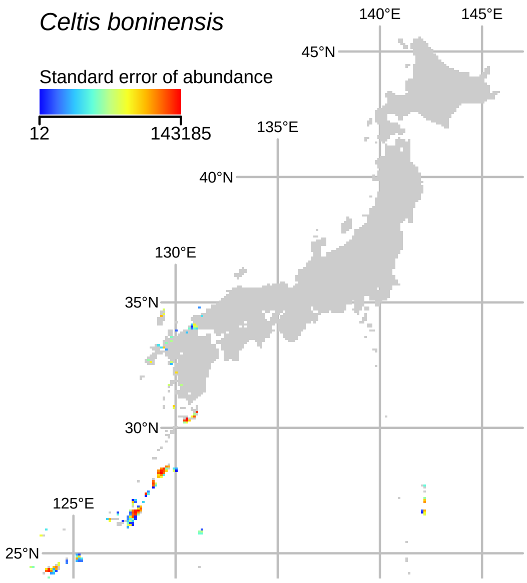
Celtis boninensis
Standard error of abundance
12
143185
140°E
145°E
45°N
135°E
40°N
130°E
35°N
30°N
125°E
25°N

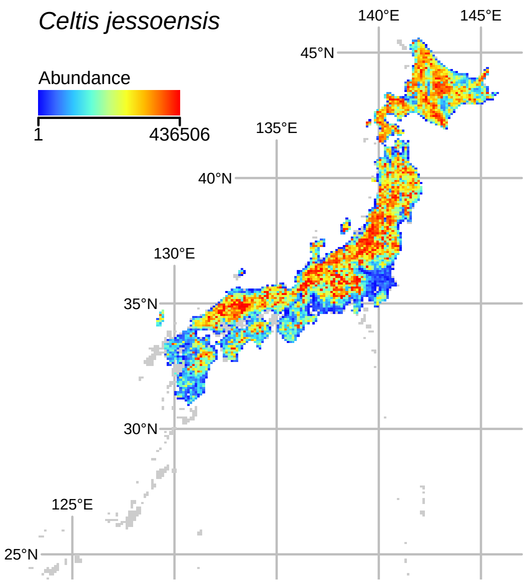
Celtis jessoensis
Abundance
1
436506
140°E
145°E
45°N
135°E
40°N
130°E
35°N
30°N
125°E
25°N

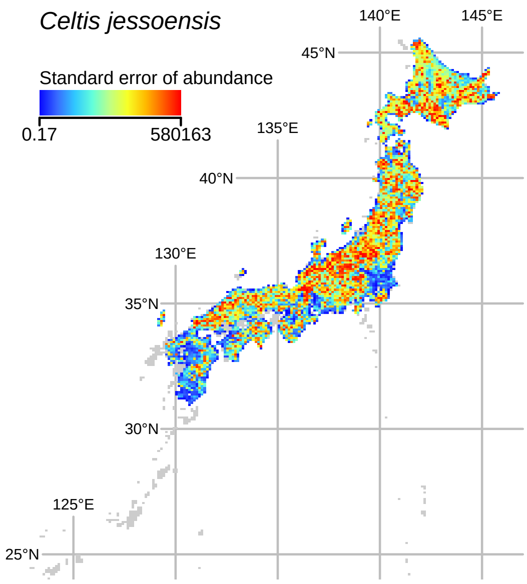
Celtis jessoensis
Standard error of abundance
0.17
580163
140°E
145°E
45°N
135°E
40°N
130°E
35°N
30°N
125°E
25°N

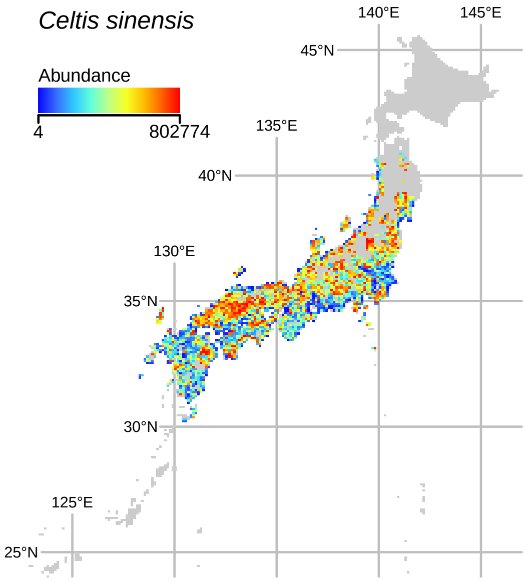
Celtis sinensis
Abundance
4
802774
140°E
145°E
45°N
135°E
40°N
130°E
35°N
30°N
125°E
25°N

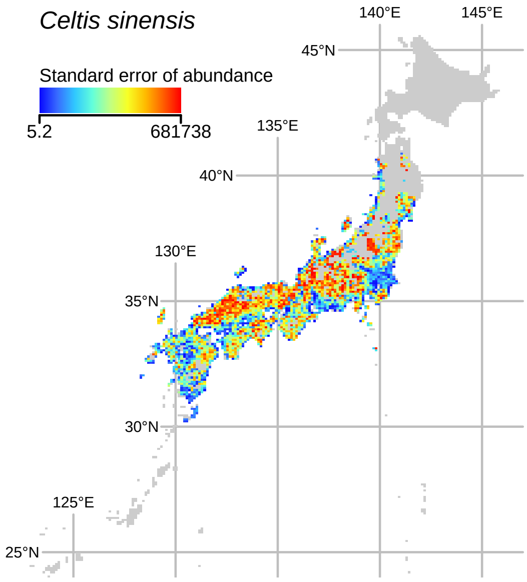
Celtis sinensis
Standard error of abundance
5.2
681738
140°E
145°E
45°N
135°E
40°N
130°E
35°N
30°N
125°E
25°N

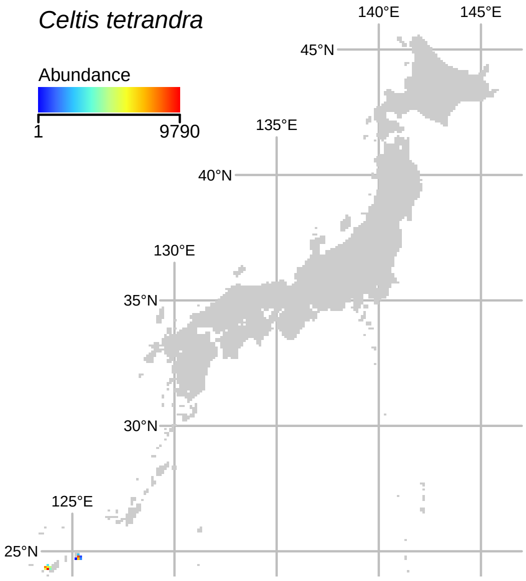
Celtis tetrandra
Abundance
1
9790
140°E
145°E
45°N
135°E
40°N
130°E
35°N
30°N
125°E
25°N

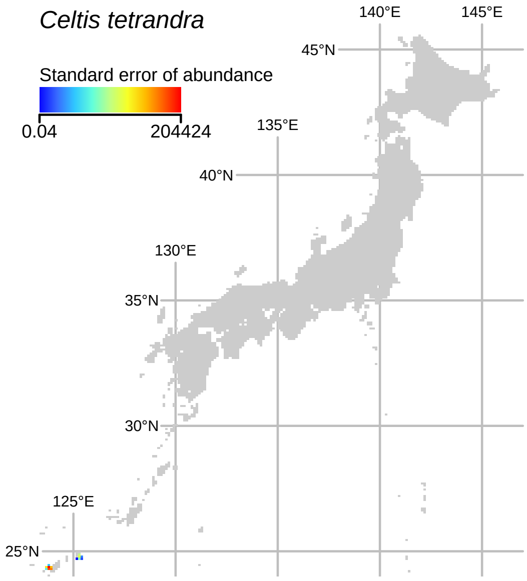
Celtis tetrandra
Standard error of abundance
0.04
204424
140°E
145°E
45°N
135°E
40°N
130°E
35°N
30°N
125°E
25°N

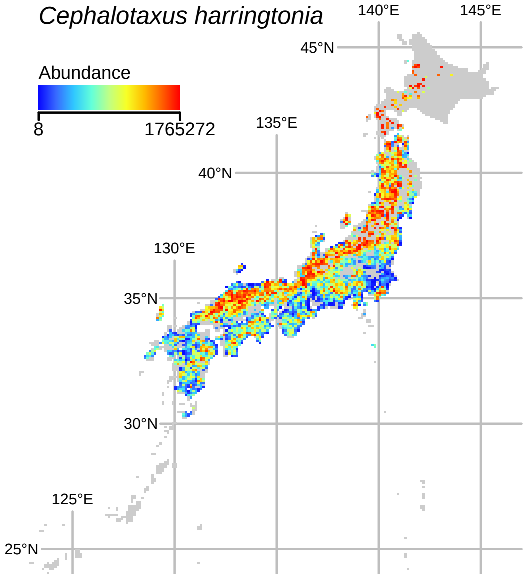
Cephalotaxus harringtonia
Abundance
8
1765272
140°E
145°E
45°N
135°E
40°N
130°E
35°N
30°N
125°E
25°N

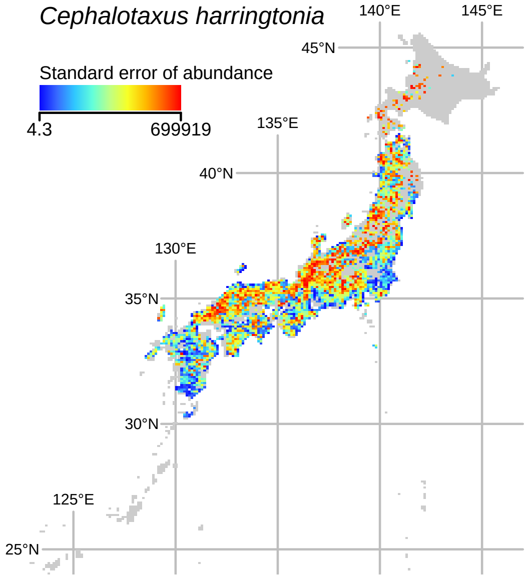
Cephalotaxus harringtonia
Standard error of abundance
4.3
699919
140°E
145°E
45°N
135°E
40°N
130°E
35°N
30°N
125°E
25°N

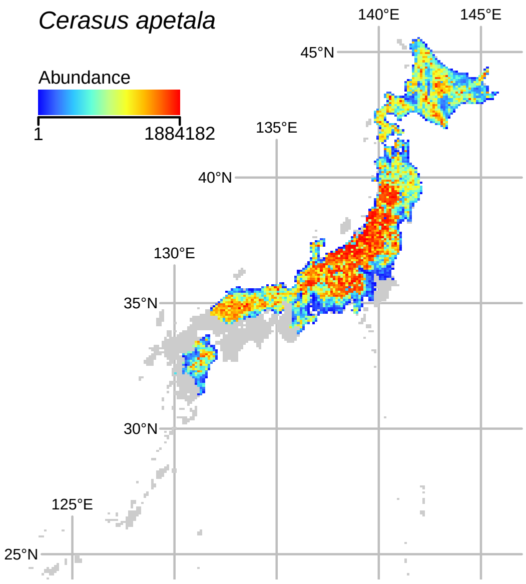
Cerasus apetala
Abundance
1
1884182
140°E
145°E
45°N
135°E
40°N
130°E
35°N
30°N
125°E
25°N

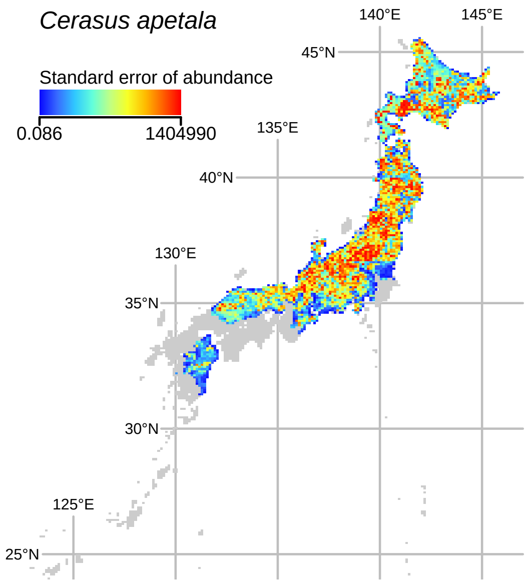
Cerasus apetala
Standard error of abundance
0.086
1404990
140°E
145°E
45°N
135°E
40°N
130°E
35°N
30°N
125°E
25°N

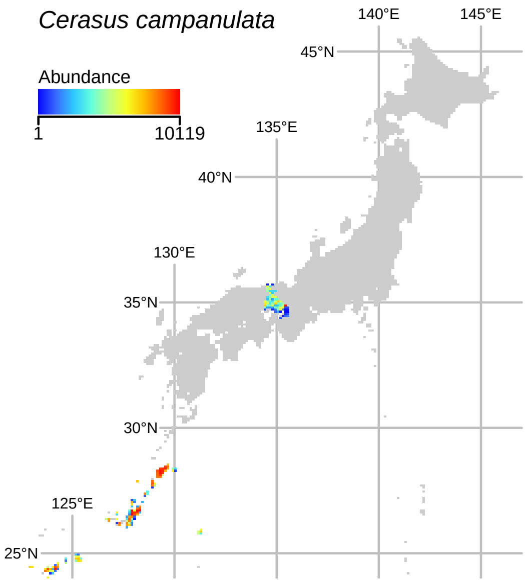
Cerasus campanulata
Abundance
1
10119
140°E
145°E
45°N
135°E
40°N
130°E
35°N
30°N
125°E
25°N

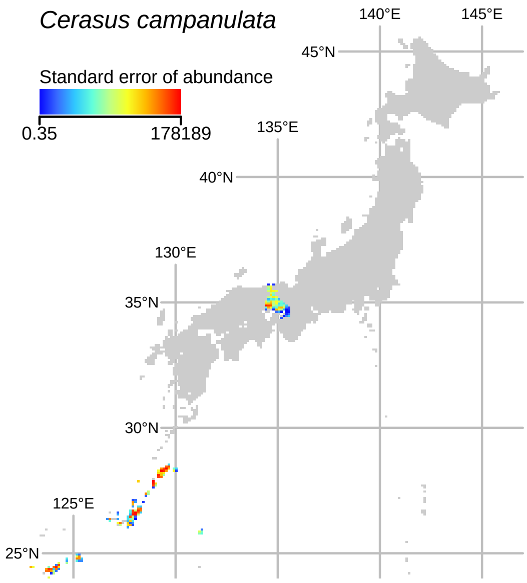
Cerasus campanulata
Standard error of abundance
0.35
178189
140°E
145°E
45°N
135°E
40°N
130°E
35°N
30°N
125°E
25°N

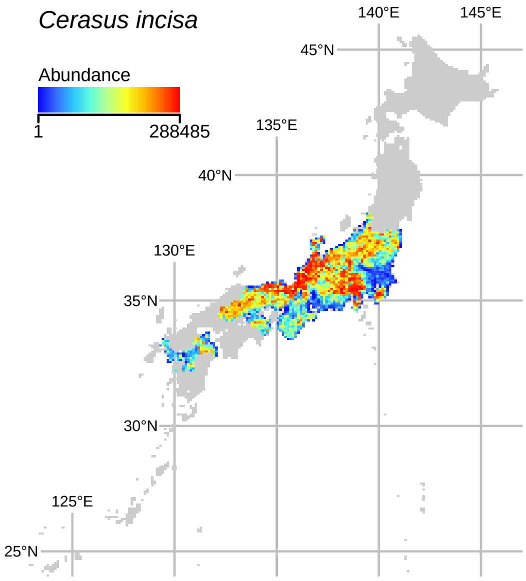
Cerasus incisa
Abundance
1
288485
140°E
145°E
45°N
135°E
40°N
130°E
35°N
30°N
125°E
25°N

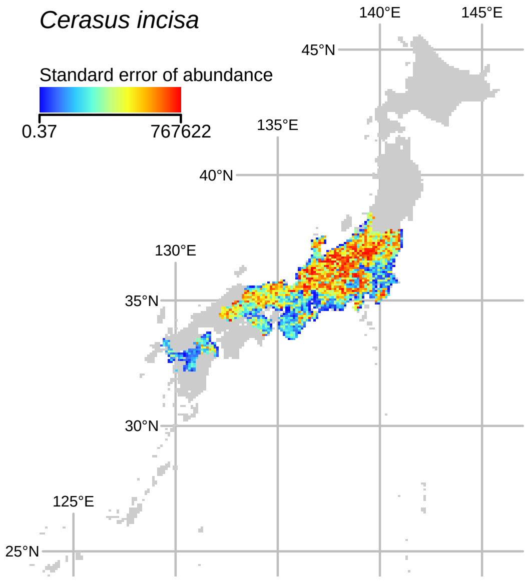
Cerasus incisa
Standard error of abundance
0.37
767622
140°E
145°E
45°N
135°E
40°N
130°E
35°N
30°N
125°E
25°N

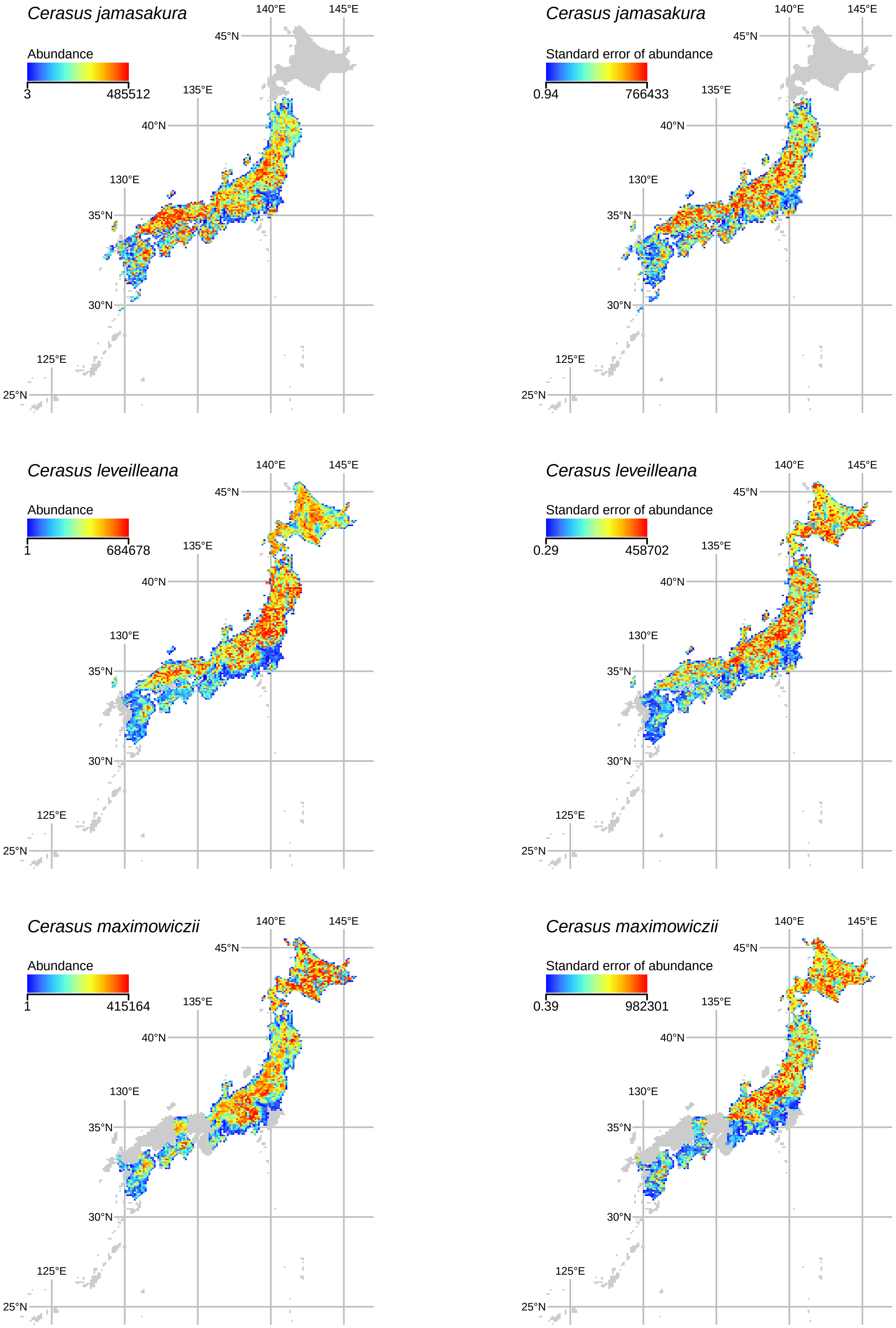
Cerasus jamasakura
Abundance
3
485512
Cerasus jamasakura
Standard error of abundance
0.94
766433
Cerasus leveilleana
Abundance
1
684678
Cerasus leveilleana
Standard error of abundance
0.29
458702
Cerasus maximowiczii
Abundance
1
415164
Cerasus maximowiczii
Standard error of abundance
0.39
982301
125°E
130°E
135°E
140°E
145°E
25°N
30°N
35°N
40°N
45°N

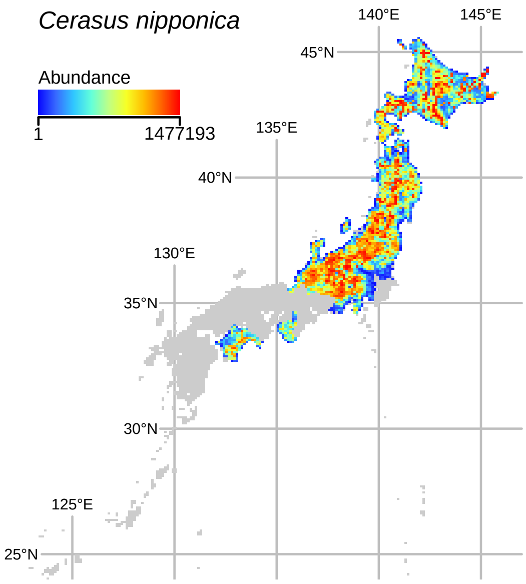
Cerasus nipponica
Abundance
1
1477193
140°E
145°E
135°E
130°E
125°E
45°N
40°N
35°N
30°N
25°N

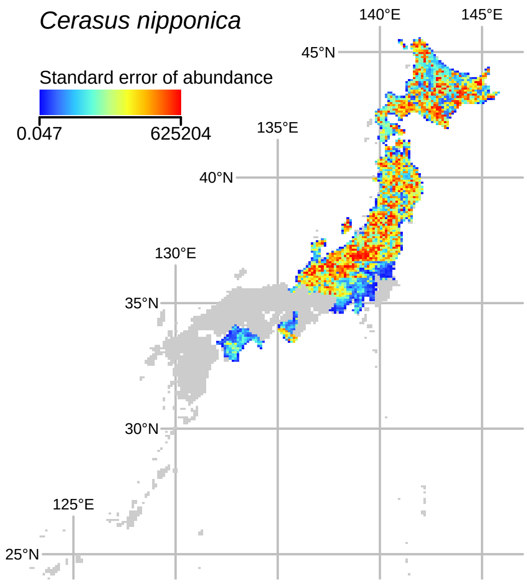
Cerasus nipponica
Standard error of abundance
0.047
625204
140°E
145°E
135°E
130°E
125°E
45°N
40°N
35°N
30°N
25°N

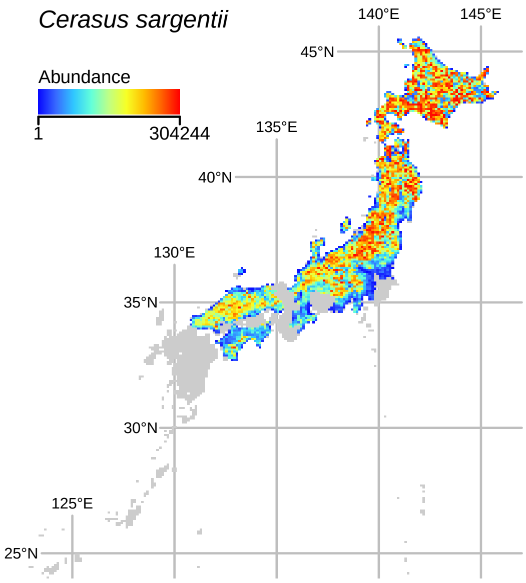
Cerasus sargentii
Abundance
1
304244
140°E
145°E
135°E
130°E
125°E
45°N
40°N
35°N
30°N
25°N

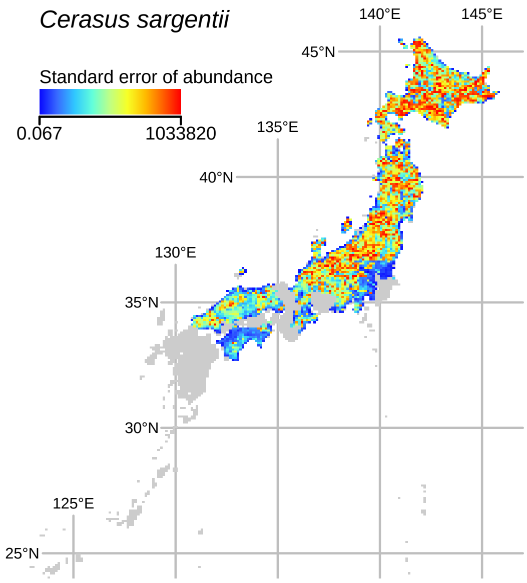
Cerasus sargentii
Standard error of abundance
0.067
1033820
140°E
145°E
135°E
130°E
125°E
45°N
40°N
35°N
30°N
25°N

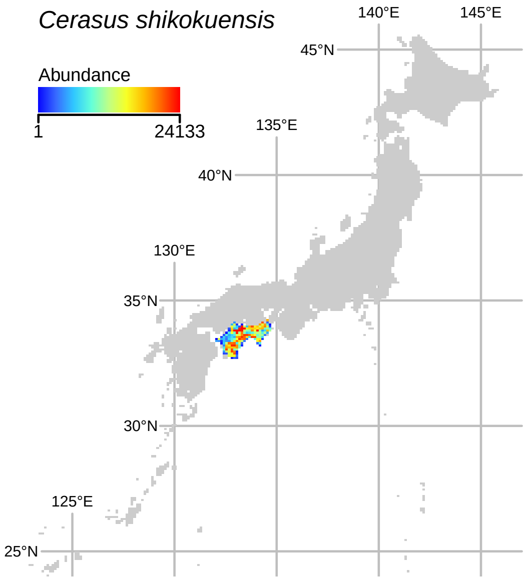
Cerasus shikokuensis
Abundance
1
24133
140°E
145°E
135°E
130°E
125°E
45°N
40°N
35°N
30°N
25°N

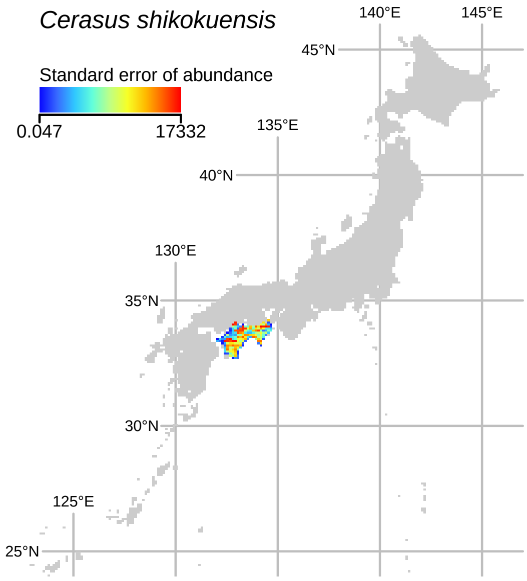
Cerasus shikokuensis
Standard error of abundance
0.047
17332
140°E
145°E
135°E
130°E
125°E
45°N
40°N
35°N
30°N
25°N

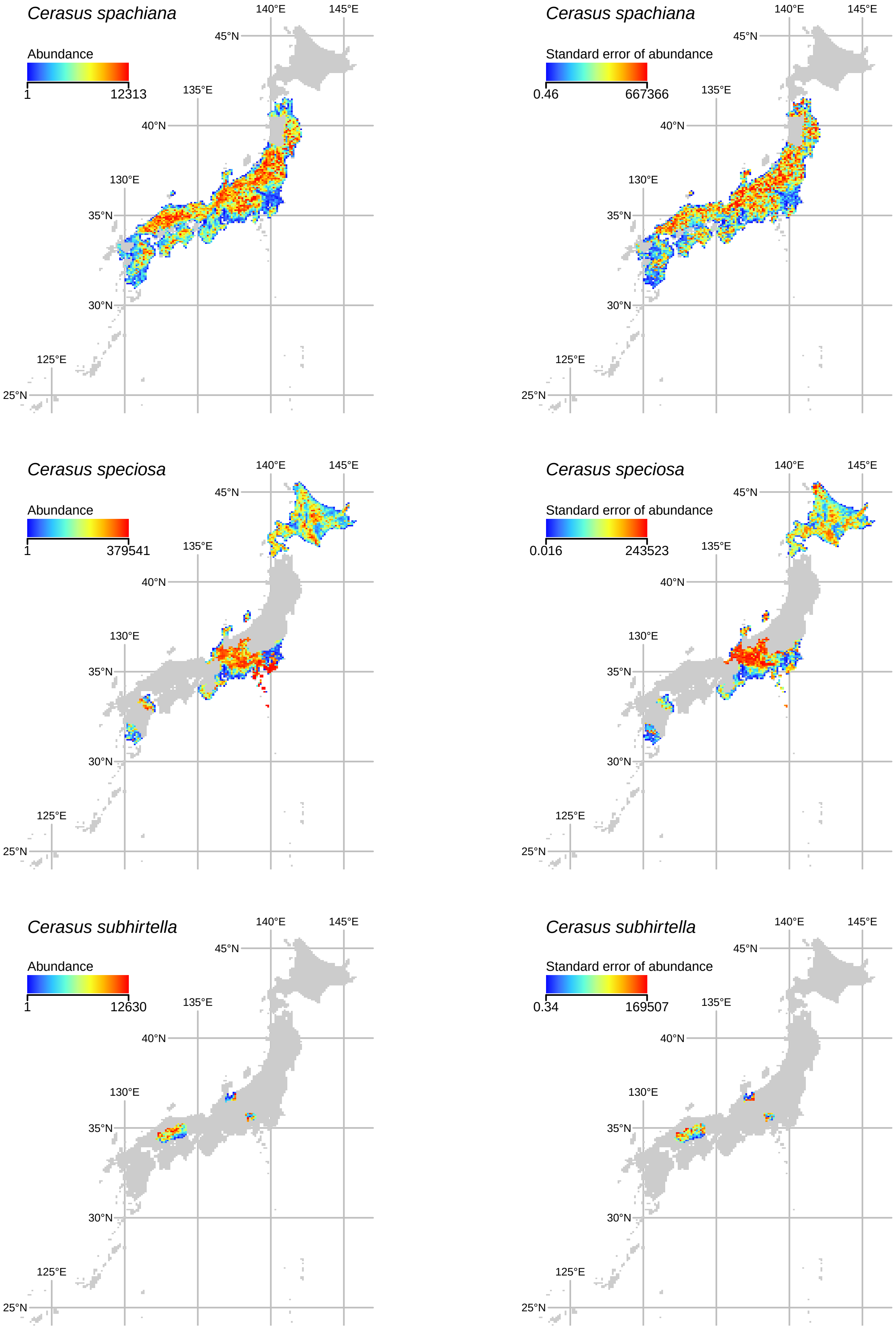
Cerasus spachiana
Abundance
1
12313
Cerasus spachiana
Standard error of abundance
0.46
667366
Cerasus speciosa
Abundance
1
379541
Cerasus speciosa
Standard error of abundance
0.016
243523
Cerasus subhirtella
Abundance
1
12630
Cerasus subhirtella
Standard error of abundance
0.34
169507
125°E
130°E
135°E
140°E
145°E
25°N
30°N
35°N
40°N
45°N

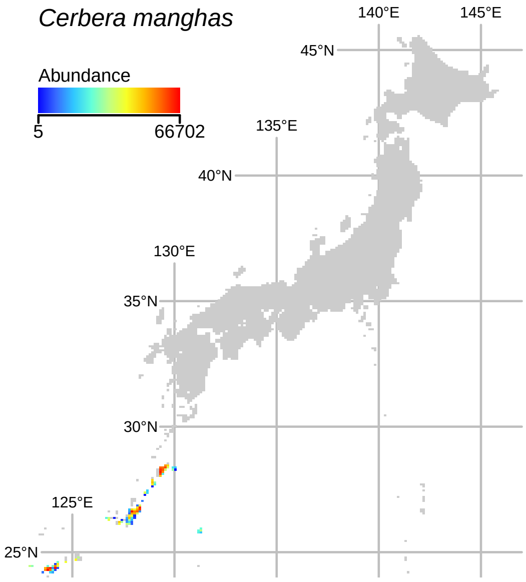
Cerbera manghas
Abundance
5
66702
140°E
145°E
45°N
135°E
40°N
130°E
35°N
30°N
125°E
25°N

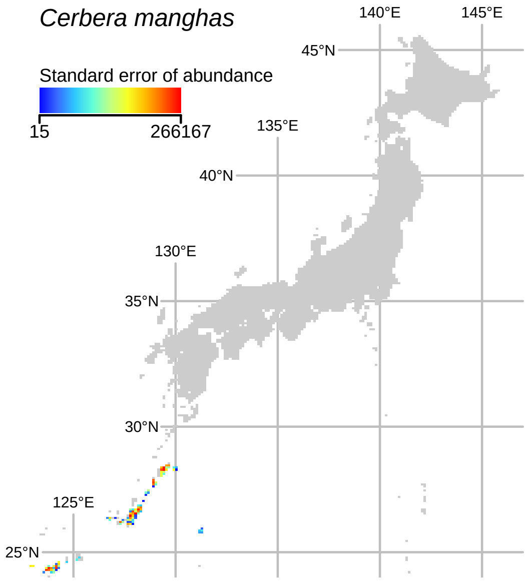
Cerbera manghas
Standard error of abundance
15
266167
140°E
145°E
45°N
135°E
40°N
130°E
35°N
30°N
125°E
25°N

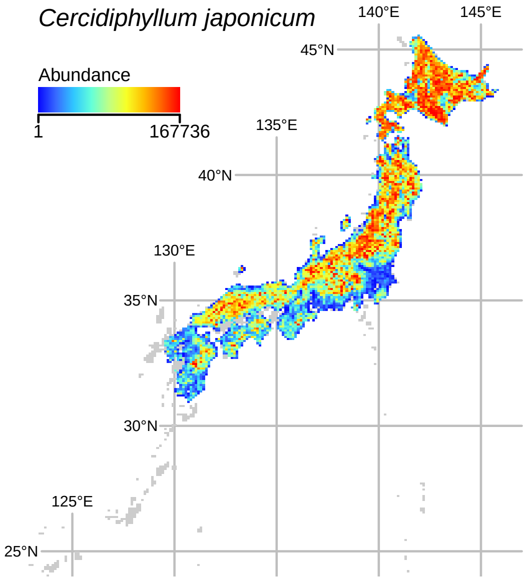
Cercidiphyllum japonicum
Abundance
1
167736
140°E
145°E
45°N
135°E
40°N
130°E
35°N
30°N
125°E
25°N

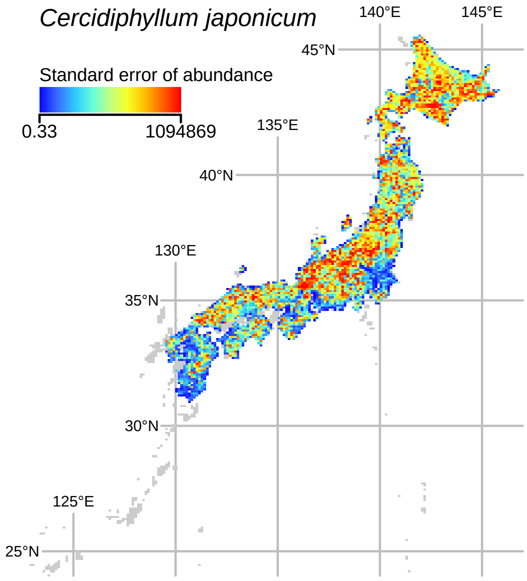
Cercidiphyllum japonicum
Standard error of abundance
0.33
1094869
140°E
145°E
45°N
135°E
40°N
130°E
35°N
30°N
125°E
25°N

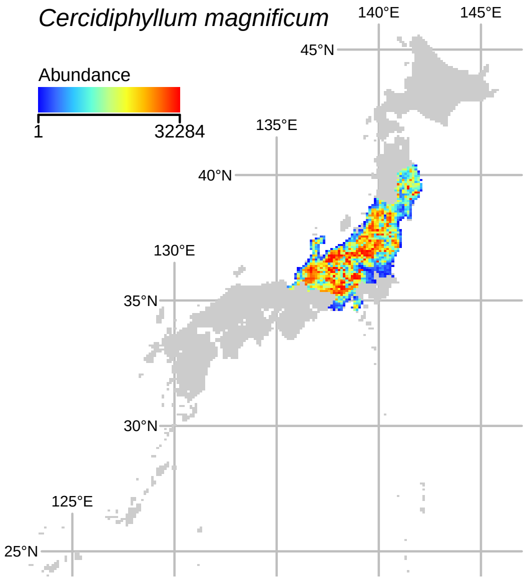
Cercidiphyllum magnificum
Abundance
1
32284
140°E
145°E
45°N
135°E
40°N
130°E
35°N
30°N
125°E
25°N

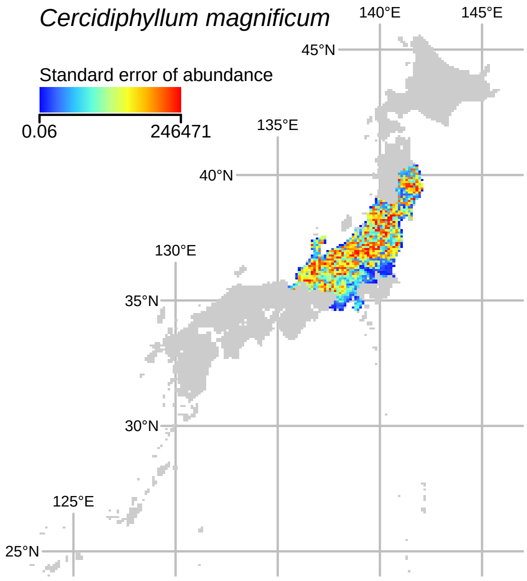
Cercidiphyllum magnificum
Standard error of abundance
0.06
246471
140°E
145°E
45°N
135°E
40°N
130°E
35°N
30°N
125°E
25°N

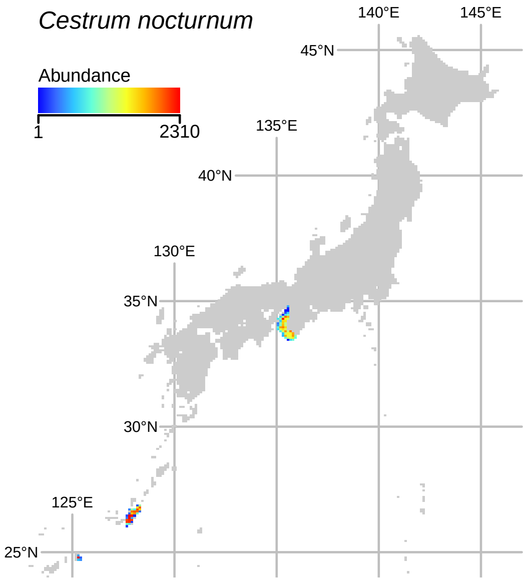
Cestrum nocturnum
Abundance
1
2310
140°E
145°E
45°N
135°E
40°N
130°E
35°N
30°N
125°E
25°N

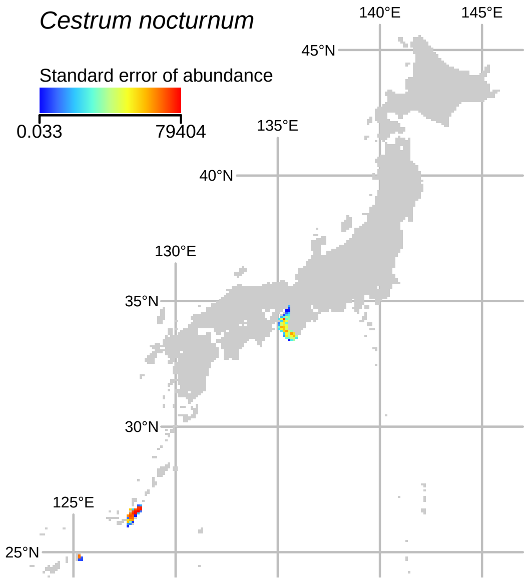
Cestrum nocturnum
Standard error of abundance
0.033
79404
140°E
145°E
45°N
135°E
40°N
130°E
35°N
30°N
125°E
25°N

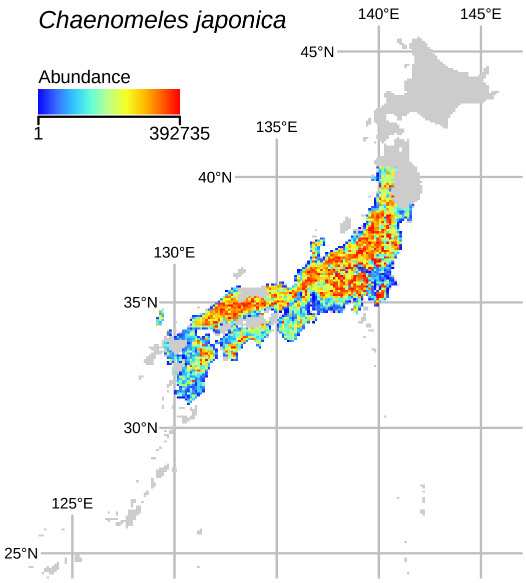
Chaenomeles japonica
Abundance
1
392735
140°E
145°E
45°N
135°E
40°N
130°E
35°N
30°N
125°E
25°N

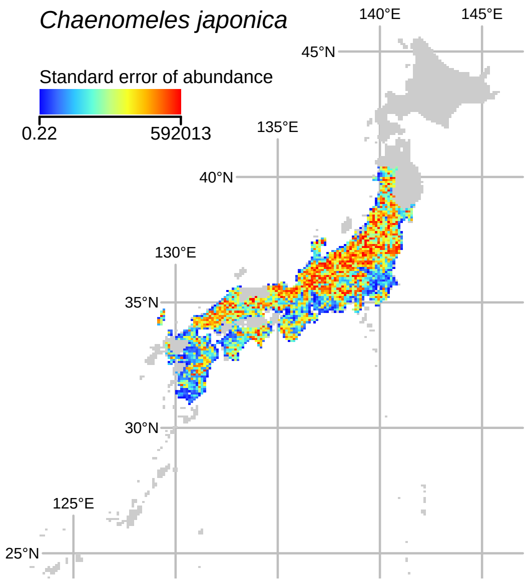
Chaenomeles japonica
Standard error of abundance
0.22
592013
140°E
145°E
45°N
135°E
40°N
130°E
35°N
30°N
125°E
25°N

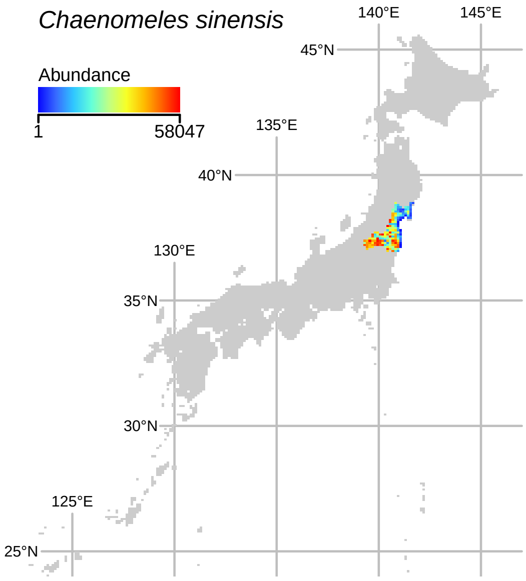
Chaenomeles sinensis
Abundance
1
58047
140°E
145°E
45°N
135°E
40°N
130°E
35°N
30°N
125°E
25°N

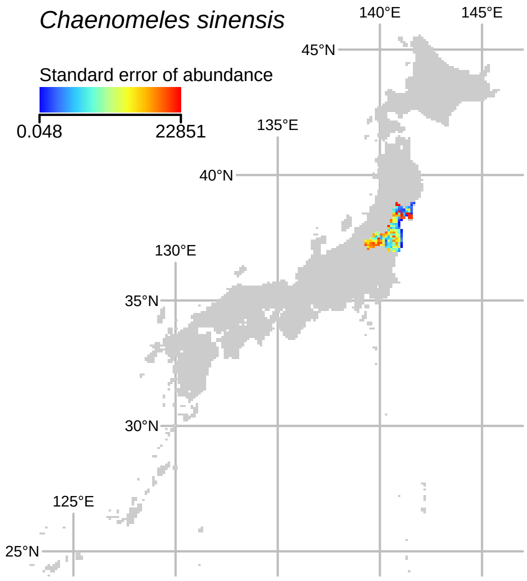
Chaenomeles sinensis
Standard error of abundance
0.048
22851
140°E
145°E
45°N
135°E
40°N
130°E
35°N
30°N
125°E
25°N

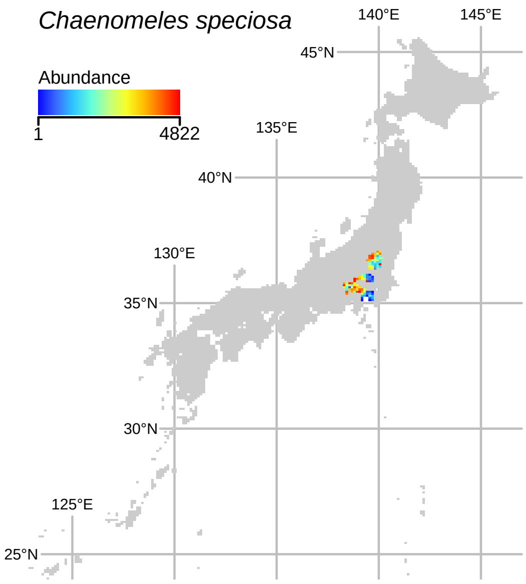
Chaenomeles speciosa
Abundance
1
4822
140°E
145°E
45°N
135°E
40°N
130°E
35°N
30°N
125°E
25°N

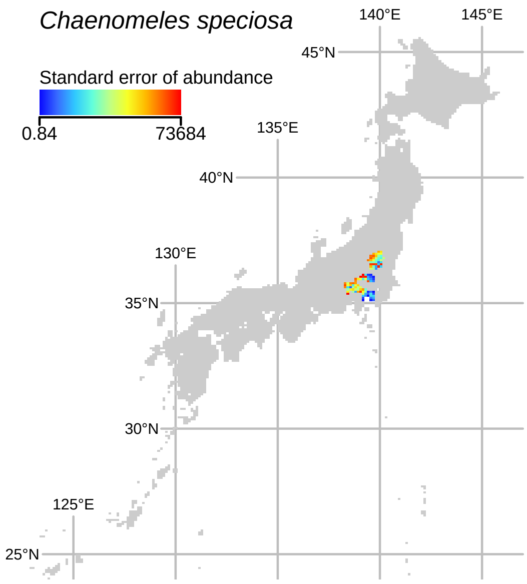
Chaenomeles speciosa
Standard error of abundance
0.84
73684
140°E
145°E
45°N
135°E
40°N
130°E
35°N
30°N
125°E
25°N

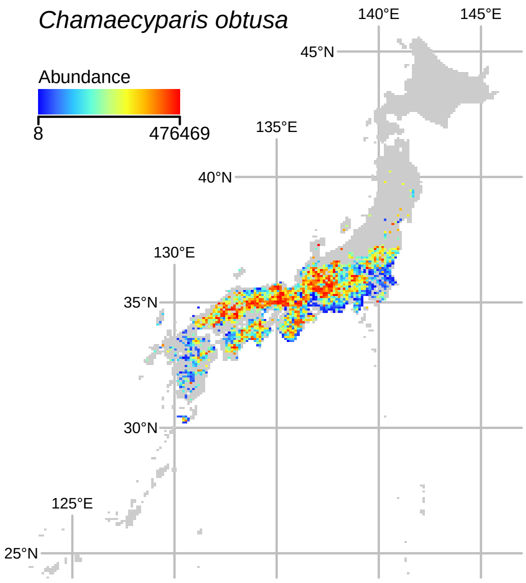
Chamaecyparis obtusa
Abundance
8
476469
140°E
145°E
45°N
135°E
40°N
130°E
35°N
30°N
125°E
25°N

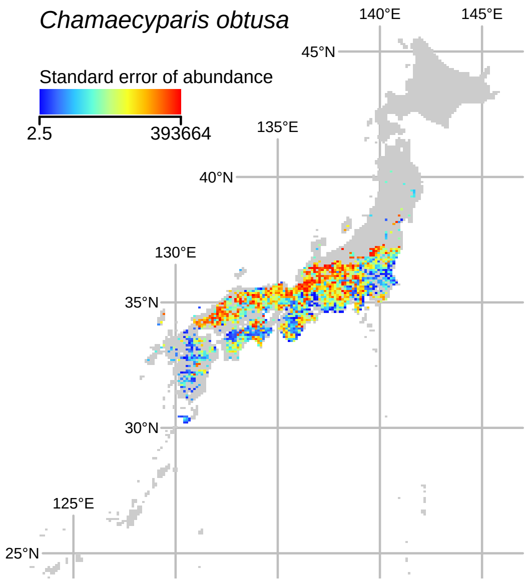
Chamaecyparis obtusa
Standard error of abundance
2.5
393664
140°E
145°E
45°N
135°E
40°N
130°E
35°N
30°N
125°E
25°N

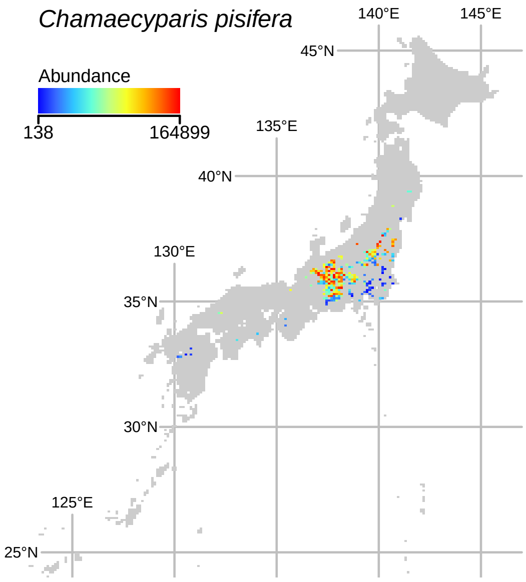
Chamaecyparis pisifera
Abundance
138
164899
140°E
145°E
45°N
135°E
40°N
130°E
35°N
30°N
125°E
25°N

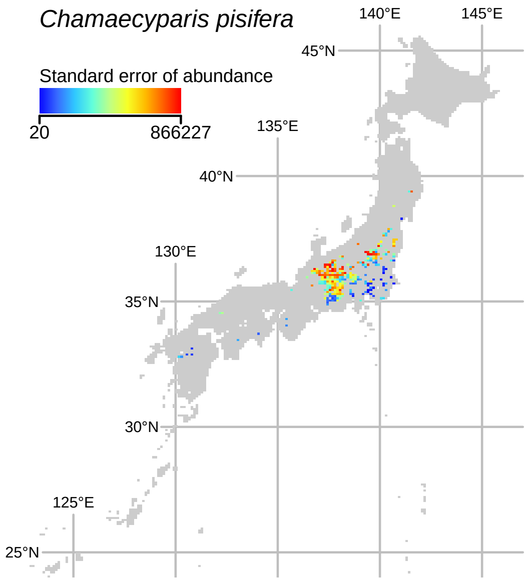
Chamaecyparis pisifera
Standard error of abundance
20
866227
140°E
145°E
45°N
135°E
40°N
130°E
35°N
30°N
125°E
25°N

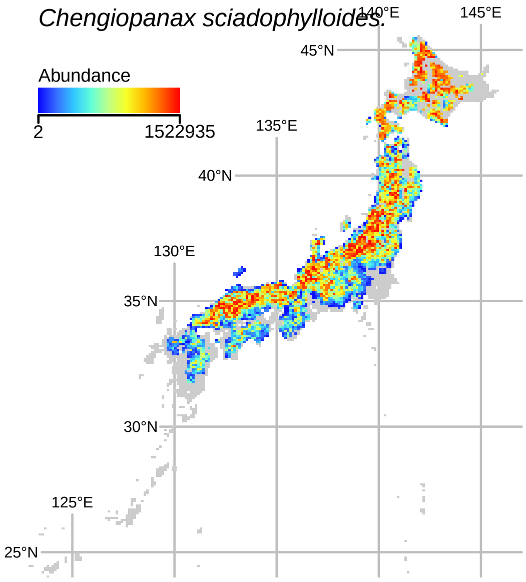
Chengiopanax sciadophylloides.
Abundance
2
1522935
125°E
130°E
135°E
140°E
145°E
25°N
30°N
35°N
40°N
45°N

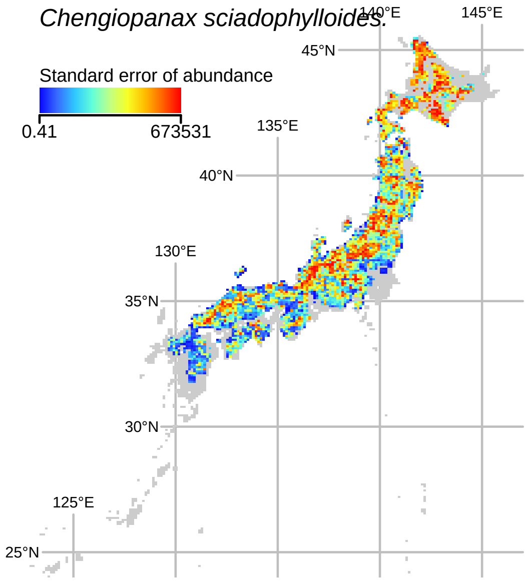
Chengiopanax sciadophylloides.
Standard error of abundance
0.41
673531
125°E
130°E
135°E
140°E
145°E
25°N
30°N
35°N
40°N
45°N

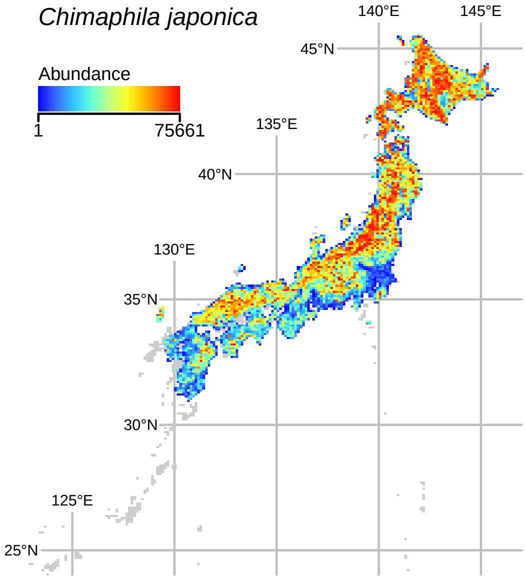
Chimaphila japonica
Abundance
1
75661
125°E
130°E
135°E
140°E
145°E
25°N
30°N
35°N
40°N
45°N

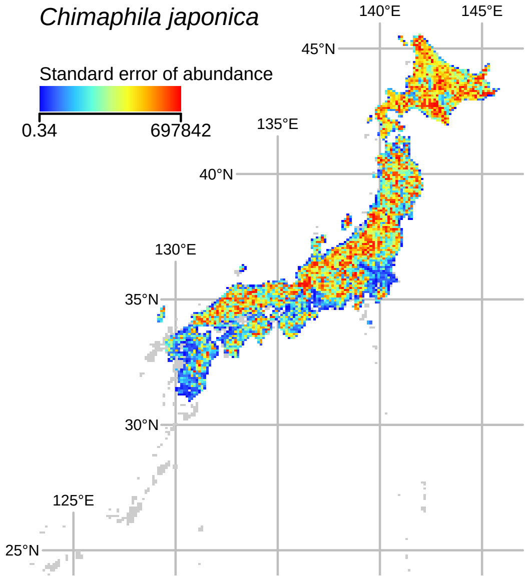
Chimaphila japonica
Standard error of abundance
0.34
697842
125°E
130°E
135°E
140°E
145°E
25°N
30°N
35°N
40°N
45°N

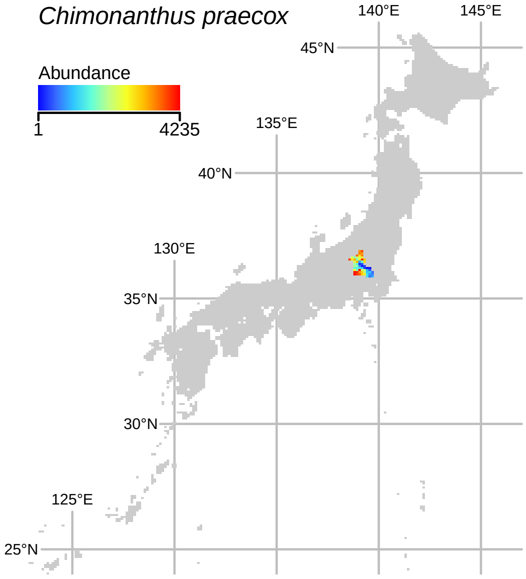
Chimonanthus praecox
Abundance
1
4235
125°E
130°E
135°E
140°E
145°E
25°N
30°N
35°N
40°N
45°N

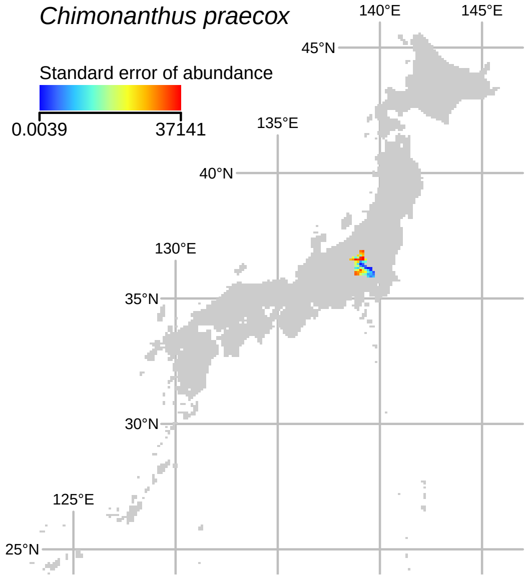
Chimonanthus praecox
Standard error of abundance
0.0039
37141
125°E
130°E
135°E
140°E
145°E
25°N
30°N
35°N
40°N
45°N

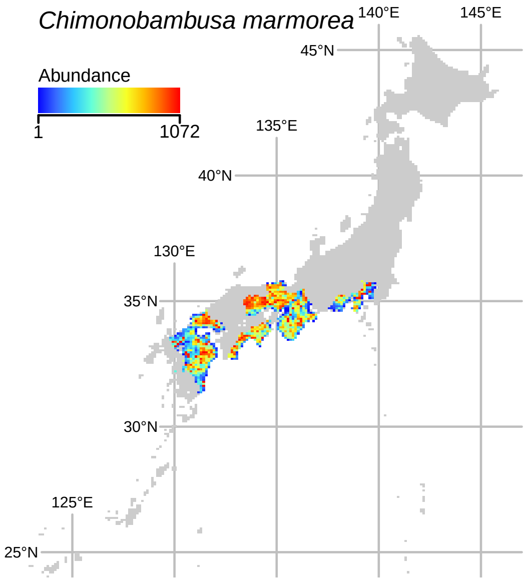
Chimonobambusa marmorea
Abundance
1
1072
140°E
145°E
45°N
135°E
40°N
130°E
35°N
30°N
125°E
25°N

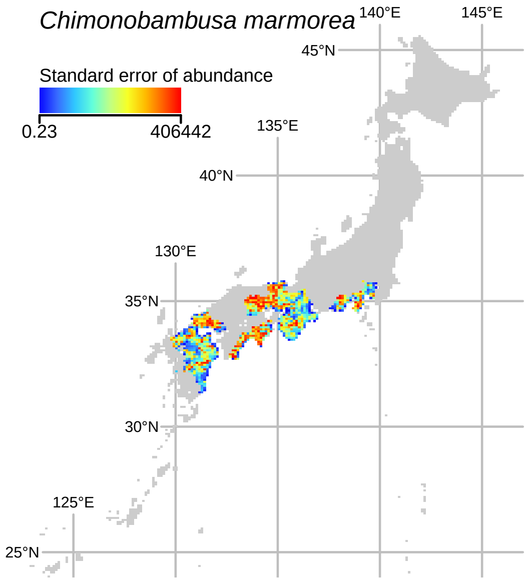
Chimonobambusa marmorea
Standard error of abundance
0.23
406442
140°E
145°E
45°N
135°E
40°N
130°E
35°N
30°N
125°E
25°N

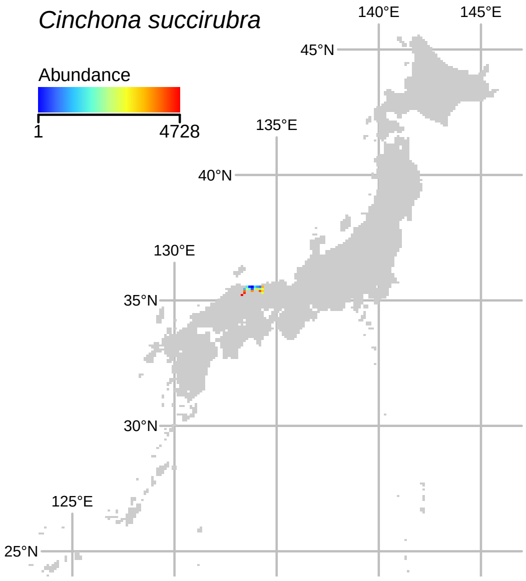
Cinchona succirubra
Abundance
1
4728
140°E
145°E
45°N
135°E
40°N
130°E
35°N
30°N
125°E
25°N

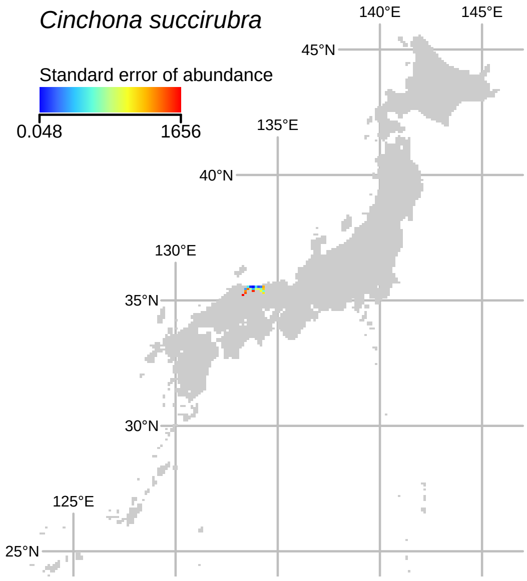
Cinchona succirubra
Standard error of abundance
0.048
1656
140°E
145°E
45°N
135°E
40°N
130°E
35°N
30°N
125°E
25°N

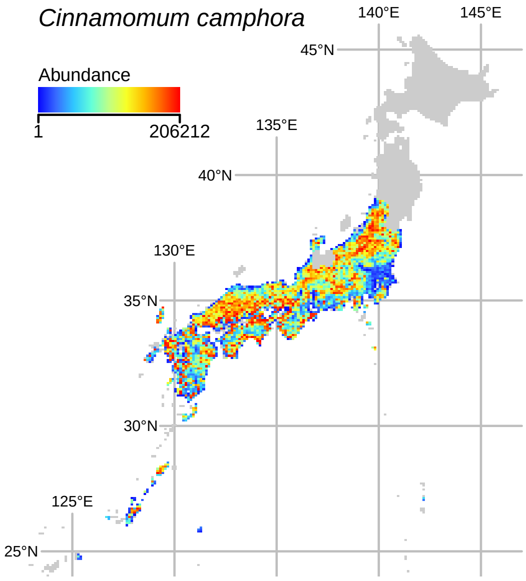
Cinnamomum camphora
Abundance
1
206212
140°E
145°E
45°N
135°E
40°N
130°E
35°N
30°N
125°E
25°N

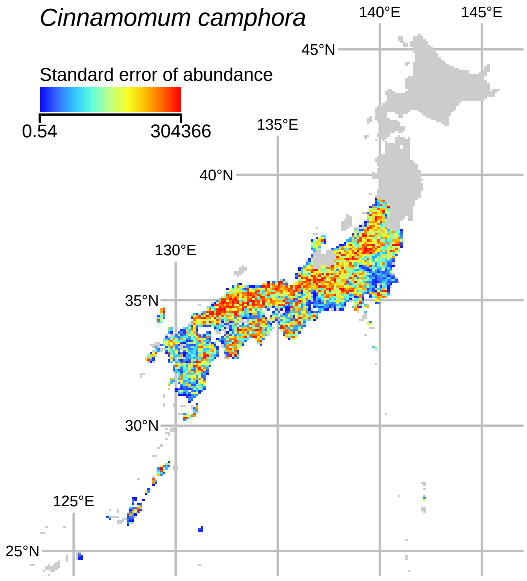
Cinnamomum camphora
Standard error of abundance
0.54
304366
140°E
145°E
45°N
135°E
40°N
130°E
35°N
30°N
125°E
25°N

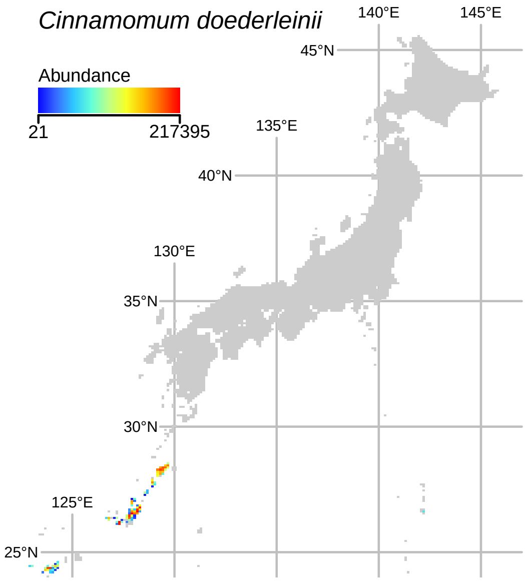
Cinnamomum doederleinii
Abundance
21
217395
140°E
145°E
45°N
135°E
40°N
130°E
35°N
30°N
125°E
25°N

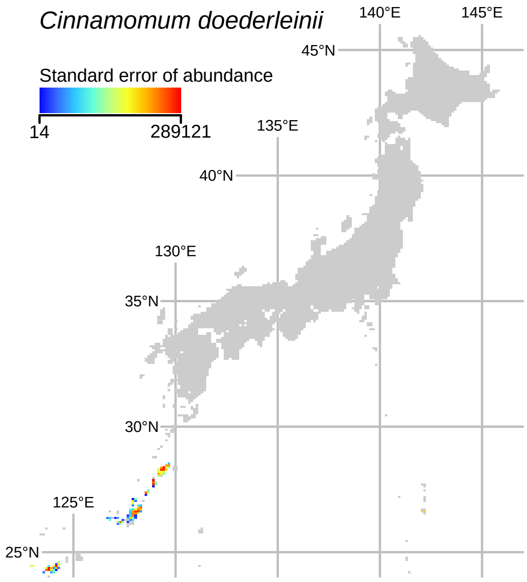
Cinnamomum doederleinii
Standard error of abundance
14
289121
140°E
145°E
45°N
135°E
40°N
130°E
35°N
30°N
125°E
25°N

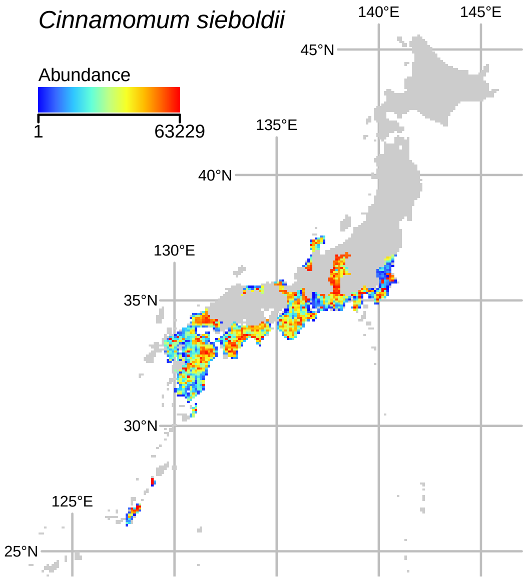
Cinnamomum sieboldii
Abundance
1
63229
140°E
145°E
45°N
135°E
40°N
130°E
35°N
30°N
125°E
25°N

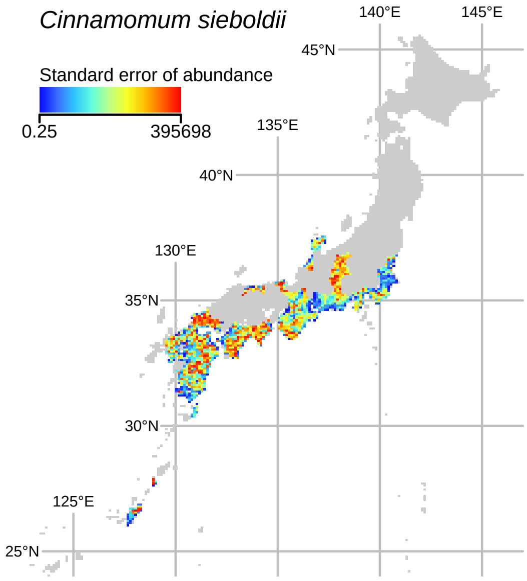
Cinnamomum sieboldii
Standard error of abundance
0.25
395698
140°E
145°E
45°N
135°E
40°N
130°E
35°N
30°N
125°E
25°N

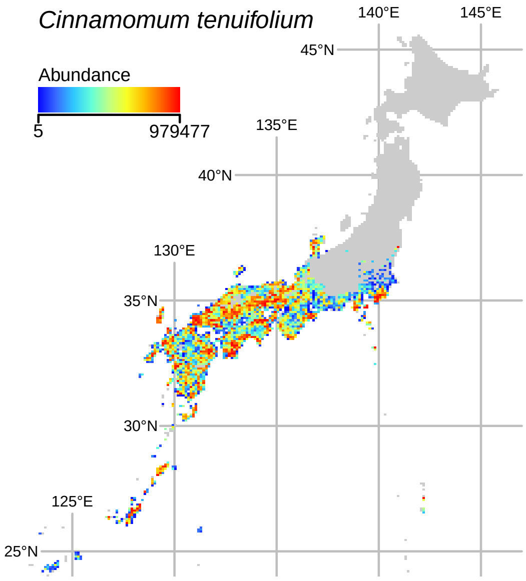
Cinnamomum tenuifolium
Abundance
5
979477
140°E
145°E
45°N
135°E
40°N
130°E
35°N
30°N
125°E
25°N

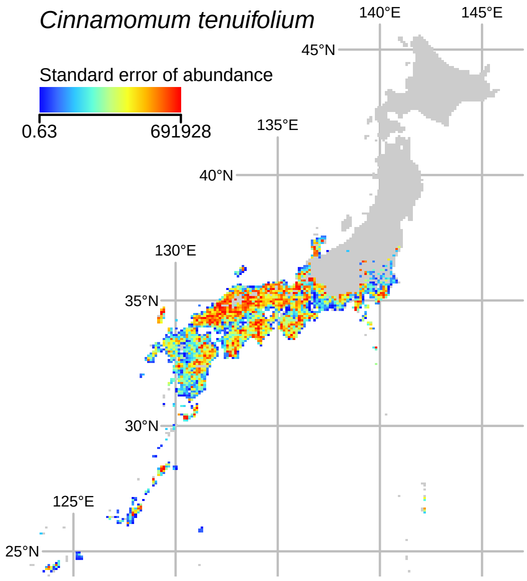
Cinnamomum tenuifolium
Standard error of abundance
0.63
691928
140°E
145°E
45°N
135°E
40°N
130°E
35°N
30°N
125°E
25°N

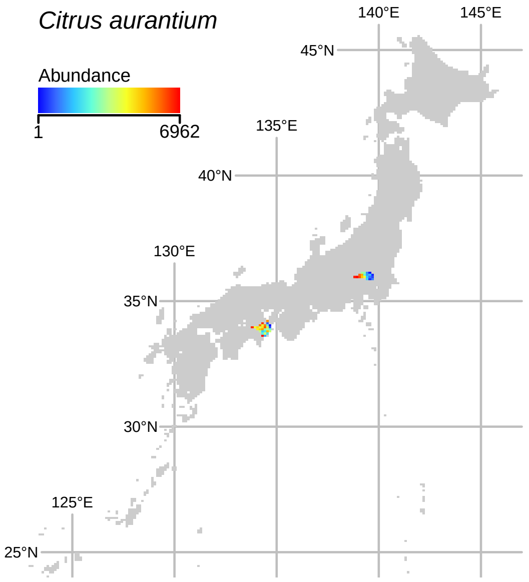
Citrus aurantium
Abundance
1
6962
140°E
145°E
45°N
135°E
40°N
130°E
35°N
30°N
125°E
25°N

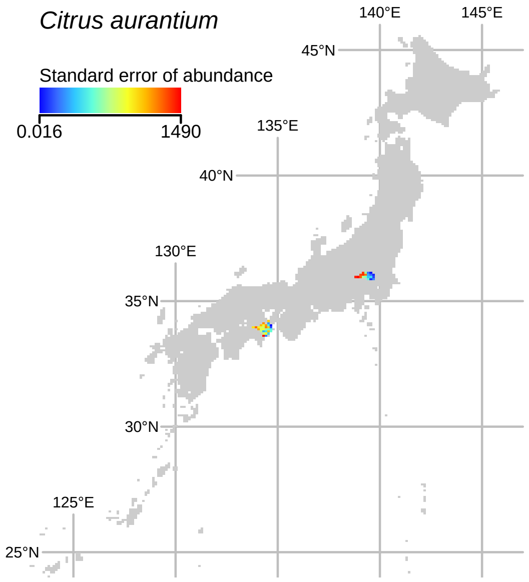
Citrus aurantium
Standard error of abundance
0.016
1490
140°E
145°E
45°N
135°E
40°N
130°E
35°N
30°N
125°E
25°N

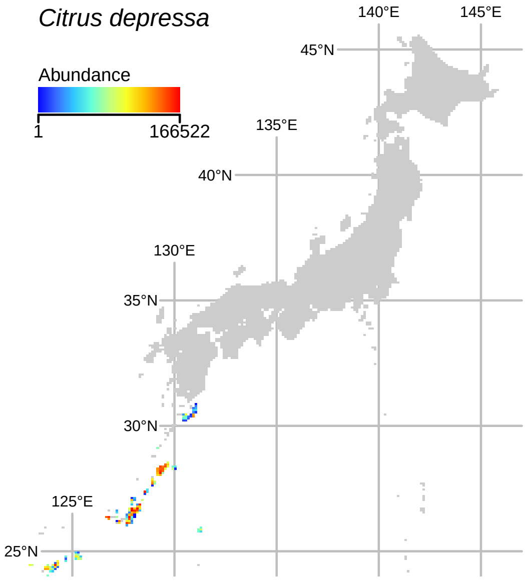
Citrus depressa
Abundance
1
166522
140°E
145°E
45°N
135°E
40°N
130°E
35°N
30°N
125°E
25°N

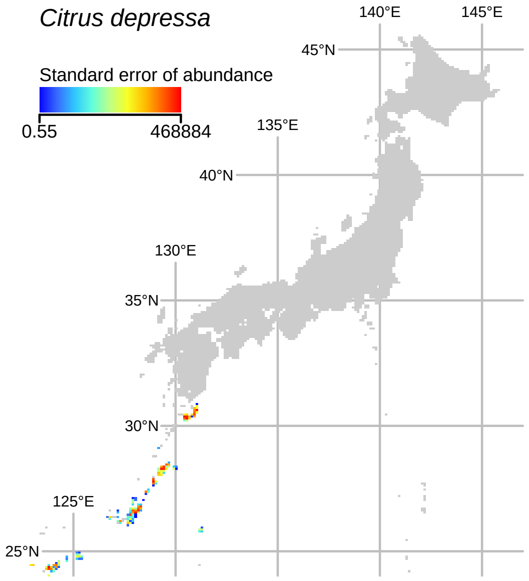
Citrus depressa
Standard error of abundance
0.55
468884
140°E
145°E
45°N
135°E
40°N
130°E
35°N
30°N
125°E
25°N

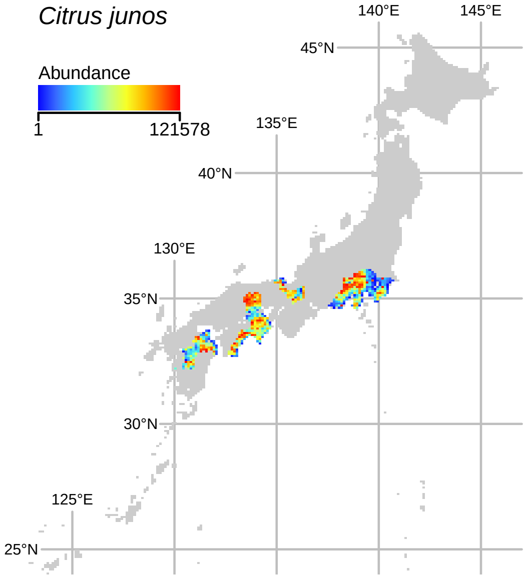
Citrus junos
Abundance
1
121578
140°E
145°E
45°N
135°E
40°N
130°E
35°N
30°N
125°E
25°N

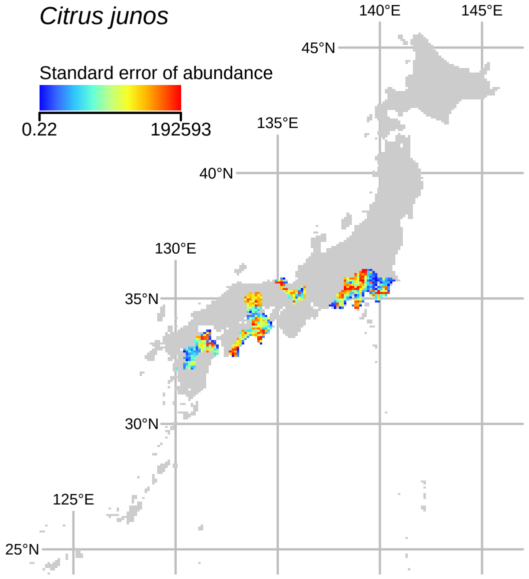
Citrus junos
Standard error of abundance
0.22
192593
140°E
145°E
45°N
135°E
40°N
130°E
35°N
30°N
125°E
25°N

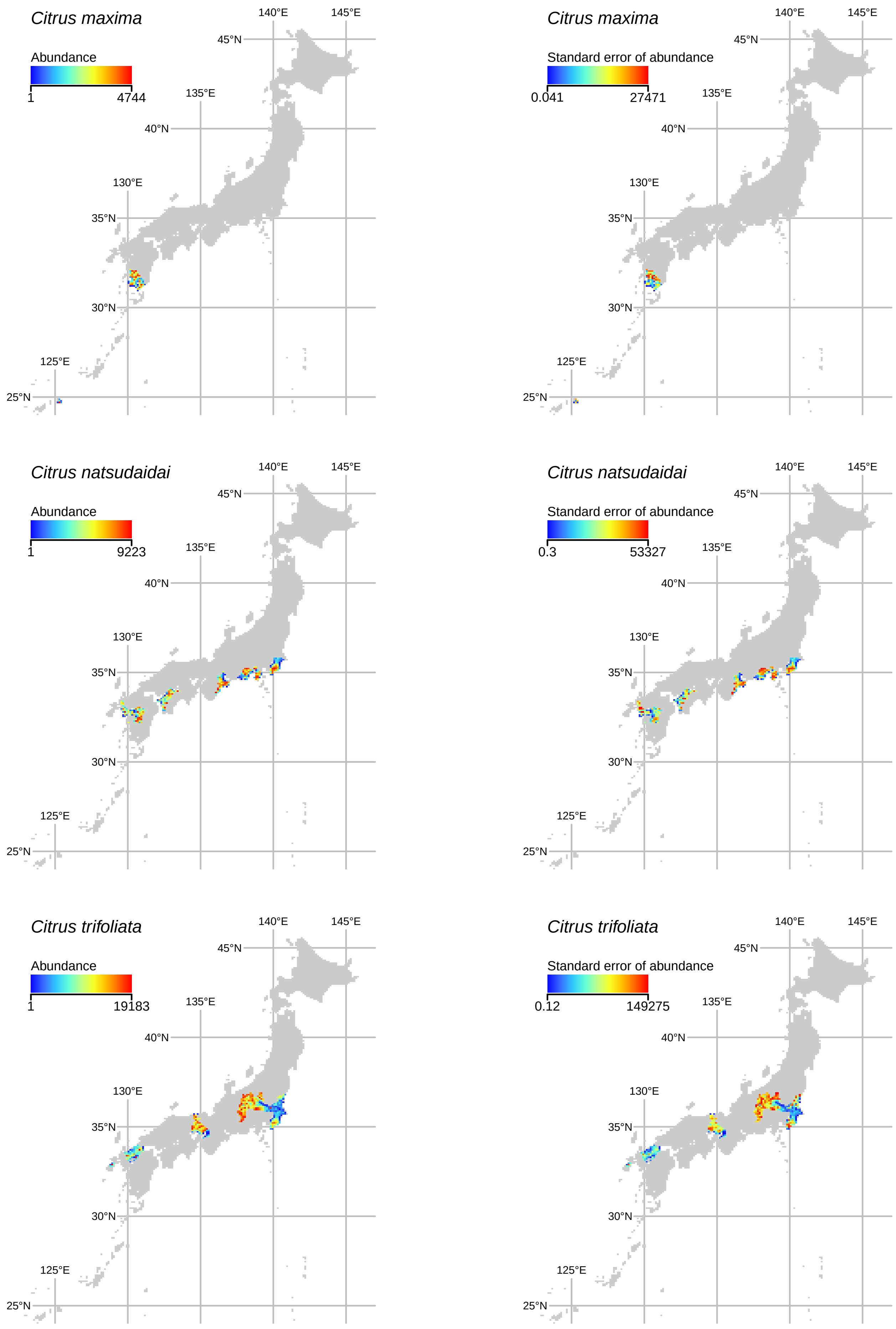
Citrus maxima
Abundance
1
4744
Citrus maxima
Standard error of abundance
0.041
27471
Citrus natsudaidai
Abundance
1
9223
Citrus natsudaidai
Standard error of abundance
0.3
53327
Citrus trifoliata
Abundance
1
19183
Citrus trifoliata
Standard error of abundance
0.12
149275
140°E
145°E
45°N
135°E
40°N
130°E
35°N
30°N
125°E
25°N

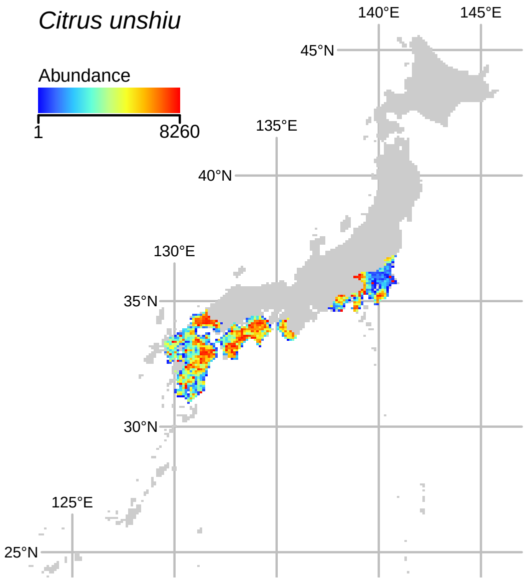
Citrus unshiu
Abundance
1
8260
140°E
145°E
45°N
135°E
40°N
130°E
35°N
30°N
125°E
25°N

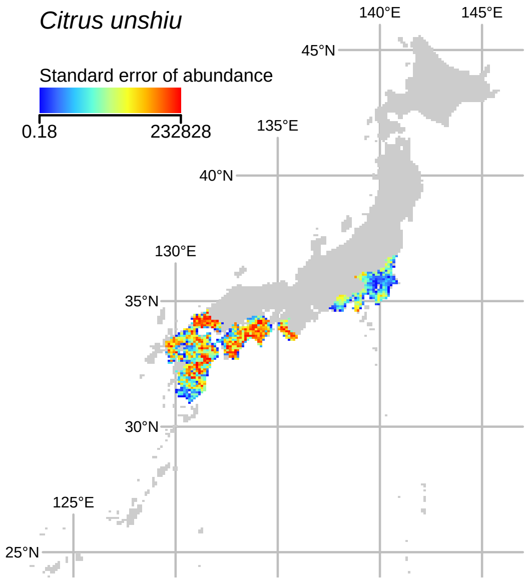
Citrus unshiu
Standard error of abundance
0.18
232828
140°E
145°E
45°N
135°E
40°N
130°E
35°N
30°N
125°E
25°N

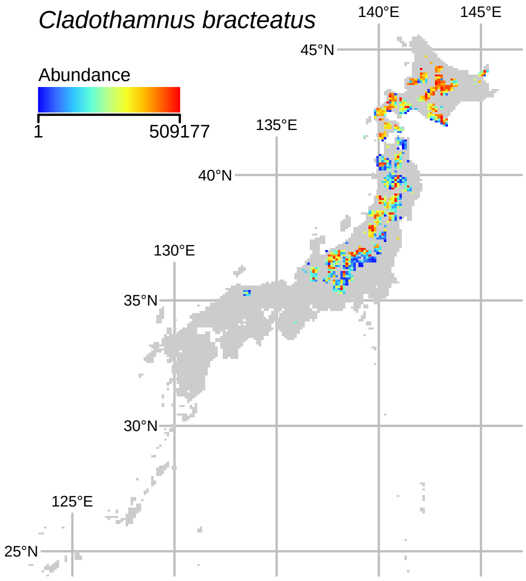
Cladothamnus bracteatus
Abundance
1
509177
140°E
145°E
45°N
135°E
40°N
130°E
35°N
30°N
125°E
25°N

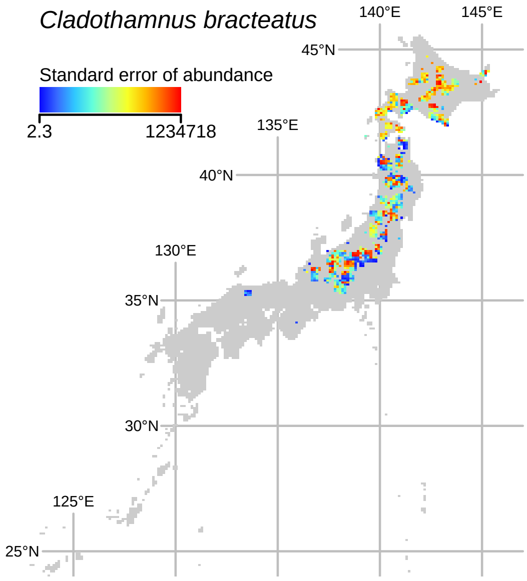
Cladothamnus bracteatus
Standard error of abundance
2.3
1234718
140°E
145°E
45°N
135°E
40°N
130°E
35°N
30°N
125°E
25°N

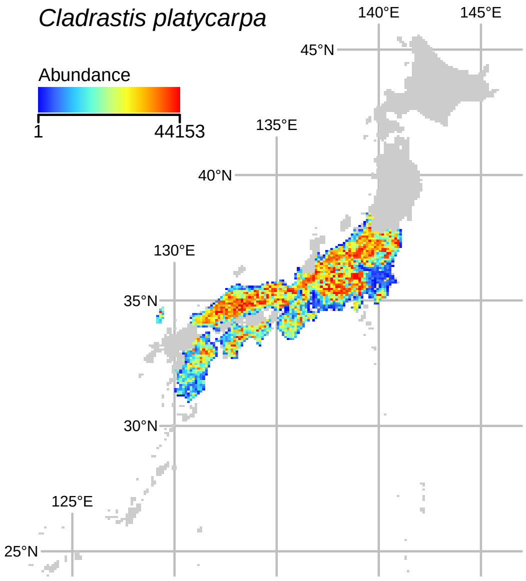
Cladrastis platycarpa
Abundance
1
44153
140°E
145°E
45°N
135°E
40°N
130°E
35°N
30°N
125°E
25°N

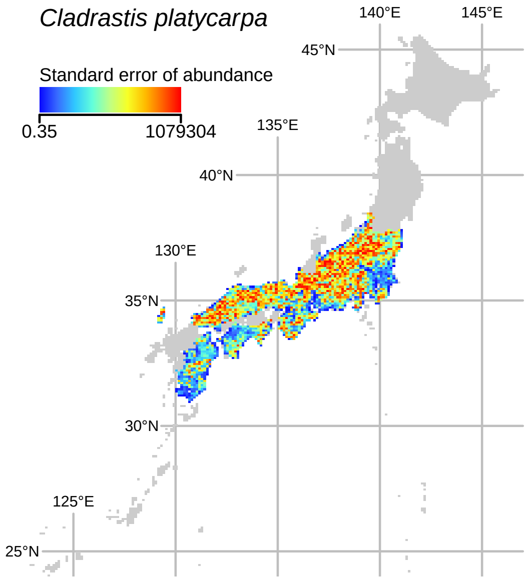
Cladrastis platycarpa
Standard error of abundance
0.35
1079304
140°E
145°E
45°N
135°E
40°N
130°E
35°N
30°N
125°E
25°N

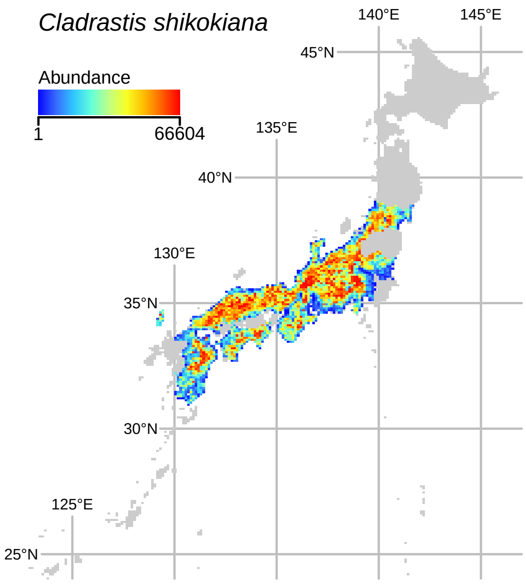
Cladrastis shikokiana
Abundance
1
66604
140°E
145°E
135°E
130°E
125°E
45°N
40°N
35°N
30°N
25°N

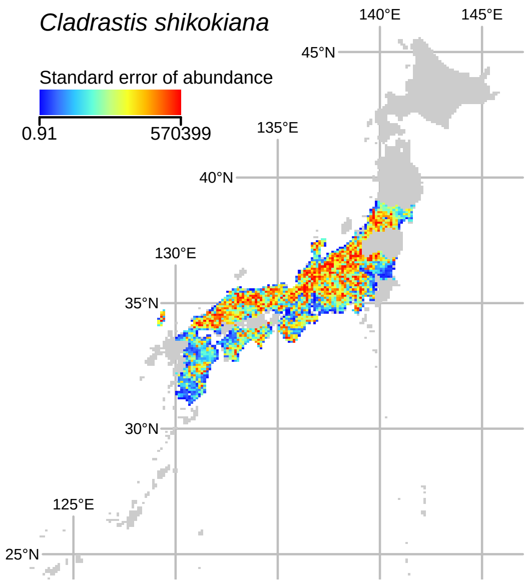
Cladrastis shikokiana
Standard error of abundance
0.91
570399
140°E
145°E
135°E
130°E
125°E
45°N
40°N
35°N
30°N
25°N

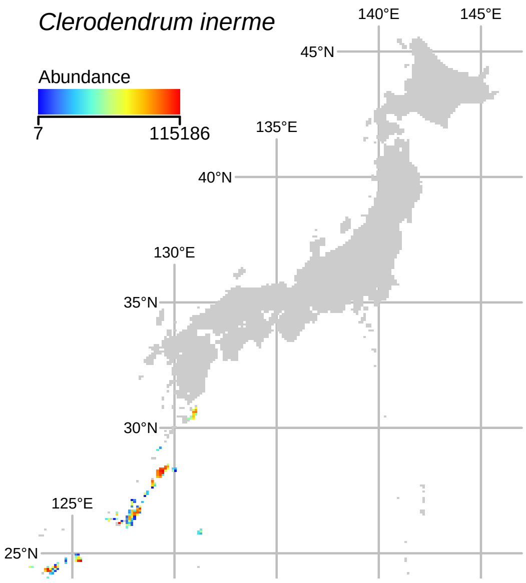
Clerodendrum inerme
Abundance
7
115186
140°E
145°E
135°E
130°E
125°E
45°N
40°N
35°N
30°N
25°N

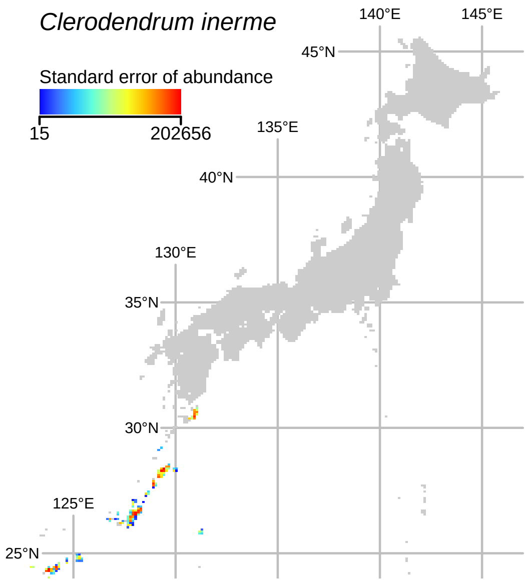
Clerodendrum inerme
Standard error of abundance
15
202656
140°E
145°E
135°E
130°E
125°E
45°N
40°N
35°N
30°N
25°N

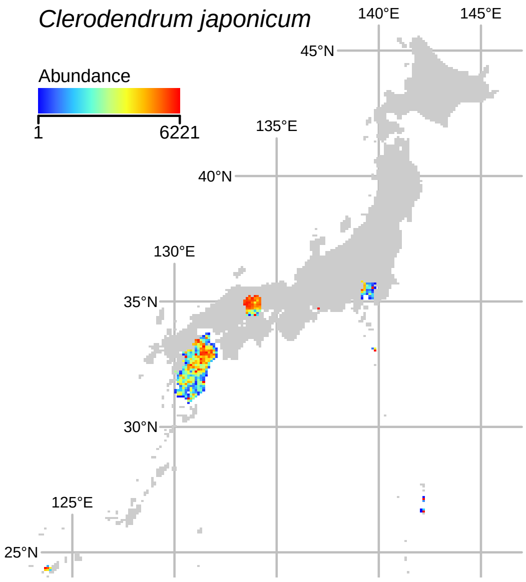
Clerodendrum japonicum
Abundance
1
6221
140°E
145°E
135°E
130°E
125°E
45°N
40°N
35°N
30°N
25°N

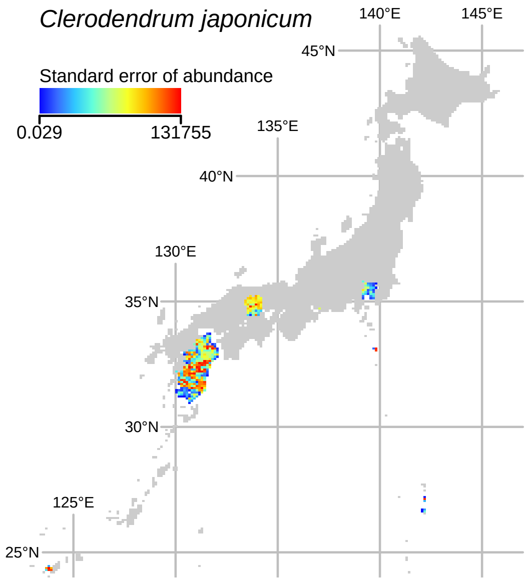
Clerodendrum japonicum
Standard error of abundance
0.029
131755
140°E
145°E
135°E
130°E
125°E
45°N
40°N
35°N
30°N
25°N

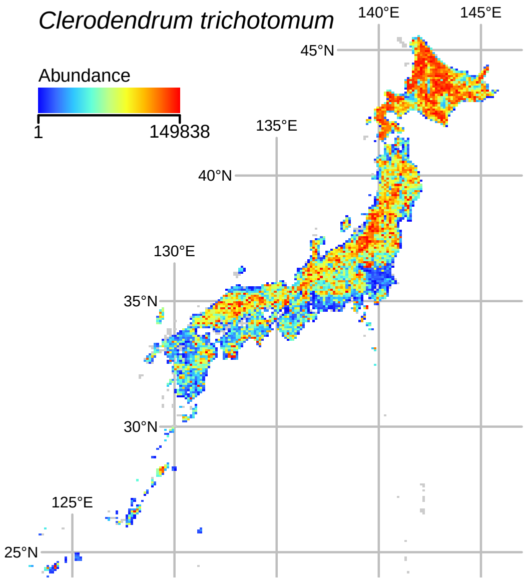
Clerodendrum trichotomum
Abundance
1
149838
140°E
145°E
45°N
135°E
40°N
130°E
35°N
30°N
125°E
25°N

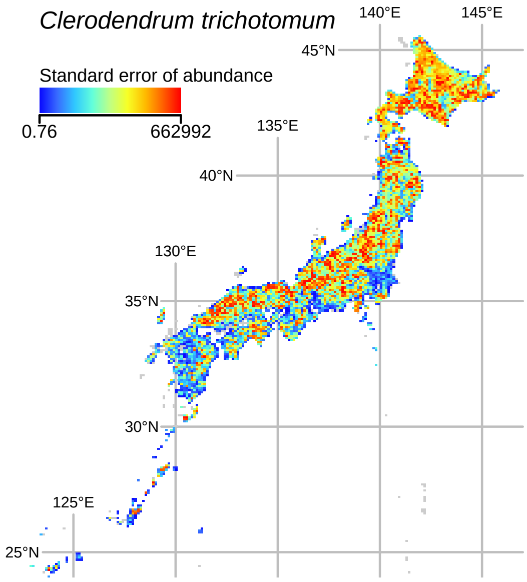
Clerodendrum trichotomum
Standard error of abundance
0.76
662992
140°E
145°E
45°N
135°E
40°N
130°E
35°N
30°N
125°E
25°N

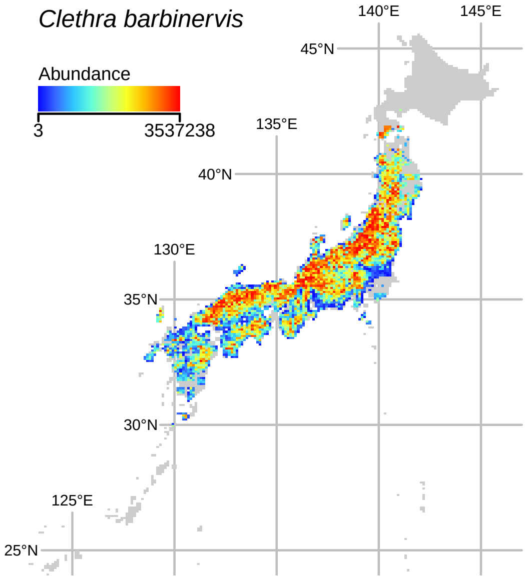
Clethra barbinervis
Abundance
3
3537238
140°E
145°E
45°N
135°E
40°N
130°E
35°N
30°N
125°E
25°N

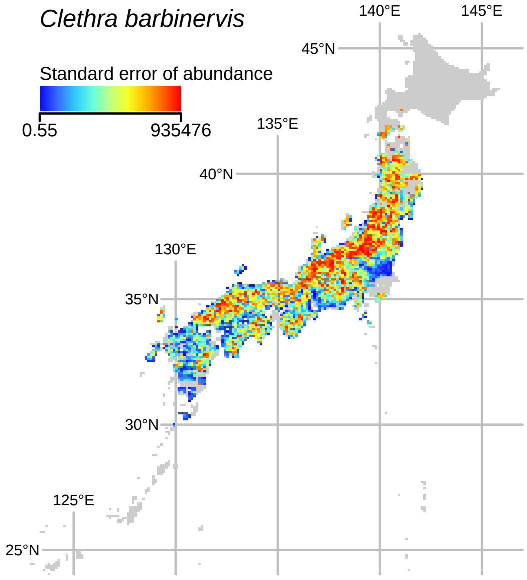
Clethra barbinervis
Standard error of abundance
0.55
935476
140°E
145°E
45°N
135°E
40°N
130°E
35°N
30°N
125°E
25°N

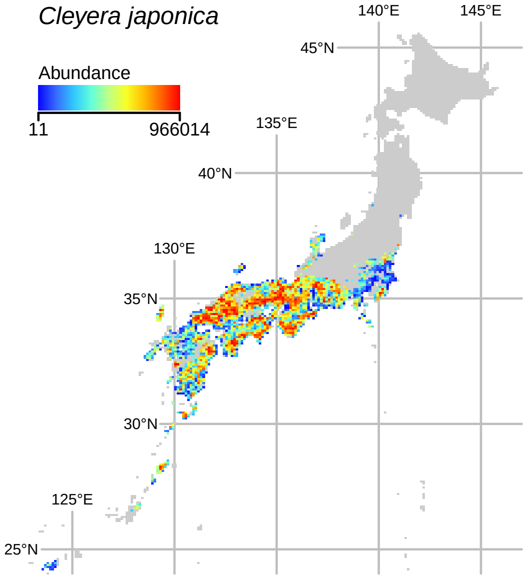
Cleyera japonica
Abundance
11
966014
140°E
145°E
45°N
135°E
40°N
130°E
35°N
30°N
125°E
25°N

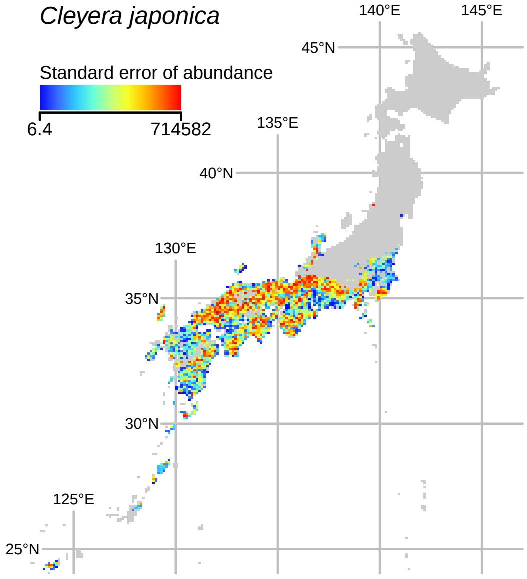
Cleyera japonica
Standard error of abundance
6.4
714582
140°E
145°E
45°N
135°E
40°N
130°E
35°N
30°N
125°E
25°N

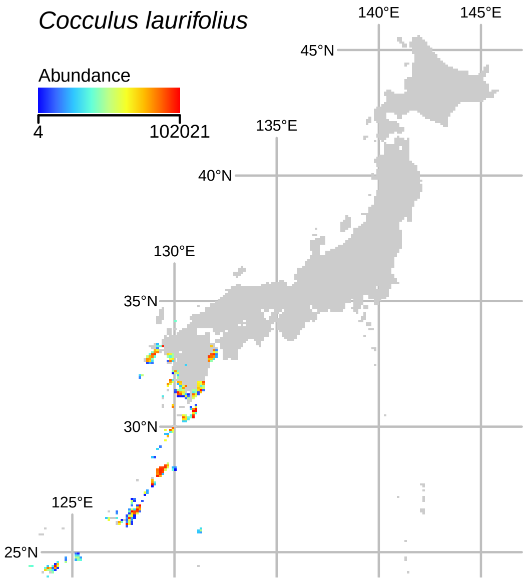
Cocculus laurifolius
Abundance
4
102021
140°E
145°E
45°N
135°E
40°N
130°E
35°N
30°N
125°E
25°N

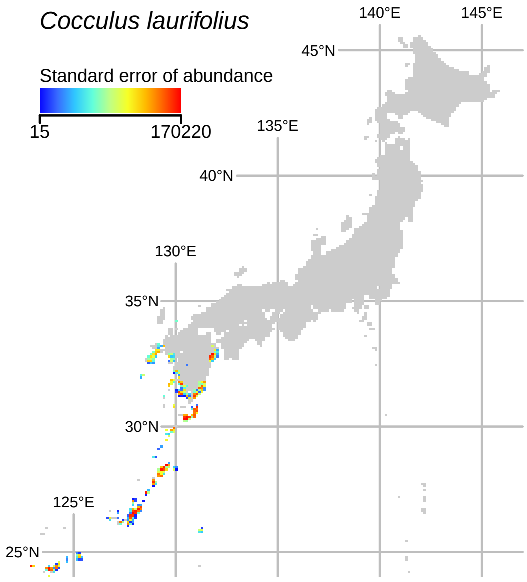
Cocculus laurifolius
Standard error of abundance
15
170220
140°E
145°E
45°N
135°E
40°N
130°E
35°N
30°N
125°E
25°N

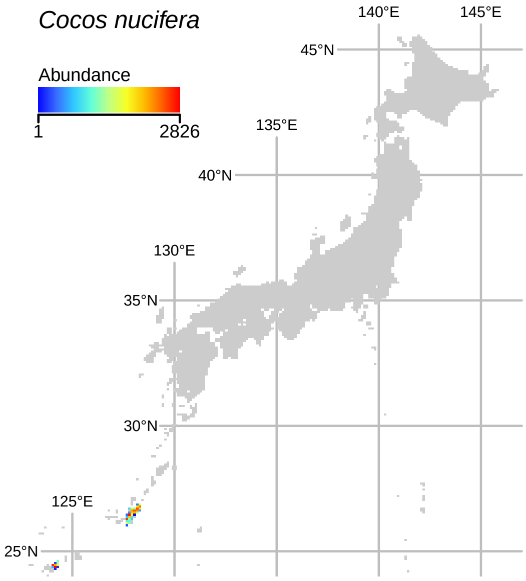
Cocos nucifera
Abundance
1
2826
140°E
145°E
45°N
135°E
40°N
130°E
35°N
30°N
125°E
25°N

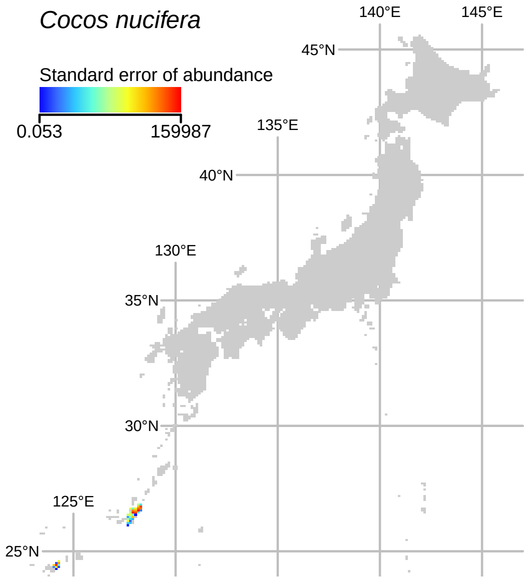
Cocos nucifera
Standard error of abundance
0.053
159987
140°E
145°E
45°N
135°E
40°N
130°E
35°N
30°N
125°E
25°N

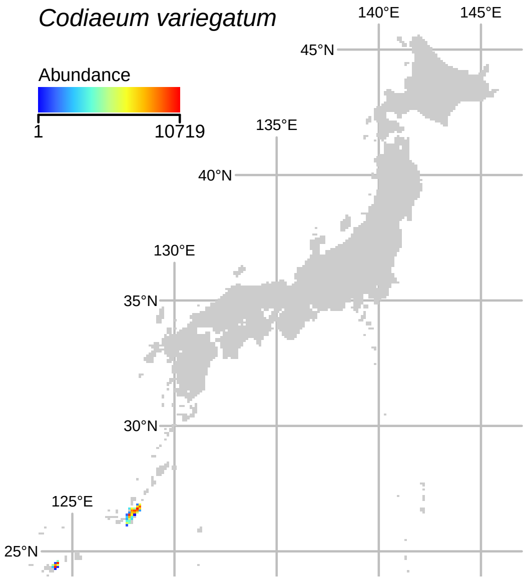
Codiaeum variegatum
Abundance
1
10719
140°E
145°E
45°N
135°E
40°N
130°E
35°N
30°N
125°E
25°N

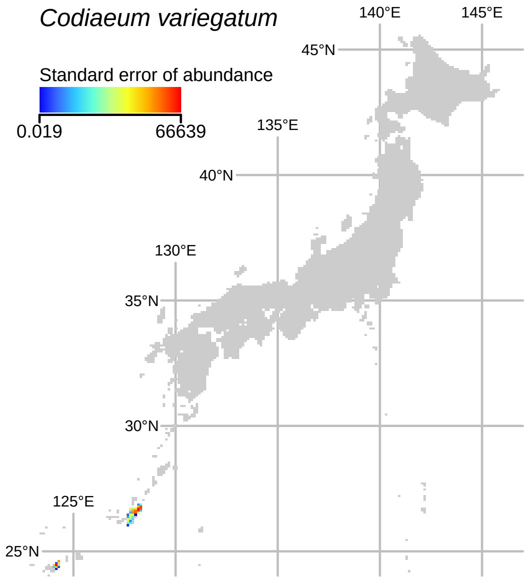
Codiaeum variegatum
Standard error of abundance
0.019
66639
140°E
145°E
45°N
135°E
40°N
130°E
35°N
30°N
125°E
25°N

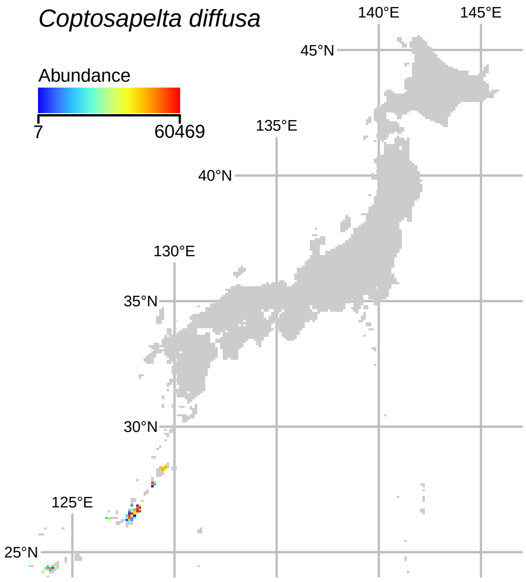
Coptosapelta diffusa
Abundance
7
60469
140°E
145°E
45°N
135°E
40°N
130°E
35°N
30°N
125°E
25°N

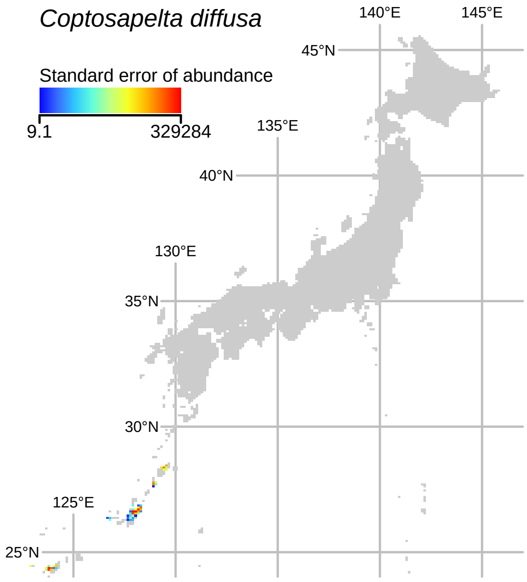
Coptosapelta diffusa
Standard error of abundance
9.1
329284
140°E
145°E
45°N
135°E
40°N
130°E
35°N
30°N
125°E
25°N

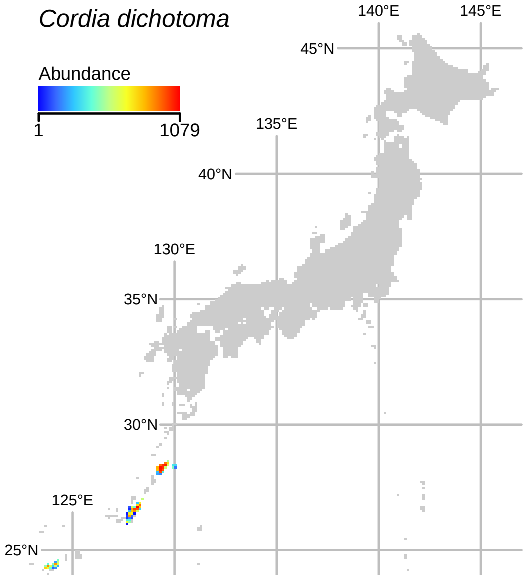
Cordia dichotoma
Abundance
1
1079
140°E
145°E
45°N
135°E
40°N
130°E
35°N
30°N
125°E
25°N

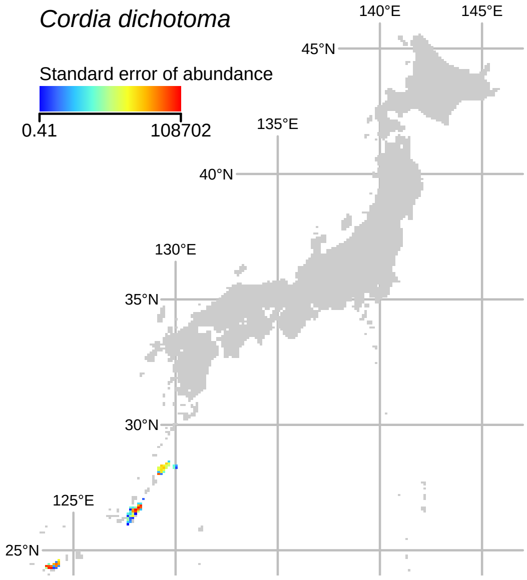
Cordia dichotoma
Standard error of abundance
0.41
108702
140°E
145°E
45°N
135°E
40°N
130°E
35°N
30°N
125°E
25°N

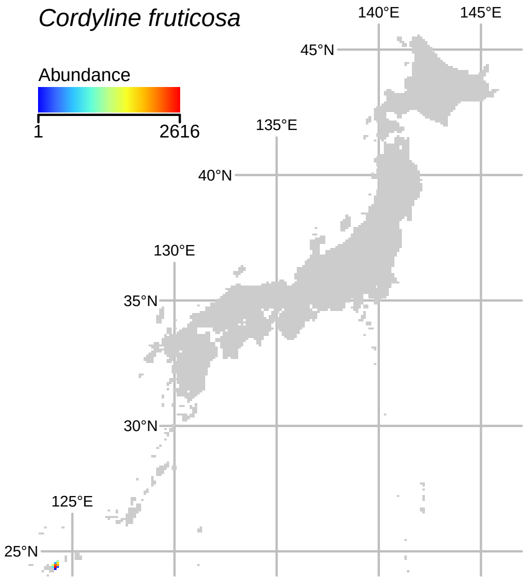
Cordyline fruticosa
Abundance
1
2616
140°E
145°E
45°N
135°E
40°N
130°E
35°N
30°N
125°E
25°N

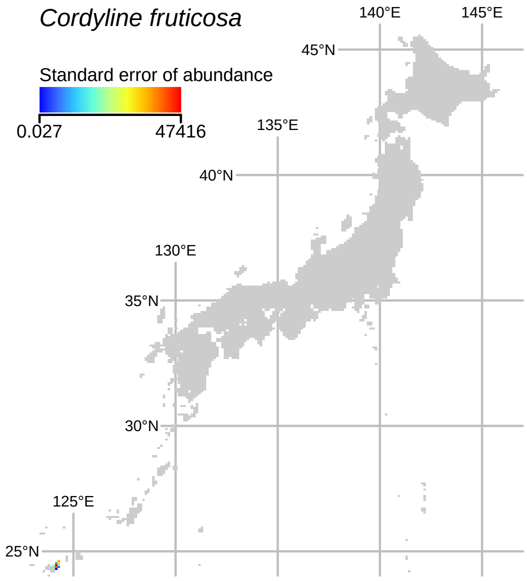
Cordyline fruticosa
Standard error of abundance
0.027
47416
140°E
145°E
45°N
135°E
40°N
130°E
35°N
30°N
125°E
25°N

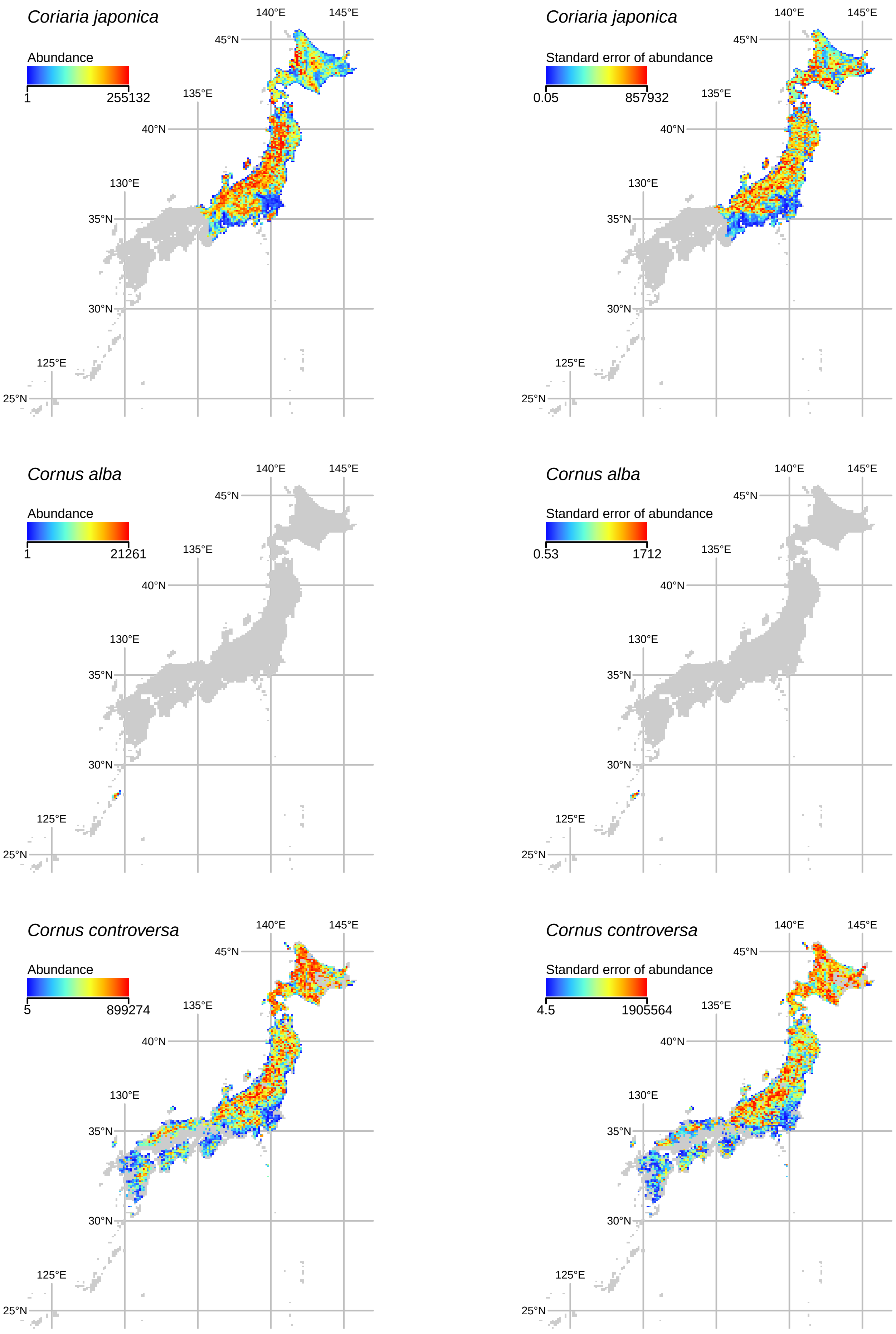

Coriaria japonica
Abundance
1
255132
Coriaria japonica
Standard error of abundance
0.05
857932
Cornus alba
Abundance
1
21261
Cornus alba
Standard error of abundance
0.53
1712
Cornus controversa
Abundance
5
899274
Cornus controversa
Standard error of abundance
4.5
1905564
125°E
130°E
135°E
140°E
145°E
25°N
30°N
35°N
40°N
45°N

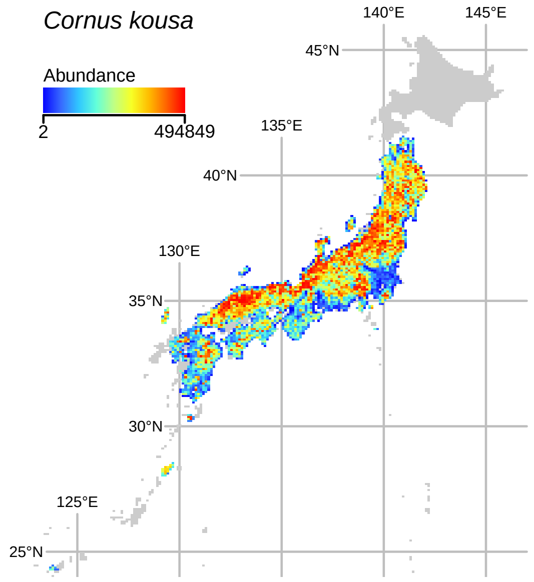
Cornus kousa
Abundance
2
494849
140°E
145°E
45°N
135°E
40°N
130°E
35°N
30°N
125°E
25°N

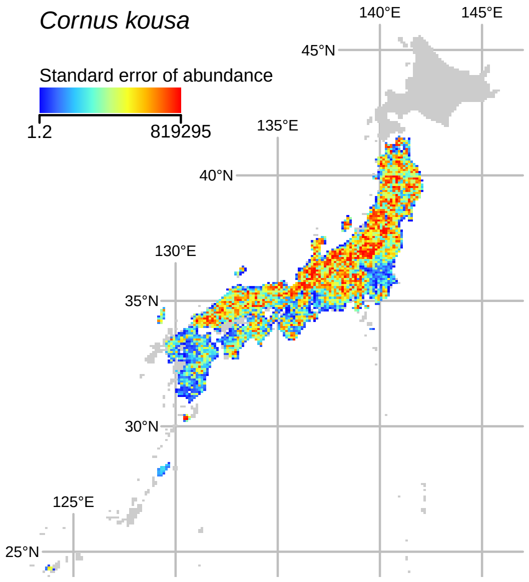
Cornus kousa
Standard error of abundance
1.2
819295
140°E
145°E
45°N
135°E
40°N
130°E
35°N
30°N
125°E
25°N

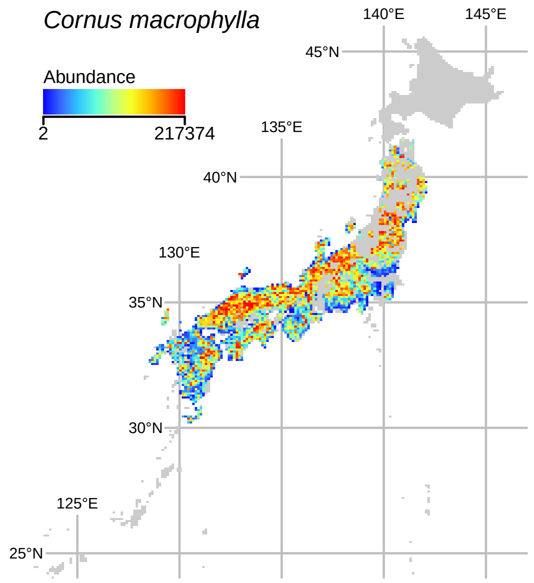
Cornus macrophylla
Abundance
2
217374
140°E
145°E
45°N
135°E
40°N
130°E
35°N
30°N
125°E
25°N

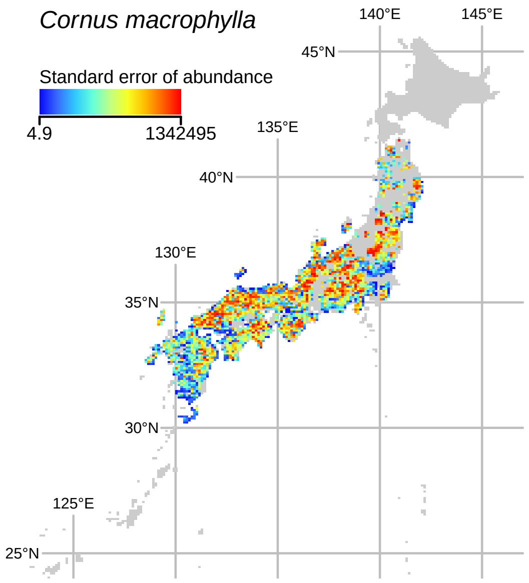
Cornus macrophylla
Standard error of abundance
4.9
1342495
140°E
145°E
45°N
135°E
40°N
130°E
35°N
30°N
125°E
25°N

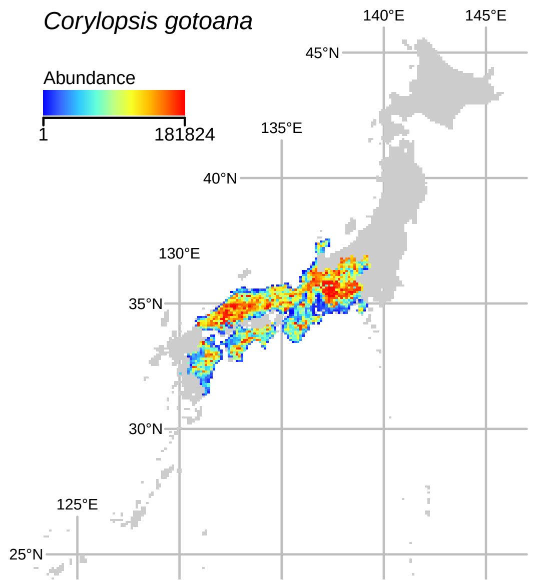
Corylopsis gotoana
Abundance
1
181824
140°E
145°E
45°N
135°E
40°N
130°E
35°N
30°N
125°E
25°N

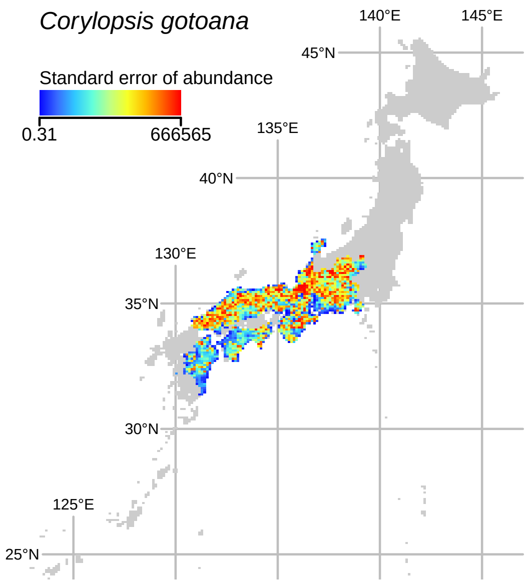
Corylopsis gotoana
Standard error of abundance
0.31
666565
140°E
145°E
45°N
135°E
40°N
130°E
35°N
30°N
125°E
25°N

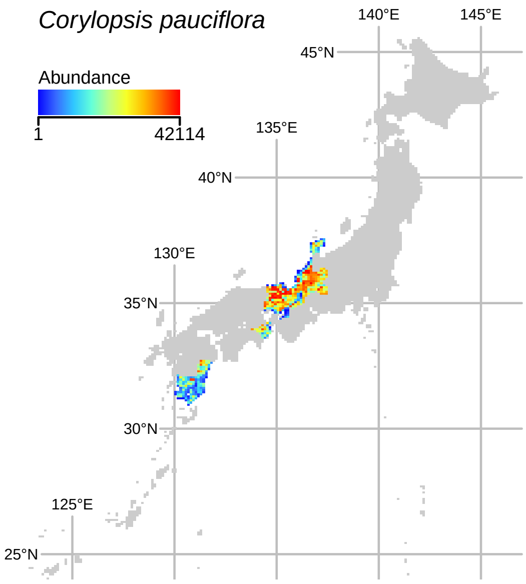
Corylopsis pauciflora
Abundance
1
42114
140°E
145°E
45°N
135°E
40°N
130°E
35°N
30°N
125°E
25°N

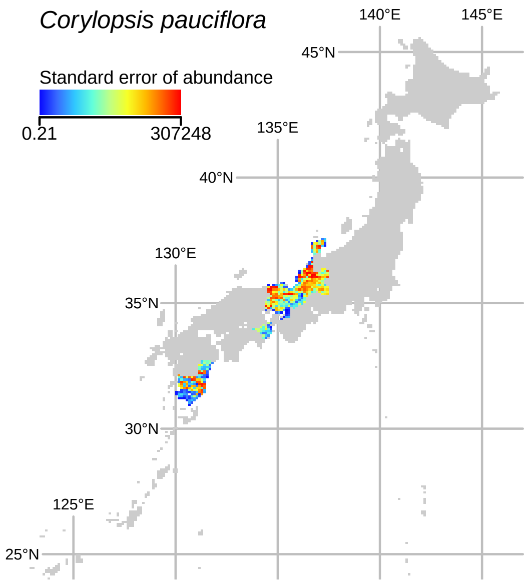
Corylopsis pauciflora
Standard error of abundance
0.21
307248
140°E
145°E
45°N
135°E
40°N
130°E
35°N
30°N
125°E
25°N

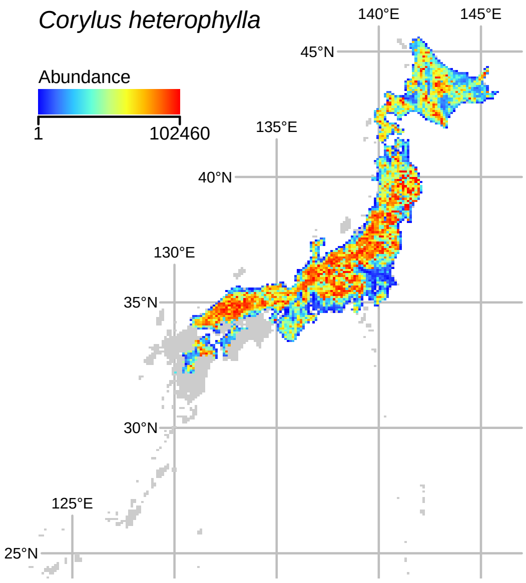
Corylus heterophylla
Abundance
1
102460
140°E
145°E
45°N
135°E
40°N
130°E
35°N
30°N
125°E
25°N

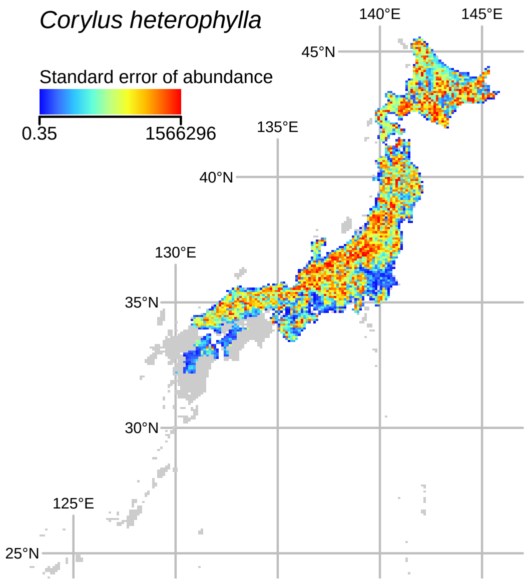
Corylus heterophylla
Standard error of abundance
0.35
1566296
140°E
145°E
45°N
135°E
40°N
130°E
35°N
30°N
125°E
25°N

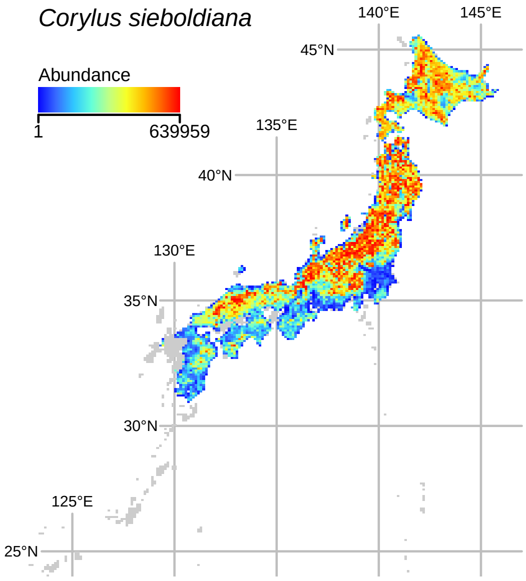
Corylus sieboldiana
Abundance
1
639959
140°E
145°E
45°N
135°E
40°N
130°E
35°N
30°N
125°E
25°N

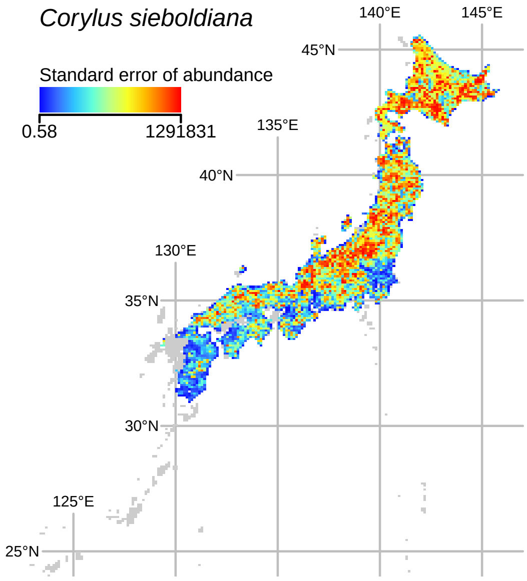
Corylus sieboldiana
Standard error of abundance
0.58
1291831
140°E
145°E
45°N
135°E
40°N
130°E
35°N
30°N
125°E
25°N

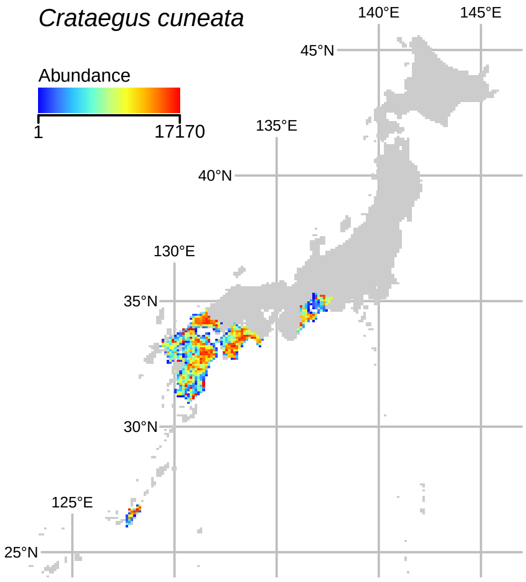
Crataegus cuneata
Abundance
1
17170
140°E
145°E
45°N
135°E
40°N
130°E
35°N
30°N
125°E
25°N

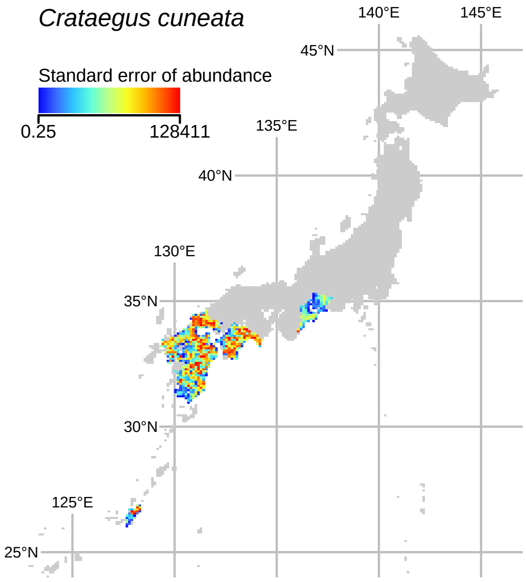
Crataegus cuneata
Standard error of abundance
0.25
128411
140°E
145°E
45°N
135°E
40°N
130°E
35°N
30°N
125°E
25°N

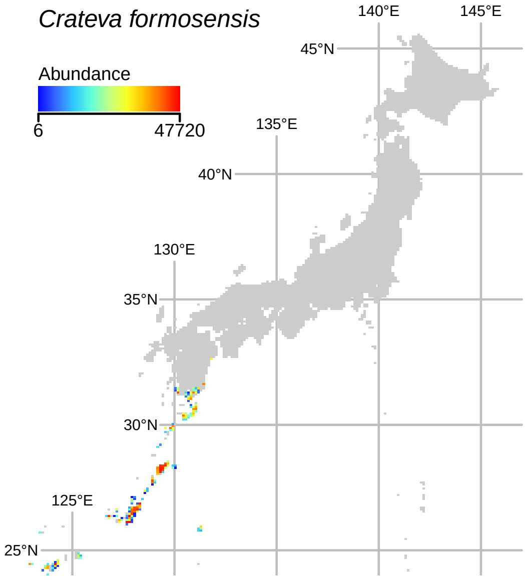
Crateva formosensis
Abundance
6
47720
140°E
145°E
45°N
135°E
40°N
130°E
35°N
30°N
125°E
25°N

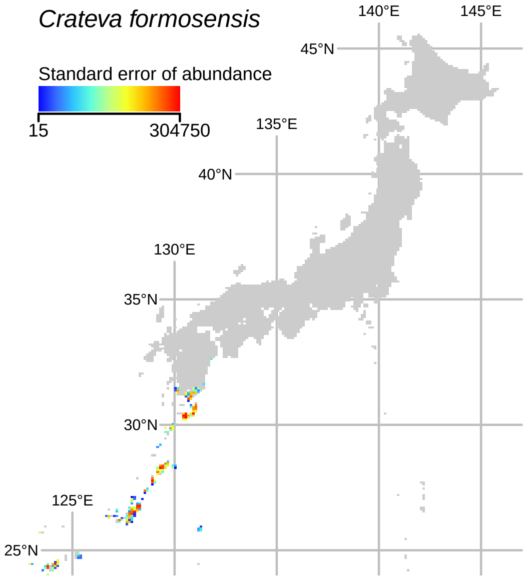
Crateva formosensis
Standard error of abundance
15
304750
140°E
145°E
45°N
135°E
40°N
130°E
35°N
30°N
125°E
25°N

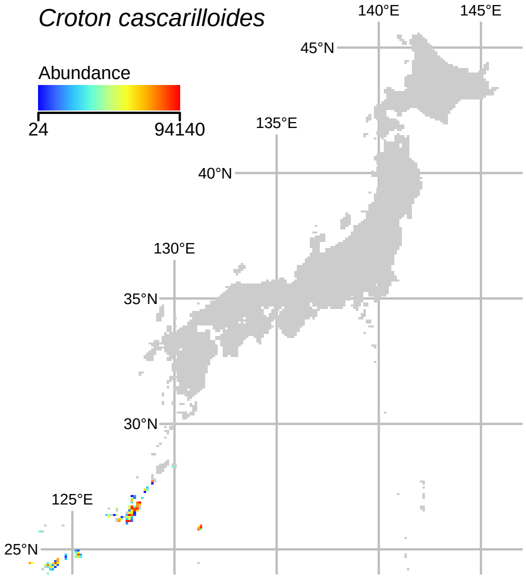
Croton cascarilloides
Abundance
24
94140
140°E
145°E
45°N
135°E
40°N
130°E
35°N
30°N
125°E
25°N

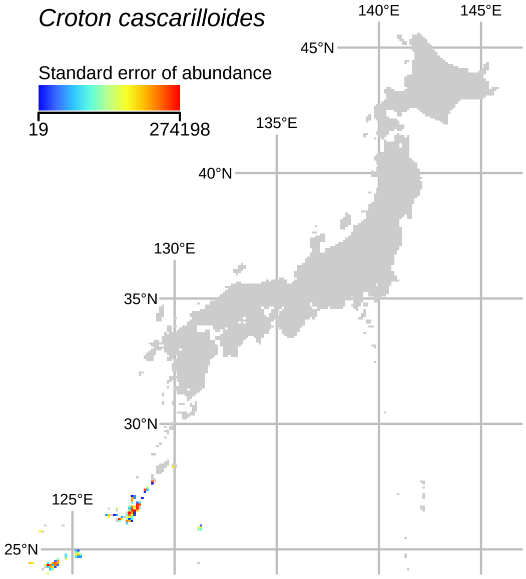
Croton cascarilloides
Standard error of abundance
19
274198
140°E
145°E
45°N
135°E
40°N
130°E
35°N
30°N
125°E
25°N

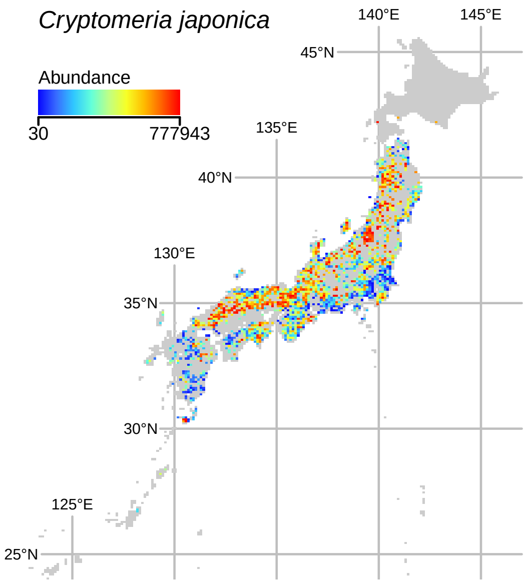
Cryptomeria japonica
Abundance
30
777943
140°E
145°E
45°N
135°E
40°N
130°E
35°N
30°N
125°E
25°N

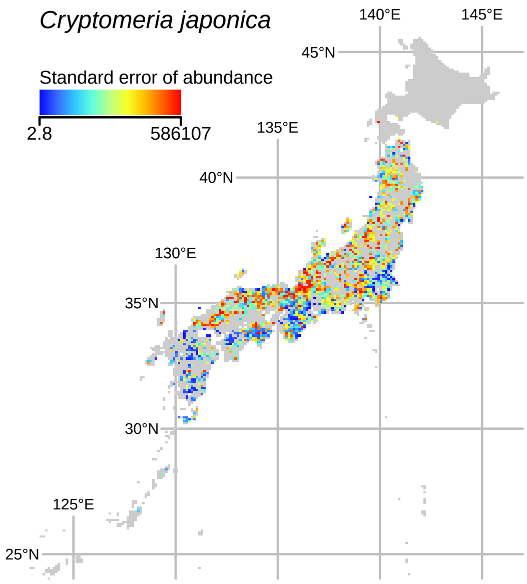
Cryptomeria japonica
Standard error of abundance
2.8
586107
140°E
145°E
45°N
135°E
40°N
130°E
35°N
30°N
125°E
25°N

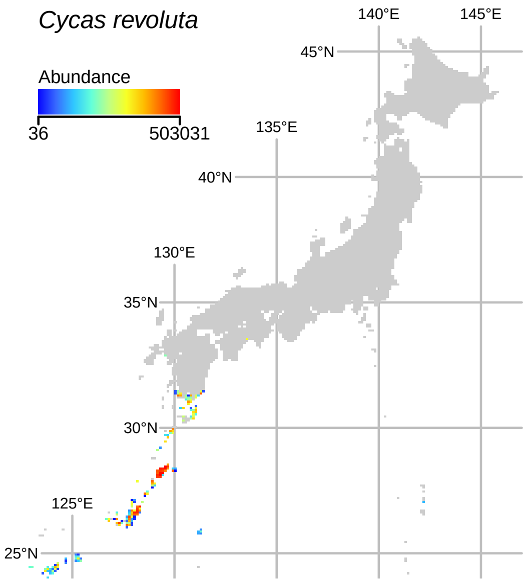
Cycas revoluta
Abundance
36
503031
140°E
145°E
45°N
135°E
40°N
130°E
35°N
30°N
125°E
25°N

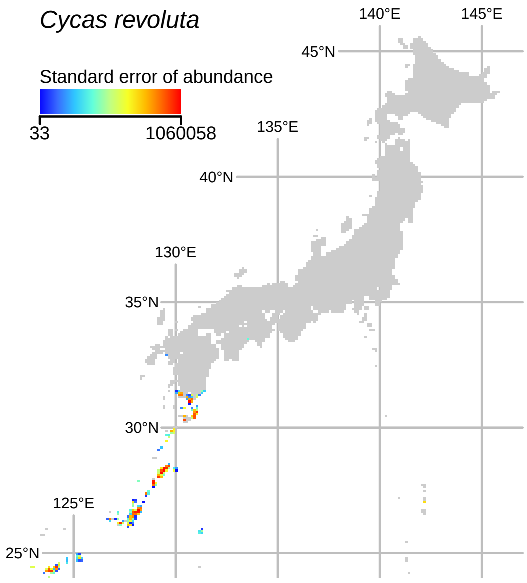
Cycas revoluta
Standard error of abundance
33
1060058
140°E
145°E
45°N
135°E
40°N
130°E
35°N
30°N
125°E
25°N

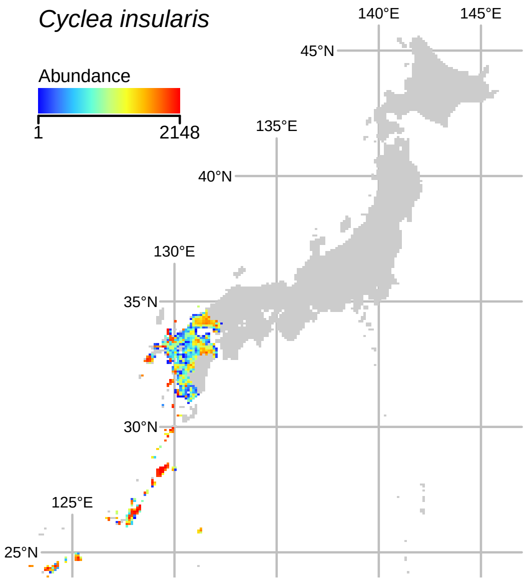
Cyclea insularis
Abundance
1
2148
140°E
145°E
45°N
135°E
40°N
130°E
35°N
30°N
125°E
25°N

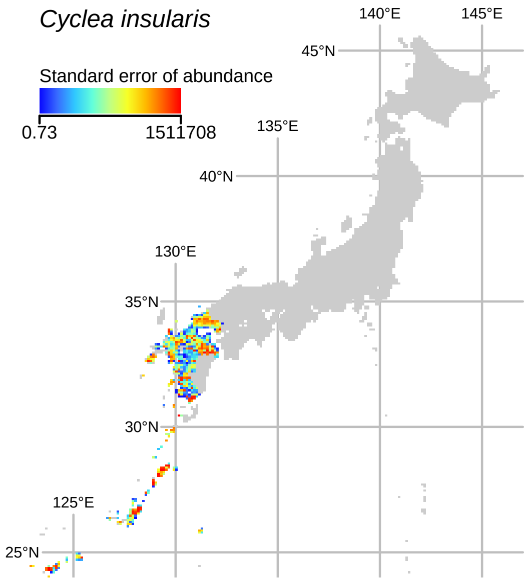
Cyclea insularis
Standard error of abundance
0.73
1511708
140°E
145°E
45°N
135°E
40°N
130°E
35°N
30°N
125°E
25°N

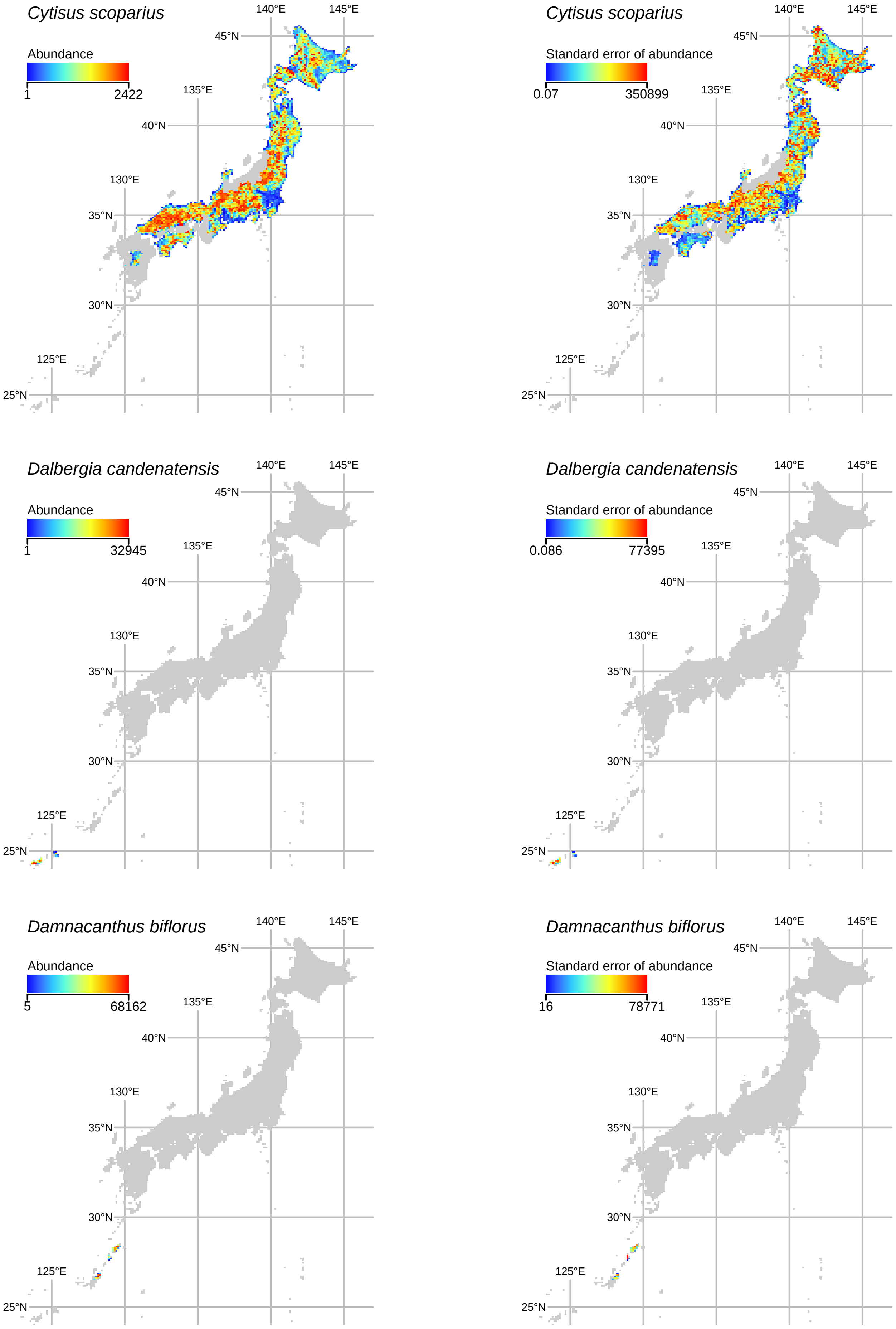

Cytisus scoparius
Abundance
1
2422
Cytisus scoparius
Standard error of abundance
0.07
350899
Dalbergia candenatensis
Abundance
1
32945
Dalbergia candenatensis
Standard error of abundance
0.086
77395
Damnacanthus biflorus
Abundance
5
68162
Damnacanthus biflorus
Standard error of abundance
16
78771
125°E
130°E
135°E
140°E
145°E
25°N
30°N
35°N
40°N
45°N

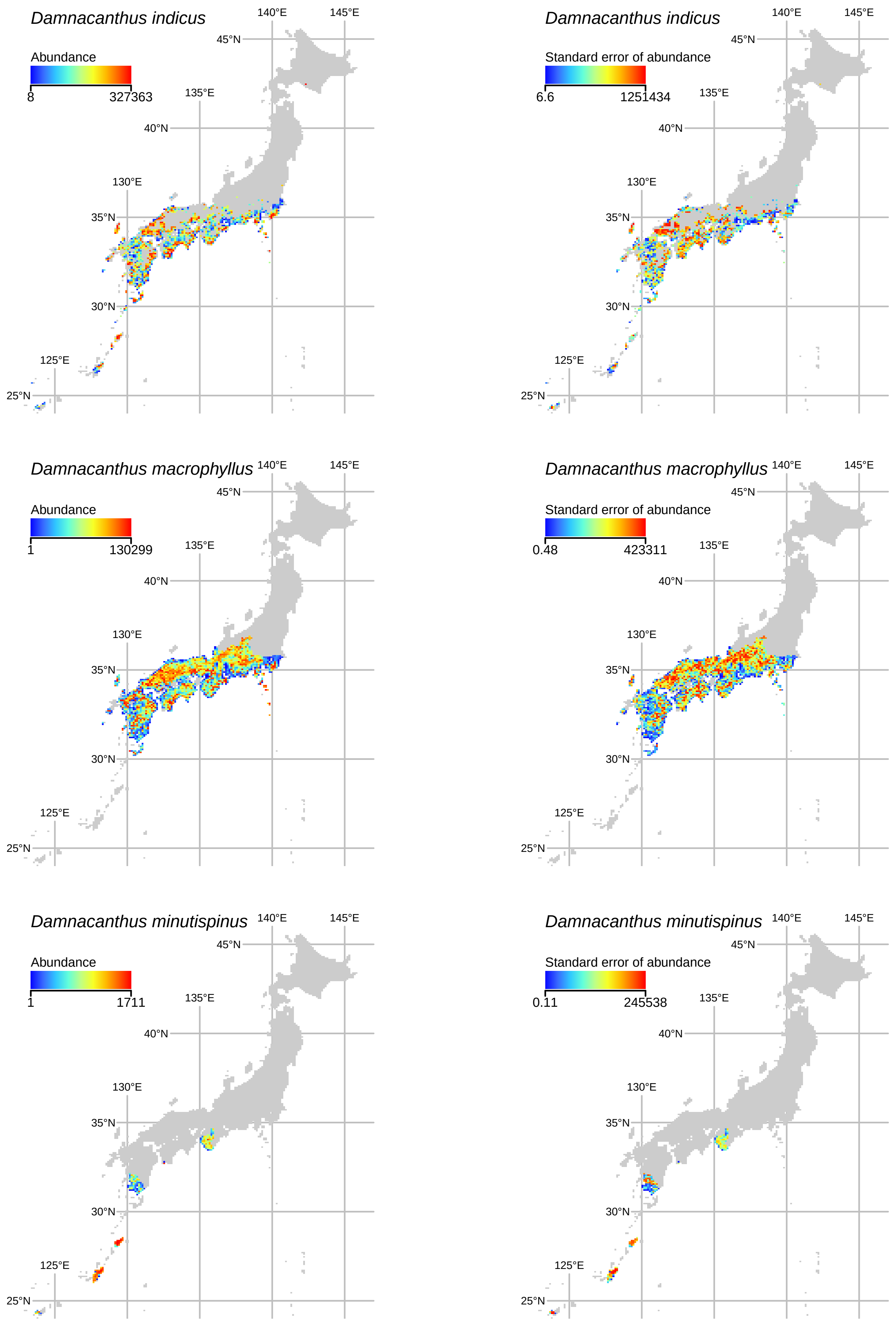

Damnacanthus indicus
Abundance
8
327363
Damnacanthus indicus
Standard error of abundance
6.6
1251434
Damnacanthus macrophyllus
Abundance
1
130299
Damnacanthus macrophyllus
Standard error of abundance
0.48
423311
Damnacanthus minutispinus
Abundance
1
1711
Damnacanthus minutispinus
Standard error of abundance
0.11
245538
140°E
145°E
45°N
135°E
40°N
130°E
35°N
30°N
125°E
25°N

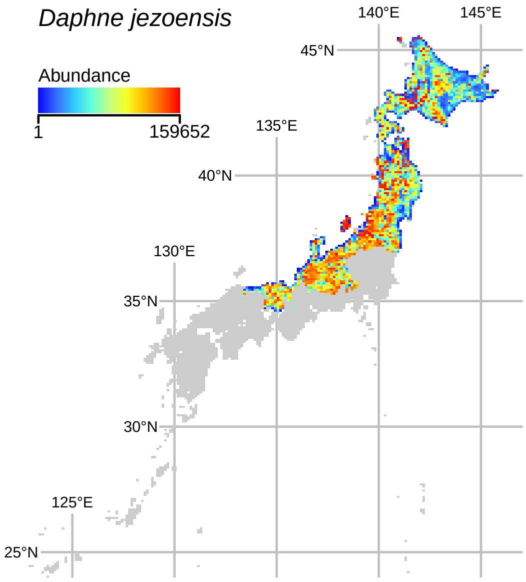
Daphne jezoensis
Abundance
1
159652
140°E
145°E
45°N
135°E
40°N
130°E
35°N
30°N
125°E
25°N

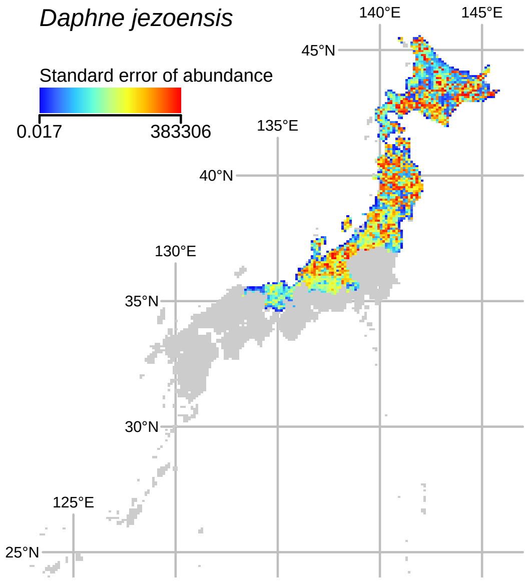
Daphne jezoensis
Standard error of abundance
0.017
383306
140°E
145°E
45°N
135°E
40°N
130°E
35°N
30°N
125°E
25°N

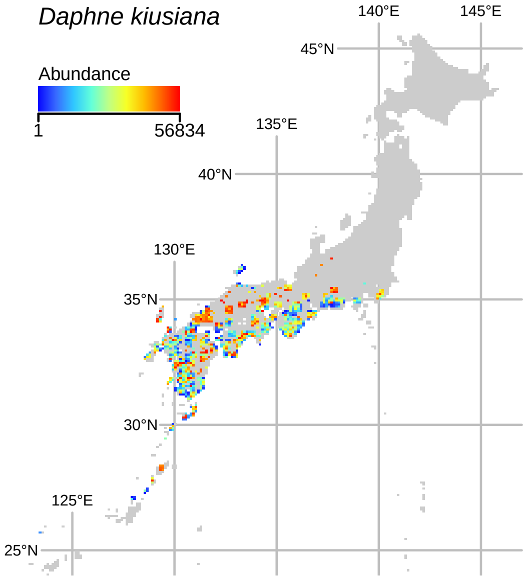
Daphne kiusiana
Abundance
1
56834
140°E
145°E
45°N
135°E
40°N
130°E
35°N
30°N
125°E
25°N

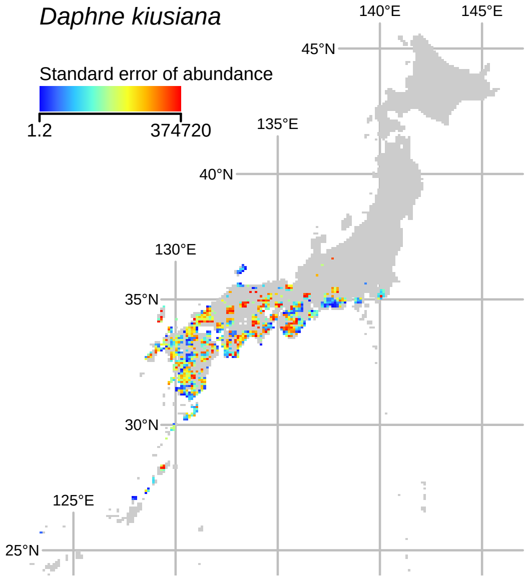
Daphne kiusiana
Standard error of abundance
1.2
374720
140°E
145°E
45°N
135°E
40°N
130°E
35°N
30°N
125°E
25°N

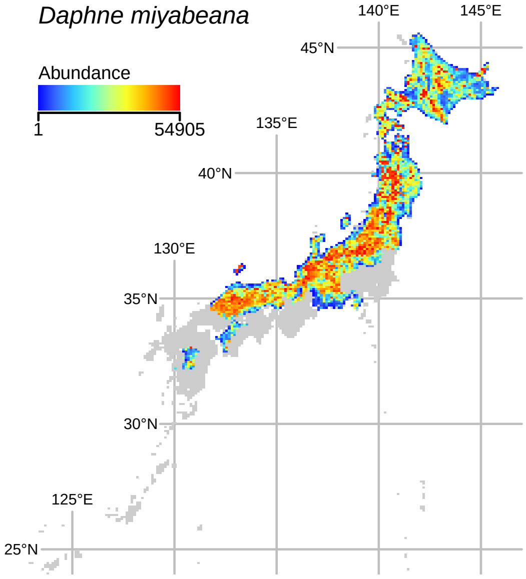
Daphne miyabeana
Abundance
1
54905
140°E
145°E
45°N
135°E
40°N
130°E
35°N
30°N
125°E
25°N

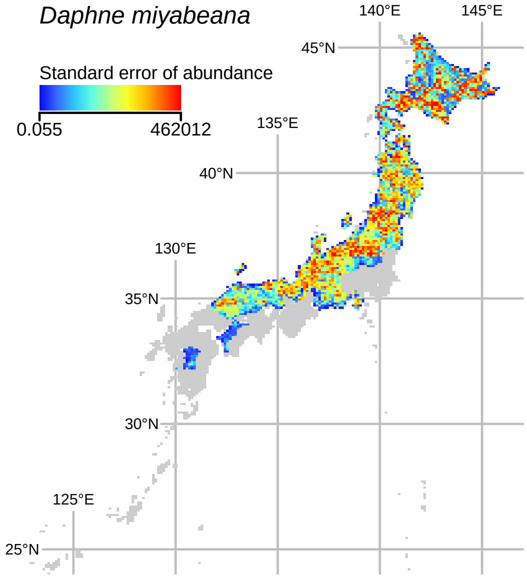
Daphne miyabeana
Standard error of abundance
0.055
462012
140°E
145°E
45°N
135°E
40°N
130°E
35°N
30°N
125°E
25°N

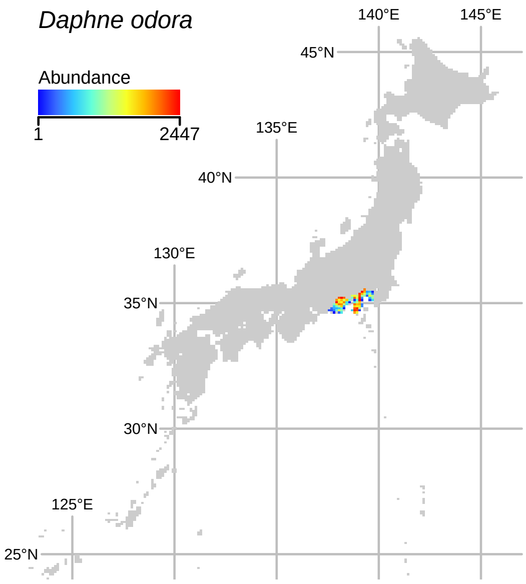
Daphne odora
Abundance
1
2447
140°E
145°E
45°N
135°E
40°N
130°E
35°N
30°N
125°E
25°N

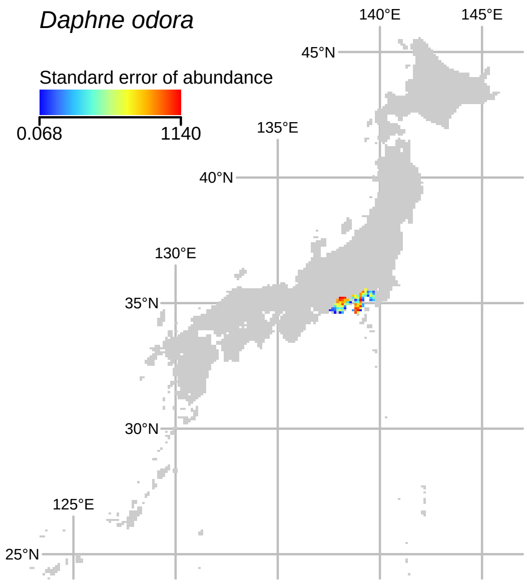
Daphne odora
Standard error of abundance
0.068
1140
140°E
145°E
45°N
135°E
40°N
130°E
35°N
30°N
125°E
25°N

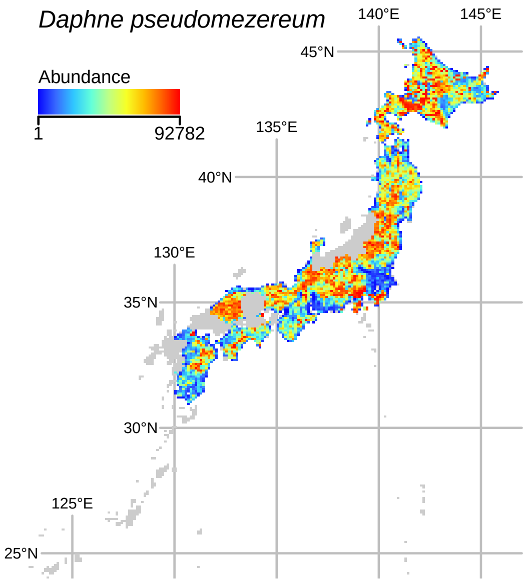
Daphne pseudomezereum
Abundance
1
92782
140°E
145°E
45°N
135°E
40°N
130°E
35°N
30°N
125°E
25°N

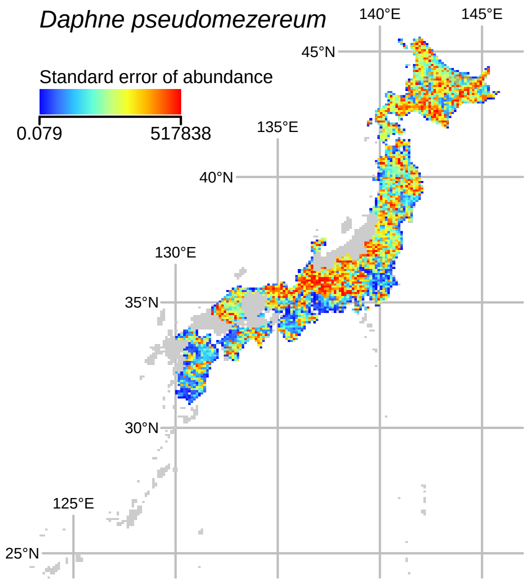
Daphne pseudomezereum
Standard error of abundance
0.079
517838
140°E
145°E
45°N
135°E
40°N
130°E
35°N
30°N
125°E
25°N

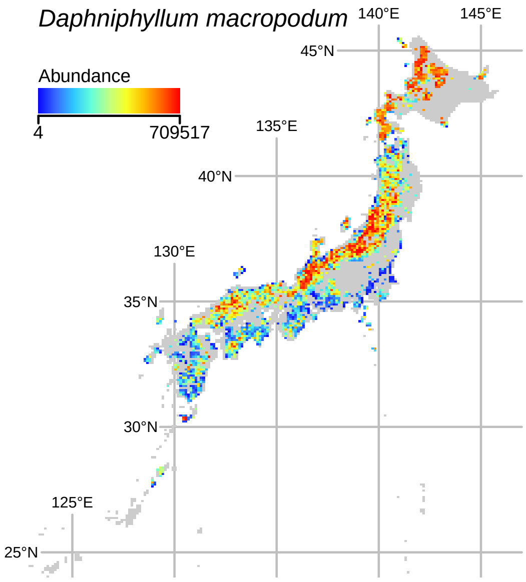
Daphniphyllum macropodum
Abundance
4
709517
140°E
145°E
45°N
135°E
40°N
130°E
35°N
30°N
125°E
25°N

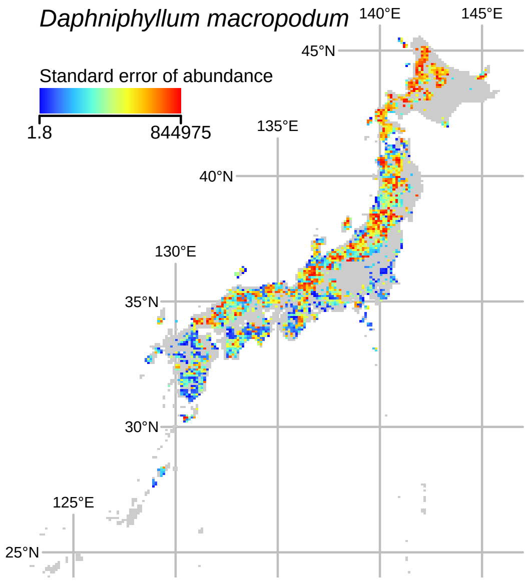
Daphniphyllum macropodum
Standard error of abundance
1.8
844975
140°E
145°E
45°N
135°E
40°N
130°E
35°N
30°N
125°E
25°N

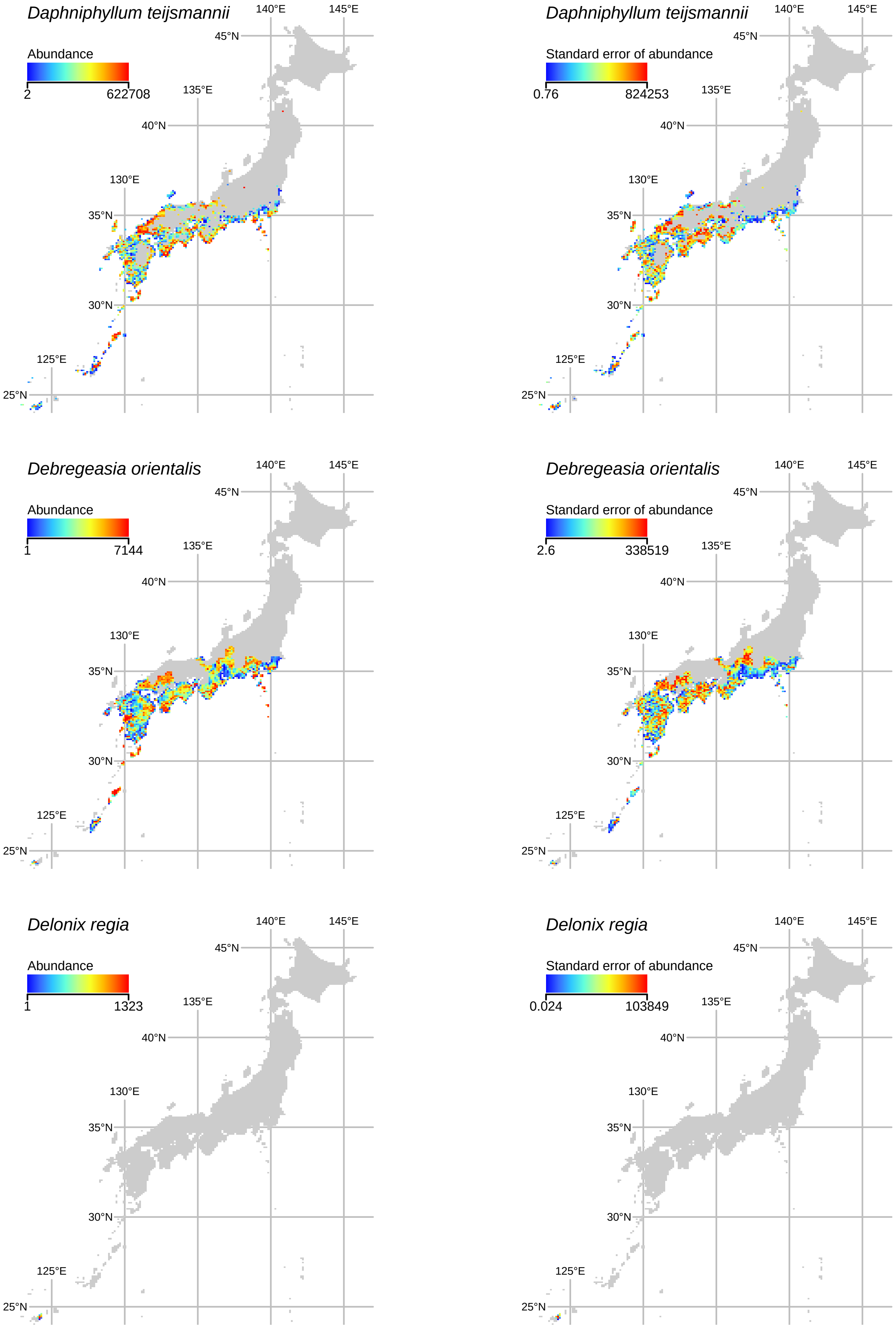
Daphniphyllum teijsmannii
Abundance
2
622708
Daphniphyllum teijsmannii
Standard error of abundance
0.76
824253
Debregeasia orientalis
Abundance
1
7144
Debregeasia orientalis
Standard error of abundance
2.6
338519
Delonix regia
Abundance
1
1323
Delonix regia
Standard error of abundance
0.024
103849
140°E
145°E
45°N
135°E
40°N
130°E
35°N
30°N
125°E
25°N

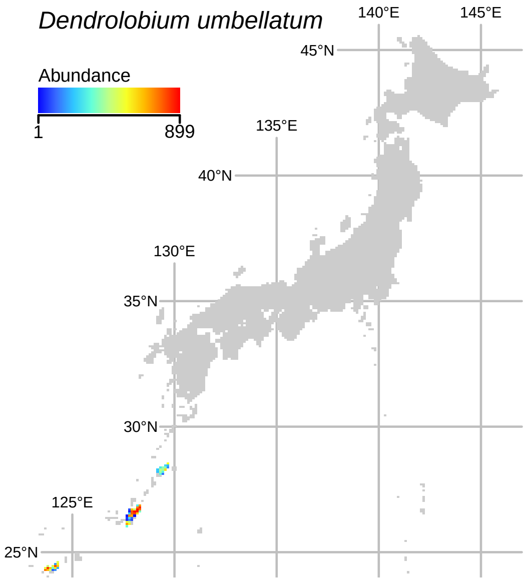
Dendrolobium umbellatum
Abundance
1
899
140°E
145°E
45°N
135°E
40°N
130°E
35°N
30°N
125°E
25°N

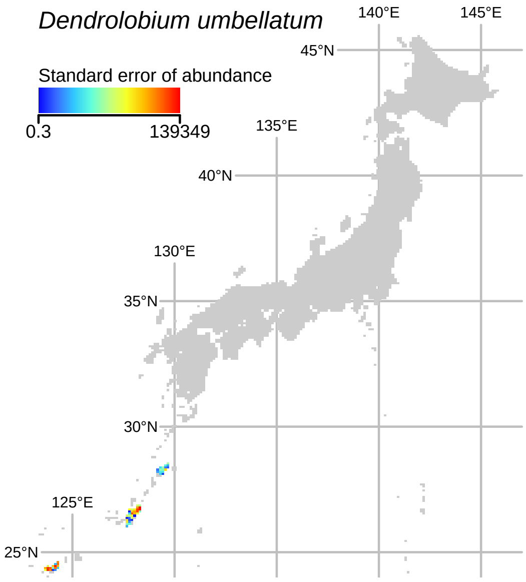
Dendrolobium umbellatum
Standard error of abundance
0.3
139349
140°E
145°E
45°N
135°E
40°N
130°E
35°N
30°N
125°E
25°N

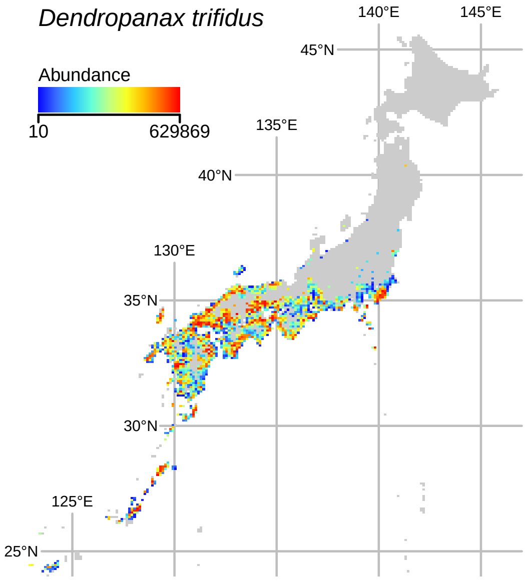
Dendropanax trifidus
Abundance
10
629869
140°E
145°E
45°N
135°E
40°N
130°E
35°N
30°N
125°E
25°N

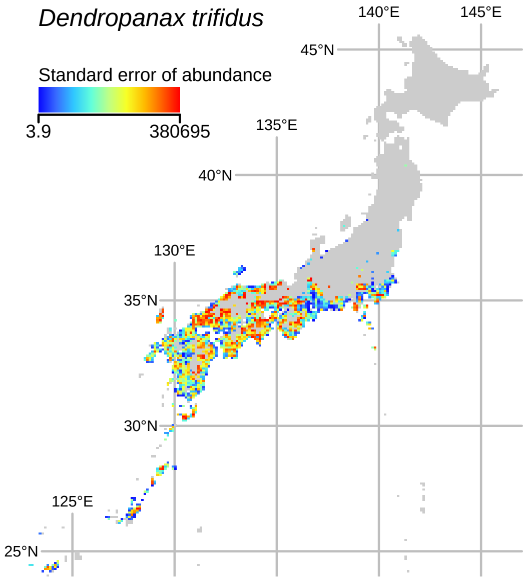
Dendropanax trifidus
Standard error of abundance
3.9
380695
140°E
145°E
45°N
135°E
40°N
130°E
35°N
30°N
125°E
25°N

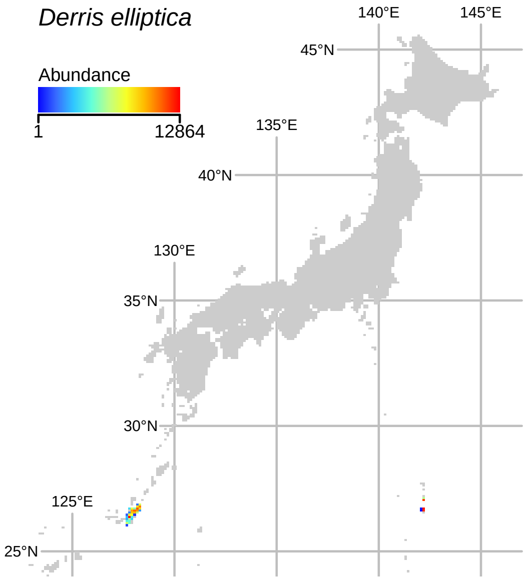
Derris elliptica
Abundance
1
12864
140°E
145°E
45°N
135°E
40°N
130°E
35°N
30°N
125°E
25°N

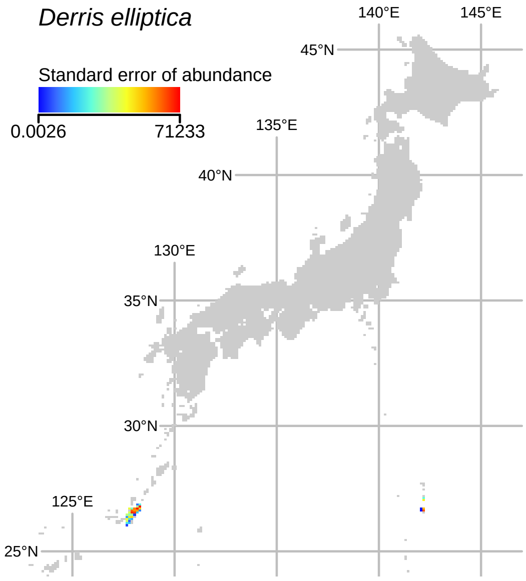
Derris elliptica
Standard error of abundance
0.0026
71233
140°E
145°E
45°N
135°E
40°N
130°E
35°N
30°N
125°E
25°N

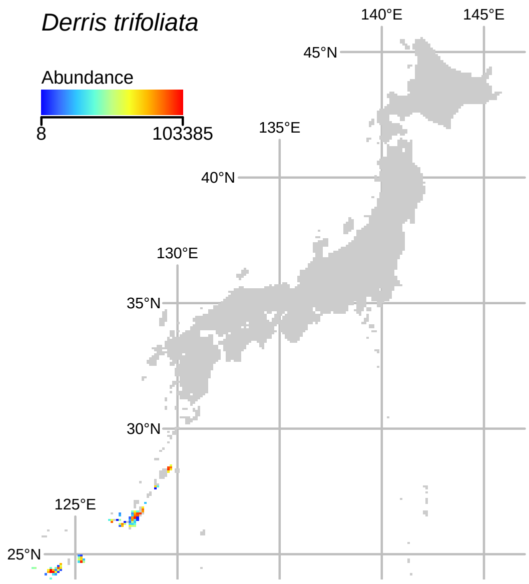
Derris trifoliata
Abundance
8
103385
140°E
145°E
45°N
135°E
40°N
130°E
35°N
30°N
125°E
25°N

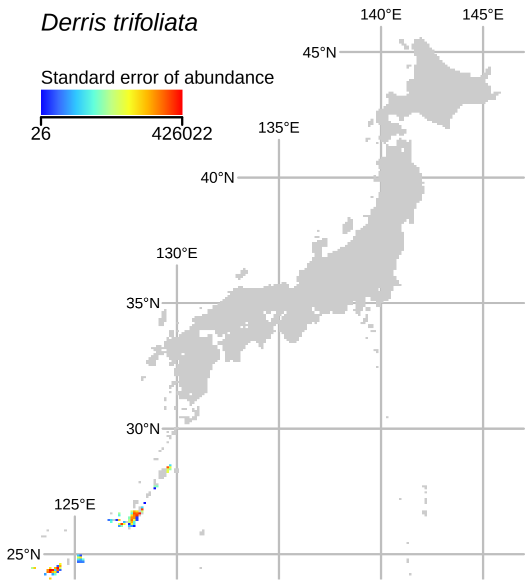
Derris trifoliata
Standard error of abundance
26
426022
140°E
145°E
45°N
135°E
40°N
130°E
35°N
30°N
125°E
25°N

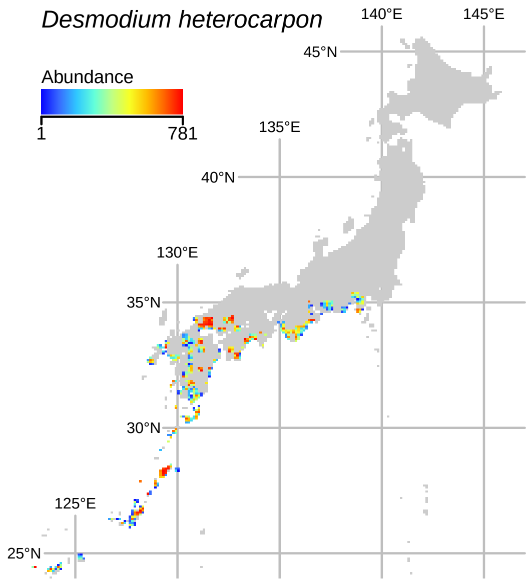
Desmodium heterocarpon
Abundance
1
781
140°E
145°E
45°N
135°E
40°N
130°E
35°N
30°N
125°E
25°N

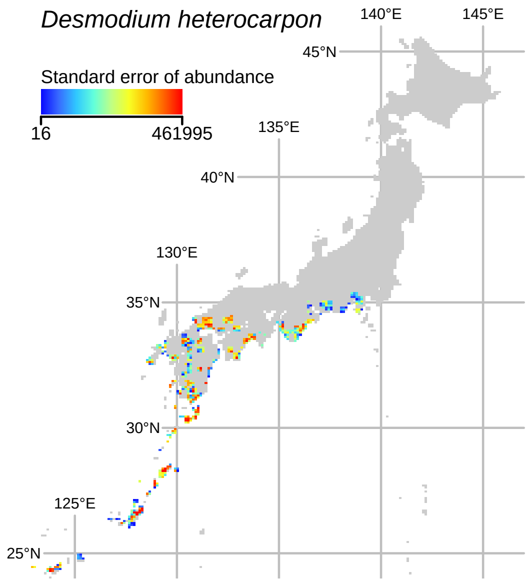
Desmodium heterocarpon
Standard error of abundance
16
461995
140°E
145°E
45°N
135°E
40°N
130°E
35°N
30°N
125°E
25°N

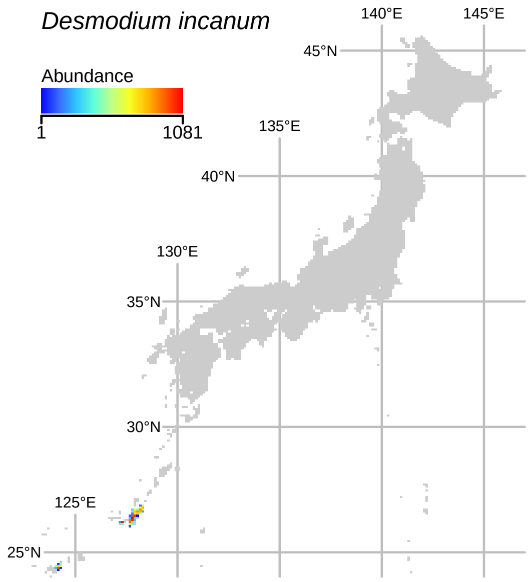
Desmodium incanum
Abundance
1
1081
140°E
145°E
45°N
135°E
40°N
130°E
35°N
30°N
125°E
25°N

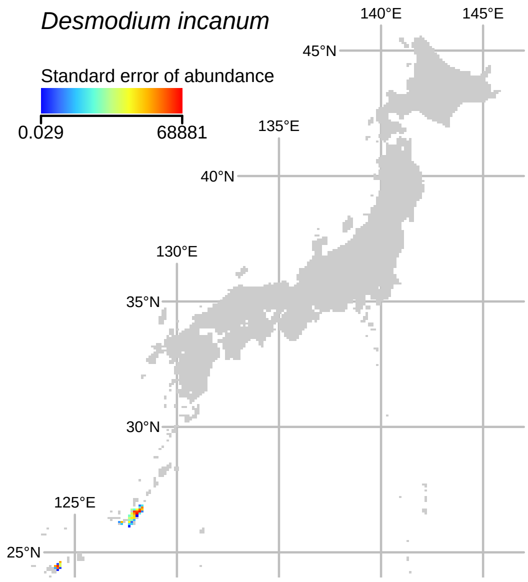
Desmodium incanum
Standard error of abundance
0.029
68881
140°E
145°E
45°N
135°E
40°N
130°E
35°N
30°N
125°E
25°N

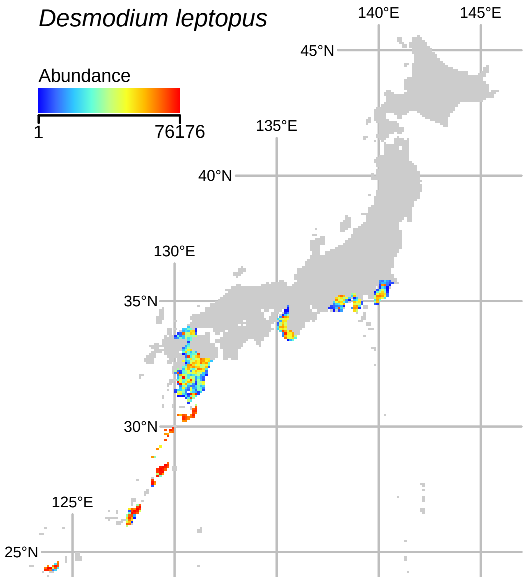
Desmodium leptopus
Abundance
1
76176
140°E
145°E
45°N
135°E
40°N
130°E
35°N
30°N
125°E
25°N

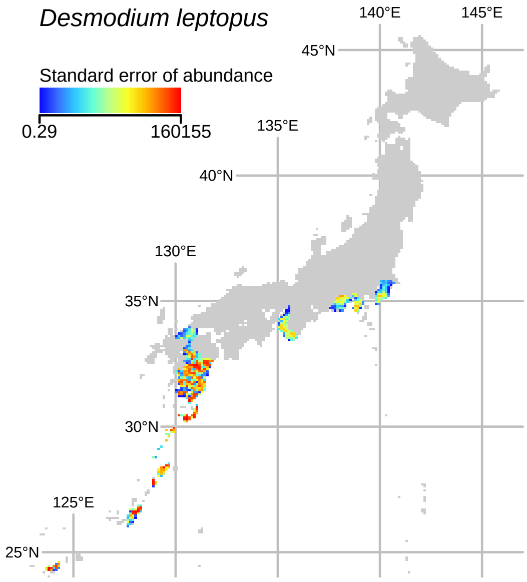
Desmodium leptopus
Standard error of abundance
0.29
160155
140°E
145°E
45°N
135°E
40°N
130°E
35°N
30°N
125°E
25°N

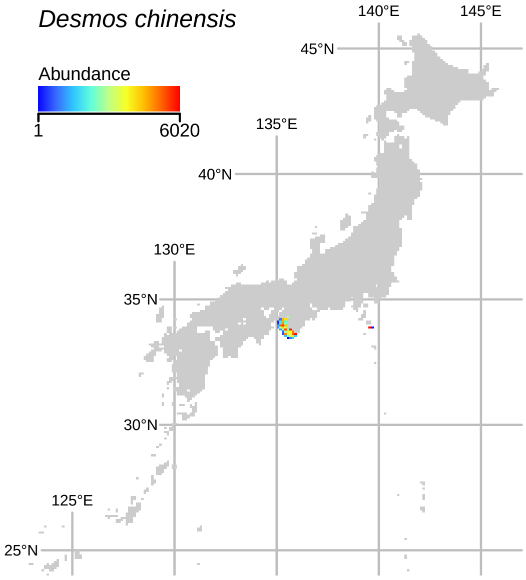
Desmos chinensis
Abundance
1
6020
140°E
145°E
45°N
135°E
40°N
130°E
35°N
30°N
125°E
25°N

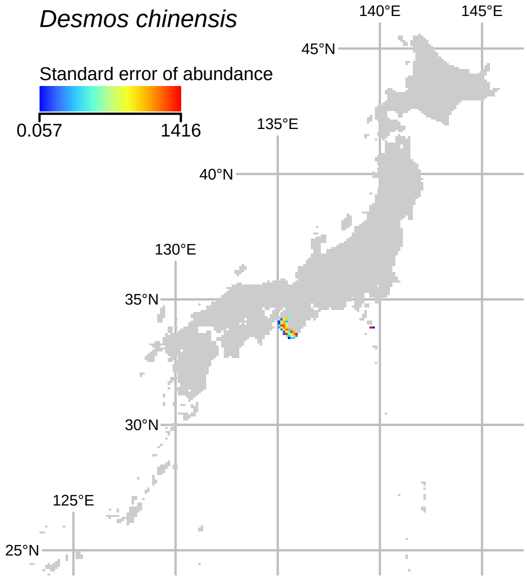
Desmos chinensis
Standard error of abundance
0.057
1416
140°E
145°E
45°N
135°E
40°N
130°E
35°N
30°N
125°E
25°N

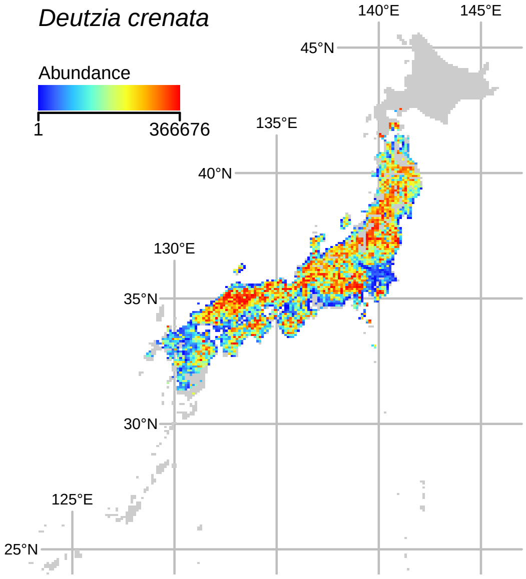
Deutzia crenata
Abundance
1
366676
140°E
145°E
45°N
135°E
40°N
130°E
35°N
30°N
125°E
25°N

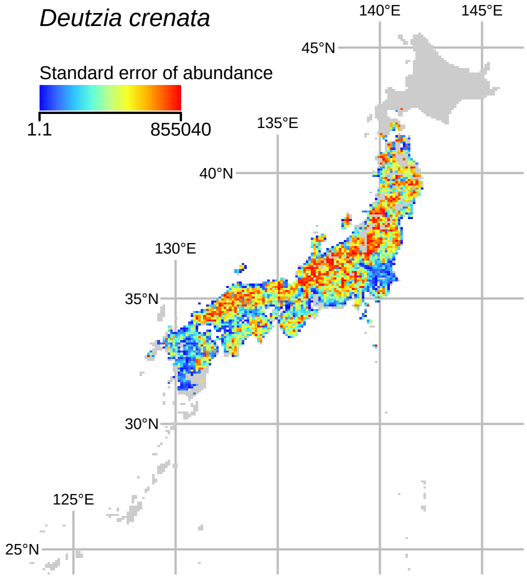
Deutzia crenata
Standard error of abundance
1.1
855040
140°E
145°E
45°N
135°E
40°N
130°E
35°N
30°N
125°E
25°N

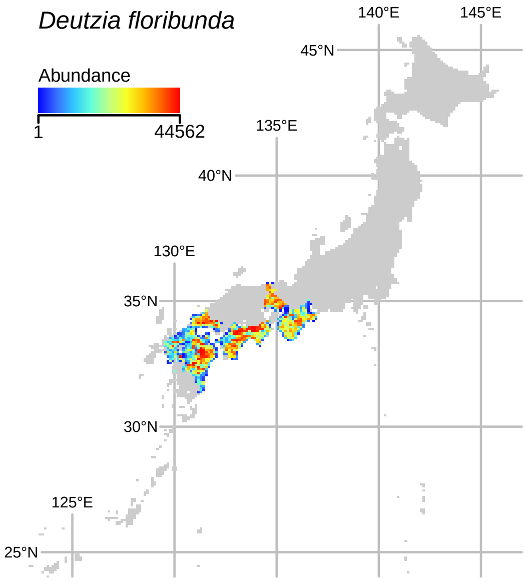
Deutzia floribunda
Abundance
1
44562
140°E
145°E
45°N
135°E
40°N
130°E
35°N
30°N
125°E
25°N

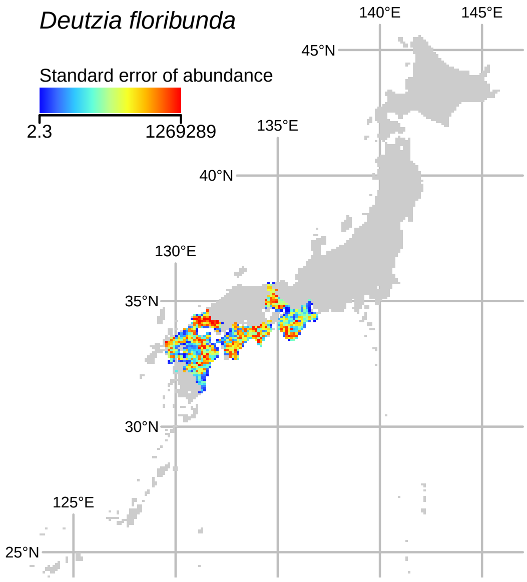
Deutzia floribunda
Standard error of abundance
2.3
1269289
140°E
145°E
45°N
135°E
40°N
130°E
35°N
30°N
125°E
25°N

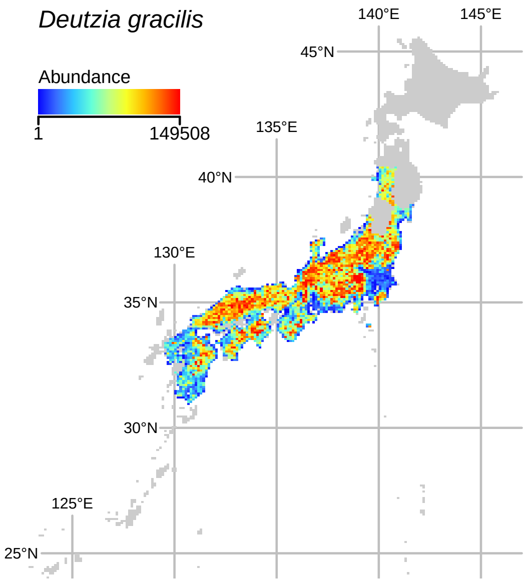
Deutzia gracilis
Abundance
1
149508
140°E
145°E
45°N
135°E
40°N
130°E
35°N
30°N
125°E
25°N

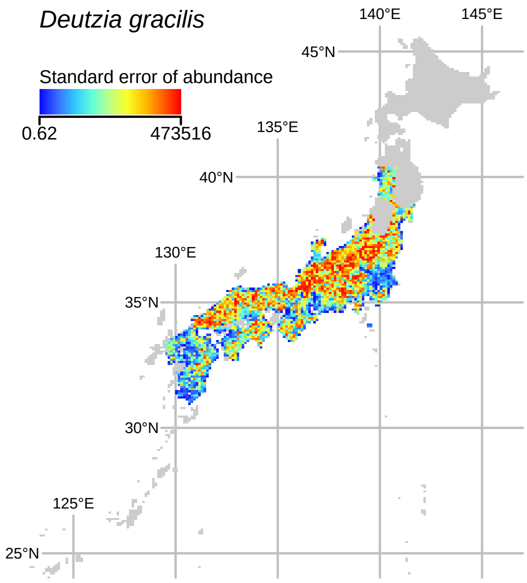
Deutzia gracilis
Standard error of abundance
0.62
473516
140°E
145°E
45°N
135°E
40°N
130°E
35°N
30°N
125°E
25°N

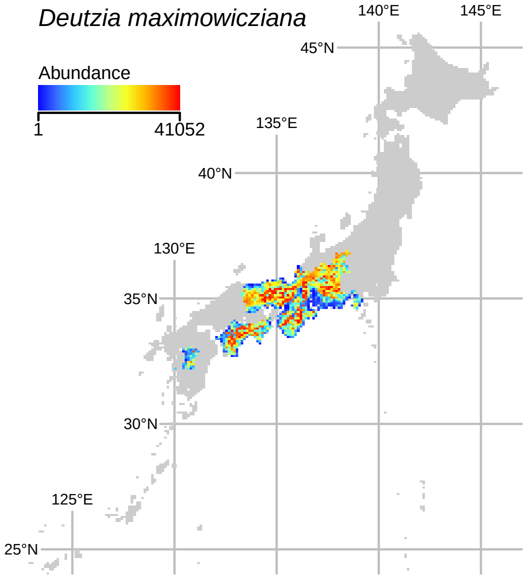
Deutzia maximowicziana
Abundance
1
41052
140°E
145°E
45°N
135°E
40°N
130°E
35°N
30°N
125°E
25°N

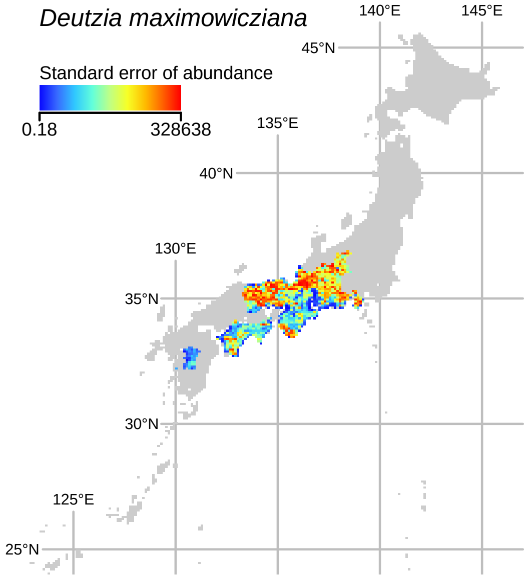
Deutzia maximowicziana
Standard error of abundance
0.18
328638
140°E
145°E
45°N
135°E
40°N
130°E
35°N
30°N
125°E
25°N

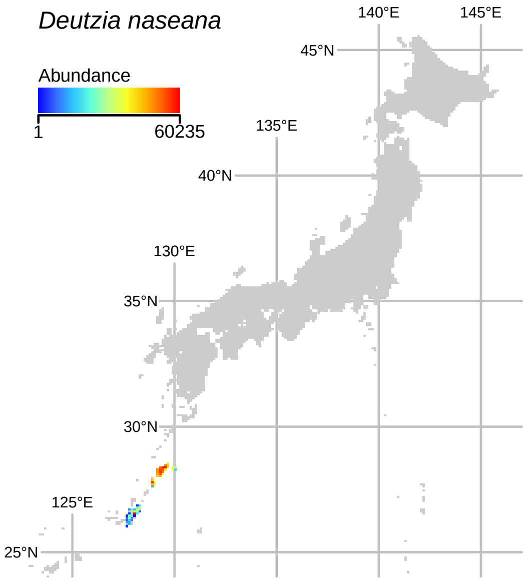
Deutzia naseana
Abundance
1
60235
140°E
145°E
45°N
135°E
40°N
130°E
35°N
30°N
125°E
25°N

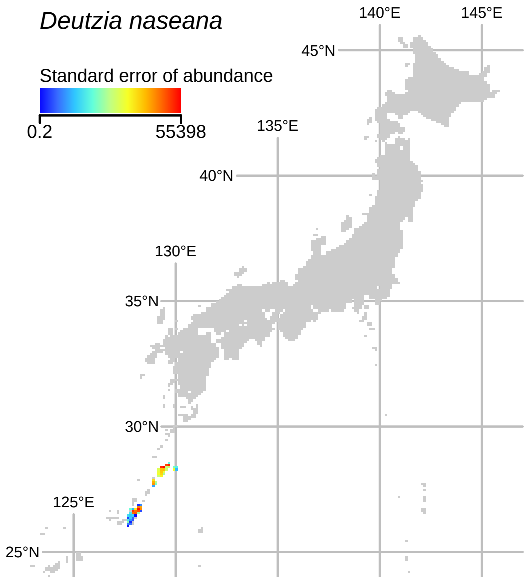
Deutzia naseana
Standard error of abundance
0.2
55398
140°E
145°E
45°N
135°E
40°N
130°E
35°N
30°N
125°E
25°N

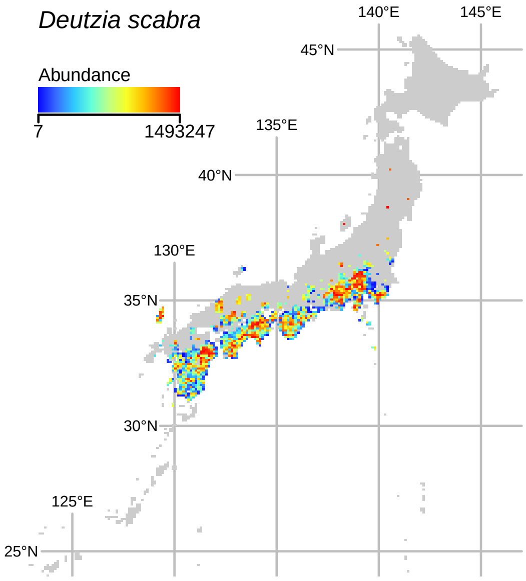
Deutzia scabra
Abundance
7
1493247
140°E
145°E
45°N
135°E
40°N
130°E
35°N
30°N
125°E
25°N

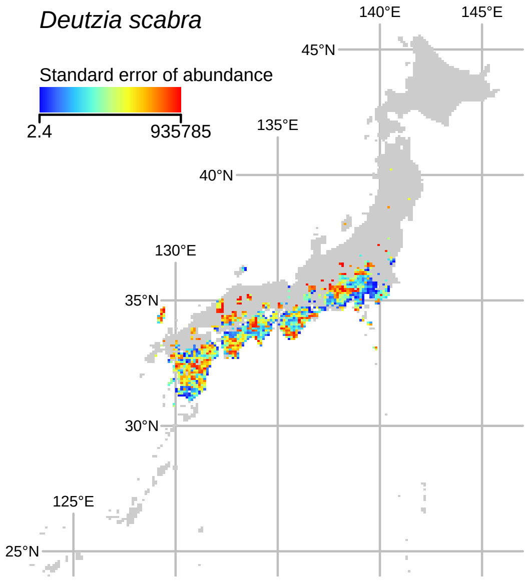
Deutzia scabra
Standard error of abundance
2.4
935785
140°E
145°E
45°N
135°E
40°N
130°E
35°N
30°N
125°E
25°N

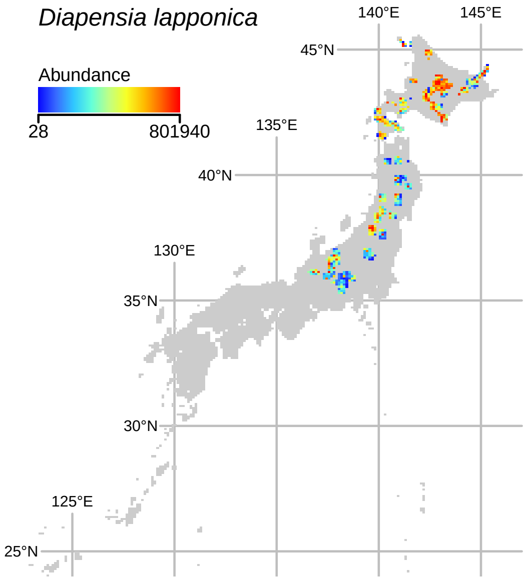
Diapensia lapponica
Abundance
28
801940
140°E
145°E
45°N
135°E
40°N
130°E
35°N
30°N
125°E
25°N

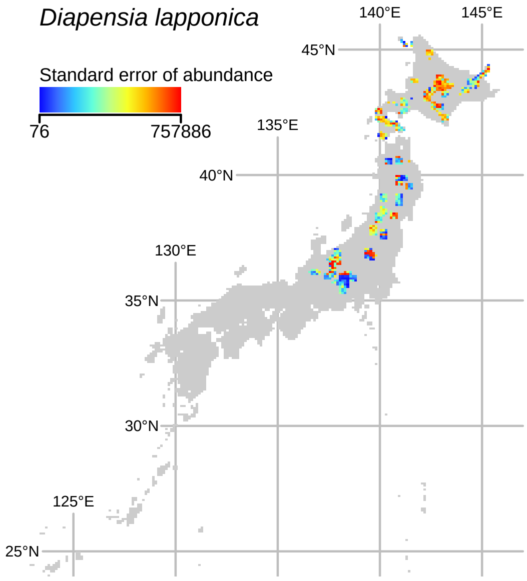
Diapensia lapponica
Standard error of abundance
76
757886
140°E
145°E
45°N
135°E
40°N
130°E
35°N
30°N
125°E
25°N

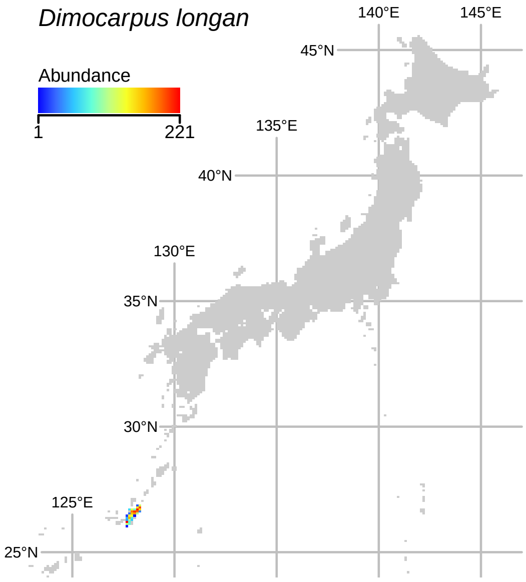
Dimocarpus longan
Abundance
1
221
140°E
145°E
45°N
135°E
40°N
130°E
35°N
30°N
125°E
25°N

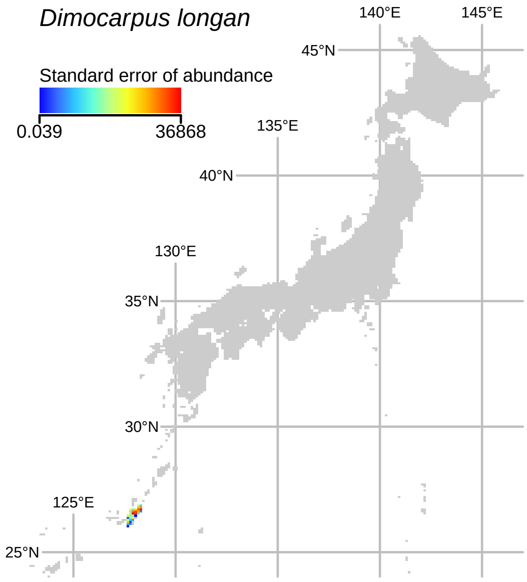
Dimocarpus longan
Standard error of abundance
0.039
36868
140°E
145°E
45°N
135°E
40°N
130°E
35°N
30°N
125°E
25°N

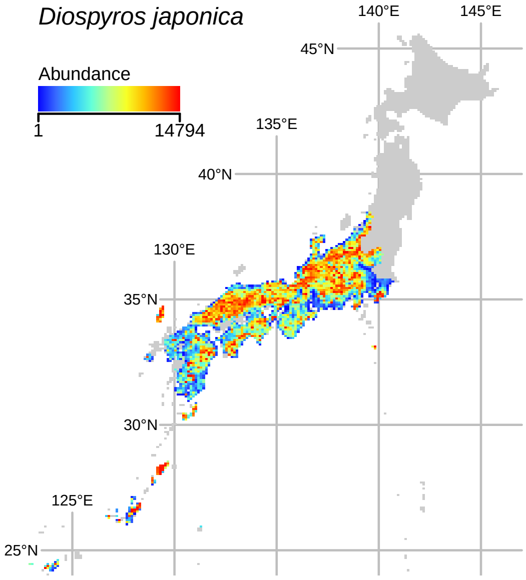
Diospyros japonica
Abundance
1
14794
140°E
145°E
45°N
135°E
40°N
130°E
35°N
30°N
125°E
25°N

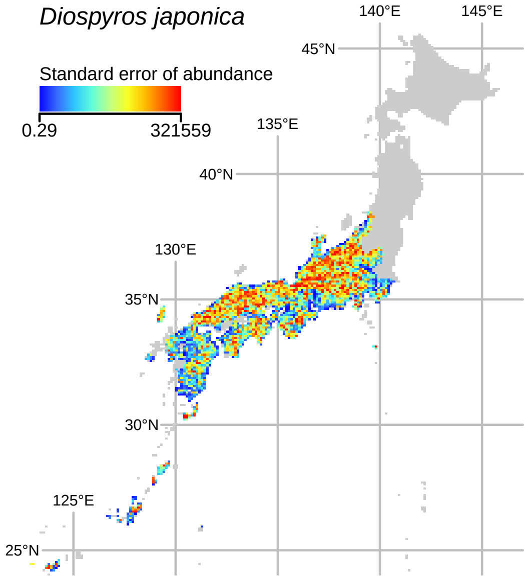
Diospyros japonica
Standard error of abundance
0.29
321559
140°E
145°E
45°N
135°E
40°N
130°E
35°N
30°N
125°E
25°N

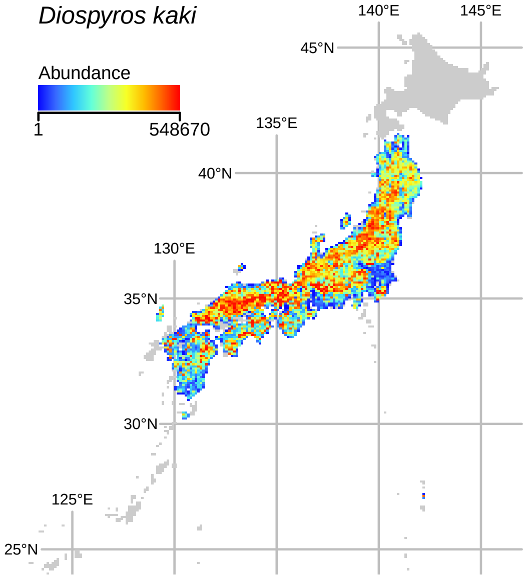
Diospyros kaki
Abundance
1
548670
140°E
145°E
45°N
135°E
40°N
130°E
35°N
30°N
125°E
25°N

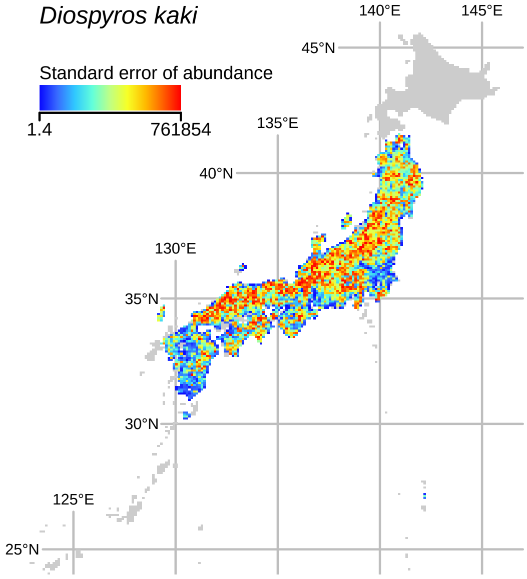
Diospyros kaki
Standard error of abundance
1.4
761854
140°E
145°E
45°N
135°E
40°N
130°E
35°N
30°N
125°E
25°N

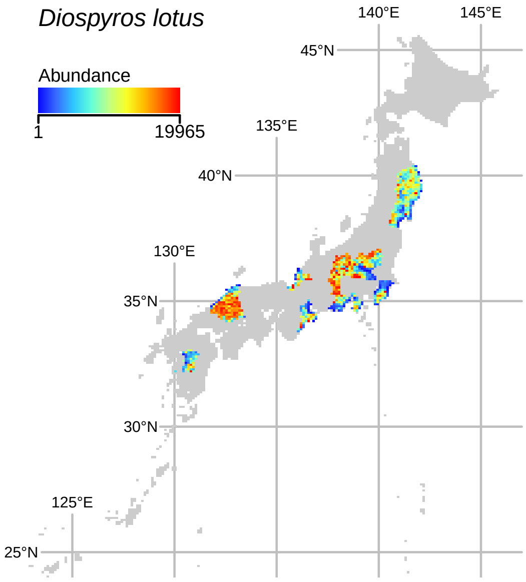
Diospyros lotus
Abundance
1
19965
140°E
145°E
45°N
135°E
40°N
130°E
35°N
30°N
125°E
25°N

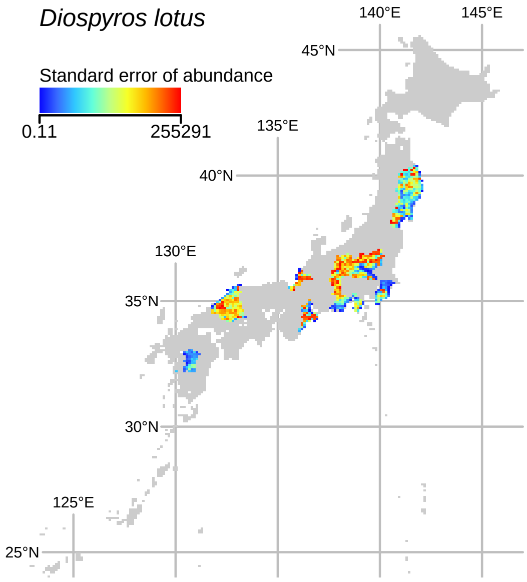
Diospyros lotus
Standard error of abundance
0.11
255291
140°E
145°E
45°N
135°E
40°N
130°E
35°N
30°N
125°E
25°N

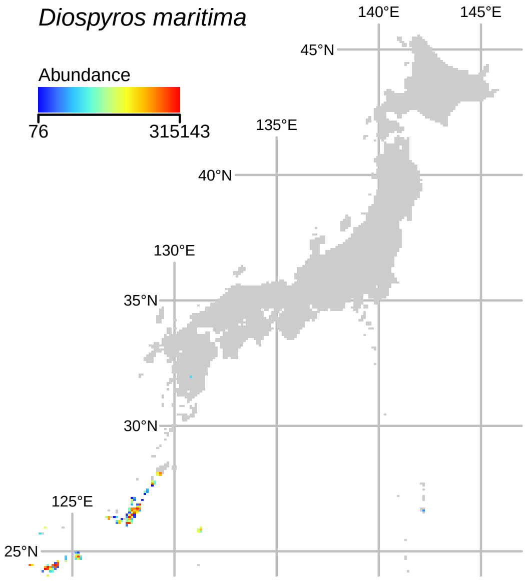
Diospyros maritima
Abundance
76
315143
140°E
145°E
45°N
135°E
40°N
130°E
35°N
30°N
125°E
25°N

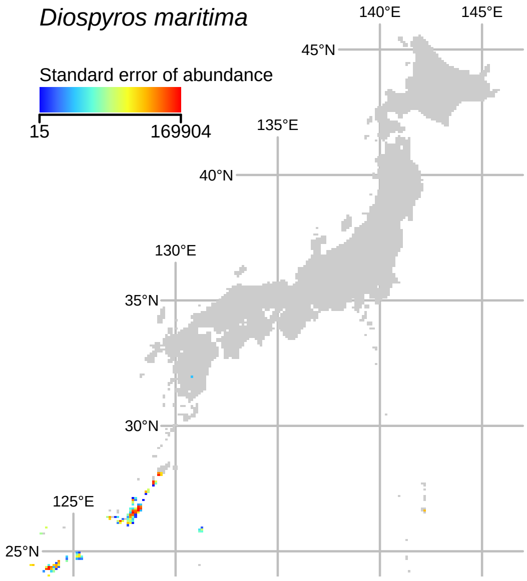
Diospyros maritima
Standard error of abundance
15
169904
140°E
145°E
45°N
135°E
40°N
130°E
35°N
30°N
125°E
25°N

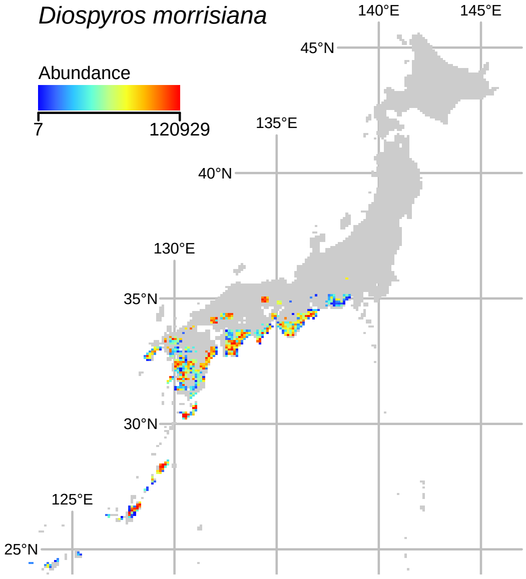
Diospyros morrisiana
Abundance
7
120929
140°E
145°E
45°N
135°E
40°N
130°E
35°N
30°N
125°E
25°N

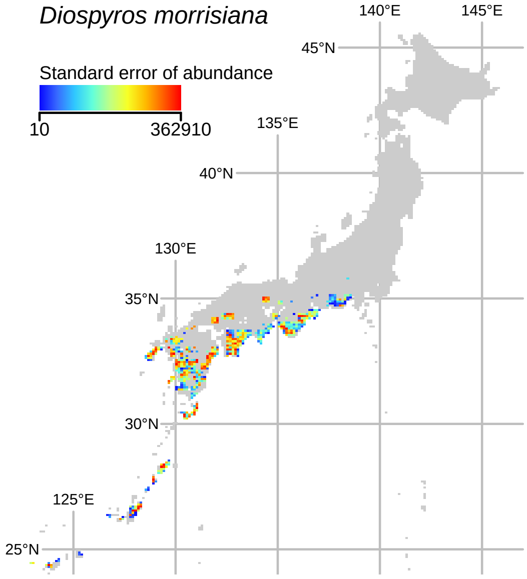
Diospyros morrisiana
Standard error of abundance
10
362910
140°E
145°E
45°N
135°E
40°N
130°E
35°N
30°N
125°E
25°N

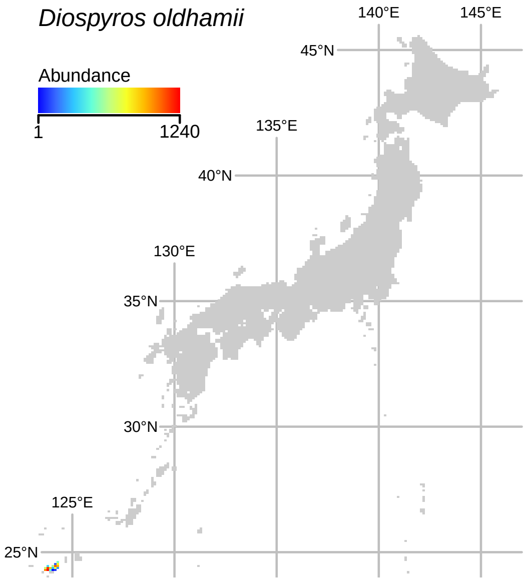
Diospyros oldhamii
Abundance
1
1240
140°E
145°E
45°N
135°E
40°N
130°E
35°N
30°N
125°E
25°N

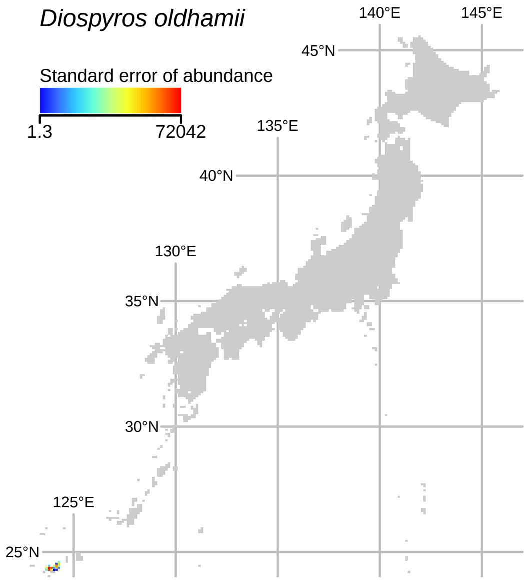
Diospyros oldhamii
Standard error of abundance
1.3
72042
140°E
145°E
45°N
135°E
40°N
130°E
35°N
30°N
125°E
25°N

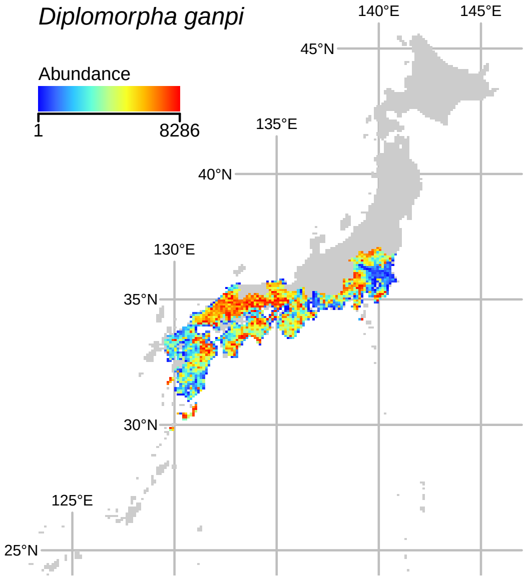
Diplomorpha ganpi
Abundance
1
8286
140°E
145°E
45°N
135°E
40°N
130°E
35°N
30°N
125°E
25°N

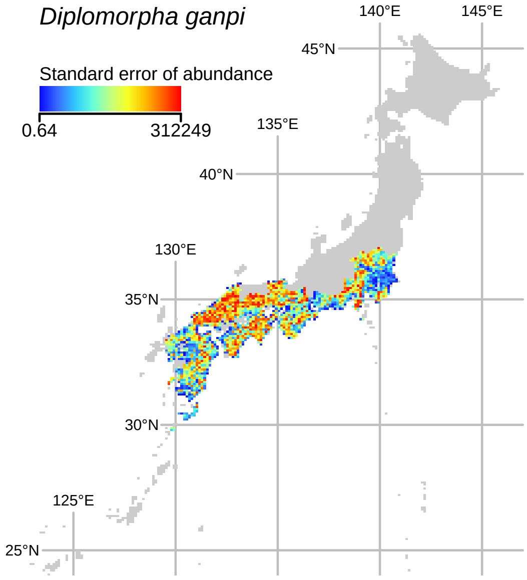
Diplomorpha ganpi
Standard error of abundance
0.64
312249
140°E
145°E
45°N
135°E
40°N
130°E
35°N
30°N
125°E
25°N

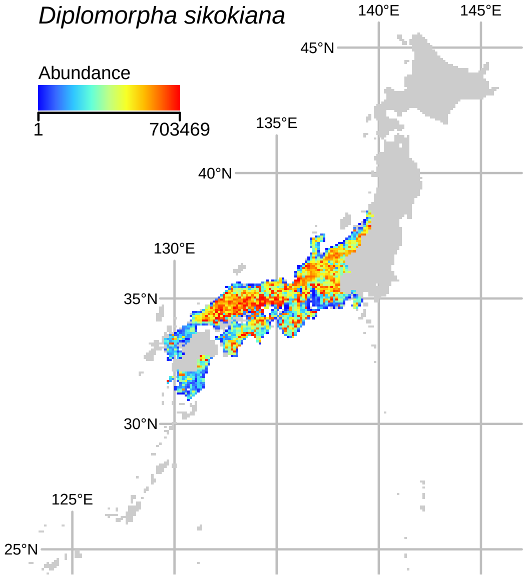
Diplomorpha sikokiana
Abundance
1
703469
140°E
145°E
45°N
135°E
40°N
130°E
35°N
30°N
125°E
25°N

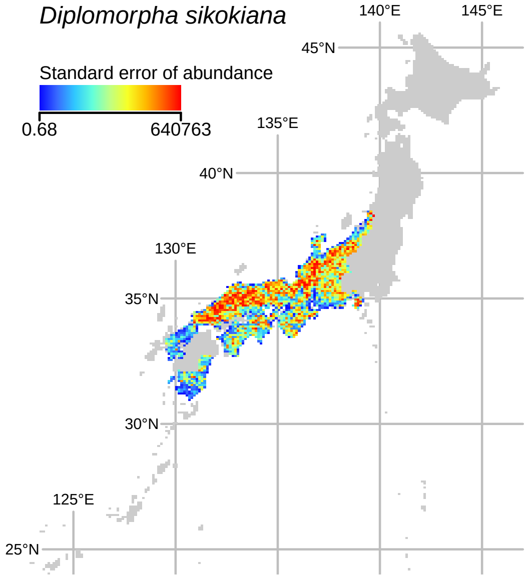
Diplomorpha sikokiana
Standard error of abundance
0.68
640763
140°E
145°E
45°N
135°E
40°N
130°E
35°N
30°N
125°E
25°N

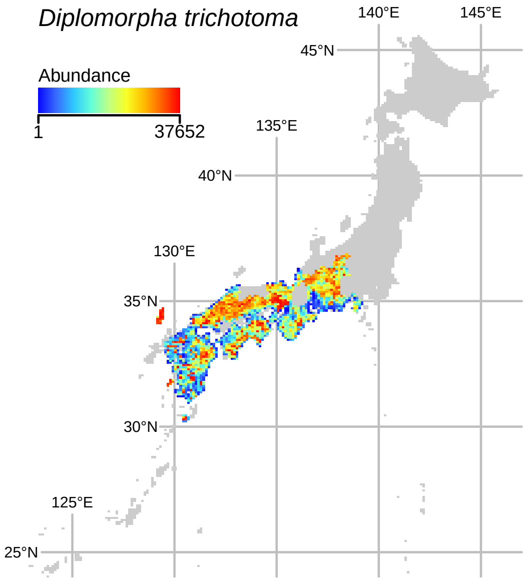
Diplomorpha trichotoma
Abundance
1
37652
140°E
145°E
45°N
135°E
40°N
130°E
35°N
30°N
125°E
25°N

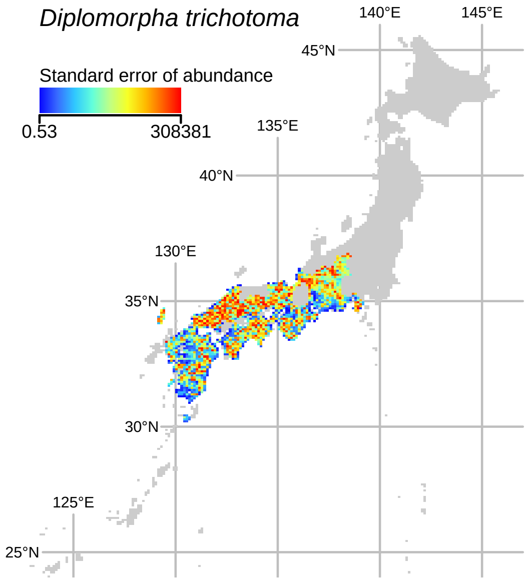
Diplomorpha trichotoma
Standard error of abundance
0.53
308381
140°E
145°E
45°N
135°E
40°N
130°E
35°N
30°N
125°E
25°N

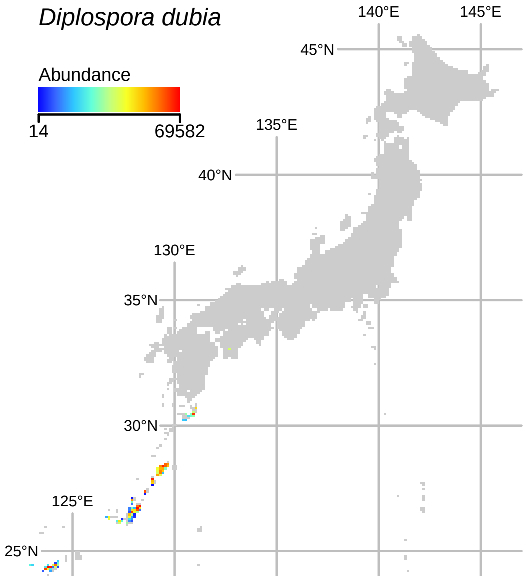
Diplospora dubia
Abundance
14
69582
140°E
145°E
45°N
135°E
40°N
130°E
35°N
30°N
125°E
25°N

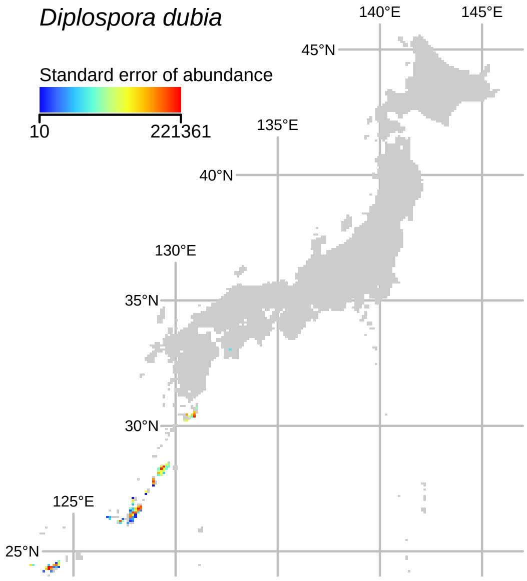
Diplospora dubia
Standard error of abundance
10
221361
140°E
145°E
45°N
135°E
40°N
130°E
35°N
30°N
125°E
25°N

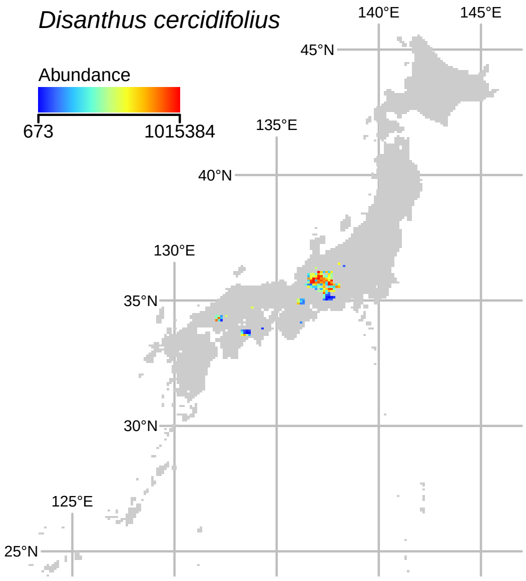
Disanthus cercidifolius
Abundance
673
1015384
140°E
145°E
45°N
135°E
40°N
130°E
35°N
30°N
125°E
25°N

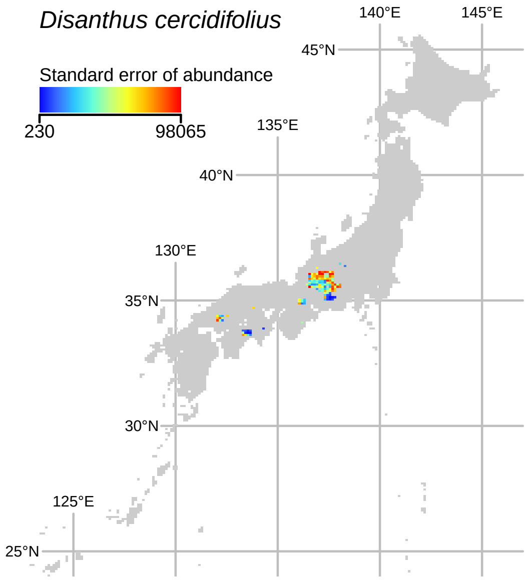
Disanthus cercidifolius
Standard error of abundance
230
98065
140°E
145°E
45°N
135°E
40°N
130°E
35°N
30°N
125°E
25°N

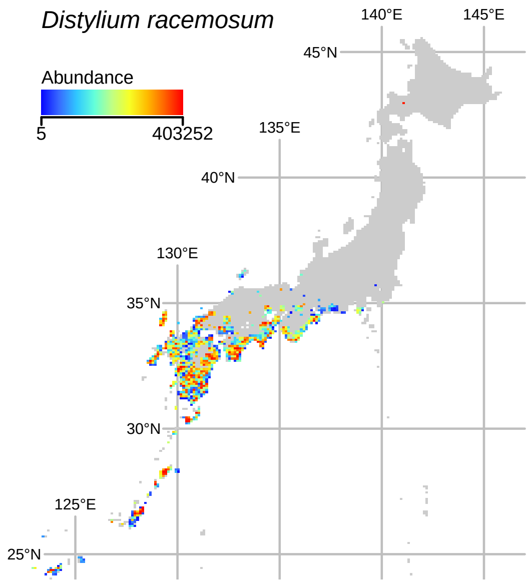

Distylium racemosum
Abundance
5
403252
140°E
145°E
45°N
135°E
40°N
130°E
35°N
30°N
125°E
25°N

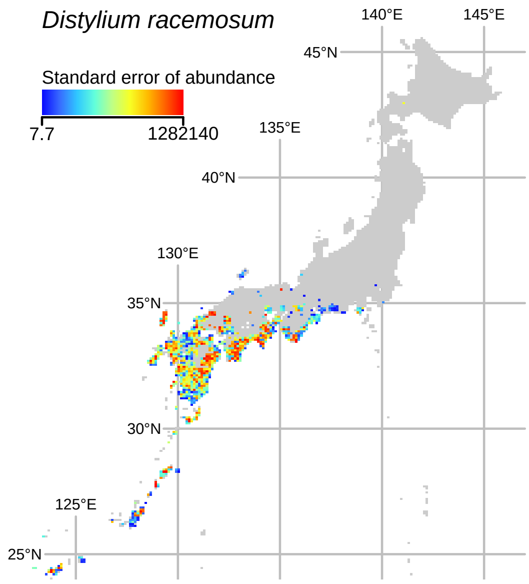

Distylium racemosum
Standard error of abundance
7.7
1282140
140°E
145°E
45°N
135°E
40°N
130°E
35°N
30°N
125°E
25°N

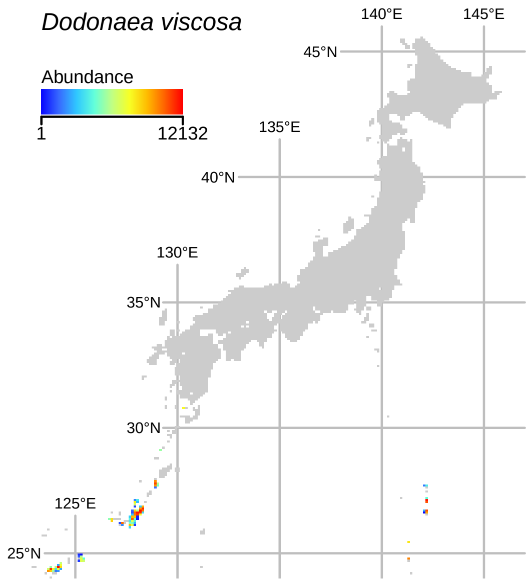

Dodonaea viscosa
Abundance
1
12132
140°E
145°E
45°N
135°E
40°N
130°E
35°N
30°N
125°E
25°N

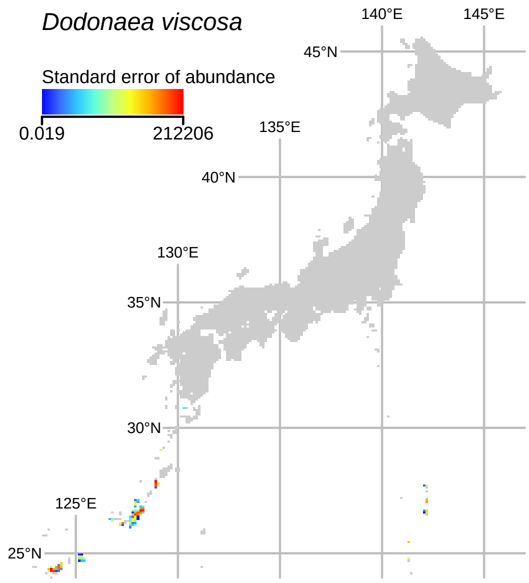

Dodonaea viscosa
Standard error of abundance
0.019
212206
140°E
145°E
45°N
135°E
40°N
130°E
35°N
30°N
125°E
25°N

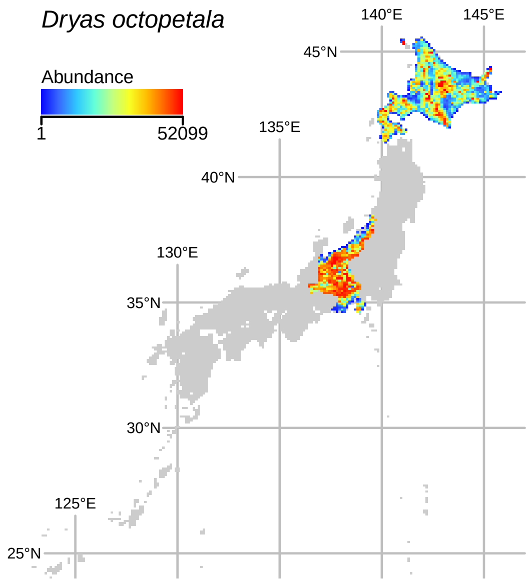

Dryas octopetala
Abundance
1
52099
140°E
145°E
45°N
135°E
40°N
130°E
35°N
30°N
125°E
25°N

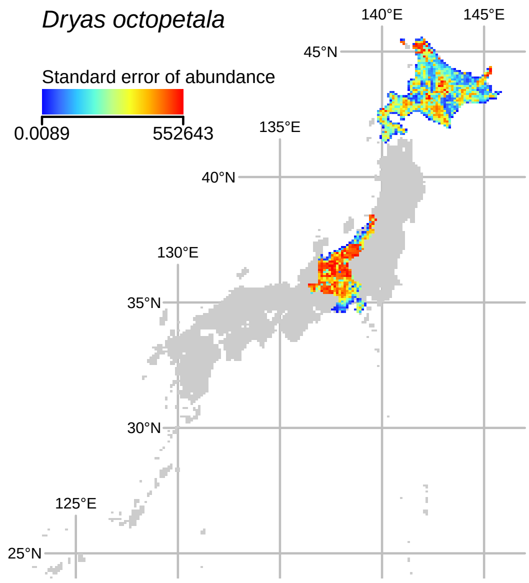

Dryas octopetala
Standard error of abundance
0.0089
552643
140°E
145°E
45°N
135°E
40°N
130°E
35°N
30°N
125°E
25°N

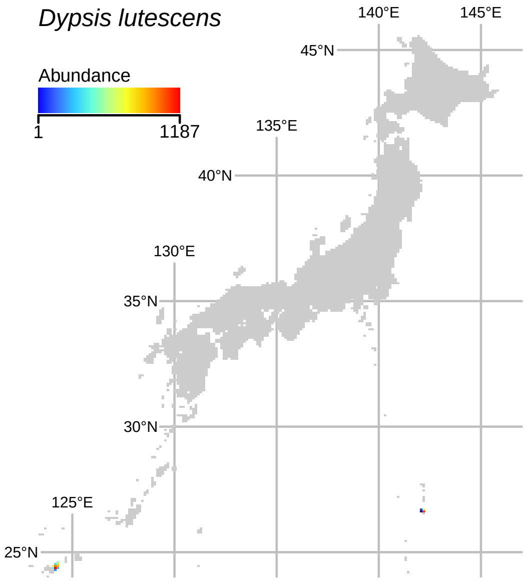
Dypsis lutescens
Abundance
1
1187
140°E
145°E
45°N
135°E
40°N
130°E
35°N
30°N
125°E
25°N

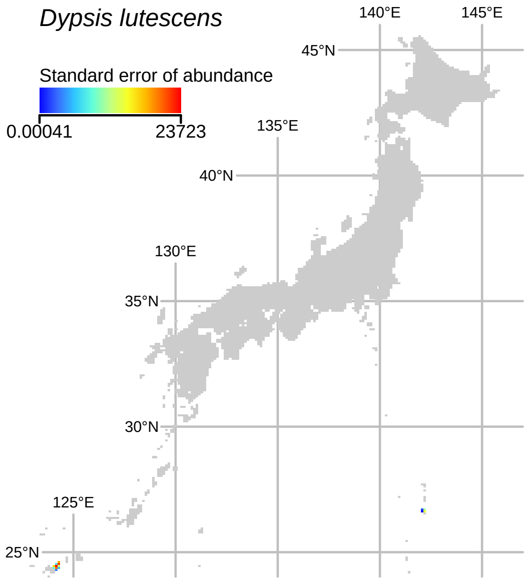
Dypsis lutescens
Standard error of abundance
0.00041
23723
140°E
145°E
45°N
135°E
40°N
130°E
35°N
30°N
125°E
25°N

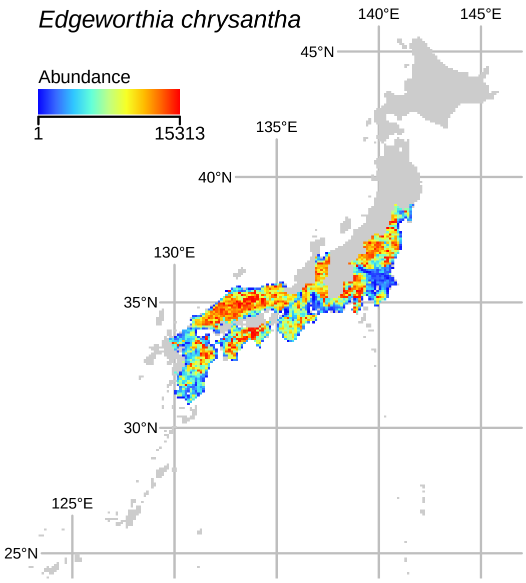
Edgeworthia chrysantha
Abundance
1
15313
140°E
145°E
45°N
135°E
40°N
130°E
35°N
30°N
125°E
25°N

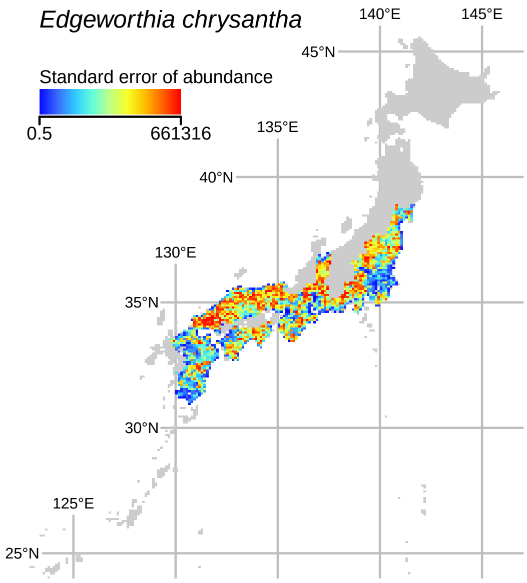
Edgeworthia chrysantha
Standard error of abundance
0.5
661316
140°E
145°E
45°N
135°E
40°N
130°E
35°N
30°N
125°E
25°N

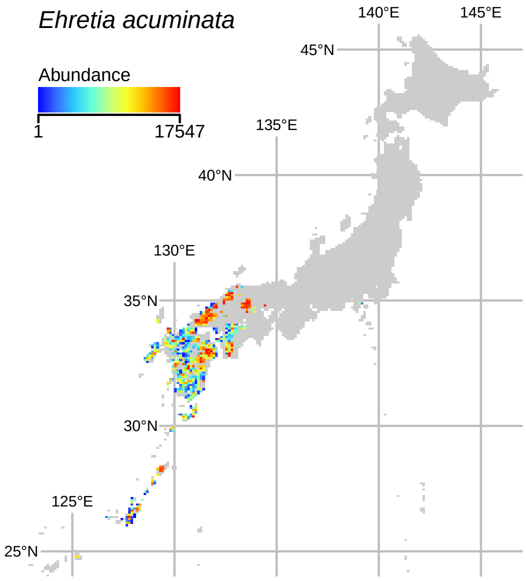
Ehretia acuminata
Abundance
1
17547
140°E
145°E
45°N
135°E
40°N
130°E
35°N
30°N
125°E
25°N

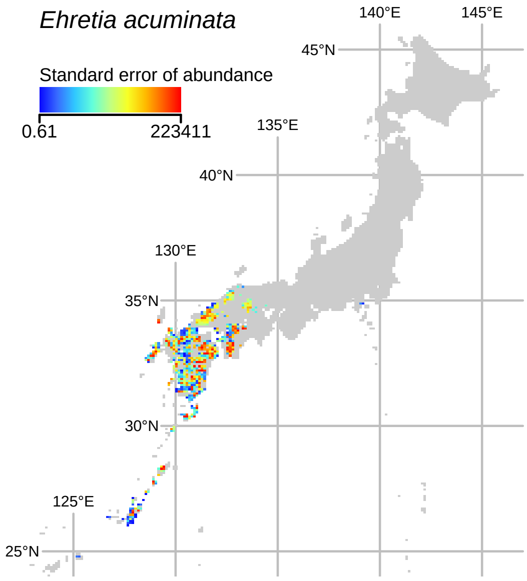
Ehretia acuminata
Standard error of abundance
0.61
223411
140°E
145°E
45°N
135°E
40°N
130°E
35°N
30°N
125°E
25°N

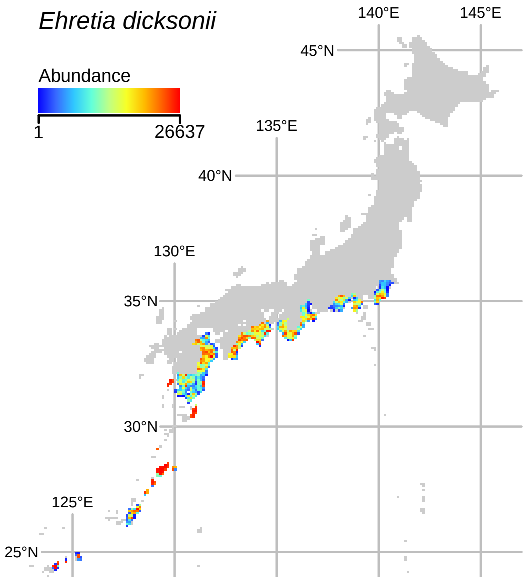
Ehretia dicksonii
Abundance
1
26637
140°E
145°E
45°N
135°E
40°N
130°E
35°N
30°N
125°E
25°N

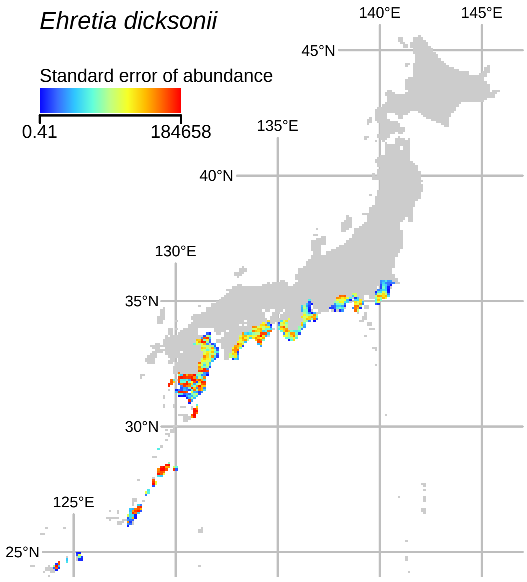
Ehretia dicksonii
Standard error of abundance
0.41
184658
140°E
145°E
45°N
135°E
40°N
130°E
35°N
30°N
125°E
25°N

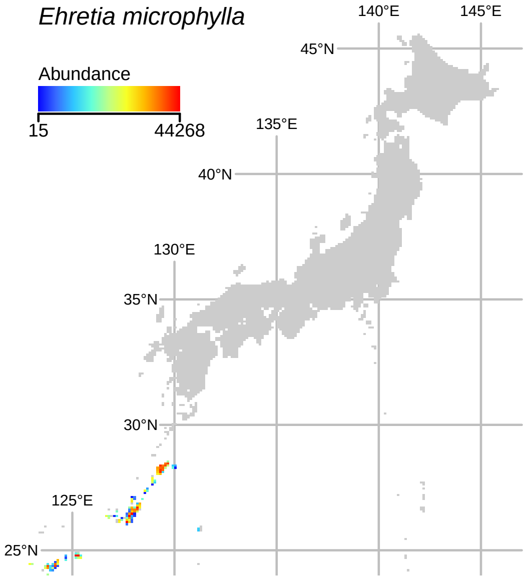
Ehretia microphylla
Abundance
15
44268
140°E
145°E
45°N
135°E
40°N
130°E
35°N
30°N
125°E
25°N

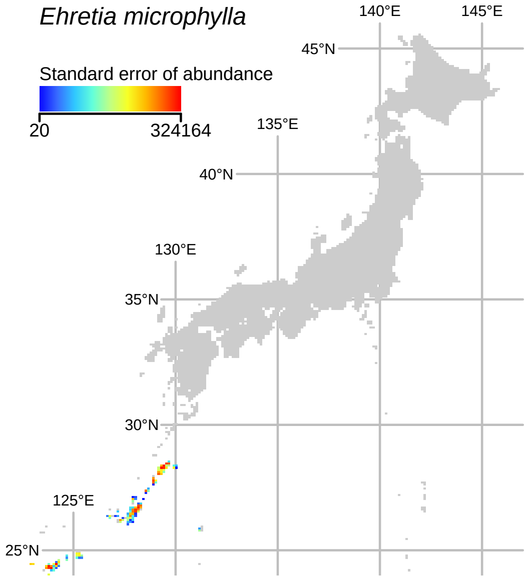
Ehretia microphylla
Standard error of abundance
20
324164
140°E
145°E
45°N
135°E
40°N
130°E
35°N
30°N
125°E
25°N

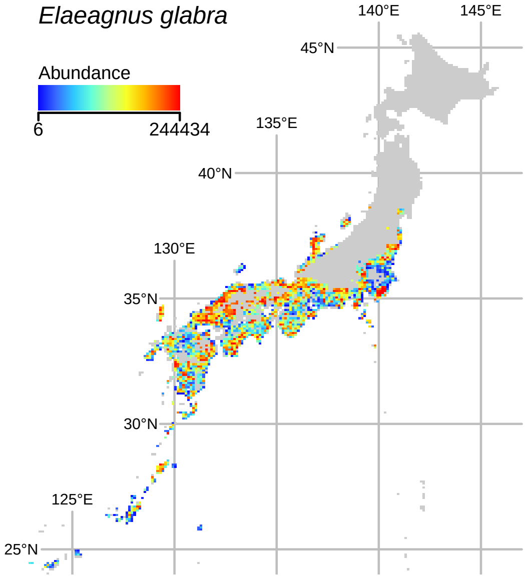
Elaeagnus glabra
Abundance
6
244434
140°E
145°E
45°N
135°E
40°N
130°E
35°N
30°N
125°E
25°N

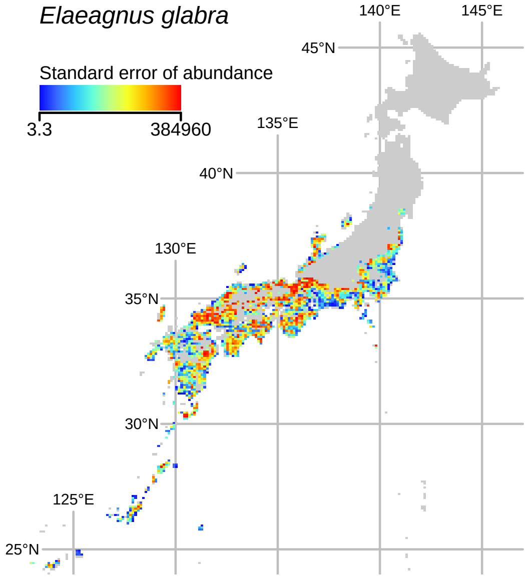
Elaeagnus glabra
Standard error of abundance
3.3
384960
140°E
145°E
45°N
135°E
40°N
130°E
35°N
30°N
125°E
25°N

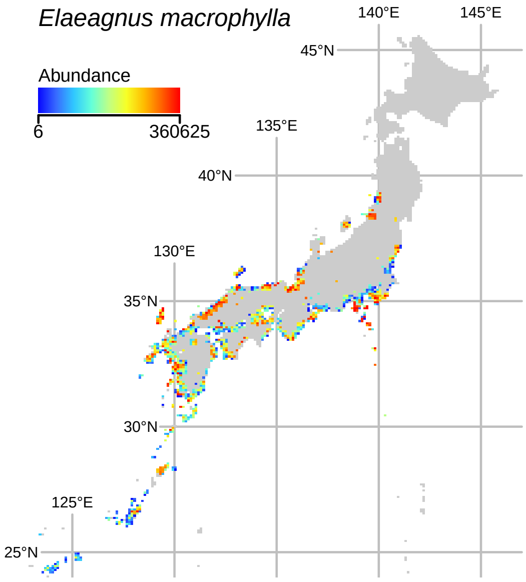
Elaeagnus macrophylla
Abundance
6
360625
140°E
145°E
45°N
135°E
40°N
130°E
35°N
30°N
125°E
25°N

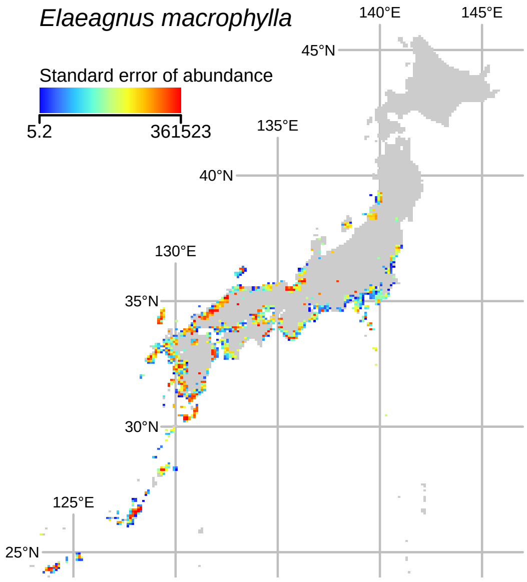
Elaeagnus macrophylla
Standard error of abundance
5.2
361523
140°E
145°E
45°N
135°E
40°N
130°E
35°N
30°N
125°E
25°N

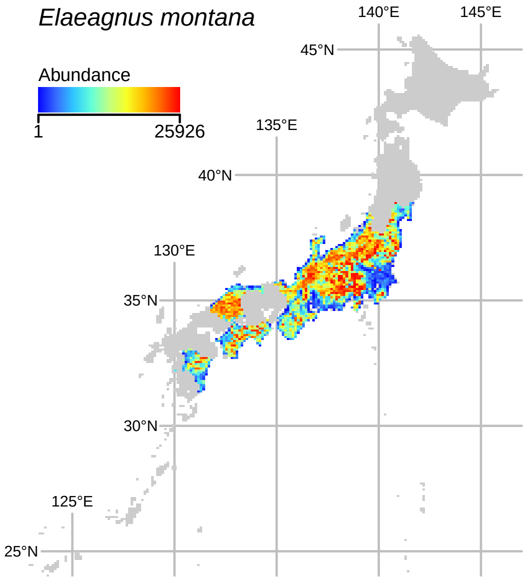
Elaeagnus montana
Abundance
1
25926
140°E
145°E
45°N
135°E
40°N
130°E
35°N
30°N
125°E
25°N

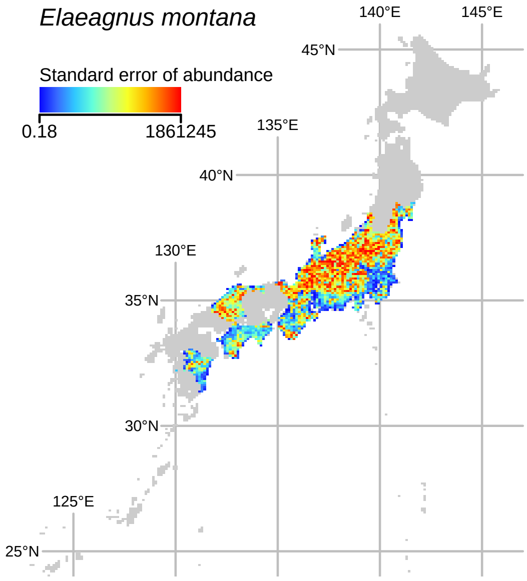
Elaeagnus montana
Standard error of abundance
0.18
1861245
140°E
145°E
45°N
135°E
40°N
130°E
35°N
30°N
125°E
25°N

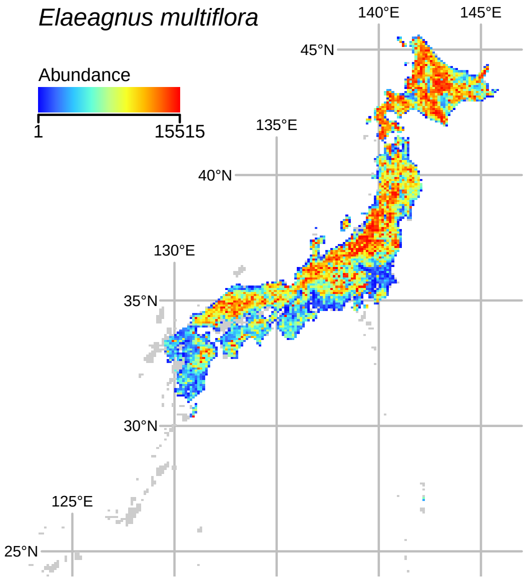
Elaeagnus multiflora
Abundance
1
15515
140°E
145°E
45°N
135°E
40°N
130°E
35°N
30°N
125°E
25°N

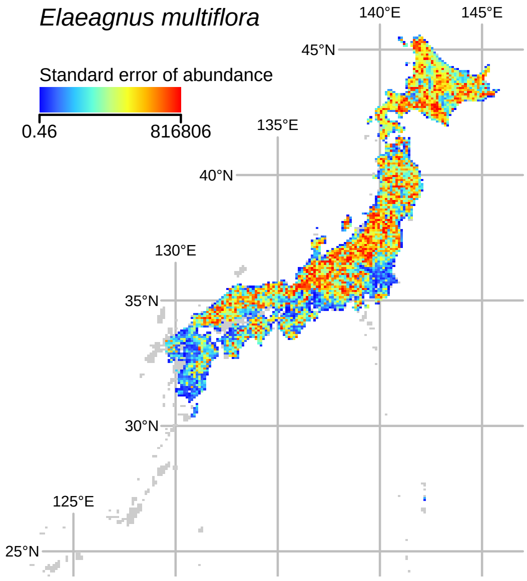
Elaeagnus multiflora
Standard error of abundance
0.46
816806
140°E
145°E
45°N
135°E
40°N
130°E
35°N
30°N
125°E
25°N

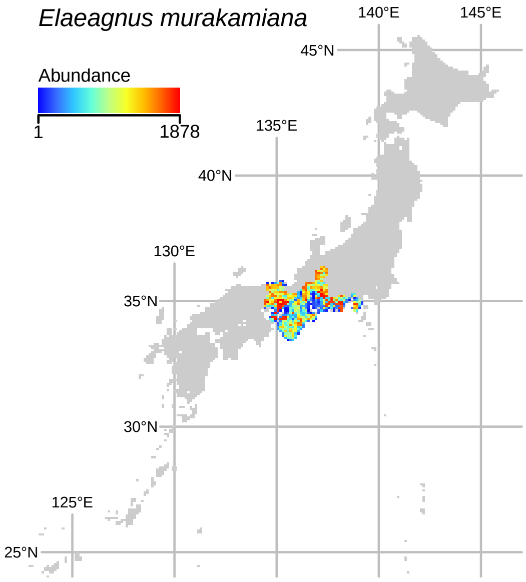
Elaeagnus murakamiana
Abundance
1
1878
140°E
145°E
45°N
135°E
40°N
130°E
35°N
30°N
125°E
25°N

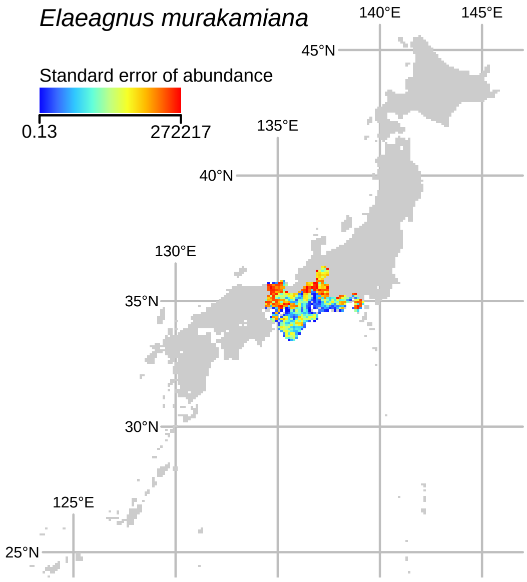
Elaeagnus murakamiana
Standard error of abundance
0.13
272217
140°E
145°E
45°N
135°E
40°N
130°E
35°N
30°N
125°E
25°N

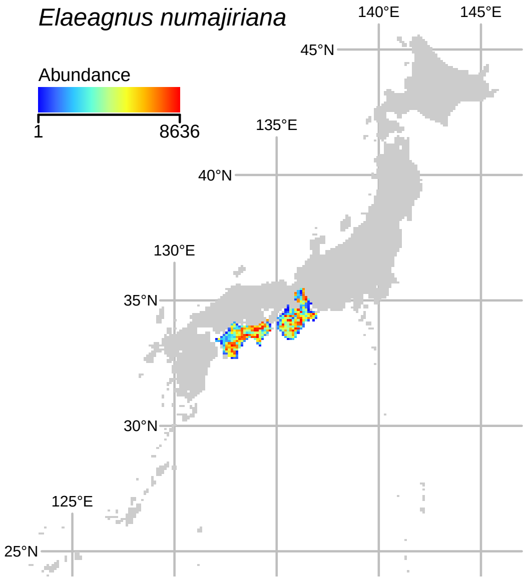
Elaeagnus numajiriana
Abundance
1
8636
140°E
145°E
45°N
135°E
40°N
130°E
35°N
30°N
125°E
25°N

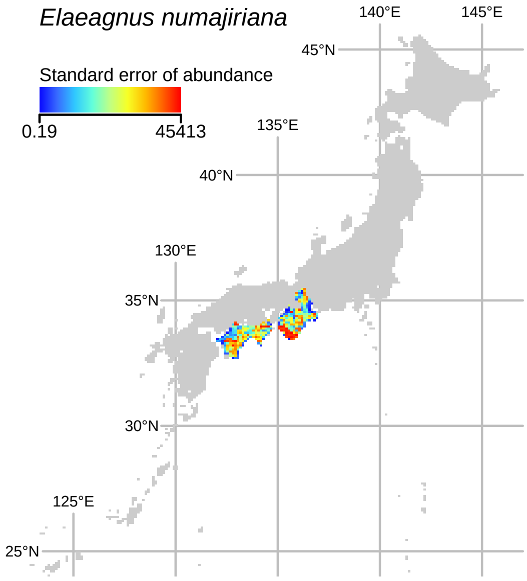
Elaeagnus numajiriana
Standard error of abundance
0.19
45413
140°E
145°E
45°N
135°E
40°N
130°E
35°N
30°N
125°E
25°N

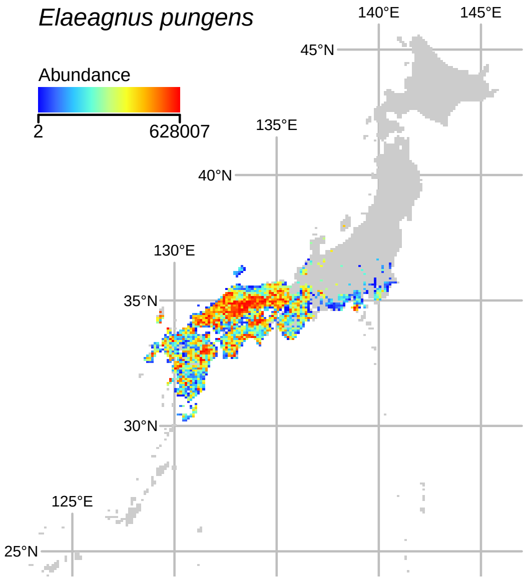
Elaeagnus pungens
Abundance
2
628007
140°E
145°E
45°N
135°E
40°N
130°E
35°N
30°N
125°E
25°N

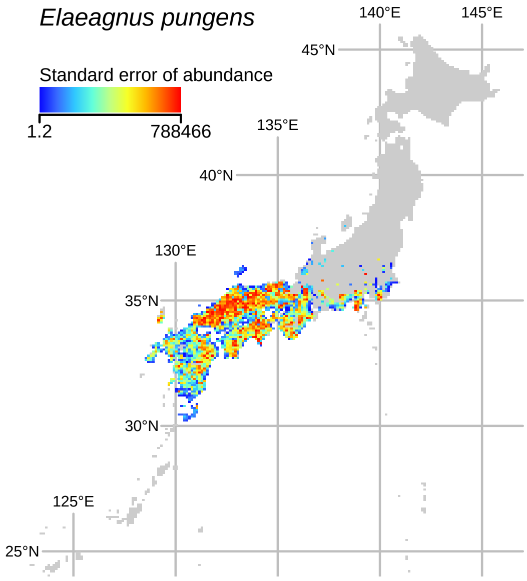
Elaeagnus pungens
Standard error of abundance
1.2
788466
140°E
145°E
45°N
135°E
40°N
130°E
35°N
30°N
125°E
25°N

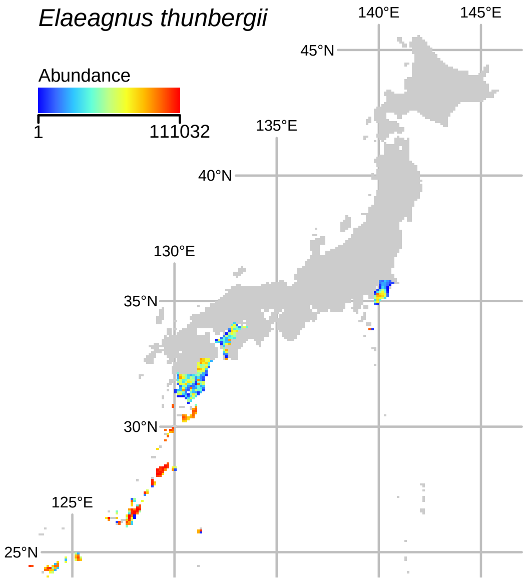
Elaeagnus thunbergii
Abundance
1
111032
140°E
145°E
45°N
135°E
40°N
130°E
35°N
30°N
125°E
25°N

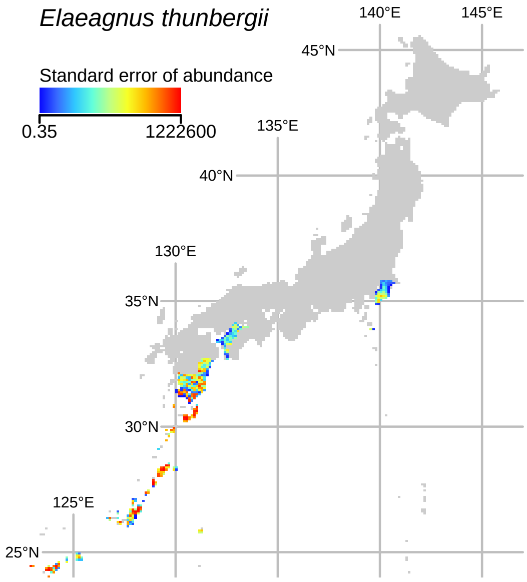
Elaeagnus thunbergii
Standard error of abundance
0.35
1222600
140°E
145°E
45°N
135°E
40°N
130°E
35°N
30°N
125°E
25°N

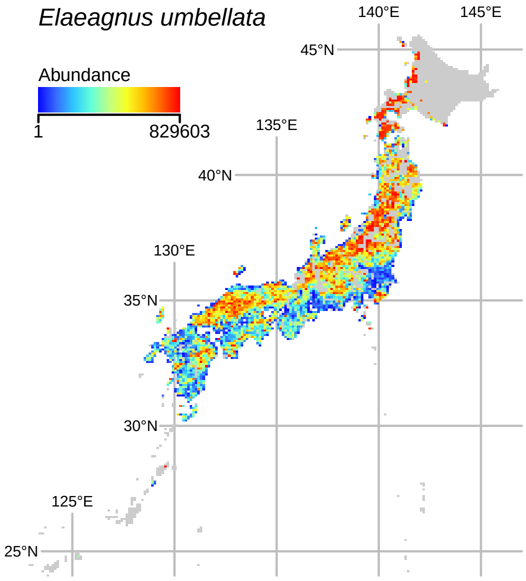
Elaeagnus umbellata
Abundance
1
829603
140°E
145°E
45°N
135°E
40°N
130°E
35°N
30°N
125°E
25°N

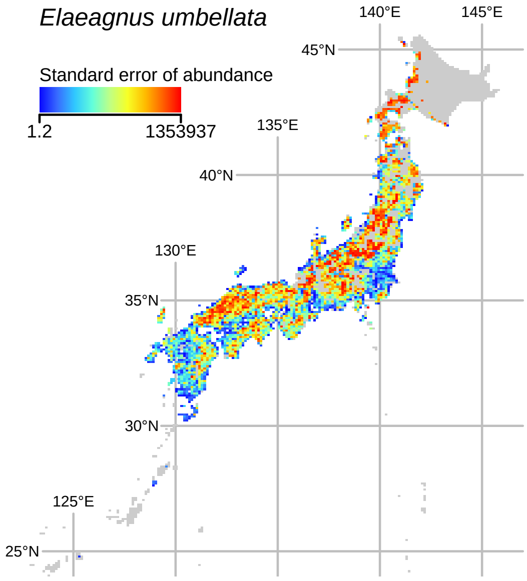
Elaeagnus umbellata
Standard error of abundance
1.2
1353937
140°E
145°E
45°N
135°E
40°N
130°E
35°N
30°N
125°E
25°N

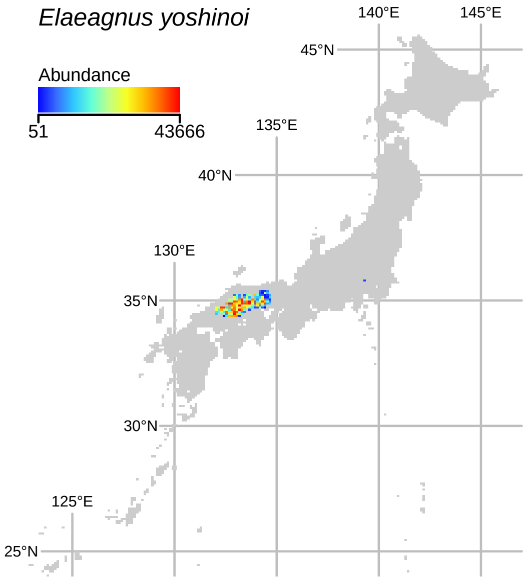
Elaeagnus yoshinoi
Abundance
51
43666
140°E
145°E
45°N
135°E
40°N
130°E
35°N
30°N
125°E
25°N

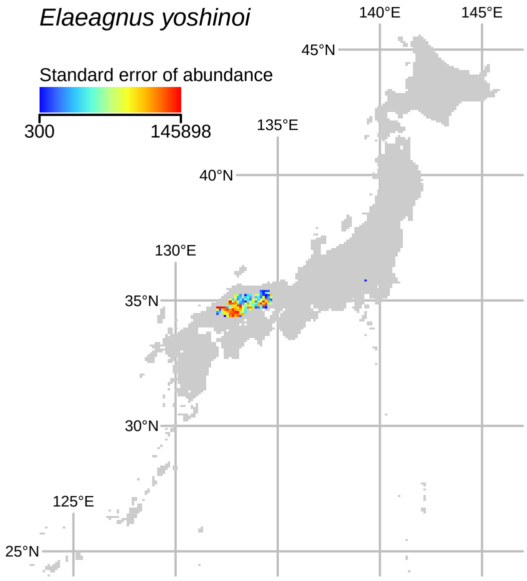
Elaeagnus yoshinoi
Standard error of abundance
300
145898
140°E
145°E
45°N
135°E
40°N
130°E
35°N
30°N
125°E
25°N

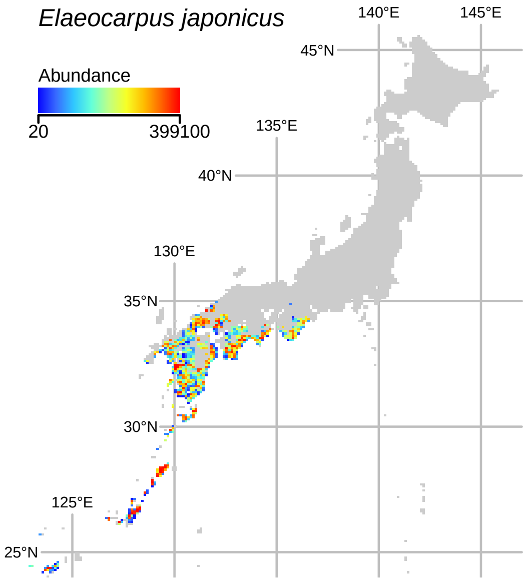
Elaeocarpus japonicus
Abundance
20
399100
140°E
145°E
45°N
135°E
40°N
130°E
35°N
30°N
125°E
25°N

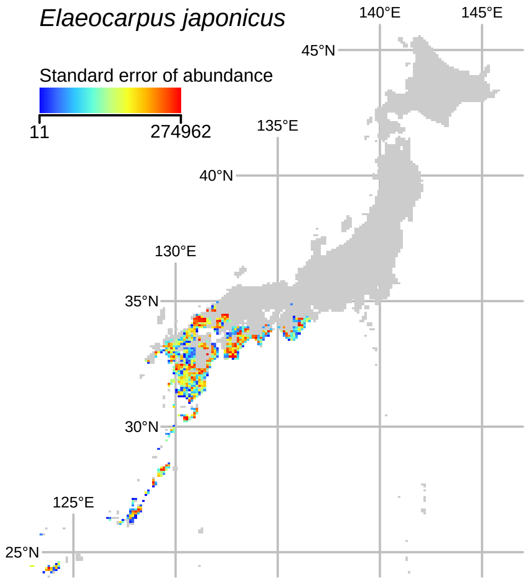
Elaeocarpus japonicus
Standard error of abundance
11
274962
140°E
145°E
45°N
135°E
40°N
130°E
35°N
30°N
125°E
25°N

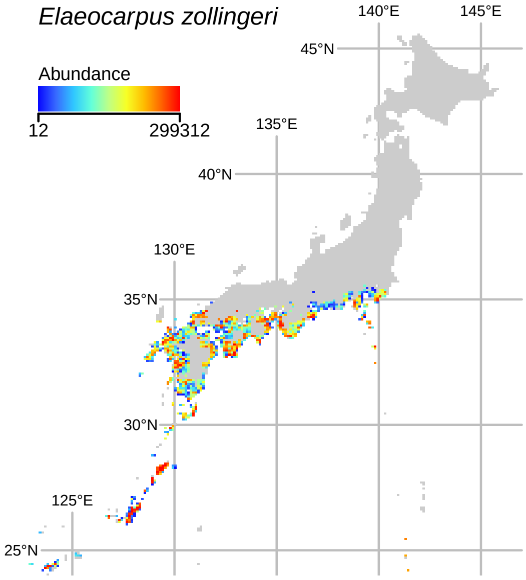
Elaeocarpus zollingeri
Abundance
12
299312
140°E
145°E
45°N
135°E
40°N
130°E
35°N
30°N
125°E
25°N

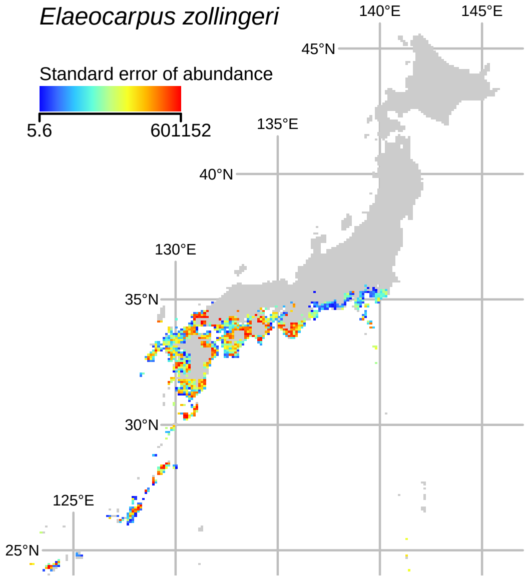
Elaeocarpus zollingeri
Standard error of abundance
5.6
601152
140°E
145°E
45°N
135°E
40°N
130°E
35°N
30°N
125°E
25°N

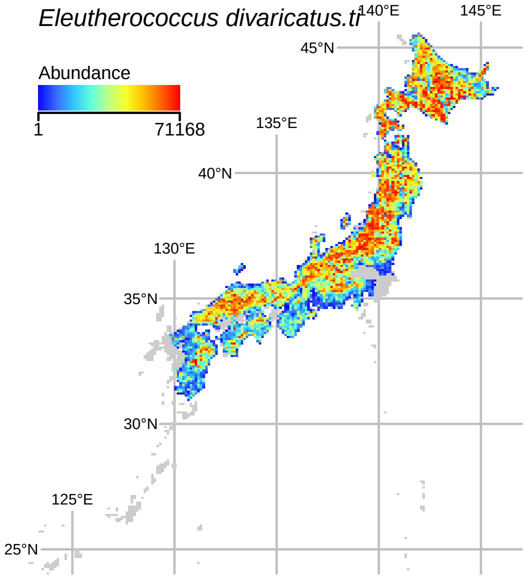
Eleutherococcus divaricatus.ti
Abundance
1
71168
140°E
145°E
45°N
135°E
40°N
130°E
35°N
30°N
125°E
25°N

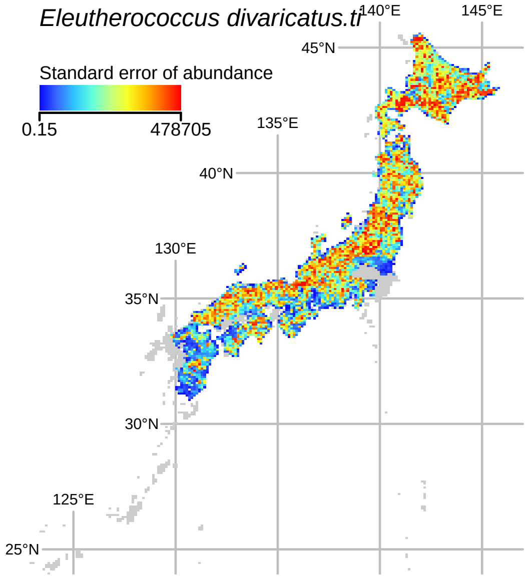
Eleutherococcus divaricatus.ti
Standard error of abundance
0.15
478705
140°E
145°E
45°N
135°E
40°N
130°E
35°N
30°N
125°E
25°N

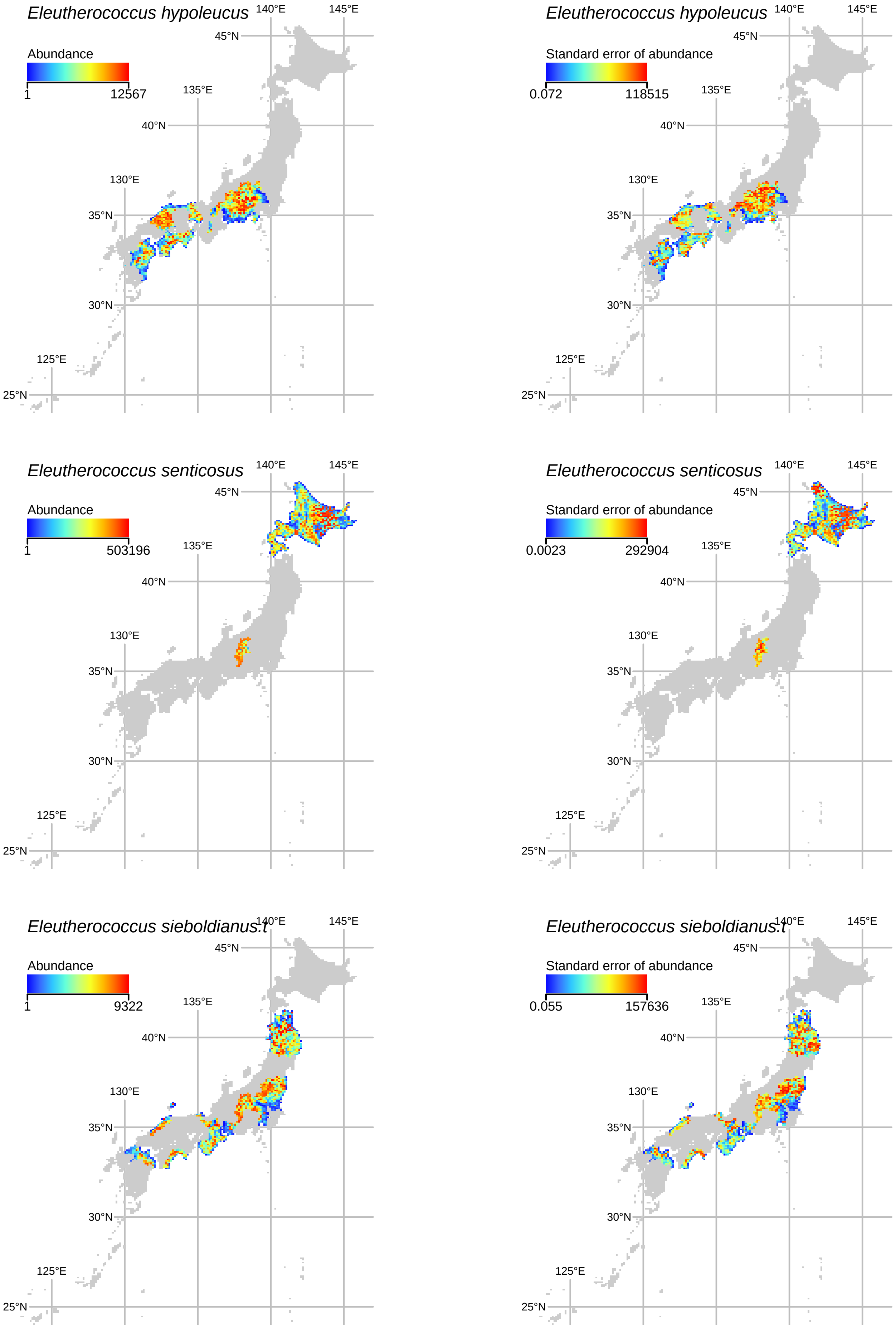
Eleutherococcus hypoleucus
Abundance
1
12567
Eleutherococcus hypoleucus
Standard error of abundance
0.072
118515
Eleutherococcus senticosus
Abundance
1
503196
Eleutherococcus senticosus
Standard error of abundance
0.0023
292904
Eleutherococcus sieboldianus.t
Abundance
1
9322
Eleutherococcus sieboldianus.t
Standard error of abundance
0.055
157636
125°E
130°E
135°E
140°E
145°E
25°N
30°N
35°N
40°N
45°N

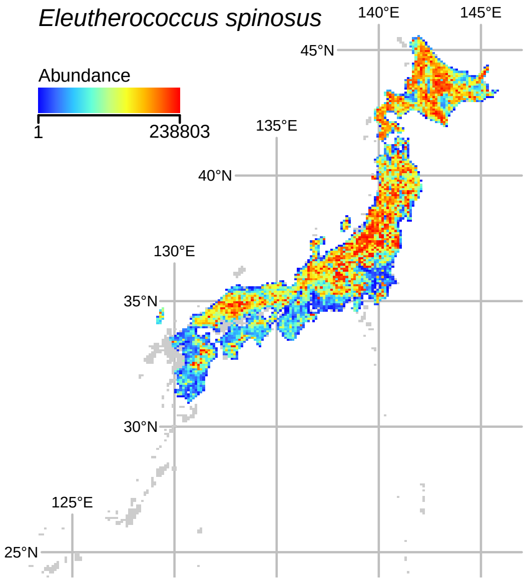
Eleutherococcus spinosus
Abundance
1
238803
140°E
145°E
135°E
130°E
125°E
45°N
40°N
35°N
30°N
25°N

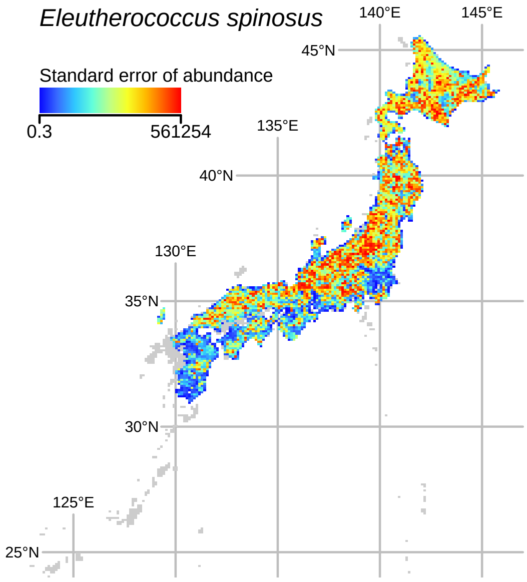
Eleutherococcus spinosus
Standard error of abundance
0.3
561254
140°E
145°E
135°E
130°E
125°E
45°N
40°N
35°N
30°N
25°N

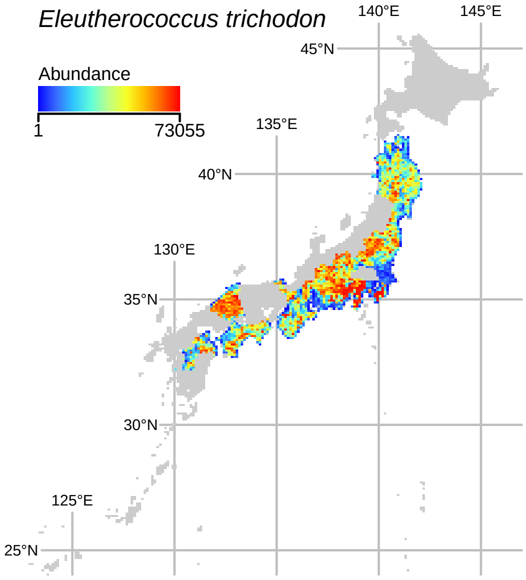
Eleutherococcus trichodon
Abundance
1
73055
140°E
145°E
135°E
130°E
125°E
45°N
40°N
35°N
30°N
25°N

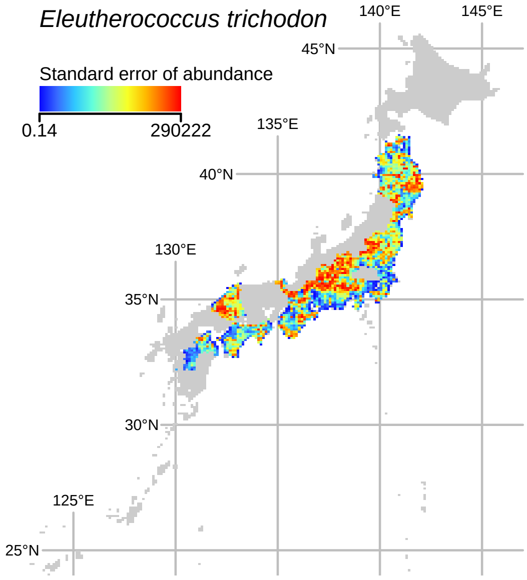
Eleutherococcus trichodon
Standard error of abundance
0.14
290222
140°E
145°E
135°E
130°E
125°E
45°N
40°N
35°N
30°N
25°N

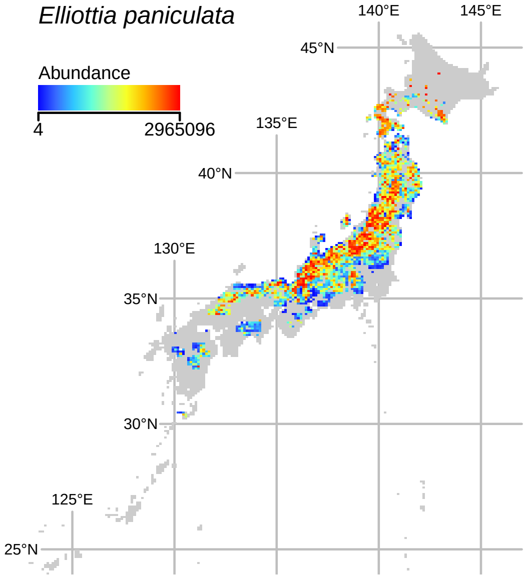
Elliottia paniculata
Abundance
4
2965096
140°E
145°E
135°E
130°E
125°E
45°N
40°N
35°N
30°N
25°N

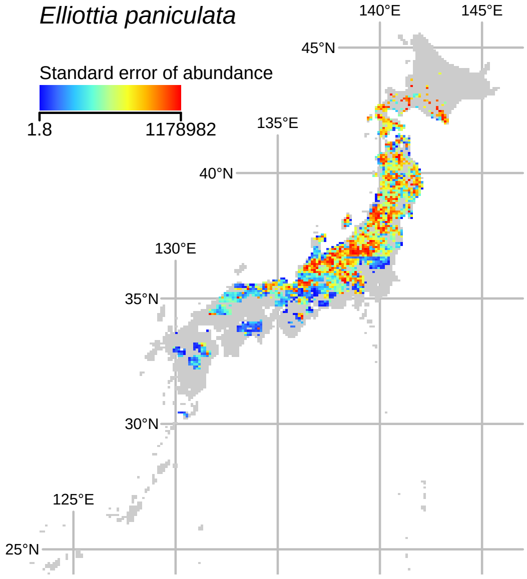
Elliottia paniculata
Standard error of abundance
1.8
1178982
140°E
145°E
135°E
130°E
125°E
45°N
40°N
35°N
30°N
25°N

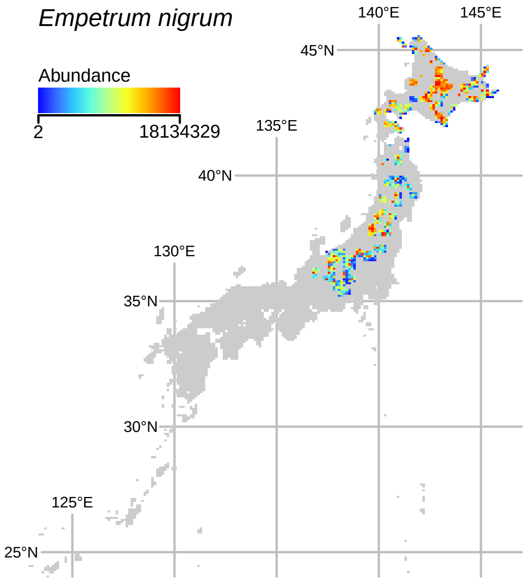
Empetrum nigrum
Abundance
2
18134329
140°E
145°E
45°N
135°E
40°N
130°E
35°N
30°N
125°E
25°N

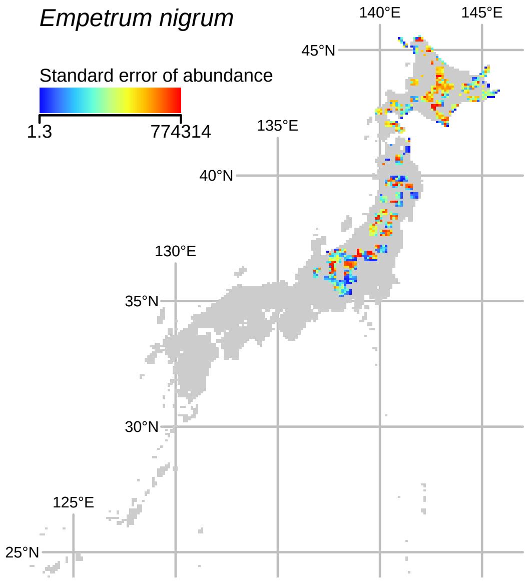
Empetrum nigrum
Standard error of abundance
1.3
774314
140°E
145°E
45°N
135°E
40°N
130°E
35°N
30°N
125°E
25°N

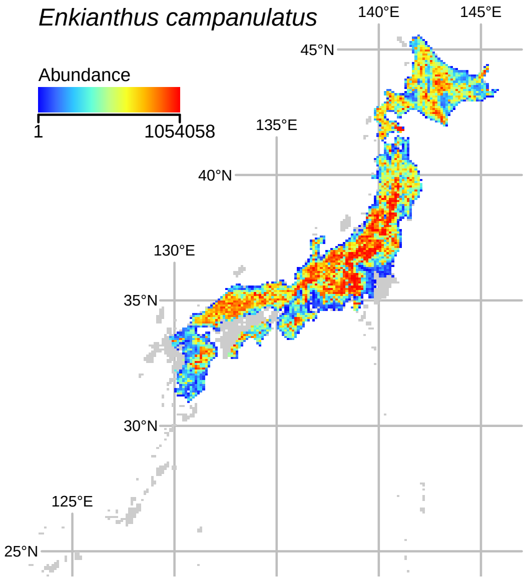
Enkianthus campanulatus
Abundance
1
1054058
140°E
145°E
45°N
135°E
40°N
130°E
35°N
30°N
125°E
25°N

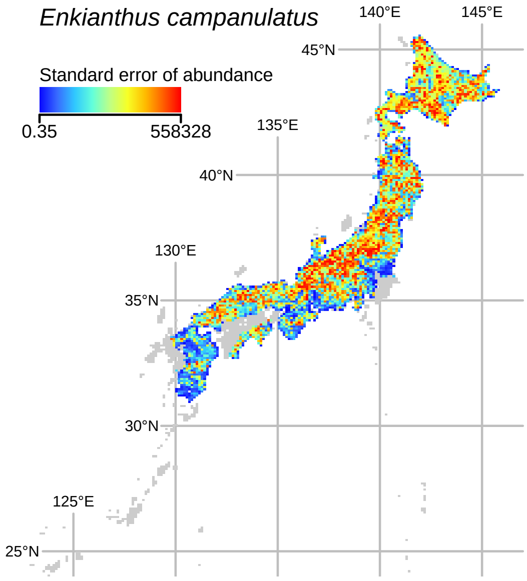
Enkianthus campanulatus
Standard error of abundance
0.35
558328
140°E
145°E
45°N
135°E
40°N
130°E
35°N
30°N
125°E
25°N

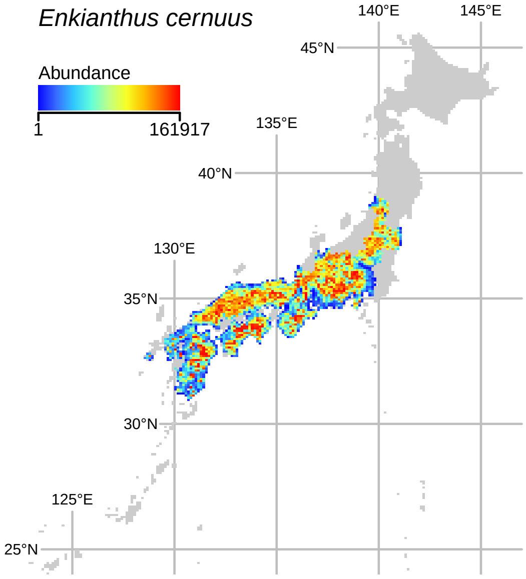
Enkianthus cernuus
Abundance
1
161917
140°E
145°E
45°N
135°E
40°N
130°E
35°N
30°N
125°E
25°N

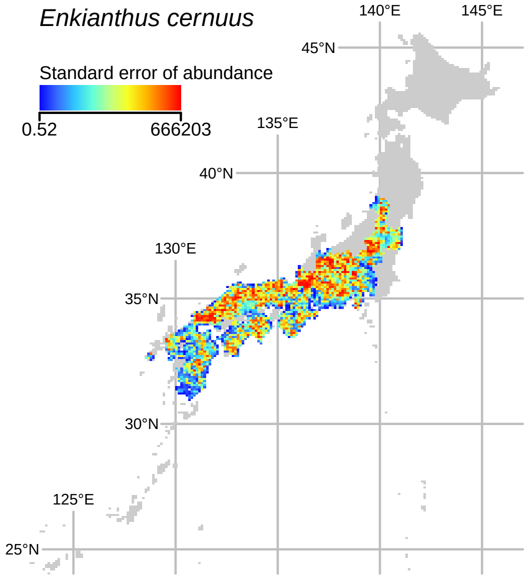
Enkianthus cernuus
Standard error of abundance
0.52
666203
140°E
145°E
45°N
135°E
40°N
130°E
35°N
30°N
125°E
25°N

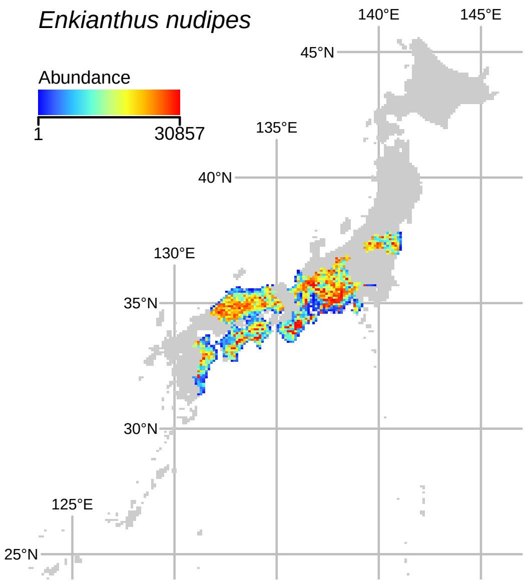
Enkianthus nudipes
Abundance
1
30857
140°E
145°E
135°E
130°E
125°E
45°N
40°N
35°N
30°N
25°N

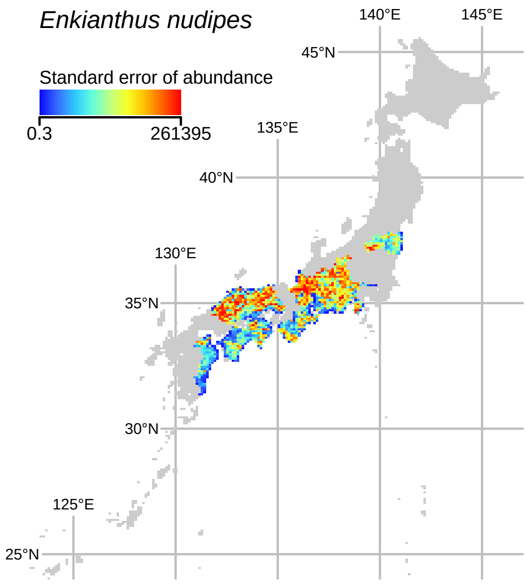
Enkianthus nudipes
Standard error of abundance
0.3
261395
140°E
145°E
135°E
130°E
125°E
45°N
40°N
35°N
30°N
25°N

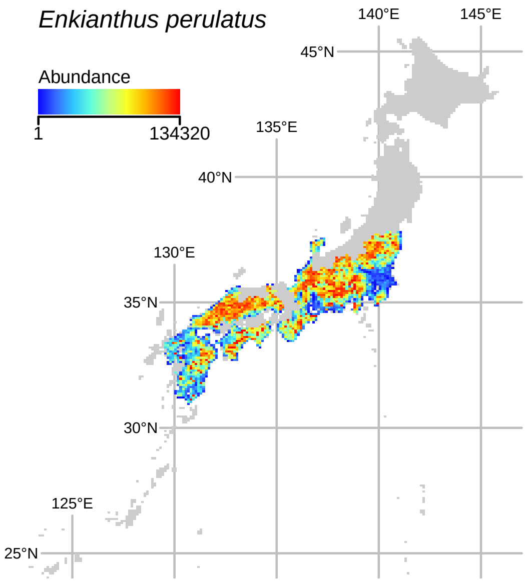
Enkianthus perulatus
Abundance
1
134320
140°E
145°E
135°E
130°E
125°E
45°N
40°N
35°N
30°N
25°N

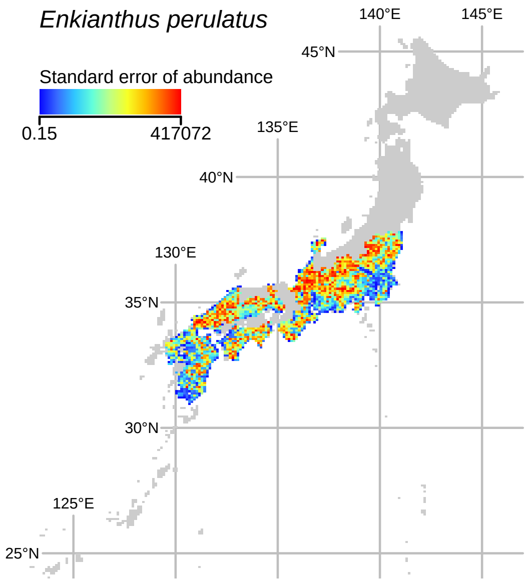
Enkianthus perulatus
Standard error of abundance
0.15
417072
140°E
145°E
135°E
130°E
125°E
45°N
40°N
35°N
30°N
25°N

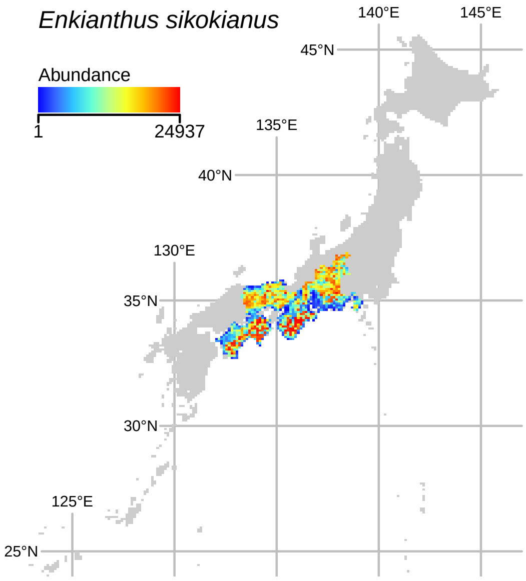
Enkianthus sikokianus
Abundance
1
24937
140°E
145°E
135°E
130°E
125°E
45°N
40°N
35°N
30°N
25°N

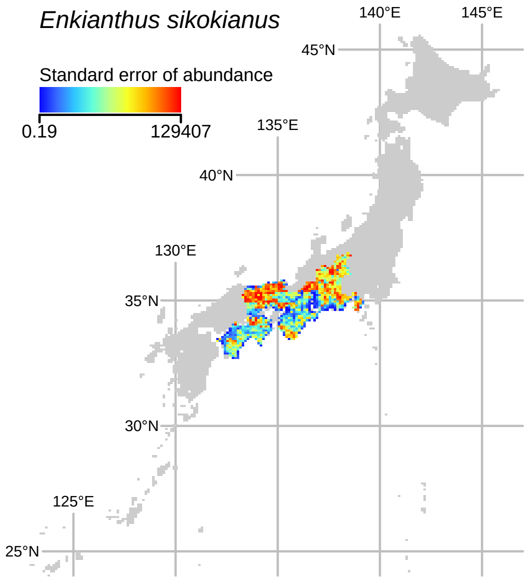
Enkianthus sikokianus
Standard error of abundance
0.19
129407
140°E
145°E
135°E
130°E
125°E
45°N
40°N
35°N
30°N
25°N

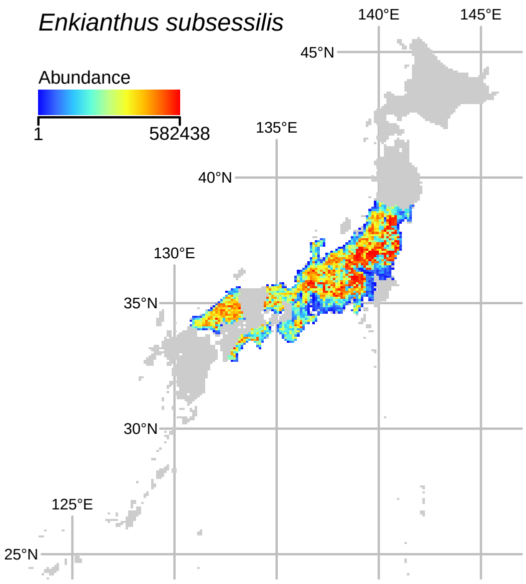
Enkianthus subsessilis
Abundance
1
582438
140°E
145°E
45°N
135°E
40°N
130°E
35°N
30°N
125°E
25°N

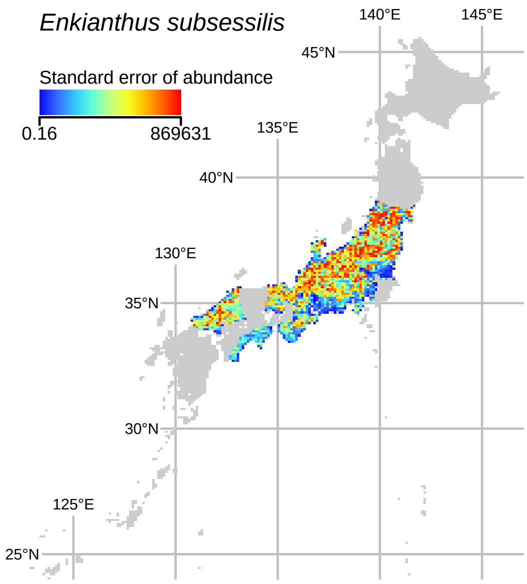
Enkianthus subsessilis
Standard error of abundance
0.16
869631
140°E
145°E
45°N
135°E
40°N
130°E
35°N
30°N
125°E
25°N

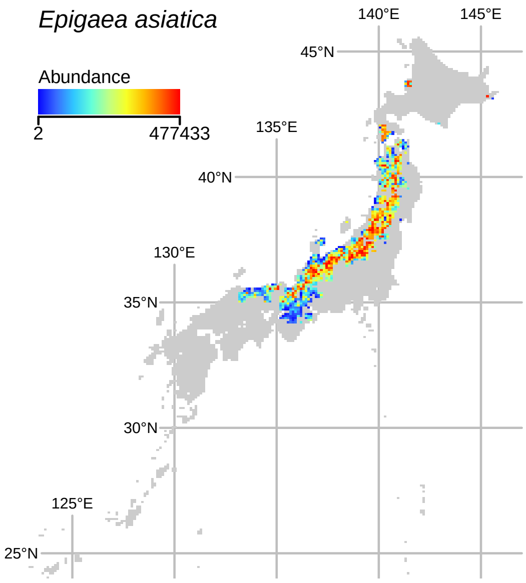
Epigaea asiatica
Abundance
2
477433
140°E
145°E
45°N
135°E
40°N
130°E
35°N
30°N
125°E
25°N

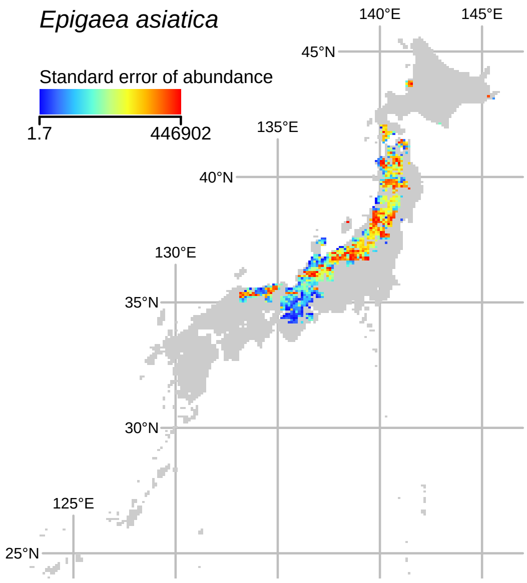
Epigaea asiatica
Standard error of abundance
1.7
446902
140°E
145°E
45°N
135°E
40°N
130°E
35°N
30°N
125°E
25°N

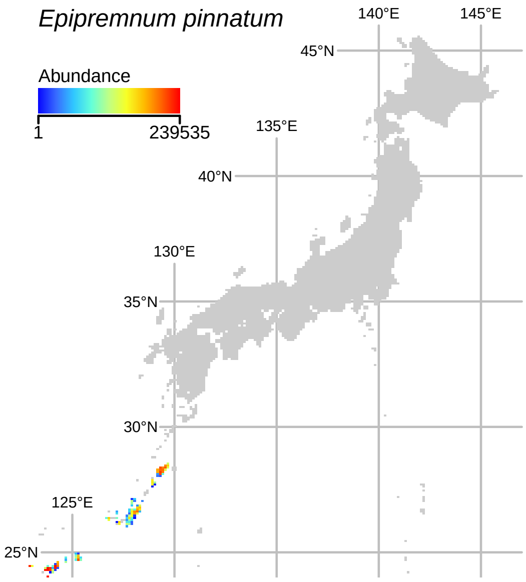
Epipremnum pinnatum
Abundance
1
239535
140°E
145°E
45°N
135°E
40°N
130°E
35°N
30°N
125°E
25°N

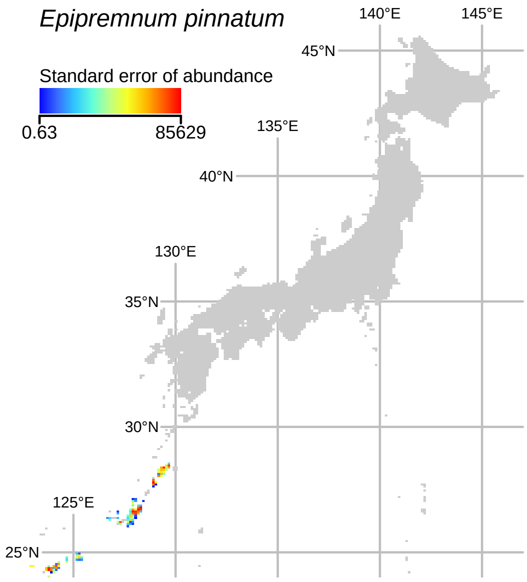
Epipremnum pinnatum
Standard error of abundance
0.63
85629
140°E
145°E
45°N
135°E
40°N
130°E
35°N
30°N
125°E
25°N

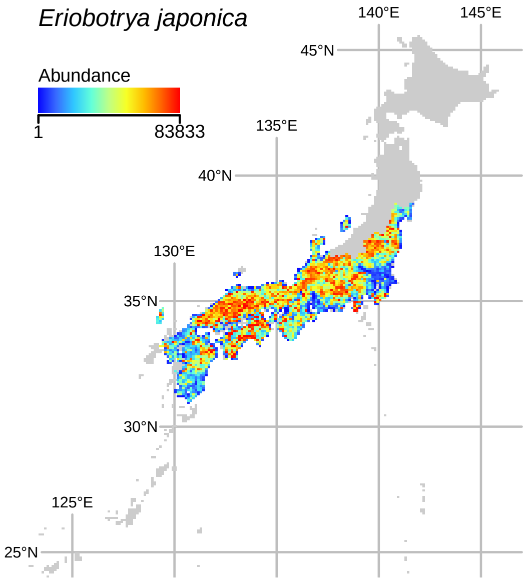
Eriobotrya japonica
Abundance
1
83833
140°E
145°E
45°N
135°E
40°N
130°E
35°N
30°N
125°E
25°N

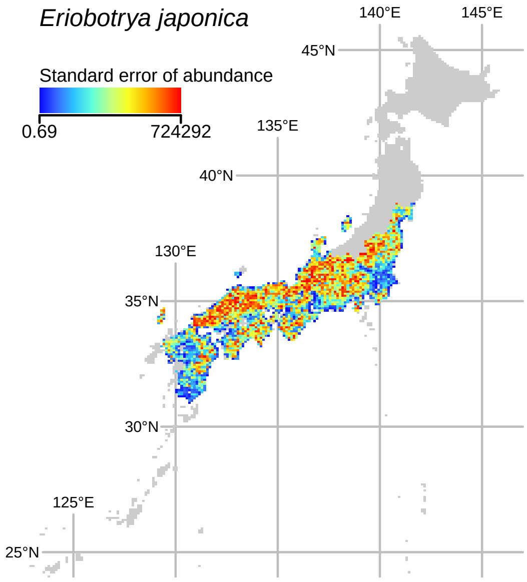
Eriobotrya japonica
Standard error of abundance
0.69
724292
140°E
145°E
45°N
135°E
40°N
130°E
35°N
30°N
125°E
25°N

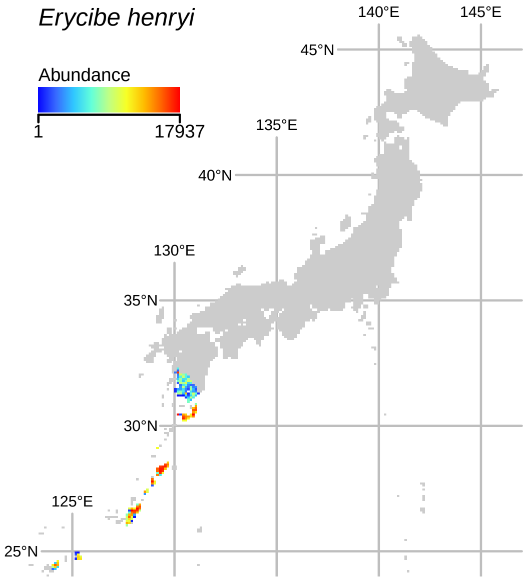
Erycibe henryi
Abundance
1
17937
140°E
145°E
45°N
135°E
40°N
130°E
35°N
30°N
125°E
25°N

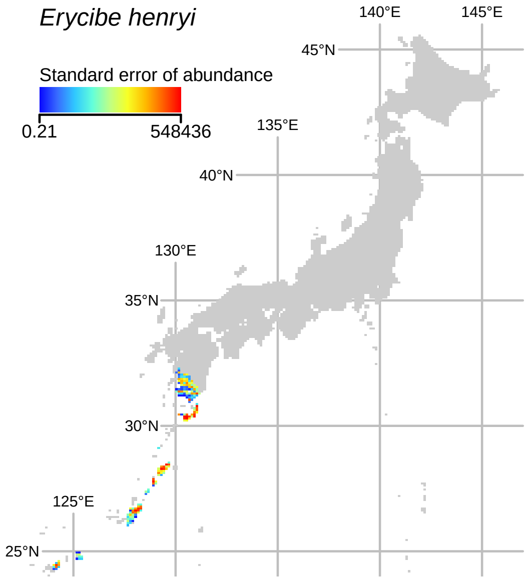
Erycibe henryi
Standard error of abundance
0.21
548436
140°E
145°E
45°N
135°E
40°N
130°E
35°N
30°N
125°E
25°N

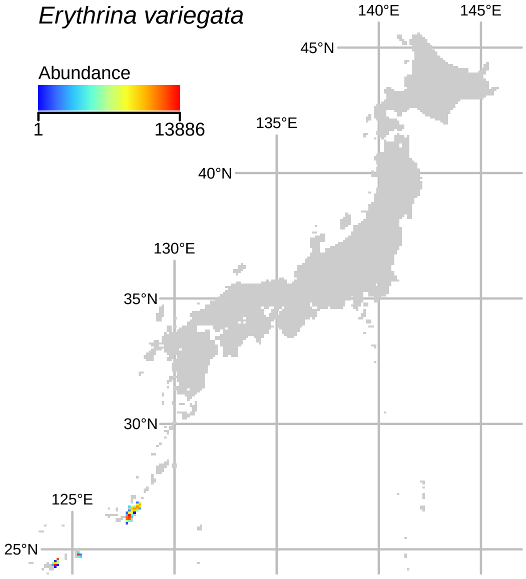
Erythrina variegata
Abundance
1
13886
140°E
145°E
45°N
135°E
40°N
130°E
35°N
30°N
125°E
25°N

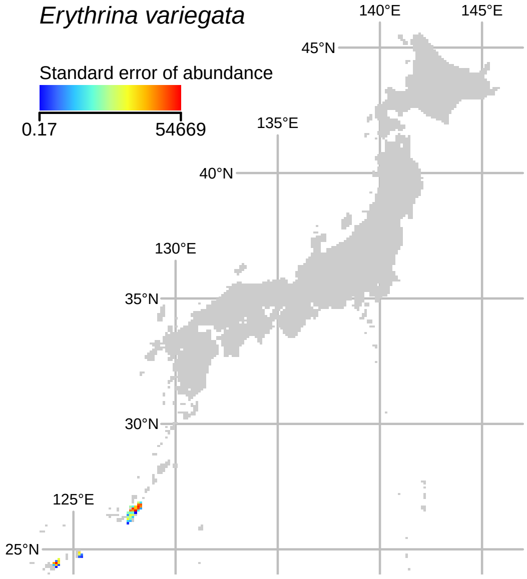
Erythrina variegata
Standard error of abundance
0.17
54669
140°E
145°E
45°N
135°E
40°N
130°E
35°N
30°N
125°E
25°N

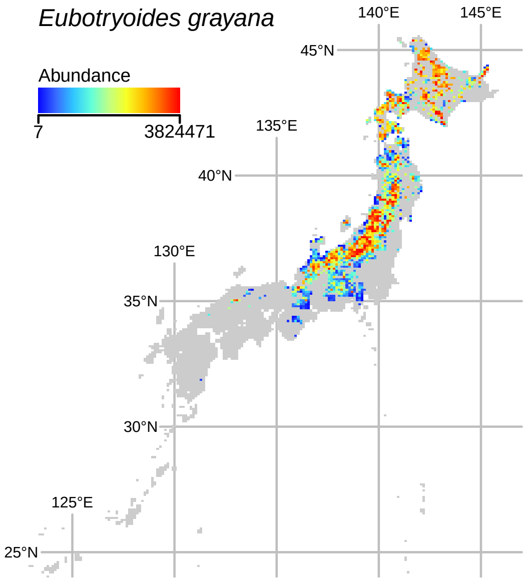
Eubotryoides grayana
Abundance
7
3824471
140°E
145°E
135°E
130°E
125°E
45°N
40°N
35°N
30°N
25°N

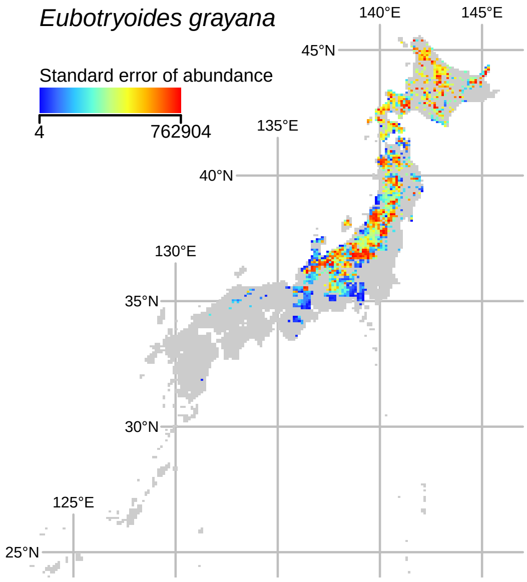
Eubotryoides grayana
Standard error of abundance
4
762904
140°E
145°E
135°E
130°E
125°E
45°N
40°N
35°N
30°N
25°N

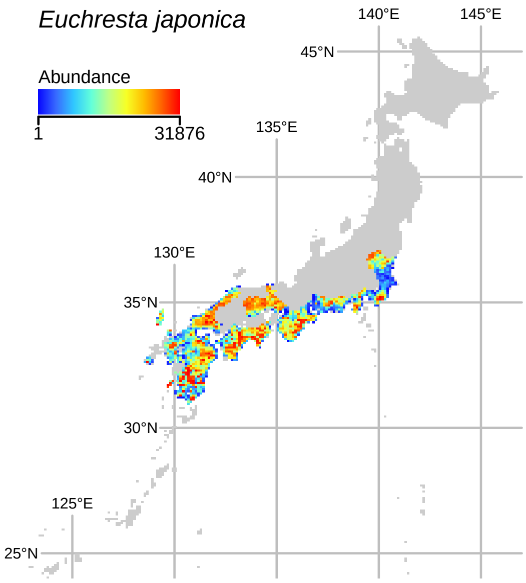
Euchresta japonica
Abundance
1
31876
140°E
145°E
135°E
130°E
125°E
45°N
40°N
35°N
30°N
25°N

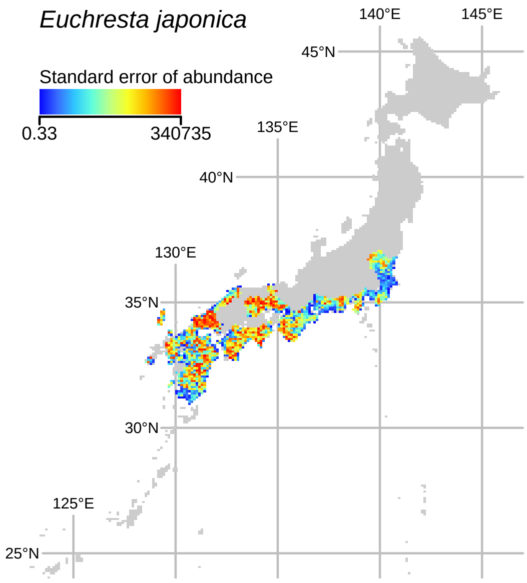
Euchresta japonica
Standard error of abundance
0.33
340735
140°E
145°E
135°E
130°E
125°E
45°N
40°N
35°N
30°N
25°N

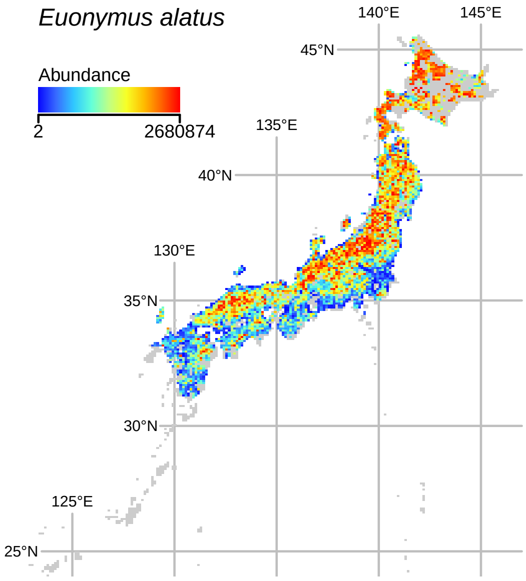
Euonymus alatus
Abundance
2
2680874
140°E
145°E
135°E
130°E
125°E
45°N
40°N
35°N
30°N
25°N

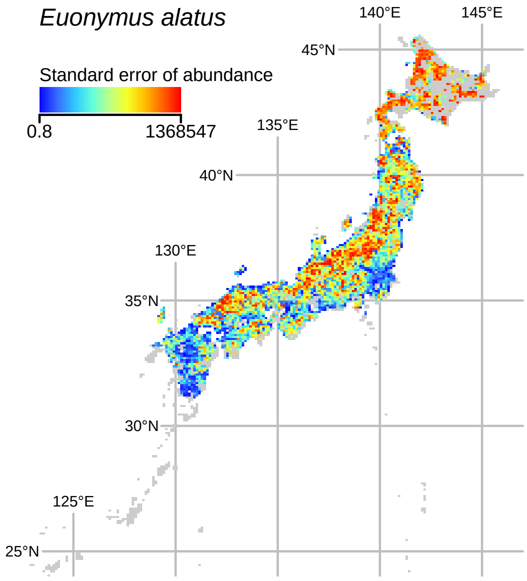
Euonymus alatus
Standard error of abundance
0.8
1368547
140°E
145°E
135°E
130°E
125°E
45°N
40°N
35°N
30°N
25°N

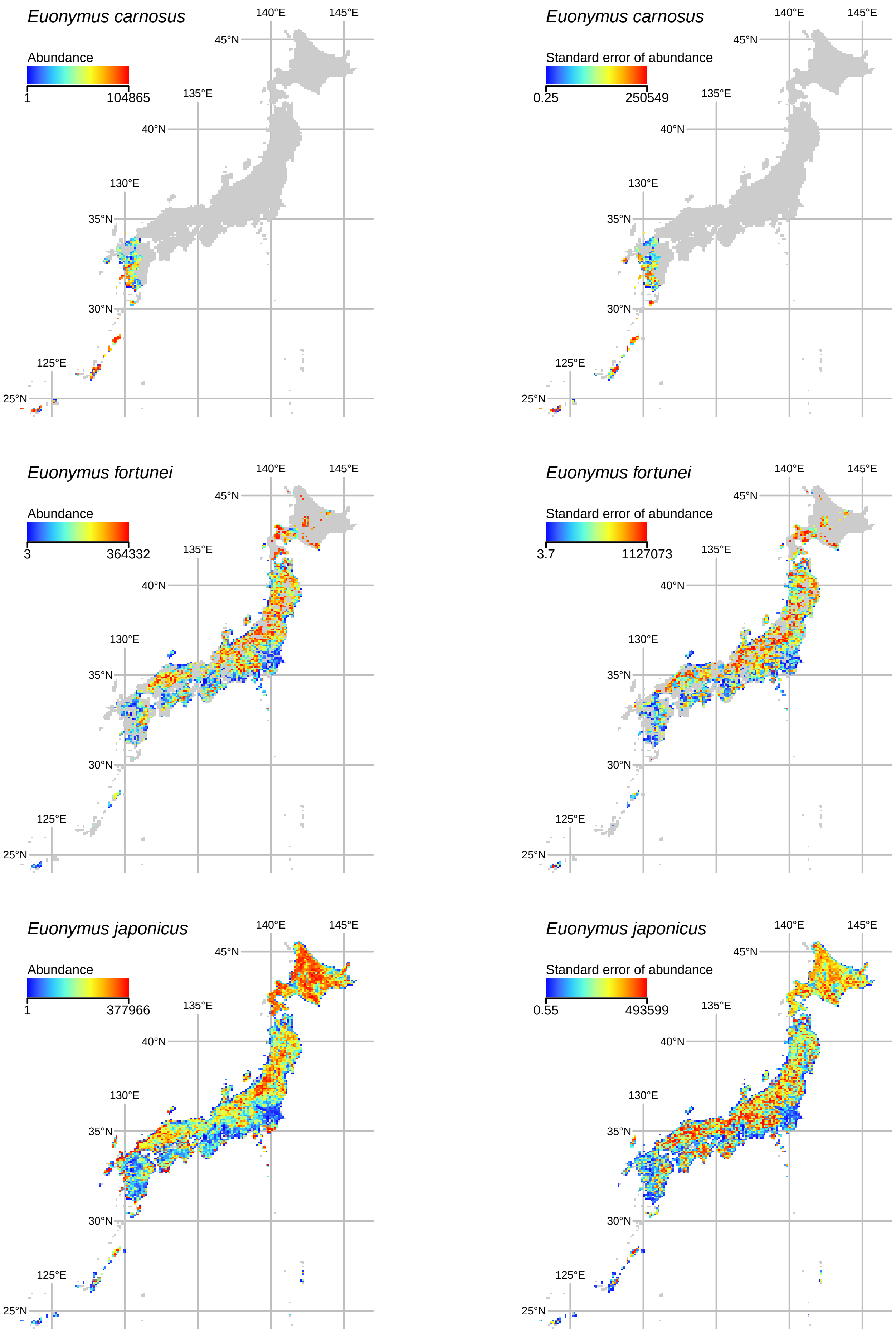
Euonymus carnosus
Abundance
1
104865
Euonymus carnosus
Standard error of abundance
0.25
250549
Euonymus fortunei
Abundance
3
364332
Euonymus fortunei
Standard error of abundance
3.7
1127073
Euonymus japonicus
Abundance
1
377966
Euonymus japonicus
Standard error of abundance
0.55
493599
125°E
130°E
135°E
140°E
145°E
25°N
30°N
35°N
40°N
45°N

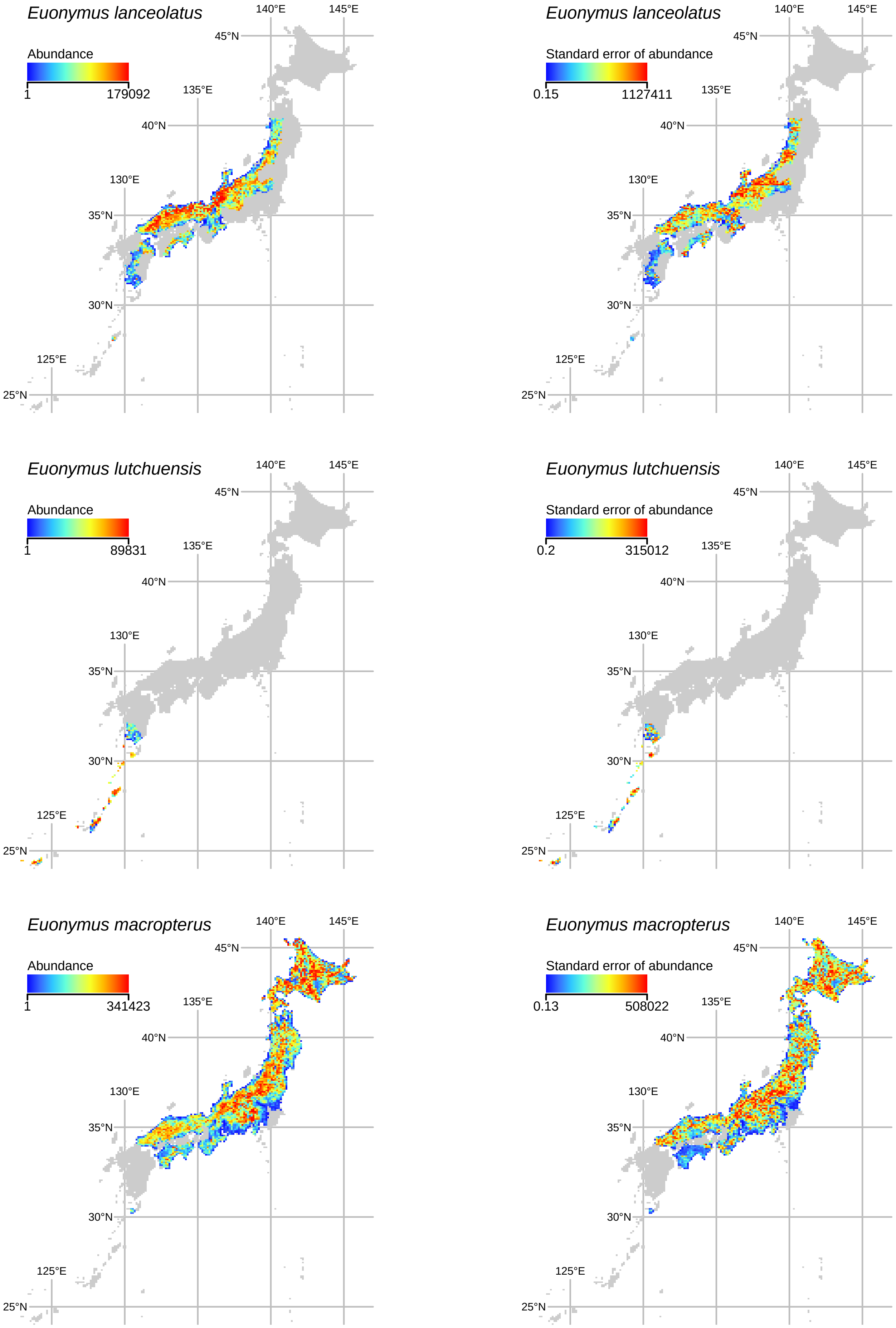
Euonymus lanceolatus
Abundance
1
179092
Euonymus lanceolatus
Standard error of abundance
0.15
1127411
Euonymus lutchuensis
Abundance
1
89831
Euonymus lutchuensis
Standard error of abundance
0.2
315012
Euonymus macropterus
Abundance
1
341423
Euonymus macropterus
Standard error of abundance
0.13
508022
125°E
130°E
135°E
140°E
145°E
25°N
30°N
35°N
40°N
45°N

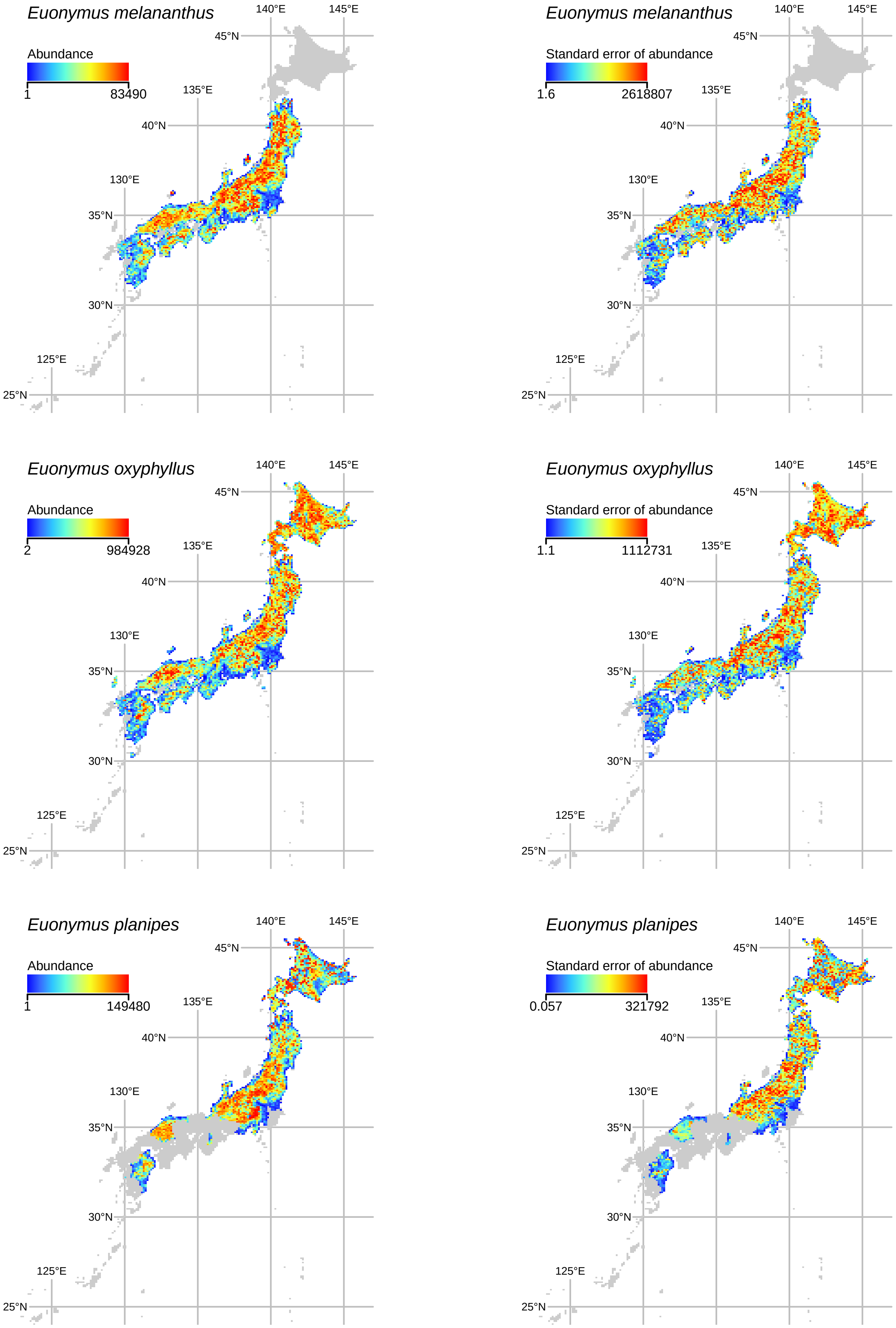
Euonymus melananthus
Abundance
1
83490
Euonymus melananthus
Standard error of abundance
1.6
2618807
Euonymus oxyphyllus
Abundance
2
984928
Euonymus oxyphyllus
Standard error of abundance
1.1
1112731
Euonymus planipes
Abundance
1
149480
Euonymus planipes
Standard error of abundance
0.057
321792
125°E
130°E
135°E
140°E
145°E
25°N
30°N
35°N
40°N
45°N

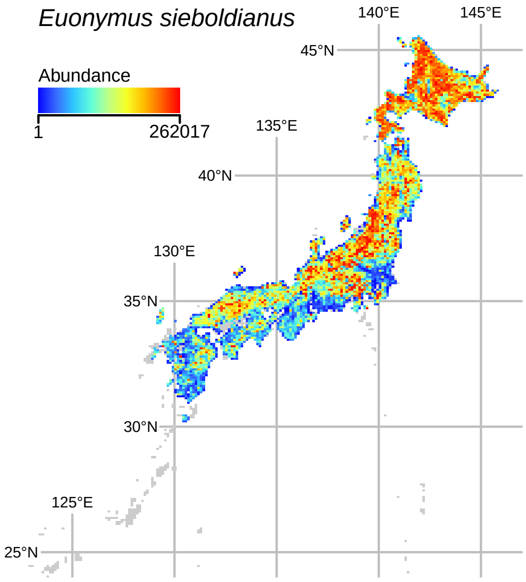
Euonymus sieboldianus
Abundance
1
262017
140°E
145°E
45°N
135°E
40°N
130°E
35°N
30°N
125°E
25°N

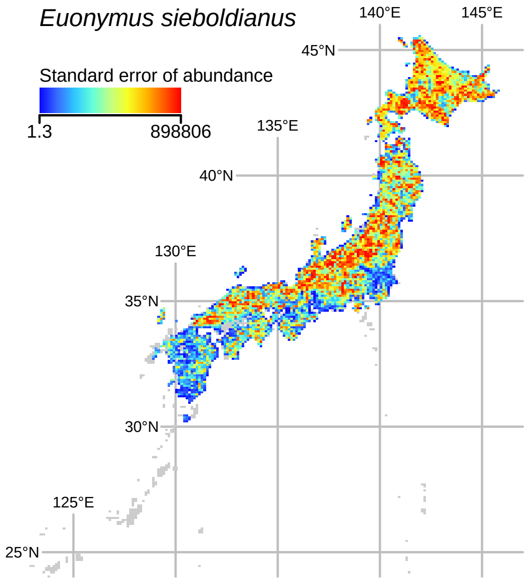
Euonymus sieboldianus
Standard error of abundance
1.3
898806
140°E
145°E
45°N
135°E
40°N
130°E
35°N
30°N
125°E
25°N

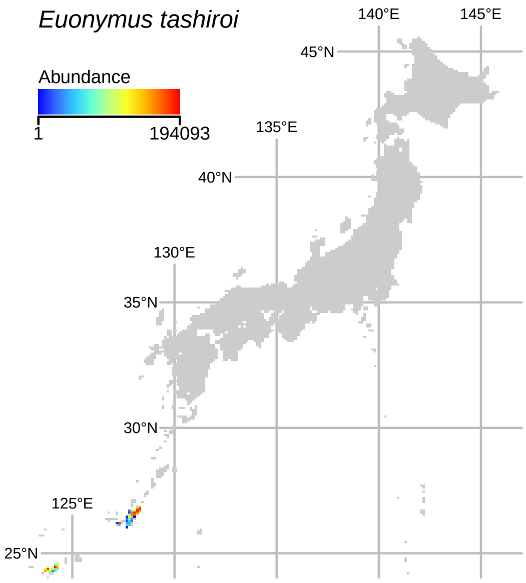
Euonymus tashiroi
Abundance
1
194093
140°E
145°E
45°N
135°E
40°N
130°E
35°N
30°N
125°E
25°N

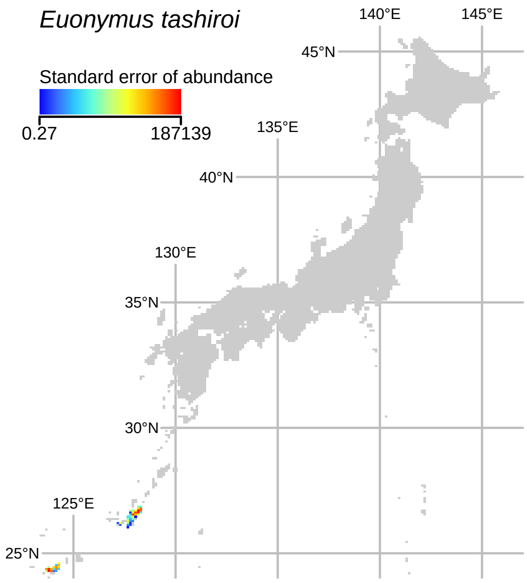
Euonymus tashiroi
Standard error of abundance
0.27
187139
140°E
145°E
45°N
135°E
40°N
130°E
35°N
30°N
125°E
25°N

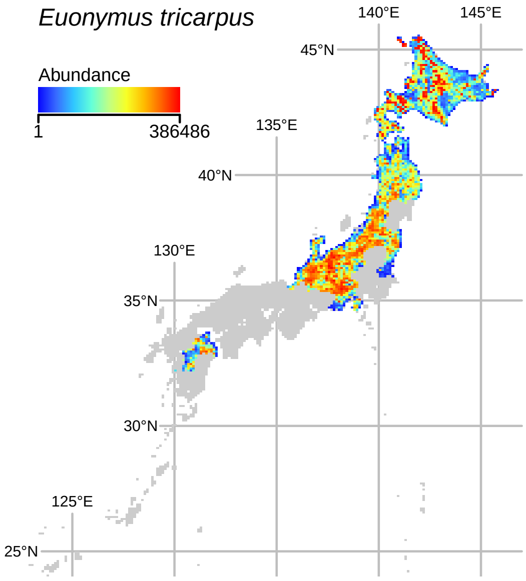
Euonymus tricarpus
Abundance
1
386486
140°E
145°E
45°N
135°E
40°N
130°E
35°N
30°N
125°E
25°N

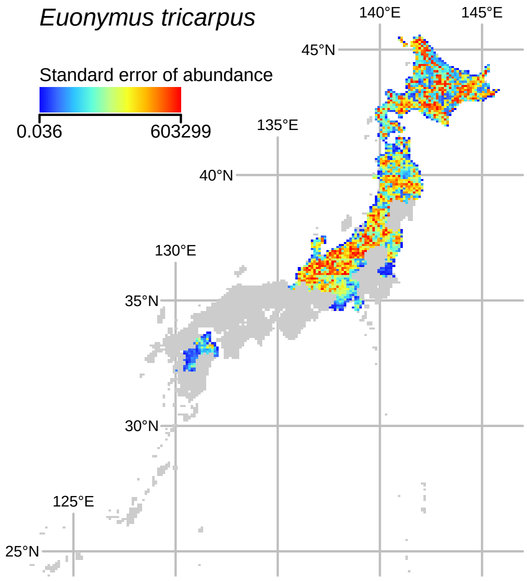
Euonymus tricarpus
Standard error of abundance
0.036
603299
140°E
145°E
45°N
135°E
40°N
130°E
35°N
30°N
125°E
25°N

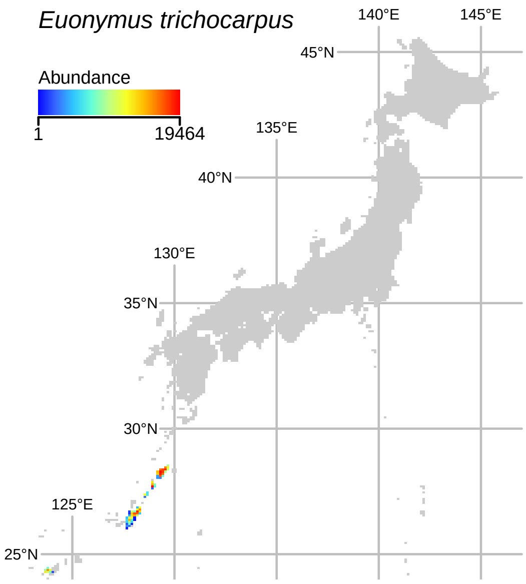
Euonymus trichocarpus
Abundance
1
19464
140°E
145°E
45°N
135°E
40°N
130°E
35°N
30°N
125°E
25°N

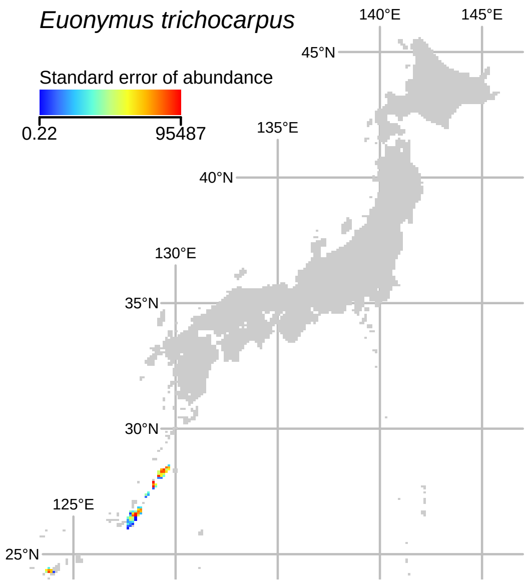
Euonymus trichocarpus
Standard error of abundance
0.22
95487
140°E
145°E
45°N
135°E
40°N
130°E
35°N
30°N
125°E
25°N

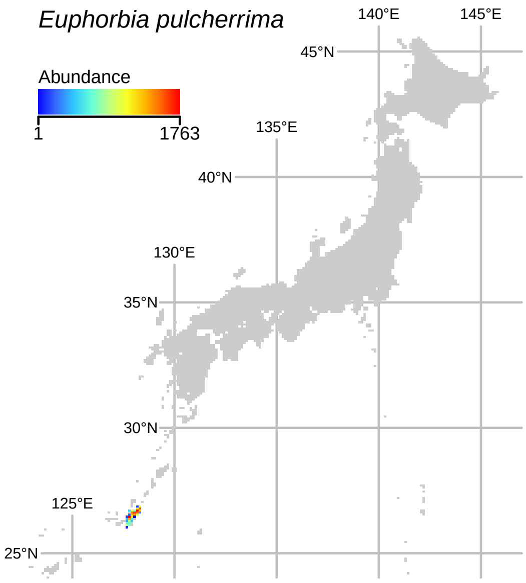
Euphorbia pulcherrima
Abundance
1
1763
140°E
145°E
45°N
135°E
40°N
130°E
35°N
30°N
125°E
25°N

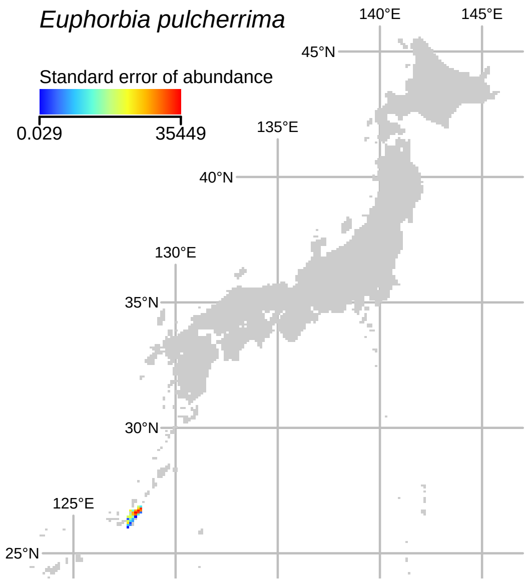
Euphorbia pulcherrima
Standard error of abundance
0.029
35449
140°E
145°E
45°N
135°E
40°N
130°E
35°N
30°N
125°E
25°N

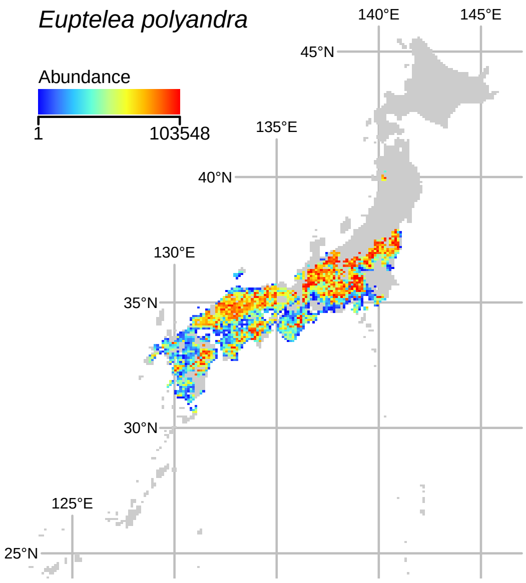
Euptelea polyandra
Abundance
1
103548
140°E
145°E
45°N
135°E
40°N
130°E
35°N
30°N
125°E
25°N

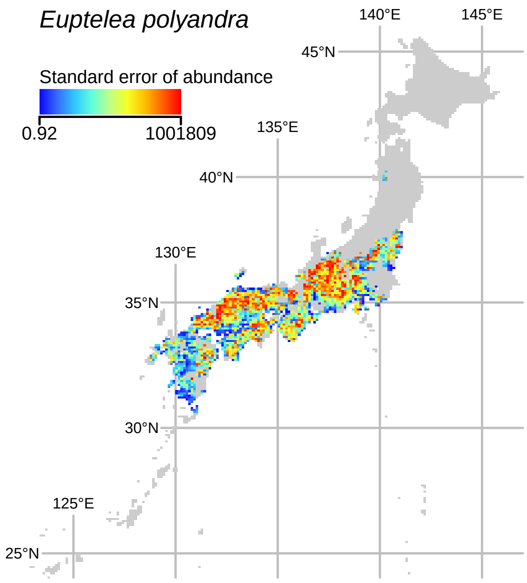
Euptelea polyandra
Standard error of abundance
0.92
1001809
140°E
145°E
45°N
135°E
40°N
130°E
35°N
30°N
125°E
25°N

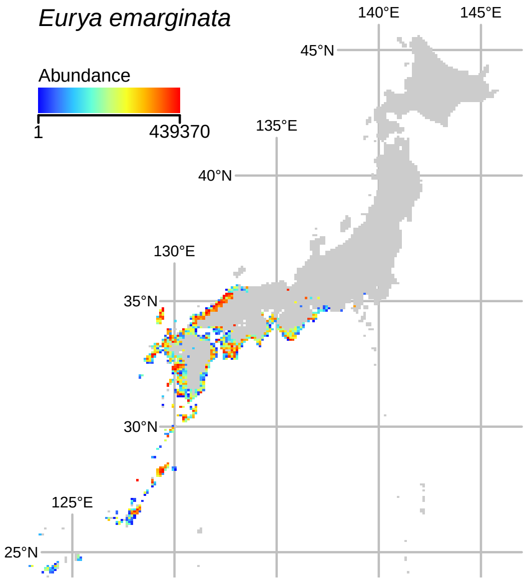
Eurya emarginata
Abundance
1
439370
140°E
145°E
45°N
135°E
40°N
130°E
35°N
30°N
125°E
25°N

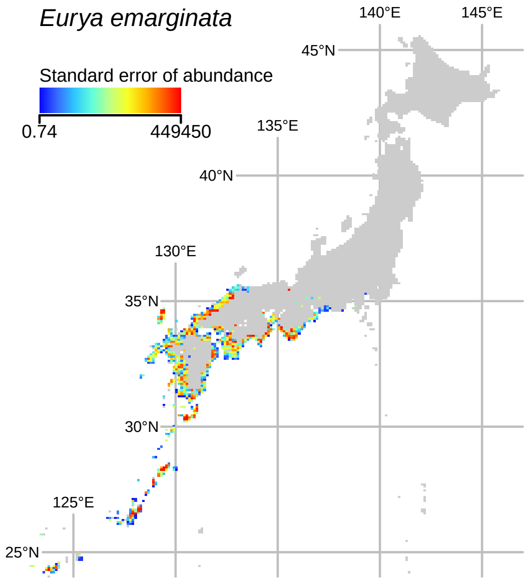
Eurya emarginata
Standard error of abundance
0.74
449450
140°E
145°E
45°N
135°E
40°N
130°E
35°N
30°N
125°E
25°N

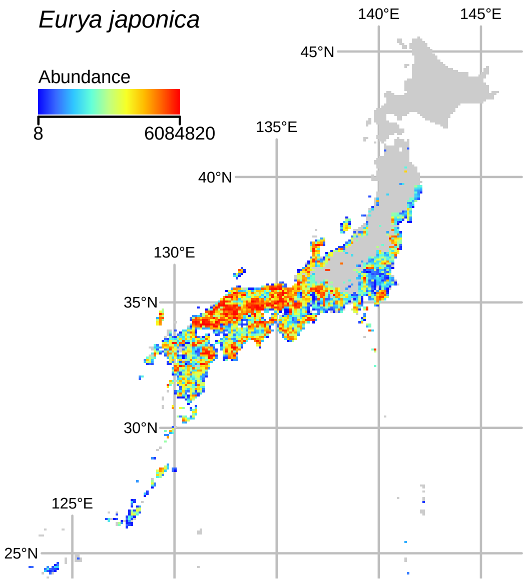
Eurya japonica
Abundance
8
6084820
140°E
145°E
45°N
135°E
40°N
130°E
35°N
30°N
125°E
25°N

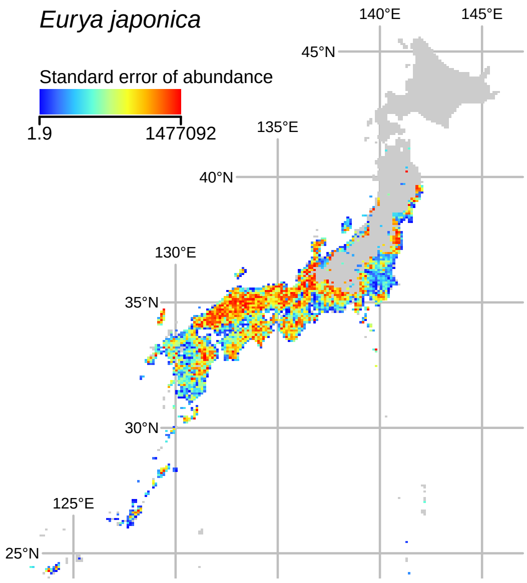
Eurya japonica
Standard error of abundance
1.9
1477092
140°E
145°E
45°N
135°E
40°N
130°E
35°N
30°N
125°E
25°N

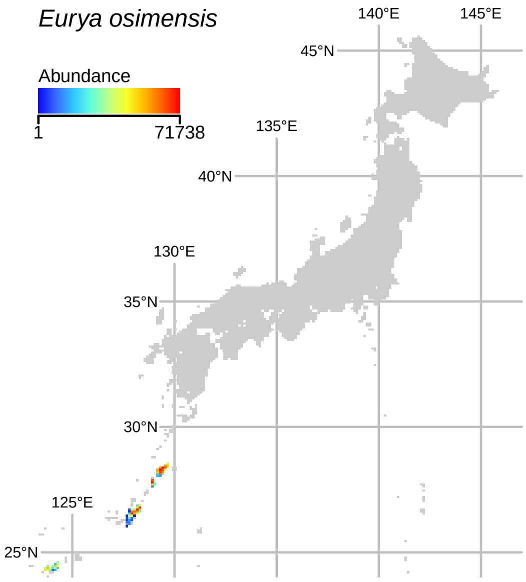
Eurya osimensis
Abundance
1
71738
140°E
145°E
45°N
135°E
40°N
130°E
35°N
30°N
125°E
25°N

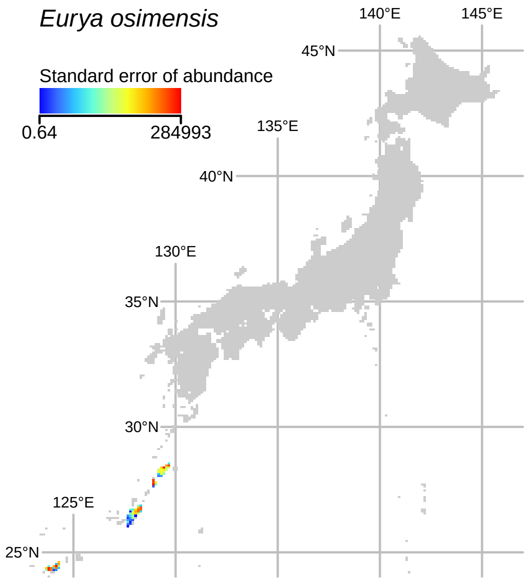
Eurya osimensis
Standard error of abundance
0.64
284993
140°E
145°E
45°N
135°E
40°N
130°E
35°N
30°N
125°E
25°N

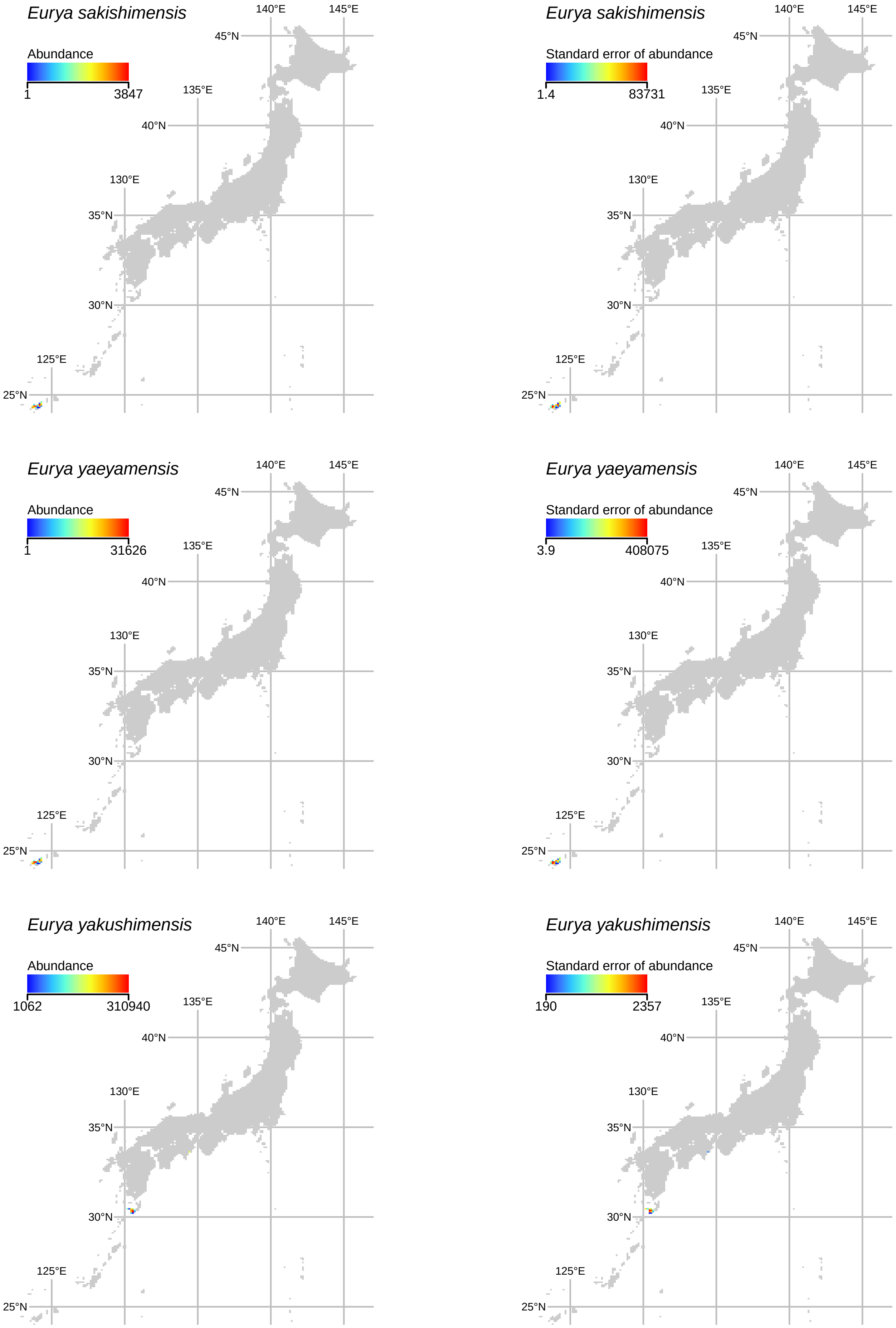
Eurya sakishimensis
Abundance
1
3847
Eurya sakishimensis
Standard error of abundance
1.4
83731
Eurya yaeyamensis
Abundance
1
31626
Eurya yaeyamensis
Standard error of abundance
3.9
408075
Eurya yakushimensis
Abundance
1062
310940
Eurya yakushimensis
Standard error of abundance
190
2357
125°E
130°E
135°E
140°E
145°E
25°N
30°N
35°N
40°N
45°N

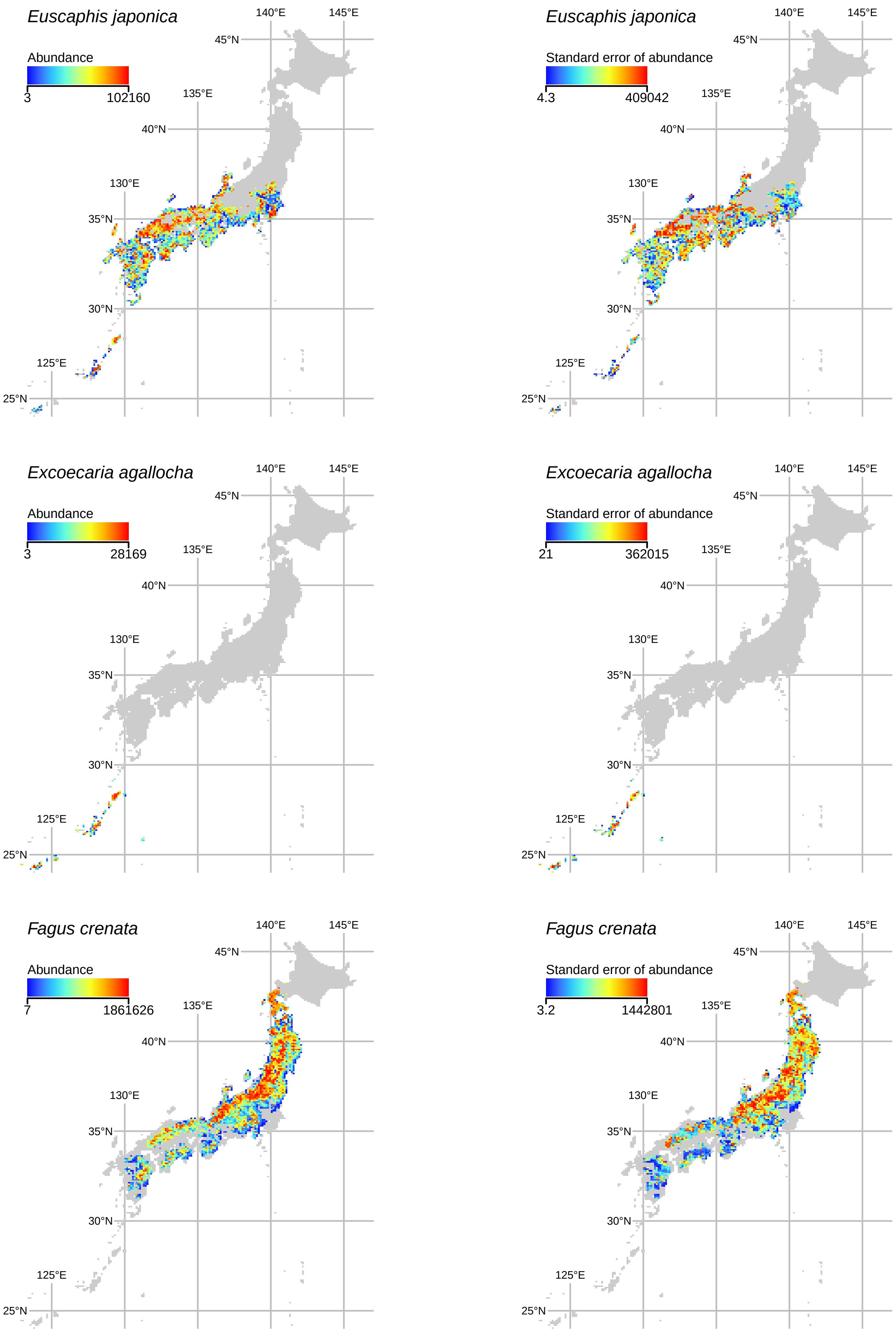
Euscaphis japonica
Abundance
3
102160
Euscaphis japonica
Standard error of abundance
4.3
409042
Excoecaria agallocha
Abundance
3
28169
Excoecaria agallocha
Standard error of abundance
21
362015
Fagus crenata
Abundance
7
1861626
Fagus crenata
Standard error of abundance
3.2
1442801
125°E
130°E
135°E
140°E
145°E
25°N
30°N
35°N
40°N
45°N

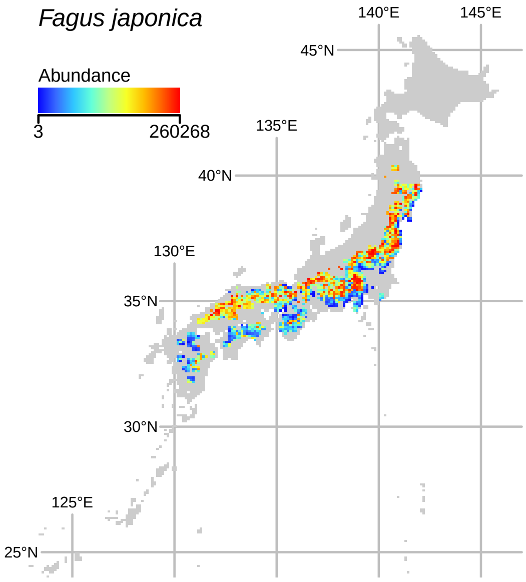
Fagus japonica
Abundance
3
260268
140°E
145°E
45°N
135°E
40°N
130°E
35°N
30°N
125°E
25°N

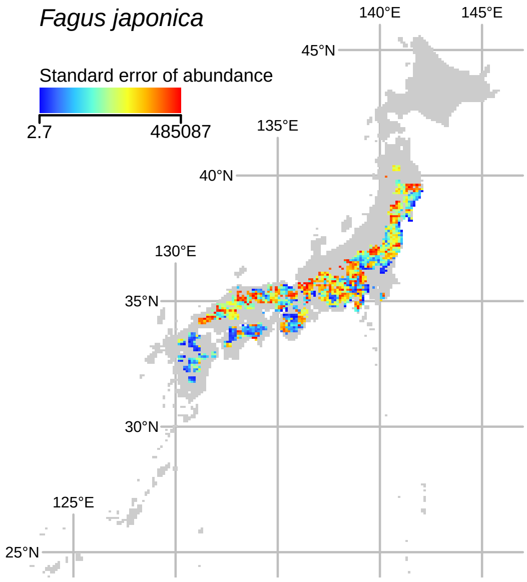
Fagus japonica
Standard error of abundance
2.7
485087
140°E
145°E
45°N
135°E
40°N
130°E
35°N
30°N
125°E
25°N

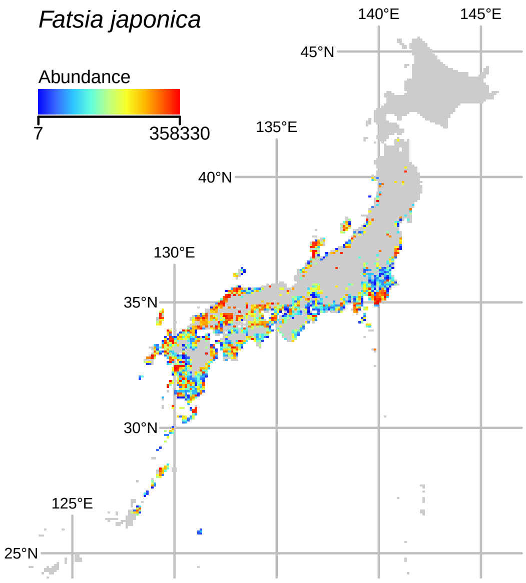
Fatsia japonica
Abundance
7
358330
140°E
145°E
45°N
135°E
40°N
130°E
35°N
30°N
125°E
25°N

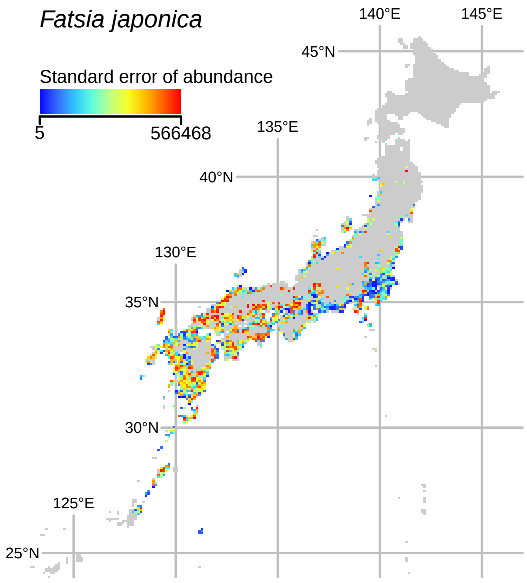
Fatsia japonica
Standard error of abundance
5
566468
140°E
145°E
45°N
135°E
40°N
130°E
35°N
30°N
125°E
25°N

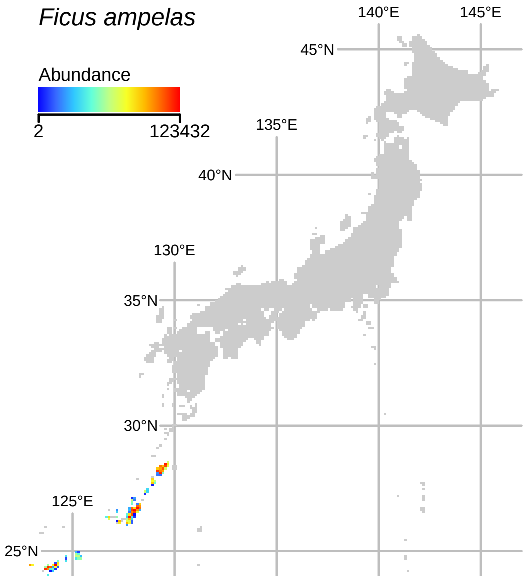
Ficus ampelas
Abundance
2
123432
140°E
145°E
45°N
135°E
40°N
130°E
35°N
30°N
125°E
25°N

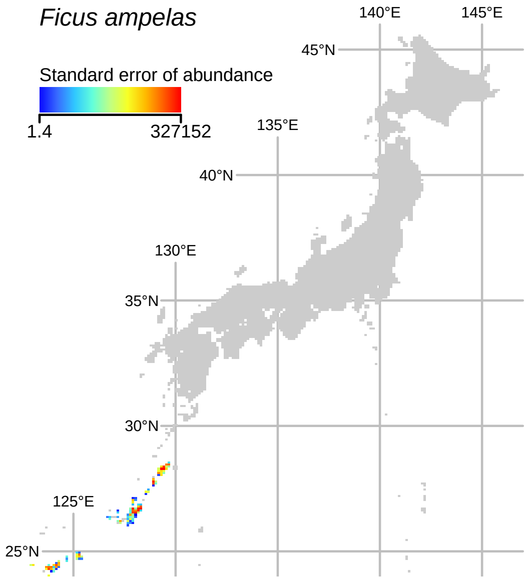
Ficus ampelas
Standard error of abundance
1.4
327152
140°E
145°E
45°N
135°E
40°N
130°E
35°N
30°N
125°E
25°N

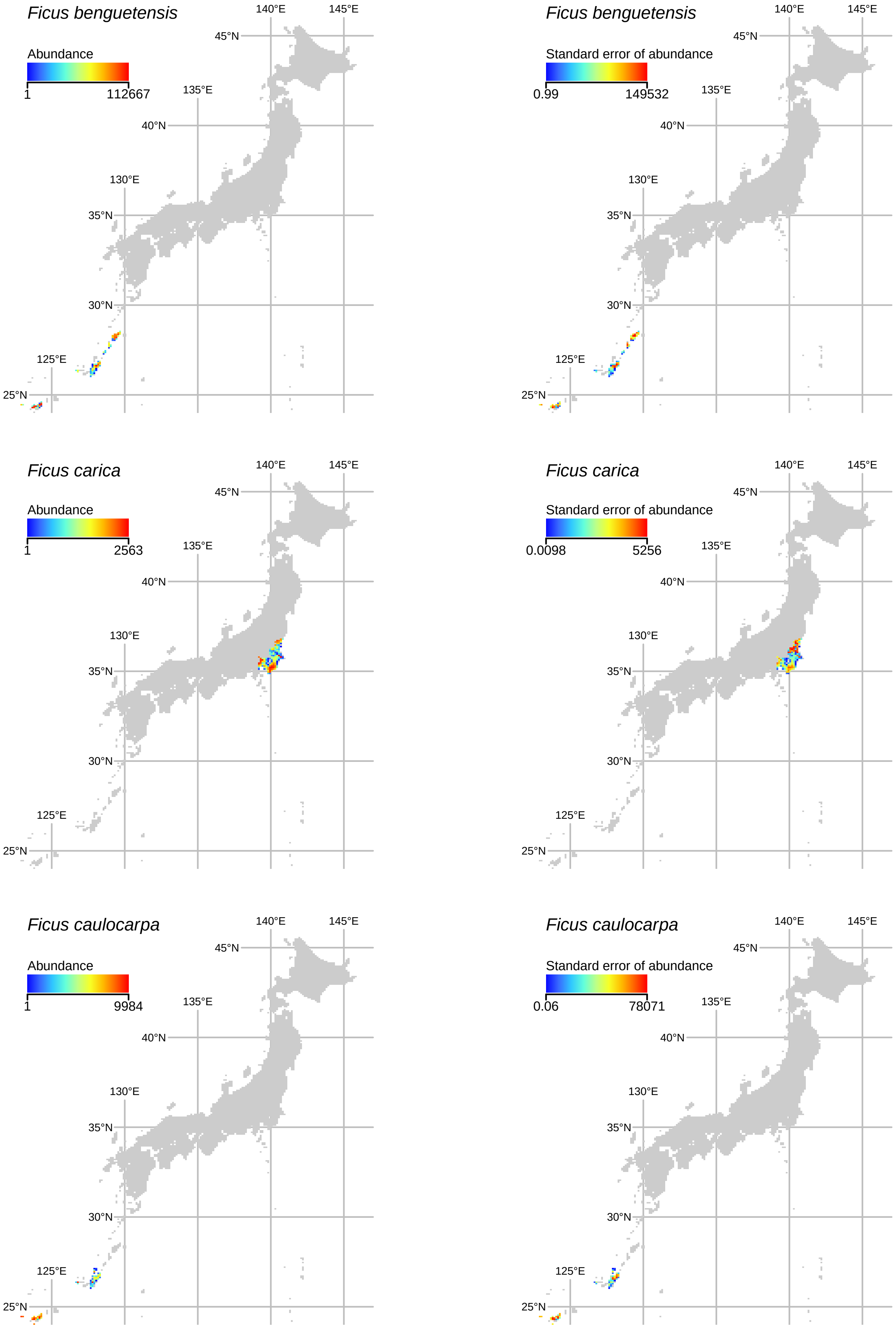
Ficus benguetensis
Abundance
1
112667
Ficus benguetensis
Standard error of abundance
0.99
149532
Ficus carica
Abundance
1
2563
Ficus carica
Standard error of abundance
0.0098
5256
Ficus caulocarpa
Abundance
1
9984
Ficus caulocarpa
Standard error of abundance
0.06
78071
125°E
130°E
135°E
140°E
145°E
25°N
30°N
35°N
40°N
45°N

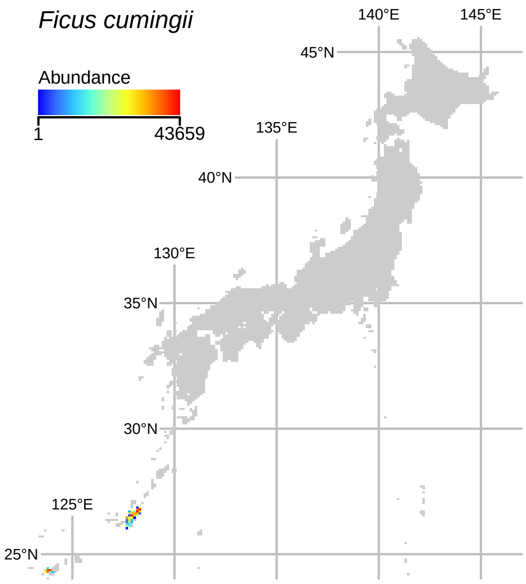
Ficus cumingii
Abundance
1
43659
140°E
145°E
45°N
135°E
40°N
130°E
35°N
30°N
125°E
25°N

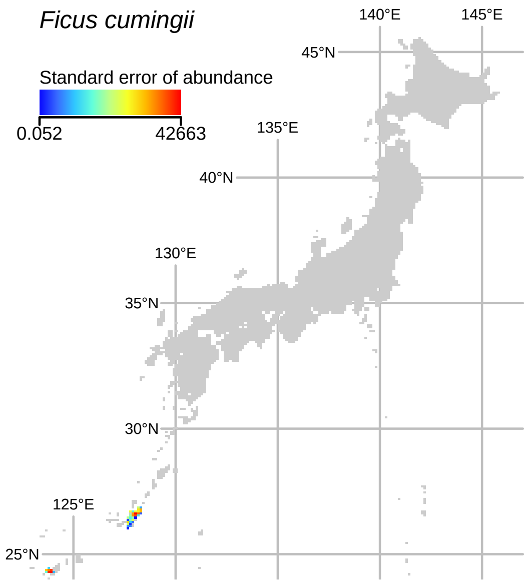
Ficus cumingii
Standard error of abundance
0.052
42663
140°E
145°E
45°N
135°E
40°N
130°E
35°N
30°N
125°E
25°N

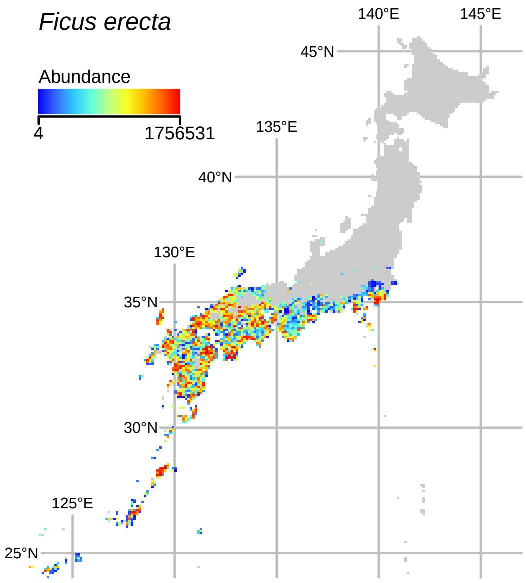
Ficus erecta
Abundance
4
1756531
140°E
145°E
45°N
135°E
40°N
130°E
35°N
30°N
125°E
25°N

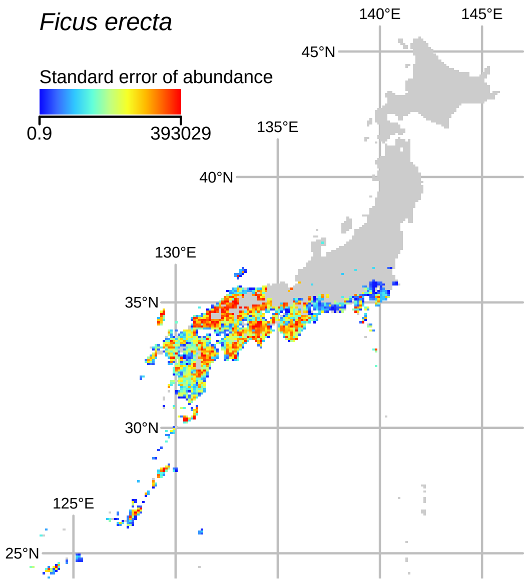
Ficus erecta
Standard error of abundance
0.9
393029
140°E
145°E
45°N
135°E
40°N
130°E
35°N
30°N
125°E
25°N

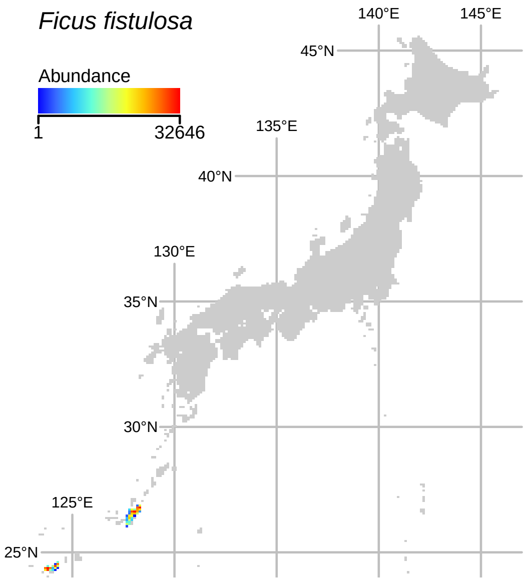
Ficus fistulosa
Abundance
1
32646
140°E
145°E
45°N
135°E
40°N
130°E
35°N
30°N
125°E
25°N

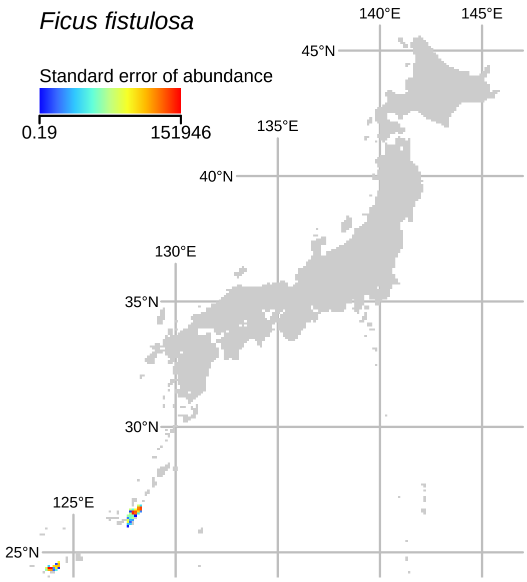
Ficus fistulosa
Standard error of abundance
0.19
151946
140°E
145°E
45°N
135°E
40°N
130°E
35°N
30°N
125°E
25°N

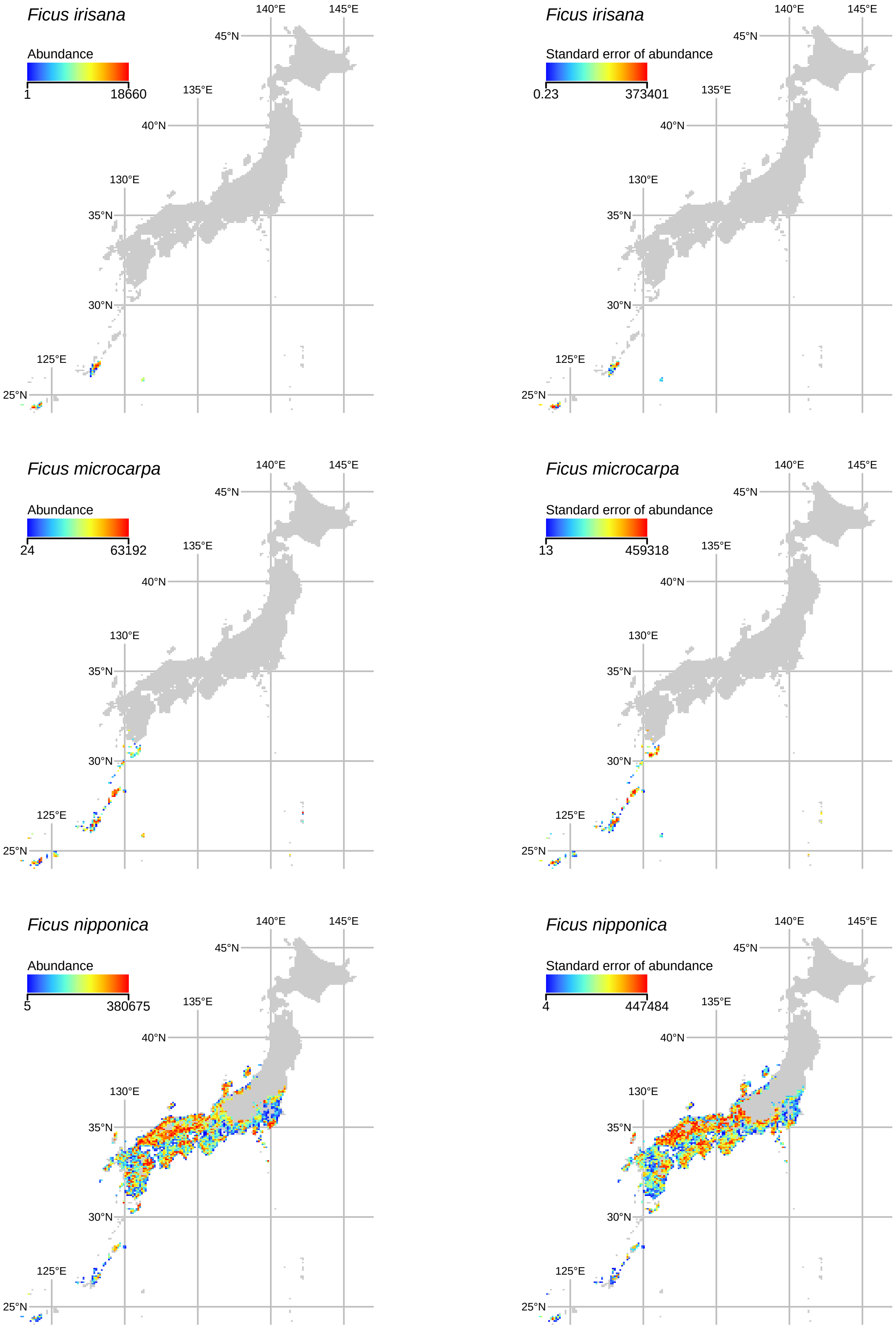
Ficus irisana
Abundance
1
18660
Ficus irisana
Standard error of abundance
0.23
373401
Ficus microcarpa
Abundance
24
63192
Ficus microcarpa
Standard error of abundance
13
459318
Ficus nipponica
Abundance
5
380675
Ficus nipponica
Standard error of abundance
4
447484
125°E
130°E
135°E
140°E
145°E
25°N
30°N
35°N
40°N
45°N

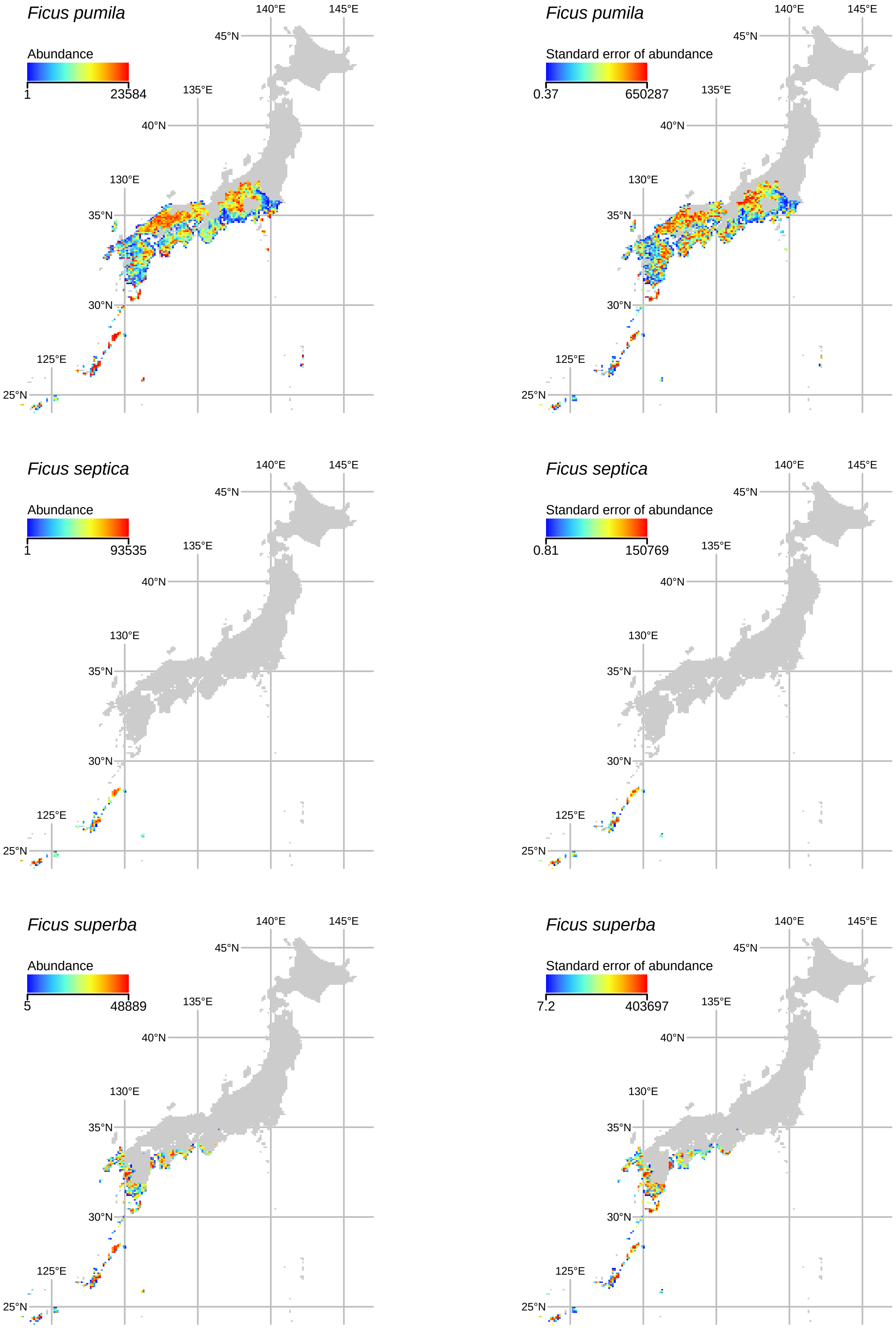
Ficus pumila
Abundance
1
23584
Ficus pumila
Standard error of abundance
0.37
650287
Ficus septica
Abundance
1
93535
Ficus septica
Standard error of abundance
0.81
150769
Ficus superba
Abundance
5
48889
Ficus superba
Standard error of abundance
7.2
403697
125°E
130°E
135°E
140°E
145°E
25°N
30°N
35°N
40°N
45°N

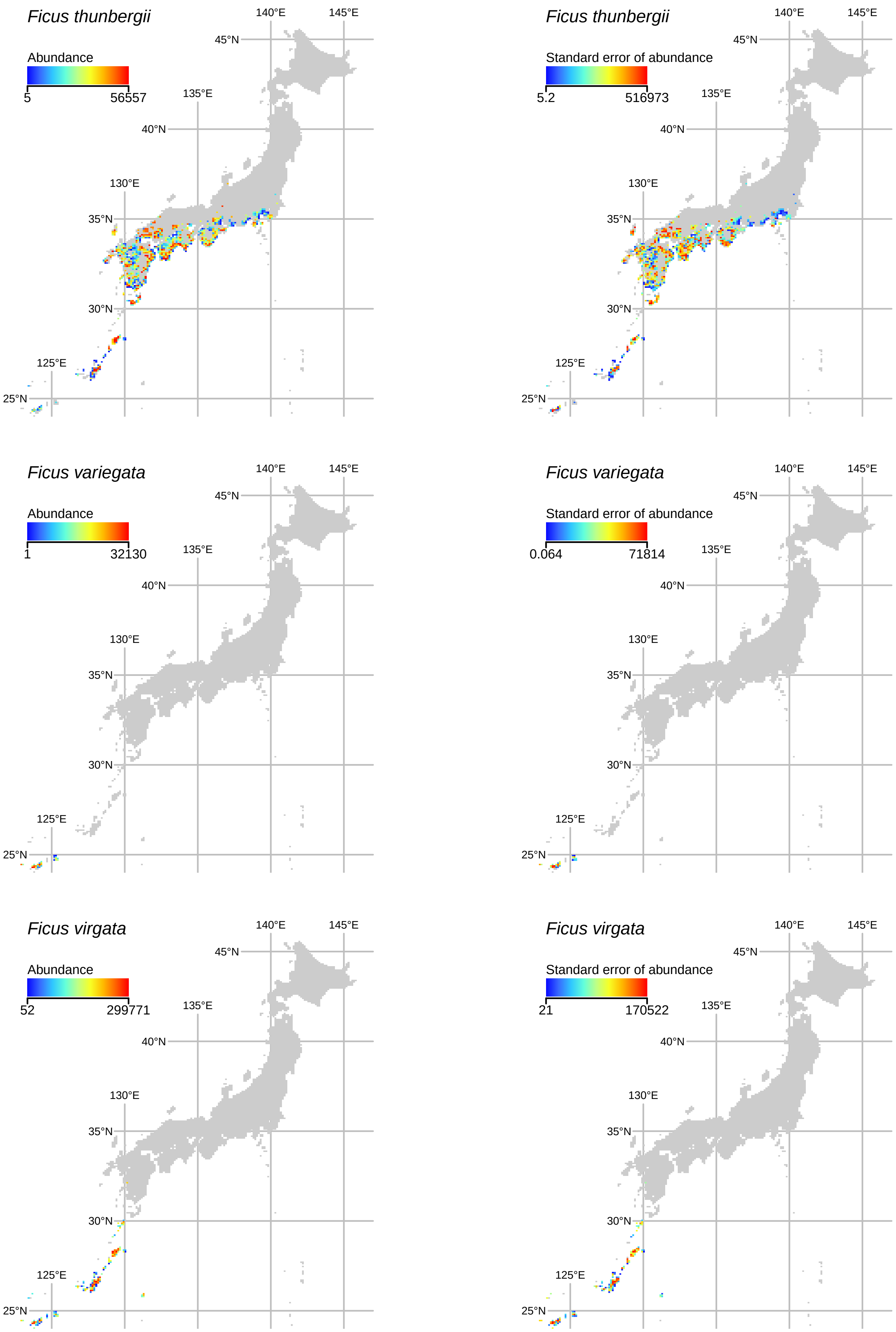
Ficus thunbergii
Abundance
5
56557
Ficus thunbergii
Standard error of abundance
5.2
516973
Ficus variegata
Abundance
1
32130
Ficus variegata
Standard error of abundance
0.064
71814
Ficus virgata
Abundance
52
299771
Ficus virgata
Standard error of abundance
21
170522
125°E
130°E
135°E
140°E
145°E
25°N
30°N
35°N
40°N
45°N

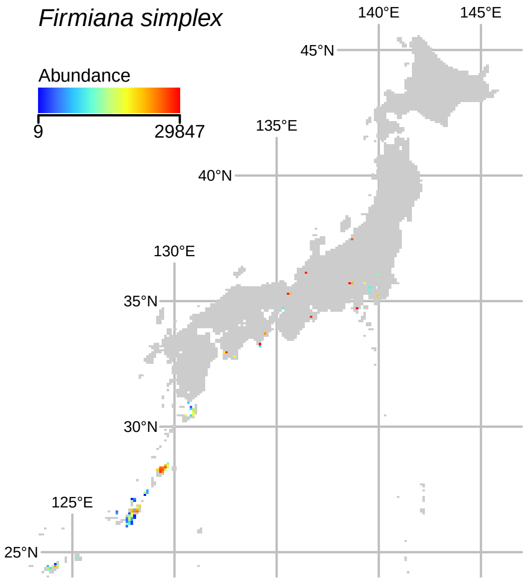
Firmiana simplex
Abundance
9
29847
140°E
145°E
45°N
135°E
40°N
130°E
35°N
30°N
125°E
25°N

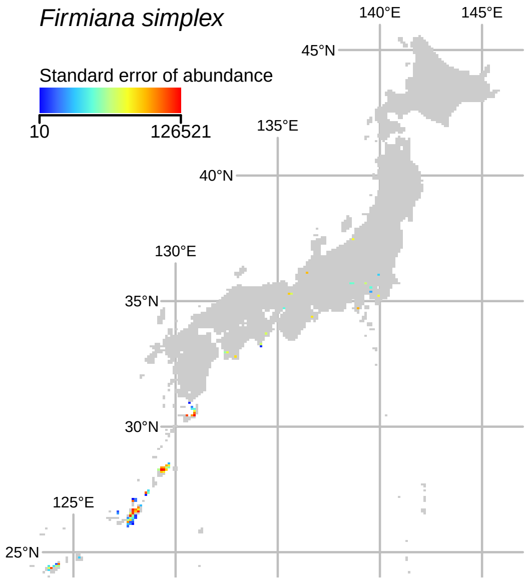
Firmiana simplex
Standard error of abundance
10
126521
140°E
145°E
45°N
135°E
40°N
130°E
35°N
30°N
125°E
25°N

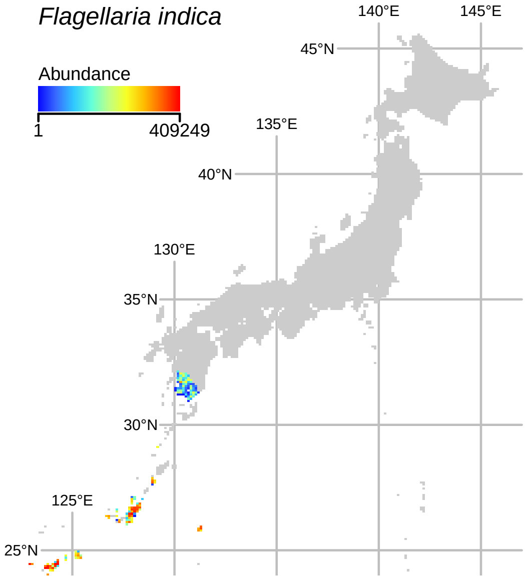
Flagellaria indica
Abundance
1
409249
140°E
145°E
45°N
135°E
40°N
130°E
35°N
30°N
125°E
25°N

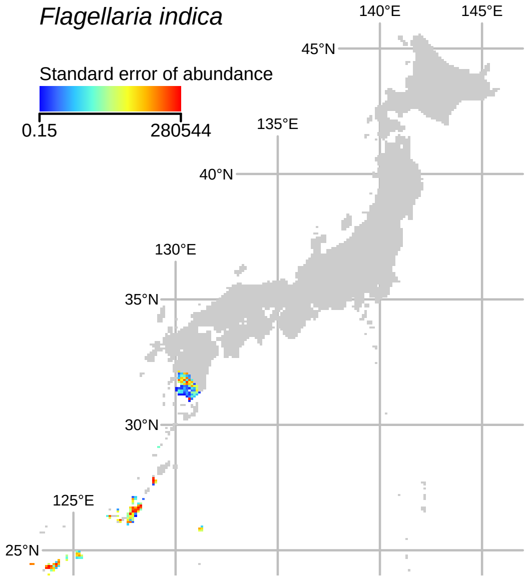
Flagellaria indica
Standard error of abundance
0.15
280544
140°E
145°E
45°N
135°E
40°N
130°E
35°N
30°N
125°E
25°N

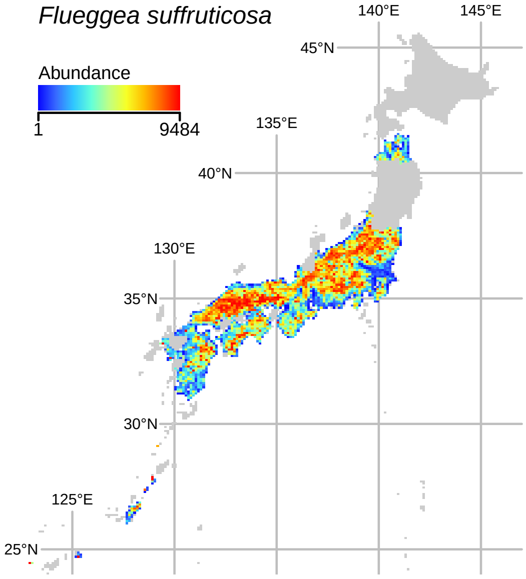
Flueggea suffruticosa
Abundance
1
9484
140°E
145°E
45°N
135°E
40°N
130°E
35°N
30°N
125°E
25°N

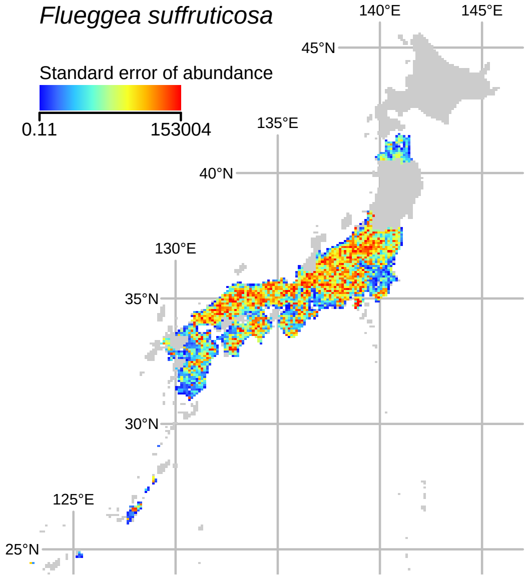
Flueggea suffruticosa
Standard error of abundance
0.11
153004
140°E
145°E
45°N
135°E
40°N
130°E
35°N
30°N
125°E
25°N

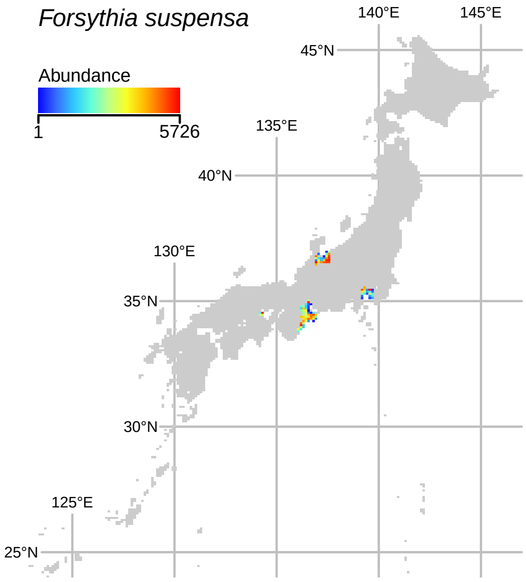
Forsythia suspensa
Abundance
1
5726
140°E
145°E
45°N
135°E
40°N
130°E
35°N
30°N
125°E
25°N

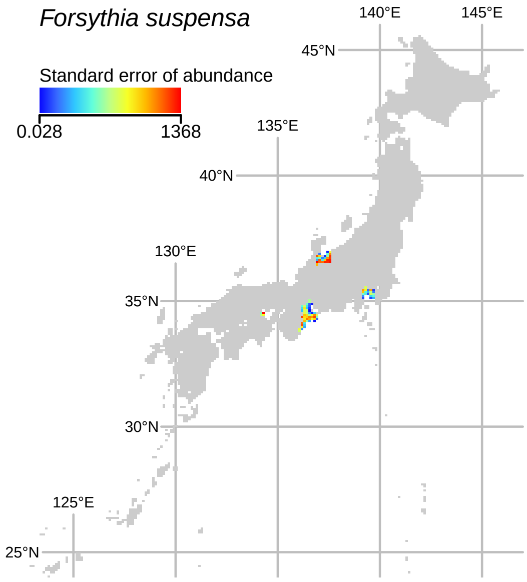
Forsythia suspensa
Standard error of abundance
0.028
1368
140°E
145°E
45°N
135°E
40°N
130°E
35°N
30°N
125°E
25°N

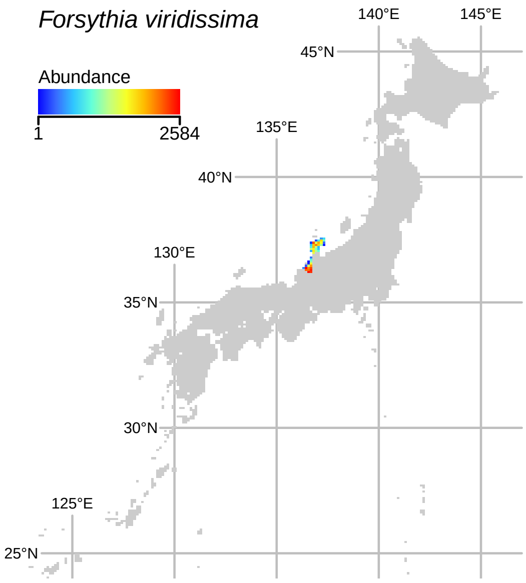
Forsythia viridissima
Abundance
1
2584
140°E
145°E
45°N
135°E
40°N
130°E
35°N
30°N
125°E
25°N

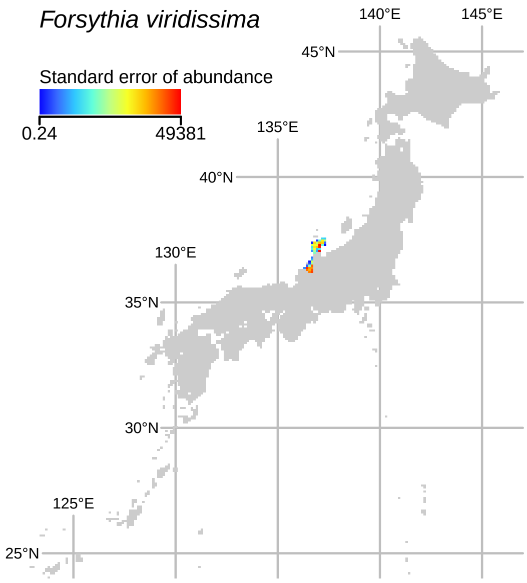
Forsythia viridissima
Standard error of abundance
0.24
49381
140°E
145°E
45°N
135°E
40°N
130°E
35°N
30°N
125°E
25°N

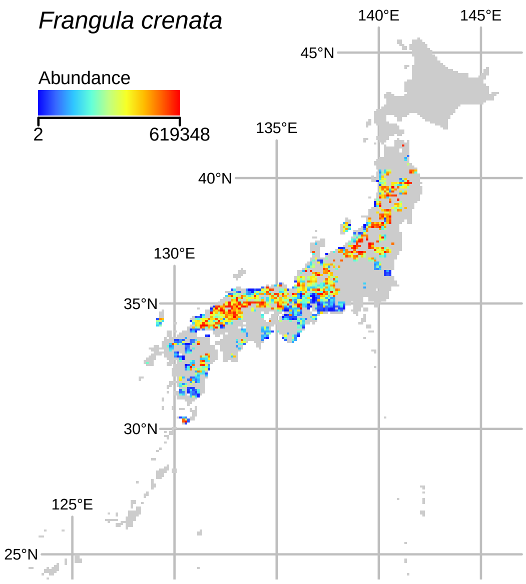
Frangula crenata
Abundance
2
619348
140°E
145°E
45°N
135°E
40°N
130°E
35°N
30°N
125°E
25°N

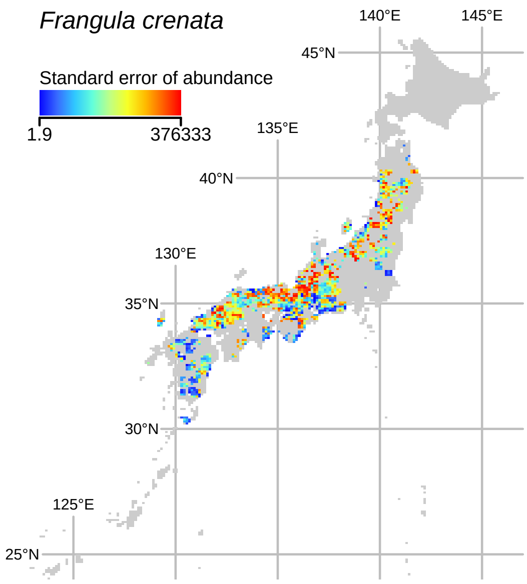
Frangula crenata
Standard error of abundance
1.9
376333
140°E
145°E
45°N
135°E
40°N
130°E
35°N
30°N
125°E
25°N

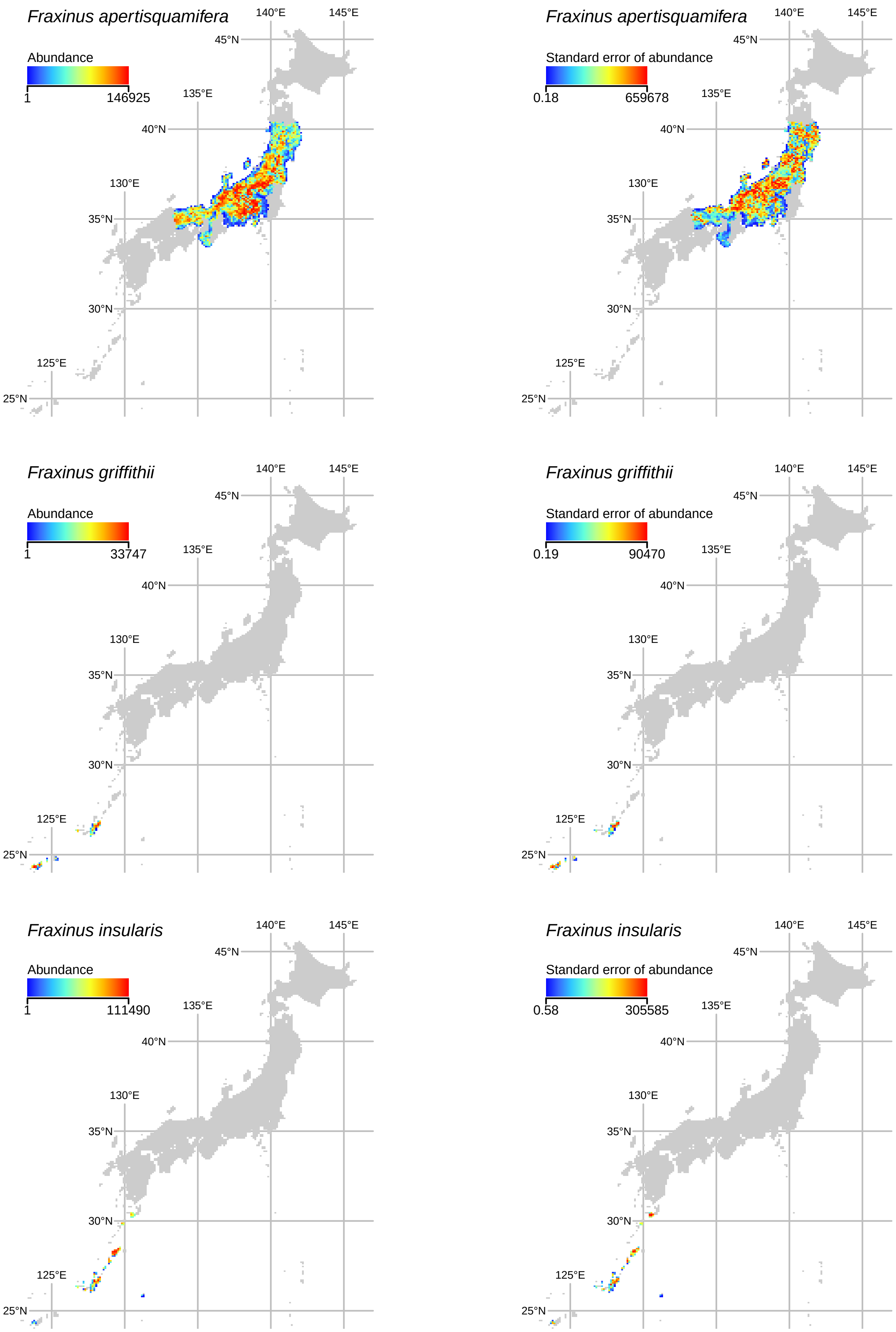
Fraxinus apertisquamifera
Abundance
1
146925
Fraxinus apertisquamifera
Standard error of abundance
0.18
659678
Fraxinus griffithii
Abundance
1
33747
Fraxinus griffithii
Standard error of abundance
0.19
90470
Fraxinus insularis
Abundance
1
111490
Fraxinus insularis
Standard error of abundance
0.58
305585
140°E
145°E
45°N
135°E
40°N
130°E
35°N
30°N
125°E
25°N

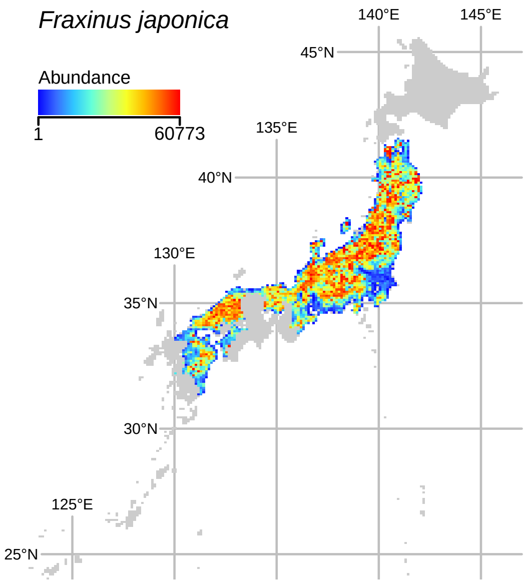
Fraxinus japonica
Abundance
1
60773
140°E
145°E
45°N
135°E
40°N
130°E
35°N
30°N
125°E
25°N

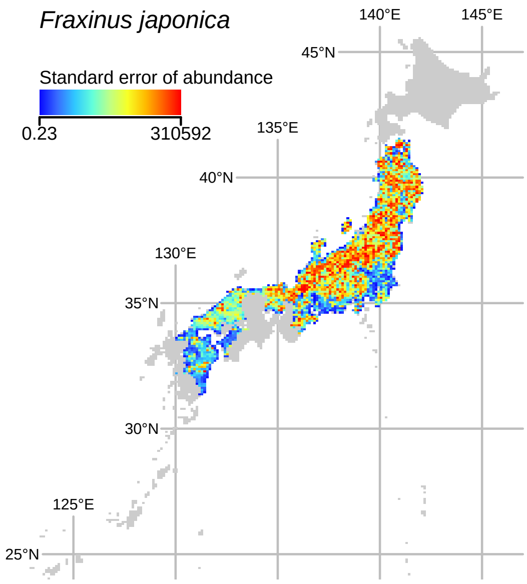
Fraxinus japonica
Standard error of abundance
0.23
310592
140°E
145°E
45°N
135°E
40°N
130°E
35°N
30°N
125°E
25°N

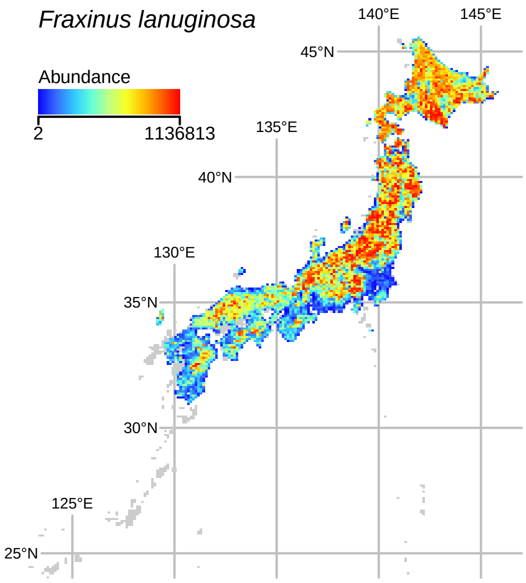
Fraxinus lanuginosa
Abundance
2
1136813
140°E
145°E
45°N
135°E
40°N
130°E
35°N
30°N
125°E
25°N

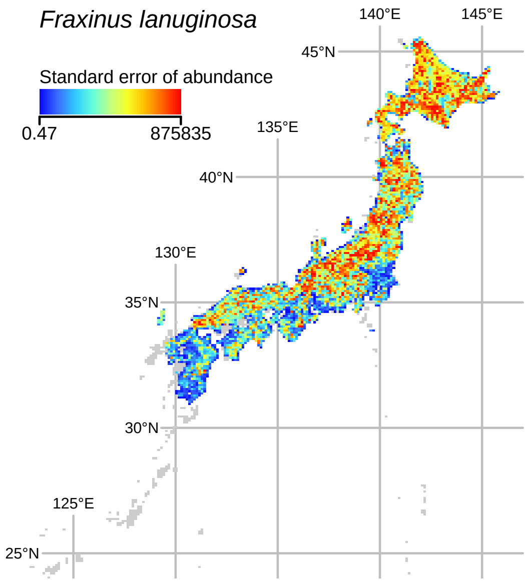
Fraxinus lanuginosa
Standard error of abundance
0.47
875835
140°E
145°E
45°N
135°E
40°N
130°E
35°N
30°N
125°E
25°N

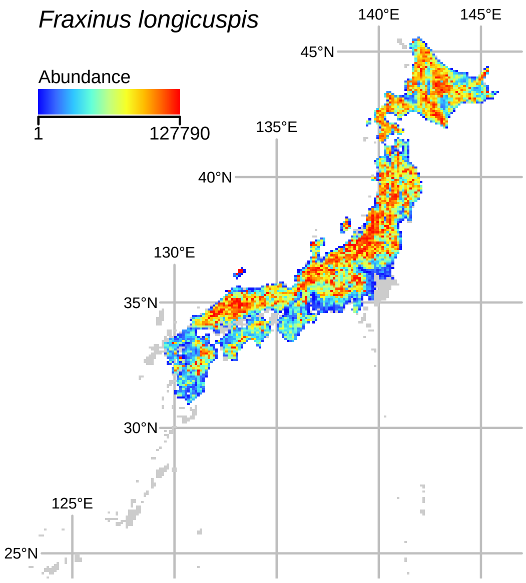
Fraxinus longicuspis
Abundance
1
127790
140°E
145°E
45°N
135°E
40°N
130°E
35°N
30°N
125°E
25°N

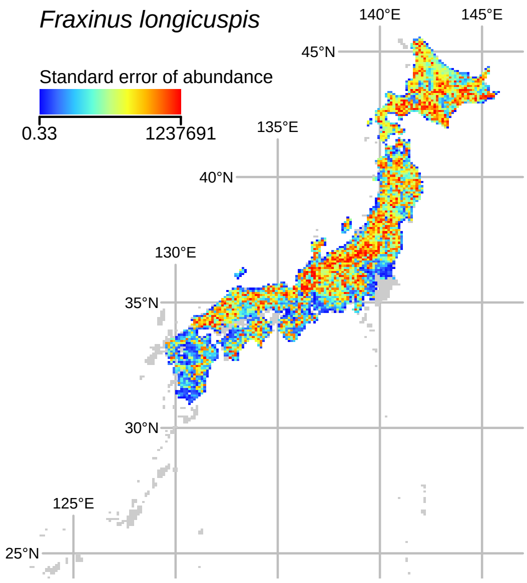
Fraxinus longicuspis
Standard error of abundance
0.33
1237691
140°E
145°E
45°N
135°E
40°N
130°E
35°N
30°N
125°E
25°N

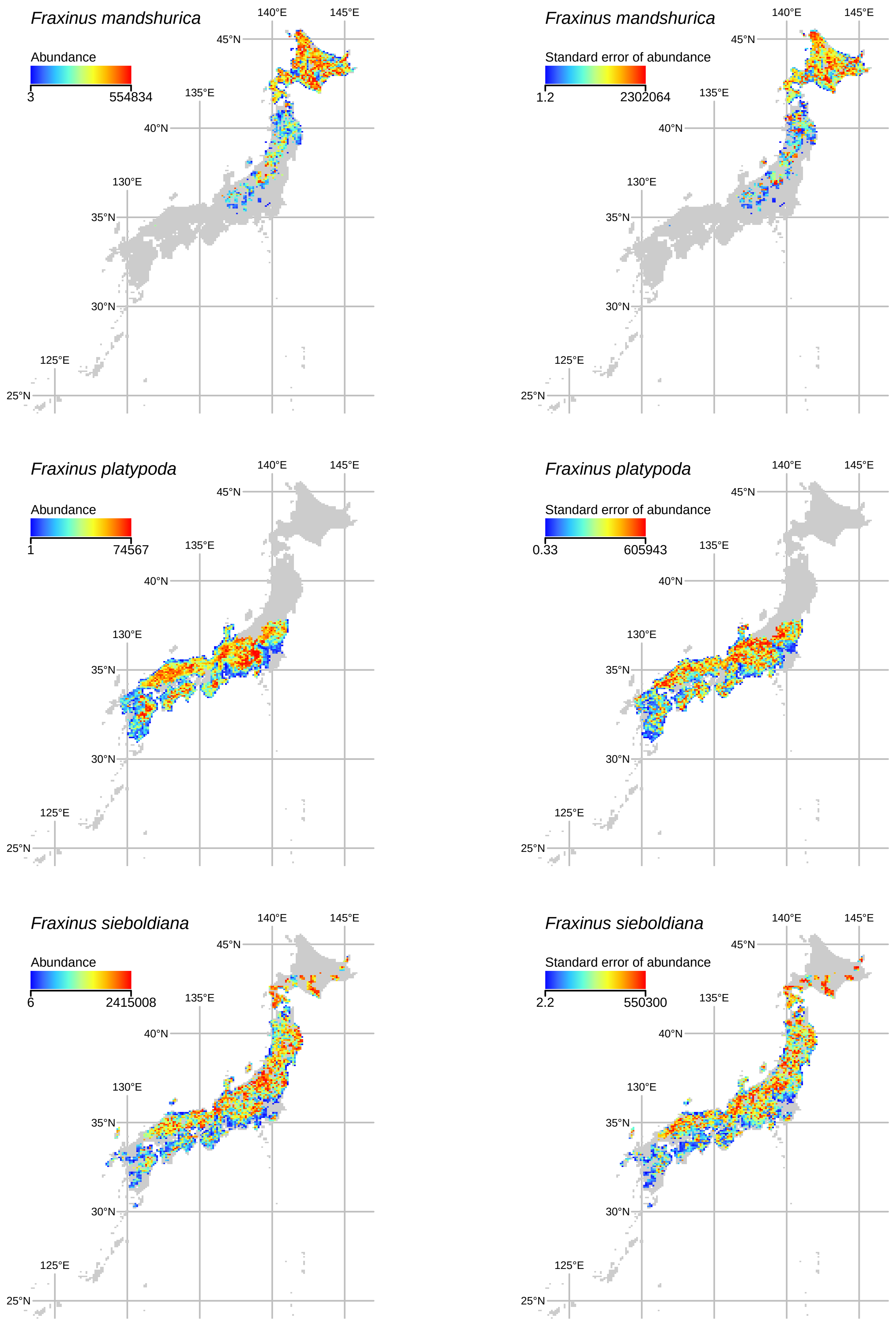
Fraxinus mandshurica
Abundance
3
554834
Fraxinus mandshurica
Standard error of abundance
1.2
2302064
Fraxinus platypoda
Abundance
1
74567
Fraxinus platypoda
Standard error of abundance
0.33
605943
Fraxinus sieboldiana
Abundance
6
2415008
Fraxinus sieboldiana
Standard error of abundance
2.2
550300
125°E
130°E
135°E
140°E
145°E
25°N
30°N
35°N
40°N
45°N

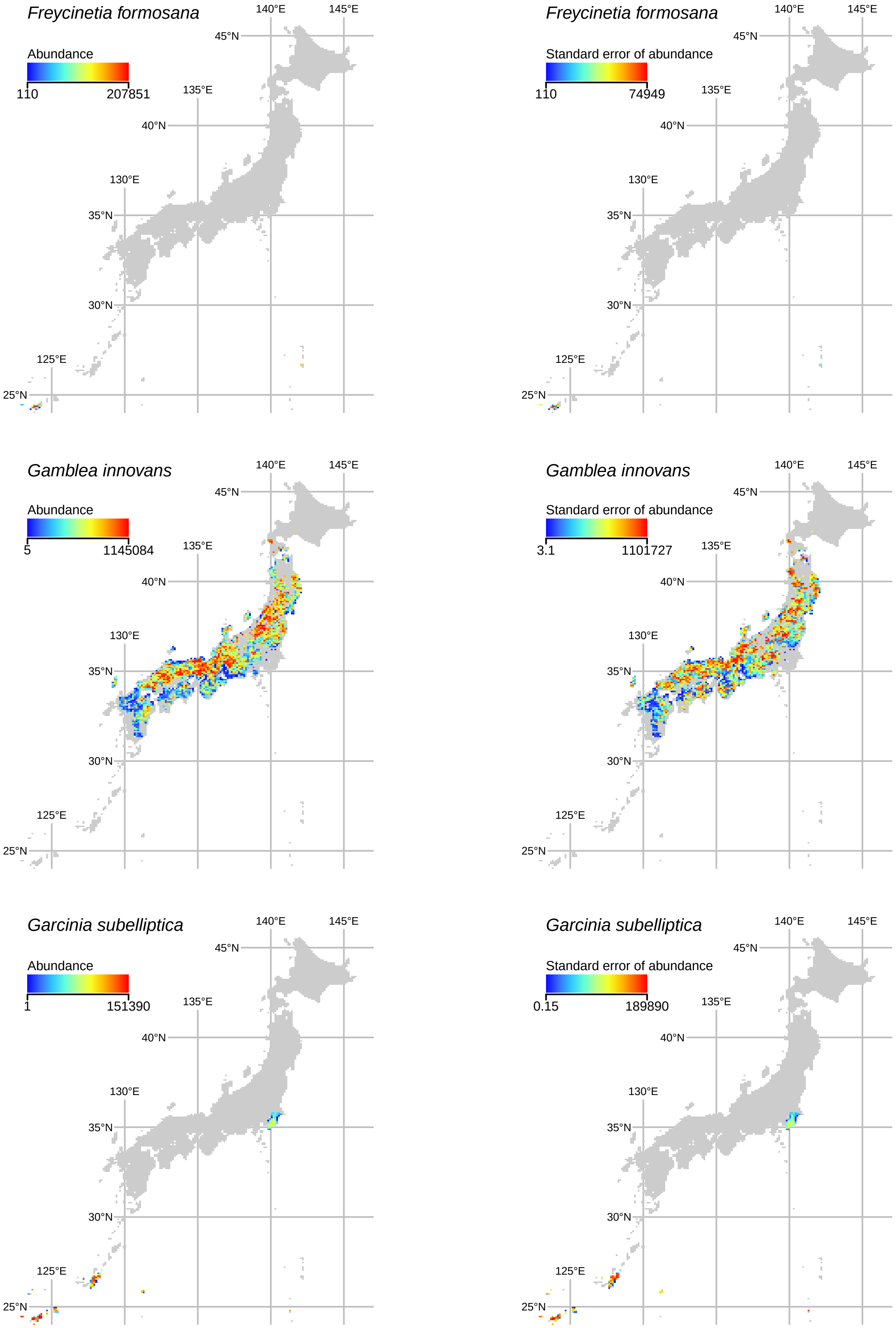
Freycinetia formosana
Abundance
110
207851
Freycinetia formosana
Standard error of abundance
110
74949
Gamblea innovans
Abundance
5
1145084
Gamblea innovans
Standard error of abundance
3.1
1101727
Garcinia subelliptica
Abundance
1
151390
Garcinia subelliptica
Standard error of abundance
0.15
189890
125°E
130°E
135°E
140°E
145°E
25°N
30°N
35°N
40°N
45°N

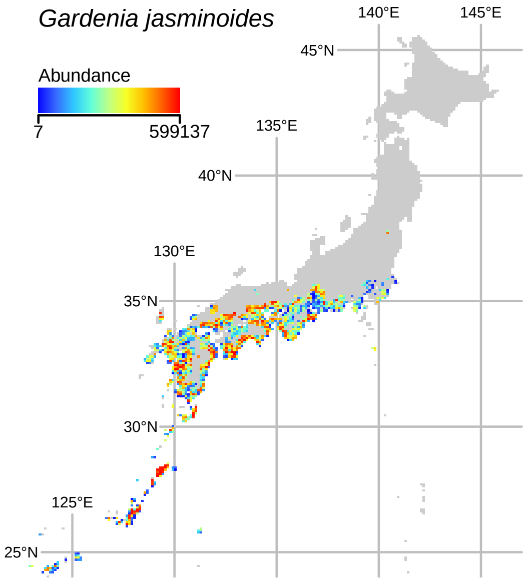
Gardenia jasminoides
Abundance
7
599137
140°E
145°E
45°N
135°E
40°N
130°E
35°N
30°N
125°E
25°N

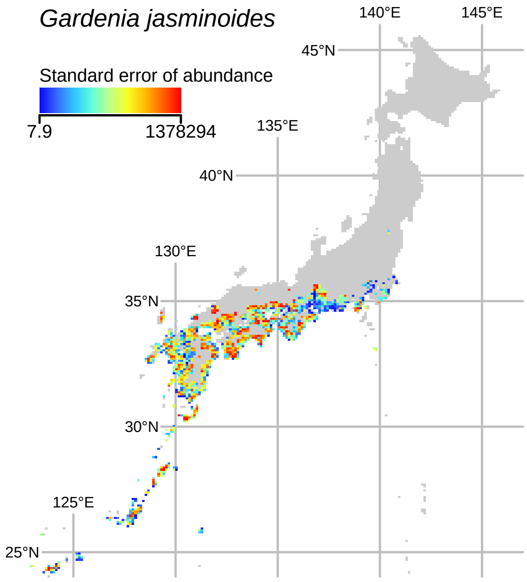
Gardenia jasminoides
Standard error of abundance
7.9
1378294
140°E
145°E
45°N
135°E
40°N
130°E
35°N
30°N
125°E
25°N

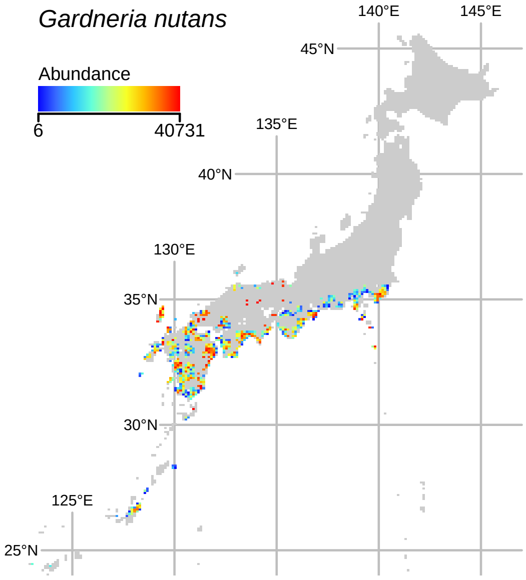
Gardneria nutans
Abundance
6
40731
140°E
145°E
45°N
135°E
40°N
130°E
35°N
30°N
125°E
25°N

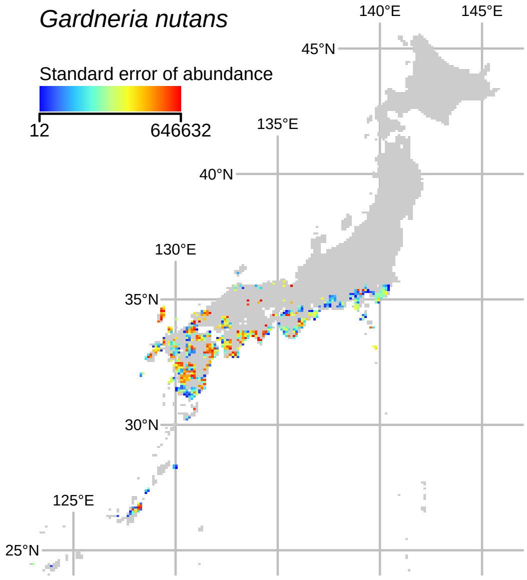
Gardneria nutans
Standard error of abundance
12
646632
140°E
145°E
45°N
135°E
40°N
130°E
35°N
30°N
125°E
25°N

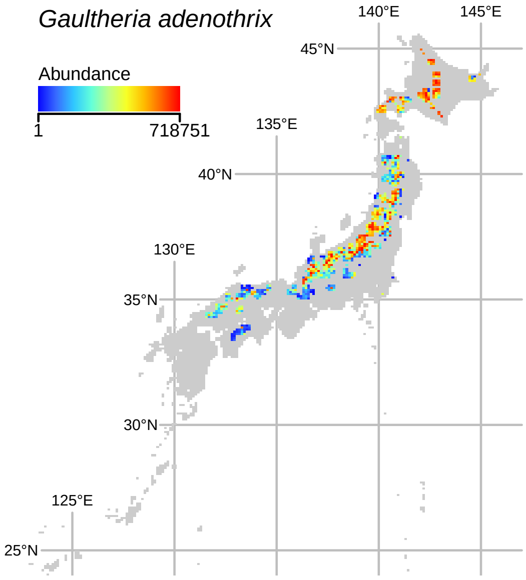
Gaultheria adenothrix
Abundance
1
718751
140°E
145°E
45°N
135°E
40°N
130°E
35°N
30°N
125°E
25°N

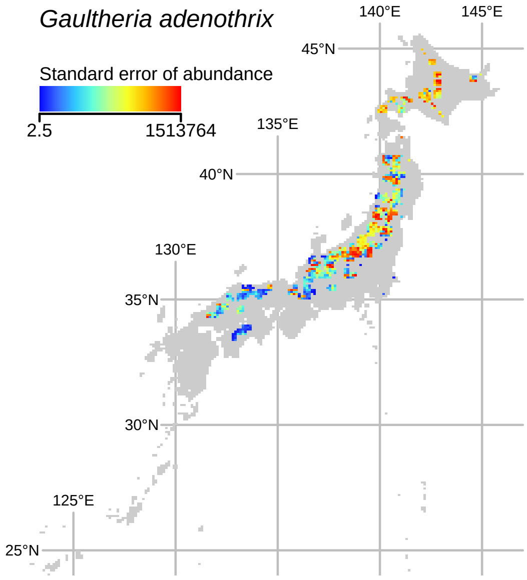
Gaultheria adenothrix
Standard error of abundance
2.5
1513764
140°E
145°E
45°N
135°E
40°N
130°E
35°N
30°N
125°E
25°N

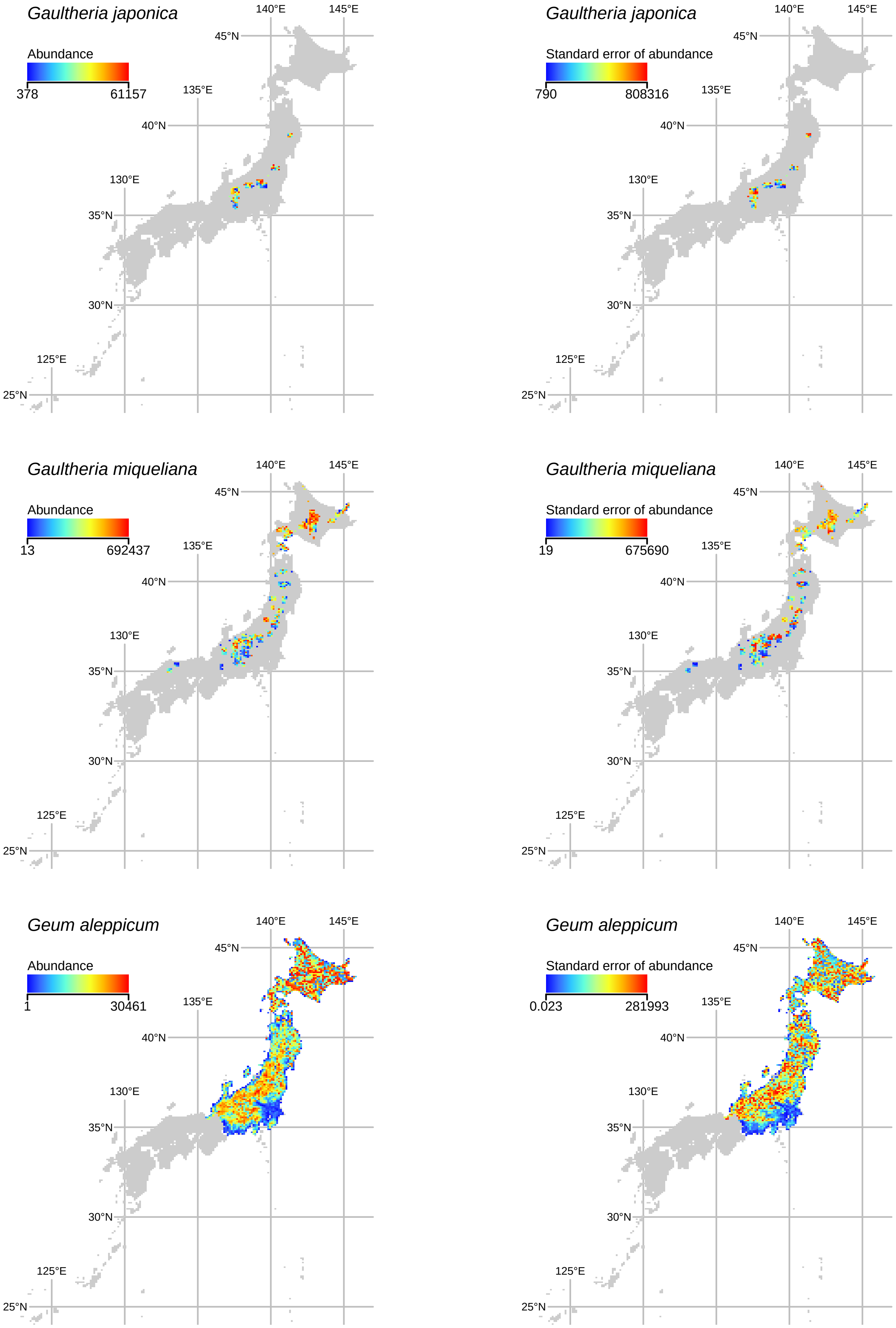
Gaultheria japonica
Abundance
378
61157
Gaultheria japonica
Standard error of abundance
790
808316
Gaultheria miqueliana
Abundance
13
692437
Gaultheria miqueliana
Standard error of abundance
19
675690
Geum aleppicum
Abundance
1
30461
Geum aleppicum
Standard error of abundance
0.023
281993
125°E
130°E
135°E
140°E
145°E
25°N
30°N
35°N
40°N
45°N

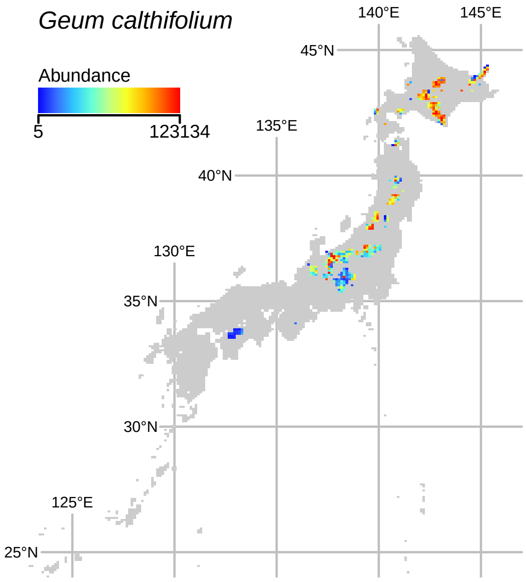
Geum calthifolium
Abundance
5
123134
140°E
145°E
45°N
135°E
40°N
130°E
35°N
30°N
125°E
25°N

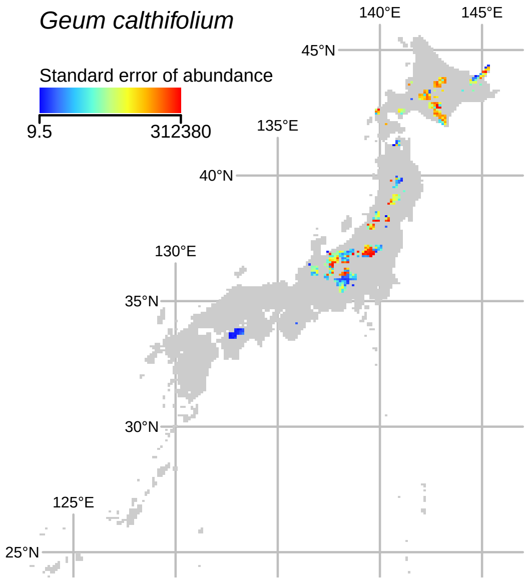
Geum calthifolium
Standard error of abundance
9.5
312380
140°E
145°E
45°N
135°E
40°N
130°E
35°N
30°N
125°E
25°N

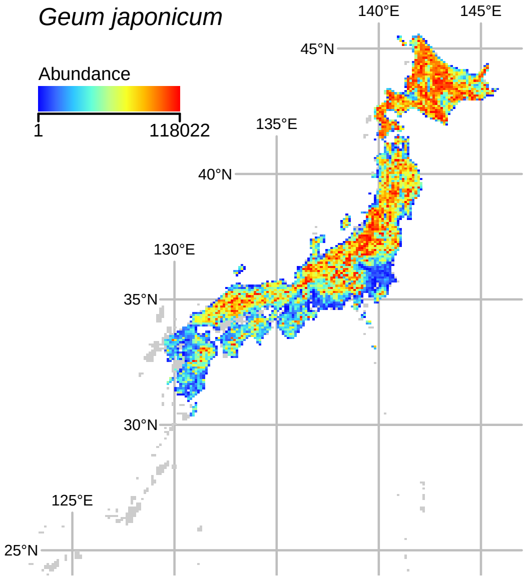
Geum japonicum
Abundance
1
118022
140°E
145°E
45°N
135°E
40°N
130°E
35°N
30°N
125°E
25°N

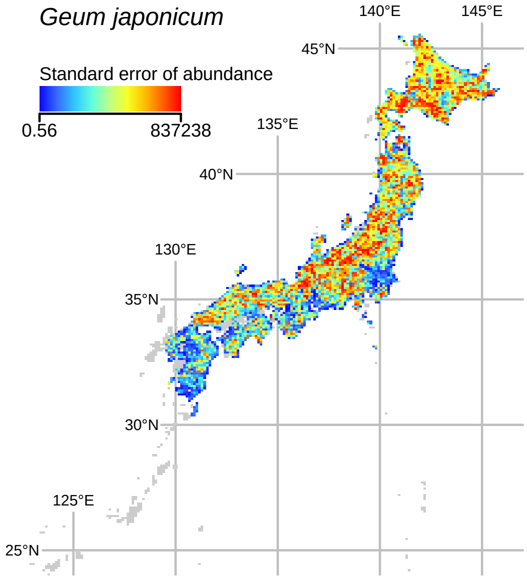
Geum japonicum
Standard error of abundance
0.56
837238
140°E
145°E
45°N
135°E
40°N
130°E
35°N
30°N
125°E
25°N

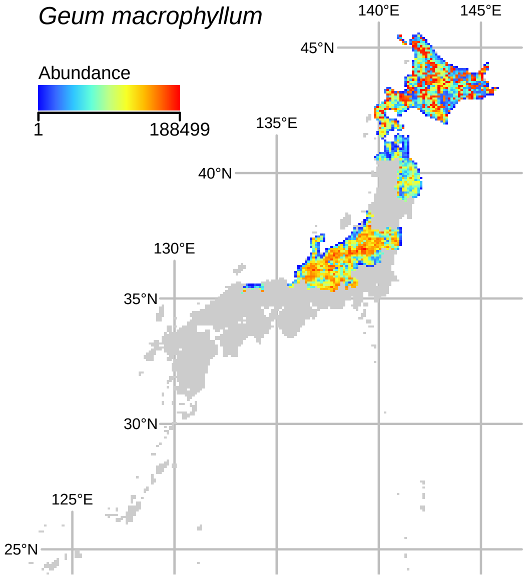
Geum macrophyllum
Abundance
1
188499
140°E
145°E
45°N
135°E
40°N
130°E
35°N
30°N
125°E
25°N

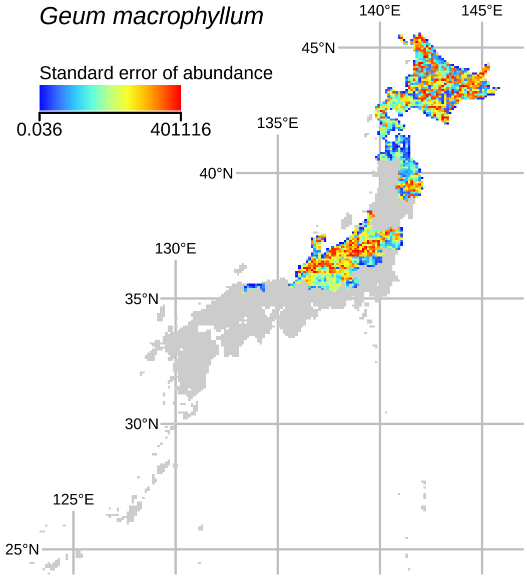
Geum macrophyllum
Standard error of abundance
0.036
401116
140°E
145°E
45°N
135°E
40°N
130°E
35°N
30°N
125°E
25°N

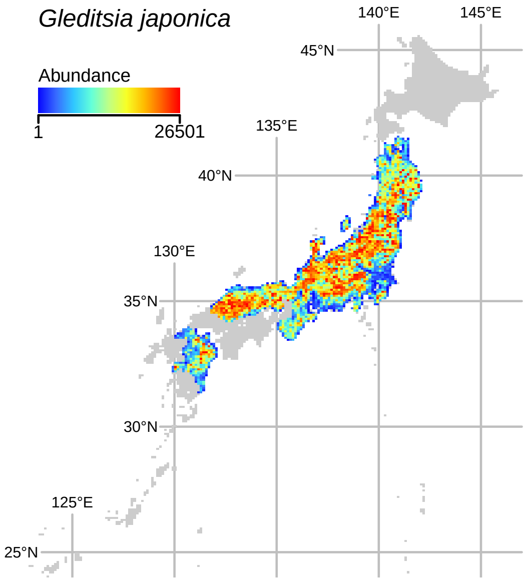
Gleditsia japonica
Abundance
1
26501
140°E
145°E
45°N
135°E
40°N
130°E
35°N
30°N
125°E
25°N

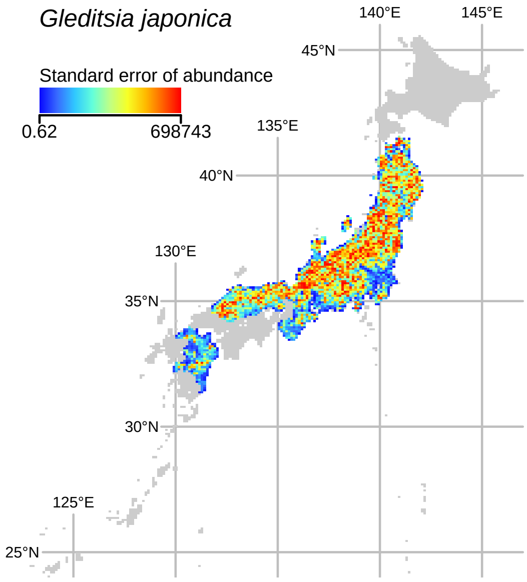
Gleditsia japonica
Standard error of abundance
0.62
698743
140°E
145°E
45°N
135°E
40°N
130°E
35°N
30°N
125°E
25°N

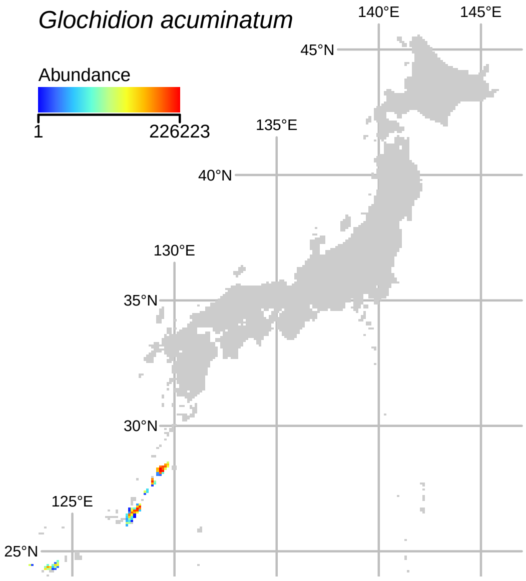
Glochidion acuminatum
Abundance
1
226223
140°E
145°E
45°N
135°E
40°N
130°E
35°N
30°N
125°E
25°N

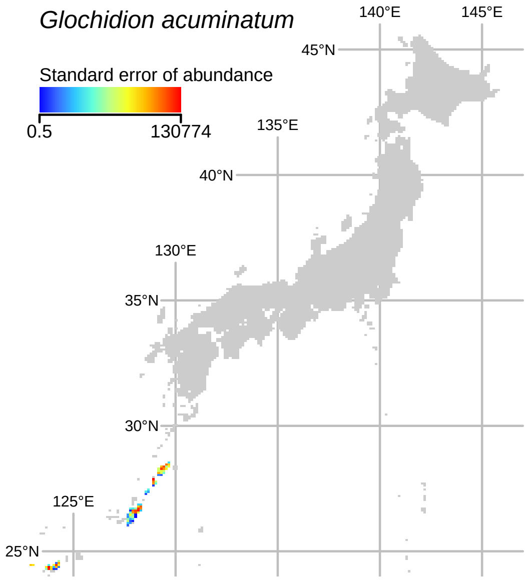
Glochidion acuminatum
Standard error of abundance
0.5
130774
140°E
145°E
45°N
135°E
40°N
130°E
35°N
30°N
125°E
25°N

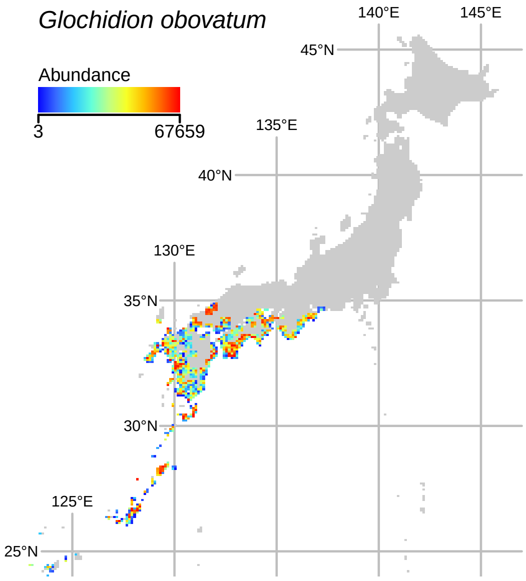
Glochidion obovatum
Abundance
3
67659
140°E
145°E
45°N
135°E
40°N
130°E
35°N
30°N
125°E
25°N

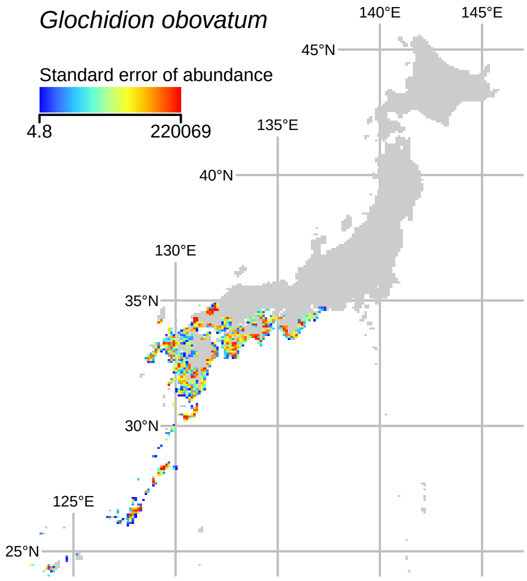
Glochidion obovatum
Standard error of abundance
4.8
220069
140°E
145°E
45°N
135°E
40°N
130°E
35°N
30°N
125°E
25°N

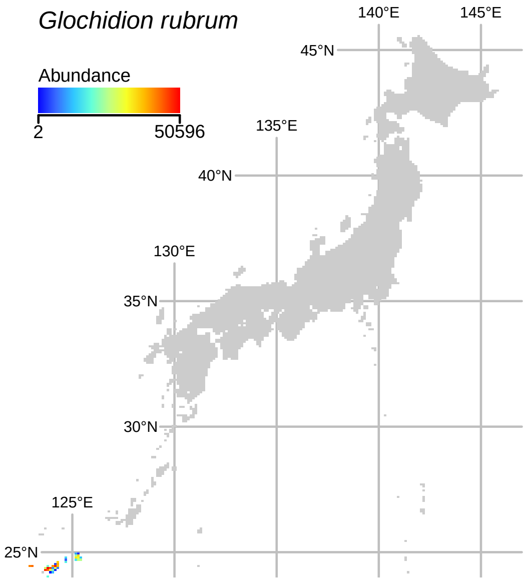
Glochidion rubrum
Abundance
2
50596
140°E
145°E
45°N
135°E
40°N
130°E
35°N
30°N
125°E
25°N

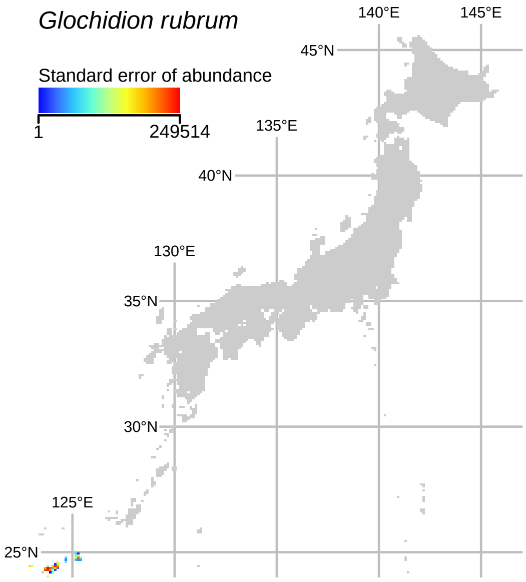
Glochidion rubrum
Standard error of abundance
1
249514
140°E
145°E
45°N
135°E
40°N
130°E
35°N
30°N
125°E
25°N

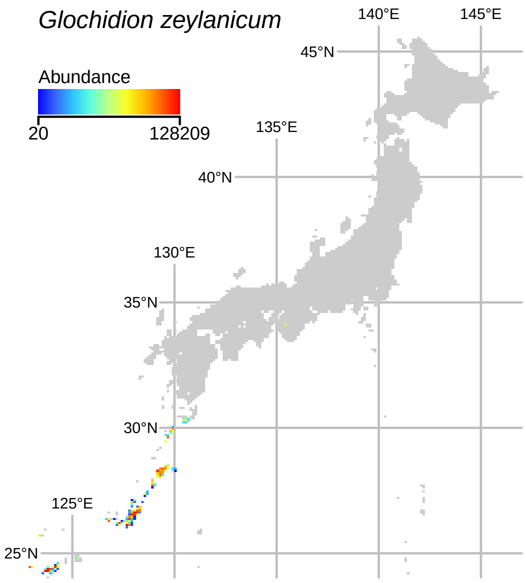
Glochidion zeylanicum
Abundance
20
128209
140°E
145°E
45°N
135°E
40°N
130°E
35°N
30°N
125°E
25°N

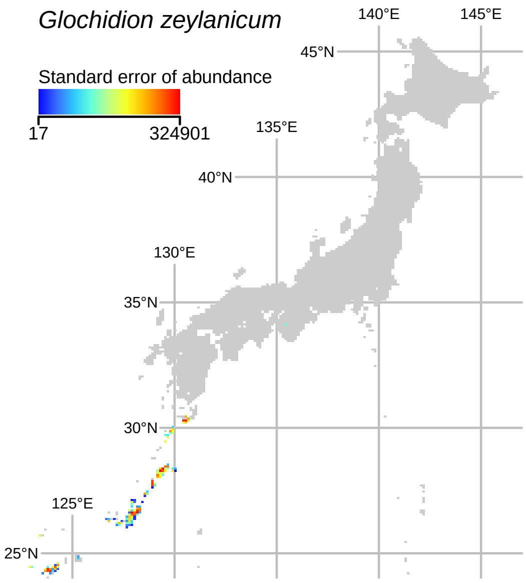
Glochidion zeylanicum
Standard error of abundance
17
324901
140°E
145°E
45°N
135°E
40°N
130°E
35°N
30°N
125°E
25°N

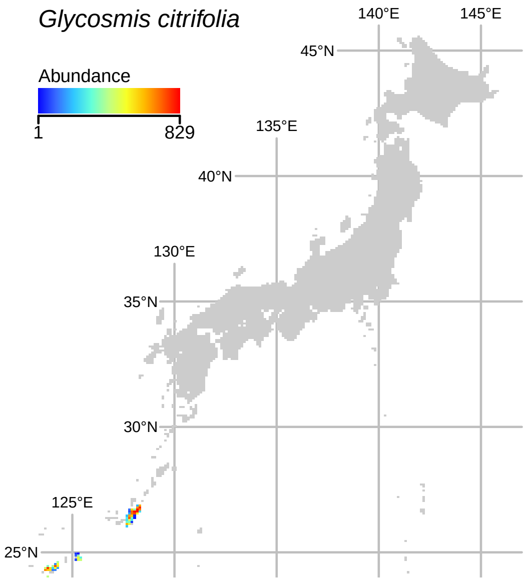
Glycosmis citrifolia
Abundance
1
829
140°E
145°E
45°N
135°E
40°N
130°E
35°N
30°N
125°E
25°N

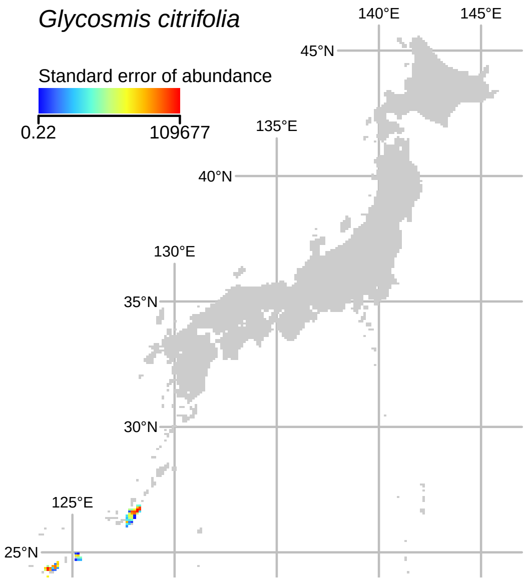
Glycosmis citrifolia
Standard error of abundance
0.22
109677
140°E
145°E
45°N
135°E
40°N
130°E
35°N
30°N
125°E
25°N

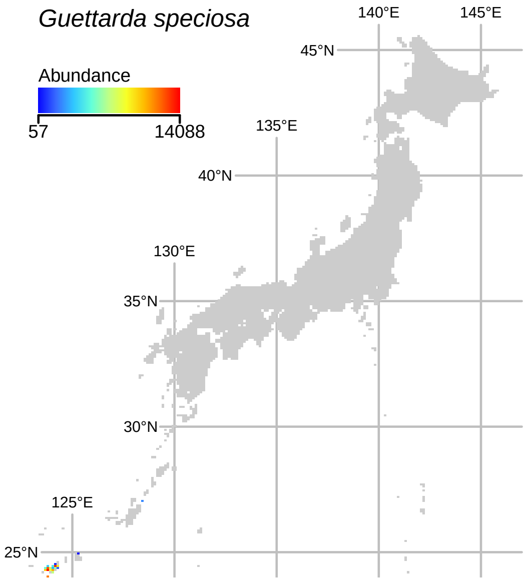
Guettarda speciosa
Abundance
57
14088
140°E
145°E
45°N
135°E
40°N
130°E
35°N
30°N
125°E
25°N

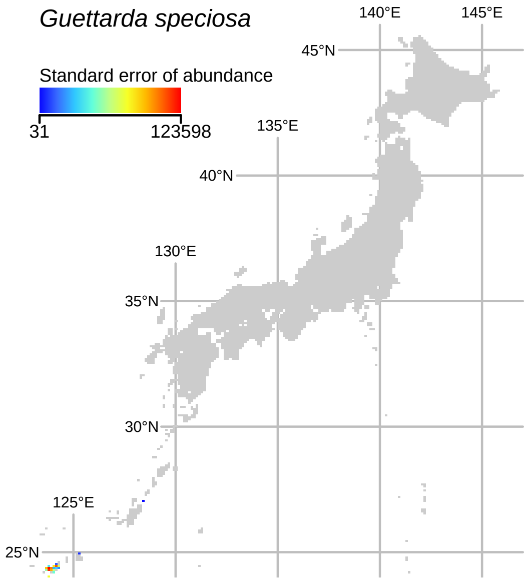
Guettarda speciosa
Standard error of abundance
31
123598
140°E
145°E
45°N
135°E
40°N
130°E
35°N
30°N
125°E
25°N

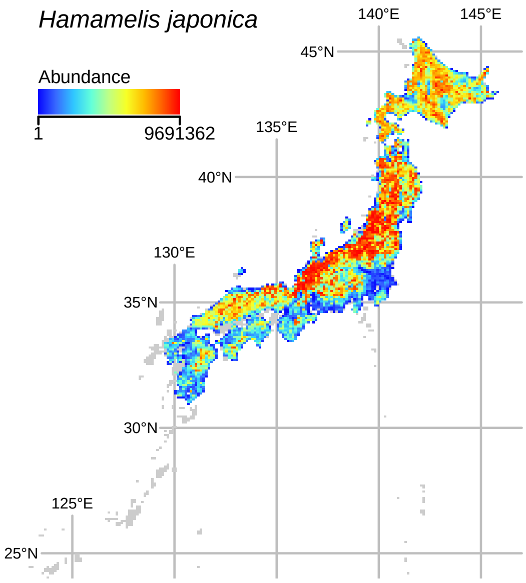
Hamamelis japonica
Abundance
1
9691362
140°E
145°E
45°N
135°E
40°N
130°E
35°N
30°N
125°E
25°N

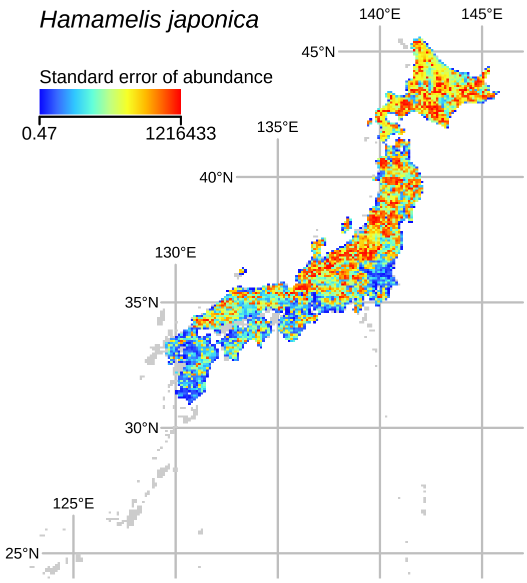
Hamamelis japonica
Standard error of abundance
0.47
1216433
140°E
145°E
45°N
135°E
40°N
130°E
35°N
30°N
125°E
25°N

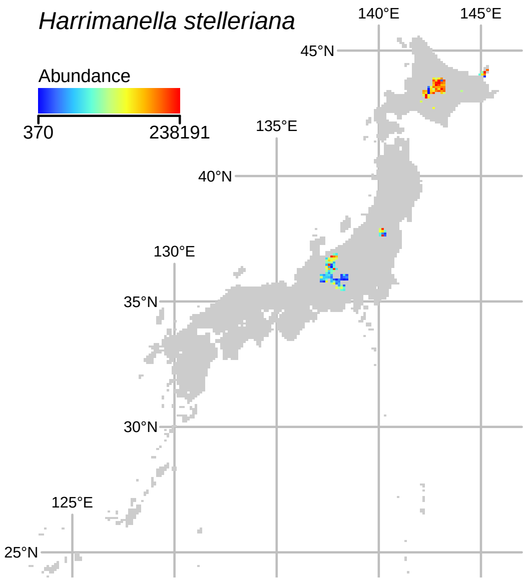
Harrimanella stelleriana
Abundance
370
238191
140°E
145°E
45°N
135°E
40°N
130°E
35°N
30°N
125°E
25°N

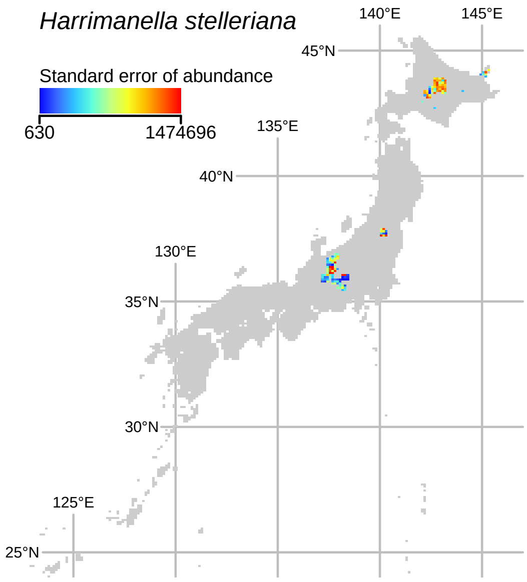
Harrimanella stelleriana
Standard error of abundance
630
1474696
140°E
145°E
45°N
135°E
40°N
130°E
35°N
30°N
125°E
25°N

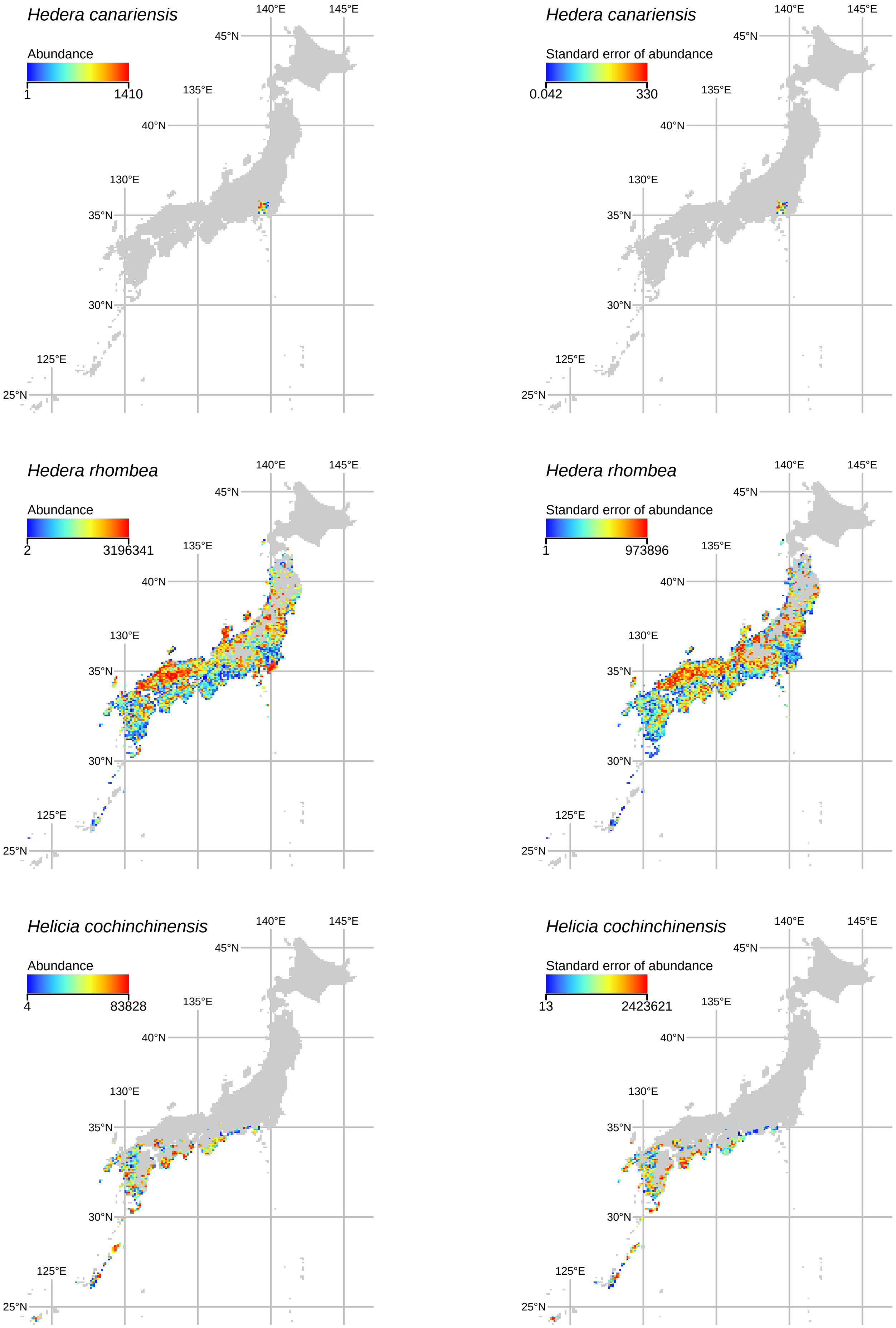
Hedera canariensis
Abundance
1
1410
Hedera canariensis
Standard error of abundance
0.042
330
Hedera rhombea
Abundance
2
3196341
Hedera rhombea
Standard error of abundance
1
973896
Helicia cochinchinensis
Abundance
4
83828
Helicia cochinchinensis
Standard error of abundance
13
2423621
125°E
130°E
135°E
140°E
145°E
25°N
30°N
35°N
40°N
45°N

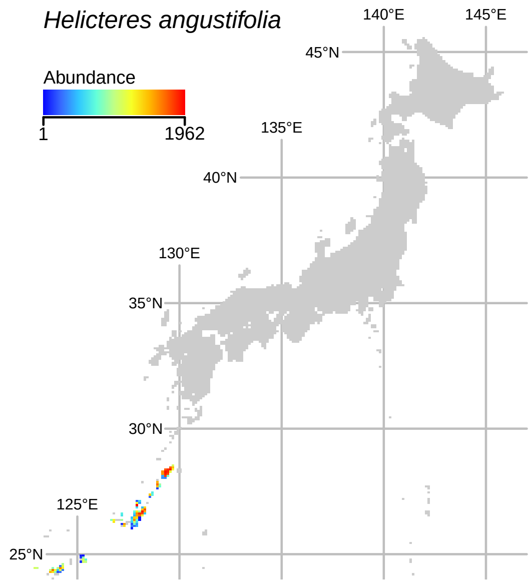
Helicteres angustifolia
Abundance
1
1962
140°E
145°E
45°N
135°E
40°N
130°E
35°N
30°N
125°E
25°N

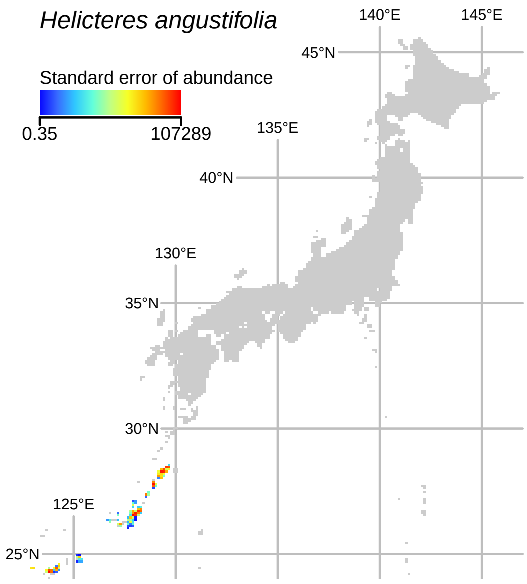
Helicteres angustifolia
Standard error of abundance
0.35
107289
140°E
145°E
45°N
135°E
40°N
130°E
35°N
30°N
125°E
25°N

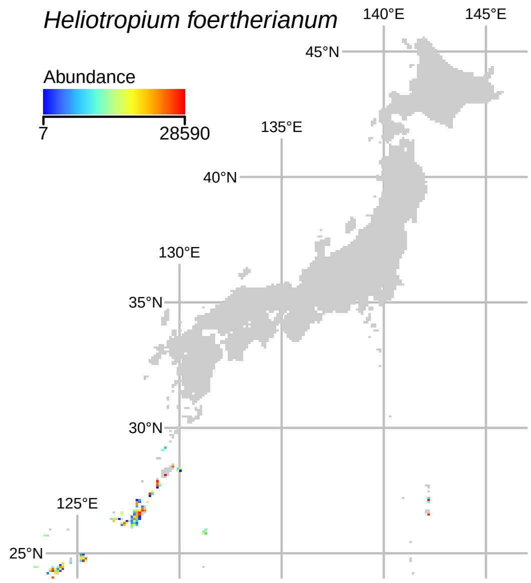
Heliotropium foertherianum
Abundance
7
28590
140°E
145°E
45°N
135°E
40°N
130°E
35°N
30°N
125°E
25°N

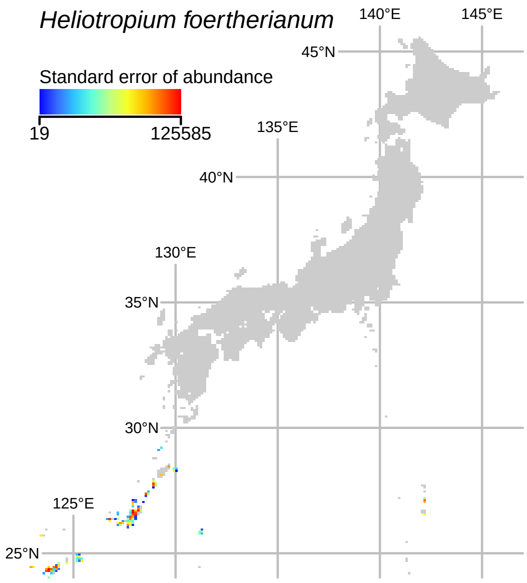
Heliotropium foertherianum
Standard error of abundance
19
125585
140°E
145°E
45°N
135°E
40°N
130°E
35°N
30°N
125°E
25°N

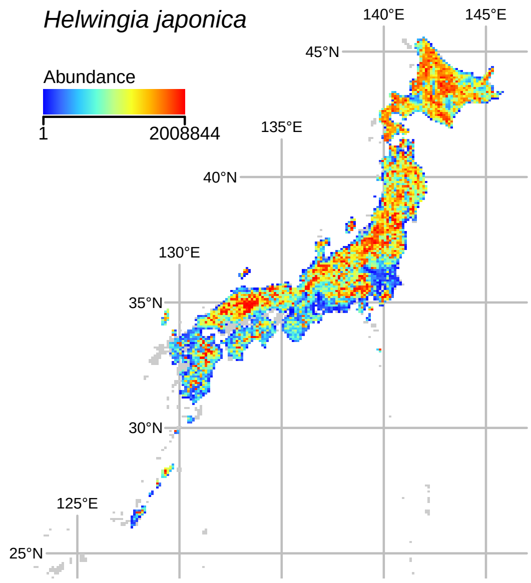
Helwingia japonica
Abundance
1
2008844
140°E
145°E
45°N
135°E
40°N
130°E
35°N
30°N
125°E
25°N

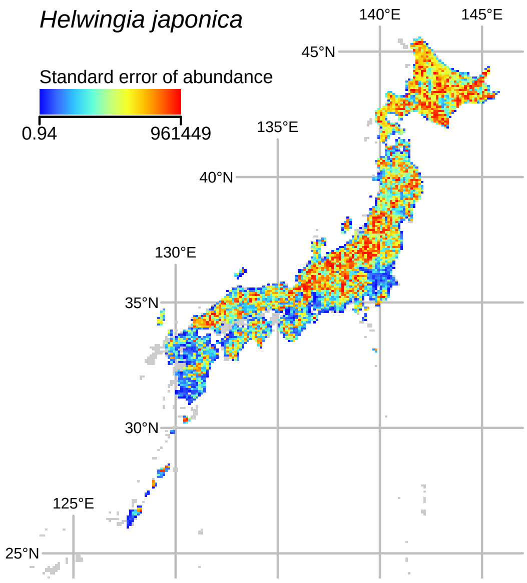
Helwingia japonica
Standard error of abundance
0.94
961449
140°E
145°E
45°N
135°E
40°N
130°E
35°N
30°N
125°E
25°N

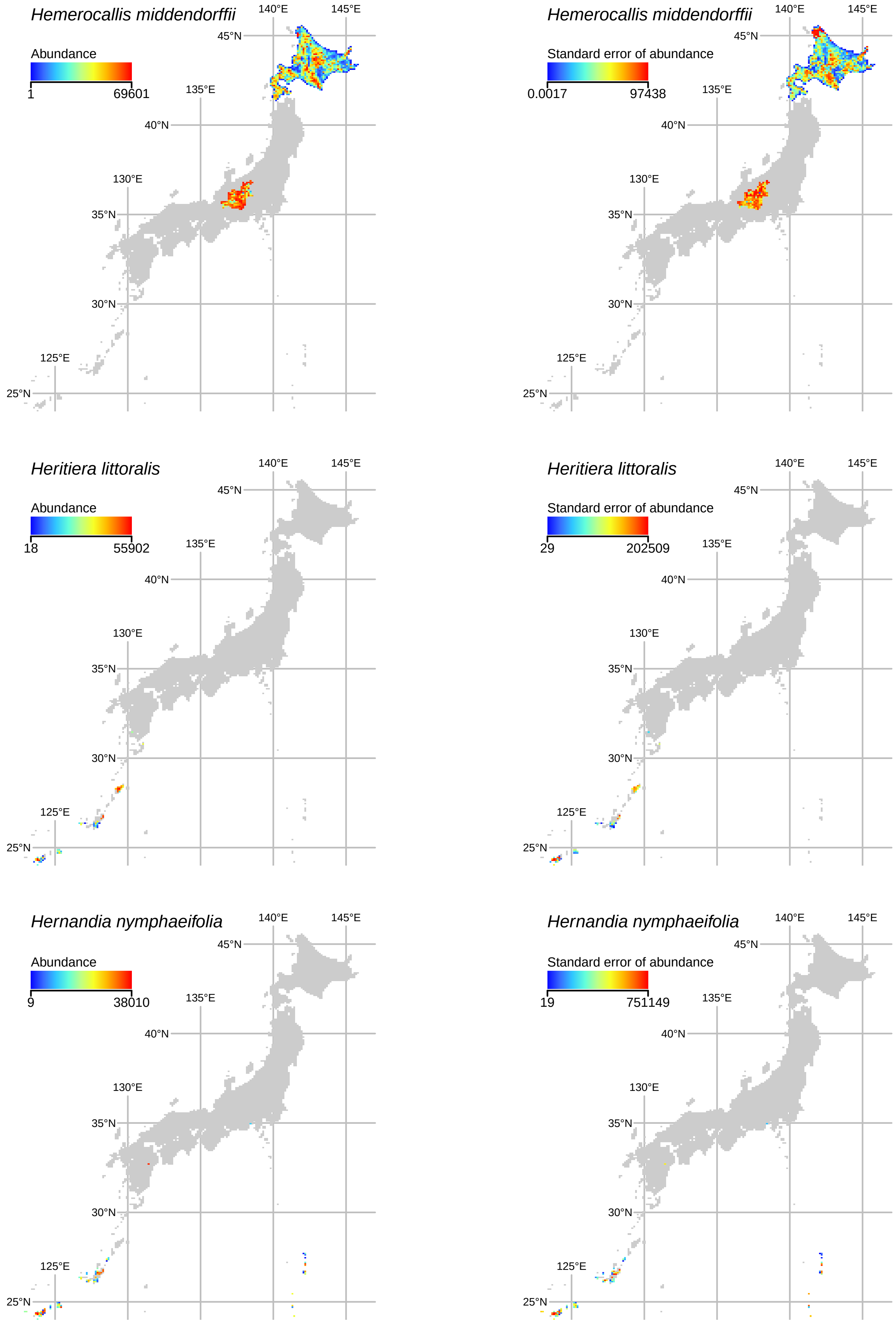
Hemerocallis middendorffii
Abundance
1
69601
Hemerocallis middendorffii
Standard error of abundance
0.0017
97438
Heritiera littoralis
Abundance
18
55902
Heritiera littoralis
Standard error of abundance
29
202509
Hernandia nymphaeifolia
Abundance
9
38010
Hernandia nymphaeifolia
Standard error of abundance
19
751149
125°E
130°E
135°E
140°E
145°E
25°N
30°N
35°N
40°N
45°N

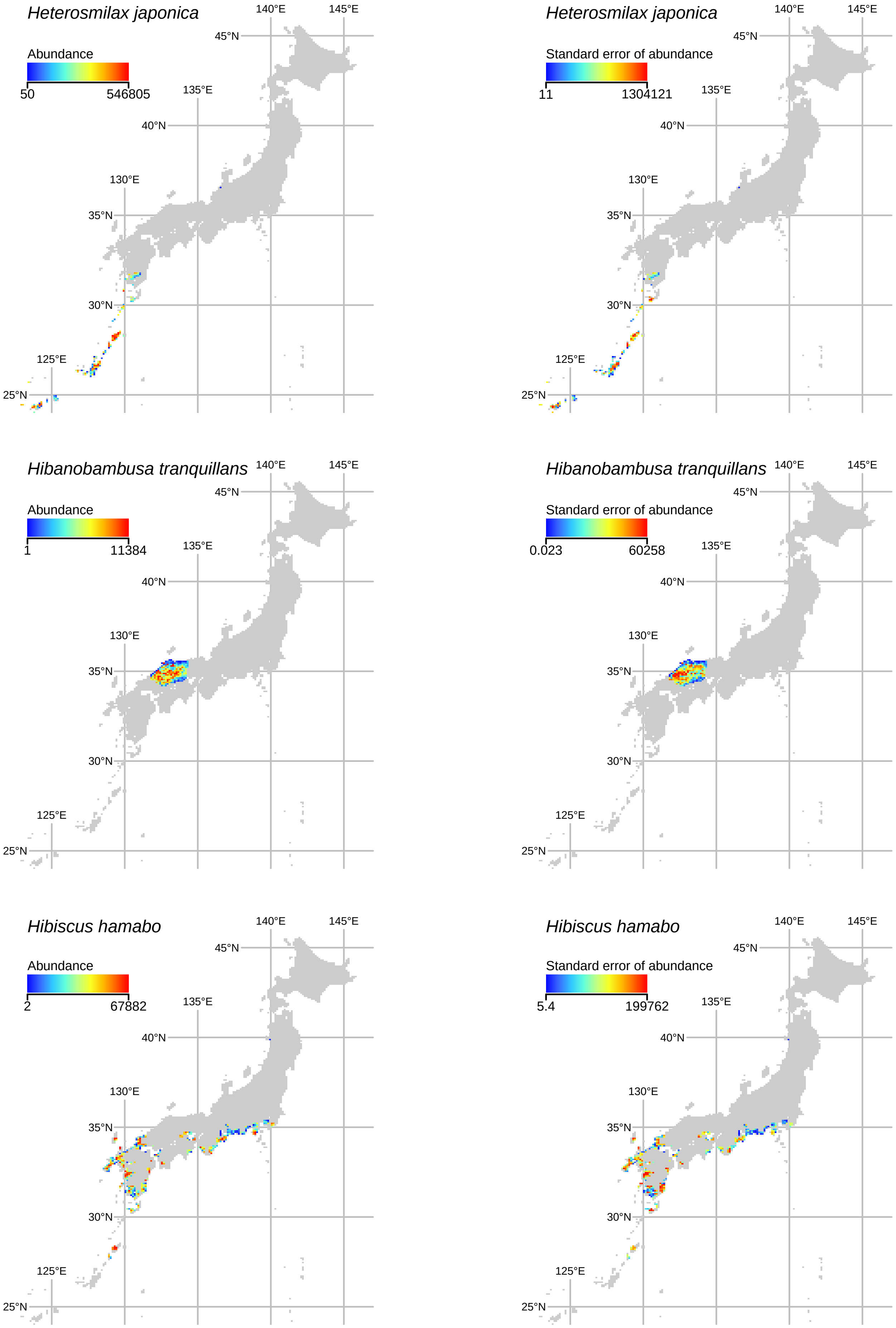
Heterosmilax japonica
Abundance
50
546805
Heterosmilax japonica
Standard error of abundance
11
1304121
Hibanobambusa tranquillans
Abundance
1
11384
Hibanobambusa tranquillans
Standard error of abundance
0.023
60258
Hibiscus hamabo
Abundance
2
67882
Hibiscus hamabo
Standard error of abundance
5.4
199762
125°E
130°E
135°E
140°E
145°E
25°N
30°N
35°N
40°N
45°N

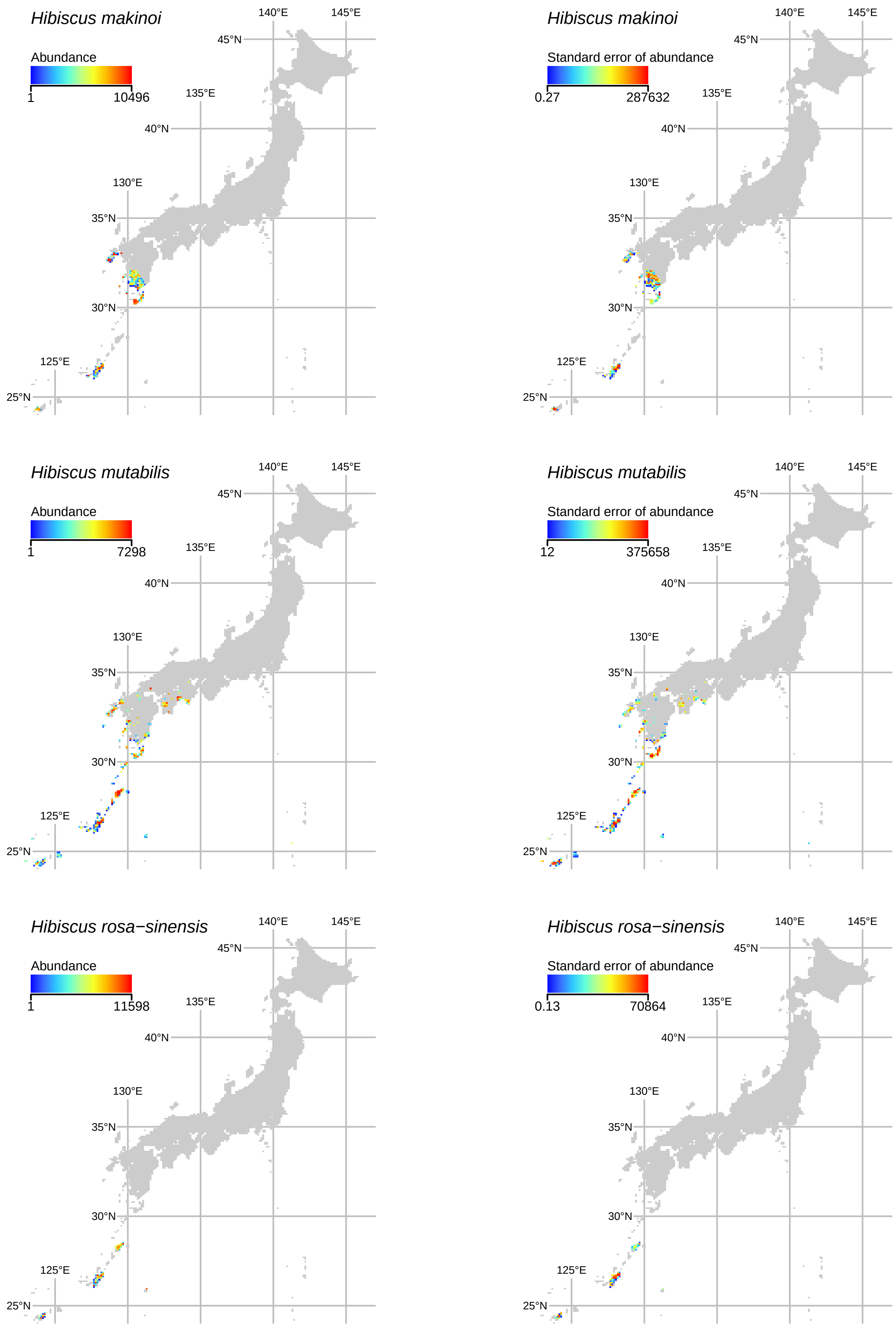
Hibiscus makinoi
Abundance
1
10496
Hibiscus makinoi
Standard error of abundance
0.27
287632
Hibiscus mutabilis
Abundance
1
7298
Hibiscus mutabilis
Standard error of abundance
12
375658
Hibiscus rosa−sinensis
Abundance
1
11598
Hibiscus rosa−sinensis
Standard error of abundance
0.13
70864
125°E
130°E
135°E
140°E
145°E
25°N
30°N
35°N
40°N
45°N

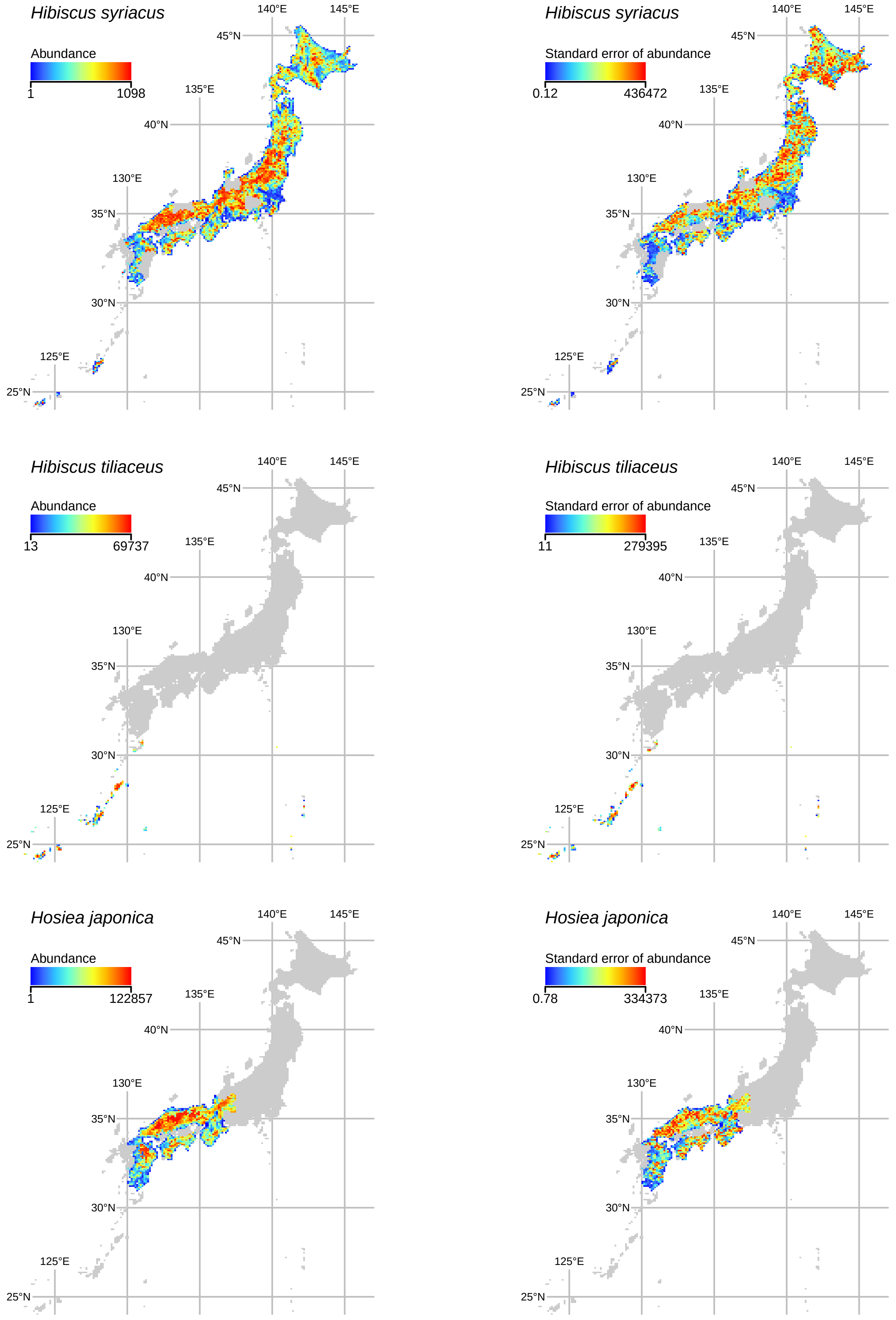
Hibiscus syriacus
Abundance
1
1098
Hibiscus syriacus
Standard error of abundance
0.12
436472
Hibiscus tiliaceus
Abundance
13
69737
Hibiscus tiliaceus
Standard error of abundance
11
279395
Hosiea japonica
Abundance
1
122857
Hosiea japonica
Standard error of abundance
0.78
334373
125°E
130°E
135°E
140°E
145°E
25°N
30°N
35°N
40°N
45°N

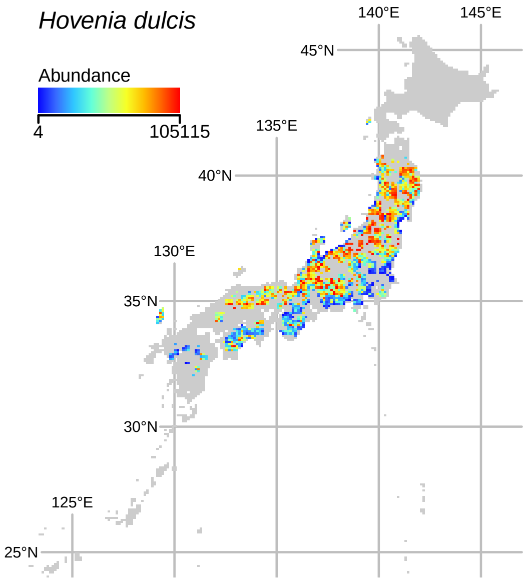
Hovenia dulcis
Abundance
4
105115
140°E
145°E
135°E
130°E
125°E
45°N
40°N
35°N
30°N
25°N

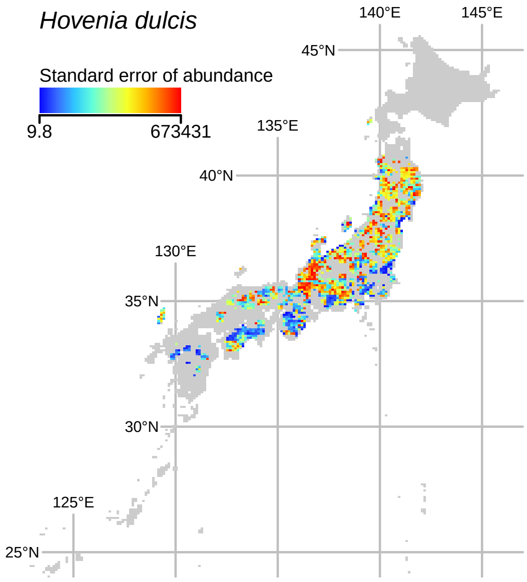
Hovenia dulcis
Standard error of abundance
9.8
673431
140°E
145°E
135°E
130°E
125°E
45°N
40°N
35°N
30°N
25°N

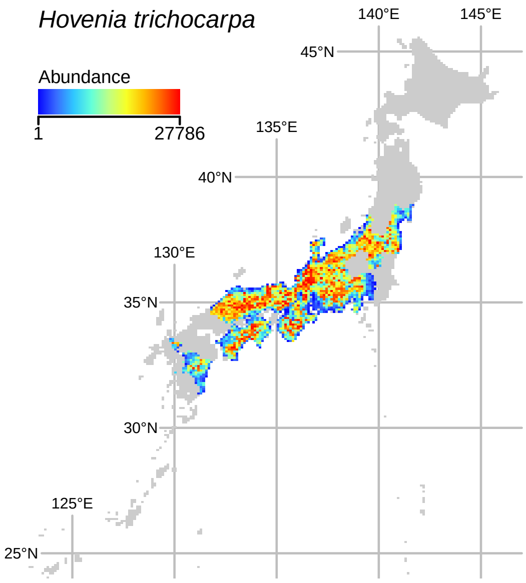
Hovenia trichocarpa
Abundance
1
27786
140°E
145°E
135°E
130°E
125°E
45°N
40°N
35°N
30°N
25°N

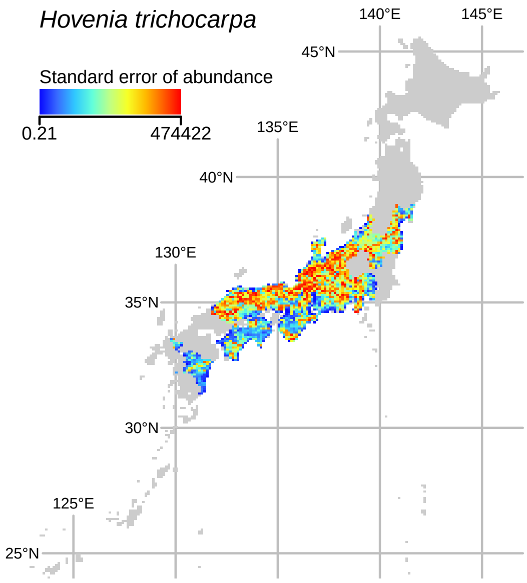
Hovenia trichocarpa
Standard error of abundance
0.21
474422
140°E
145°E
135°E
130°E
125°E
45°N
40°N
35°N
30°N
25°N

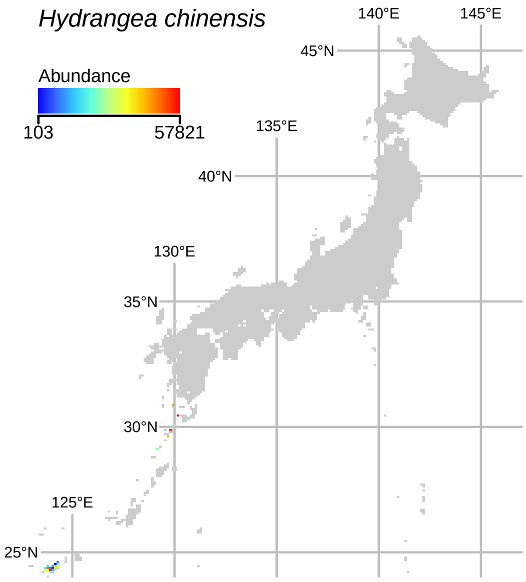
Hydrangea chinensis
Abundance
103
57821
140°E
145°E
135°E
130°E
125°E
45°N
40°N
35°N
30°N
25°N

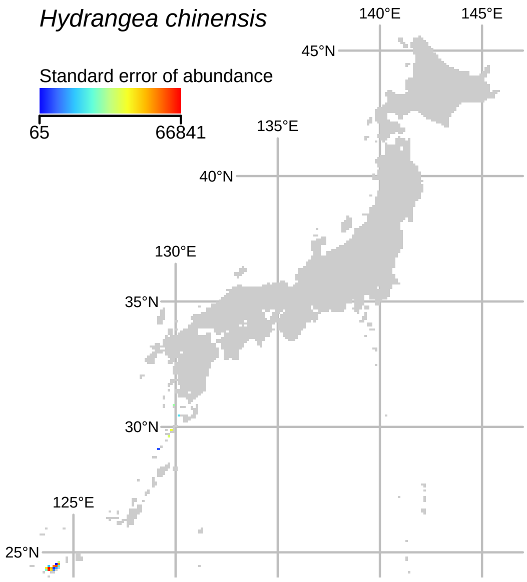
Hydrangea chinensis
Standard error of abundance
65
66841
140°E
145°E
135°E
130°E
125°E
45°N
40°N
35°N
30°N
25°N

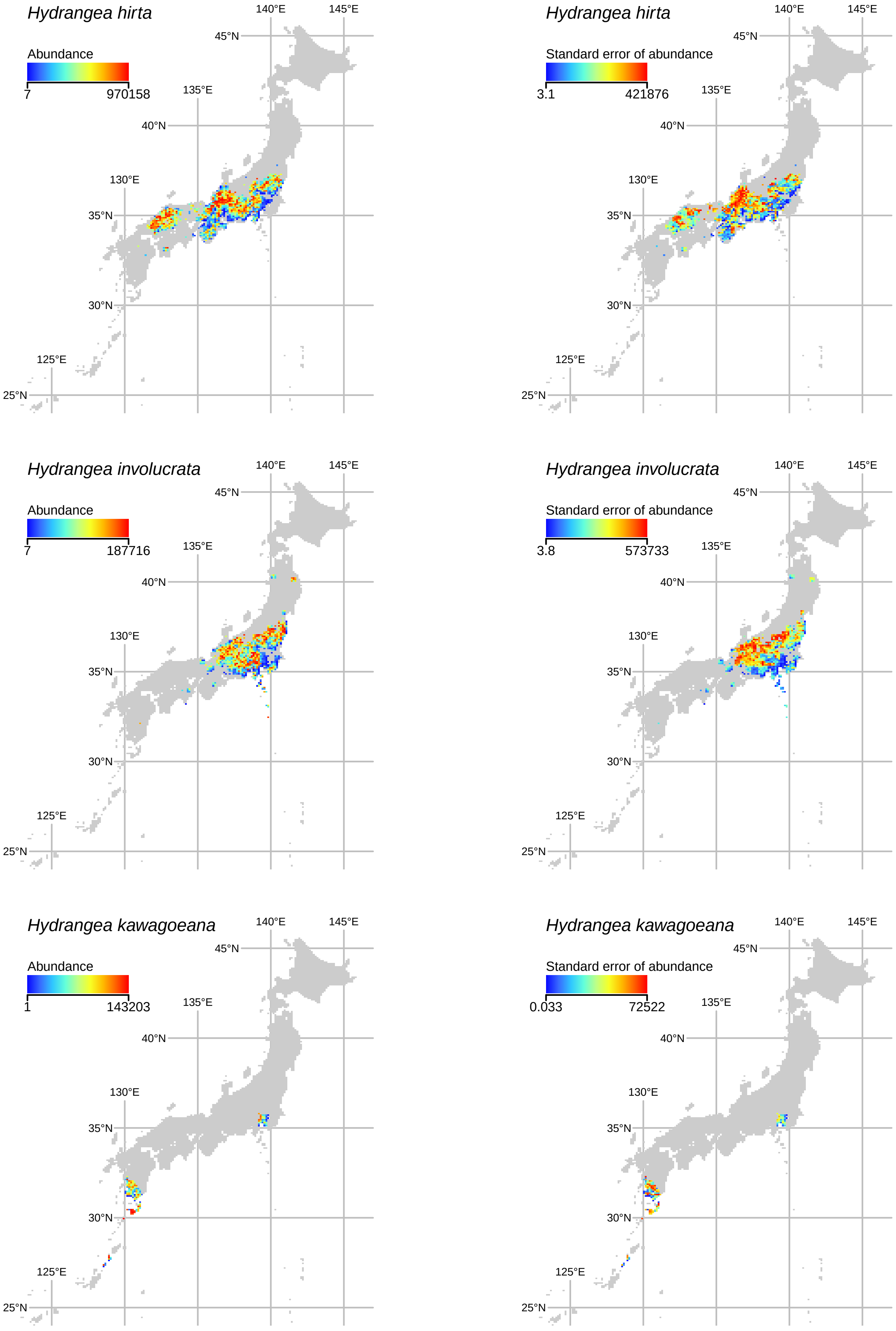
Hydrangea hirta
Abundance
7
970158
Hydrangea hirta
Standard error of abundance
3.1
421876
Hydrangea involucrata
Abundance
7
187716
Hydrangea involucrata
Standard error of abundance
3.8
573733
Hydrangea kawagoeana
Abundance
1
143203
Hydrangea kawagoeana
Standard error of abundance
0.033
72522
125°E
130°E
135°E
140°E
145°E
25°N
30°N
35°N
40°N
45°N

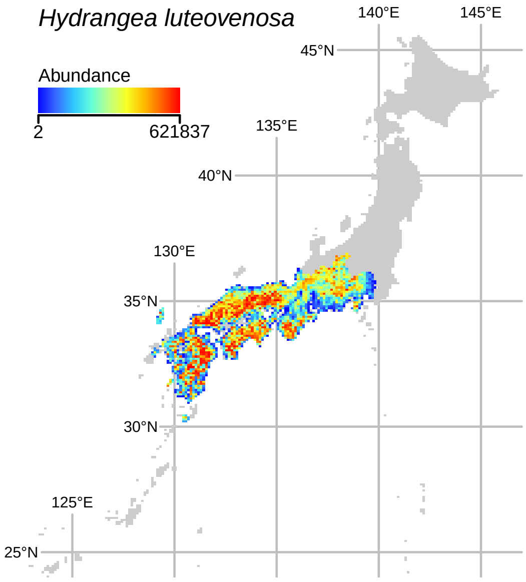
Hydrangea luteovenosa
Abundance
2
621837
140°E
145°E
45°N
135°E
40°N
130°E
35°N
30°N
125°E
25°N

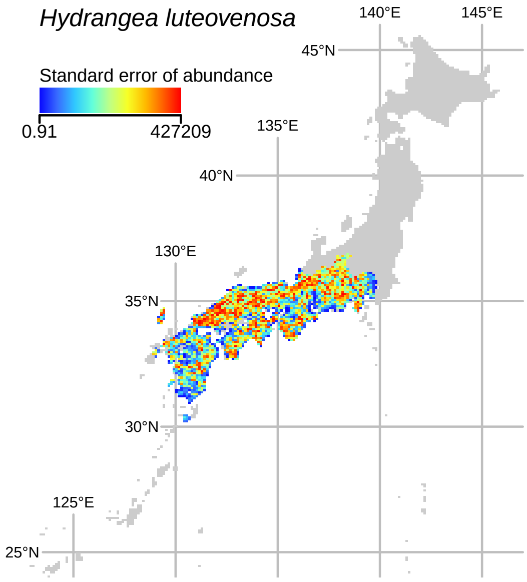
Hydrangea luteovenosa
Standard error of abundance
0.91
427209
140°E
145°E
45°N
135°E
40°N
130°E
35°N
30°N
125°E
25°N

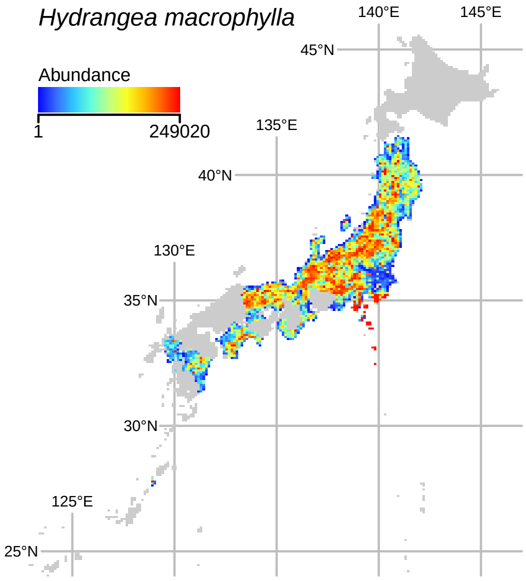
Hydrangea macrophylla
Abundance
1
249020
140°E
145°E
45°N
135°E
40°N
130°E
35°N
30°N
125°E
25°N

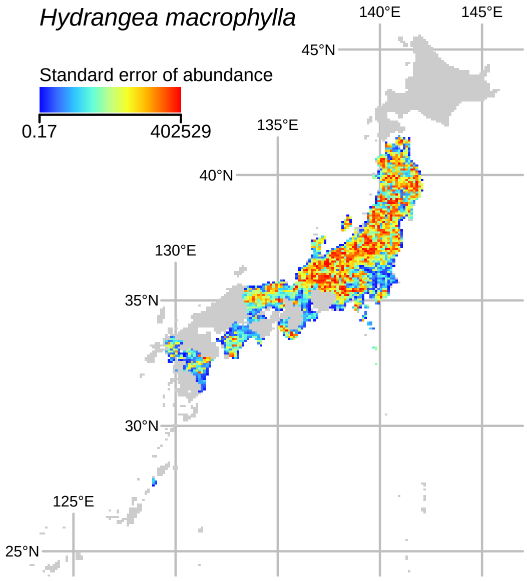
Hydrangea macrophylla
Standard error of abundance
0.17
402529
140°E
145°E
45°N
135°E
40°N
130°E
35°N
30°N
125°E
25°N

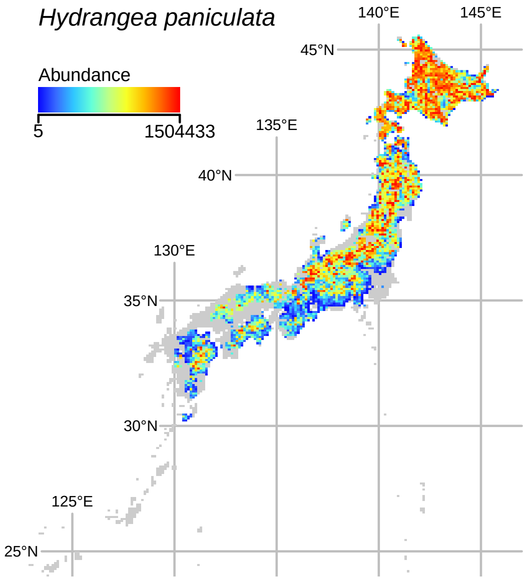
Hydrangea paniculata
Abundance
5
1504433
140°E
145°E
45°N
135°E
40°N
130°E
35°N
30°N
125°E
25°N

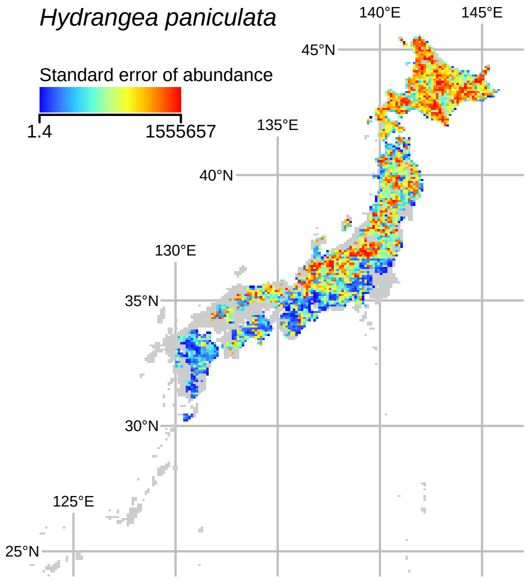
Hydrangea paniculata
Standard error of abundance
1.4
1555657
140°E
145°E
45°N
135°E
40°N
130°E
35°N
30°N
125°E
25°N

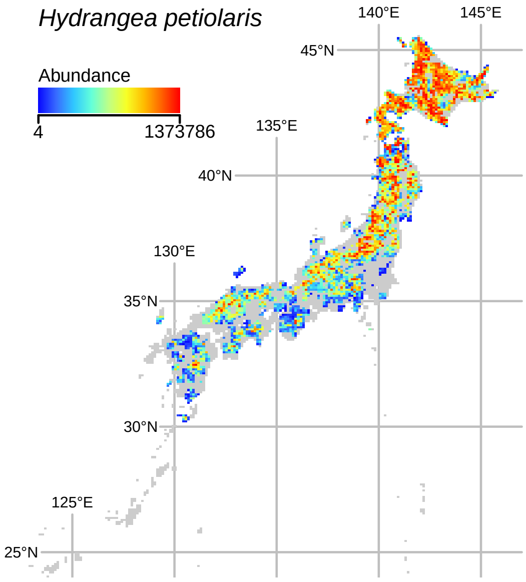
Hydrangea petiolaris
Abundance
4
1373786
140°E
145°E
45°N
135°E
40°N
130°E
35°N
30°N
125°E
25°N

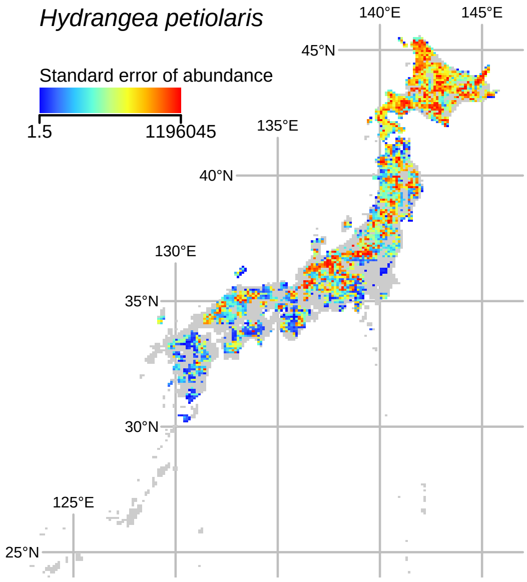
Hydrangea petiolaris
Standard error of abundance
1.5
1196045
140°E
145°E
45°N
135°E
40°N
130°E
35°N
30°N
125°E
25°N

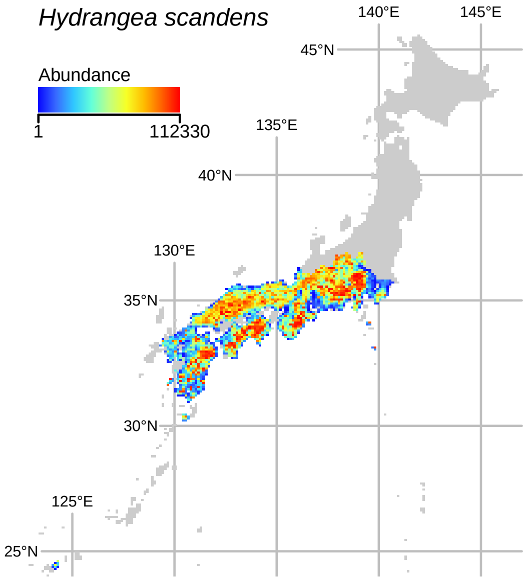
Hydrangea scandens
Abundance
1
112330
140°E
145°E
45°N
135°E
40°N
130°E
35°N
30°N
125°E
25°N

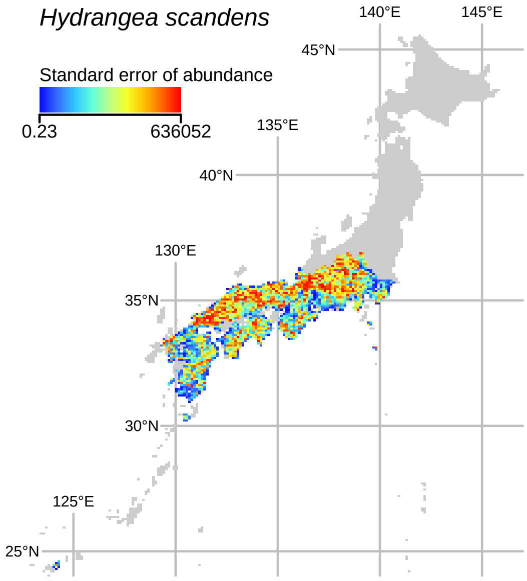
Hydrangea scandens
Standard error of abundance
0.23
636052
140°E
145°E
45°N
135°E
40°N
130°E
35°N
30°N
125°E
25°N

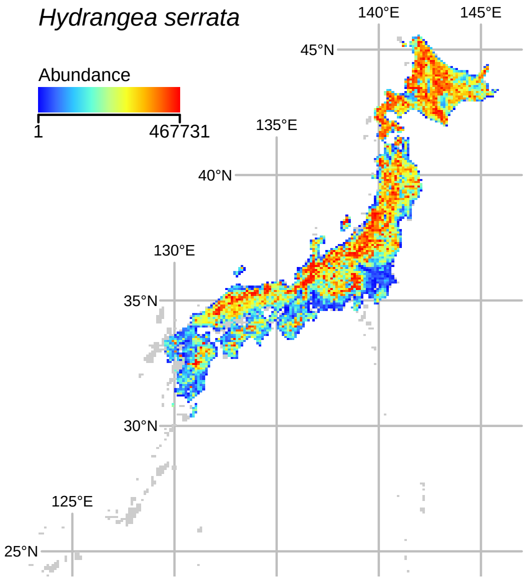
Hydrangea serrata
Abundance
1
467731
140°E
145°E
45°N
135°E
40°N
130°E
35°N
30°N
125°E
25°N

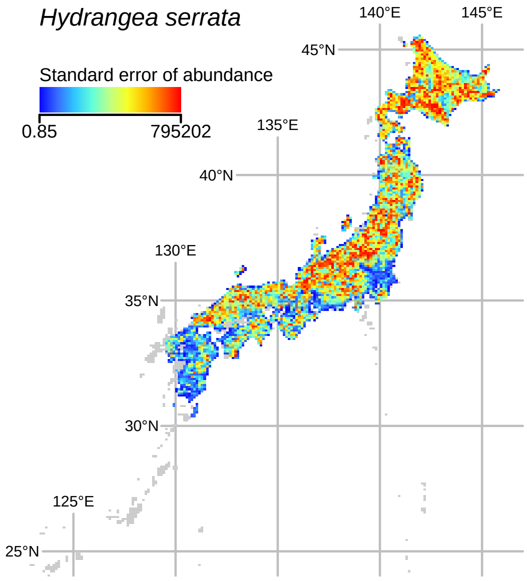
Hydrangea serrata
Standard error of abundance
0.85
795202
140°E
145°E
45°N
135°E
40°N
130°E
35°N
30°N
125°E
25°N

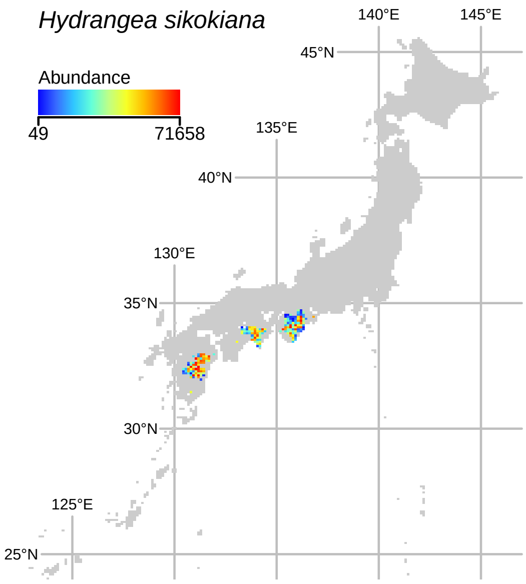
Hydrangea sikokiana
Abundance
49
71658
140°E
145°E
45°N
135°E
40°N
130°E
35°N
30°N
125°E
25°N

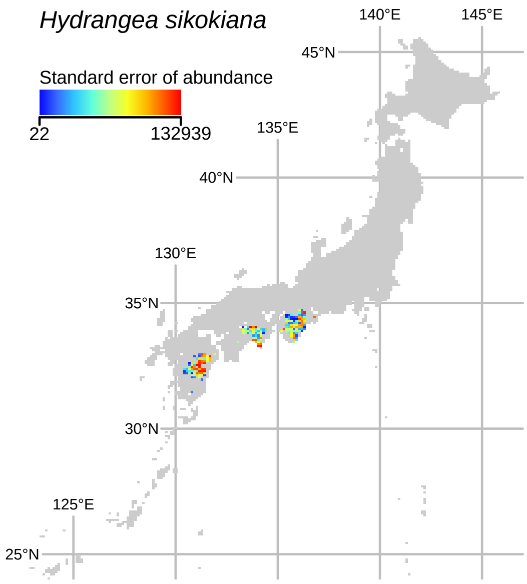
Hydrangea sikokiana
Standard error of abundance
22
132939
140°E
145°E
45°N
135°E
40°N
130°E
35°N
30°N
125°E
25°N

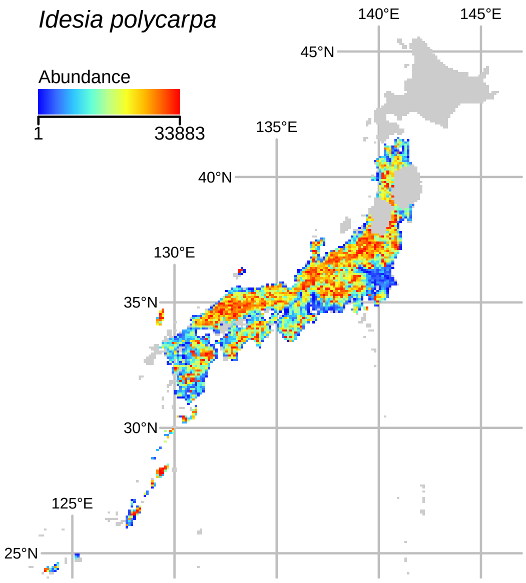
Idesia polycarpa
Abundance
1
33883
140°E
145°E
45°N
135°E
40°N
130°E
35°N
30°N
125°E
25°N

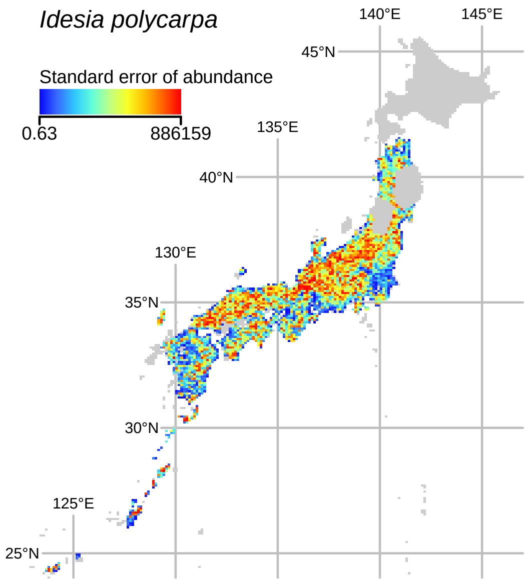
Idesia polycarpa
Standard error of abundance
0.63
886159
140°E
145°E
45°N
135°E
40°N
130°E
35°N
30°N
125°E
25°N

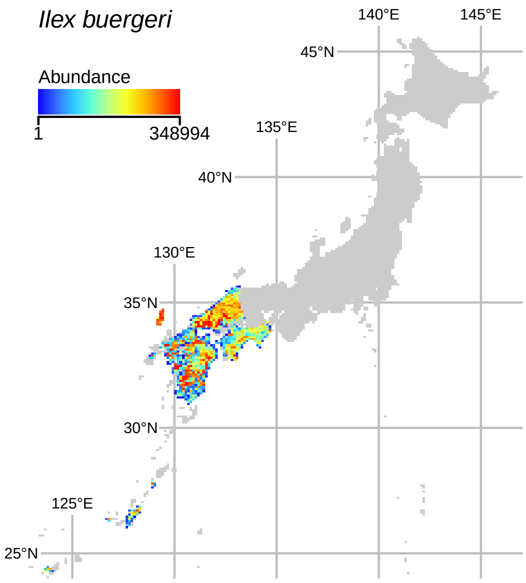
Ilex buergeri
Abundance
1
348994
140°E
145°E
45°N
135°E
40°N
130°E
35°N
30°N
125°E
25°N

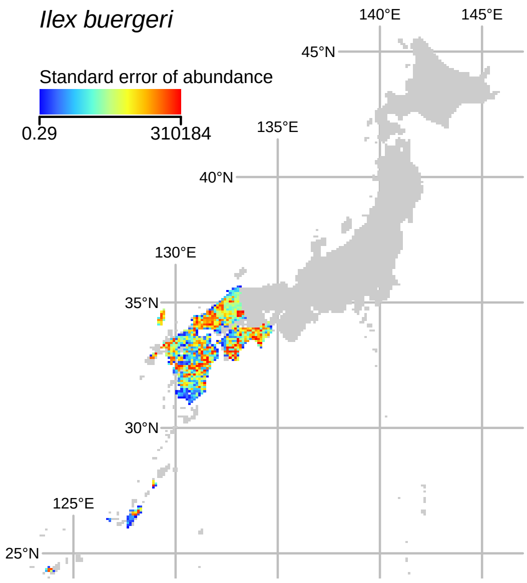
Ilex buergeri
Standard error of abundance
0.29
310184
140°E
145°E
45°N
135°E
40°N
130°E
35°N
30°N
125°E
25°N

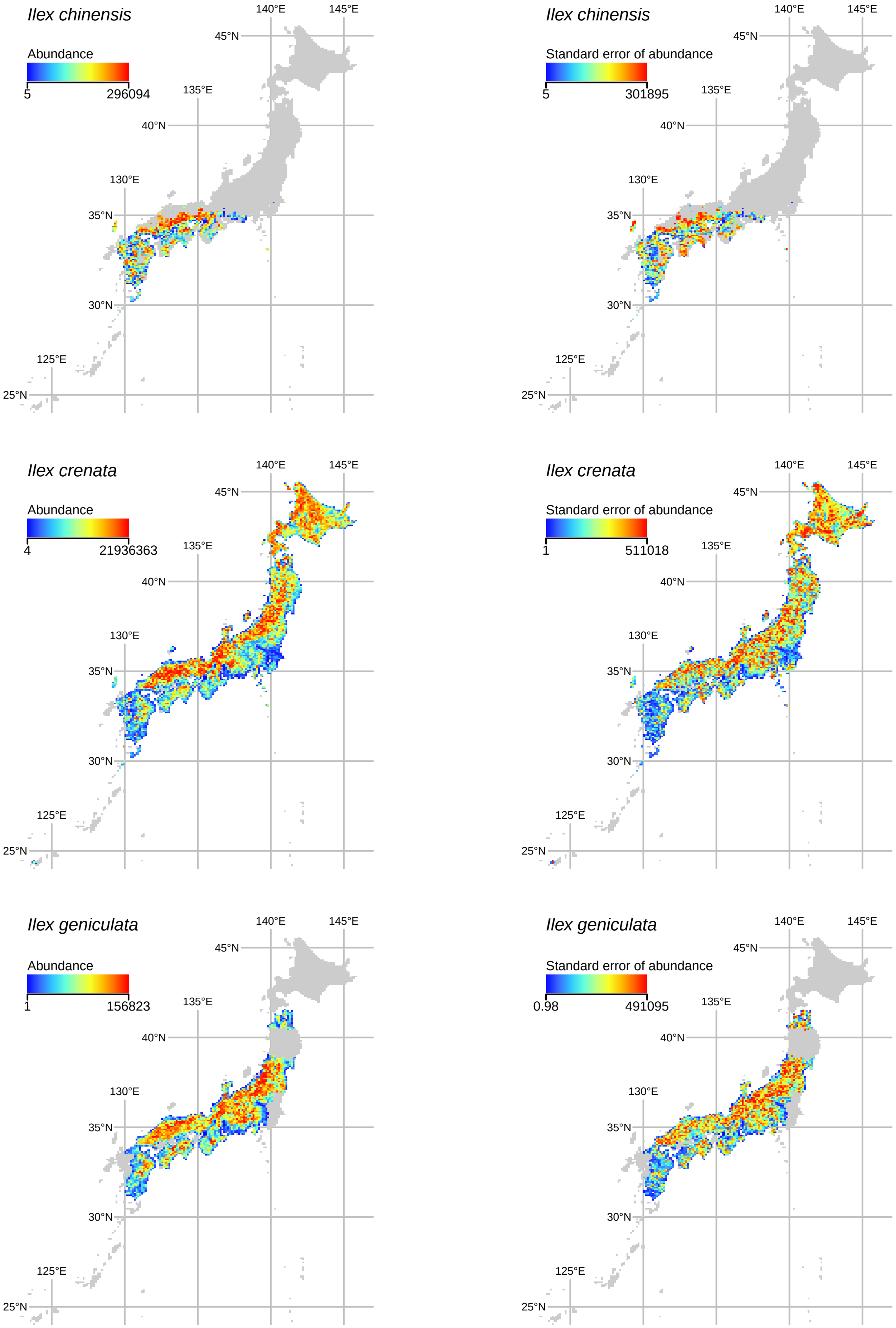

Ilex chinensis
Abundance
5
296094
Ilex chinensis
Standard error of abundance
5
301895
Ilex crenata
Abundance
4
21936363
Ilex crenata
Standard error of abundance
1
511018
Ilex geniculata
Abundance
1
156823
Ilex geniculata
Standard error of abundance
0.98
491095
125°E
130°E
135°E
140°E
145°E
25°N
30°N
35°N
40°N
45°N

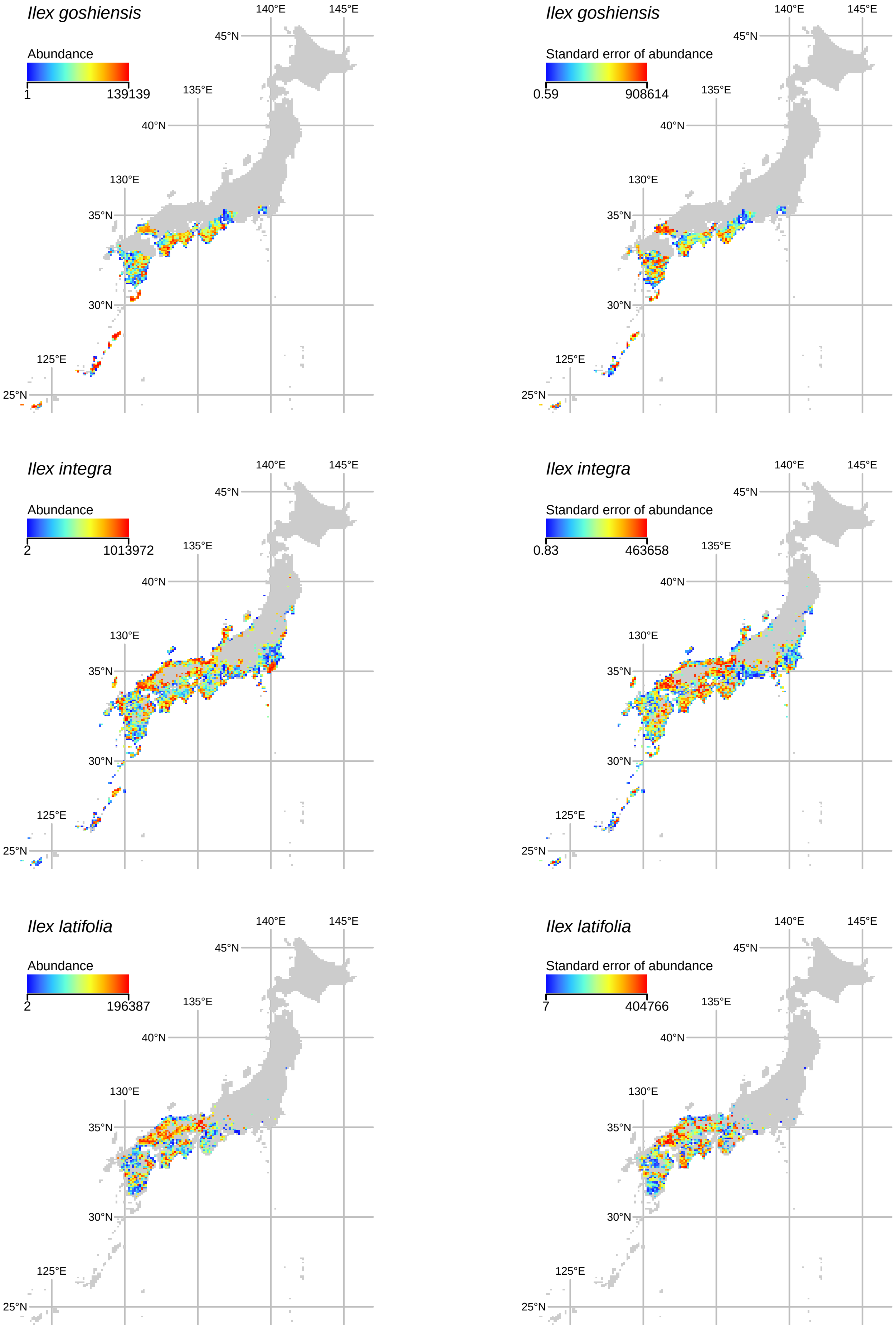
Ilex goshiensis
Abundance
1
139139
Ilex goshiensis
Standard error of abundance
0.59
908614
Ilex integra
Abundance
2
1013972
Ilex integra
Standard error of abundance
0.83
463658
Ilex latifolia
Abundance
2
196387
Ilex latifolia
Standard error of abundance
7
404766
125°E
130°E
135°E
140°E
145°E
25°N
30°N
35°N
40°N
45°N

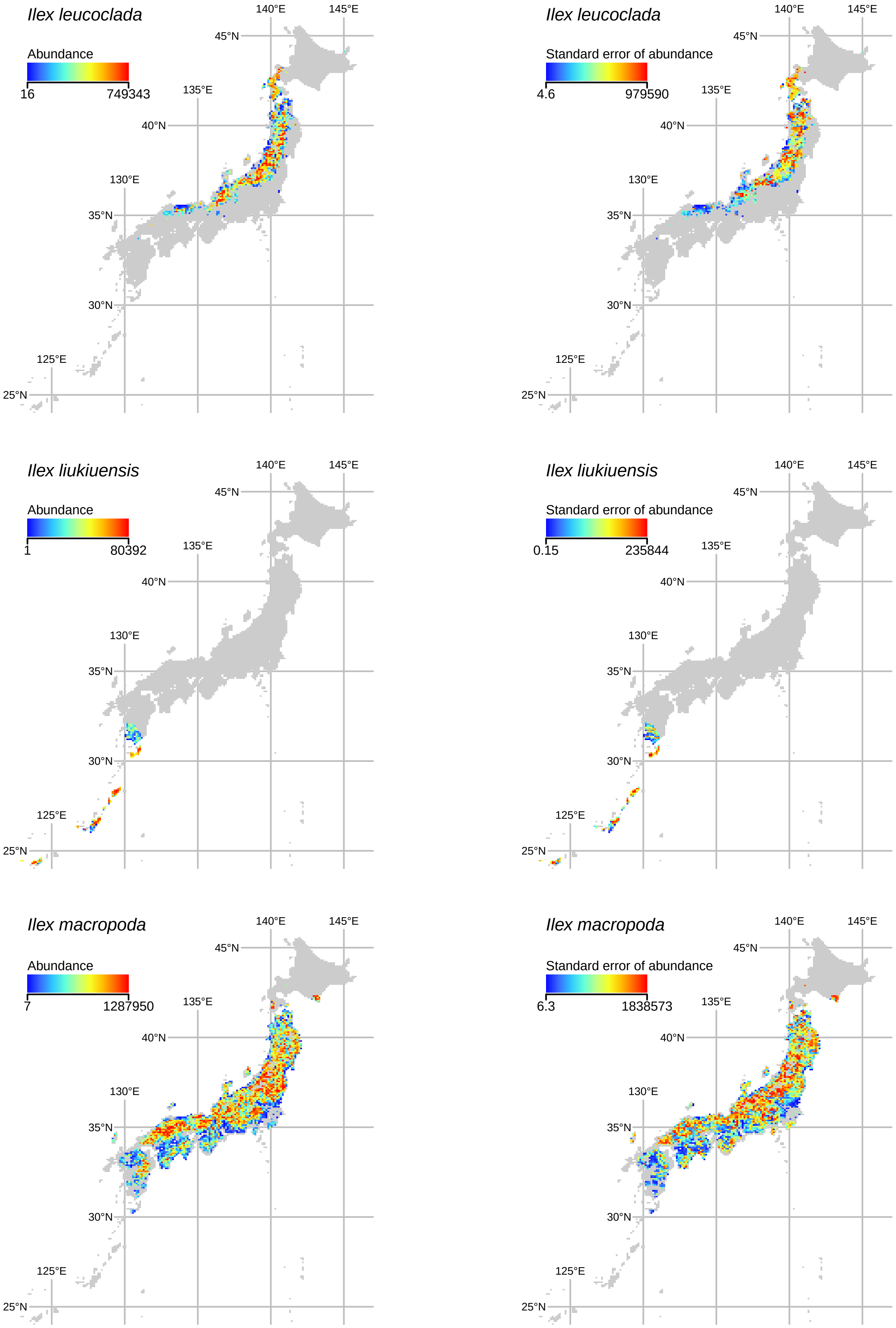
Ilex leucoclada
Abundance
16
749343
Ilex leucoclada
Standard error of abundance
4.6
979590
Ilex liukiuensis
Abundance
1
80392
Ilex liukiuensis
Standard error of abundance
0.15
235844
Ilex macropoda
Abundance
7
1287950
Ilex macropoda
Standard error of abundance
6.3
1838573
140°E
145°E
135°E
130°E
125°E
45°N
40°N
35°N
30°N
25°N

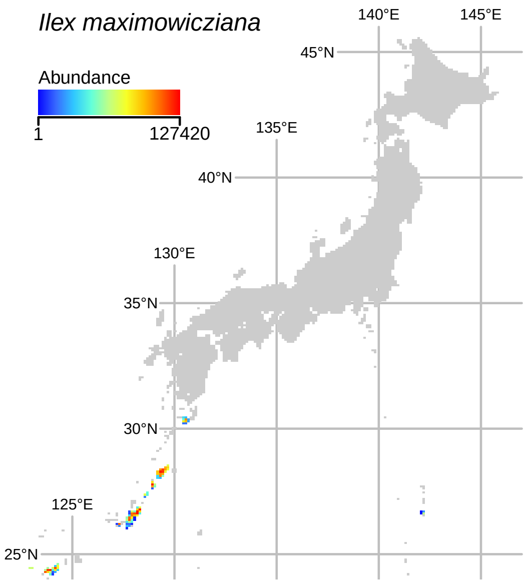
Ilex maximowicziana
Abundance
1
127420
140°E
145°E
45°N
135°E
40°N
130°E
35°N
30°N
125°E
25°N

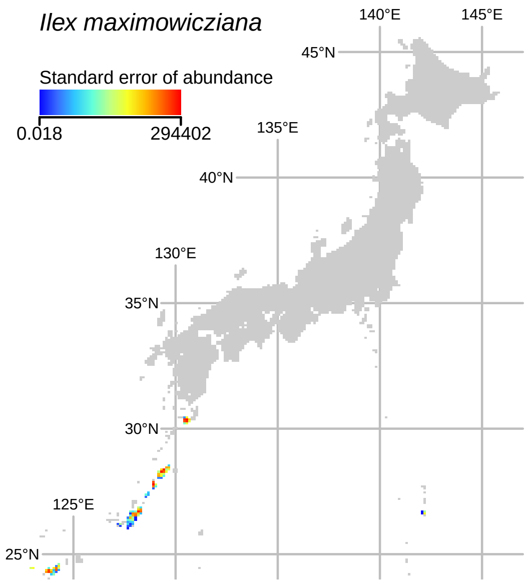
Ilex maximowicziana
Standard error of abundance
0.018
294402
140°E
145°E
45°N
135°E
40°N
130°E
35°N
30°N
125°E
25°N

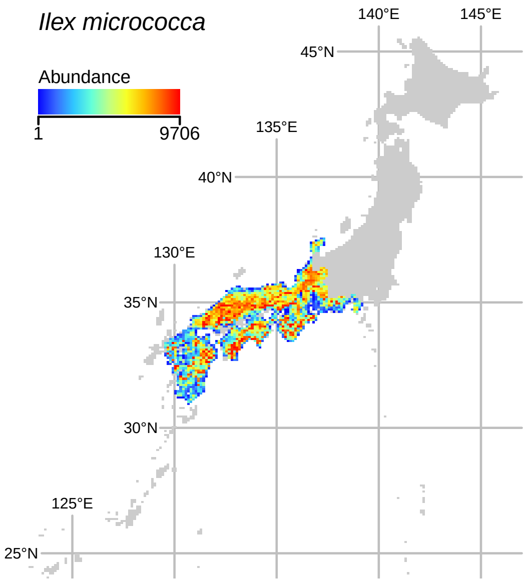
Ilex micrococca
Abundance
1
9706
140°E
145°E
45°N
135°E
40°N
130°E
35°N
30°N
125°E
25°N

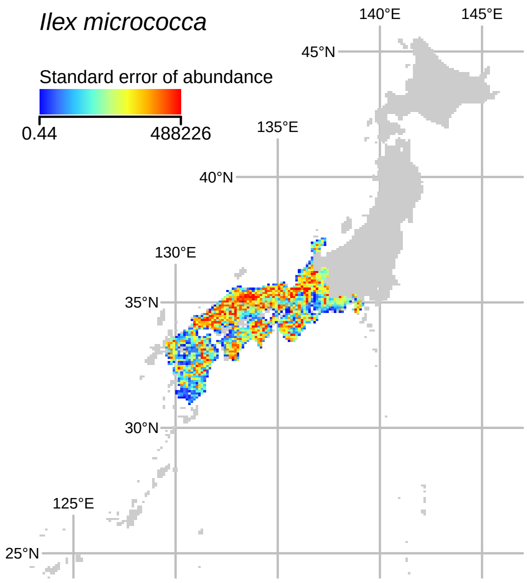
Ilex micrococca
Standard error of abundance
0.44
488226
140°E
145°E
45°N
135°E
40°N
130°E
35°N
30°N
125°E
25°N

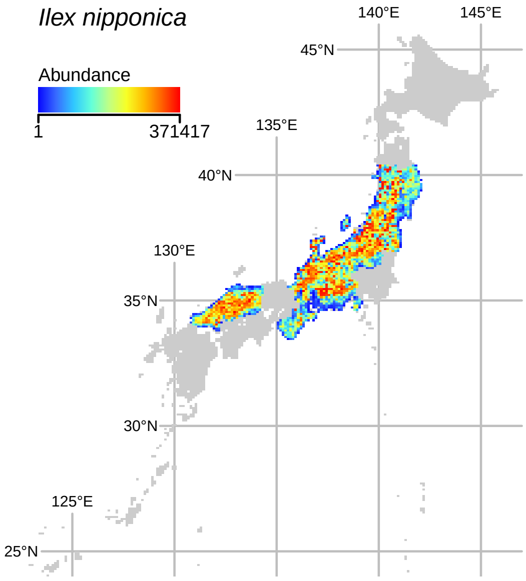
Ilex nipponica
Abundance
1
371417
140°E
145°E
45°N
135°E
40°N
130°E
35°N
30°N
125°E
25°N

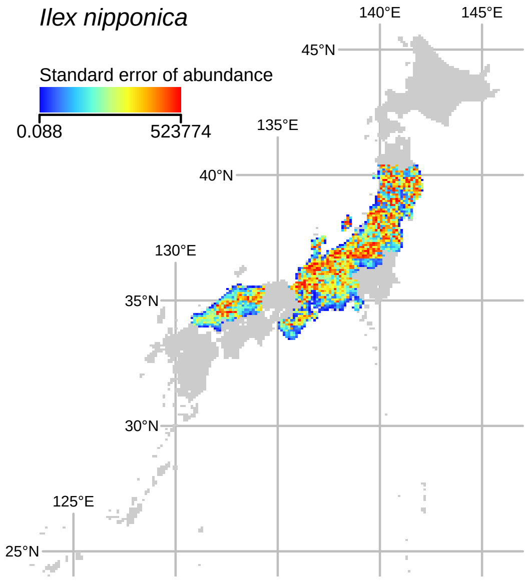
Ilex nipponica
Standard error of abundance
0.088
523774
140°E
145°E
45°N
135°E
40°N
130°E
35°N
30°N
125°E
25°N

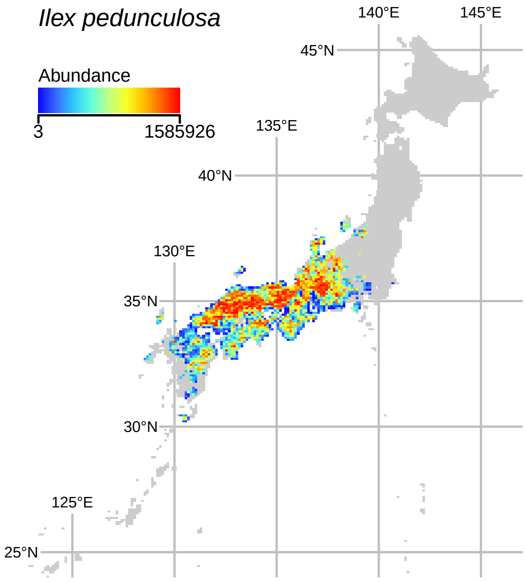
Ilex pedunculosa
Abundance
3
1585926
140°E
145°E
45°N
135°E
40°N
130°E
35°N
30°N
125°E
25°N

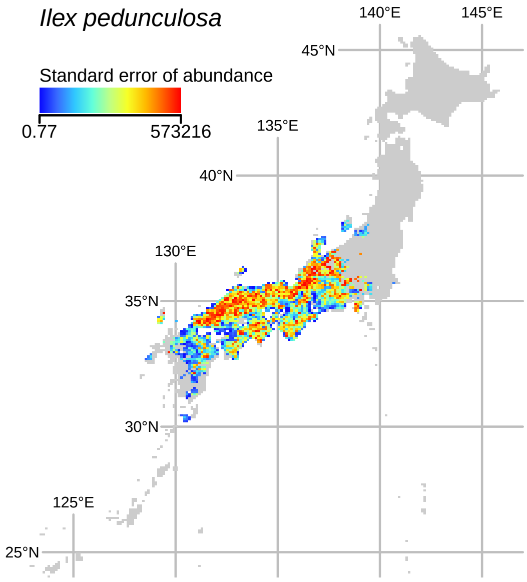
Ilex pedunculosa
Standard error of abundance
0.77
573216
140°E
145°E
45°N
135°E
40°N
130°E
35°N
30°N
125°E
25°N

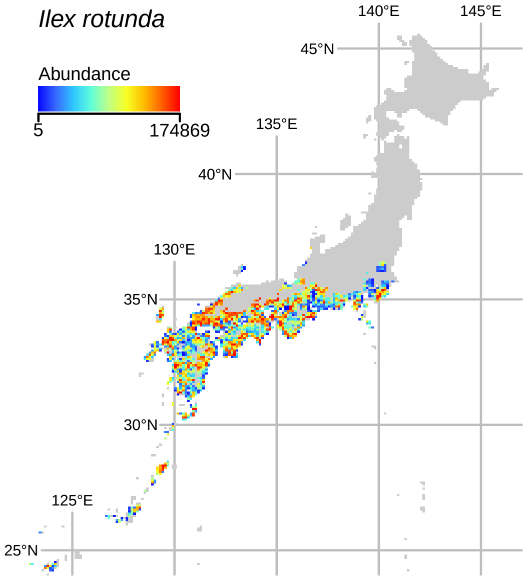
Ilex rotunda
Abundance
5
174869
140°E
145°E
45°N
135°E
40°N
130°E
35°N
30°N
125°E
25°N

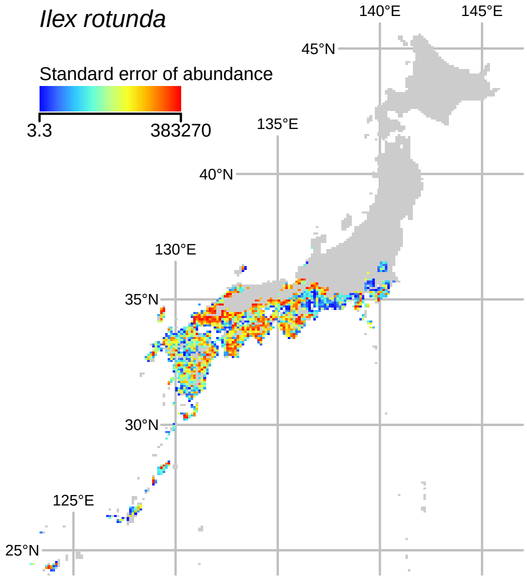
Ilex rotunda
Standard error of abundance
3.3
383270
140°E
145°E
45°N
135°E
40°N
130°E
35°N
30°N
125°E
25°N

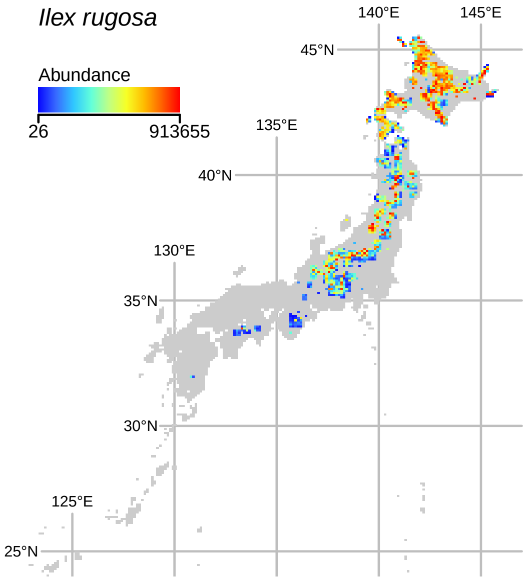
Ilex rugosa
Abundance
26
913655
140°E
145°E
45°N
135°E
40°N
130°E
35°N
30°N
125°E
25°N

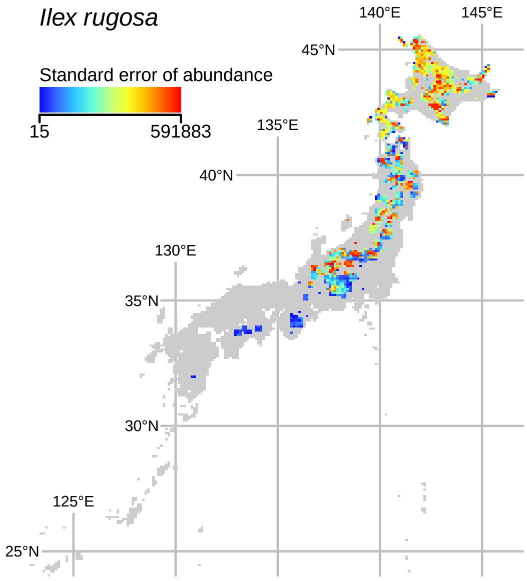
Ilex rugosa
Standard error of abundance
15
591883
140°E
145°E
45°N
135°E
40°N
130°E
35°N
30°N
125°E
25°N

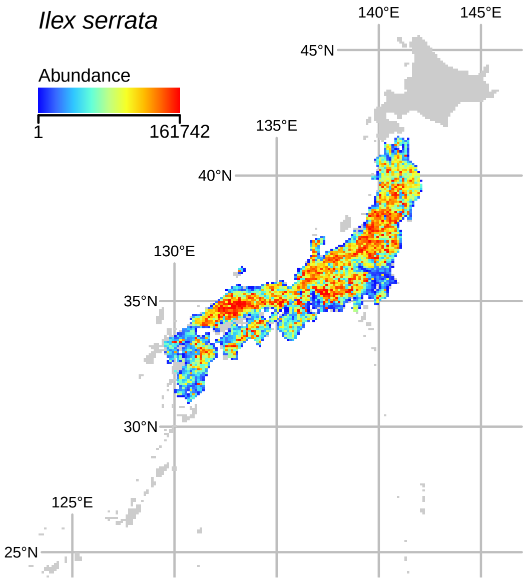
Ilex serrata
Abundance
1
161742
140°E
145°E
45°N
135°E
40°N
130°E
35°N
30°N
125°E
25°N

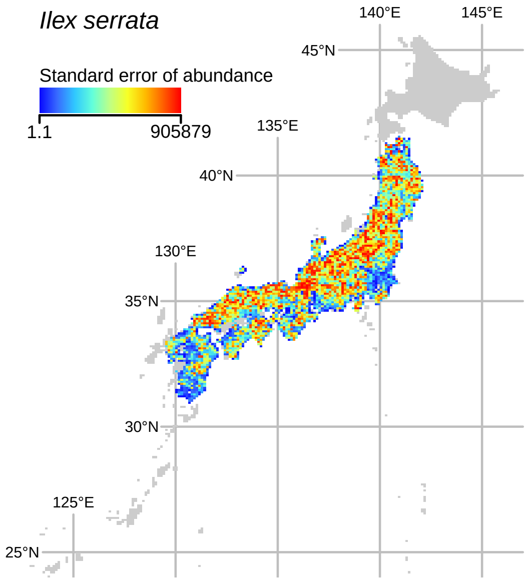
Ilex serrata
Standard error of abundance
1.1
905879
140°E
145°E
45°N
135°E
40°N
130°E
35°N
30°N
125°E
25°N

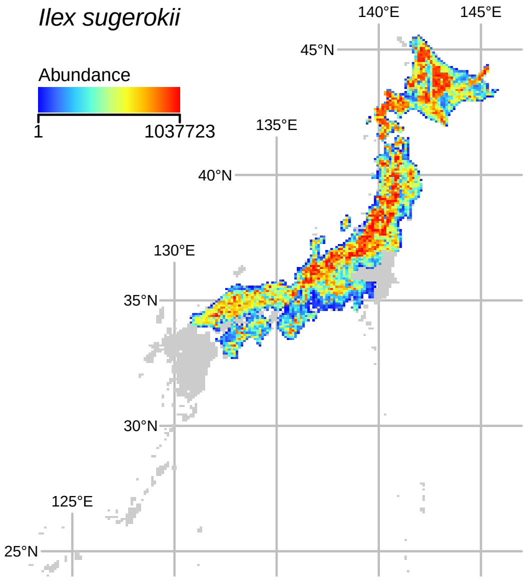
Ilex sugerokii
Abundance
1
1037723
140°E
145°E
45°N
135°E
40°N
130°E
35°N
30°N
125°E
25°N

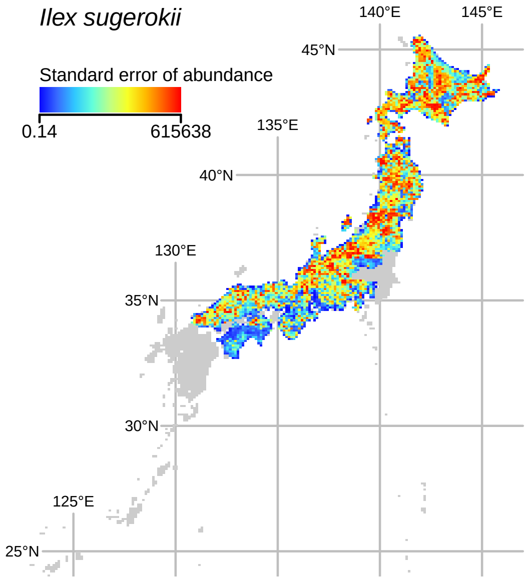
Ilex sugerokii
Standard error of abundance
0.14
615638
140°E
145°E
45°N
135°E
40°N
130°E
35°N
30°N
125°E
25°N

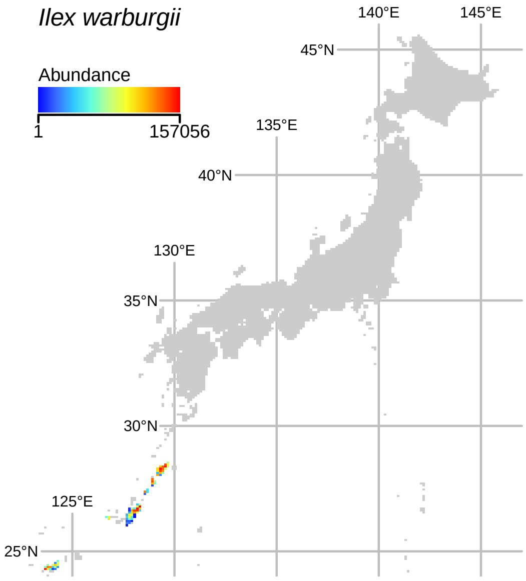
Ilex warburgii
Abundance
1
157056
140°E
145°E
45°N
135°E
40°N
130°E
35°N
30°N
125°E
25°N

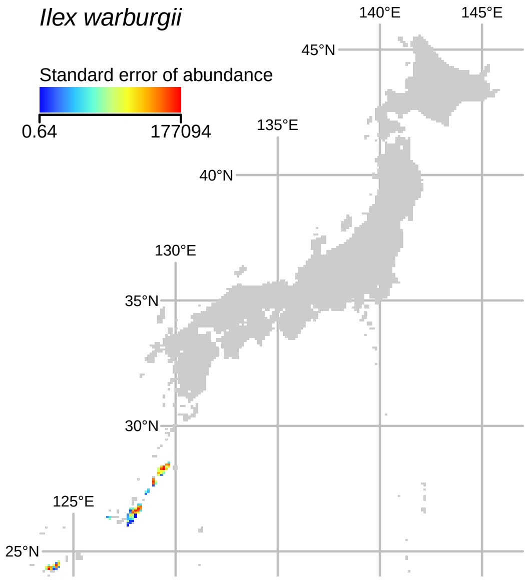
Ilex warburgii
Standard error of abundance
0.64
177094
140°E
145°E
45°N
135°E
40°N
130°E
35°N
30°N
125°E
25°N

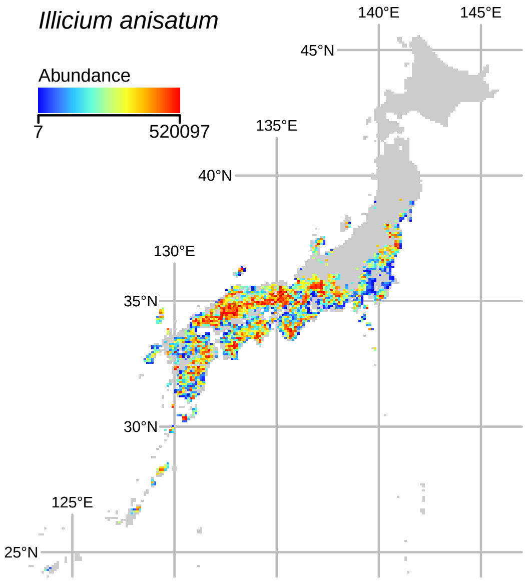
Illicium anisatum
Abundance
7
520097
140°E
145°E
45°N
135°E
40°N
130°E
35°N
30°N
125°E
25°N

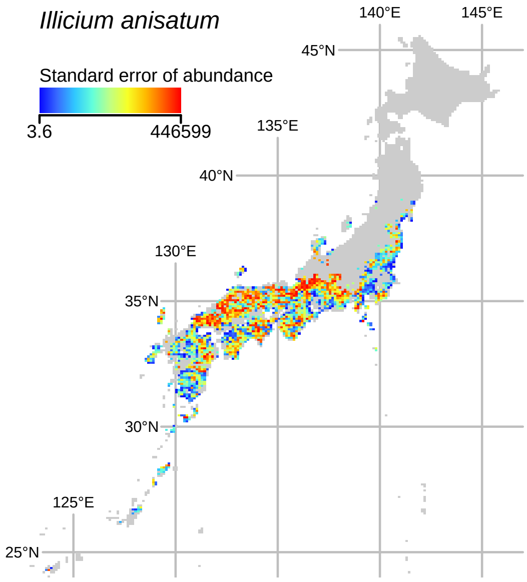
Illicium anisatum
Standard error of abundance
3.6
446599
140°E
145°E
45°N
135°E
40°N
130°E
35°N
30°N
125°E
25°N

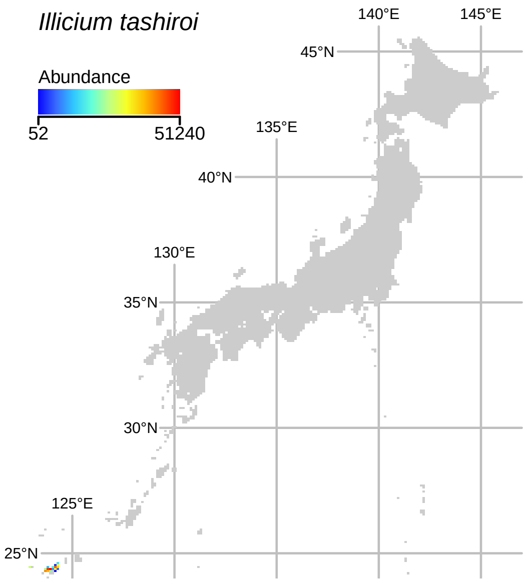
Illicium tashiroi
Abundance
52
51240
140°E
145°E
45°N
135°E
40°N
130°E
35°N
30°N
125°E
25°N

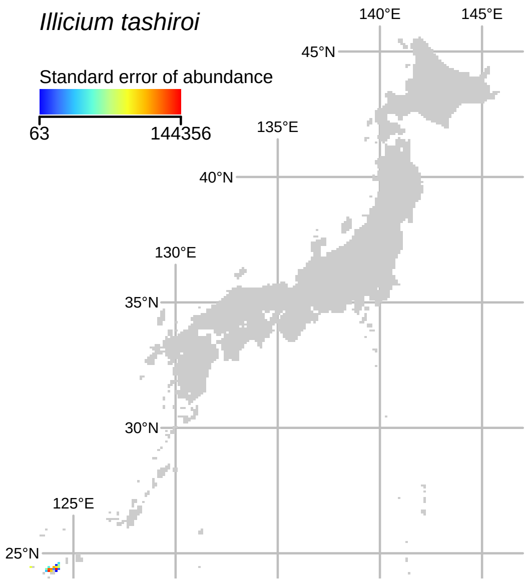
Illicium tashiroi
Standard error of abundance
63
144356
140°E
145°E
45°N
135°E
40°N
130°E
35°N
30°N
125°E
25°N

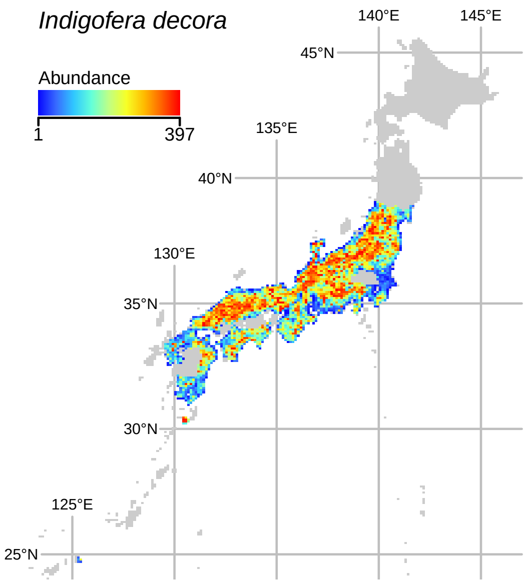
Indigofera decora
Abundance
1
397
140°E
145°E
45°N
135°E
40°N
130°E
35°N
30°N
125°E
25°N

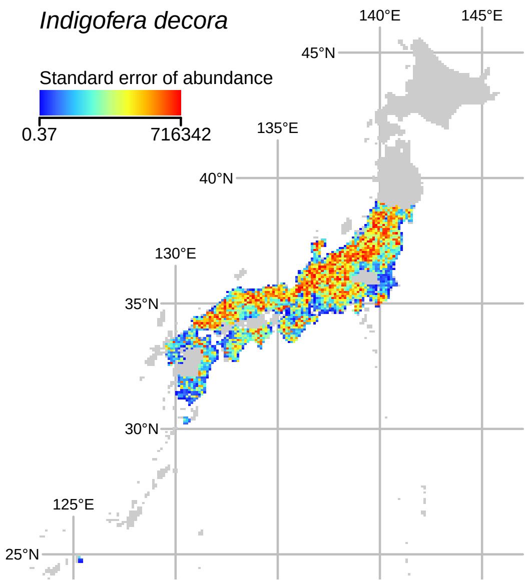
Indigofera decora
Standard error of abundance
0.37
716342
140°E
145°E
45°N
135°E
40°N
130°E
35°N
30°N
125°E
25°N

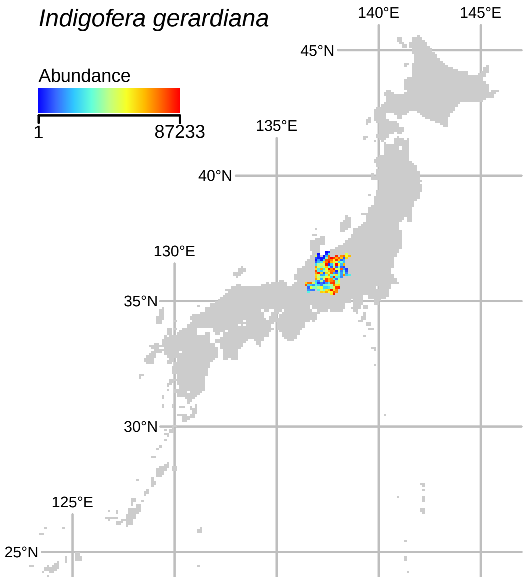
Indigofera gerardiana
Abundance
1
87233
140°E
145°E
45°N
135°E
40°N
130°E
35°N
30°N
125°E
25°N

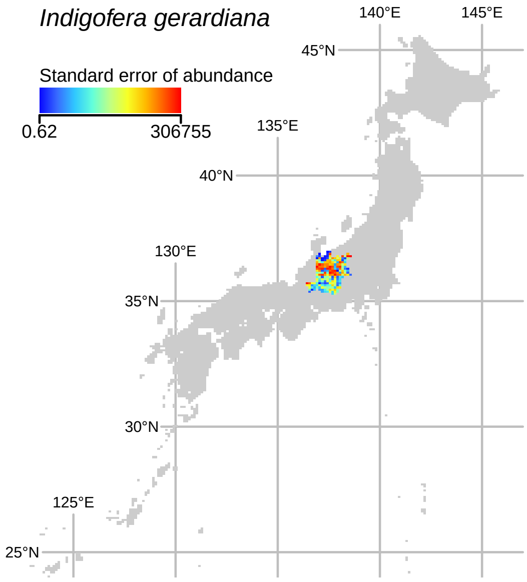
Indigofera gerardiana
Standard error of abundance
0.62
306755
140°E
145°E
45°N
135°E
40°N
130°E
35°N
30°N
125°E
25°N

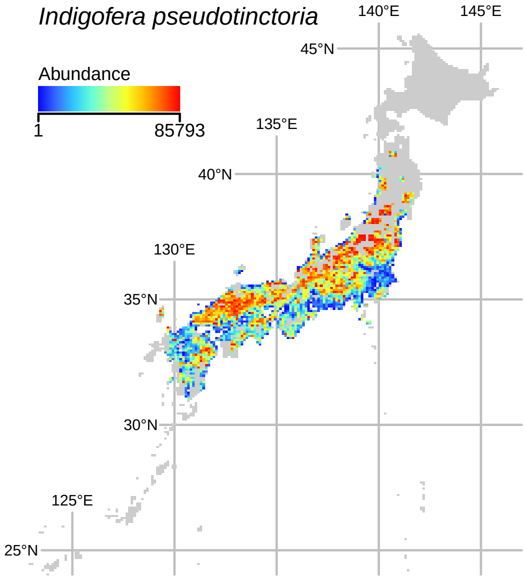
Indigofera pseudotinctoria
Abundance
1
85793
140°E
145°E
45°N
135°E
40°N
130°E
35°N
30°N
125°E
25°N

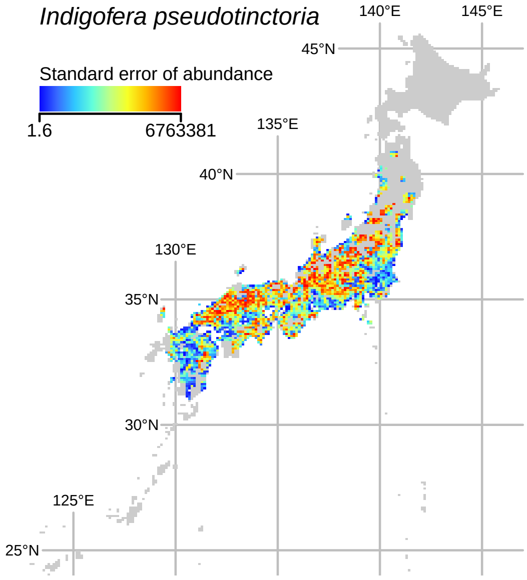
Indigofera pseudotinctoria
Standard error of abundance
1.6
6763381
140°E
145°E
45°N
135°E
40°N
130°E
35°N
30°N
125°E
25°N

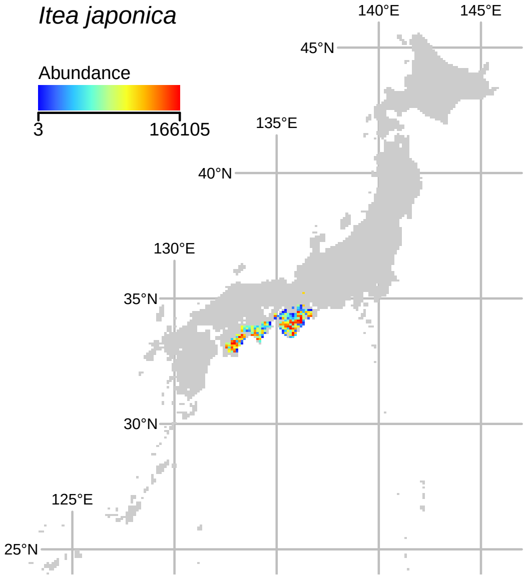
Itea japonica
Abundance
3
166105
140°E
145°E
45°N
135°E
40°N
130°E
35°N
30°N
125°E
25°N

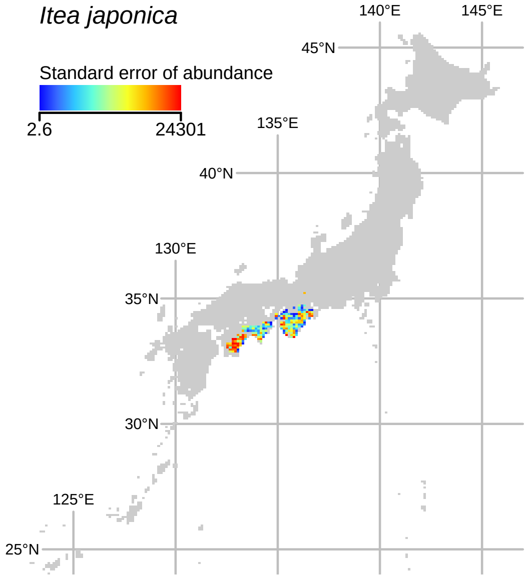
Itea japonica
Standard error of abundance
2.6
24301
140°E
145°E
45°N
135°E
40°N
130°E
35°N
30°N
125°E
25°N

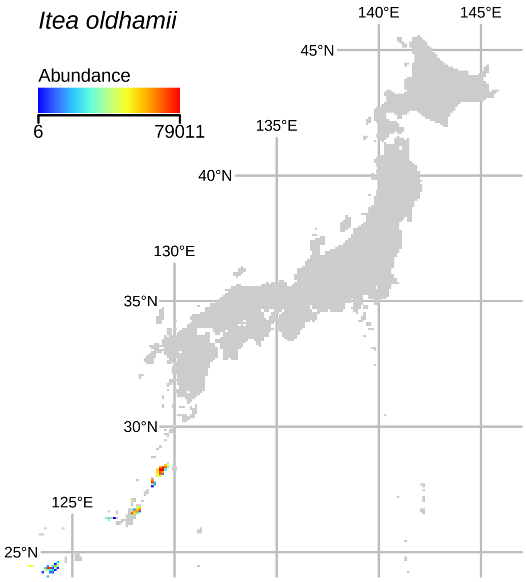
Itea oldhamii
Abundance
6
79011
140°E
145°E
45°N
135°E
40°N
130°E
35°N
30°N
125°E
25°N

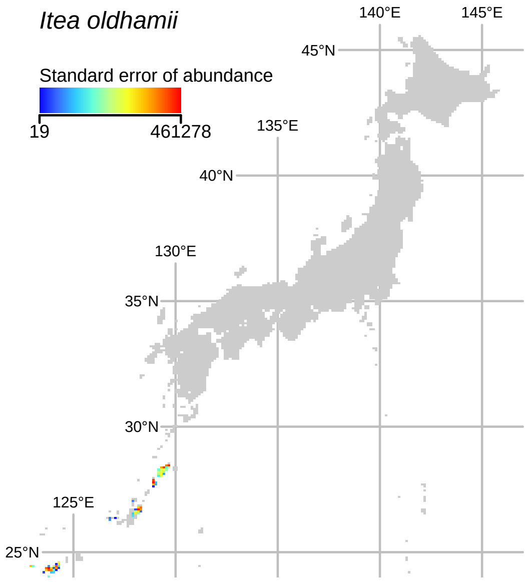
Itea oldhamii
Standard error of abundance
19
461278
140°E
145°E
45°N
135°E
40°N
130°E
35°N
30°N
125°E
25°N

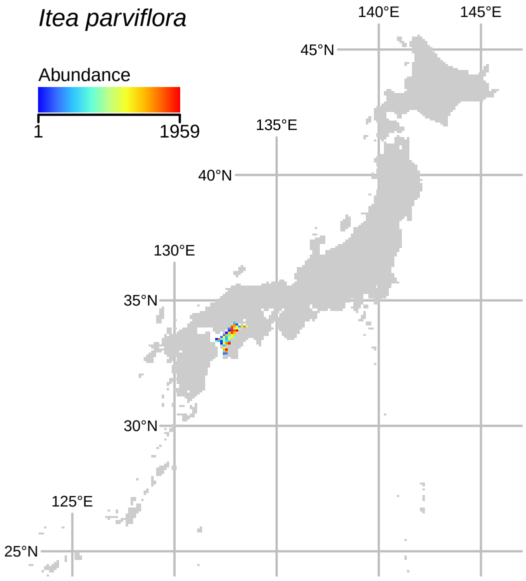
Itea parviflora
Abundance
1
1959
140°E
145°E
45°N
135°E
40°N
130°E
35°N
30°N
125°E
25°N

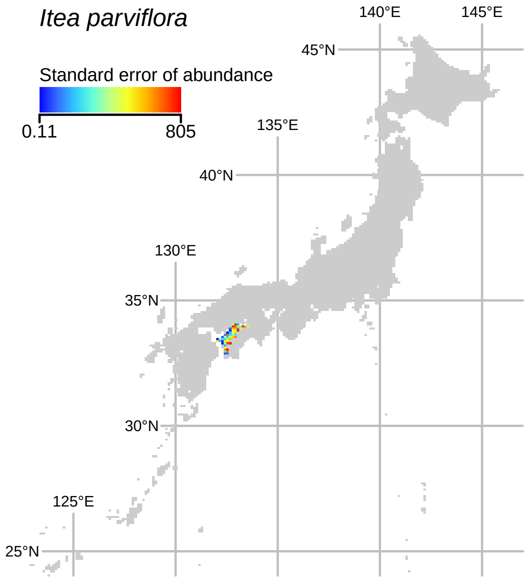
Itea parviflora
Standard error of abundance
0.11
805
140°E
145°E
45°N
135°E
40°N
130°E
35°N
30°N
125°E
25°N

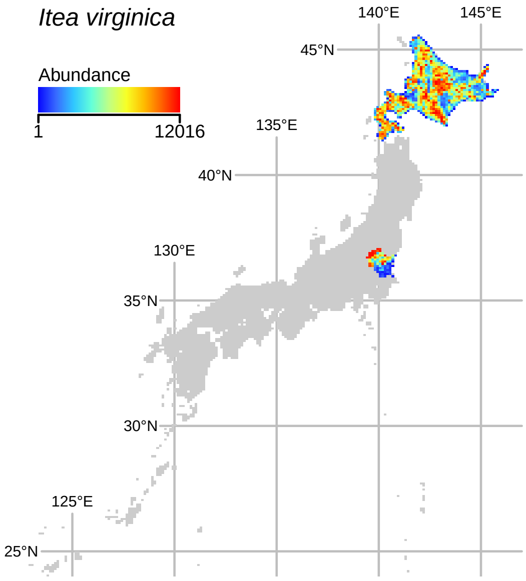
Itea virginica
Abundance
1
12016
140°E
145°E
45°N
135°E
40°N
130°E
35°N
30°N
125°E
25°N

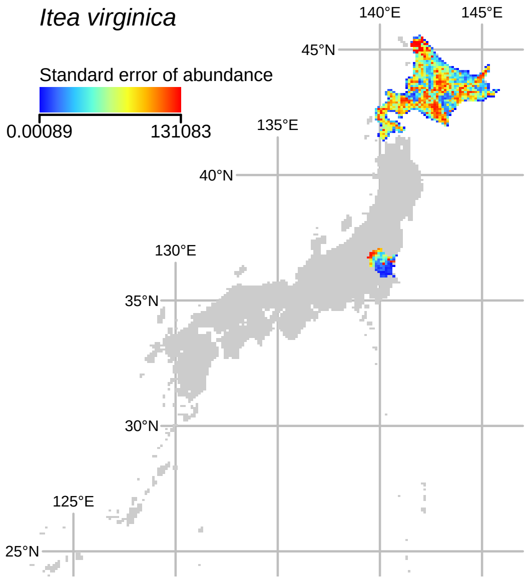
Itea virginica
Standard error of abundance
0.00089
131083
140°E
145°E
45°N
135°E
40°N
130°E
35°N
30°N
125°E
25°N

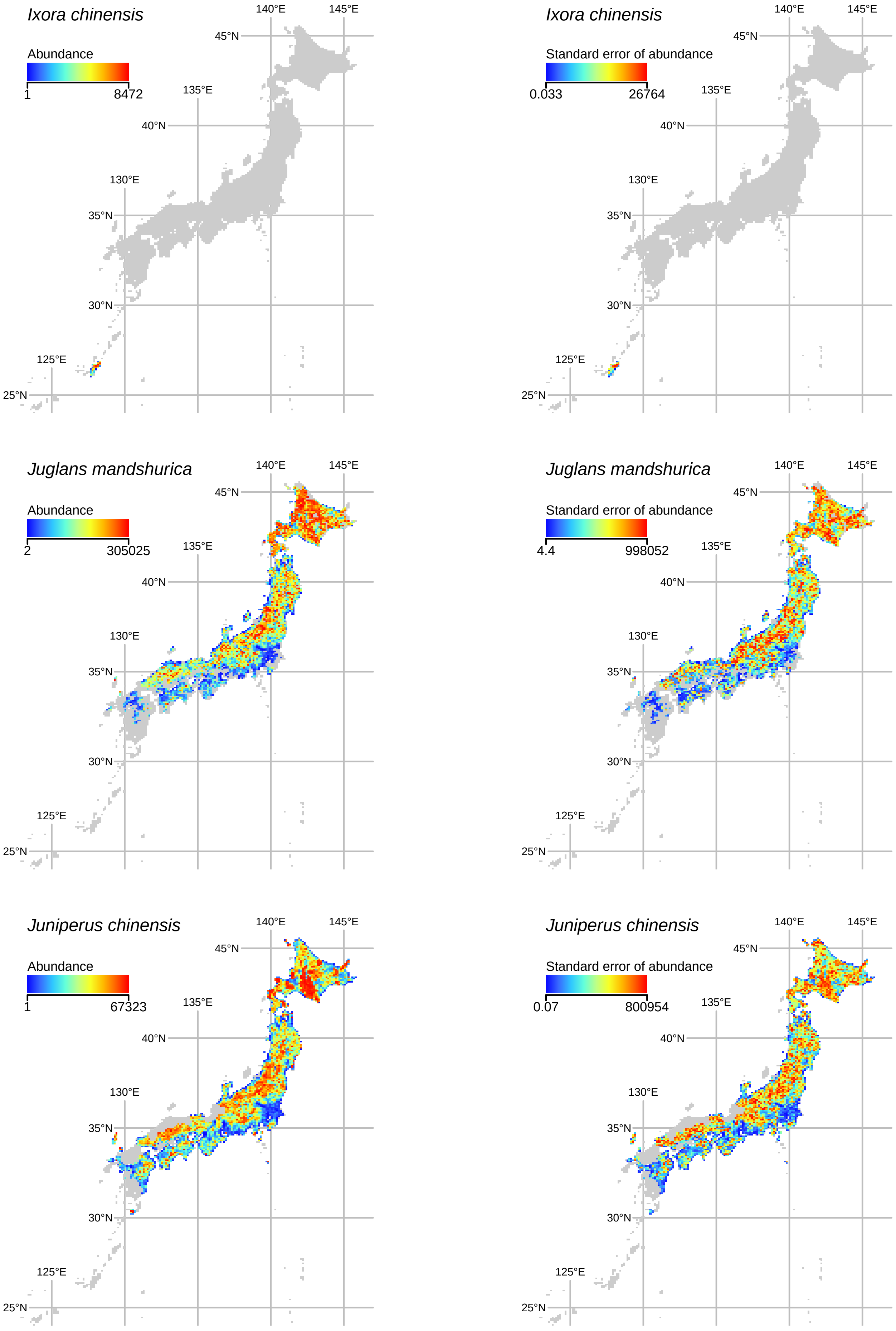
Ixora chinensis
Abundance
1
8472
Ixora chinensis
Standard error of abundance
0.033
26764
Juglans mandshurica
Abundance
2
305025
Juglans mandshurica
Standard error of abundance
4.4
998052
Juniperus chinensis
Abundance
1
67323
Juniperus chinensis
Standard error of abundance
0.07
800954
125°E
130°E
135°E
140°E
145°E
25°N
30°N
35°N
40°N
45°N

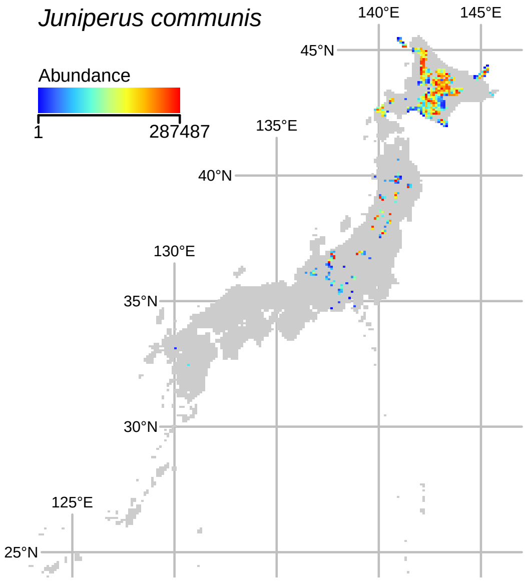
Juniperus communis
Abundance
1
287487
140°E
145°E
45°N
135°E
40°N
130°E
35°N
30°N
125°E
25°N

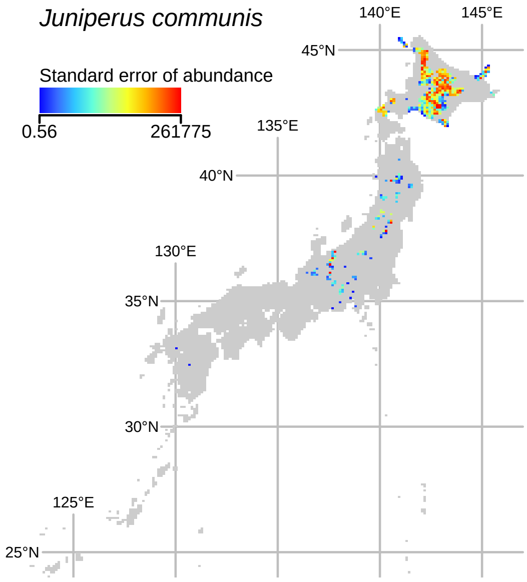
Juniperus communis
Standard error of abundance
0.56
261775
140°E
145°E
45°N
135°E
40°N
130°E
35°N
30°N
125°E
25°N

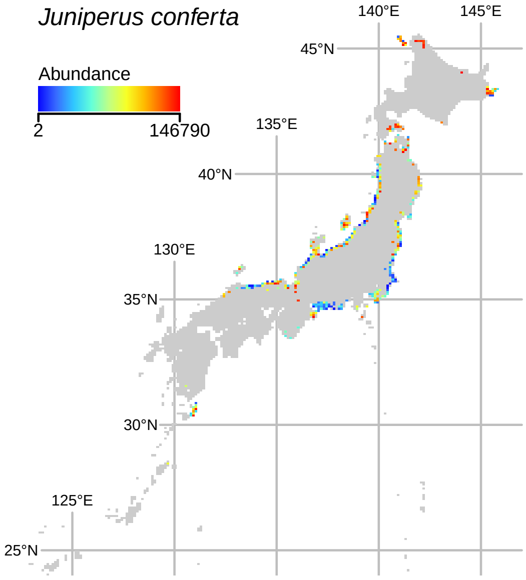
Juniperus conferta
Abundance
2
146790
140°E
145°E
45°N
135°E
40°N
130°E
35°N
30°N
125°E
25°N

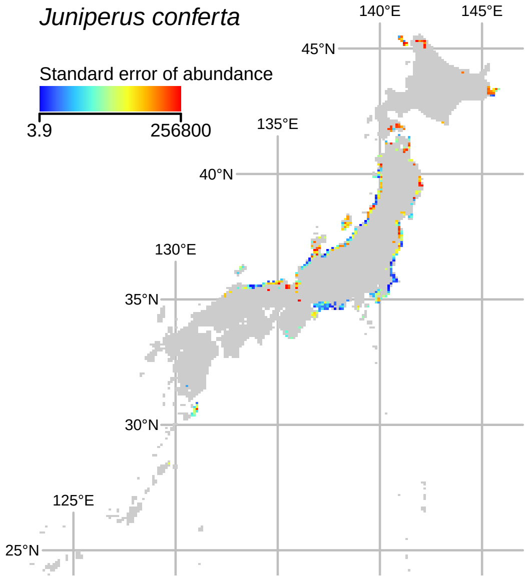
Juniperus conferta
Standard error of abundance
3.9
256800
140°E
145°E
45°N
135°E
40°N
130°E
35°N
30°N
125°E
25°N

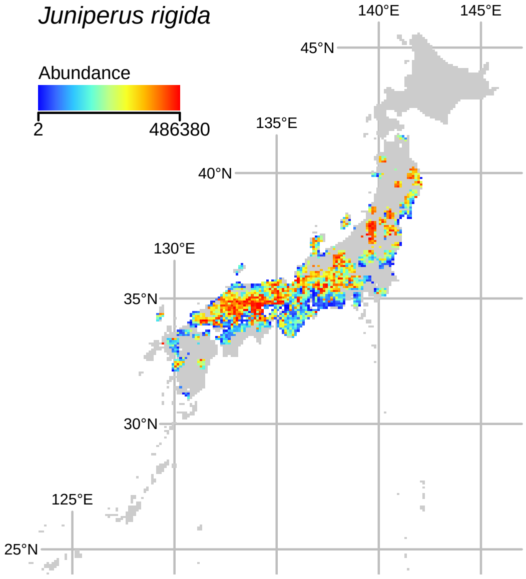
Juniperus rigida
Abundance
2
486380
140°E
145°E
45°N
135°E
40°N
130°E
35°N
30°N
125°E
25°N

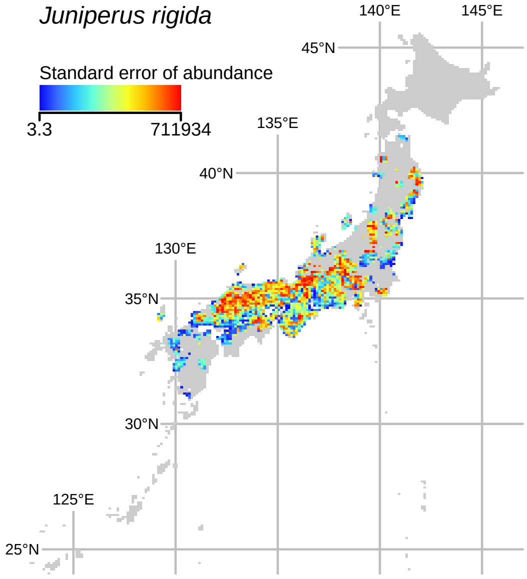
Juniperus rigida
Standard error of abundance
3.3
711934
140°E
145°E
45°N
135°E
40°N
130°E
35°N
30°N
125°E
25°N

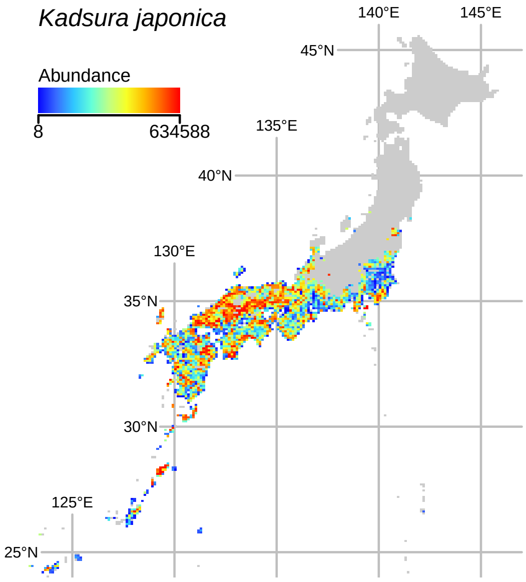
Kadsura japonica
Abundance
8
634588
140°E
145°E
45°N
135°E
40°N
130°E
35°N
30°N
125°E
25°N

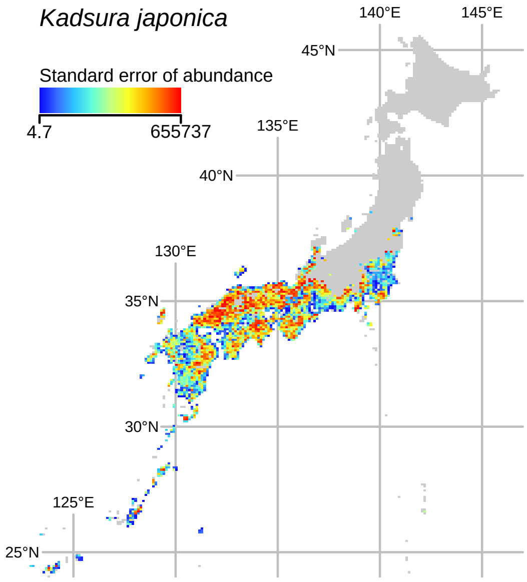
Kadsura japonica
Standard error of abundance
4.7
655737
140°E
145°E
45°N
135°E
40°N
130°E
35°N
30°N
125°E
25°N

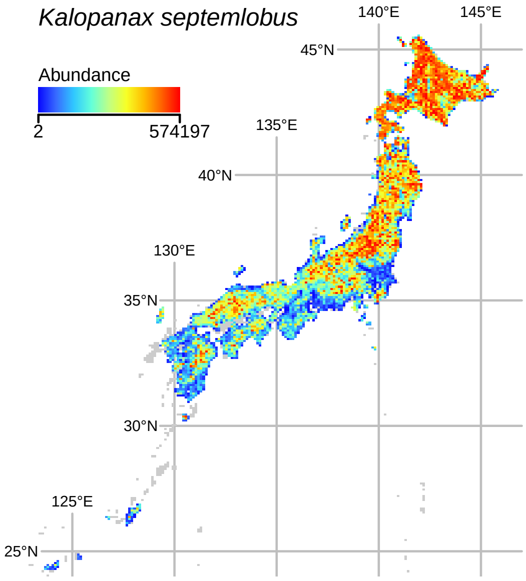
Kalopanax septemlobus
Abundance
2
574197
140°E
145°E
45°N
135°E
40°N
130°E
35°N
30°N
125°E
25°N

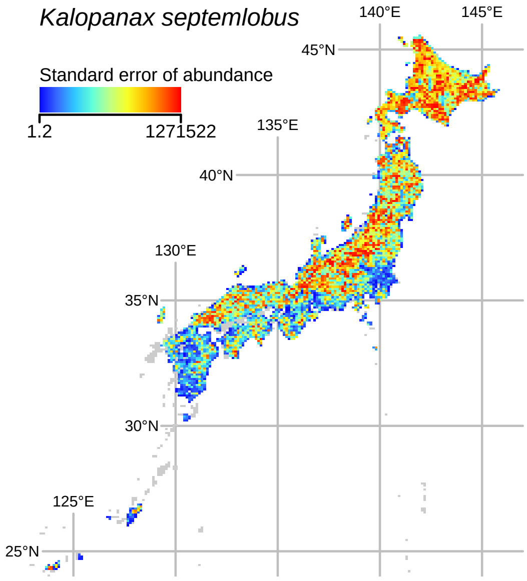
Kalopanax septemlobus
Standard error of abundance
1.2
1271522
140°E
145°E
45°N
135°E
40°N
130°E
35°N
30°N
125°E
25°N

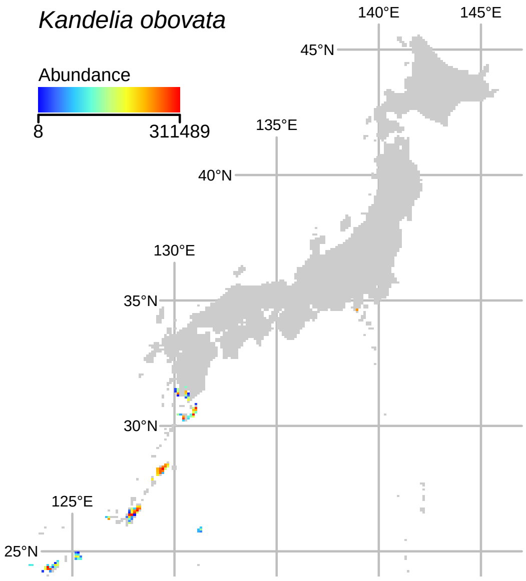
Kandelia obovata
Abundance
8
311489
140°E
145°E
45°N
135°E
40°N
130°E
35°N
30°N
125°E
25°N

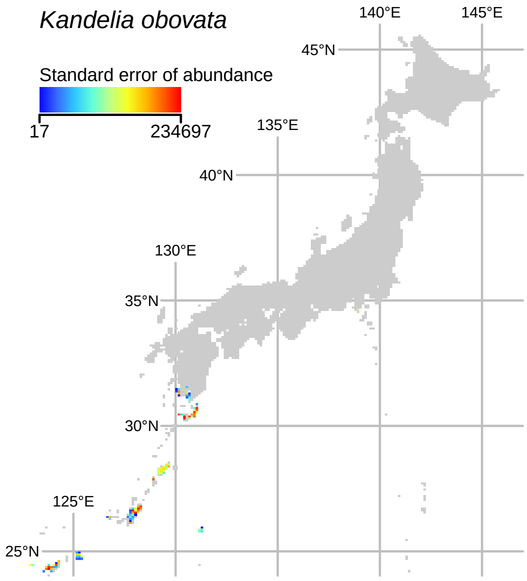
Kandelia obovata
Standard error of abundance
17
234697
140°E
145°E
45°N
135°E
40°N
130°E
35°N
30°N
125°E
25°N

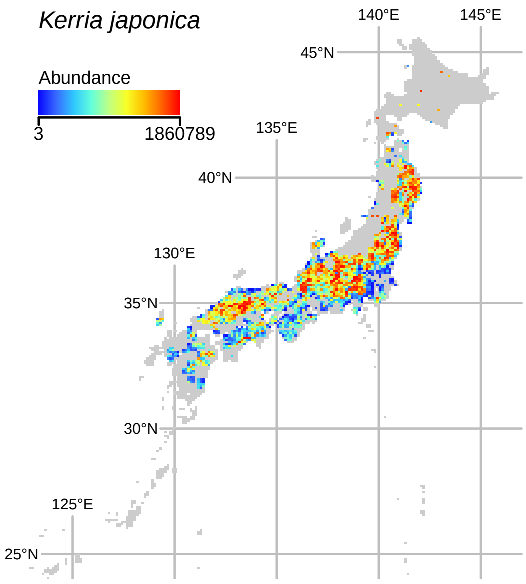
Kerria japonica
Abundance
3
1860789
140°E
145°E
135°E
130°E
125°E
45°N
40°N
35°N
30°N
25°N

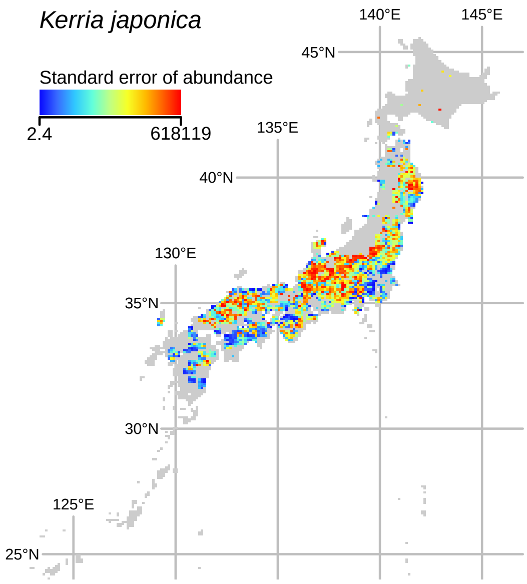
Kerria japonica
Standard error of abundance
2.4
618119
140°E
145°E
135°E
130°E
125°E
45°N
40°N
35°N
30°N
25°N

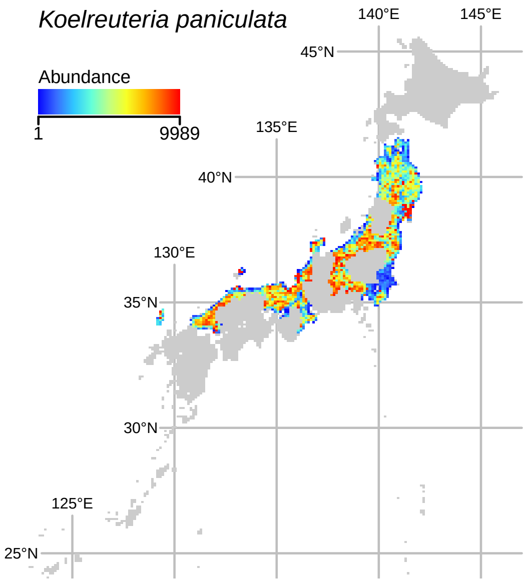
Koelreuteria paniculata
Abundance
1
9989
140°E
145°E
135°E
130°E
125°E
45°N
40°N
35°N
30°N
25°N

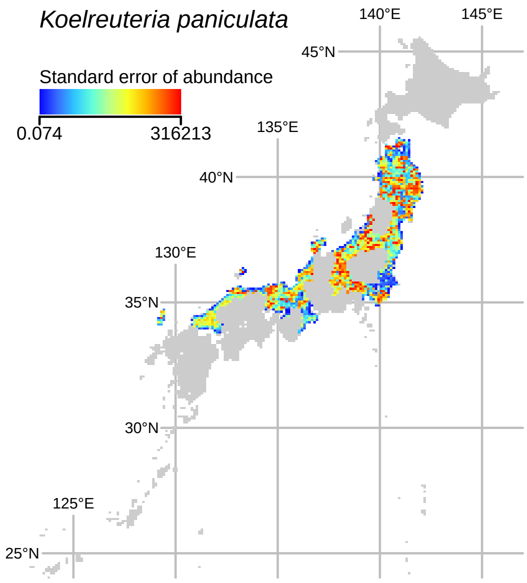
Koelreuteria paniculata
Standard error of abundance
0.074
316213
140°E
145°E
135°E
130°E
125°E
45°N
40°N
35°N
30°N
25°N

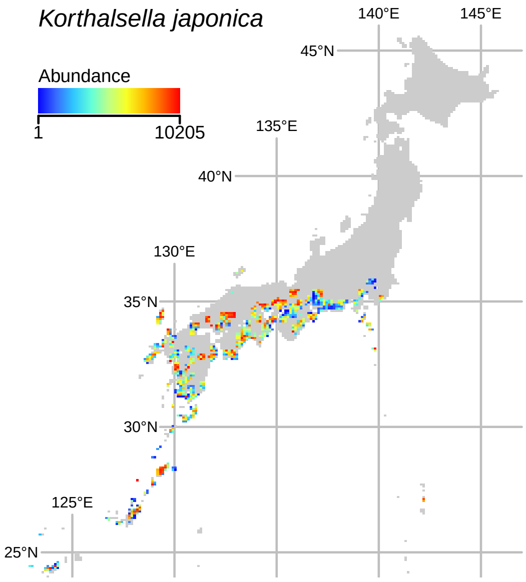
Korthalsella japonica
Abundance
1
10205
140°E
145°E
135°E
130°E
125°E
45°N
40°N
35°N
30°N
25°N

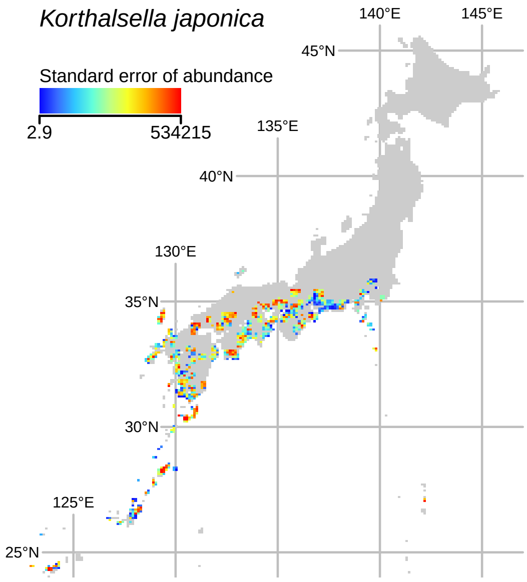
Korthalsella japonica
Standard error of abundance
2.9
534215
140°E
145°E
135°E
130°E
125°E
45°N
40°N
35°N
30°N
25°N

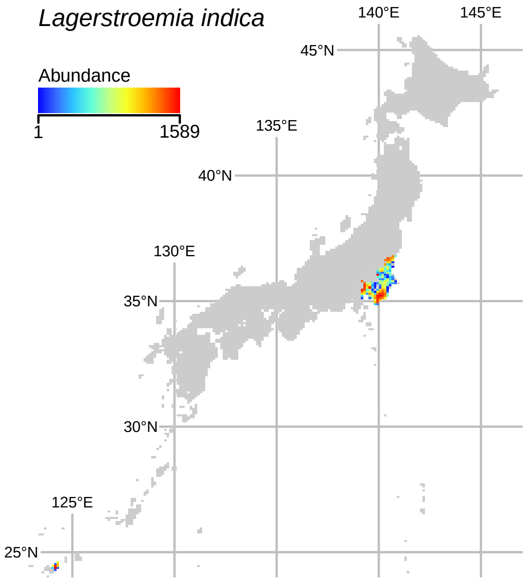
Lagerstroemia indica
Abundance
1
1589
140°E
145°E
45°N
135°E
40°N
130°E
35°N
30°N
125°E
25°N

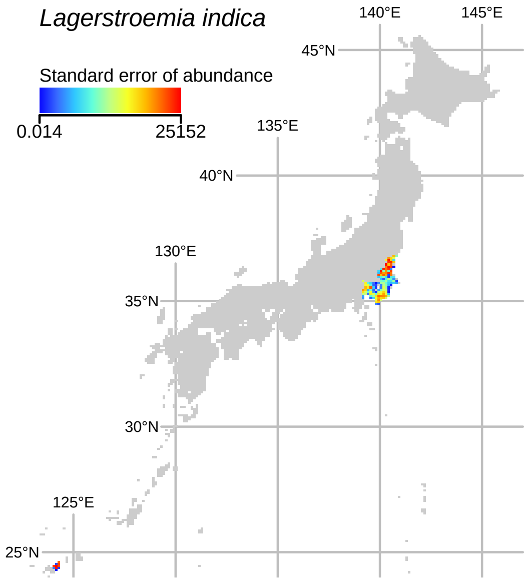
Lagerstroemia indica
Standard error of abundance
0.014
25152
140°E
145°E
45°N
135°E
40°N
130°E
35°N
30°N
125°E
25°N

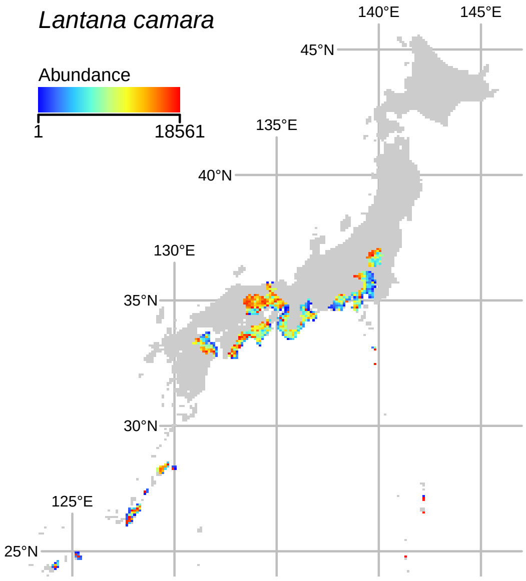
Lantana camara
Abundance
1
18561
140°E
145°E
45°N
135°E
40°N
130°E
35°N
30°N
125°E
25°N

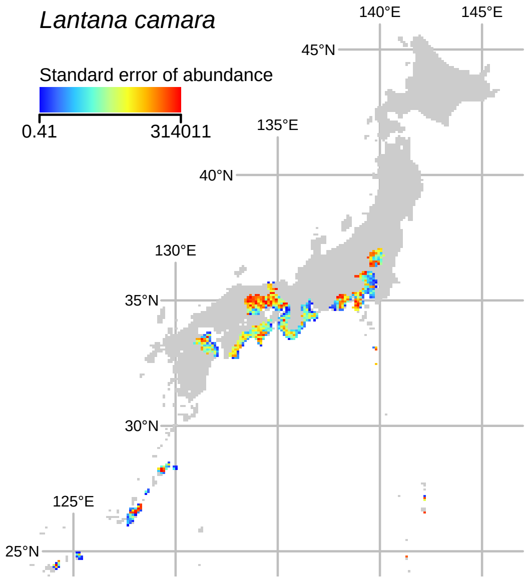
Lantana camara
Standard error of abundance
0.41
314011
140°E
145°E
45°N
135°E
40°N
130°E
35°N
30°N
125°E
25°N

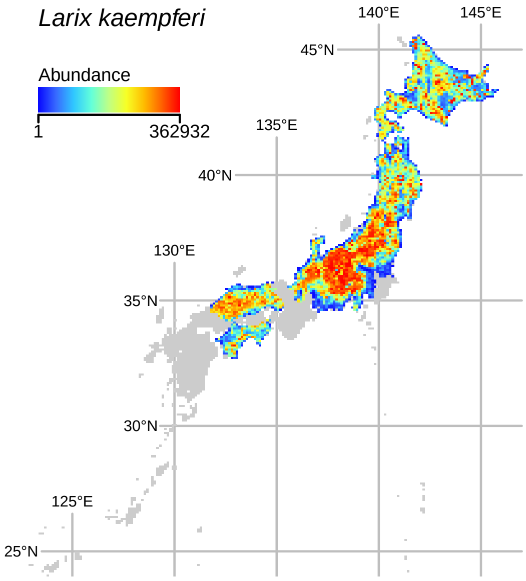
Larix kaempferi
Abundance
1
362932
140°E
145°E
45°N
135°E
40°N
130°E
35°N
30°N
125°E
25°N

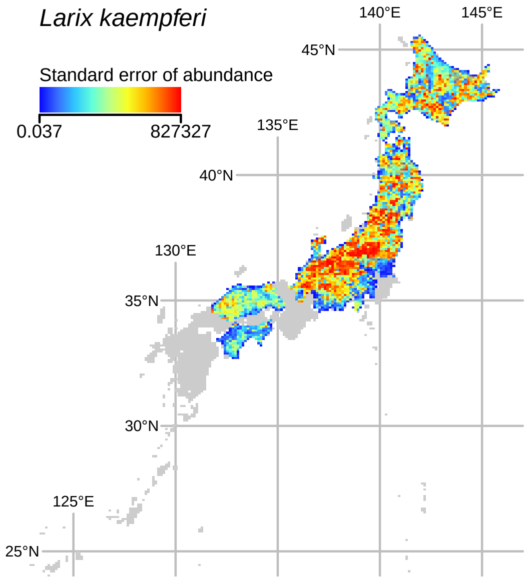
Larix kaempferi
Standard error of abundance
0.037
827327
140°E
145°E
45°N
135°E
40°N
130°E
35°N
30°N
125°E
25°N

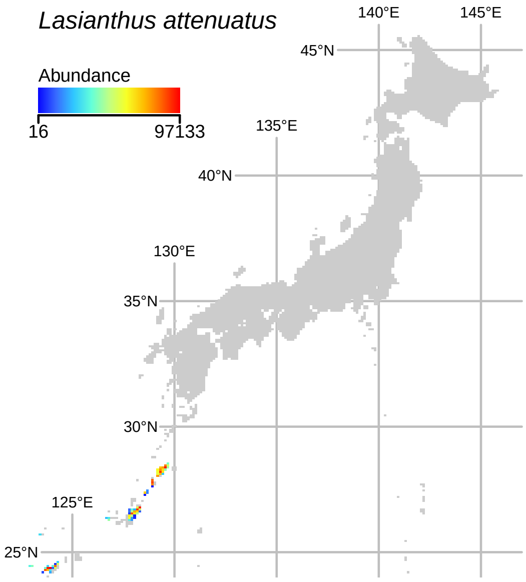
Lasianthus attenuatus
Abundance
16
97133
140°E
145°E
45°N
135°E
40°N
130°E
35°N
30°N
125°E
25°N

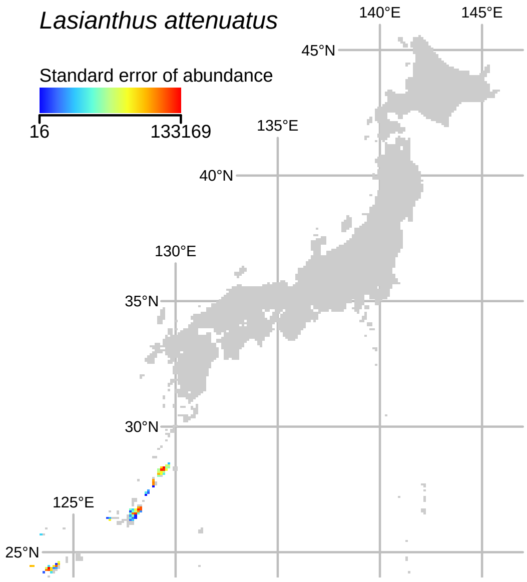
Lasianthus attenuatus
Standard error of abundance
16
133169
140°E
145°E
45°N
135°E
40°N
130°E
35°N
30°N
125°E
25°N

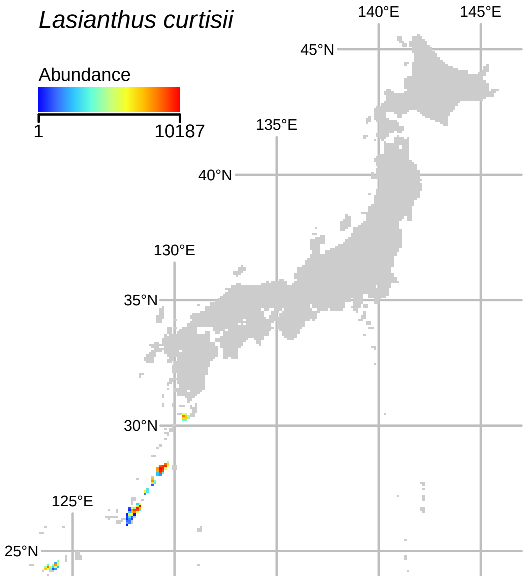
Lasianthus curtisii
Abundance
1
10187
140°E
145°E
45°N
135°E
40°N
130°E
35°N
30°N
125°E
25°N

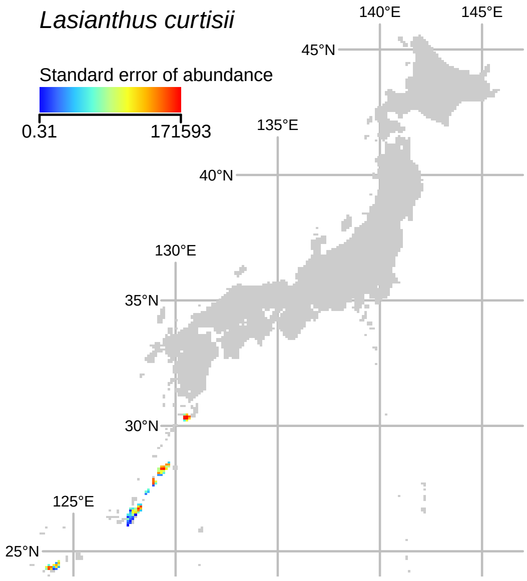
Lasianthus curtisii
Standard error of abundance
0.31
171593
140°E
145°E
45°N
135°E
40°N
130°E
35°N
30°N
125°E
25°N

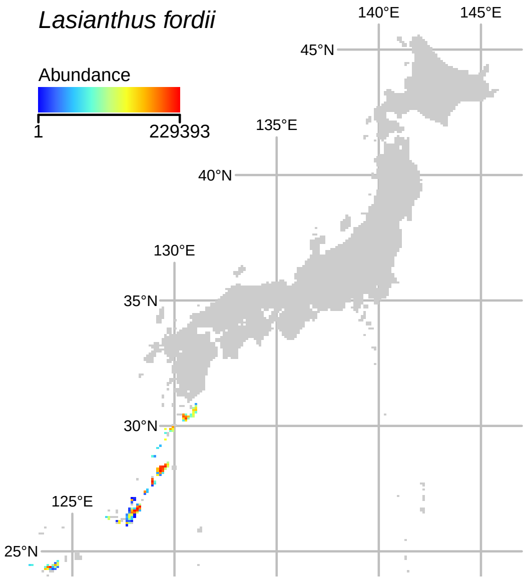
Lasianthus fordii
Abundance
1
229393
140°E
145°E
45°N
135°E
40°N
130°E
35°N
30°N
125°E
25°N

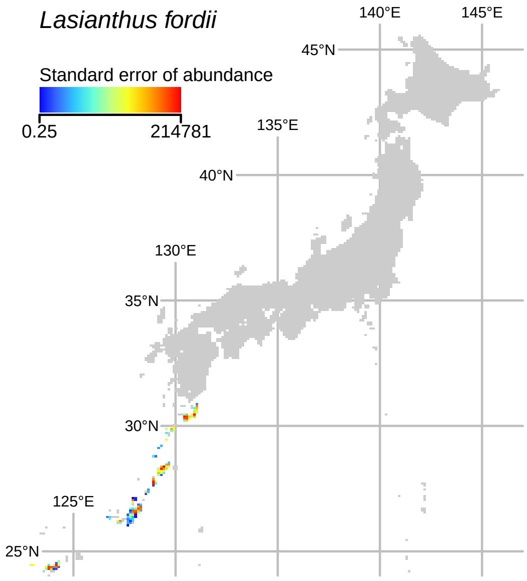
Lasianthus fordii
Standard error of abundance
0.25
214781
140°E
145°E
45°N
135°E
40°N
130°E
35°N
30°N
125°E
25°N

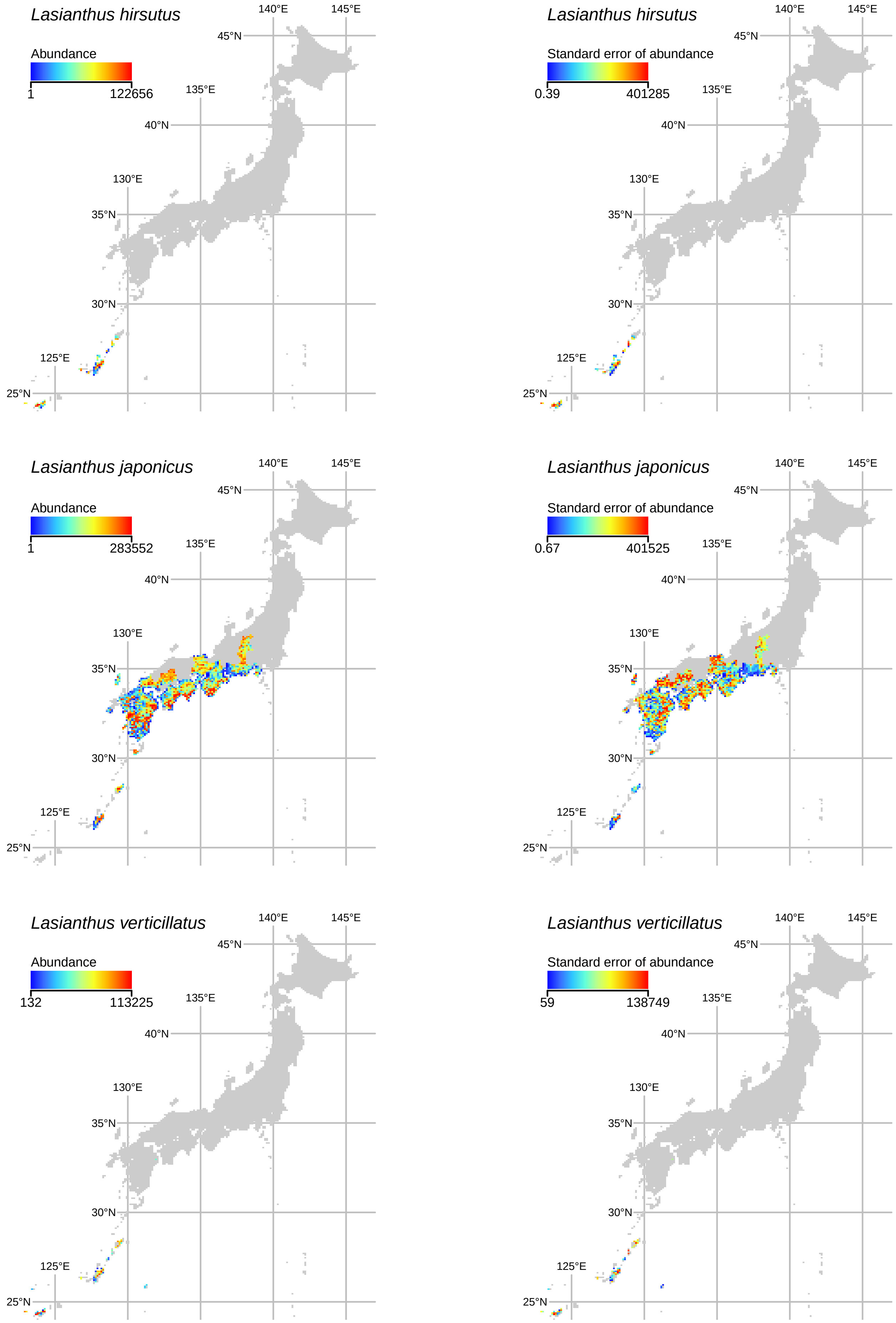
Lasianthus hirsutus
Abundance
1
122656
Lasianthus hirsutus
Standard error of abundance
0.39
401285
Lasianthus japonicus
Abundance
1
283552
Lasianthus japonicus
Standard error of abundance
0.67
401525
Lasianthus verticillatus
Abundance
132
113225
Lasianthus verticillatus
Standard error of abundance
59
138749
125°E
130°E
135°E
140°E
145°E
25°N
30°N
35°N
40°N
45°N

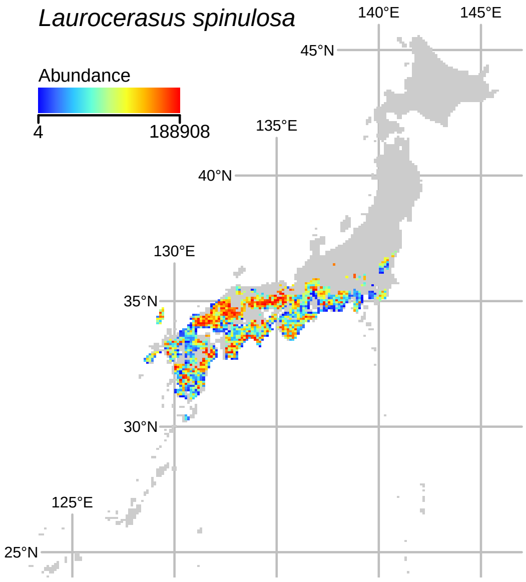
Laurocerasus spinulosa
Abundance
4
188908
140°E
145°E
45°N
135°E
40°N
130°E
35°N
30°N
125°E
25°N

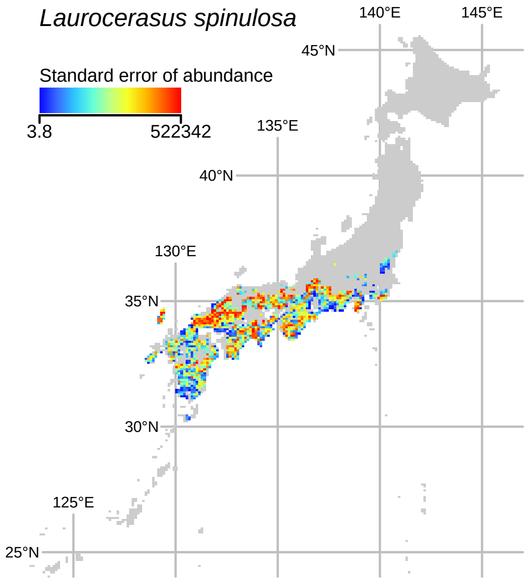
Laurocerasus spinulosa
Standard error of abundance
3.8
522342
140°E
145°E
45°N
135°E
40°N
130°E
35°N
30°N
125°E
25°N

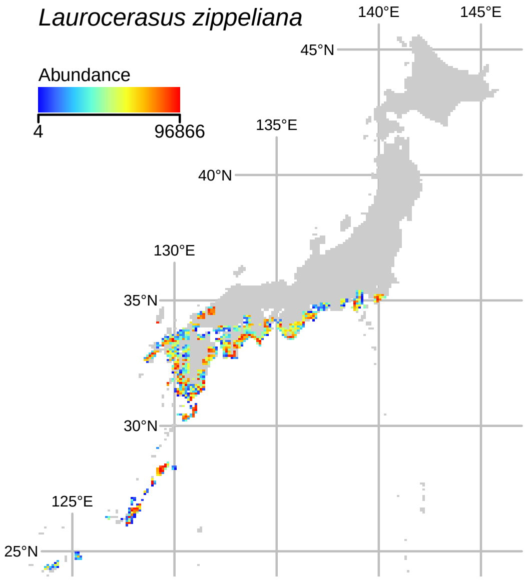
Laurocerasus zippeliana
Abundance
4
96866
140°E
145°E
45°N
135°E
40°N
130°E
35°N
30°N
125°E
25°N

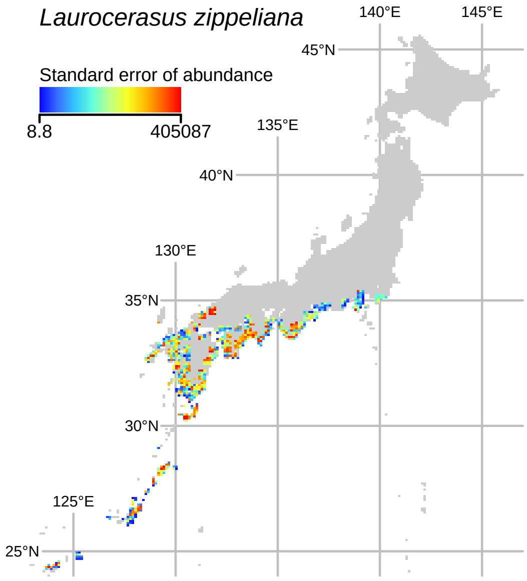
Laurocerasus zippeliana
Standard error of abundance
8.8
405087
140°E
145°E
45°N
135°E
40°N
130°E
35°N
30°N
125°E
25°N

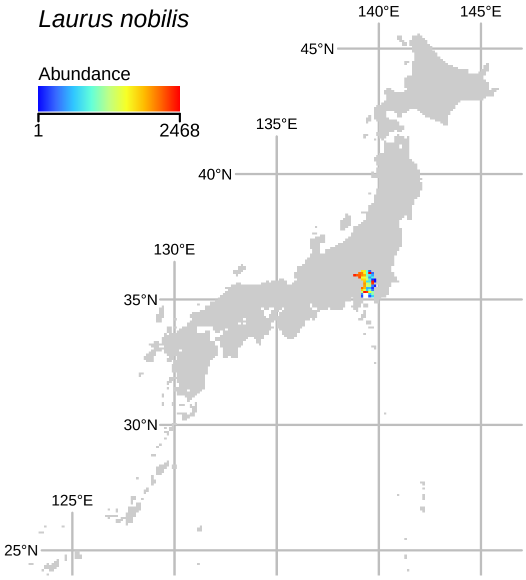
Laurus nobilis
Abundance
1
2468
140°E
145°E
45°N
135°E
40°N
130°E
35°N
30°N
125°E
25°N

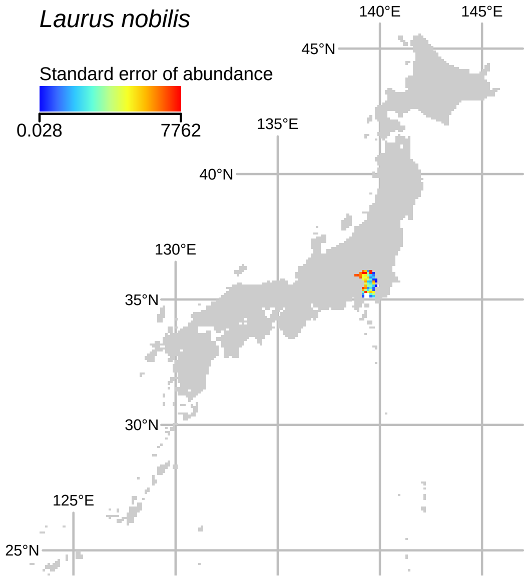
Laurus nobilis
Standard error of abundance
0.028
7762
140°E
145°E
45°N
135°E
40°N
130°E
35°N
30°N
125°E
25°N

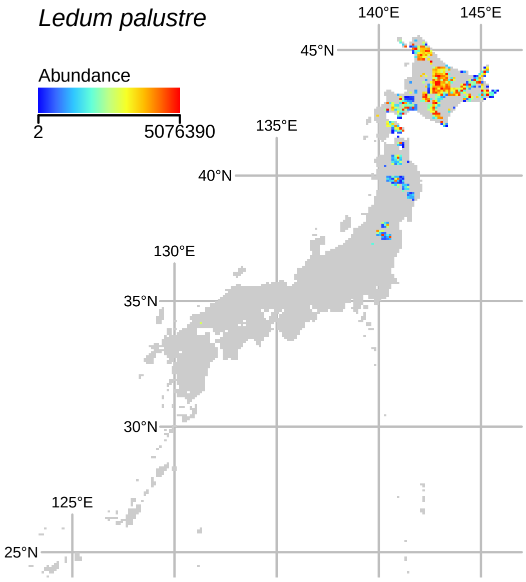
Ledum palustre
Abundance
2
5076390
140°E
145°E
45°N
135°E
40°N
130°E
35°N
30°N
125°E
25°N

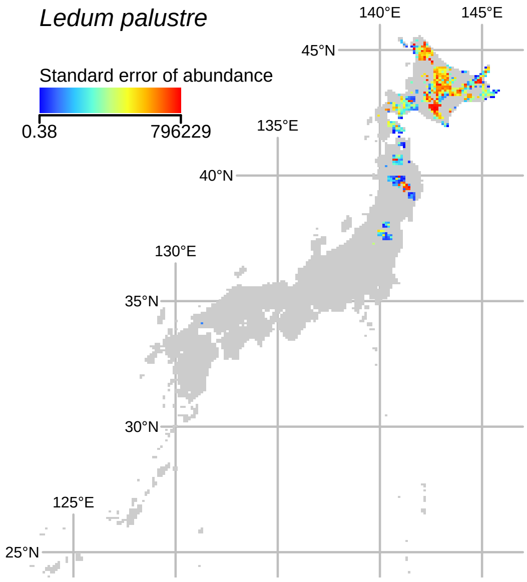
Ledum palustre
Standard error of abundance
0.38
796229
140°E
145°E
45°N
135°E
40°N
130°E
35°N
30°N
125°E
25°N

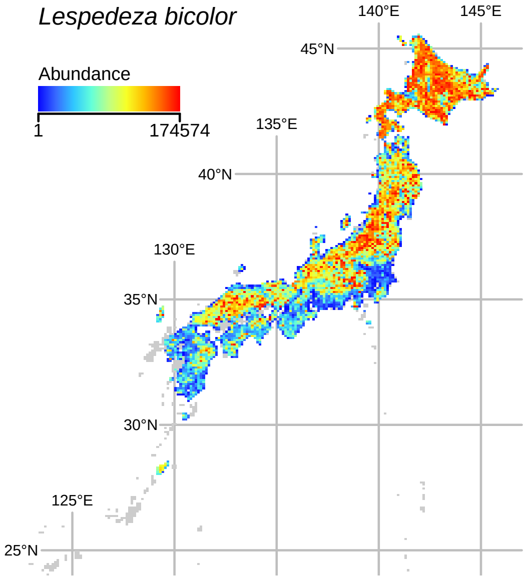
Lespedeza bicolor
Abundance
1
174574
140°E
145°E
45°N
135°E
40°N
130°E
35°N
30°N
125°E
25°N

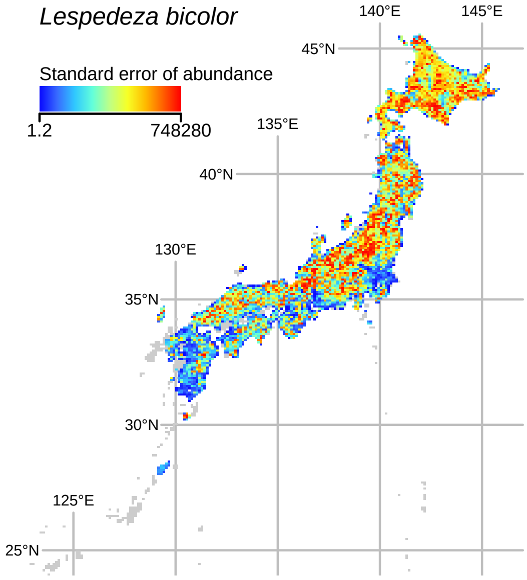
Lespedeza bicolor
Standard error of abundance
1.2
748280
140°E
145°E
45°N
135°E
40°N
130°E
35°N
30°N
125°E
25°N

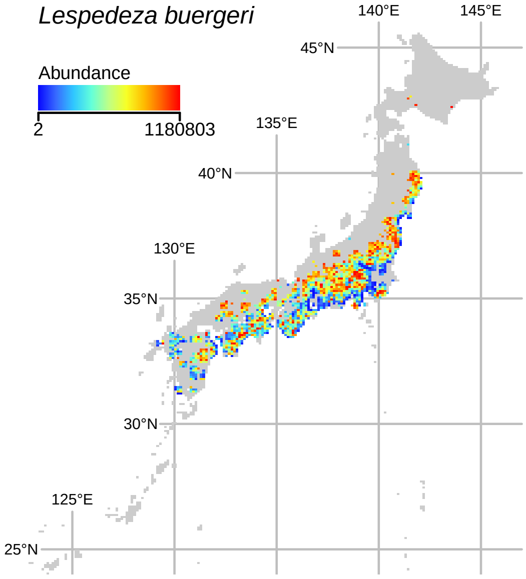
Lespedeza buergeri
Abundance
2
1180803
140°E
145°E
45°N
135°E
40°N
130°E
35°N
30°N
125°E
25°N

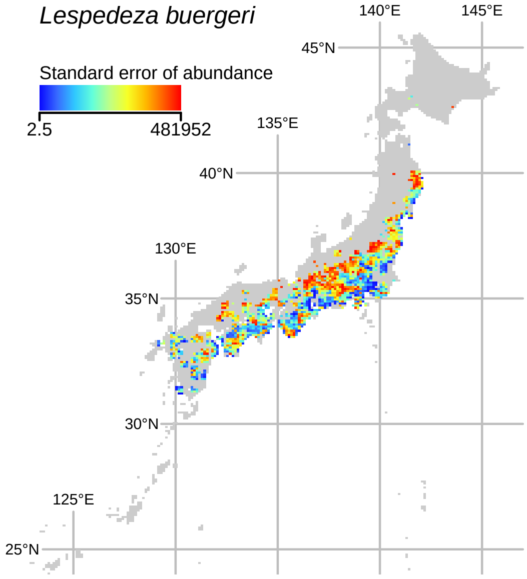
Lespedeza buergeri
Standard error of abundance
2.5
481952
140°E
145°E
45°N
135°E
40°N
130°E
35°N
30°N
125°E
25°N

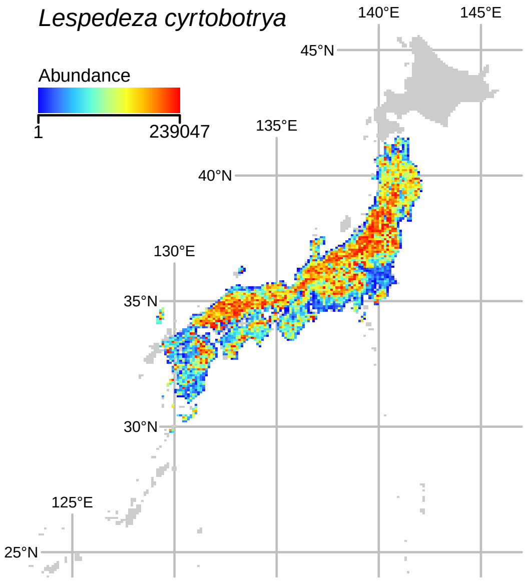
Lespedeza cyrtobotrya
Abundance
1
239047
125°E
130°E
135°E
140°E
145°E
25°N
30°N
35°N
40°N
45°N

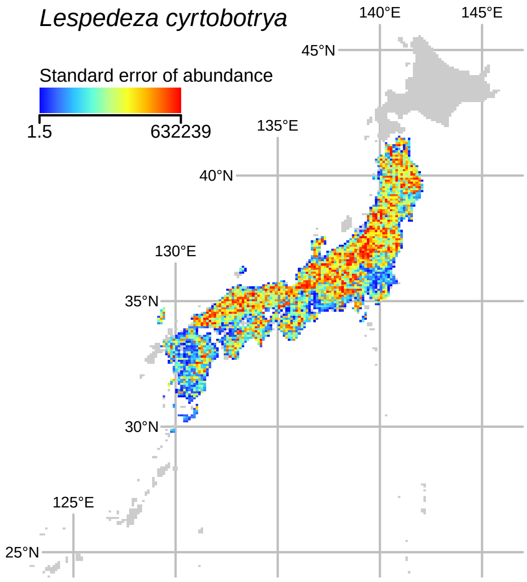
Lespedeza cyrtobotrya
Standard error of abundance
1.5
632239
125°E
130°E
135°E
140°E
145°E
25°N
30°N
35°N
40°N
45°N

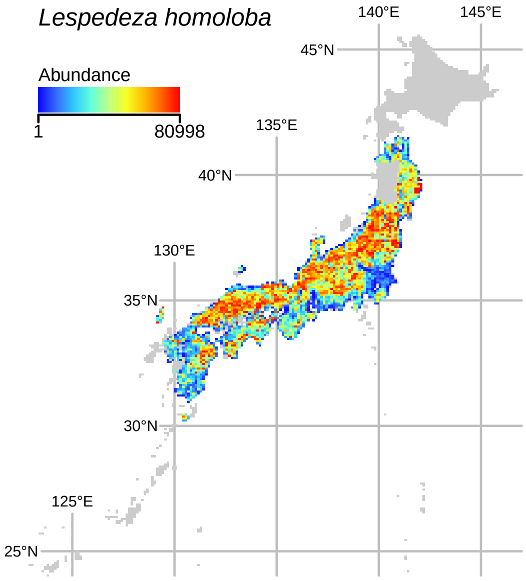
Lespedeza homoloba
Abundance
1
80998
125°E
130°E
135°E
140°E
145°E
25°N
30°N
35°N
40°N
45°N

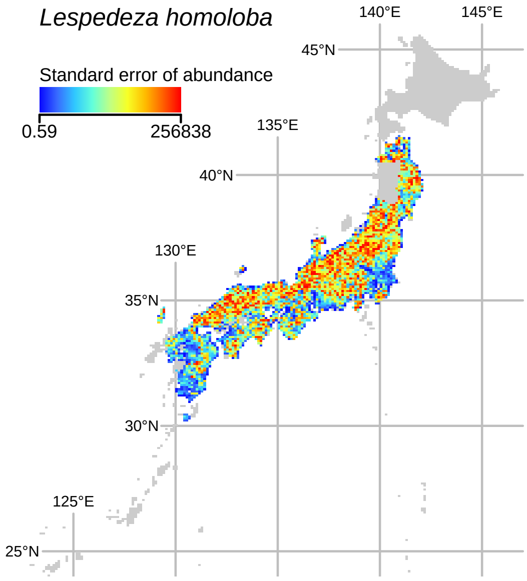
Lespedeza homoloba
Standard error of abundance
0.59
256838
125°E
130°E
135°E
140°E
145°E
25°N
30°N
35°N
40°N
45°N

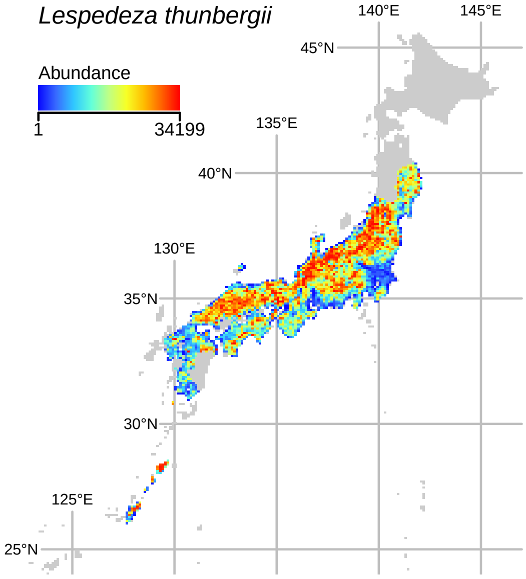
Lespedeza thunbergii
Abundance
1
34199
125°E
130°E
135°E
140°E
145°E
25°N
30°N
35°N
40°N
45°N

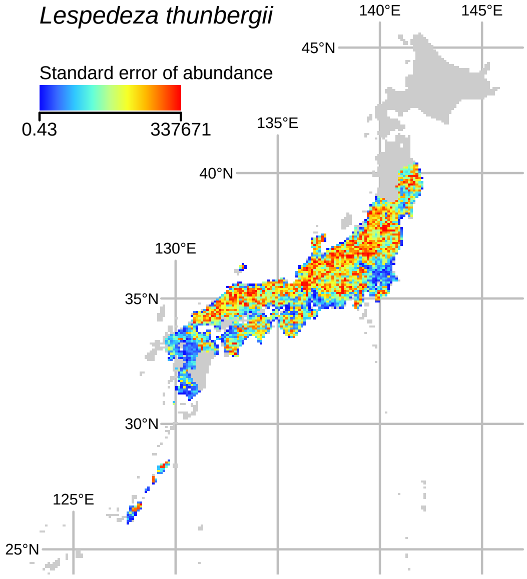
Lespedeza thunbergii
Standard error of abundance
0.43
337671
125°E
130°E
135°E
140°E
145°E
25°N
30°N
35°N
40°N
45°N

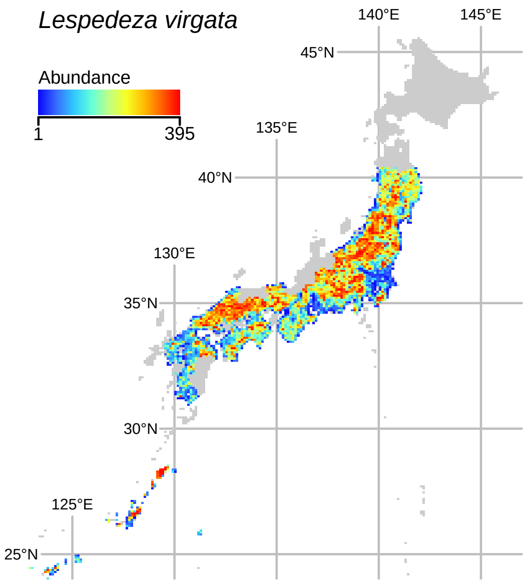
Lespedeza virgata
Abundance
1
395
140°E
145°E
45°N
135°E
40°N
130°E
35°N
30°N
125°E
25°N

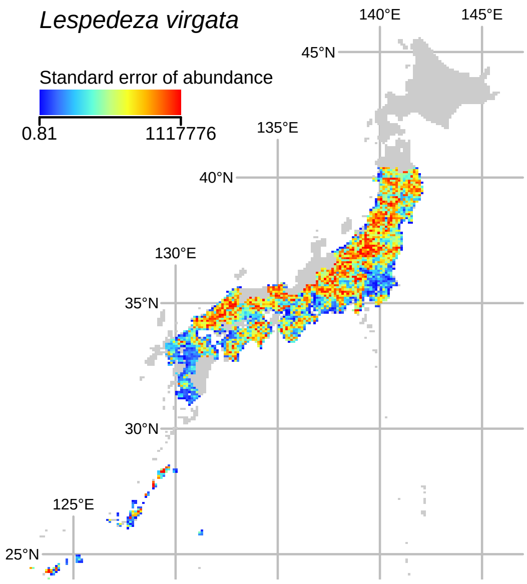
Lespedeza virgata
Standard error of abundance
0.81
1117776
140°E
145°E
45°N
135°E
40°N
130°E
35°N
30°N
125°E
25°N

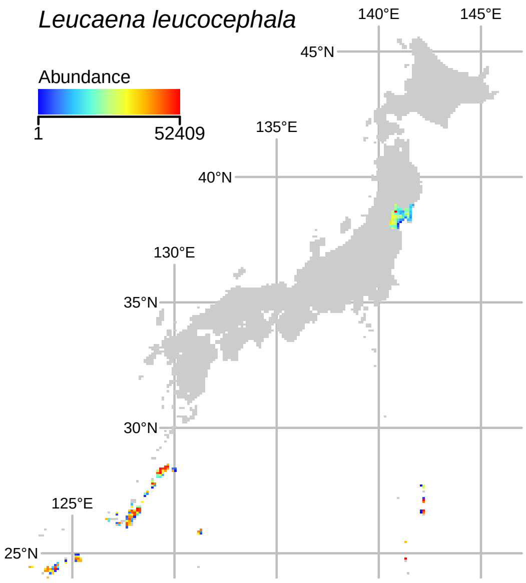
Leucaena leucocephala
Abundance
1
52409
140°E
145°E
45°N
135°E
40°N
130°E
35°N
30°N
125°E
25°N

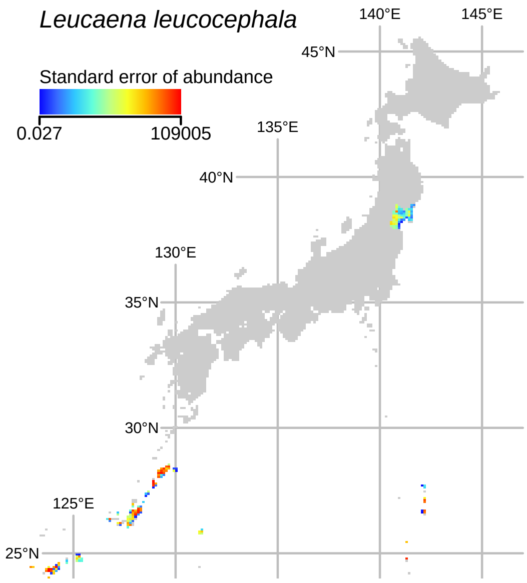
Leucaena leucocephala
Standard error of abundance
0.027
109005
140°E
145°E
45°N
135°E
40°N
130°E
35°N
30°N
125°E
25°N

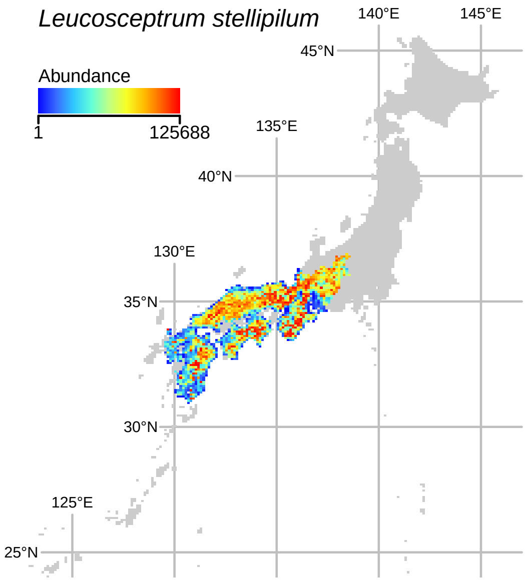
Leucosceptrum stellipilum
Abundance
1
125688
140°E
145°E
45°N
135°E
40°N
130°E
35°N
30°N
125°E
25°N

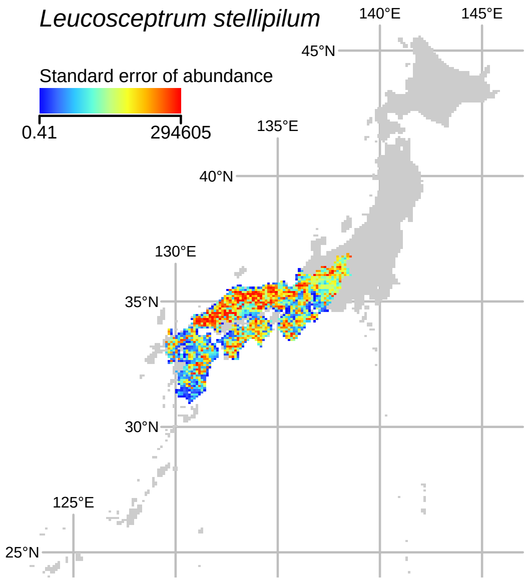
Leucosceptrum stellipilum
Standard error of abundance
0.41
294605
140°E
145°E
45°N
135°E
40°N
130°E
35°N
30°N
125°E
25°N

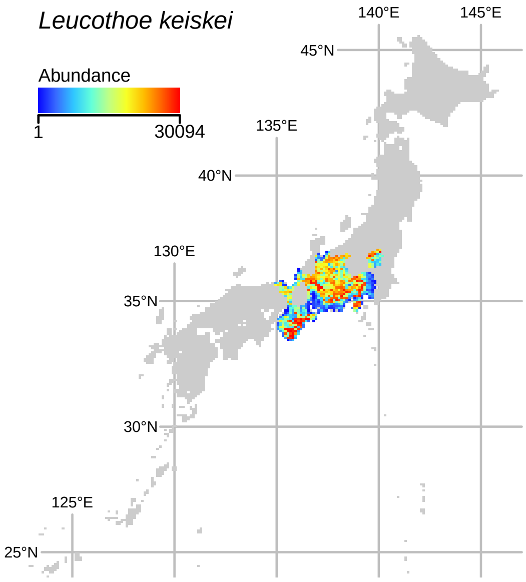
Leucothoe keiskei
Abundance
1
30094
140°E
145°E
45°N
135°E
40°N
130°E
35°N
30°N
125°E
25°N

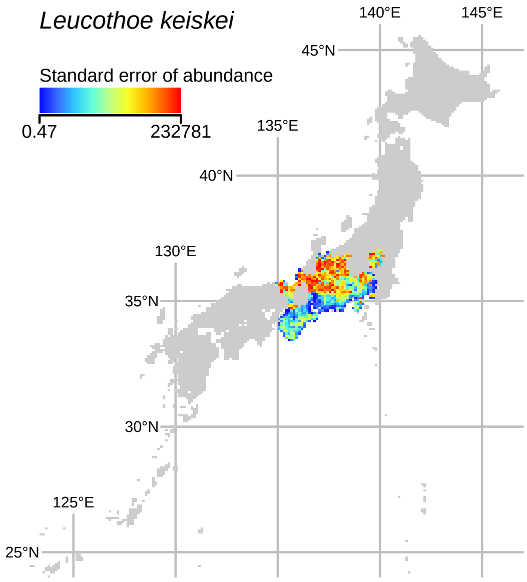
Leucothoe keiskei
Standard error of abundance
0.47
232781
140°E
145°E
45°N
135°E
40°N
130°E
35°N
30°N
125°E
25°N

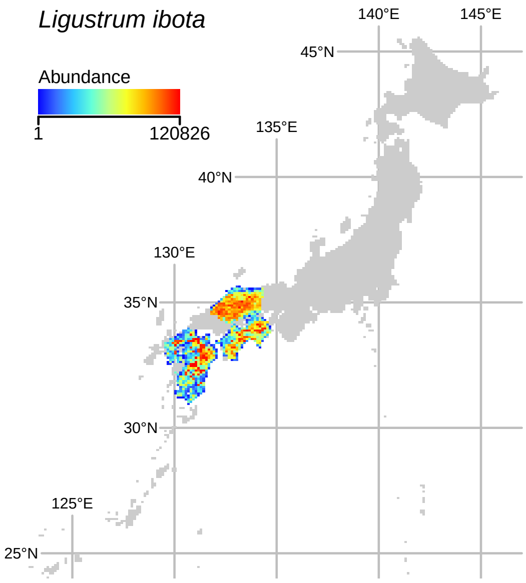
Ligustrum ibota
Abundance
1
120826
140°E
145°E
45°N
135°E
40°N
130°E
35°N
30°N
125°E
25°N

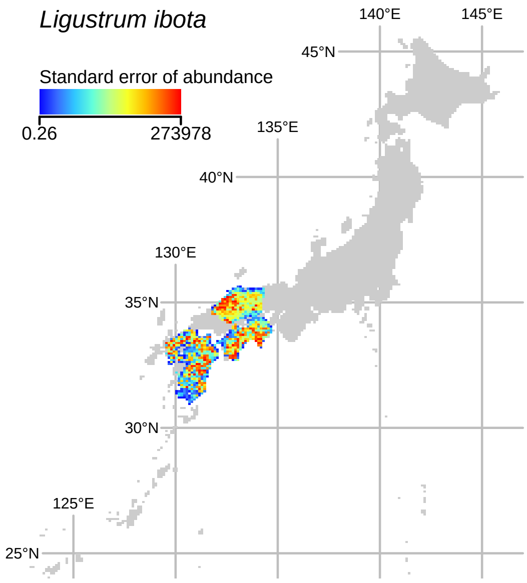
Ligustrum ibota
Standard error of abundance
0.26
273978
140°E
145°E
45°N
135°E
40°N
130°E
35°N
30°N
125°E
25°N

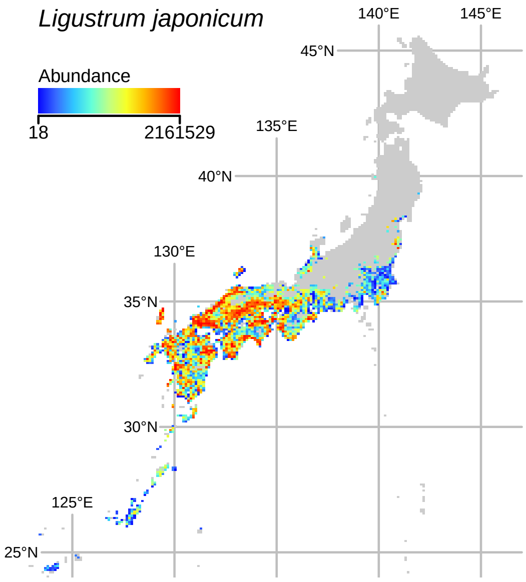
Ligustrum japonicum
Abundance
18
2161529
140°E
145°E
45°N
135°E
40°N
130°E
35°N
30°N
125°E
25°N

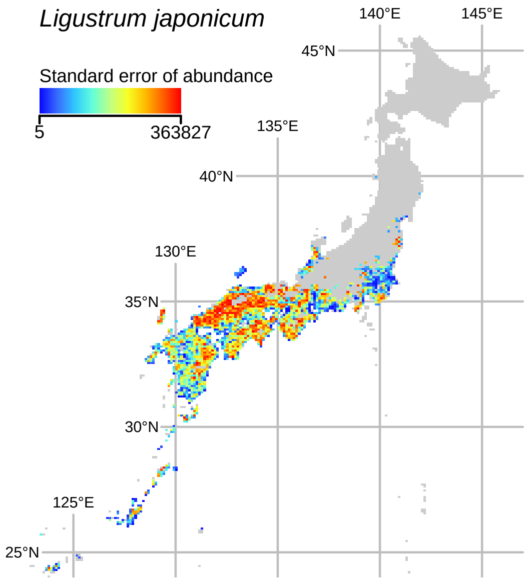
Ligustrum japonicum
Standard error of abundance
5
363827
140°E
145°E
45°N
135°E
40°N
130°E
35°N
30°N
125°E
25°N

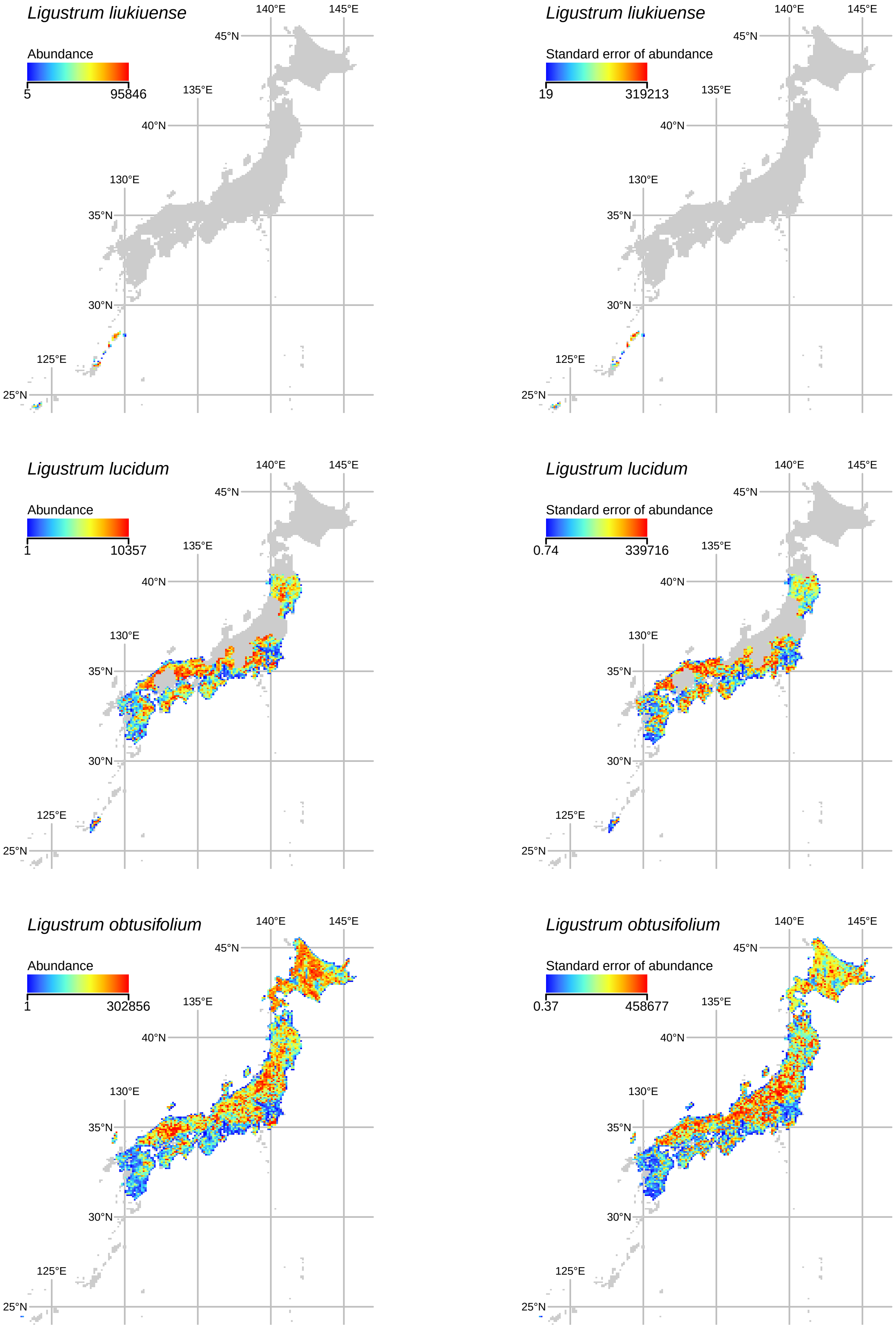

Ligustrum liukiuense
Abundance
5
95846
Ligustrum liukiuense
Standard error of abundance
19
319213
Ligustrum lucidum
Abundance
1
10357
Ligustrum lucidum
Standard error of abundance
0.74
339716
Ligustrum obtusifolium
Abundance
1
302856
Ligustrum obtusifolium
Standard error of abundance
0.37
458677
125°E
130°E
135°E
140°E
145°E
25°N
30°N
35°N
40°N
45°N

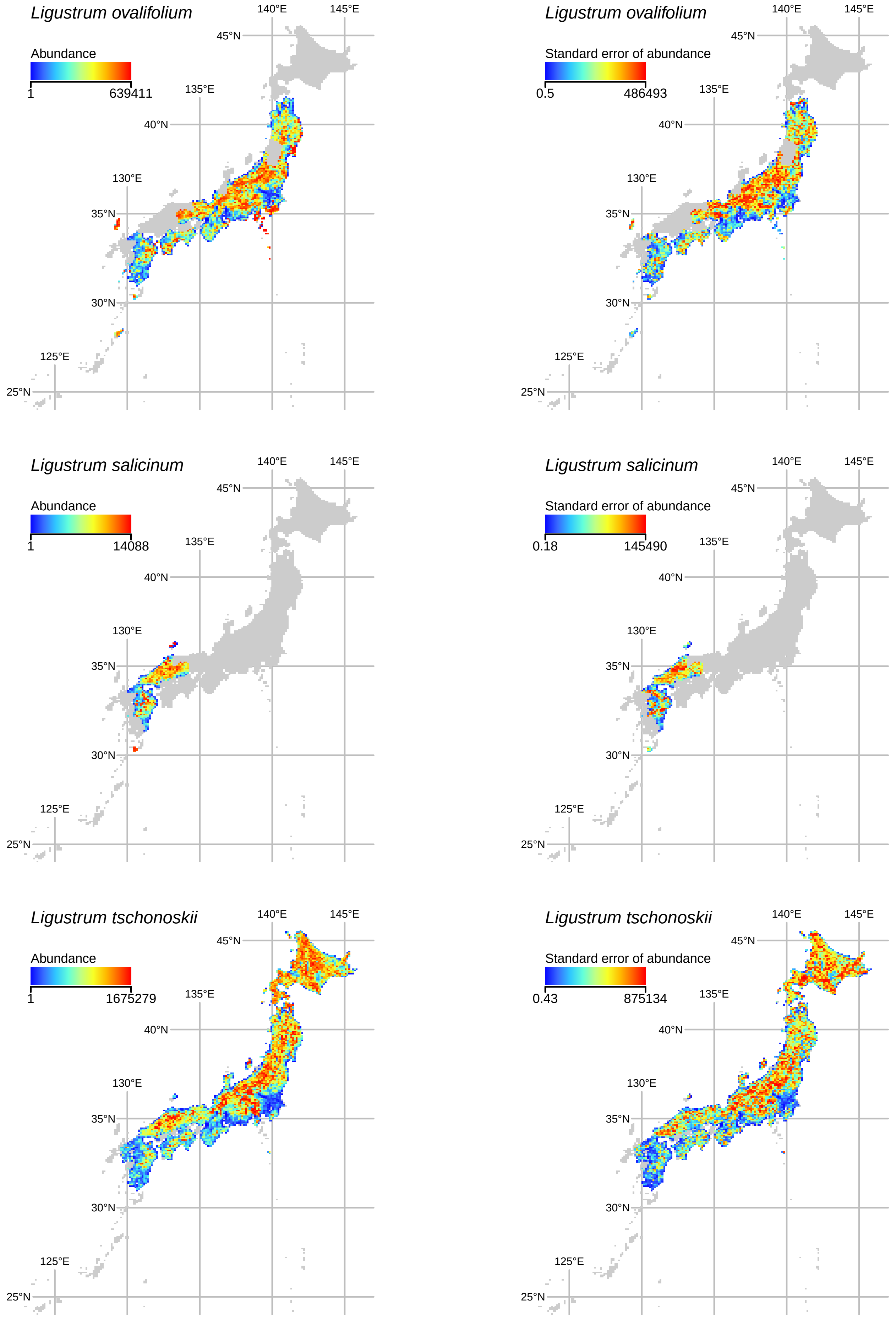
Ligustrum ovalifolium
Abundance
1
639411
Ligustrum ovalifolium
Standard error of abundance
0.5
486493
Ligustrum salicinum
Abundance
1
14088
Ligustrum salicinum
Standard error of abundance
0.18
145490
Ligustrum tschonoskii
Abundance
1
1675279
Ligustrum tschonoskii
Standard error of abundance
0.43
875134
125°E
130°E
135°E
140°E
145°E
25°N
30°N
35°N
40°N
45°N

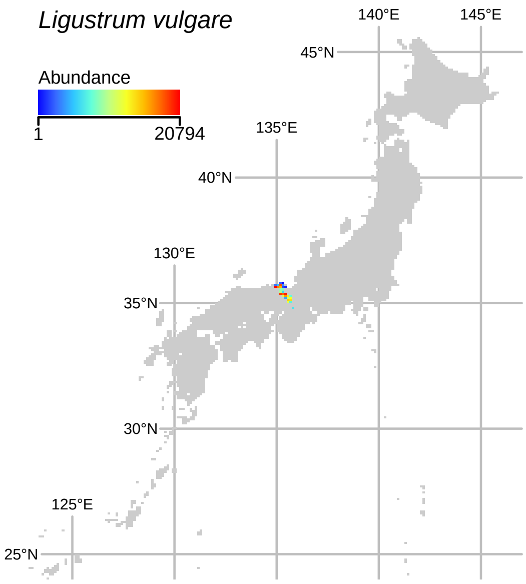
Ligustrum vulgare
Abundance
1
20794
140°E
145°E
45°N
135°E
40°N
130°E
35°N
30°N
125°E
25°N

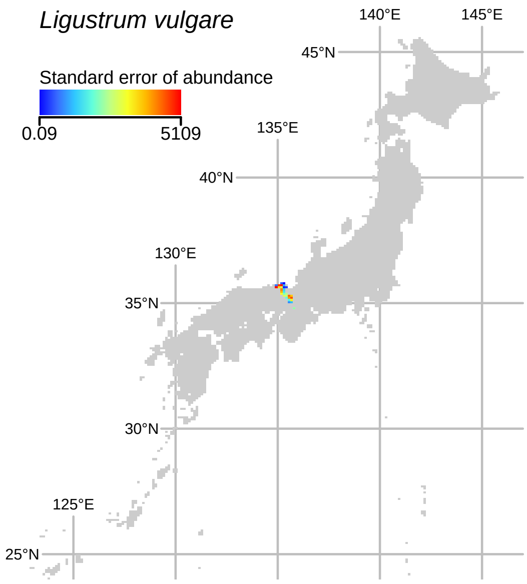
Ligustrum vulgare
Standard error of abundance
0.09
5109
140°E
145°E
45°N
135°E
40°N
130°E
35°N
30°N
125°E
25°N

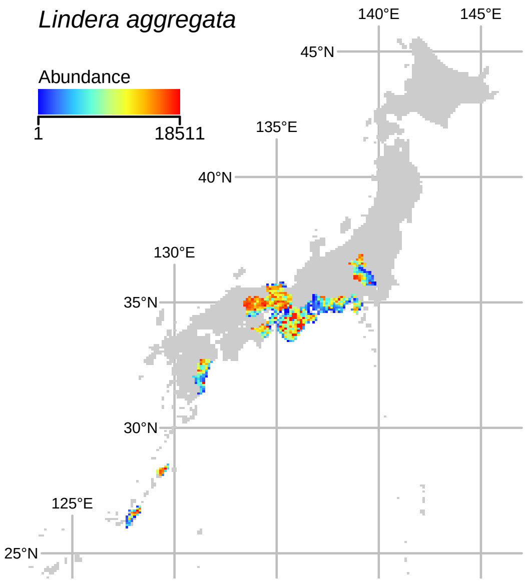
Lindera aggregata
Abundance
1
18511
140°E
145°E
45°N
135°E
40°N
130°E
35°N
30°N
125°E
25°N

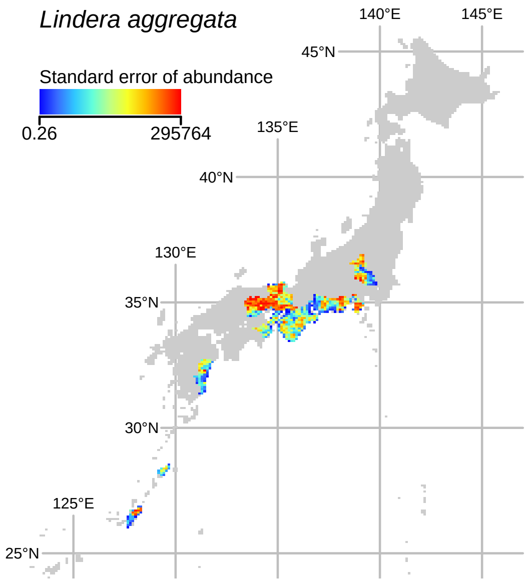
Lindera aggregata
Standard error of abundance
0.26
295764
140°E
145°E
45°N
135°E
40°N
130°E
35°N
30°N
125°E
25°N

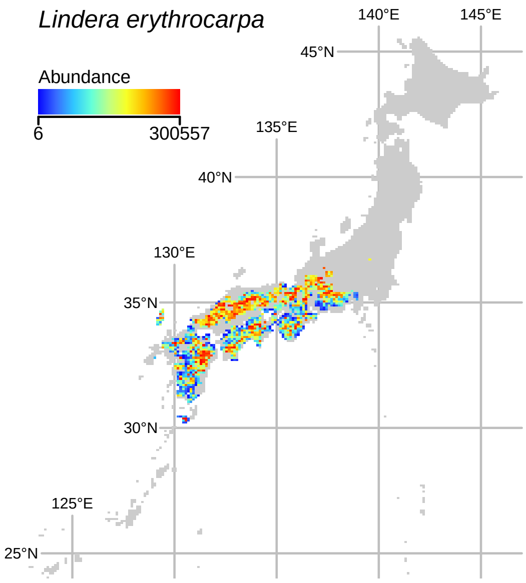
Lindera erythrocarpa
Abundance
6
300557
140°E
145°E
45°N
135°E
40°N
130°E
35°N
30°N
125°E
25°N

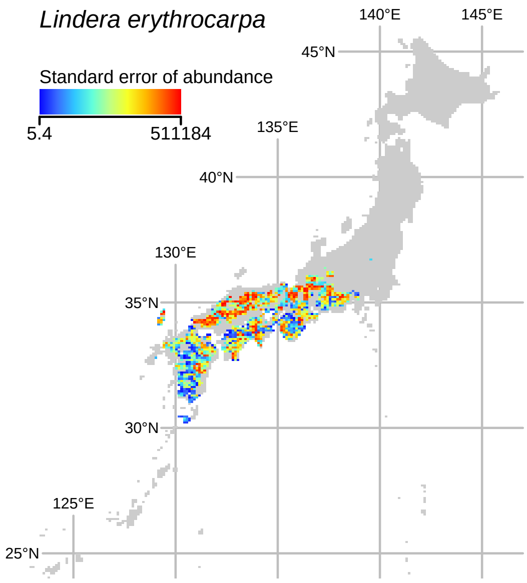
Lindera erythrocarpa
Standard error of abundance
5.4
511184
140°E
145°E
45°N
135°E
40°N
130°E
35°N
30°N
125°E
25°N

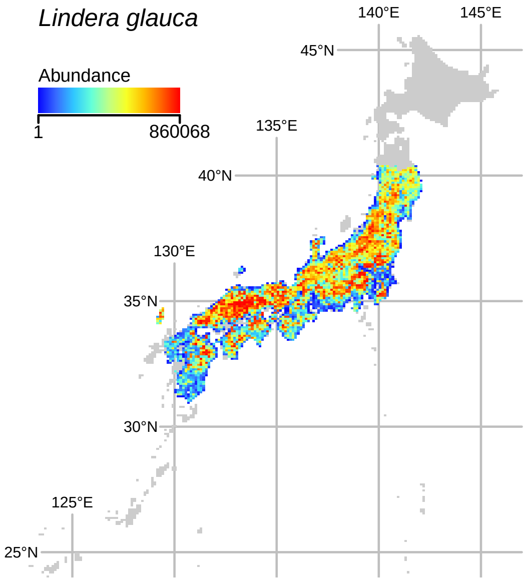
Lindera glauca
Abundance
1
860068
140°E
145°E
45°N
135°E
40°N
130°E
35°N
30°N
125°E
25°N

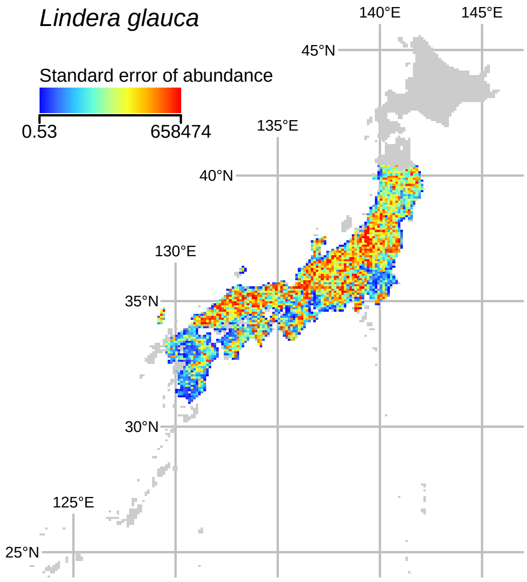
Lindera glauca
Standard error of abundance
0.53
658474
140°E
145°E
45°N
135°E
40°N
130°E
35°N
30°N
125°E
25°N

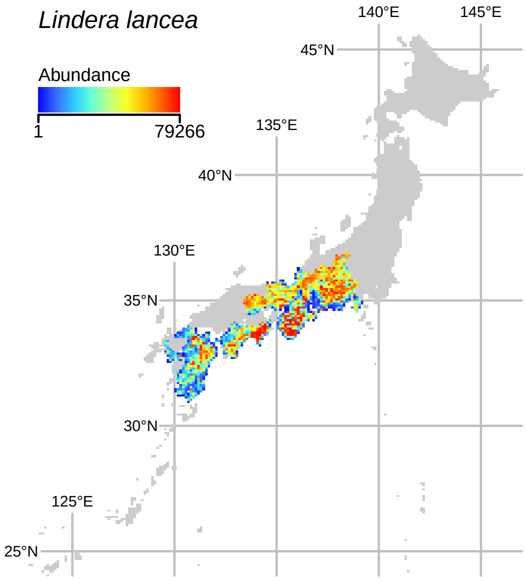
Lindera lancea
Abundance
1
79266
140°E
145°E
45°N
135°E
40°N
130°E
35°N
30°N
125°E
25°N

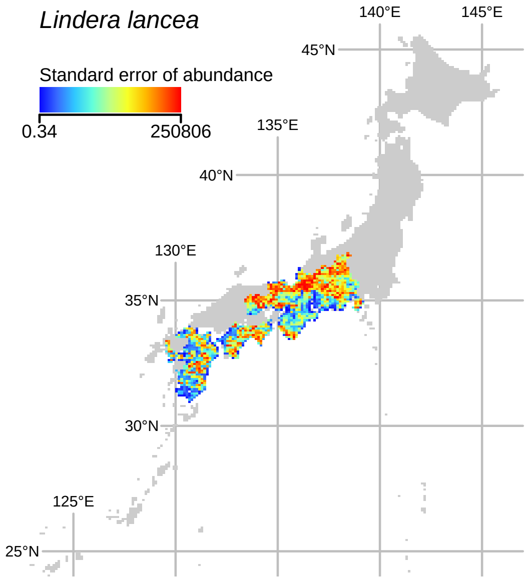
Lindera lancea
Standard error of abundance
0.34
250806
140°E
145°E
45°N
135°E
40°N
130°E
35°N
30°N
125°E
25°N

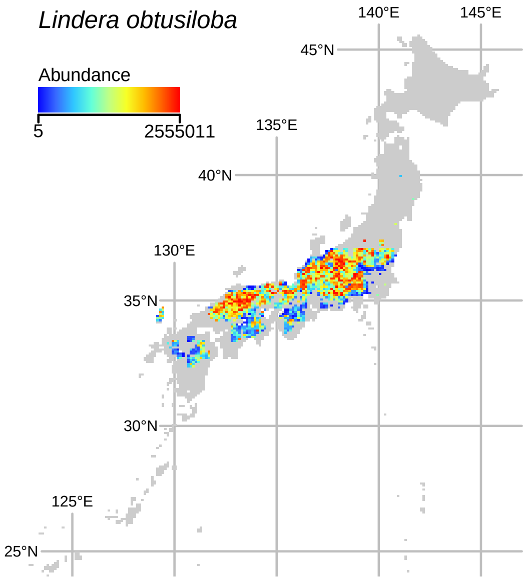
Lindera obtusiloba
Abundance
5
2555011
140°E
145°E
45°N
135°E
40°N
130°E
35°N
30°N
125°E
25°N

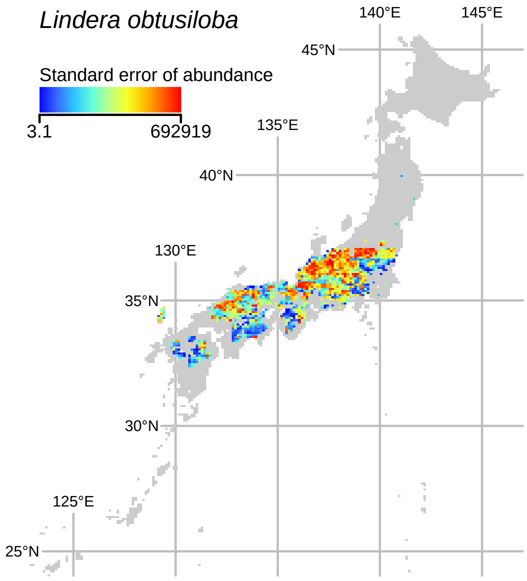
Lindera obtusiloba
Standard error of abundance
3.1
692919
140°E
145°E
45°N
135°E
40°N
130°E
35°N
30°N
125°E
25°N

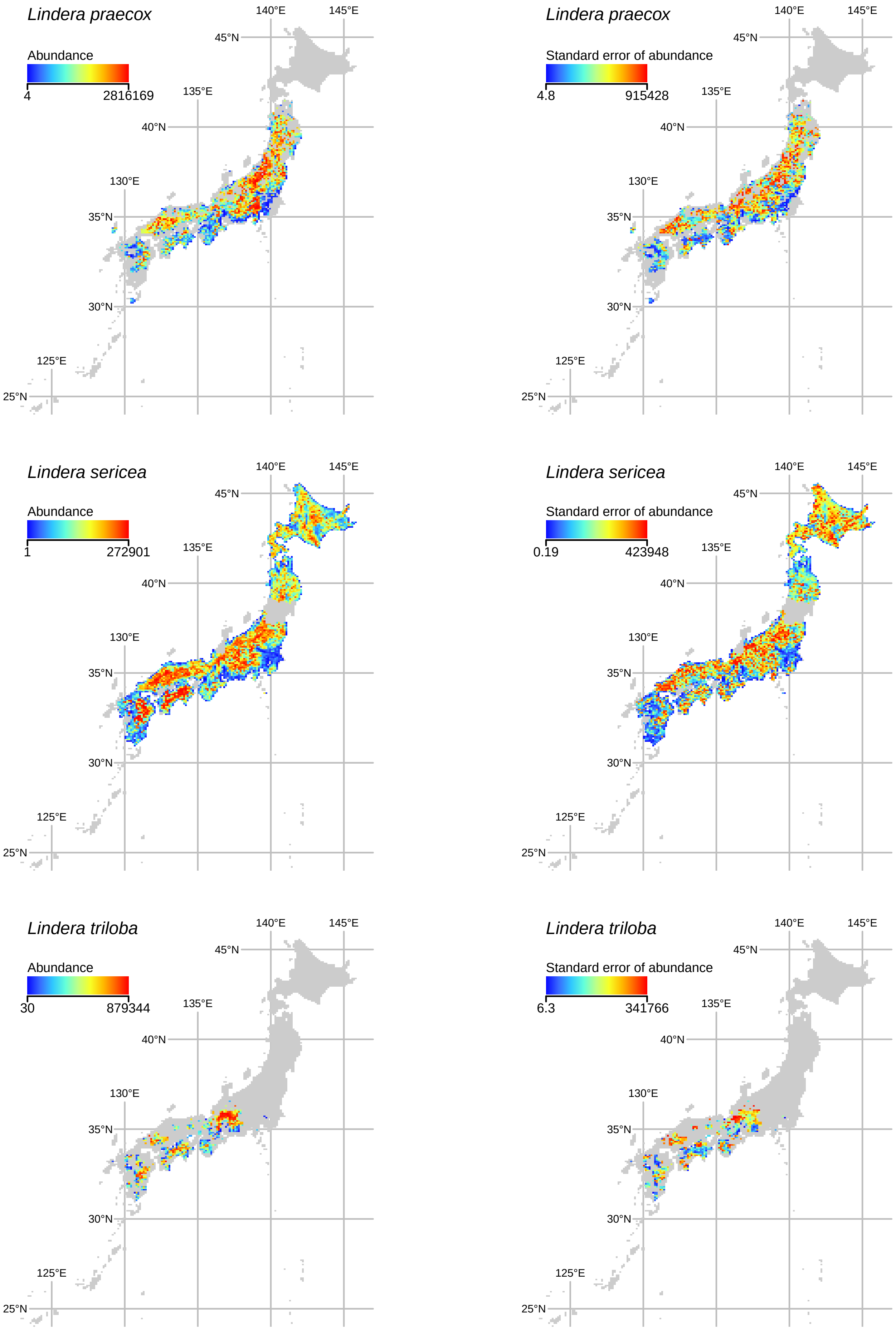

Lindera praecox
Abundance
4
2816169
Lindera praecox
Standard error of abundance
4.8
915428
Lindera sericea
Abundance
1
272901
Lindera sericea
Standard error of abundance
0.19
423948
Lindera triloba
Abundance
30
879344
Lindera triloba
Standard error of abundance
6.3
341766
125°E
130°E
135°E
140°E
145°E
25°N
30°N
35°N
40°N
45°N

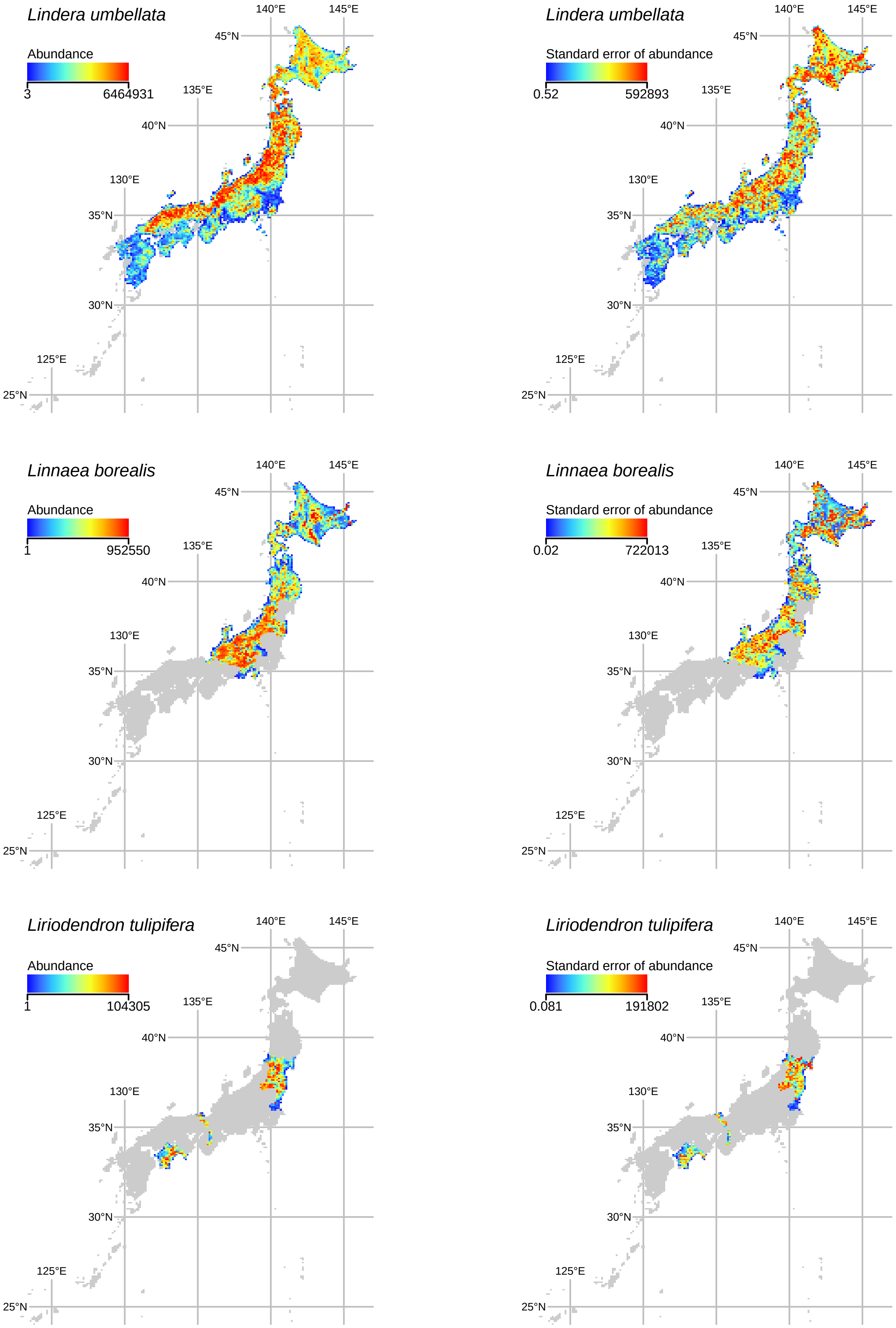
Lindera umbellata
Abundance
3
6464931
Lindera umbellata
Standard error of abundance
0.52
592893
Linnaea borealis
Abundance
1
952550
Linnaea borealis
Standard error of abundance
0.02
722013
Liriodendron tulipifera
Abundance
1
104305
Liriodendron tulipifera
Standard error of abundance
0.081
191802
140°E
145°E
45°N
135°E
40°N
130°E
35°N
30°N
125°E
25°N

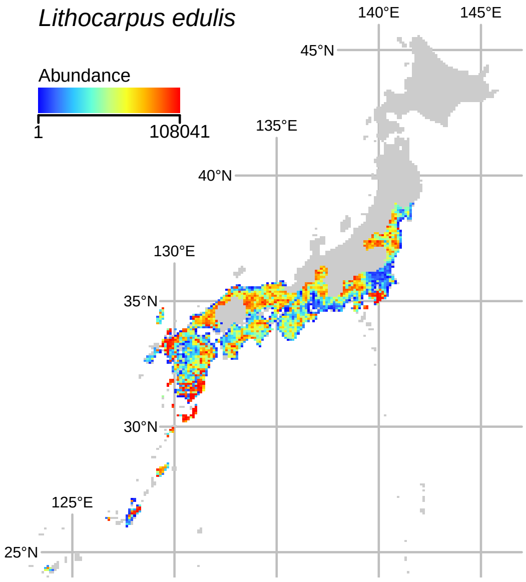

Lithocarpus edulis
Abundance
1
108041
140°E
145°E
45°N
135°E
40°N
130°E
35°N
30°N
125°E
25°N

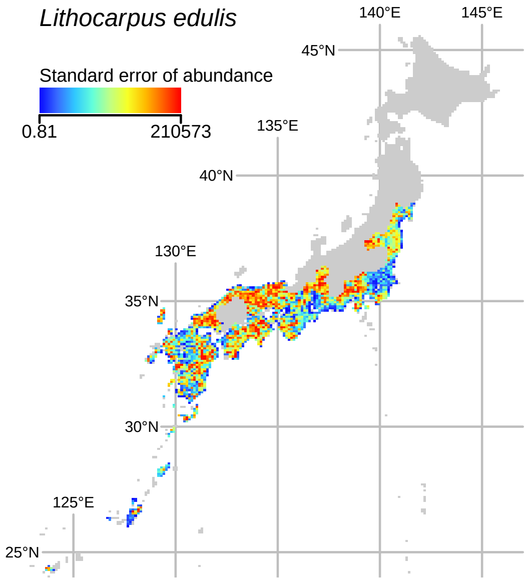

Lithocarpus edulis
Standard error of abundance
0.81
210573
140°E
145°E
45°N
135°E
40°N
130°E
35°N
30°N
125°E
25°N

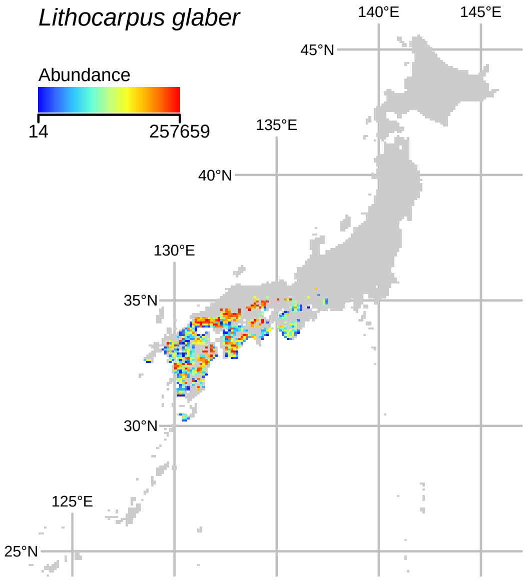

Lithocarpus glaber
Abundance
14
257659
140°E
145°E
45°N
135°E
40°N
130°E
35°N
30°N
125°E
25°N

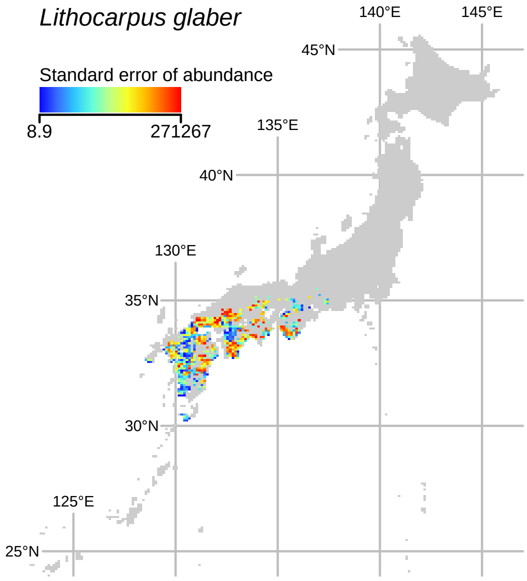

Lithocarpus glaber
Standard error of abundance
8.9
271267
140°E
145°E
45°N
135°E
40°N
130°E
35°N
30°N
125°E
25°N

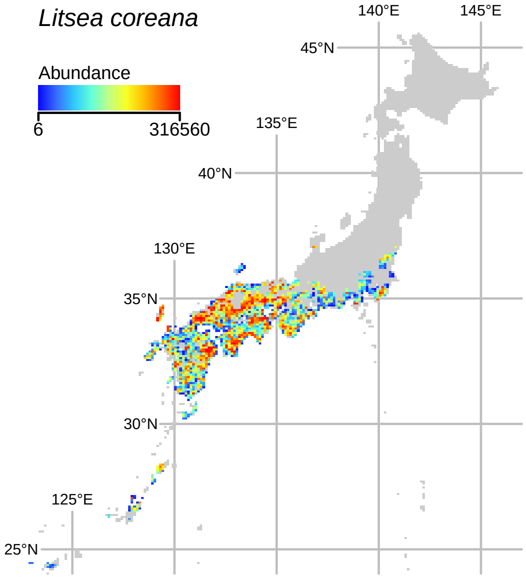

Litsea coreana
Abundance
6
316560
140°E
145°E
45°N
135°E
40°N
130°E
35°N
30°N
125°E
25°N

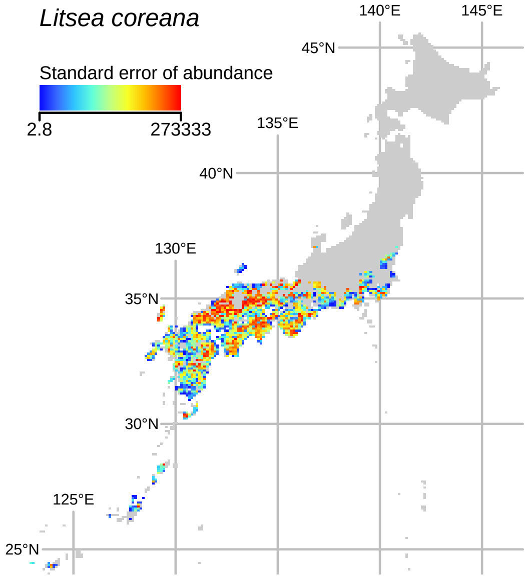

Litsea coreana
Standard error of abundance
2.8
273333
140°E
145°E
45°N
135°E
40°N
130°E
35°N
30°N
125°E
25°N

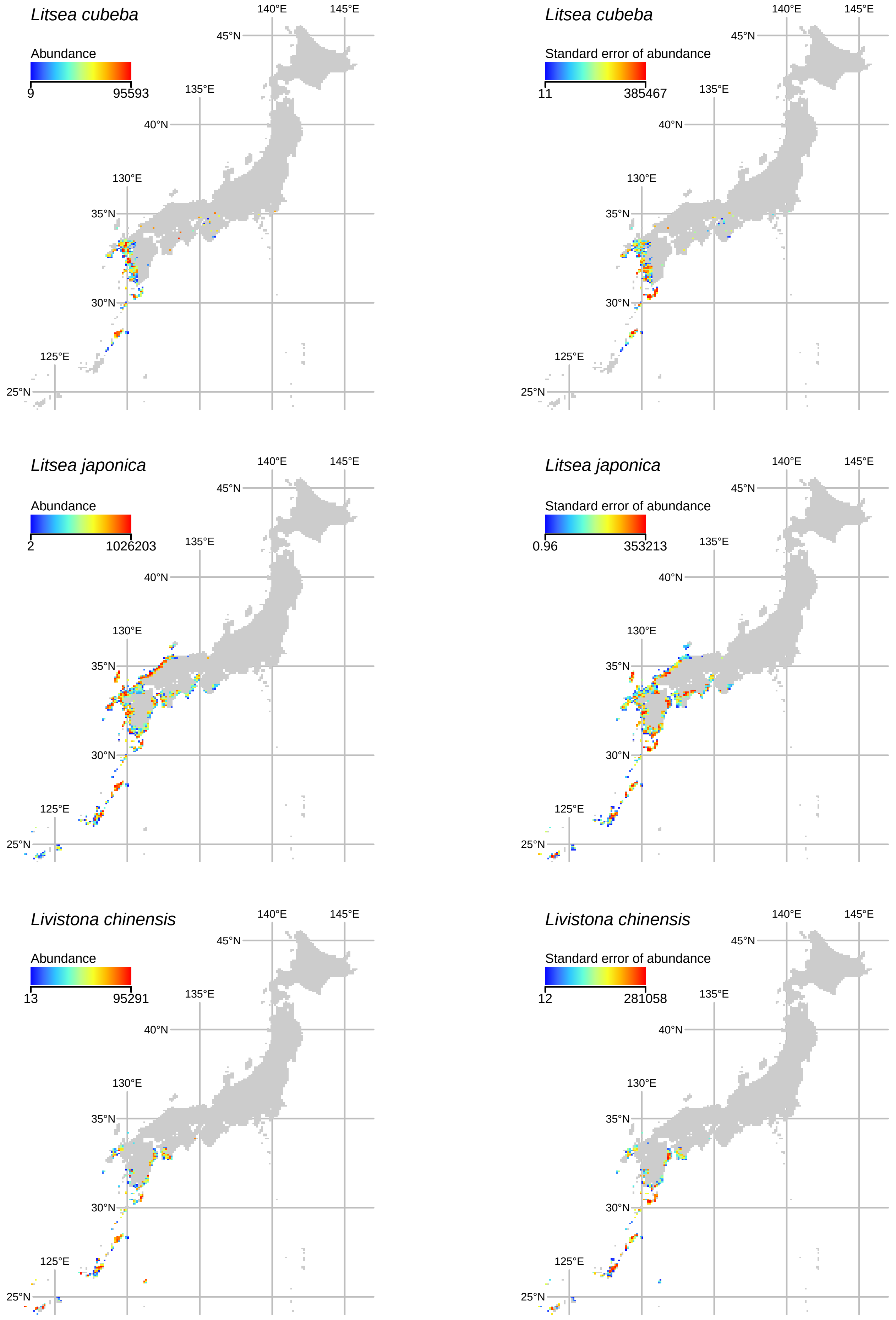
Litsea cubeba
Abundance
9
95593
Litsea cubeba
Standard error of abundance
11
385467
Litsea japonica
Abundance
2
1026203
Litsea japonica
Standard error of abundance
0.96
353213
Livistona chinensis
Abundance
13
95291
Livistona chinensis
Standard error of abundance
12
281058
140°E
145°E
45°N
135°E
40°N
130°E
35°N
30°N
125°E
25°N

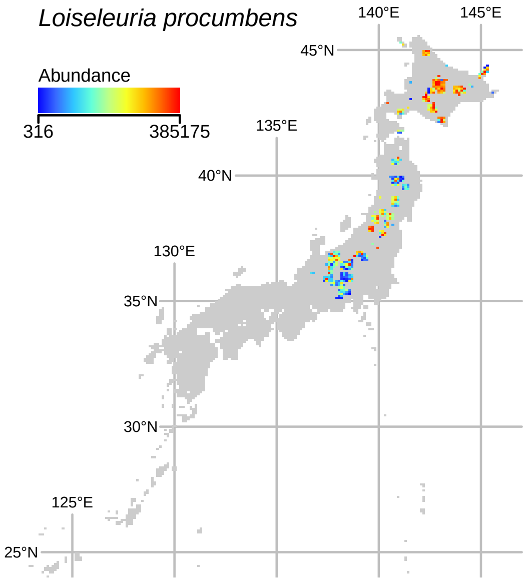
Loiseleuria procumbens
Abundance
316
385175
140°E
145°E
135°E
130°E
125°E
45°N
40°N
35°N
30°N
25°N

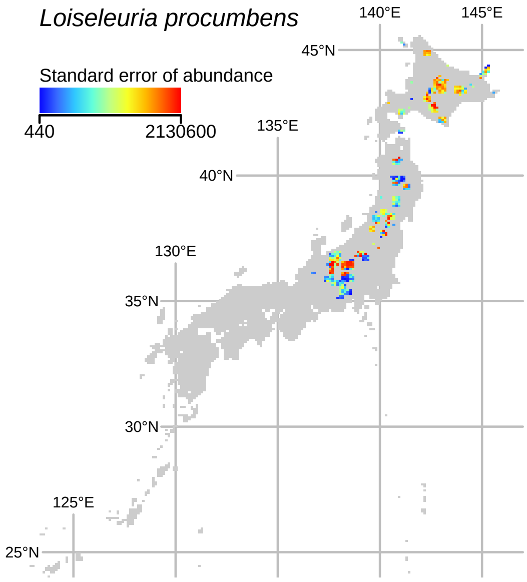
Loiseleuria procumbens
Standard error of abundance
440
2130600
140°E
145°E
135°E
130°E
125°E
45°N
40°N
35°N
30°N
25°N

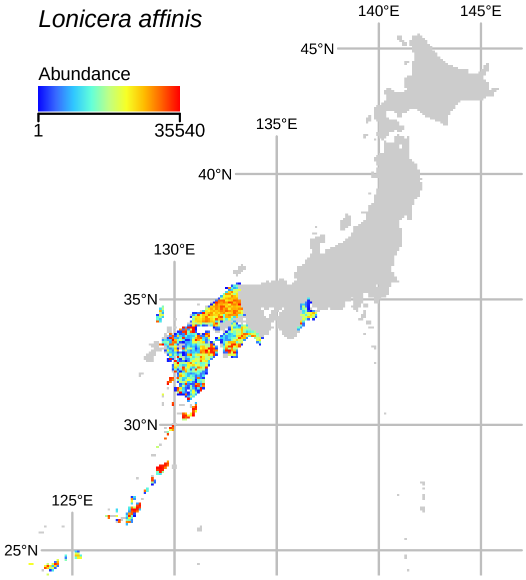
Lonicera affinis
Abundance
1
35540
140°E
145°E
135°E
130°E
125°E
45°N
40°N
35°N
30°N
25°N

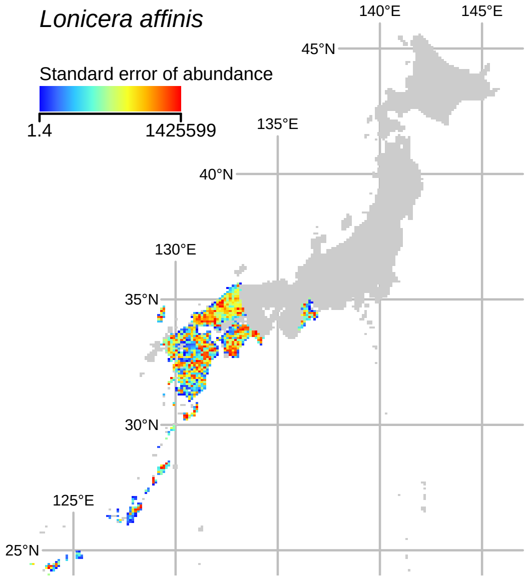
Lonicera affinis
Standard error of abundance
1.4
1425599
140°E
145°E
135°E
130°E
125°E
45°N
40°N
35°N
30°N
25°N

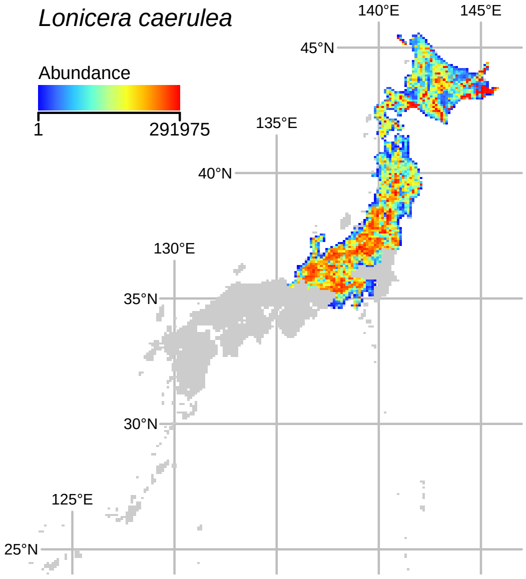
Lonicera caerulea
Abundance
1
291975
140°E
145°E
135°E
130°E
125°E
45°N
40°N
35°N
30°N
25°N

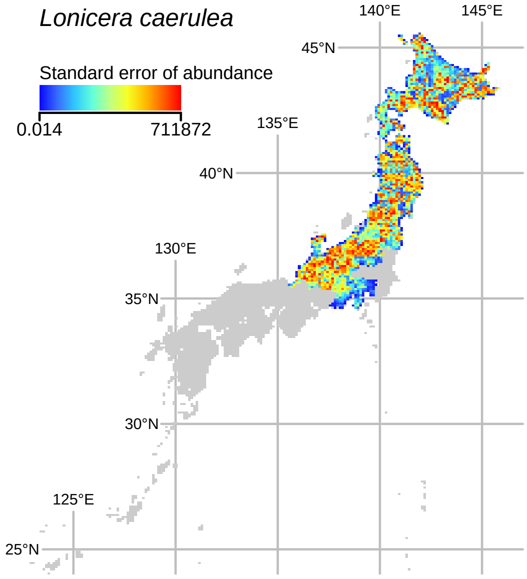
Lonicera caerulea
Standard error of abundance
0.014
711872
140°E
145°E
135°E
130°E
125°E
45°N
40°N
35°N
30°N
25°N

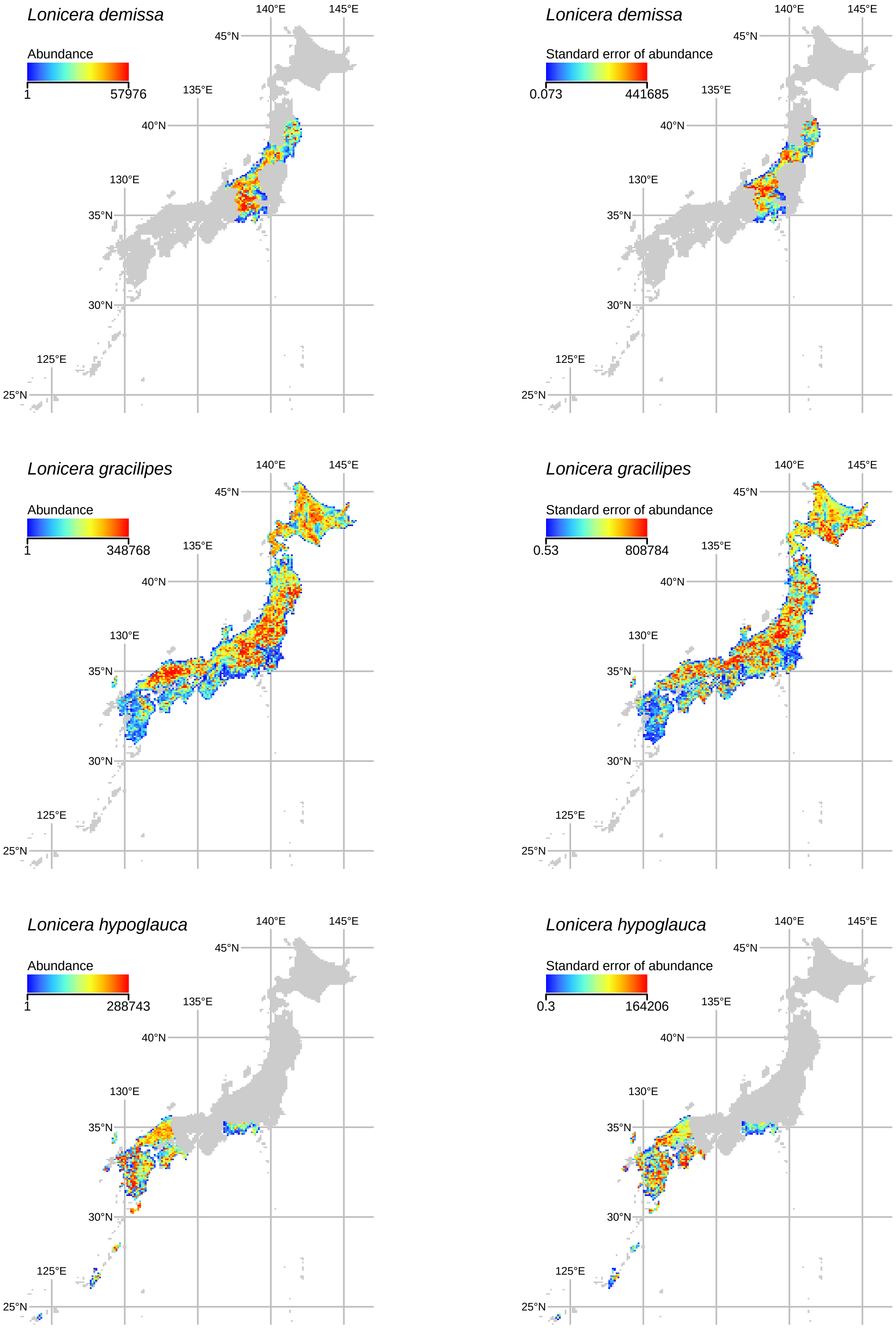
Lonicera demissa
Abundance
1
57976
Lonicera demissa
Standard error of abundance
0.073
441685
Lonicera gracilipes
Abundance
1
348768
Lonicera gracilipes
Standard error of abundance
0.53
808784
Lonicera hypoglauca
Abundance
1
288743
Lonicera hypoglauca
Standard error of abundance
0.3
164206
140°E
145°E
45°N
135°E
40°N
130°E
35°N
30°N
125°E
25°N

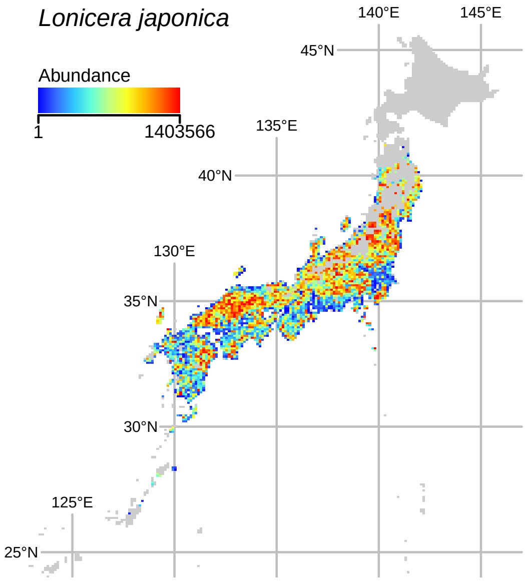
Lonicera japonica
Abundance
1
1403566
140°E
145°E
45°N
135°E
40°N
130°E
35°N
30°N
125°E
25°N

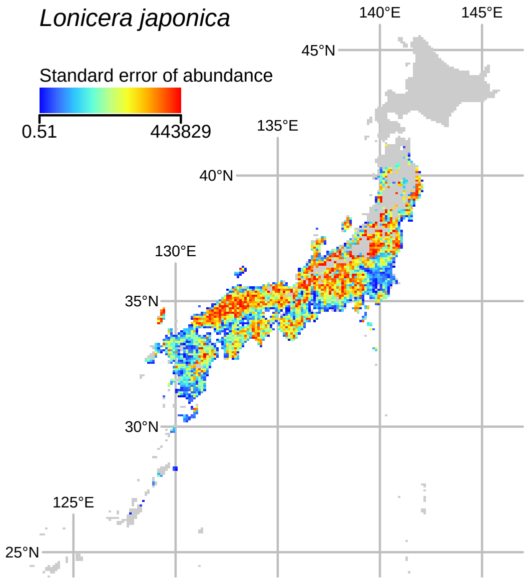
Lonicera japonica
Standard error of abundance
0.51
443829
140°E
145°E
45°N
135°E
40°N
130°E
35°N
30°N
125°E
25°N

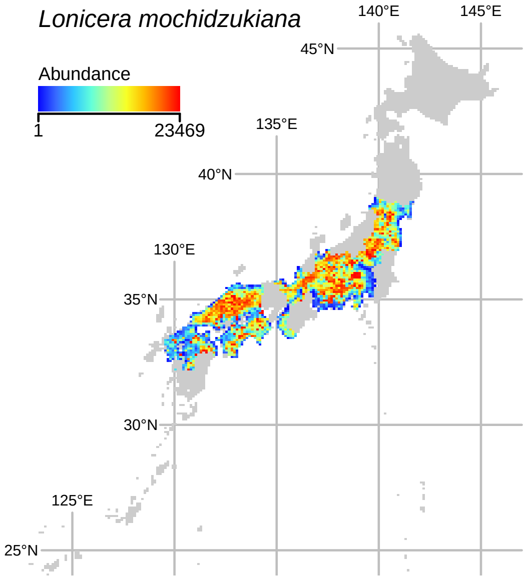
Lonicera mochidzukiana
Abundance
1
23469
140°E
145°E
45°N
135°E
40°N
130°E
35°N
30°N
125°E
25°N

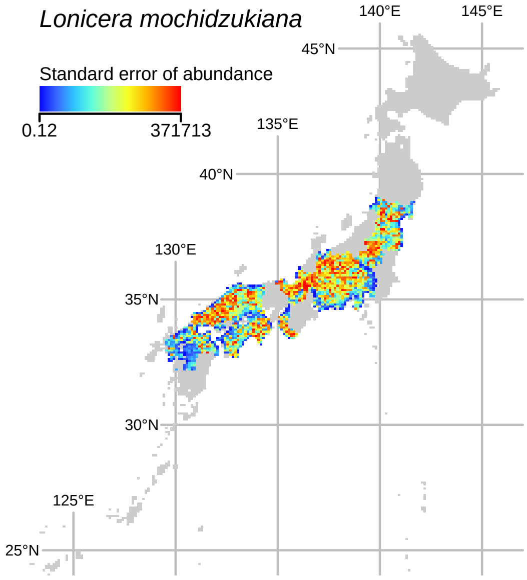
Lonicera mochidzukiana
Standard error of abundance
0.12
371713
140°E
145°E
45°N
135°E
40°N
130°E
35°N
30°N
125°E
25°N

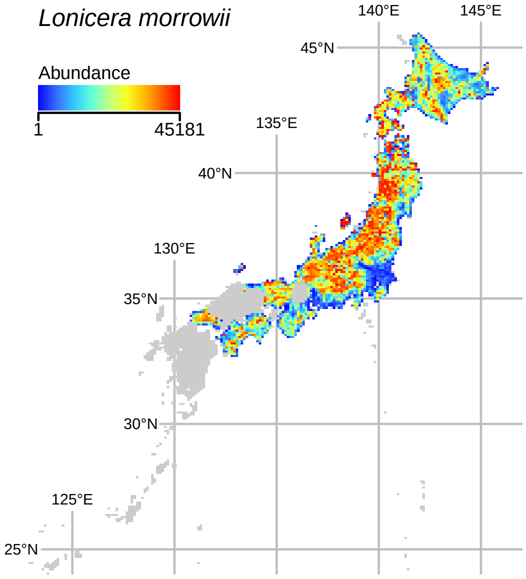
Lonicera morrowii
Abundance
1
45181
140°E
145°E
45°N
135°E
40°N
130°E
35°N
30°N
125°E
25°N

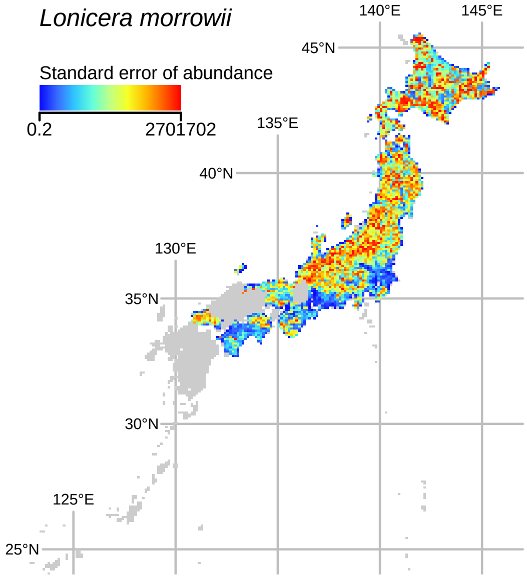
Lonicera morrowii
Standard error of abundance
0.2
2701702
140°E
145°E
45°N
135°E
40°N
130°E
35°N
30°N
125°E
25°N

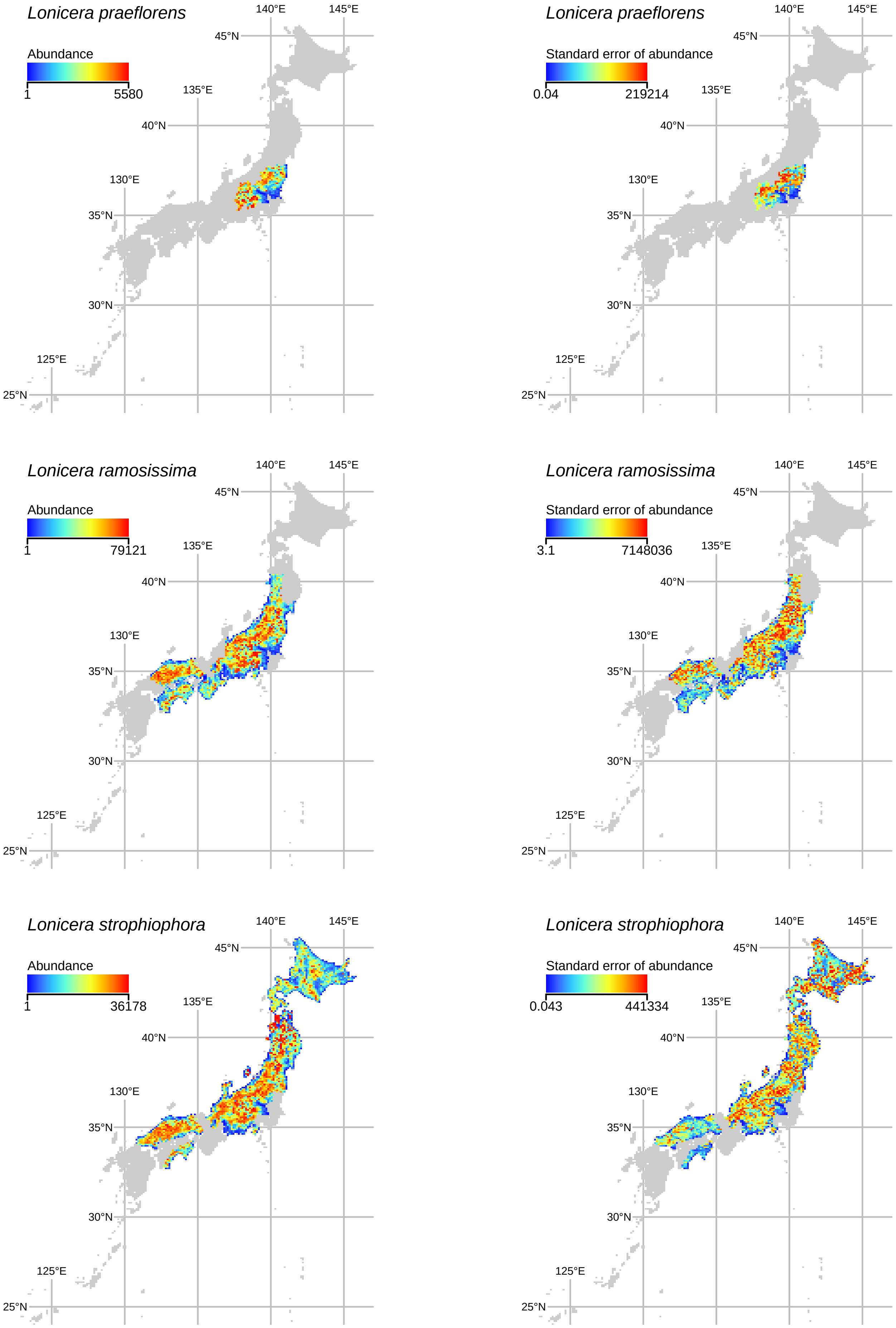
Lonicera praeflorens
Abundance
1
5580
Lonicera praeflorens
Standard error of abundance
0.04
219214
Lonicera ramosissima
Abundance
1
79121
Lonicera ramosissima
Standard error of abundance
3.1
7148036
Lonicera strophiophora
Abundance
1
36178
Lonicera strophiophora
Standard error of abundance
0.043
441334
125°E
130°E
135°E
140°E
145°E
25°N
30°N
35°N
40°N
45°N

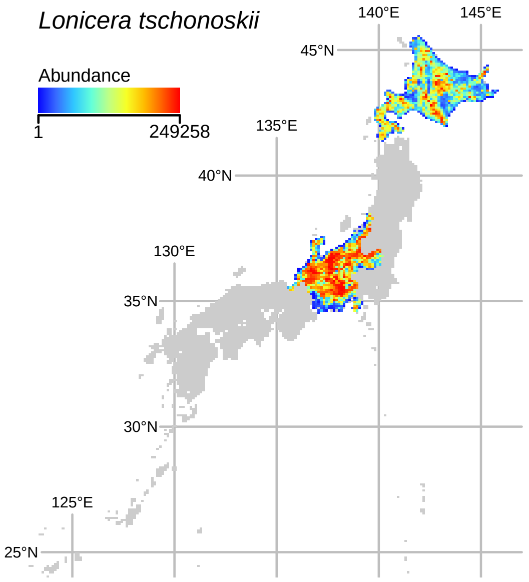
Lonicera tschonoskii
Abundance
1
249258
140°E
145°E
45°N
135°E
40°N
130°E
35°N
30°N
125°E
25°N

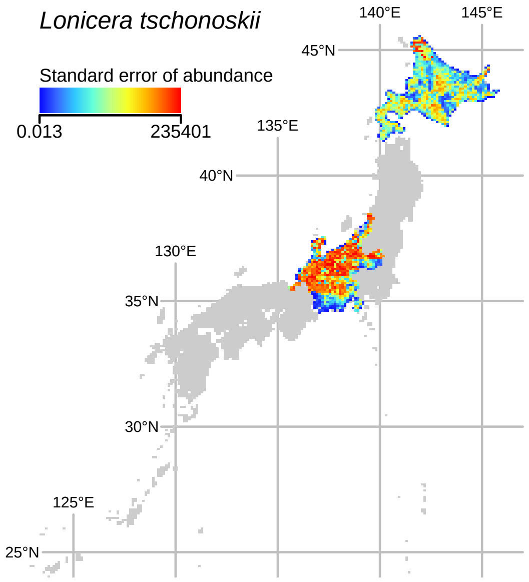
Lonicera tschonoskii
Standard error of abundance
0.013
235401
140°E
145°E
45°N
135°E
40°N
130°E
35°N
30°N
125°E
25°N

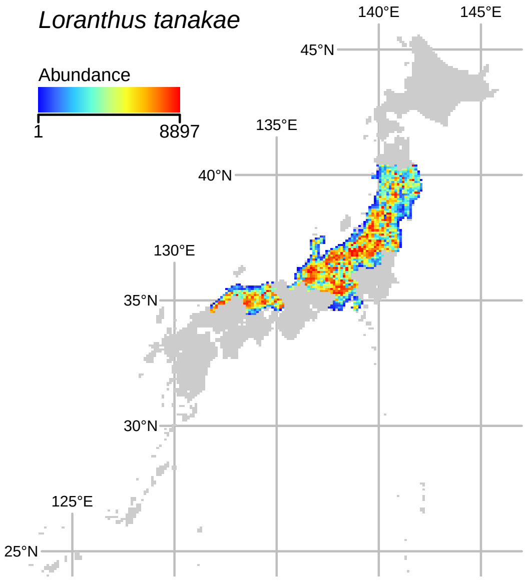
Loranthus tanakae
Abundance
1
8897
140°E
145°E
45°N
135°E
40°N
130°E
35°N
30°N
125°E
25°N

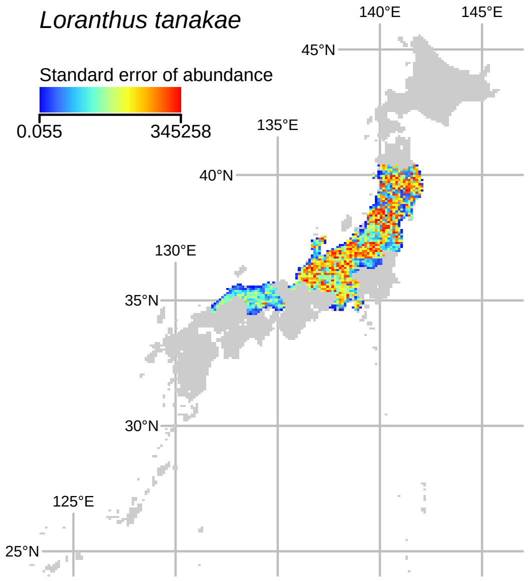
Loranthus tanakae
Standard error of abundance
0.055
345258
140°E
145°E
45°N
135°E
40°N
130°E
35°N
30°N
125°E
25°N

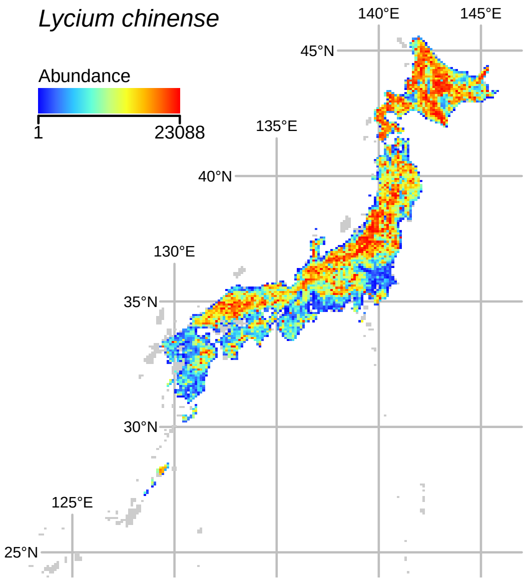
Lycium chinense
Abundance
1
23088
140°E
145°E
45°N
135°E
40°N
130°E
35°N
30°N
125°E
25°N

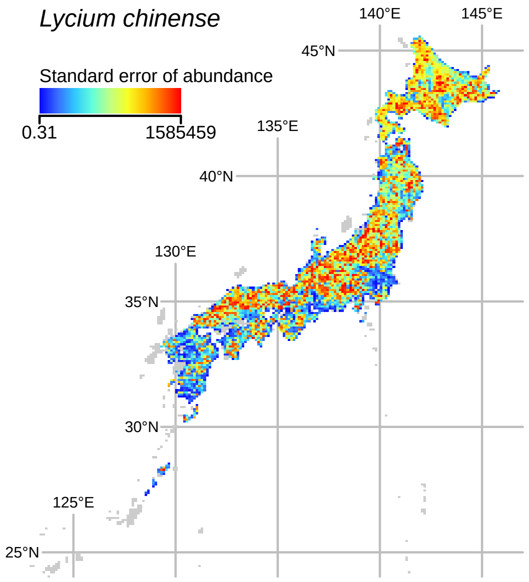
Lycium chinense
Standard error of abundance
0.31
1585459
140°E
145°E
45°N
135°E
40°N
130°E
35°N
30°N
125°E
25°N

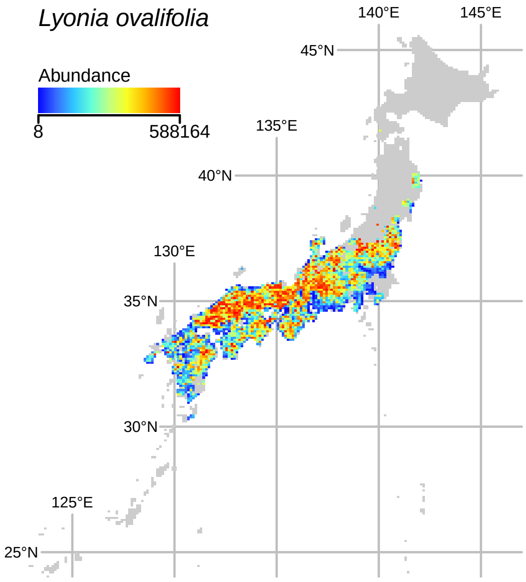
Lyonia ovalifolia
Abundance
8
588164
140°E
145°E
45°N
135°E
40°N
130°E
35°N
30°N
125°E
25°N

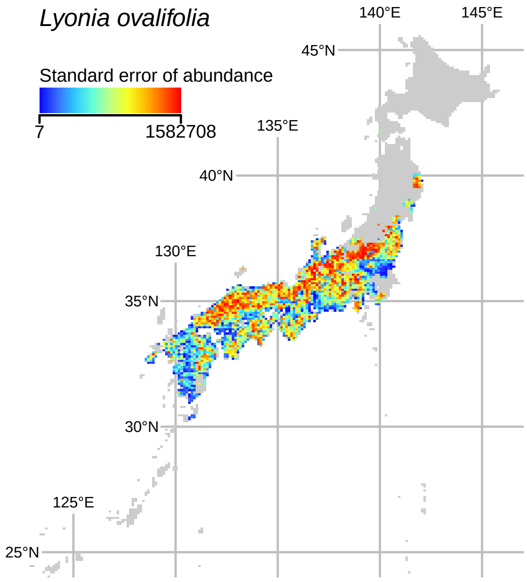
Lyonia ovalifolia
Standard error of abundance
7
1582708
140°E
145°E
45°N
135°E
40°N
130°E
35°N
30°N
125°E
25°N

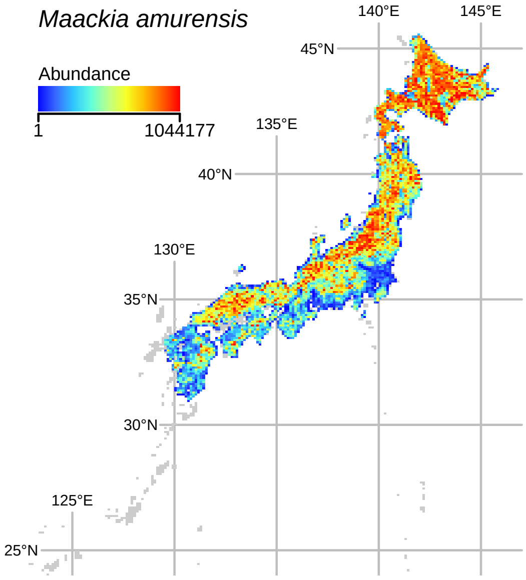
Maackia amurensis
Abundance
1
1044177
140°E
145°E
45°N
135°E
40°N
130°E
35°N
30°N
125°E
25°N

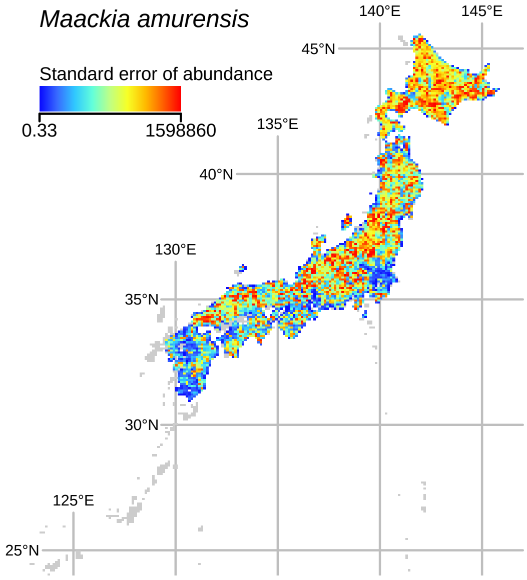
Maackia amurensis
Standard error of abundance
0.33
1598860
140°E
145°E
45°N
135°E
40°N
130°E
35°N
30°N
125°E
25°N

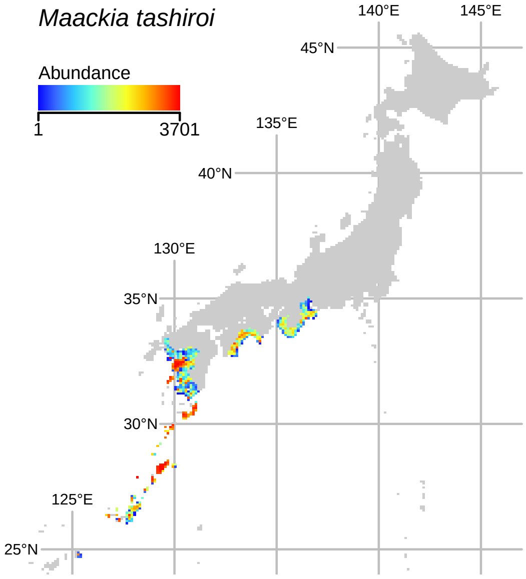
Maackia tashiroi
Abundance
1
3701
140°E
145°E
45°N
135°E
40°N
130°E
35°N
30°N
125°E
25°N

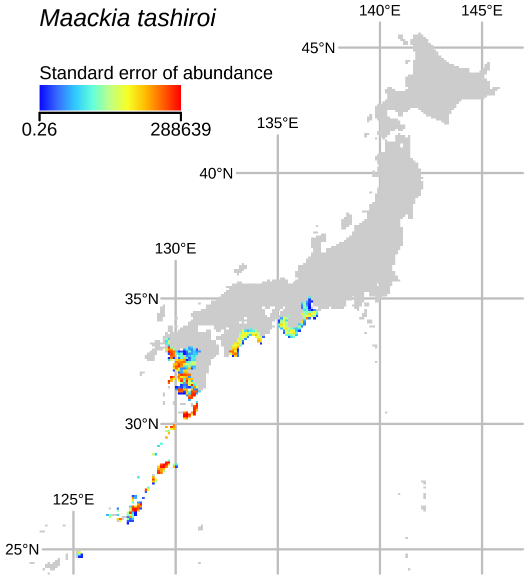
Maackia tashiroi
Standard error of abundance
0.26
288639
140°E
145°E
45°N
135°E
40°N
130°E
35°N
30°N
125°E
25°N

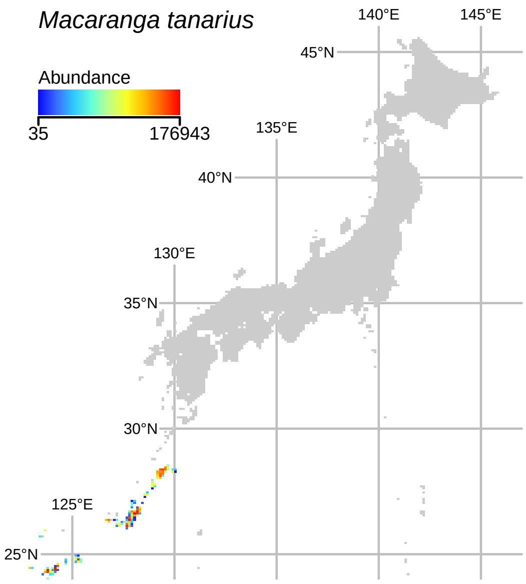
Macaranga tanarius
Abundance
35
176943
140°E
145°E
45°N
135°E
40°N
130°E
35°N
30°N
125°E
25°N

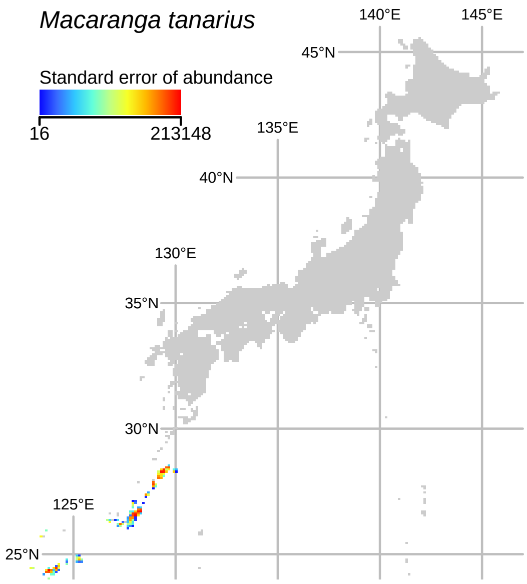
Macaranga tanarius
Standard error of abundance
16
213148
140°E
145°E
45°N
135°E
40°N
130°E
35°N
30°N
125°E
25°N

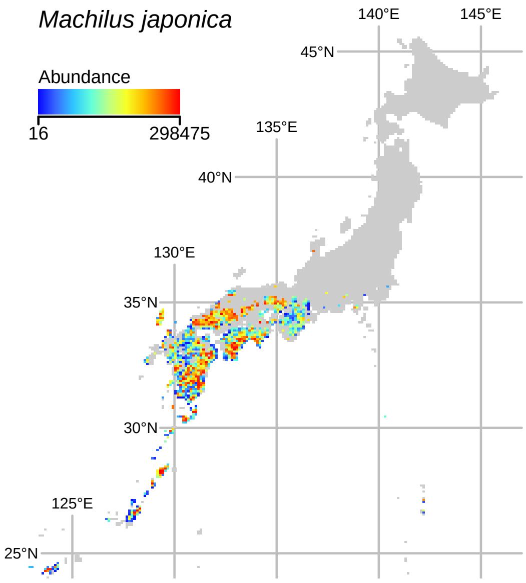
Machilus japonica
Abundance
16
298475
140°E
145°E
45°N
135°E
40°N
130°E
35°N
30°N
125°E
25°N

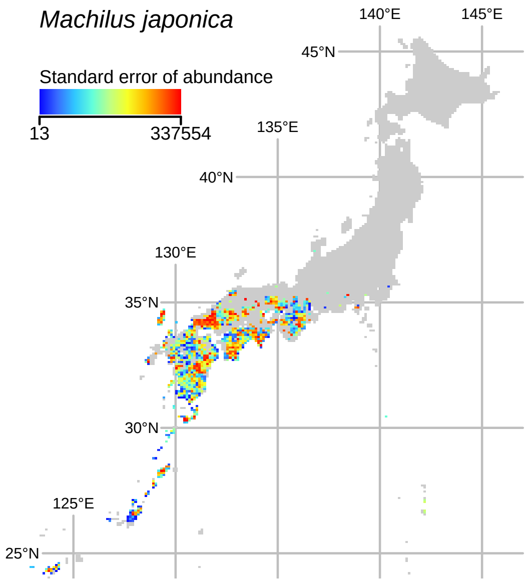
Machilus japonica
Standard error of abundance
13
337554
140°E
145°E
45°N
135°E
40°N
130°E
35°N
30°N
125°E
25°N

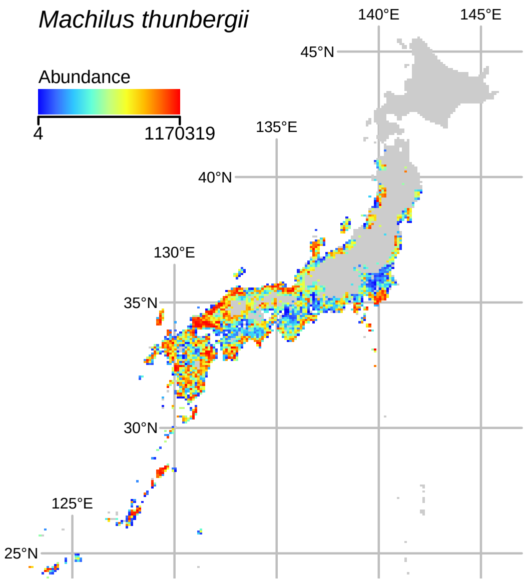
Machilus thunbergii
Abundance
4
1170319
140°E
145°E
45°N
135°E
40°N
130°E
35°N
30°N
125°E
25°N

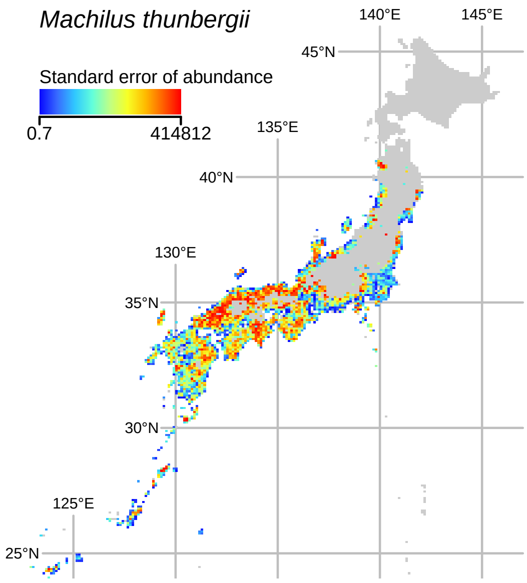
Machilus thunbergii
Standard error of abundance
0.7
414812
140°E
145°E
45°N
135°E
40°N
130°E
35°N
30°N
125°E
25°N

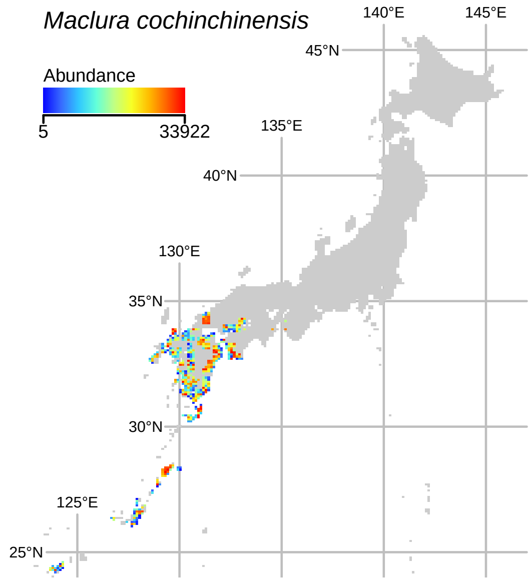

Maclura cochinchinensis
Abundance
5
33922
140°E
145°E
45°N
135°E
40°N
130°E
35°N
30°N
125°E
25°N

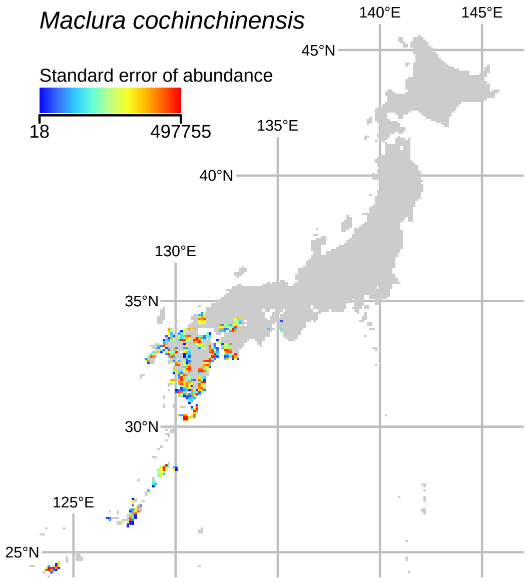

Maclura cochinchinensis
Standard error of abundance
18
497755
140°E
145°E
45°N
135°E
40°N
130°E
35°N
30°N
125°E
25°N

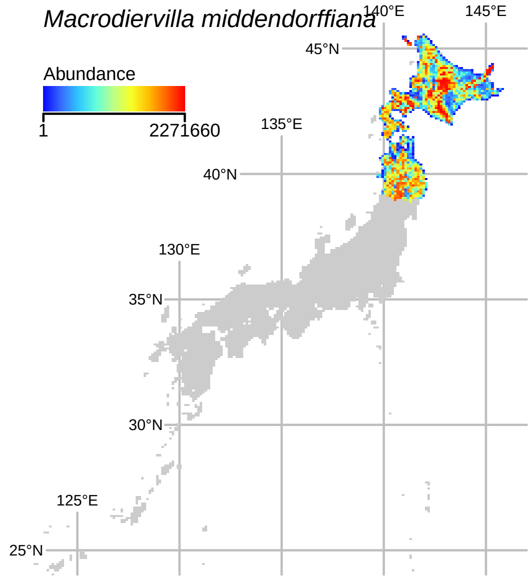

Macrodiervilla middendorffiana
Abundance
1
2271660
140°E
145°E
45°N
135°E
40°N
130°E
35°N
30°N
125°E
25°N

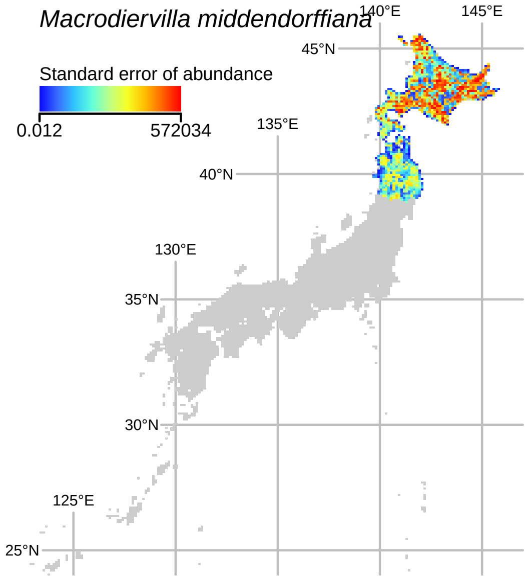

Macrodiervilla middendorffiana
Standard error of abundance
0.012
572034
140°E
145°E
45°N
135°E
40°N
130°E
35°N
30°N
125°E
25°N

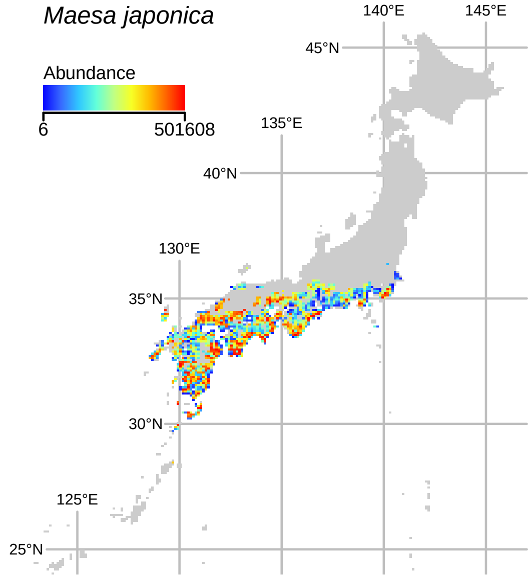

Maesa japonica
Abundance
6
501608
140°E
145°E
45°N
135°E
40°N
130°E
35°N
30°N
125°E
25°N

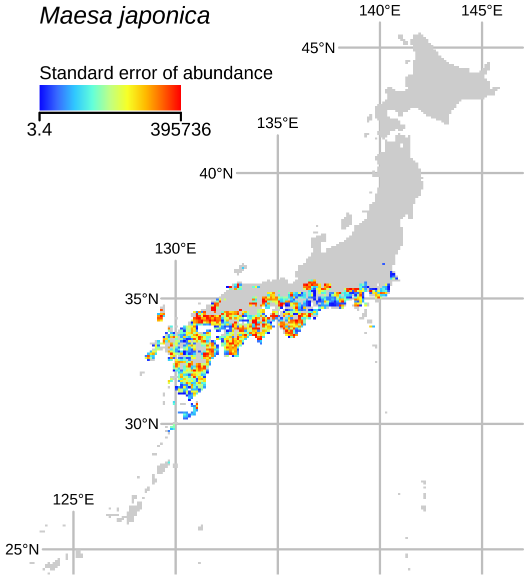

Maesa japonica
Standard error of abundance
3.4
395736
140°E
145°E
45°N
135°E
40°N
130°E
35°N
30°N
125°E
25°N

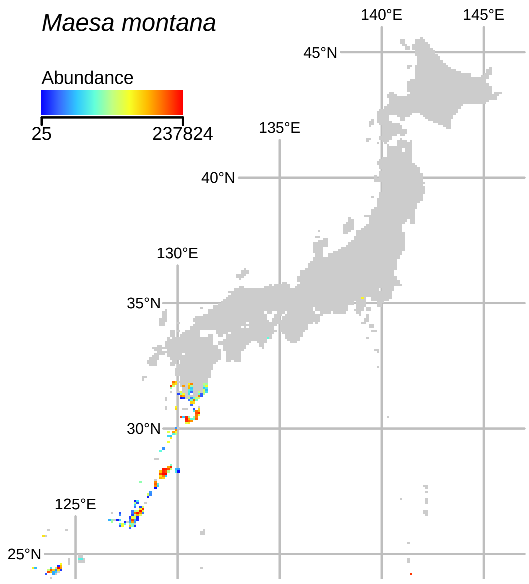
Maesa montana
Abundance
25
237824
140°E
145°E
45°N
135°E
40°N
130°E
35°N
30°N
125°E
25°N

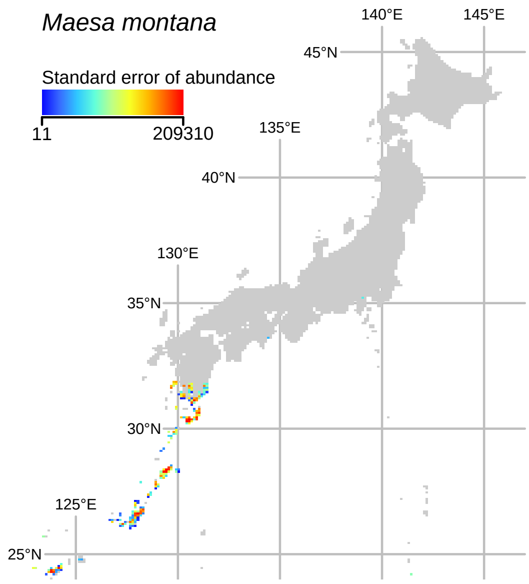
Maesa montana
Standard error of abundance
11
209310
140°E
145°E
45°N
135°E
40°N
130°E
35°N
30°N
125°E
25°N

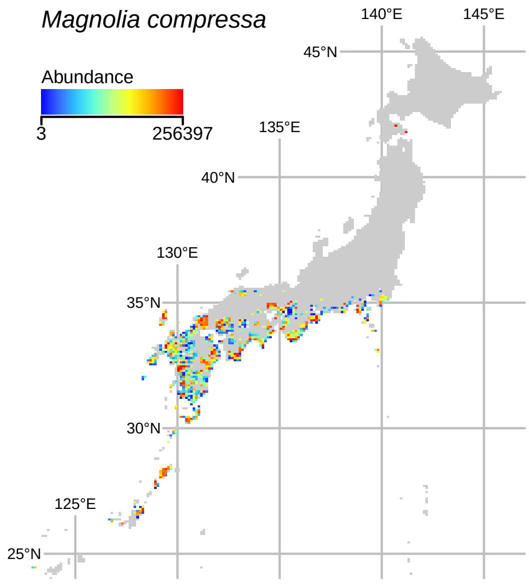
Magnolia compressa
Abundance
3
256397
140°E
145°E
45°N
135°E
40°N
130°E
35°N
30°N
125°E
25°N

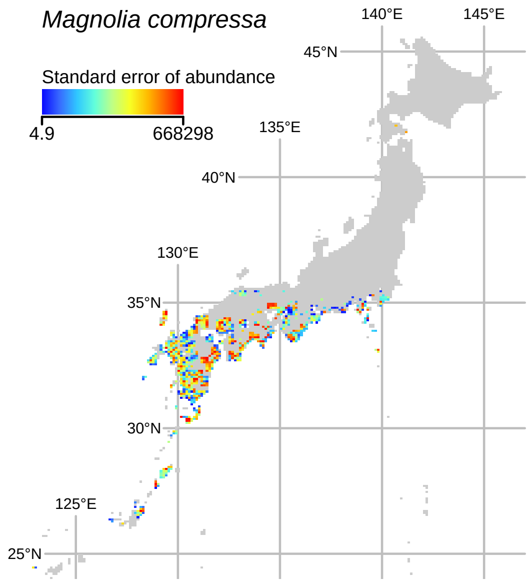
Magnolia compressa
Standard error of abundance
4.9
668298
140°E
145°E
45°N
135°E
40°N
130°E
35°N
30°N
125°E
25°N

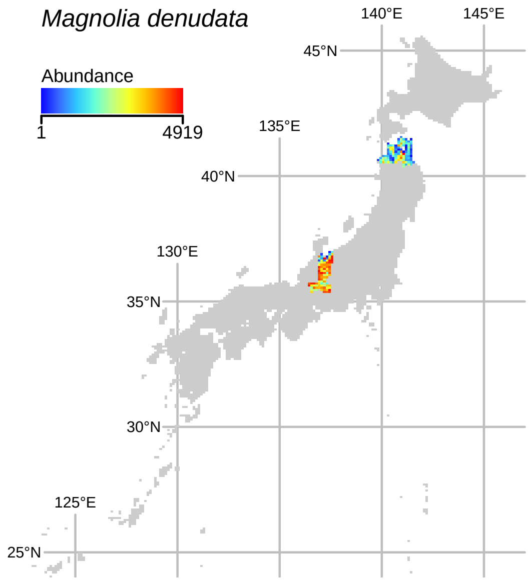
Magnolia denudata
Abundance
1
4919
140°E
145°E
45°N
135°E
40°N
130°E
35°N
30°N
125°E
25°N

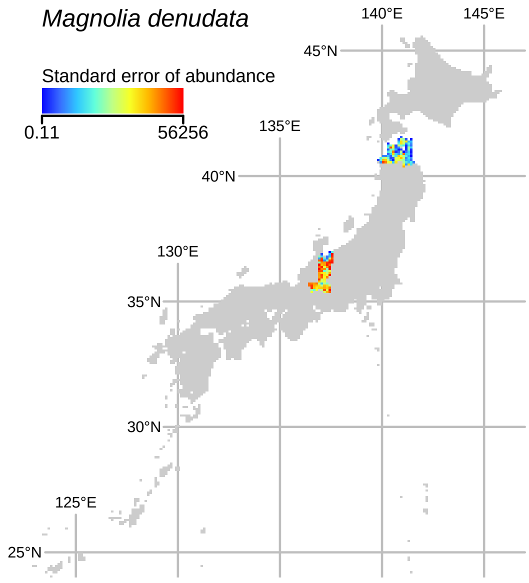
Magnolia denudata
Standard error of abundance
0.11
56256
140°E
145°E
45°N
135°E
40°N
130°E
35°N
30°N
125°E
25°N

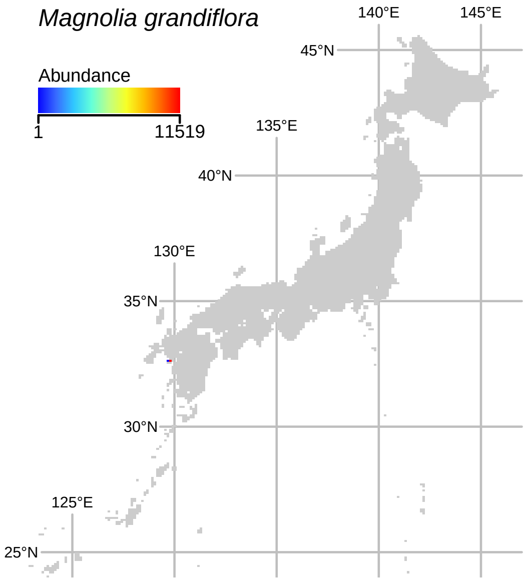
Magnolia grandiflora
Abundance
1
11519
140°E
145°E
45°N
135°E
40°N
130°E
35°N
30°N
125°E
25°N

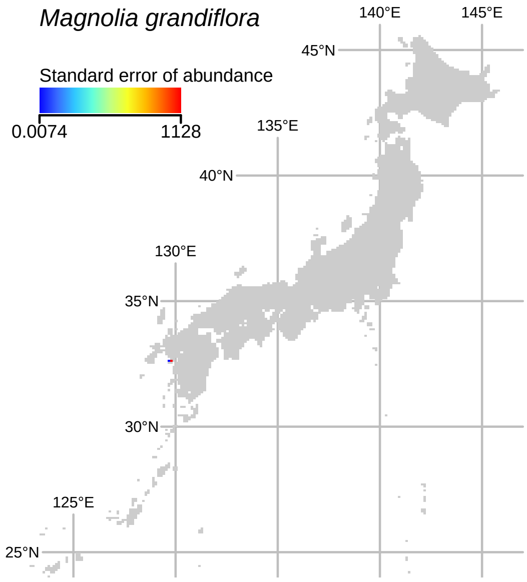
Magnolia grandiflora
Standard error of abundance
0.0074
1128
140°E
145°E
45°N
135°E
40°N
130°E
35°N
30°N
125°E
25°N

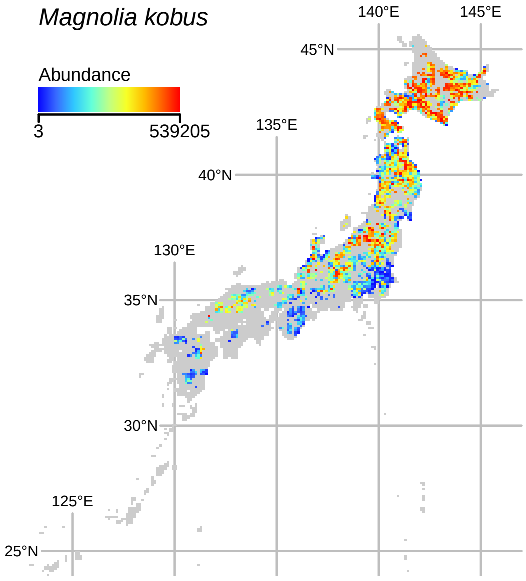
Magnolia kobus
Abundance
3
539205
140°E
145°E
45°N
135°E
40°N
130°E
35°N
30°N
125°E
25°N

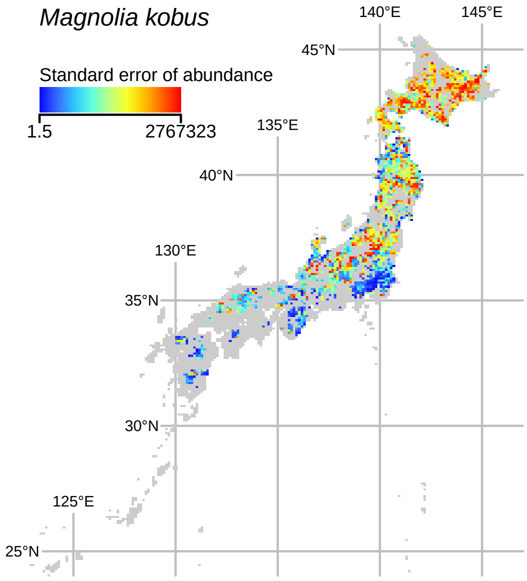
Magnolia kobus
Standard error of abundance
1.5
2767323
140°E
145°E
45°N
135°E
40°N
130°E
35°N
30°N
125°E
25°N

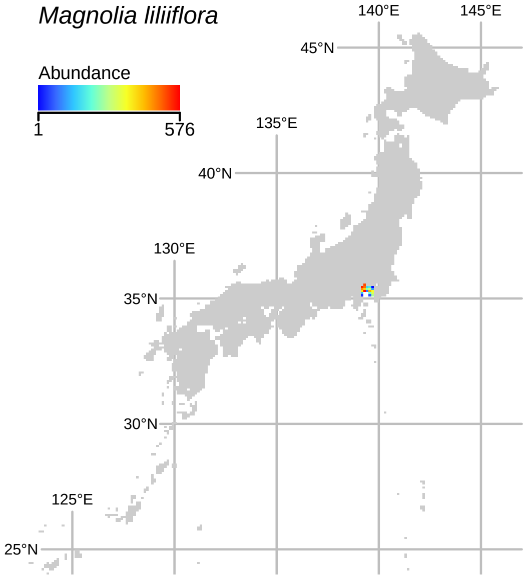
Magnolia liliiflora
Abundance
1
576
140°E
145°E
45°N
135°E
40°N
130°E
35°N
30°N
125°E
25°N

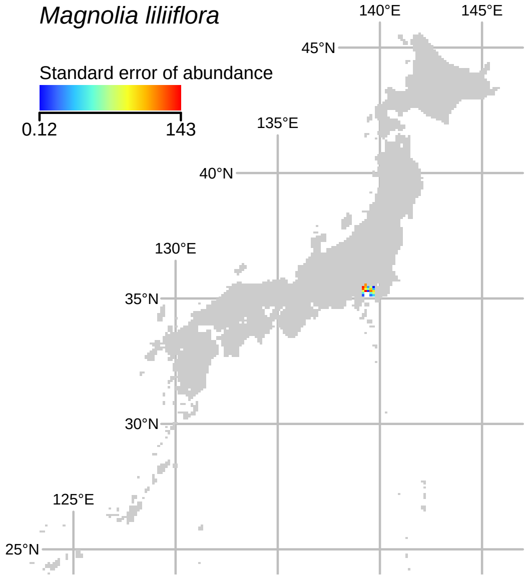
Magnolia liliiflora
Standard error of abundance
0.12
143
140°E
145°E
45°N
135°E
40°N
130°E
35°N
30°N
125°E
25°N

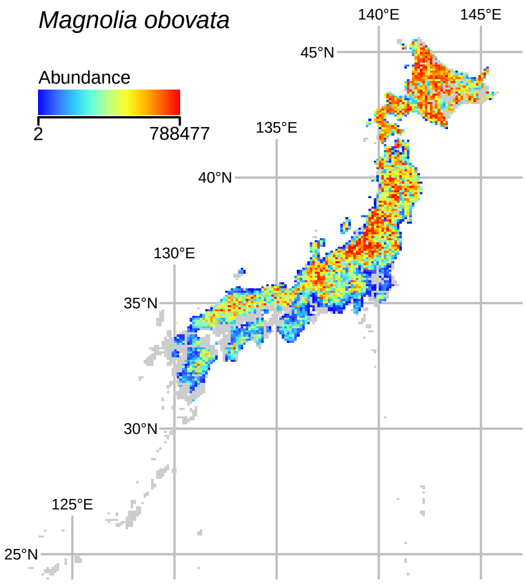
Magnolia obovata
Abundance
2
788477
140°E
145°E
45°N
135°E
40°N
130°E
35°N
30°N
125°E
25°N

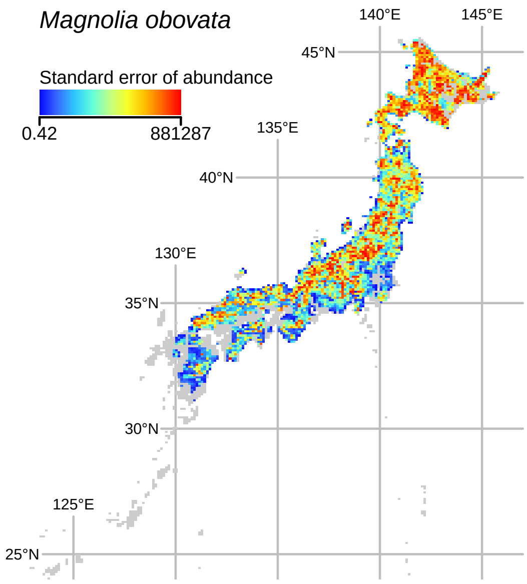
Magnolia obovata
Standard error of abundance
0.42
881287
140°E
145°E
45°N
135°E
40°N
130°E
35°N
30°N
125°E
25°N

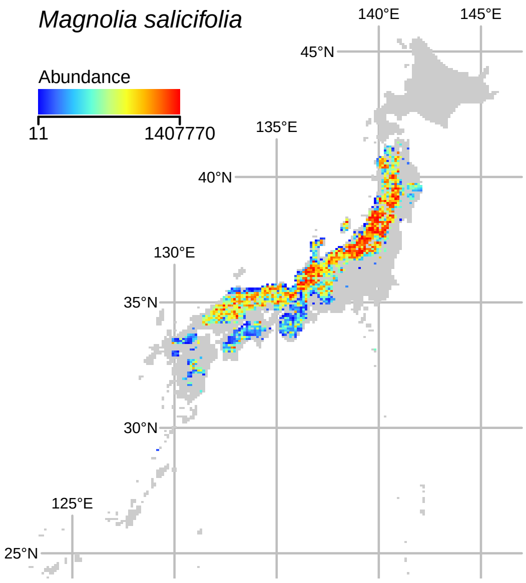
Magnolia salicifolia
Abundance
11
1407770
140°E
145°E
45°N
135°E
40°N
130°E
35°N
30°N
125°E
25°N

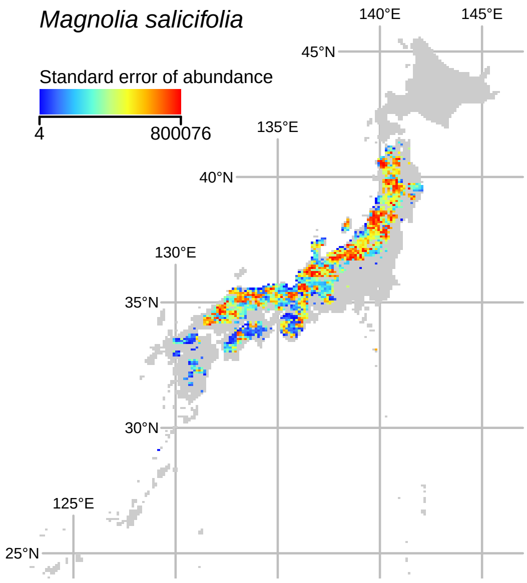
Magnolia salicifolia
Standard error of abundance
4
800076
140°E
145°E
45°N
135°E
40°N
130°E
35°N
30°N
125°E
25°N

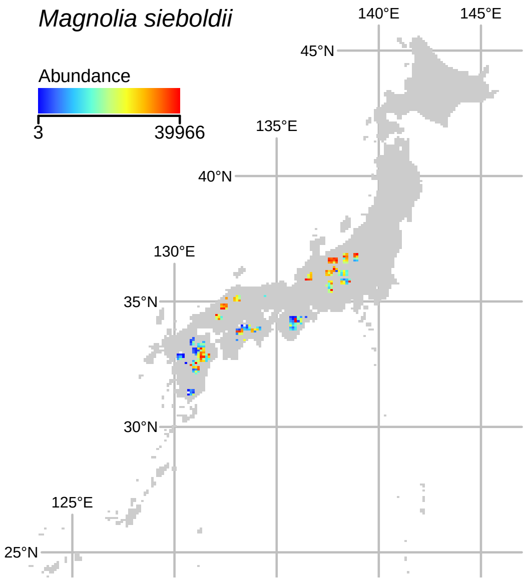
Magnolia sieboldii
Abundance
3
39966
140°E
145°E
45°N
135°E
40°N
130°E
35°N
30°N
125°E
25°N

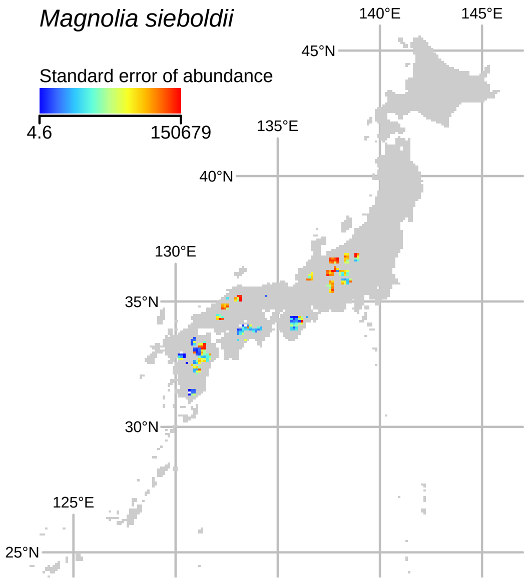
Magnolia sieboldii
Standard error of abundance
4.6
150679
140°E
145°E
45°N
135°E
40°N
130°E
35°N
30°N
125°E
25°N

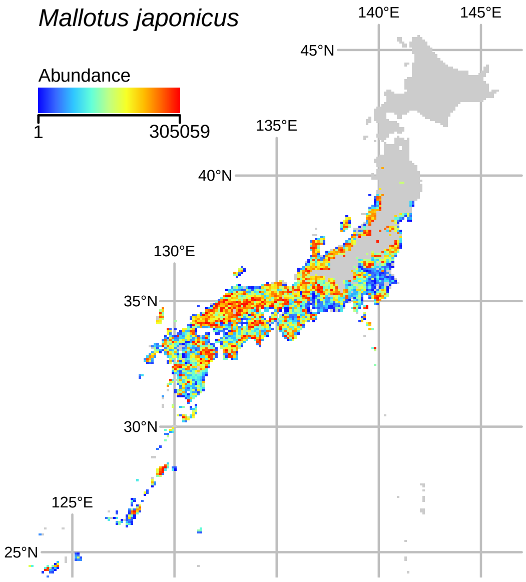
Mallotus japonicus
Abundance
1
305059
140°E
145°E
45°N
135°E
40°N
130°E
35°N
30°N
125°E
25°N

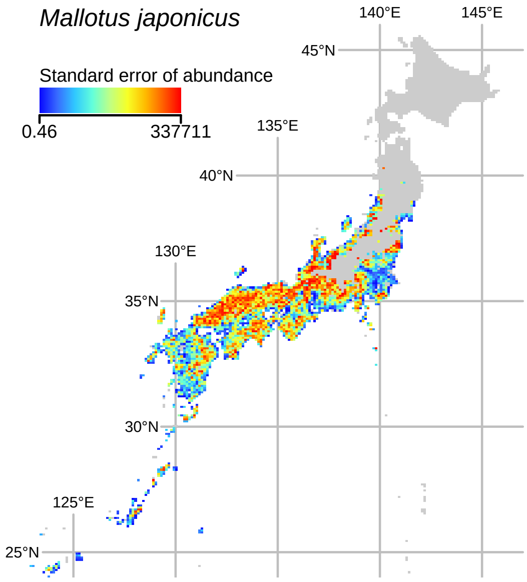
Mallotus japonicus
Standard error of abundance
0.46
337711
140°E
145°E
45°N
135°E
40°N
130°E
35°N
30°N
125°E
25°N

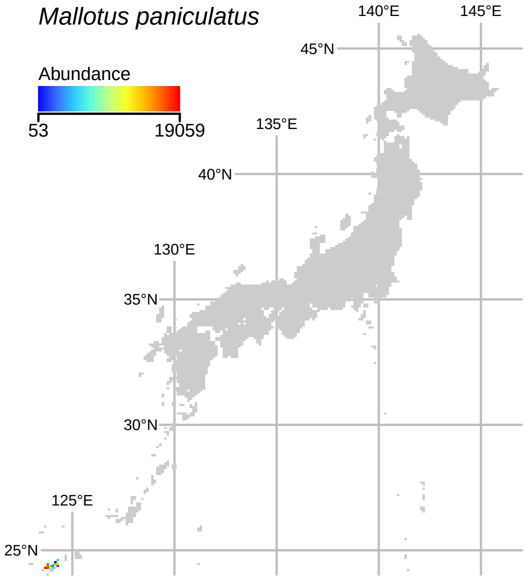
Mallotus paniculatus
Abundance
53
19059
140°E
145°E
45°N
135°E
40°N
130°E
35°N
30°N
125°E
25°N

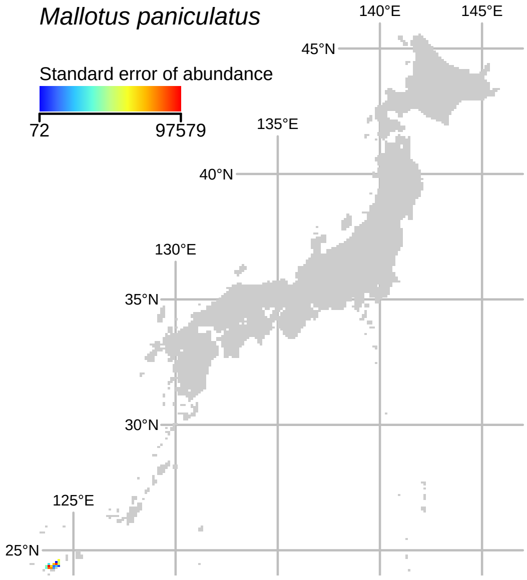
Mallotus paniculatus
Standard error of abundance
72
97579
140°E
145°E
45°N
135°E
40°N
130°E
35°N
30°N
125°E
25°N

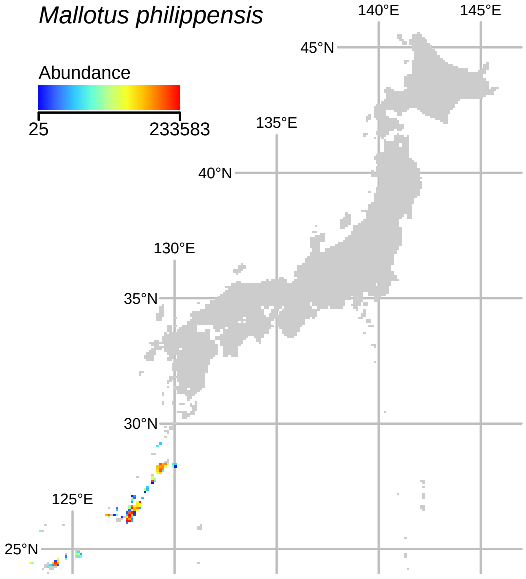
Mallotus philippensis
Abundance
25
233583
140°E
145°E
45°N
135°E
40°N
130°E
35°N
30°N
125°E
25°N

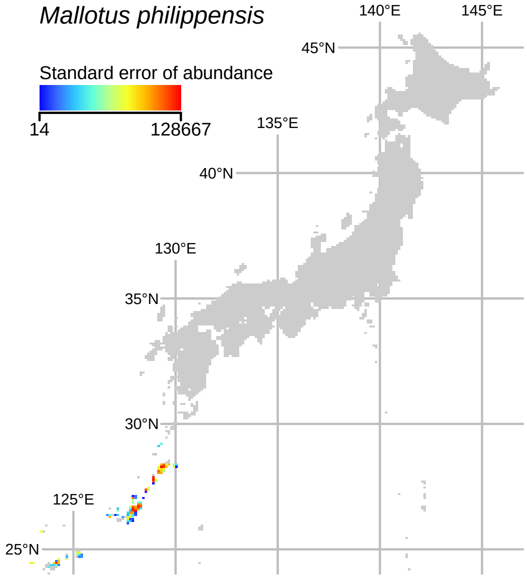
Mallotus philippensis
Standard error of abundance
14
128667
140°E
145°E
45°N
135°E
40°N
130°E
35°N
30°N
125°E
25°N

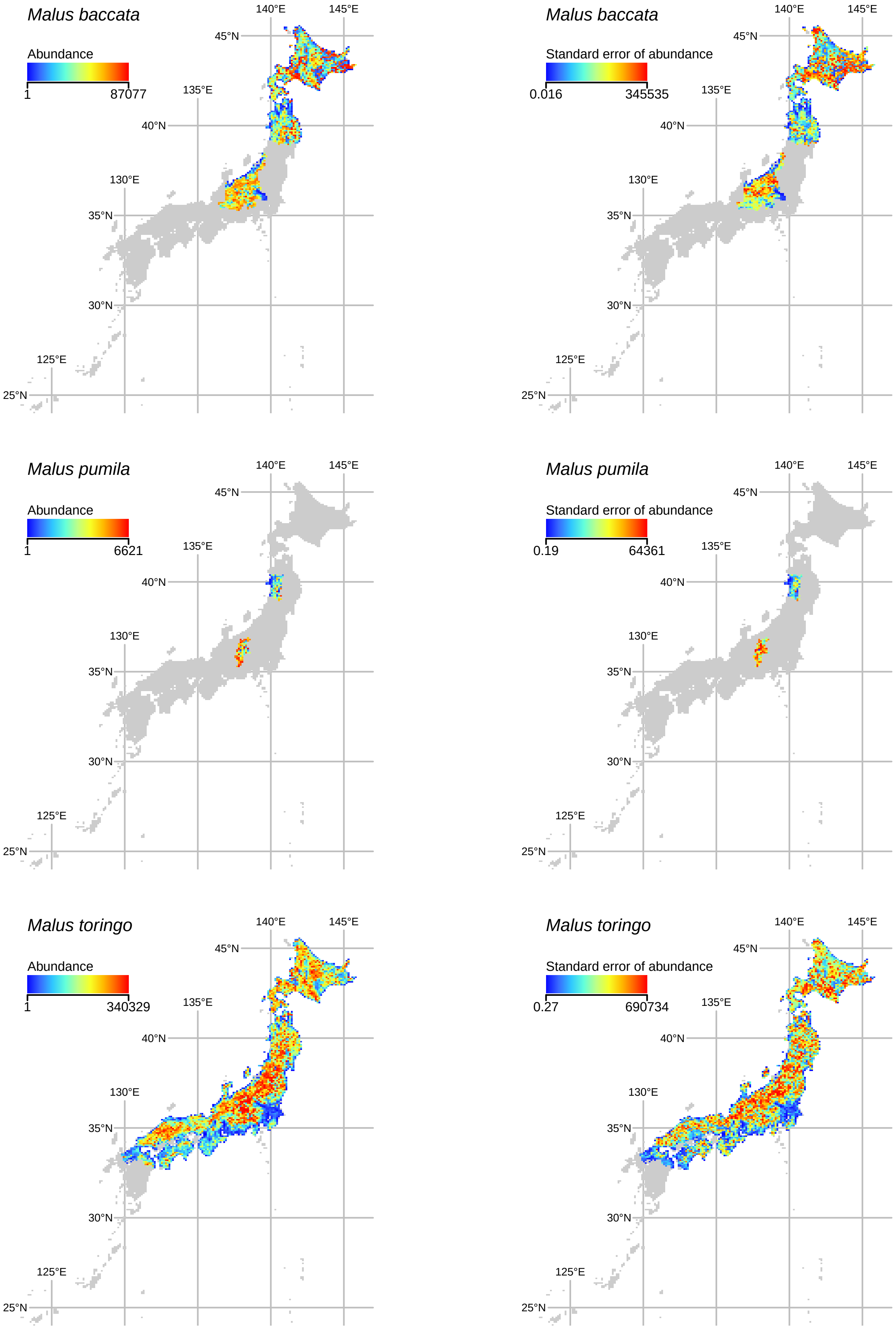
Malus baccata
Abundance
1
87077
Malus baccata
Standard error of abundance
0.016
345535
Malus pumila
Abundance
1
6621
Malus pumila
Standard error of abundance
0.19
64361
Malus toringo
Abundance
1
340329
Malus toringo
Standard error of abundance
0.27
690734
125°E
130°E
135°E
140°E
145°E
25°N
30°N
35°N
40°N
45°N

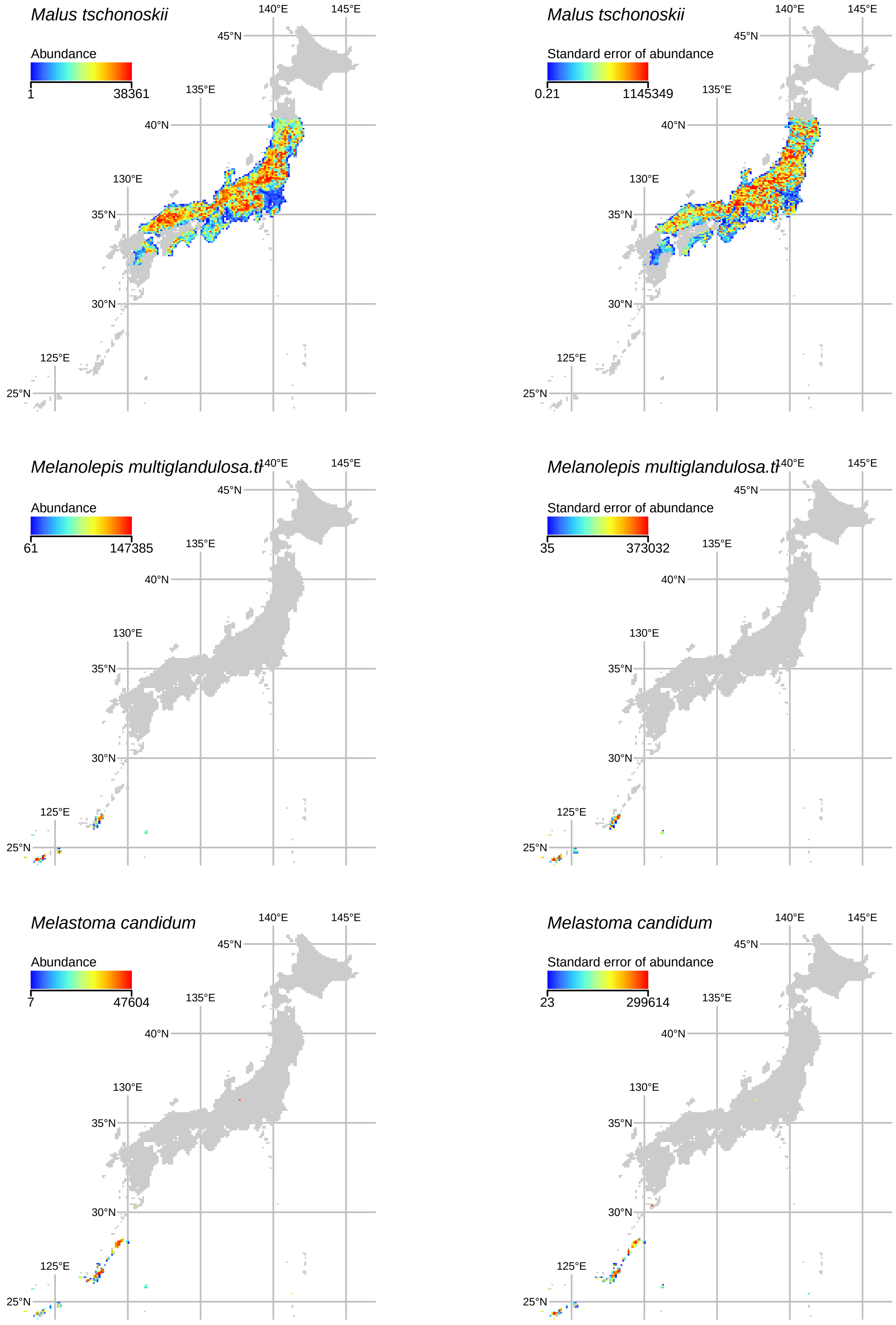
Malus tschonoskii
Abundance
1
38361
Malus tschonoskii
Standard error of abundance
0.21
1145349
Melanolepis multiglandulosa.ti
Abundance
61
147385
Melanolepis multiglandulosa.ti
Standard error of abundance
35
373032
Melastoma candidum
Abundance
7
47604
Melastoma candidum
Standard error of abundance
23
299614
125°E
130°E
135°E
140°E
145°E
25°N
30°N
35°N
40°N
45°N

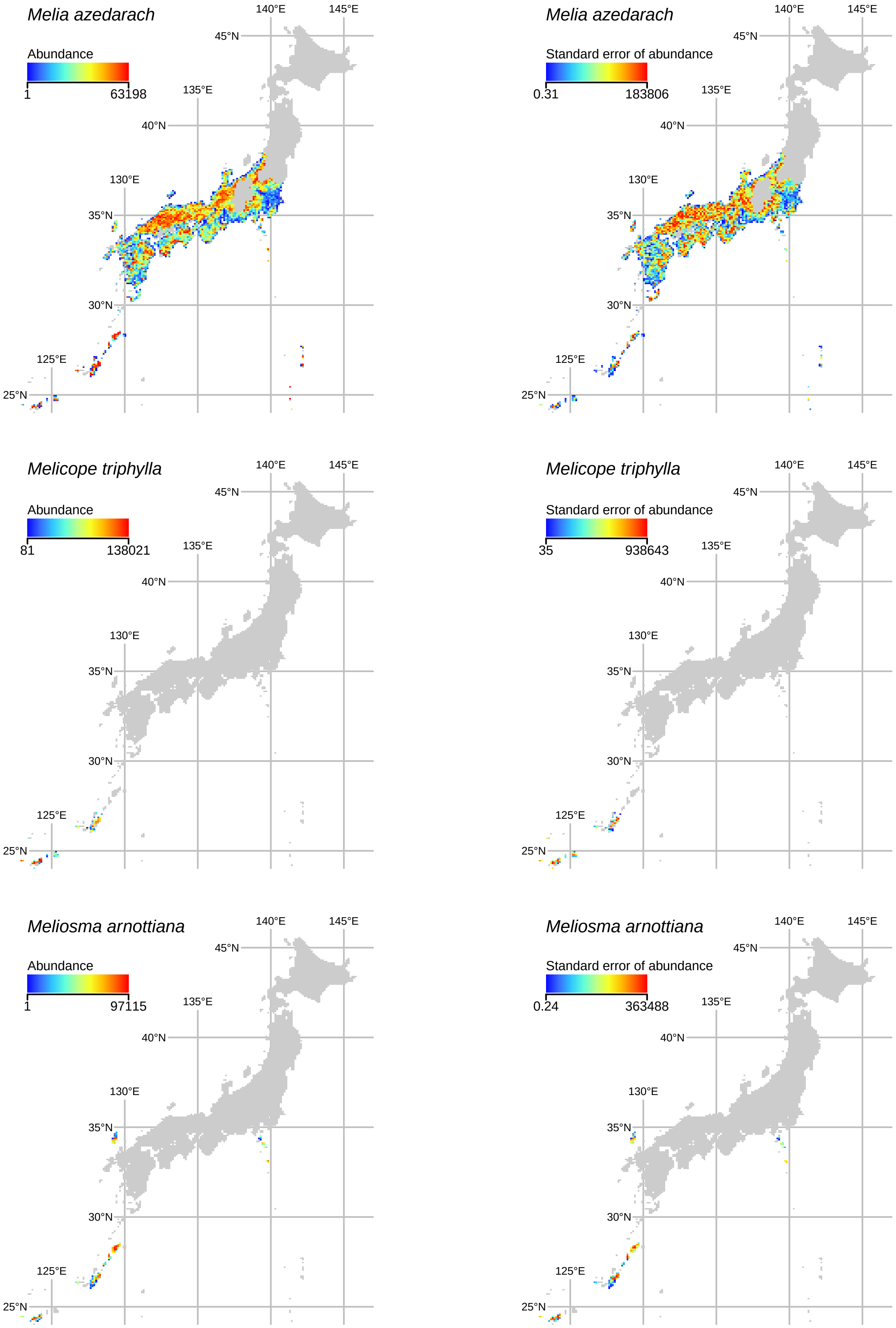
Melia azedarach
Abundance
1
63198
Melia azedarach
Standard error of abundance
0.31
183806
Melicope triphylla
Abundance
81
138021
Melicope triphylla
Standard error of abundance
35
938643
Meliosma arnottiana
Abundance
1
97115
Meliosma arnottiana
Standard error of abundance
0.24
363488
140°E
145°E
135°E
130°E
125°E
45°N
40°N
35°N
30°N
25°N

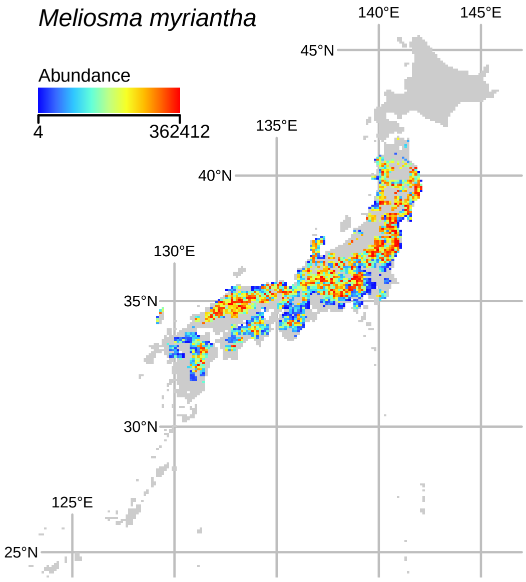
Meliosma myriantha
Abundance
4
362412
140°E
145°E
45°N
135°E
40°N
130°E
35°N
30°N
125°E
25°N

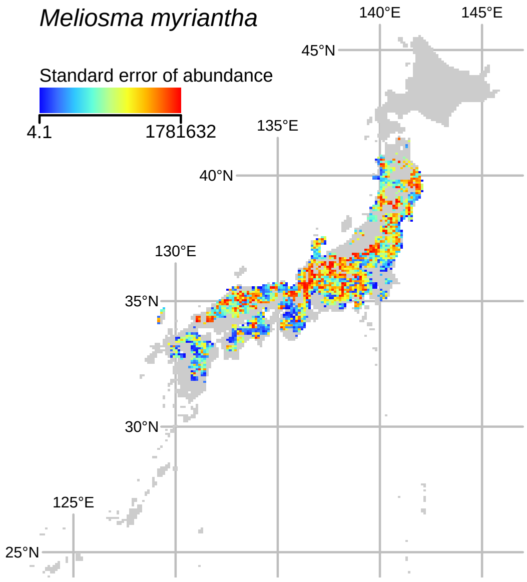
Meliosma myriantha
Standard error of abundance
4.1
1781632
140°E
145°E
45°N
135°E
40°N
130°E
35°N
30°N
125°E
25°N

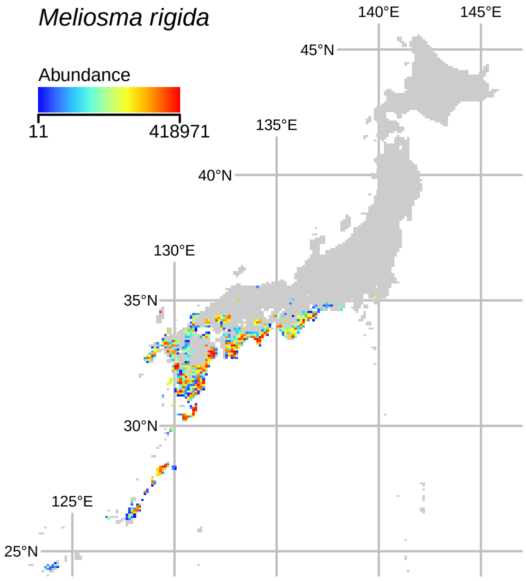
Meliosma rigida
Abundance
11
418971
140°E
145°E
45°N
135°E
40°N
130°E
35°N
30°N
125°E
25°N

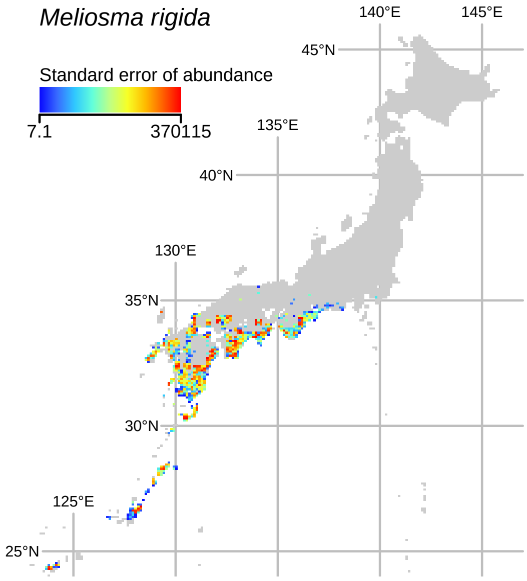
Meliosma rigida
Standard error of abundance
7.1
370115
140°E
145°E
45°N
135°E
40°N
130°E
35°N
30°N
125°E
25°N

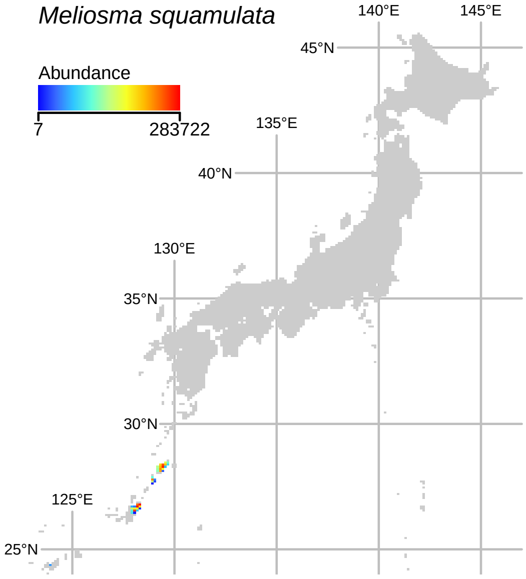
Meliosma squamulata
Abundance
7
283722
140°E
145°E
45°N
135°E
40°N
130°E
35°N
30°N
125°E
25°N

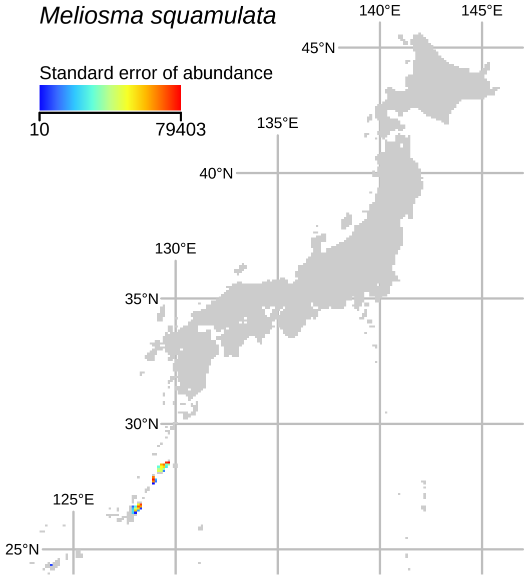
Meliosma squamulata
Standard error of abundance
10
79403
140°E
145°E
45°N
135°E
40°N
130°E
35°N
30°N
125°E
25°N

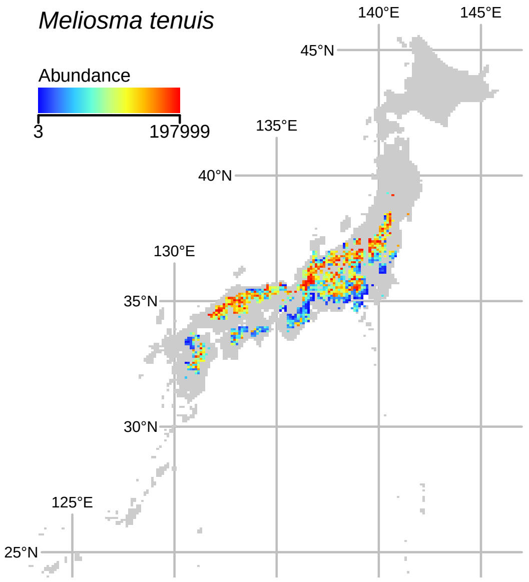
Meliosma tenuis
Abundance
3
197999
140°E
145°E
45°N
135°E
40°N
130°E
35°N
30°N
125°E
25°N

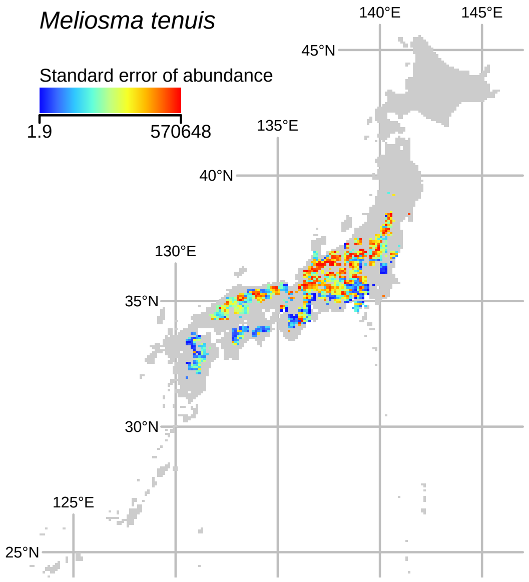
Meliosma tenuis
Standard error of abundance
1.9
570648
140°E
145°E
45°N
135°E
40°N
130°E
35°N
30°N
125°E
25°N

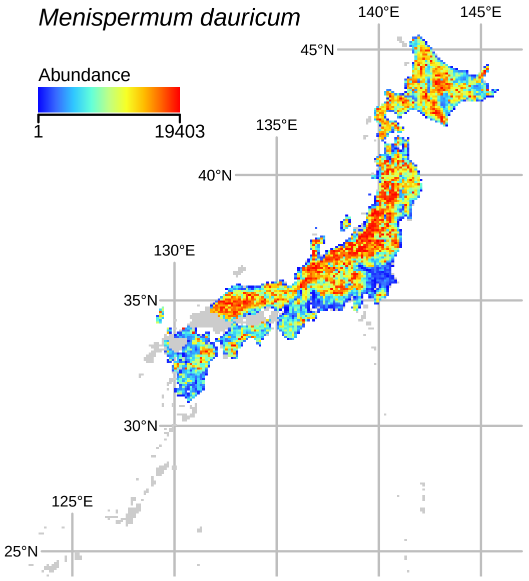
Menispermum dauricum
Abundance
1
19403
140°E
145°E
45°N
135°E
40°N
130°E
35°N
30°N
125°E
25°N

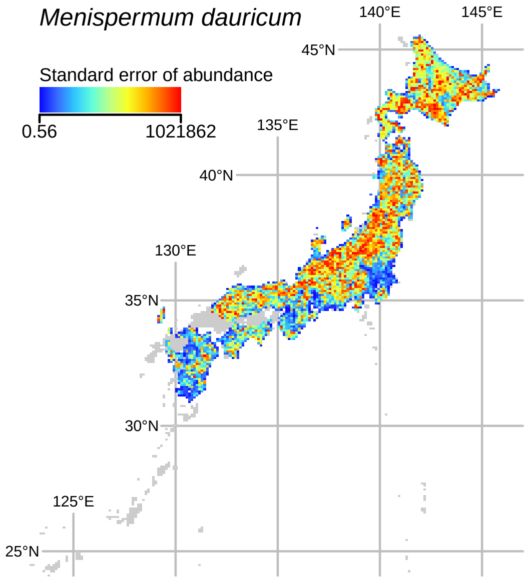
Menispermum dauricum
Standard error of abundance
0.56
1021862
140°E
145°E
45°N
135°E
40°N
130°E
35°N
30°N
125°E
25°N

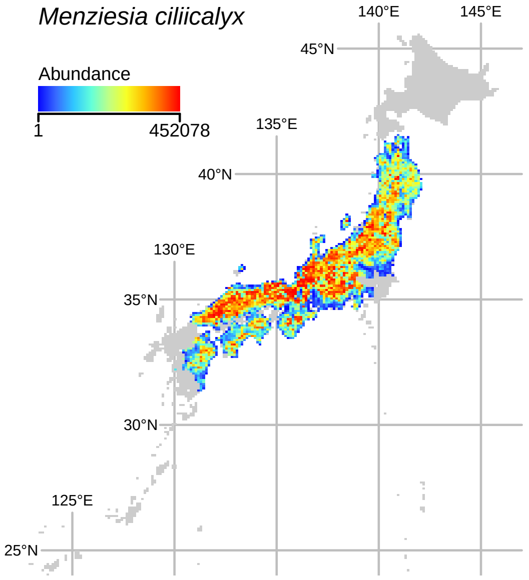
Menziesia ciliicalyx
Abundance
1
452078
140°E
145°E
45°N
135°E
40°N
130°E
35°N
30°N
125°E
25°N

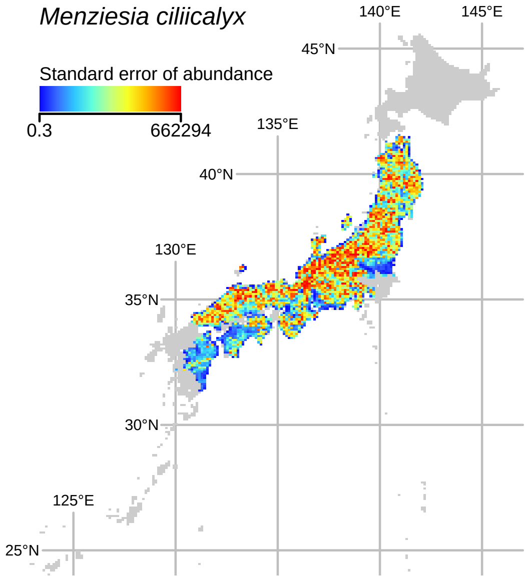
Menziesia ciliicalyx
Standard error of abundance
0.3
662294
140°E
145°E
45°N
135°E
40°N
130°E
35°N
30°N
125°E
25°N

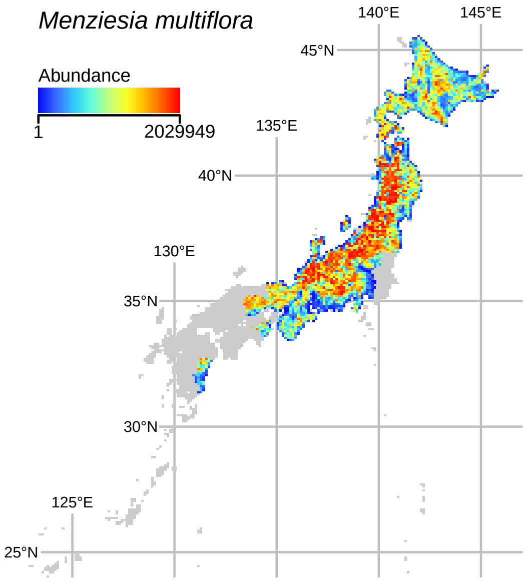
Menziesia multiflora
Abundance
1
2029949
140°E
145°E
45°N
135°E
40°N
130°E
35°N
30°N
125°E
25°N

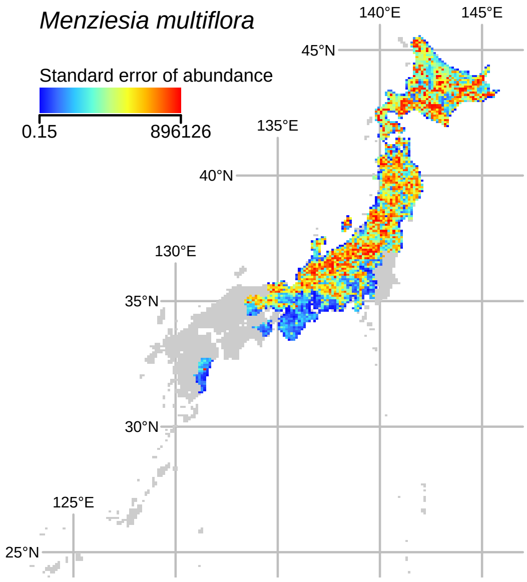
Menziesia multiflora
Standard error of abundance
0.15
896126
140°E
145°E
45°N
135°E
40°N
130°E
35°N
30°N
125°E
25°N

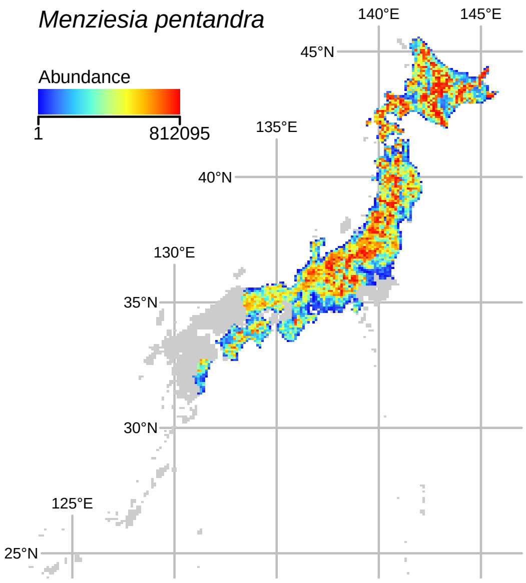
Menziesia pentandra
Abundance
1
812095
140°E
145°E
45°N
135°E
40°N
130°E
35°N
30°N
125°E
25°N

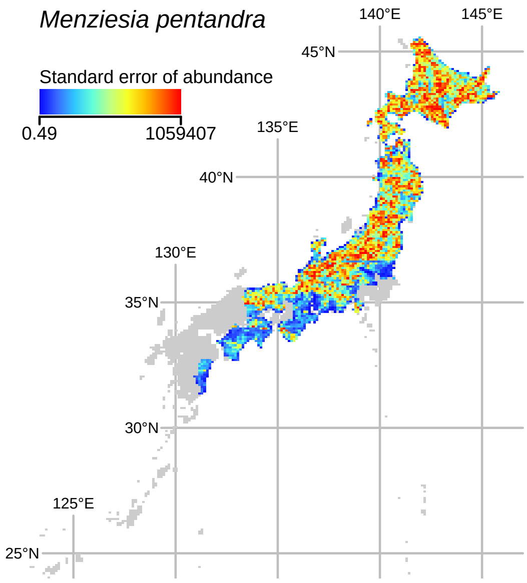
Menziesia pentandra
Standard error of abundance
0.49
1059407
140°E
145°E
45°N
135°E
40°N
130°E
35°N
30°N
125°E
25°N

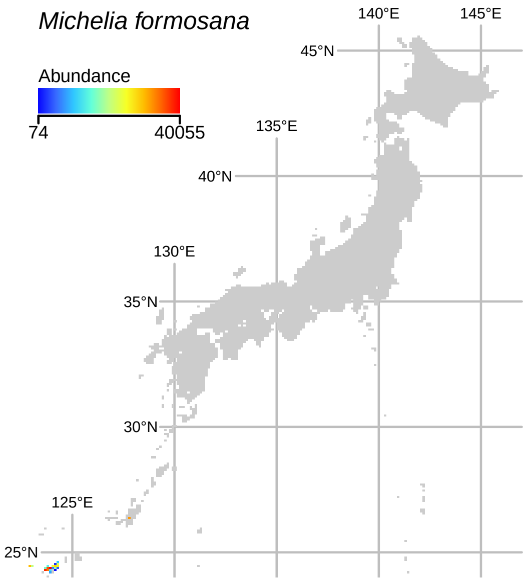
Michelia formosana
Abundance
74
40055
140°E
145°E
45°N
135°E
40°N
130°E
35°N
30°N
125°E
25°N

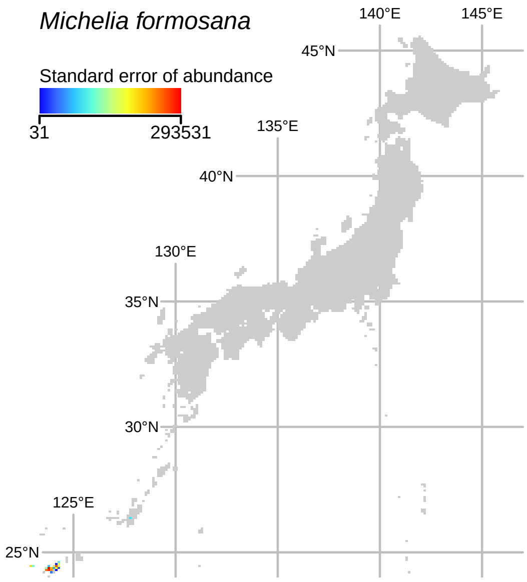
Michelia formosana
Standard error of abundance
31
293531
140°E
145°E
45°N
135°E
40°N
130°E
35°N
30°N
125°E
25°N

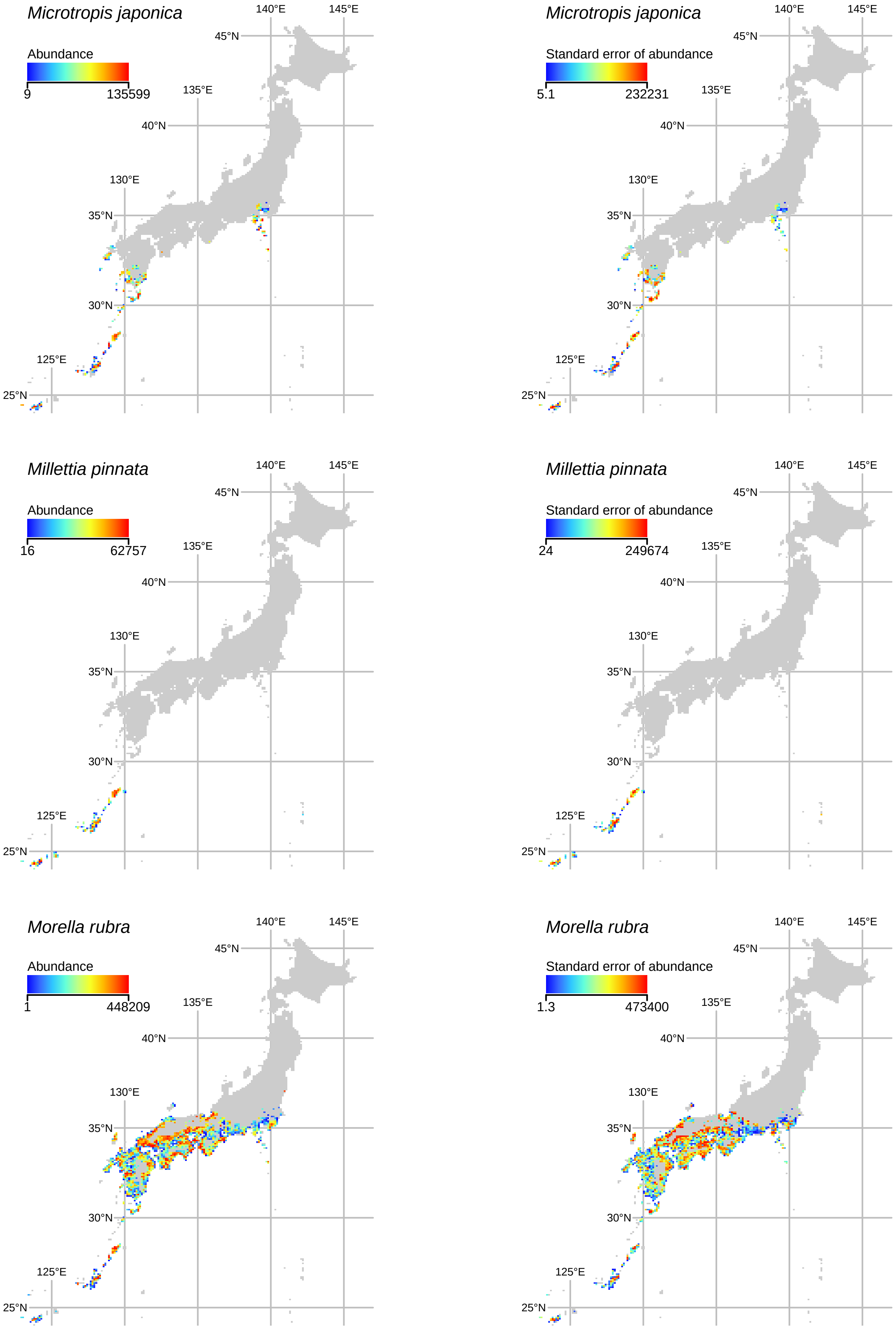
Microtropis japonica
Abundance
9
135599
Microtropis japonica
Standard error of abundance
5.1
232231
Millettia pinnata
Abundance
16
62757
Millettia pinnata
Standard error of abundance
24
249674
Morella rubra
Abundance
1
448209
Morella rubra
Standard error of abundance
1.3
473400
140°E
145°E
135°E
130°E
125°E
45°N
40°N
35°N
30°N
25°N

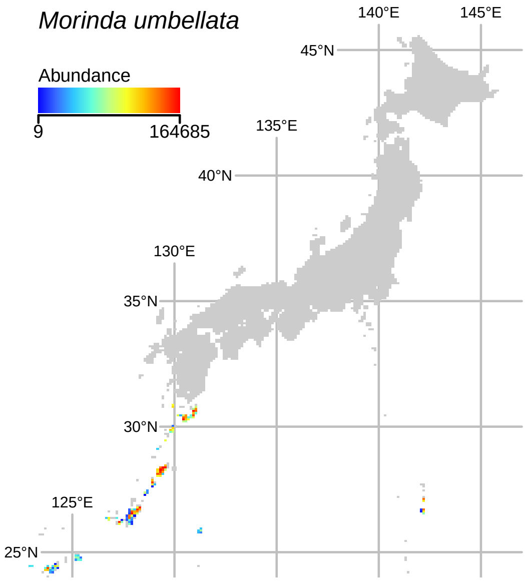
Morinda umbellata
Abundance
9
164685
140°E
145°E
45°N
135°E
40°N
130°E
35°N
30°N
125°E
25°N

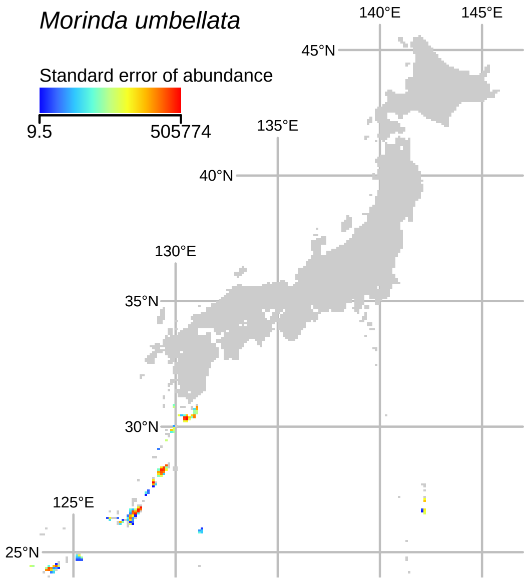
Morinda umbellata
Standard error of abundance
9.5
505774
140°E
145°E
45°N
135°E
40°N
130°E
35°N
30°N
125°E
25°N

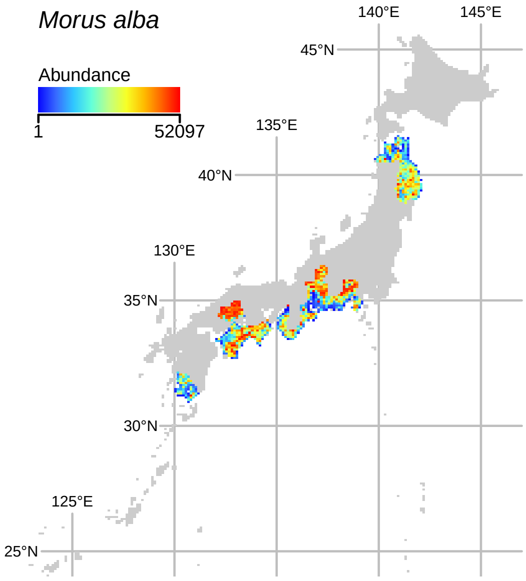
Morus alba
Abundance
1
52097
140°E
145°E
45°N
135°E
40°N
130°E
35°N
30°N
125°E
25°N

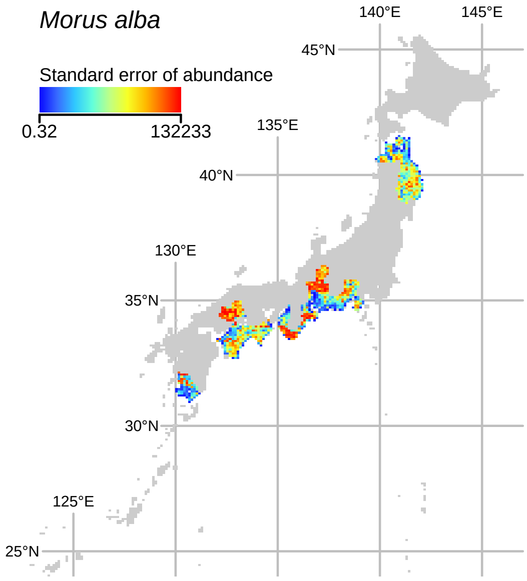
Morus alba
Standard error of abundance
0.32
132233
140°E
145°E
45°N
135°E
40°N
130°E
35°N
30°N
125°E
25°N

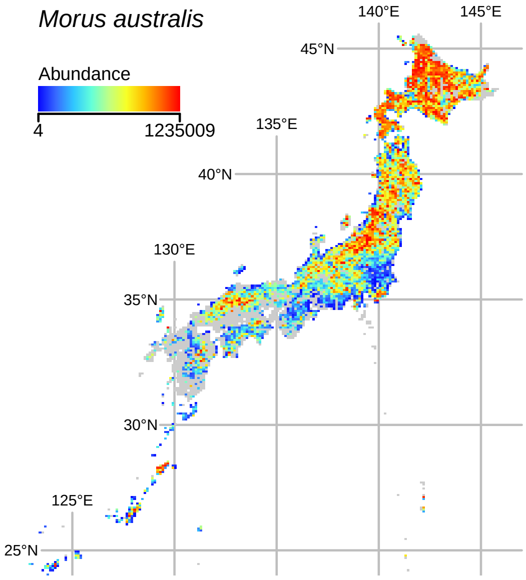
Morus australis
Abundance
4
1235009
140°E
145°E
45°N
135°E
40°N
130°E
35°N
30°N
125°E
25°N

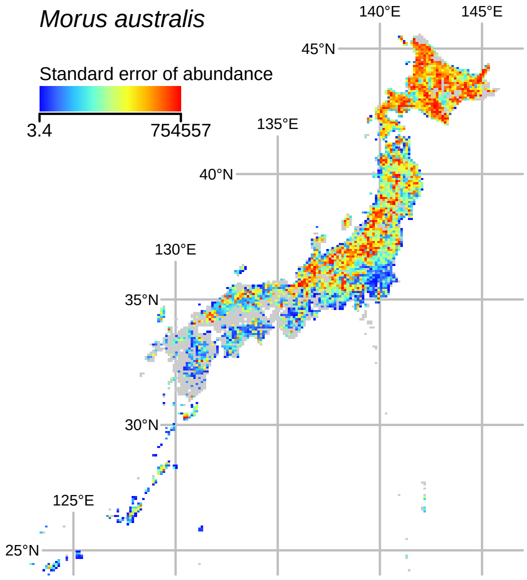
Morus australis
Standard error of abundance
3.4
754557
140°E
145°E
45°N
135°E
40°N
130°E
35°N
30°N
125°E
25°N

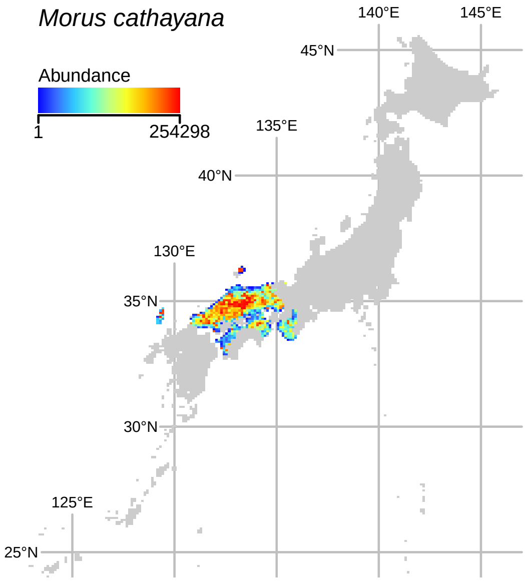
Morus cathayana
Abundance
1
254298
140°E
145°E
45°N
135°E
40°N
130°E
35°N
30°N
125°E
25°N

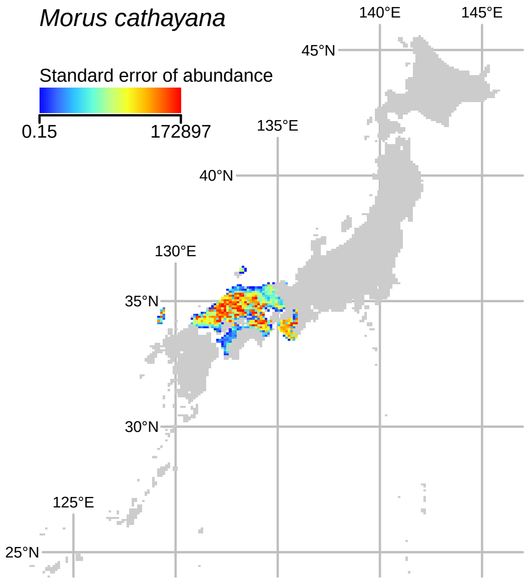
Morus cathayana
Standard error of abundance
0.15
172897
140°E
145°E
45°N
135°E
40°N
130°E
35°N
30°N
125°E
25°N

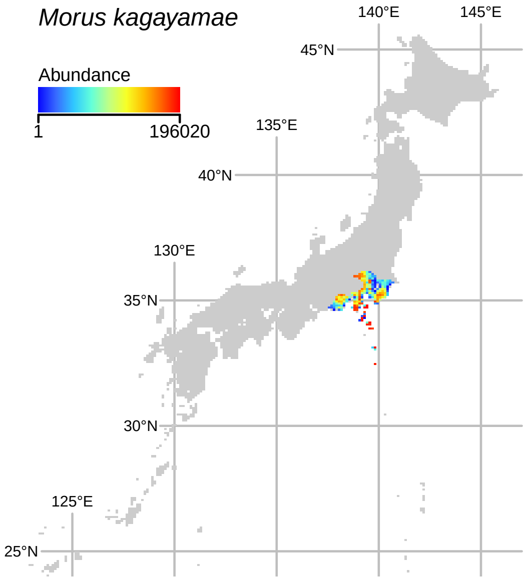
Morus kagayamae
Abundance
1
196020
140°E
145°E
45°N
135°E
40°N
130°E
35°N
30°N
125°E
25°N

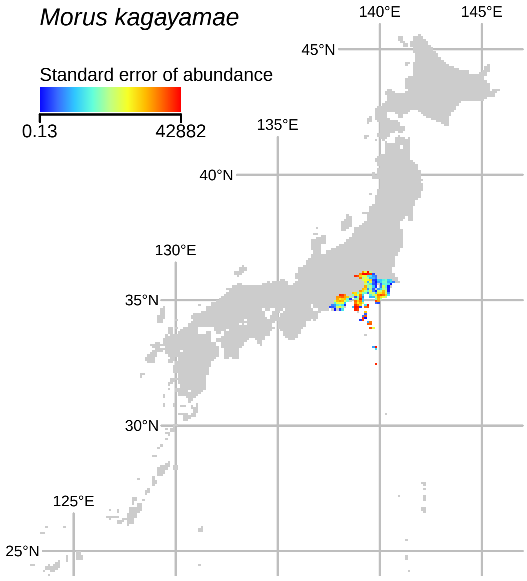
Morus kagayamae
Standard error of abundance
0.13
42882
140°E
145°E
45°N
135°E
40°N
130°E
35°N
30°N
125°E
25°N

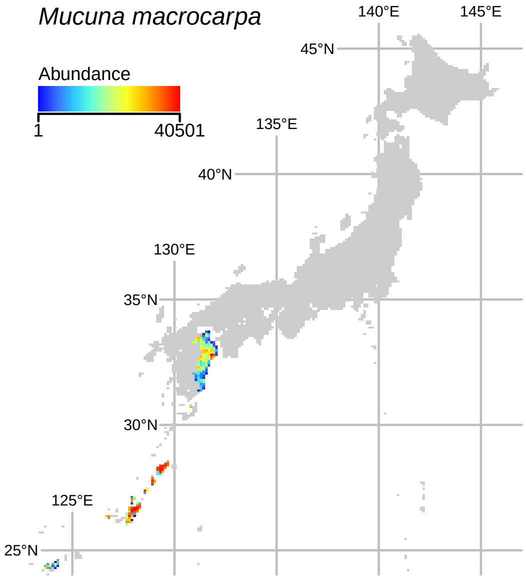
Mucuna macrocarpa
Abundance
1
40501
140°E
145°E
45°N
135°E
40°N
130°E
35°N
30°N
125°E
25°N

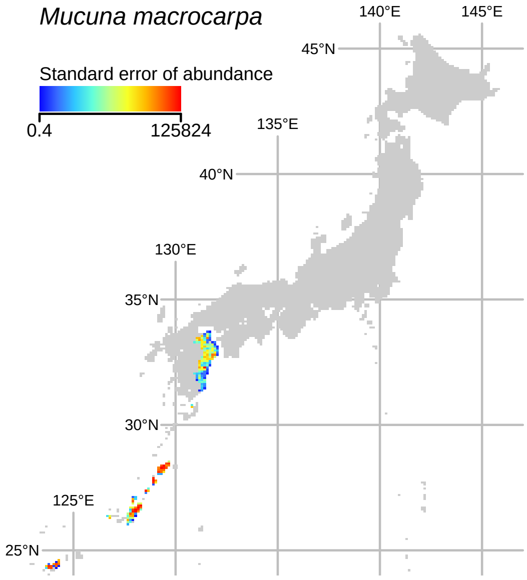
Mucuna macrocarpa
Standard error of abundance
0.4
125824
140°E
145°E
45°N
135°E
40°N
130°E
35°N
30°N
125°E
25°N

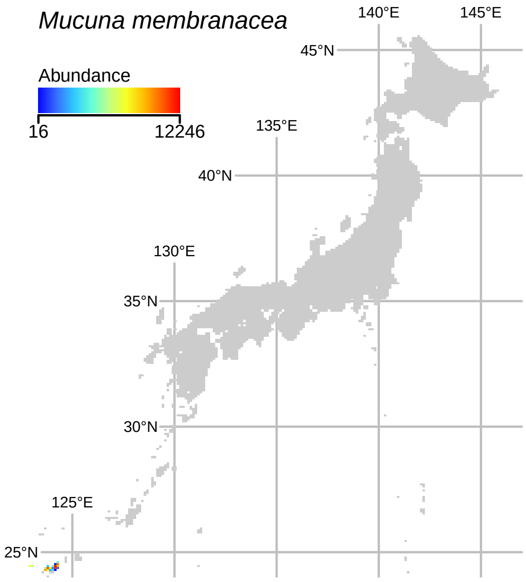
Mucuna membranacea
Abundance
16
12246
140°E
145°E
45°N
135°E
40°N
130°E
35°N
30°N
125°E
25°N

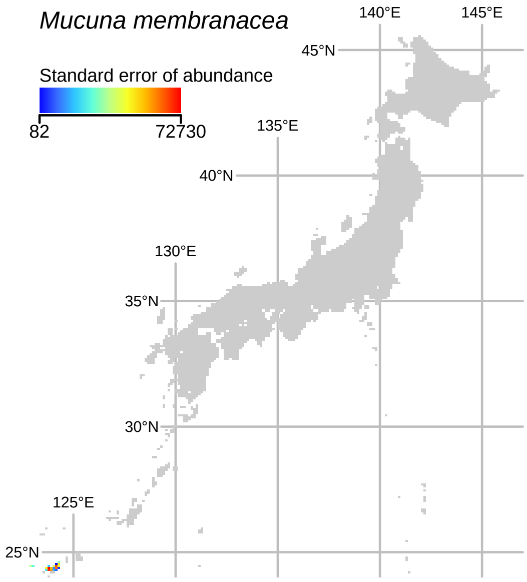
Mucuna membranacea
Standard error of abundance
82
72730
140°E
145°E
45°N
135°E
40°N
130°E
35°N
30°N
125°E
25°N

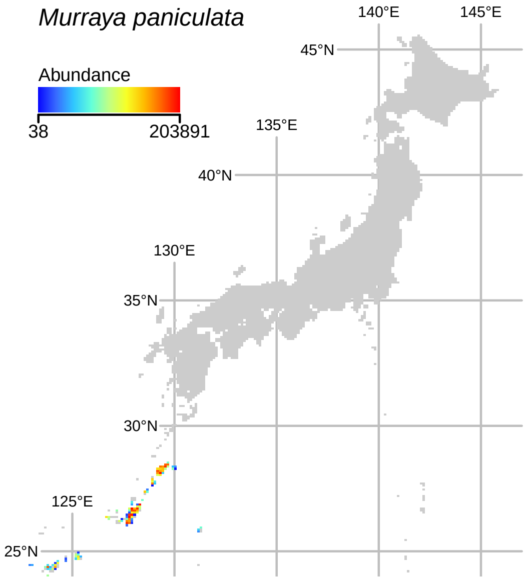
Murraya paniculata
Abundance
38
203891
140°E
145°E
45°N
135°E
40°N
130°E
35°N
30°N
125°E
25°N

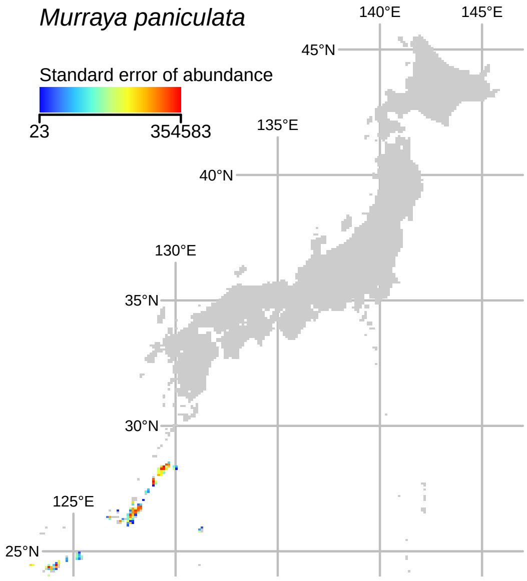
Murraya paniculata
Standard error of abundance
23
354583
140°E
145°E
45°N
135°E
40°N
130°E
35°N
30°N
125°E
25°N

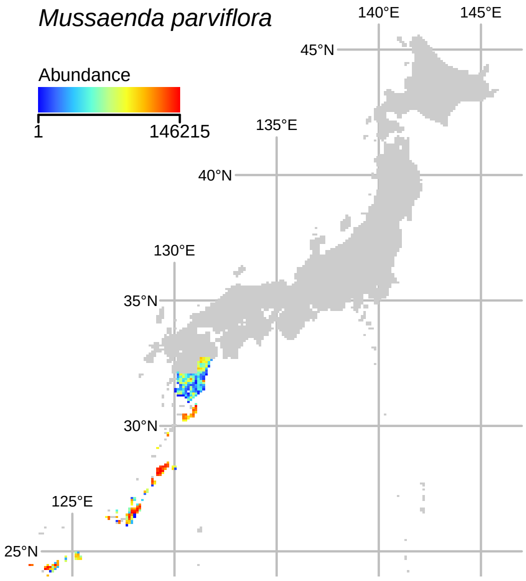
Mussaenda parviflora
Abundance
1
146215
140°E
145°E
45°N
135°E
40°N
130°E
35°N
30°N
125°E
25°N

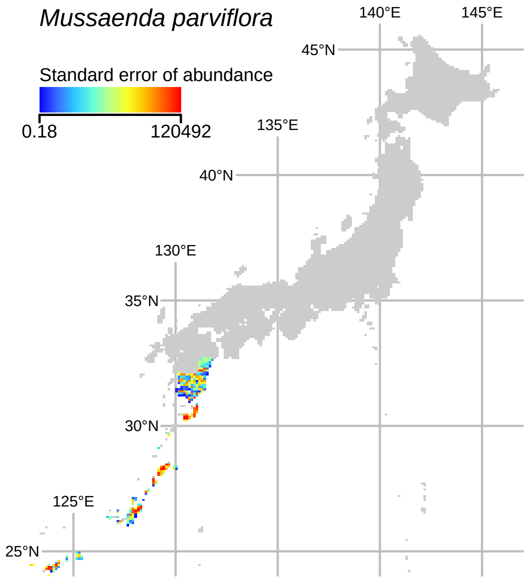
Mussaenda parviflora
Standard error of abundance
0.18
120492
140°E
145°E
45°N
135°E
40°N
130°E
35°N
30°N
125°E
25°N

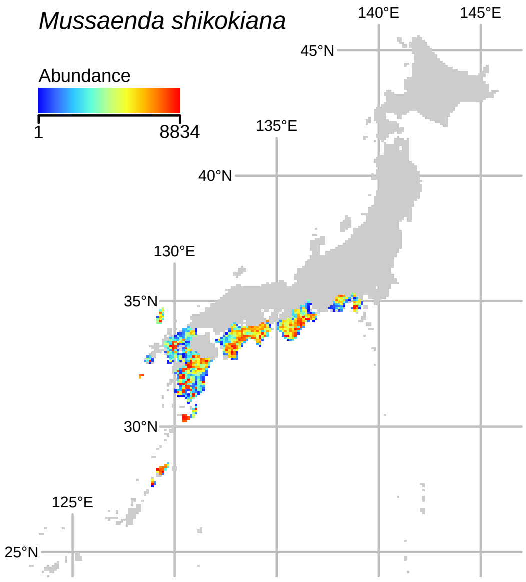
Mussaenda shikokiana
Abundance
1
8834
140°E
145°E
45°N
135°E
40°N
130°E
35°N
30°N
125°E
25°N

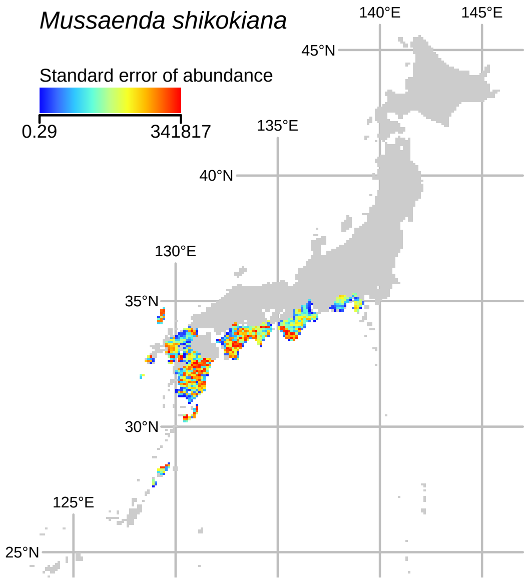
Mussaenda shikokiana
Standard error of abundance
0.29
341817
140°E
145°E
45°N
135°E
40°N
130°E
35°N
30°N
125°E
25°N

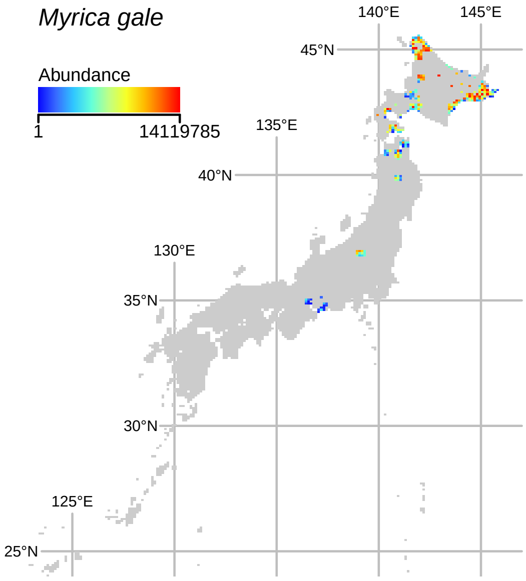
Myrica gale
Abundance
1
14119785
140°E
145°E
45°N
135°E
40°N
130°E
35°N
30°N
125°E
25°N

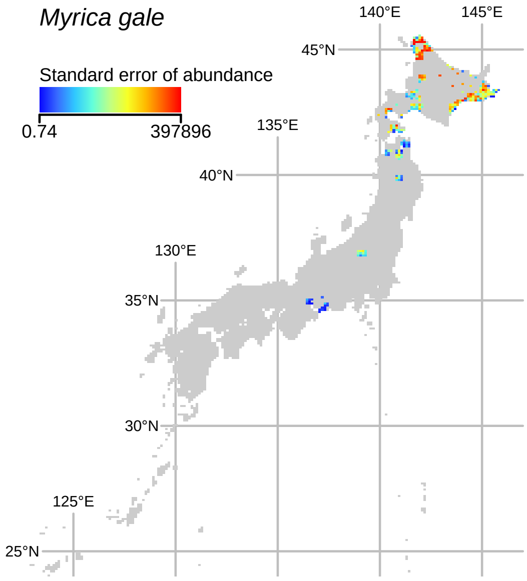
Myrica gale
Standard error of abundance
0.74
397896
140°E
145°E
45°N
135°E
40°N
130°E
35°N
30°N
125°E
25°N

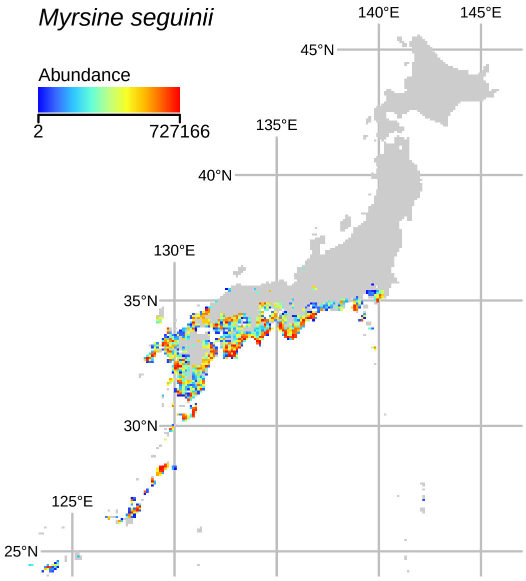
Myrsine seguinii
Abundance
2
727166
140°E
145°E
45°N
135°E
40°N
130°E
35°N
30°N
125°E
25°N

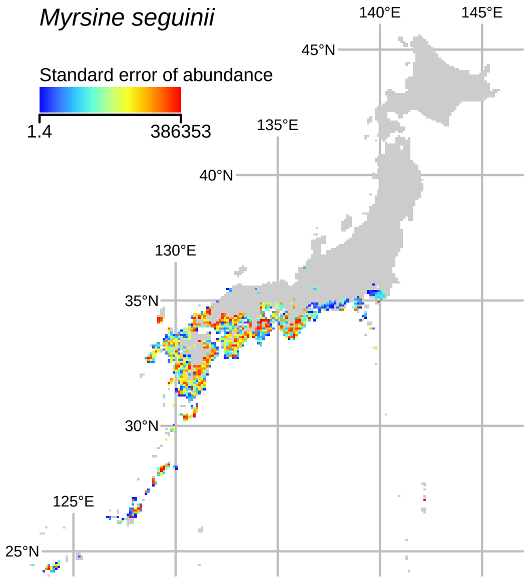
Myrsine seguinii
Standard error of abundance
1.4
386353
140°E
145°E
45°N
135°E
40°N
130°E
35°N
30°N
125°E
25°N

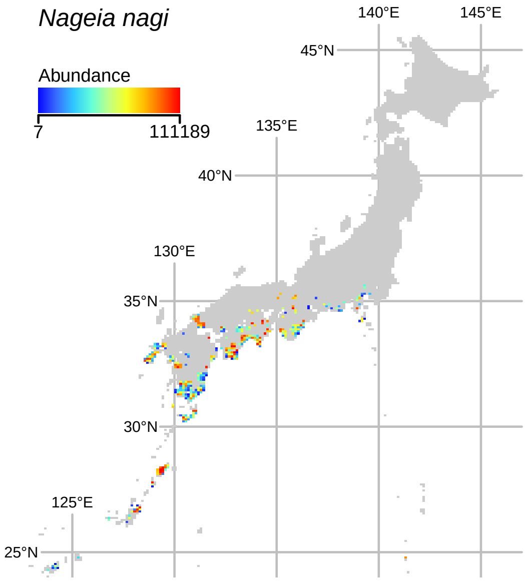
Nageia nagi
Abundance
7
111189
140°E
145°E
45°N
135°E
40°N
130°E
35°N
30°N
125°E
25°N

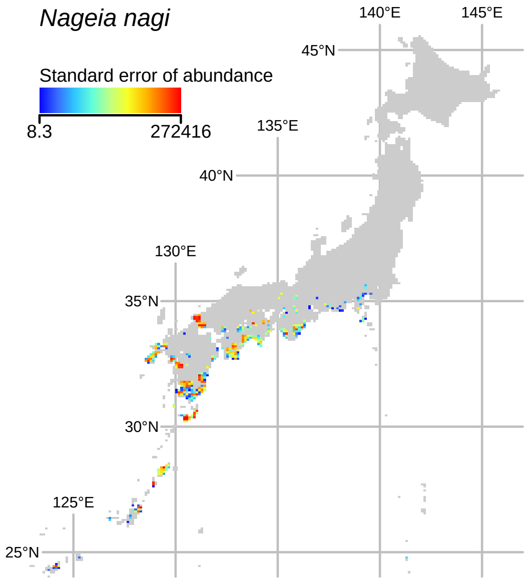
Nageia nagi
Standard error of abundance
8.3
272416
140°E
145°E
45°N
135°E
40°N
130°E
35°N
30°N
125°E
25°N

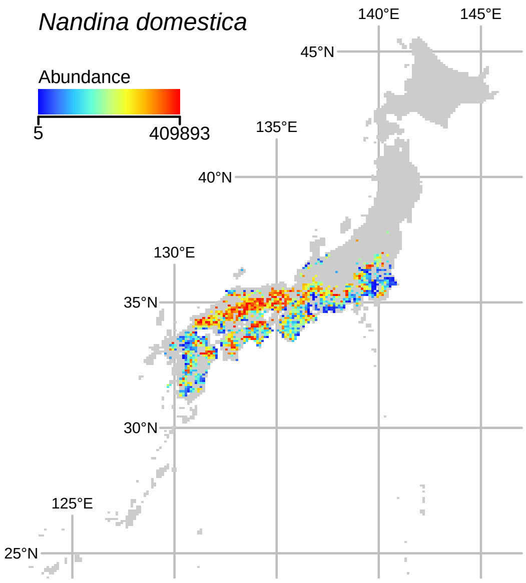
Nandina domestica
Abundance
5
409893
140°E
145°E
45°N
135°E
40°N
130°E
35°N
30°N
125°E
25°N

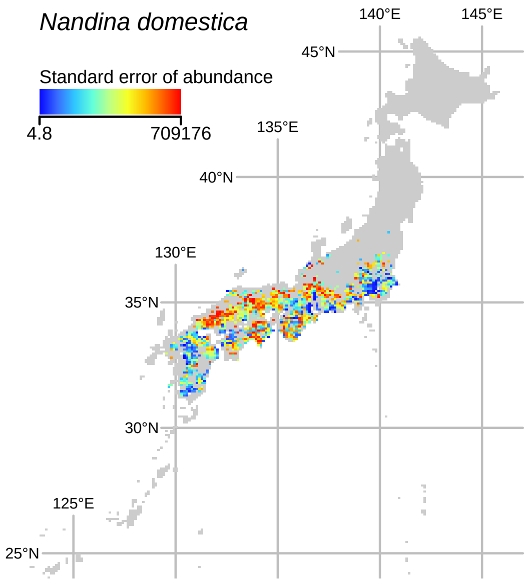
Nandina domestica
Standard error of abundance
4.8
709176
140°E
145°E
45°N
135°E
40°N
130°E
35°N
30°N
125°E
25°N

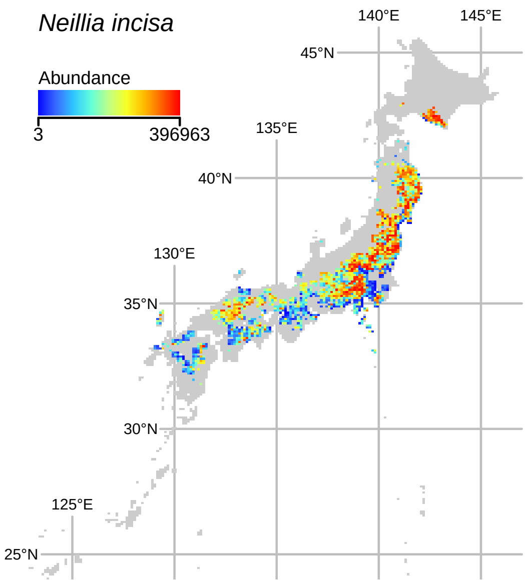
Neillia incisa
Abundance
3
396963
140°E
145°E
45°N
135°E
40°N
130°E
35°N
30°N
125°E
25°N

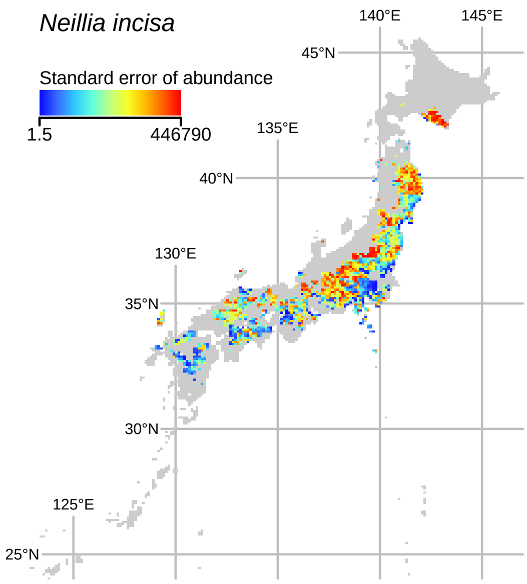
Neillia incisa
Standard error of abundance
1.5
446790
140°E
145°E
45°N
135°E
40°N
130°E
35°N
30°N
125°E
25°N

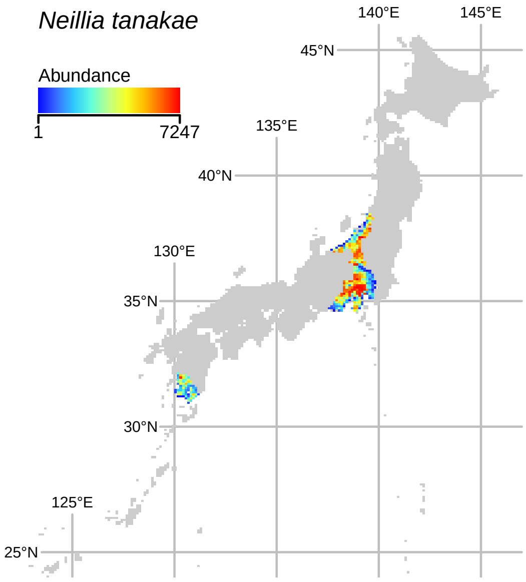
Neillia tanakae
Abundance
1
7247
140°E
145°E
45°N
135°E
40°N
130°E
35°N
30°N
125°E
25°N

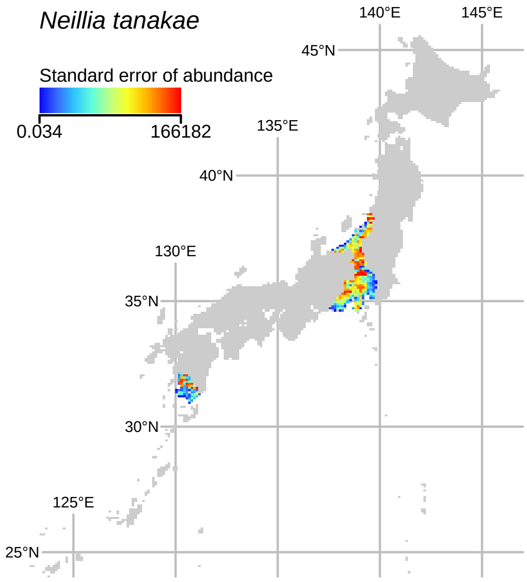
Neillia tanakae
Standard error of abundance
0.034
166182
140°E
145°E
45°N
135°E
40°N
130°E
35°N
30°N
125°E
25°N

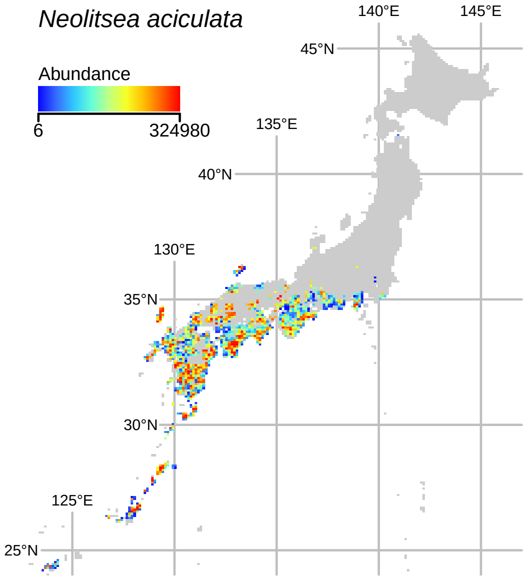
Neolitsea aciculata
Abundance
6
324980
140°E
145°E
45°N
135°E
40°N
130°E
35°N
30°N
125°E
25°N

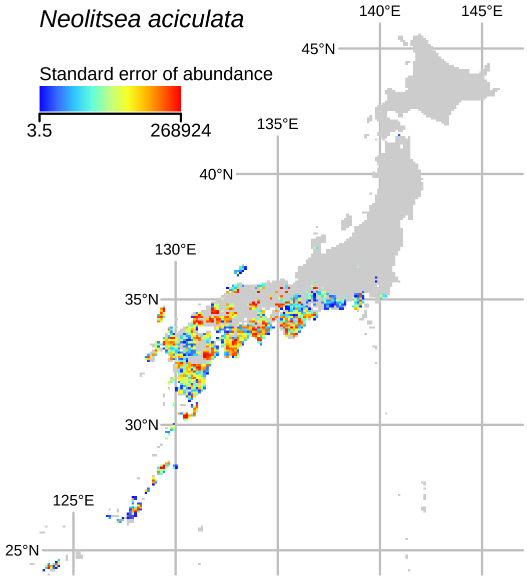
Neolitsea aciculata
Standard error of abundance
3.5
268924
140°E
145°E
45°N
135°E
40°N
130°E
35°N
30°N
125°E
25°N

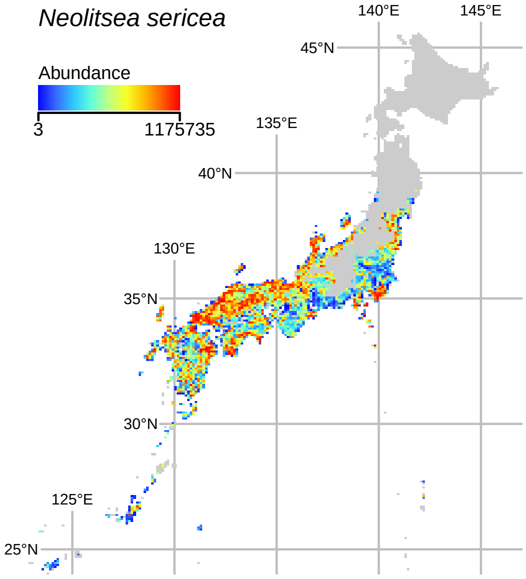
Neolitsea sericea
Abundance
3
1175735
140°E
145°E
45°N
135°E
40°N
130°E
35°N
30°N
125°E
25°N

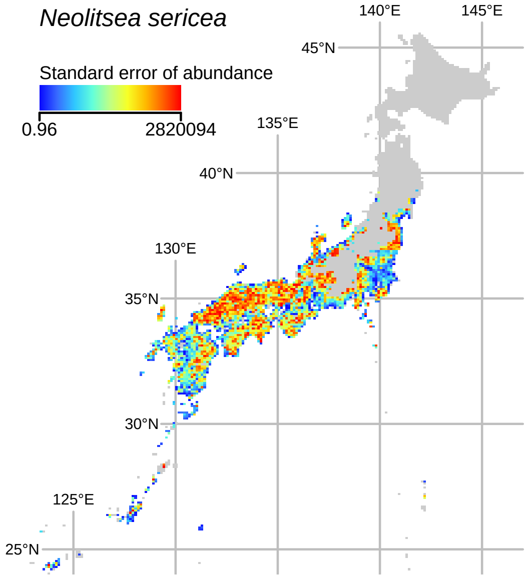
Neolitsea sericea
Standard error of abundance
0.96
2820094
140°E
145°E
45°N
135°E
40°N
130°E
35°N
30°N
125°E
25°N

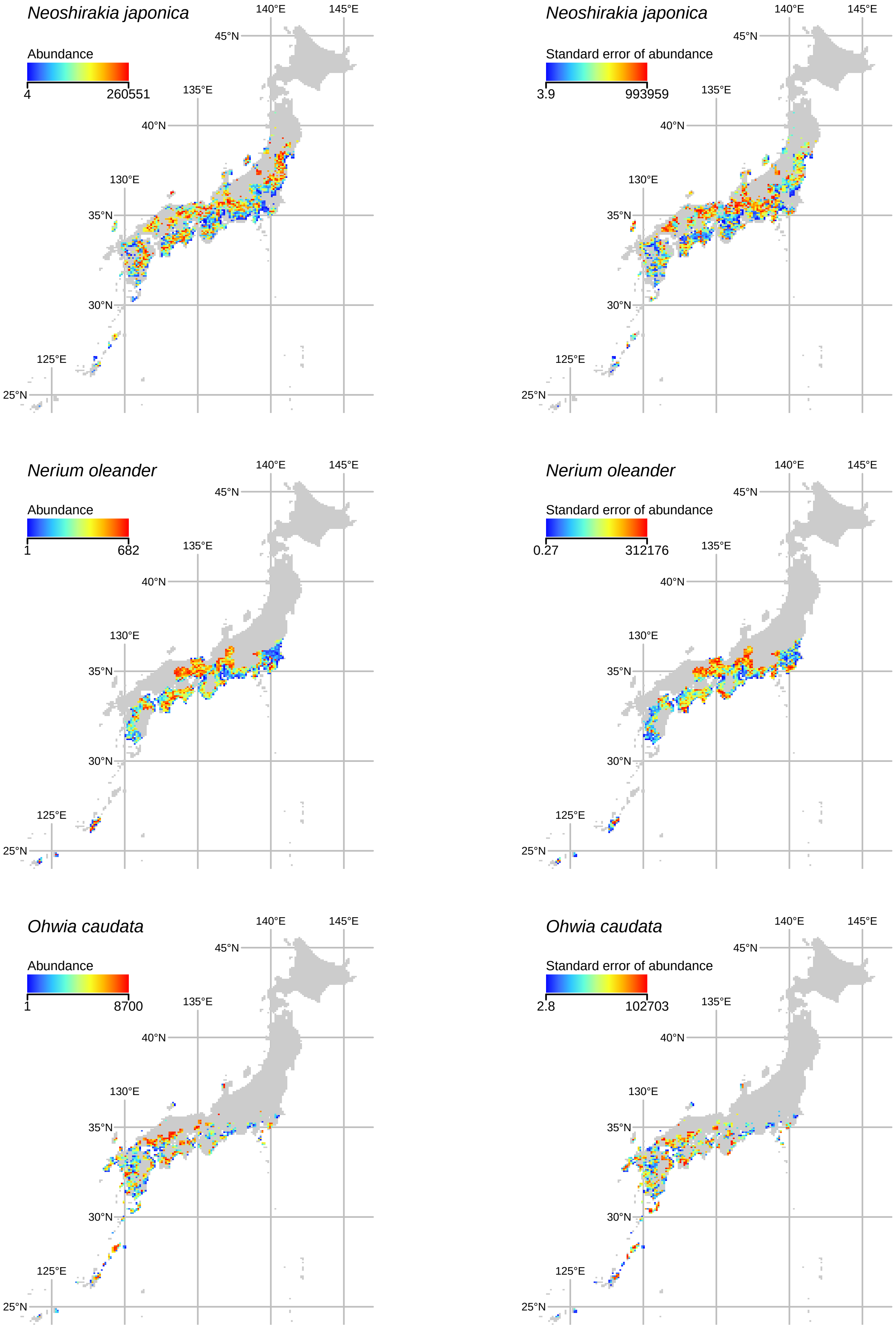

Neoshirakia japonica
Abundance
4
260551
Neoshirakia japonica
Standard error of abundance
3.9
993959
Nerium oleander
Abundance
1
682
Nerium oleander
Standard error of abundance
0.27
312176
Ohwia caudata
Abundance
1
8700
Ohwia caudata
Standard error of abundance
2.8
102703
125°E
130°E
135°E
140°E
145°E
25°N
30°N
35°N
40°N
45°N

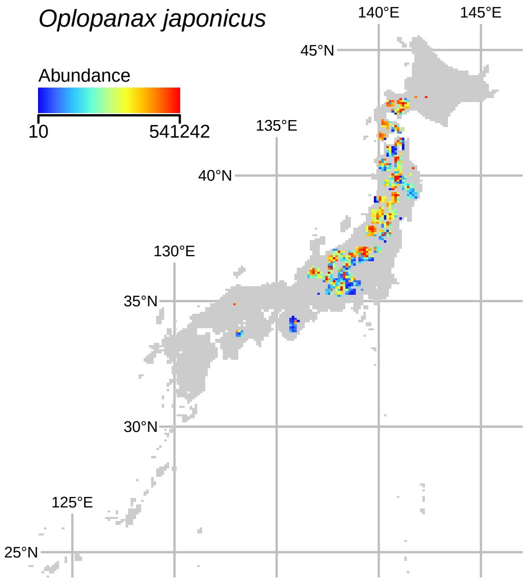
Oplopanax japonicus
Abundance
10
541242
140°E
145°E
45°N
135°E
40°N
130°E
35°N
30°N
125°E
25°N

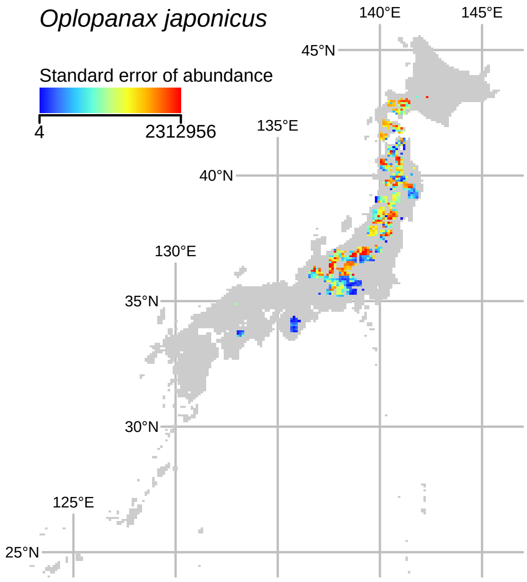
Oplopanax japonicus
Standard error of abundance
4
2312956
140°E
145°E
45°N
135°E
40°N
130°E
35°N
30°N
125°E
25°N

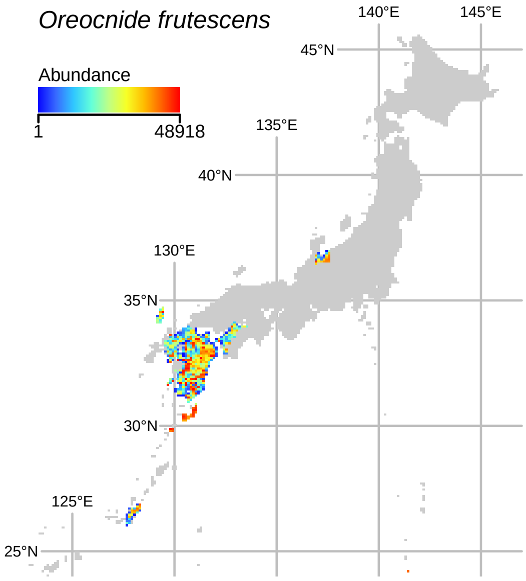
Oreocnide frutescens
Abundance
1
48918
140°E
145°E
45°N
135°E
40°N
130°E
35°N
30°N
125°E
25°N

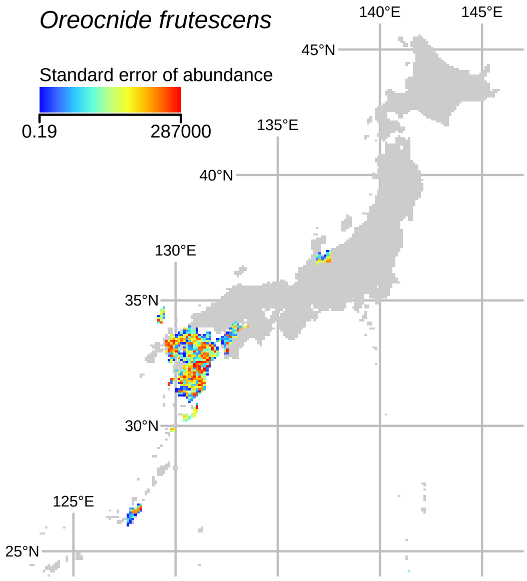
Oreocnide frutescens
Standard error of abundance
0.19
287000
140°E
145°E
45°N
135°E
40°N
130°E
35°N
30°N
125°E
25°N

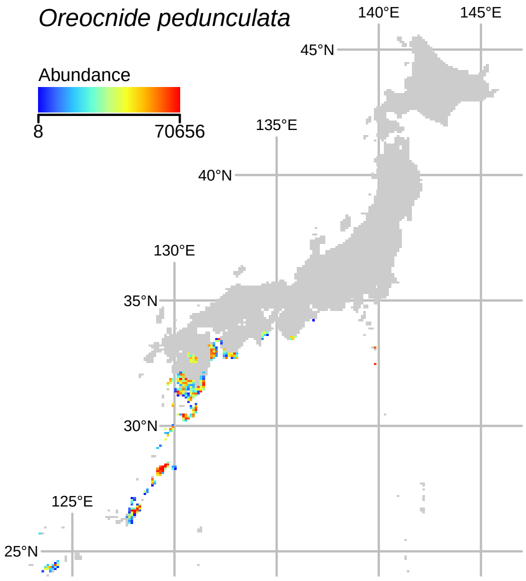
Oreocnide pedunculata
Abundance
8
70656
140°E
145°E
45°N
135°E
40°N
130°E
35°N
30°N
125°E
25°N

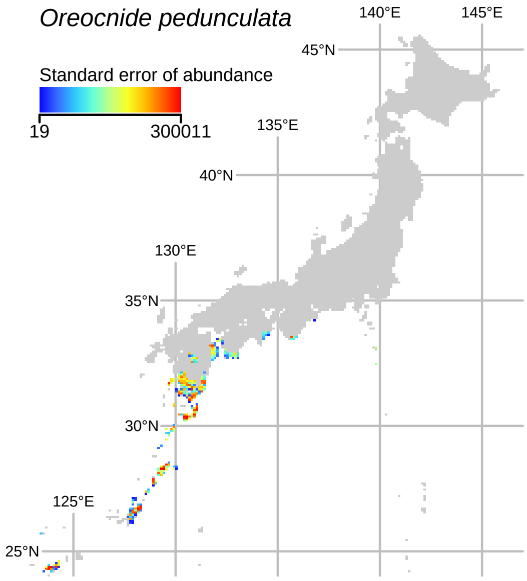
Oreocnide pedunculata
Standard error of abundance
19
300011
140°E
145°E
45°N
135°E
40°N
130°E
35°N
30°N
125°E
25°N

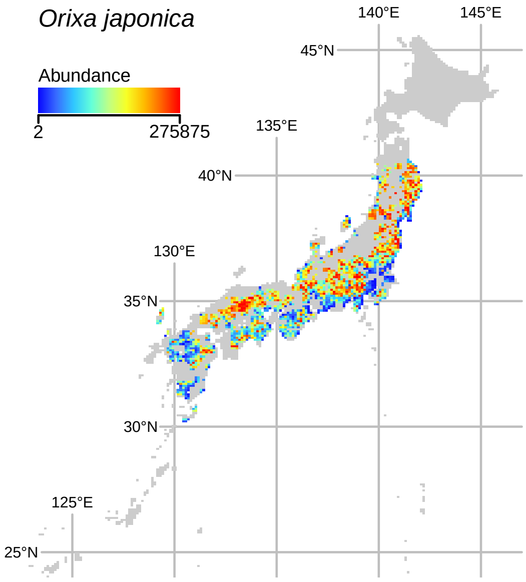
Orixa japonica
Abundance
2
275875
140°E
145°E
45°N
135°E
40°N
130°E
35°N
30°N
125°E
25°N

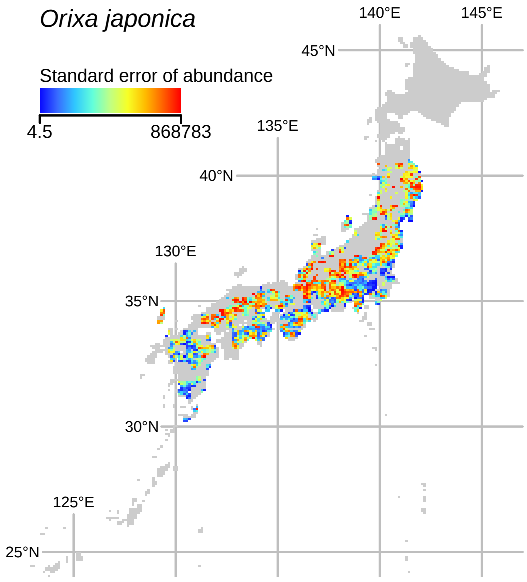
Orixa japonica
Standard error of abundance
4.5
868783
140°E
145°E
45°N
135°E
40°N
130°E
35°N
30°N
125°E
25°N

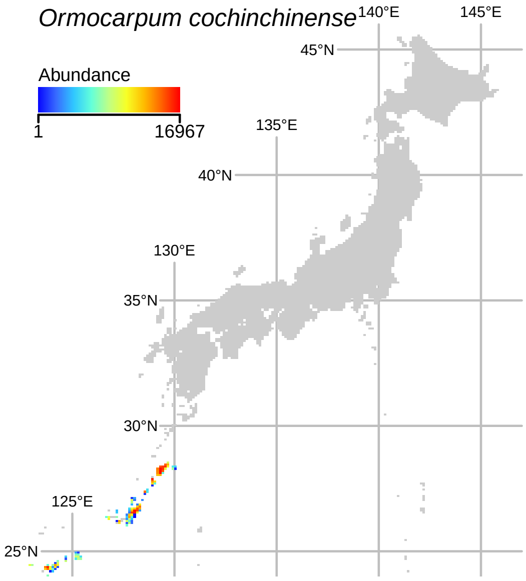
Ormocarpum cochinchinense
Abundance
1
16967
140°E
145°E
45°N
135°E
40°N
130°E
35°N
30°N
125°E
25°N

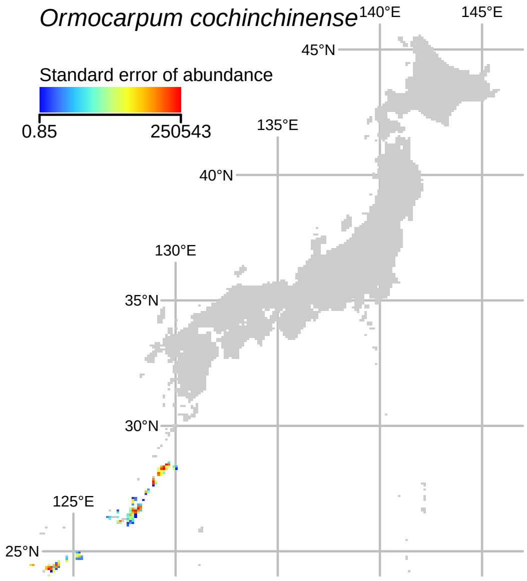
Ormocarpum cochinchinense
Standard error of abundance
0.85
250543
140°E
145°E
45°N
135°E
40°N
130°E
35°N
30°N
125°E
25°N

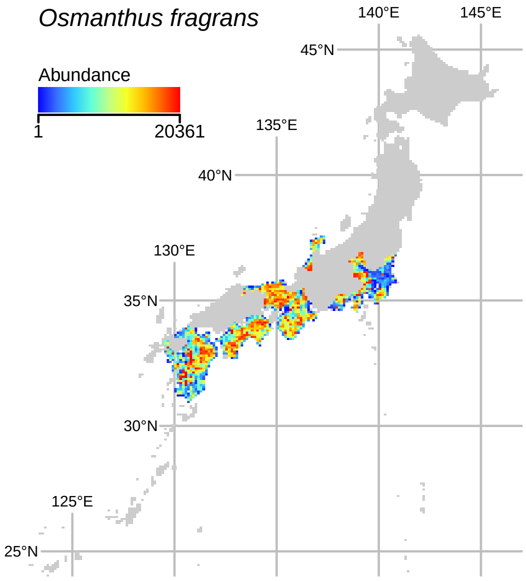
Osmanthus fragrans
Abundance
1
20361
140°E
145°E
45°N
135°E
40°N
130°E
35°N
30°N
125°E
25°N

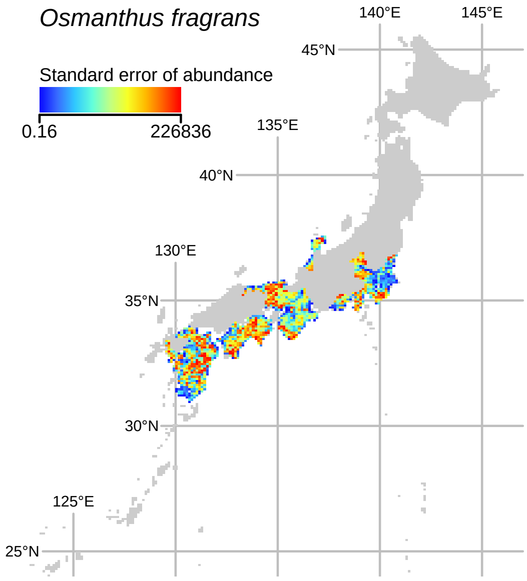
Osmanthus fragrans
Standard error of abundance
0.16
226836
140°E
145°E
45°N
135°E
40°N
130°E
35°N
30°N
125°E
25°N

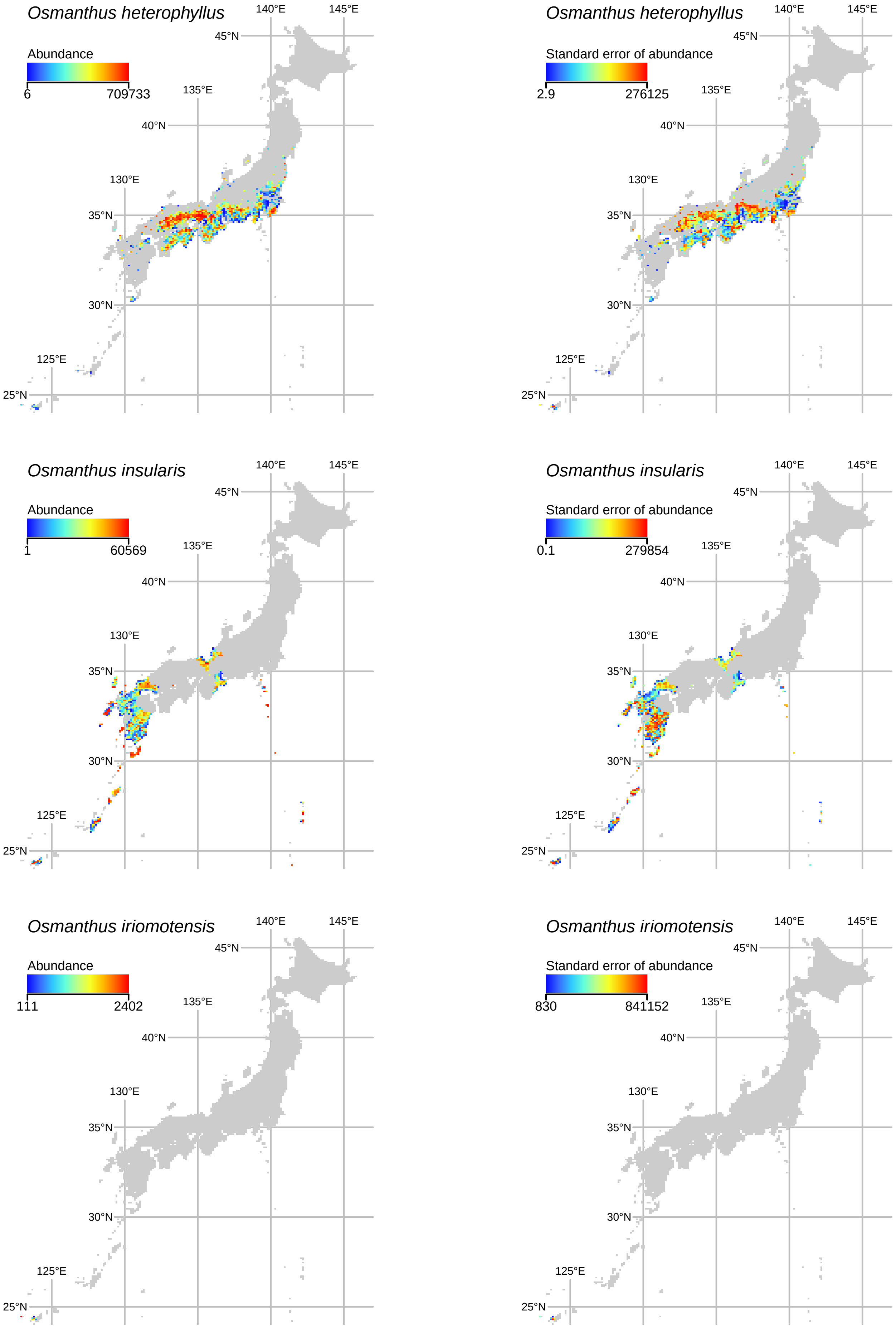
Osmanthus heterophyllus
Abundance
6
709733
Osmanthus heterophyllus
Standard error of abundance
2.9
276125
Osmanthus insularis
Abundance
1
60569
Osmanthus insularis
Standard error of abundance
0.1
279854
Osmanthus iriomotensis
Abundance
111
2402
Osmanthus iriomotensis
Standard error of abundance
830
841152
125°E
130°E
135°E
140°E
145°E
25°N
30°N
35°N
40°N
45°N

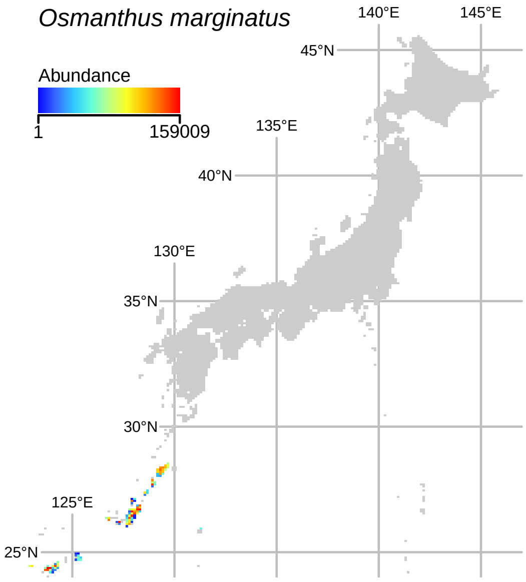
Osmanthus marginatus
Abundance
1
159009
140°E
145°E
45°N
135°E
40°N
130°E
35°N
30°N
125°E
25°N

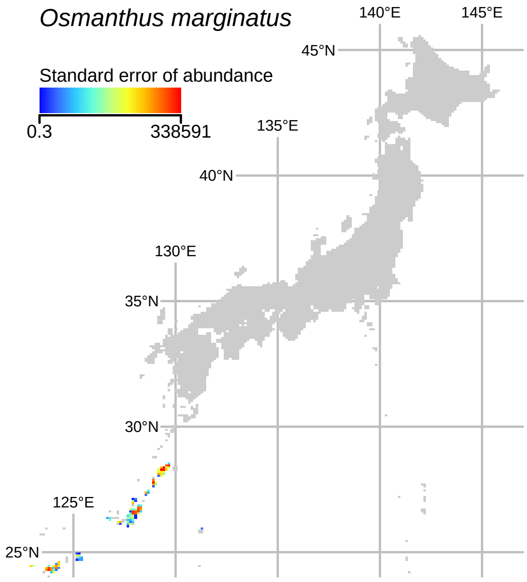
Osmanthus marginatus
Standard error of abundance
0.3
338591
140°E
145°E
45°N
135°E
40°N
130°E
35°N
30°N
125°E
25°N

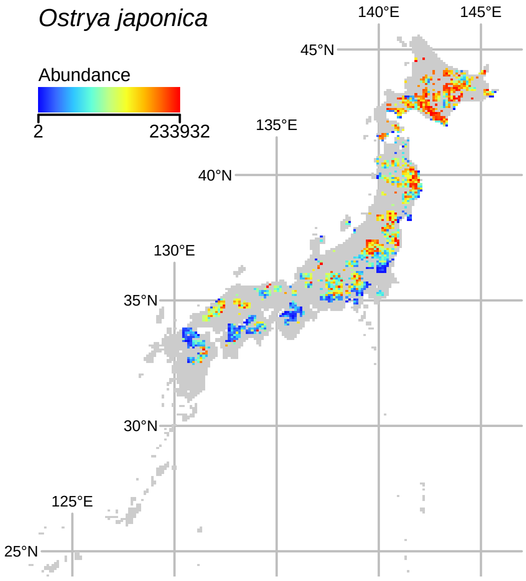
Ostrya japonica
Abundance
2
233932
140°E
145°E
45°N
135°E
40°N
130°E
35°N
30°N
125°E
25°N

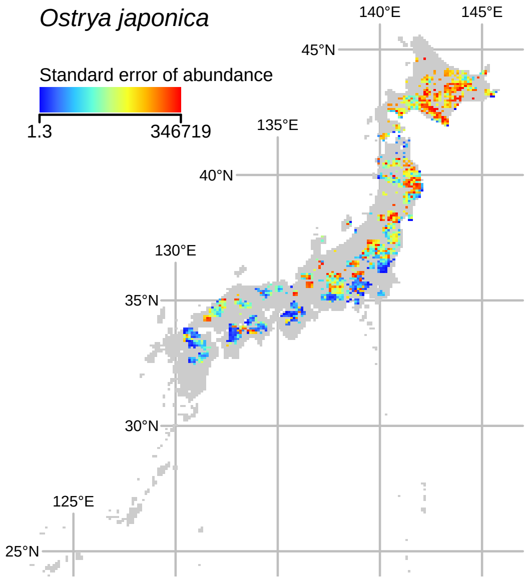
Ostrya japonica
Standard error of abundance
1.3
346719
140°E
145°E
45°N
135°E
40°N
130°E
35°N
30°N
125°E
25°N

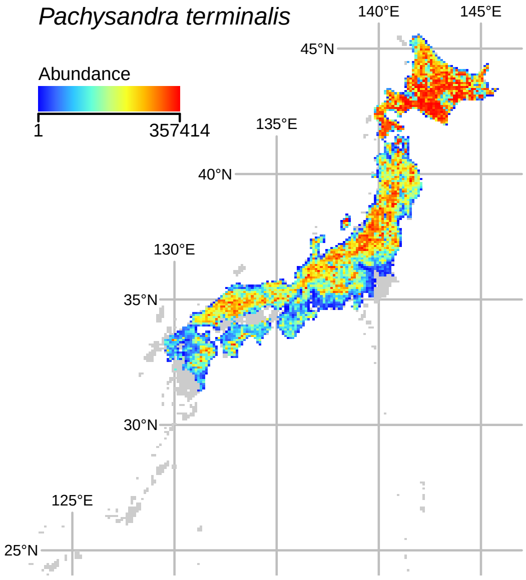
Pachysandra terminalis
Abundance
1
357414
140°E
145°E
45°N
135°E
40°N
130°E
35°N
30°N
125°E
25°N

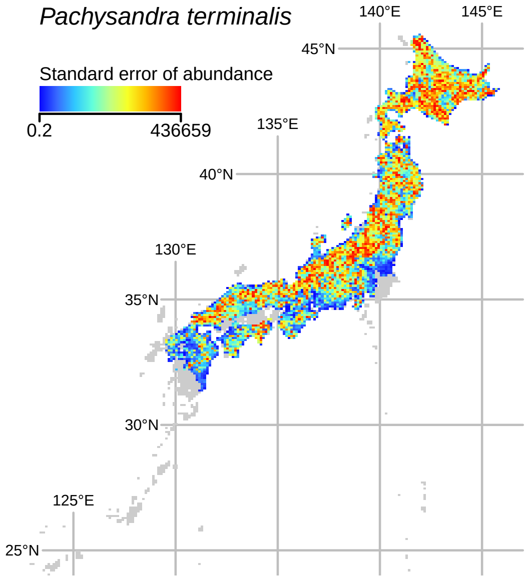
Pachysandra terminalis
Standard error of abundance
0.2
436659
140°E
145°E
45°N
135°E
40°N
130°E
35°N
30°N
125°E
25°N

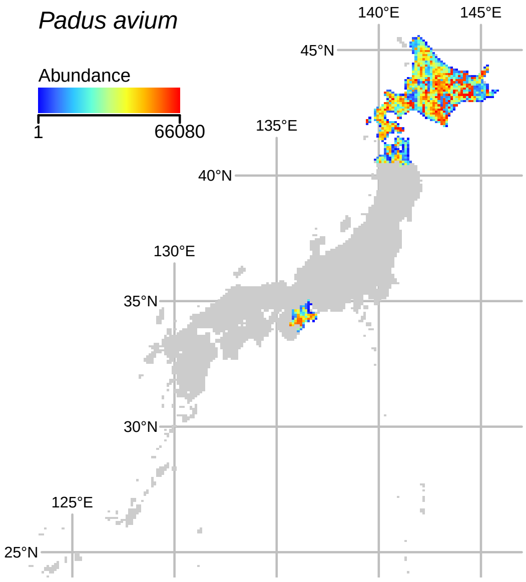
Padus avium
Abundance
1
66080
140°E
145°E
45°N
135°E
40°N
130°E
35°N
30°N
125°E
25°N

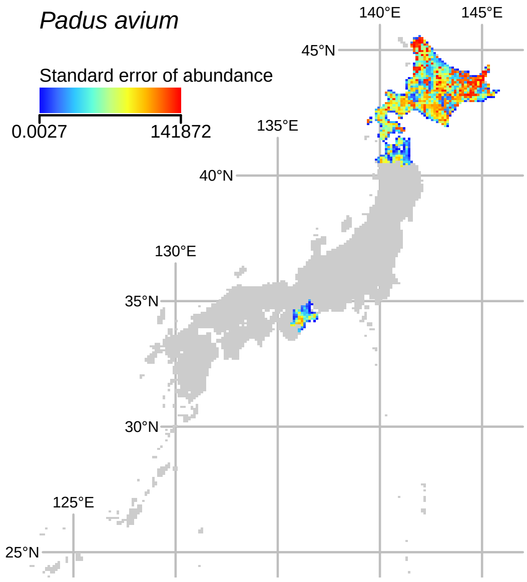
Padus avium
Standard error of abundance
0.0027
141872
140°E
145°E
45°N
135°E
40°N
130°E
35°N
30°N
125°E
25°N

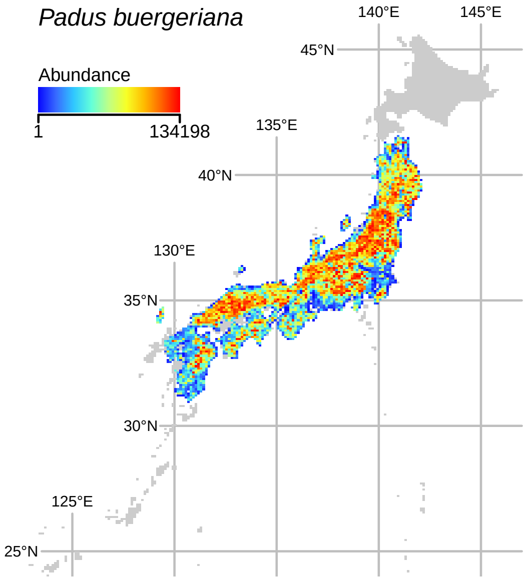
Padus buergeriana
Abundance
1
134198
140°E
145°E
45°N
135°E
40°N
130°E
35°N
30°N
125°E
25°N

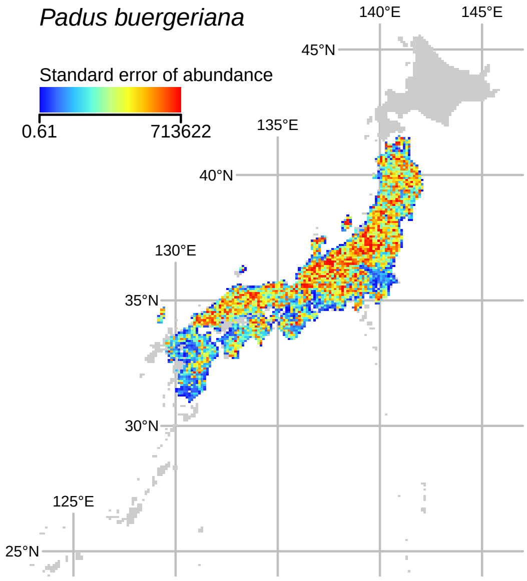
Padus buergeriana
Standard error of abundance
0.61
713622
140°E
145°E
45°N
135°E
40°N
130°E
35°N
30°N
125°E
25°N

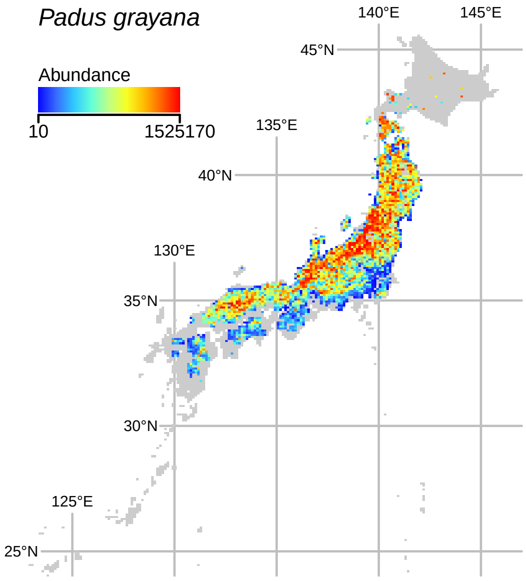
Padus grayana
Abundance
10
1525170
140°E
145°E
45°N
135°E
40°N
130°E
35°N
30°N
125°E
25°N

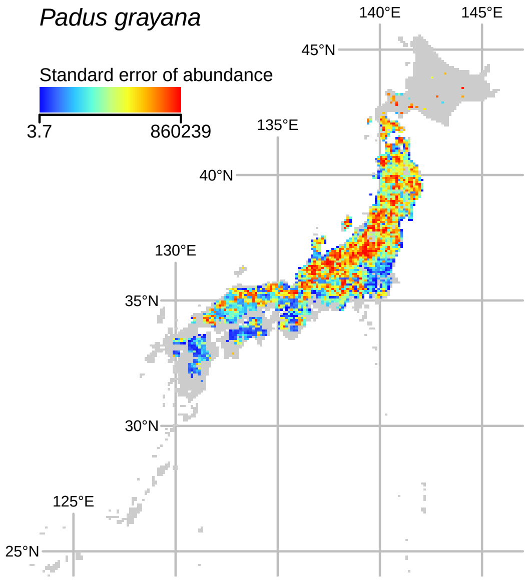
Padus grayana
Standard error of abundance
3.7
860239
140°E
145°E
45°N
135°E
40°N
130°E
35°N
30°N
125°E
25°N

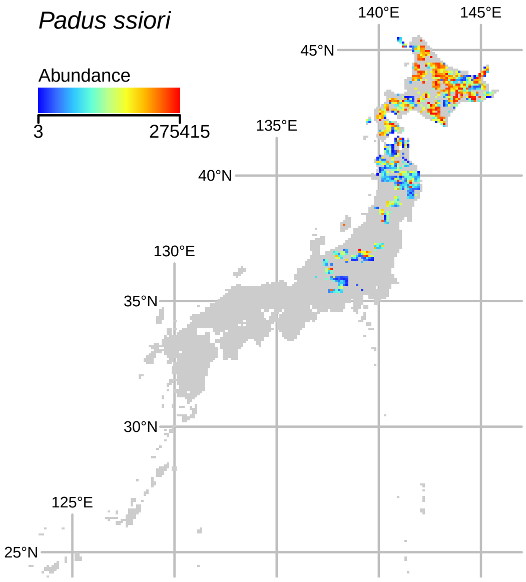
Padus ssiori
Abundance
3
275415
140°E
145°E
135°E
130°E
125°E
45°N
40°N
35°N
30°N
25°N

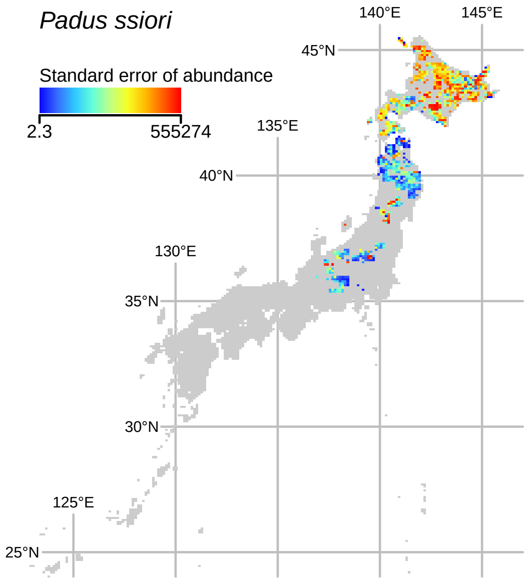
Padus ssiori
Standard error of abundance
2.3
555274
140°E
145°E
135°E
130°E
125°E
45°N
40°N
35°N
30°N
25°N

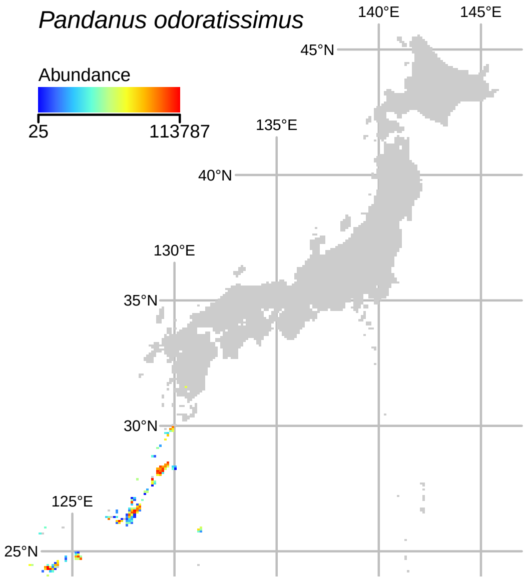
Pandanus odoratissimus
Abundance
25
113787
140°E
145°E
135°E
130°E
125°E
45°N
40°N
35°N
30°N
25°N

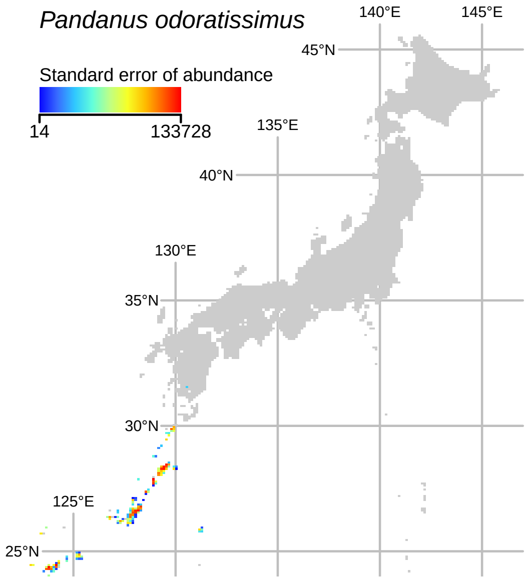
Pandanus odoratissimus
Standard error of abundance
14
133728
140°E
145°E
135°E
130°E
125°E
45°N
40°N
35°N
30°N
25°N

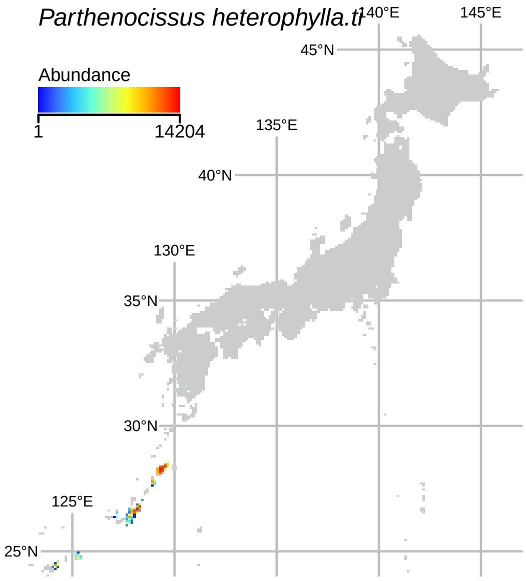
Parthenocissus heterophylla.tI
Abundance
1
14204
140°E
145°E
135°E
130°E
125°E
45°N
40°N
35°N
30°N
25°N

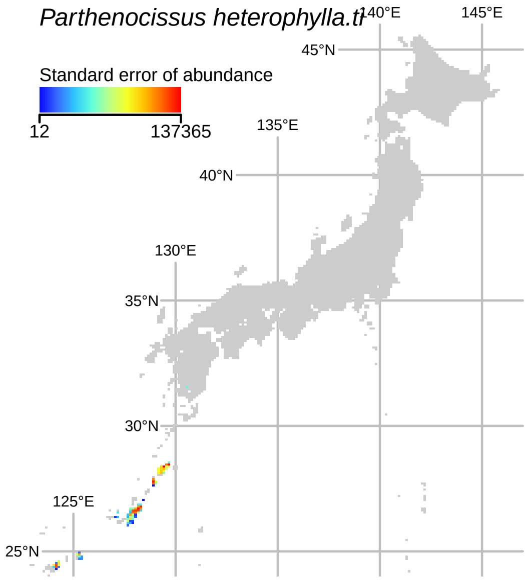
Parthenocissus heterophylla.tI
Standard error of abundance
12
137365
140°E
145°E
135°E
130°E
125°E
45°N
40°N
35°N
30°N
25°N

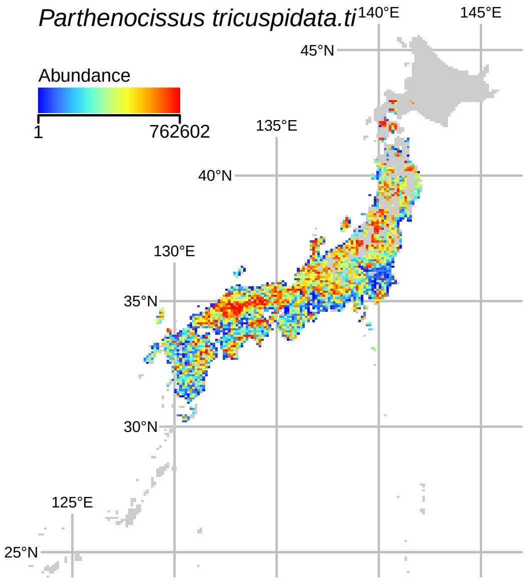
Parthenocissus tricuspidata.ti
Abundance
1
762602
140°E
145°E
135°E
130°E
125°E
45°N
40°N
35°N
30°N
25°N

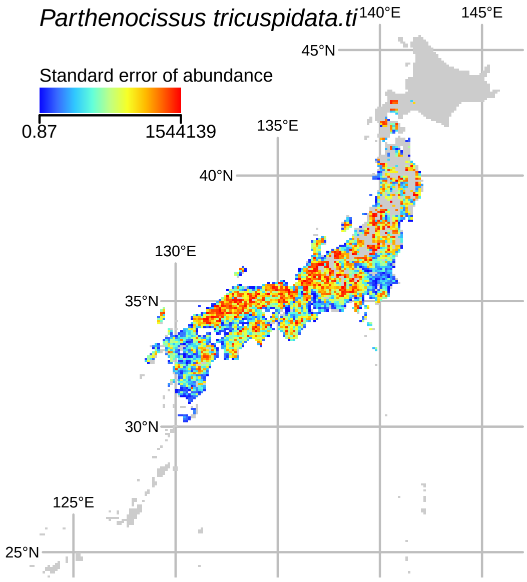
Parthenocissus tricuspidata.ti
Standard error of abundance
0.87
1544139
140°E
145°E
135°E
130°E
125°E
45°N
40°N
35°N
30°N
25°N

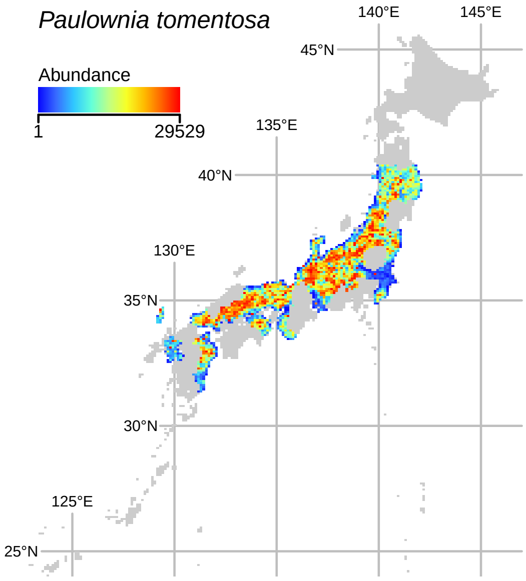
Paulownia tomentosa
Abundance
1
29529
140°E
145°E
135°E
130°E
125°E
45°N
40°N
35°N
30°N
25°N

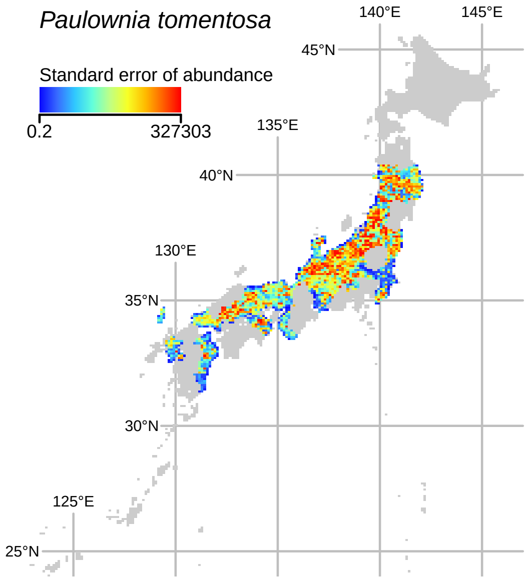
Paulownia tomentosa
Standard error of abundance
0.2
327303
140°E
145°E
135°E
130°E
125°E
45°N
40°N
35°N
30°N
25°N

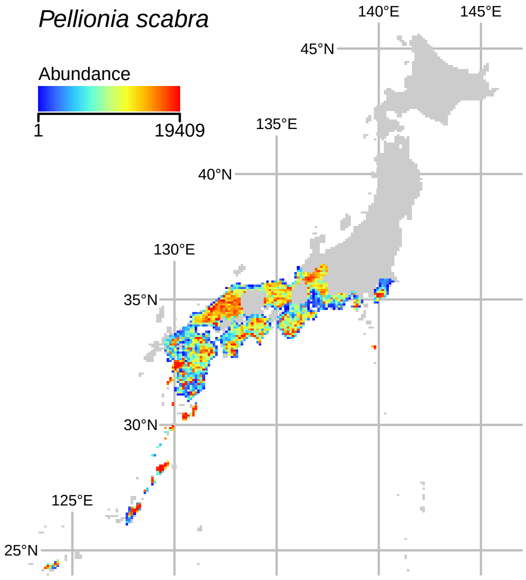
Pellionia scabra
Abundance
1
19409
140°E
145°E
135°E
130°E
125°E
45°N
40°N
35°N
30°N
25°N

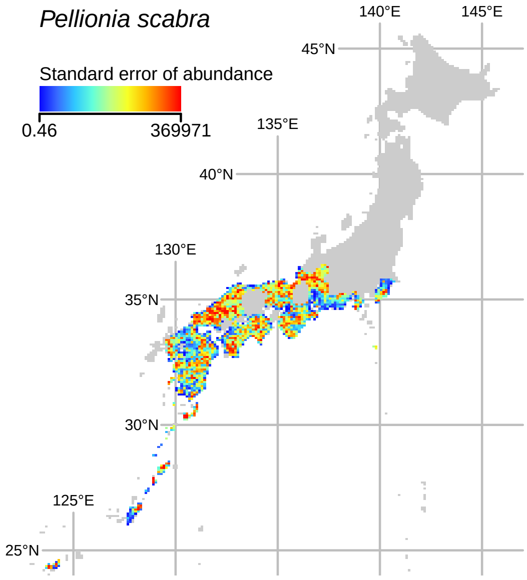
Pellionia scabra
Standard error of abundance
0.46
369971
140°E
145°E
135°E
130°E
125°E
45°N
40°N
35°N
30°N
25°N

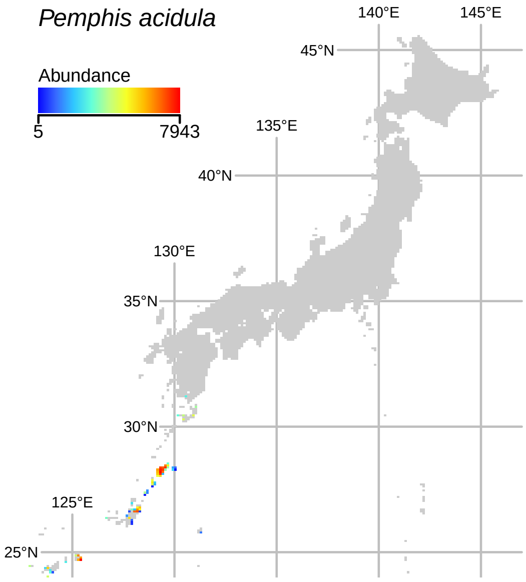
Pemphis acidula
Abundance
5
7943
140°E
145°E
45°N
135°E
40°N
130°E
35°N
30°N
125°E
25°N

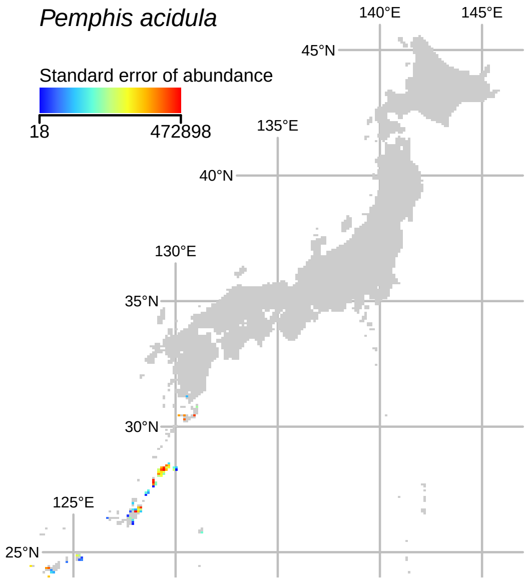
Pemphis acidula
Standard error of abundance
18
472898
140°E
145°E
45°N
135°E
40°N
130°E
35°N
30°N
125°E
25°N

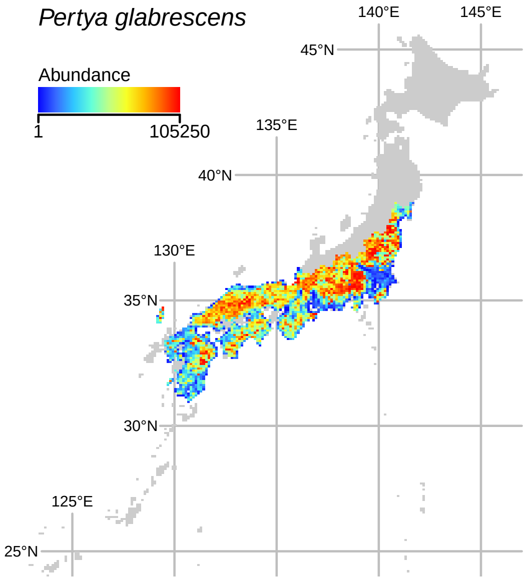
Pertya glabrescens
Abundance
1
105250
140°E
145°E
45°N
135°E
40°N
130°E
35°N
30°N
125°E
25°N

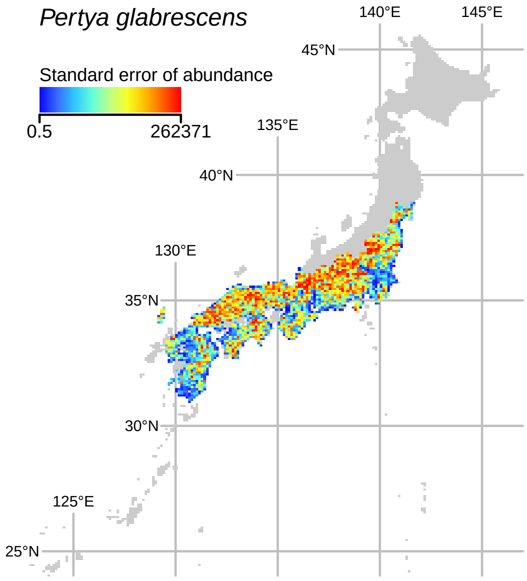
Pertya glabrescens
Standard error of abundance
0.5
262371
140°E
145°E
45°N
135°E
40°N
130°E
35°N
30°N
125°E
25°N

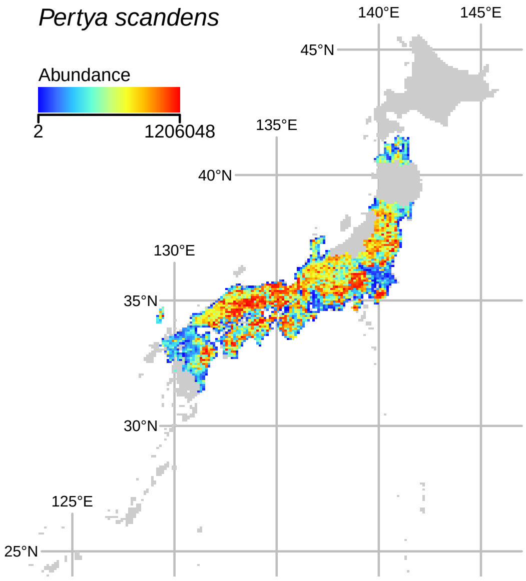
Pertya scandens
Abundance
2
1206048
140°E
145°E
45°N
135°E
40°N
130°E
35°N
30°N
125°E
25°N

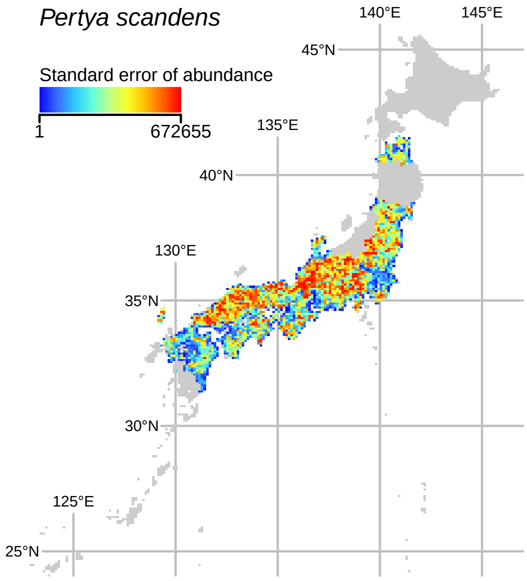
Pertya scandens
Standard error of abundance
1
672655
140°E
145°E
45°N
135°E
40°N
130°E
35°N
30°N
125°E
25°N

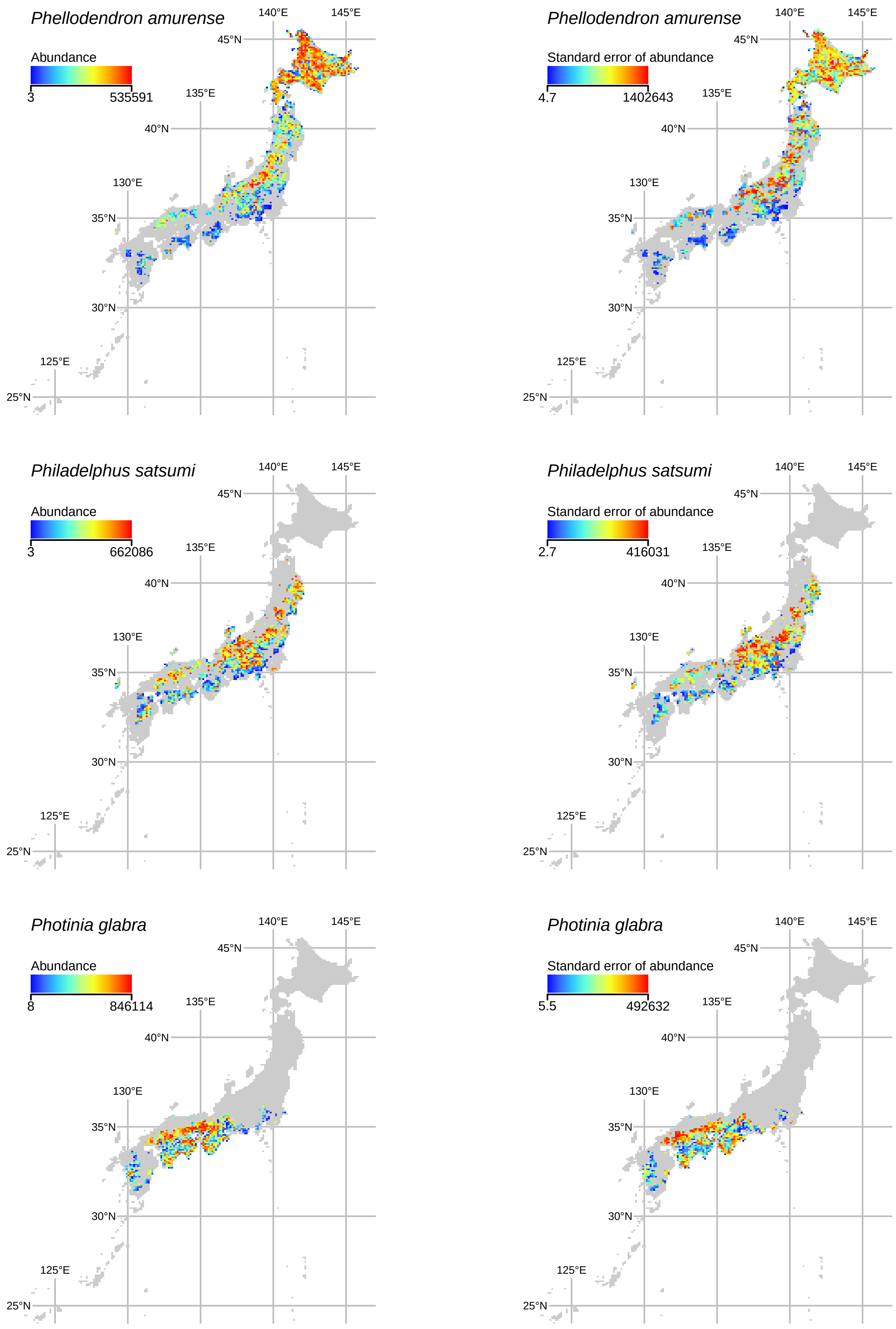
Phellodendron amurense
Abundance
3
535591
Phellodendron amurense
Standard error of abundance
4.7
1402643
Philadelphus satsumi
Abundance
3
662086
Philadelphus satsumi
Standard error of abundance
2.7
416031
Photinia glabra
Abundance
8
846114
Photinia glabra
Standard error of abundance
5.5
492632
140°E
145°E
135°E
130°E
125°E
45°N
40°N
35°N
30°N
25°N

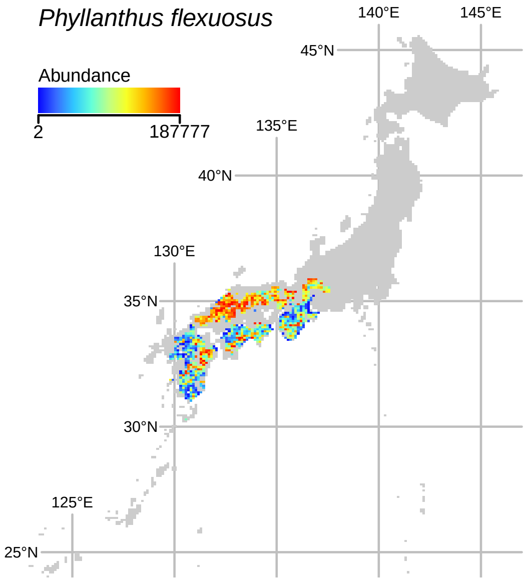
Phyllanthus flexuosus
Abundance
2
187777
140°E
145°E
45°N
135°E
40°N
130°E
35°N
30°N
125°E
25°N

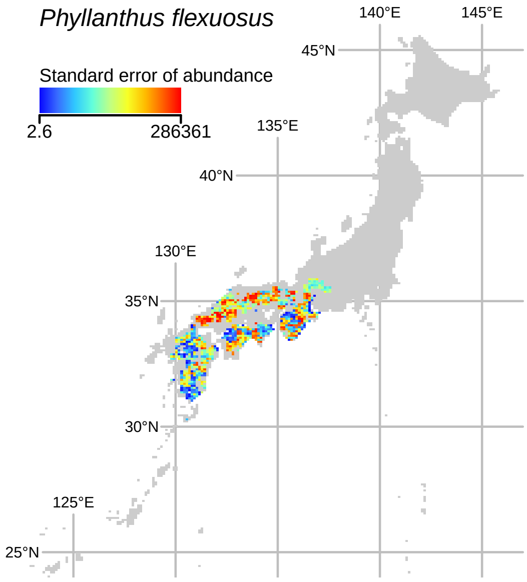
Phyllanthus flexuosus
Standard error of abundance
2.6
286361
140°E
145°E
45°N
135°E
40°N
130°E
35°N
30°N
125°E
25°N

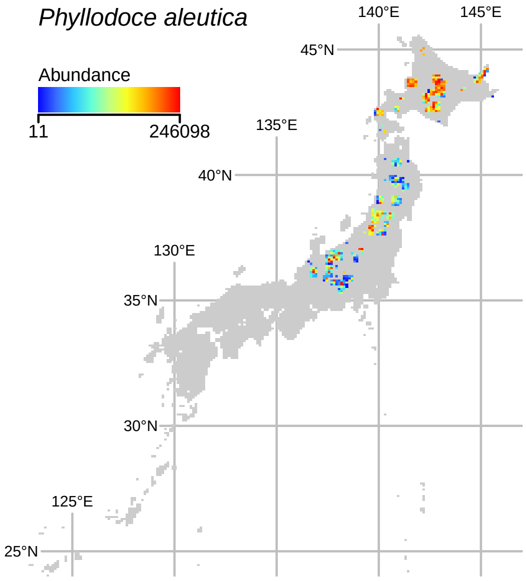
Phyllodoce aleutica
Abundance
11
246098
140°E
145°E
45°N
135°E
40°N
130°E
35°N
30°N
125°E
25°N

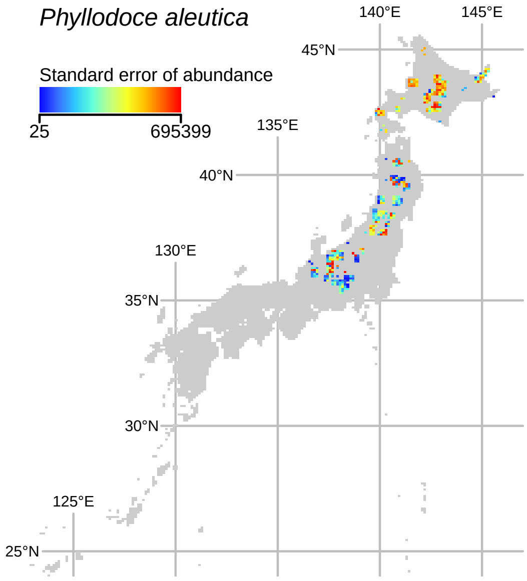
Phyllodoce aleutica
Standard error of abundance
25
695399
140°E
145°E
45°N
135°E
40°N
130°E
35°N
30°N
125°E
25°N

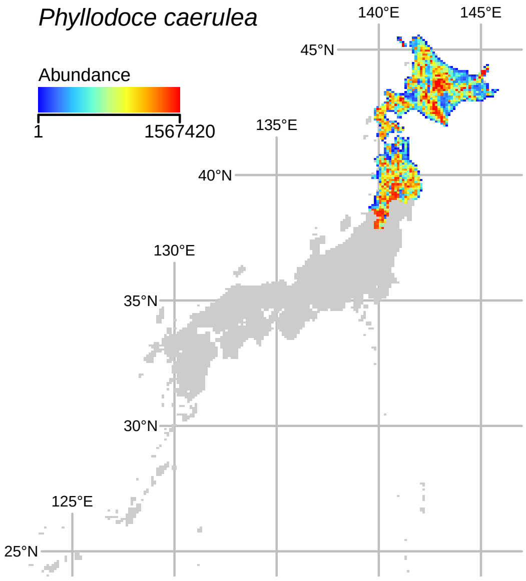
Phyllodoce caerulea
Abundance
1
1567420
140°E
145°E
45°N
135°E
40°N
130°E
35°N
30°N
125°E
25°N

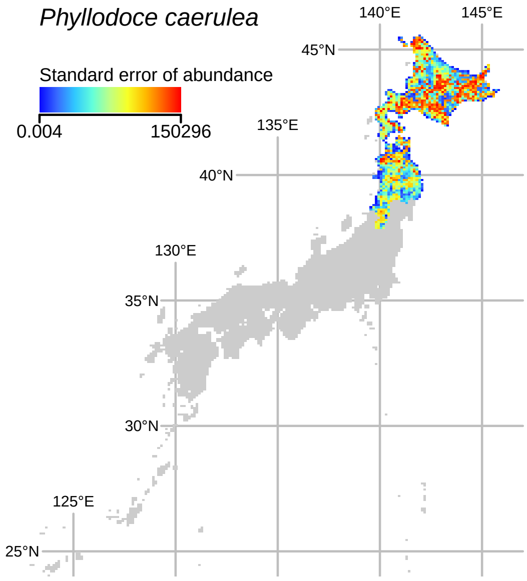
Phyllodoce caerulea
Standard error of abundance
0.004
150296
140°E
145°E
45°N
135°E
40°N
130°E
35°N
30°N
125°E
25°N

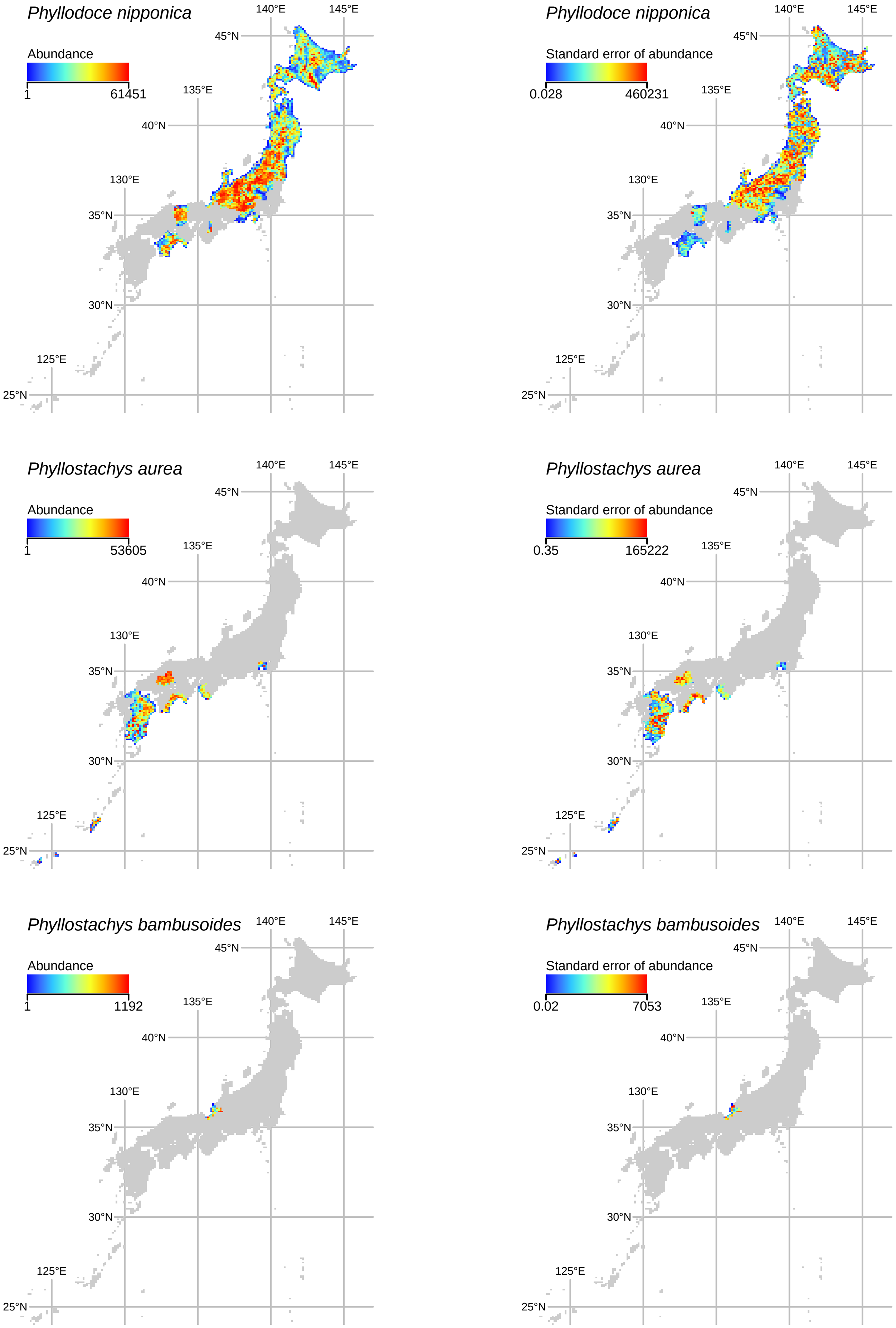
Phyllodoce nipponica
Abundance
1
61451
Phyllodoce nipponica
Standard error of abundance
0.028
460231
Phyllostachys aurea
Abundance
1
53605
Phyllostachys aurea
Standard error of abundance
0.35
165222
Phyllostachys bambusoides
Abundance
1
1192
Phyllostachys bambusoides
Standard error of abundance
0.02
7053
125°E
130°E
135°E
140°E
145°E
25°N
30°N
35°N
40°N
45°N

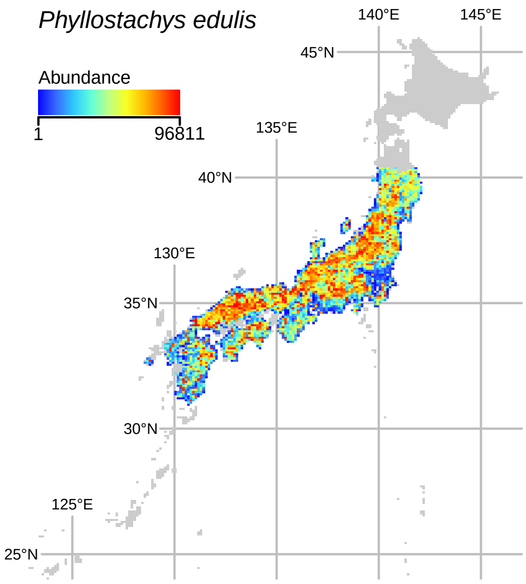
Phyllostachys edulis
Abundance
1
96811
140°E
145°E
45°N
135°E
40°N
130°E
35°N
30°N
125°E
25°N

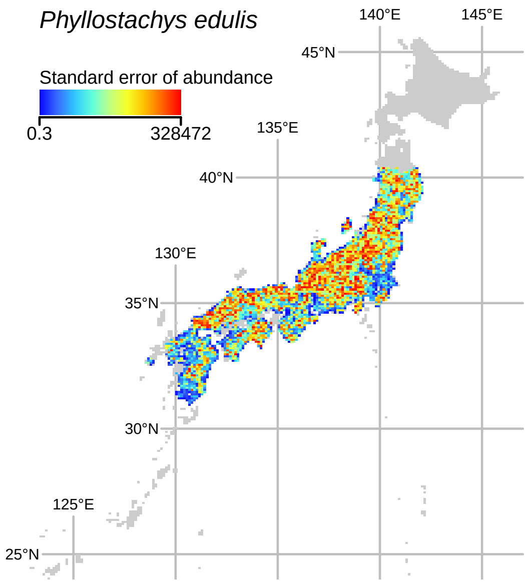
Phyllostachys edulis
Standard error of abundance
0.3
328472
140°E
145°E
45°N
135°E
40°N
130°E
35°N
30°N
125°E
25°N

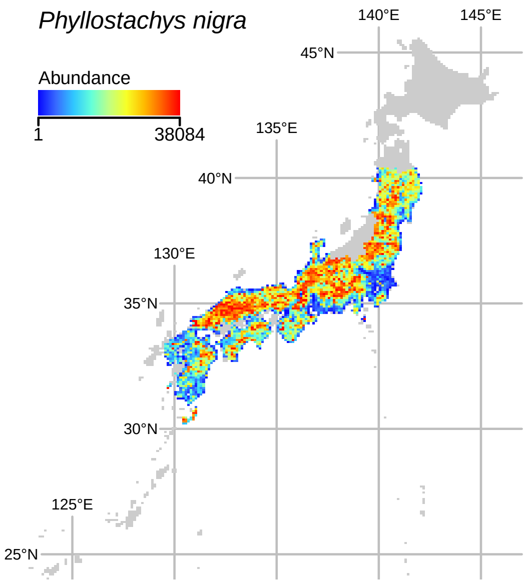
Phyllostachys nigra
Abundance
1
38084
140°E
145°E
45°N
135°E
40°N
130°E
35°N
30°N
125°E
25°N

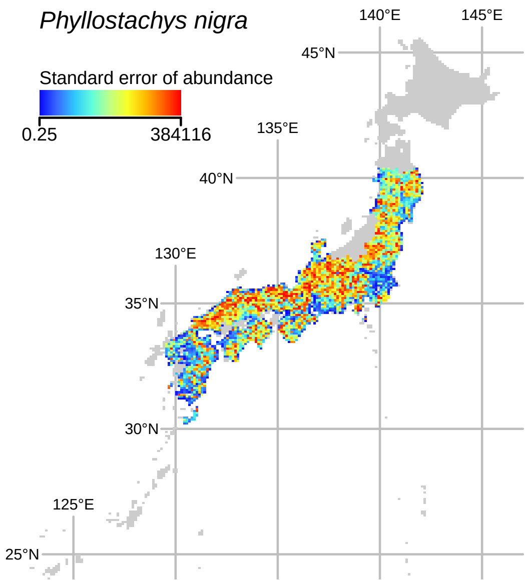
Phyllostachys nigra
Standard error of abundance
0.25
384116
140°E
145°E
45°N
135°E
40°N
130°E
35°N
30°N
125°E
25°N

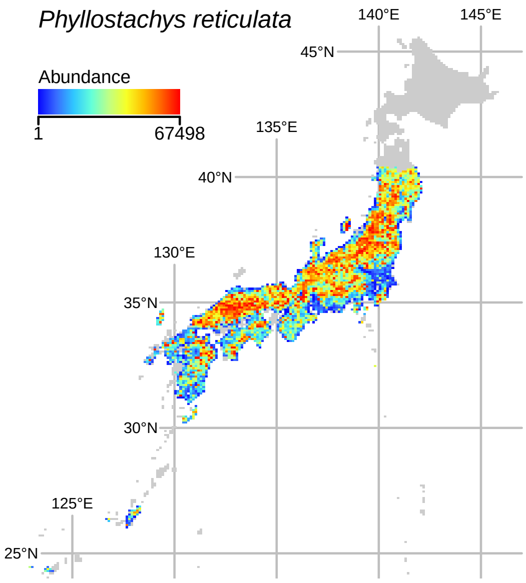
Phyllostachys reticulata
Abundance
1
67498
140°E
145°E
45°N
135°E
40°N
130°E
35°N
30°N
125°E
25°N

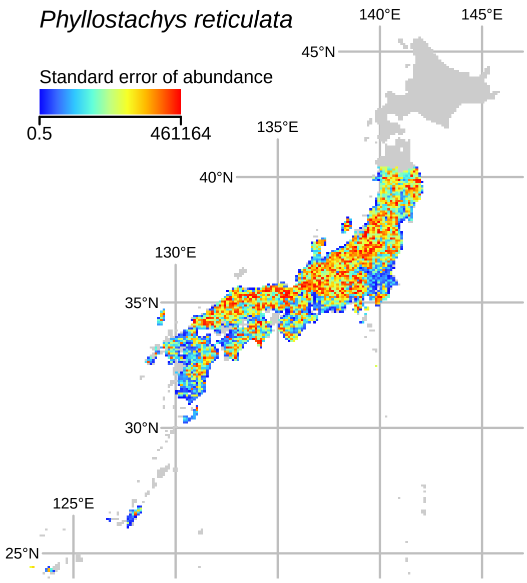
Phyllostachys reticulata
Standard error of abundance
0.5
461164
140°E
145°E
45°N
135°E
40°N
130°E
35°N
30°N
125°E
25°N

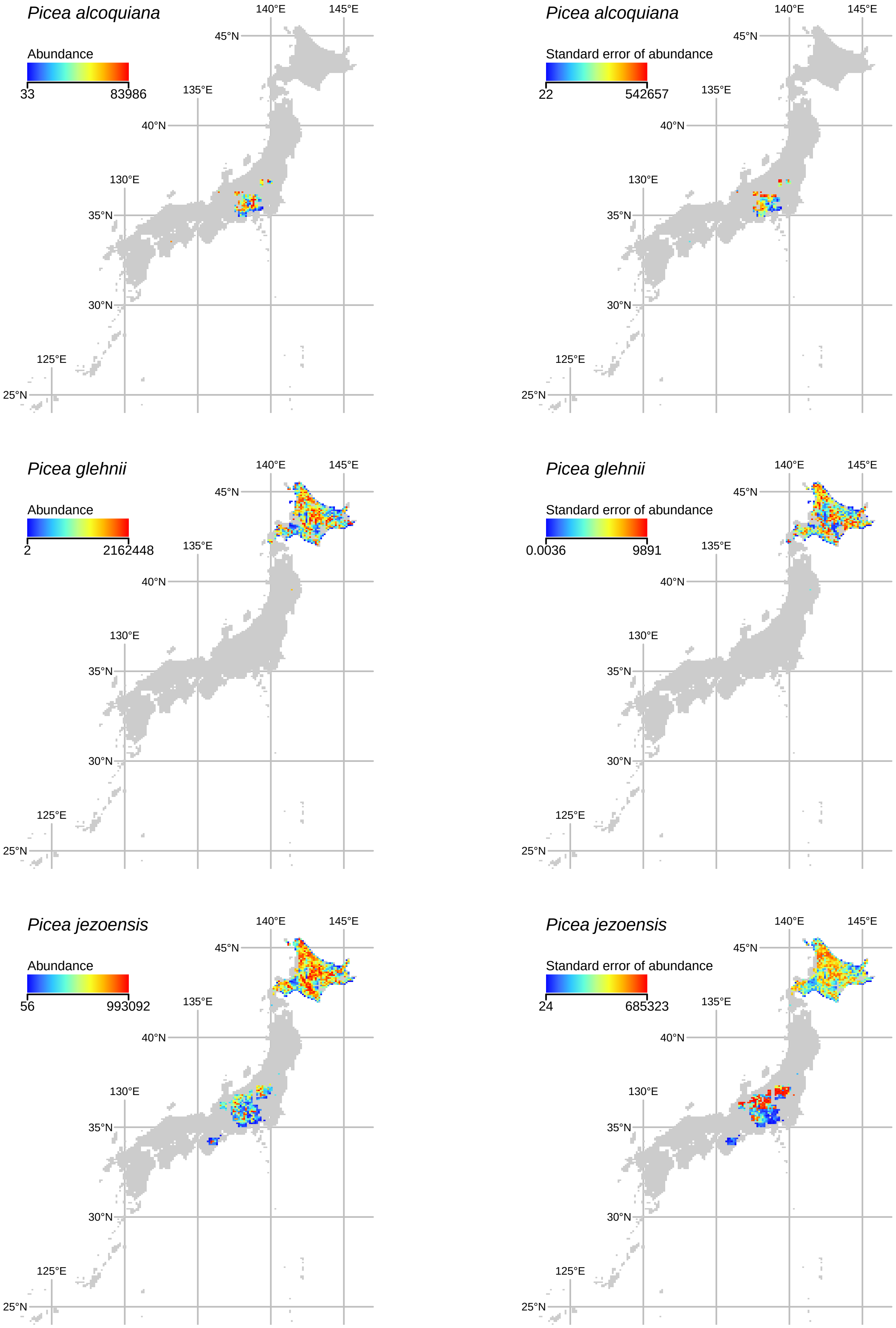
Picea alcoquiana
Abundance
33
83986
Picea alcoquiana
Standard error of abundance
22
542657
Picea glehnii
Abundance
2
2162448
Picea glehnii
Standard error of abundance
0.0036
9891
Picea jezoensis
Abundance
56
993092
Picea jezoensis
Standard error of abundance
24
685323
125°E
130°E
135°E
140°E
145°E
25°N
30°N
35°N
40°N
45°N

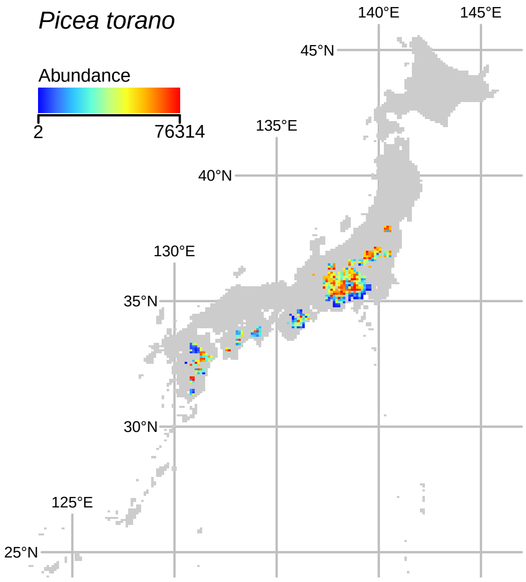
Picea torano
Abundance
2
76314
140°E
145°E
45°N
135°E
40°N
130°E
35°N
30°N
125°E
25°N

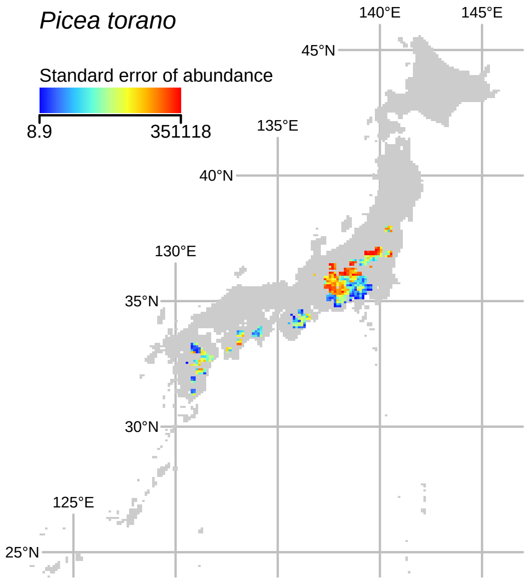
Picea torano
Standard error of abundance
8.9
351118
140°E
145°E
45°N
135°E
40°N
130°E
35°N
30°N
125°E
25°N

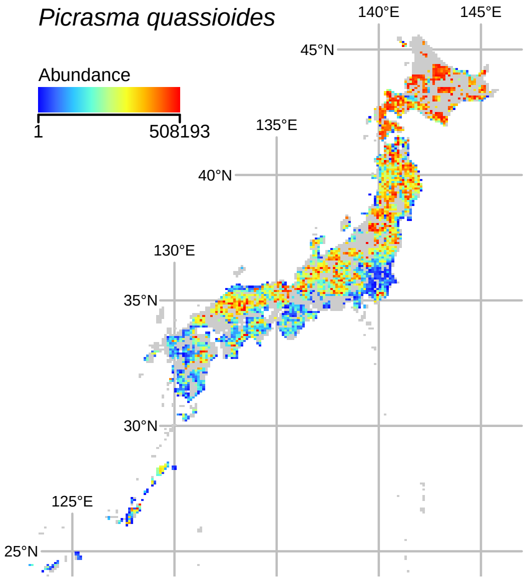
Picrasma quassioides
Abundance
1
508193
140°E
145°E
45°N
135°E
40°N
130°E
35°N
30°N
125°E
25°N

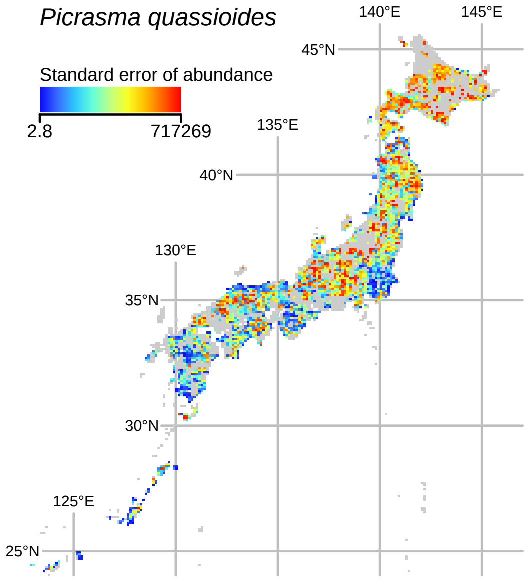
Picrasma quassioides
Standard error of abundance
2.8
717269
140°E
145°E
45°N
135°E
40°N
130°E
35°N
30°N
125°E
25°N

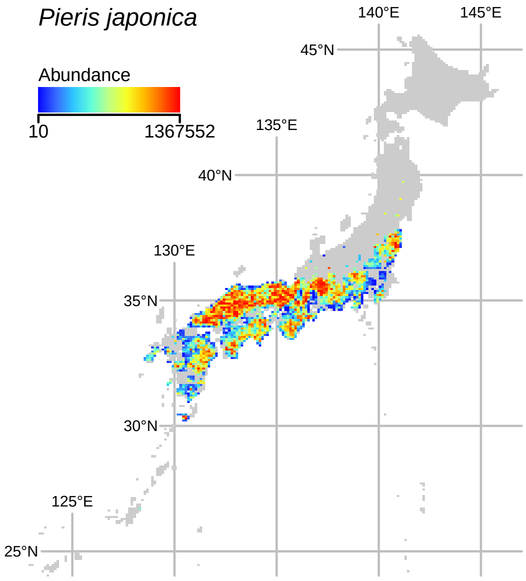
Pieris japonica
Abundance
10
1367552
140°E
145°E
45°N
135°E
40°N
130°E
35°N
30°N
125°E
25°N

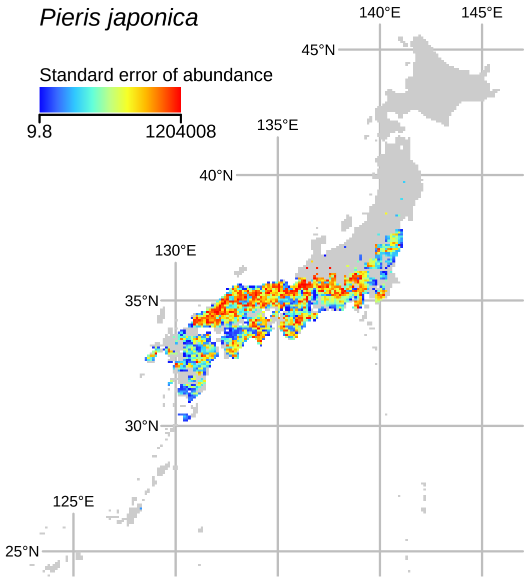
Pieris japonica
Standard error of abundance
9.8
1204008
140°E
145°E
45°N
135°E
40°N
130°E
35°N
30°N
125°E
25°N

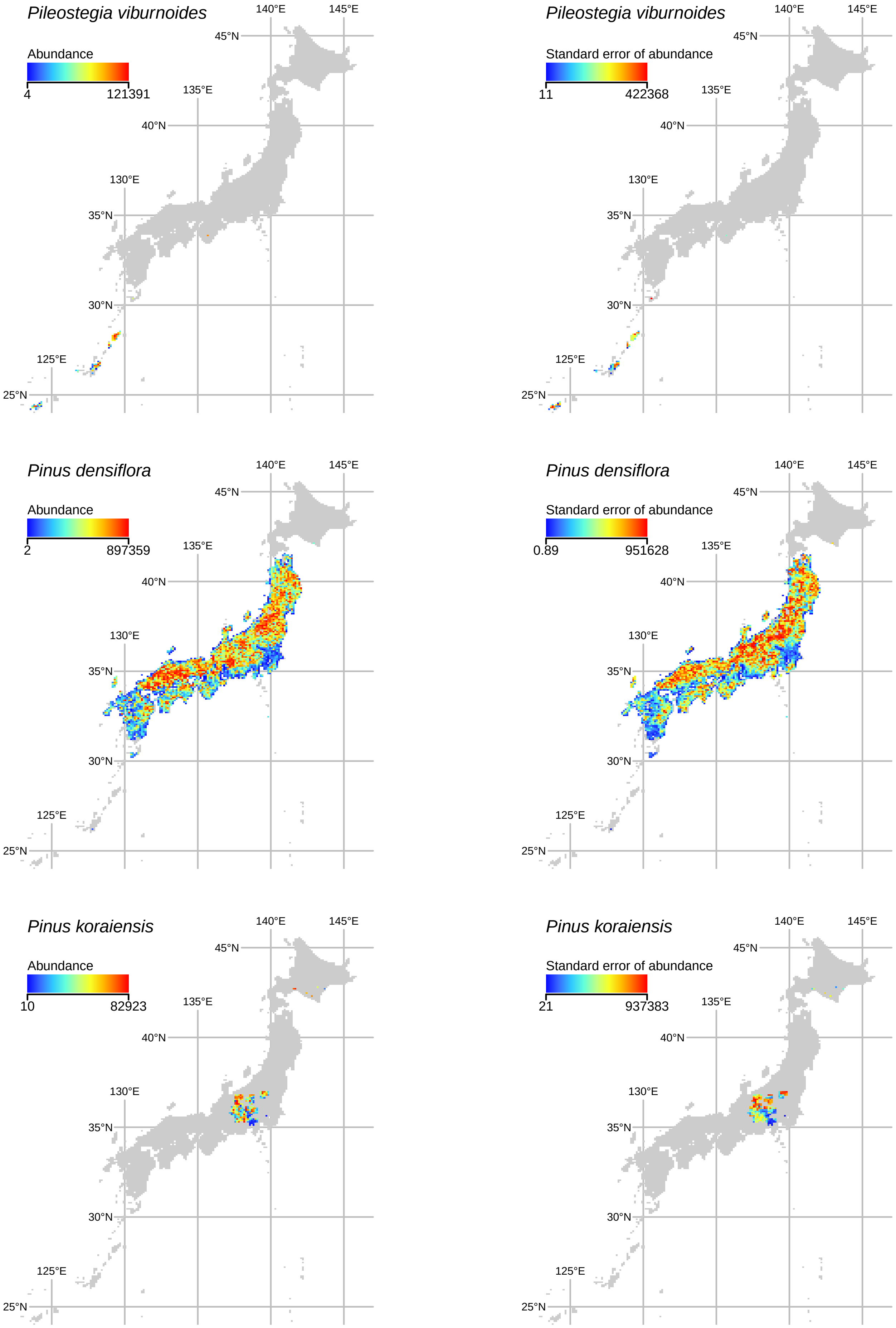
Pileostegia viburnoides
Abundance
4
121391
Pileostegia viburnoides
Standard error of abundance
11
422368
Pinus densiflora
Abundance
2
897359
Pinus densiflora
Standard error of abundance
0.89
951628
Pinus koraiensis
Abundance
10
82923
Pinus koraiensis
Standard error of abundance
21
937383
125°E
130°E
135°E
140°E
145°E
25°N
30°N
35°N
40°N
45°N

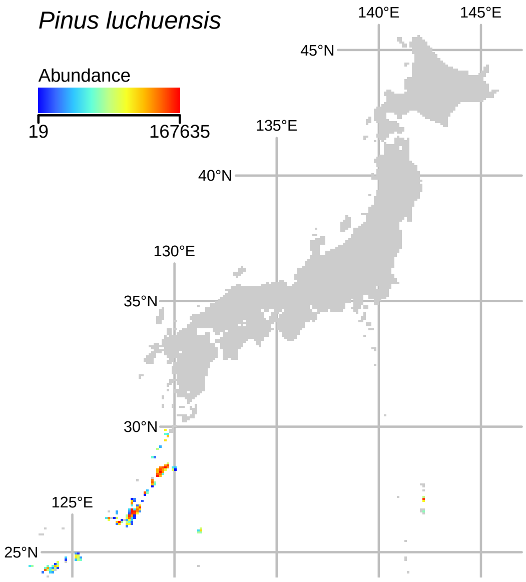
Pinus luchuensis
Abundance
19
167635
140°E
145°E
45°N
135°E
40°N
130°E
35°N
30°N
125°E
25°N

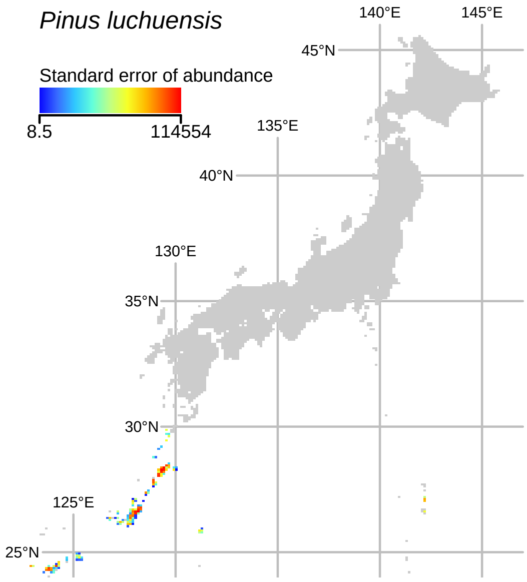
Pinus luchuensis
Standard error of abundance
8.5
114554
140°E
145°E
45°N
135°E
40°N
130°E
35°N
30°N
125°E
25°N

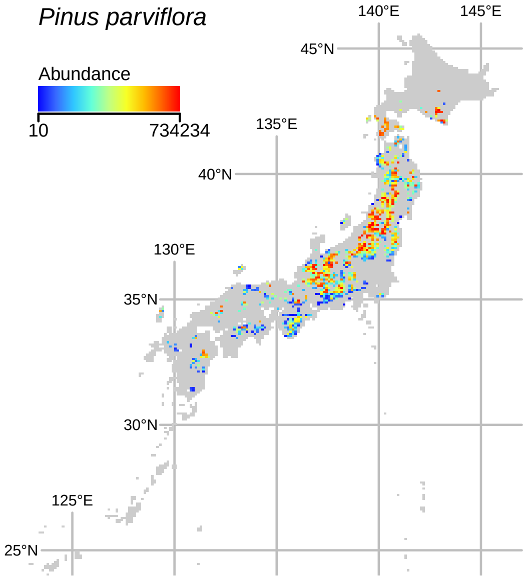
Pinus parviflora
Abundance
10
734234
140°E
145°E
45°N
135°E
40°N
130°E
35°N
30°N
125°E
25°N

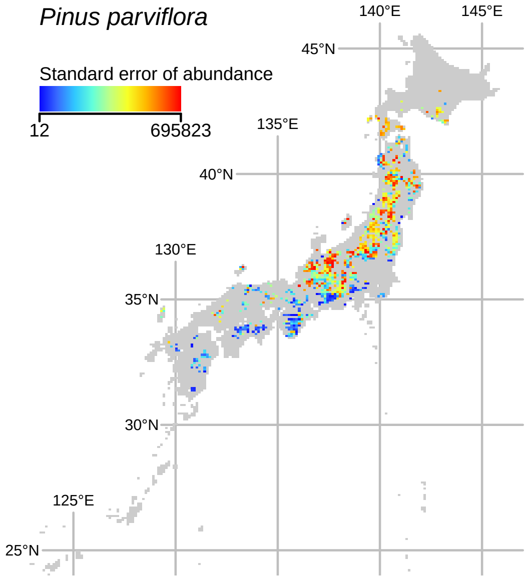
Pinus parviflora
Standard error of abundance
12
695823
140°E
145°E
45°N
135°E
40°N
130°E
35°N
30°N
125°E
25°N

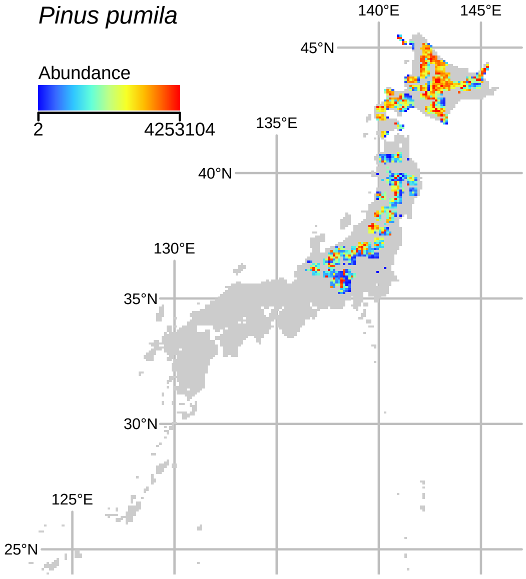
Pinus pumila
Abundance
2
4253104
140°E
145°E
45°N
135°E
40°N
130°E
35°N
30°N
125°E
25°N

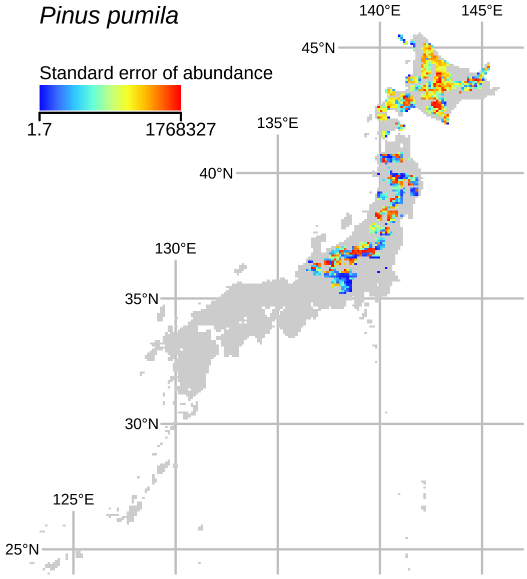
Pinus pumila
Standard error of abundance
1.7
1768327
140°E
145°E
45°N
135°E
40°N
130°E
35°N
30°N
125°E
25°N

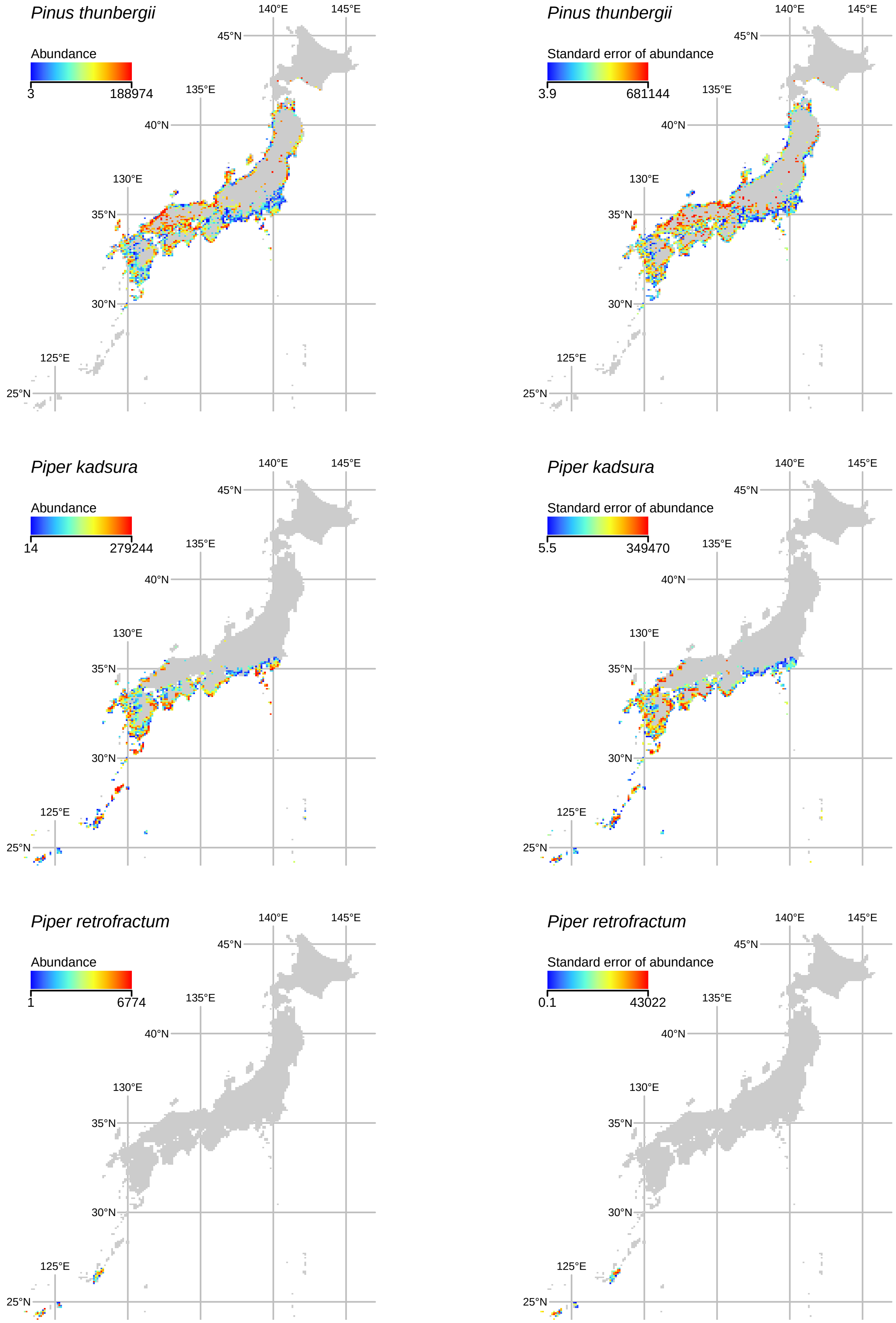
Pinus thunbergii
Abundance
3
188974
Pinus thunbergii
Standard error of abundance
3.9
681144
Piper kadsura
Abundance
14
279244
Piper kadsura
Standard error of abundance
5.5
349470
Piper retrofractum
Abundance
1
6774
Piper retrofractum
Standard error of abundance
0.1
43022
140°E
145°E
45°N
135°E
40°N
130°E
35°N
30°N
125°E
25°N

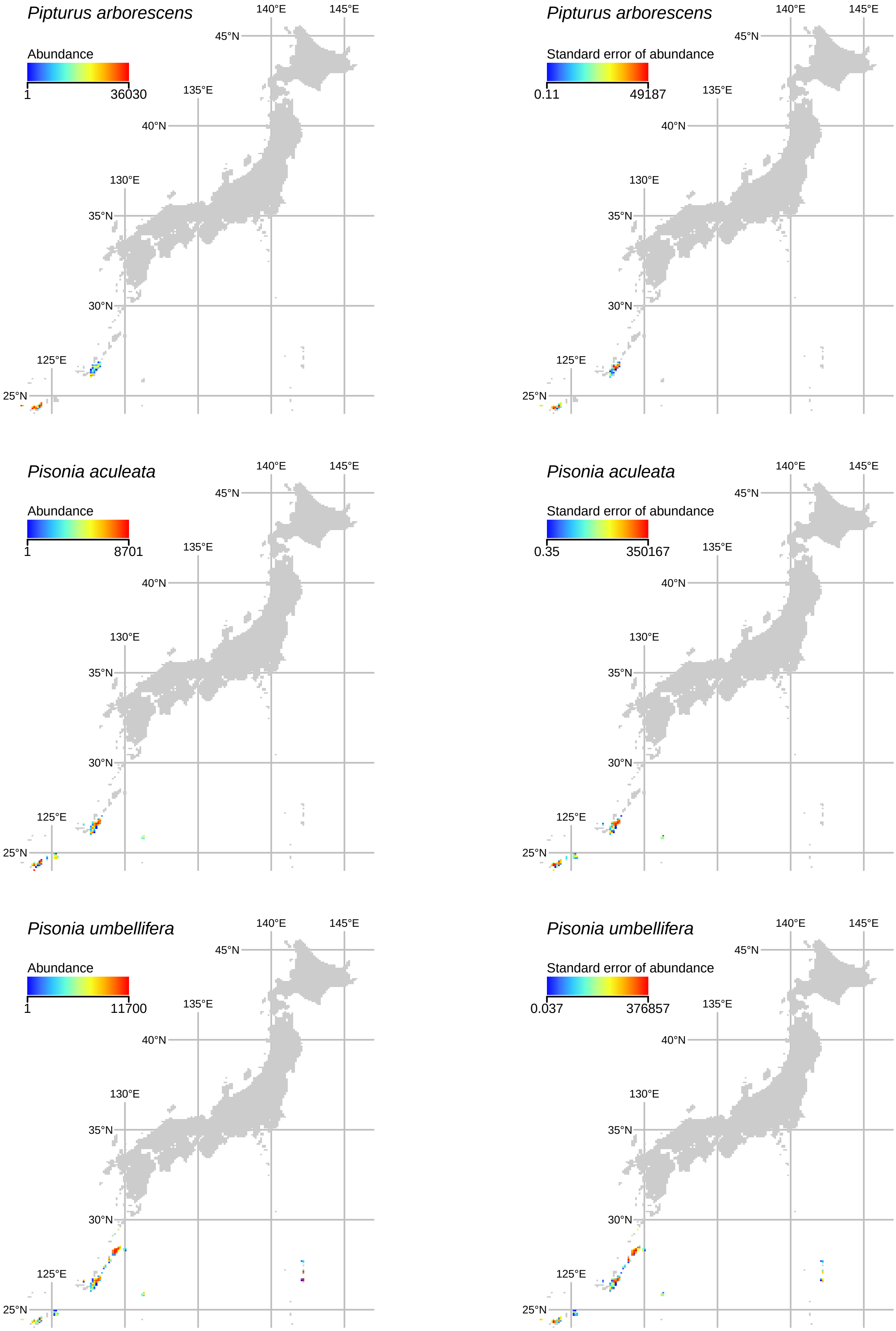
Pipturus arborescens
Abundance
1
36030
Pipturus arborescens
Standard error of abundance
0.11
49187
Pisonia aculeata
Abundance
1
8701
Pisonia aculeata
Standard error of abundance
0.35
350167
Pisonia umbellifera
Abundance
1
11700
Pisonia umbellifera
Standard error of abundance
0.037
376857
140°E
145°E
45°N
135°E
40°N
130°E
35°N
30°N
125°E
25°N

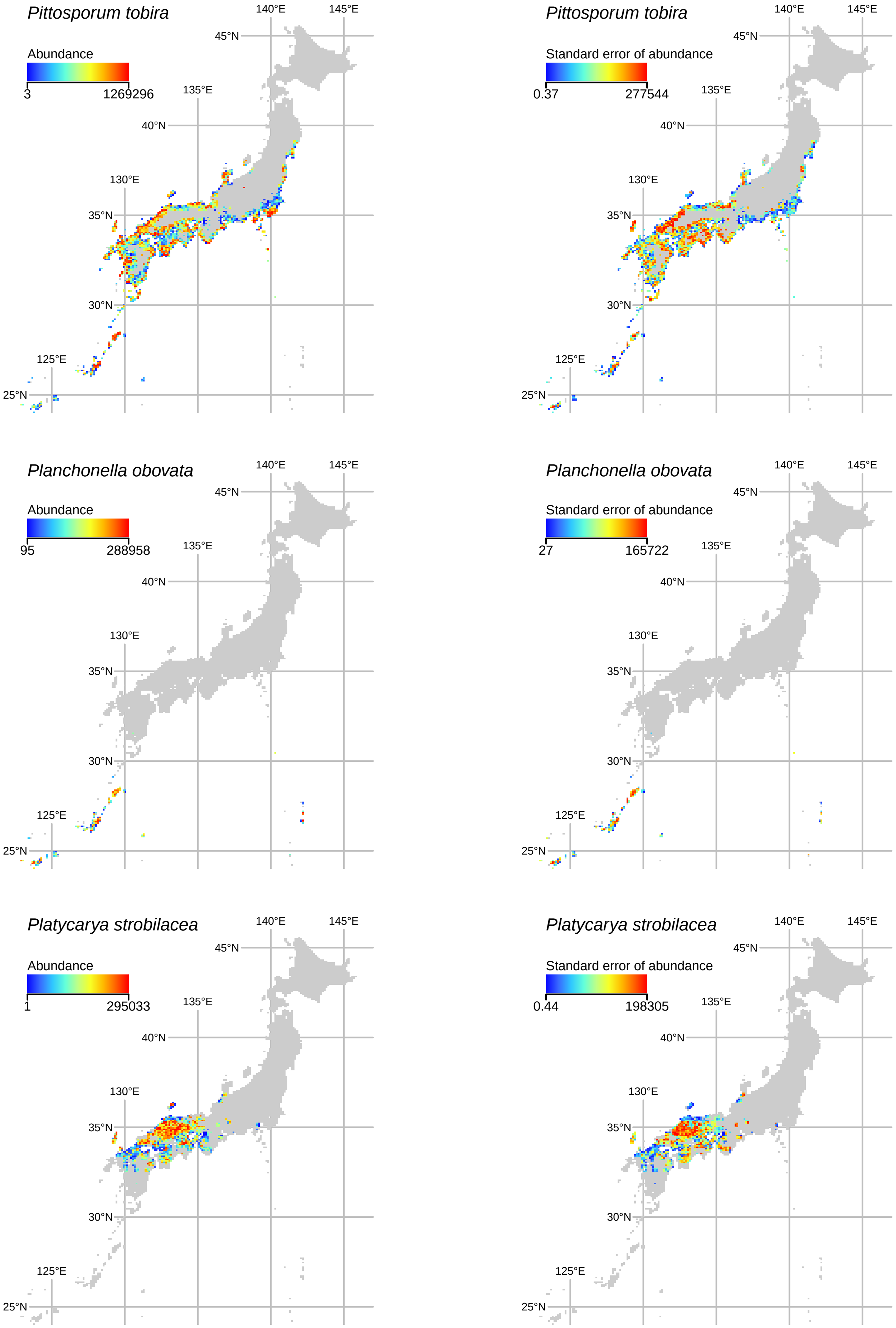
Pittosporum tobira
Abundance
3
1269296
Pittosporum tobira
Standard error of abundance
0.37
277544
Planchonella obovata
Abundance
95
288958
Planchonella obovata
Standard error of abundance
27
165722
Platycarya strobilacea
Abundance
1
295033
Platycarya strobilacea
Standard error of abundance
0.44
198305
125°E
130°E
135°E
140°E
145°E
25°N
30°N
35°N
40°N
45°N

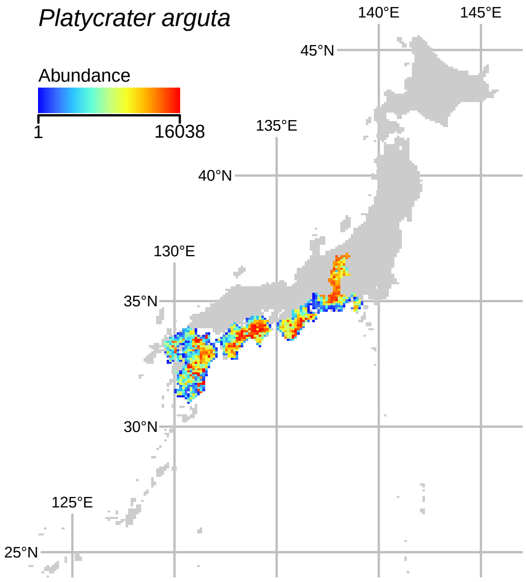
Platycrater arguta
Abundance
1
16038
140°E
145°E
45°N
135°E
40°N
130°E
35°N
30°N
125°E
25°N

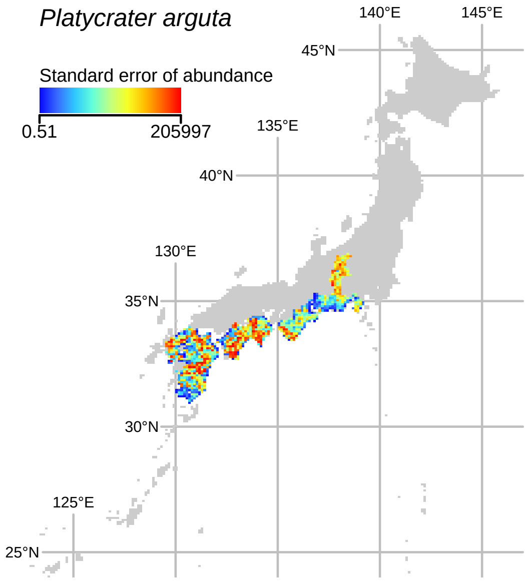
Platycrater arguta
Standard error of abundance
0.51
205997
140°E
145°E
45°N
135°E
40°N
130°E
35°N
30°N
125°E
25°N

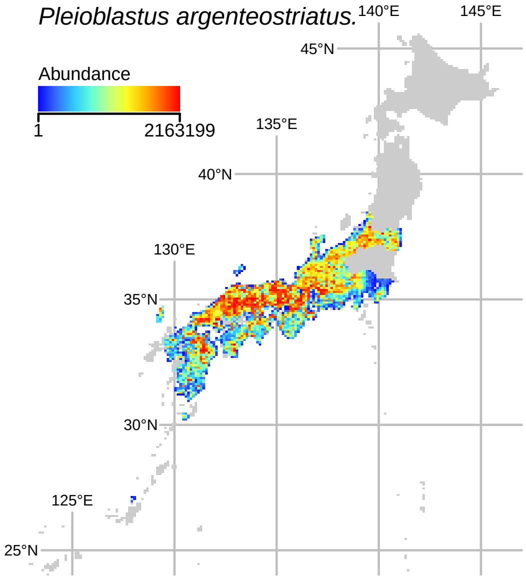
Pleioblastus argenteostriatus.
Abundance
1
2163199
140°E
145°E
45°N
135°E
40°N
130°E
35°N
30°N
125°E
25°N

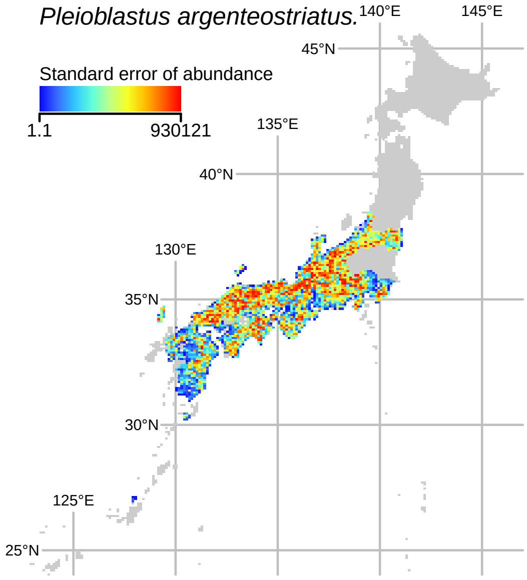
Pleioblastus argenteostriatus.
Standard error of abundance
1.1
930121
140°E
145°E
45°N
135°E
40°N
130°E
35°N
30°N
125°E
25°N

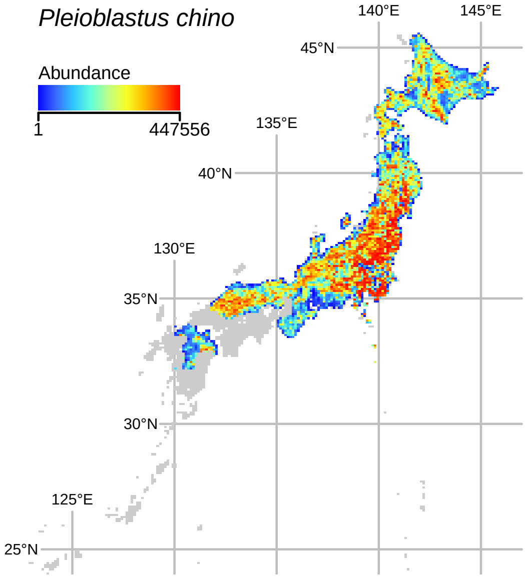
Pleioblastus chino
Abundance
1
447556
140°E
145°E
45°N
135°E
40°N
130°E
35°N
30°N
125°E
25°N

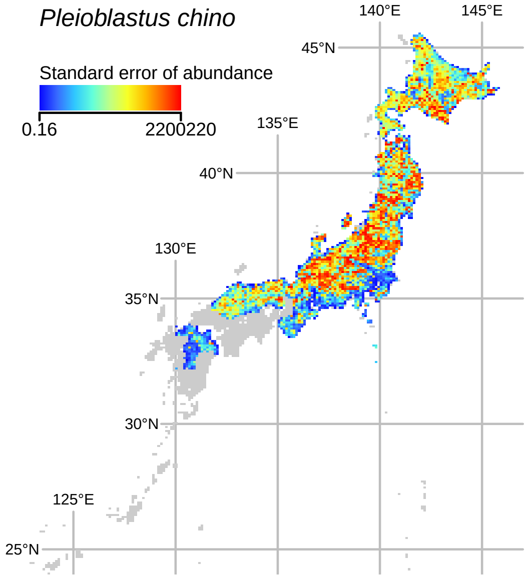
Pleioblastus chino
Standard error of abundance
0.16
2200220
140°E
145°E
45°N
135°E
40°N
130°E
35°N
30°N
125°E
25°N

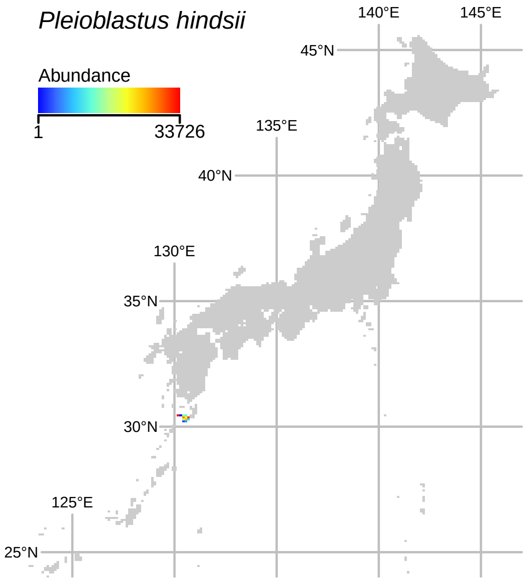
Pleioblastus hindsii
Abundance
1
33726
140°E
145°E
45°N
135°E
40°N
130°E
35°N
30°N
125°E
25°N

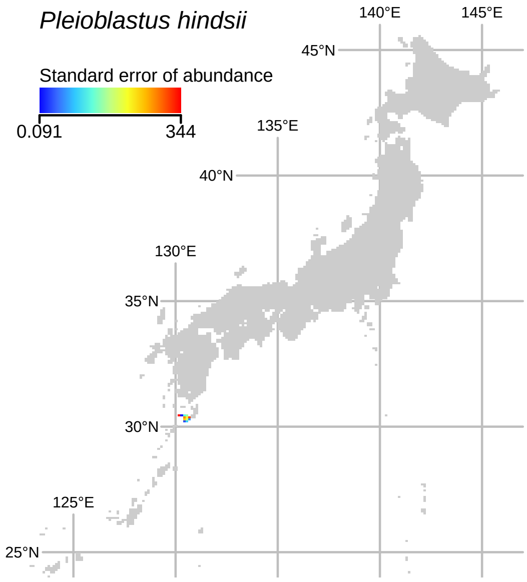
Pleioblastus hindsii
Standard error of abundance
0.091
344
140°E
145°E
45°N
135°E
40°N
130°E
35°N
30°N
125°E
25°N

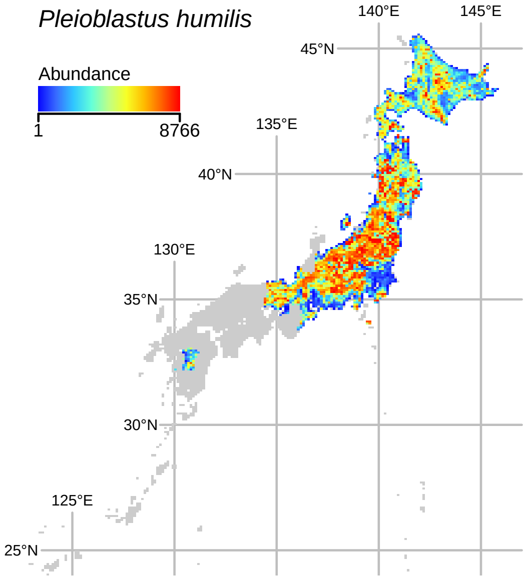
Pleioblastus humilis
Abundance
1
8766
140°E
145°E
45°N
135°E
40°N
130°E
35°N
30°N
125°E
25°N

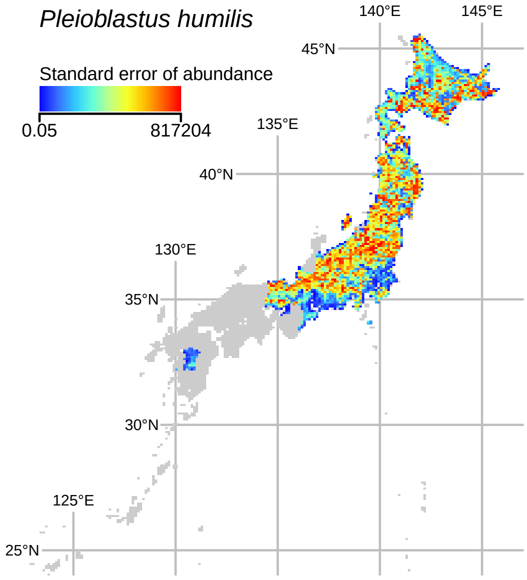
Pleioblastus humilis
Standard error of abundance
0.05
817204
140°E
145°E
45°N
135°E
40°N
130°E
35°N
30°N
125°E
25°N

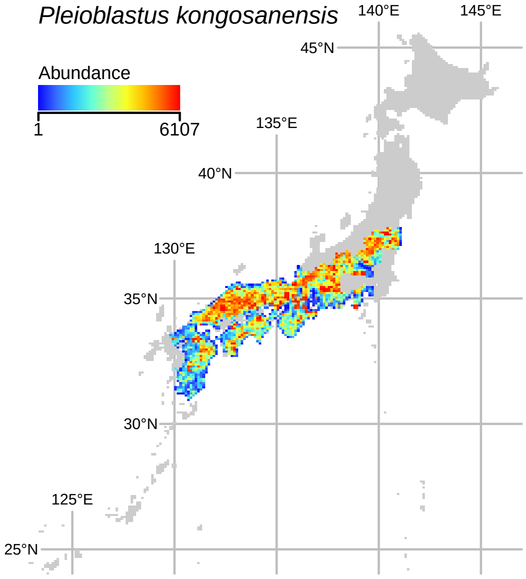
Pleioblastus kongosanensis
Abundance
1
6107
140°E
145°E
45°N
135°E
40°N
130°E
35°N
30°N
125°E
25°N

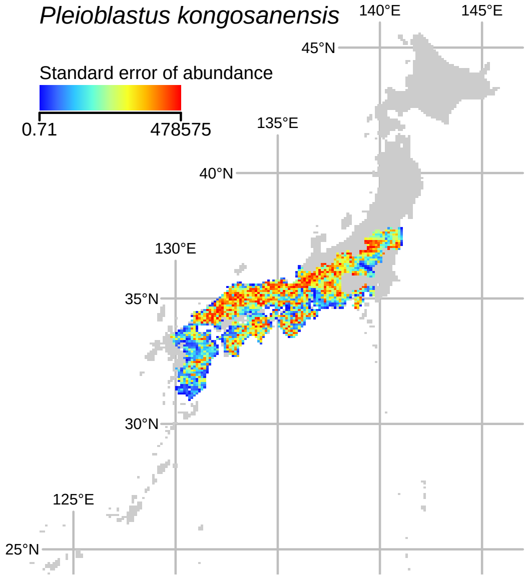
Pleioblastus kongosanensis
Standard error of abundance
0.71
478575
140°E
145°E
45°N
135°E
40°N
130°E
35°N
30°N
125°E
25°N

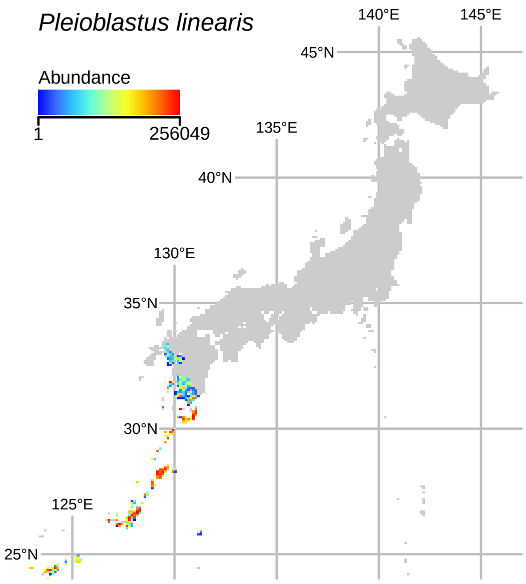
Pleioblastus linearis
Abundance
1
256049
140°E
145°E
45°N
135°E
40°N
130°E
35°N
30°N
125°E
25°N

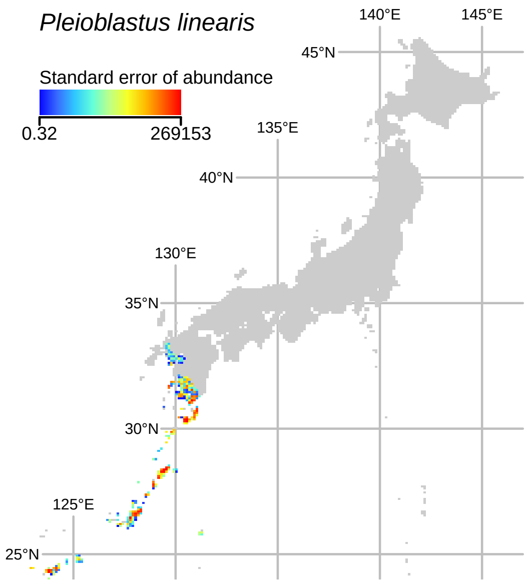
Pleioblastus linearis
Standard error of abundance
0.32
269153
140°E
145°E
45°N
135°E
40°N
130°E
35°N
30°N
125°E
25°N

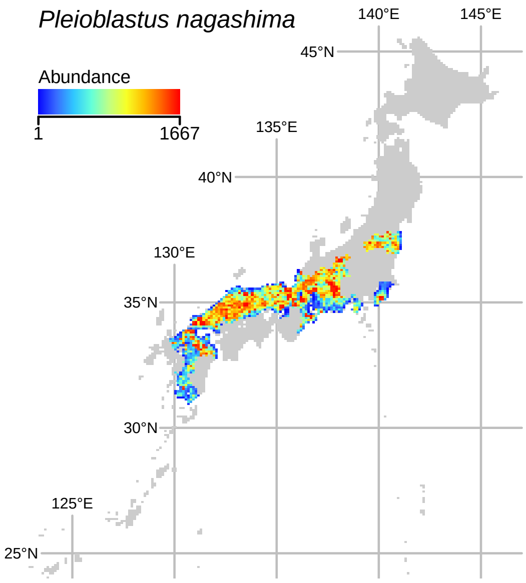
Pleioblastus nagashima
Abundance
1
1667
140°E
145°E
45°N
135°E
40°N
130°E
35°N
30°N
125°E
25°N

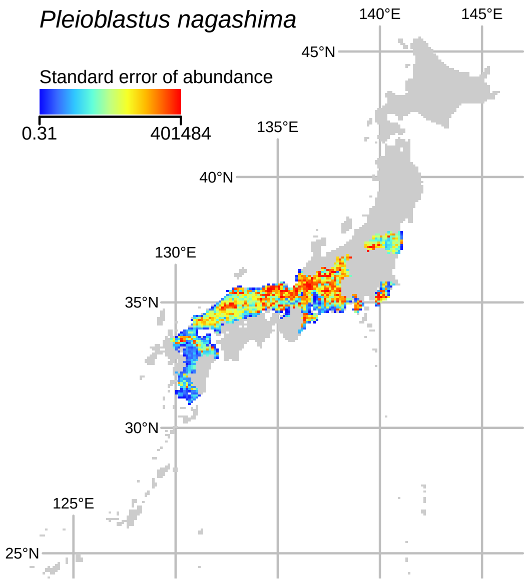
Pleioblastus nagashima
Standard error of abundance
0.31
401484
140°E
145°E
45°N
135°E
40°N
130°E
35°N
30°N
125°E
25°N

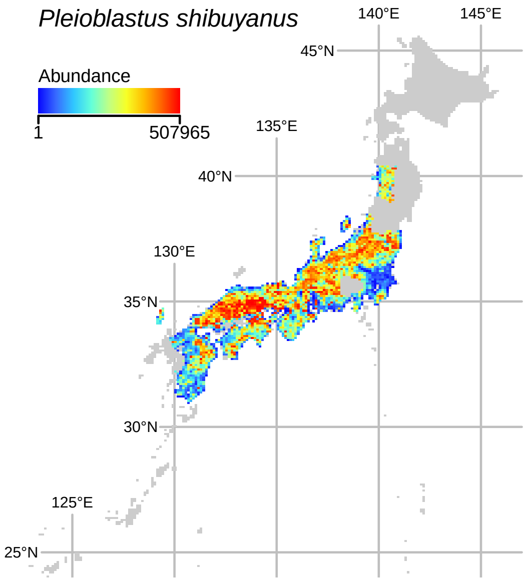
Pleioblastus shibuyanus
Abundance
1
507965
140°E
145°E
45°N
135°E
40°N
130°E
35°N
30°N
125°E
25°N

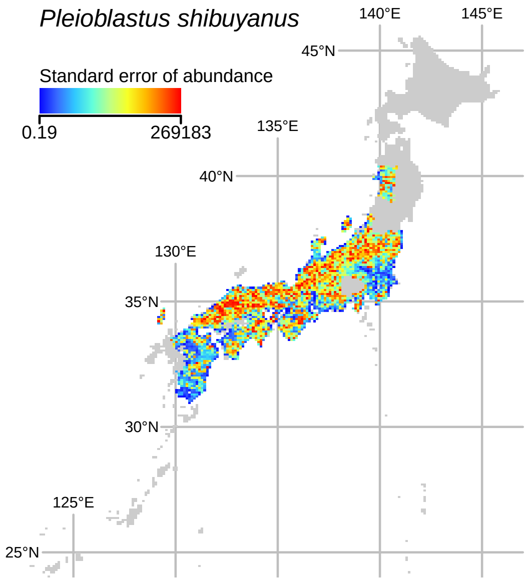
Pleioblastus shibuyanus
Standard error of abundance
0.19
269183
140°E
145°E
45°N
135°E
40°N
130°E
35°N
30°N
125°E
25°N

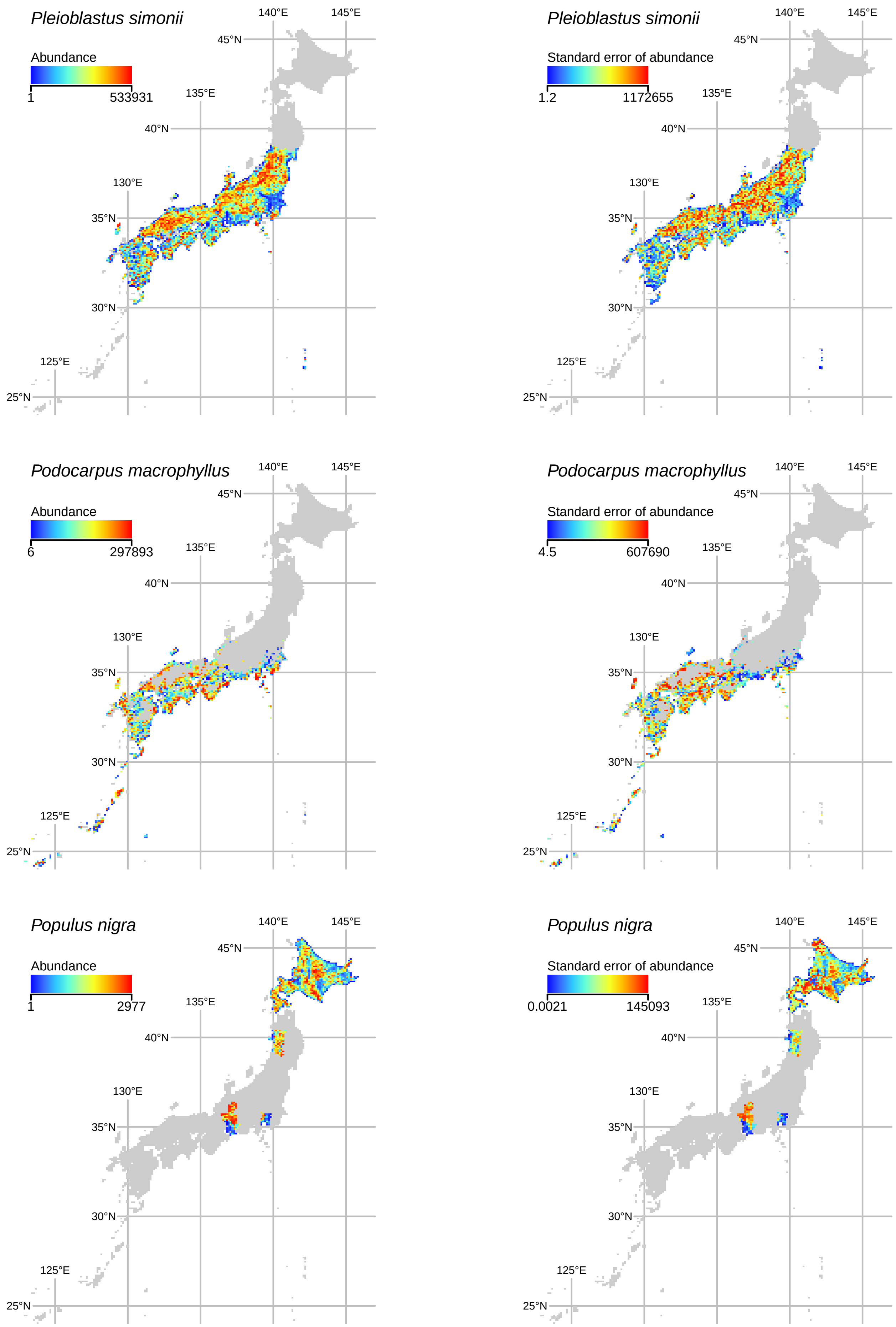

Pleioblastus simonii
Abundance
1
533931
Pleioblastus simonii
Standard error of abundance
1.2
1172655
Podocarpus macrophyllus
Abundance
6
297893
Podocarpus macrophyllus
Standard error of abundance
4.5
607690
Populus nigra
Abundance
1
2977
Populus nigra
Standard error of abundance
0.0021
145093
125°E
130°E
135°E
140°E
145°E
25°N
30°N
35°N
40°N
45°N

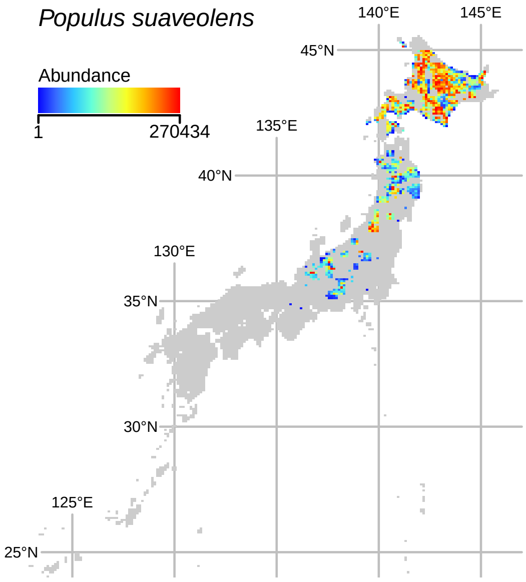
Populus suaveolens
Abundance
1
270434
140°E
145°E
45°N
135°E
40°N
130°E
35°N
30°N
125°E
25°N

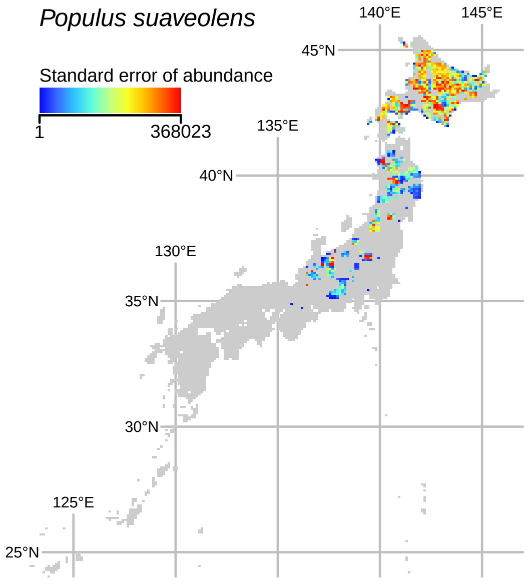
Populus suaveolens
Standard error of abundance
1
368023
140°E
145°E
45°N
135°E
40°N
130°E
35°N
30°N
125°E
25°N

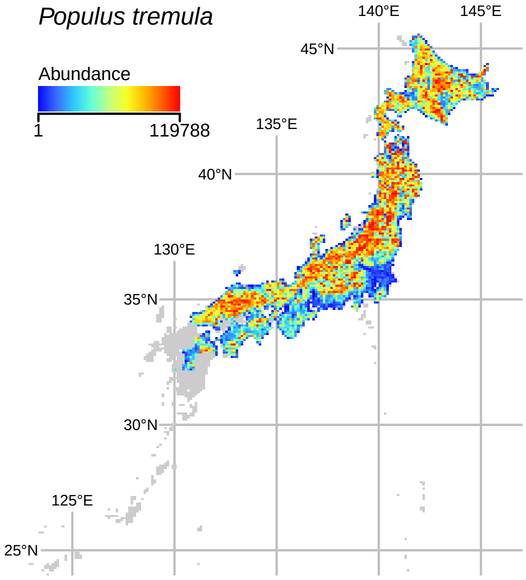
Populus tremula
Abundance
1
119788
140°E
145°E
45°N
135°E
40°N
130°E
35°N
30°N
125°E
25°N

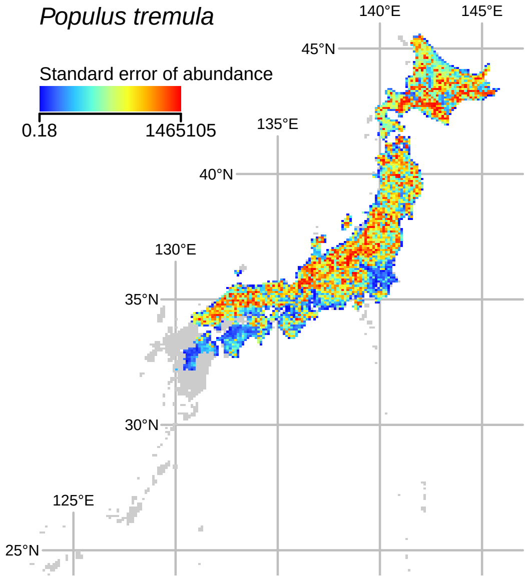
Populus tremula
Standard error of abundance
0.18
1465105
140°E
145°E
45°N
135°E
40°N
130°E
35°N
30°N
125°E
25°N

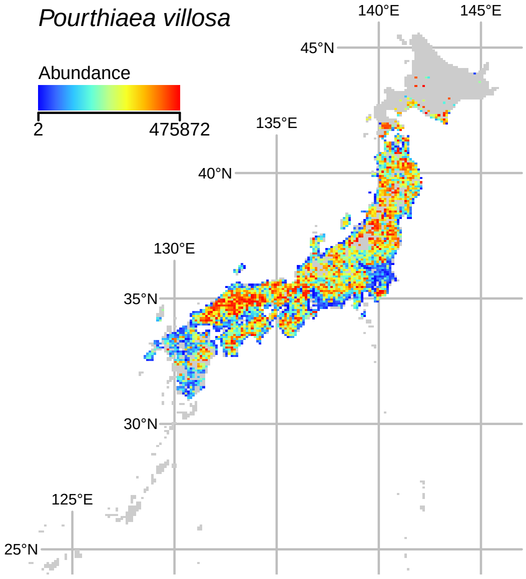
Pourthiaea villosa
Abundance
2
475872
140°E
145°E
45°N
135°E
40°N
130°E
35°N
30°N
125°E
25°N

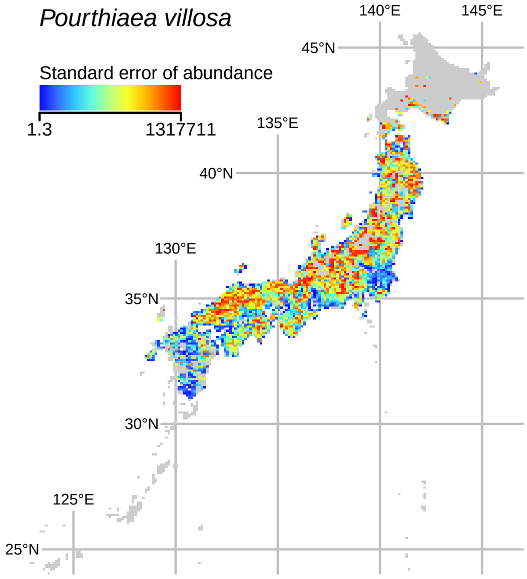
Pourthiaea villosa
Standard error of abundance
1.3
1317711
140°E
145°E
45°N
135°E
40°N
130°E
35°N
30°N
125°E
25°N

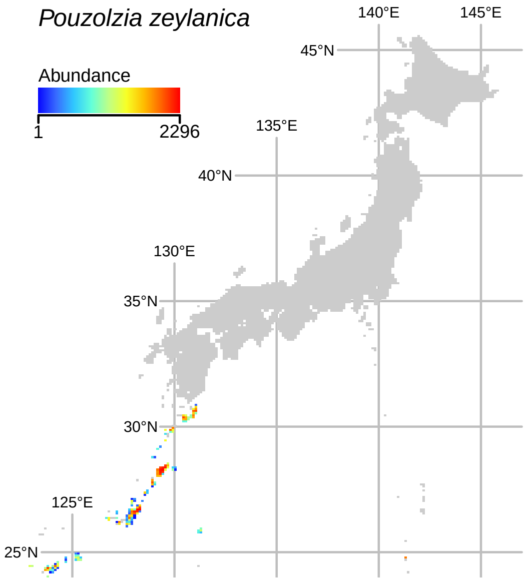
Pouzolzia zeylanica
Abundance
1
2296
140°E
145°E
45°N
135°E
40°N
130°E
35°N
30°N
125°E
25°N

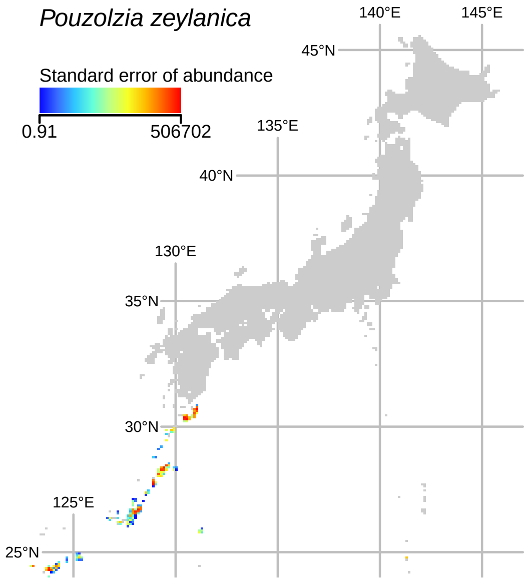
Pouzolzia zeylanica
Standard error of abundance
0.91
506702
140°E
145°E
45°N
135°E
40°N
130°E
35°N
30°N
125°E
25°N

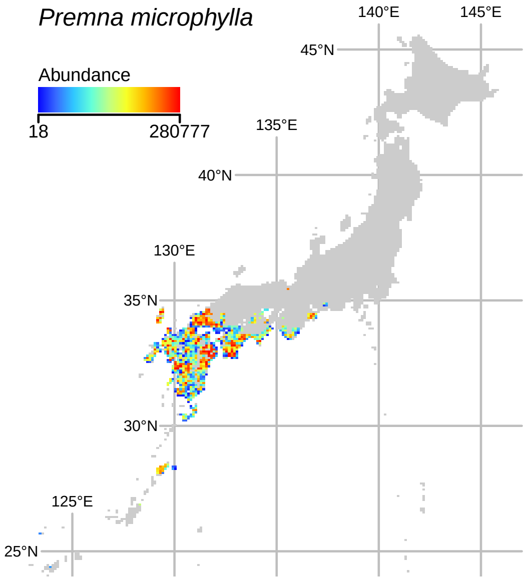
Premna microphylla
Abundance
18
280777
140°E
145°E
45°N
135°E
40°N
130°E
35°N
30°N
125°E
25°N

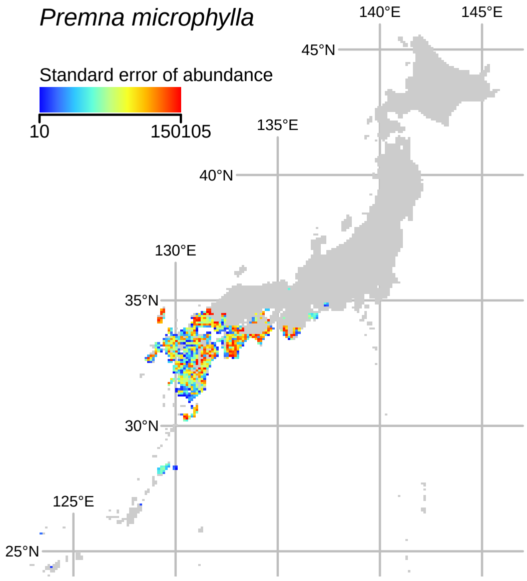
Premna microphylla
Standard error of abundance
10
150105
140°E
145°E
45°N
135°E
40°N
130°E
35°N
30°N
125°E
25°N

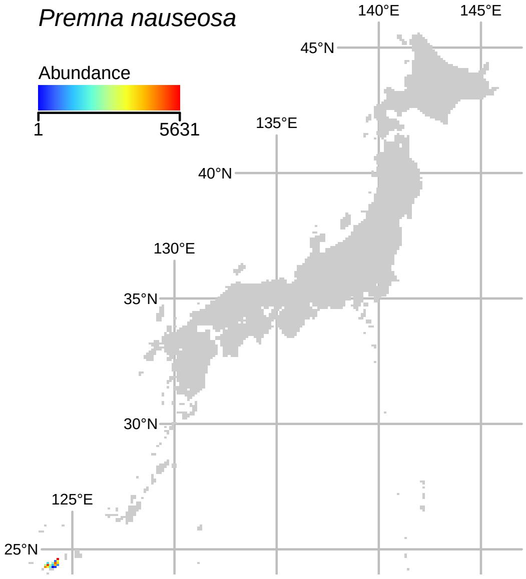
Premna nauseosa
Abundance
1
5631
140°E
145°E
45°N
135°E
40°N
130°E
35°N
30°N
125°E
25°N

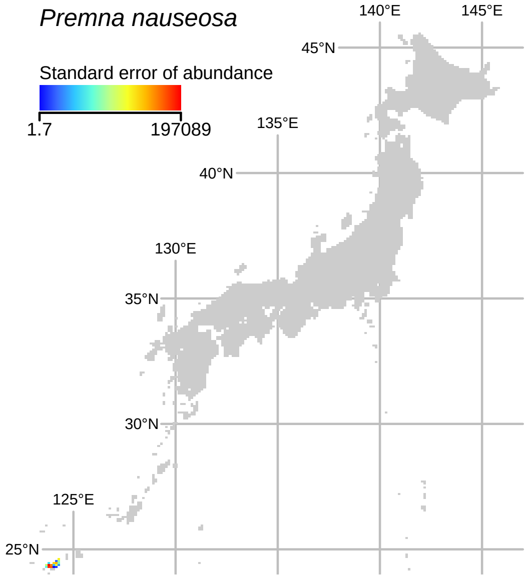
Premna nauseosa
Standard error of abundance
1.7
197089
140°E
145°E
45°N
135°E
40°N
130°E
35°N
30°N
125°E
25°N

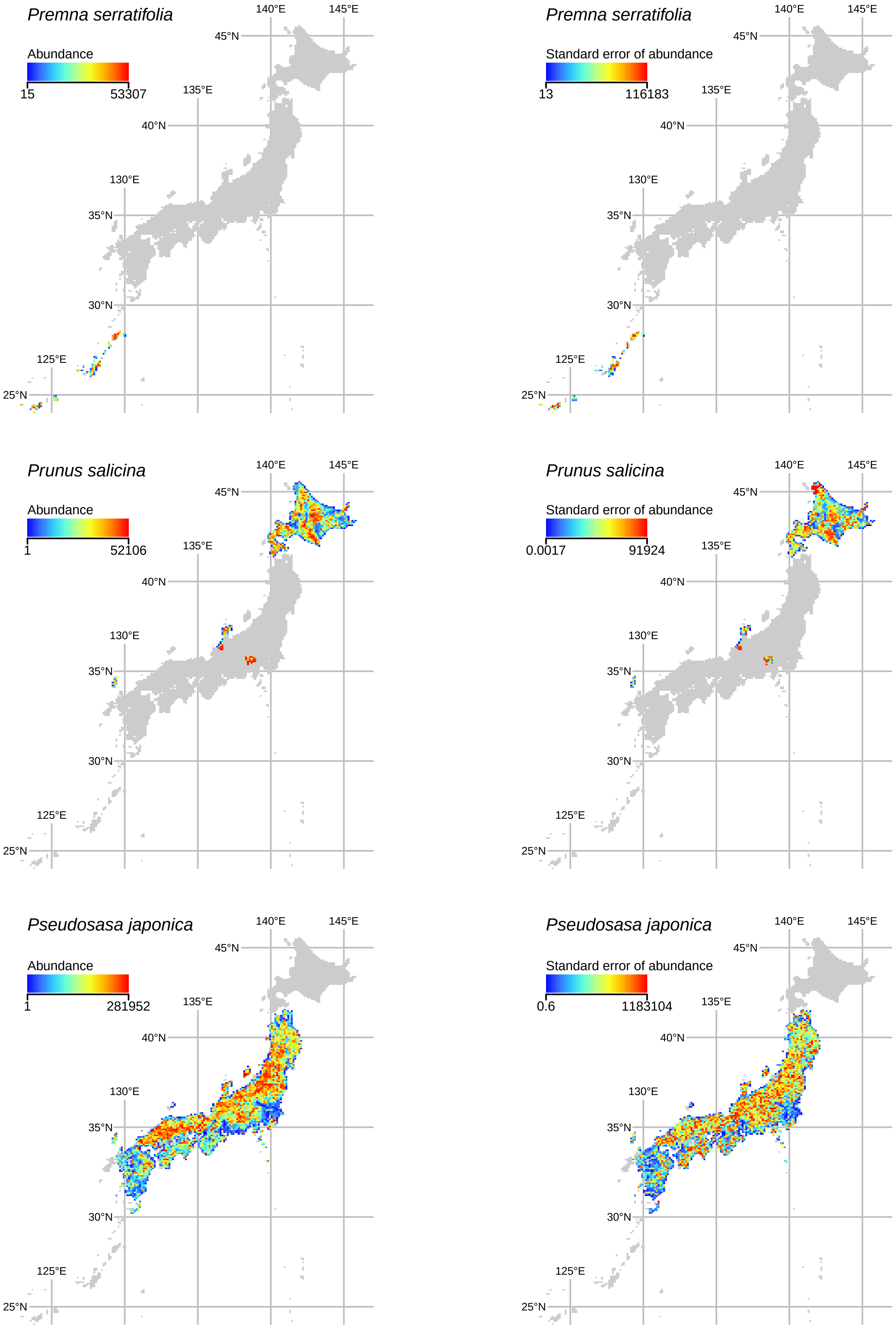

Premna serratifolia
Abundance
15
53307
Premna serratifolia
Standard error of abundance
13
116183
Prunus salicina
Abundance
1
52106
Prunus salicina
Standard error of abundance
0.0017
91924
Pseudosasa japonica
Abundance
1
281952
Pseudosasa japonica
Standard error of abundance
0.6
1183104
125°E
130°E
135°E
140°E
145°E
25°N
30°N
35°N
40°N
45°N

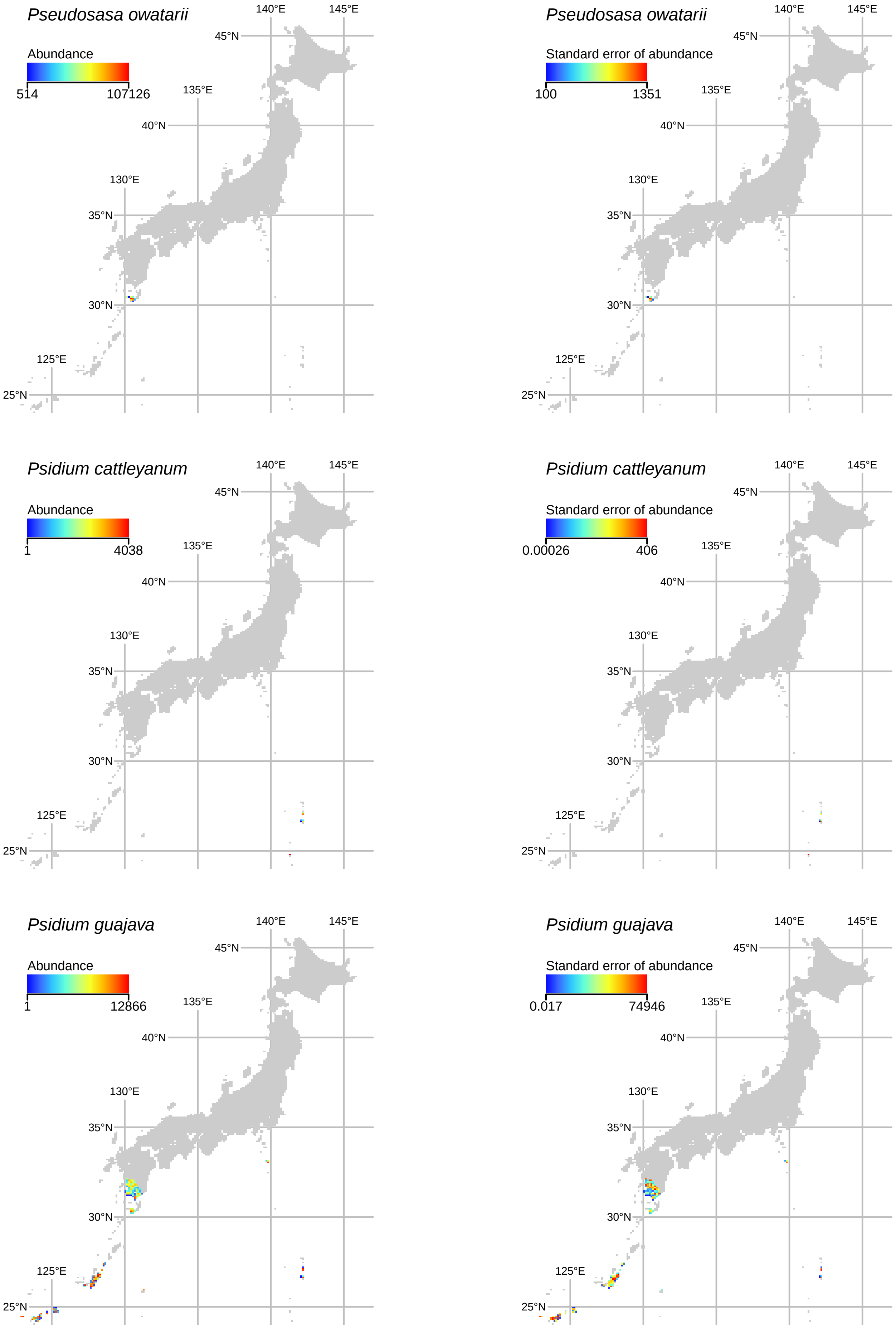
Pseudosasa owatarii
Abundance
514
107126
Pseudosasa owatarii
Standard error of abundance
100
1351
Psidium cattleyanum
Abundance
1
4038
Psidium cattleyanum
Standard error of abundance
0.00026
406
Psidium guajava
Abundance
1
12866
Psidium guajava
Standard error of abundance
0.017
74946
125°E
130°E
135°E
140°E
145°E
25°N
30°N
35°N
40°N
45°N

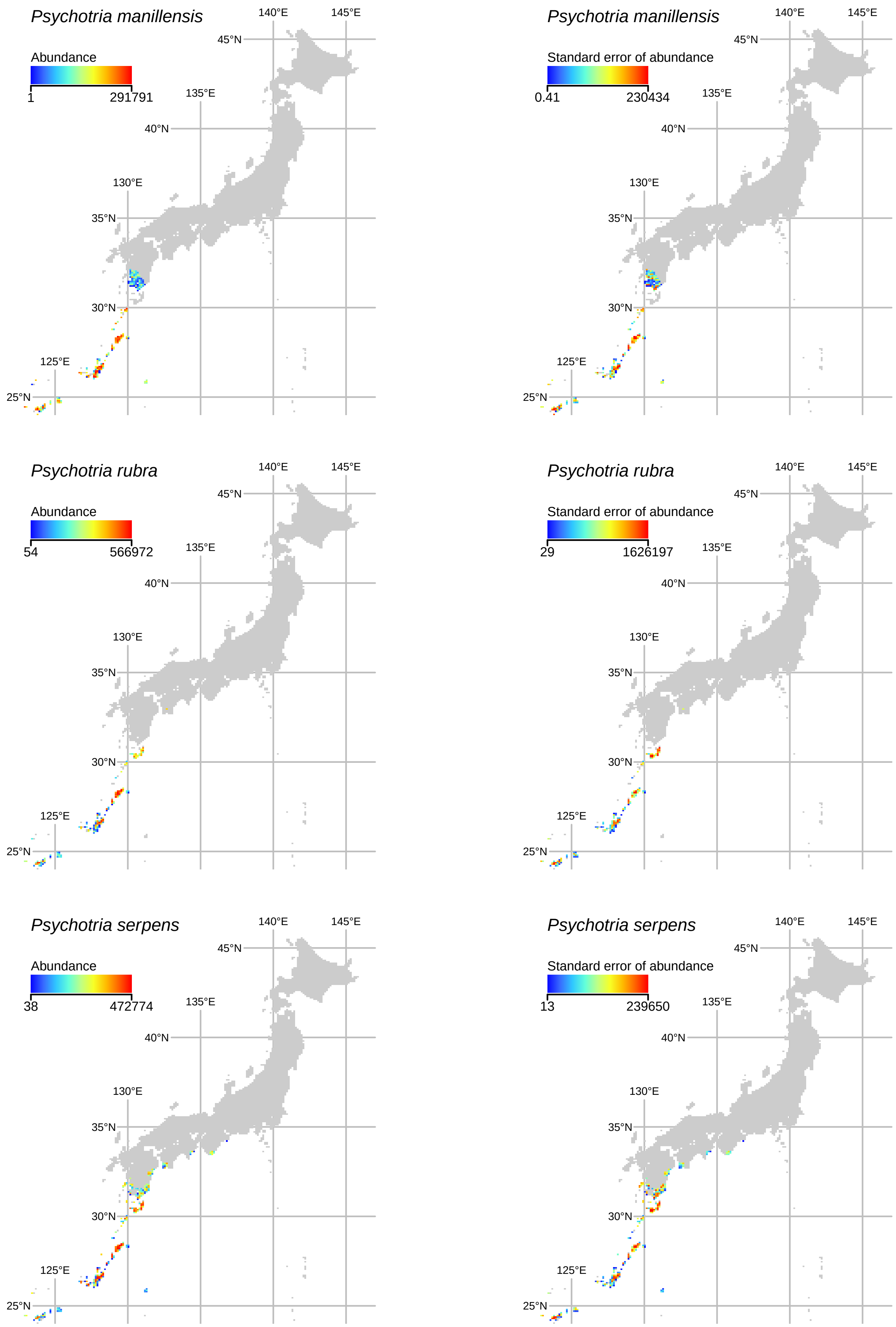

Psychotria manillensis
Abundance
1
291791
Psychotria manillensis
Standard error of abundance
0.41
230434
Psychotria rubra
Abundance
54
566972
Psychotria rubra
Standard error of abundance
29
1626197
Psychotria serpens
Abundance
38
472774
Psychotria serpens
Standard error of abundance
13
239650
125°E
130°E
135°E
140°E
145°E
25°N
30°N
35°N
40°N
45°N

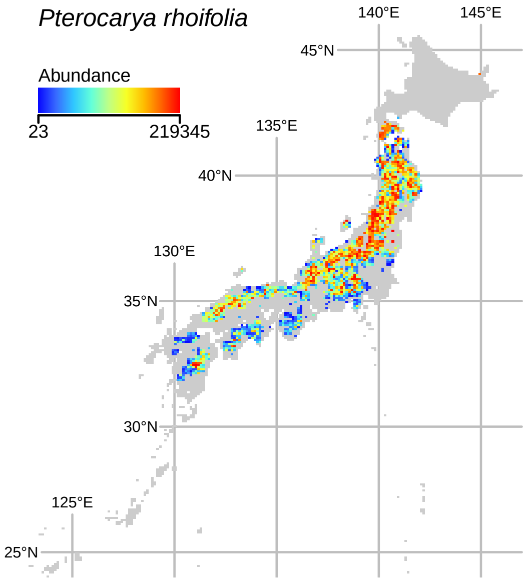
Pterocarya rhoifolia
Abundance
23
219345
140°E
145°E
135°E
130°E
125°E
45°N
40°N
35°N
30°N
25°N

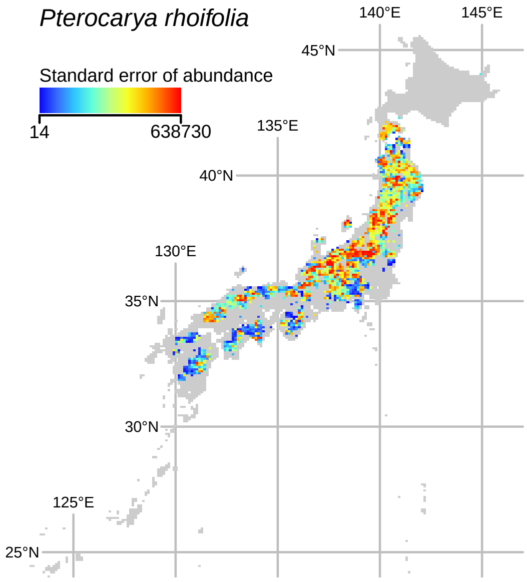
Pterocarya rhoifolia
Standard error of abundance
14
638730
140°E
145°E
135°E
130°E
125°E
45°N
40°N
35°N
30°N
25°N

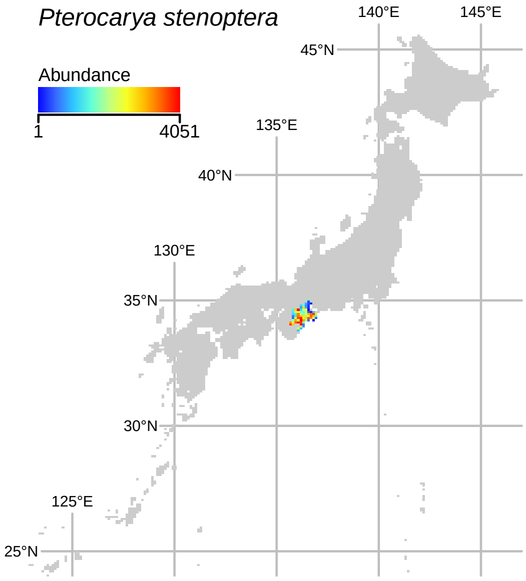
Pterocarya stenoptera
Abundance
1
4051
140°E
145°E
135°E
130°E
125°E
45°N
40°N
35°N
30°N
25°N

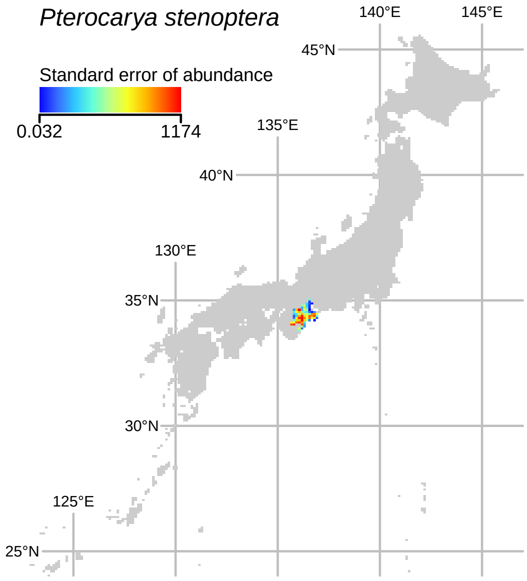
Pterocarya stenoptera
Standard error of abundance
0.032
1174
140°E
145°E
135°E
130°E
125°E
45°N
40°N
35°N
30°N
25°N

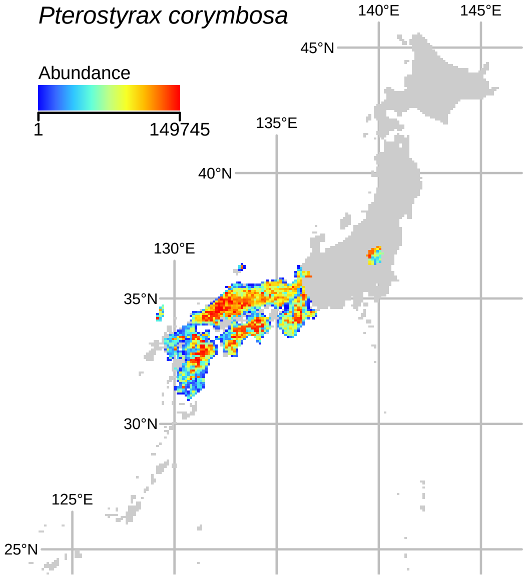
Pterostyrax corymbosa
Abundance
1
149745
140°E
145°E
135°E
130°E
125°E
45°N
40°N
35°N
30°N
25°N

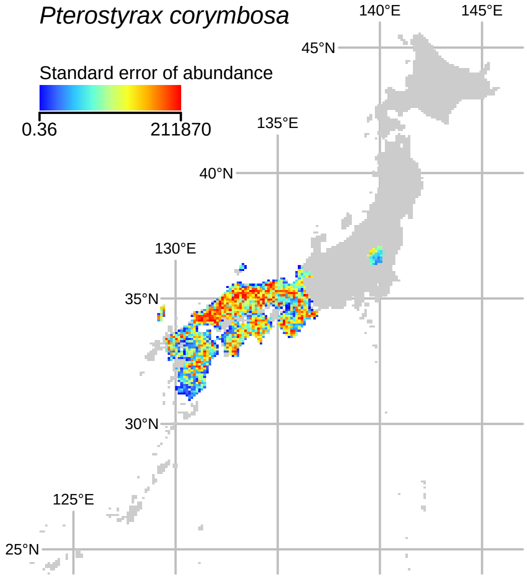
Pterostyrax corymbosa
Standard error of abundance
0.36
211870
140°E
145°E
135°E
130°E
125°E
45°N
40°N
35°N
30°N
25°N

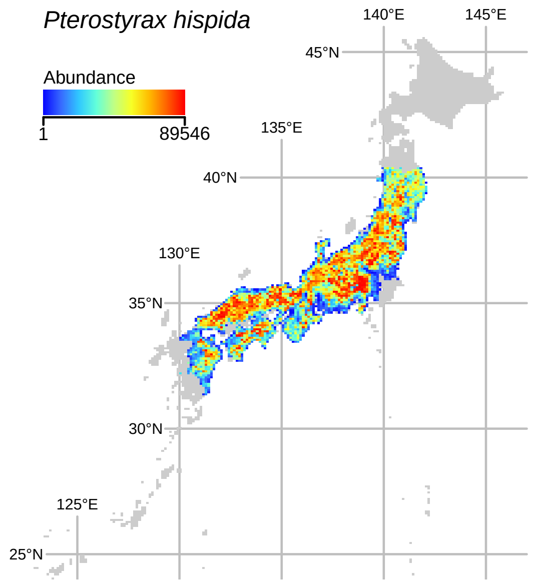
Pterostyrax hispida
Abundance
1
89546
140°E
145°E
45°N
135°E
40°N
130°E
35°N
30°N
125°E
25°N

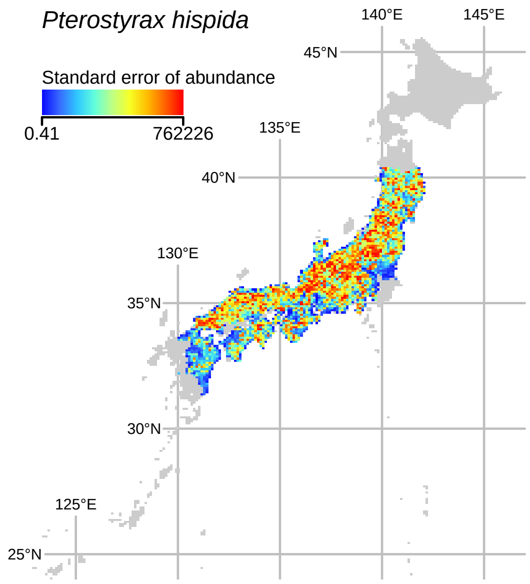
Pterostyrax hispida
Standard error of abundance
0.41
762226
140°E
145°E
45°N
135°E
40°N
130°E
35°N
30°N
125°E
25°N

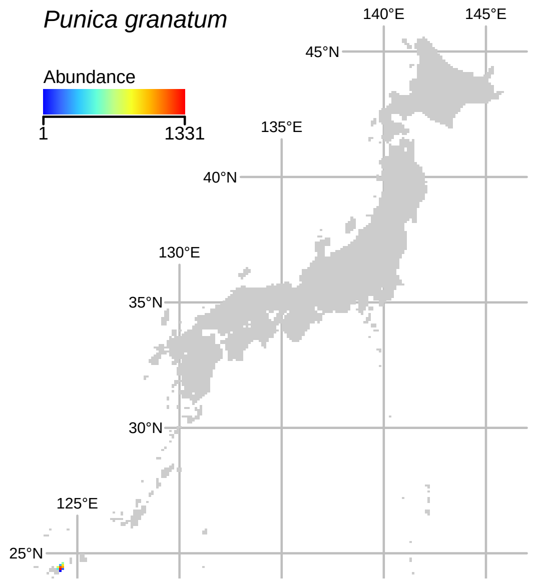
Punica granatum
Abundance
1
1331
140°E
145°E
45°N
135°E
40°N
130°E
35°N
30°N
125°E
25°N

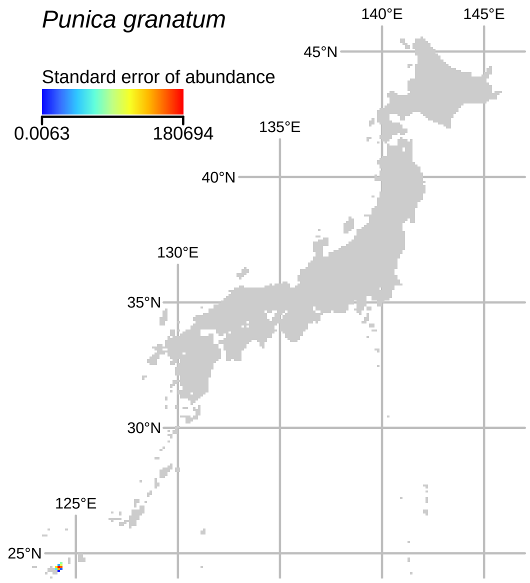
Punica granatum
Standard error of abundance
0.0063
180694
140°E
145°E
45°N
135°E
40°N
130°E
35°N
30°N
125°E
25°N

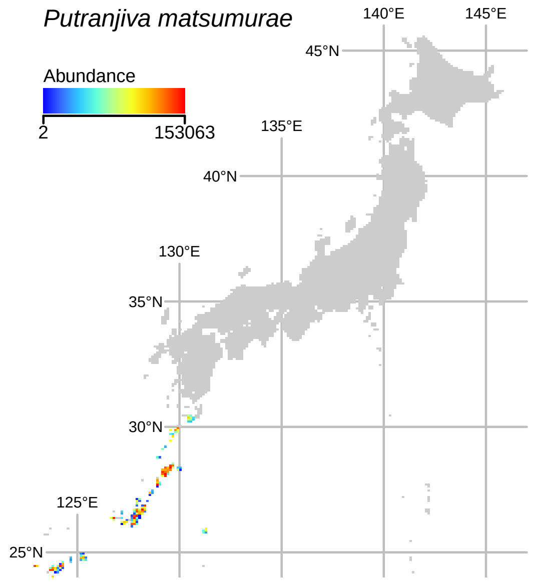
Putranjiva matsumurae
Abundance
2
153063
140°E
145°E
45°N
135°E
40°N
130°E
35°N
30°N
125°E
25°N

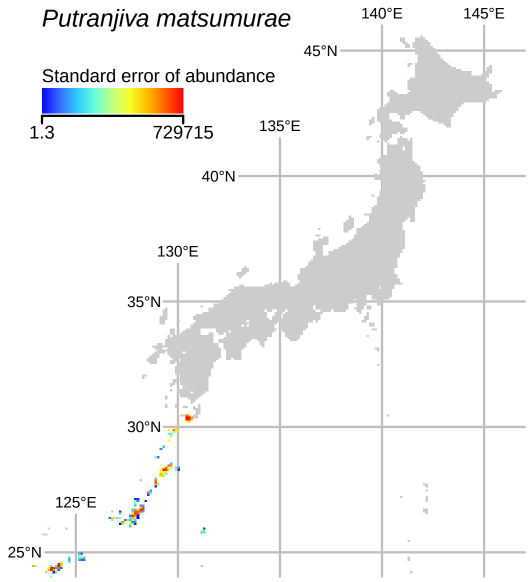
Putranjiva matsumurae
Standard error of abundance
1.3
729715
140°E
145°E
45°N
135°E
40°N
130°E
35°N
30°N
125°E
25°N

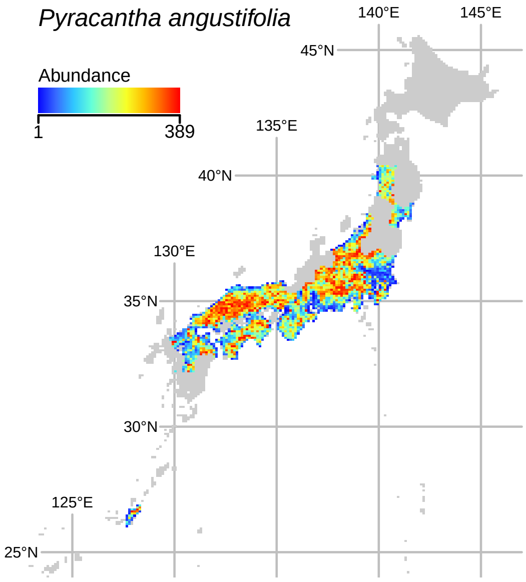
Pyracantha angustifolia
Abundance
1
389
140°E
145°E
45°N
135°E
40°N
130°E
35°N
30°N
125°E
25°N

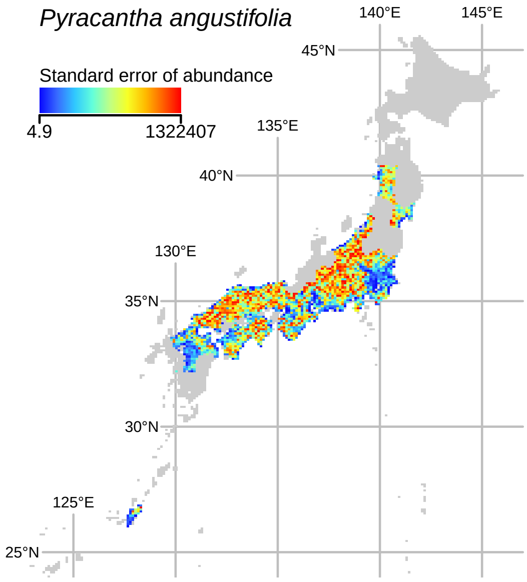
Pyracantha angustifolia
Standard error of abundance
4.9
1322407
140°E
145°E
45°N
135°E
40°N
130°E
35°N
30°N
125°E
25°N

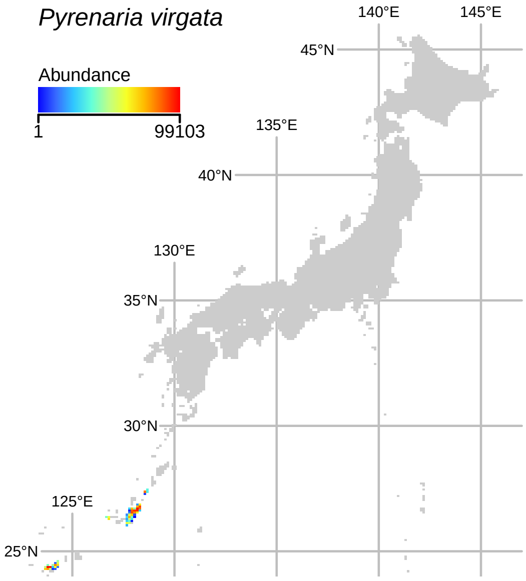
Pyrenaria virgata
Abundance
1
99103
140°E
145°E
45°N
135°E
40°N
130°E
35°N
30°N
125°E
25°N

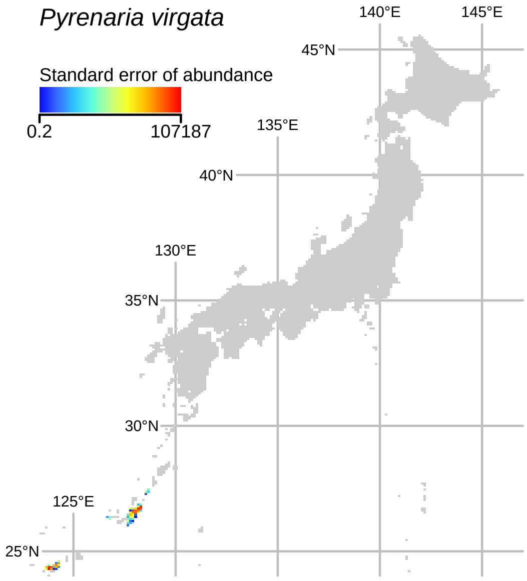
Pyrenaria virgata
Standard error of abundance
0.2
107187
140°E
145°E
45°N
135°E
40°N
130°E
35°N
30°N
125°E
25°N

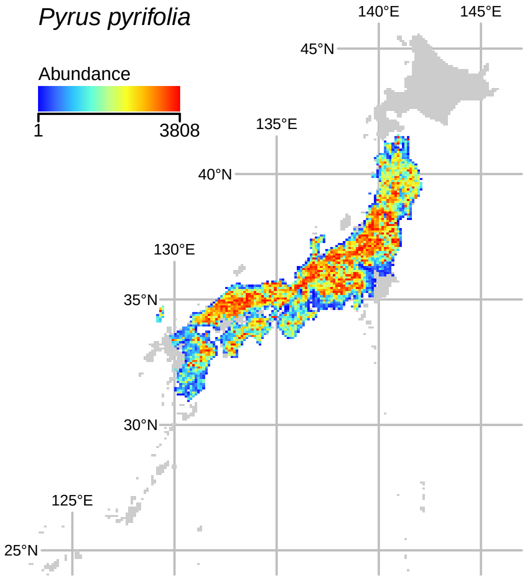
Pyrus pyrifolia
Abundance
1
3808
140°E
145°E
45°N
135°E
40°N
130°E
35°N
30°N
125°E
25°N

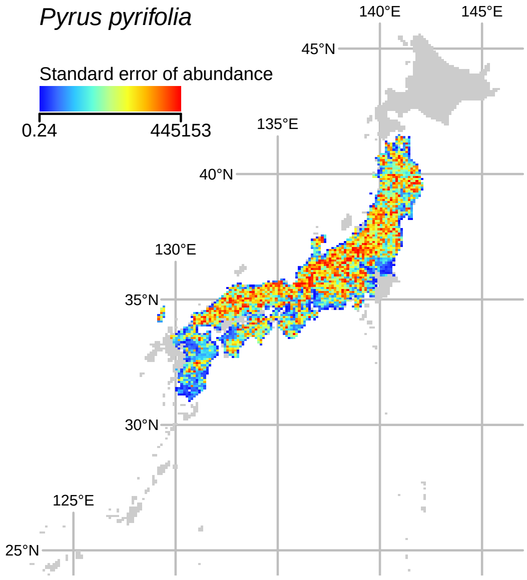
Pyrus pyrifolia
Standard error of abundance
0.24
445153
140°E
145°E
45°N
135°E
40°N
130°E
35°N
30°N
125°E
25°N

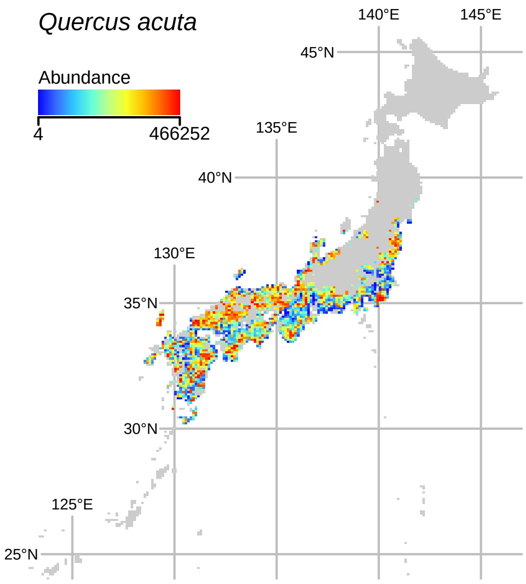
Quercus acuta
Abundance
4
466252
140°E
145°E
45°N
135°E
40°N
130°E
35°N
30°N
125°E
25°N

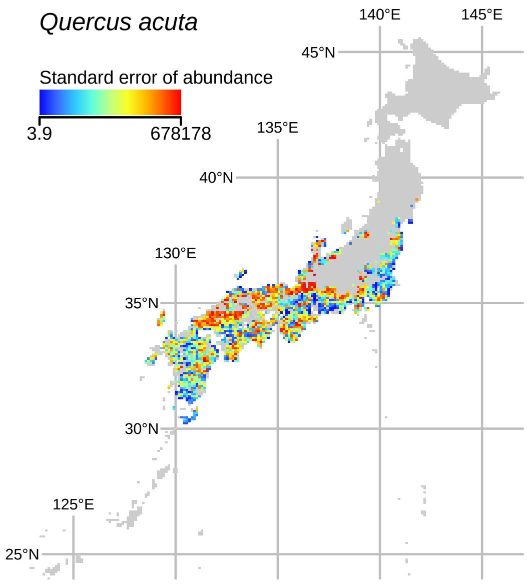
Quercus acuta
Standard error of abundance
3.9
678178
140°E
145°E
45°N
135°E
40°N
130°E
35°N
30°N
125°E
25°N

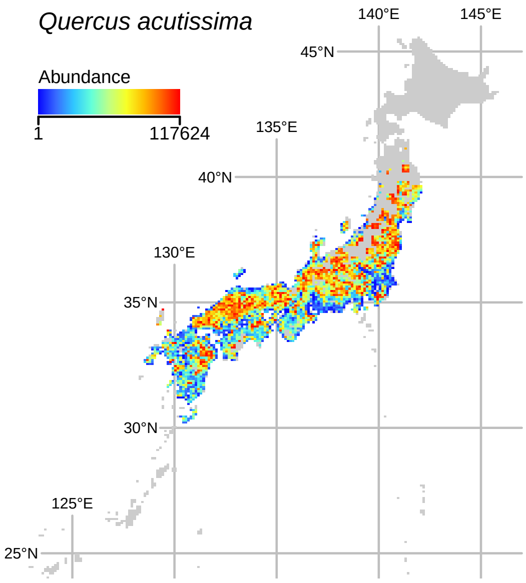
Quercus acutissima
Abundance
1
117624
140°E
145°E
45°N
135°E
40°N
130°E
35°N
30°N
125°E
25°N

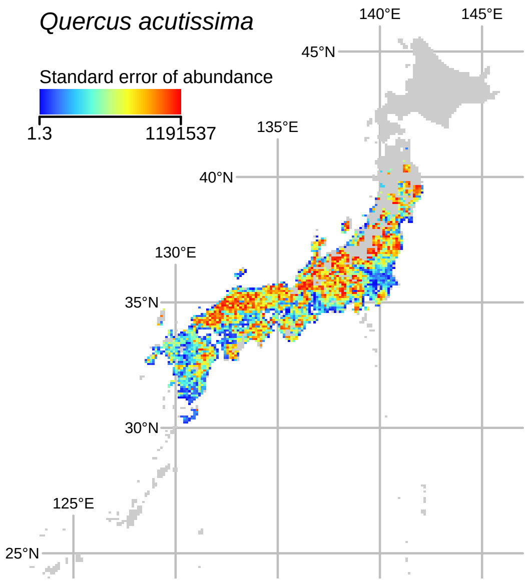
Quercus acutissima
Standard error of abundance
1.3
1191537
140°E
145°E
45°N
135°E
40°N
130°E
35°N
30°N
125°E
25°N

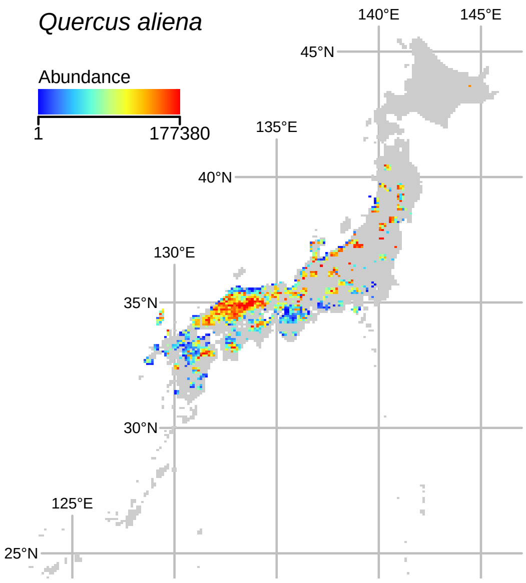
Quercus aliena
Abundance
1
177380
140°E
145°E
45°N
135°E
40°N
130°E
35°N
30°N
125°E
25°N

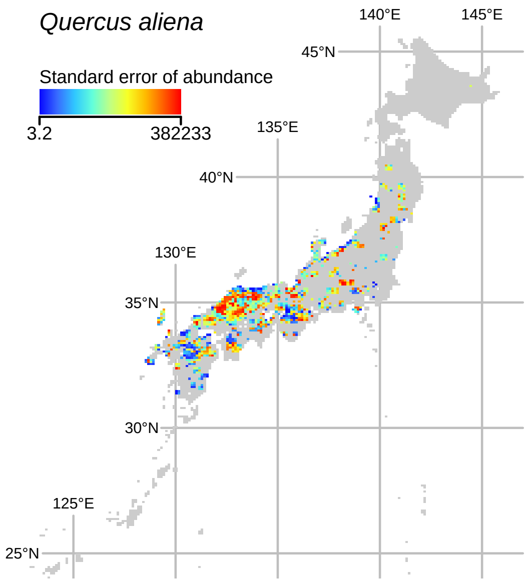
Quercus aliena
Standard error of abundance
3.2
382233
140°E
145°E
45°N
135°E
40°N
130°E
35°N
30°N
125°E
25°N

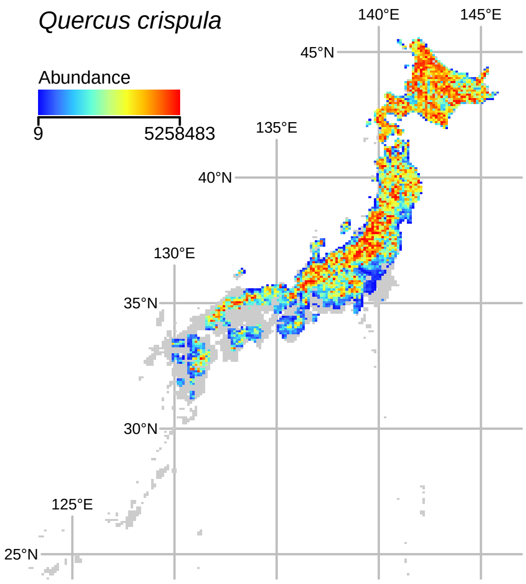
Quercus crispula
Abundance
9
5258483
140°E
145°E
135°E
130°E
125°E
45°N
40°N
35°N
30°N
25°N

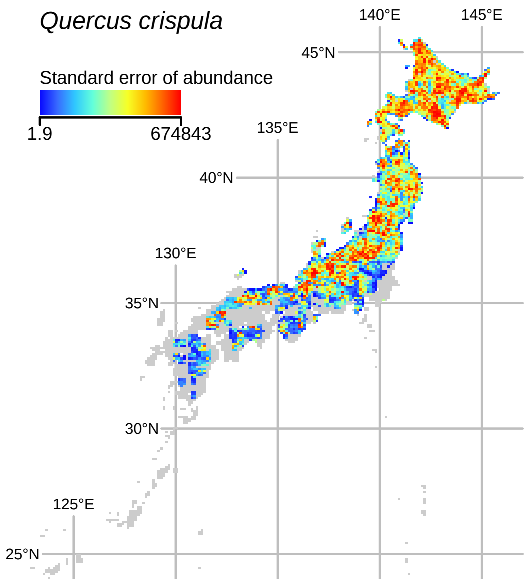
Quercus crispula
Standard error of abundance
1.9
674843
140°E
145°E
135°E
130°E
125°E
45°N
40°N
35°N
30°N
25°N

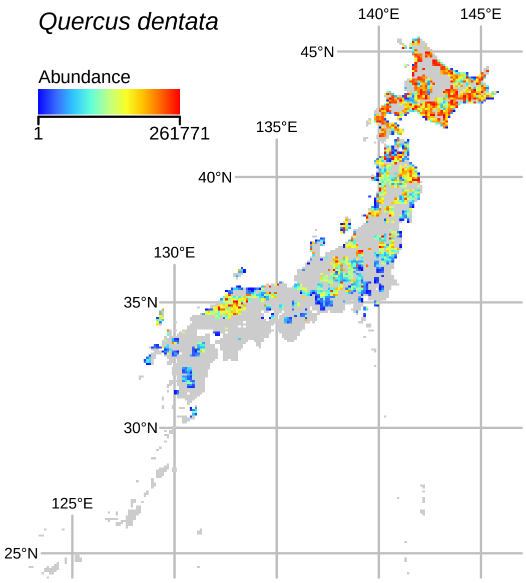
Quercus dentata
Abundance
1
261771
140°E
145°E
135°E
130°E
125°E
45°N
40°N
35°N
30°N
25°N

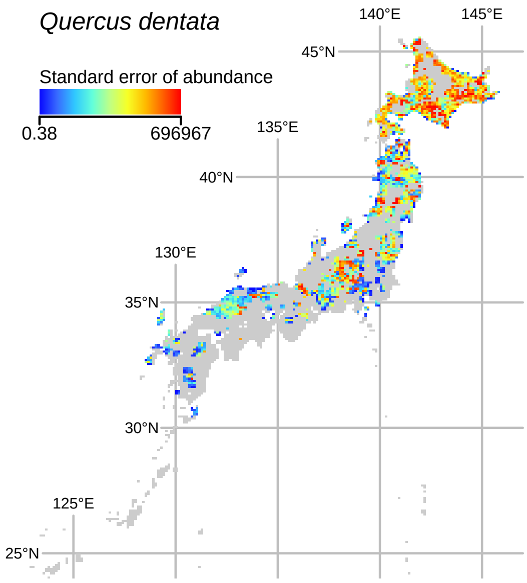
Quercus dentata
Standard error of abundance
0.38
696967
140°E
145°E
135°E
130°E
125°E
45°N
40°N
35°N
30°N
25°N

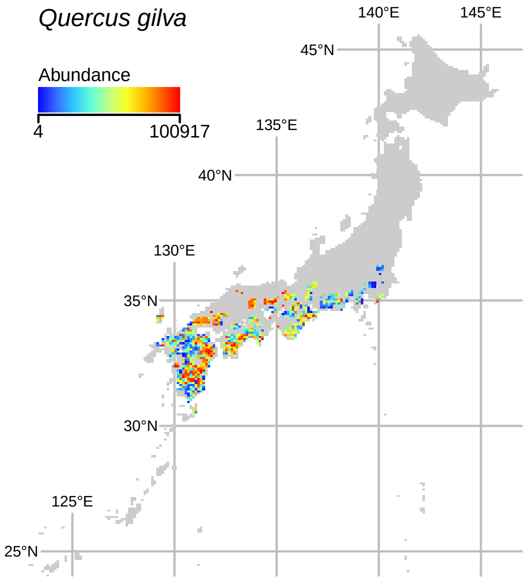
Quercus gilva
Abundance
4
100917
140°E
145°E
135°E
130°E
125°E
45°N
40°N
35°N
30°N
25°N

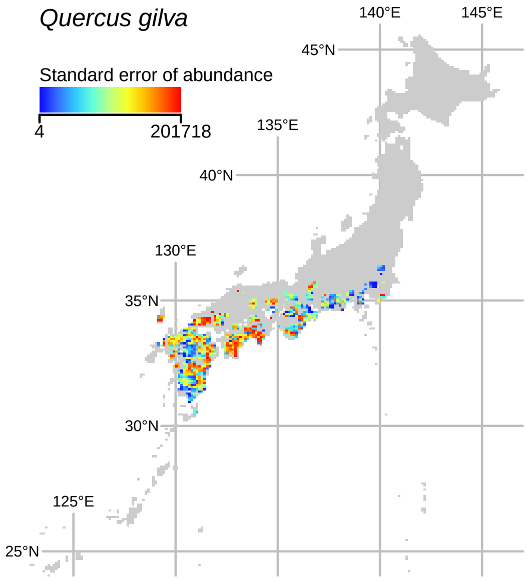
Quercus gilva
Standard error of abundance
4
201718
140°E
145°E
135°E
130°E
125°E
45°N
40°N
35°N
30°N
25°N

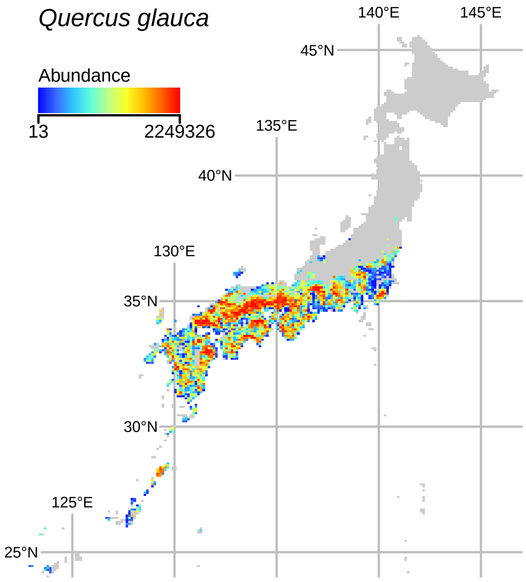
Quercus glauca
Abundance
13
2249326
140°E
145°E
135°E
130°E
125°E
45°N
40°N
35°N
30°N
25°N

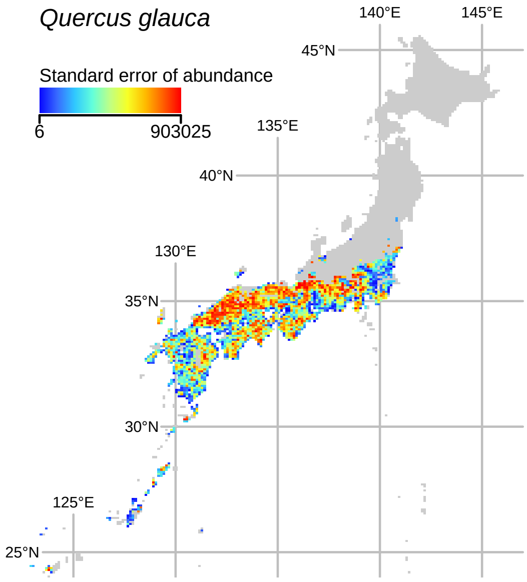
Quercus glauca
Standard error of abundance
6
903025
140°E
145°E
135°E
130°E
125°E
45°N
40°N
35°N
30°N
25°N

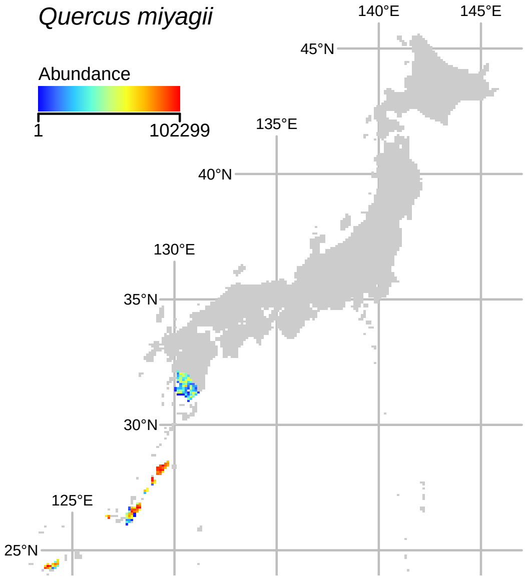
Quercus miyagii
Abundance
1
102299
140°E
145°E
135°E
130°E
125°E
45°N
40°N
35°N
30°N
25°N

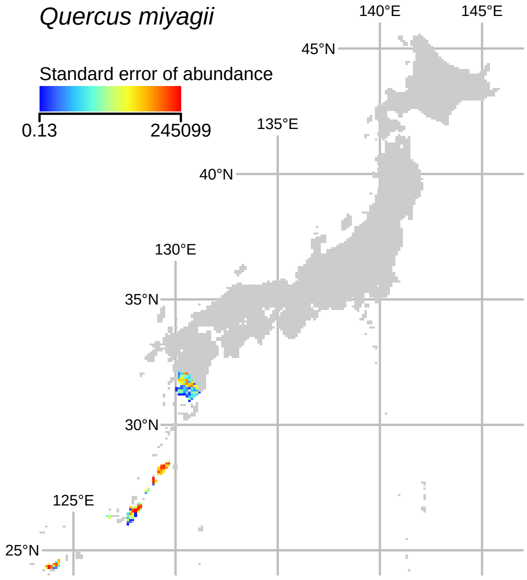
Quercus miyagii
Standard error of abundance
0.13
245099
140°E
145°E
135°E
130°E
125°E
45°N
40°N
35°N
30°N
25°N

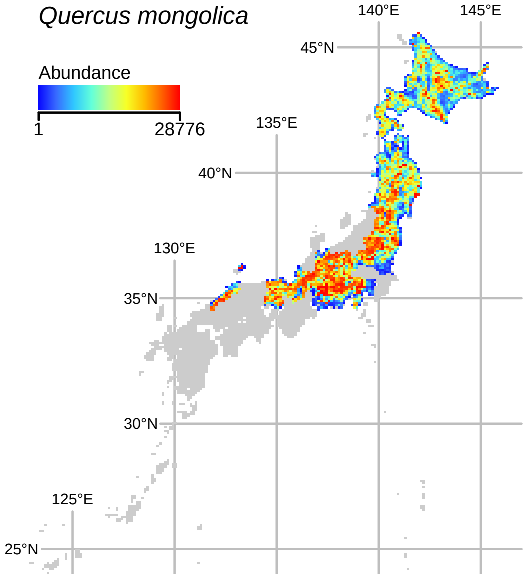
Quercus mongolica
Abundance
1
28776
140°E
145°E
135°E
130°E
125°E
45°N
40°N
35°N
30°N
25°N

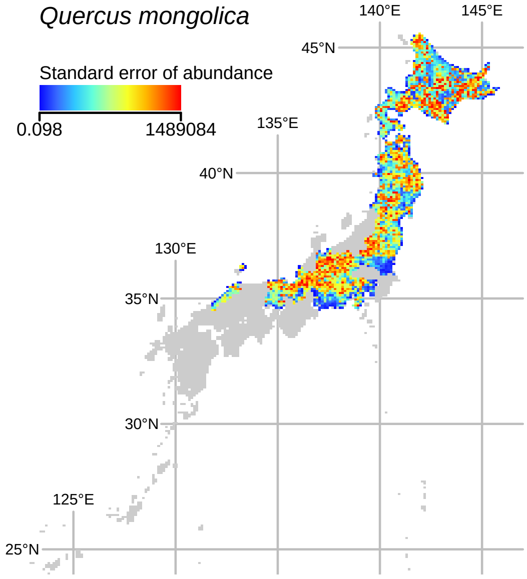
Quercus mongolica
Standard error of abundance
0.098
1489084
140°E
145°E
135°E
130°E
125°E
45°N
40°N
35°N
30°N
25°N

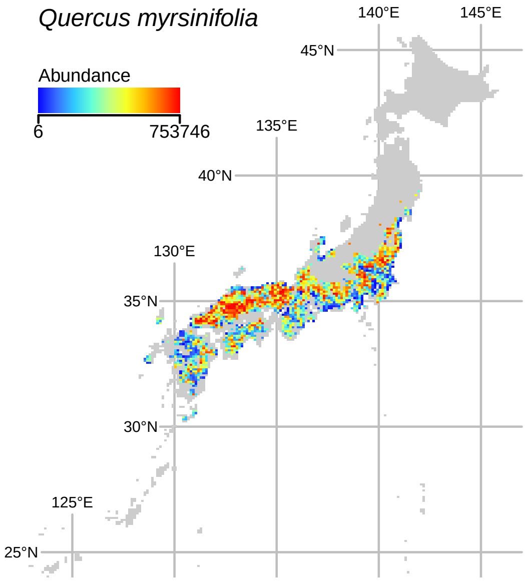
Quercus myrsinifolia
Abundance
6
753746
140°E
145°E
45°N
135°E
40°N
130°E
35°N
30°N
125°E
25°N

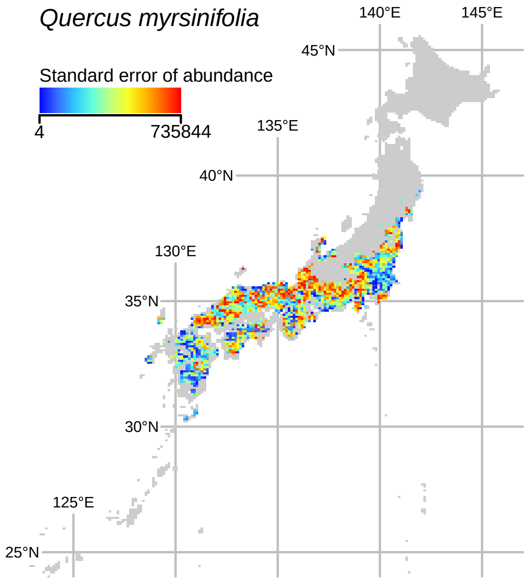
Quercus myrsinifolia
Standard error of abundance
4
735844
140°E
145°E
45°N
135°E
40°N
130°E
35°N
30°N
125°E
25°N

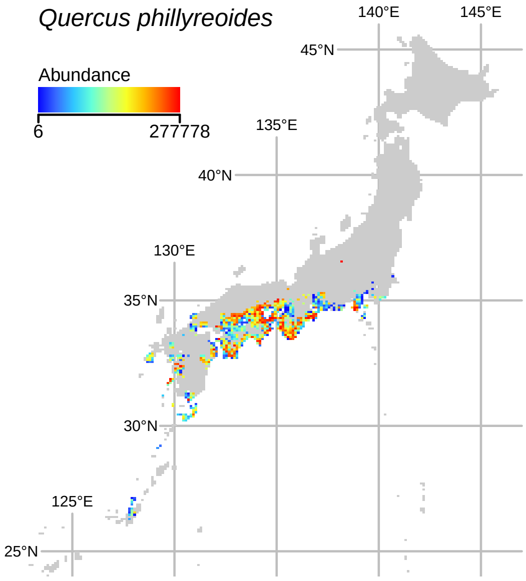
Quercus phillyreoides
Abundance
6
277778
140°E
145°E
45°N
135°E
40°N
130°E
35°N
30°N
125°E
25°N

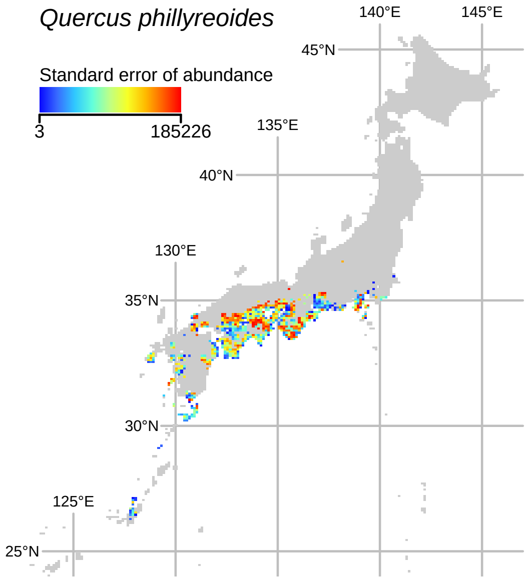
Quercus phillyreoides
Standard error of abundance
3
185226
140°E
145°E
45°N
135°E
40°N
130°E
35°N
30°N
125°E
25°N

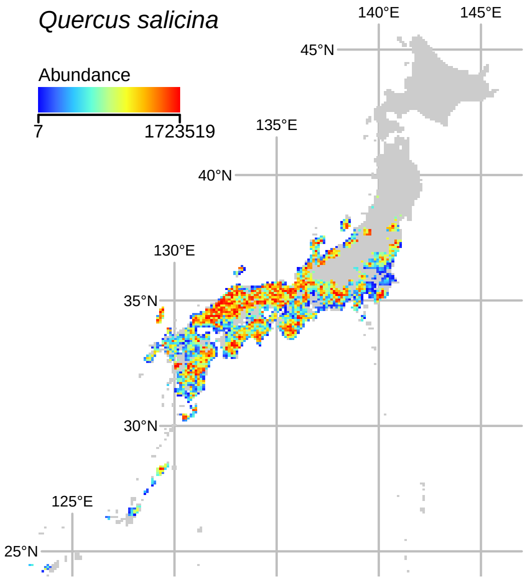
Quercus salicina
Abundance
7
1723519
140°E
145°E
45°N
135°E
40°N
130°E
35°N
30°N
125°E
25°N

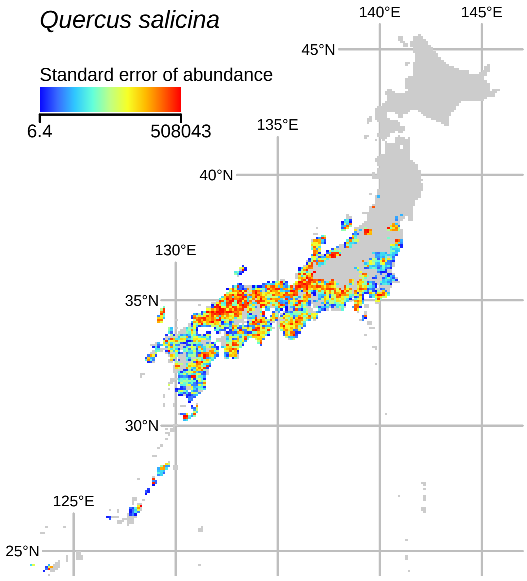
Quercus salicina
Standard error of abundance
6.4
508043
140°E
145°E
45°N
135°E
40°N
130°E
35°N
30°N
125°E
25°N

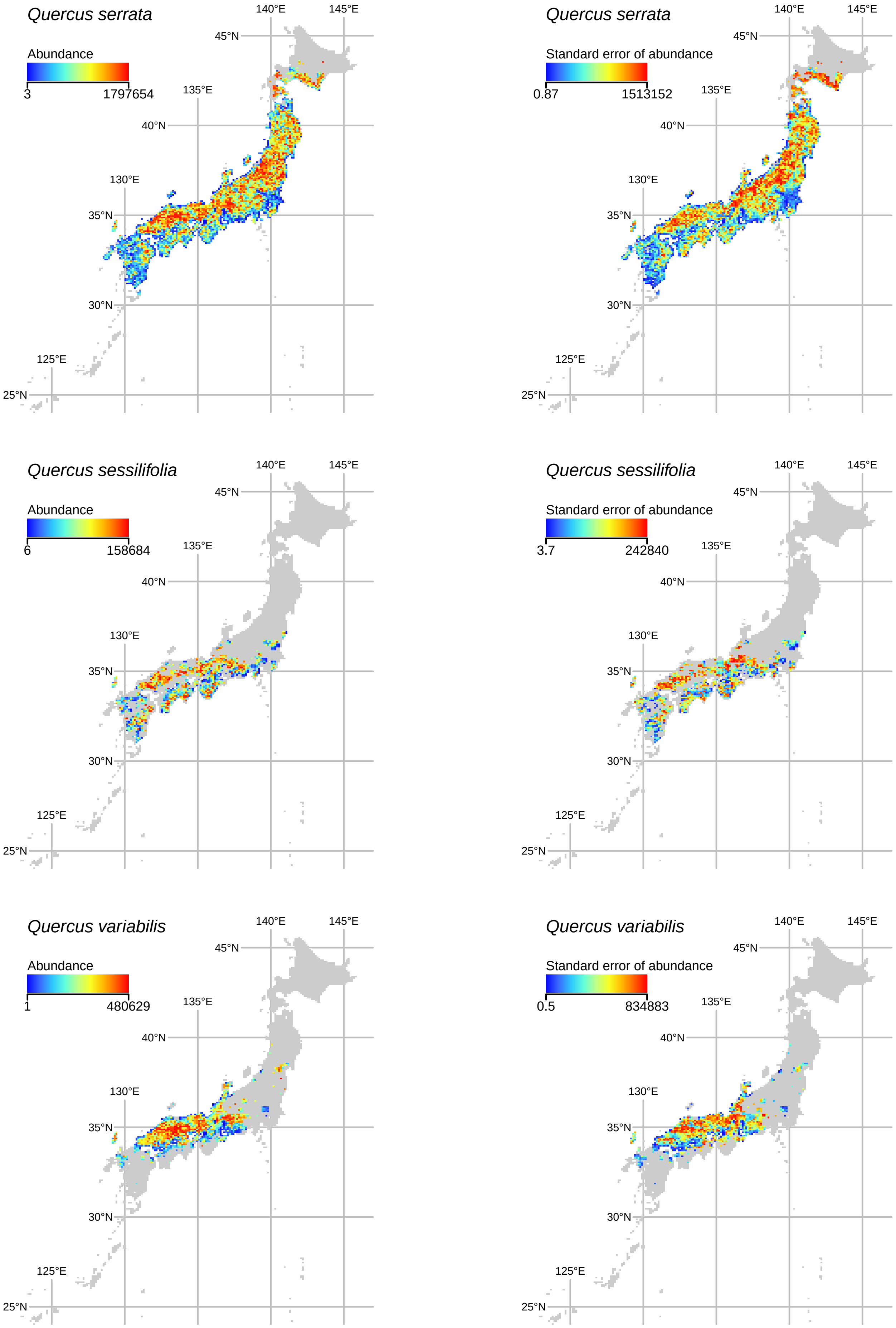
Quercus serrata
Abundance
3
1797654
Quercus serrata
Standard error of abundance
0.87
1513152
Quercus sessilifolia
Abundance
6
158684
Quercus sessilifolia
Standard error of abundance
3.7
242840
Quercus variabilis
Abundance
1
480629
Quercus variabilis
Standard error of abundance
0.5
834883
125°E
130°E
135°E
140°E
145°E
25°N
30°N
35°N
40°N
45°N

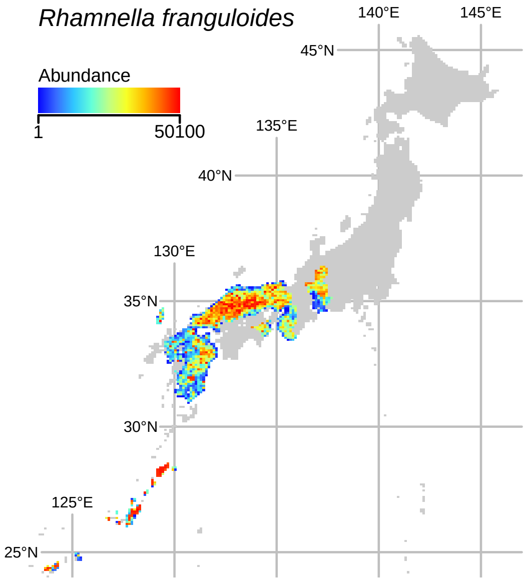
Rhamnella franguloides
Abundance
1
50100
140°E
145°E
45°N
135°E
40°N
130°E
35°N
30°N
125°E
25°N

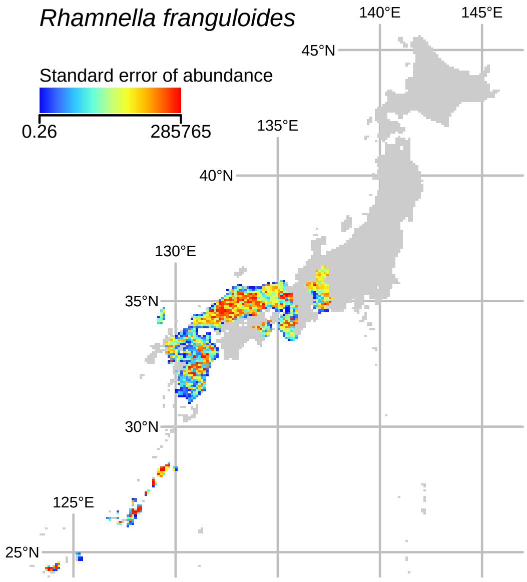
Rhamnella franguloides
Standard error of abundance
0.26
285765
140°E
145°E
45°N
135°E
40°N
130°E
35°N
30°N
125°E
25°N

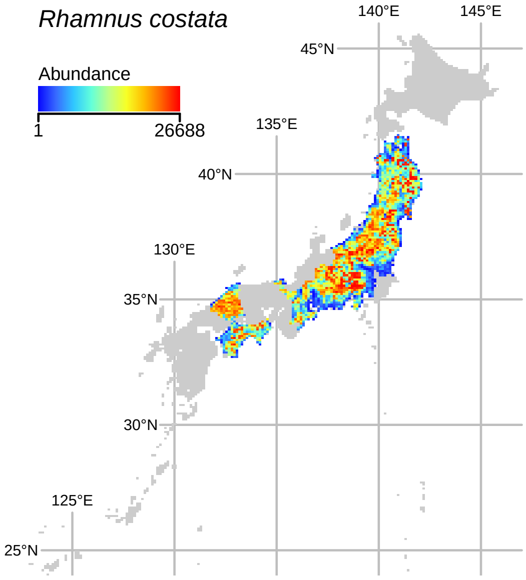
Rhamnus costata
Abundance
1
26688
140°E
145°E
45°N
135°E
40°N
130°E
35°N
30°N
125°E
25°N

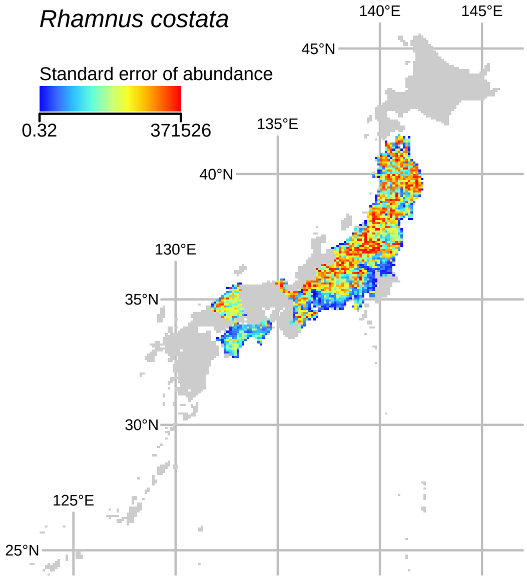
Rhamnus costata
Standard error of abundance
0.32
371526
140°E
145°E
45°N
135°E
40°N
130°E
35°N
30°N
125°E
25°N

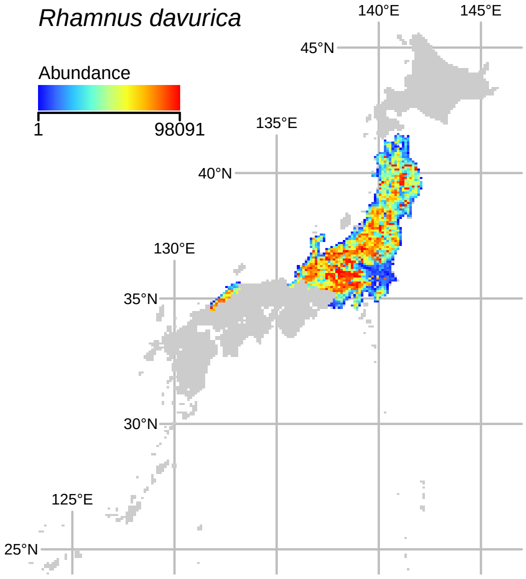
Rhamnus davurica
Abundance
1
98091
140°E
145°E
45°N
135°E
40°N
130°E
35°N
30°N
125°E
25°N

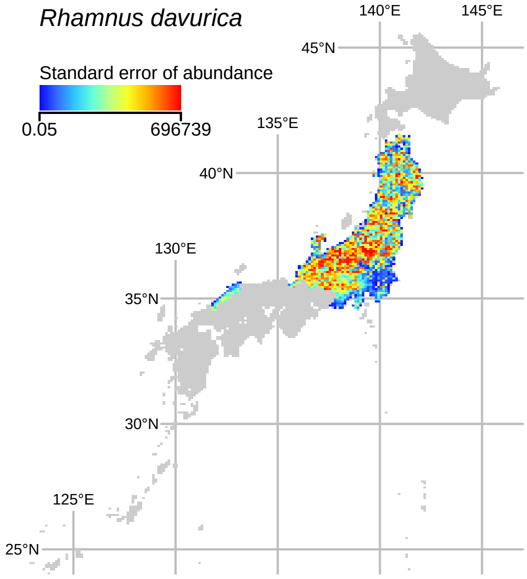
Rhamnus davurica
Standard error of abundance
0.05
696739
140°E
145°E
45°N
135°E
40°N
130°E
35°N
30°N
125°E
25°N

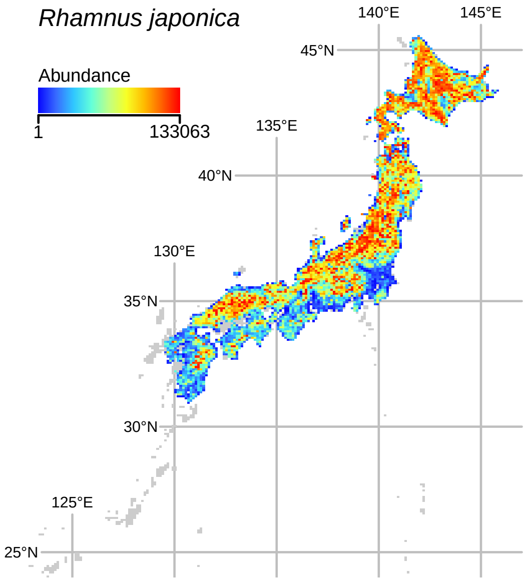
Rhamnus japonica
Abundance
1
133063
140°E
145°E
45°N
135°E
40°N
130°E
35°N
30°N
125°E
25°N

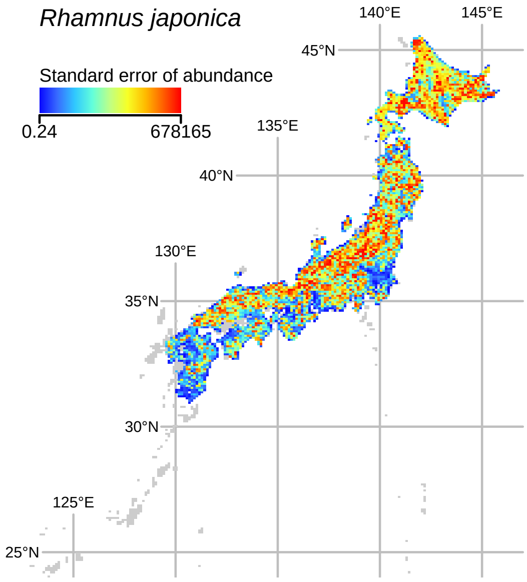
Rhamnus japonica
Standard error of abundance
0.24
678165
140°E
145°E
45°N
135°E
40°N
130°E
35°N
30°N
125°E
25°N

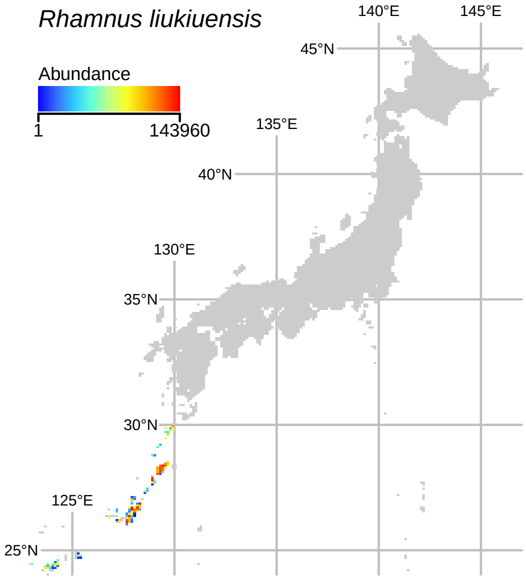
Rhamnus liukiuensis
Abundance
1
143960
140°E
145°E
45°N
135°E
40°N
130°E
35°N
30°N
125°E
25°N

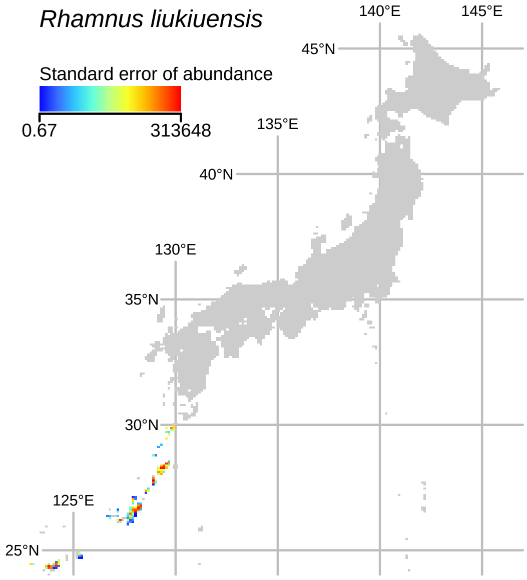
Rhamnus liukiuensis
Standard error of abundance
0.67
313648
140°E
145°E
45°N
135°E
40°N
130°E
35°N
30°N
125°E
25°N

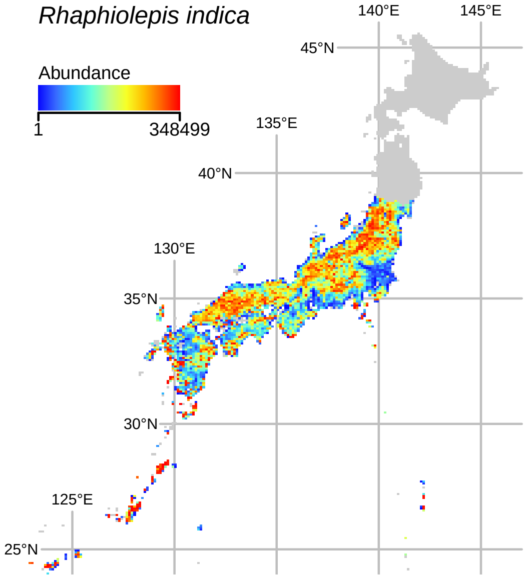
Rhaphiolepis indica
Abundance
1
348499
140°E
145°E
45°N
135°E
40°N
130°E
35°N
30°N
125°E
25°N

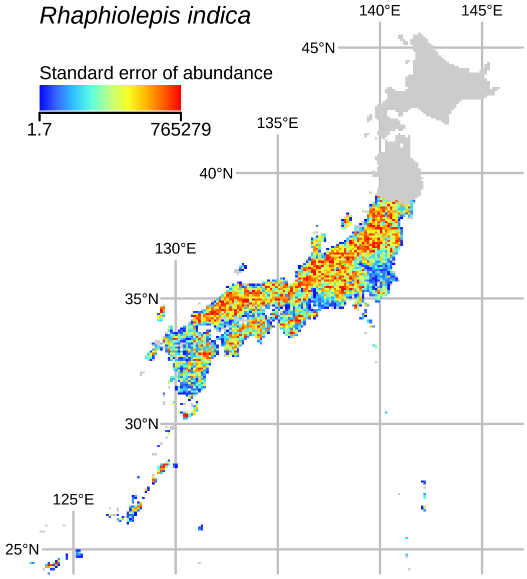
Rhaphiolepis indica
Standard error of abundance
1.7
765279
140°E
145°E
45°N
135°E
40°N
130°E
35°N
30°N
125°E
25°N

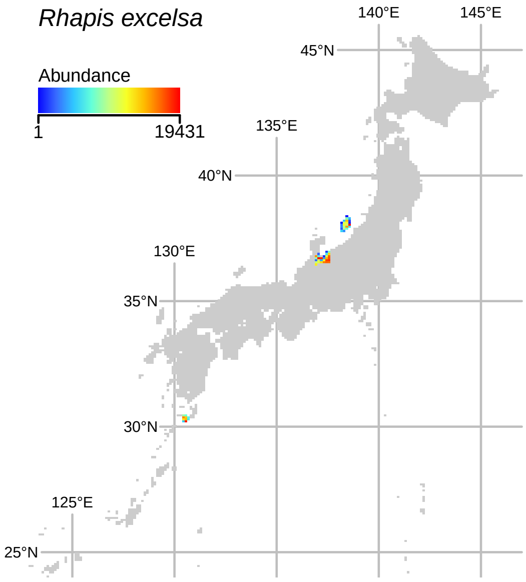
Rhapis excelsa
Abundance
1
19431
140°E
145°E
135°E
130°E
125°E
45°N
40°N
35°N
30°N
25°N

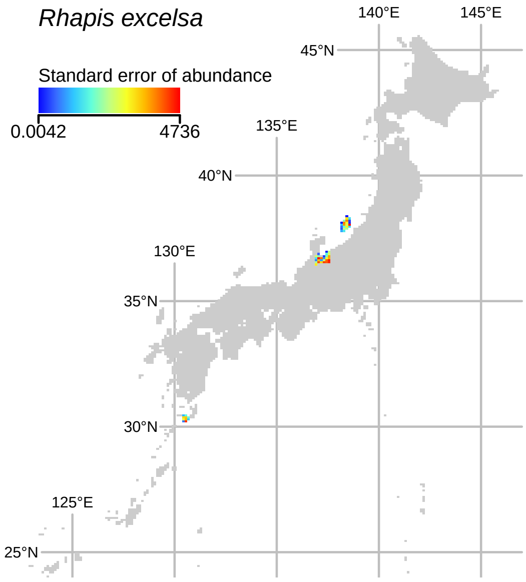
Rhapis excelsa
Standard error of abundance
0.0042
4736
140°E
145°E
135°E
130°E
125°E
45°N
40°N
35°N
30°N
25°N

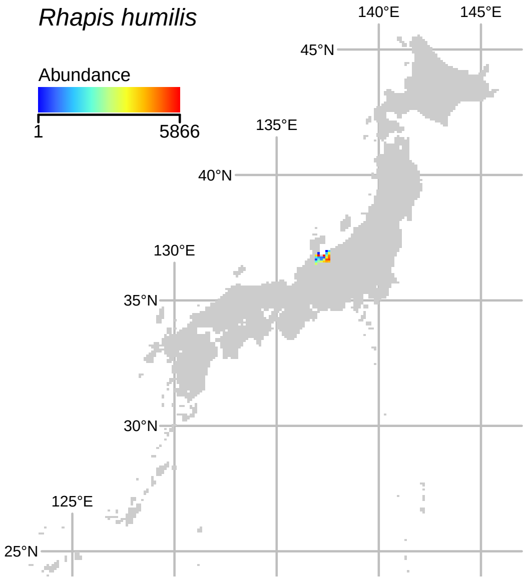
Rhapis humilis
Abundance
1
5866
140°E
145°E
135°E
130°E
125°E
45°N
40°N
35°N
30°N
25°N

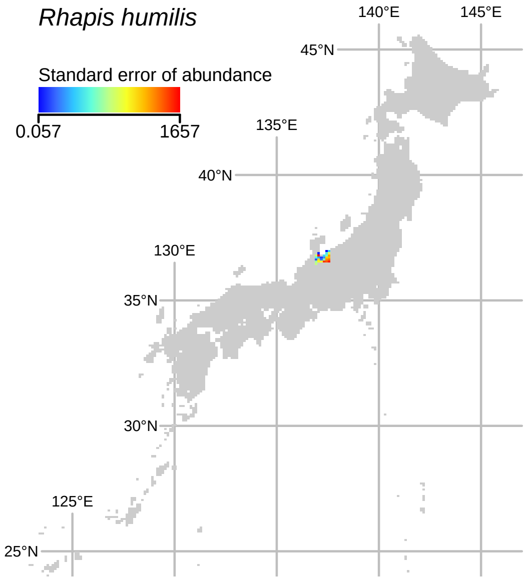
Rhapis humilis
Standard error of abundance
0.057
1657
140°E
145°E
135°E
130°E
125°E
45°N
40°N
35°N
30°N
25°N

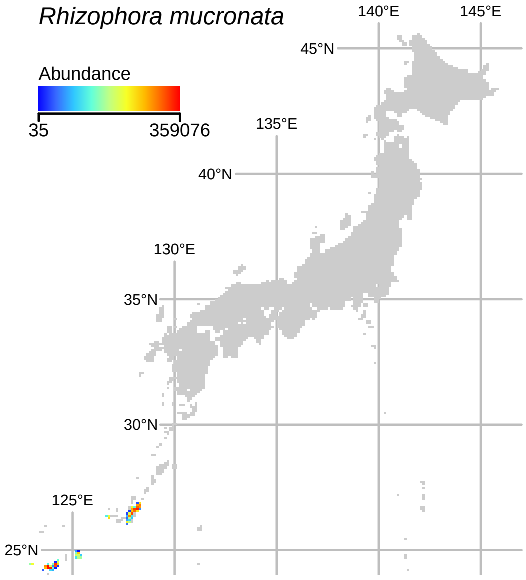
Rhizophora mucronata
Abundance
35
359076
140°E
145°E
135°E
130°E
125°E
45°N
40°N
35°N
30°N
25°N

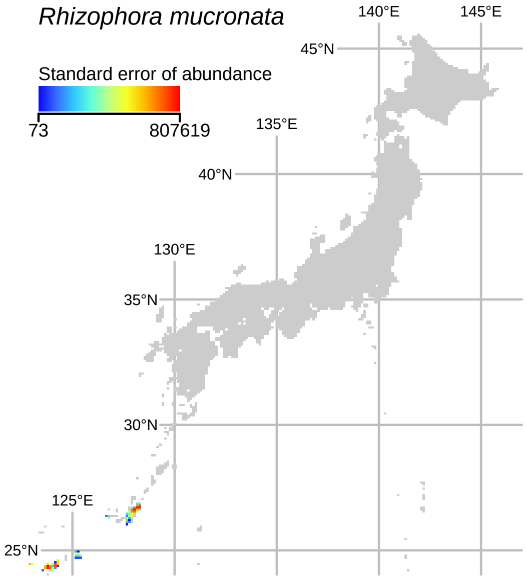
Rhizophora mucronata
Standard error of abundance
73
807619
140°E
145°E
135°E
130°E
125°E
45°N
40°N
35°N
30°N
25°N

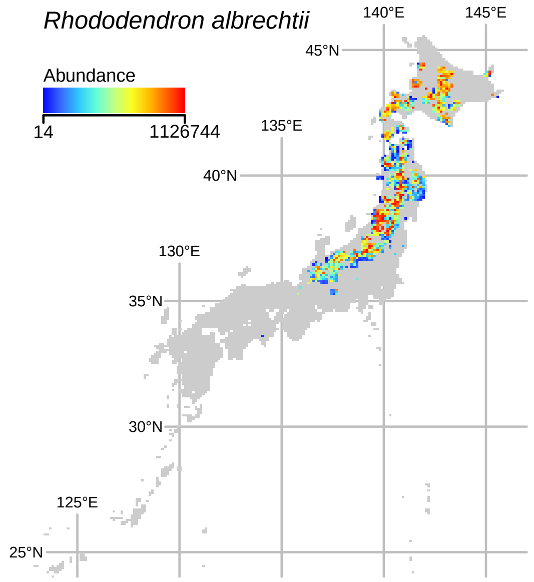
Rhododendron albrechtii
Abundance
14
1126744
140°E
145°E
135°E
130°E
125°E
45°N
40°N
35°N
30°N
25°N

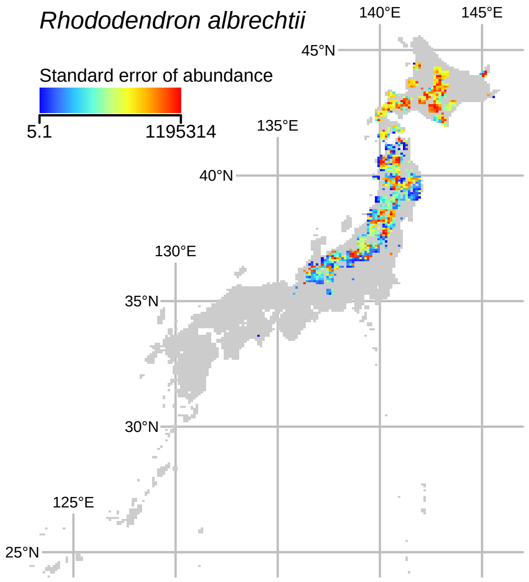
Rhododendron albrechtii
Standard error of abundance
5.1
1195314
140°E
145°E
135°E
130°E
125°E
45°N
40°N
35°N
30°N
25°N

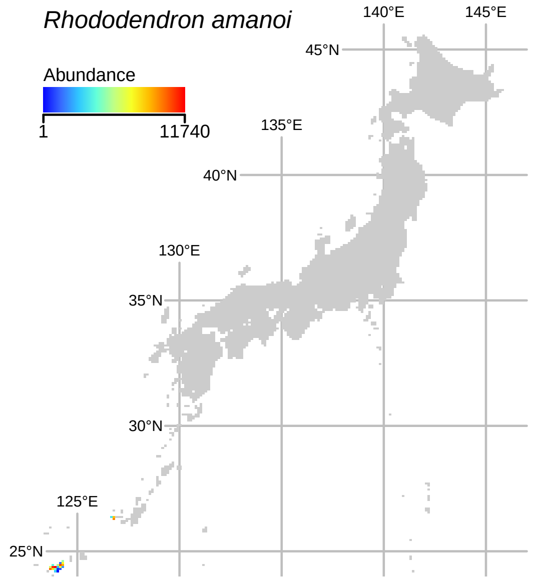
Rhododendron amanoi
Abundance
1
11740
140°E
145°E
135°E
130°E
125°E
45°N
40°N
35°N
30°N
25°N

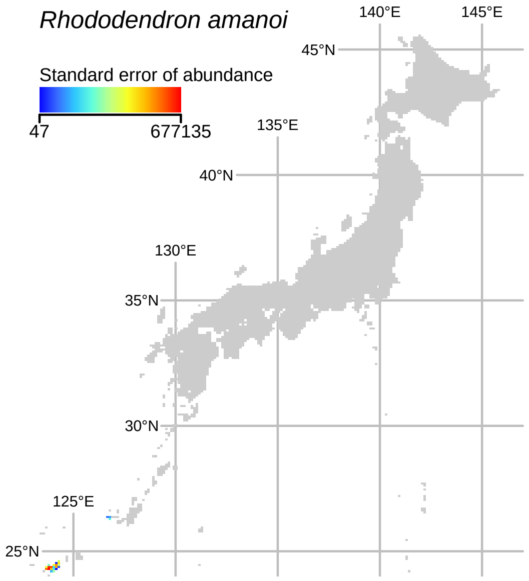
Rhododendron amanoi
Standard error of abundance
47
677135
140°E
145°E
135°E
130°E
125°E
45°N
40°N
35°N
30°N
25°N

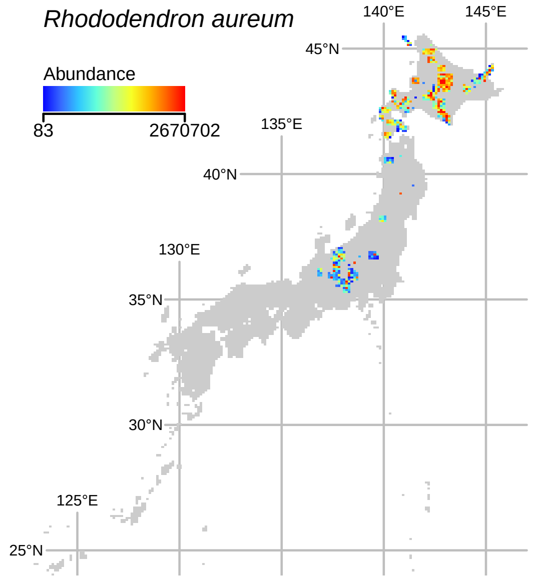
Rhododendron aureum
Abundance
83
2670702
140°E
145°E
135°E
130°E
125°E
45°N
40°N
35°N
30°N
25°N

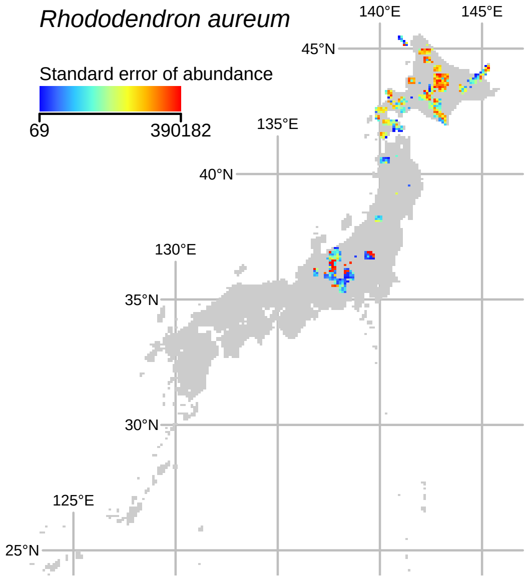
Rhododendron aureum
Standard error of abundance
69
390182
140°E
145°E
135°E
130°E
125°E
45°N
40°N
35°N
30°N
25°N

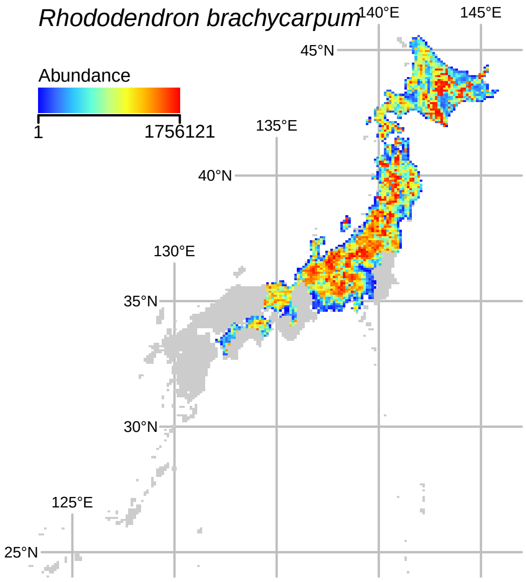
Rhododendron brachycarpum
Abundance
1
1756121
140°E
145°E
135°E
130°E
125°E
45°N
40°N
35°N
30°N
25°N

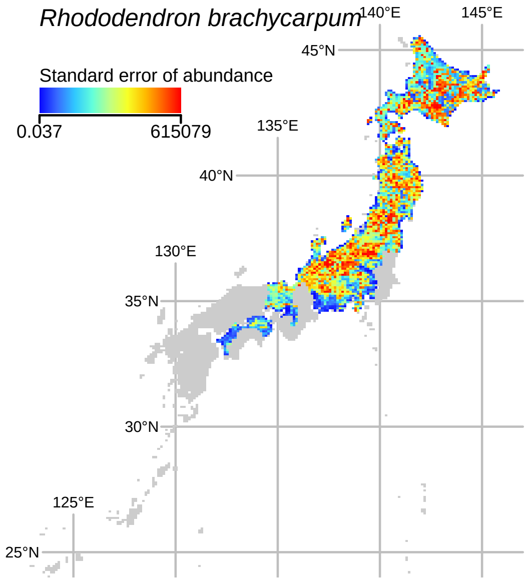
Rhododendron brachycarpum
Standard error of abundance
0.037
615079
140°E
145°E
135°E
130°E
125°E
45°N
40°N
35°N
30°N
25°N

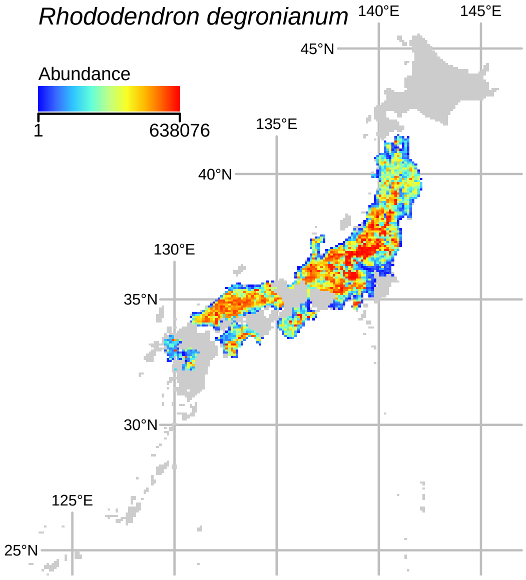
Rhododendron degronianum
Abundance
1
638076
140°E
145°E
135°E
130°E
125°E
45°N
40°N
35°N
30°N
25°N

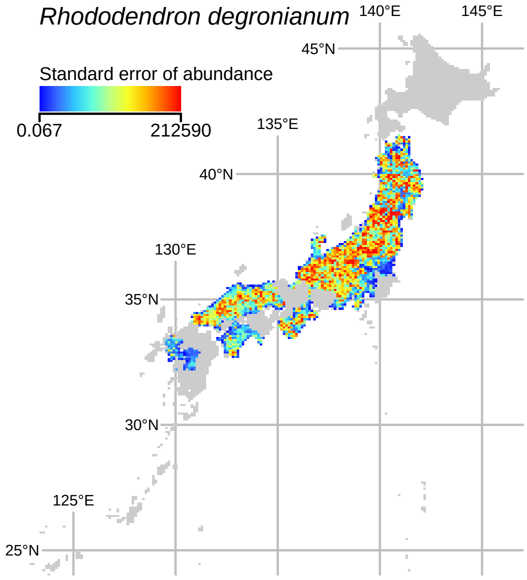
Rhododendron degronianum
Standard error of abundance
0.067
212590
140°E
145°E
135°E
130°E
125°E
45°N
40°N
35°N
30°N
25°N

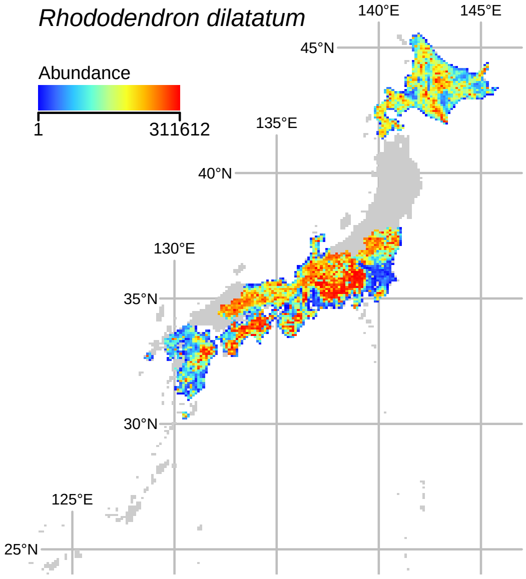
Rhododendron dilatatum
Abundance
1
311612
140°E
145°E
135°E
130°E
125°E
45°N
40°N
35°N
30°N
25°N

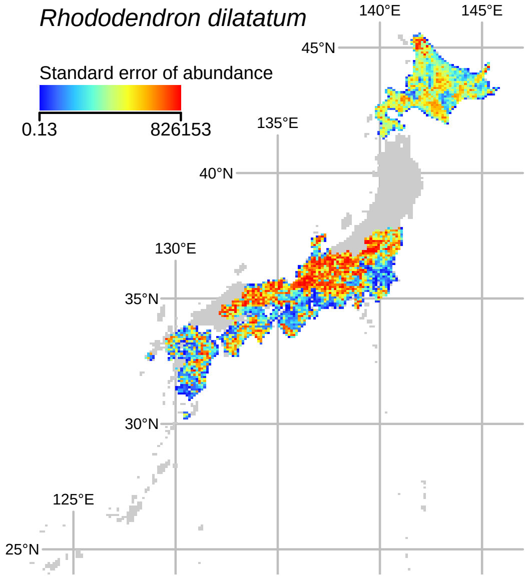
Rhododendron dilatatum
Standard error of abundance
0.13
826153
140°E
145°E
135°E
130°E
125°E
45°N
40°N
35°N
30°N
25°N

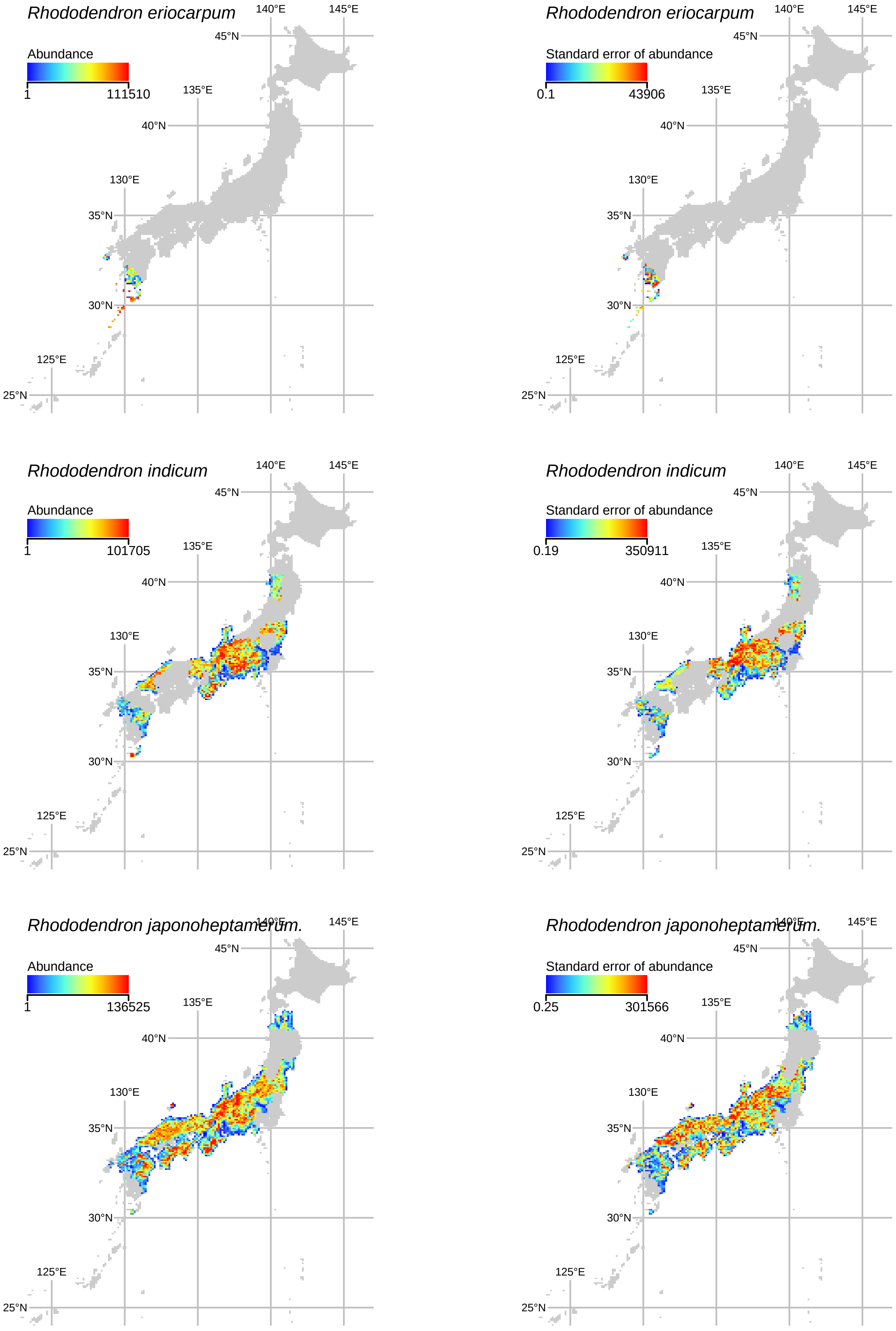
Rhododendron eriocarpum
Abundance
1
111510
Rhododendron eriocarpum
Standard error of abundance
0.1
43906
Rhododendron indicum
Abundance
1
101705
Rhododendron indicum
Standard error of abundance
0.19
350911
Rhododendron japonoheptamerum.
Abundance
1
136525
Rhododendron japonoheptamerum.
Standard error of abundance
0.25
301566
125°E
130°E
135°E
140°E
145°E
25°N
30°N
35°N
40°N
45°N

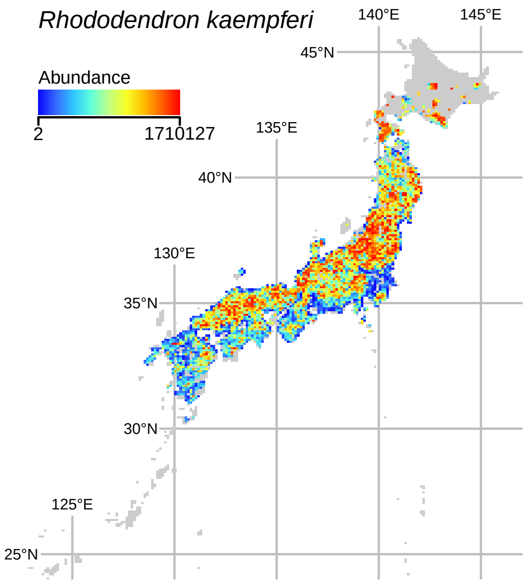
Rhododendron kaempferi
Abundance
2
1710127
140°E
145°E
45°N
135°E
40°N
130°E
35°N
30°N
125°E
25°N

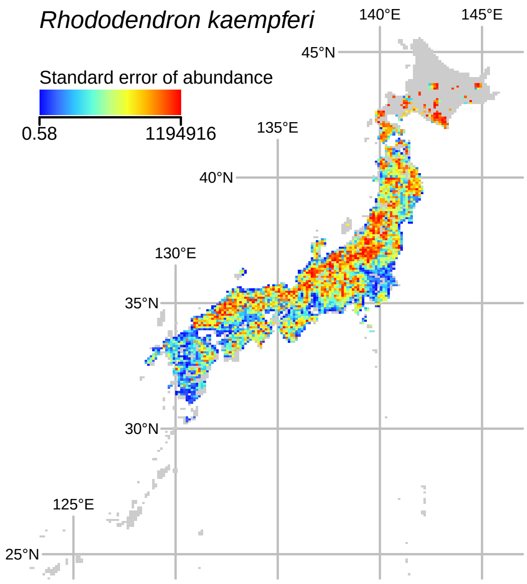
Rhododendron kaempferi
Standard error of abundance
0.58
1194916
140°E
145°E
45°N
135°E
40°N
130°E
35°N
30°N
125°E
25°N

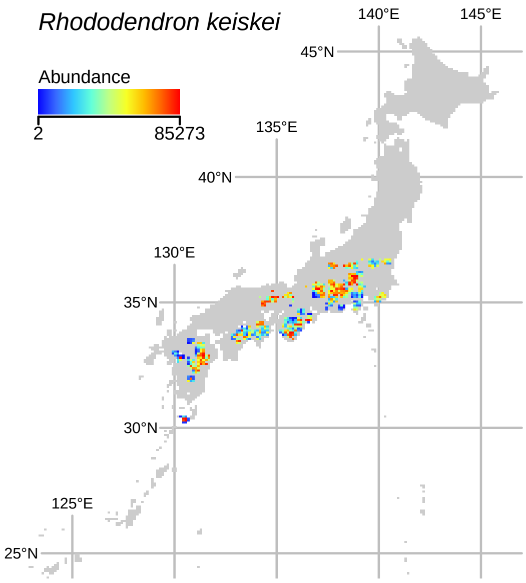
Rhododendron keiskei
Abundance
2
85273
140°E
145°E
45°N
135°E
40°N
130°E
35°N
30°N
125°E
25°N

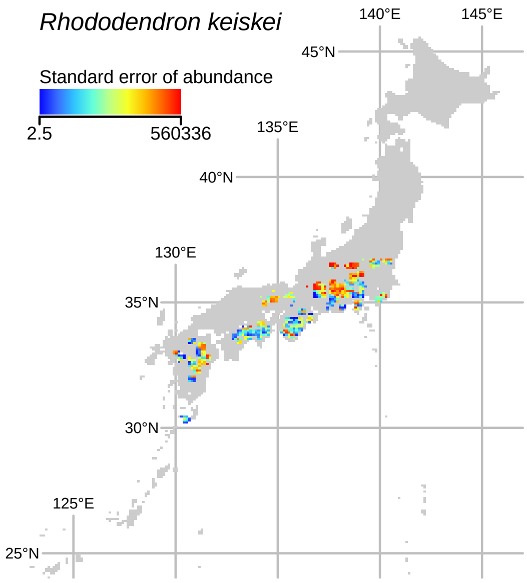
Rhododendron keiskei
Standard error of abundance
2.5
560336
140°E
145°E
45°N
135°E
40°N
130°E
35°N
30°N
125°E
25°N

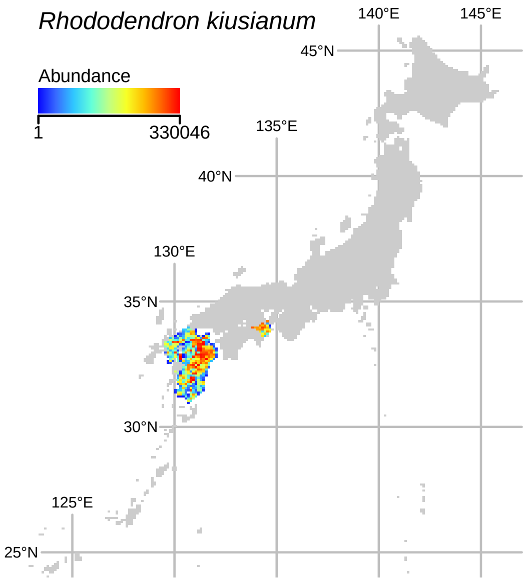
Rhododendron kiusianum
Abundance
1
330046
140°E
145°E
45°N
135°E
40°N
130°E
35°N
30°N
125°E
25°N

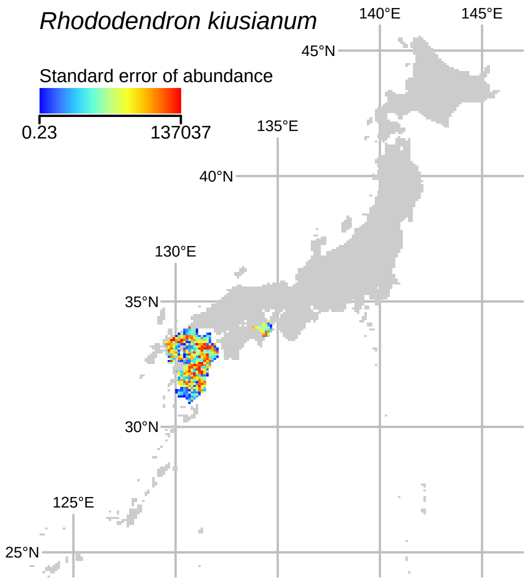
Rhododendron kiusianum
Standard error of abundance
0.23
137037
140°E
145°E
45°N
135°E
40°N
130°E
35°N
30°N
125°E
25°N

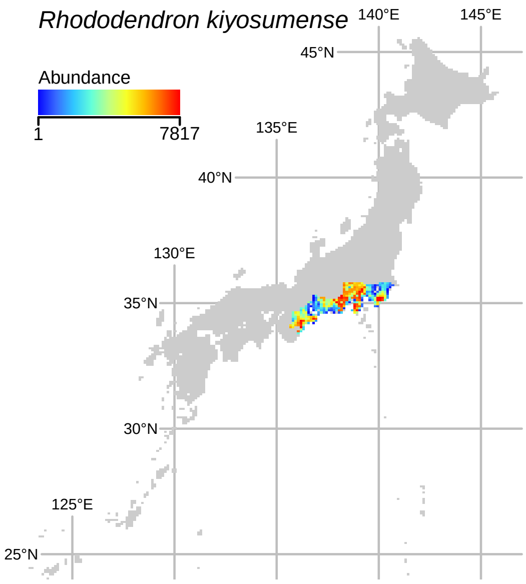
Rhododendron kiyosumense
Abundance
1
7817
140°E
145°E
45°N
135°E
40°N
130°E
35°N
30°N
125°E
25°N

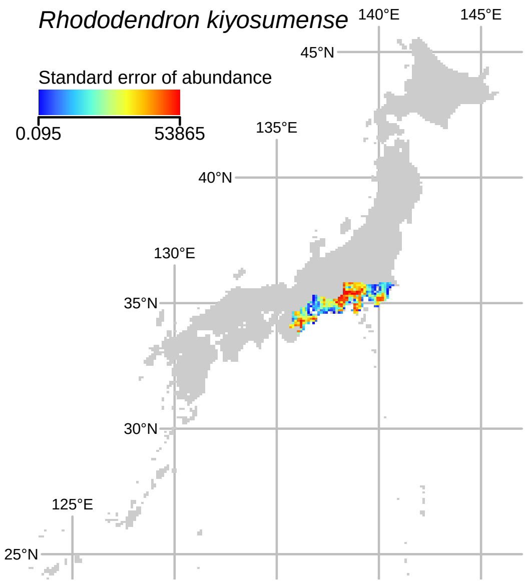
Rhododendron kiyosumense
Standard error of abundance
0.095
53865
140°E
145°E
45°N
135°E
40°N
130°E
35°N
30°N
125°E
25°N

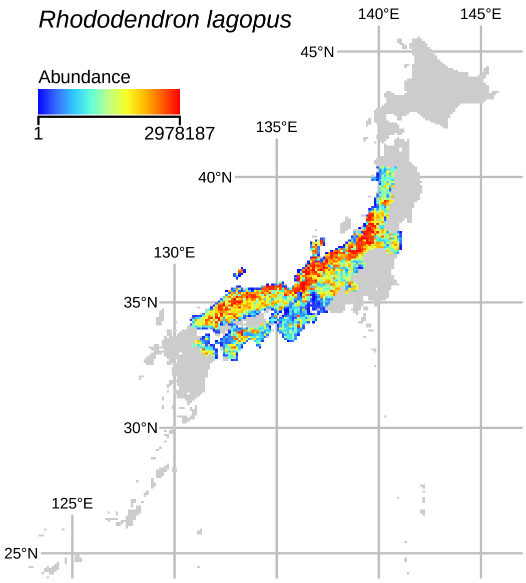
Rhododendron lagopus
Abundance
1
2978187
140°E
145°E
45°N
135°E
40°N
130°E
35°N
30°N
125°E
25°N

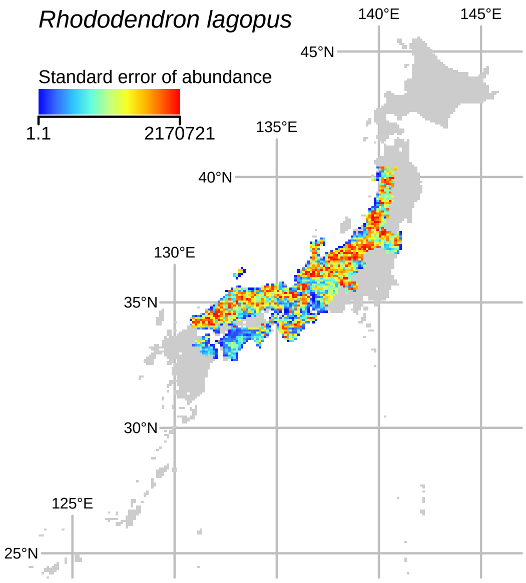
Rhododendron lagopus
Standard error of abundance
1.1
2170721
140°E
145°E
45°N
135°E
40°N
130°E
35°N
30°N
125°E
25°N

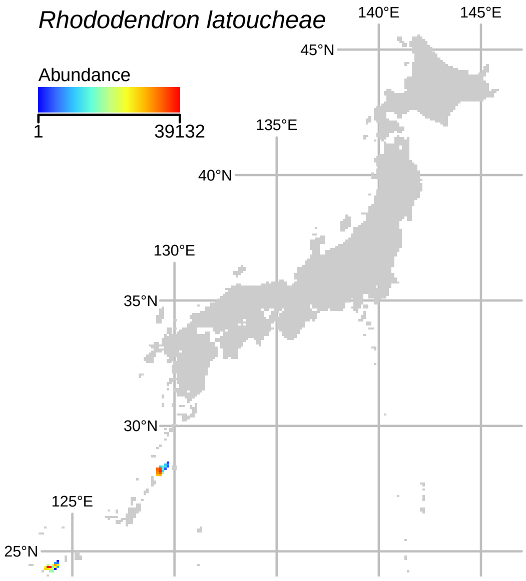
Rhododendron latoucheae
Abundance
1
39132
140°E
145°E
45°N
135°E
40°N
130°E
35°N
30°N
125°E
25°N

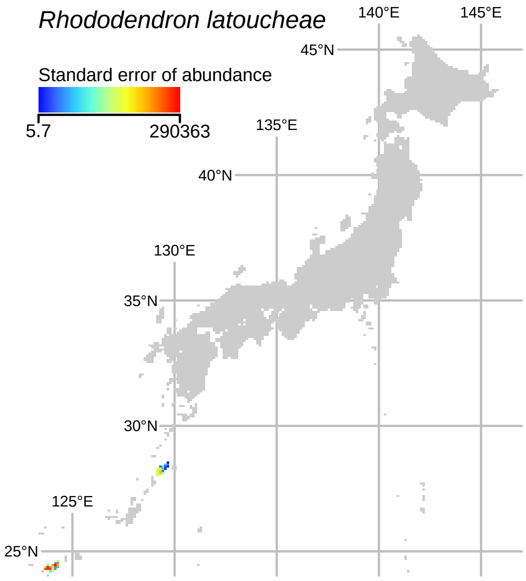
Rhododendron latoucheae
Standard error of abundance
5.7
290363
140°E
145°E
45°N
135°E
40°N
130°E
35°N
30°N
125°E
25°N

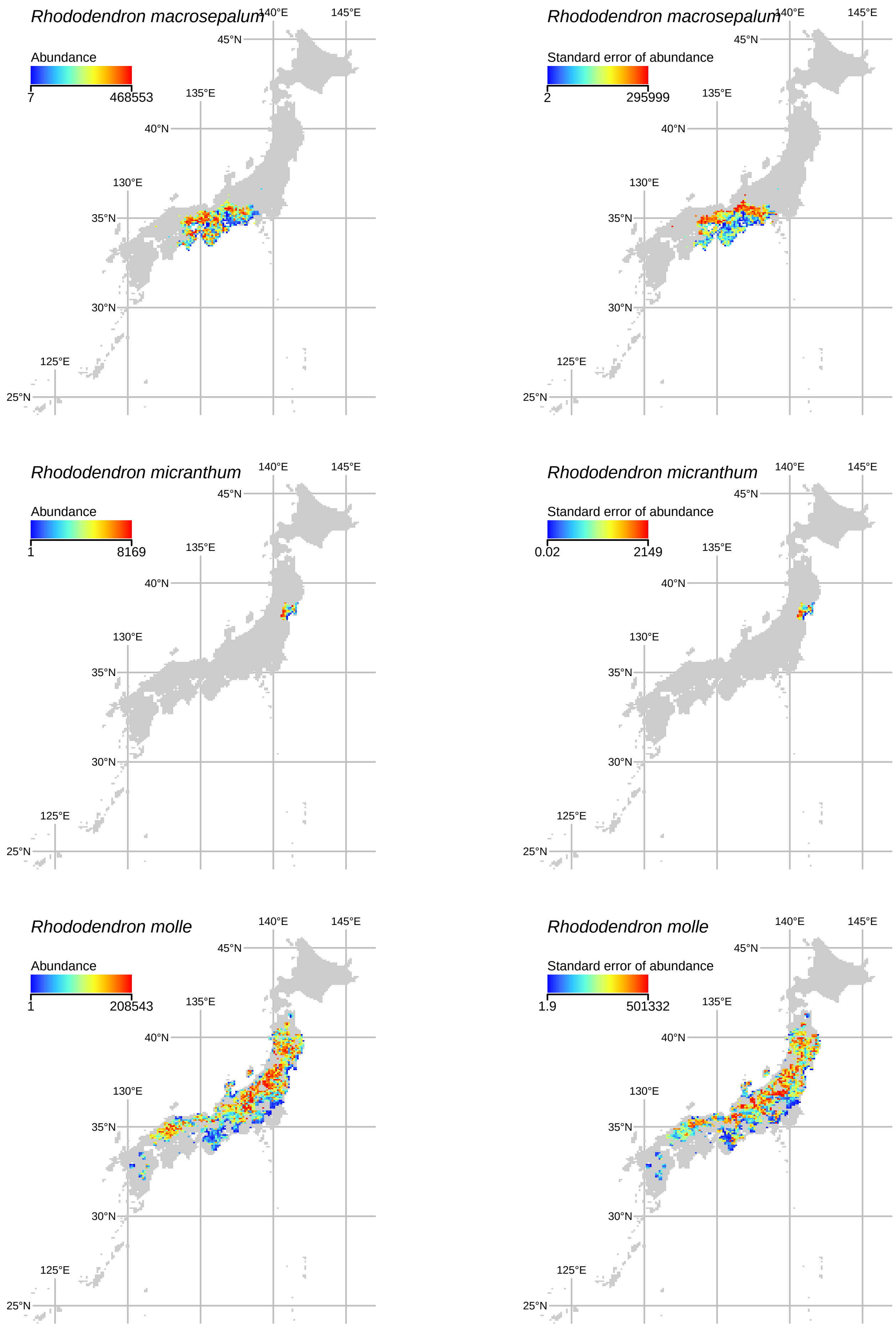
Rhododendron macrosepalum
Abundance
7
468553
Rhododendron macrosepalum
Standard error of abundance
2
295999
Rhododendron micranthum
Abundance
1
8169
Rhododendron micranthum
Standard error of abundance
0.02
2149
Rhododendron molle
Abundance
1
208543
Rhododendron molle
Standard error of abundance
1.9
501332
125°E
130°E
135°E
140°E
145°E
25°N
30°N
35°N
40°N
45°N

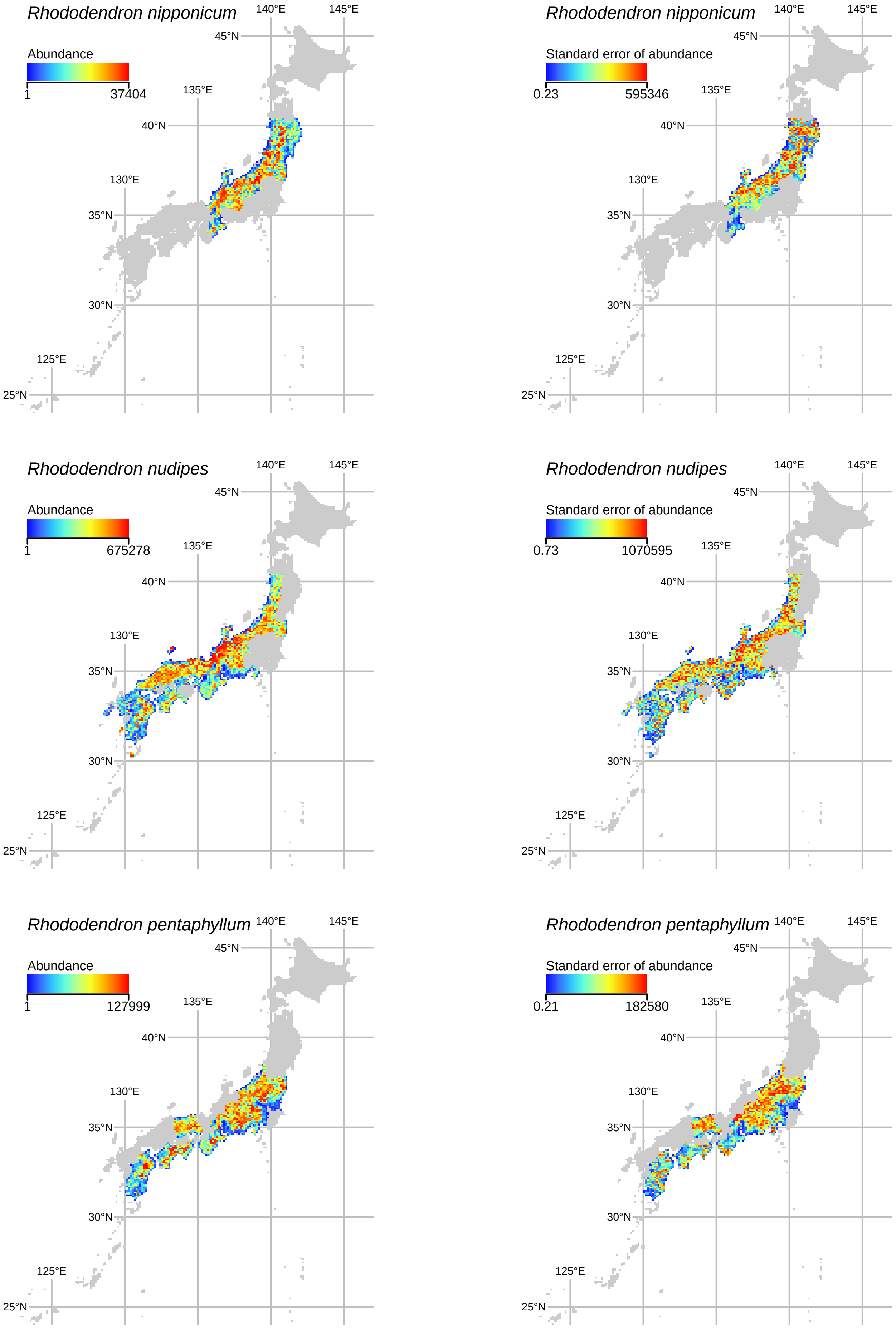
Rhododendron nipponicum
Abundance
1
37404
Rhododendron nipponicum
Standard error of abundance
0.23
595346
Rhododendron nudipes
Abundance
1
675278
Rhododendron nudipes
Standard error of abundance
0.73
1070595
Rhododendron pentaphyllum
Abundance
1
127999
Rhododendron pentaphyllum
Standard error of abundance
0.21
182580
125°E
130°E
135°E
140°E
145°E
25°N
30°N
35°N
40°N
45°N

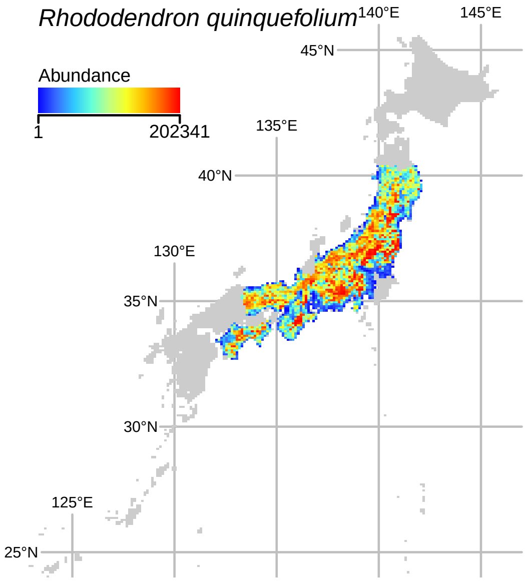
Rhododendron quinquefolium
Abundance
1
202341
140°E
145°E
45°N
135°E
40°N
130°E
35°N
30°N
125°E
25°N

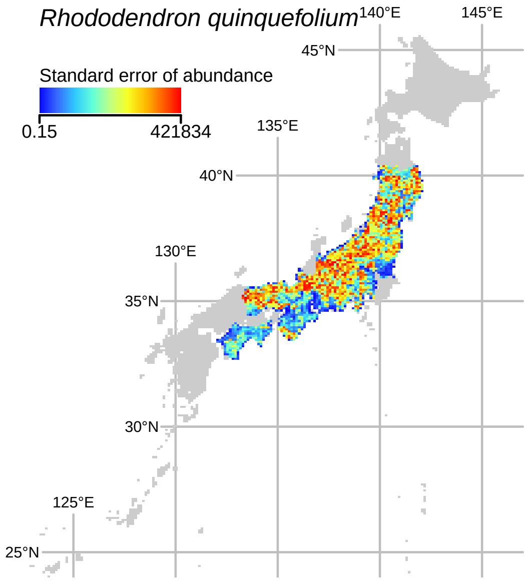
Rhododendron quinquefolium
Standard error of abundance
0.15
421834
140°E
145°E
45°N
135°E
40°N
130°E
35°N
30°N
125°E
25°N

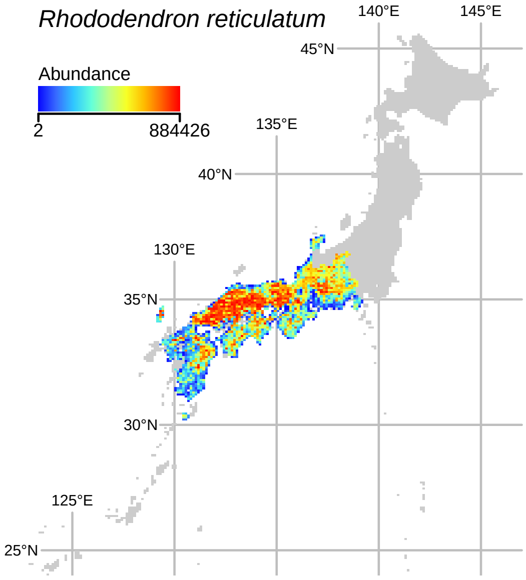
Rhododendron reticulatum
Abundance
2
884426
140°E
145°E
45°N
135°E
40°N
130°E
35°N
30°N
125°E
25°N

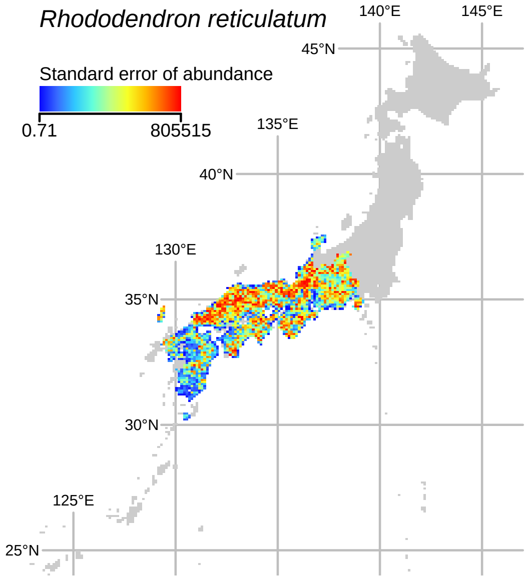
Rhododendron reticulatum
Standard error of abundance
0.71
805515
140°E
145°E
45°N
135°E
40°N
130°E
35°N
30°N
125°E
25°N

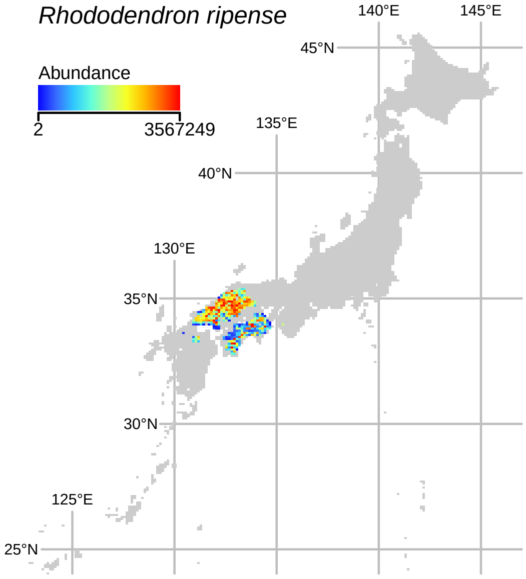
Rhododendron ripense
Abundance
2
3567249
140°E
145°E
45°N
135°E
40°N
130°E
35°N
30°N
125°E
25°N

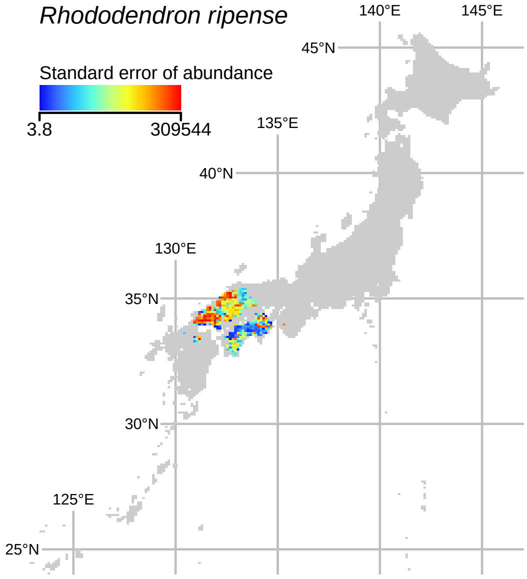
Rhododendron ripense
Standard error of abundance
3.8
309544
140°E
145°E
45°N
135°E
40°N
130°E
35°N
30°N
125°E
25°N

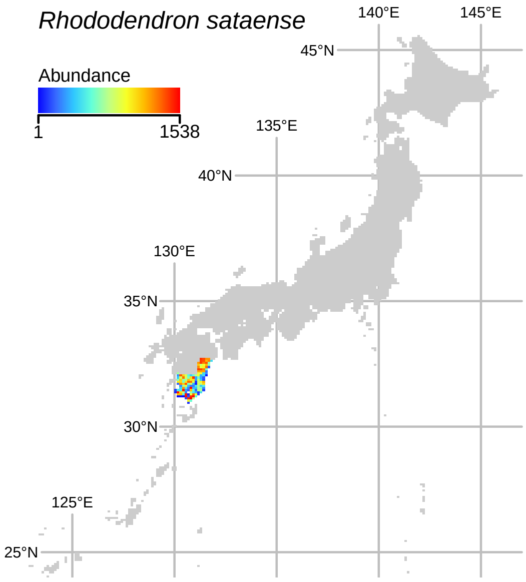
Rhododendron sataense
Abundance
1
1538
140°E
145°E
45°N
135°E
40°N
130°E
35°N
30°N
125°E
25°N

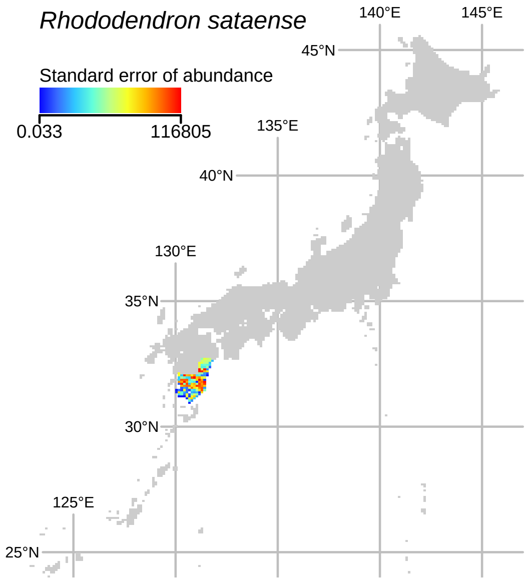
Rhododendron sataense
Standard error of abundance
0.033
116805
140°E
145°E
45°N
135°E
40°N
130°E
35°N
30°N
125°E
25°N

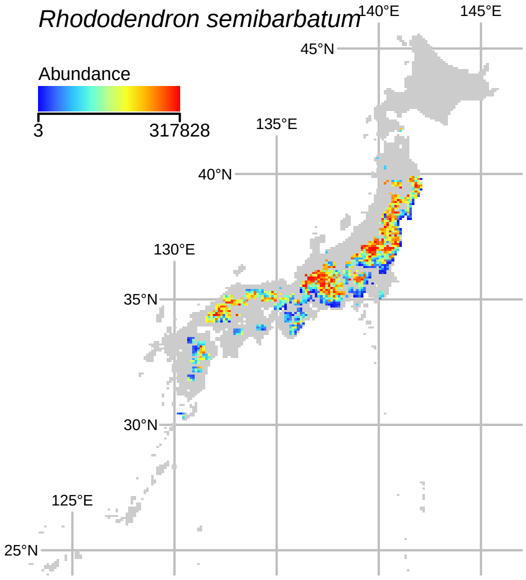
Rhododendron semibarbatum
Abundance
3
317828
140°E
145°E
45°N
135°E
40°N
130°E
35°N
30°N
125°E
25°N

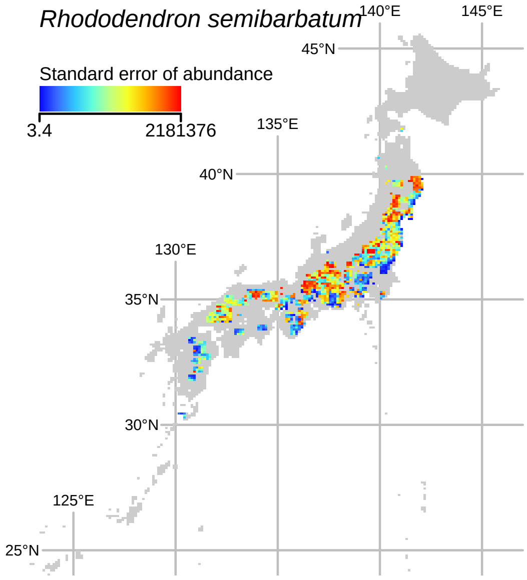
Rhododendron semibarbatum
Standard error of abundance
3.4
2181376
140°E
145°E
45°N
135°E
40°N
130°E
35°N
30°N
125°E
25°N

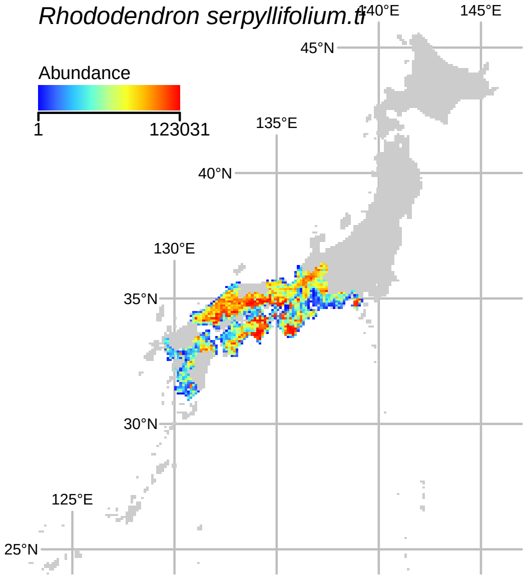
Rhododendron serpyllifolium.ti
Abundance
1
123031
140°E
145°E
45°N
135°E
40°N
130°E
35°N
30°N
125°E
25°N

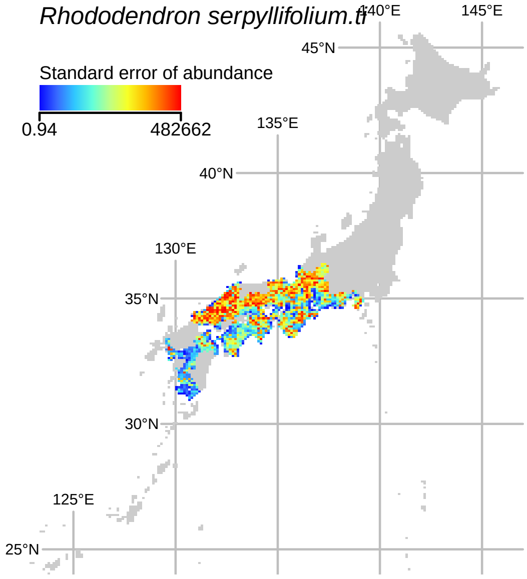
Rhododendron serpyllifolium.ti
Standard error of abundance
0.94
482662
140°E
145°E
45°N
135°E
40°N
130°E
35°N
30°N
125°E
25°N

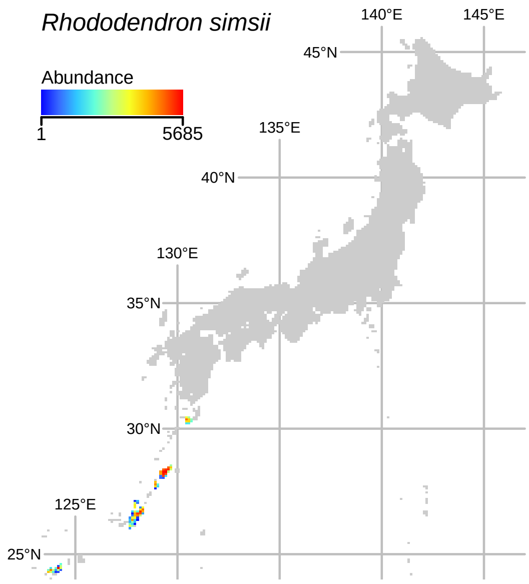
Rhododendron simsii
Abundance
1
5685
140°E
145°E
45°N
135°E
40°N
130°E
35°N
30°N
125°E
25°N

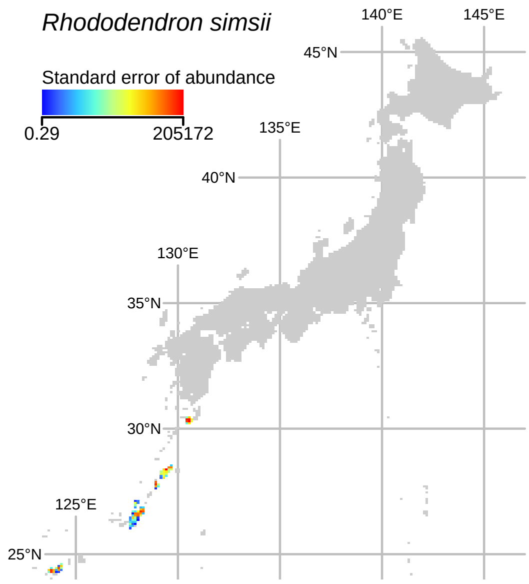
Rhododendron simsii
Standard error of abundance
0.29
205172
140°E
145°E
45°N
135°E
40°N
130°E
35°N
30°N
125°E
25°N

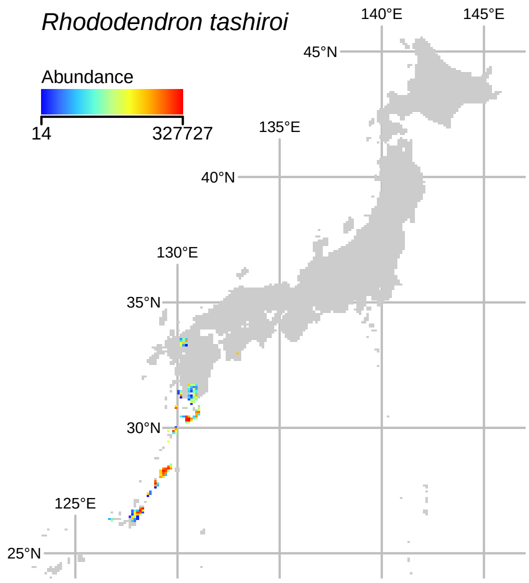
Rhododendron tashiroi
Abundance
14
327727
140°E
145°E
45°N
135°E
40°N
130°E
35°N
30°N
125°E
25°N

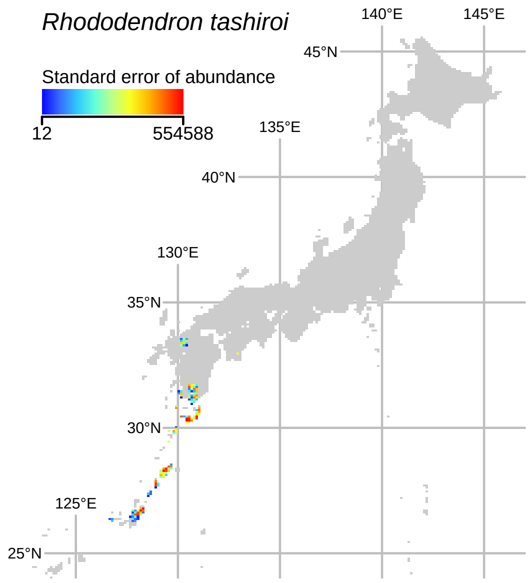
Rhododendron tashiroi
Standard error of abundance
12
554588
140°E
145°E
45°N
135°E
40°N
130°E
35°N
30°N
125°E
25°N

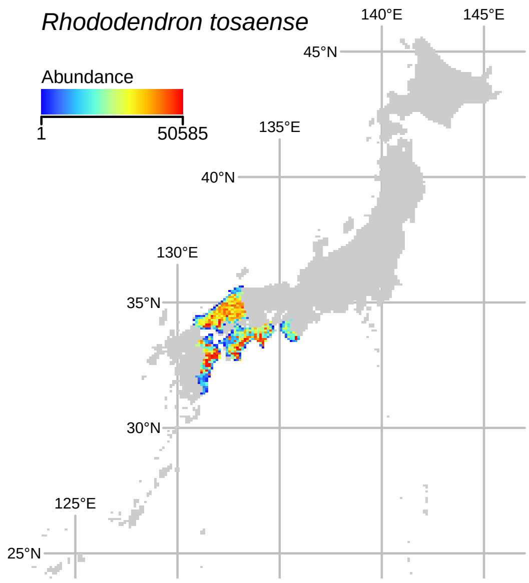
Rhododendron tosaense
Abundance
1
50585
140°E
145°E
45°N
135°E
40°N
130°E
35°N
30°N
125°E
25°N

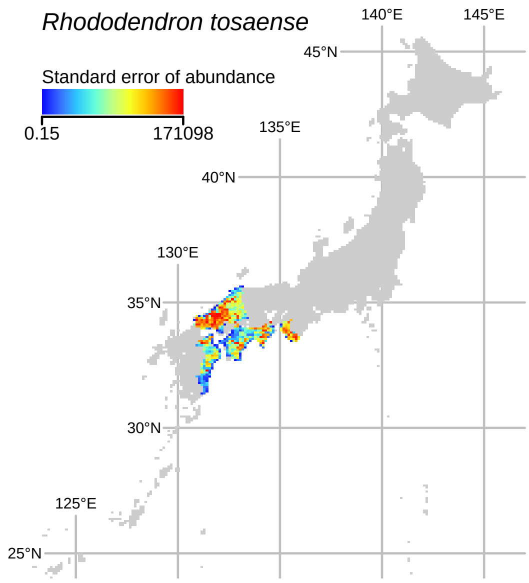
Rhododendron tosaense
Standard error of abundance
0.15
171098
140°E
145°E
45°N
135°E
40°N
130°E
35°N
30°N
125°E
25°N

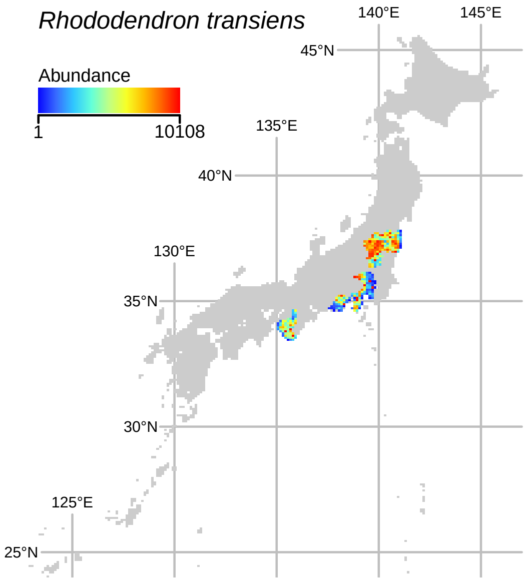
Rhododendron transiens
Abundance
1
10108
140°E
145°E
135°E
130°E
125°E
45°N
40°N
35°N
30°N
25°N

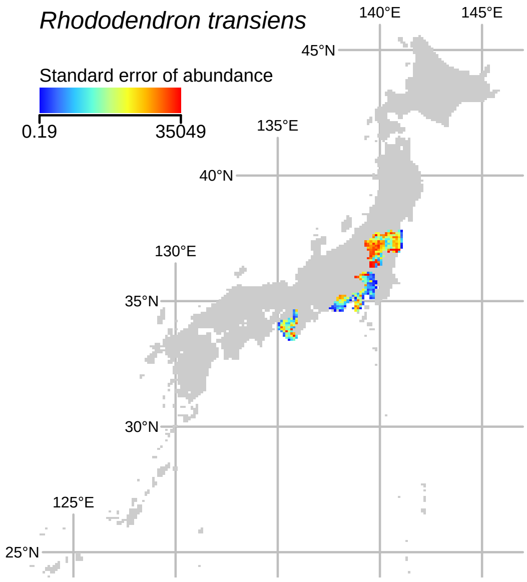
Rhododendron transiens
Standard error of abundance
0.19
35049
140°E
145°E
135°E
130°E
125°E
45°N
40°N
35°N
30°N
25°N

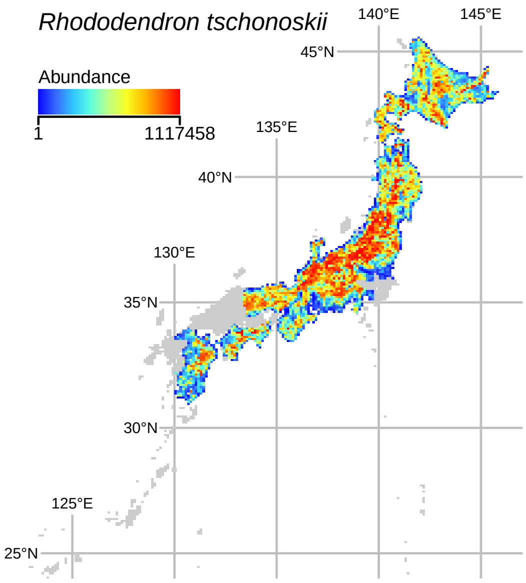
Rhododendron tschonoskii
Abundance
1
1117458
140°E
145°E
135°E
130°E
125°E
45°N
40°N
35°N
30°N
25°N

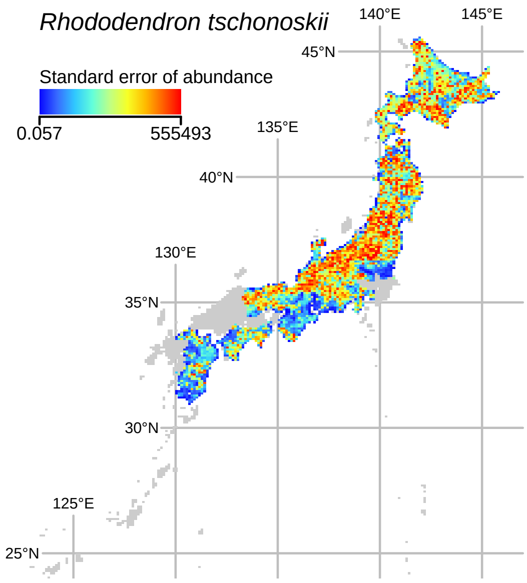
Rhododendron tschonoskii
Standard error of abundance
0.057
555493
140°E
145°E
135°E
130°E
125°E
45°N
40°N
35°N
30°N
25°N

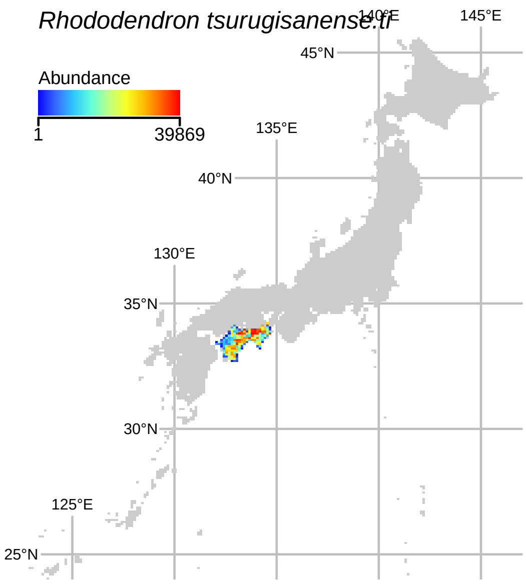
Rhododendron tsurugisanense.tr
Abundance
1
39869
140°E
145°E
135°E
130°E
125°E
45°N
40°N
35°N
30°N
25°N

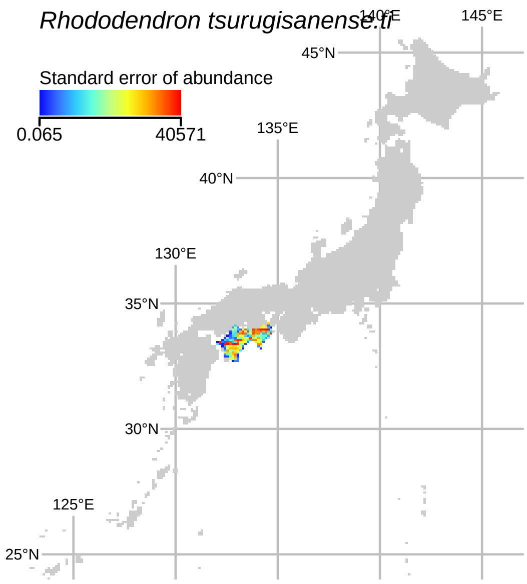
Rhododendron tsurugisanense.tr
Standard error of abundance
0.065
40571
140°E
145°E
135°E
130°E
125°E
45°N
40°N
35°N
30°N
25°N

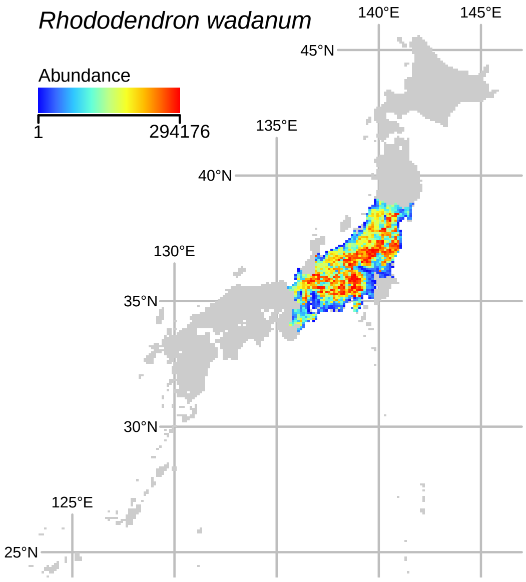
Rhododendron wadanum
Abundance
1
294176
140°E
145°E
45°N
135°E
40°N
130°E
35°N
30°N
125°E
25°N

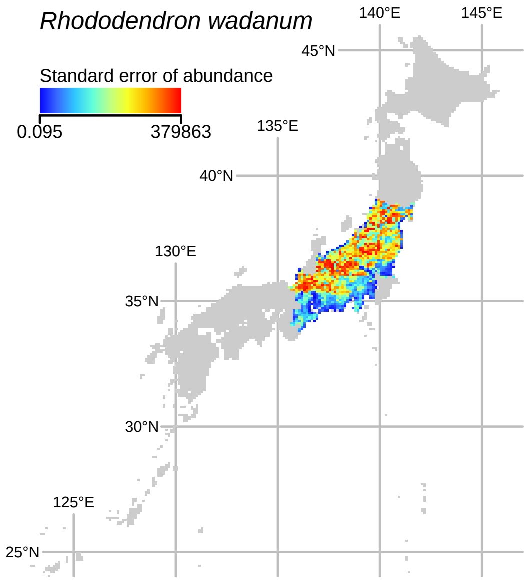
Rhododendron wadanum
Standard error of abundance
0.095
379863
140°E
145°E
45°N
135°E
40°N
130°E
35°N
30°N
125°E
25°N

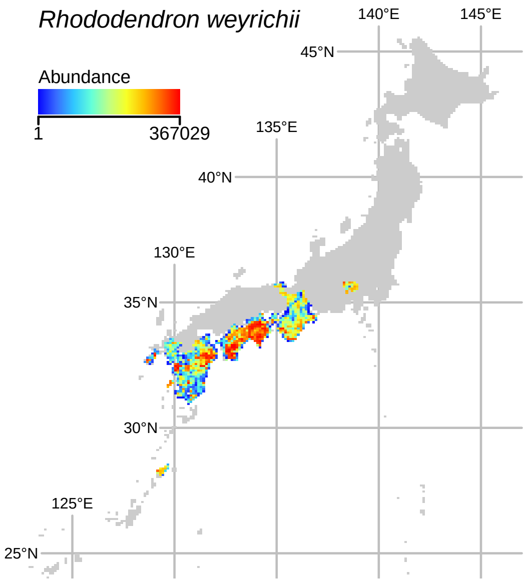
Rhododendron weyrichii
Abundance
1
367029
140°E
145°E
45°N
135°E
40°N
130°E
35°N
30°N
125°E
25°N

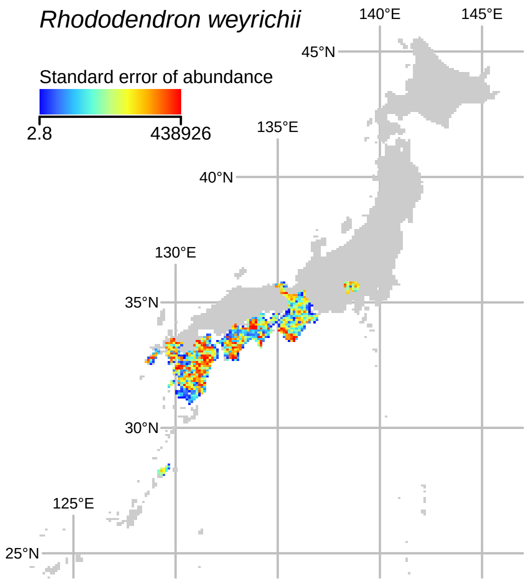
Rhododendron weyrichii
Standard error of abundance
2.8
438926
140°E
145°E
45°N
135°E
40°N
130°E
35°N
30°N
125°E
25°N

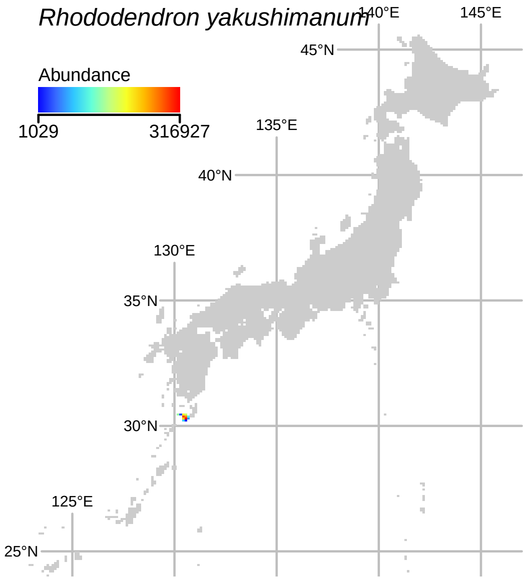
Rhododendron yakushimanum
Abundance
1029
316927
140°E
145°E
45°N
135°E
40°N
130°E
35°N
30°N
125°E
25°N

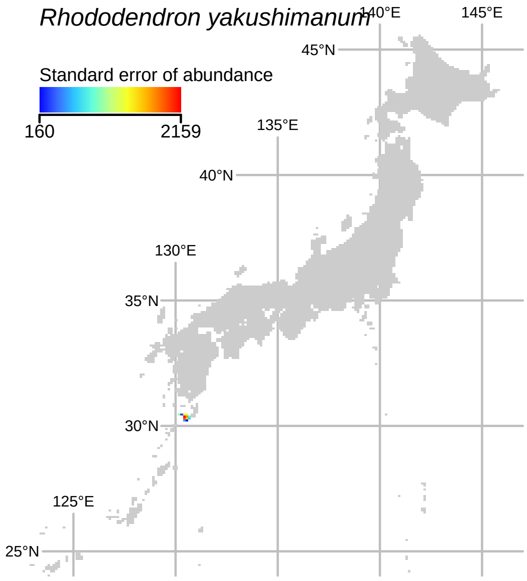
Rhododendron yakushimanum
Standard error of abundance
160
2159
140°E
145°E
45°N
135°E
40°N
130°E
35°N
30°N
125°E
25°N

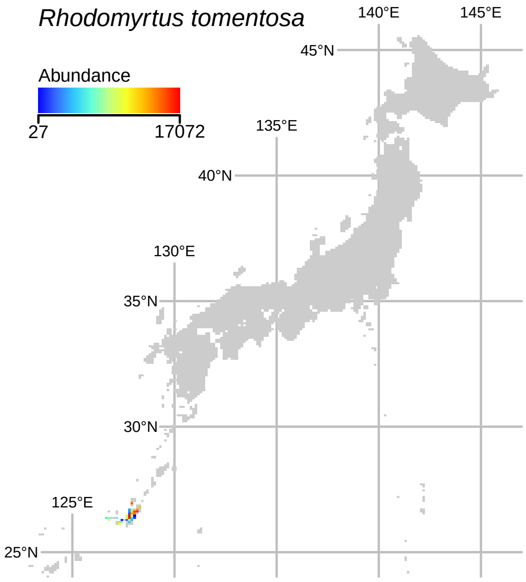
Rhodomyrtus tomentosa
Abundance
27
17072
140°E
145°E
45°N
135°E
40°N
130°E
35°N
30°N
125°E
25°N

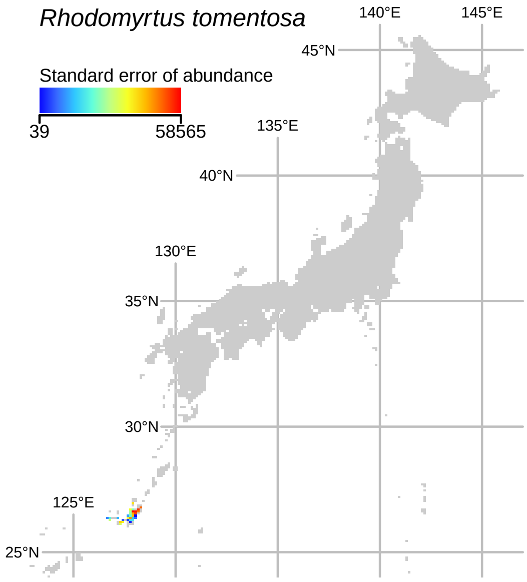
Rhodomyrtus tomentosa
Standard error of abundance
39
58565
140°E
145°E
45°N
135°E
40°N
130°E
35°N
30°N
125°E
25°N

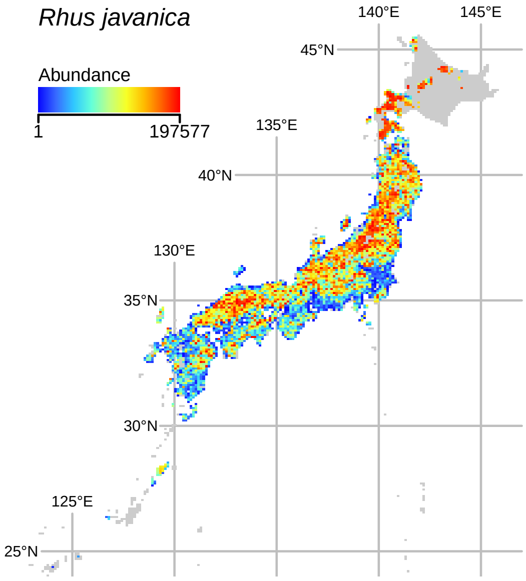
Rhus javanica
Abundance
1
197577
140°E
145°E
45°N
135°E
40°N
130°E
35°N
30°N
125°E
25°N

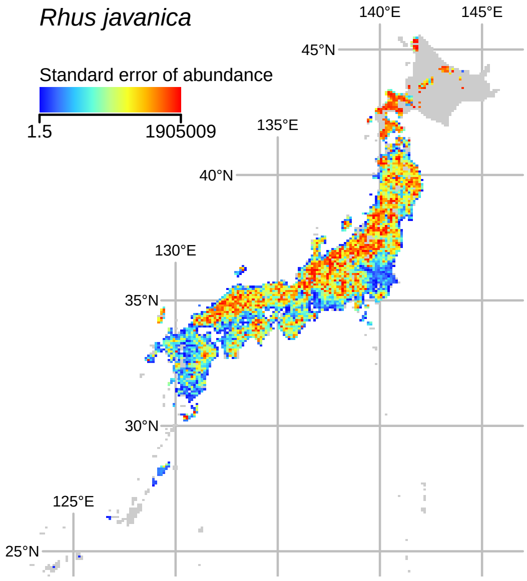
Rhus javanica
Standard error of abundance
1.5
1905009
140°E
145°E
45°N
135°E
40°N
130°E
35°N
30°N
125°E
25°N

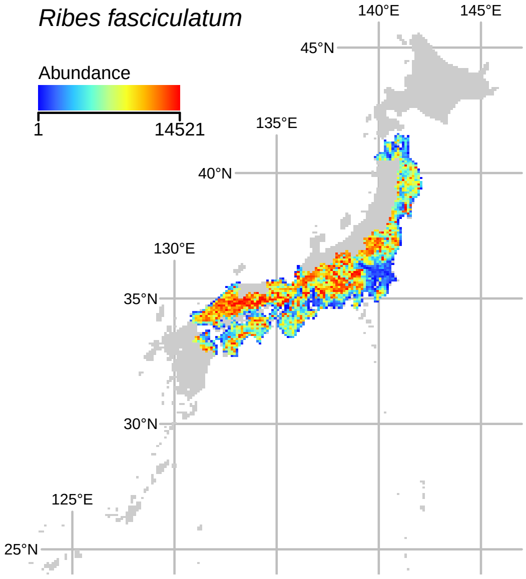
Ribes fasciculatum
Abundance
1
14521
140°E
145°E
45°N
135°E
40°N
130°E
35°N
30°N
125°E
25°N

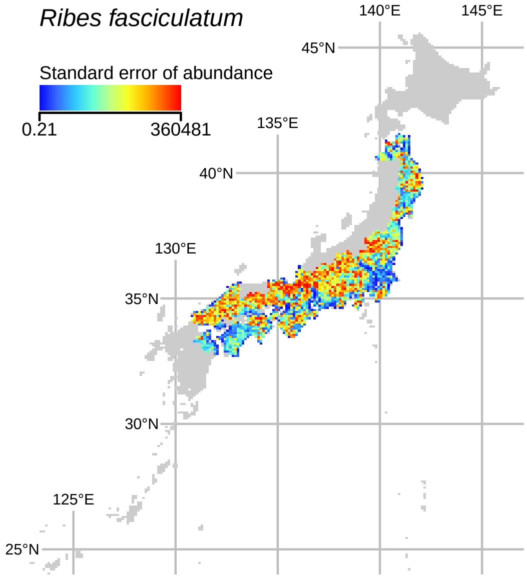
Ribes fasciculatum
Standard error of abundance
0.21
360481
140°E
145°E
45°N
135°E
40°N
130°E
35°N
30°N
125°E
25°N

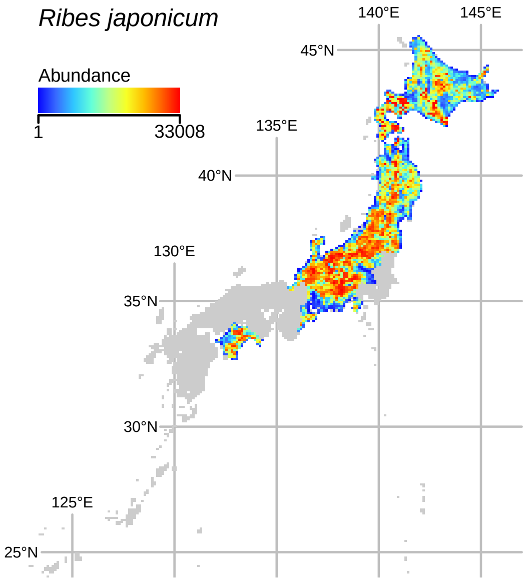
Ribes japonicum
Abundance
1
33008
140°E
145°E
45°N
135°E
40°N
130°E
35°N
30°N
125°E
25°N

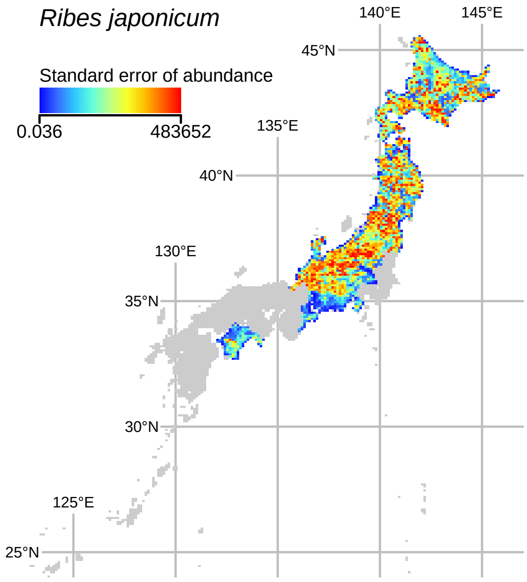
Ribes japonicum
Standard error of abundance
0.036
483652
140°E
145°E
45°N
135°E
40°N
130°E
35°N
30°N
125°E
25°N

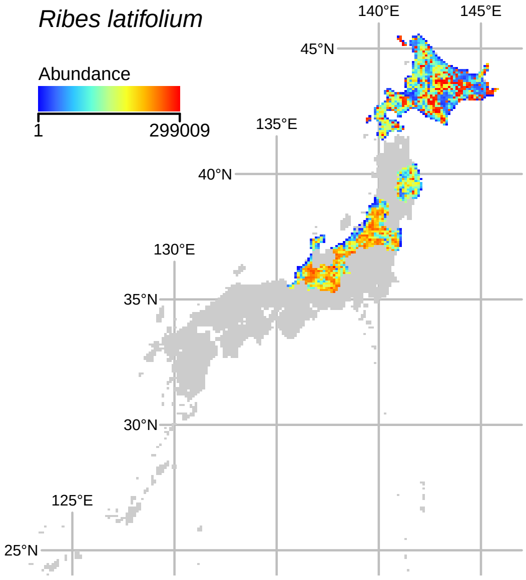
Ribes latifolium
Abundance
1
299009
140°E
145°E
45°N
135°E
40°N
130°E
35°N
30°N
125°E
25°N

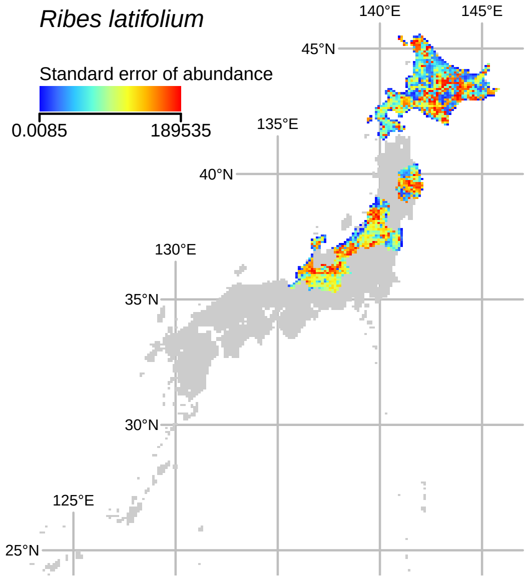
Ribes latifolium
Standard error of abundance
0.0085
189535
140°E
145°E
45°N
135°E
40°N
130°E
35°N
30°N
125°E
25°N

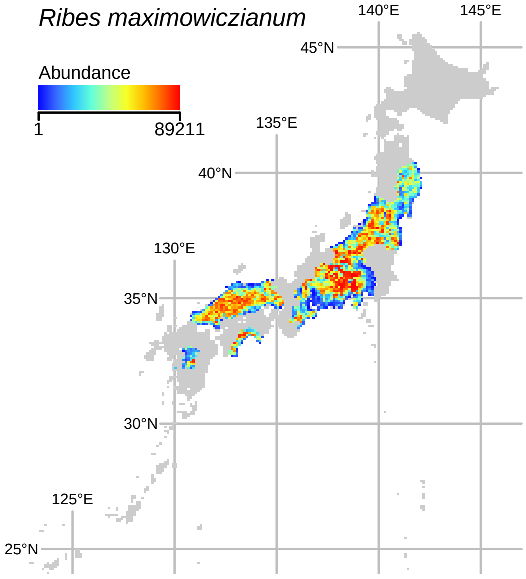
Ribes maximowiczianum
Abundance
1
89211
140°E
145°E
45°N
135°E
40°N
130°E
35°N
30°N
125°E
25°N

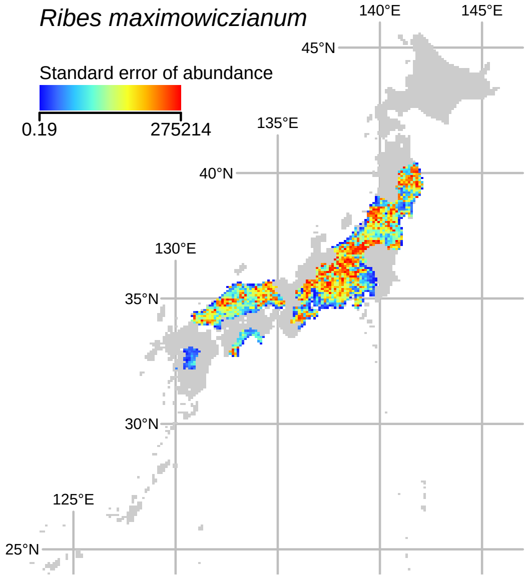
Ribes maximowiczianum
Standard error of abundance
0.19
275214
140°E
145°E
45°N
135°E
40°N
130°E
35°N
30°N
125°E
25°N

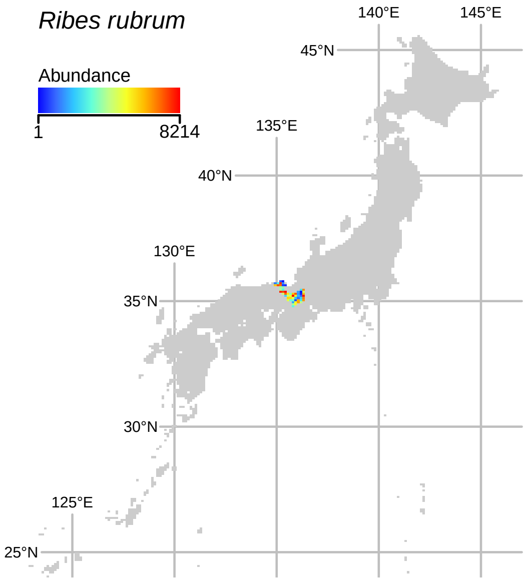
Ribes rubrum
Abundance
1
8214
140°E
145°E
45°N
135°E
40°N
130°E
35°N
30°N
125°E
25°N

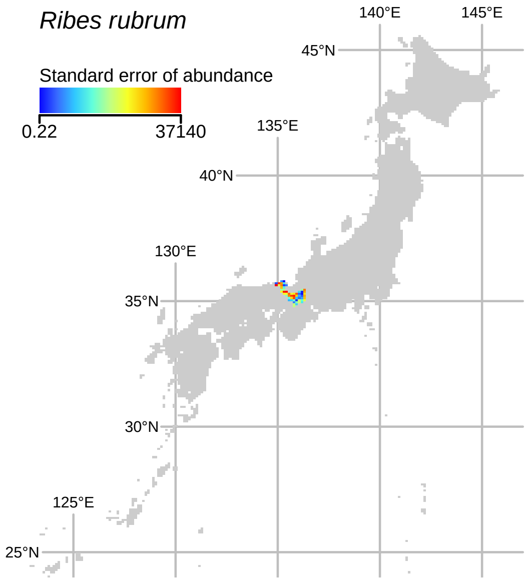
Ribes rubrum
Standard error of abundance
0.22
37140
140°E
145°E
45°N
135°E
40°N
130°E
35°N
30°N
125°E
25°N

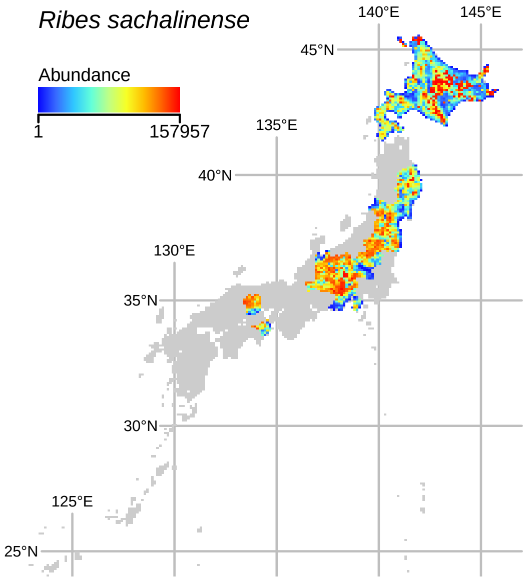
Ribes sachalinense
Abundance
1
157957
140°E
145°E
45°N
135°E
40°N
130°E
35°N
30°N
125°E
25°N

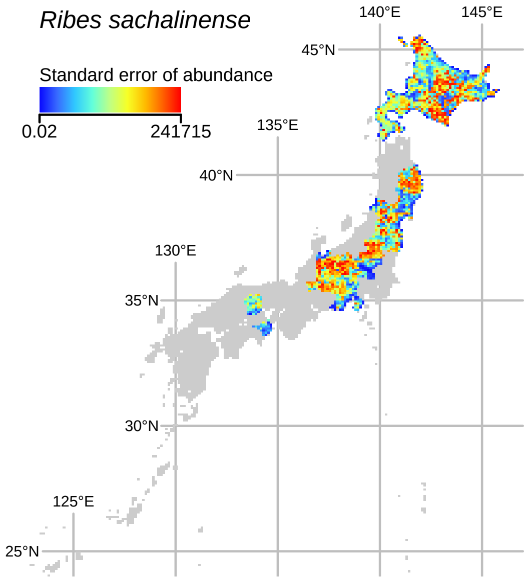
Ribes sachalinense
Standard error of abundance
0.02
241715
140°E
145°E
45°N
135°E
40°N
130°E
35°N
30°N
125°E
25°N

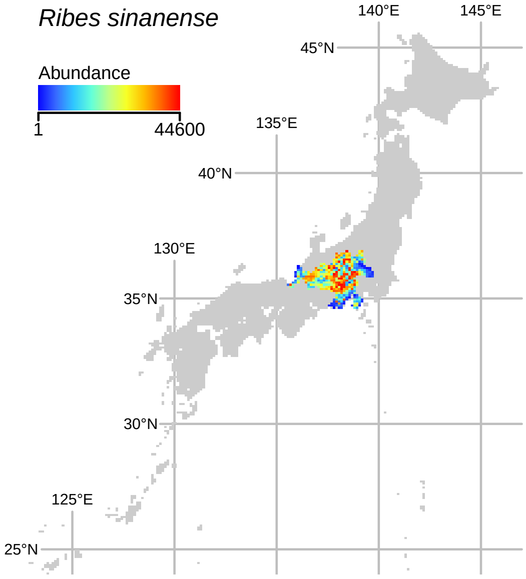
Ribes sinanense
Abundance
1
44600
140°E
145°E
45°N
135°E
40°N
130°E
35°N
30°N
125°E
25°N

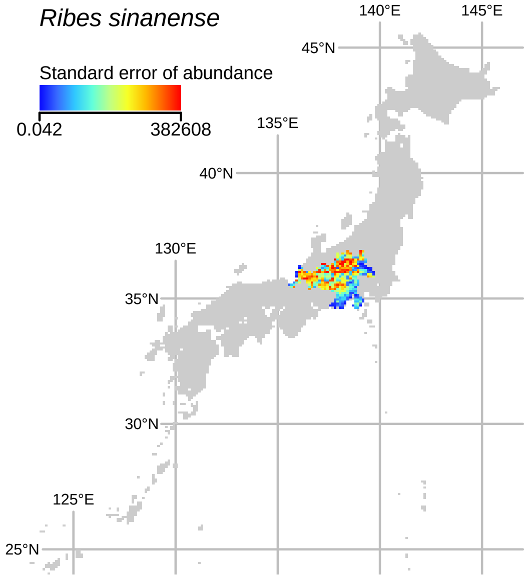
Ribes sinanense
Standard error of abundance
0.042
382608
140°E
145°E
45°N
135°E
40°N
130°E
35°N
30°N
125°E
25°N

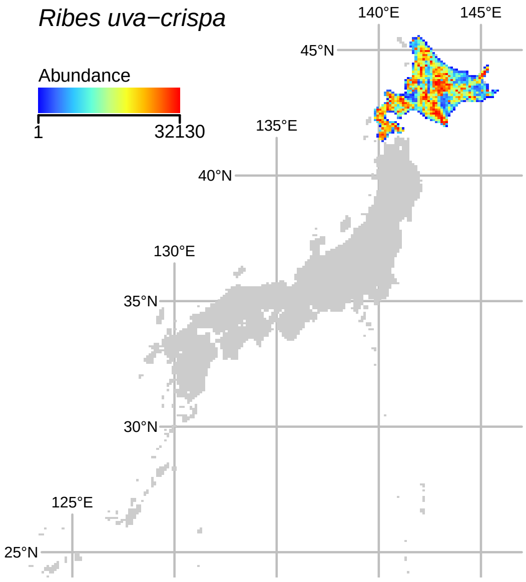
Ribes uva−crispa
Abundance
1
32130
140°E
145°E
45°N
135°E
40°N
130°E
35°N
30°N
125°E
25°N

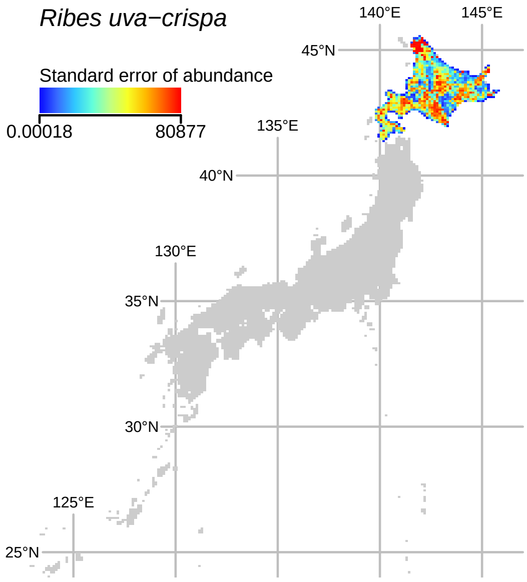
Ribes uva−crispa
Standard error of abundance
0.00018
80877
140°E
145°E
45°N
135°E
40°N
130°E
35°N
30°N
125°E
25°N

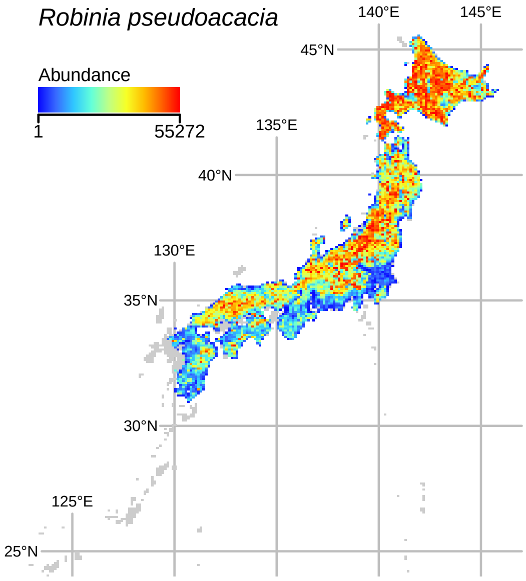
Robinia pseudoacacia
Abundance
1
55272
140°E
145°E
45°N
135°E
40°N
130°E
35°N
30°N
125°E
25°N

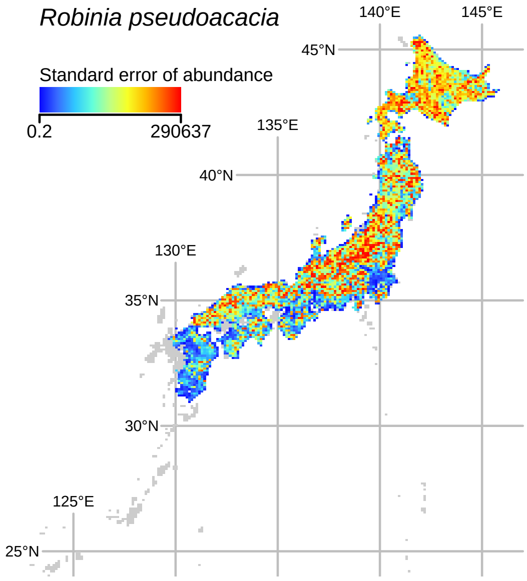
Robinia pseudoacacia
Standard error of abundance
0.2
290637
140°E
145°E
45°N
135°E
40°N
130°E
35°N
30°N
125°E
25°N

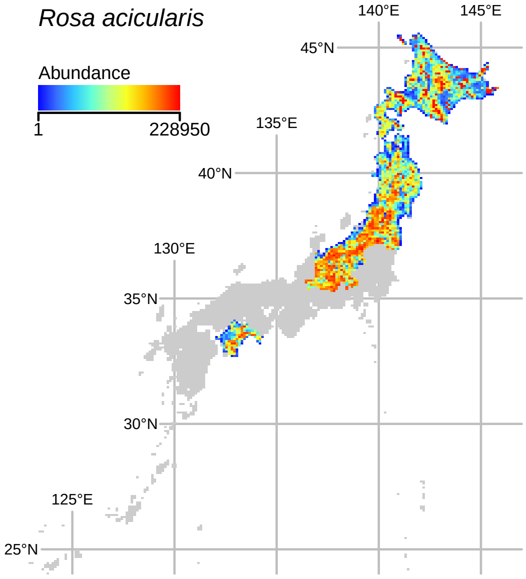
Rosa acicularis
Abundance
1
228950
140°E
145°E
45°N
135°E
40°N
130°E
35°N
30°N
125°E
25°N

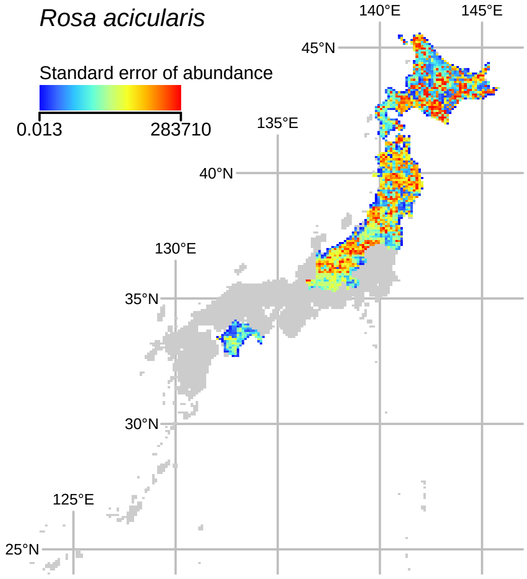
Rosa acicularis
Standard error of abundance
0.013
283710
140°E
145°E
45°N
135°E
40°N
130°E
35°N
30°N
125°E
25°N

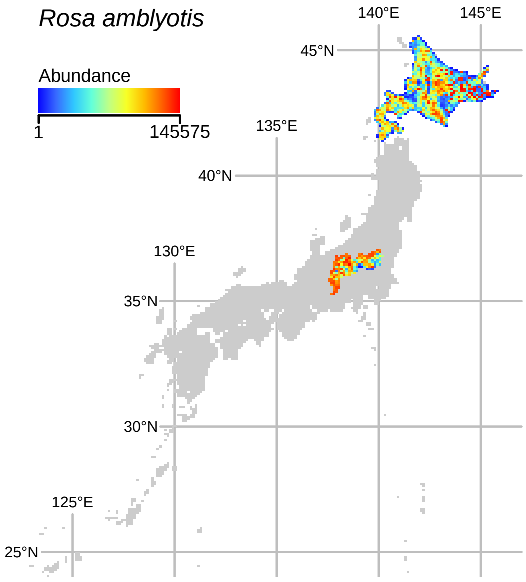
Rosa amblyotis
Abundance
1
145575
140°E
145°E
135°E
130°E
125°E
45°N
40°N
35°N
30°N
25°N

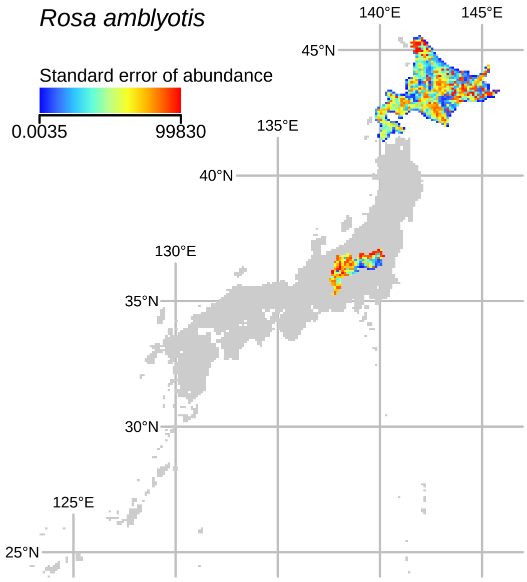
Rosa amblyotis
Standard error of abundance
0.0035
99830
140°E
145°E
135°E
130°E
125°E
45°N
40°N
35°N
30°N
25°N

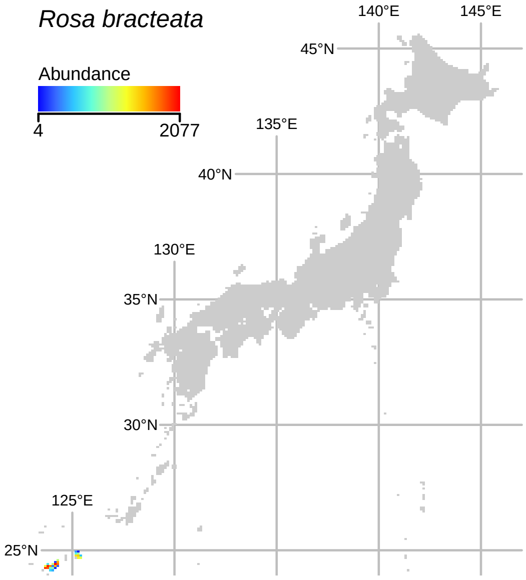
Rosa bracteata
Abundance
4
2077
140°E
145°E
135°E
130°E
125°E
45°N
40°N
35°N
30°N
25°N

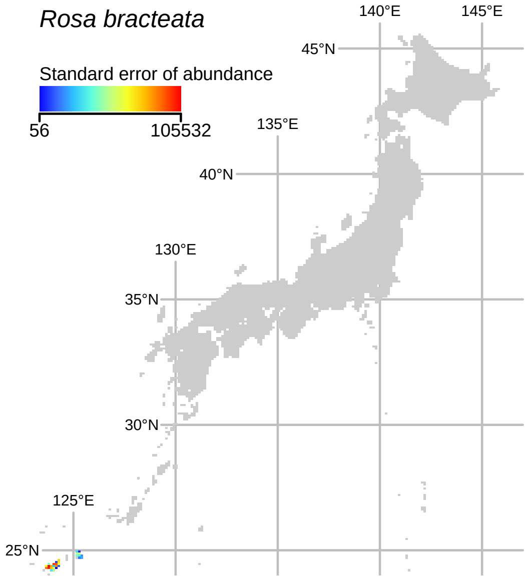
Rosa bracteata
Standard error of abundance
56
105532
140°E
145°E
135°E
130°E
125°E
45°N
40°N
35°N
30°N
25°N

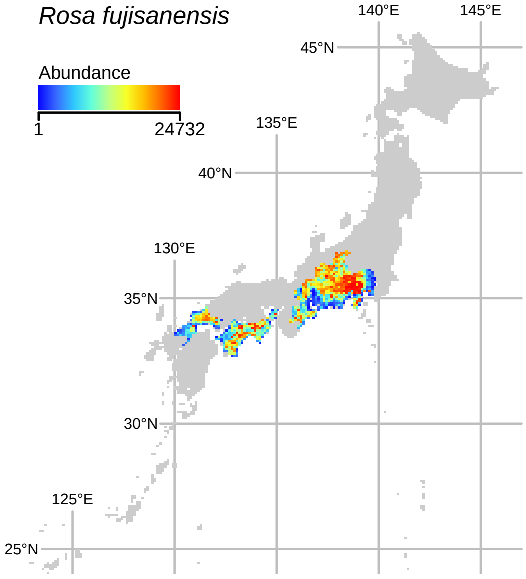
Rosa fujisanensis
Abundance
1
24732
140°E
145°E
135°E
130°E
125°E
45°N
40°N
35°N
30°N
25°N

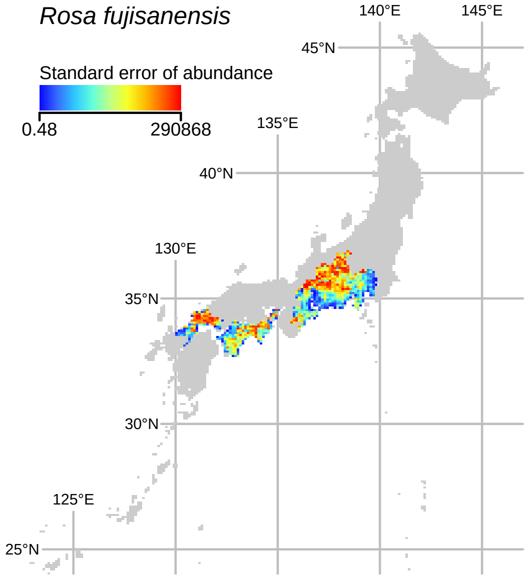
Rosa fujisanensis
Standard error of abundance
0.48
290868
140°E
145°E
135°E
130°E
125°E
45°N
40°N
35°N
30°N
25°N

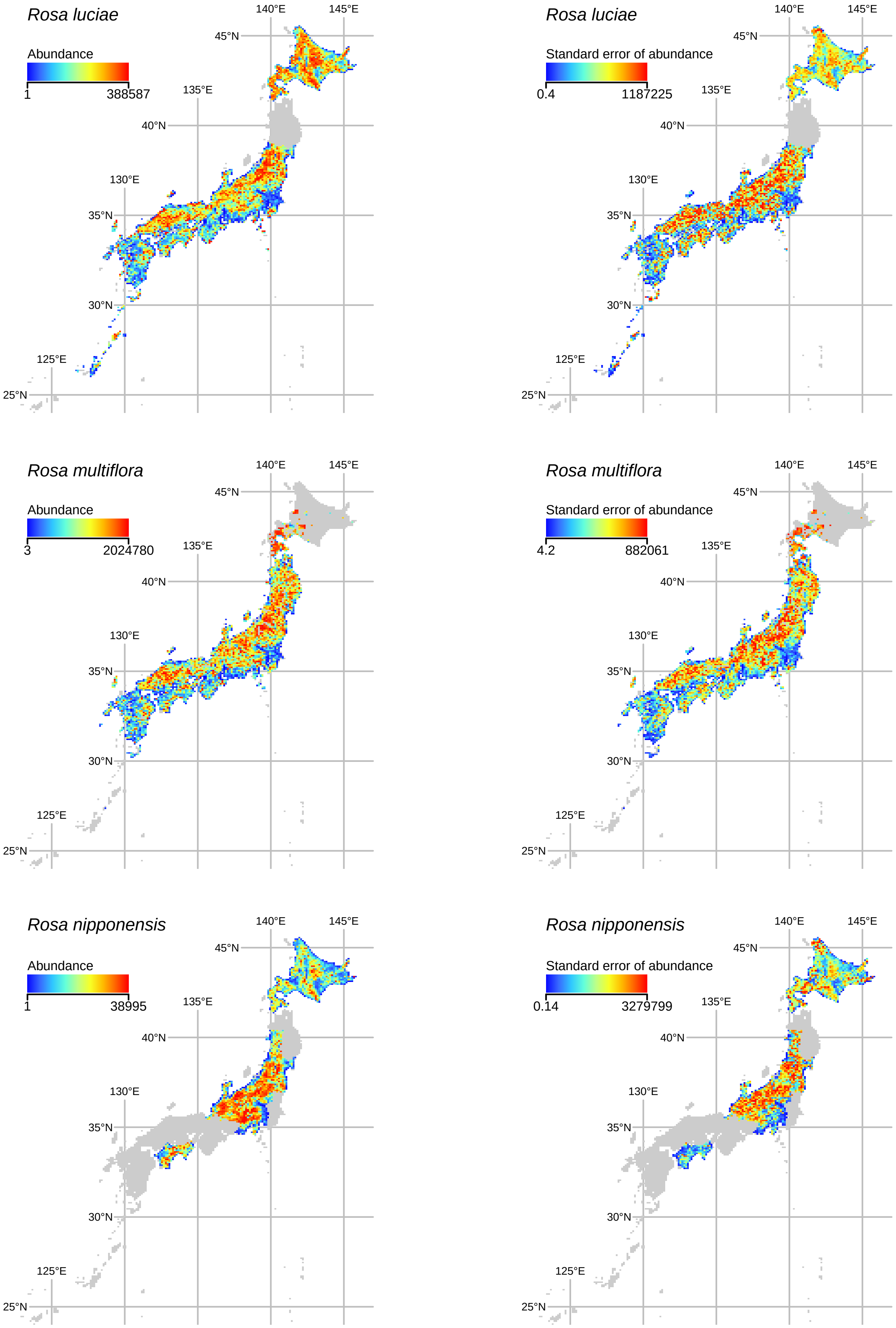
Rosa luciae
Abundance
1
388587
Rosa luciae
Standard error of abundance
0.4
1187225
Rosa multiflora
Abundance
3
2024780
Rosa multiflora
Standard error of abundance
4.2
882061
Rosa nipponensis
Abundance
1
38995
Rosa nipponensis
Standard error of abundance
0.14
3279799
125°E
130°E
135°E
140°E
145°E
25°N
30°N
35°N
40°N
45°N

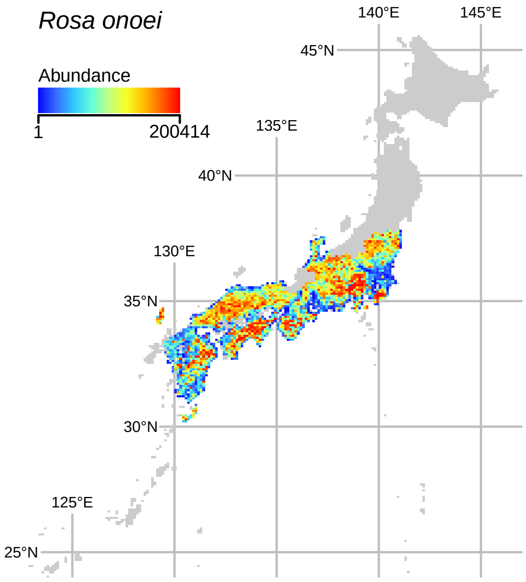
Rosa onoei
Abundance
1
200414
140°E
145°E
45°N
135°E
40°N
130°E
35°N
30°N
125°E
25°N

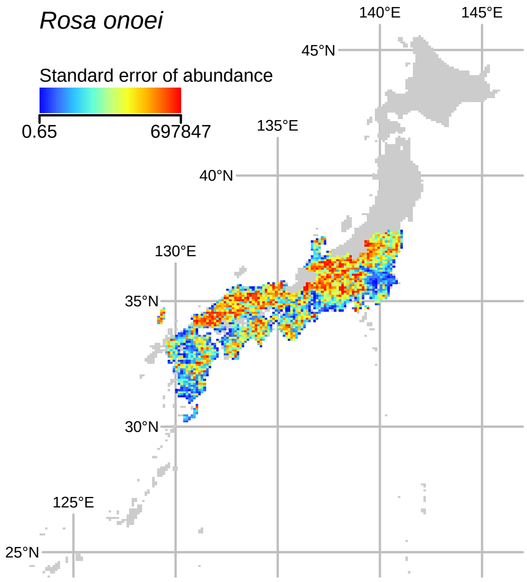
Rosa onoei
Standard error of abundance
0.65
697847
140°E
145°E
45°N
135°E
40°N
130°E
35°N
30°N
125°E
25°N

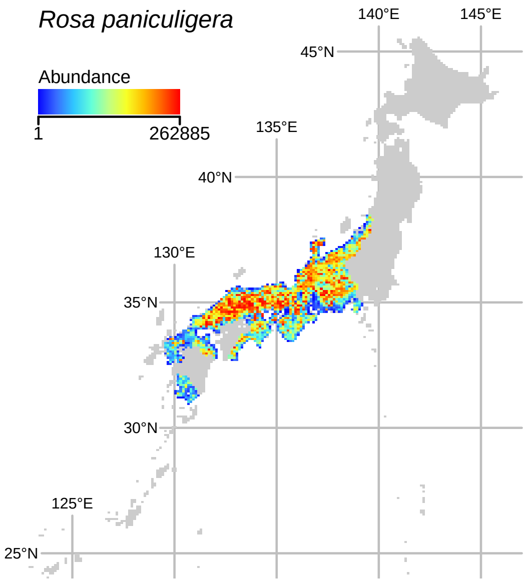
Rosa paniculigera
Abundance
1
262885
140°E
145°E
45°N
135°E
40°N
130°E
35°N
30°N
125°E
25°N

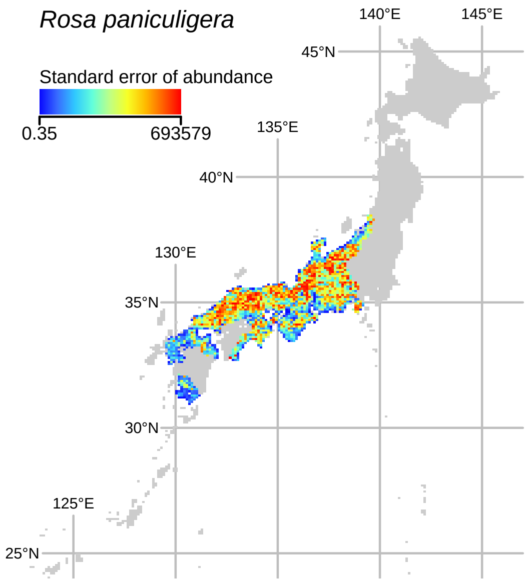
Rosa paniculigera
Standard error of abundance
0.35
693579
140°E
145°E
45°N
135°E
40°N
130°E
35°N
30°N
125°E
25°N

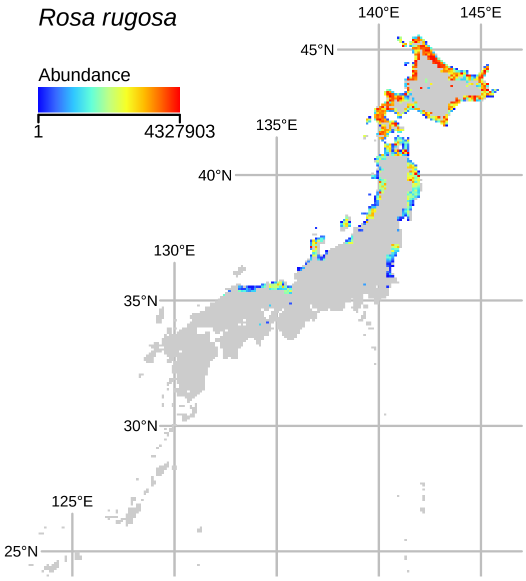
Rosa rugosa
Abundance
1
4327903
140°E
145°E
45°N
135°E
40°N
130°E
35°N
30°N
125°E
25°N

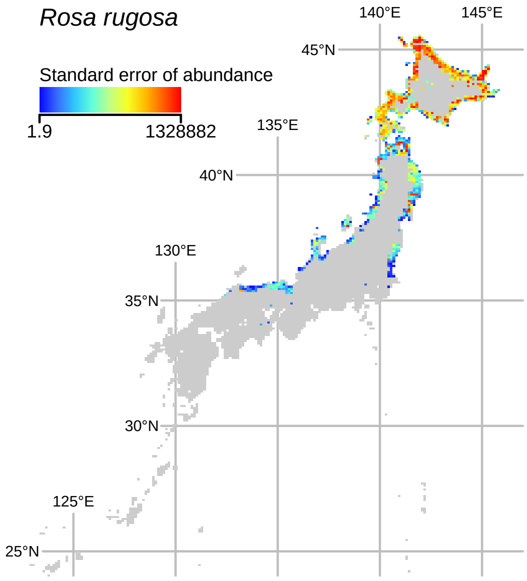
Rosa rugosa
Standard error of abundance
1.9
1328882
140°E
145°E
45°N
135°E
40°N
130°E
35°N
30°N
125°E
25°N

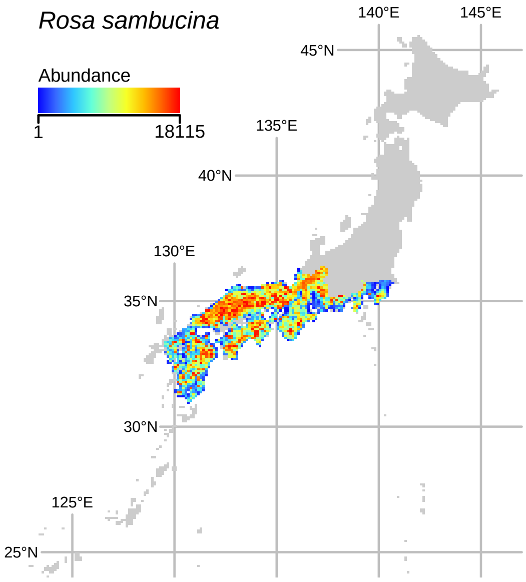
Rosa sambucina
Abundance
1
18115
140°E
145°E
45°N
135°E
40°N
130°E
35°N
30°N
125°E
25°N

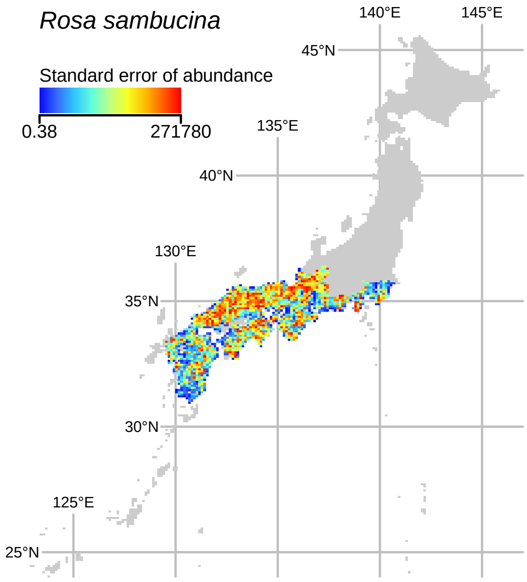
Rosa sambucina
Standard error of abundance
0.38
271780
140°E
145°E
45°N
135°E
40°N
130°E
35°N
30°N
125°E
25°N

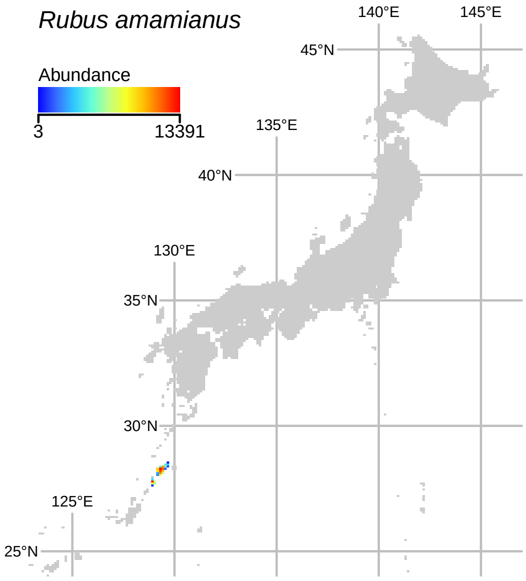
Rubus amamianus
Abundance
3
13391
140°E
145°E
45°N
135°E
40°N
130°E
35°N
30°N
125°E
25°N

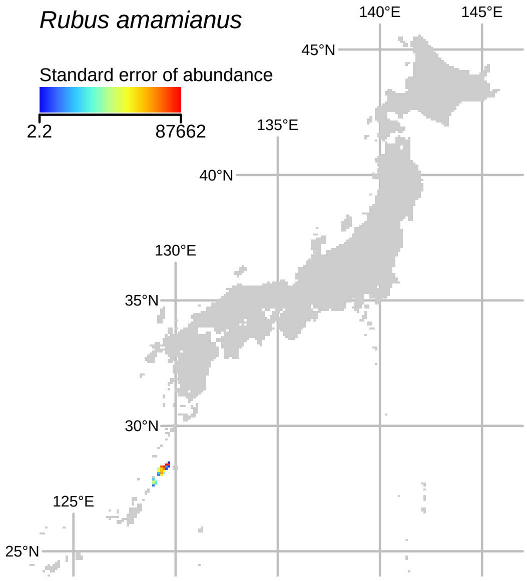
Rubus amamianus
Standard error of abundance
2.2
87662
140°E
145°E
45°N
135°E
40°N
130°E
35°N
30°N
125°E
25°N

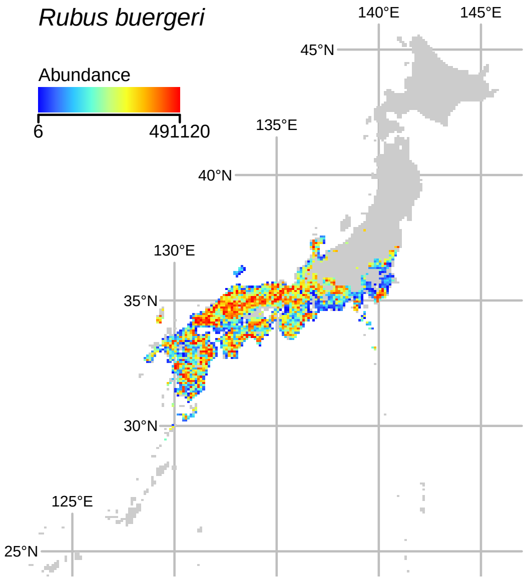
Rubus buergeri
Abundance
6
491120
140°E
145°E
45°N
135°E
40°N
130°E
35°N
30°N
125°E
25°N

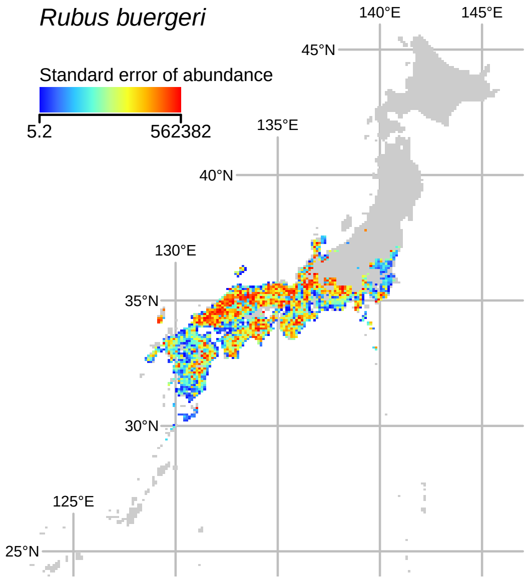
Rubus buergeri
Standard error of abundance
5.2
562382
140°E
145°E
45°N
135°E
40°N
130°E
35°N
30°N
125°E
25°N

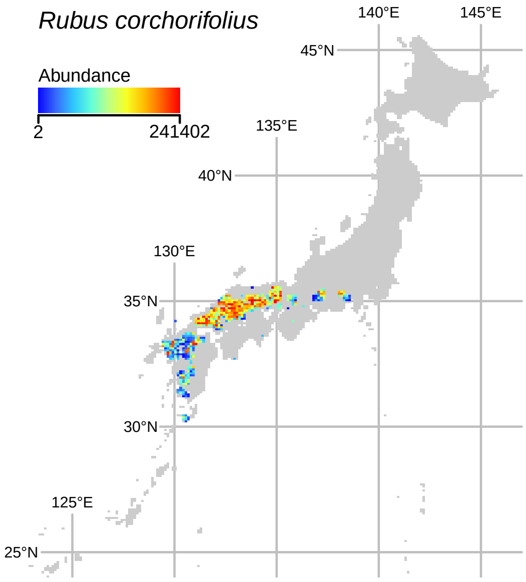
Rubus corchorifolius
Abundance
2
241402
140°E
145°E
45°N
135°E
40°N
130°E
35°N
30°N
125°E
25°N

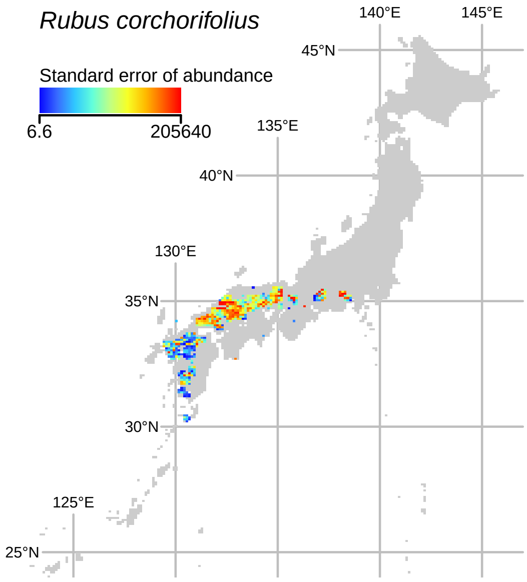
Rubus corchorifolius
Standard error of abundance
6.6
205640
140°E
145°E
45°N
135°E
40°N
130°E
35°N
30°N
125°E
25°N

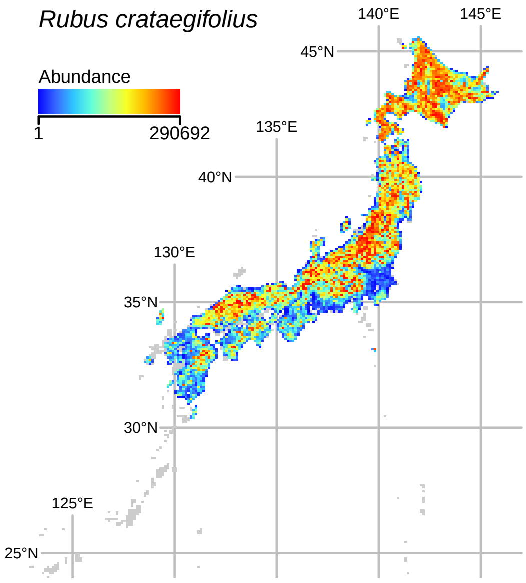
Rubus crataegifolius
Abundance
1
290692
140°E
145°E
45°N
135°E
40°N
130°E
35°N
30°N
125°E
25°N

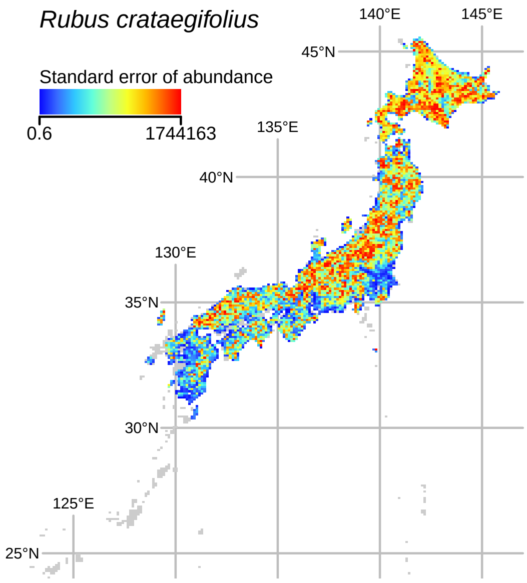
Rubus crataegifolius
Standard error of abundance
0.6
1744163
140°E
145°E
45°N
135°E
40°N
130°E
35°N
30°N
125°E
25°N

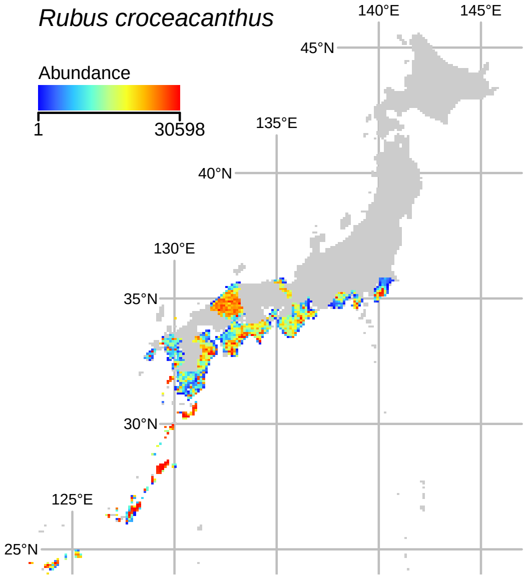
Rubus croceacanthus
Abundance
1
30598
140°E
145°E
45°N
135°E
40°N
130°E
35°N
30°N
125°E
25°N

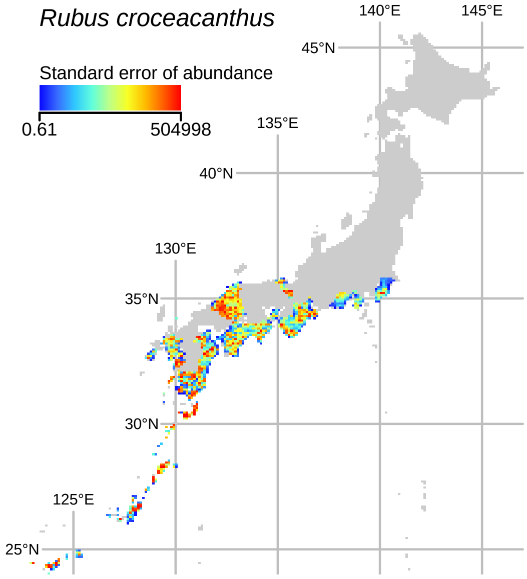
Rubus croceacanthus
Standard error of abundance
0.61
504998
140°E
145°E
45°N
135°E
40°N
130°E
35°N
30°N
125°E
25°N

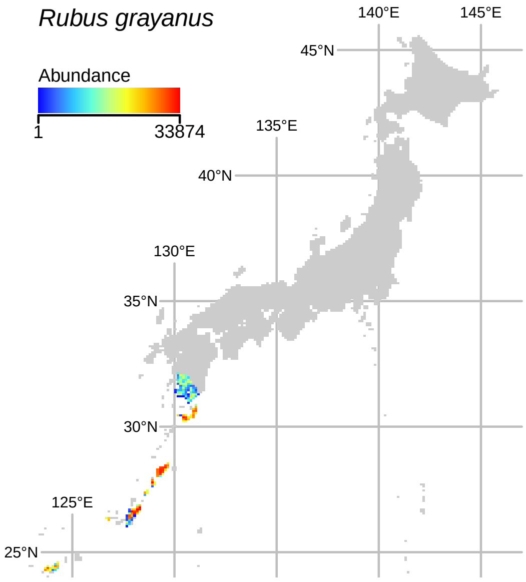
Rubus grayanus
Abundance
1
33874
140°E
145°E
45°N
135°E
40°N
130°E
35°N
30°N
125°E
25°N

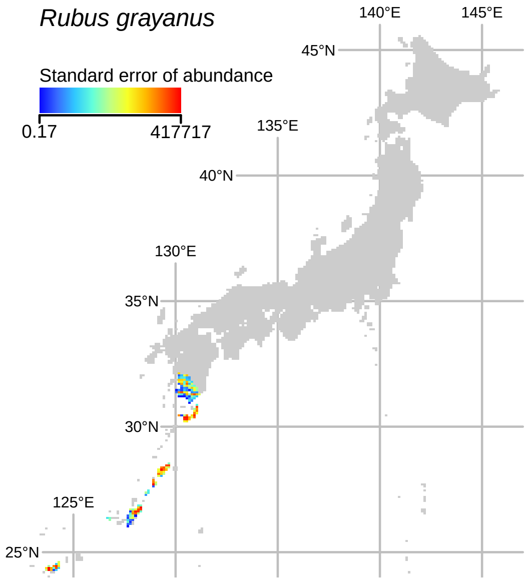
Rubus grayanus
Standard error of abundance
0.17
417717
140°E
145°E
45°N
135°E
40°N
130°E
35°N
30°N
125°E
25°N

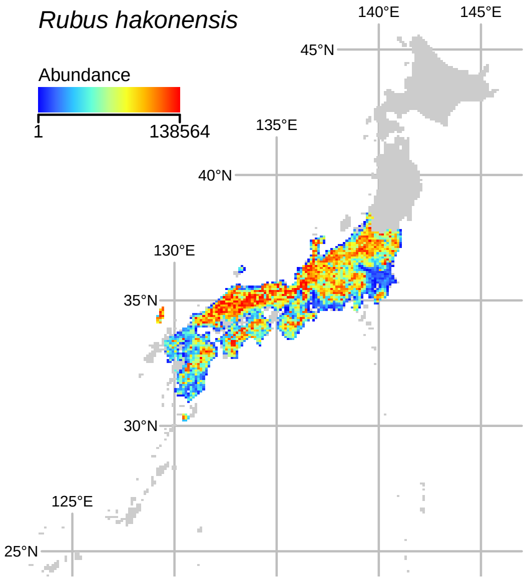
Rubus hakonensis
Abundance
1
138564
140°E
145°E
45°N
135°E
40°N
130°E
35°N
30°N
125°E
25°N

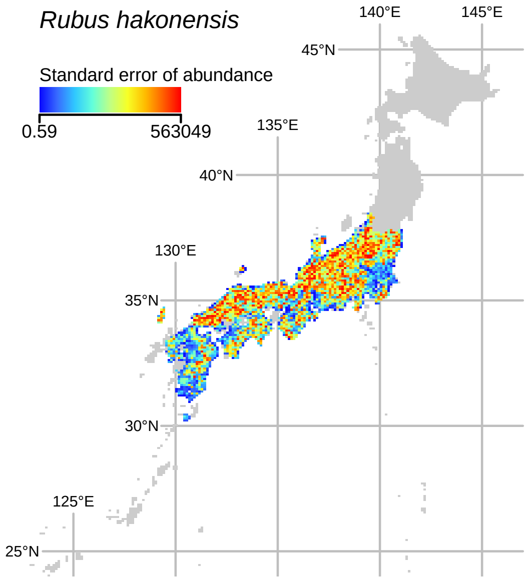
Rubus hakonensis
Standard error of abundance
0.59
563049
140°E
145°E
45°N
135°E
40°N
130°E
35°N
30°N
125°E
25°N

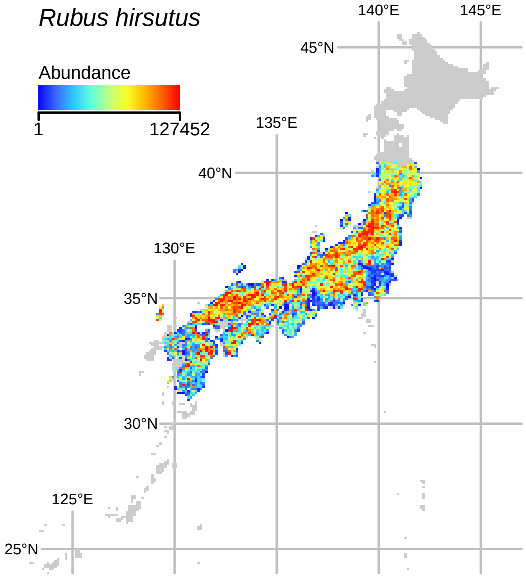
Rubus hirsutus
Abundance
1
127452
140°E
145°E
45°N
135°E
40°N
130°E
35°N
30°N
125°E
25°N

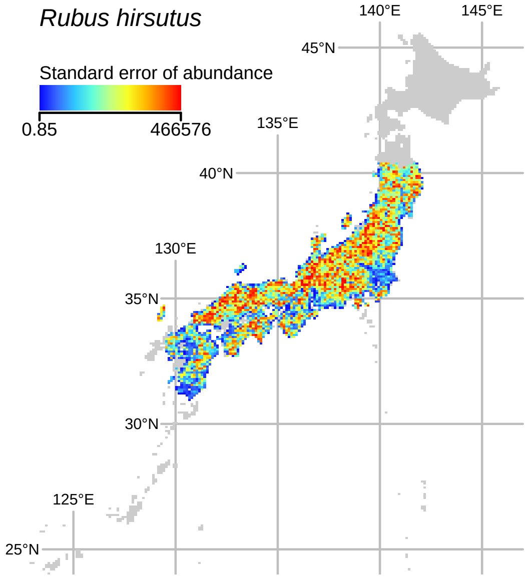
Rubus hirsutus
Standard error of abundance
0.85
466576
140°E
145°E
45°N
135°E
40°N
130°E
35°N
30°N
125°E
25°N

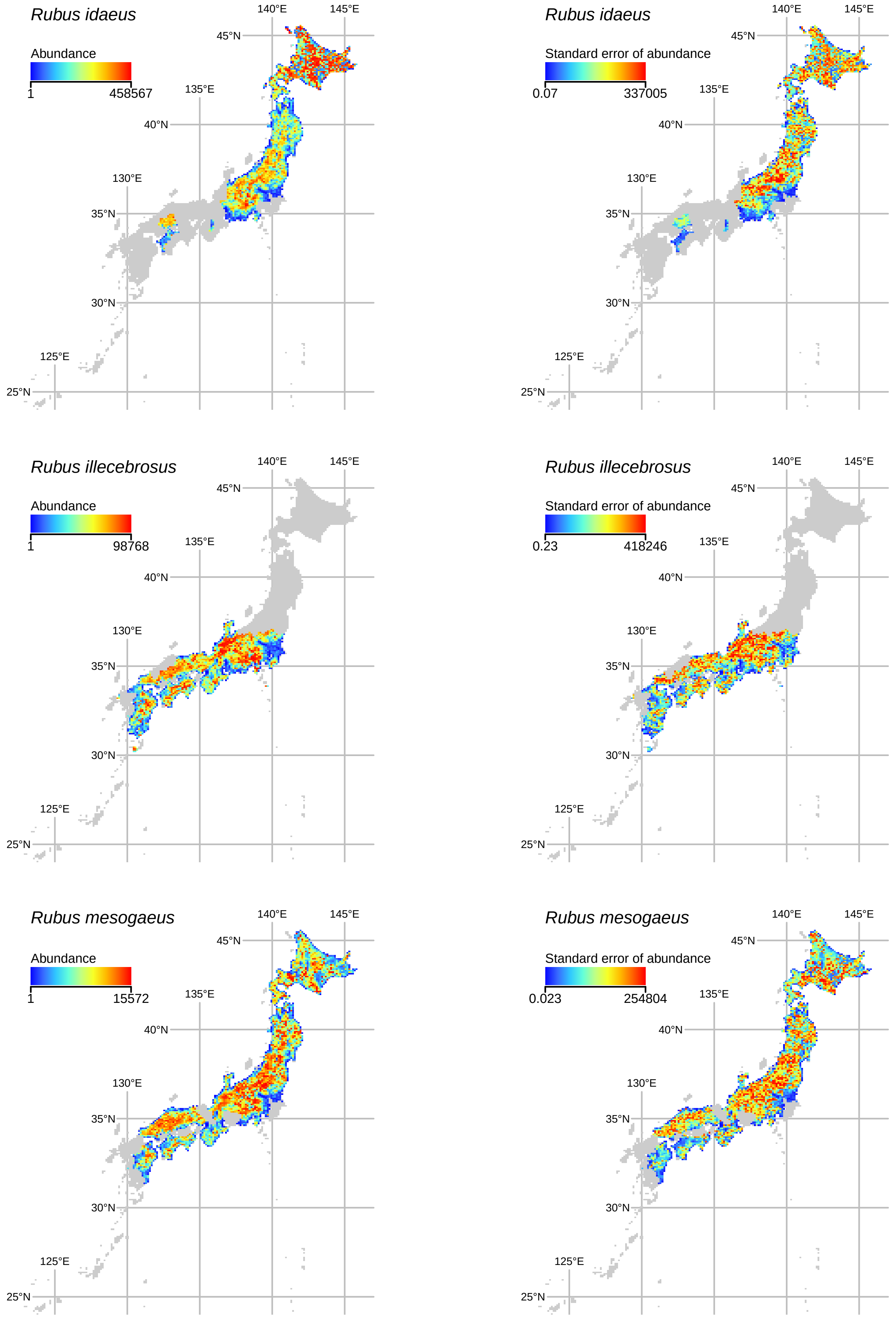
Rubus idaeus
Abundance
1
458567
Rubus idaeus
Standard error of abundance
0.07
337005
Rubus illecebrosus
Abundance
1
98768
Rubus illecebrosus
Standard error of abundance
0.23
418246
Rubus mesogaeus
Abundance
1
15572
Rubus mesogaeus
Standard error of abundance
0.023
254804
125°E
130°E
135°E
140°E
145°E
25°N
30°N
35°N
40°N
45°N

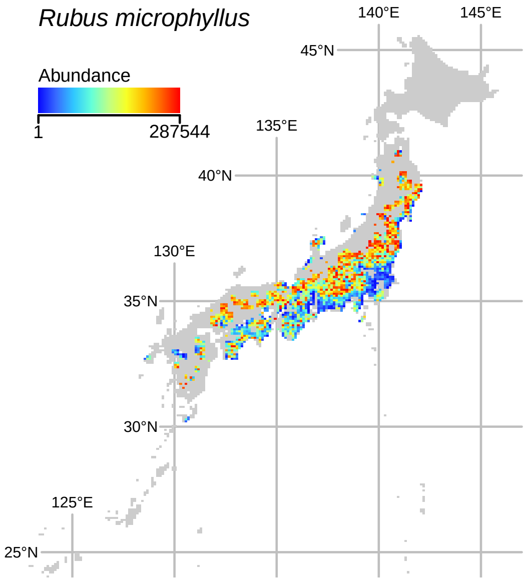
Rubus microphyllus
Abundance
1
287544
140°E
145°E
45°N
135°E
40°N
130°E
35°N
30°N
125°E
25°N

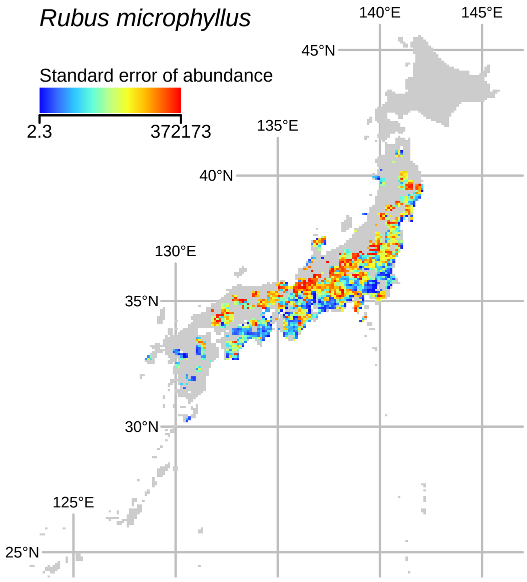
Rubus microphyllus
Standard error of abundance
2.3
372173
140°E
145°E
45°N
135°E
40°N
130°E
35°N
30°N
125°E
25°N

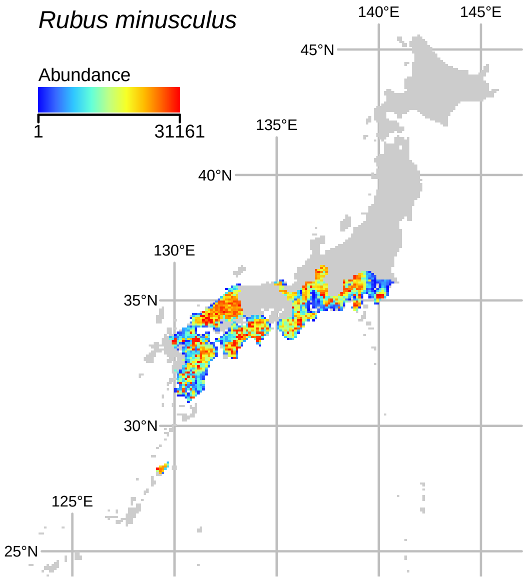
Rubus minusculus
Abundance
1
31161
140°E
145°E
45°N
135°E
40°N
130°E
35°N
30°N
125°E
25°N

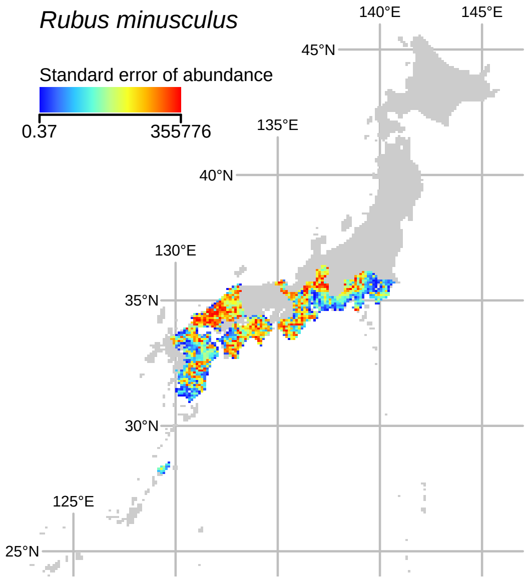
Rubus minusculus
Standard error of abundance
0.37
355776
140°E
145°E
45°N
135°E
40°N
130°E
35°N
30°N
125°E
25°N

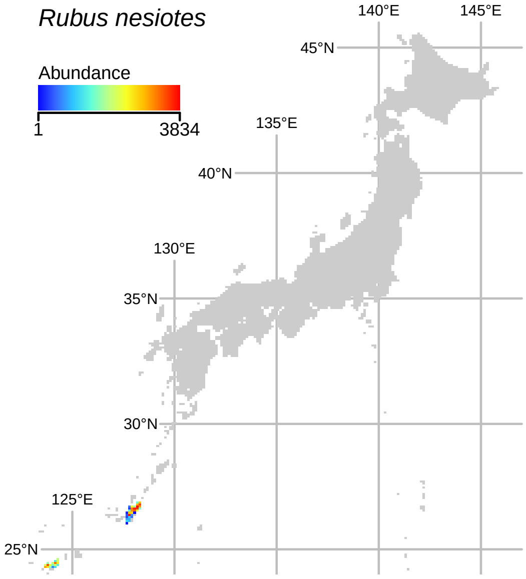
Rubus nesiotes
Abundance
1
3834
140°E
145°E
45°N
135°E
40°N
130°E
35°N
30°N
125°E
25°N

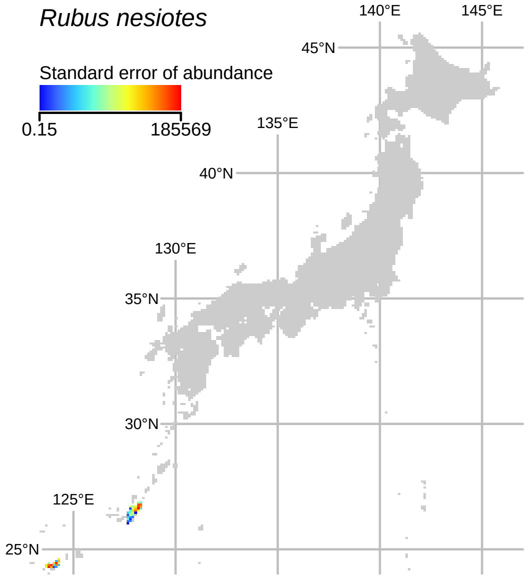
Rubus nesiotes
Standard error of abundance
0.15
185569
140°E
145°E
45°N
135°E
40°N
130°E
35°N
30°N
125°E
25°N

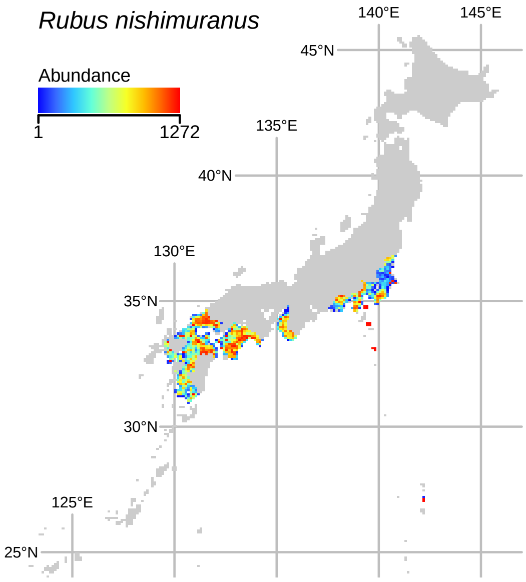
Rubus nishimuranus
Abundance
1
1272
140°E
145°E
135°E
130°E
125°E
45°N
40°N
35°N
30°N
25°N

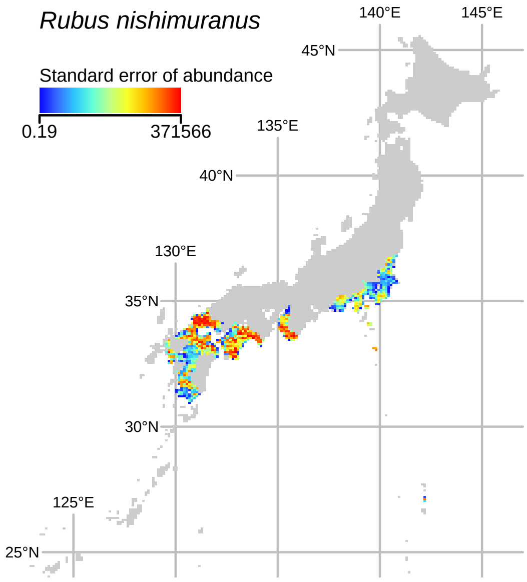
Rubus nishimuranus
Standard error of abundance
0.19
371566
140°E
145°E
135°E
130°E
125°E
45°N
40°N
35°N
30°N
25°N

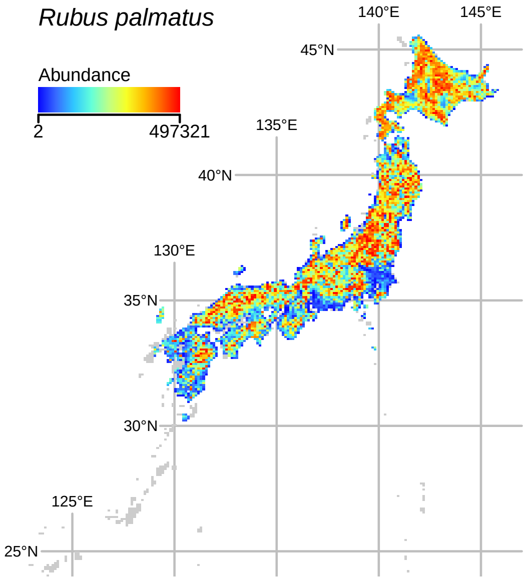
Rubus palmatus
Abundance
2
497321
140°E
145°E
135°E
130°E
125°E
45°N
40°N
35°N
30°N
25°N

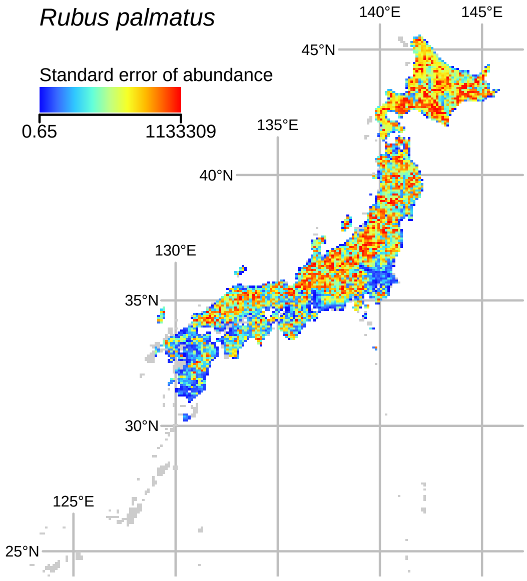
Rubus palmatus
Standard error of abundance
0.65
1133309
140°E
145°E
135°E
130°E
125°E
45°N
40°N
35°N
30°N
25°N

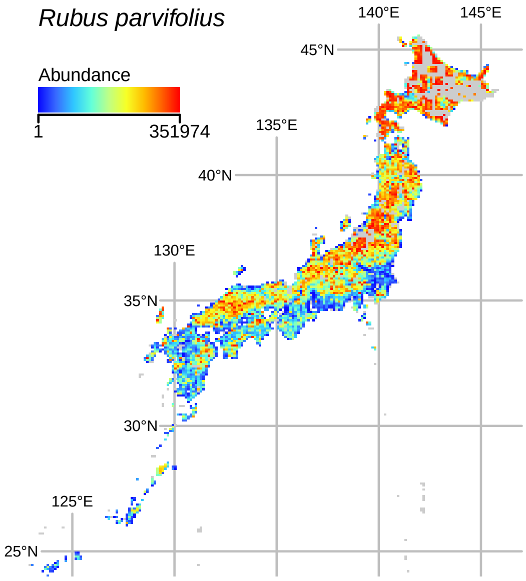
Rubus parvifolius
Abundance
1
351974
140°E
145°E
135°E
130°E
125°E
45°N
40°N
35°N
30°N
25°N

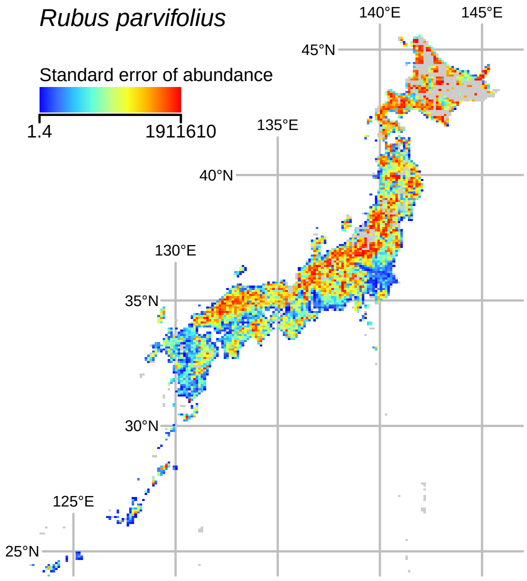
Rubus parvifolius
Standard error of abundance
1.4
1911610
140°E
145°E
135°E
130°E
125°E
45°N
40°N
35°N
30°N
25°N

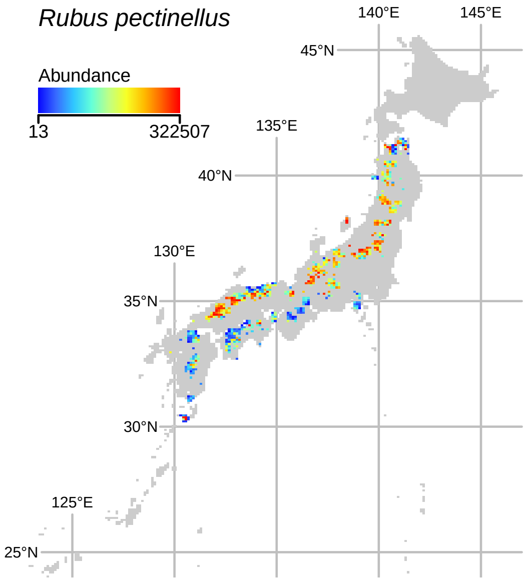
Rubus pectinellus
Abundance
13
322507
140°E
145°E
45°N
135°E
40°N
130°E
35°N
30°N
125°E
25°N

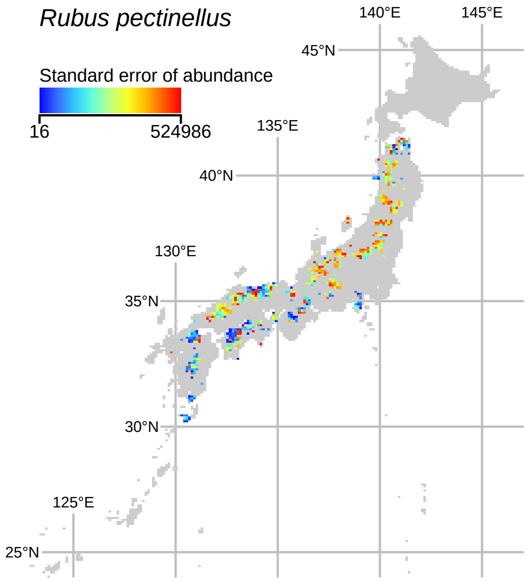
Rubus pectinellus
Standard error of abundance
16
524986
140°E
145°E
45°N
135°E
40°N
130°E
35°N
30°N
125°E
25°N

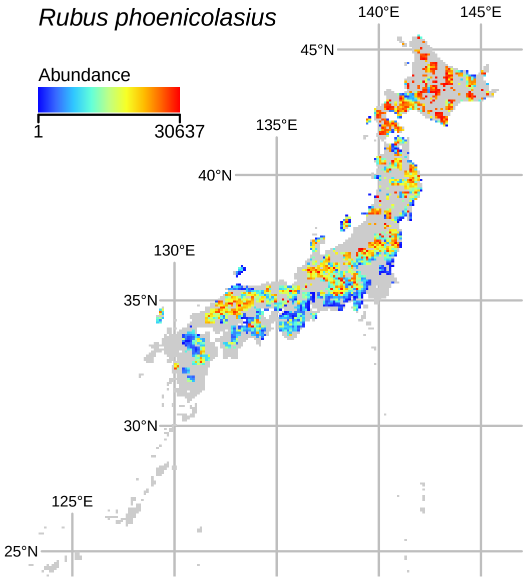
Rubus phoenicolasius
Abundance
1
30637
140°E
145°E
45°N
135°E
40°N
130°E
35°N
30°N
125°E
25°N

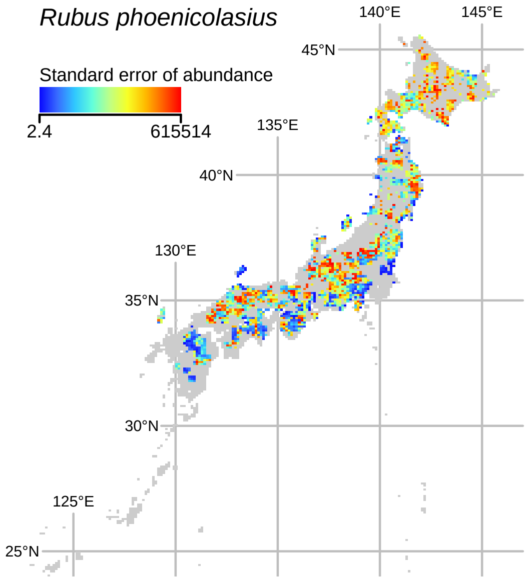
Rubus phoenicolasius
Standard error of abundance
2.4
615514
140°E
145°E
45°N
135°E
40°N
130°E
35°N
30°N
125°E
25°N

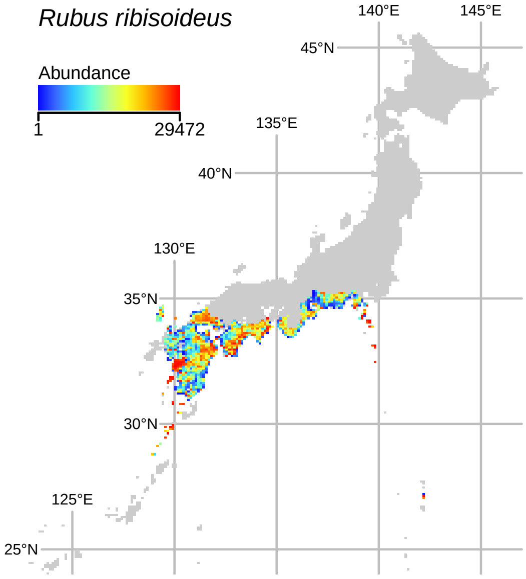
Rubus ribisoideus
Abundance
1
29472
140°E
145°E
45°N
135°E
40°N
130°E
35°N
30°N
125°E
25°N

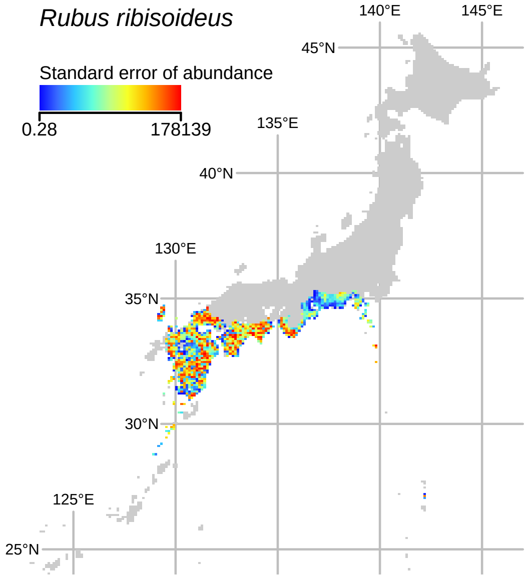
Rubus ribisoideus
Standard error of abundance
0.28
178139
140°E
145°E
45°N
135°E
40°N
130°E
35°N
30°N
125°E
25°N

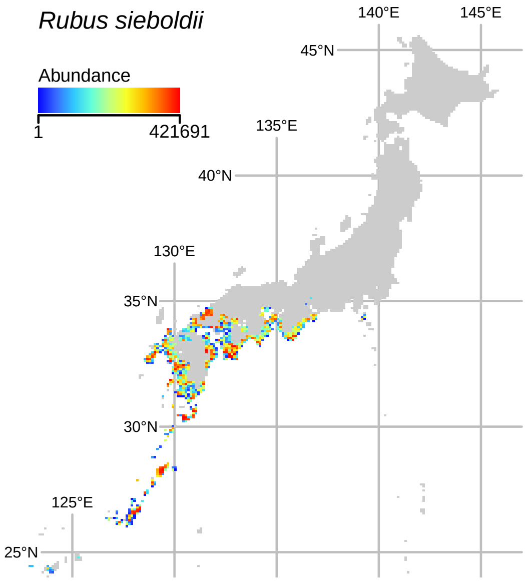
Rubus sieboldii
Abundance
1
421691
140°E
145°E
45°N
135°E
40°N
130°E
35°N
30°N
125°E
25°N

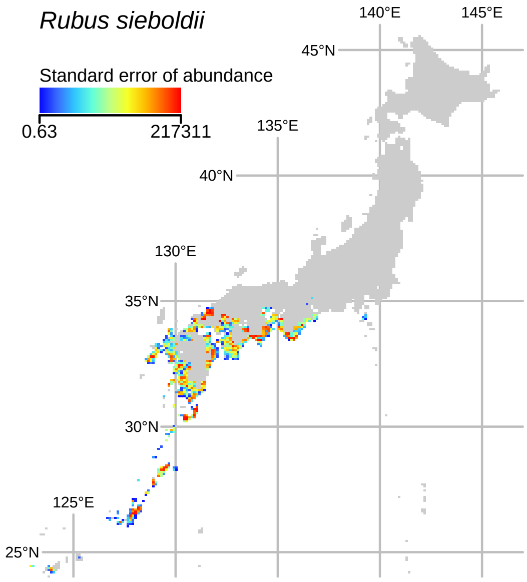
Rubus sieboldii
Standard error of abundance
0.63
217311
140°E
145°E
45°N
135°E
40°N
130°E
35°N
30°N
125°E
25°N

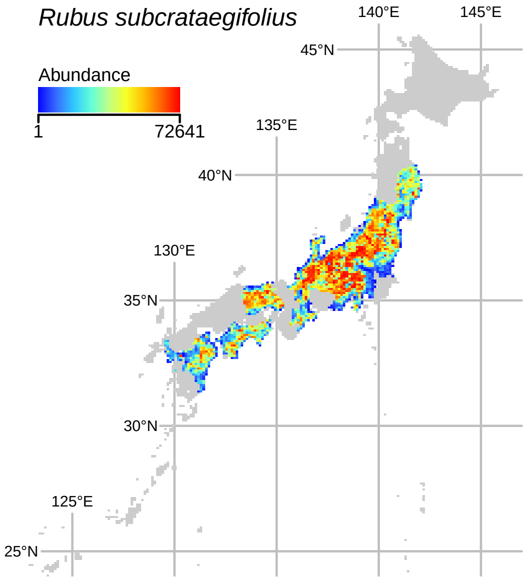
Rubus subcrataegifolius
Abundance
1
72641
140°E
145°E
45°N
135°E
40°N
130°E
35°N
30°N
125°E
25°N

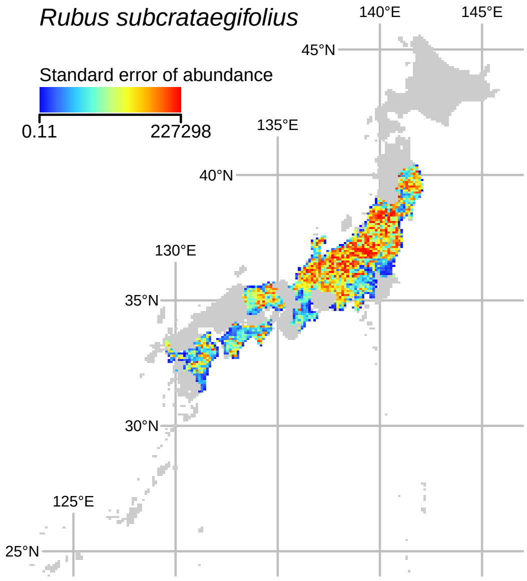
Rubus subcrataegifolius
Standard error of abundance
0.11
227298
140°E
145°E
45°N
135°E
40°N
130°E
35°N
30°N
125°E
25°N

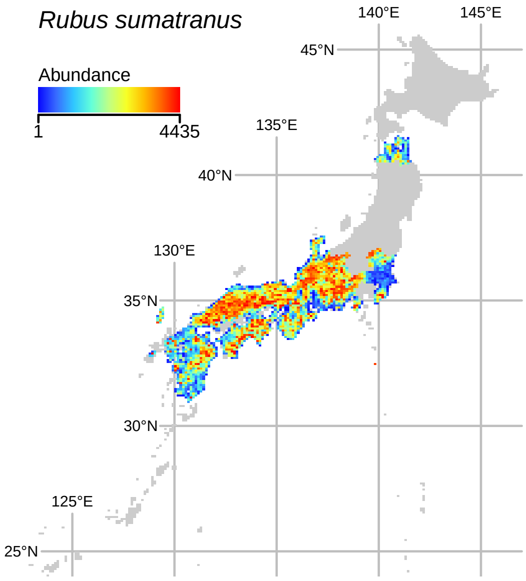
Rubus sumatranus
Abundance
1
4435
140°E
145°E
45°N
135°E
40°N
130°E
35°N
30°N
125°E
25°N

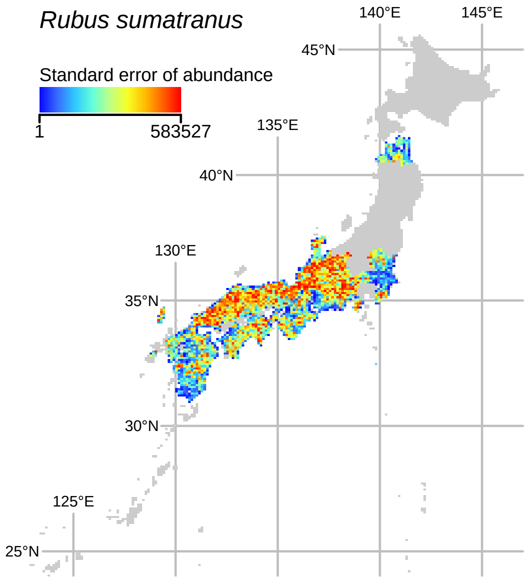
Rubus sumatranus
Standard error of abundance
1
583527
140°E
145°E
45°N
135°E
40°N
130°E
35°N
30°N
125°E
25°N

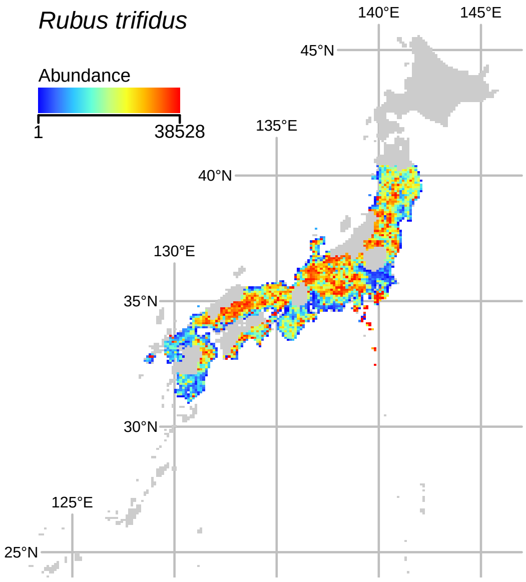
Rubus trifidus
Abundance
1
38528
140°E
145°E
45°N
135°E
40°N
130°E
35°N
30°N
125°E
25°N

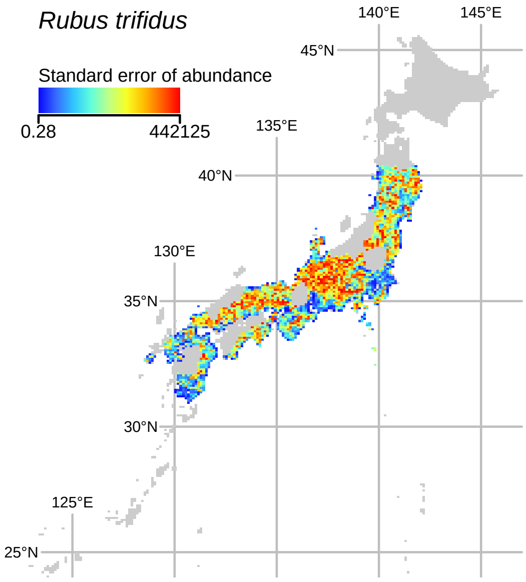
Rubus trifidus
Standard error of abundance
0.28
442125
140°E
145°E
45°N
135°E
40°N
130°E
35°N
30°N
125°E
25°N

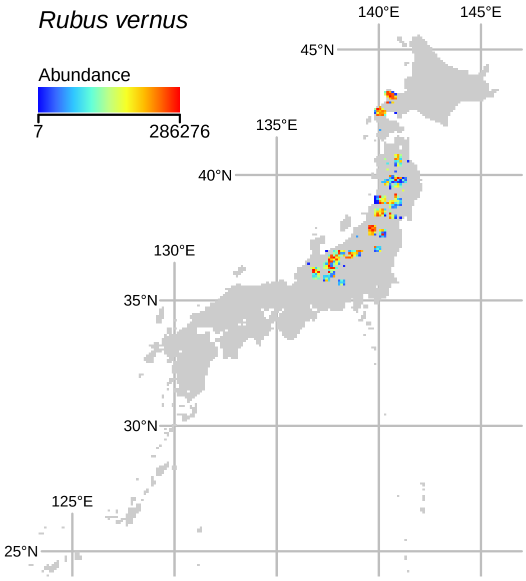
Rubus vernus
Abundance
7
286276
140°E
145°E
45°N
135°E
40°N
130°E
35°N
30°N
125°E
25°N

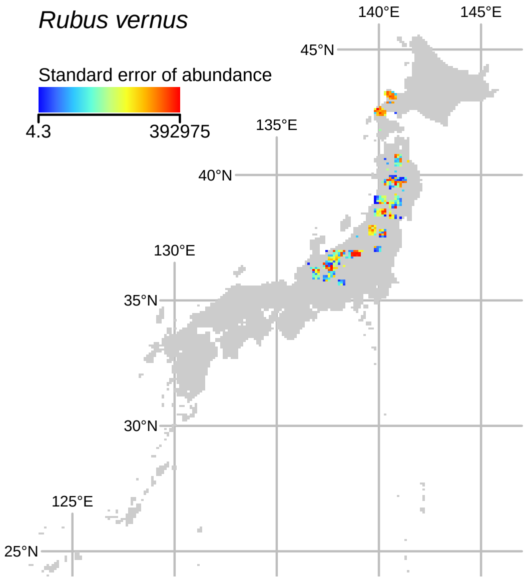
Rubus vernus
Standard error of abundance
4.3
392975
140°E
145°E
45°N
135°E
40°N
130°E
35°N
30°N
125°E
25°N

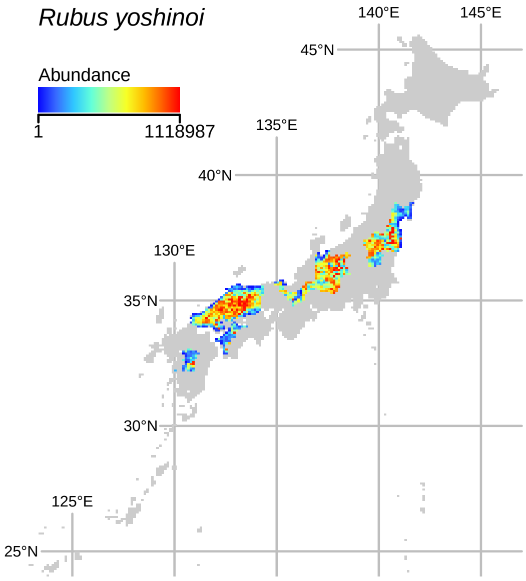
Rubus yoshinoi
Abundance
1
1118987
140°E
145°E
45°N
135°E
40°N
130°E
35°N
30°N
125°E
25°N

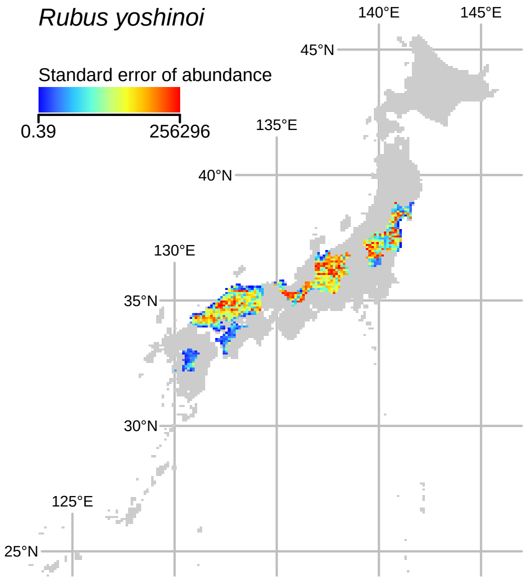
Rubus yoshinoi
Standard error of abundance
0.39
256296
140°E
145°E
45°N
135°E
40°N
130°E
35°N
30°N
125°E
25°N

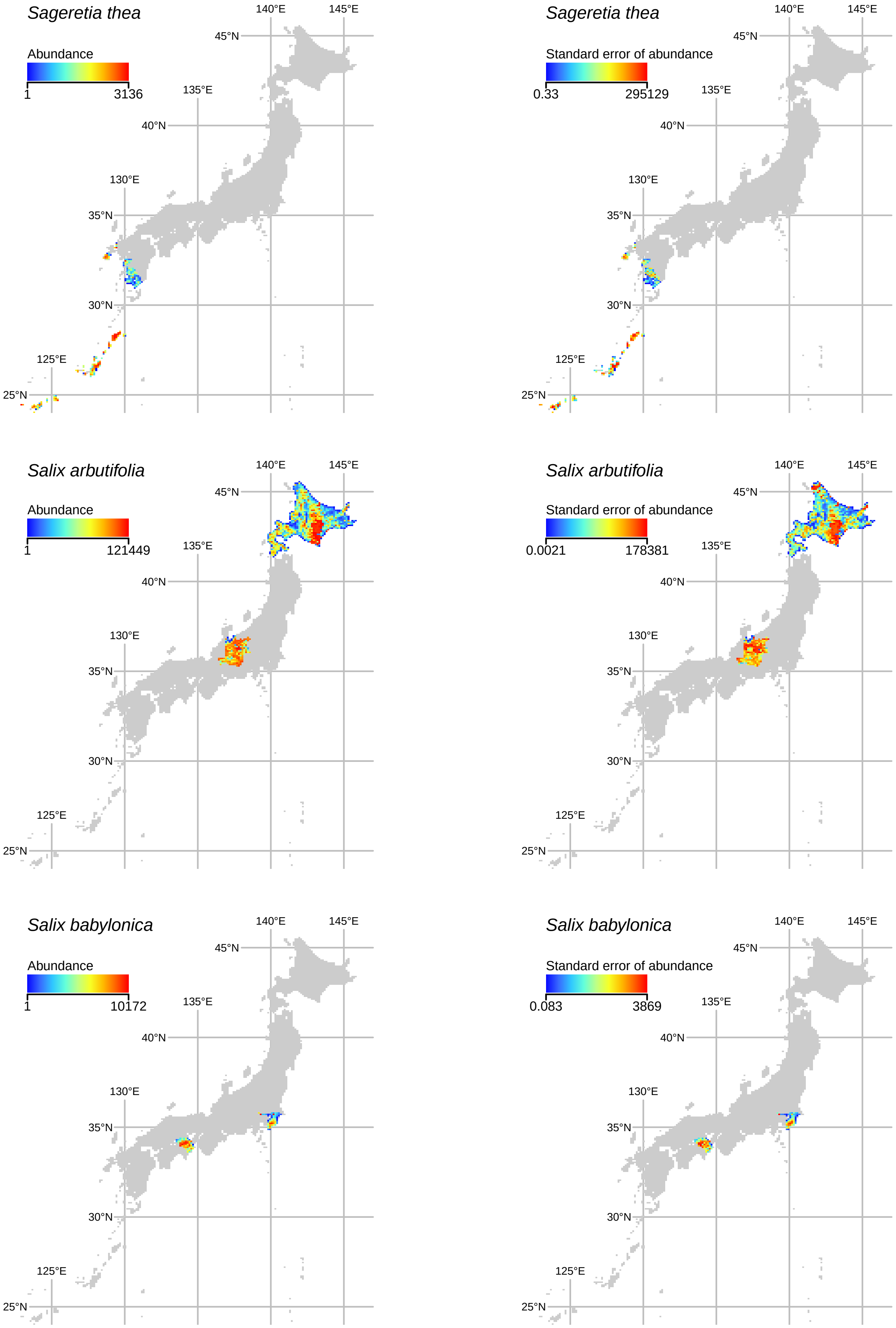
Sageretia thea
Abundance
1
3136
Sageretia thea
Standard error of abundance
0.33
295129
Salix arbutifolia
Abundance
1
121449
Salix arbutifolia
Standard error of abundance
0.0021
178381
Salix babylonica
Abundance
1
10172
Salix babylonica
Standard error of abundance
0.083
3869
140°E
145°E
45°N
135°E
40°N
130°E
35°N
30°N
125°E
25°N

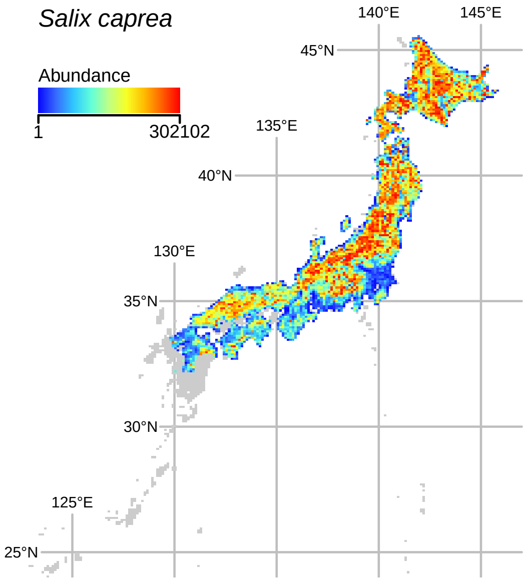
Salix caprea
Abundance
1
302102
140°E
145°E
45°N
135°E
40°N
130°E
35°N
30°N
125°E
25°N

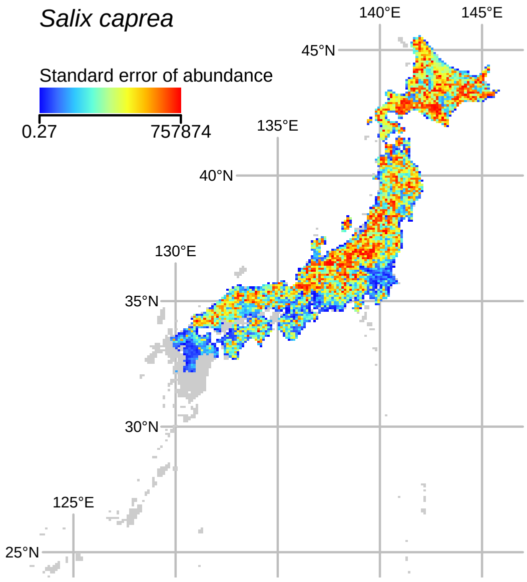
Salix caprea
Standard error of abundance
0.27
757874
140°E
145°E
45°N
135°E
40°N
130°E
35°N
30°N
125°E
25°N

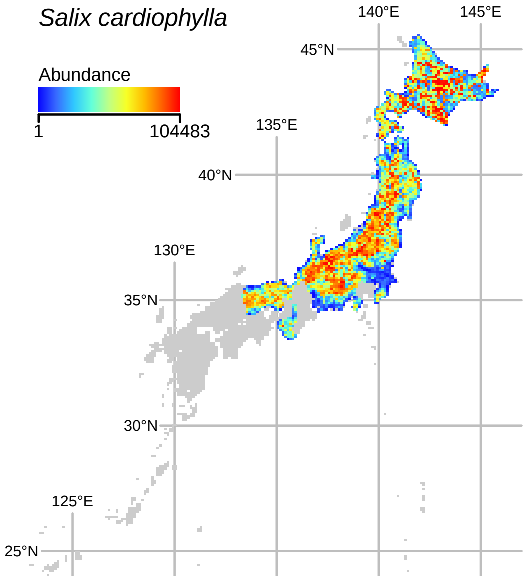
Salix cardiophylla
Abundance
1
104483
140°E
145°E
45°N
135°E
40°N
130°E
35°N
30°N
125°E
25°N

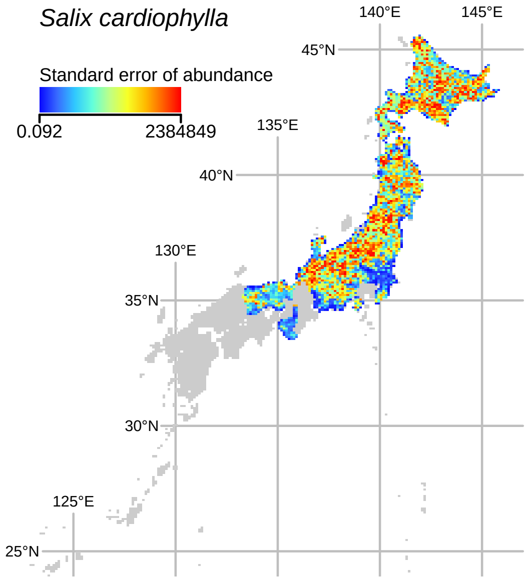
Salix cardiophylla
Standard error of abundance
0.092
2384849
140°E
145°E
45°N
135°E
40°N
130°E
35°N
30°N
125°E
25°N

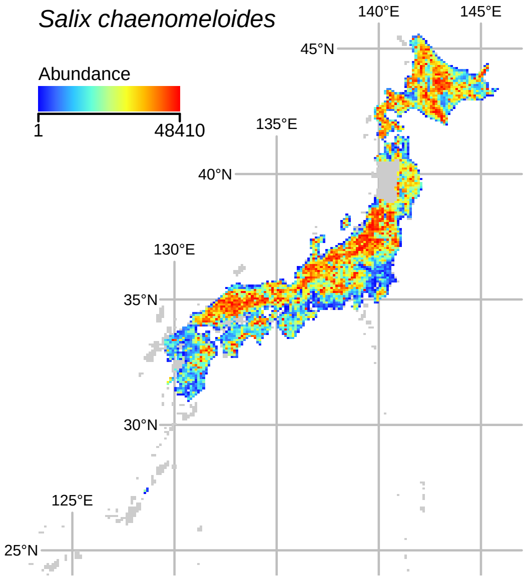
Salix chaenomeloides
Abundance
1
48410
140°E
145°E
45°N
135°E
40°N
130°E
35°N
30°N
125°E
25°N

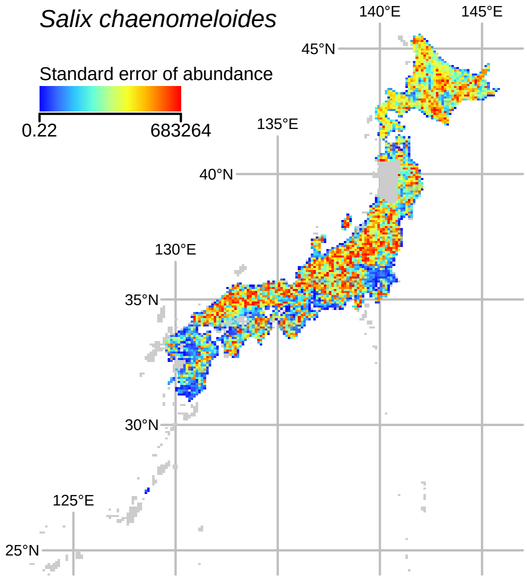
Salix chaenomeloides
Standard error of abundance
0.22
683264
140°E
145°E
45°N
135°E
40°N
130°E
35°N
30°N
125°E
25°N

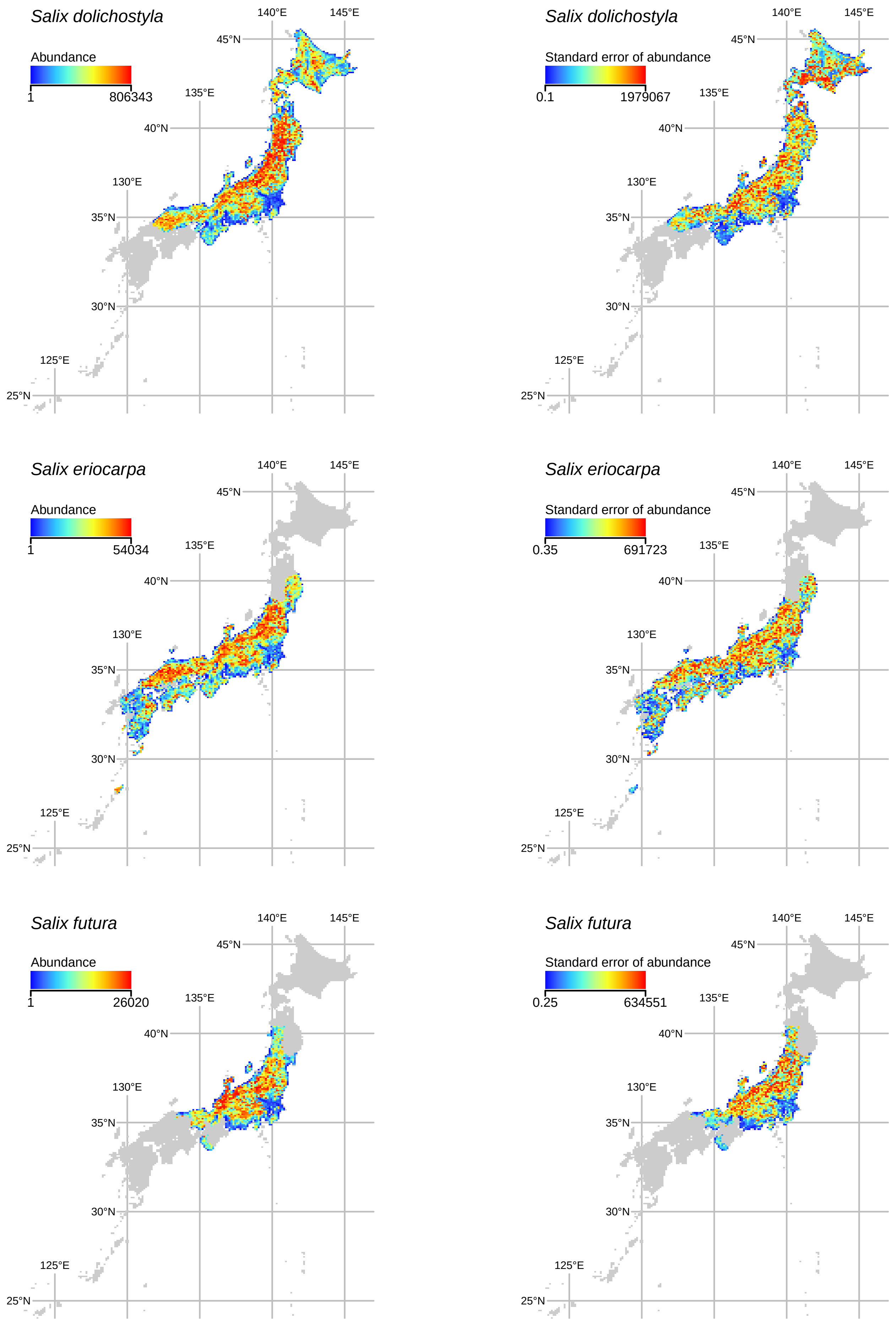
Salix dolichostyla
Abundance
1
806343
Salix dolichostyla
Standard error of abundance
0.1
1979067
Salix eriocarpa
Abundance
1
54034
Salix eriocarpa
Standard error of abundance
0.35
691723
Salix futura
Abundance
1
26020
Salix futura
Standard error of abundance
0.25
634551
125°E
130°E
135°E
140°E
145°E
25°N
30°N
35°N
40°N
45°N

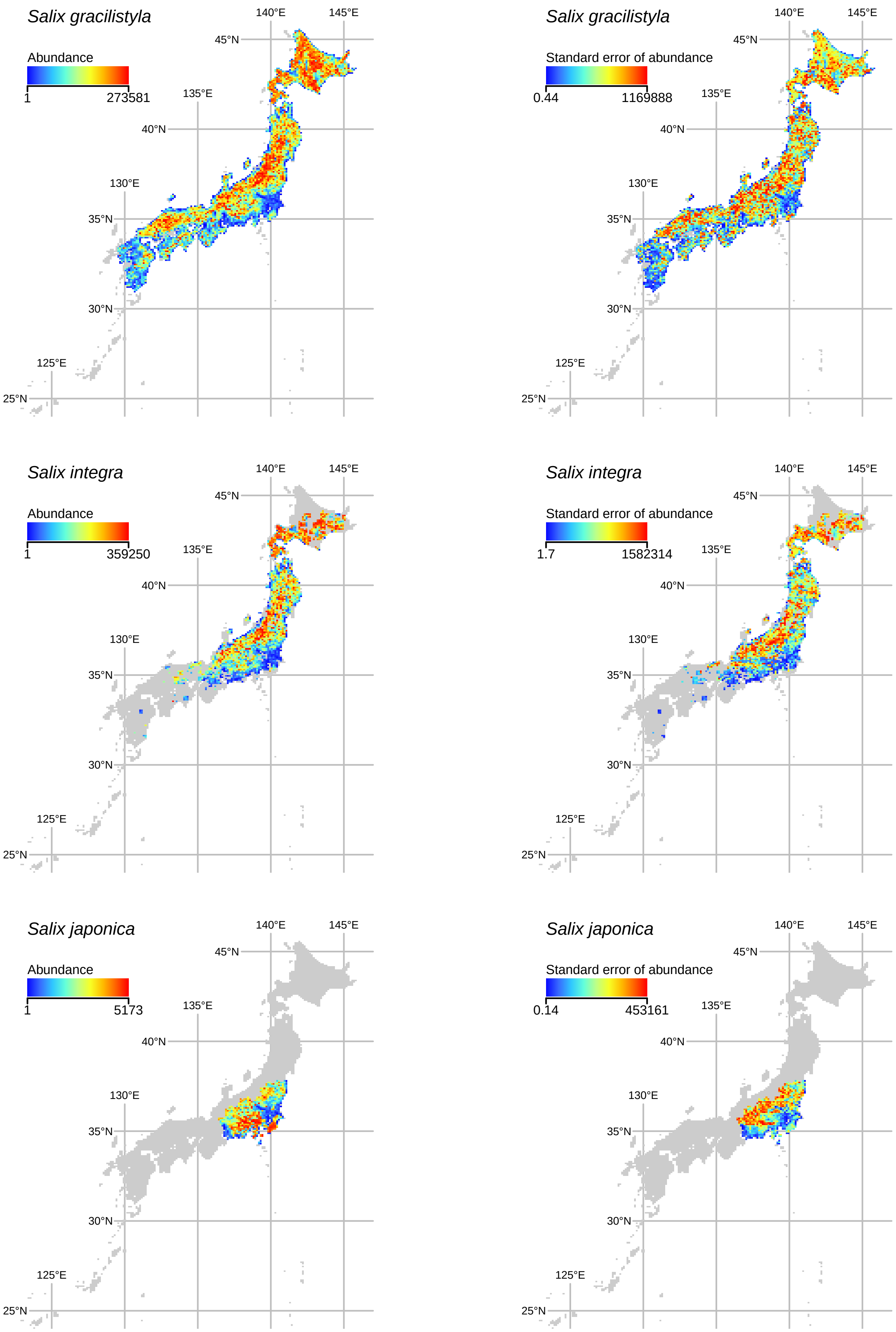
Salix gracilistyla
Abundance
1
273581
Salix gracilistyla
Standard error of abundance
0.44
1169888
Salix integra
Abundance
1
359250
Salix integra
Standard error of abundance
1.7
1582314
Salix japonica
Abundance
1
5173
Salix japonica
Standard error of abundance
0.14
453161
125°E
130°E
135°E
140°E
145°E
25°N
30°N
35°N
40°N
45°N

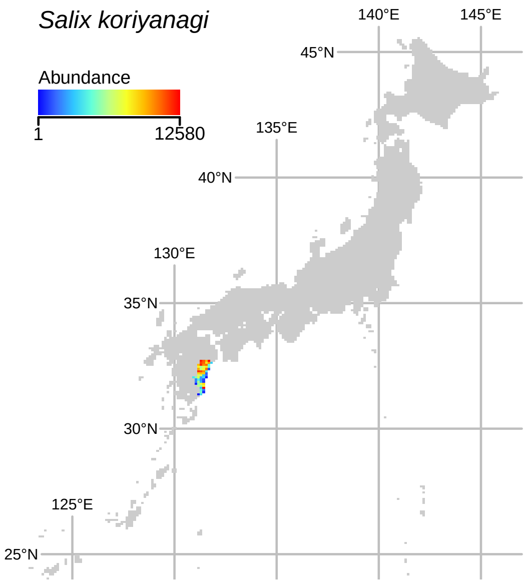
Salix koriyanagi
Abundance
1
12580
140°E
145°E
45°N
135°E
40°N
130°E
35°N
30°N
125°E
25°N

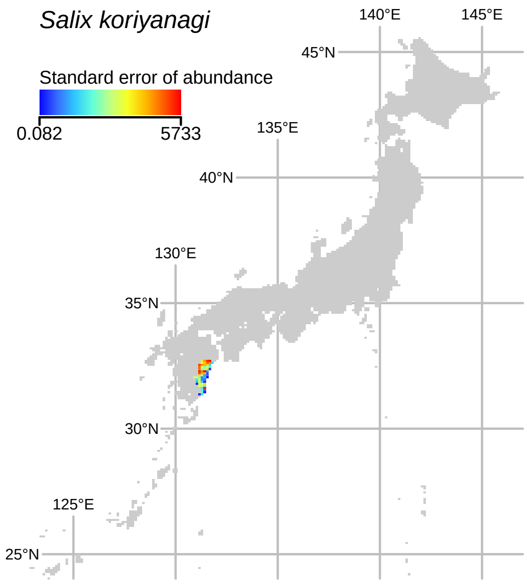
Salix koriyanagi
Standard error of abundance
0.082
5733
140°E
145°E
45°N
135°E
40°N
130°E
35°N
30°N
125°E
25°N

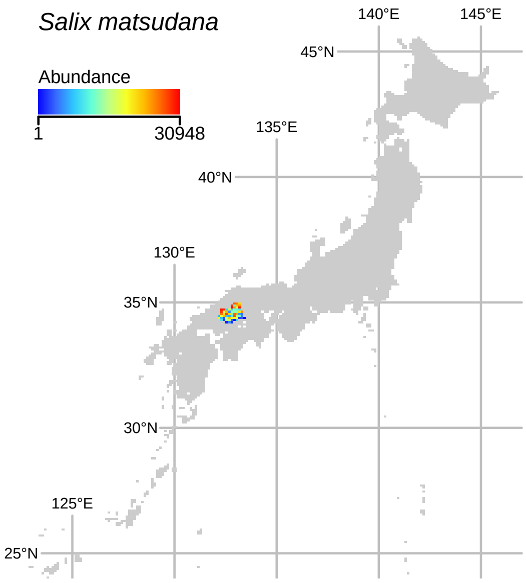
Salix matsudana
Abundance
1
30948
140°E
145°E
45°N
135°E
40°N
130°E
35°N
30°N
125°E
25°N

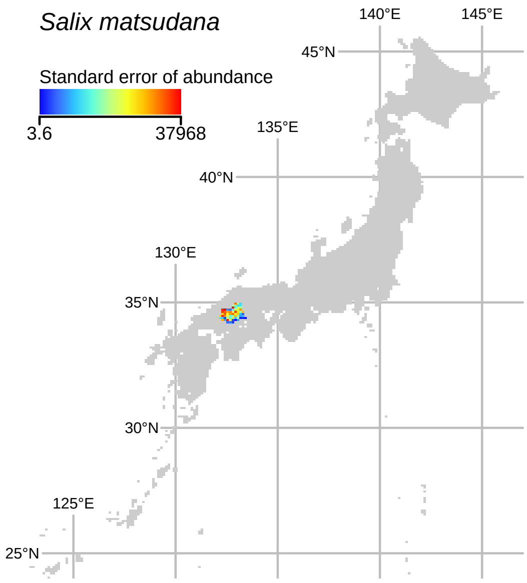
Salix matsudana
Standard error of abundance
3.6
37968
140°E
145°E
45°N
135°E
40°N
130°E
35°N
30°N
125°E
25°N

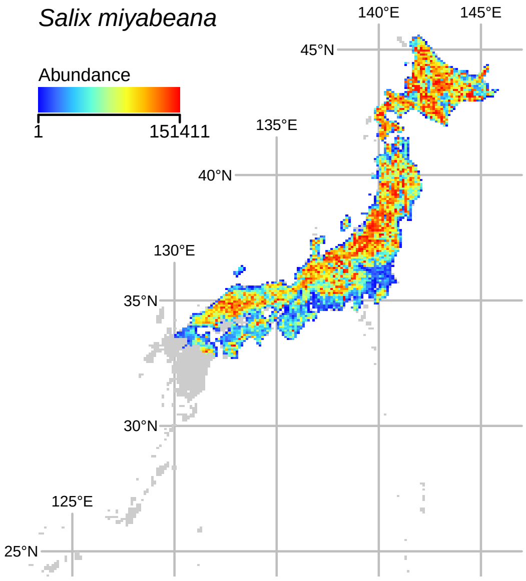
Salix miyabeana
Abundance
1
151411
140°E
145°E
45°N
135°E
40°N
130°E
35°N
30°N
125°E
25°N

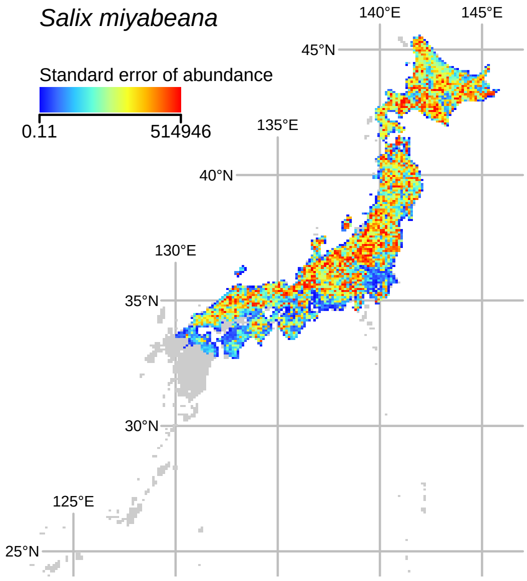
Salix miyabeana
Standard error of abundance
0.11
514946
140°E
145°E
45°N
135°E
40°N
130°E
35°N
30°N
125°E
25°N

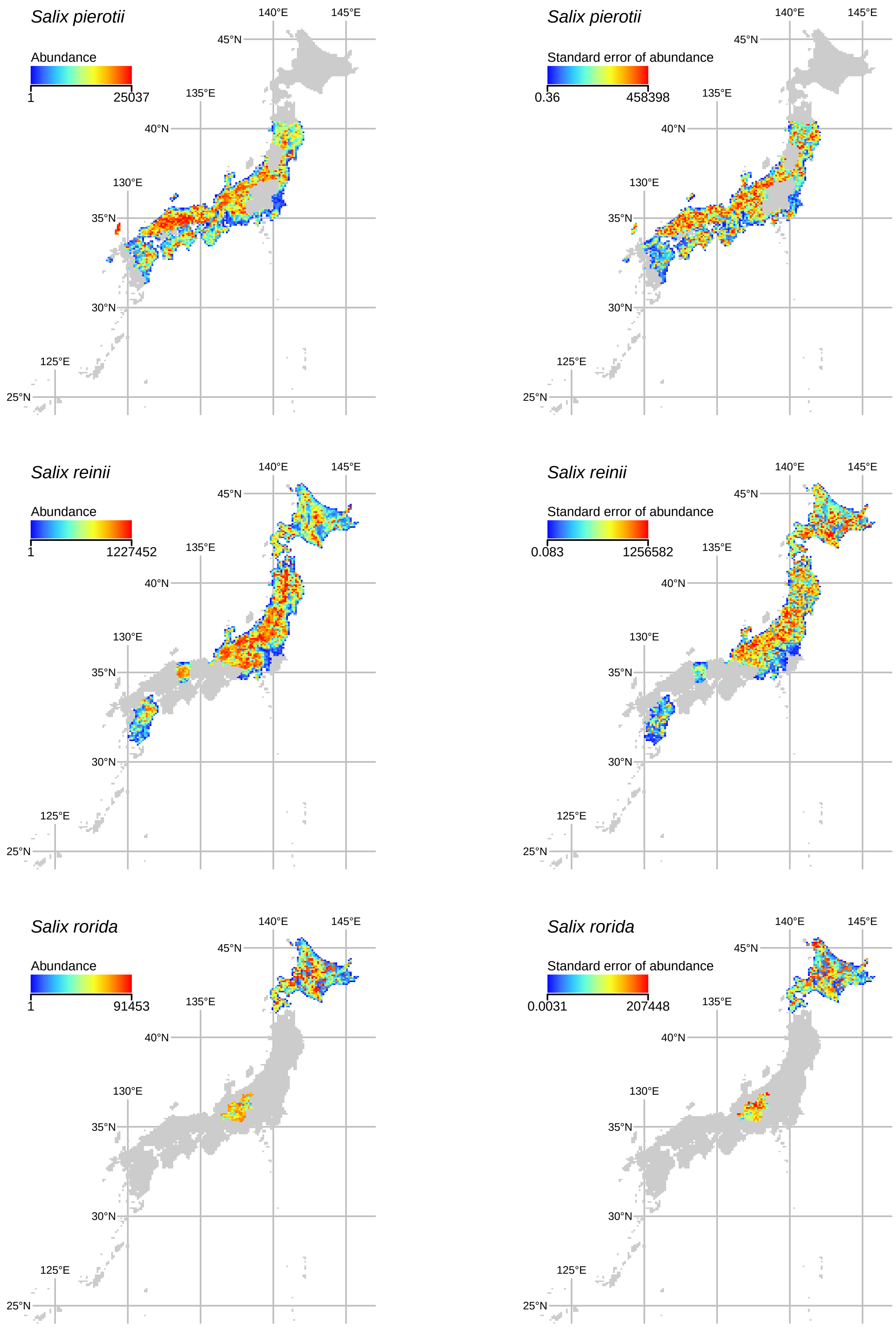
Salix pierotii
Abundance
1
25037
Salix pierotii
Standard error of abundance
0.36
458398
Salix reinii
Abundance
1
1227452
Salix reinii
Standard error of abundance
0.083
1256582
Salix rorida
Abundance
1
91453
Salix rorida
Standard error of abundance
0.0031
207448
125°E
130°E
135°E
140°E
145°E
25°N
30°N
35°N
40°N
45°N

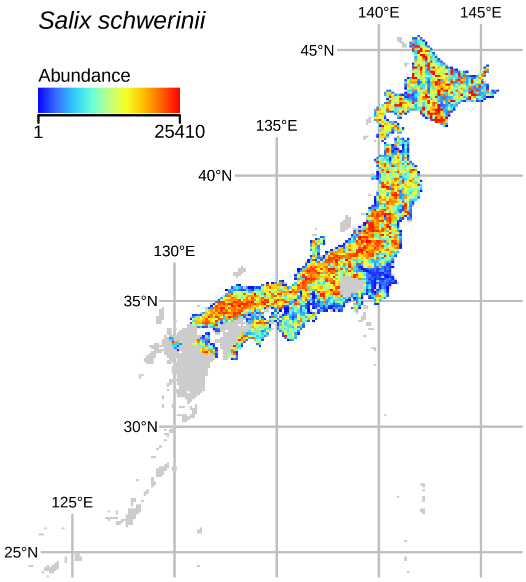
Salix schwerinii
Abundance
1
25410
140°E
145°E
135°E
130°E
125°E
45°N
40°N
35°N
30°N
25°N

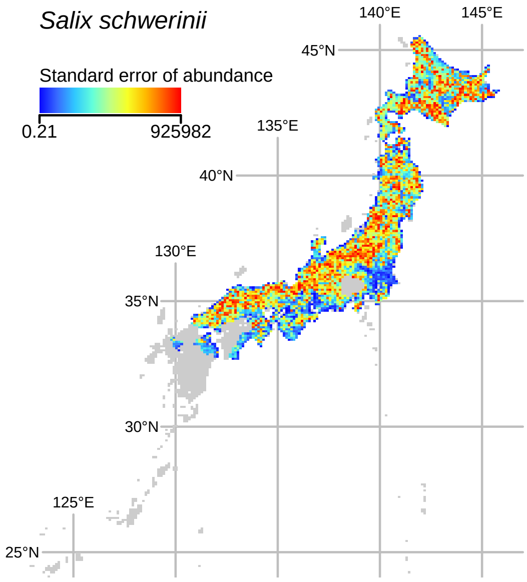
Salix schwerinii
Standard error of abundance
0.21
925982
140°E
145°E
135°E
130°E
125°E
45°N
40°N
35°N
30°N
25°N

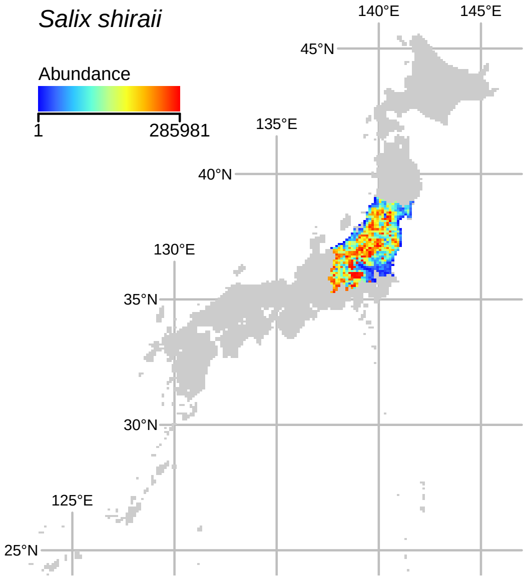
Salix shiraii
Abundance
1
285981
140°E
145°E
135°E
130°E
125°E
45°N
40°N
35°N
30°N
25°N

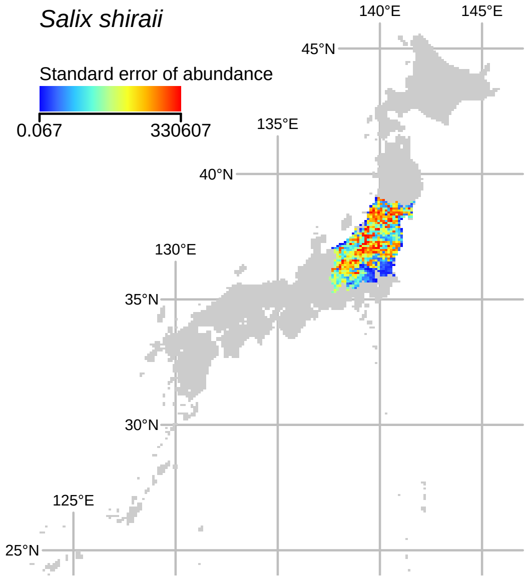
Salix shiraii
Standard error of abundance
0.067
330607
140°E
145°E
135°E
130°E
125°E
45°N
40°N
35°N
30°N
25°N

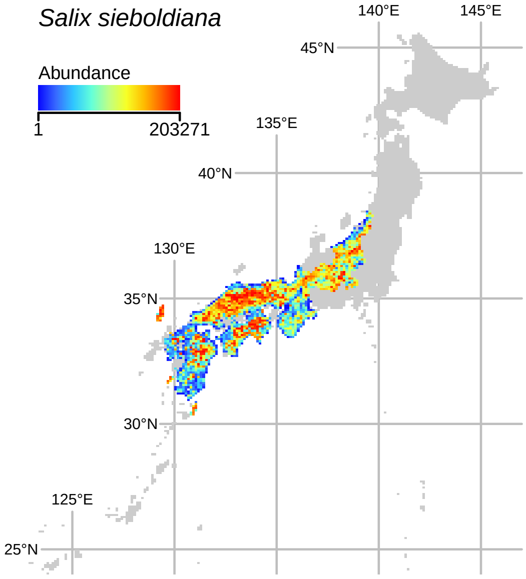
Salix sieboldiana
Abundance
1
203271
140°E
145°E
135°E
130°E
125°E
45°N
40°N
35°N
30°N
25°N

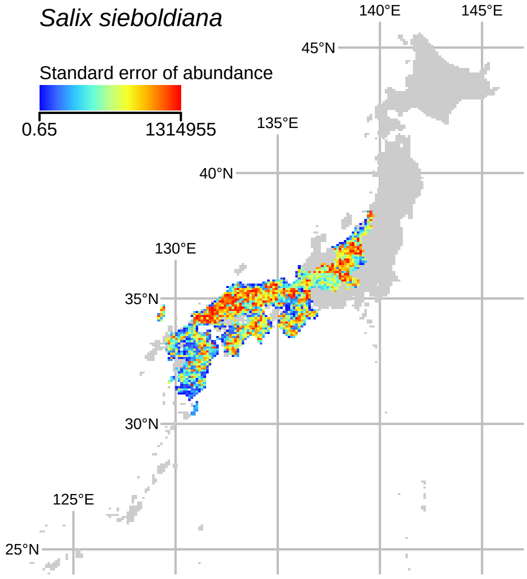
Salix sieboldiana
Standard error of abundance
0.65
1314955
140°E
145°E
135°E
130°E
125°E
45°N
40°N
35°N
30°N
25°N

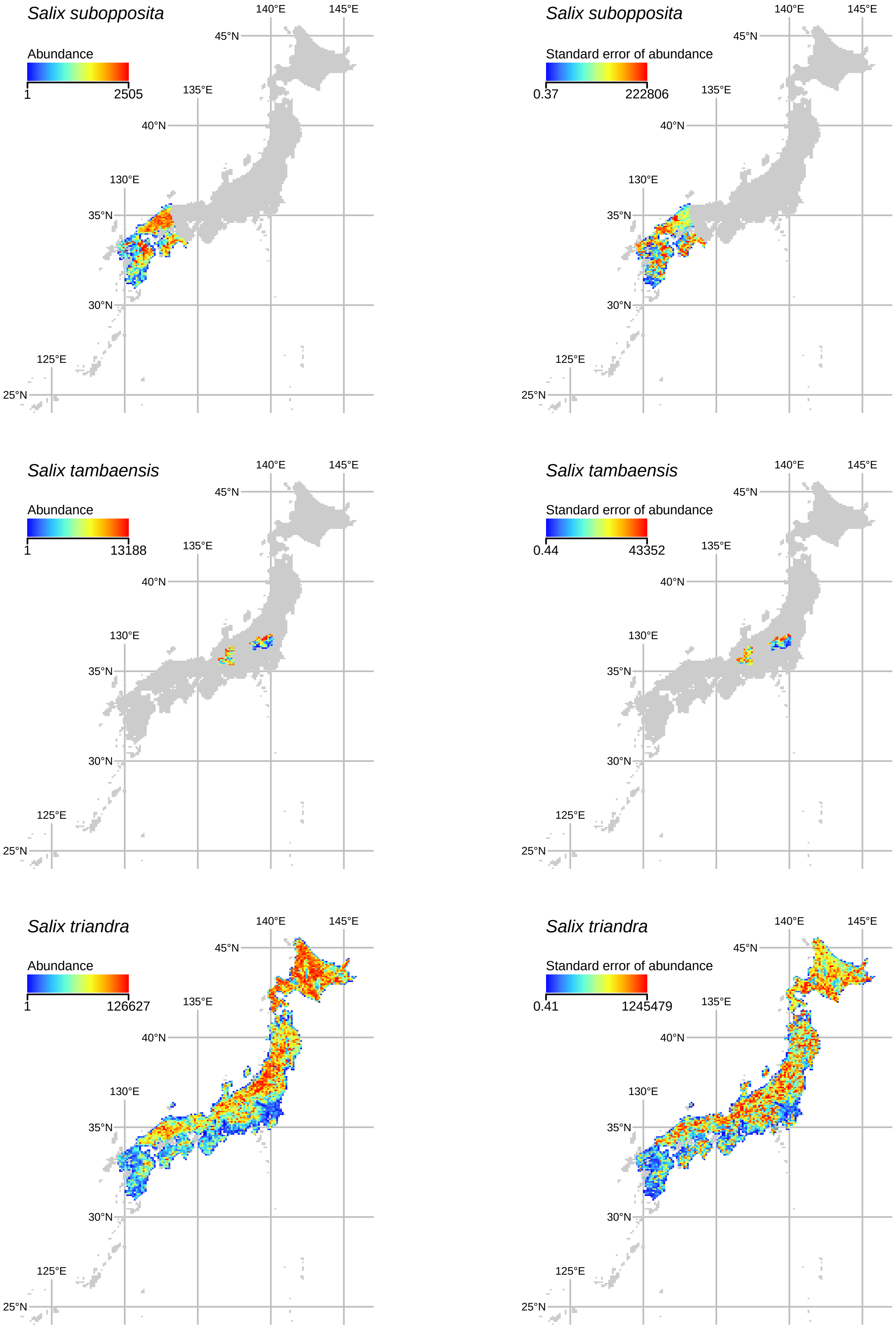
Salix subopposita
Abundance
1
2505
Salix subopposita
Standard error of abundance
0.37
222806
Salix tambaensis
Abundance
1
13188
Salix tambaensis
Standard error of abundance
0.44
43352
Salix triandra
Abundance
1
126627
Salix triandra
Standard error of abundance
0.41
1245479
125°E
130°E
135°E
140°E
145°E
25°N
30°N
35°N
40°N
45°N

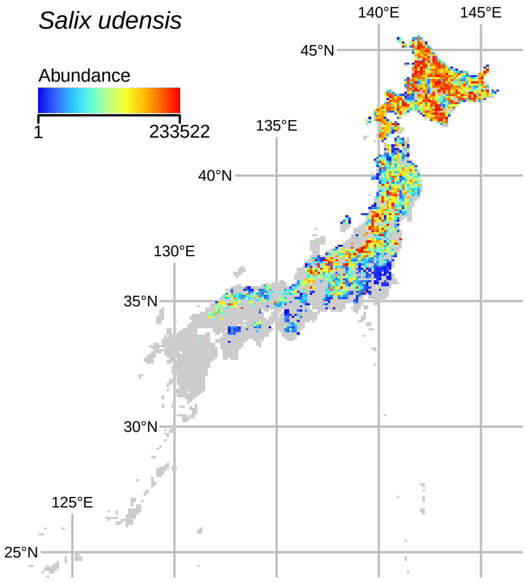
Salix udensis
Abundance
1
233522
140°E
145°E
135°E
130°E
125°E
45°N
40°N
35°N
30°N
25°N

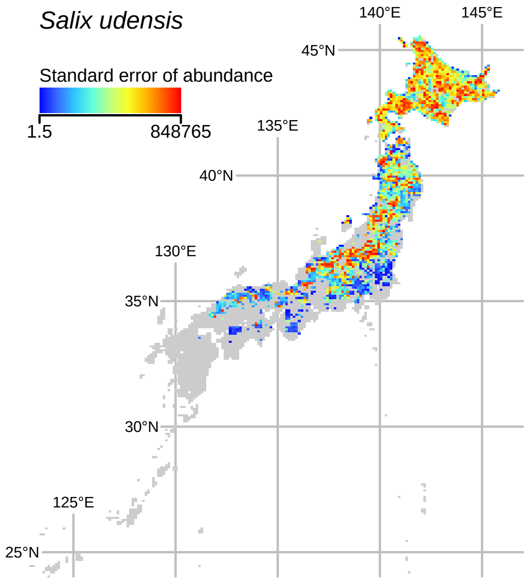
Salix udensis
Standard error of abundance
1.5
848765
140°E
145°E
135°E
130°E
125°E
45°N
40°N
35°N
30°N
25°N

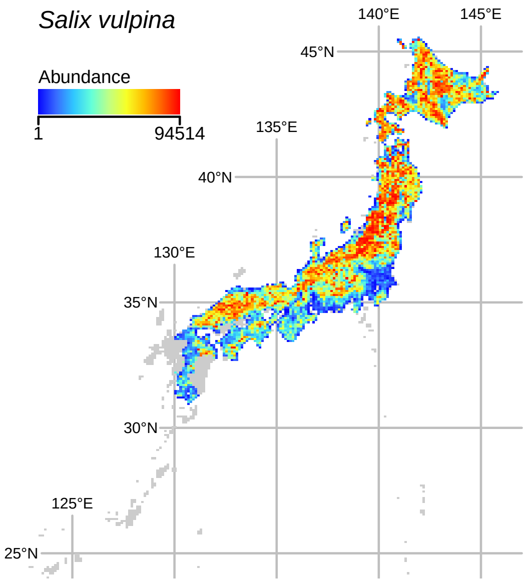
Salix vulpina
Abundance
1
94514
140°E
145°E
135°E
130°E
125°E
45°N
40°N
35°N
30°N
25°N

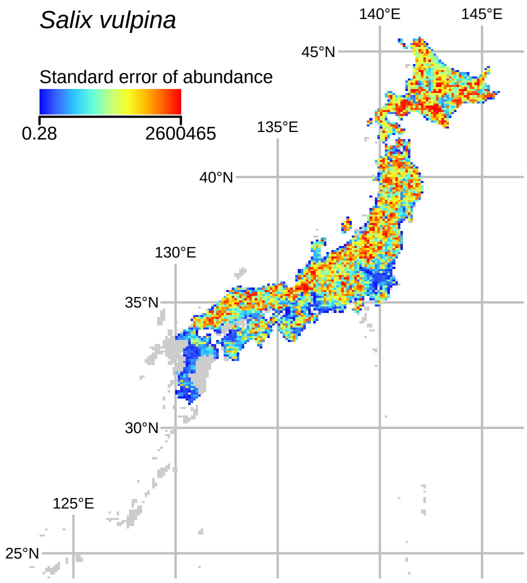
Salix vulpina
Standard error of abundance
0.28
2600465
140°E
145°E
135°E
130°E
125°E
45°N
40°N
35°N
30°N
25°N

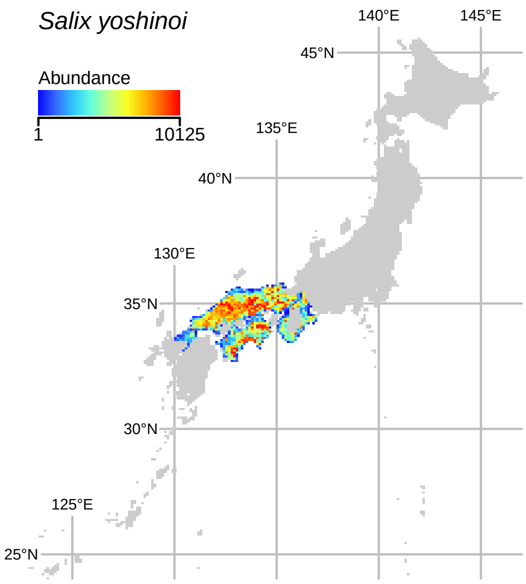
Salix yoshinoi
Abundance
1
10125
140°E
145°E
135°E
130°E
125°E
45°N
40°N
35°N
30°N
25°N

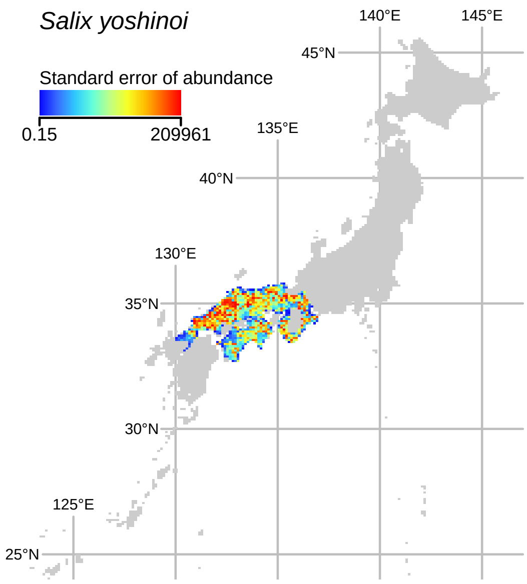
Salix yoshinoi
Standard error of abundance
0.15
209961
140°E
145°E
135°E
130°E
125°E
45°N
40°N
35°N
30°N
25°N

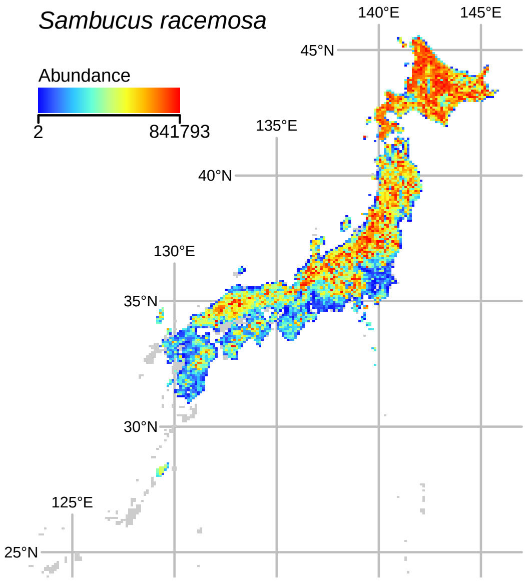
Sambucus racemosa
Abundance
2
841793
140°E
145°E
45°N
135°E
40°N
130°E
35°N
30°N
125°E
25°N

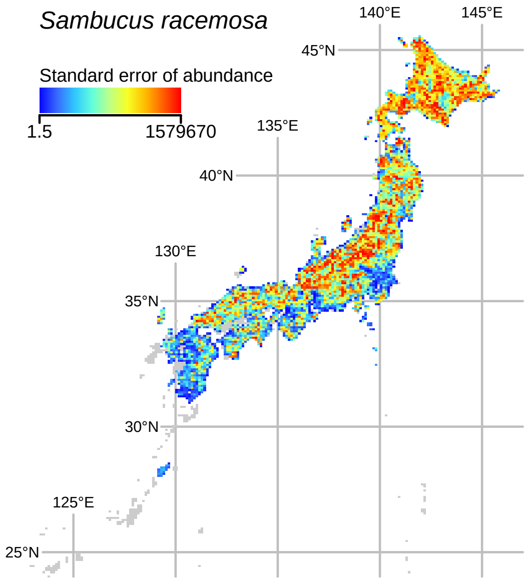
Sambucus racemosa
Standard error of abundance
1.5
1579670
140°E
145°E
45°N
135°E
40°N
130°E
35°N
30°N
125°E
25°N

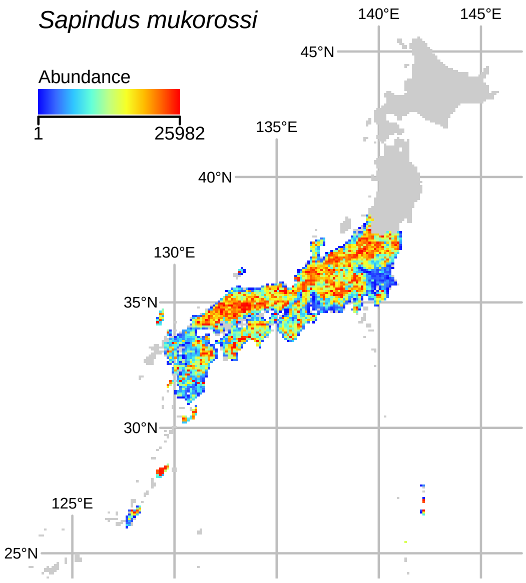
Sapindus mukorossi
Abundance
1
25982
140°E
145°E
45°N
135°E
40°N
130°E
35°N
30°N
125°E
25°N

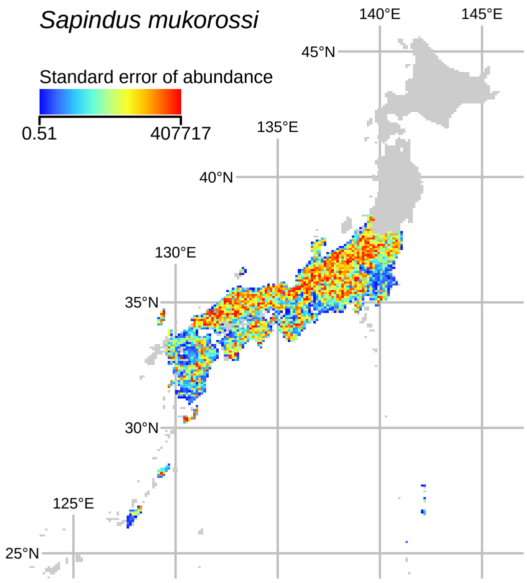
Sapindus mukorossi
Standard error of abundance
0.51
407717
140°E
145°E
45°N
135°E
40°N
130°E
35°N
30°N
125°E
25°N

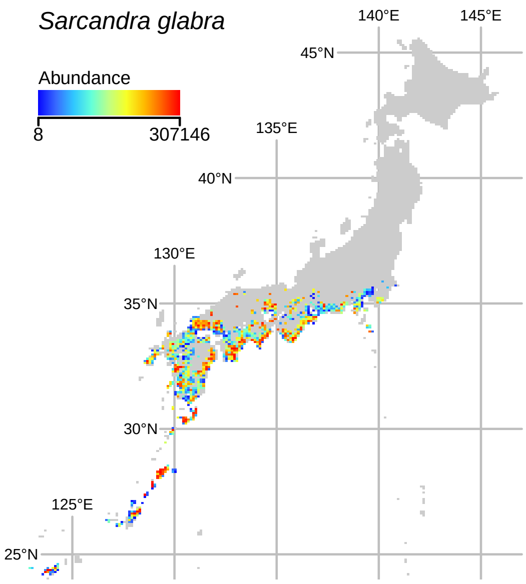
Sarcandra glabra
Abundance
8
307146
140°E
145°E
45°N
135°E
40°N
130°E
35°N
30°N
125°E
25°N

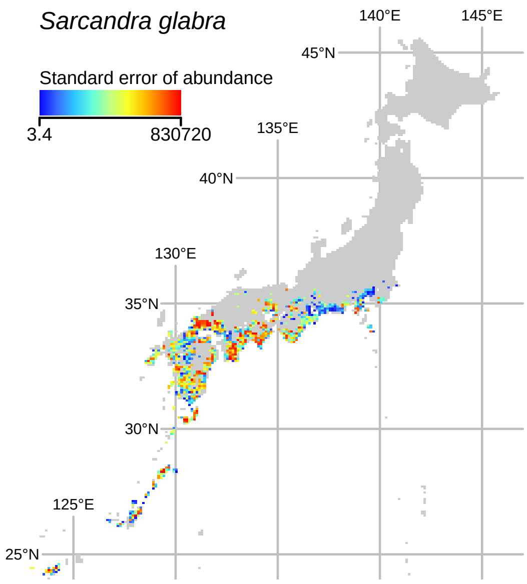
Sarcandra glabra
Standard error of abundance
3.4
830720
140°E
145°E
45°N
135°E
40°N
130°E
35°N
30°N
125°E
25°N

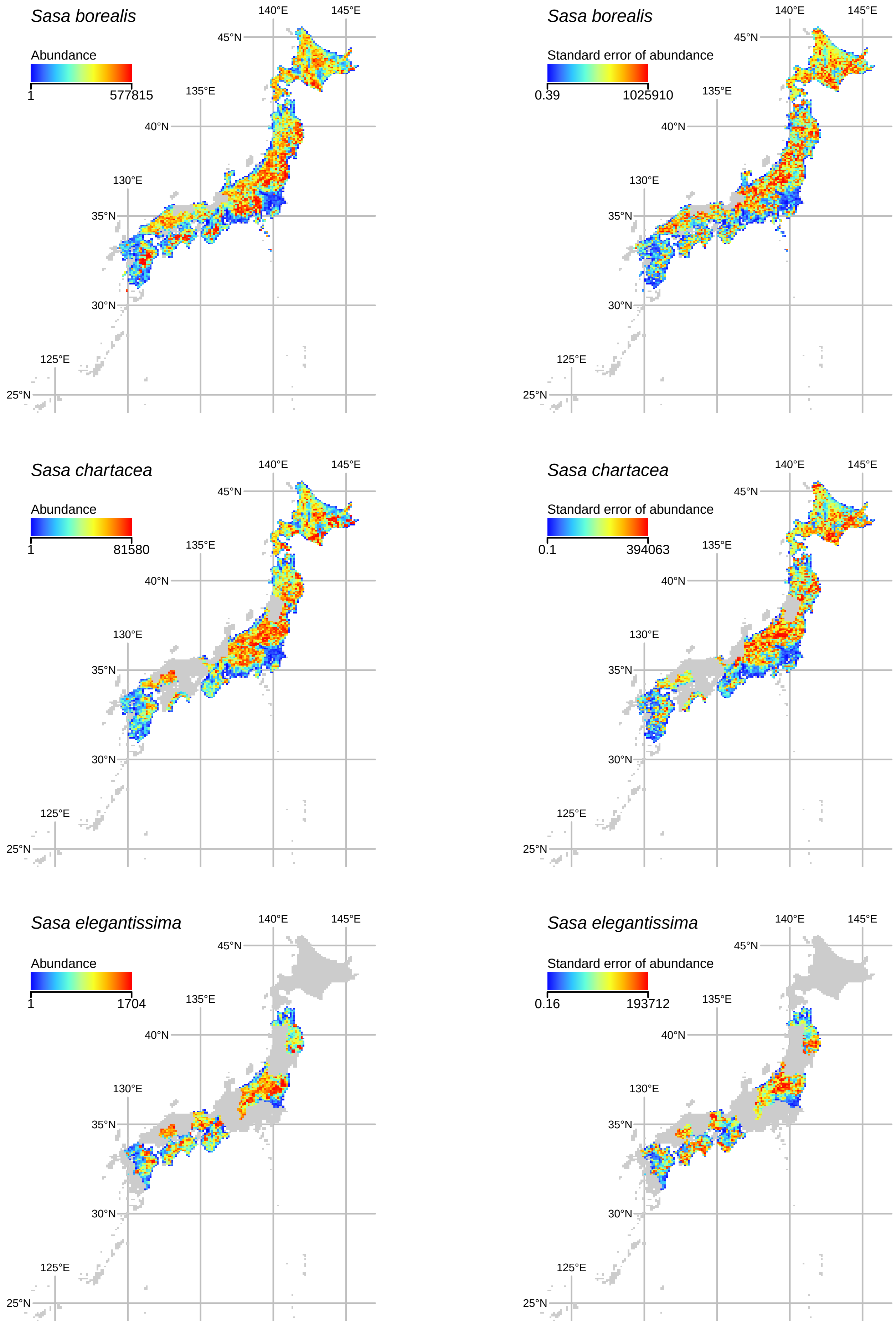
Sasa borealis
Abundance
1
577815
Sasa borealis
Standard error of abundance
0.39
1025910
Sasa chartacea
Abundance
1
81580
Sasa chartacea
Standard error of abundance
0.1
394063
Sasa elegantissima
Abundance
1
1704
Sasa elegantissima
Standard error of abundance
0.16
193712
125°E
130°E
135°E
140°E
145°E
25°N
30°N
35°N
40°N
45°N

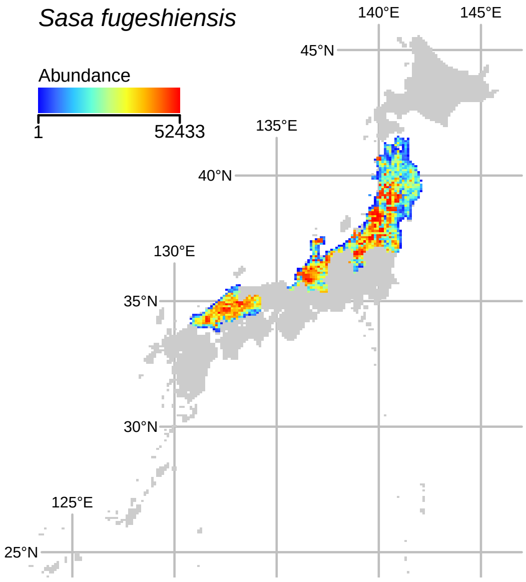
Sasa fugeshiensis
Abundance
1
52433
140°E
145°E
135°E
130°E
125°E
45°N
40°N
35°N
30°N
25°N

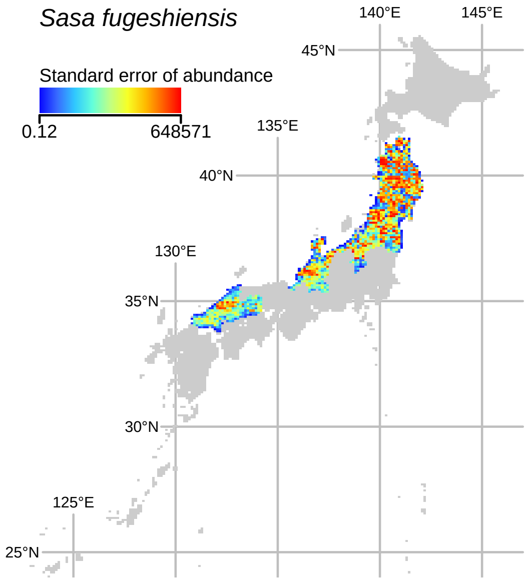
Sasa fugeshiensis
Standard error of abundance
0.12
648571
140°E
145°E
135°E
130°E
125°E
45°N
40°N
35°N
30°N
25°N

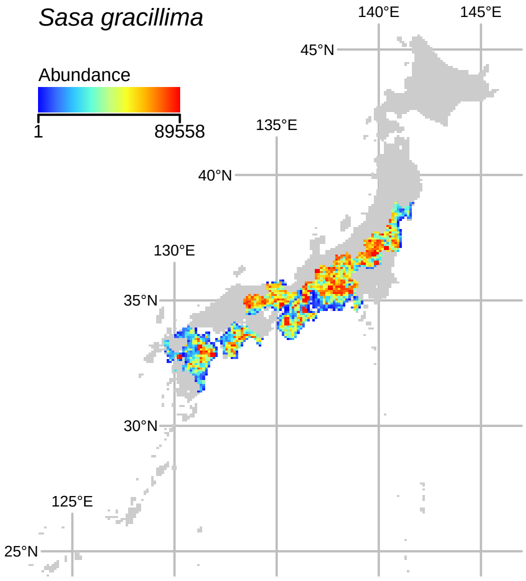
Sasa gracillima
Abundance
1
89558
140°E
145°E
135°E
130°E
125°E
45°N
40°N
35°N
30°N
25°N

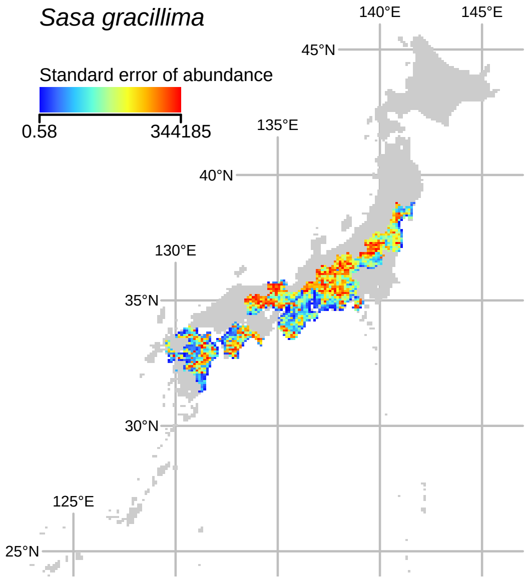
Sasa gracillima
Standard error of abundance
0.58
344185
140°E
145°E
135°E
130°E
125°E
45°N
40°N
35°N
30°N
25°N

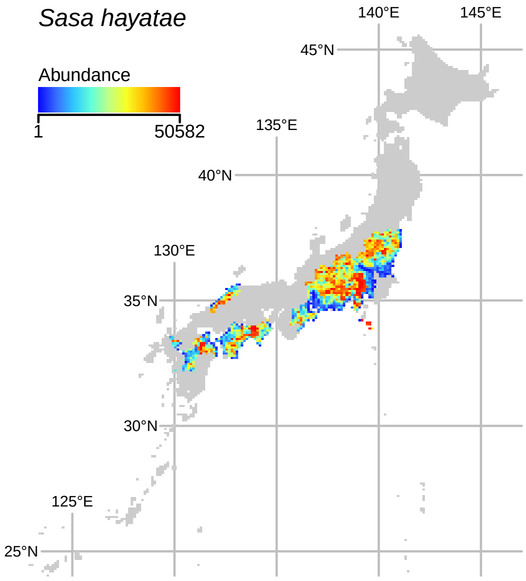
Sasa hayatae
Abundance
1
50582
140°E
145°E
135°E
130°E
125°E
45°N
40°N
35°N
30°N
25°N

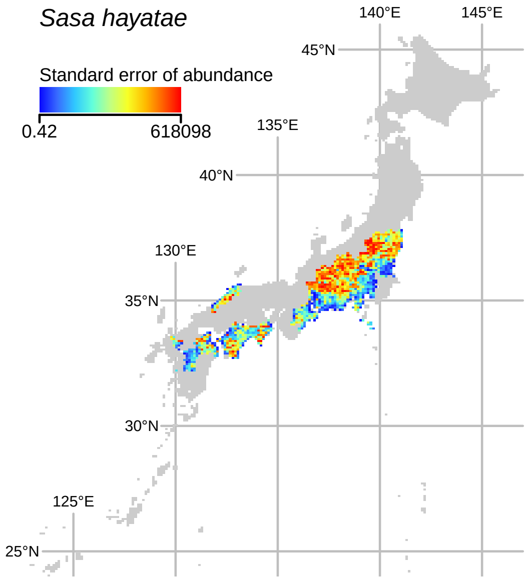
Sasa hayatae
Standard error of abundance
0.42
618098
140°E
145°E
135°E
130°E
125°E
45°N
40°N
35°N
30°N
25°N

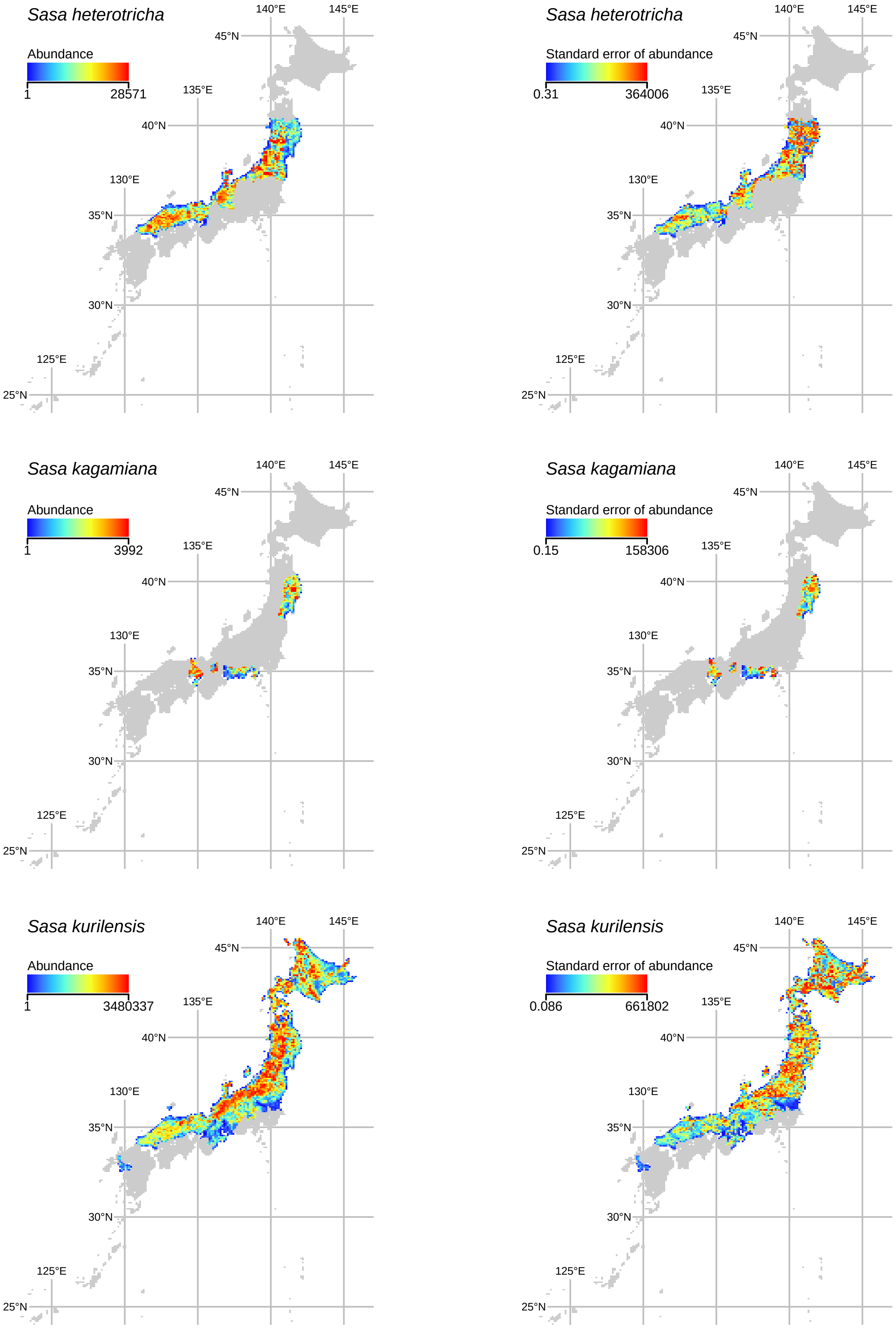
Sasa heterotricha
Abundance
1
28571
Sasa heterotricha
Standard error of abundance
0.31
364006
Sasa kagamiana
Abundance
1
3992
Sasa kagamiana
Standard error of abundance
0.15
158306
Sasa kurilensis
Abundance
1
3480337
Sasa kurilensis
Standard error of abundance
0.086
661802
125°E
130°E
135°E
140°E
145°E
25°N
30°N
35°N
40°N
45°N

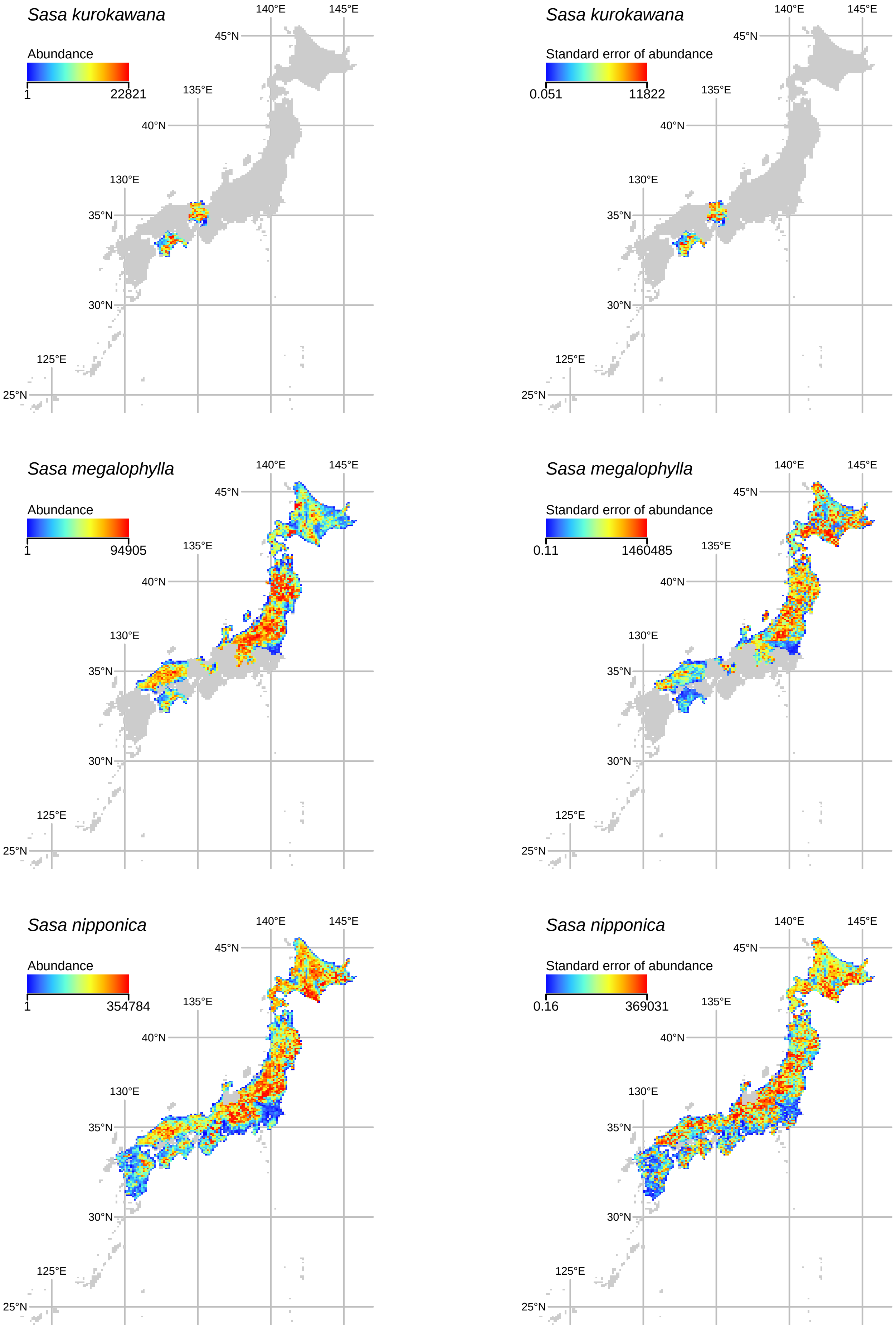

Sasa kurokawana
Abundance
1
22821
Sasa kurokawana
Standard error of abundance
0.051
11822
Sasa megalophylla
Abundance
1
94905
Sasa megalophylla
Standard error of abundance
0.11
1460485
Sasa nipponica
Abundance
1
354784
Sasa nipponica
Standard error of abundance
0.16
369031
125°E
130°E
135°E
140°E
145°E
25°N
30°N
35°N
40°N
45°N

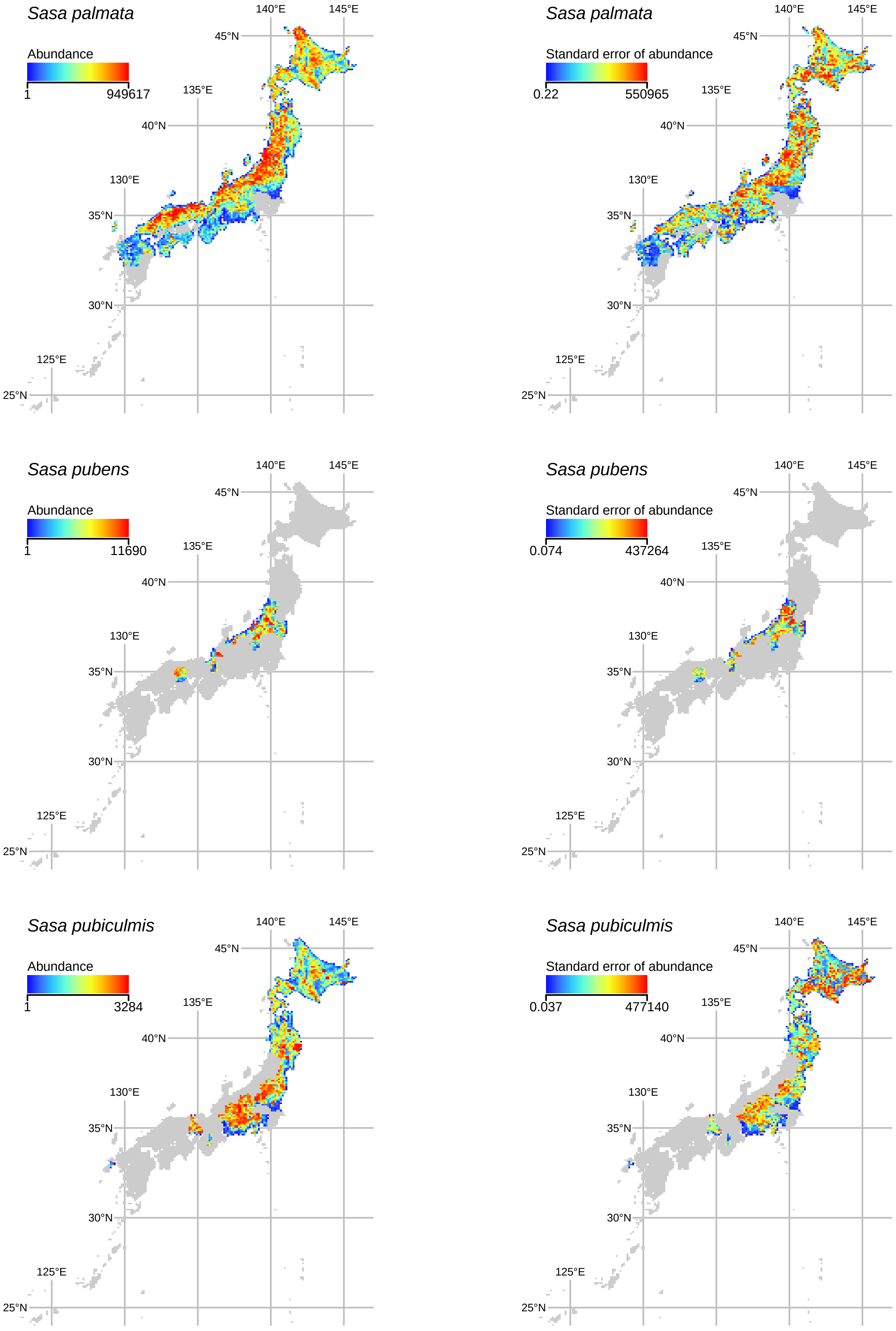
Sasa palmata
Abundance
1
949617
Sasa palmata
Standard error of abundance
0.22
550965
Sasa pubens
Abundance
1
11690
Sasa pubens
Standard error of abundance
0.074
437264
Sasa pubiculmis
Abundance
1
3284
Sasa pubiculmis
Standard error of abundance
0.037
477140
125°E
130°E
135°E
140°E
145°E
25°N
30°N
35°N
40°N
45°N

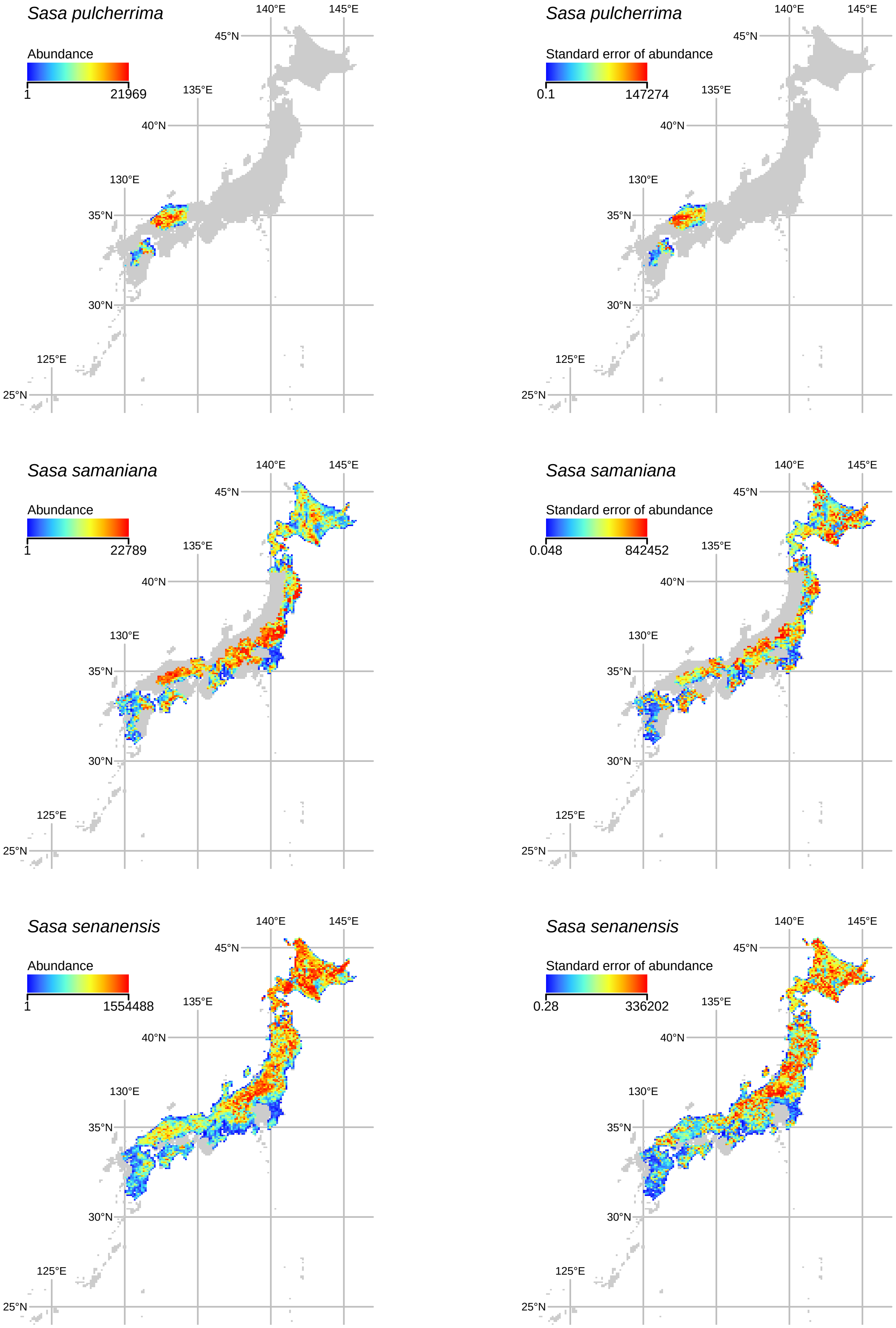
Sasa pulcherrima
Abundance
1
21969
Sasa pulcherrima
Standard error of abundance
0.1
147274
Sasa samaniana
Abundance
1
22789
Sasa samaniana
Standard error of abundance
0.048
842452
Sasa senanensis
Abundance
1
1554488
Sasa senanensis
Standard error of abundance
0.28
336202
125°E
130°E
135°E
140°E
145°E
25°N
30°N
35°N
40°N
45°N

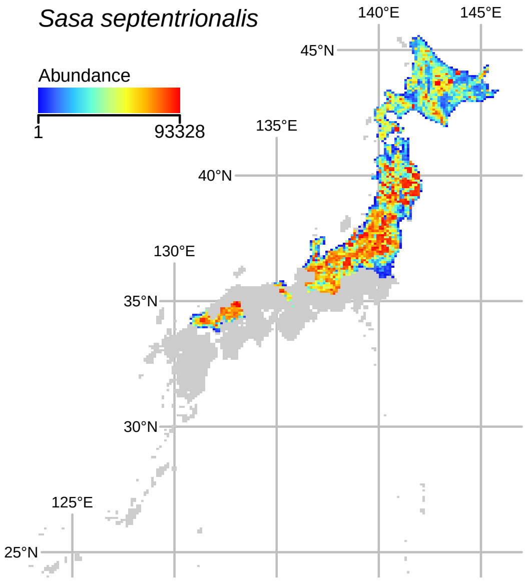
Sasa septentrionalis
Abundance
1
93328
140°E
145°E
45°N
135°E
40°N
130°E
35°N
30°N
125°E
25°N

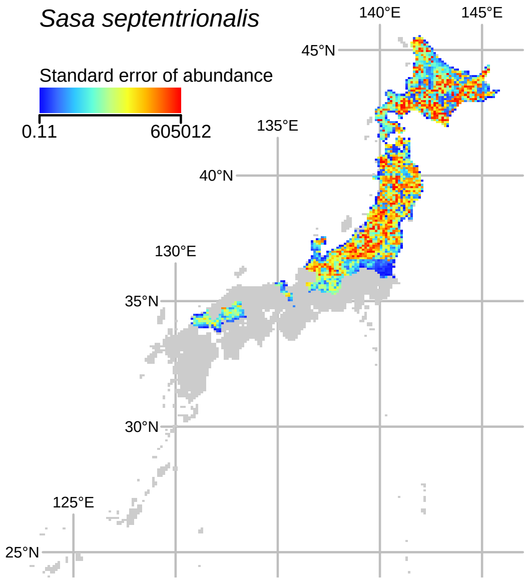
Sasa septentrionalis
Standard error of abundance
0.11
605012
140°E
145°E
45°N
135°E
40°N
130°E
35°N
30°N
125°E
25°N

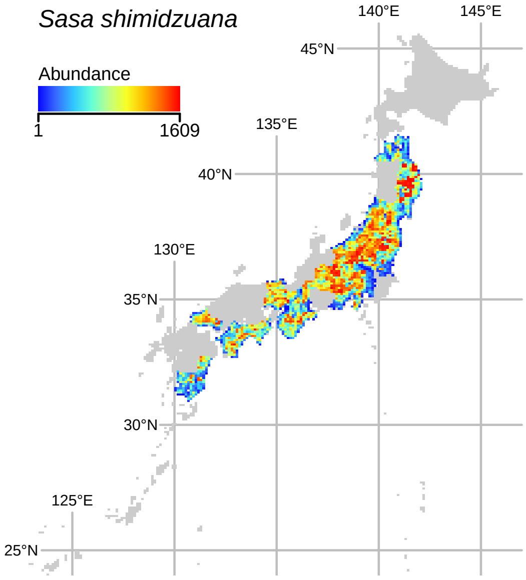
Sasa shimidzuana
Abundance
1
1609
140°E
145°E
45°N
135°E
40°N
130°E
35°N
30°N
125°E
25°N

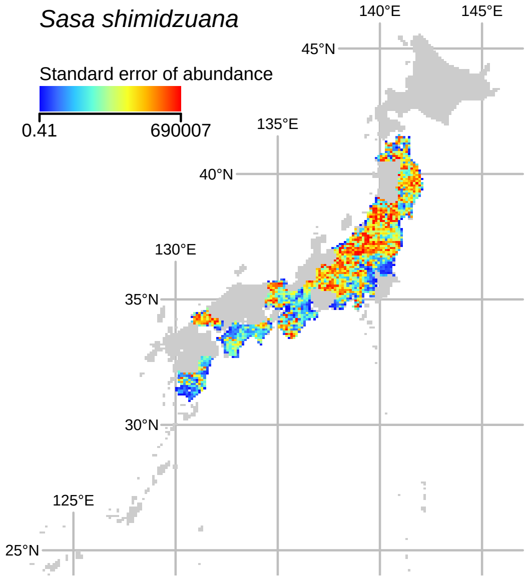
Sasa shimidzuana
Standard error of abundance
0.41
690007
140°E
145°E
45°N
135°E
40°N
130°E
35°N
30°N
125°E
25°N

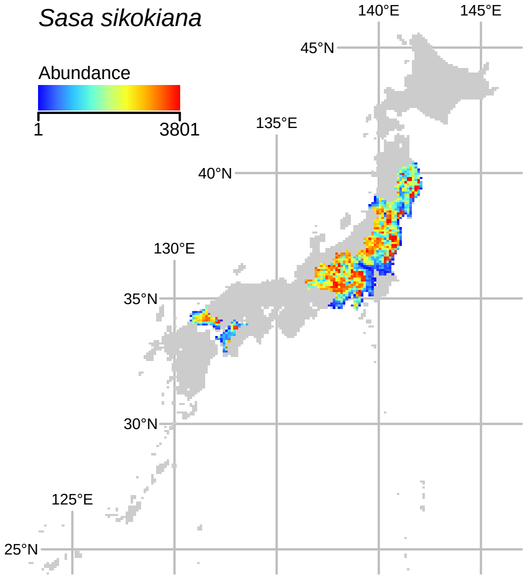
Sasa sikokiana
Abundance
1
3801
140°E
145°E
45°N
135°E
40°N
130°E
35°N
30°N
125°E
25°N

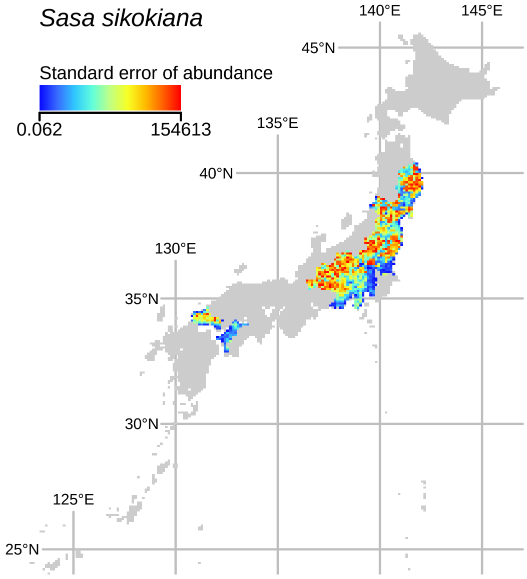
Sasa sikokiana
Standard error of abundance
0.062
154613
140°E
145°E
45°N
135°E
40°N
130°E
35°N
30°N
125°E
25°N

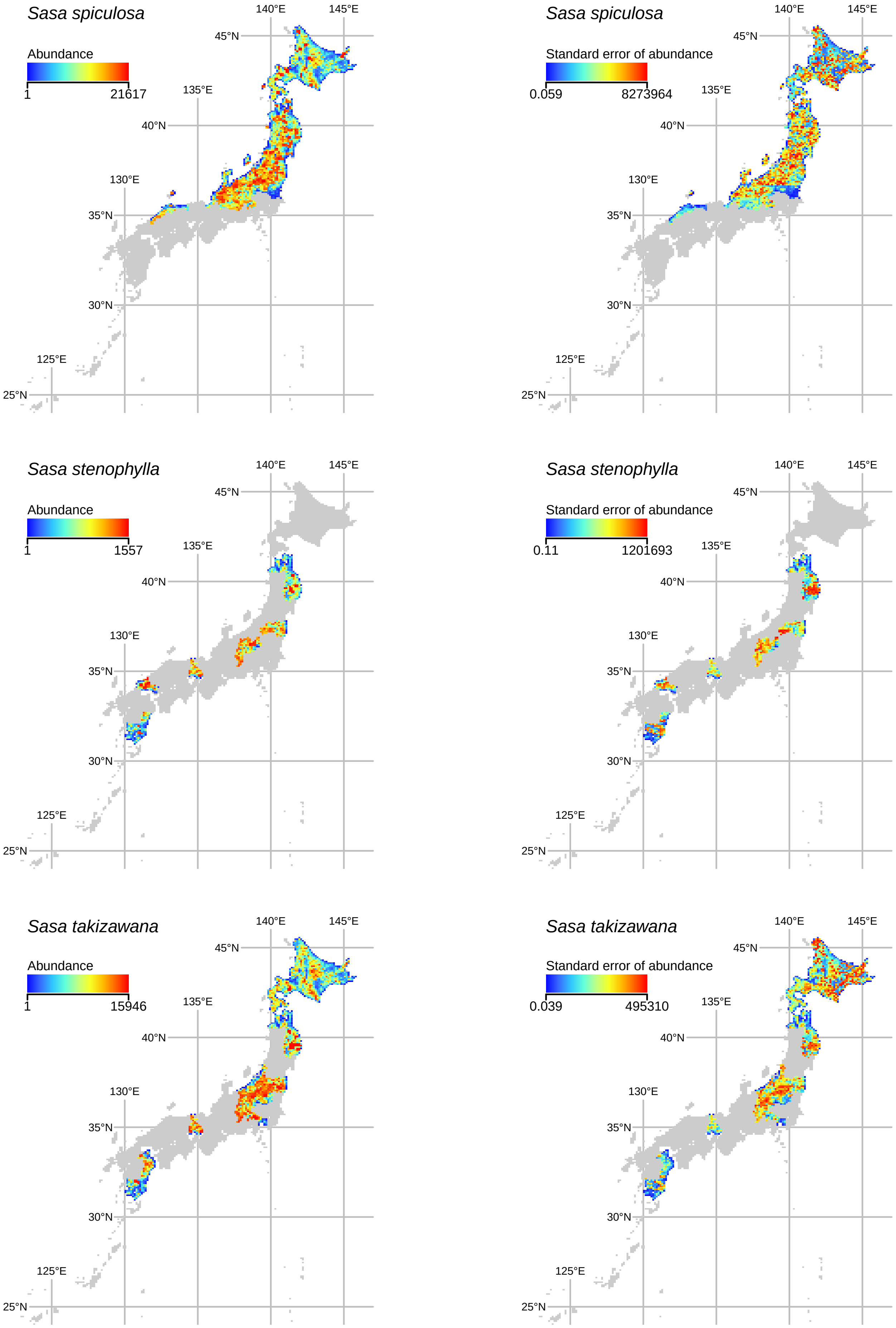
Sasa spiculosa
Abundance
1
21617
Sasa spiculosa
Standard error of abundance
0.059
8273964
Sasa stenophylla
Abundance
1
1557
Sasa stenophylla
Standard error of abundance
0.11
1201693
Sasa takizawana
Abundance
1
15946
Sasa takizawana
Standard error of abundance
0.039
495310
140°E
145°E
135°E
130°E
125°E
45°N
40°N
35°N
30°N
25°N

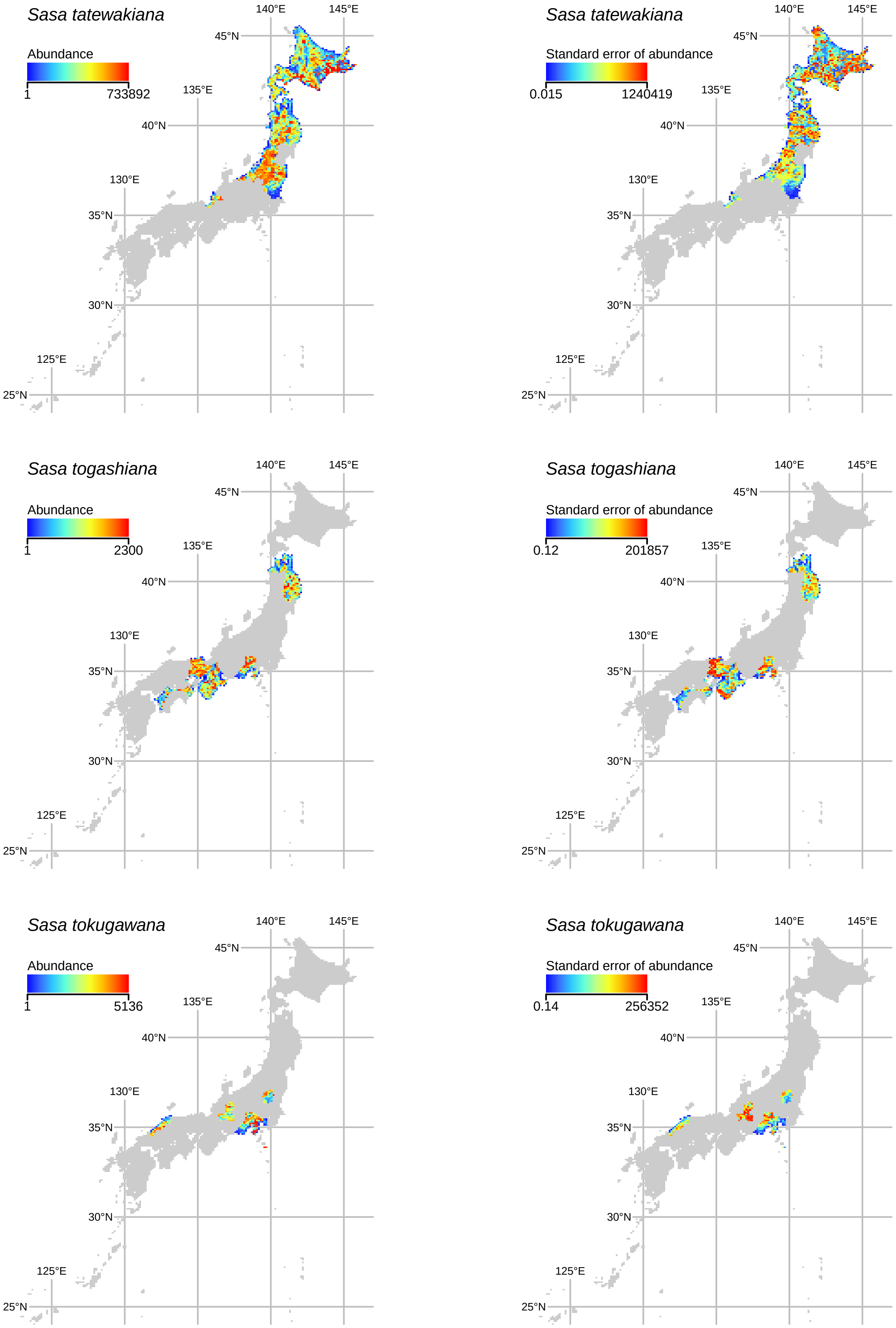
Sasa tatewakiana
Abundance
1
733892
Sasa tatewakiana
Standard error of abundance
0.015
1240419
Sasa togashiana
Abundance
1
2300
Sasa togashiana
Standard error of abundance
0.12
201857
Sasa tokugawana
Abundance
1
5136
Sasa tokugawana
Standard error of abundance
0.14
256352
125°E
130°E
135°E
140°E
145°E
25°N
30°N
35°N
40°N
45°N

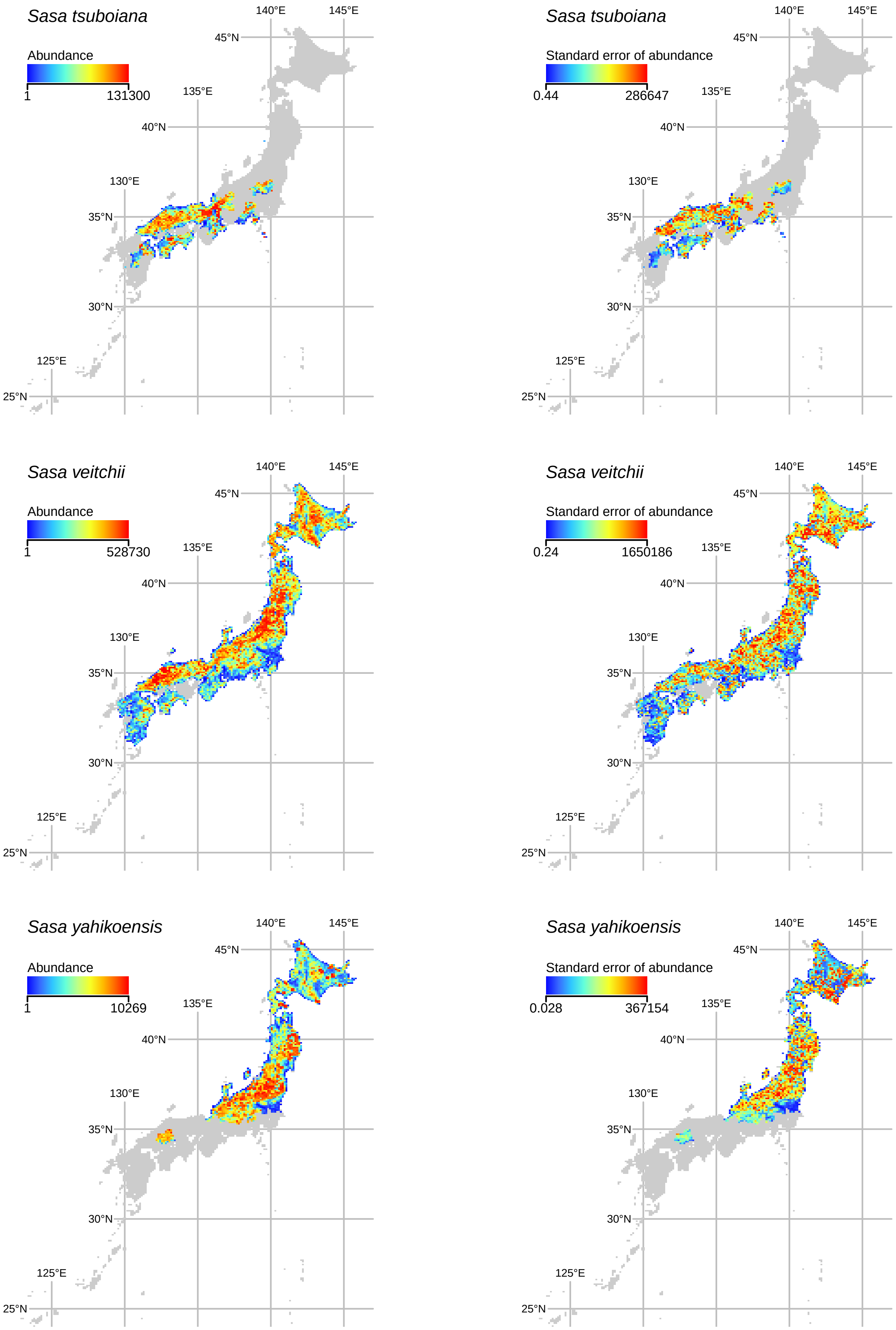
Sasa tsuboiana
Abundance
1
131300
Sasa tsuboiana
Standard error of abundance
0.44
286647
Sasa veitchii
Abundance
1
528730
Sasa veitchii
Standard error of abundance
0.24
1650186
Sasa yahikoensis
Abundance
1
10269
Sasa yahikoensis
Standard error of abundance
0.028
367154
140°E
145°E
45°N
135°E
40°N
130°E
35°N
30°N
125°E
25°N

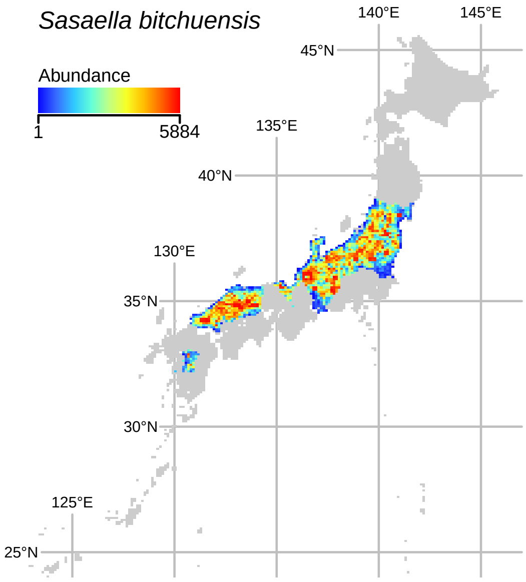
Sasaella bitchuensis
Abundance
1
5884
140°E
145°E
135°E
130°E
125°E
45°N
40°N
35°N
30°N
25°N

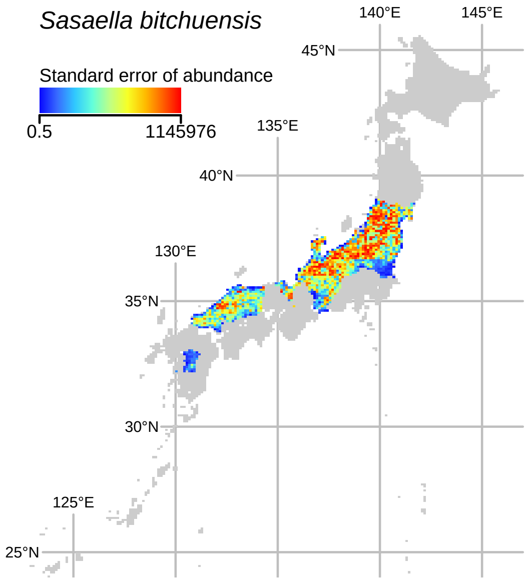
Sasaella bitchuensis
Standard error of abundance
0.5
1145976
140°E
145°E
135°E
130°E
125°E
45°N
40°N
35°N
30°N
25°N

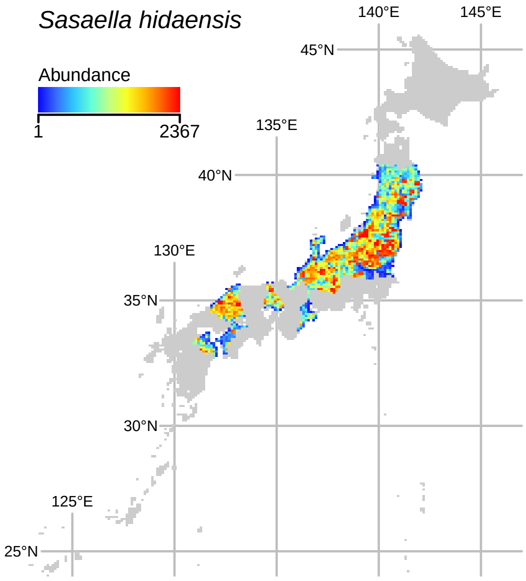
Sasaella hidaensis
Abundance
1
2367
140°E
145°E
135°E
130°E
125°E
45°N
40°N
35°N
30°N
25°N

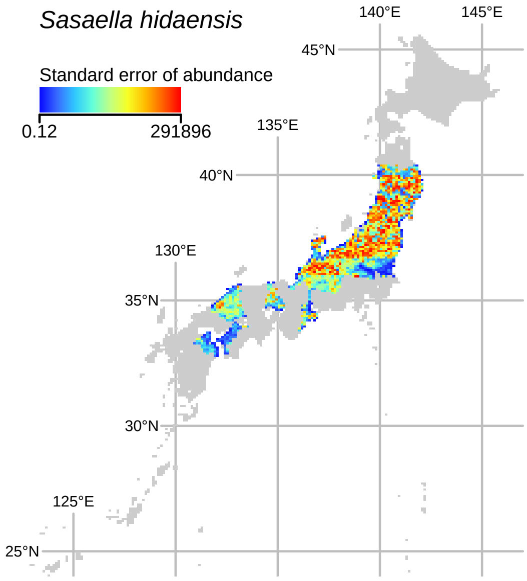
Sasaella hidaensis
Standard error of abundance
0.12
291896
140°E
145°E
135°E
130°E
125°E
45°N
40°N
35°N
30°N
25°N

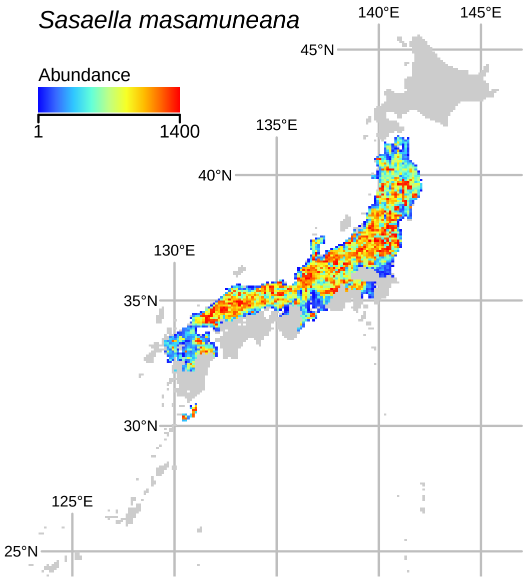
Sasaella masamuneana
Abundance
1
1400
140°E
145°E
135°E
130°E
125°E
45°N
40°N
35°N
30°N
25°N

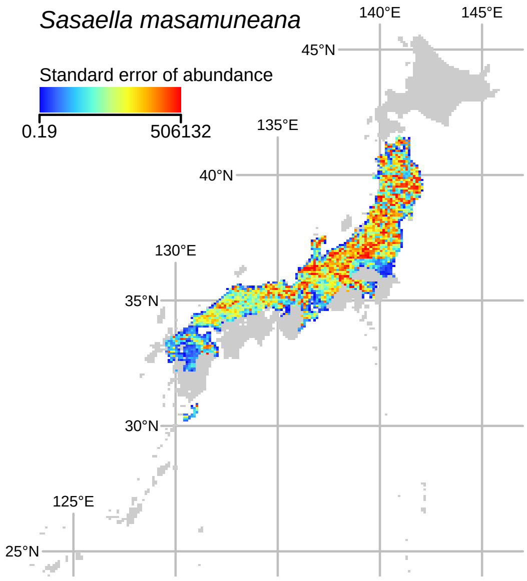
Sasaella masamuneana
Standard error of abundance
0.19
506132
140°E
145°E
135°E
130°E
125°E
45°N
40°N
35°N
30°N
25°N

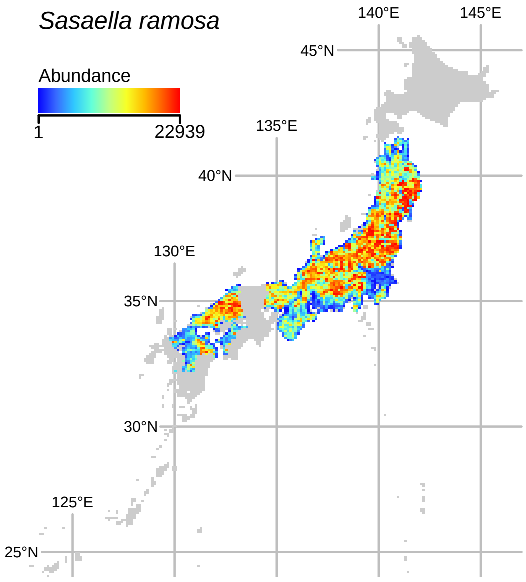
Sasaella ramosa
Abundance
1
22939
140°E
145°E
135°E
130°E
125°E
45°N
40°N
35°N
30°N
25°N

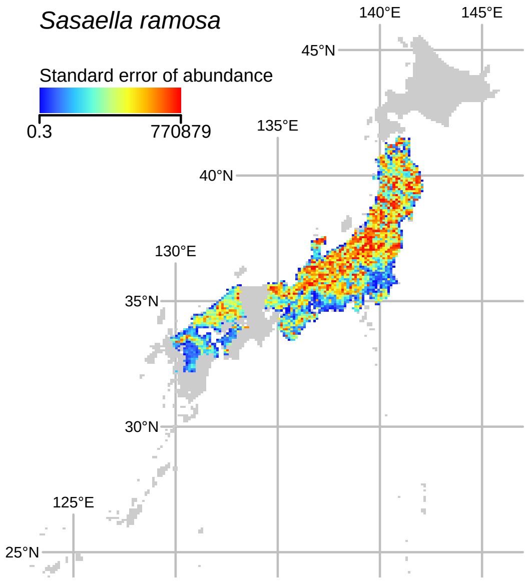
Sasaella ramosa
Standard error of abundance
0.3
770879
140°E
145°E
135°E
130°E
125°E
45°N
40°N
35°N
30°N
25°N

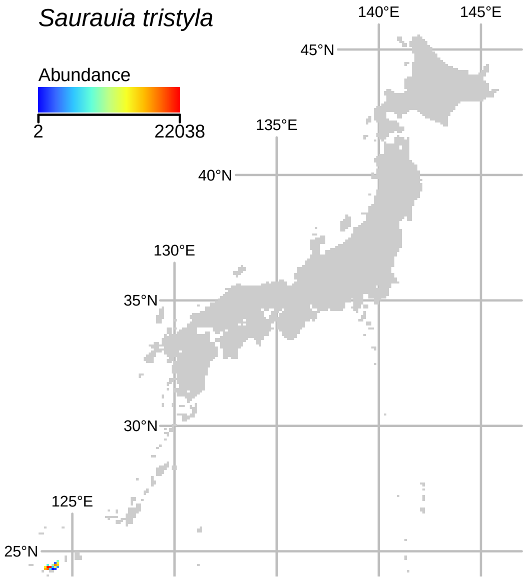
Saurauia tristyla
Abundance
2
22038
140°E
145°E
135°E
130°E
125°E
45°N
40°N
35°N
30°N
25°N

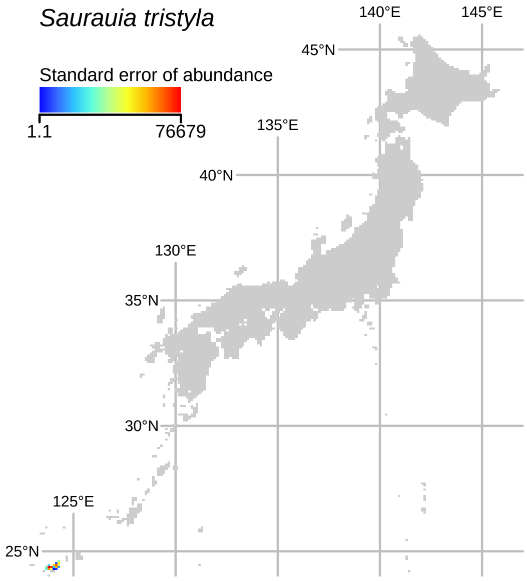
Saurauia tristyla
Standard error of abundance
1.1
76679
140°E
145°E
135°E
130°E
125°E
45°N
40°N
35°N
30°N
25°N

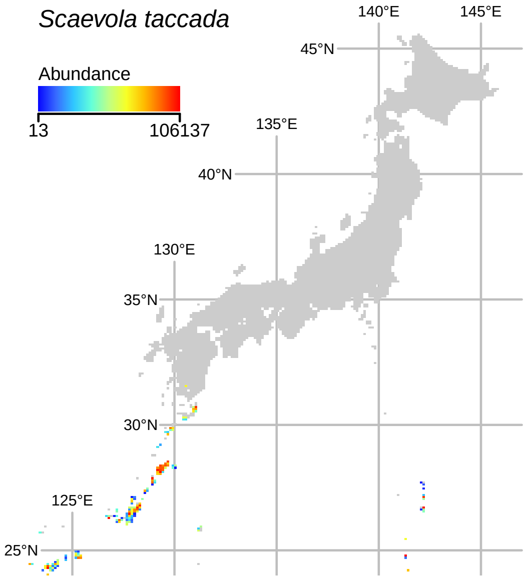
Scaevola taccada
Abundance
13
106137
140°E
145°E
135°E
130°E
125°E
45°N
40°N
35°N
30°N
25°N

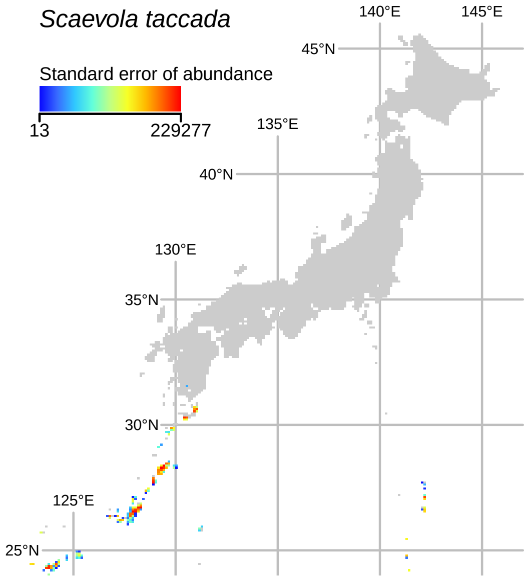
Scaevola taccada
Standard error of abundance
13
229277
140°E
145°E
135°E
130°E
125°E
45°N
40°N
35°N
30°N
25°N

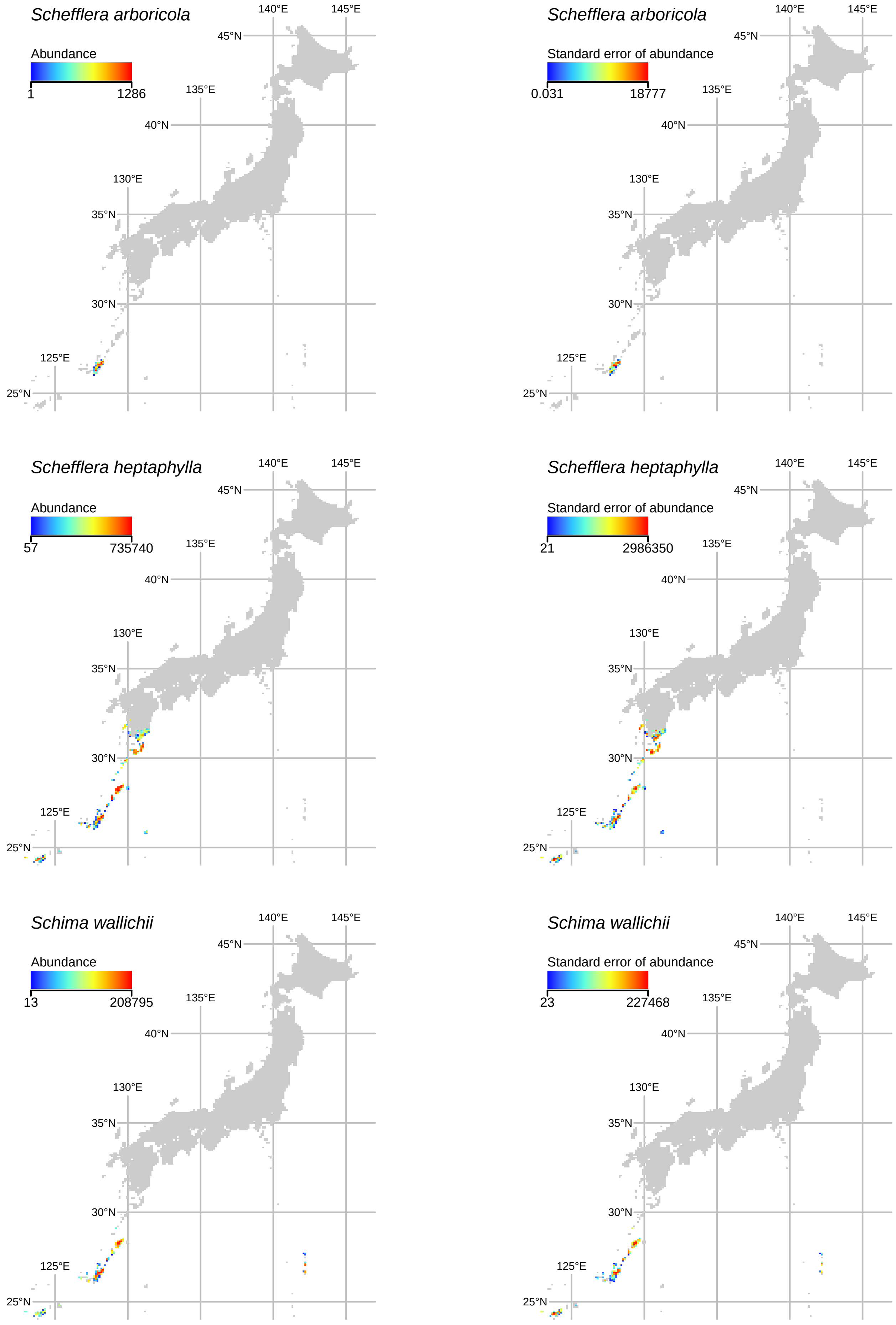
Schefflera arboricola
Abundance
1
1286
Schefflera arboricola
Standard error of abundance
0.031
18777
Schefflera heptaphylla
Abundance
57
735740
Schefflera heptaphylla
Standard error of abundance
21
2986350
Schima wallichii
Abundance
13
208795
Schima wallichii
Standard error of abundance
23
227468
140°E
145°E
45°N
135°E
40°N
130°E
35°N
30°N
125°E
25°N

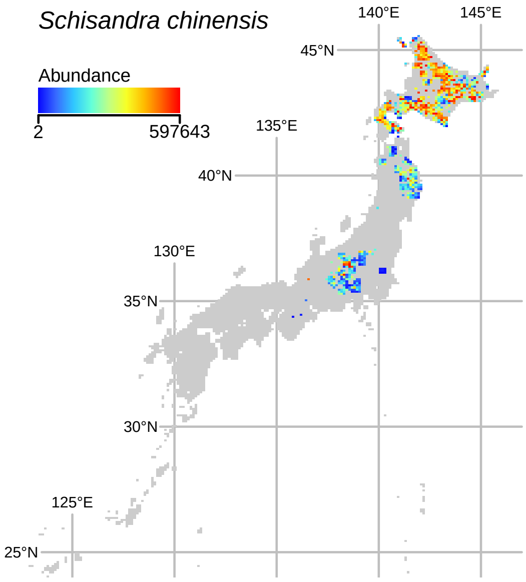
Schisandra chinensis
Abundance
2
597643
140°E
145°E
45°N
135°E
40°N
130°E
35°N
30°N
125°E
25°N

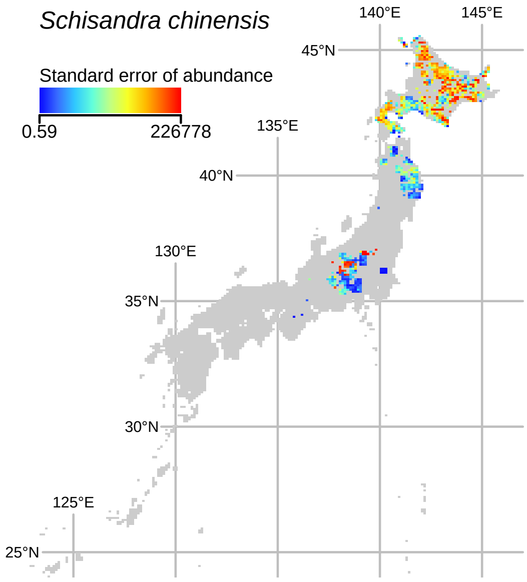
Schisandra chinensis
Standard error of abundance
0.59
226778
140°E
145°E
45°N
135°E
40°N
130°E
35°N
30°N
125°E
25°N

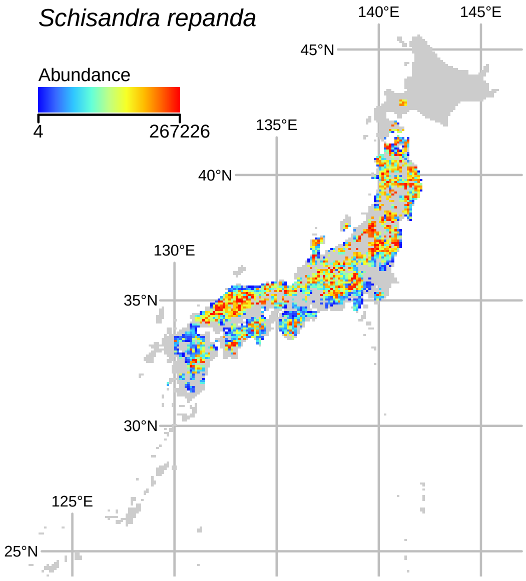
Schisandra repanda
Abundance
4
267226
140°E
145°E
45°N
135°E
40°N
130°E
35°N
30°N
125°E
25°N

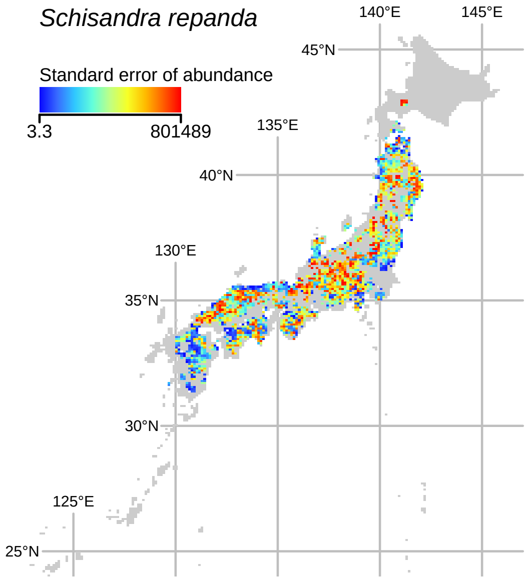
Schisandra repanda
Standard error of abundance
3.3
801489
140°E
145°E
45°N
135°E
40°N
130°E
35°N
30°N
125°E
25°N

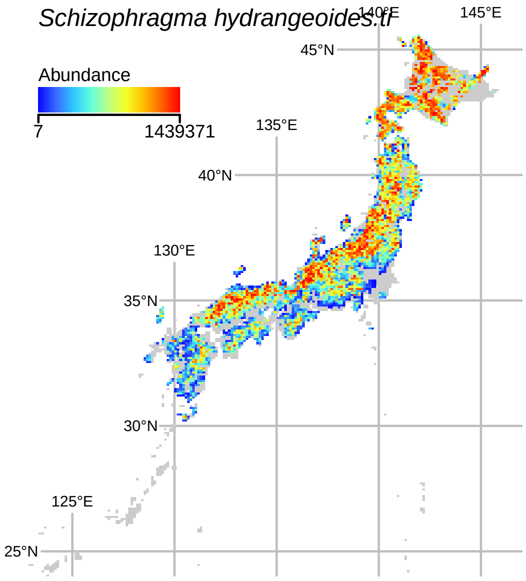
Schizophragma hydrangeoides.tF
Abundance
7
1439371
140°E
145°E
45°N
135°E
40°N
130°E
35°N
30°N
125°E
25°N

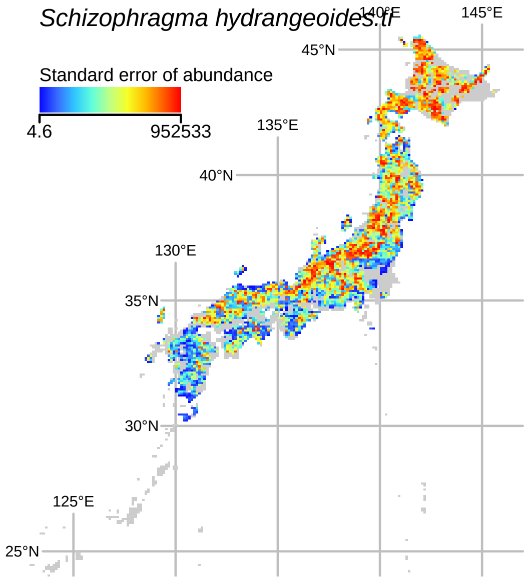
Schizophragma hydrangeoides.tF
Standard error of abundance
4.6
952533
140°E
145°E
45°N
135°E
40°N
130°E
35°N
30°N
125°E
25°N

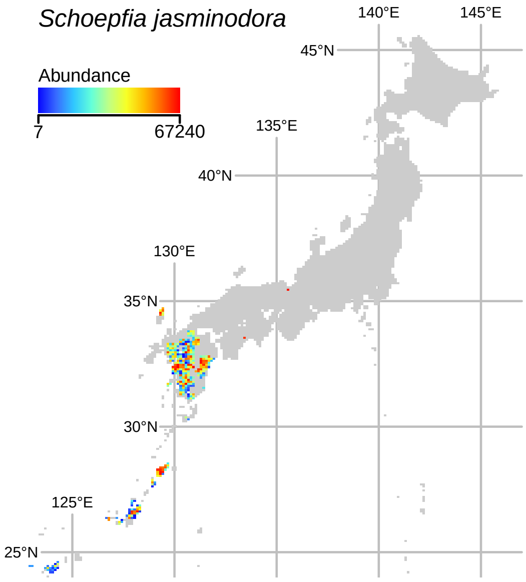
Schoepfia jasminodora
Abundance
7
67240
140°E
145°E
45°N
135°E
40°N
130°E
35°N
30°N
125°E
25°N

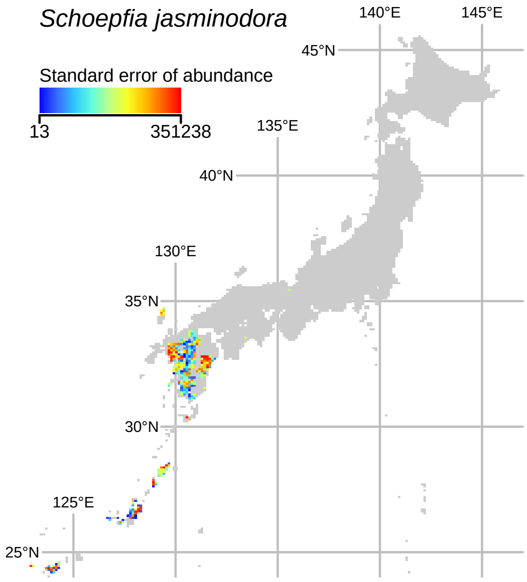
Schoepfia jasminodora
Standard error of abundance
13
351238
140°E
145°E
45°N
135°E
40°N
130°E
35°N
30°N
125°E
25°N

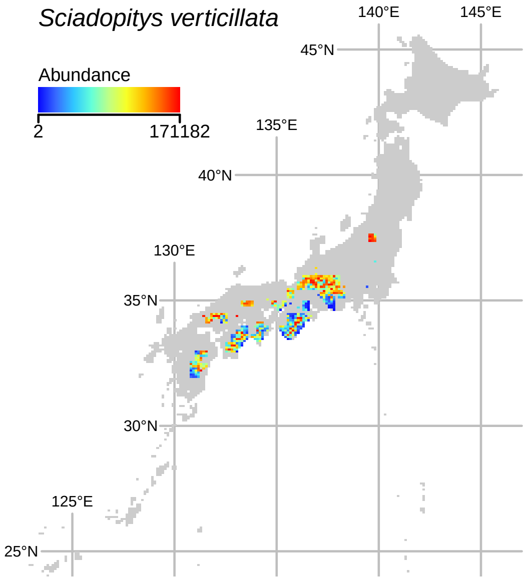
Sciadopitys verticillata
Abundance
2
171182
140°E
145°E
45°N
135°E
40°N
130°E
35°N
30°N
125°E
25°N

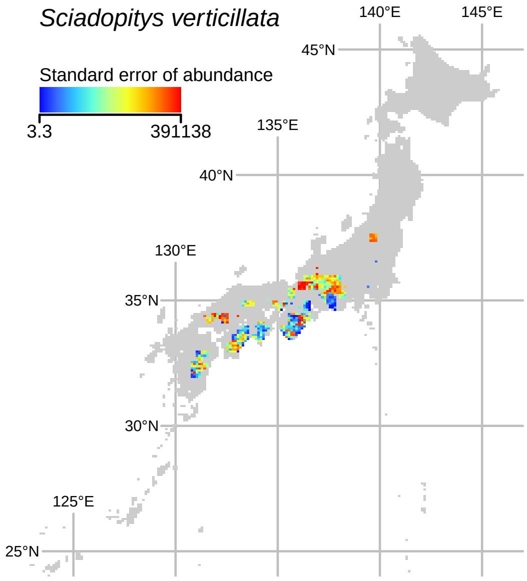
Sciadopitys verticillata
Standard error of abundance
3.3
391138
140°E
145°E
45°N
135°E
40°N
130°E
35°N
30°N
125°E
25°N

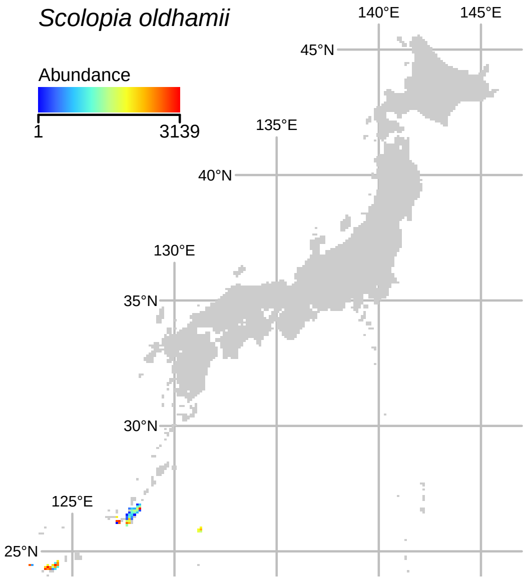
Scolopia oldhamii
Abundance
1
3139
140°E
145°E
45°N
135°E
40°N
130°E
35°N
30°N
125°E
25°N

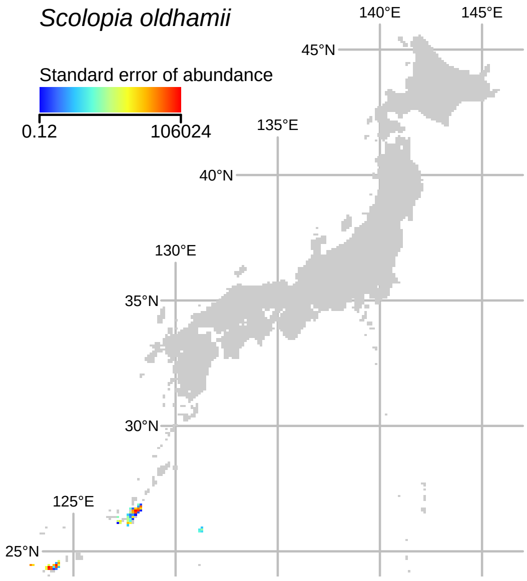
Scolopia oldhamii
Standard error of abundance
0.12
106024
140°E
145°E
45°N
135°E
40°N
130°E
35°N
30°N
125°E
25°N

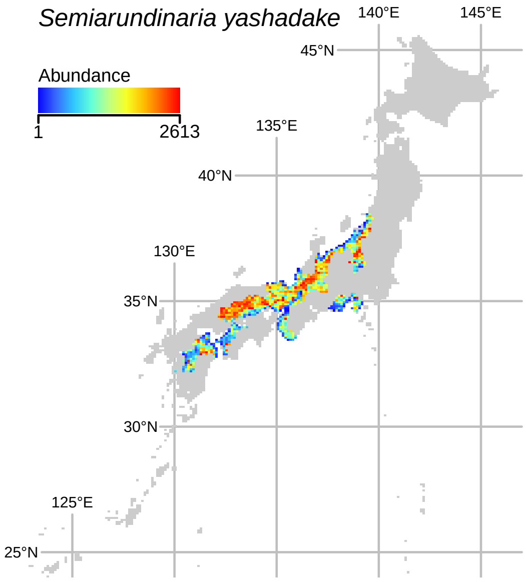
Semiarundinaria yashadake
Abundance
1
2613
140°E
145°E
45°N
135°E
40°N
130°E
35°N
30°N
125°E
25°N

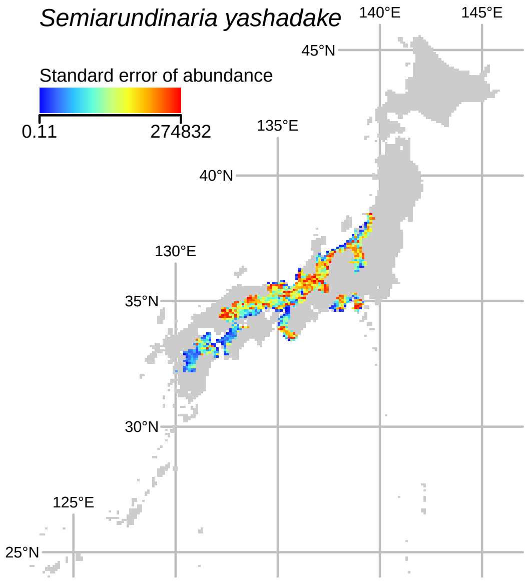
Semiarundinaria yashadake
Standard error of abundance
0.11
274832
140°E
145°E
45°N
135°E
40°N
130°E
35°N
30°N
125°E
25°N

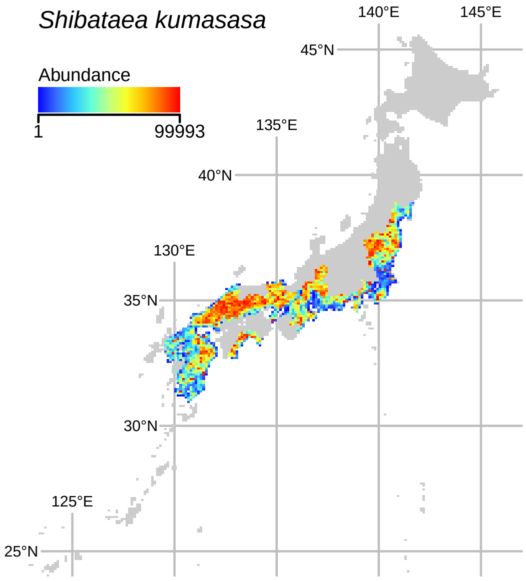
Shibataea kumasasa
Abundance
1
99993
140°E
145°E
45°N
135°E
40°N
130°E
35°N
30°N
125°E
25°N

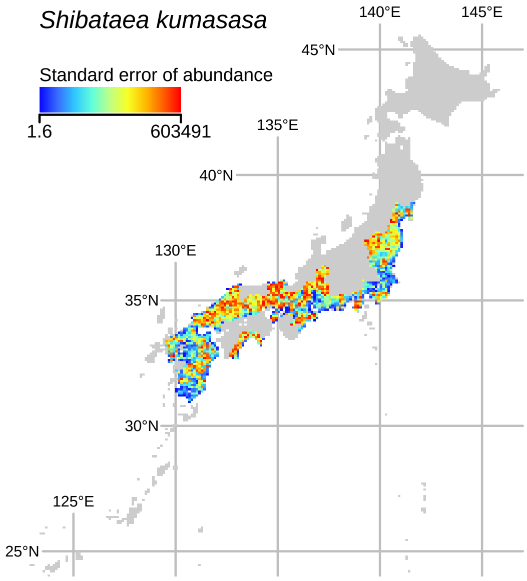
Shibataea kumasasa
Standard error of abundance
1.6
603491
140°E
145°E
45°N
135°E
40°N
130°E
35°N
30°N
125°E
25°N

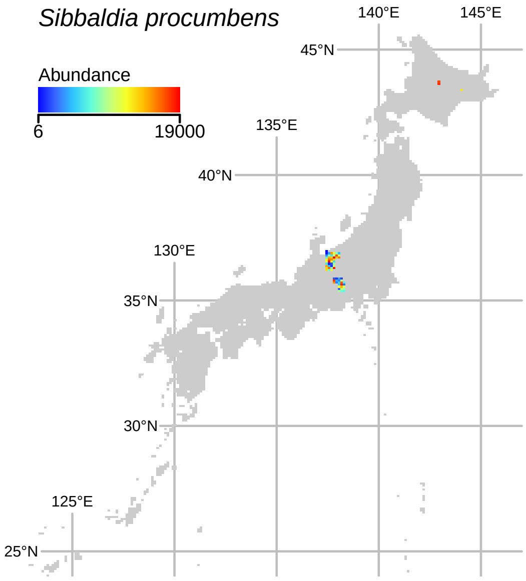
Sibbaldia procumbens
Abundance
6
19000
140°E
145°E
45°N
135°E
40°N
130°E
35°N
30°N
125°E
25°N

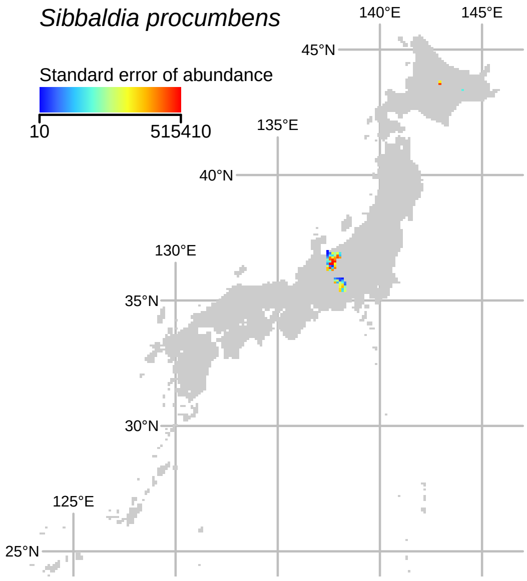
Sibbaldia procumbens
Standard error of abundance
10
515410
140°E
145°E
45°N
135°E
40°N
130°E
35°N
30°N
125°E
25°N

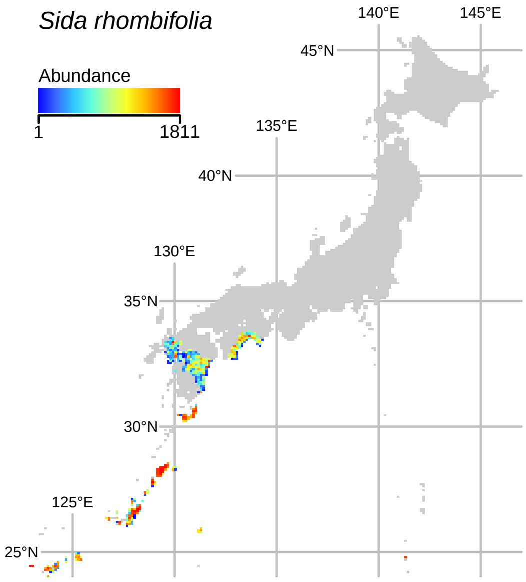
Sida rhombifolia
Abundance
1
1811
140°E
145°E
45°N
135°E
40°N
130°E
35°N
30°N
125°E
25°N

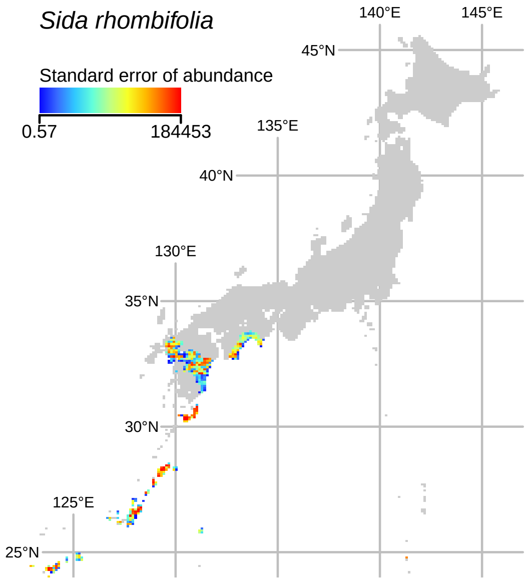
Sida rhombifolia
Standard error of abundance
0.57
184453
140°E
145°E
45°N
135°E
40°N
130°E
35°N
30°N
125°E
25°N

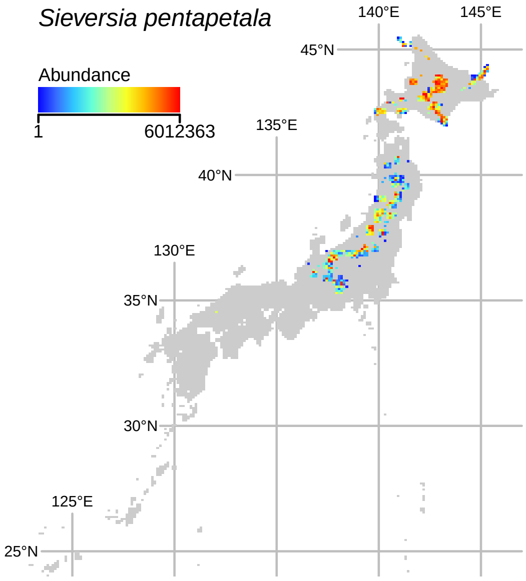
Sieversia pentapetala
Abundance
1
6012363
140°E
145°E
45°N
135°E
40°N
130°E
35°N
30°N
125°E
25°N

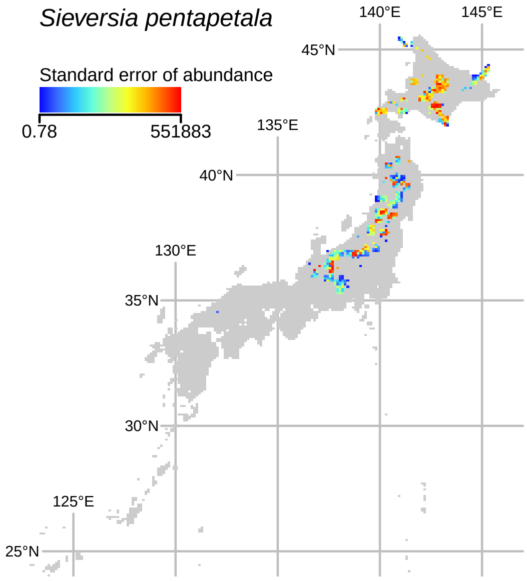
Sieversia pentapetala
Standard error of abundance
0.78
551883
140°E
145°E
45°N
135°E
40°N
130°E
35°N
30°N
125°E
25°N

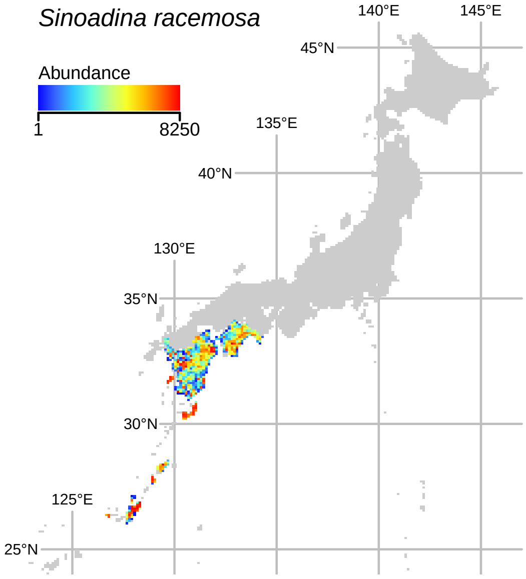
Sinoadina racemosa
Abundance
1
8250
140°E
145°E
45°N
135°E
40°N
130°E
35°N
30°N
125°E
25°N

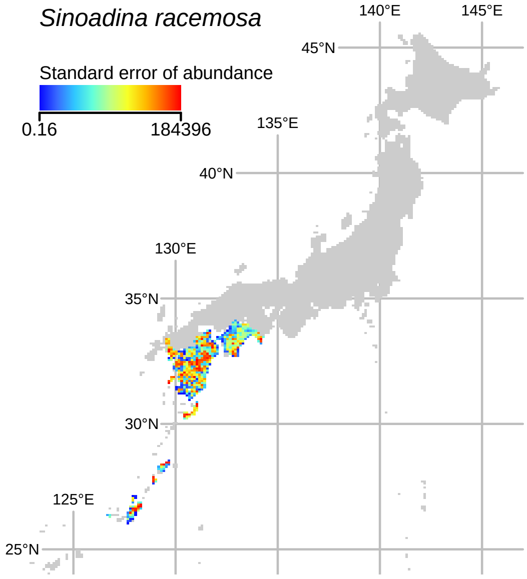
Sinoadina racemosa
Standard error of abundance
0.16
184396
140°E
145°E
45°N
135°E
40°N
130°E
35°N
30°N
125°E
25°N

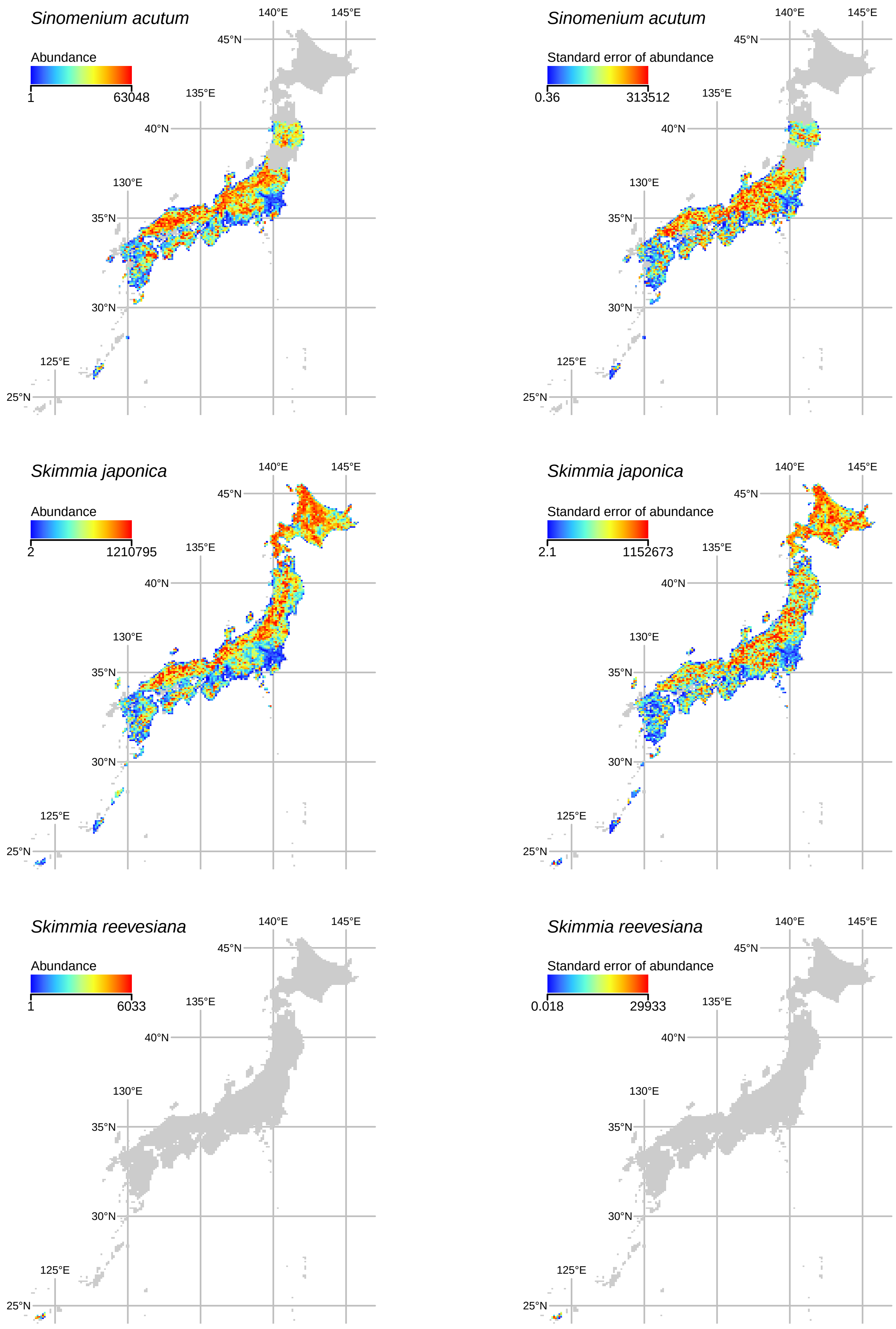
Sinomenium acutum
Abundance
1
63048
Sinomenium acutum
Standard error of abundance
0.36
313512
Skimmia japonica
Abundance
2
1210795
Skimmia japonica
Standard error of abundance
2.1
1152673
Skimmia reevesiana
Abundance
1
6033
Skimmia reevesiana
Standard error of abundance
0.018
29933
125°E
130°E
135°E
140°E
145°E
25°N
30°N
35°N
40°N
45°N

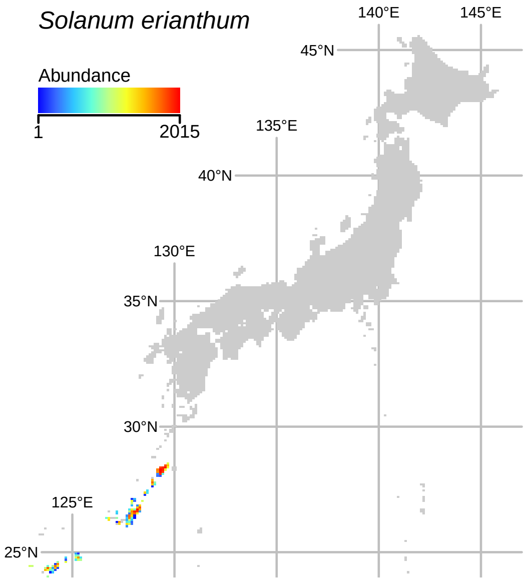
Solanum erianthum
Abundance
1
2015
140°E
145°E
45°N
135°E
40°N
130°E
35°N
30°N
125°E
25°N

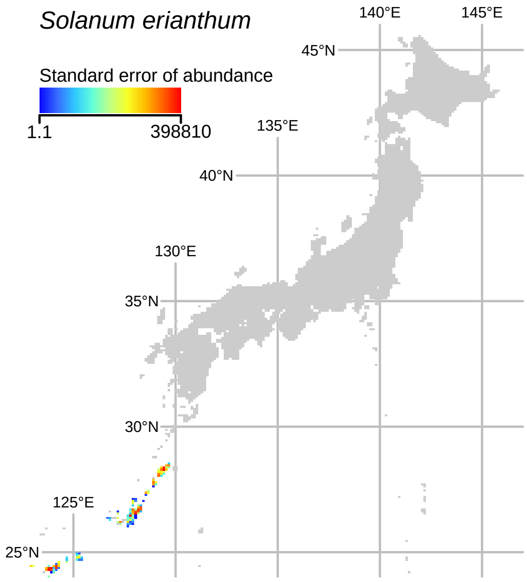
Solanum erianthum
Standard error of abundance
1.1
398810
140°E
145°E
45°N
135°E
40°N
130°E
35°N
30°N
125°E
25°N

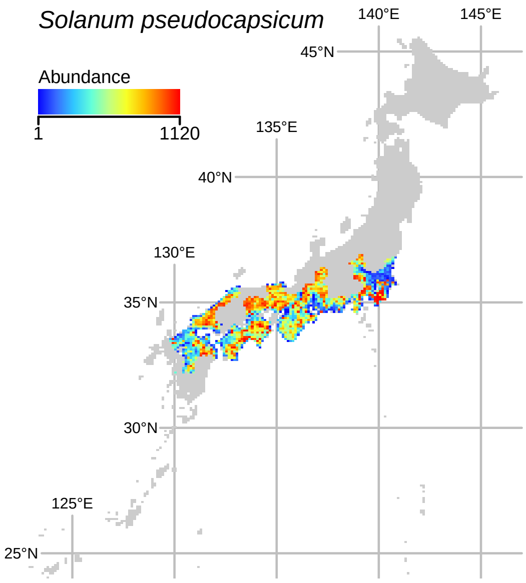
Solanum pseudocapsicum
Abundance
1
1120
140°E
145°E
45°N
135°E
40°N
130°E
35°N
30°N
125°E
25°N

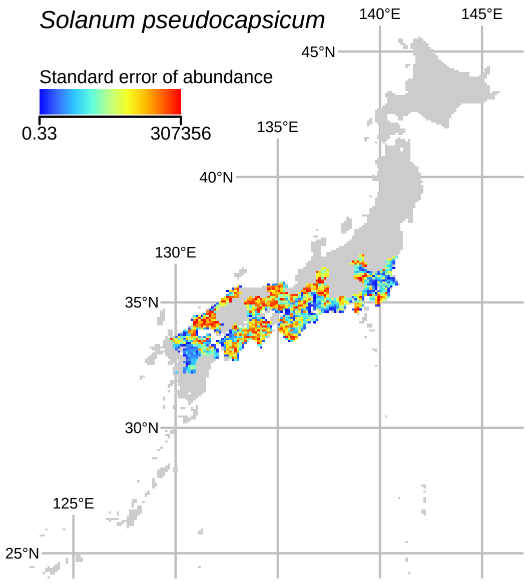
Solanum pseudocapsicum
Standard error of abundance
0.33
307356
140°E
145°E
45°N
135°E
40°N
130°E
35°N
30°N
125°E
25°N

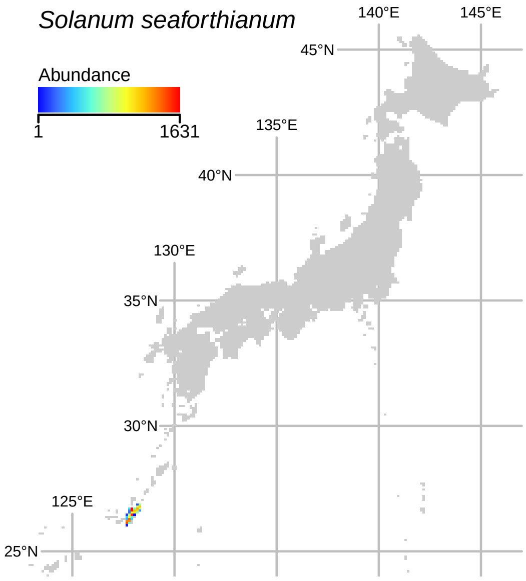
Solanum seaforthianum
Abundance
1
1631
140°E
145°E
45°N
135°E
40°N
130°E
35°N
30°N
125°E
25°N

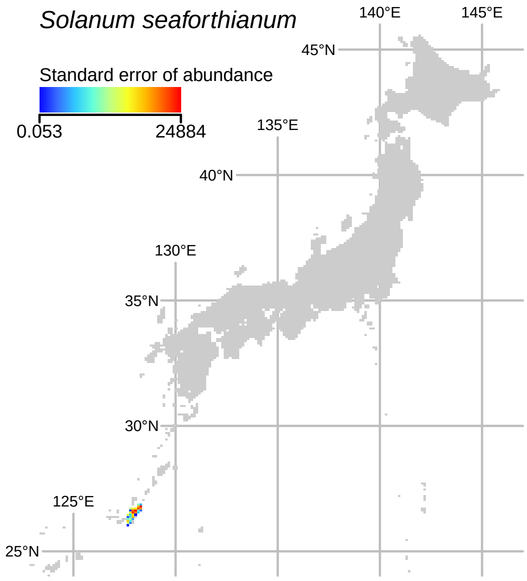
Solanum seaforthianum
Standard error of abundance
0.053
24884
140°E
145°E
45°N
135°E
40°N
130°E
35°N
30°N
125°E
25°N

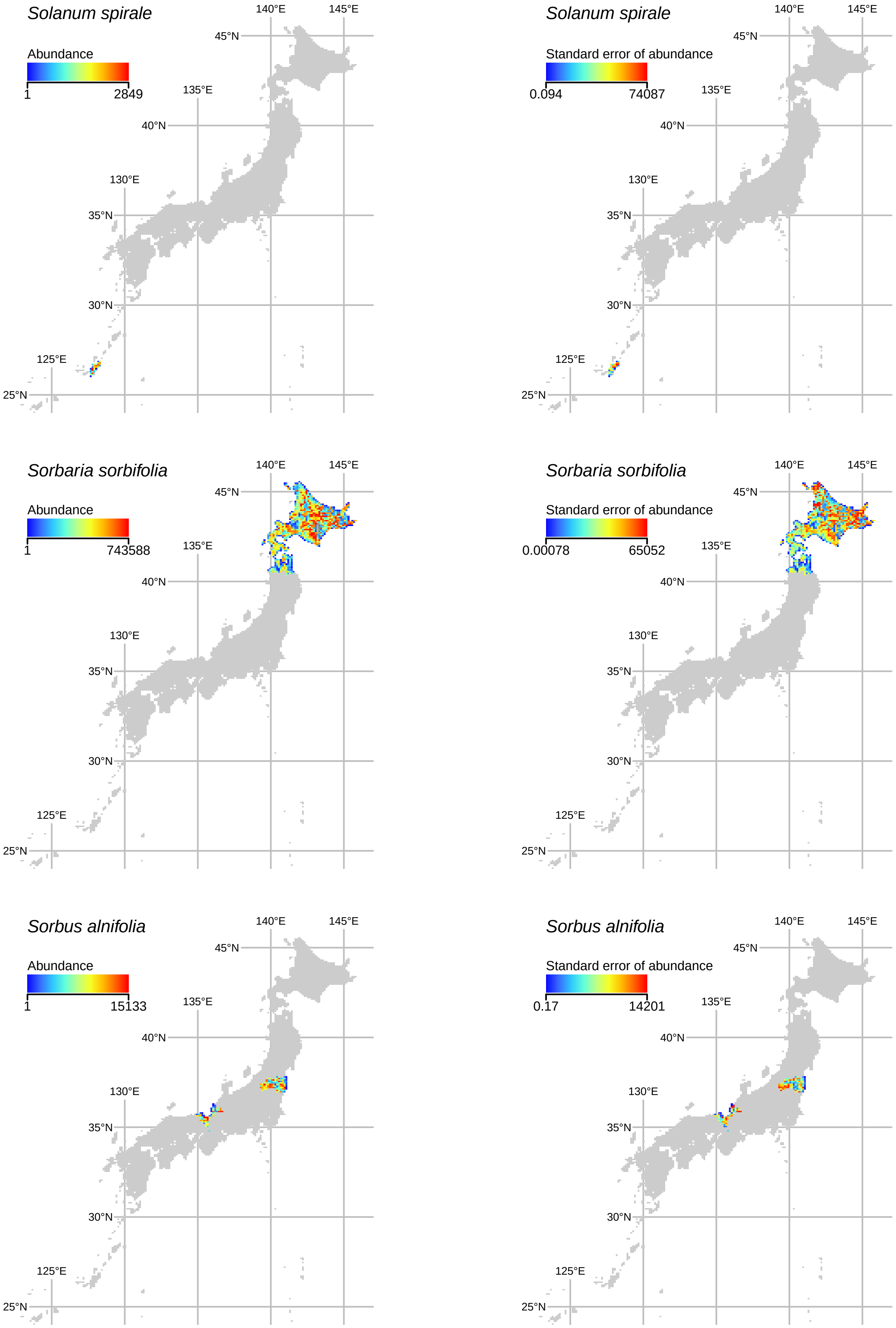
Solanum spirale
Abundance
1
2849
Solanum spirale
Standard error of abundance
0.094
74087
Sorbaria sorbifolia
Abundance
1
743588
Sorbaria sorbifolia
Standard error of abundance
0.00078
65052
Sorbus alnifolia
Abundance
1
15133
Sorbus alnifolia
Standard error of abundance
0.17
14201
125°E
130°E
135°E
140°E
145°E
25°N
30°N
35°N
40°N
45°N

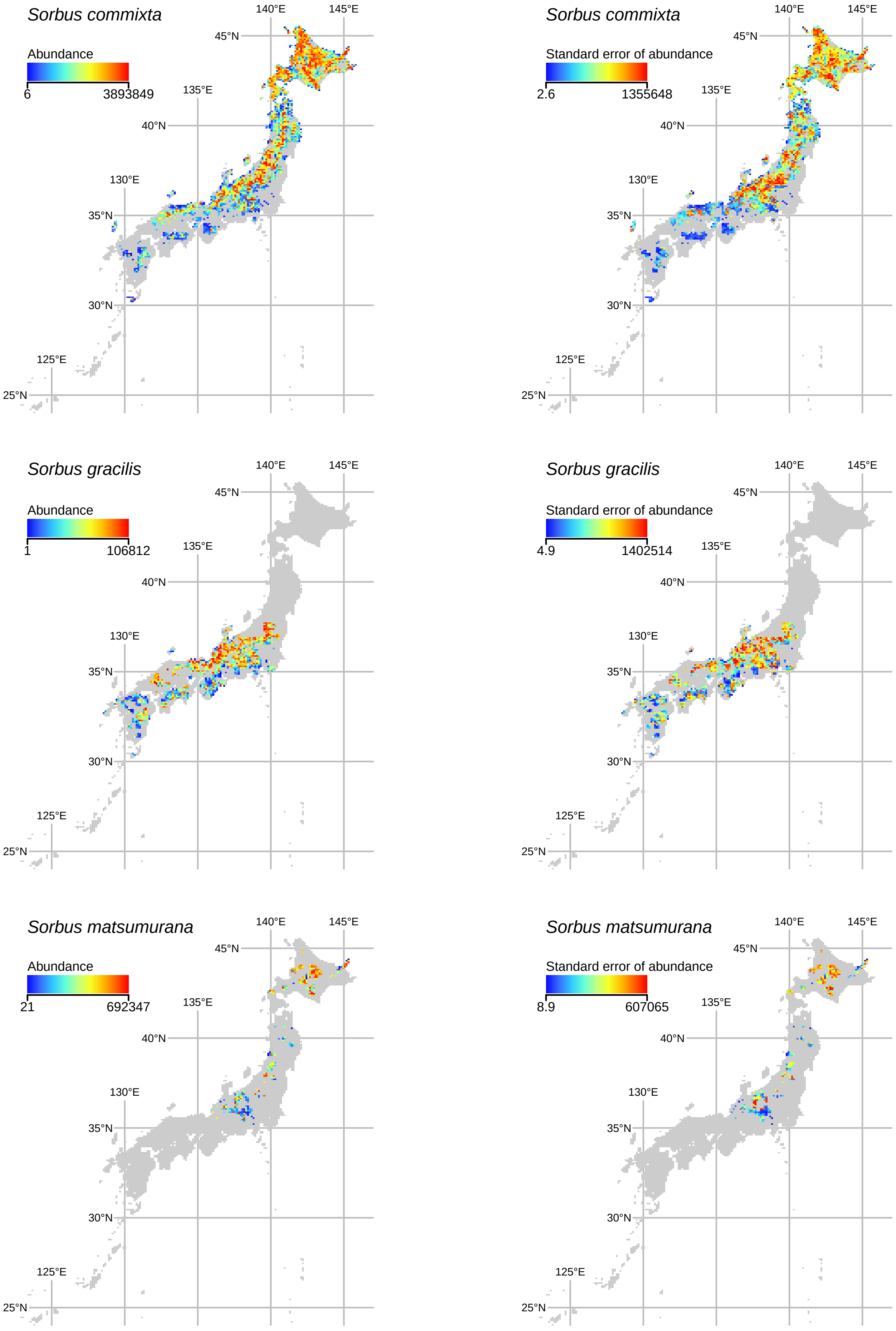
Sorbus commixta
Abundance
6
3893849
Sorbus commixta
Standard error of abundance
2.6
1355648
Sorbus gracilis
Abundance
1
106812
Sorbus gracilis
Standard error of abundance
4.9
1402514
Sorbus matsumurana
Abundance
21
692347
Sorbus matsumurana
Standard error of abundance
8.9
607065
125°E
130°E
135°E
140°E
145°E
25°N
30°N
35°N
40°N
45°N

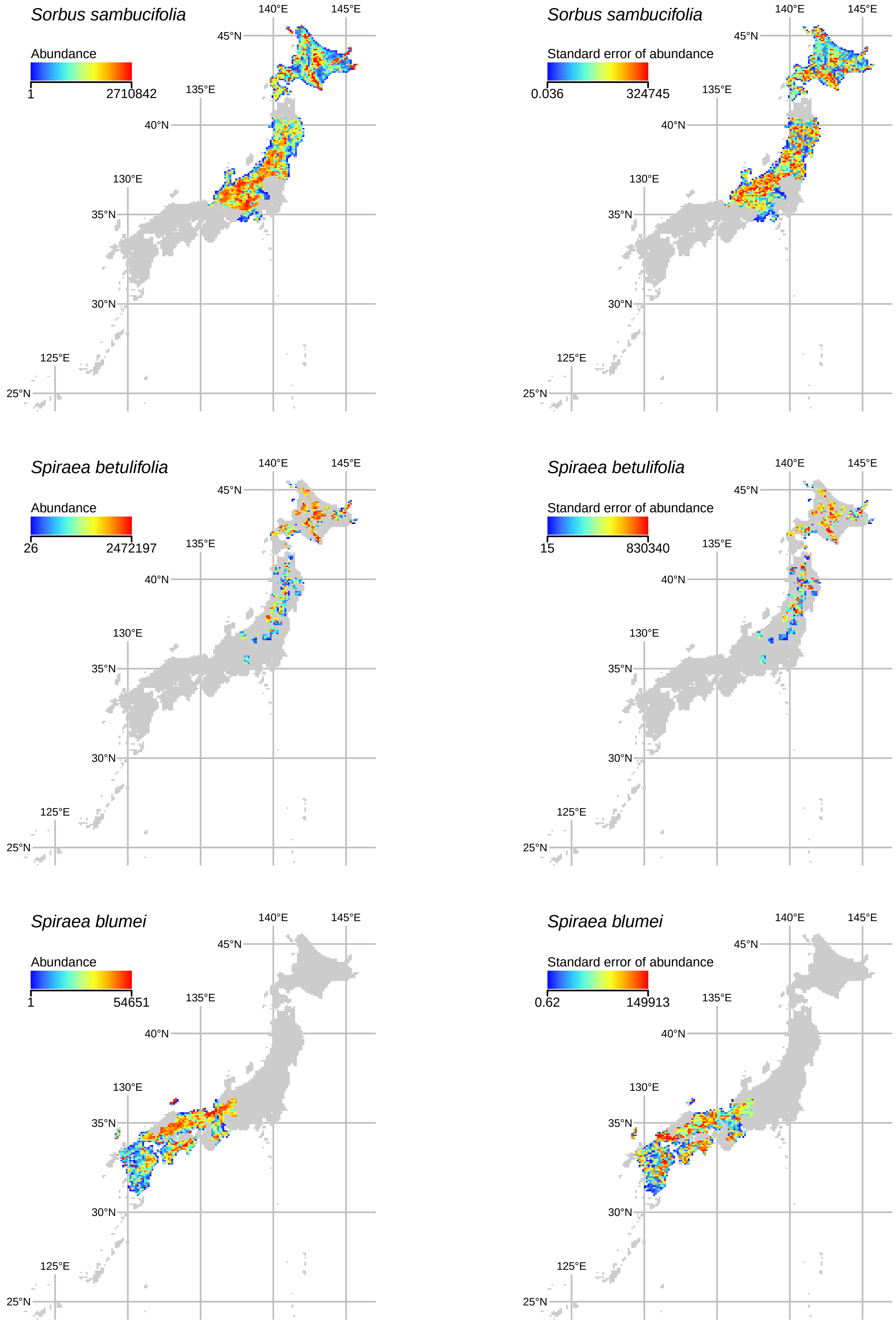
Sorbus sambucifolia
Abundance
1
2710842
Sorbus sambucifolia
Standard error of abundance
0.036
324745
Spiraea betulifolia
Abundance
26
2472197
Spiraea betulifolia
Standard error of abundance
15
830340
Spiraea blumei
Abundance
1
54651
Spiraea blumei
Standard error of abundance
0.62
149913
125°E
130°E
135°E
140°E
145°E
25°N
30°N
35°N
40°N
45°N

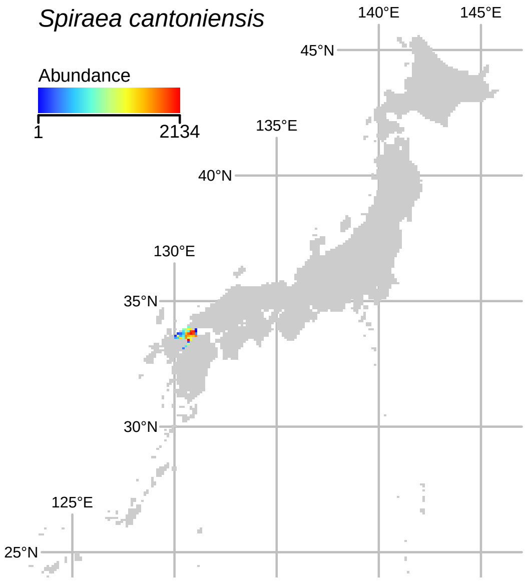
Spiraea cantoniensis
Abundance
1
2134
140°E
145°E
45°N
135°E
40°N
130°E
35°N
30°N
125°E
25°N

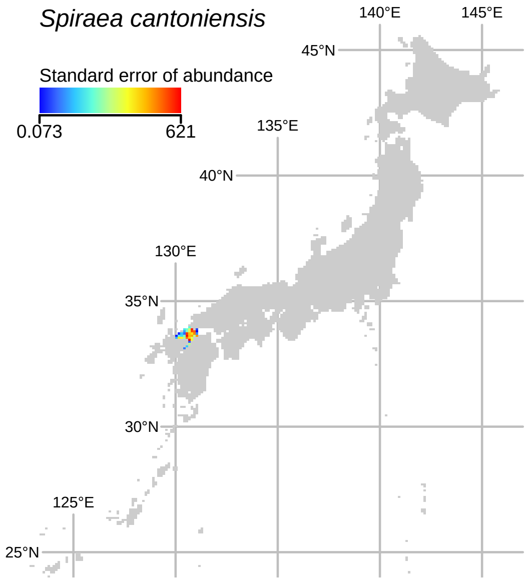
Spiraea cantoniensis
Standard error of abundance
0.073
621
140°E
145°E
45°N
135°E
40°N
130°E
35°N
30°N
125°E
25°N

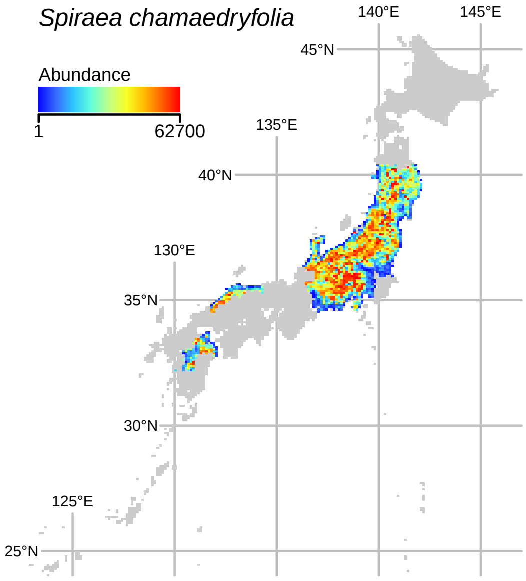
Spiraea chamaedryfolia
Abundance
1
62700
140°E
145°E
45°N
135°E
40°N
130°E
35°N
30°N
125°E
25°N

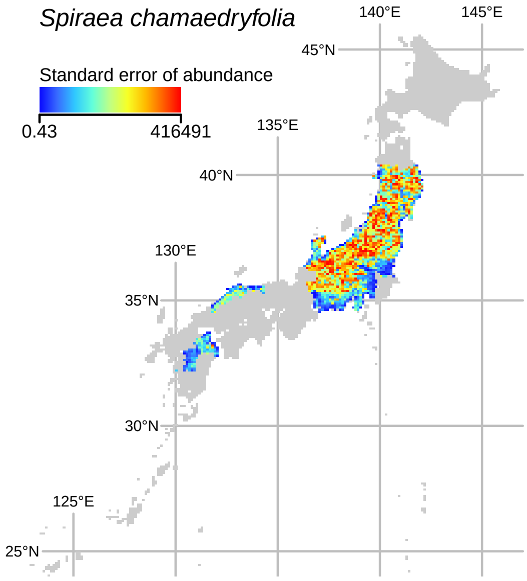
Spiraea chamaedryfolia
Standard error of abundance
0.43
416491
140°E
145°E
45°N
135°E
40°N
130°E
35°N
30°N
125°E
25°N

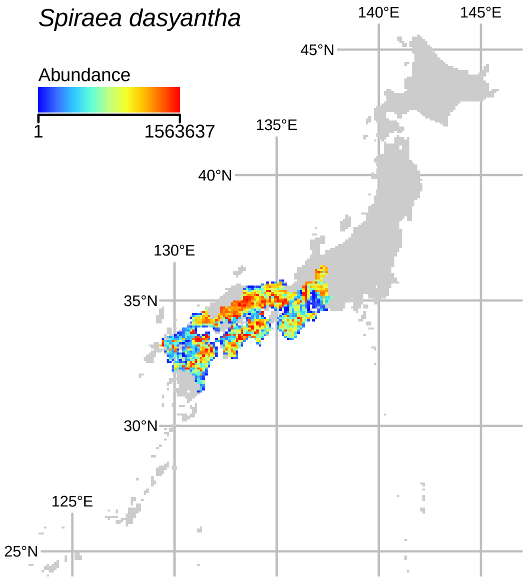
Spiraea dasyantha
Abundance
1
1563637
140°E
145°E
45°N
135°E
40°N
130°E
35°N
30°N
125°E
25°N

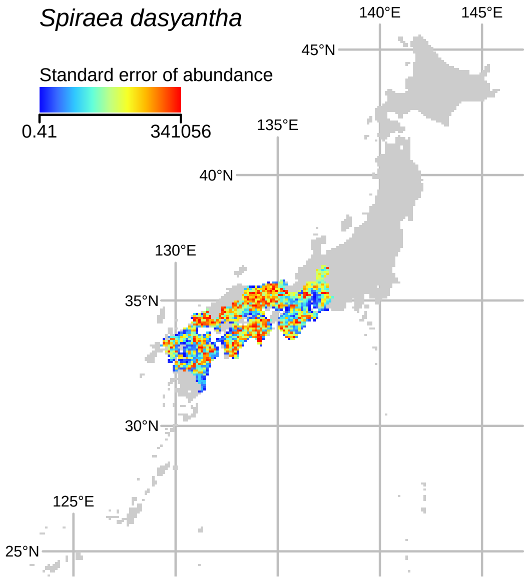
Spiraea dasyantha
Standard error of abundance
0.41
341056
140°E
145°E
45°N
135°E
40°N
130°E
35°N
30°N
125°E
25°N

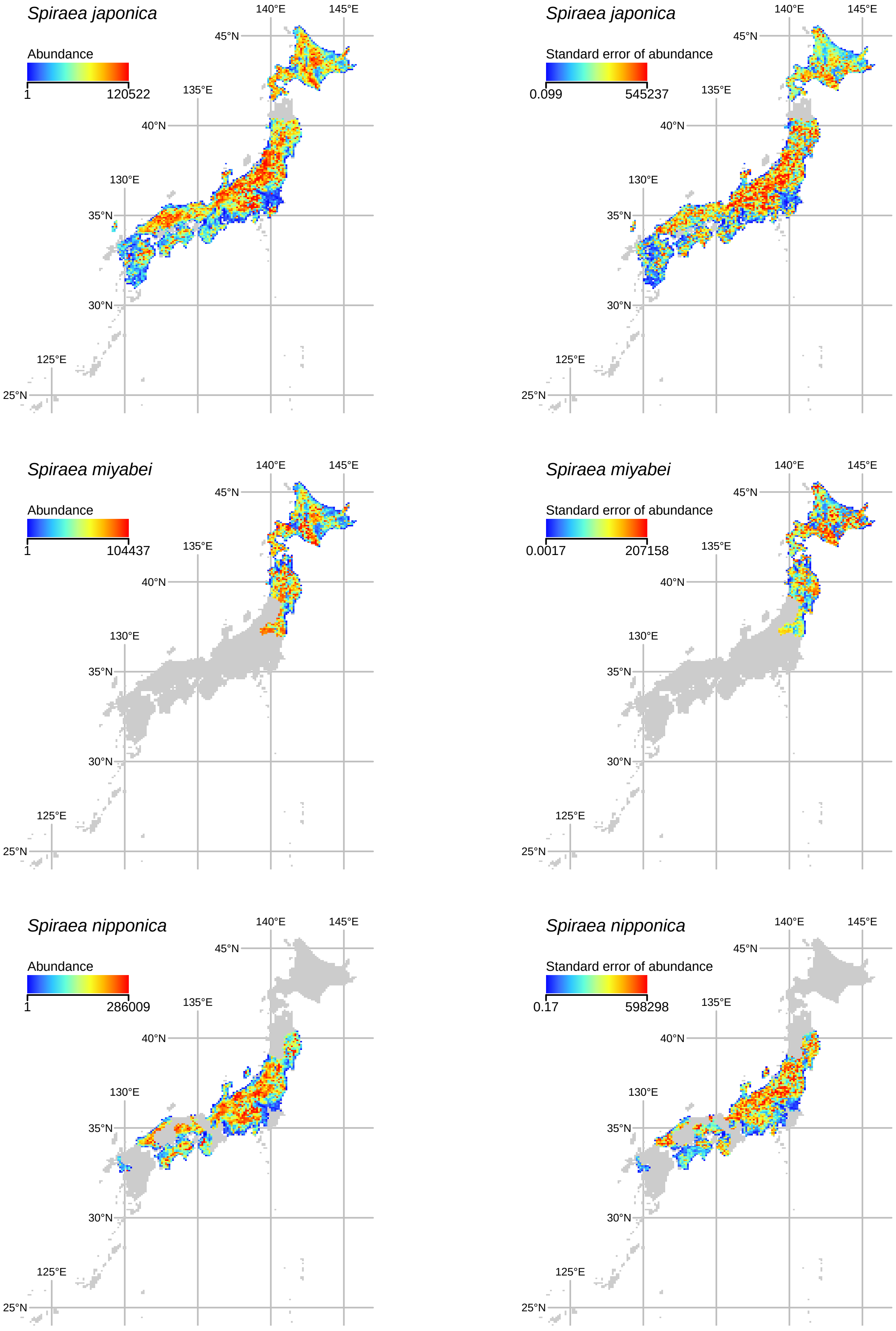
Spiraea japonica
Abundance
1
120522
Spiraea japonica
Standard error of abundance
0.099
545237
Spiraea miyabei
Abundance
1
104437
Spiraea miyabei
Standard error of abundance
0.0017
207158
Spiraea nipponica
Abundance
1
286009
Spiraea nipponica
Standard error of abundance
0.17
598298
125°E
130°E
135°E
140°E
145°E
25°N
30°N
35°N
40°N
45°N

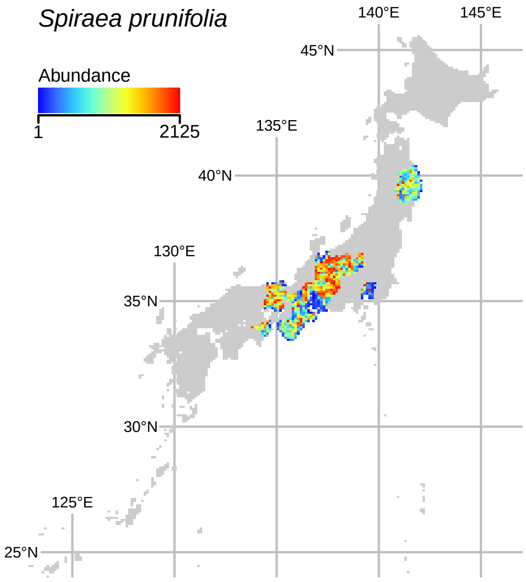
Spiraea prunifolia
Abundance
1
2125
140°E
145°E
135°E
130°E
125°E
45°N
40°N
35°N
30°N
25°N

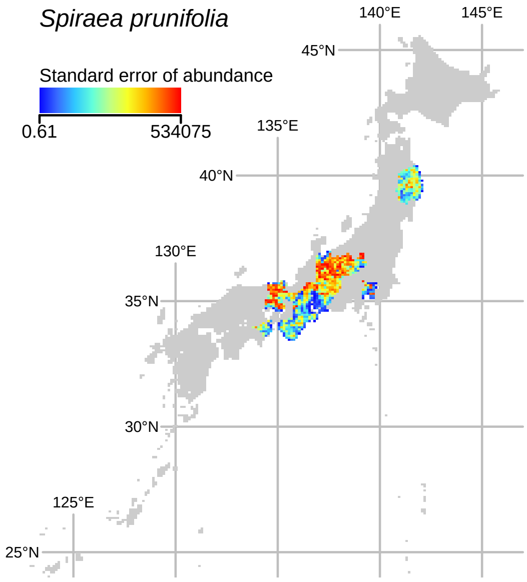
Spiraea prunifolia
Standard error of abundance
0.61
534075
140°E
145°E
135°E
130°E
125°E
45°N
40°N
35°N
30°N
25°N

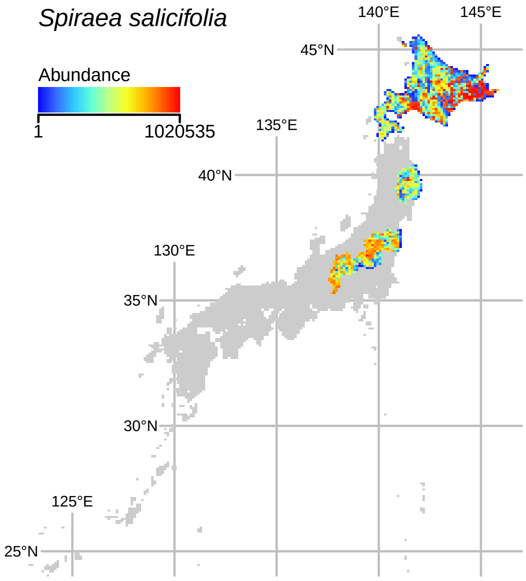
Spiraea salicifolia
Abundance
1
1020535
140°E
145°E
135°E
130°E
125°E
45°N
40°N
35°N
30°N
25°N

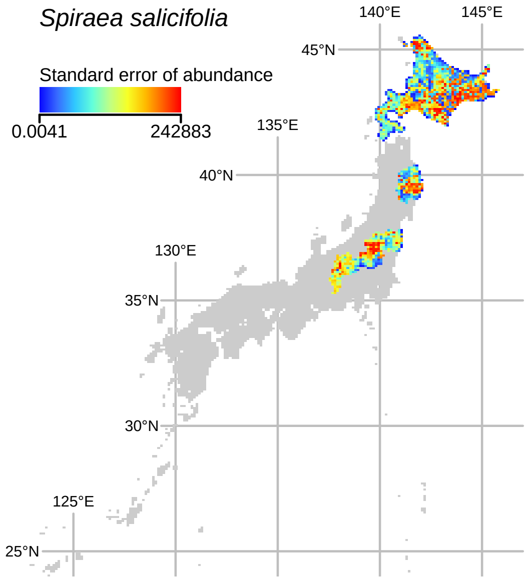
Spiraea salicifolia
Standard error of abundance
0.0041
242883
140°E
145°E
135°E
130°E
125°E
45°N
40°N
35°N
30°N
25°N

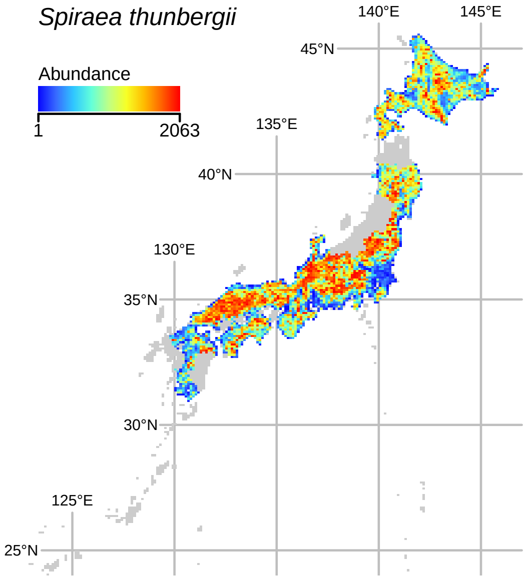
Spiraea thunbergii
Abundance
1
2063
140°E
145°E
135°E
130°E
125°E
45°N
40°N
35°N
30°N
25°N

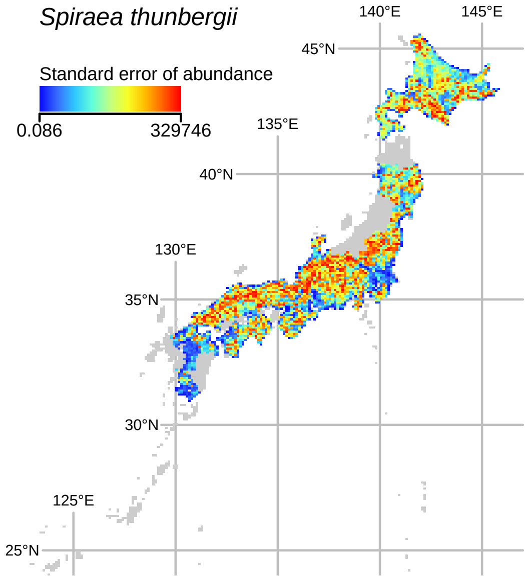
Spiraea thunbergii
Standard error of abundance
0.086
329746
140°E
145°E
135°E
130°E
125°E
45°N
40°N
35°N
30°N
25°N

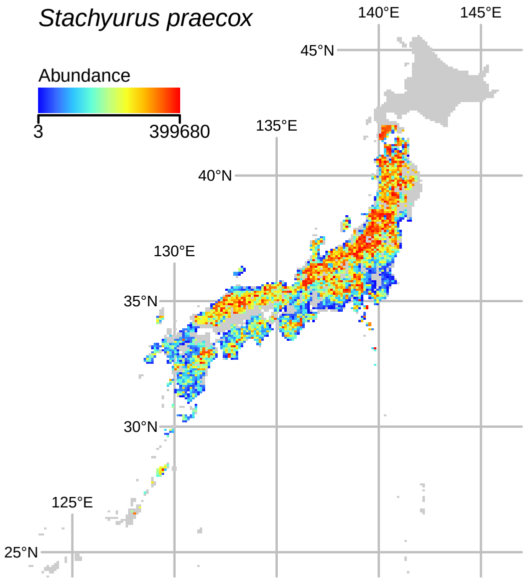
Stachyurus praecox
Abundance
3
399680
140°E
145°E
45°N
135°E
40°N
130°E
35°N
30°N
125°E
25°N

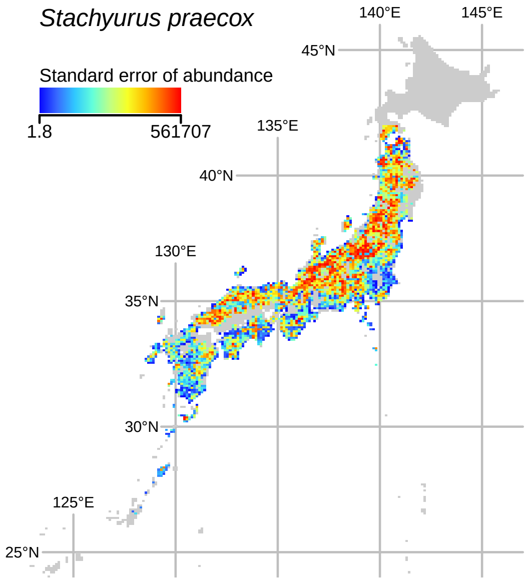
Stachyurus praecox
Standard error of abundance
1.8
561707
140°E
145°E
45°N
135°E
40°N
130°E
35°N
30°N
125°E
25°N

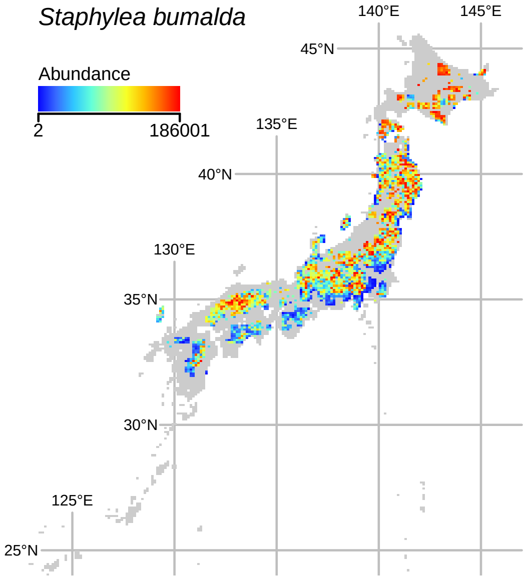
Staphylea bumalda
Abundance
2
186001
140°E
145°E
45°N
135°E
40°N
130°E
35°N
30°N
125°E
25°N

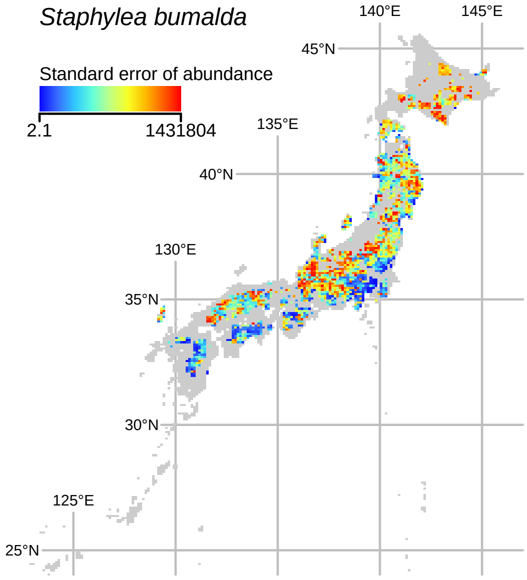
Staphylea bumalda
Standard error of abundance
2.1
1431804
140°E
145°E
45°N
135°E
40°N
130°E
35°N
30°N
125°E
25°N

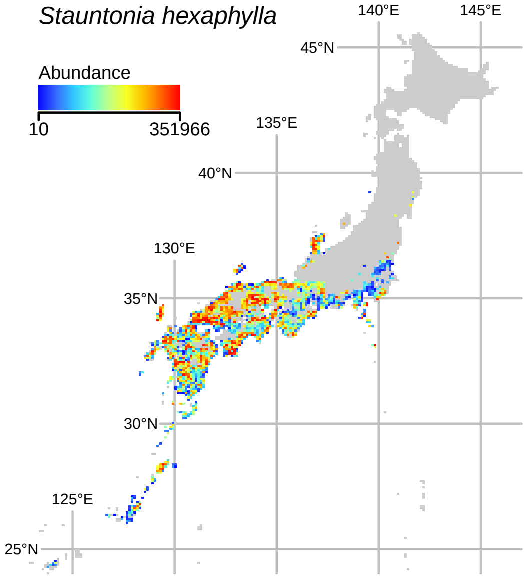
Stauntonia hexaphylla
Abundance
10
351966
140°E
145°E
45°N
135°E
40°N
130°E
35°N
30°N
125°E
25°N

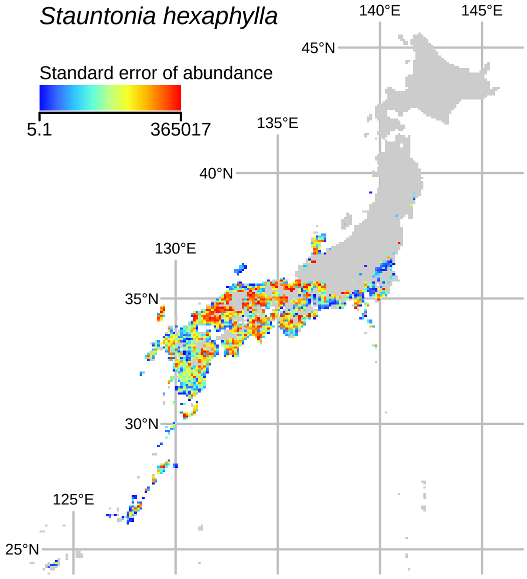
Stauntonia hexaphylla
Standard error of abundance
5.1
365017
140°E
145°E
45°N
135°E
40°N
130°E
35°N
30°N
125°E
25°N

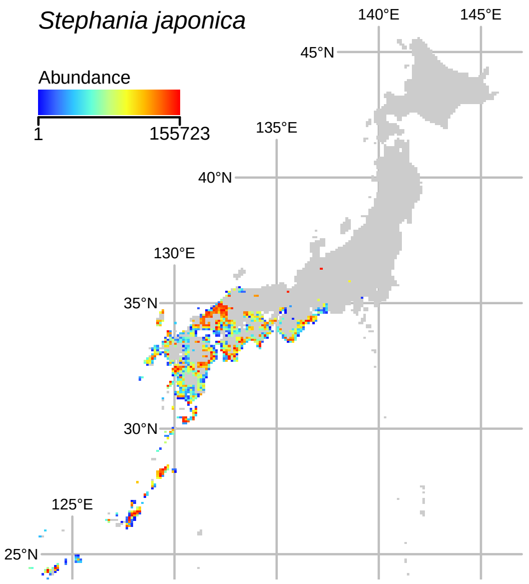
Stephania japonica
Abundance
1
155723
140°E
145°E
45°N
135°E
40°N
130°E
35°N
30°N
125°E
25°N

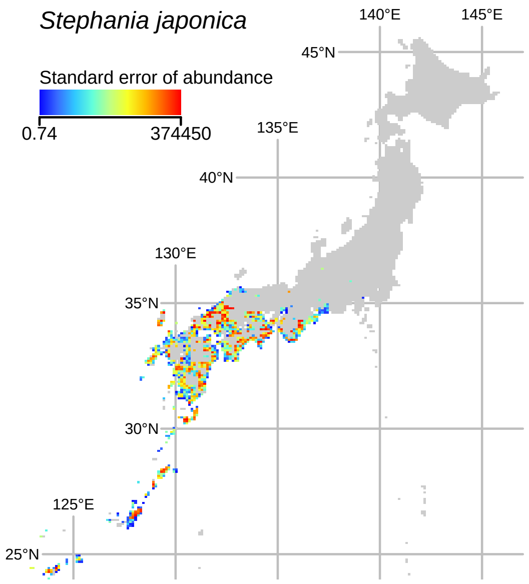
Stephania japonica
Standard error of abundance
0.74
374450
140°E
145°E
45°N
135°E
40°N
130°E
35°N
30°N
125°E
25°N

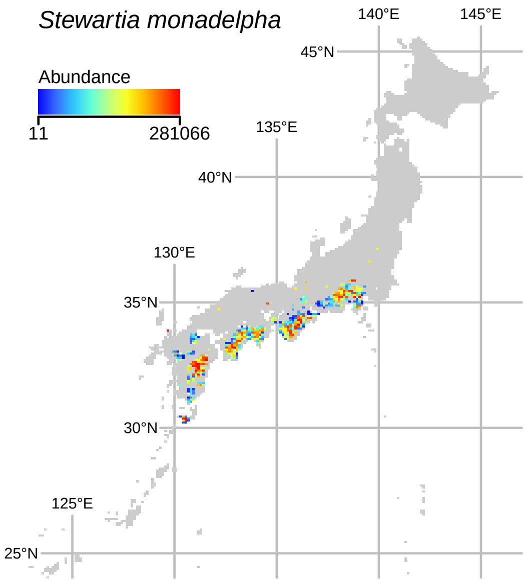
Stewartia monadelpha
Abundance
11
281066
140°E
145°E
45°N
135°E
40°N
130°E
35°N
30°N
125°E
25°N

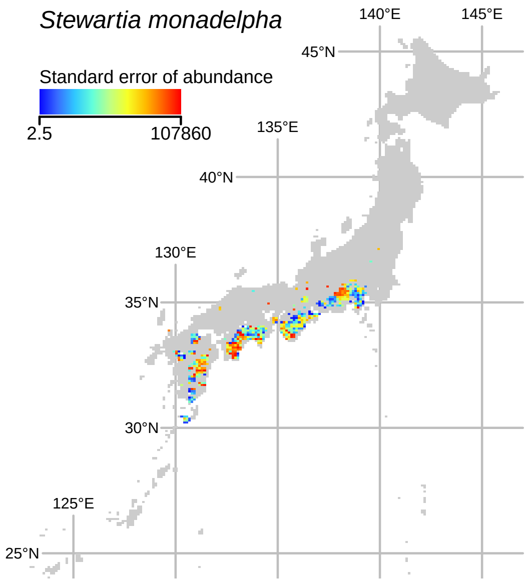
Stewartia monadelpha
Standard error of abundance
2.5
107860
140°E
145°E
45°N
135°E
40°N
130°E
35°N
30°N
125°E
25°N

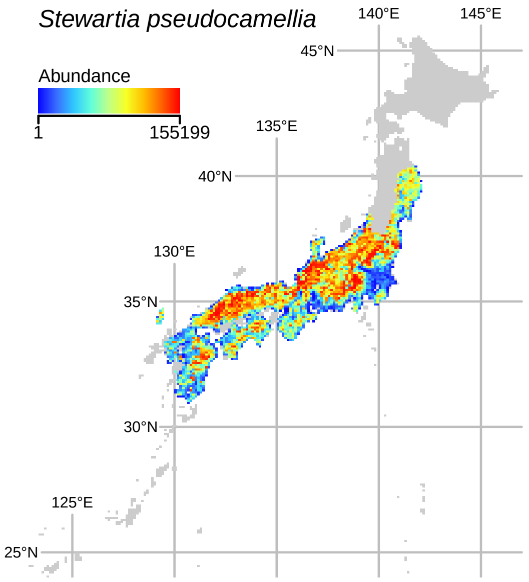
Stewartia pseudocamellia
Abundance
1
155199
140°E
145°E
45°N
135°E
40°N
130°E
35°N
30°N
125°E
25°N

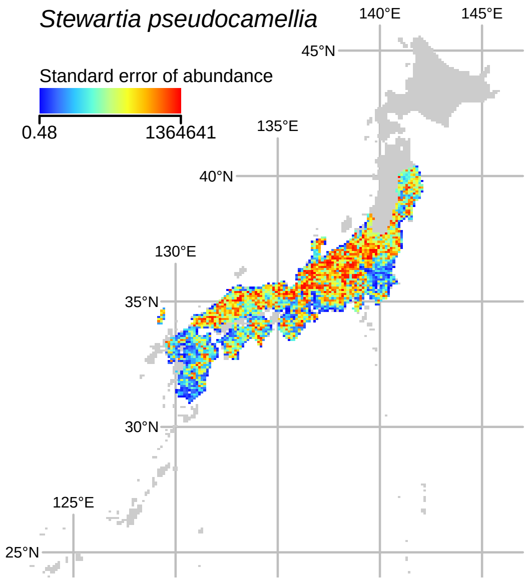
Stewartia pseudocamellia
Standard error of abundance
0.48
1364641
140°E
145°E
45°N
135°E
40°N
130°E
35°N
30°N
125°E
25°N

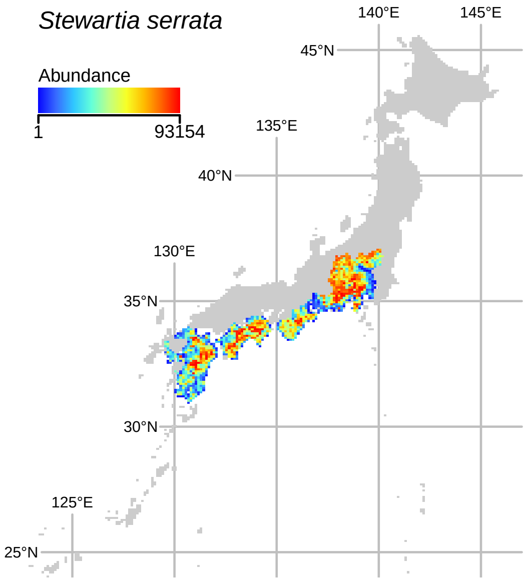
Stewartia serrata
Abundance
1
93154
140°E
145°E
45°N
135°E
40°N
130°E
35°N
30°N
125°E
25°N

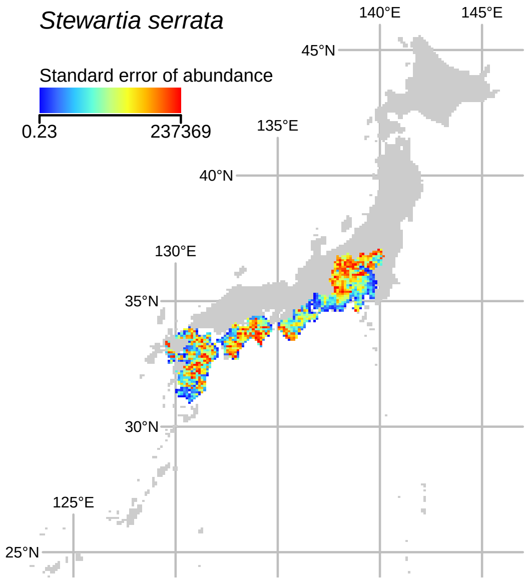
Stewartia serrata
Standard error of abundance
0.23
237369
140°E
145°E
45°N
135°E
40°N
130°E
35°N
30°N
125°E
25°N

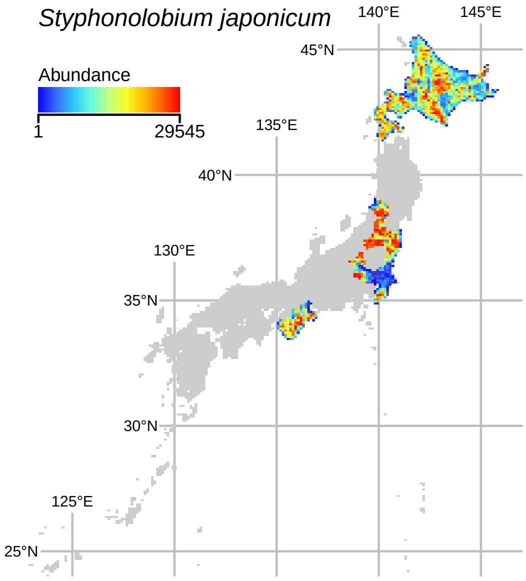
Styphonolobium japonicum
Abundance
1
29545
140°E
145°E
45°N
135°E
40°N
130°E
35°N
30°N
125°E
25°N

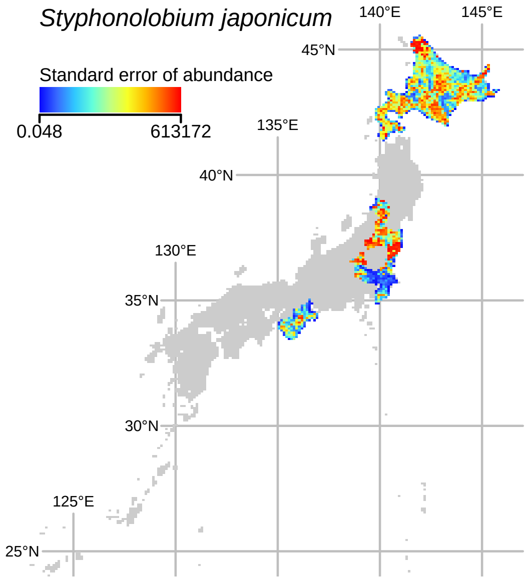
Styphonolobium japonicum
Standard error of abundance
0.048
613172
140°E
145°E
45°N
135°E
40°N
130°E
35°N
30°N
125°E
25°N

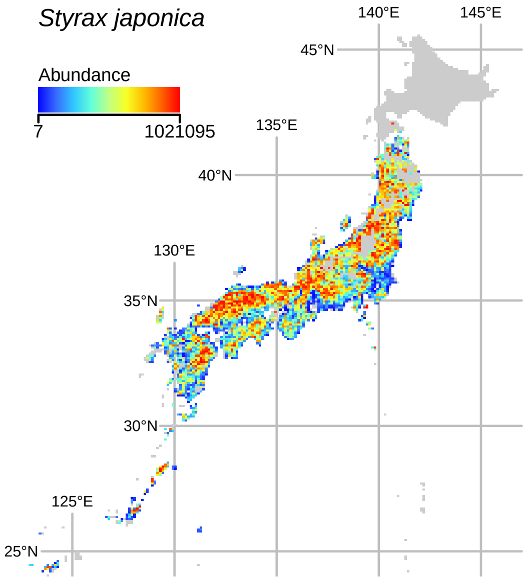
Styrax japonica
Abundance
7
1021095
140°E
145°E
45°N
135°E
40°N
130°E
35°N
30°N
125°E
25°N

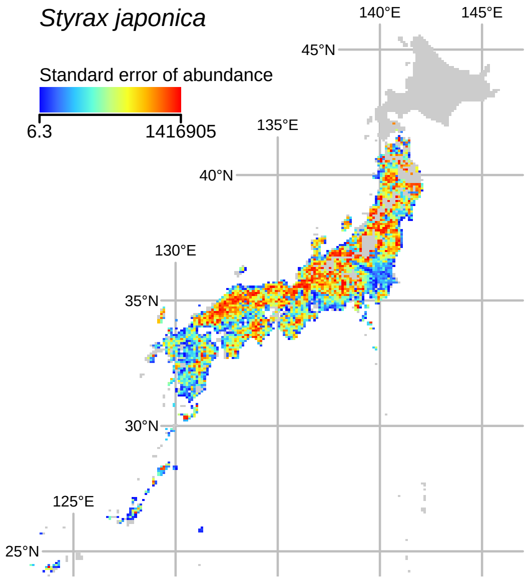
Styrax japonica
Standard error of abundance
6.3
1416905
140°E
145°E
45°N
135°E
40°N
130°E
35°N
30°N
125°E
25°N

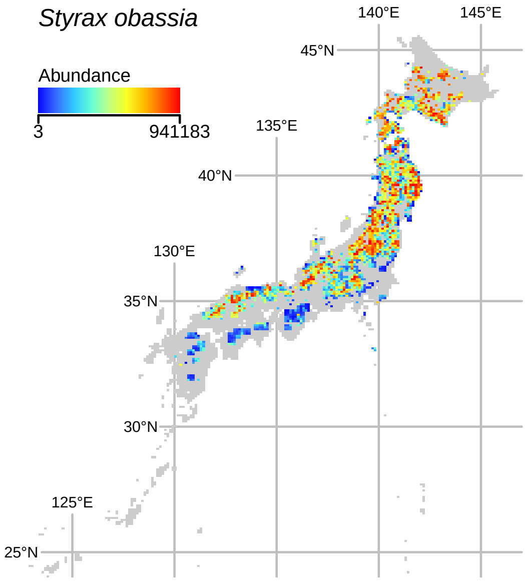

Styrax obassia
Abundance
3
941183
140°E
145°E
45°N
135°E
40°N
130°E
35°N
30°N
125°E
25°N

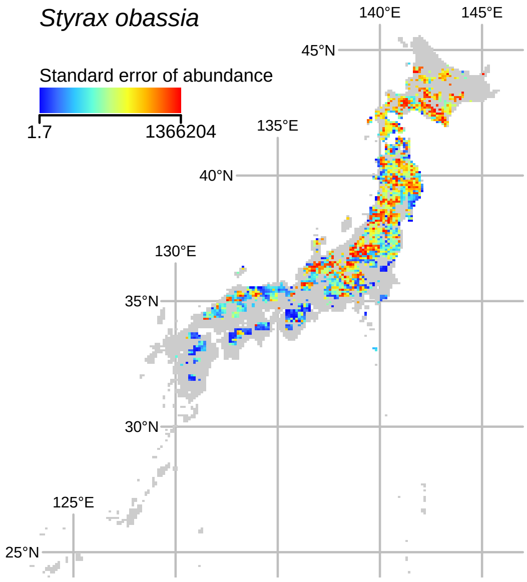

Styrax obassia
Standard error of abundance
1.7
1366204
140°E
145°E
45°N
135°E
40°N
130°E
35°N
30°N
125°E
25°N

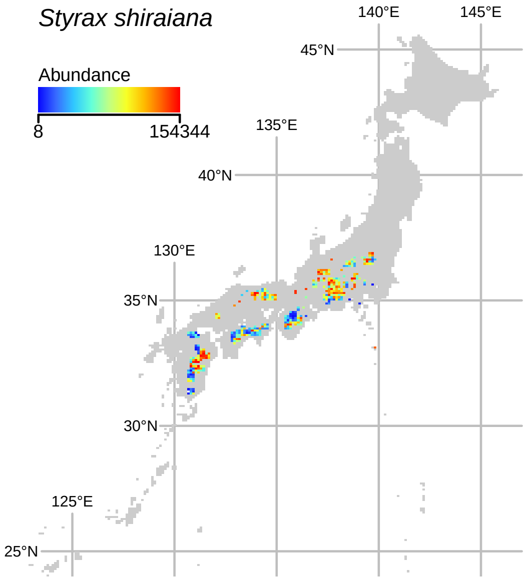

Styrax shiraiana
Abundance
8
154344
140°E
145°E
45°N
135°E
40°N
130°E
35°N
30°N
125°E
25°N

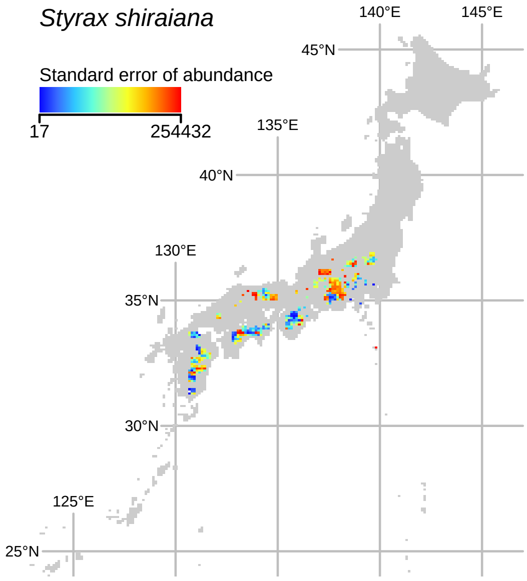

Styrax shiraiana
Standard error of abundance
17
254432
140°E
145°E
45°N
135°E
40°N
130°E
35°N
30°N
125°E
25°N

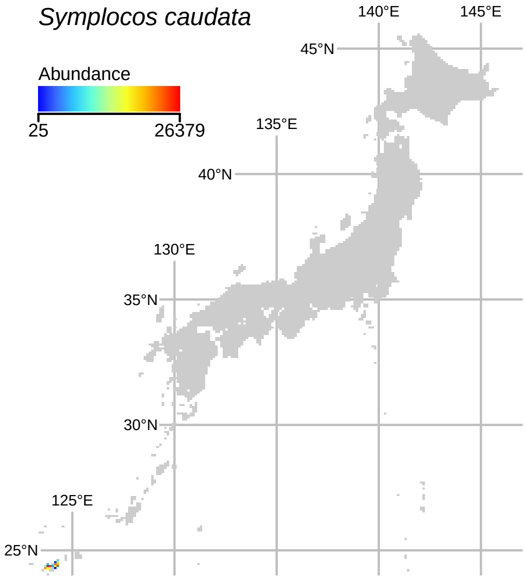

Symplocos caudata
Abundance
25
26379
140°E
145°E
45°N
135°E
40°N
130°E
35°N
30°N
125°E
25°N

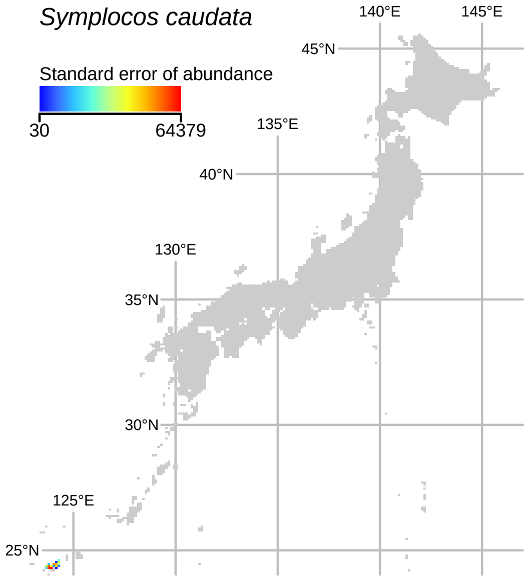

Symplocos caudata
Standard error of abundance
30
64379
140°E
145°E
45°N
135°E
40°N
130°E
35°N
30°N
125°E
25°N

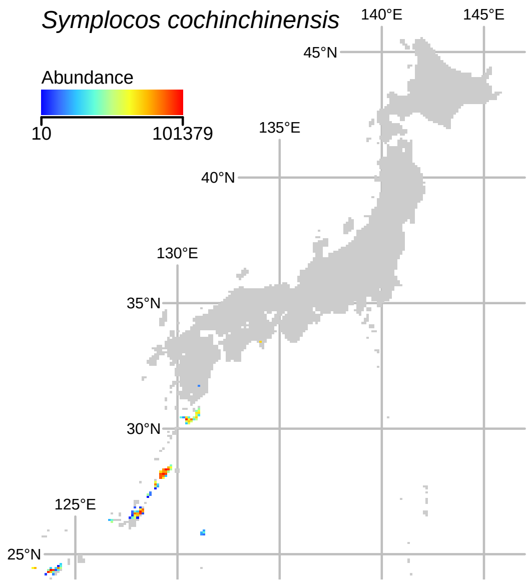
Symplocos cochinchinensis
Abundance
10
101379
140°E
145°E
135°E
130°E
125°E
45°N
40°N
35°N
30°N
25°N

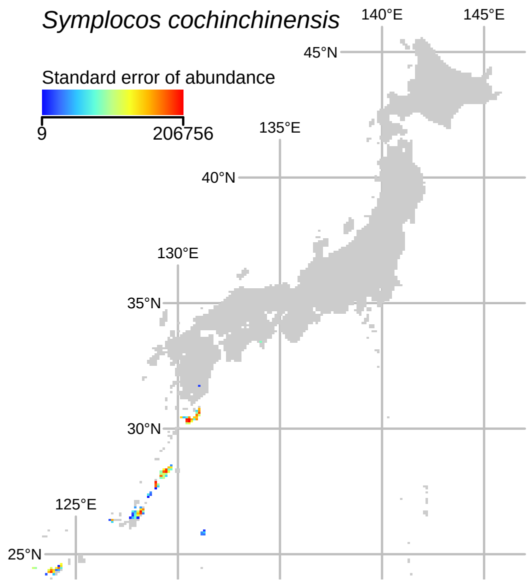
Symplocos cochinchinensis
Standard error of abundance
9
206756
140°E
145°E
135°E
130°E
125°E
45°N
40°N
35°N
30°N
25°N

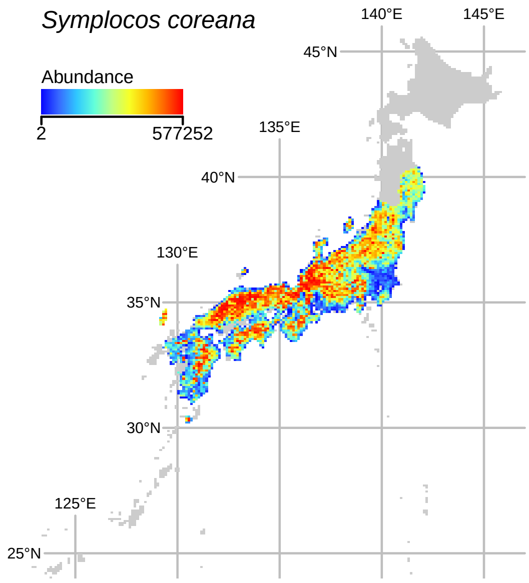
Symplocos coreana
Abundance
2
577252
140°E
145°E
135°E
130°E
125°E
45°N
40°N
35°N
30°N
25°N

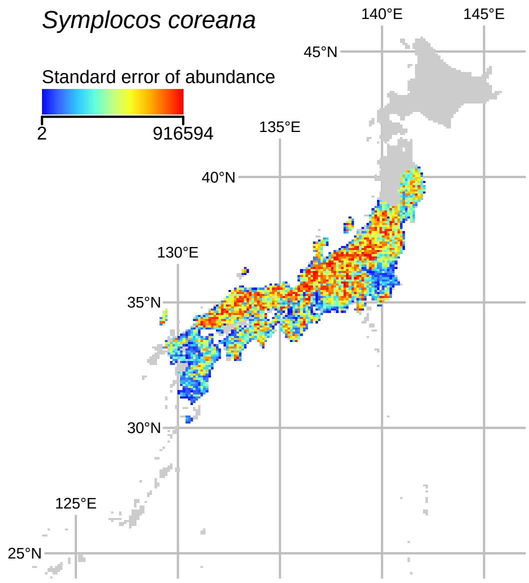
Symplocos coreana
Standard error of abundance
2
916594
140°E
145°E
135°E
130°E
125°E
45°N
40°N
35°N
30°N
25°N

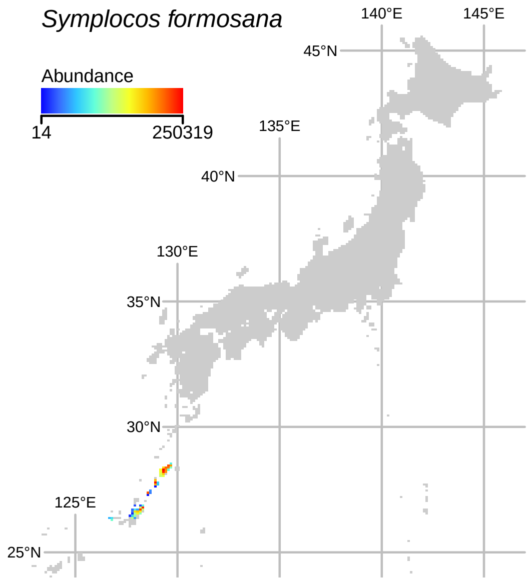
Symplocos formosana
Abundance
14
250319
140°E
145°E
135°E
130°E
125°E
45°N
40°N
35°N
30°N
25°N

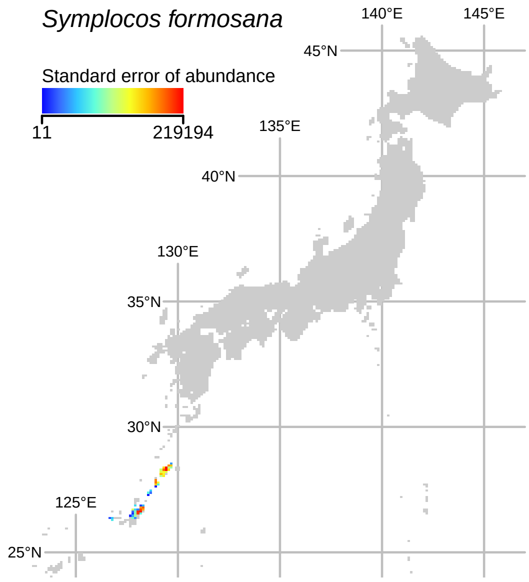
Symplocos formosana
Standard error of abundance
11
219194
140°E
145°E
135°E
130°E
125°E
45°N
40°N
35°N
30°N
25°N

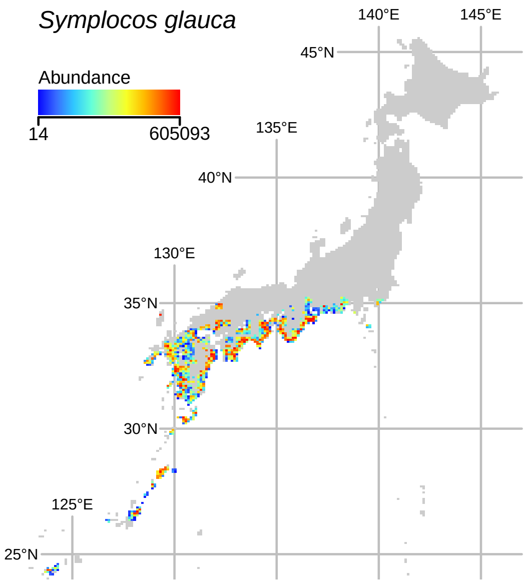
Symplocos glauca
Abundance
14
605093
140°E
145°E
45°N
135°E
40°N
130°E
35°N
30°N
125°E
25°N

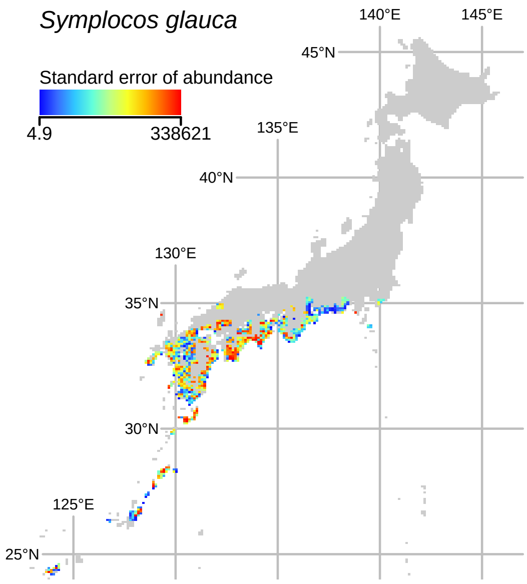
Symplocos glauca
Standard error of abundance
4.9
338621
140°E
145°E
45°N
135°E
40°N
130°E
35°N
30°N
125°E
25°N

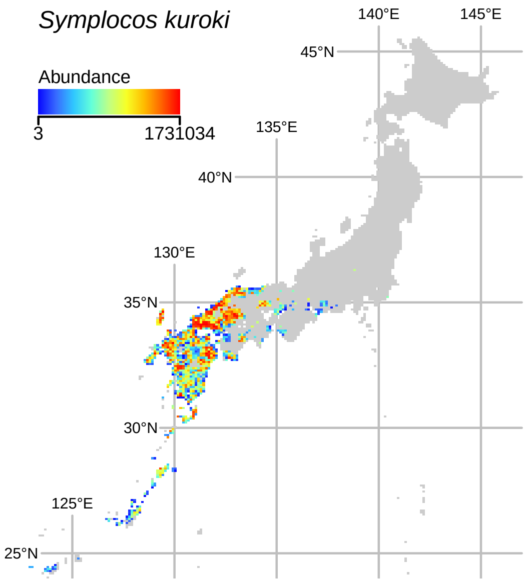
Symplocos kuroki
Abundance
3
1731034
140°E
145°E
45°N
135°E
40°N
130°E
35°N
30°N
125°E
25°N

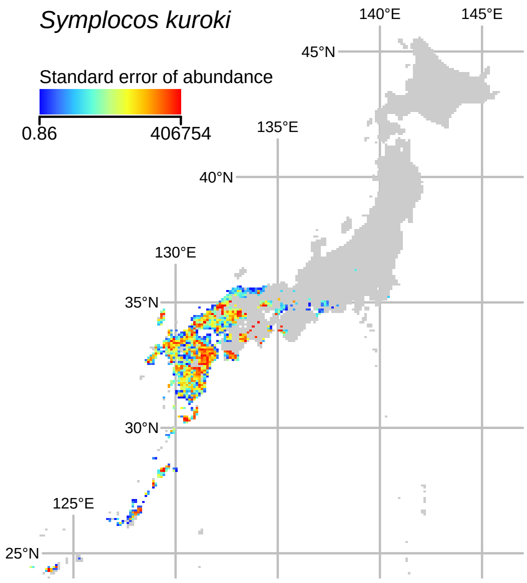
Symplocos kuroki
Standard error of abundance
0.86
406754
140°E
145°E
45°N
135°E
40°N
130°E
35°N
30°N
125°E
25°N

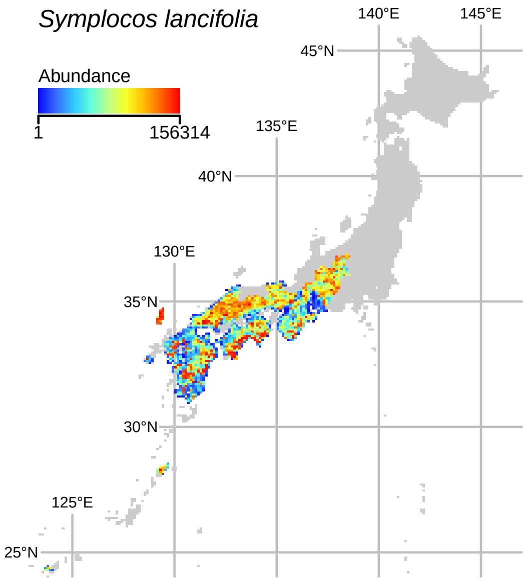
Symplocos lancifolia
Abundance
1
156314
140°E
145°E
45°N
135°E
40°N
130°E
35°N
30°N
125°E
25°N

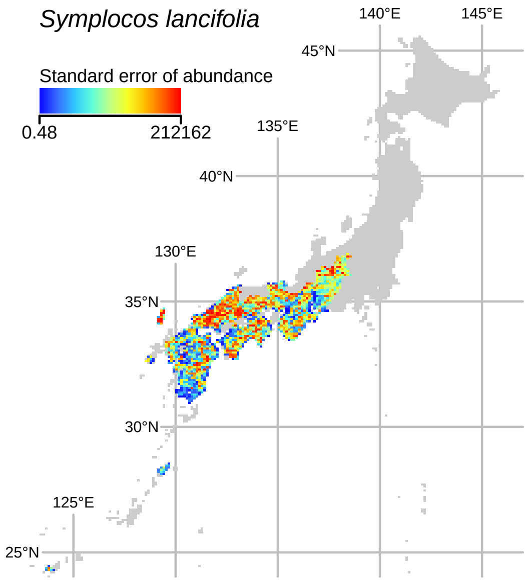
Symplocos lancifolia
Standard error of abundance
0.48
212162
140°E
145°E
45°N
135°E
40°N
130°E
35°N
30°N
125°E
25°N

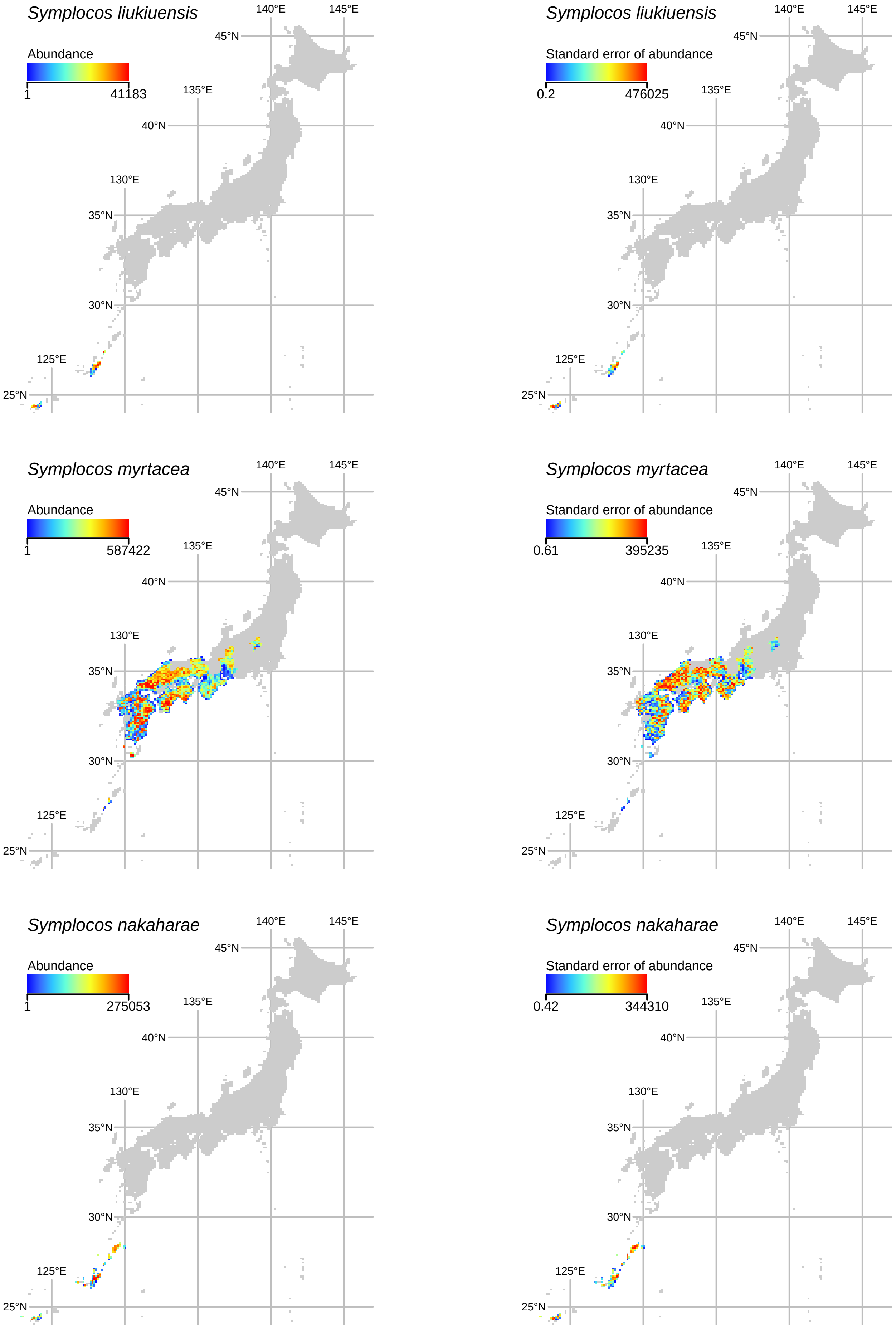
Symplocos liukiuensis
Abundance
1
41183
Symplocos liukiuensis
Standard error of abundance
0.2
476025
Symplocos myrtacea
Abundance
1
587422
Symplocos myrtacea
Standard error of abundance
0.61
395235
Symplocos nakaharae
Abundance
1
275053
Symplocos nakaharae
Standard error of abundance
0.42
344310
125°E
130°E
135°E
140°E
145°E
25°N
30°N
35°N
40°N
45°N

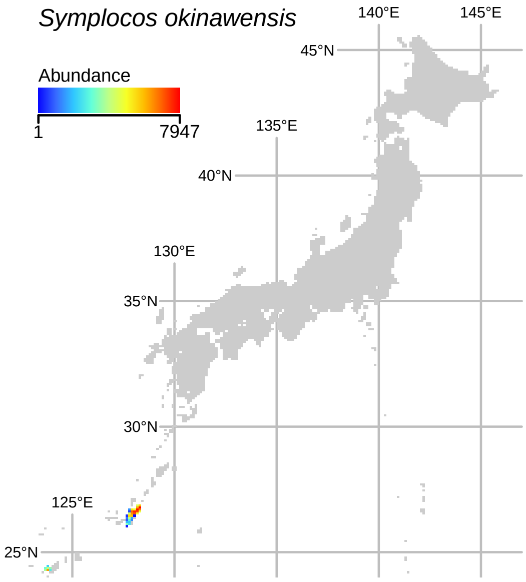
Symplocos okinawensis
Abundance
1
7947
140°E
145°E
45°N
135°E
40°N
130°E
35°N
30°N
125°E
25°N

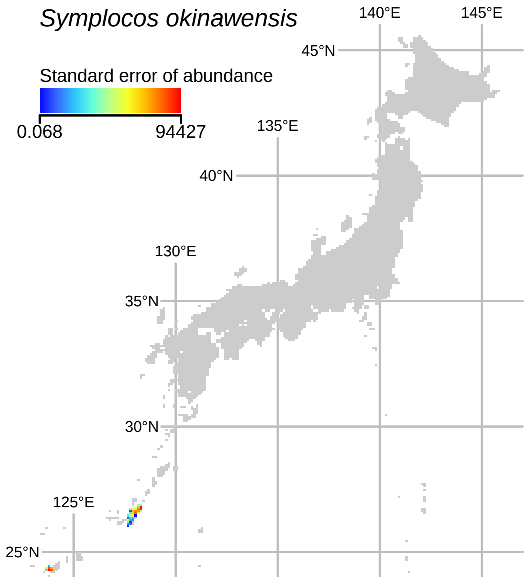
Symplocos okinawensis
Standard error of abundance
0.068
94427
140°E
145°E
45°N
135°E
40°N
130°E
35°N
30°N
125°E
25°N

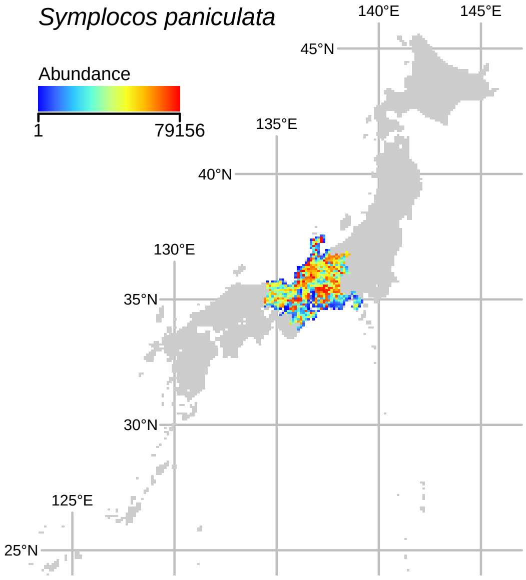
Symplocos paniculata
Abundance
1
79156
140°E
145°E
45°N
135°E
40°N
130°E
35°N
30°N
125°E
25°N

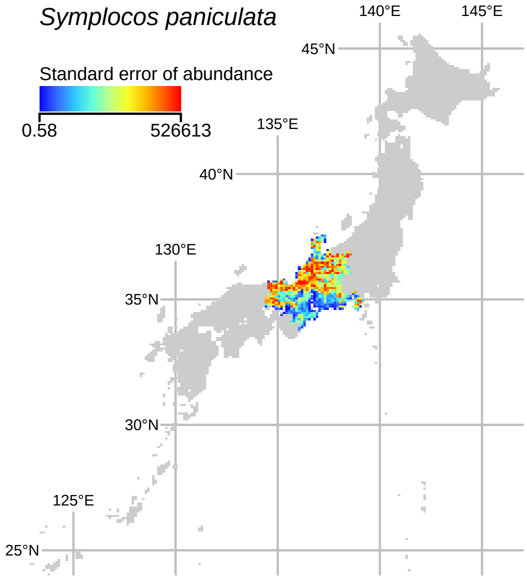
Symplocos paniculata
Standard error of abundance
0.58
526613
140°E
145°E
45°N
135°E
40°N
130°E
35°N
30°N
125°E
25°N

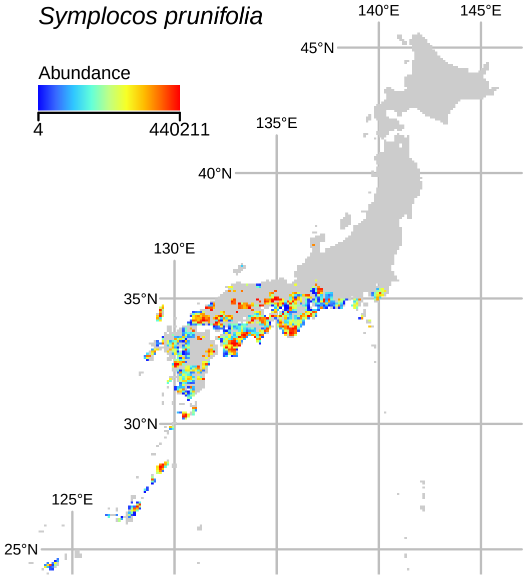
Symplocos prunifolia
Abundance
4
440211
140°E
145°E
45°N
135°E
40°N
130°E
35°N
30°N
125°E
25°N

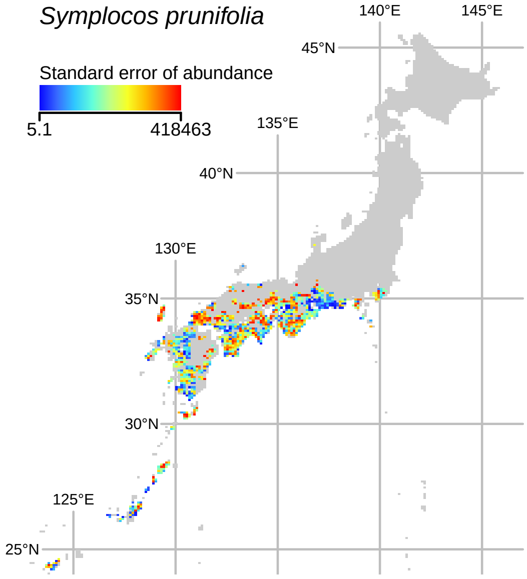
Symplocos prunifolia
Standard error of abundance
5.1
418463
140°E
145°E
45°N
135°E
40°N
130°E
35°N
30°N
125°E
25°N

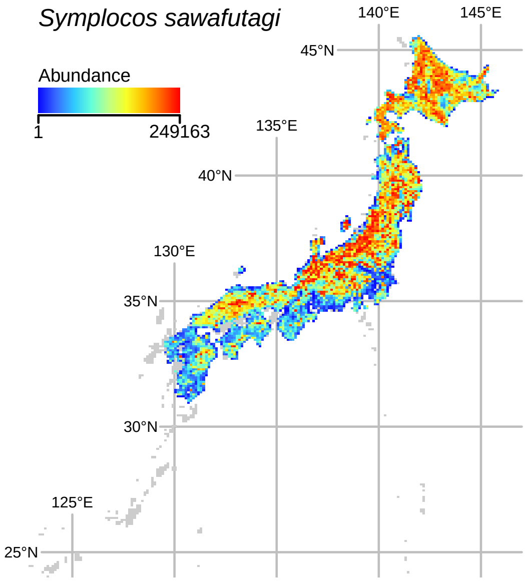
Symplocos sawafutagi
Abundance
1
249163
140°E
145°E
45°N
135°E
40°N
130°E
35°N
30°N
125°E
25°N

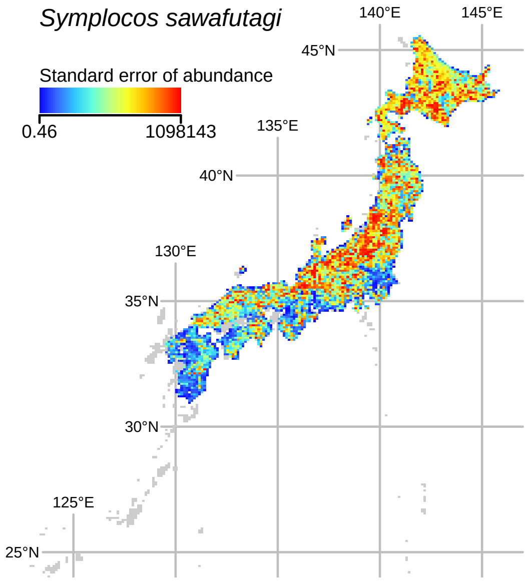
Symplocos sawafutagi
Standard error of abundance
0.46
1098143
140°E
145°E
45°N
135°E
40°N
130°E
35°N
30°N
125°E
25°N

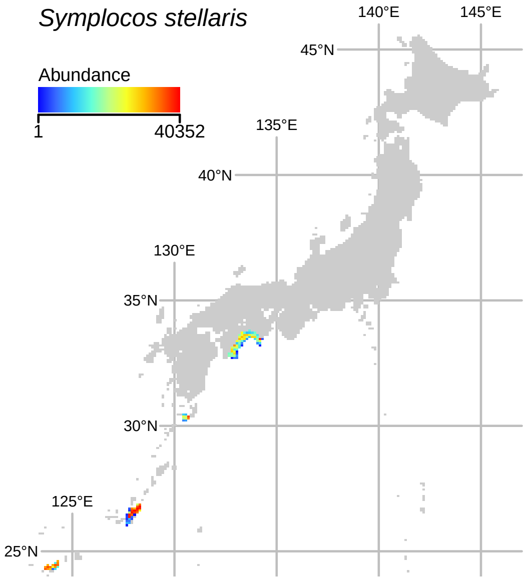
Symplocos stellaris
Abundance
1
40352
140°E
145°E
45°N
135°E
40°N
130°E
35°N
30°N
125°E
25°N

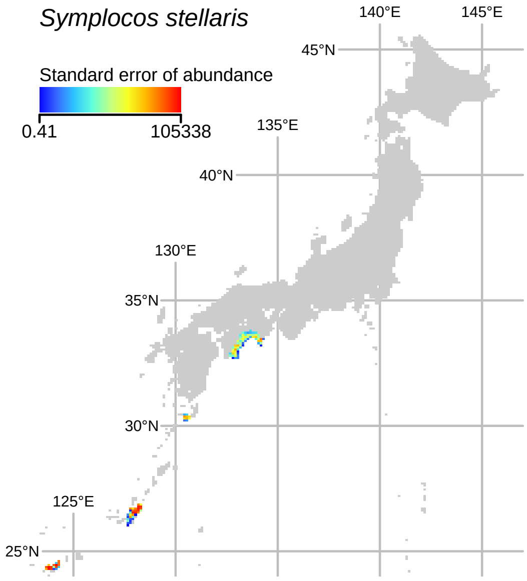
Symplocos stellaris
Standard error of abundance
0.41
105338
140°E
145°E
45°N
135°E
40°N
130°E
35°N
30°N
125°E
25°N

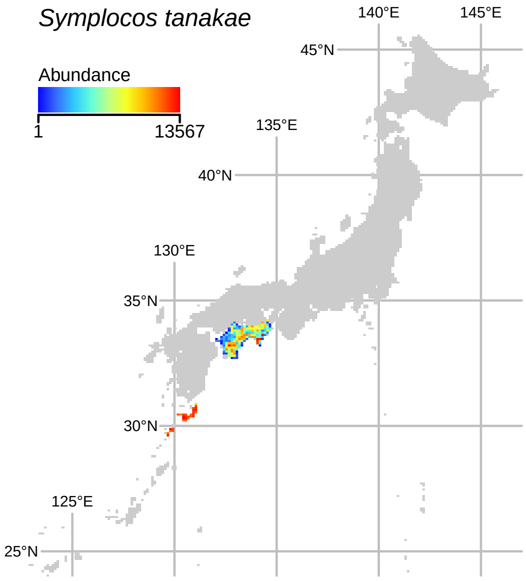
Symplocos tanakae
Abundance
1
13567
140°E
145°E
45°N
135°E
40°N
130°E
35°N
30°N
125°E
25°N

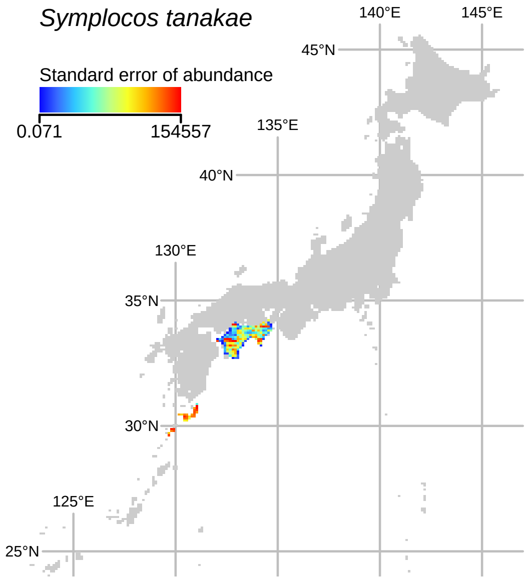
Symplocos tanakae
Standard error of abundance
0.071
154557
140°E
145°E
45°N
135°E
40°N
130°E
35°N
30°N
125°E
25°N

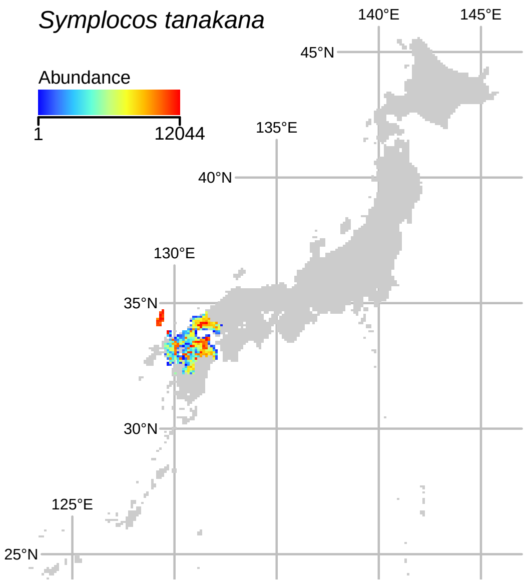
Symplocos tanakana
Abundance
1
12044
140°E
145°E
45°N
135°E
40°N
130°E
35°N
30°N
125°E
25°N

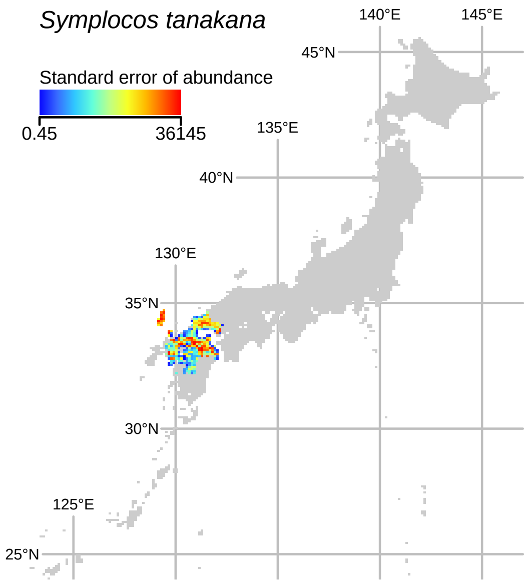
Symplocos tanakana
Standard error of abundance
0.45
36145
140°E
145°E
45°N
135°E
40°N
130°E
35°N
30°N
125°E
25°N

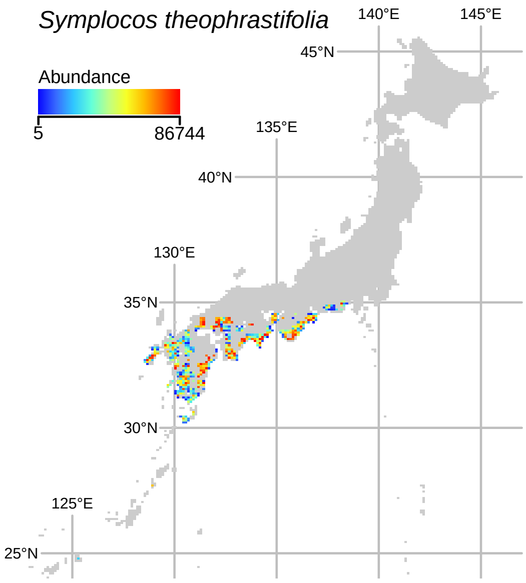
Symplocos theophrastifolia
Abundance
5
86744
140°E
145°E
45°N
135°E
40°N
130°E
35°N
30°N
125°E
25°N

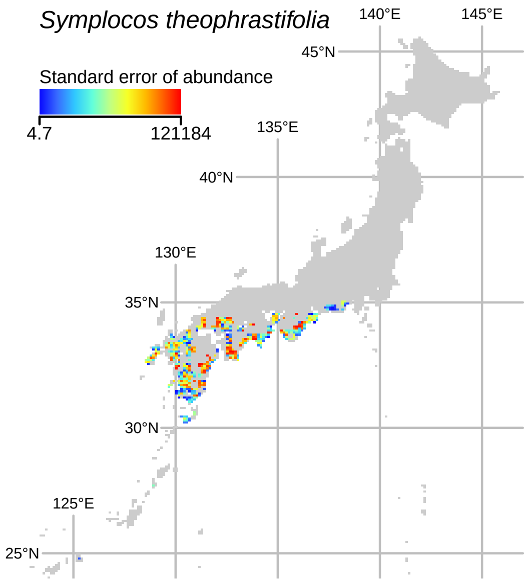
Symplocos theophrastifolia
Standard error of abundance
4.7
121184
140°E
145°E
45°N
135°E
40°N
130°E
35°N
30°N
125°E
25°N

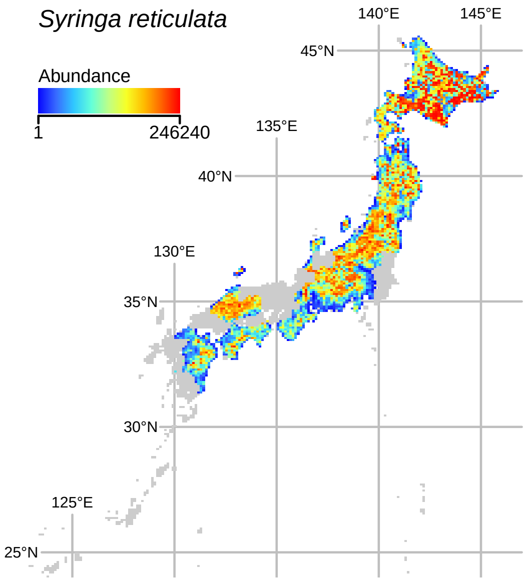
Syringa reticulata
Abundance
1
246240
140°E
145°E
45°N
135°E
40°N
130°E
35°N
30°N
125°E
25°N

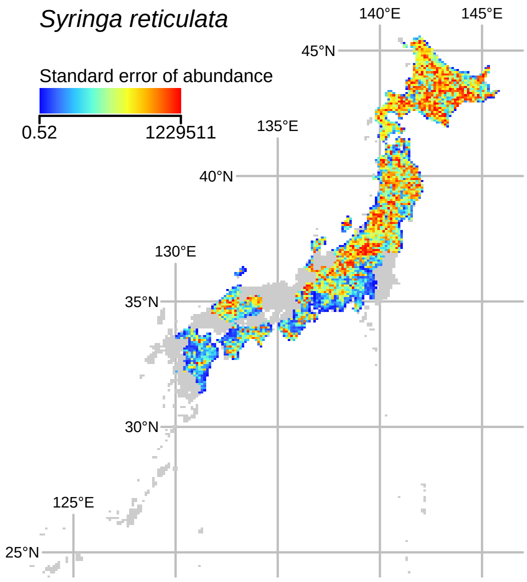
Syringa reticulata
Standard error of abundance
0.52
1229511
140°E
145°E
45°N
135°E
40°N
130°E
35°N
30°N
125°E
25°N

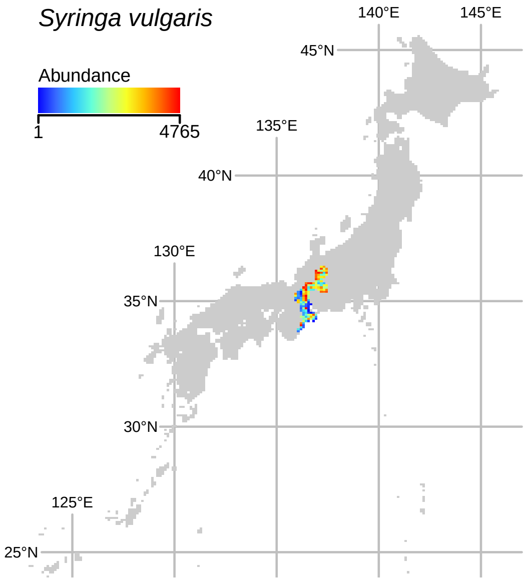
Syringa vulgaris
Abundance
1
4765
140°E
145°E
45°N
135°E
40°N
130°E
35°N
30°N
125°E
25°N

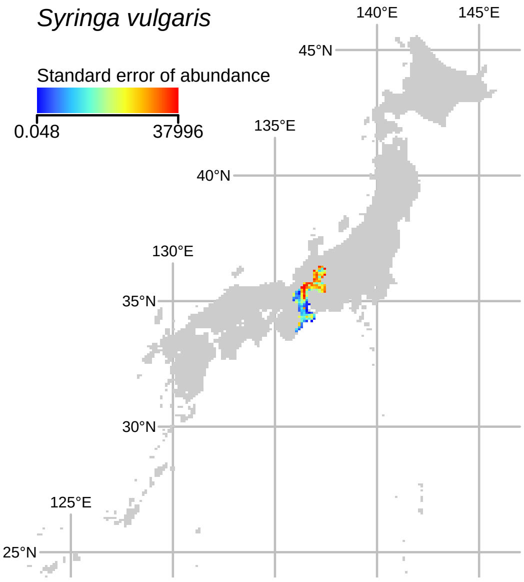
Syringa vulgaris
Standard error of abundance
0.048
37996
140°E
145°E
45°N
135°E
40°N
130°E
35°N
30°N
125°E
25°N

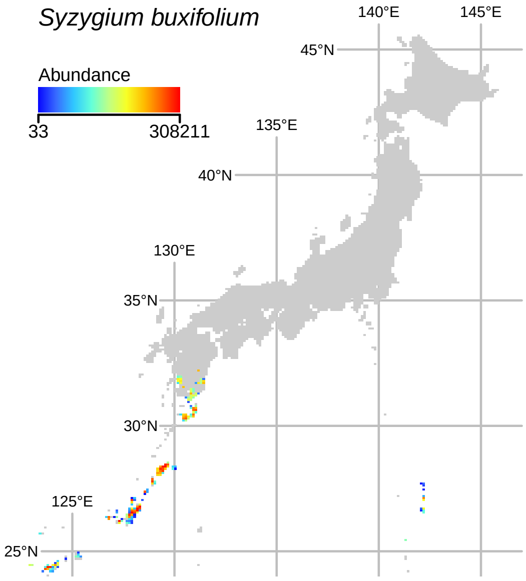
Syzygium buxifolium
Abundance
33
308211
140°E
145°E
45°N
135°E
40°N
130°E
35°N
30°N
125°E
25°N

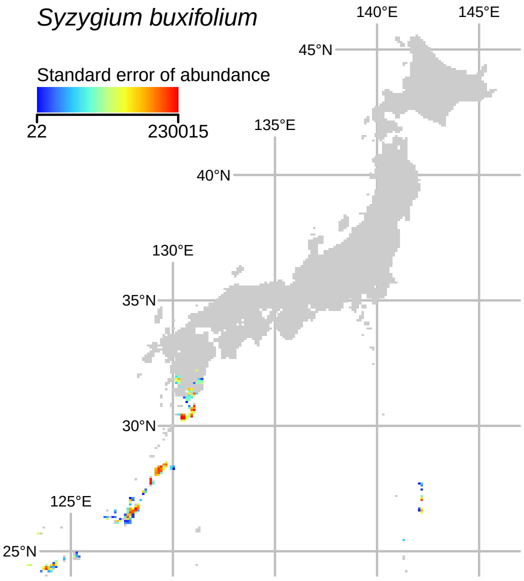
Syzygium buxifolium
Standard error of abundance
22
230015
140°E
145°E
45°N
135°E
40°N
130°E
35°N
30°N
125°E
25°N

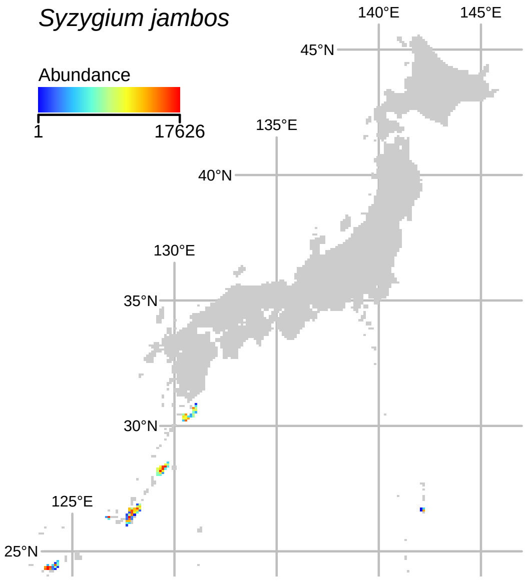
Syzygium jambos
Abundance
1
17626
140°E
145°E
45°N
135°E
40°N
130°E
35°N
30°N
125°E
25°N

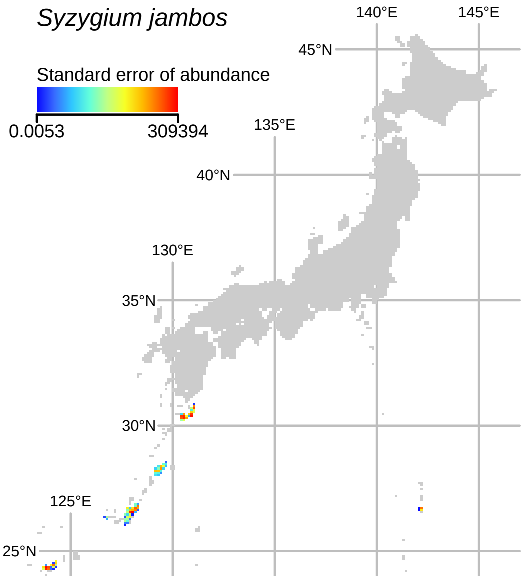
Syzygium jambos
Standard error of abundance
0.0053
309394
140°E
145°E
45°N
135°E
40°N
130°E
35°N
30°N
125°E
25°N

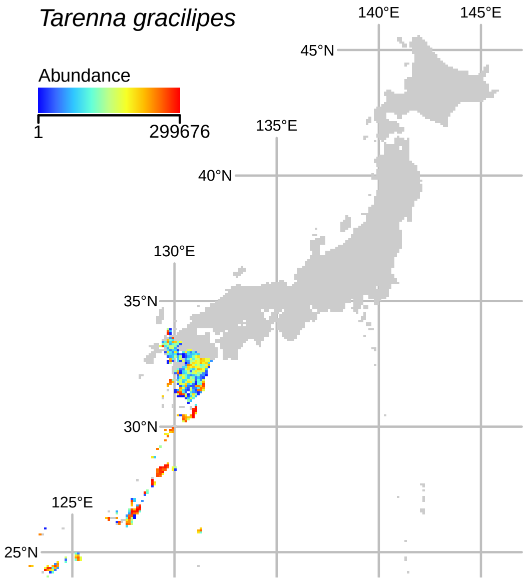
Tarenna gracilipes
Abundance
1
299676
140°E
145°E
45°N
135°E
40°N
130°E
35°N
30°N
125°E
25°N

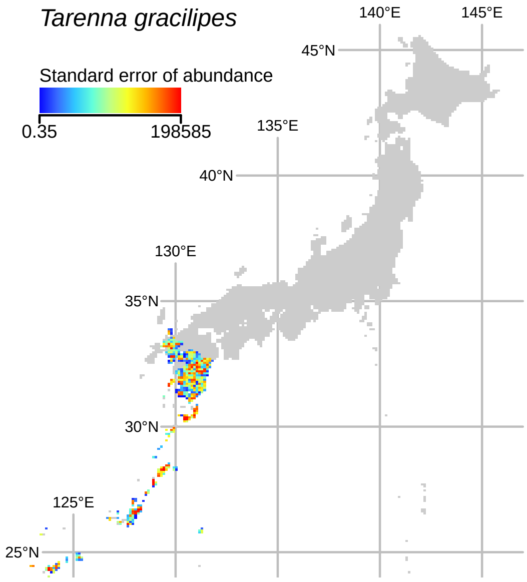
Tarenna gracilipes
Standard error of abundance
0.35
198585
140°E
145°E
45°N
135°E
40°N
130°E
35°N
30°N
125°E
25°N

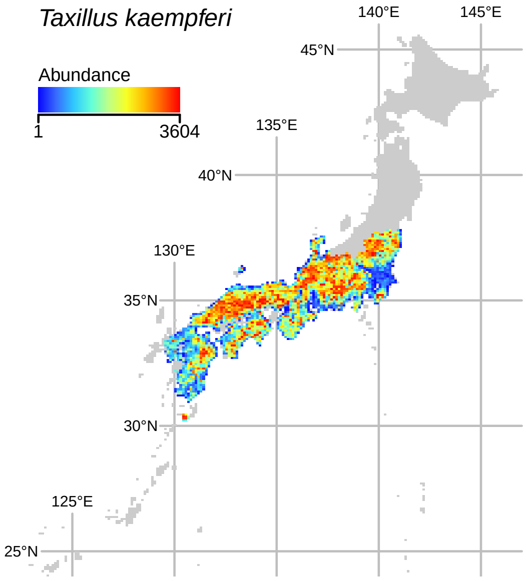
Taxillus kaempferi
Abundance
1
3604
140°E
145°E
45°N
135°E
40°N
130°E
35°N
30°N
125°E
25°N

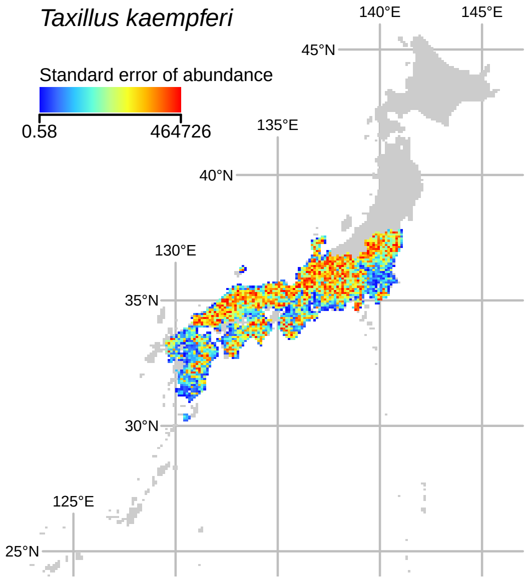
Taxillus kaempferi
Standard error of abundance
0.58
464726
140°E
145°E
45°N
135°E
40°N
130°E
35°N
30°N
125°E
25°N

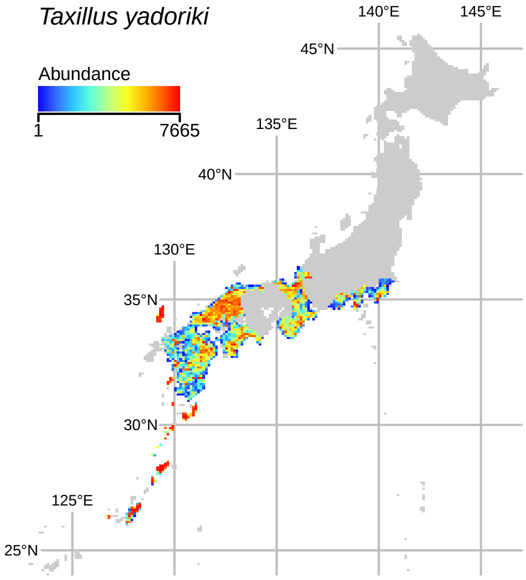
Taxillus yadoriki
Abundance
1
7665
140°E
145°E
45°N
135°E
40°N
130°E
35°N
30°N
125°E
25°N

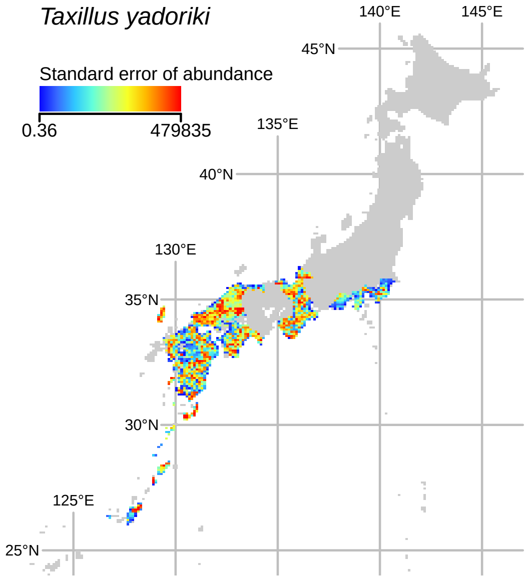
Taxillus yadoriki
Standard error of abundance
0.36
479835
140°E
145°E
45°N
135°E
40°N
130°E
35°N
30°N
125°E
25°N

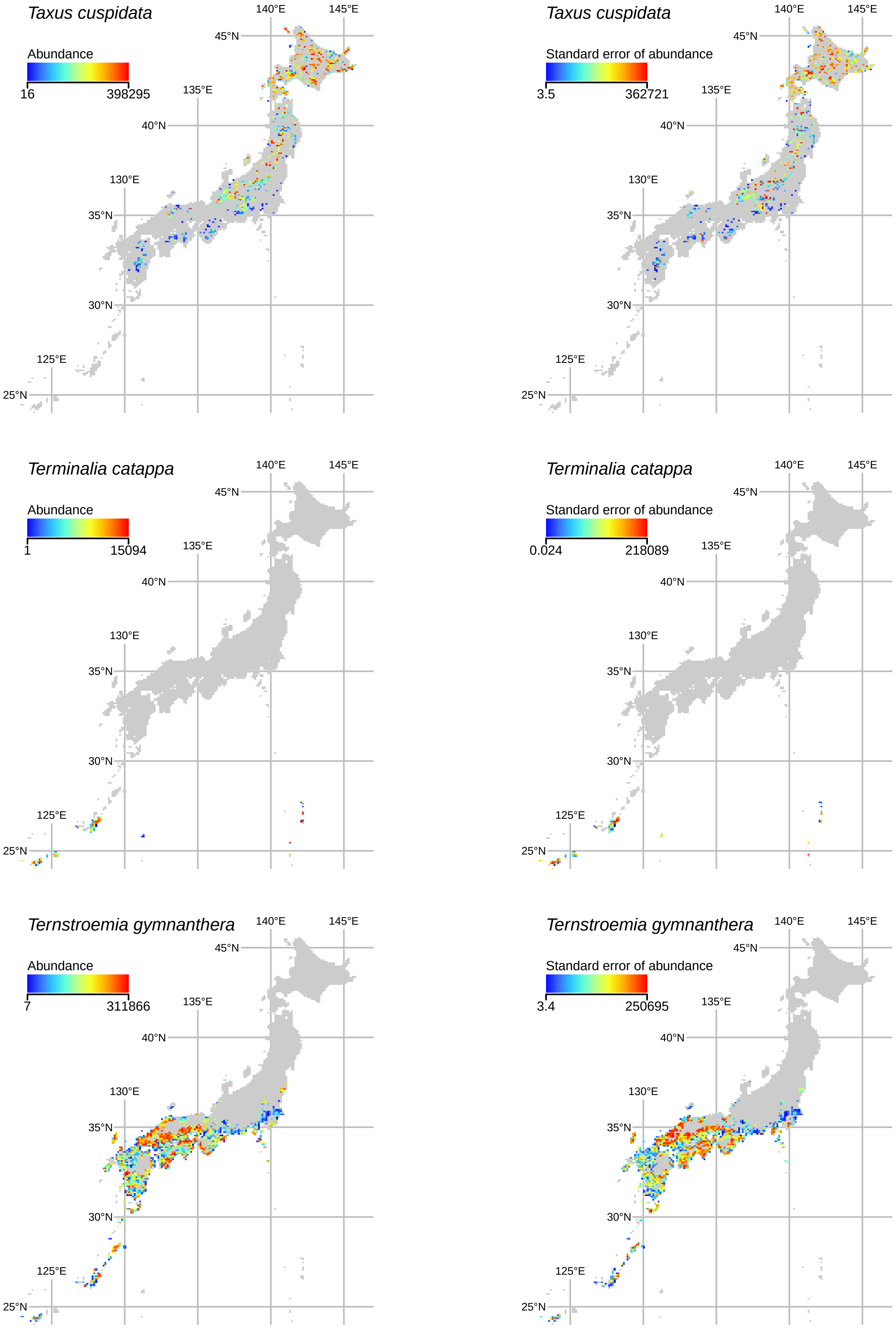

Taxus cuspidata
Abundance
16
398295
Taxus cuspidata
Standard error of abundance
3.5
362721
Terminalia catappa
Abundance
1
15094
Terminalia catappa
Standard error of abundance
0.024
218089
Ternstroemia gymnanthera
Abundance
7
311866
Ternstroemia gymnanthera
Standard error of abundance
3.4
250695
125°E
130°E
135°E
140°E
145°E
25°N
30°N
35°N
40°N
45°N

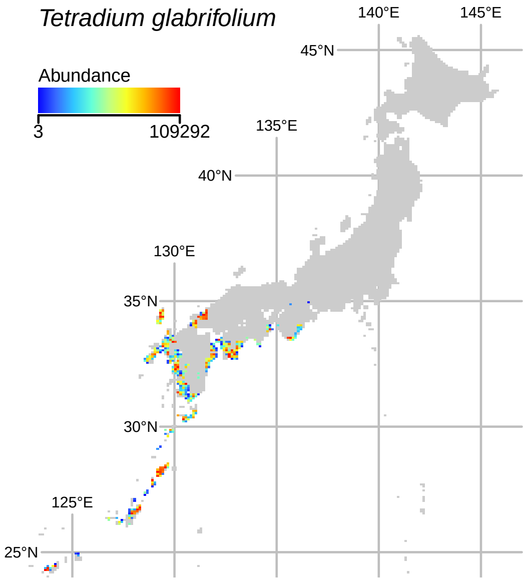
Tetradium glabrifolium
Abundance
3
109292
140°E
145°E
45°N
135°E
40°N
130°E
35°N
30°N
125°E
25°N

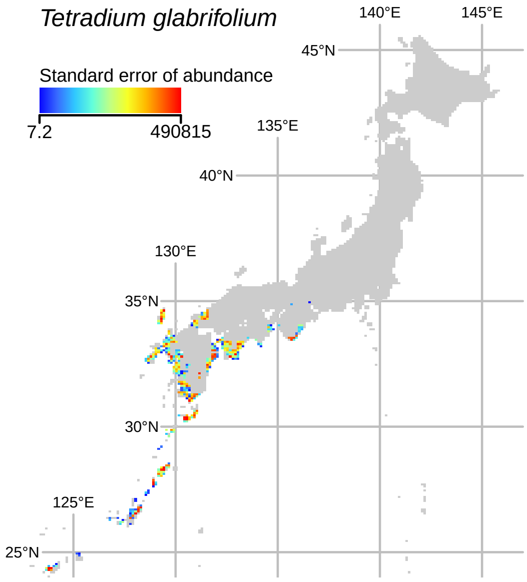
Tetradium glabrifolium
Standard error of abundance
7.2
490815
140°E
145°E
45°N
135°E
40°N
130°E
35°N
30°N
125°E
25°N

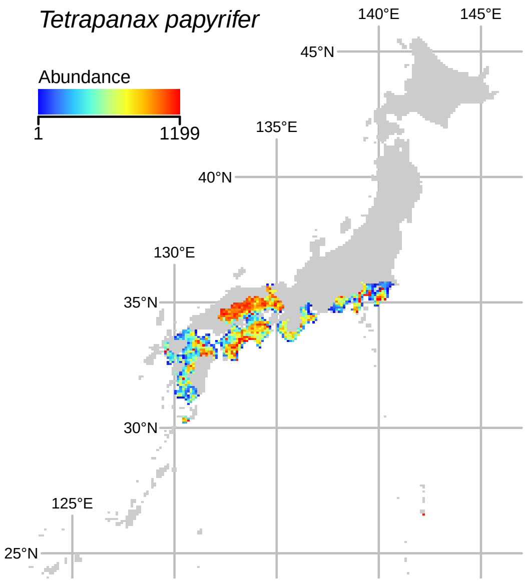
Tetrapanax papyrifer
Abundance
1
1199
140°E
145°E
45°N
135°E
40°N
130°E
35°N
30°N
125°E
25°N

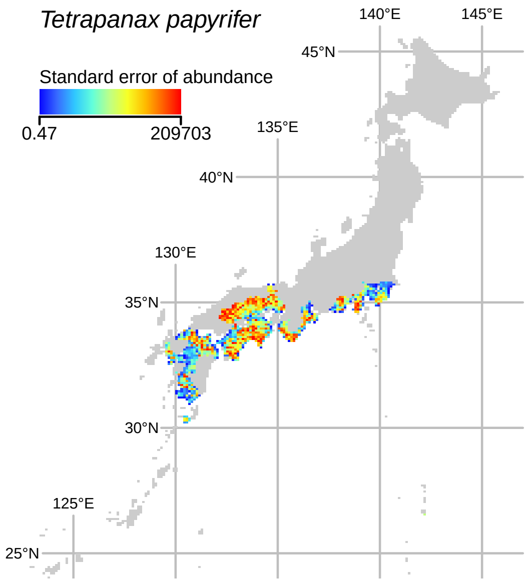
Tetrapanax papyrifer
Standard error of abundance
0.47
209703
140°E
145°E
45°N
135°E
40°N
130°E
35°N
30°N
125°E
25°N

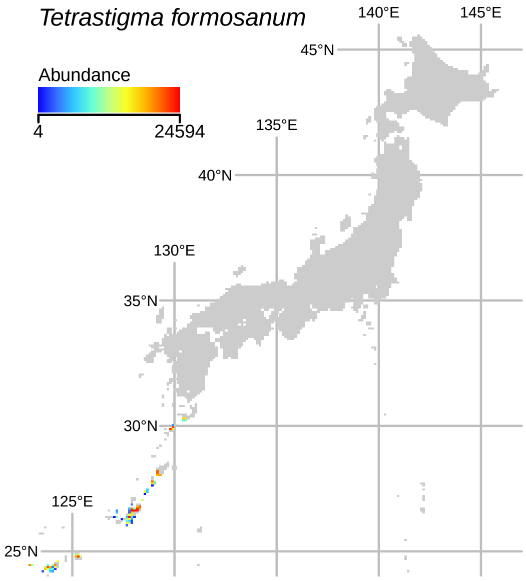
Tetrastigma formosanum
Abundance
4
24594
140°E
145°E
45°N
135°E
40°N
130°E
35°N
30°N
125°E
25°N

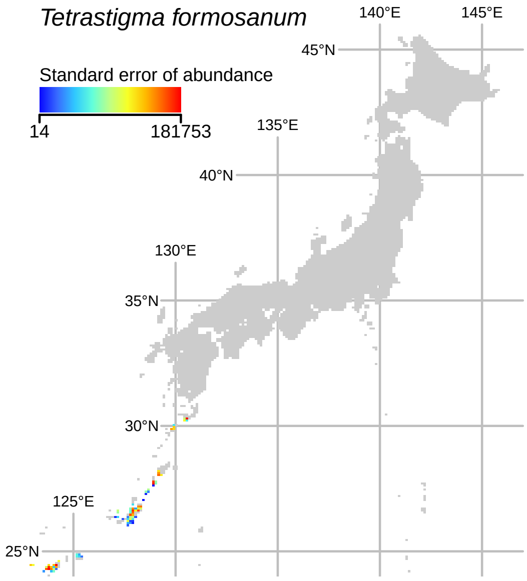
Tetrastigma formosanum
Standard error of abundance
14
181753
140°E
145°E
45°N
135°E
40°N
130°E
35°N
30°N
125°E
25°N

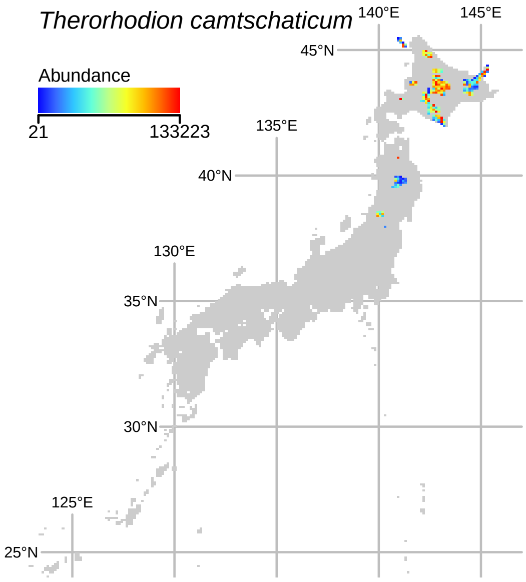
Therorhodion camtschaticum
Abundance
21
133223
140°E
145°E
135°E
130°E
125°E
45°N
40°N
35°N
30°N
25°N

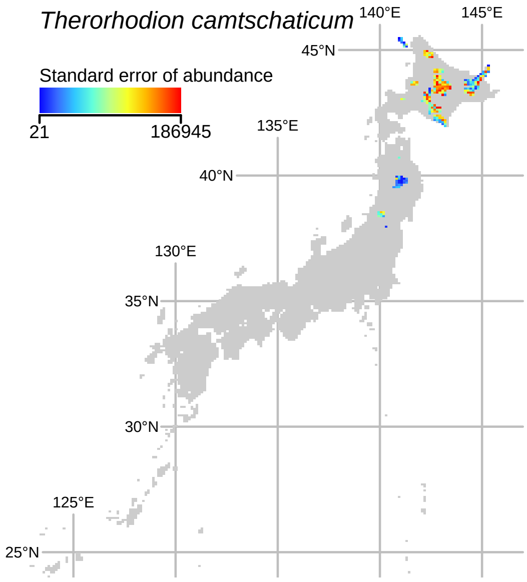
Therorhodion camtschaticum
Standard error of abundance
21
186945
140°E
145°E
135°E
130°E
125°E
45°N
40°N
35°N
30°N
25°N

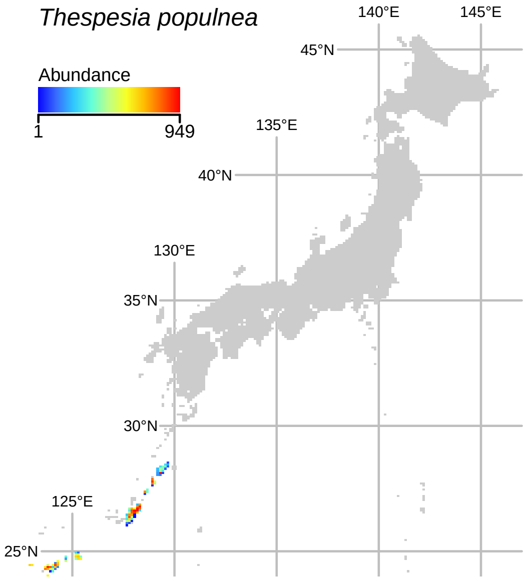
Thespesia populnea
Abundance
1
949
140°E
145°E
135°E
130°E
125°E
45°N
40°N
35°N
30°N
25°N

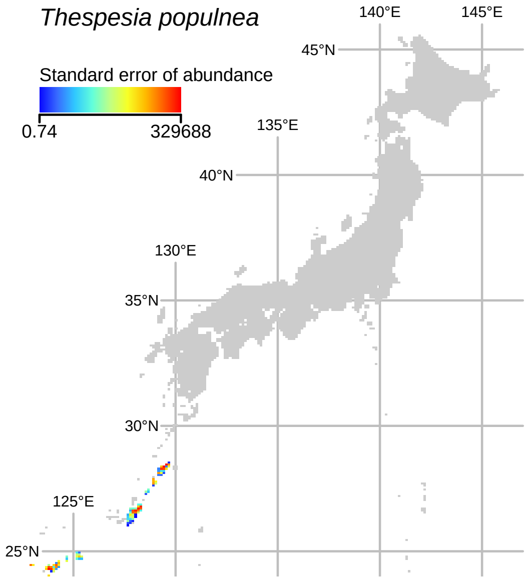
Thespesia populnea
Standard error of abundance
0.74
329688
140°E
145°E
135°E
130°E
125°E
45°N
40°N
35°N
30°N
25°N

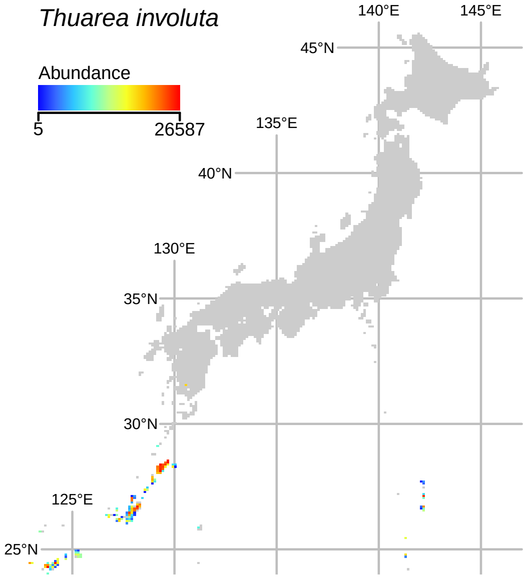
Thuarea involuta
Abundance
5
26587
140°E
145°E
135°E
130°E
125°E
45°N
40°N
35°N
30°N
25°N

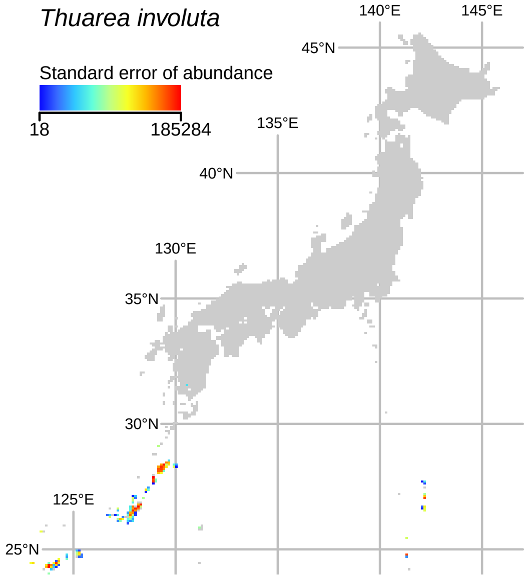
Thuarea involuta
Standard error of abundance
18
185284
140°E
145°E
135°E
130°E
125°E
45°N
40°N
35°N
30°N
25°N

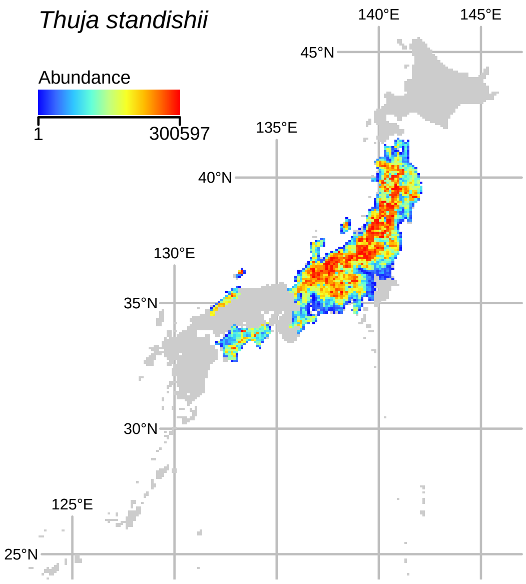
Thuja standishii
Abundance
1
300597
140°E
145°E
45°N
135°E
40°N
130°E
35°N
30°N
125°E
25°N

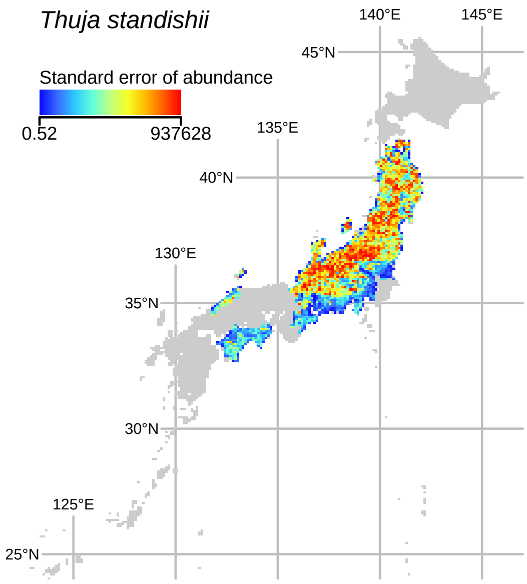
Thuja standishii
Standard error of abundance
0.52
937628
140°E
145°E
45°N
135°E
40°N
130°E
35°N
30°N
125°E
25°N

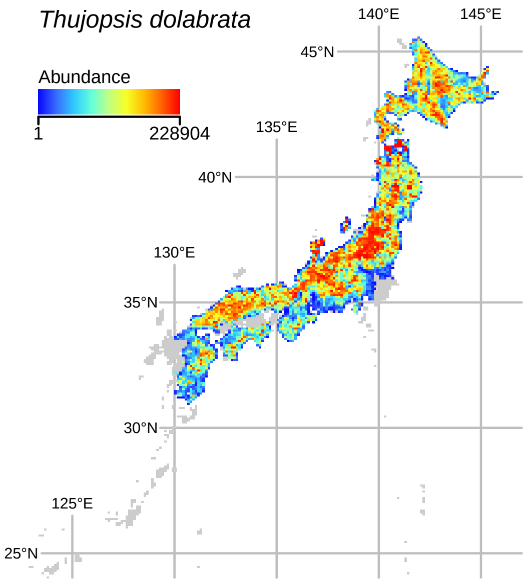
Thujopsis dolabrata
Abundance
1
228904
140°E
145°E
45°N
135°E
40°N
130°E
35°N
30°N
125°E
25°N

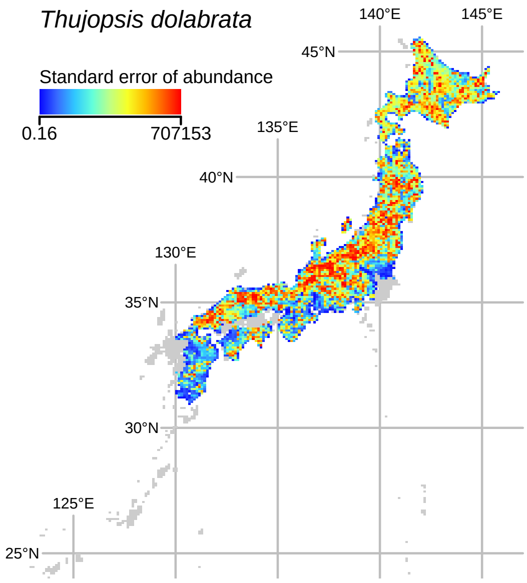
Thujopsis dolabrata
Standard error of abundance
0.16
707153
140°E
145°E
45°N
135°E
40°N
130°E
35°N
30°N
125°E
25°N

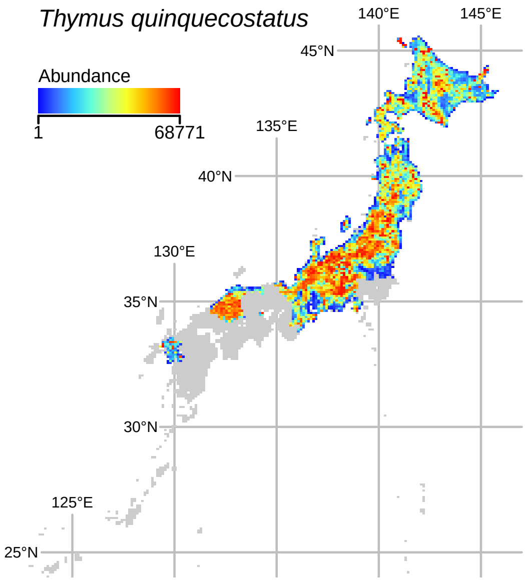
Thymus quinquecostatus
Abundance
1
68771
140°E
145°E
45°N
135°E
40°N
130°E
35°N
30°N
125°E
25°N

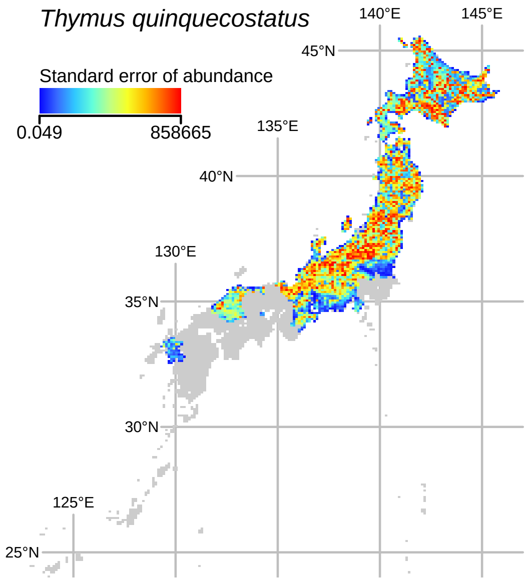
Thymus quinquecostatus
Standard error of abundance
0.049
858665
140°E
145°E
45°N
135°E
40°N
130°E
35°N
30°N
125°E
25°N

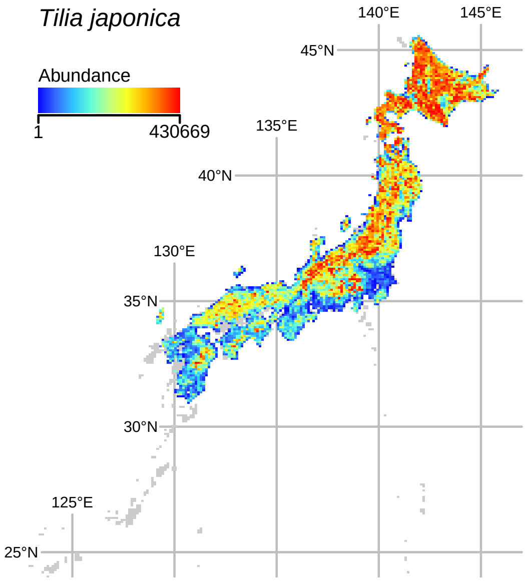
Tilia japonica
Abundance
1
430669
140°E
145°E
45°N
135°E
40°N
130°E
35°N
30°N
125°E
25°N

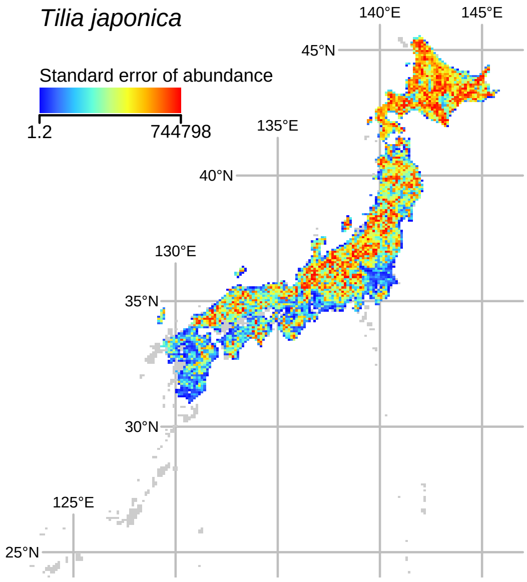
Tilia japonica
Standard error of abundance
1.2
744798
140°E
145°E
45°N
135°E
40°N
130°E
35°N
30°N
125°E
25°N

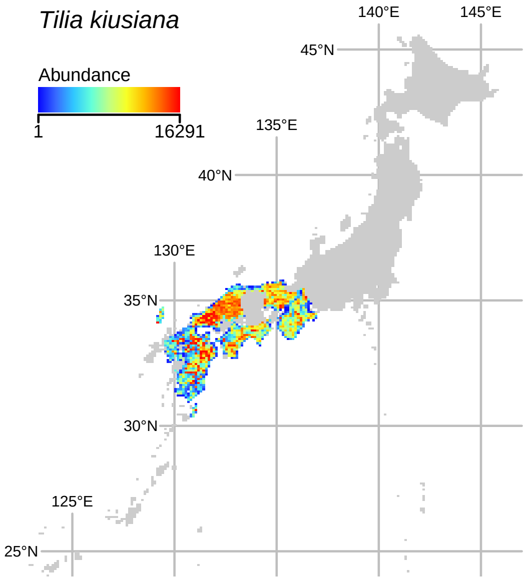
Tilia kiusiana
Abundance
1
16291
140°E
145°E
45°N
135°E
40°N
130°E
35°N
30°N
125°E
25°N

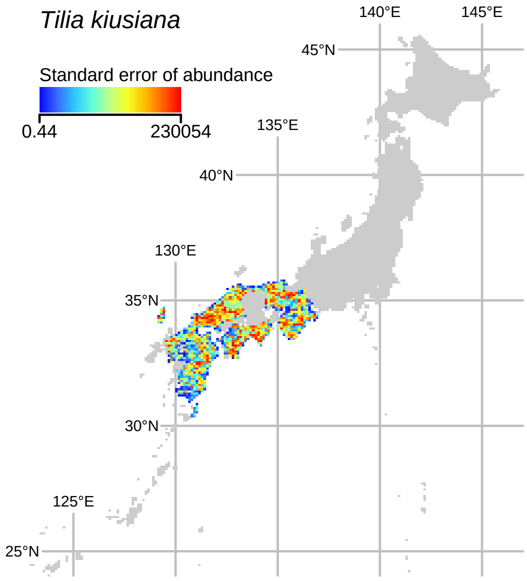
Tilia kiusiana
Standard error of abundance
0.44
230054
140°E
145°E
45°N
135°E
40°N
130°E
35°N
30°N
125°E
25°N

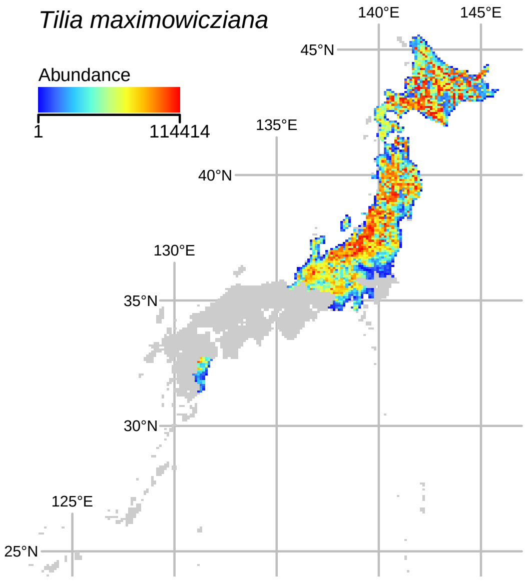
Tilia maximowicziana
Abundance
1
114414
140°E
145°E
45°N
135°E
40°N
130°E
35°N
30°N
125°E
25°N

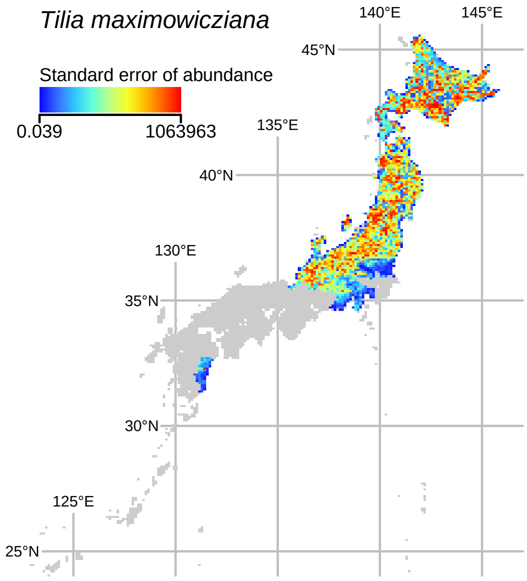
Tilia maximowicziana
Standard error of abundance
0.039
1063963
140°E
145°E
45°N
135°E
40°N
130°E
35°N
30°N
125°E
25°N

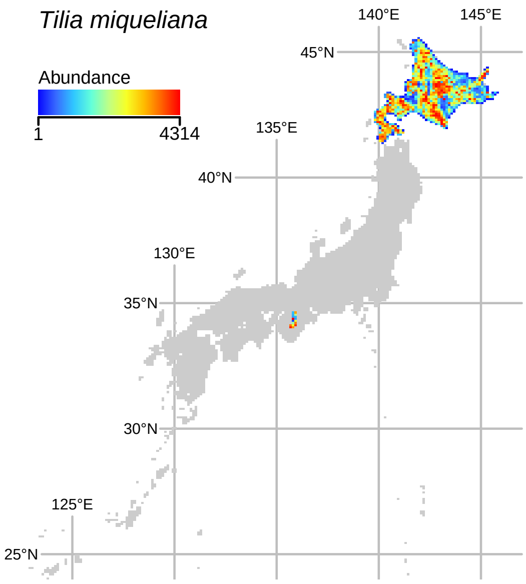
Tilia miqueliana
Abundance
1
4314
140°E
145°E
45°N
135°E
40°N
130°E
35°N
30°N
125°E
25°N

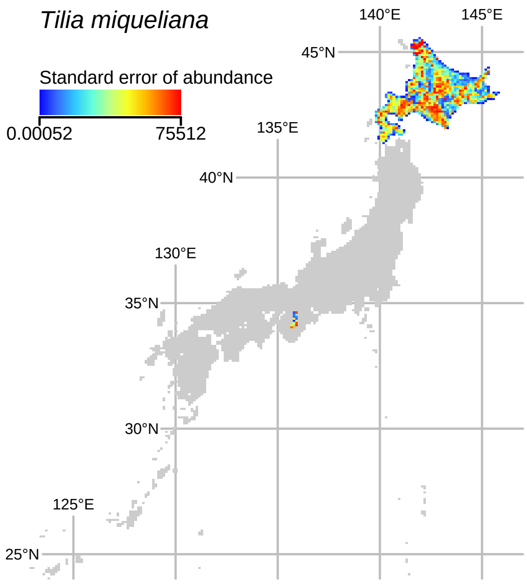
Tilia miqueliana
Standard error of abundance
0.00052
75512
140°E
145°E
45°N
135°E
40°N
130°E
35°N
30°N
125°E
25°N

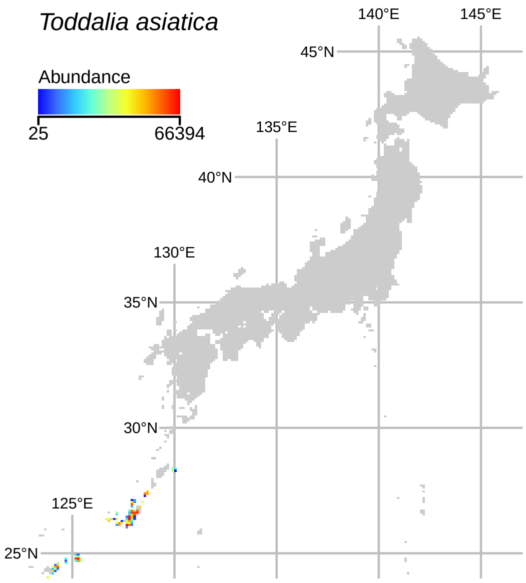
Toddalia asiatica
Abundance
25
66394
140°E
145°E
45°N
135°E
40°N
130°E
35°N
30°N
125°E
25°N

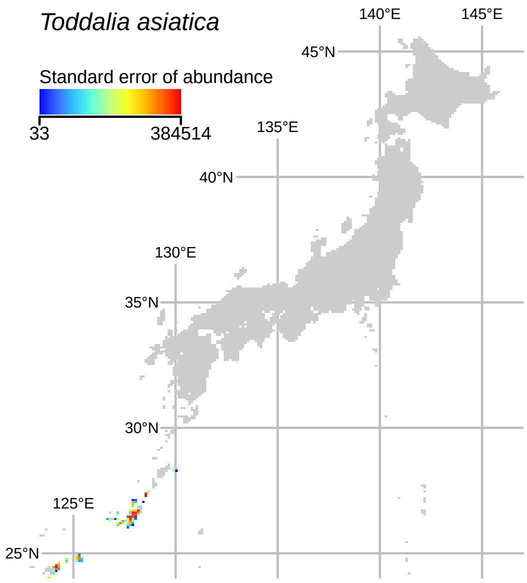
Toddalia asiatica
Standard error of abundance
33
384514
140°E
145°E
45°N
135°E
40°N
130°E
35°N
30°N
125°E
25°N

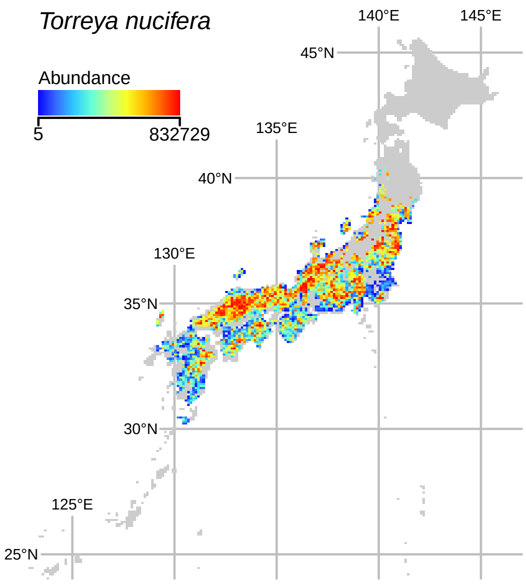
Torreya nucifera
Abundance
5
832729
140°E
145°E
45°N
135°E
40°N
130°E
35°N
30°N
125°E
25°N

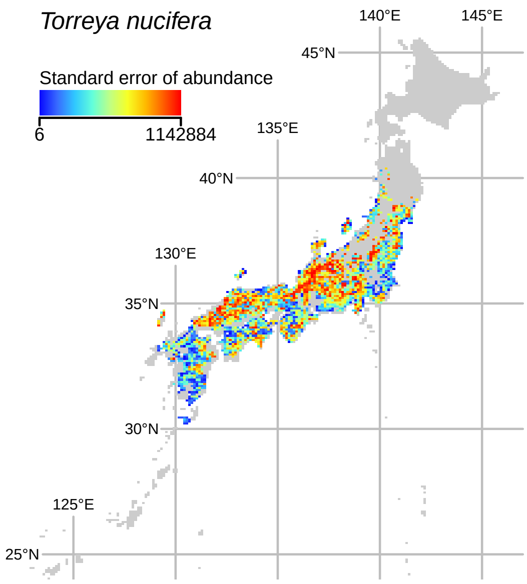
Torreya nucifera
Standard error of abundance
6
1142884
140°E
145°E
45°N
135°E
40°N
130°E
35°N
30°N
125°E
25°N

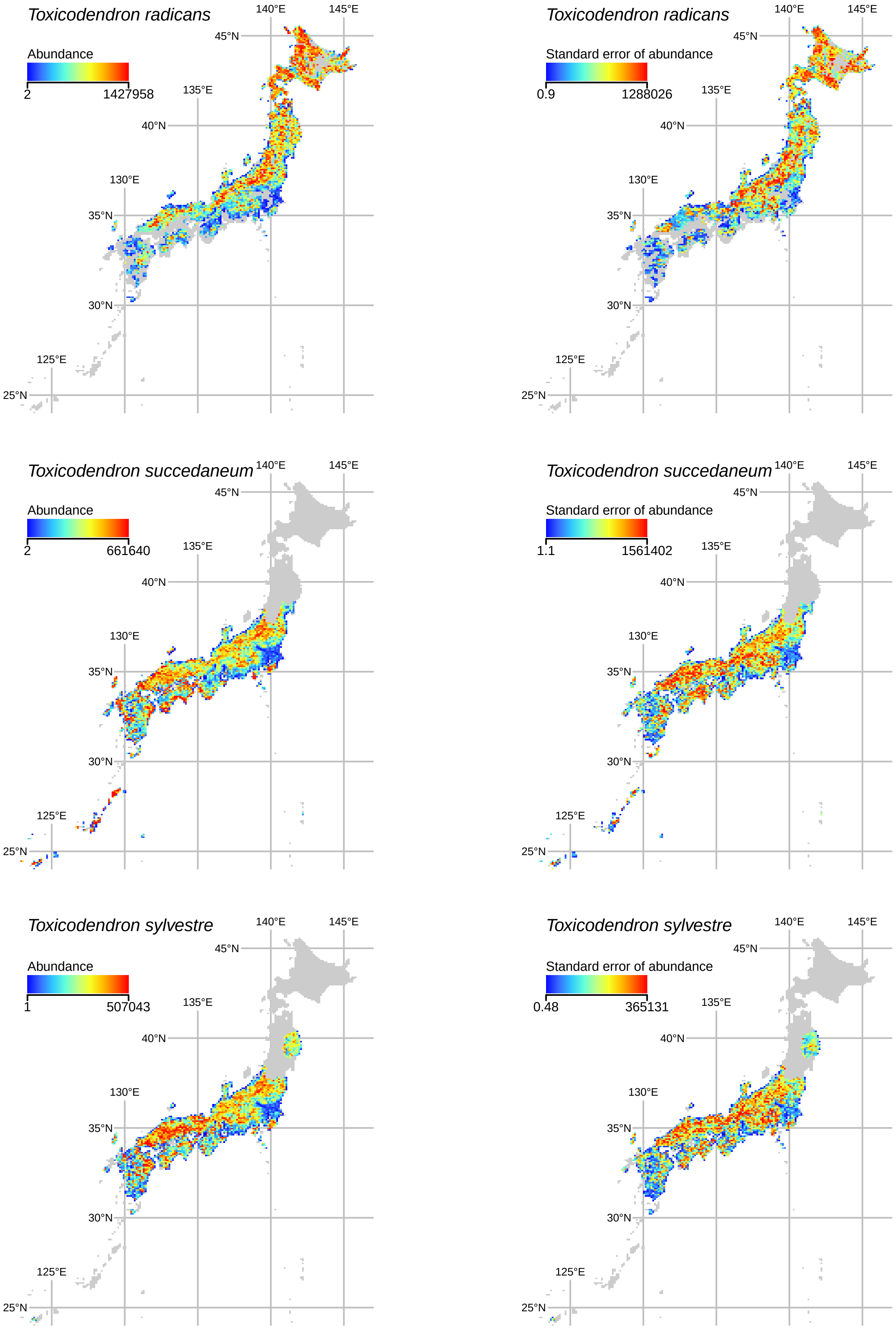
Toxicodendron radicans
Abundance
2
1427958
Toxicodendron radicans
Standard error of abundance
0.9
1288026
Toxicodendron succedaneum
Abundance
2
661640
Toxicodendron succedaneum
Standard error of abundance
1.1
1561402
Toxicodendron sylvestre
Abundance
1
507043
Toxicodendron sylvestre
Standard error of abundance
0.48
365131
125°E
130°E
135°E
140°E
145°E
25°N
30°N
35°N
40°N
45°N

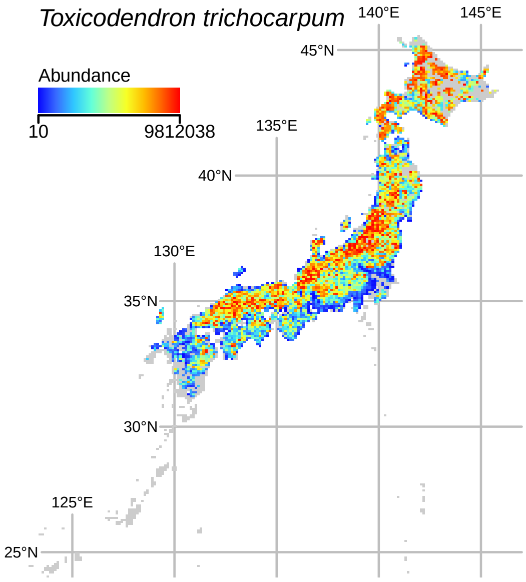
Toxicodendron trichocarpum
Abundance
10
9812038
140°E
145°E
45°N
135°E
40°N
130°E
35°N
30°N
125°E
25°N

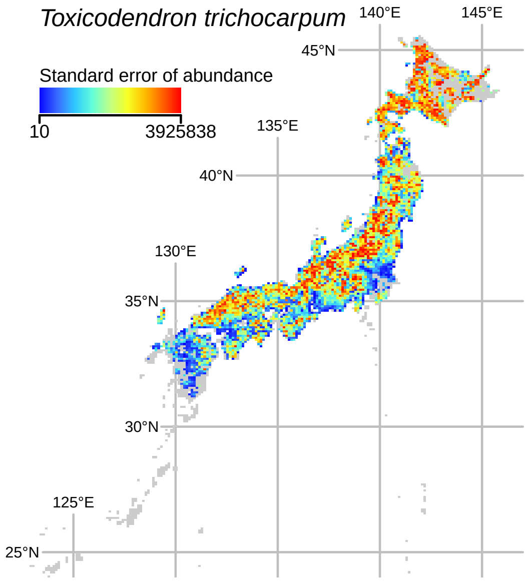
Toxicodendron trichocarpum
Standard error of abundance
10
3925838
140°E
145°E
45°N
135°E
40°N
130°E
35°N
30°N
125°E
25°N

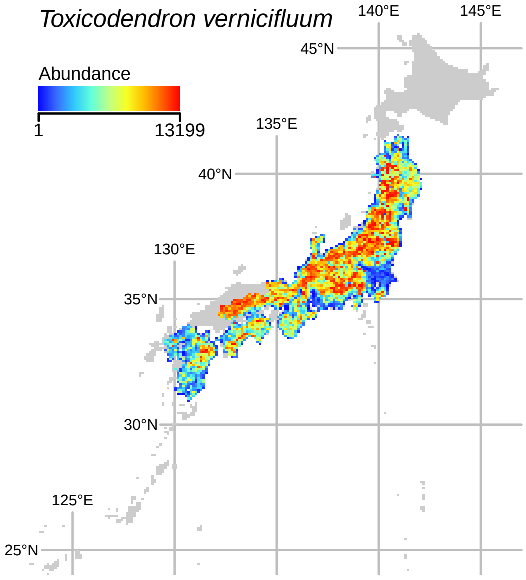
Toxicodendron vernicifluum
Abundance
1
13199
140°E
145°E
45°N
135°E
40°N
130°E
35°N
30°N
125°E
25°N

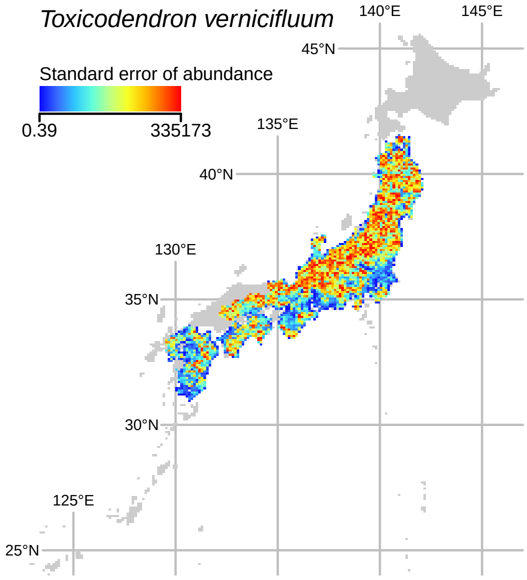
Toxicodendron vernicifluum
Standard error of abundance
0.39
335173
140°E
145°E
45°N
135°E
40°N
130°E
35°N
30°N
125°E
25°N

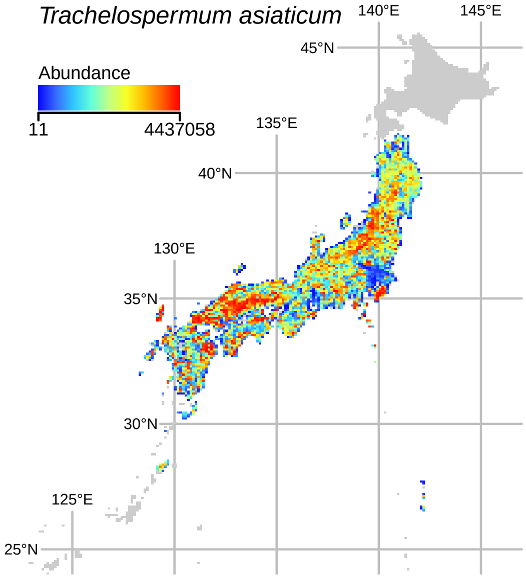
Trachelospermum asiaticum
Abundance
11
4437058
140°E
145°E
45°N
135°E
40°N
130°E
35°N
30°N
125°E
25°N

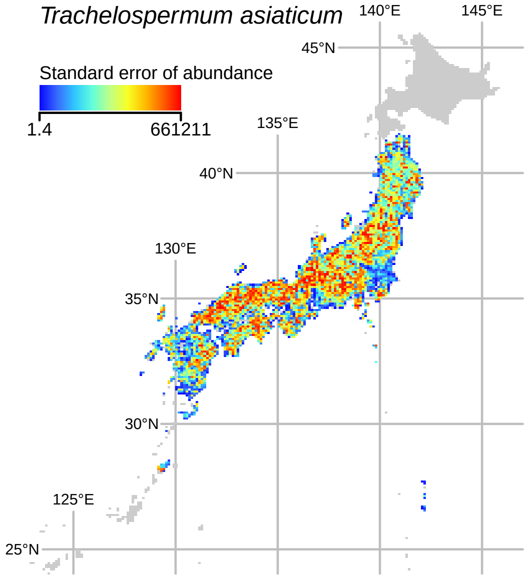
Trachelospermum asiaticum
Standard error of abundance
1.4
661211
140°E
145°E
45°N
135°E
40°N
130°E
35°N
30°N
125°E
25°N

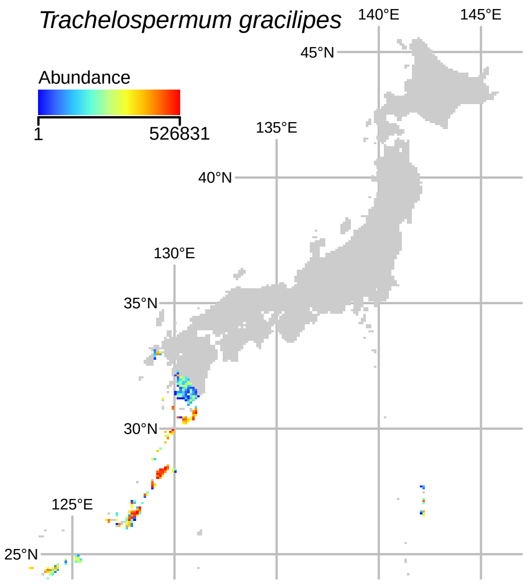
Trachelospermum gracilipes
Abundance
1
526831
140°E
145°E
45°N
135°E
40°N
130°E
35°N
30°N
125°E
25°N

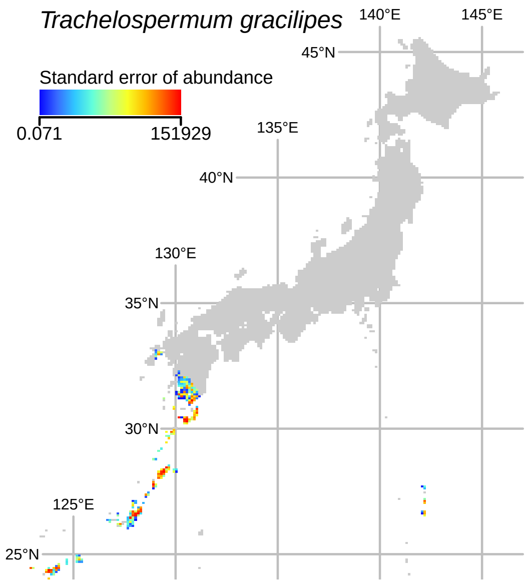
Trachelospermum gracilipes
Standard error of abundance
0.071
151929
140°E
145°E
45°N
135°E
40°N
130°E
35°N
30°N
125°E
25°N

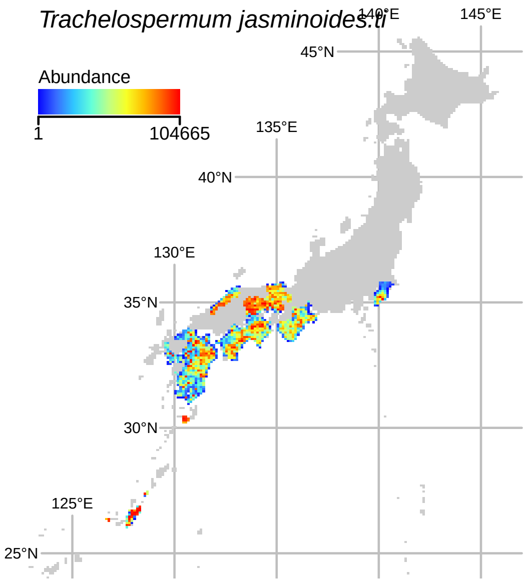
Trachelospermum jasminoides.ti
Abundance
1
104665
140°E
145°E
45°N
135°E
40°N
130°E
35°N
30°N
125°E
25°N

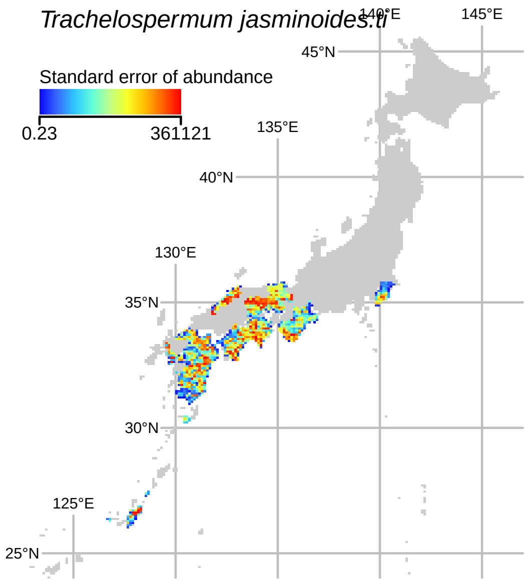
Trachelospermum jasminoides.ti
Standard error of abundance
0.23
361121
140°E
145°E
45°N
135°E
40°N
130°E
35°N
30°N
125°E
25°N

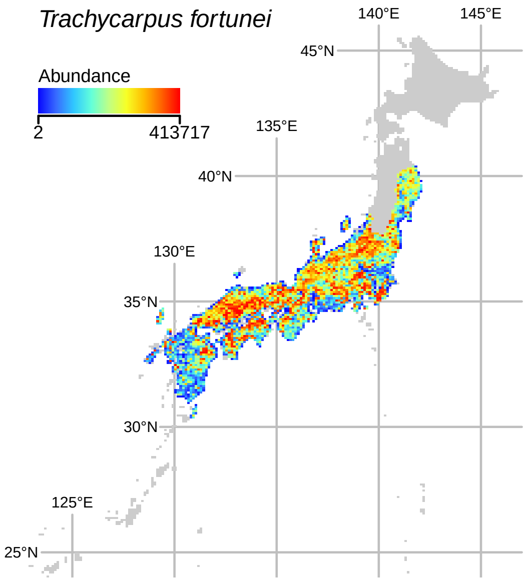
Trachycarpus fortunei
Abundance
2
413717
140°E
145°E
45°N
135°E
40°N
130°E
35°N
30°N
125°E
25°N

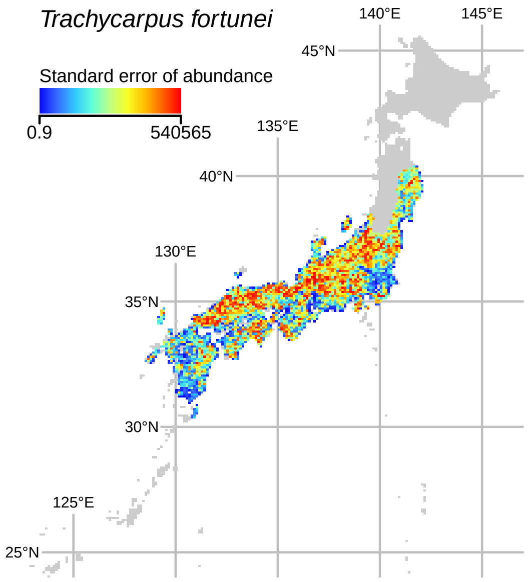
Trachycarpus fortunei
Standard error of abundance
0.9
540565
140°E
145°E
45°N
135°E
40°N
130°E
35°N
30°N
125°E
25°N

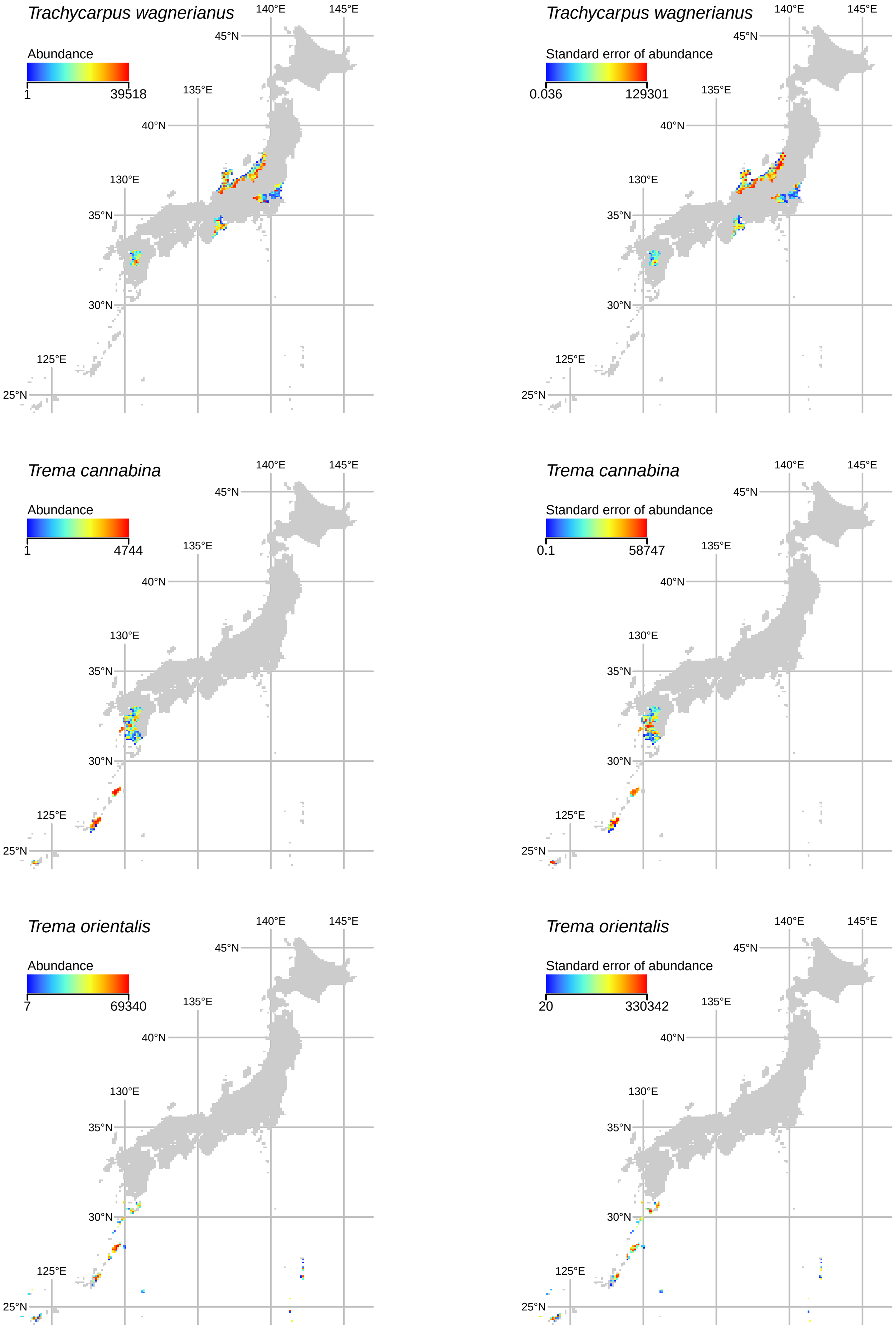
Trachycarpus wagnerianus
Abundance
1
39518
Trachycarpus wagnerianus
Standard error of abundance
0.036
129301
Trema cannabina
Abundance
1
4744
Trema cannabina
Standard error of abundance
0.1
58747
Trema orientalis
Abundance
7
69340
Trema orientalis
Standard error of abundance
20
330342
125°E
130°E
135°E
140°E
145°E
25°N
30°N
35°N
40°N
45°N

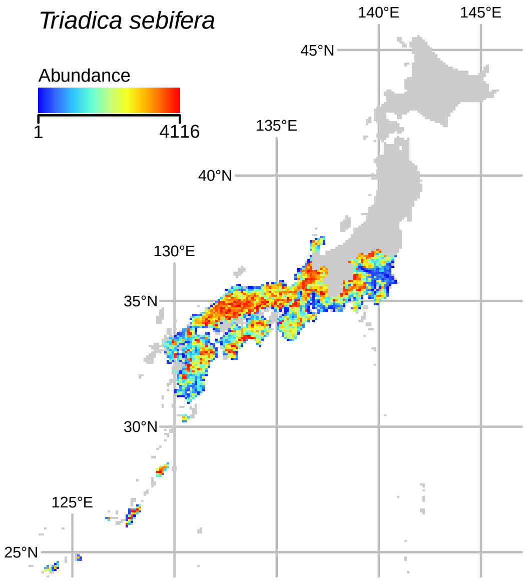
Triadica sebifera
Abundance
1
4116
140°E
145°E
45°N
135°E
40°N
130°E
35°N
30°N
125°E
25°N

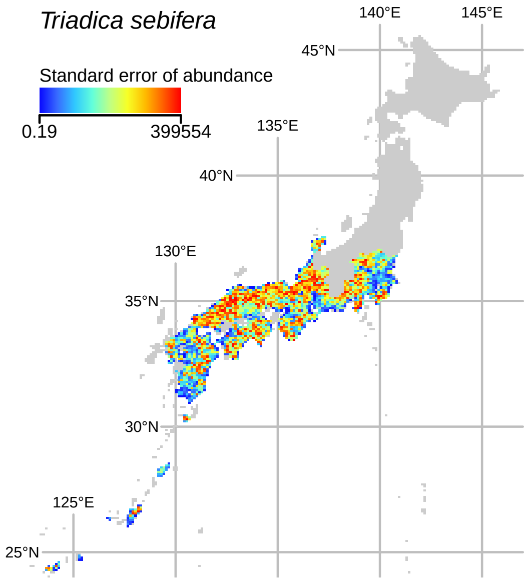
Triadica sebifera
Standard error of abundance
0.19
399554
140°E
145°E
45°N
135°E
40°N
130°E
35°N
30°N
125°E
25°N

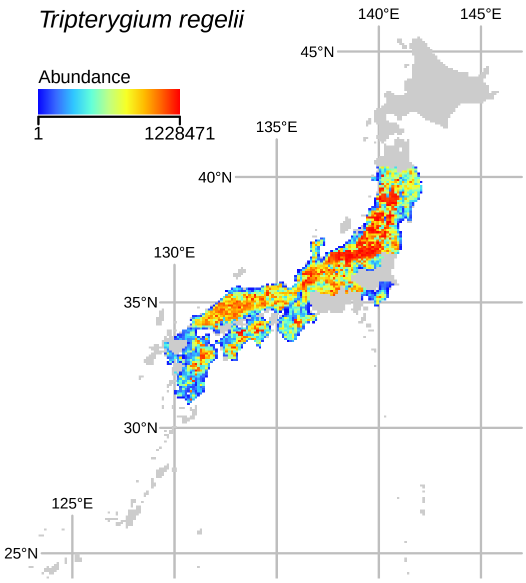
Tripterygium regelii
Abundance
1
1228471
140°E
145°E
45°N
135°E
40°N
130°E
35°N
30°N
125°E
25°N

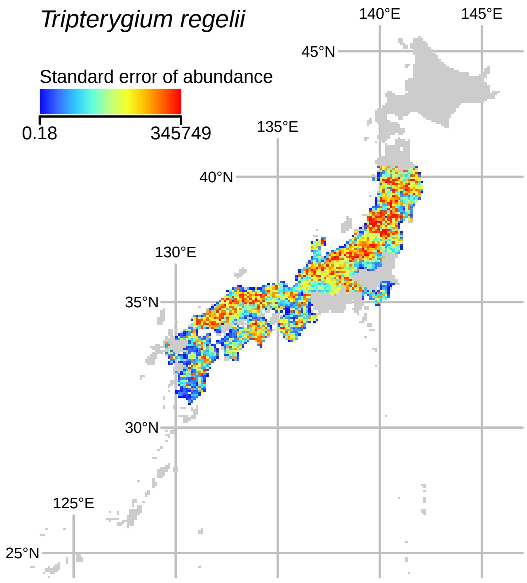
Tripterygium regelii
Standard error of abundance
0.18
345749
140°E
145°E
45°N
135°E
40°N
130°E
35°N
30°N
125°E
25°N

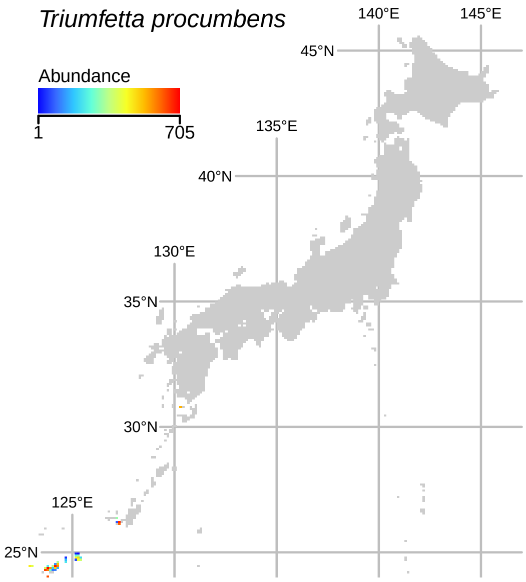
Triumfetta procumbens
Abundance
1
705
140°E
145°E
45°N
135°E
40°N
130°E
35°N
30°N
125°E
25°N

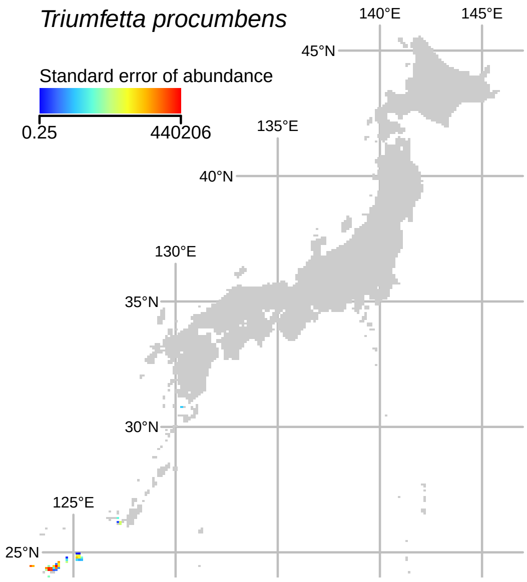
Triumfetta procumbens
Standard error of abundance
0.25
440206
140°E
145°E
45°N
135°E
40°N
130°E
35°N
30°N
125°E
25°N

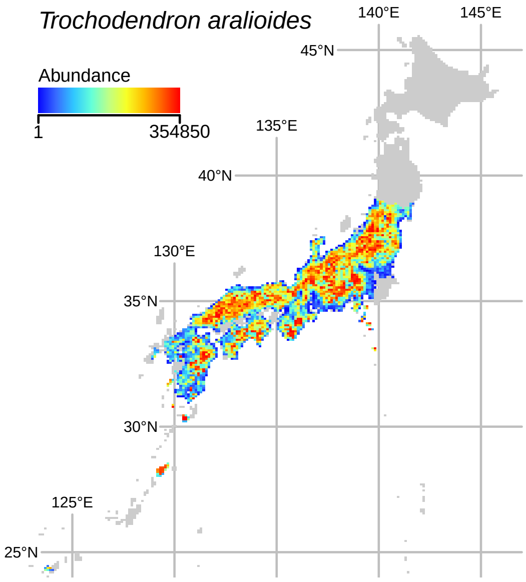
Trochodendron aralioides
Abundance
1
354850
140°E
145°E
45°N
135°E
40°N
130°E
35°N
30°N
125°E
25°N

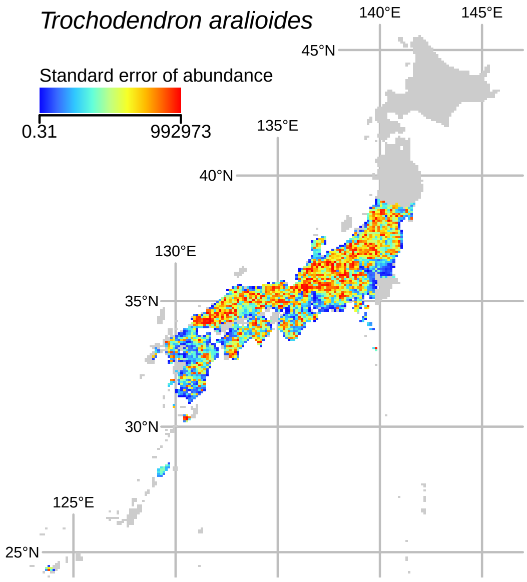
Trochodendron aralioides
Standard error of abundance
0.31
992973
140°E
145°E
45°N
135°E
40°N
130°E
35°N
30°N
125°E
25°N

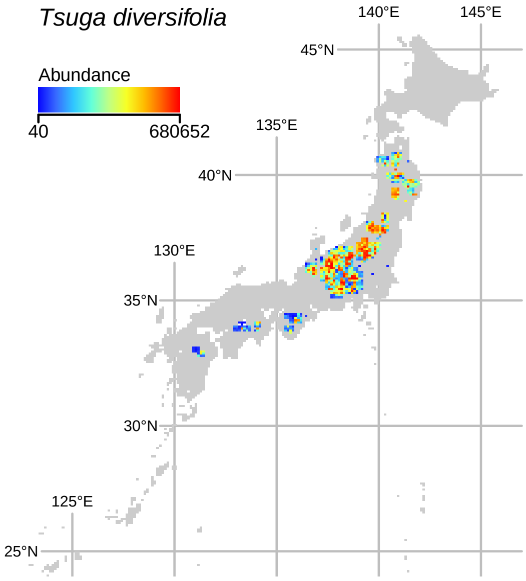
Tsuga diversifolia
Abundance
40
680652
140°E
145°E
45°N
135°E
40°N
130°E
35°N
30°N
125°E
25°N

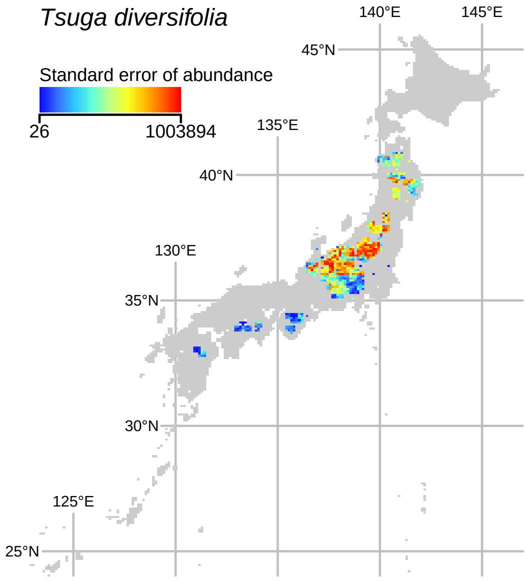
Tsuga diversifolia
Standard error of abundance
26
1003894
140°E
145°E
45°N
135°E
40°N
130°E
35°N
30°N
125°E
25°N

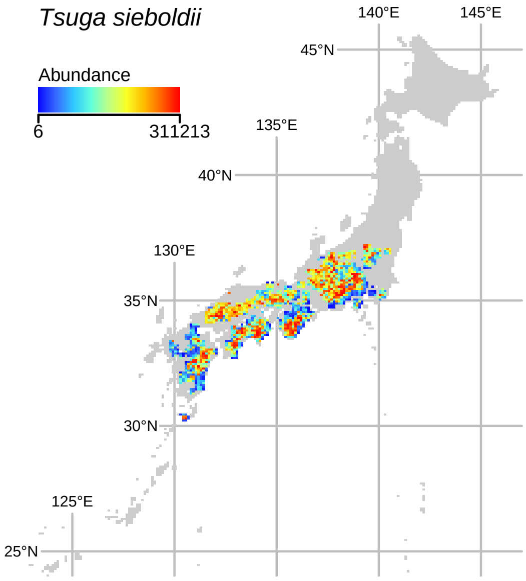
Tsuga sieboldii
Abundance
6
311213
140°E
145°E
45°N
135°E
40°N
130°E
35°N
30°N
125°E
25°N

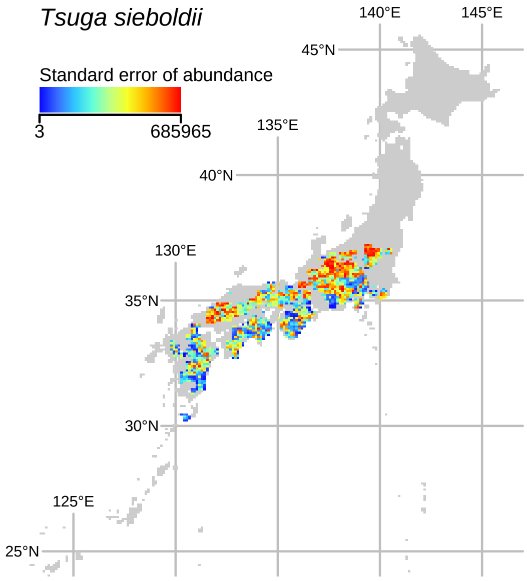
Tsuga sieboldii
Standard error of abundance
3
685965
140°E
145°E
45°N
135°E
40°N
130°E
35°N
30°N
125°E
25°N

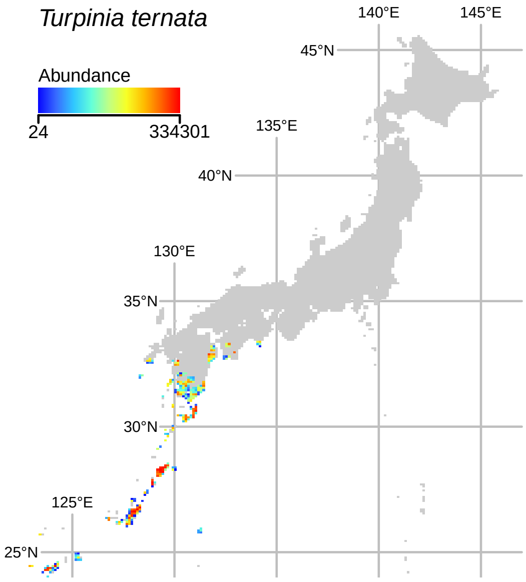
Turpinia ternata
Abundance
24
334301
140°E
145°E
45°N
135°E
40°N
130°E
35°N
30°N
125°E
25°N

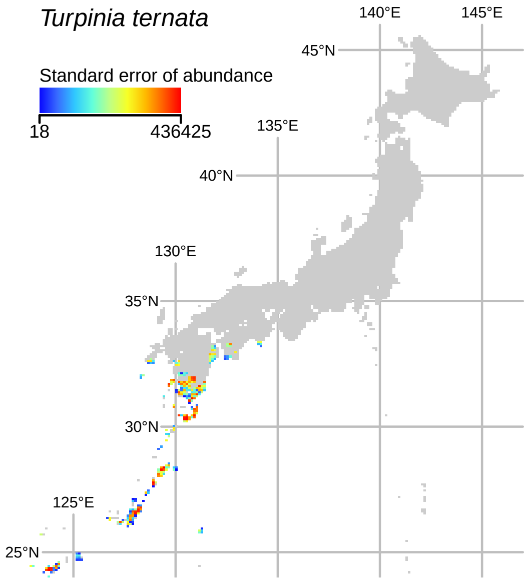
Turpinia ternata
Standard error of abundance
18
436425
140°E
145°E
45°N
135°E
40°N
130°E
35°N
30°N
125°E
25°N

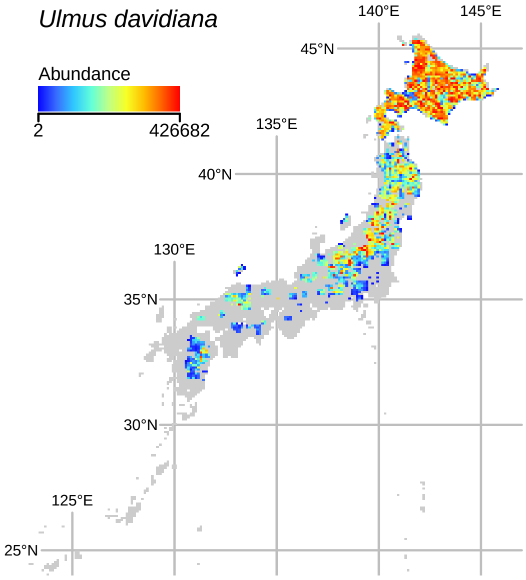
Ulmus davidiana
Abundance
2
426682
140°E
145°E
45°N
135°E
40°N
130°E
35°N
30°N
125°E
25°N

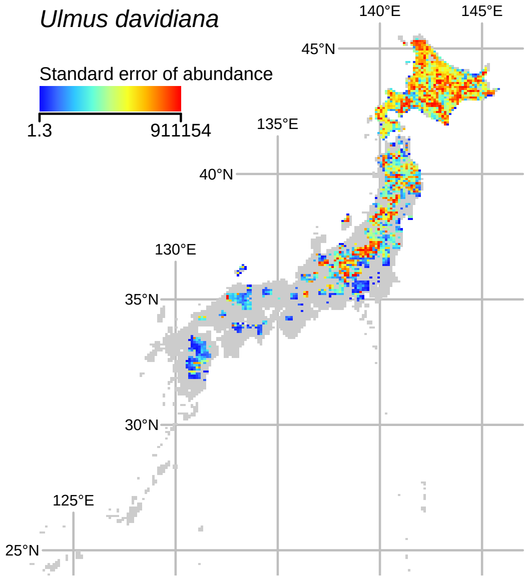
Ulmus davidiana
Standard error of abundance
1.3
911154
140°E
145°E
45°N
135°E
40°N
130°E
35°N
30°N
125°E
25°N

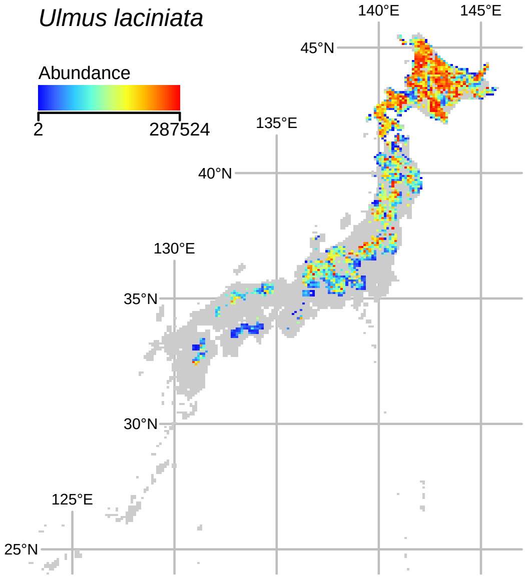
Ulmus laciniata
Abundance
2
287524
140°E
145°E
45°N
135°E
40°N
130°E
35°N
30°N
125°E
25°N

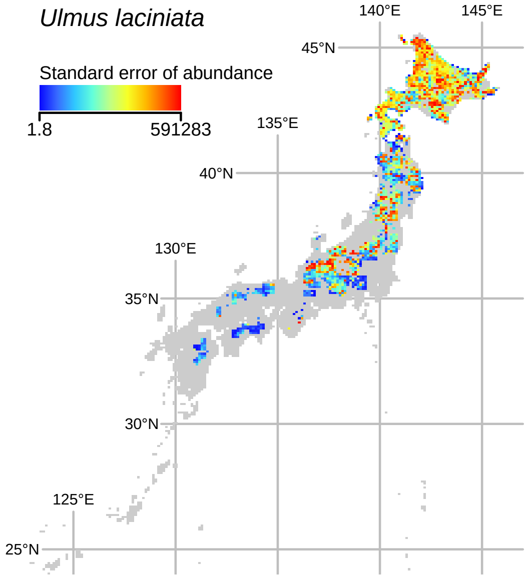
Ulmus laciniata
Standard error of abundance
1.8
591283
140°E
145°E
45°N
135°E
40°N
130°E
35°N
30°N
125°E
25°N

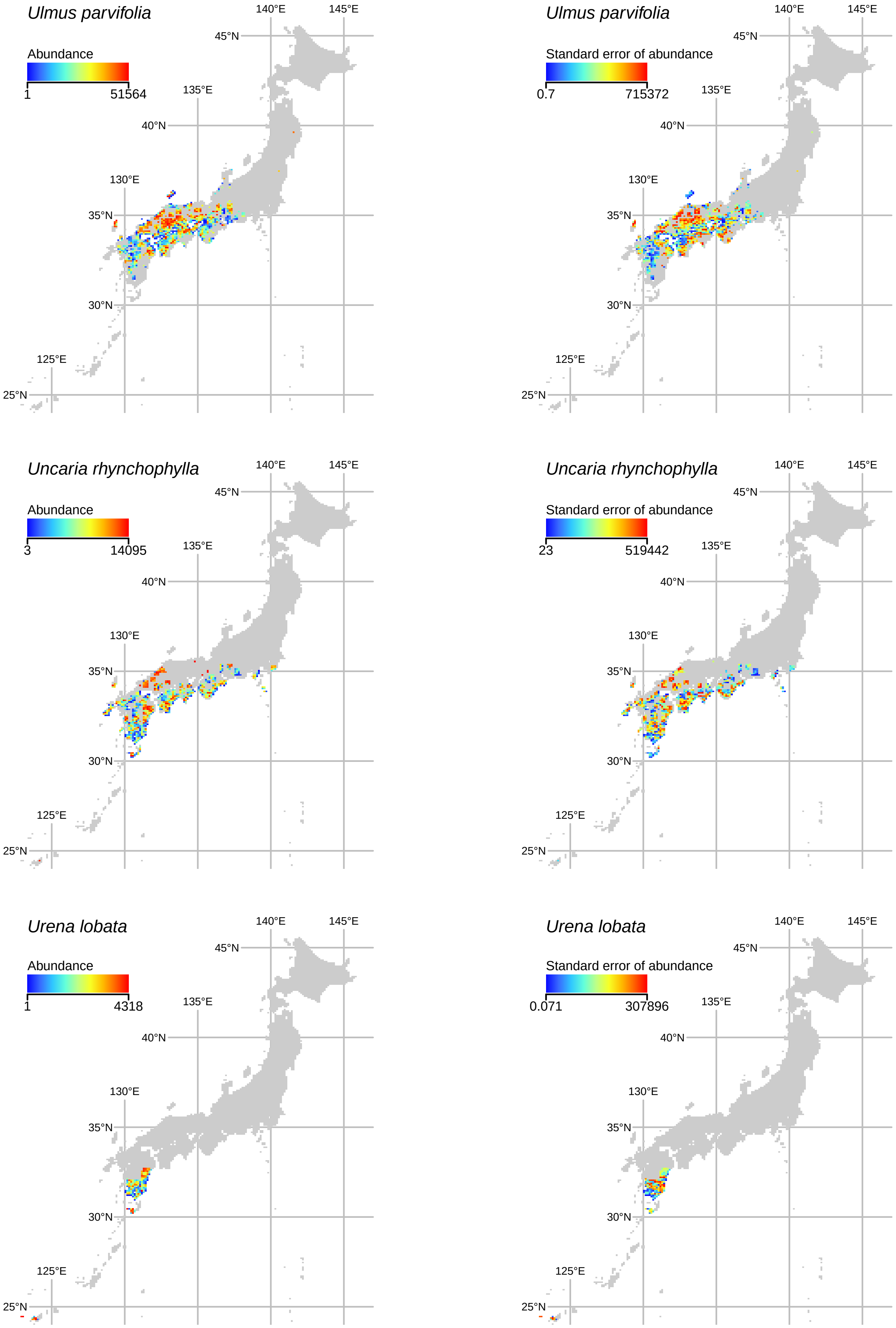
Ulmus parvifolia
Abundance
1
51564
Ulmus parvifolia
Standard error of abundance
0.7
715372
Uncaria rhynchophylla
Abundance
3
14095
Uncaria rhynchophylla
Standard error of abundance
23
519442
Urena lobata
Abundance
1
4318
Urena lobata
Standard error of abundance
0.071
307896
125°E
130°E
135°E
140°E
145°E
25°N
30°N
35°N
40°N
45°N

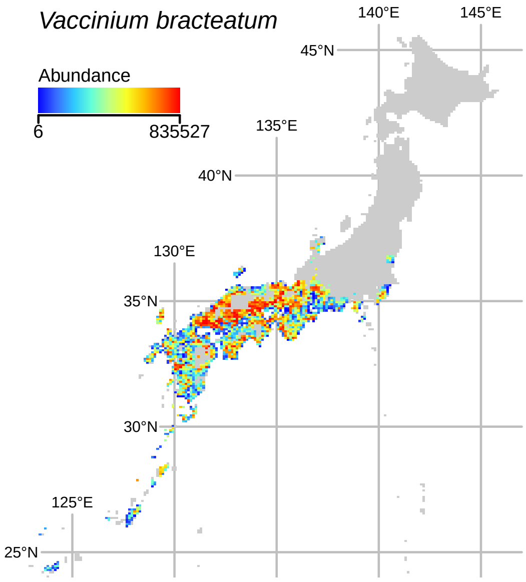
Vaccinium bracteatum
Abundance
6
835527
140°E
145°E
45°N
135°E
40°N
130°E
35°N
30°N
125°E
25°N

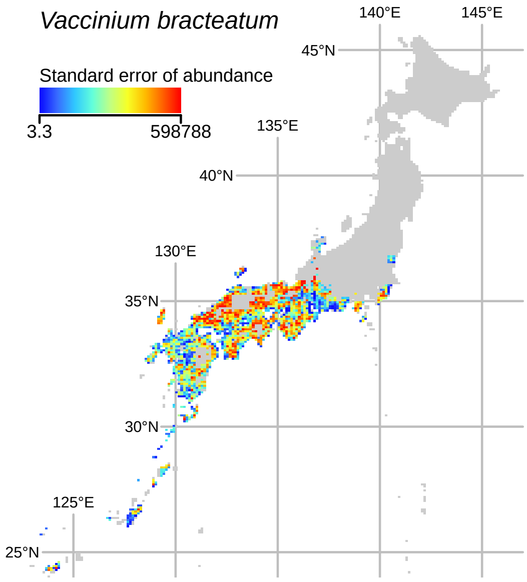
Vaccinium bracteatum
Standard error of abundance
3.3
598788
140°E
145°E
45°N
135°E
40°N
130°E
35°N
30°N
125°E
25°N

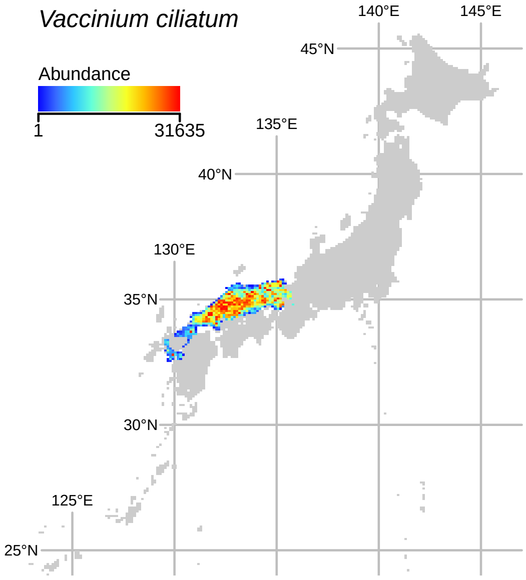
Vaccinium ciliatum
Abundance
1
31635
140°E
145°E
45°N
135°E
40°N
130°E
35°N
30°N
125°E
25°N

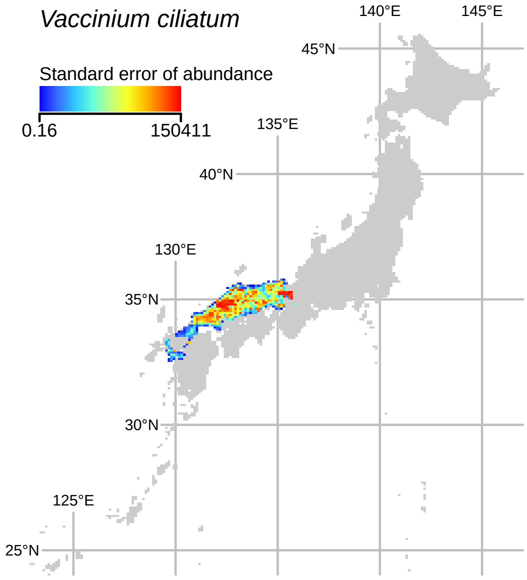
Vaccinium ciliatum
Standard error of abundance
0.16
150411
140°E
145°E
45°N
135°E
40°N
130°E
35°N
30°N
125°E
25°N

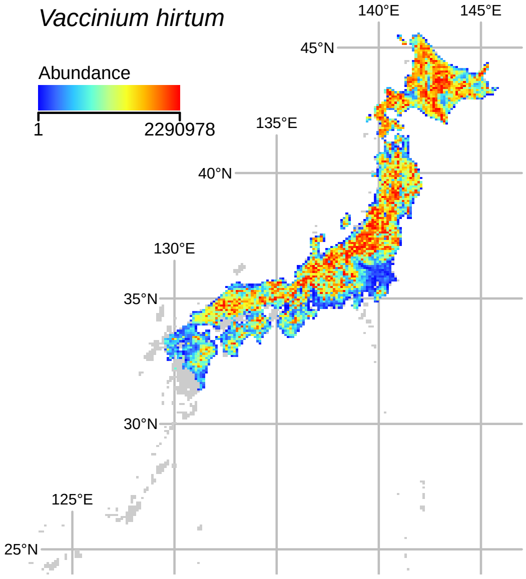
Vaccinium hirtum
Abundance
1
2290978
140°E
145°E
45°N
135°E
40°N
130°E
35°N
30°N
125°E
25°N

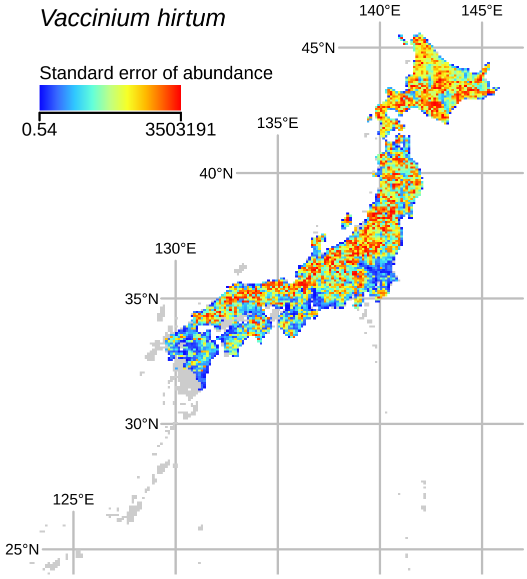
Vaccinium hirtum
Standard error of abundance
0.54
3503191
140°E
145°E
45°N
135°E
40°N
130°E
35°N
30°N
125°E
25°N

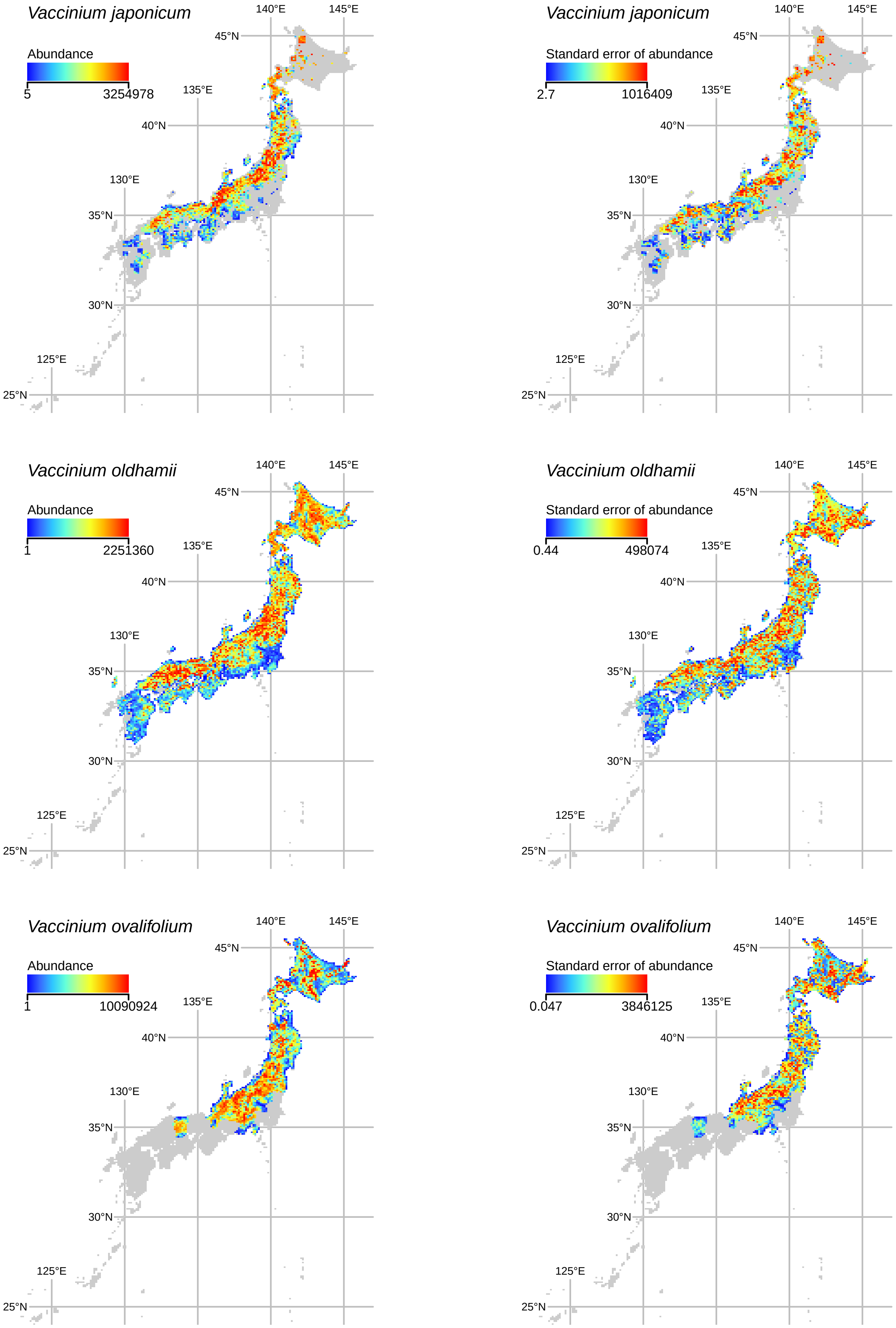
Vaccinium japonicum
Abundance
5
3254978
Vaccinium japonicum
Standard error of abundance
2.7
1016409
Vaccinium oldhamii
Abundance
1
2251360
Vaccinium oldhamii
Standard error of abundance
0.44
498074
Vaccinium ovalifolium
Abundance
1
10090924
Vaccinium ovalifolium
Standard error of abundance
0.047
3846125
125°E
130°E
135°E
140°E
145°E
25°N
30°N
35°N
40°N
45°N

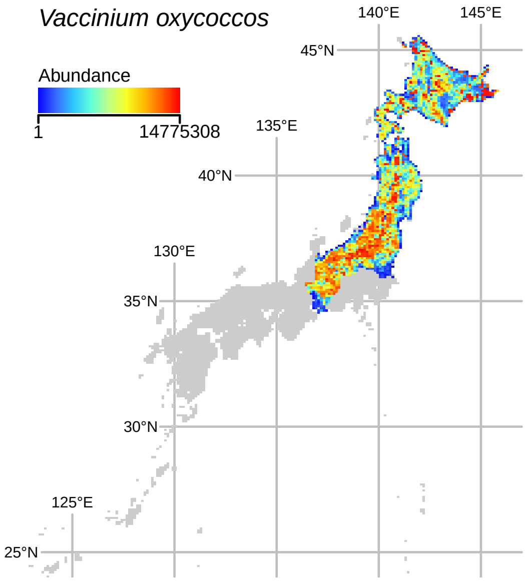
Vaccinium oxycoccos
Abundance
1
14775308
140°E
145°E
45°N
135°E
40°N
130°E
35°N
30°N
125°E
25°N

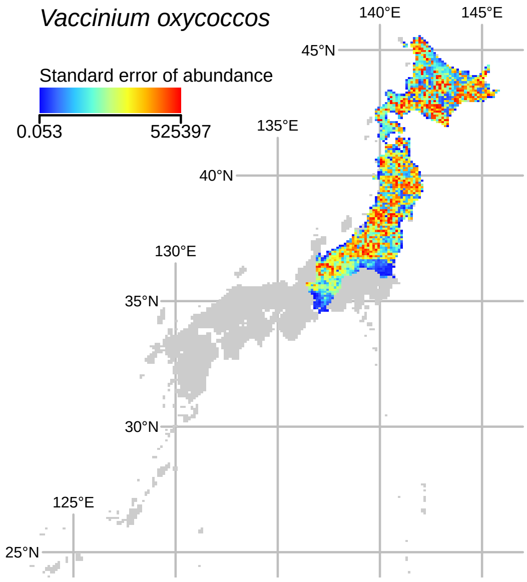
Vaccinium oxycoccos
Standard error of abundance
0.053
525397
140°E
145°E
45°N
135°E
40°N
130°E
35°N
30°N
125°E
25°N

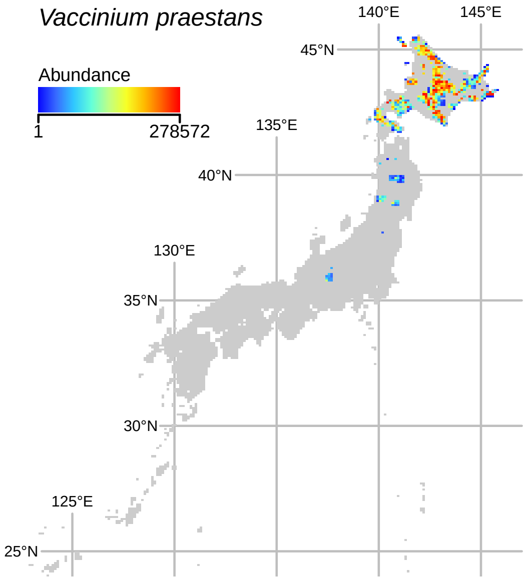
Vaccinium praestans
Abundance
1
278572
140°E
145°E
45°N
135°E
40°N
130°E
35°N
30°N
125°E
25°N

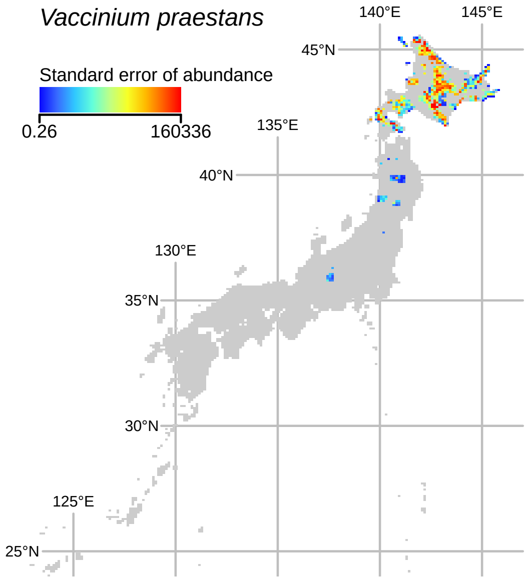
Vaccinium praestans
Standard error of abundance
0.26
160336
140°E
145°E
45°N
135°E
40°N
130°E
35°N
30°N
125°E
25°N

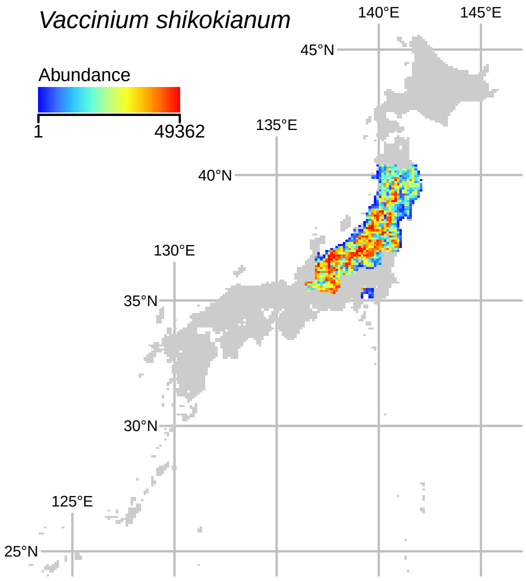
Vaccinium shikokianum
Abundance
1
49362
140°E
145°E
45°N
135°E
40°N
130°E
35°N
30°N
125°E
25°N

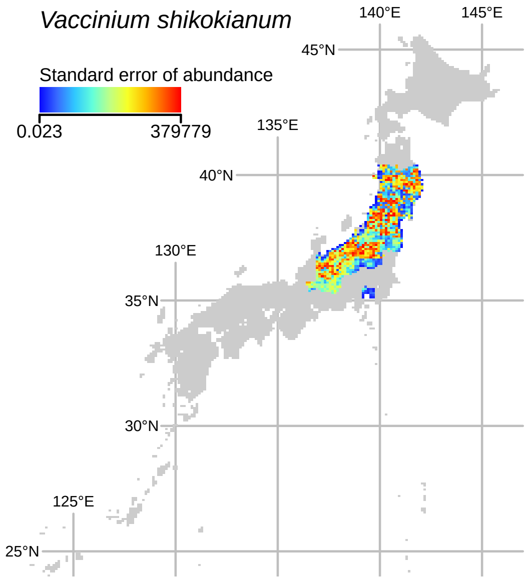
Vaccinium shikokianum
Standard error of abundance
0.023
379779
140°E
145°E
45°N
135°E
40°N
130°E
35°N
30°N
125°E
25°N

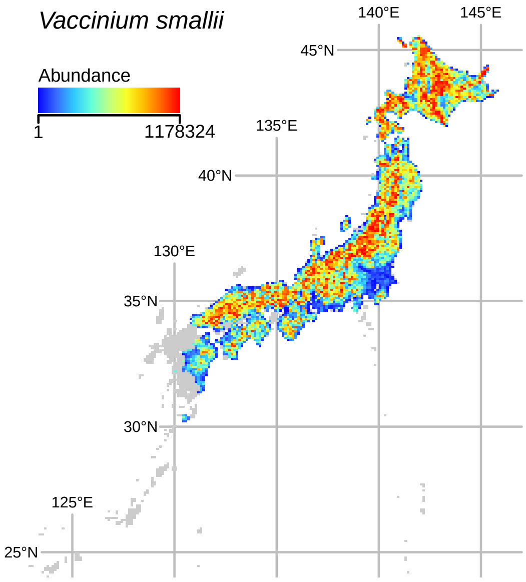
Vaccinium smallii
Abundance
1
1178324
140°E
145°E
45°N
135°E
40°N
130°E
35°N
30°N
125°E
25°N

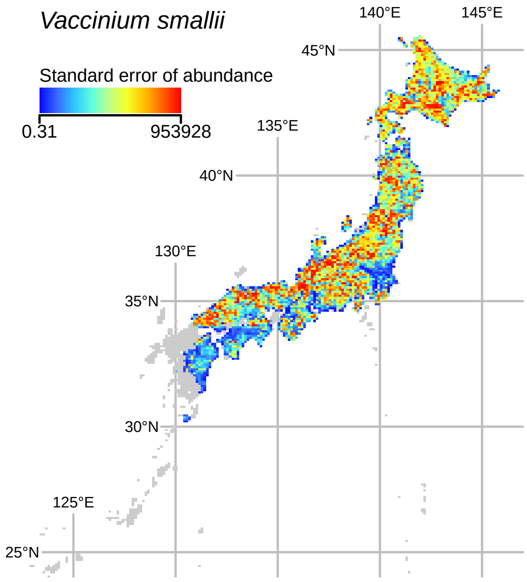
Vaccinium smallii
Standard error of abundance
0.31
953928
140°E
145°E
45°N
135°E
40°N
130°E
35°N
30°N
125°E
25°N

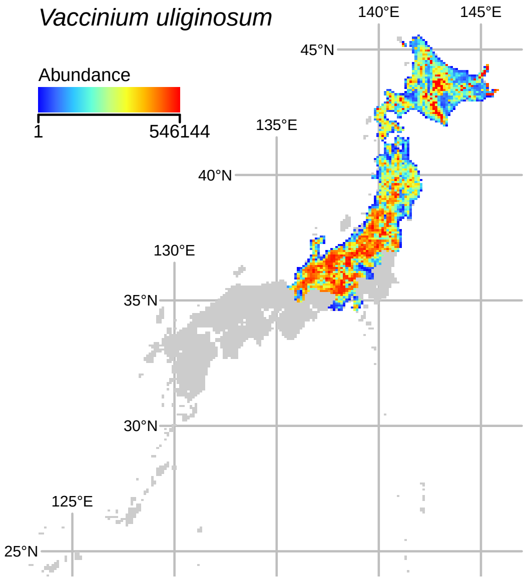
Vaccinium uliginosum
Abundance
1
546144
140°E
145°E
45°N
135°E
40°N
130°E
35°N
30°N
125°E
25°N

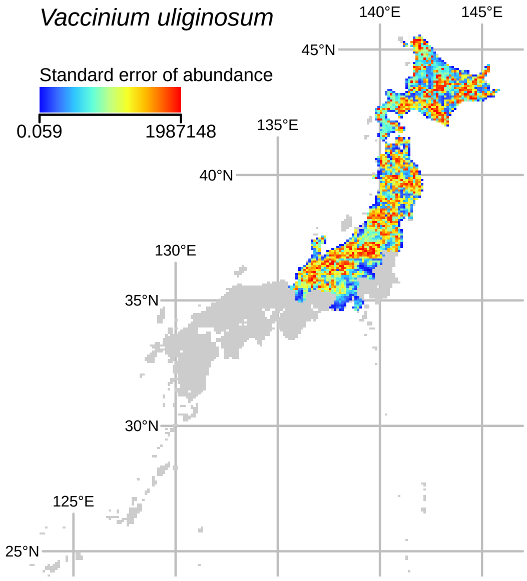
Vaccinium uliginosum
Standard error of abundance
0.059
1987148
140°E
145°E
45°N
135°E
40°N
130°E
35°N
30°N
125°E
25°N

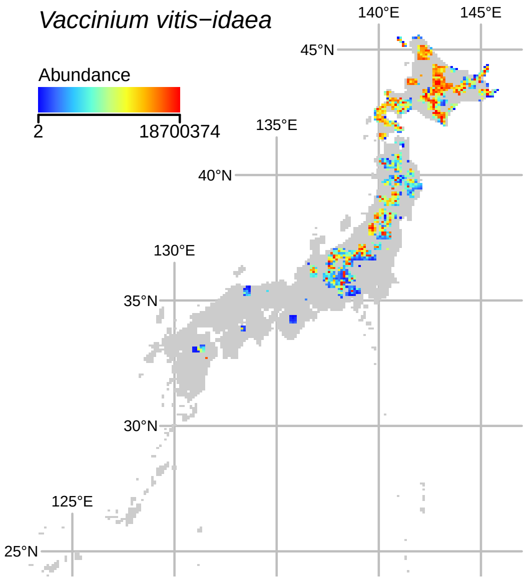
Vaccinium vitis−idaea
Abundance
2
18700374
140°E
145°E
45°N
135°E
40°N
130°E
35°N
30°N
125°E
25°N

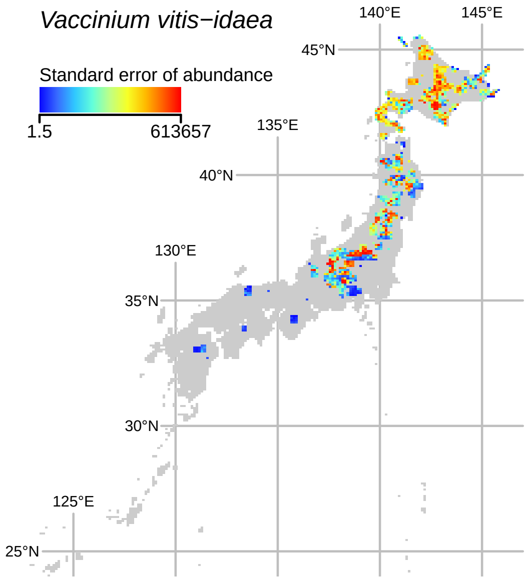
Vaccinium vitis−idaea
Standard error of abundance
1.5
613657
140°E
145°E
45°N
135°E
40°N
130°E
35°N
30°N
125°E
25°N

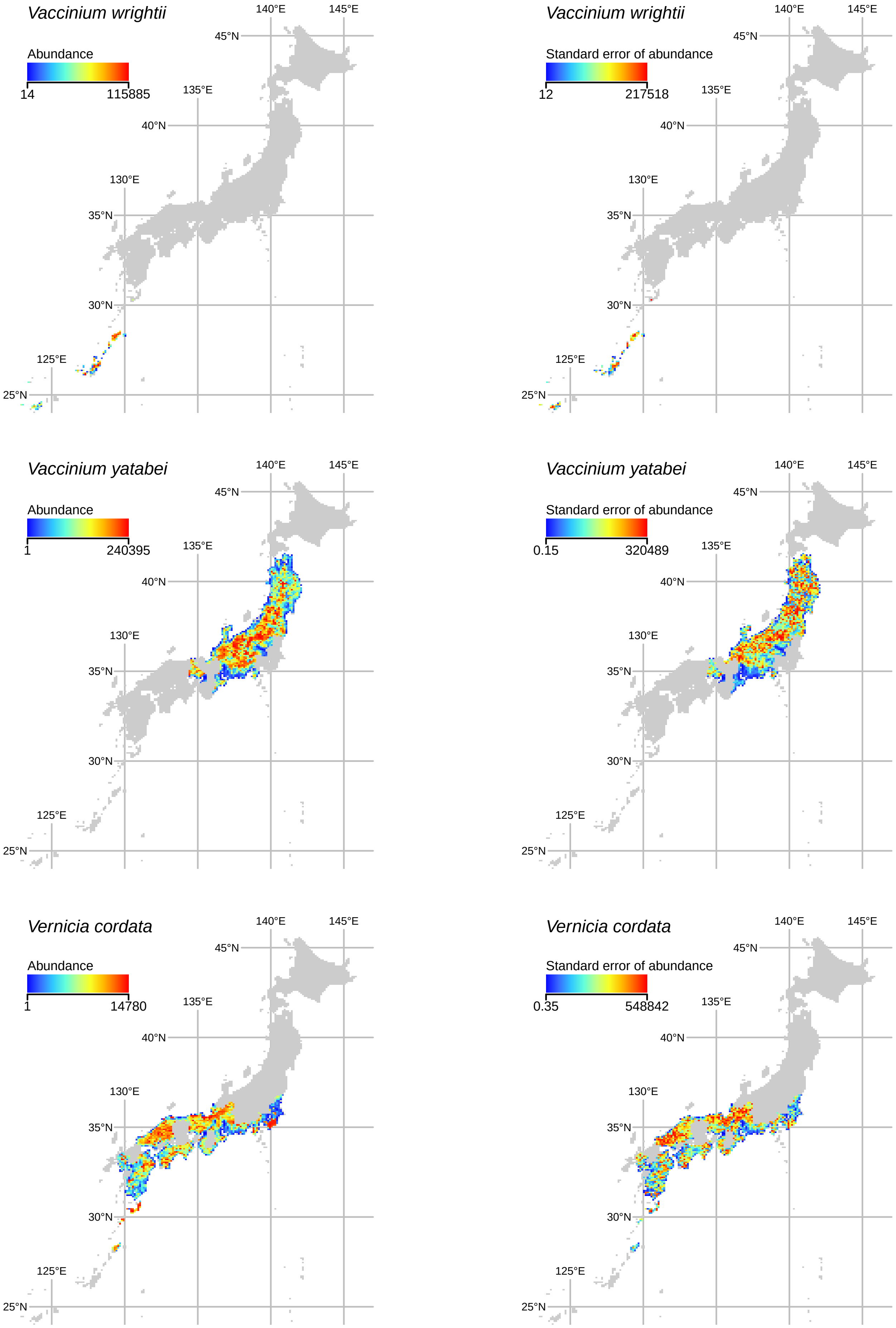
Vaccinium wrightii
Abundance
14
115885
Vaccinium wrightii
Standard error of abundance
12
217518
Vaccinium yatabei
Abundance
1
240395
Vaccinium yatabei
Standard error of abundance
0.15
320489
Vernicia cordata
Abundance
1
14780
Vernicia cordata
Standard error of abundance
0.35
548842
125°E
130°E
135°E
140°E
145°E
25°N
30°N
35°N
40°N
45°N

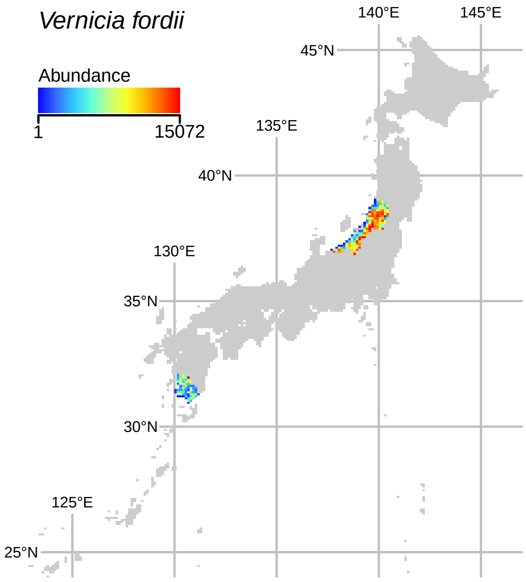
Vernicia fordii
Abundance
1
15072
140°E
145°E
45°N
135°E
40°N
130°E
35°N
30°N
125°E
25°N

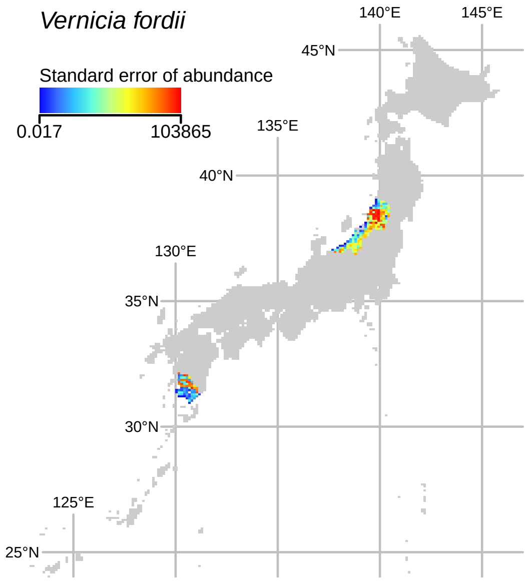
Vernicia fordii
Standard error of abundance
0.017
103865
140°E
145°E
45°N
135°E
40°N
130°E
35°N
30°N
125°E
25°N

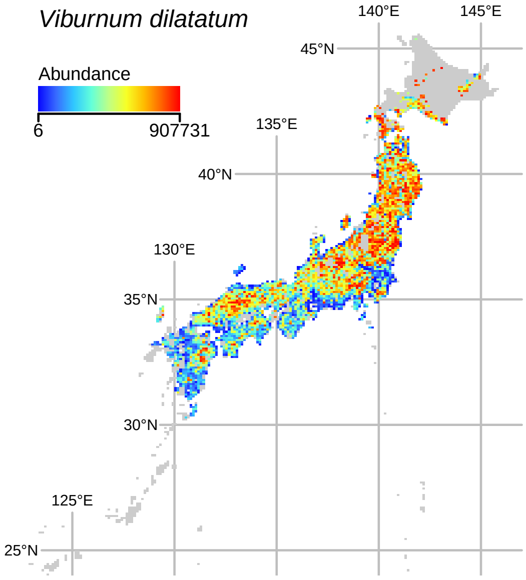
Viburnum dilatatum
Abundance
6
907731
140°E
145°E
45°N
135°E
40°N
130°E
35°N
30°N
125°E
25°N

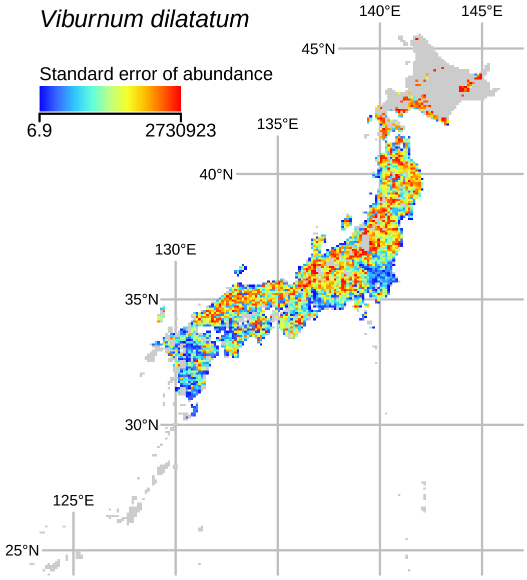
Viburnum dilatatum
Standard error of abundance
6.9
2730923
140°E
145°E
45°N
135°E
40°N
130°E
35°N
30°N
125°E
25°N

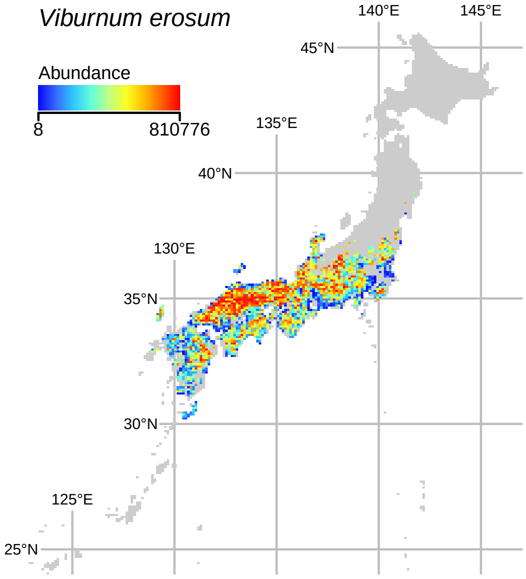
Viburnum erosum
Abundance
8
810776
140°E
145°E
45°N
135°E
40°N
130°E
35°N
30°N
125°E
25°N

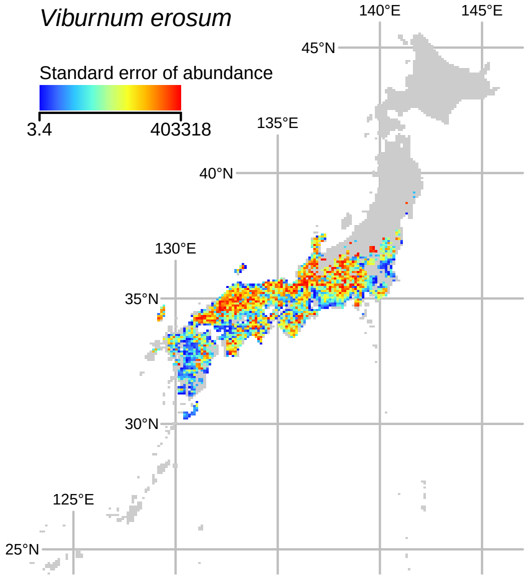
Viburnum erosum
Standard error of abundance
3.4
403318
140°E
145°E
45°N
135°E
40°N
130°E
35°N
30°N
125°E
25°N

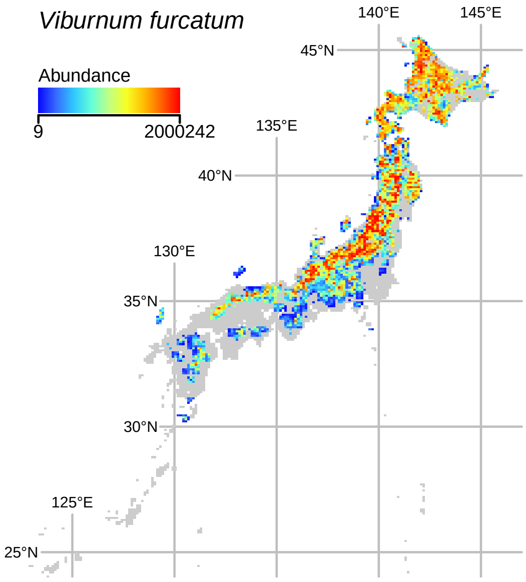
Viburnum furcatum
Abundance
9
2000242
140°E
145°E
45°N
135°E
40°N
130°E
35°N
30°N
125°E
25°N

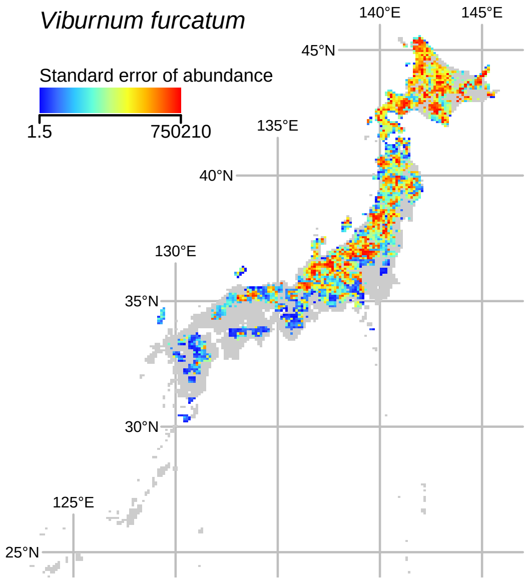
Viburnum furcatum
Standard error of abundance
1.5
750210
140°E
145°E
45°N
135°E
40°N
130°E
35°N
30°N
125°E
25°N

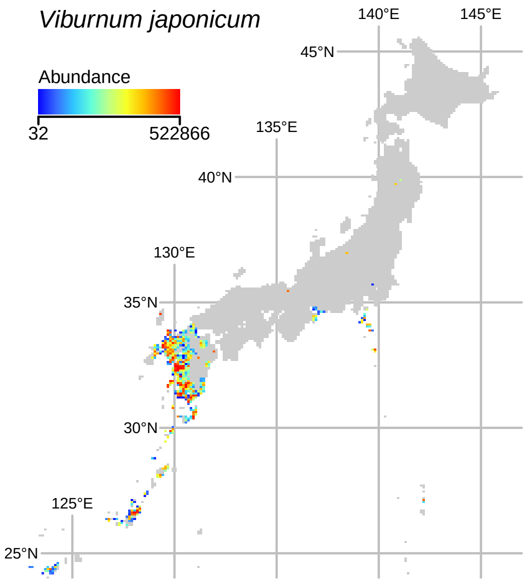
Viburnum japonicum
Abundance
32
522866
140°E
145°E
45°N
135°E
40°N
130°E
35°N
30°N
125°E
25°N

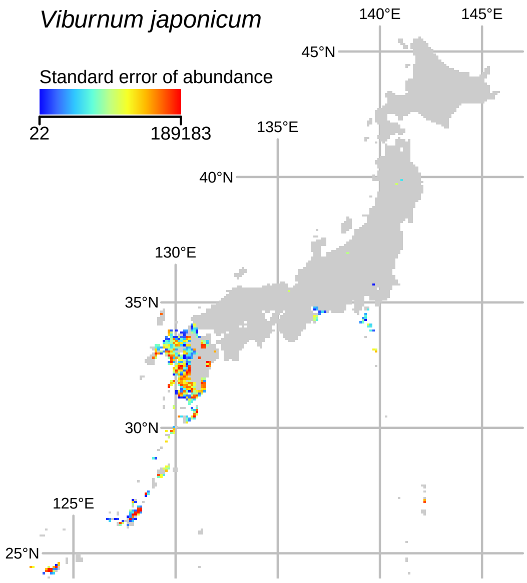
Viburnum japonicum
Standard error of abundance
22
189183
140°E
145°E
45°N
135°E
40°N
130°E
35°N
30°N
125°E
25°N

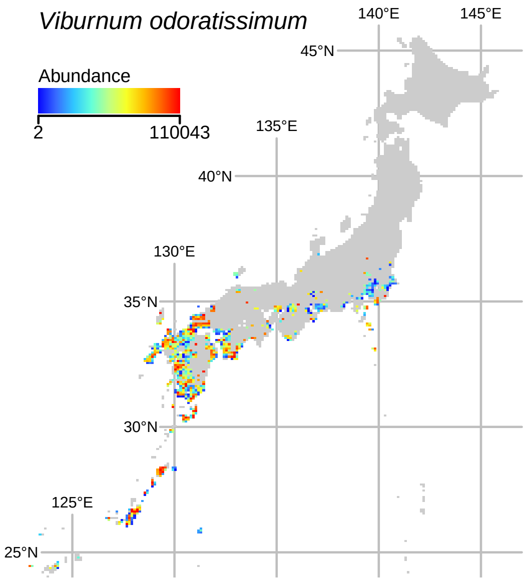
Viburnum odoratissimum
Abundance
2
110043
140°E
145°E
45°N
135°E
40°N
130°E
35°N
30°N
125°E
25°N

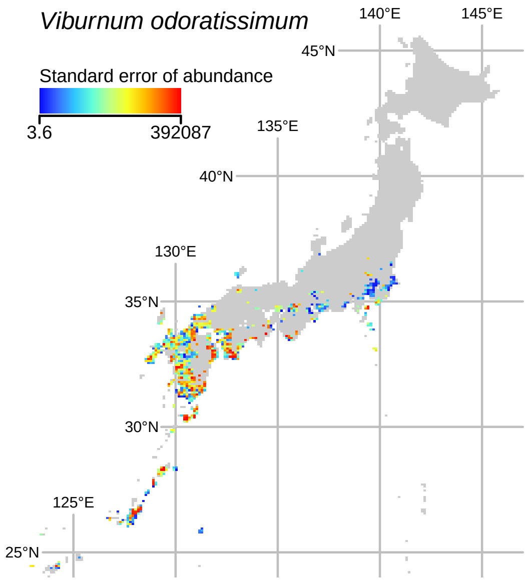
Viburnum odoratissimum
Standard error of abundance
3.6
392087
140°E
145°E
45°N
135°E
40°N
130°E
35°N
30°N
125°E
25°N

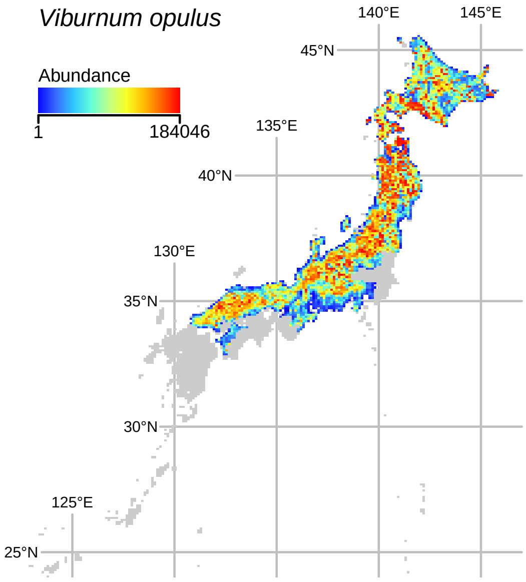
Viburnum opulus
Abundance
1
184046
140°E
145°E
45°N
135°E
40°N
130°E
35°N
30°N
125°E
25°N

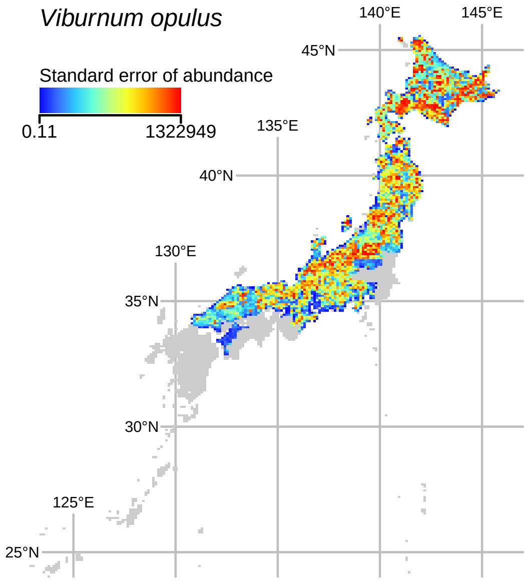
Viburnum opulus
Standard error of abundance
0.11
1322949
140°E
145°E
45°N
135°E
40°N
130°E
35°N
30°N
125°E
25°N

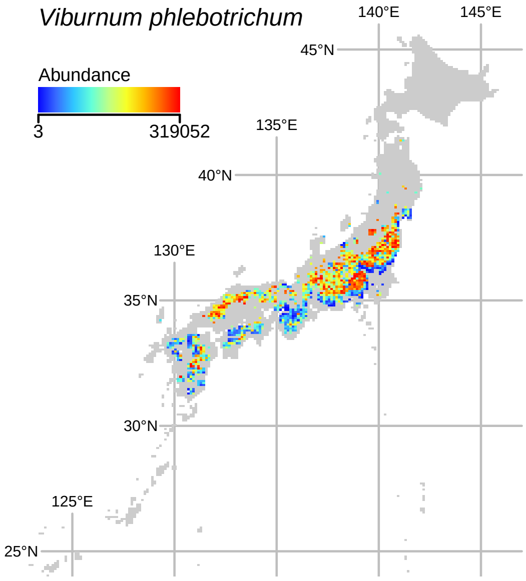
Viburnum phlebotrichum
Abundance
3
319052
140°E
145°E
45°N
135°E
40°N
130°E
35°N
30°N
125°E
25°N

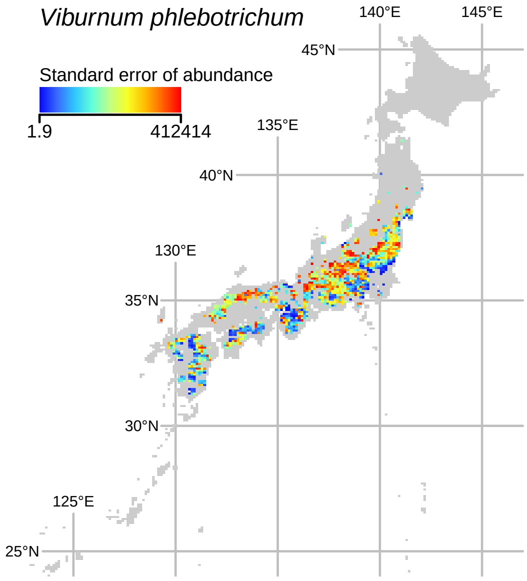
Viburnum phlebotrichum
Standard error of abundance
1.9
412414
140°E
145°E
45°N
135°E
40°N
130°E
35°N
30°N
125°E
25°N

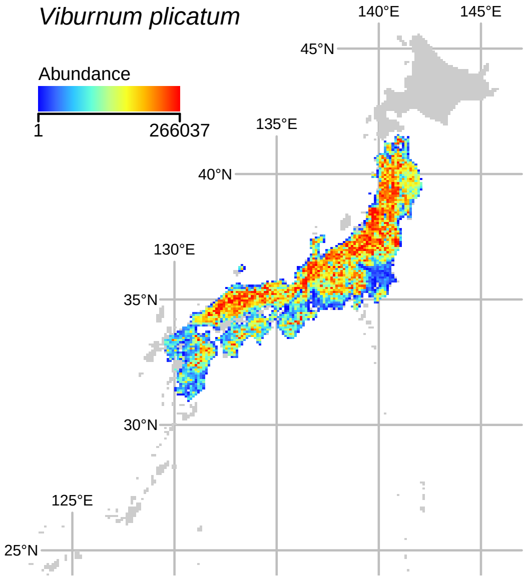
Viburnum plicatum
Abundance
1
266037
140°E
145°E
45°N
135°E
40°N
130°E
35°N
30°N
125°E
25°N

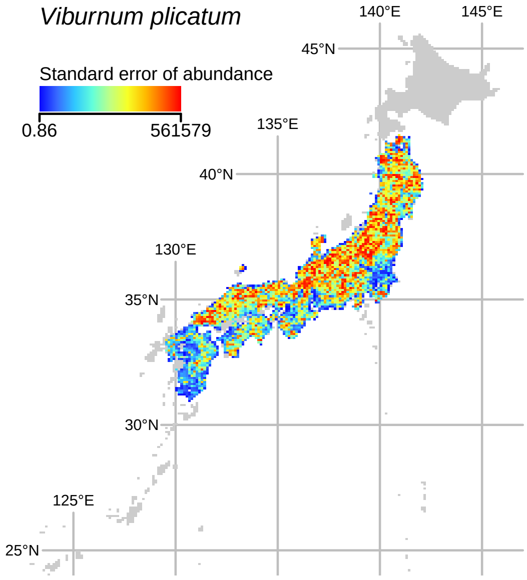
Viburnum plicatum
Standard error of abundance
0.86
561579
140°E
145°E
45°N
135°E
40°N
130°E
35°N
30°N
125°E
25°N

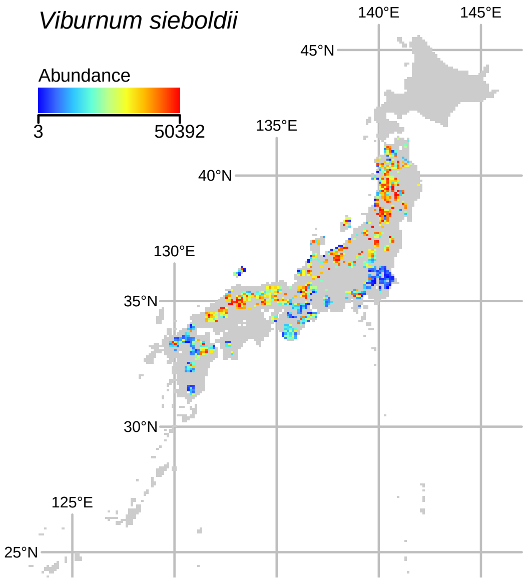
Viburnum sieboldii
Abundance
3
50392
140°E
145°E
45°N
135°E
40°N
130°E
35°N
30°N
125°E
25°N

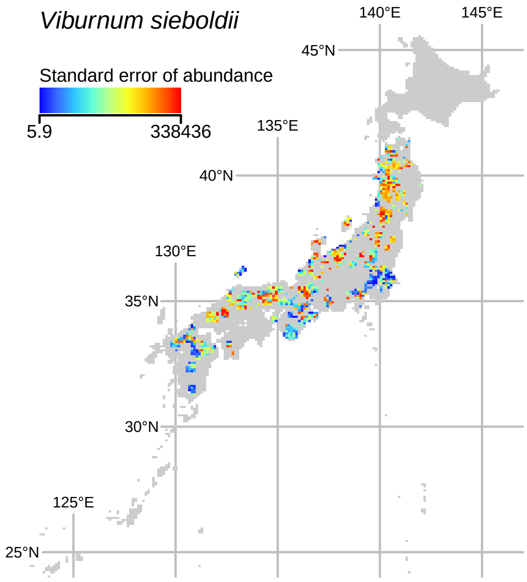
Viburnum sieboldii
Standard error of abundance
5.9
338436
140°E
145°E
45°N
135°E
40°N
130°E
35°N
30°N
125°E
25°N

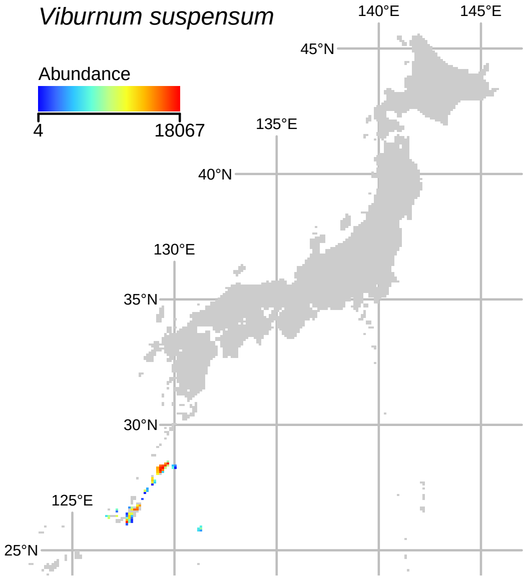
Viburnum suspensum
Abundance
4
18067
140°E
145°E
45°N
135°E
40°N
130°E
35°N
30°N
125°E
25°N

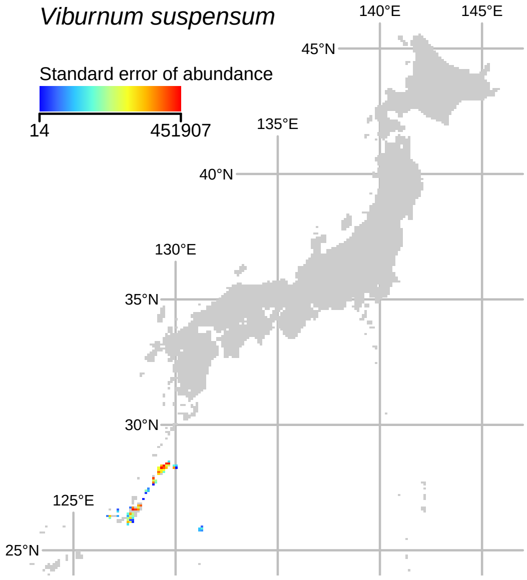
Viburnum suspensum
Standard error of abundance
14
451907
140°E
145°E
45°N
135°E
40°N
130°E
35°N
30°N
125°E
25°N

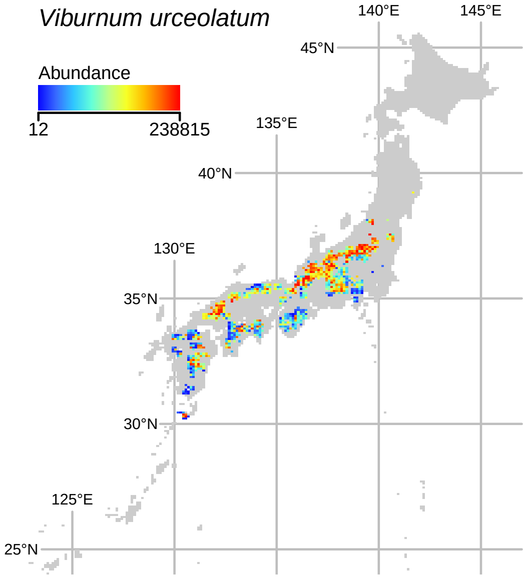
Viburnum urceolatum
Abundance
12
238815
140°E
145°E
45°N
135°E
40°N
130°E
35°N
30°N
125°E
25°N

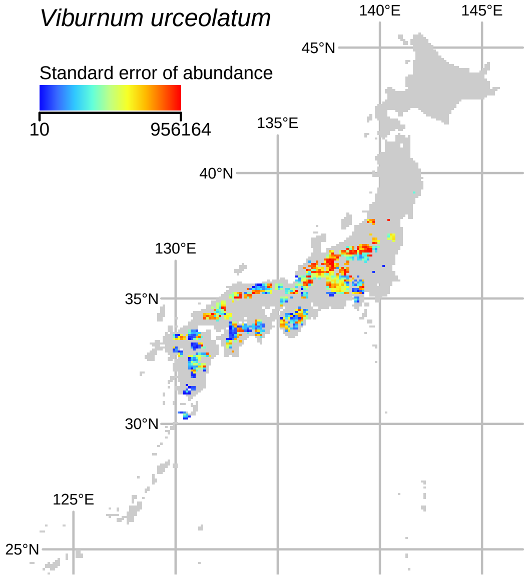
Viburnum urceolatum
Standard error of abundance
10
956164
140°E
145°E
45°N
135°E
40°N
130°E
35°N
30°N
125°E
25°N

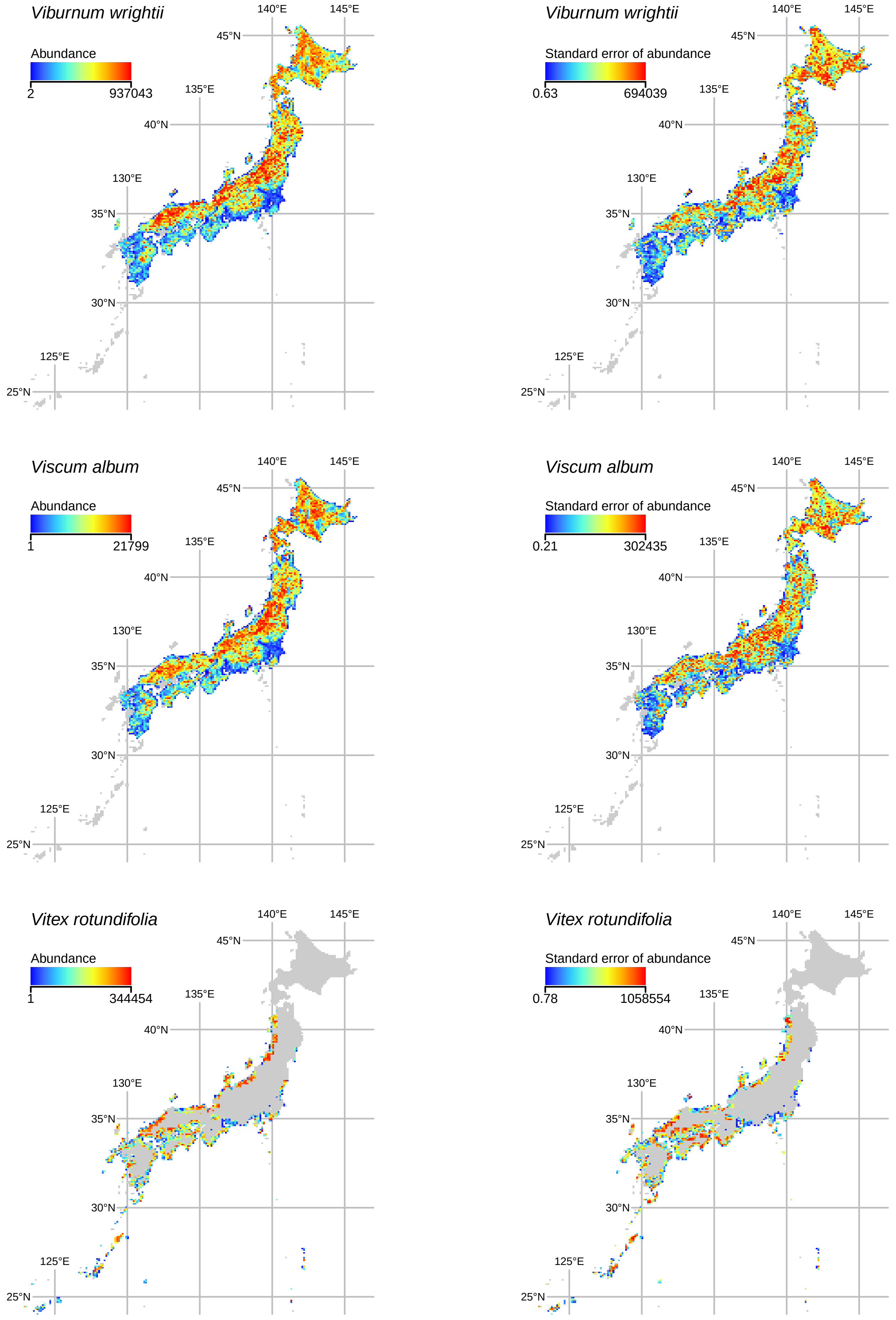
Viburnum wrightii
Abundance
2
937043
Viburnum wrightii
Standard error of abundance
0.63
694039
Viscum album
Abundance
1
21799
Viscum album
Standard error of abundance
0.21
302435
Vitex rotundifolia
Abundance
1
344454
Vitex rotundifolia
Standard error of abundance
0.78
1058554
125°E
130°E
135°E
140°E
145°E
25°N
30°N
35°N
40°N
45°N

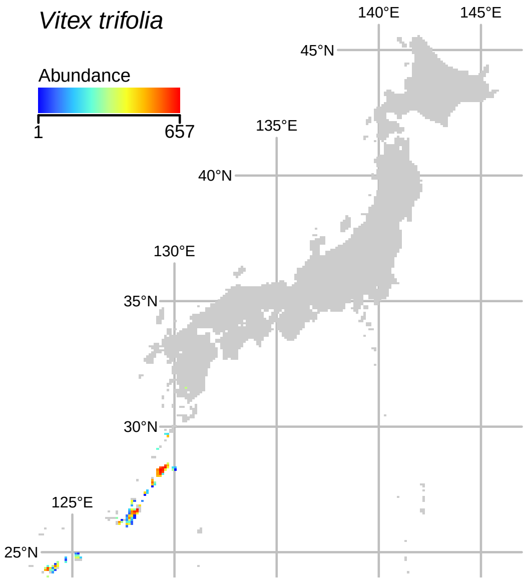
Vitex trifolia
Abundance
1
657
140°E
145°E
45°N
135°E
40°N
130°E
35°N
30°N
125°E
25°N

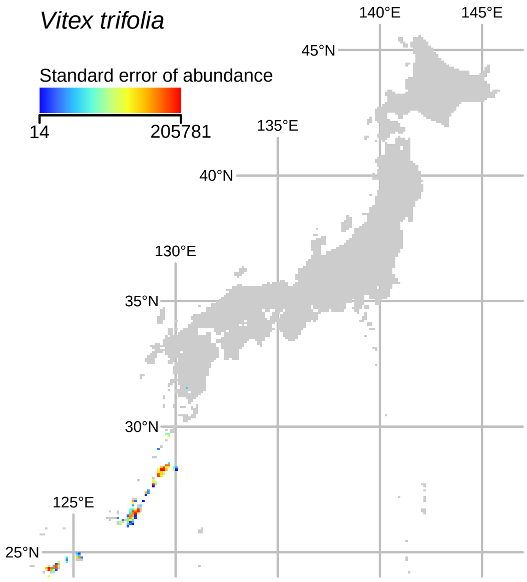
Vitex trifolia
Standard error of abundance
14
205781
140°E
145°E
45°N
135°E
40°N
130°E
35°N
30°N
125°E
25°N

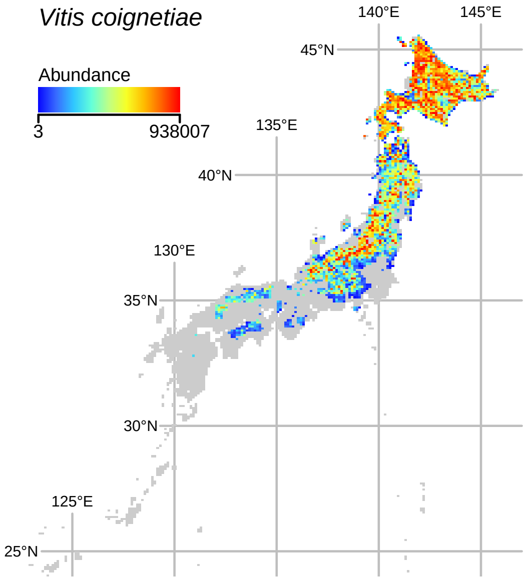
Vitis coignetiae
Abundance
3
938007
140°E
145°E
45°N
135°E
40°N
130°E
35°N
30°N
125°E
25°N

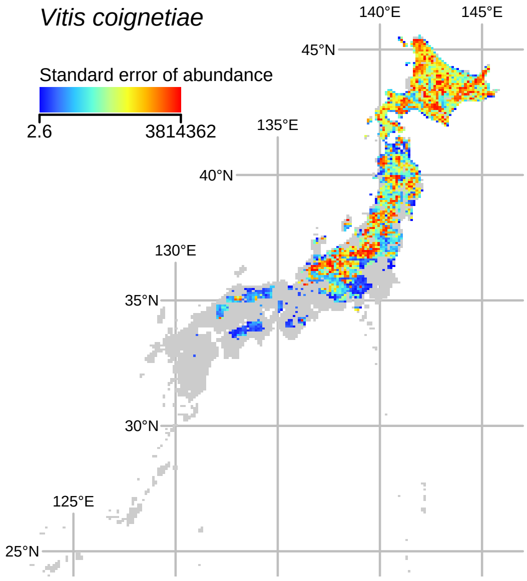
Vitis coignetiae
Standard error of abundance
2.6
3814362
140°E
145°E
45°N
135°E
40°N
130°E
35°N
30°N
125°E
25°N

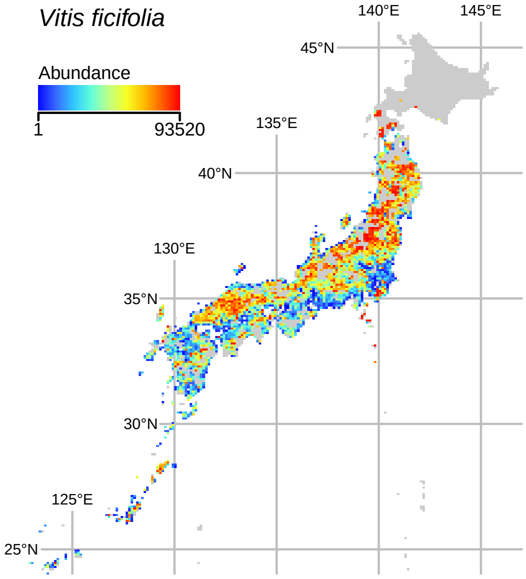
Vitis ficifolia
Abundance
1
93520
140°E
145°E
45°N
135°E
40°N
130°E
35°N
30°N
125°E
25°N

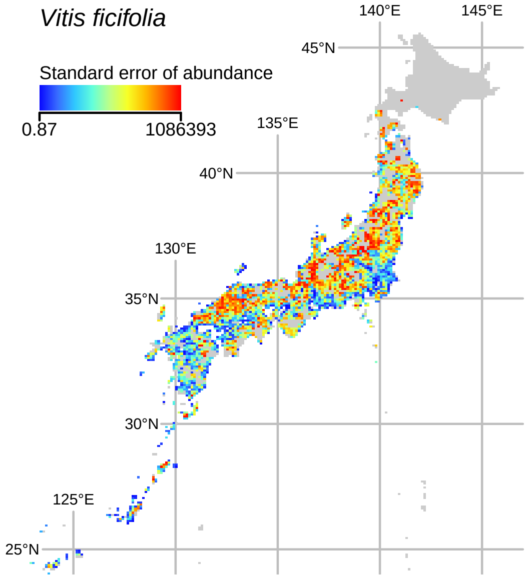
Vitis ficifolia
Standard error of abundance
0.87
1086393
140°E
145°E
45°N
135°E
40°N
130°E
35°N
30°N
125°E
25°N

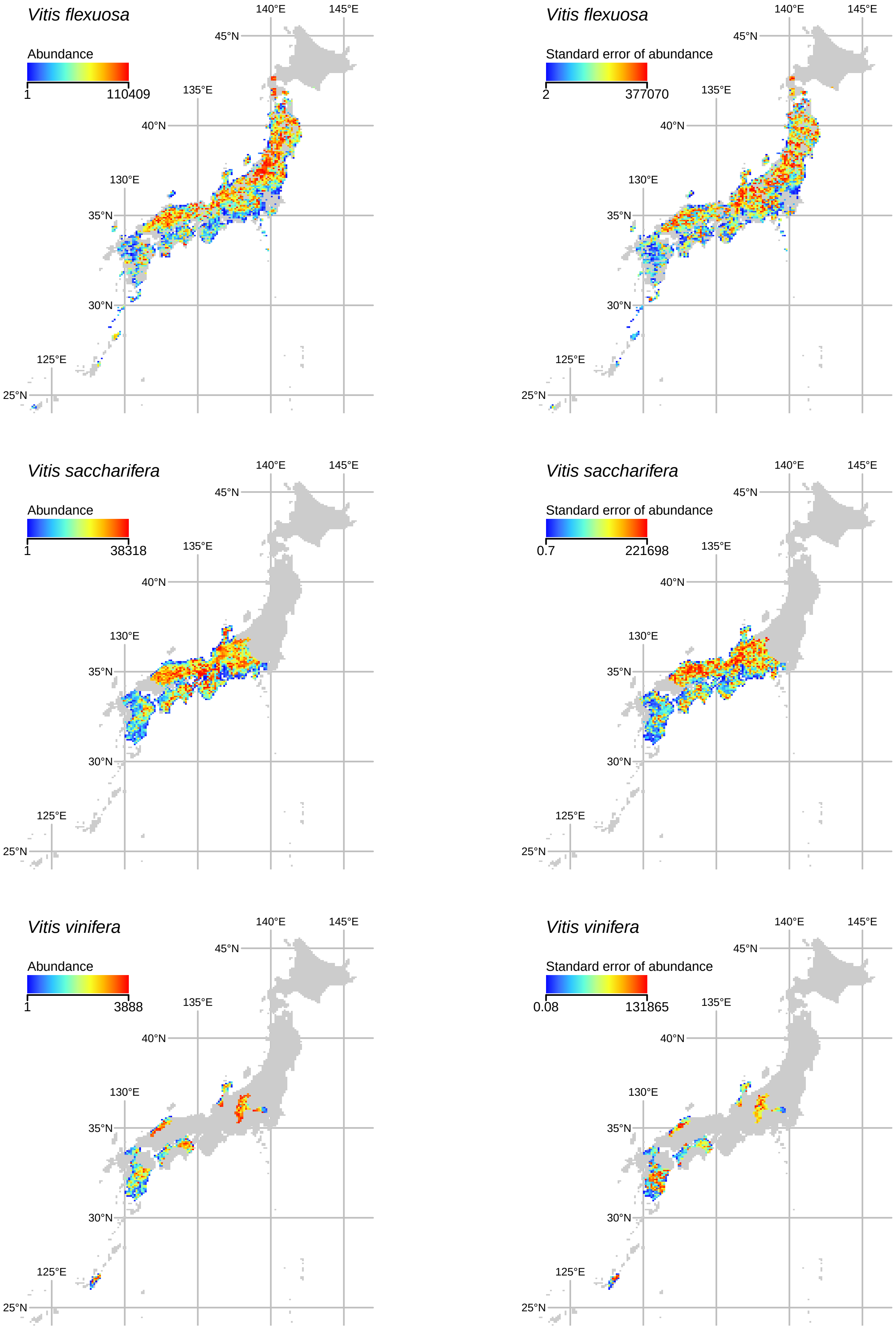
Vitis flexuosa
Abundance
1
110409
Vitis flexuosa
Standard error of abundance
2
377070
Vitis saccharifera
Abundance
1
38318
Vitis saccharifera
Standard error of abundance
0.7
221698
Vitis vinifera
Abundance
1
3888
Vitis vinifera
Standard error of abundance
0.08
131865
125°E
130°E
135°E
140°E
145°E
25°N
30°N
35°N
40°N
45°N

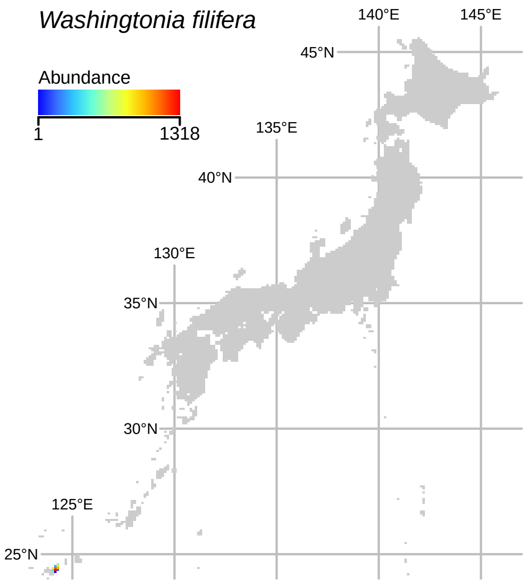
Washingtonia filifera
Abundance
1
1318
140°E
145°E
45°N
135°E
40°N
130°E
35°N
30°N
125°E
25°N

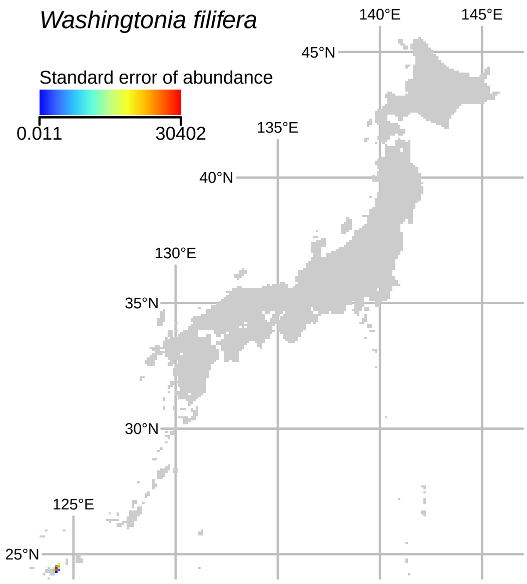
Washingtonia filifera
Standard error of abundance
0.011
30402
140°E
145°E
45°N
135°E
40°N
130°E
35°N
30°N
125°E
25°N

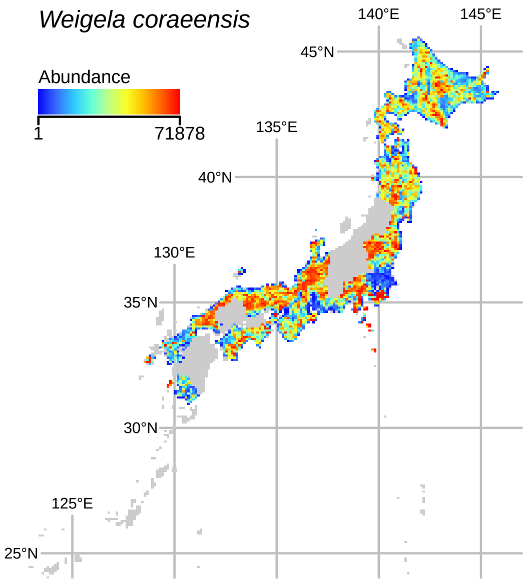
Weigela coraeensis
Abundance
1
71878
140°E
145°E
45°N
135°E
40°N
130°E
35°N
30°N
125°E
25°N

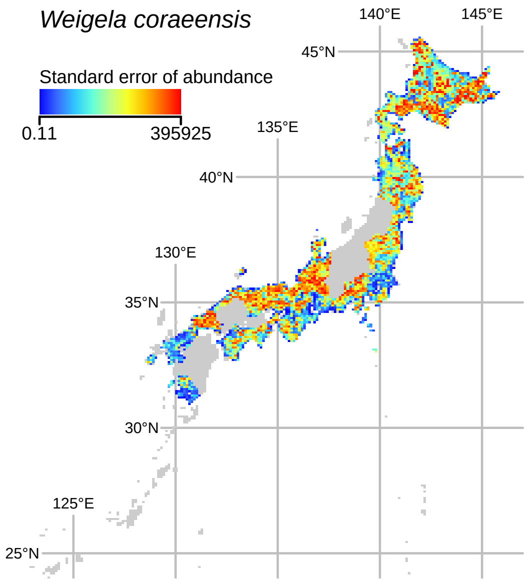
Weigela coraeensis
Standard error of abundance
0.11
395925
140°E
145°E
45°N
135°E
40°N
130°E
35°N
30°N
125°E
25°N

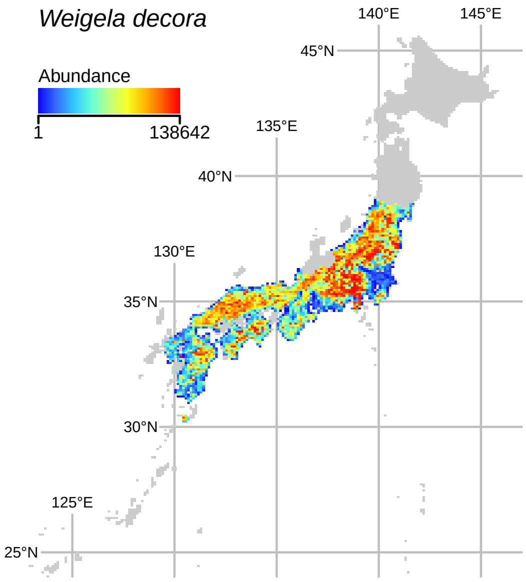
Weigela decora
Abundance
1
138642
140°E
145°E
45°N
135°E
40°N
130°E
35°N
30°N
125°E
25°N

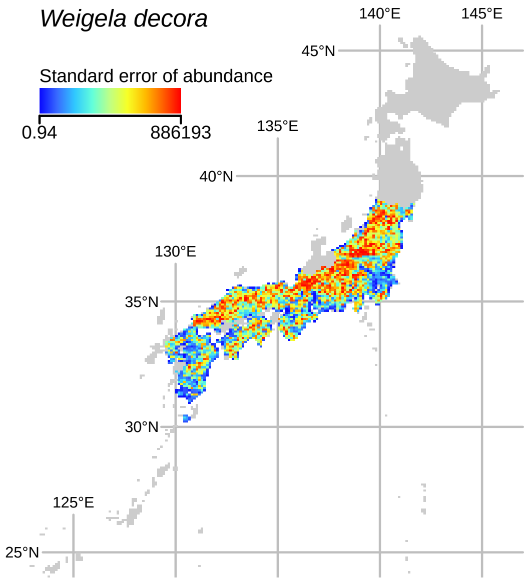
Weigela decora
Standard error of abundance
0.94
886193
140°E
145°E
45°N
135°E
40°N
130°E
35°N
30°N
125°E
25°N

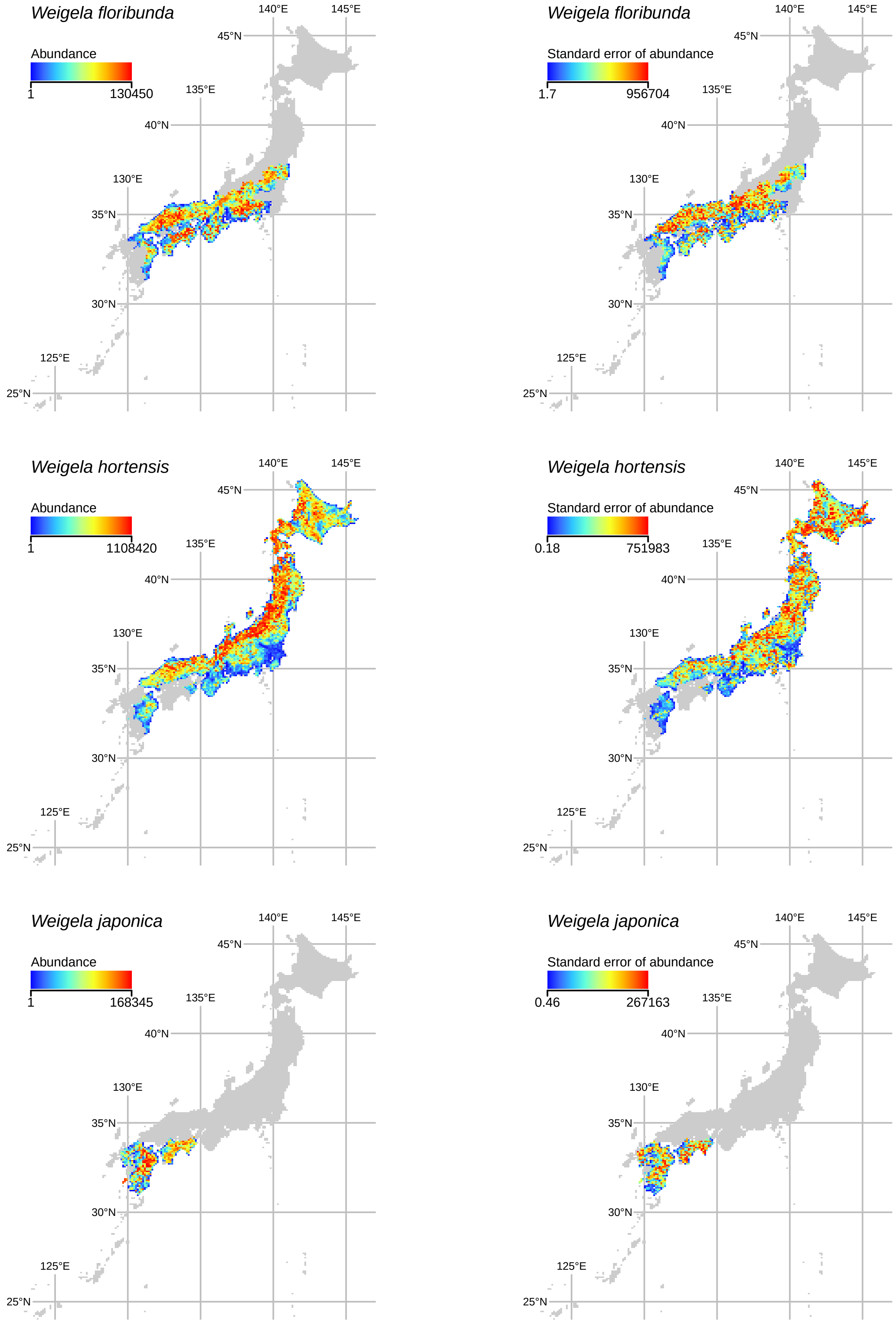
Weigela floribunda
Abundance
1
130450
Weigela floribunda
Standard error of abundance
1.7
956704
Weigela hortensis
Abundance
1
1108420
Weigela hortensis
Standard error of abundance
0.18
751983
Weigela japonica
Abundance
1
168345
Weigela japonica
Standard error of abundance
0.46
267163
140°E
145°E
135°E
130°E
125°E
45°N
40°N
35°N
30°N
25°N

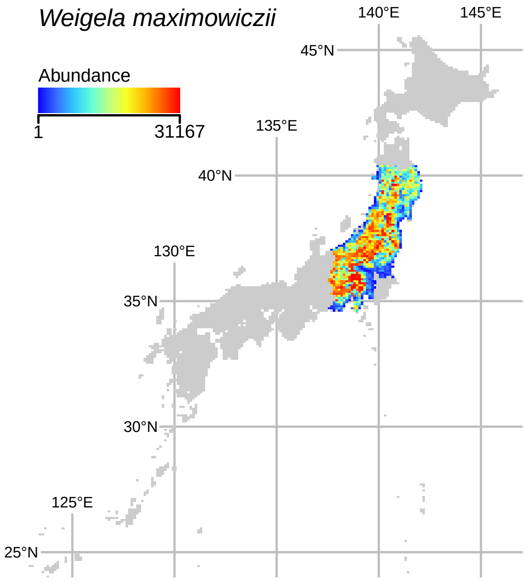
Weigela maximowiczii
Abundance
1
31167
140°E
145°E
45°N
135°E
40°N
130°E
35°N
30°N
125°E
25°N

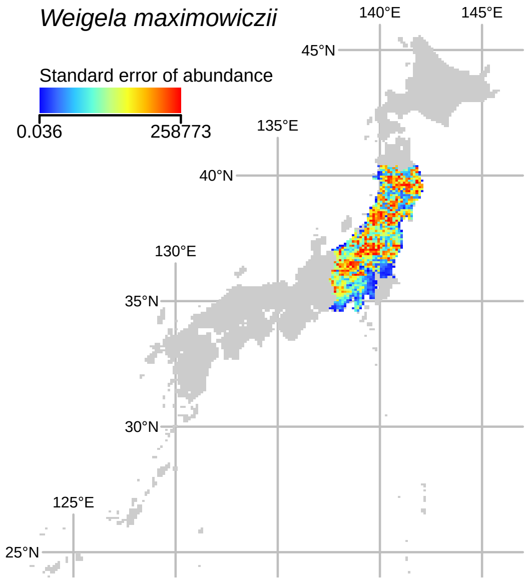
Weigela maximowiczii
Standard error of abundance
0.036
258773
140°E
145°E
45°N
135°E
40°N
130°E
35°N
30°N
125°E
25°N

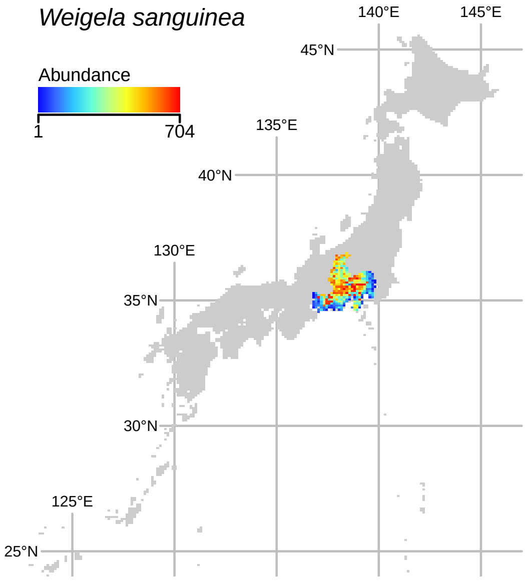
Weigela sanguinea
Abundance
1
704
140°E
145°E
45°N
135°E
40°N
130°E
35°N
30°N
125°E
25°N

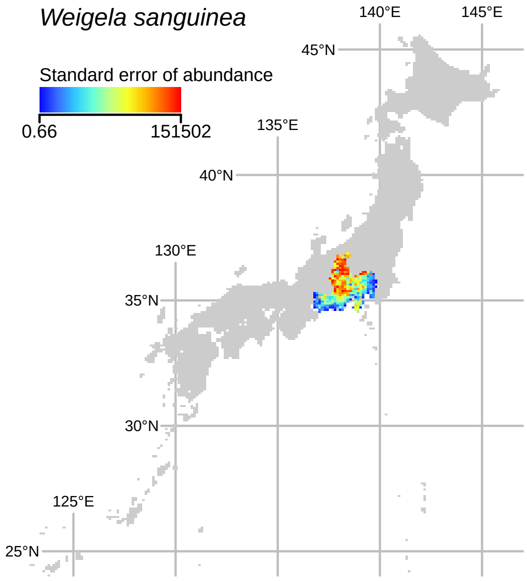
Weigela sanguinea
Standard error of abundance
0.66
151502
140°E
145°E
45°N
135°E
40°N
130°E
35°N
30°N
125°E
25°N

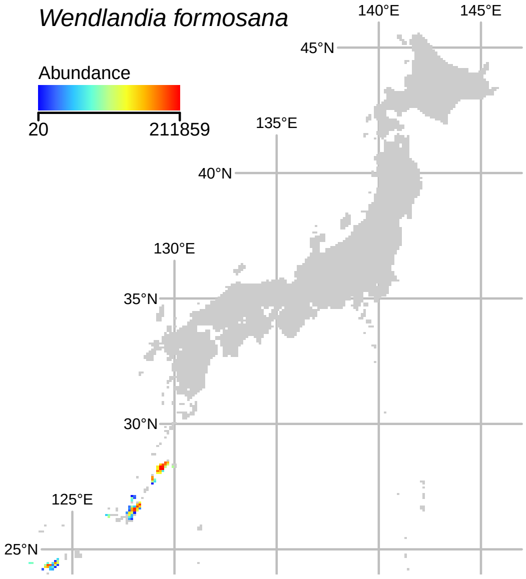
Wendlandia formosana
Abundance
20
211859
140°E
145°E
45°N
135°E
40°N
130°E
35°N
30°N
125°E
25°N

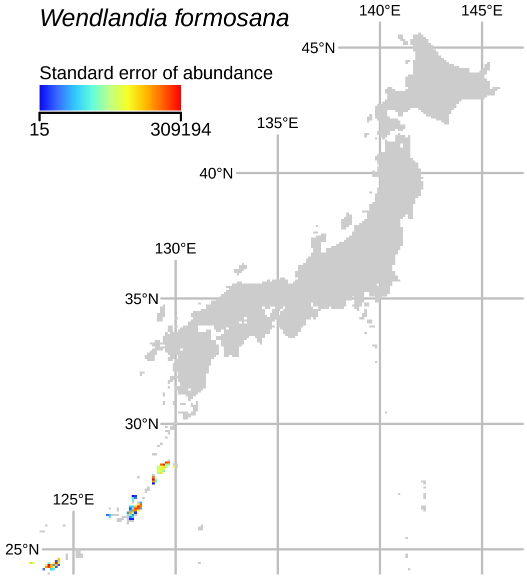
Wendlandia formosana
Standard error of abundance
15
309194
140°E
145°E
45°N
135°E
40°N
130°E
35°N
30°N
125°E
25°N

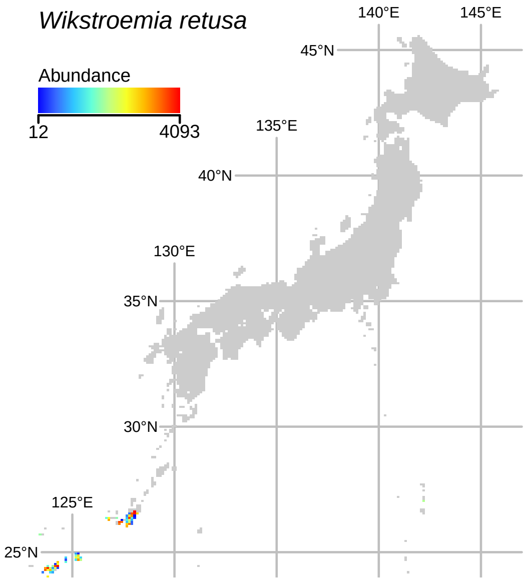
Wikstroemia retusa
Abundance
12
4093
140°E
145°E
45°N
135°E
40°N
130°E
35°N
30°N
125°E
25°N

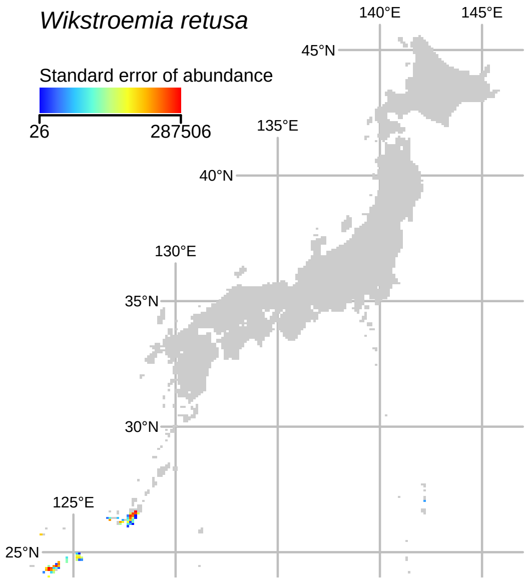
Wikstroemia retusa
Standard error of abundance
26
287506
140°E
145°E
45°N
135°E
40°N
130°E
35°N
30°N
125°E
25°N

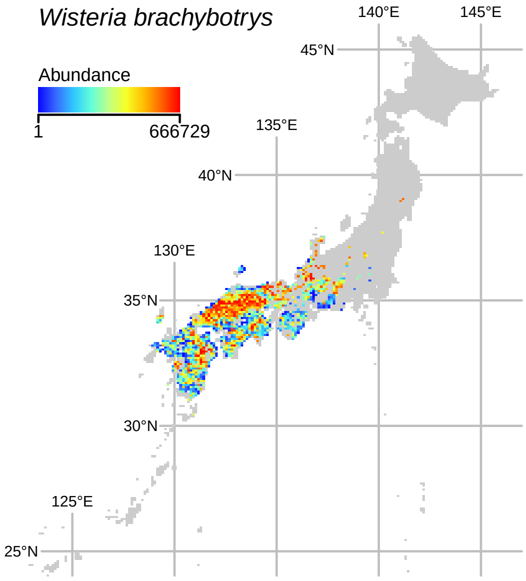
Wisteria brachybotrys
Abundance
1
666729
140°E
145°E
45°N
135°E
40°N
130°E
35°N
30°N
125°E
25°N

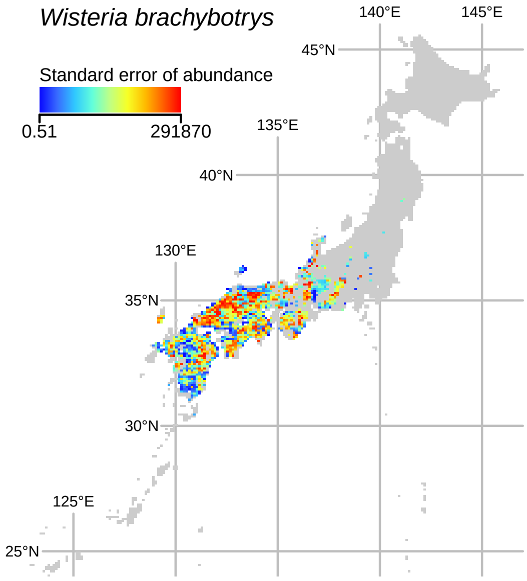
Wisteria brachybotrys
Standard error of abundance
0.51
291870
140°E
145°E
45°N
135°E
40°N
130°E
35°N
30°N
125°E
25°N

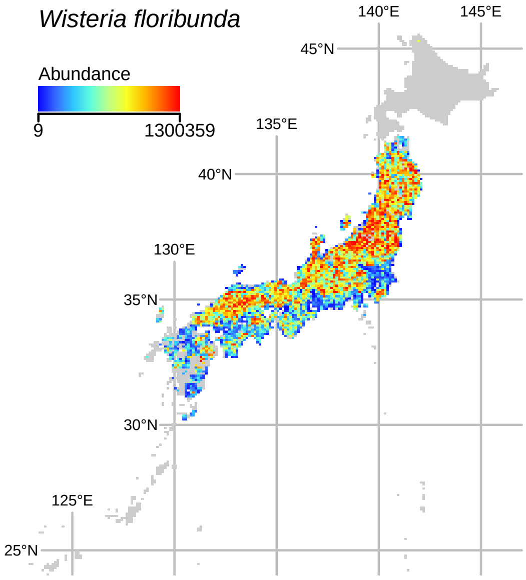
Wisteria floribunda
Abundance
9
1300359
140°E
145°E
45°N
135°E
40°N
130°E
35°N
30°N
125°E
25°N

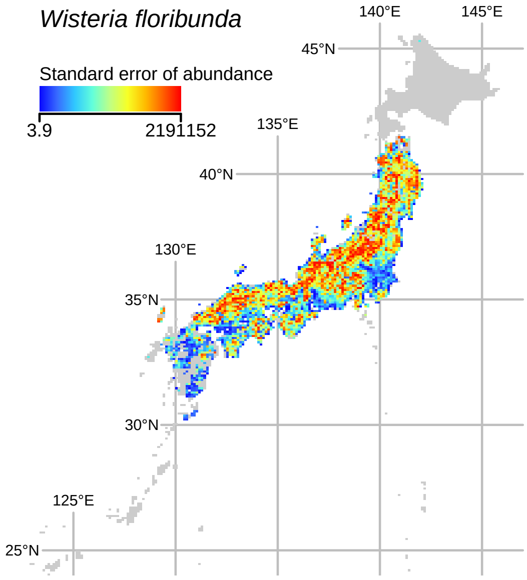
Wisteria floribunda
Standard error of abundance
3.9
2191152
140°E
145°E
45°N
135°E
40°N
130°E
35°N
30°N
125°E
25°N

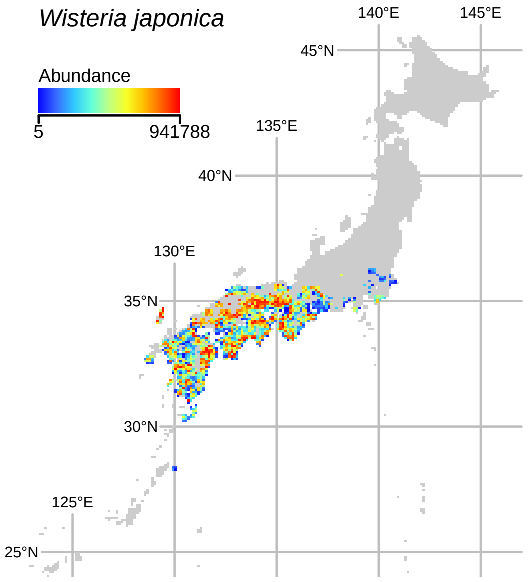
Wisteria japonica
Abundance
5
941788
140°E
145°E
45°N
135°E
40°N
130°E
35°N
30°N
125°E
25°N

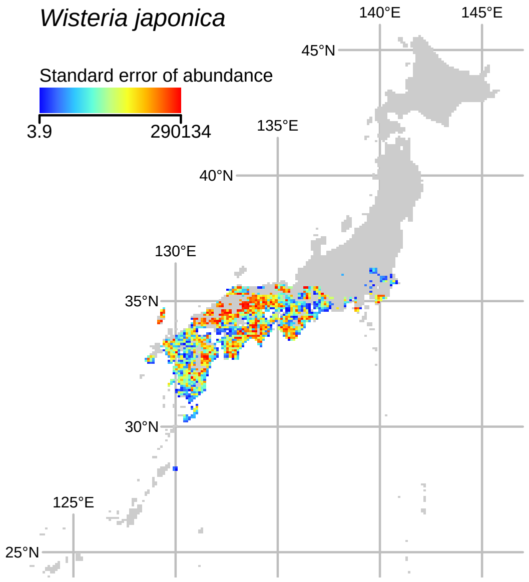
Wisteria japonica
Standard error of abundance
3.9
290134
140°E
145°E
45°N
135°E
40°N
130°E
35°N
30°N
125°E
25°N

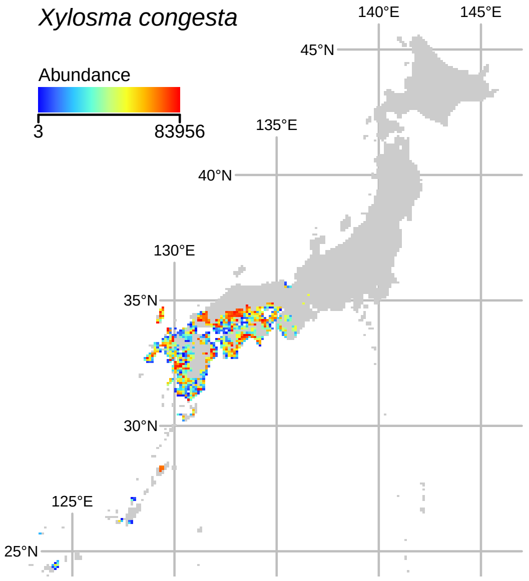
Xylosma congesta
Abundance
3
83956
140°E
145°E
45°N
135°E
40°N
130°E
35°N
30°N
125°E
25°N

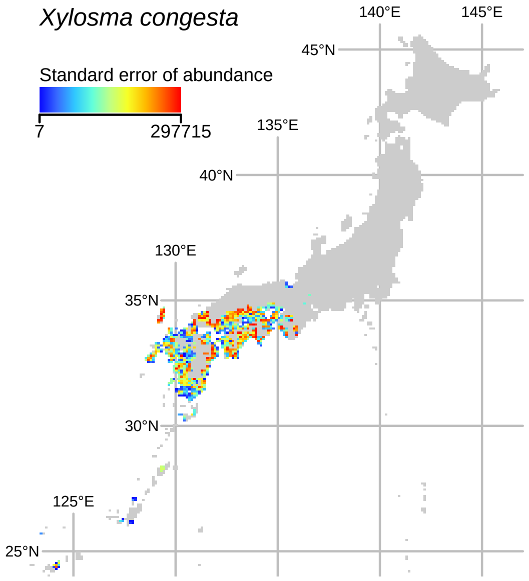
Xylosma congesta
Standard error of abundance
7
297715
140°E
145°E
45°N
135°E
40°N
130°E
35°N
30°N
125°E
25°N

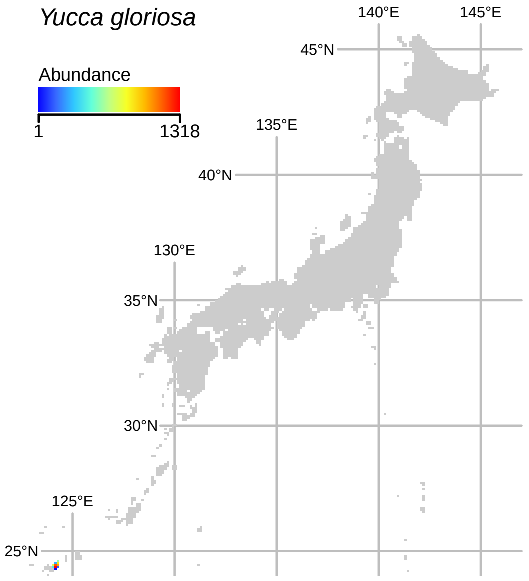
Yucca gloriosa
Abundance
1
1318
140°E
145°E
45°N
135°E
40°N
130°E
35°N
30°N
125°E
25°N

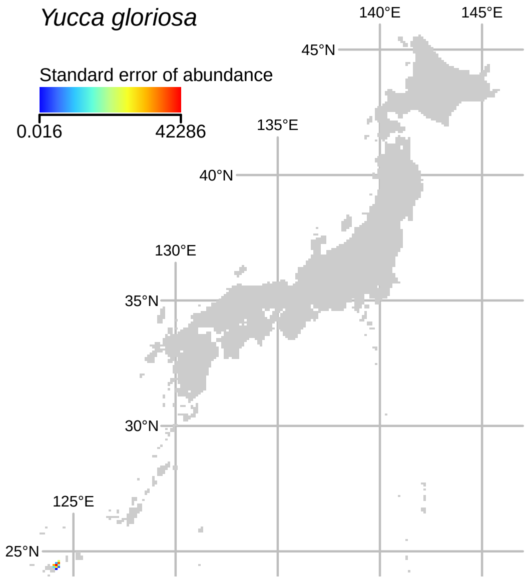
Yucca gloriosa
Standard error of abundance
0.016
42286
140°E
145°E
45°N
135°E
40°N
130°E
35°N
30°N
125°E
25°N

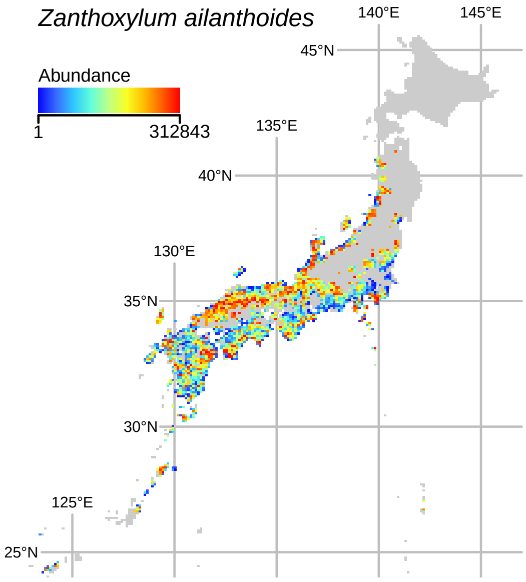
Zanthoxylum ailanthoides
Abundance
1
312843
140°E
145°E
45°N
135°E
40°N
130°E
35°N
30°N
125°E
25°N

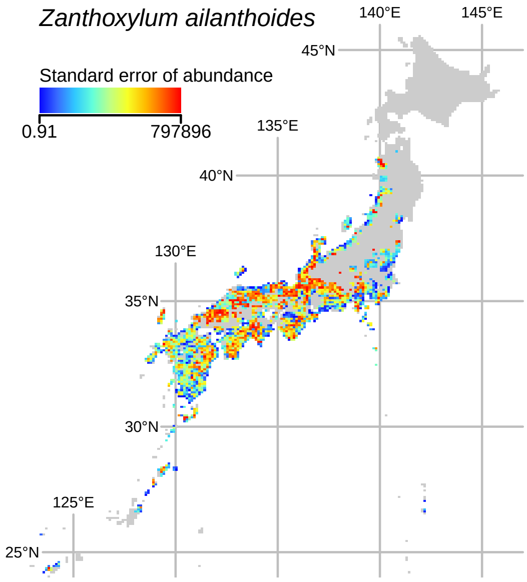
Zanthoxylum ailanthoides
Standard error of abundance
0.91
797896
140°E
145°E
45°N
135°E
40°N
130°E
35°N
30°N
125°E
25°N

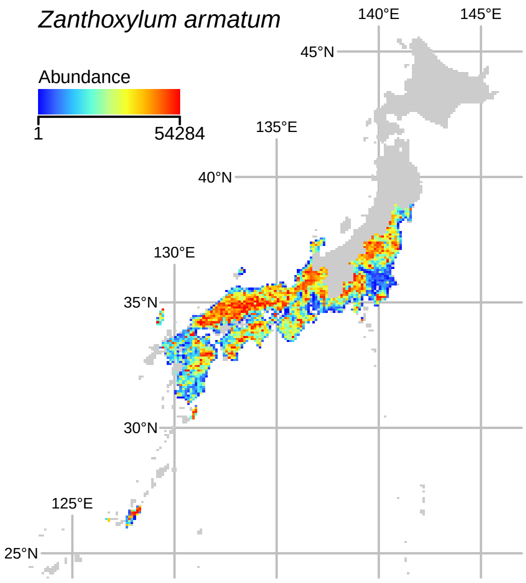
Zanthoxylum armatum
Abundance
1
54284
140°E
145°E
45°N
135°E
40°N
130°E
35°N
30°N
125°E
25°N

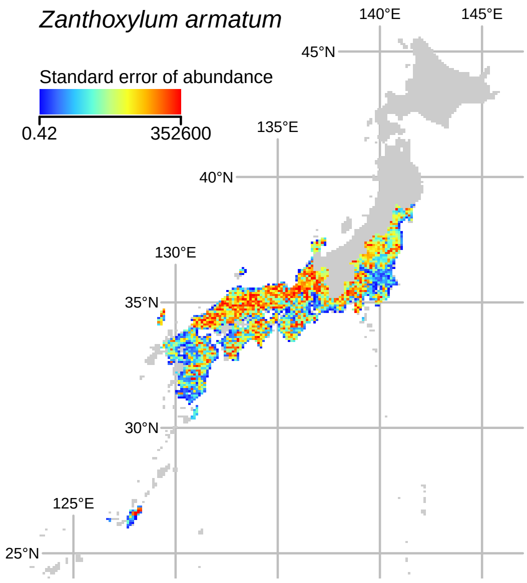
Zanthoxylum armatum
Standard error of abundance
0.42
352600
140°E
145°E
45°N
135°E
40°N
130°E
35°N
30°N
125°E
25°N

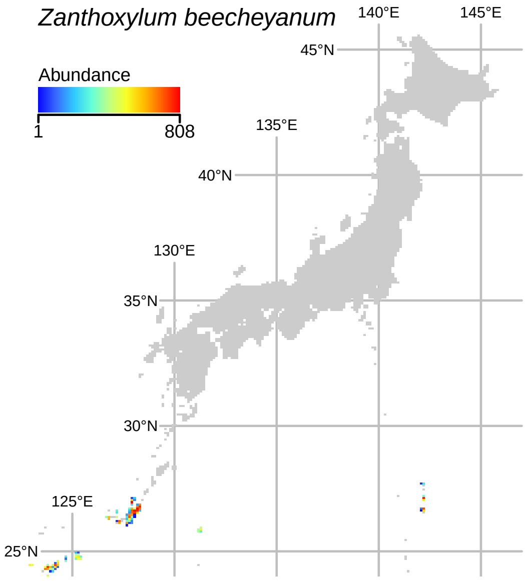
Zanthoxylum beecheyanum
Abundance
1
808
140°E
145°E
45°N
135°E
40°N
130°E
35°N
30°N
125°E
25°N

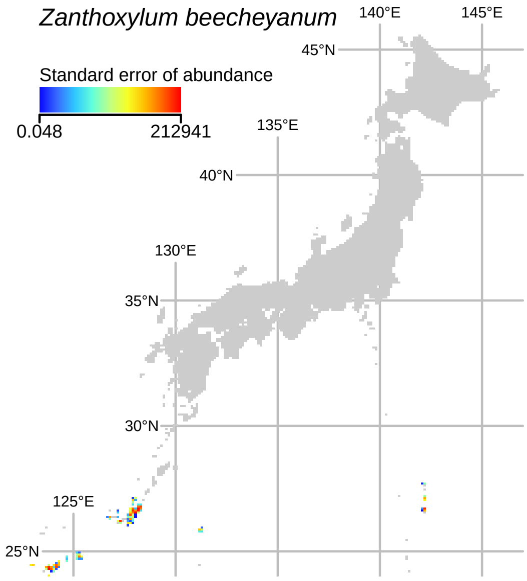
Zanthoxylum beecheyanum
Standard error of abundance
0.048
212941
140°E
145°E
45°N
135°E
40°N
130°E
35°N
30°N
125°E
25°N

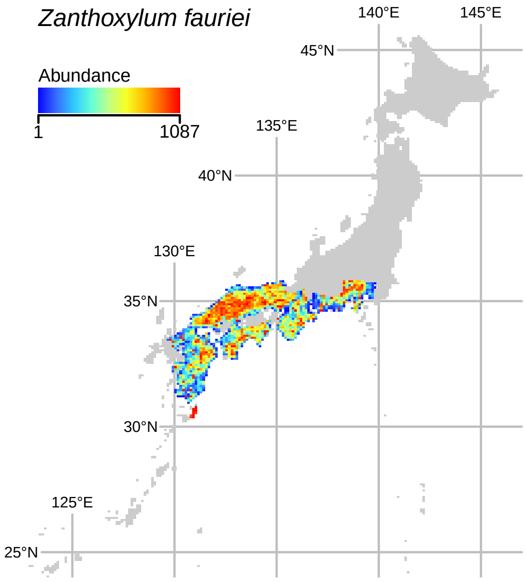
Zanthoxylum fauriei
Abundance
1
1087
140°E
145°E
135°E
130°E
125°E
45°N
40°N
35°N
30°N
25°N

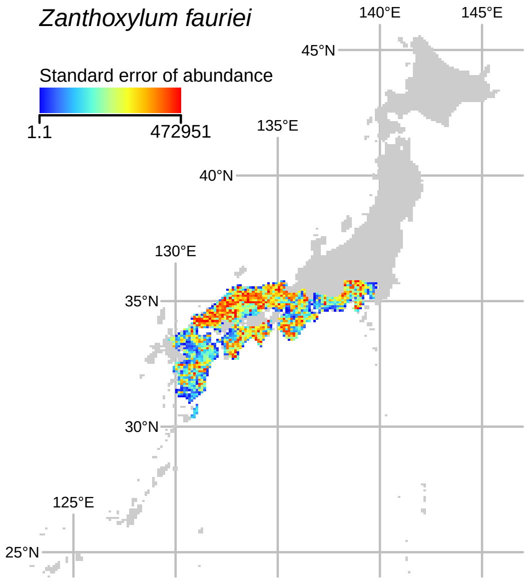
Zanthoxylum fauriei
Standard error of abundance
1.1
472951
140°E
145°E
135°E
130°E
125°E
45°N
40°N
35°N
30°N
25°N

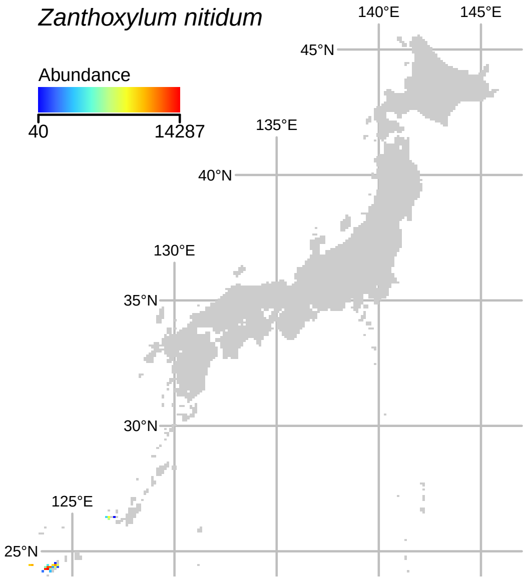
Zanthoxylum nitidum
Abundance
40
14287
140°E
145°E
135°E
130°E
125°E
45°N
40°N
35°N
30°N
25°N

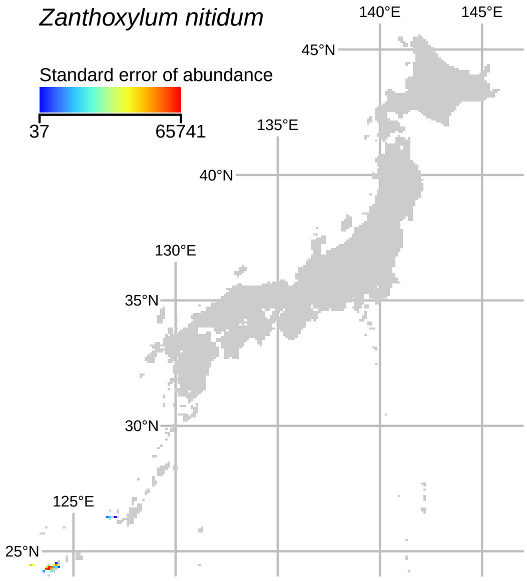
Zanthoxylum nitidum
Standard error of abundance
37
65741
140°E
145°E
135°E
130°E
125°E
45°N
40°N
35°N
30°N
25°N

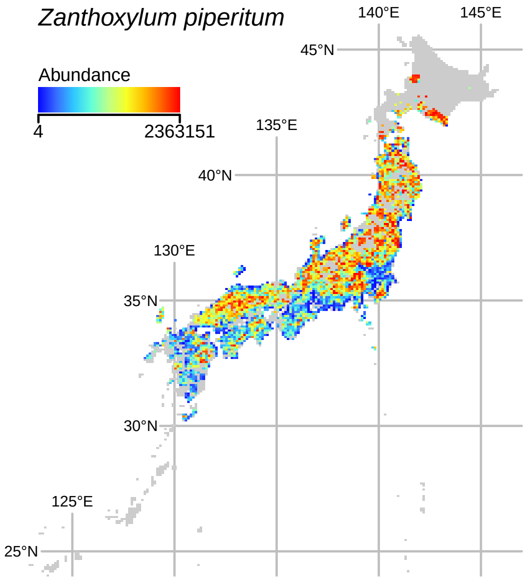
Zanthoxylum piperitum
Abundance
4
2363151
140°E
145°E
135°E
130°E
125°E
45°N
40°N
35°N
30°N
25°N

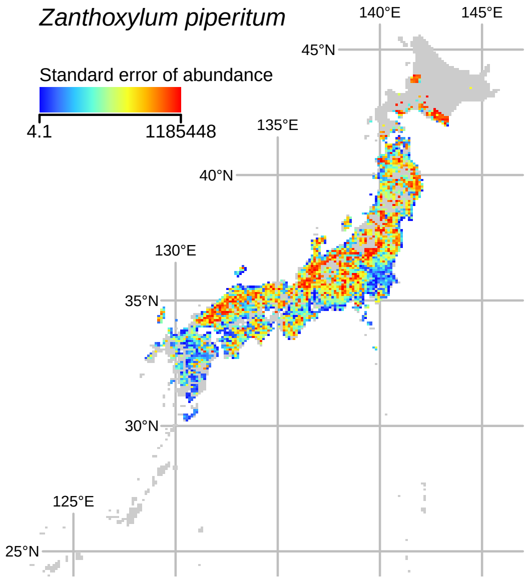
Zanthoxylum piperitum
Standard error of abundance
4.1
1185448
140°E
145°E
135°E
130°E
125°E
45°N
40°N
35°N
30°N
25°N

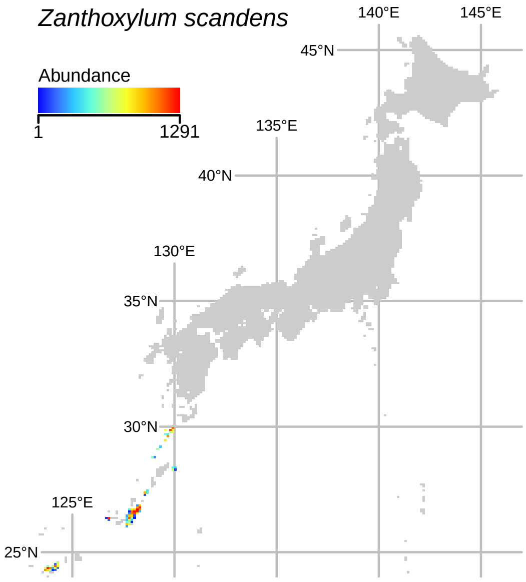
Zanthoxylum scandens
Abundance
1
1291
140°E
145°E
45°N
135°E
40°N
130°E
35°N
30°N
125°E
25°N

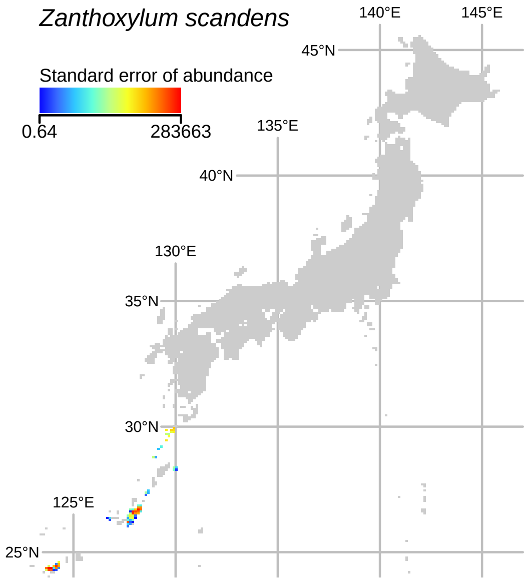
Zanthoxylum scandens
Standard error of abundance
0.64
283663
140°E
145°E
45°N
135°E
40°N
130°E
35°N
30°N
125°E
25°N

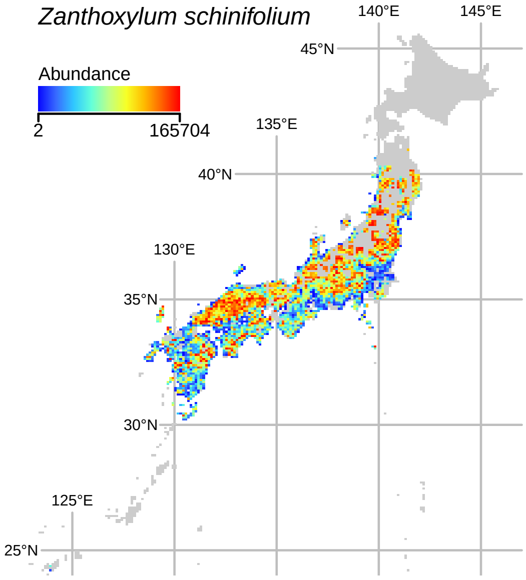
Zanthoxylum schinifolium
Abundance
2
165704
140°E
145°E
45°N
135°E
40°N
130°E
35°N
30°N
125°E
25°N

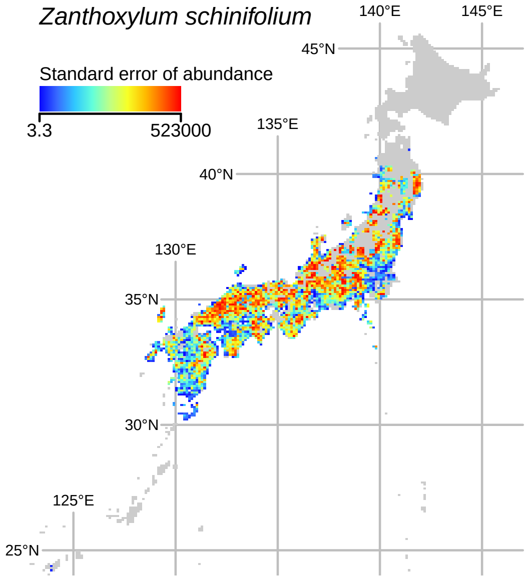
Zanthoxylum schinifolium
Standard error of abundance
3.3
523000
140°E
145°E
45°N
135°E
40°N
130°E
35°N
30°N
125°E
25°N

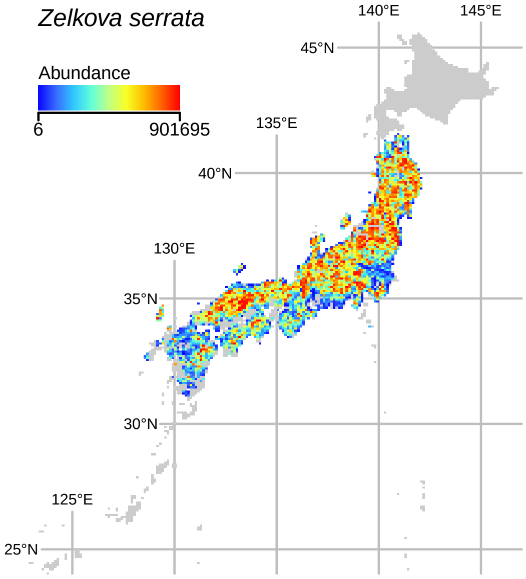
Zelkova serrata
Abundance
6
901695
140°E
145°E
45°N
135°E
40°N
130°E
35°N
30°N
125°E
25°N

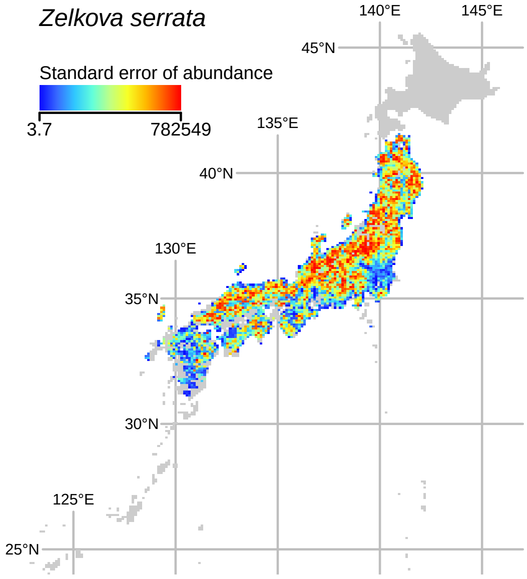
Zelkova serrata
Standard error of abundance
3.7
782549
140°E
145°E
45°N
135°E
40°N
130°E
35°N
30°N
125°E
25°N

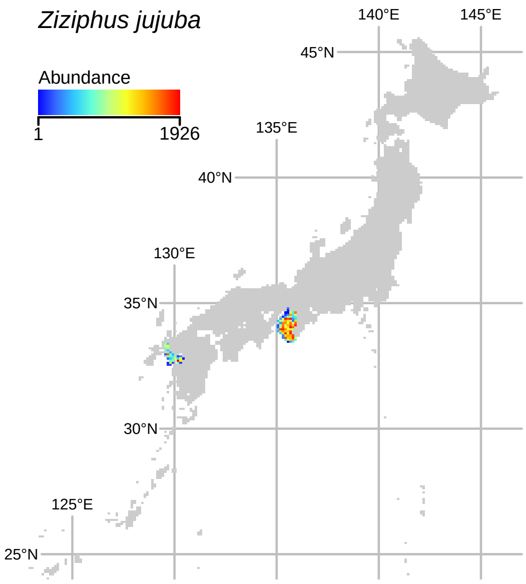

Ziziphus jujuba
Abundance
1
1926
140°E
145°E
45°N
135°E
40°N
130°E
35°N
30°N
125°E
25°N

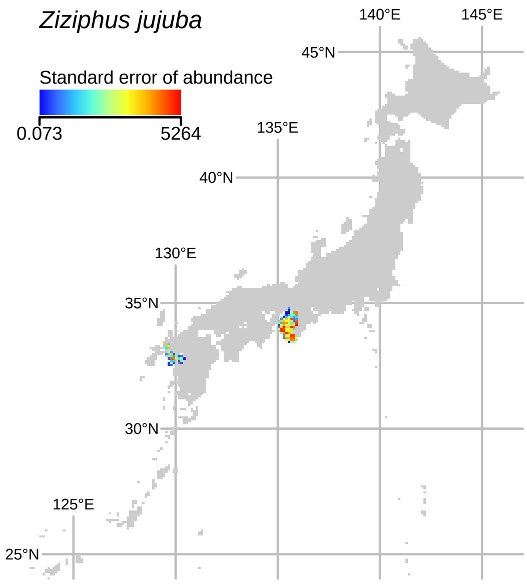

Ziziphus jujuba
Standard error of abundance
0.073
5264
140°E
145°E
45°N
135°E
40°N
130°E
35°N
30°N
125°E
25°N

Supplementary References

[1] Carpenter, B., Gelman, A., Hoffman, M.D., Lee, D., Goodrich, B., Betancourt, M., Brubaker, M.A., Guo, J., Li, P. & Riddell, A. (2017) Stan: a probabilistic programming language. *Journal of Statistical Software*, **76**, 1–32.

[2] Fournier, D.A., Skaug, H.J., Ancheta, J., Ianelli, J., Magnusson, A., Maunder, M.N., Nielsen, A. & Sibert, J. (2012) AD Model Builder: using automatic differentiation for statistical inference of highly parameterized complex nonlinear models. *Optimization Methods and Software*, **27**, 233–249.

[3] King, R. (2014) Statistical ecology. *Annual Review of Statistics and Its Application*, **1**, 401–426.

[4] Kristensen, K., Nielsen, A., Berg, C.W., Skaug, H. & Bell, B.M. (2016) TMB: automatic differentiation and Laplace approximation. *Journal of Statistical Software*, **70**, 1–21.

[5] Kusumoto, B., Shiono, T., Konoshima, M., Yoshimoto, A., Tanaka, T. & Kubota, Y. (2017) How well are biodiversity drivers reflected in protected areas? A representativeness assessment of the geohistorical gradients that shaped endemic flora in Japan. *Ecological Research*, **32**, 299–311.

[6] Plummer, M. (2003) JAGS: A program for analysis of Bayesian graphical models using Gibbs sampling. *Proceedings of the 3rd international workshop on distributed statistical computing (DSC 2003)*, volume 124, p. 125. Technische Universit at Wien, Austria.

[7] Sanderson, E.W., Jaiteh, M., Levy, M.A., Redford, K.H., Wannebo, A.V. & Woolmer, G. (2002) The human footprint and the last of the wild. *BioScience*, **52**, 891–904.
