# Supplementary material for: Integrating multiple sources of ecological data to unveil macroscale species abundance
Source: Nat Commun. 2020 Apr 3;11:1695. doi: 10.1038/s41467-020-15407-5 (PMC7125090; doi:10.1038/s41467-020-15407-5)
Supplement: Supplementary file 3 — Reporting Summary [file 41467_2020_15407_MOESM3_ESM.pdf]

natureresearch

Corresponding author(s): Keiichi Fukaya
Last updated by author(s): 2020/2/14

Reporting Summary

Nature Research wishes to improve the reproducibility of the work that we publish. This form provides structure for consistency and transparency in reporting. For further information on Nature Research policies, see Authors & Referees and the Editorial Policy Checklist .

Statistics

For all statistical analyses, confirm that the following items are present in the figure legend, table legend, main text, or Methods section.

n/a	Confirmed	
☐	☒	The exact sample size (n) for each experimental group/condition, given as a discrete number and unit of measurement
☐	☒	A statement on whether measurements were taken from distinct samples or whether the same sample was measured repeatedly
☒	☐	The statistical test(s) used AND whether they are one- or two-sided<br>*Only common tests should be described solely by name; describe more complex techniques in the Methods section.*
☐	☒	A description of all covariates tested
☐	☒	A description of any assumptions or corrections, such as tests of normality and adjustment for multiple comparisons
☐	☒	A full description of the statistical parameters including central tendency (e.g. means) or other basic estimates (e.g. regression coefficient) AND variation (e.g. standard deviation) or associated estimates of uncertainty (e.g. confidence intervals)
☒	☐	For null hypothesis testing, the test statistic (e.g. F, t, r) with confidence intervals, effect sizes, degrees of freedom and P value noted<br>*Give P values as exact values whenever suitable.*
☒	☐	For Bayesian analysis, information on the choice of priors and Markov chain Monte Carlo settings
☐	☒	For hierarchical and complex designs, identification of the appropriate level for tests and full reporting of outcomes
☒	☐	Estimates of effect sizes (e.g. Cohen's d, Pearson's r), indicating how they were calculated

Our web collection on statistics for biologists contains articles on many of the points above.

Software and code

Policy information about availability of computer code

Data collection	No software was used.
Data analysis	R (versions 3.5.0 and 3.5.1) and TMB package (version 1.7.15) were used to analyse the data. The Template Model Builder code for the fitted model is available in the supplementary files (Supplementary Data 1) and from GitHub (https://github.com/fukayak/mSAD_TMB).

For manuscripts utilizing custom algorithms or software that are central to the research but not yet described in published literature, software must be made available to editors/reviewers. We strongly encourage code deposition in a community repository (e.g. GitHub). See the Nature Research guidelines for submitting code & software for further information.

Data

Policy information about availability of data

All manuscripts must include a data availability statement. This statement should provide the following information, where applicable:

- Accession codes, unique identifiers, or web links for publicly available datasets
- A list of figures that have associated raw data
- A description of any restrictions on data availability

The datasets of vegetation survey and species geographic distribution, that were analysed in the current study, are available at https://www.givd.info/ID/AS-JP-002 upon request from the Global Index of Vegetation-Plot Databases (GIVD). See the GIVD rules for details at: https://www.givd.info/index.xhtml. Other data sources include the national survey of the natural environment (http://www.biodic.go.jp/trialSystem/EN/info/vg.html), the forest dynamics plots (FDP: http://www.biodic.go.jp/moni1000/findings/data/index_file.html), the national forest inventory plots (NFI: https://www.rinya.maff.go.jp/j/keikaku/tayouseichousa/chousadeta.html), and the global map of forest trees (GMFT: https://elischolar.library.yale.edu/yale fes data/1/). The forest sampling plots along latitudinal and elevational gradients (FSLE) dataset is unpublished data available upon reasonable request.

Field-specific reporting

Please select the one below that is the best fit for your research. If you are not sure, read the appropriate sections before making your selection.

☐ Life sciences ☐ Behavioural & social sciences ☒ Ecological, evolutionary & environmental sciences

For a reference copy of the document with all sections, see nature.com/documents/nr-reporting-summary-flat.pdf

Ecological, evolutionary & environmental sciences study design

All studies must disclose on these points even when the disclosure is negative.

Study description	In order to estimate large scale patterns of tree species abundance, we modelled a large dataset of woody plant communities along with geographical ranges of species from various data sources.
Research sample	A research sample is considered to be detection-nondetection observations of species obtained from a vegetation survey, which is used to estimate species abundances. The data sources are Kusumoto et al. (2015) and the national vegetation survey of Japan. We also used geographic species occurrence information collected from various data sources including museum and herbarium specimens, species occurrence records, distribution maps of plant species, and regional species checklists (Horikawa et al. 1972, Kubota et al. 2014). The validation datasets include the forest inventory data (FDP, NFI, and FSLE) and a global estimate of tree density (GMFT; Crowther et al. 2015).
Sampling strategy	Available survey records were collected exhaustively. No sample-size calculation was performed prior to the analysis because a statistical hypothesis testing was not the major aim of this study.
Data collection	The dataset was compiled based on the existing records of vegetation surveys, museum and herbarium specimens, species occurrence records, distribution maps of plant species, and regional species checklists.
Timing and spatial scale	Spatial scale: Mid-latitude forests on East Asian islands, including the Japanese archipelago. The vegetation plots were placed in natural forests in various successional stages between 24◦02′- 45◦ 30′ N and 122◦ 56′- 153◦ 59′ E. Temporal scale: The time period of the vegetation surveys spans from 1954 to 2013.
Data exclusions	Some of the vegetation survey data were excluded before the analysis because required information such as plot area was not available.
Reproducibility	Computer codes are available for reproduction of the results.
Randomization	No randomization was required in this study.
Blinding	Not applicable to this study.

Did the study involve field work? ☐ Yes ☒ No

Reporting for specific materials, systems and methods

We require information from authors about some types of materials, experimental systems and methods used in many studies. Here, indicate whether each material, system or method listed is relevant to your study. If you are not sure if a list item applies to your research, read the appropriate section before selecting a response.

Materials & experimental systems

n/a	Involved in the study	
☒	☐	Antibodies
☒	☐	Eukaryotic cell lines
☒	☐	Palaeontology
☒	☐	Animals and other organisms
☒	☐	Human research participants
☒	☐	Clinical data

Methods

n/a	Involved in the study	
☒	☐	ChIP-seq
☒	☐	Flow cytometry
☒	☐	MRI-based neuroimaging
