# Supplementary material for: Integrating multiple sources of ecological data to unveil macroscale species abundance
Source: Nat Commun. 2020 Apr 3;11:1695. doi: 10.1038/s41467-020-15407-5 (PMC7125090; doi:10.1038/s41467-020-15407-5)
Supplement: Supplementary file 4 — Description of Additional Supplementary Files [file 41467_2020_15407_MOESM4_ESM.pdf]

Description of Additional Supplementary Files

File Name: Supplementary Software 1
Description: The **Template Model Builder** code for the fitted model. It is supplied to the main function in the **TMB** package to calculate the marginal likelihood of the model described in the *Methods* section (subsection, *Model fitting and inference*), and thus can be used to obtain maximum likelihood estimates in **R**.
